# An efficient material search for room-temperature topological magnons

**DOI:** 10.1126/sciadv.ade7731

**Published:** 2023-02-17

**Authors:** Mohammed J. Karaki, Xu Yang, Archibald J. Williams, Mohamed Nawwar, Vicky Doan-Nguyen, Joshua E. Goldberger, Yuan-Ming Lu

**Affiliations:** ^1^ Department of Physics, The Ohio State University, Columbus, OH 43210, USA.; ^2^ Department of Chemistry and Biochemistry, The Ohio State University, Columbus, OH 43210, USA.; ^3^ Department of Material Science and Engineering, The Ohio State University, Columbus, OH 43210, USA.

## Abstract

Topologically protected magnon surface states are highly desirable as an ideal platform to engineer low-dissipation spintronics devices. However, theoretical prediction of topological magnons in strongly correlated materials proves to be challenging because the ab initio density functional theory calculations fail to reliably predict magnetic interactions in correlated materials. Here, we present a symmetry-based approach, which predicts topological magnons in magnetically ordered crystals, upon applying external perturbations such as magnetic/electric fields and/or mechanical strains. We apply this approach to carry out an efficient search for magnetic materials in the Bilbao Crystallographic Server, where, among 198 compounds with an over 300-K transition temperature, we identify 12 magnetic insulators that support room-temperature topological magnons. They feature Weyl magnons with surface magnon arcs and magnon axion insulators with either chiral surface or hinge magnon modes, offering a route to realize energy-efficient devices based on protected surface magnons.

## Teaser

A symmetry-based approach leads to the efficient discovery of magnets hosting topological magnons.

## INTRODUCTION

Since the discovery of topological insulators and associated *Z*_2_ topological invariants (*1*–*3*), much progress has been made to reveal topological properties encoded in the electronic band structures of weakly correlated quantum materials (*4*–*7*). Not only has a full classification of topological bands been achieved, but recent efforts have also led to a complete catalog of topological electronic materials based on ab initio calculations of their band structures (*8*–*12*). One natural question arises: Can the huge success of topological band theory be extended to predict the topology of strongly correlated materials?

In materials with long-range magnetic orders, the spin-wave excitations can exhibit nontrivial topology and robust surface states, known as topological magnons [see (*13*) and references therein for a review of the literature]. Because of protected surface states insensitive to system geometry and defects, topological magnons provide a new route toward robust and low-dissipation magnon-based circuitry in magnon spintronics (*14*, *15*), making high-quality materials hosting topological magnons very desirable. However, in contrast to efficiently searched and completely cataloged topological electronic materials, a systematic search for topological magnon materials has not been achieved so far (*13*, *16*, *17*). In particular, two main challenges lie ahead of us.

First of all, unlike the electronic band topology, which has been thoroughly understood and classified, much less is known about the magnon band topology. In electronic bands, after the 10-fold way classification of strong topological invariants protected by global symmetries (*7*), a complete classification of band topology in any space group has been achieved by the theoretical developments of symmetry indicators (SIs) (*18*, *19*) and topological quantum chemistry (*8*, *20*). In contrast, the spin-wave bands are described by a quadratic boson Hamiltonian, known to be very different from a fermion system (*21*). Despite recent progress, which established a map connecting the bosonic band topology to fermionic ones (*13*, *22*, *23*), a complete classification of magnon band topology is still not available (*13*, *16*, *17*).

Second, strong correlations of magnetic materials make it a challenging task to systematically predict the topology of magnon bands in materials. In electronic materials, with the help of ab initio density functional theory calculations of band structures in existing materials, a catalog of topological electronic materials has recently been achieved (*8*–*12*). In contrast, because of the lack of reliable ab initio calculations, theoretically determining the microscopic spin model of a magnetic material, and hence its magnon bands, has been a longstanding problem in strongly correlated systems. Typically, we rely on experimental inputs, for example, by fitting inelastic neutron scattering data, to determine the exchange interactions on a material-by-material basis. This severely hinders any large-scale search for materials hosting topological magnons.

**Fig. 1.**
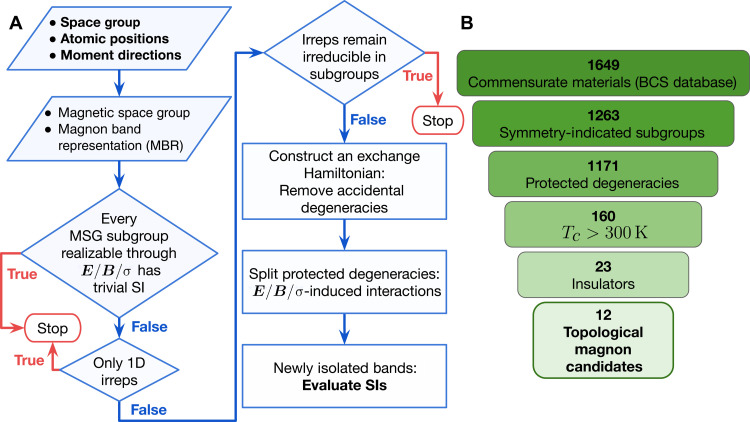
Workflow and summary of results. (**A**) Flowchart diagram highlighting the steps for systematic diagnosis of topological magnons in a generic magnetic material. MSG, magnetic space group; irreps, irreducible representations. (**B**) A summary of the search outcome. Explicit spin-wave calculations are performed in the last step to the 23 room-temperature magnetic insulators that pass all the group-theoretical filters. 1D, one-dimensional.

In this work, we show that a systematic material search for topological magnons can be carried out efficiently using a symmetry-based approach summarized in Fig. 1. We performed such a semiautomated search among all insulating materials with a room temperature (*T_c_ >* 300 K) magnetic order in the Bilbao Crystallographic Server (BCS) and identified 12 candidate materials that can host room-temperature topological magnons. In the main text, we outline our strategy, the search algorithm, and outcome. We highlight two examples among the search results and comment on the synthesis of candidate materials. We list the detailed search process and data in the Supplementary Materials.

## RESULTS

### Theory framework

As shown previously (*21*–*23*), the linear spin-wave (LSW) problem of diagonalizing a quadratic Hamiltonian of bosons can be exactly mapped to the diagonalization of a fermionic Bogoliubov-de Gennes Hamiltonian, with the same symmetry group and band topology. This motivated us to apply the theory of SIs and topological quantum chemistry developed in electronic bands to magnon excitations described by the LSW theory. In the theory of topological quantum chemistry (*8*, *20*), a generic band structure always arises from localized orbitals located at symmetry-respecting locations known as the Wyckoff positions of a space group. The band representations in momentum space are strongly constrained by the possible Wyckoff positions and orbital symmetries. In particular, the band representations of a trivial atomic insulator can be enumerated for a given space group. If a band representation does not fit into any atomic insulator or their composites, the associated band structure must be topological, and these nontrivial band representations at high-symmetry momenta are known as SIs for topological bands (*18*, *19*, *24*, *25*). In the case of electronic bands in (weakly correlated) solid-state materials, ab initio calculations based on the density functional theory is used to compute the SIs at high-symmetry momenta. If one set of connected bands is separated from other bands at the high-symmetry momenta, its topology can be implied by its SIs, which enabled a complete search and catalog for topological electronic materials (*8*–*12*).

In our problem of spin waves in magnetic insulators, the localized spin moments of ordered magnetic ions play the role of localized electronic orbitals in the theory of topological quantum chemistry. To be specific, for a magnetic ion whose spin ***S*** aligns along the *z* axis with $\langle S\rangle =S\hat{z.}$

(*S* is the spin magnetic moment), its LSW variable $b=(S^{x}+iS^{y})/\sqrt{2S}$ (known as Holstein-Primakoff boson) corresponds to the electron annihilation operator for the localized orbital in the electronic problem, and its symmetry transformations under the site symmetry group of the magnetic ion determine the orbital symmetry at its Wyckoff site. This allows us to constrain and classify all possible band representations of magnons in a magnetic space group (MSG) by mapping to its electronic counterparts discussed in (*19*, *20*, *26*).

This, unfortunately, is not enough for a large-scale material search for topological magnons, mostly obstructed by the lack of microscopic spin models for a given magnetic material. In weakly correlated electronic materials, ab initio calculations are readily available to reliably predict the energy and SIs of electronic bands. On the contrary, although magnon band representations are constrained by the space group and magnetic order as mentioned above, the connectivity and SIs of magnon bands are usually not uniquely fixed by symmetry and generally depend on the microscopic spin-spin interactions in the material. How do we overcome this difficulty and systematically search for topological magnons?

Our strategy is to start from materials with symmetry-enforced magnon band degeneracy. More precisely, using the theory of topological quantum chemistry, we search for the MSGs and Wyckoff positions that host at least two connected bands in every possible band representation. Next, among the search results, we further identify those MSGs and Wyckoff positions where the protected degeneracy is lifted (i.e., the band crossings are gapped out) by external perturbations, including electric field ***E***, magnetic (Zeeman) field ***B***, and/or mechanical strains σ, which break the MSG down to a subgroup with separated magnon bands. The SIs of these separated bands will be used to diagnose the magnon band topology. These search results are robust predictions from symmetry and representation theory and independent of the microscopic spin models. Last, we ask how the SIs of the separated bands depend on the external perturbations and therefore determine the topological magnons induced by external perturbations. Our predictions are then consolidated by concrete spin models, which incorporate dominant nearest-neighbor Heisenberg exchange interactions and other symmetry-allowed terms.

### Search algorithm

We now describe the workflow of our material search process summarized in Fig. 1A. Our starting point requires basic input about the structure and symmetry, which consists of the space group of the crystal structure, the positions of the magnetic atoms, and their magnetic moments of the long-range magnetic order. This information is sufficient to fix the MSG describing the magnetic structure and the magnetic Wyckoff positions of the magnetic atoms. Using the theory of topological quantum chemistry, we can classify and determine all possible magnon band representations for a given MSG and the associated Wyckoff positions.

In the next step, following the strategy described previously, we need to identify (i) connected magnon bands with protected degeneracy in the magnetically ordered material; (ii) separated magnon bands when the degeneracy is split by external perturbations, including electric field ***E***, magnetic (Zeeman) field ***B***, and mechanical strains σ; and (iii) nontrivial SIs of the separated bands supported by the magnetic subgroup reduced from the original MSG by the symmetry-breaking perturbations. This step is implemented by two filters on the MSG and Wyckoff positions. First, we search for the MSGs and magnetic Wyckoff positions—at least one of whose magnetic subgroups support a nontrivial SI group. In other words, we screen out those MSGs whose subgroups all have a trivial (ℤ_1_) SI group. Among the 1651 MSGs, only 345 (including 50 gray groups) violate the first constraint (see the Supplementary Materials). Second, we require the MSG and Wyckoff positions to host symmetry-protected magnon band degeneracy, which can be split by perturbations that break the original MSG to a subgroup with nontrivial SIs. More precisely, we require the magnon bands constrained by the MSG and Wyckoff positions to have at least one multidimensional irreducible representation, which will split when the MSG is broken down to a subgroup. Of all 9182 Wyckoff positions compatible with a commensurate magnetic order in all MSGs, only 1094 violate the second constraint.

We have applied these theoretical constraints on MSGs and Wyckoff positions to all magnetic materials in the BCS database. Among 1649 commensurate structures, we find that 1263 entries pass the first filter, and 1171 entries pass both filters, as shown in Fig. 1B. These structures are summarized in the Supplementary Materials. Here, of the 1171 compounds passing the group-theoretical filters, we focus on the 23 room-temperature magnetic insulators with *T_c_ >* 300 K and a charge gap.

The last step of the search process is to determine the SIs of magnon bands after external perturbations are applied to the magnetic materials. We applied LSW theory to generic spin models, with dominant Heisenberg exchange interactions supplemented by other symmetry-allowed terms, to determine the SIs and topology of the resulting magnon bands. The search outcomes are 12 magnetic insulators that host either Weyl magnons (*27*) or the magnonic analog of axion insulators (labeled as “magnon axion insulators”), listed in Table 1 (*28*, *29*). We categorize the 12 candidate materials into three types, (I), (II), and (III) as labeled in Table 1:

**Table 1.** Room-temperature magnetic insulator candidates for topological magnons. The superscripts in the last column indicate whether a candidate is type I, II, or III, as explained in the main text.

Material [*T*_c_ (K)]	Original MSG	Perturbation	MSG subgroup	Predicted topology
NdFeO_3_ (760) CeFeO_3_ (720) NaOsO_3_ (410) TbFeO_3_ (681) SmFeO_3_ (670) LaCrO_3_ (310)	*Pn*^′^*m*a^′^ (62.448)	$\vec{B}∥[001]$	$P2_{1}^{\prime }/c^{\prime }\;(14.79)$	Weyl magnons
		Strain ⊥ [001]	$P2_{1}^{\prime }/c^{\prime }\;(14.79)$	Weyl magnons^I^
		Strain ⊥ [100]	$P2_{1}^{\prime }/c^{\prime }\;(14.79)$	Weyl magnons^I^
		Strain ⊥ [010]	*P*2_1_/*m* (11.50)	Trivial
α-Fe_2_O_3_ (955)	*C*2^′^/*c*^′^(15.89)	$\vec{B}∥[010]$	$P\bar{1}\;(2.4)$	Weyl magnons^III^
SrRu_2_O_6_ (565)	$P_{c}\bar{3}1m\;(162.78)$	$\vec{B}∥[001]$ and generic strain	$P\bar{1}\;(2.4)$	Weyl/magnon axion insulator^III^
CoF_3_ (460)	$R\bar{3}c\;(167.103)$	$\vec{B}∥[001]$ and strain ∥[110]	$P\bar{1}\;(2.4)$	Weyl magnons^III^
FeF_3_ (394)	*C*2^′^/*c*^′^(15.89)	$\vec{B}∥[010]$	$P\bar{1}\;(2.4)\;P\bar{1}\;(2.4)$	Weyl magnons^II^
		$\vec{B}∥[010]$ and generic strain		Magnon axion insulator^II^
LaSrFeO_4_ (380)	*P_C_*4_2_/*nnm* (134.481)	$\vec{E}∥[110]$ and strain⊥[110]	*C_a_*2 (5.17)	Weyl magnons^III^
MnTe (323)	*Cmcm* (63.457)	strain⊥[100]	*C*2/*m* (12.58)	Weyl/magnon axion insulator^II^
		$\vec{B}∥[100]$ and strain ⊥[010]	$P\bar{1}\;(2.4)$	Weyl magnons^II^

(I) Topological magnons can be obtained irrespective of the form of the perturbations, as long as the original MSG is broken down to a certain subgroup. The examples include the rare-earth perovskites RMO_3_ in the top row of Table 1.

(II) The type of topological magnons or, more precisely, the SIs of separated magnon bands depend on the type of external perturbations applied to the system. The examples include FeF_3_ and MnTe in Table 1, where different perturbations can give rise to either Weyl magnons or magnon axion insulators.

(III) The SIs of magnon bands depend not only on the form of external perturbations but also on the unperturbed magnon band structure. In this case, we make predictions based on microscopic spin models, with a dominant Heisenberg exchange interaction and other symmetry-allowed terms. For example, we use a *J*_1_-*J*_2_-*J*_3_ Heisenberg model plus perturbations to predict the magnon band topology in the case of α-Fe_2_O_3_ (Fig. 2).

**Fig. 2.**
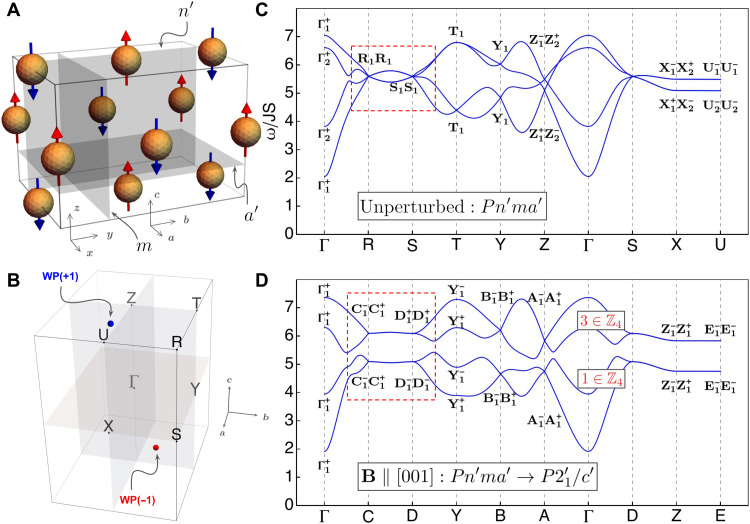
Weyl magnons induced by a magnetic field or uniaxial strain in TbFeO_3_. (**A** and **B**) Magnetic unit cell and Brillouin zone of TbFeO_3_ showing only the ordered Fe3^+^ moments. WP, Weyl point. (**C**) Magnon spectrum in the absence of external perturbations, showing two fourfold degeneracies at momenta R and S, protected by the MSG symmetry. The labels at each high-symmetry momentum indicate the irreducible representation of each state, and the ± superscripts indicate whether the state is even or odd under inversion, respectively. (**D**) Magnon spectrum upon the application of a magnetic field, reducing the MSG and splitting the four connected bands into two pairs of bands. Each pair of bands acquires a nontrivial ν = 1,3 ∈ ℤ_4_ inversion SI, corresponding to Weyl magnons shown in (B) and Fermi arcs on the material surface.

Type (I) topological magnons are the most robust ones among our search results for their presence depends neither on the form of the unperturbed Hamiltonian nor the perturbations but only on the MSGs before and after external perturbations are applied. Below, we present two candidate materials as examples of our search results in Table 1.

### Examples

Below, we highlight two examples of room-temperature topological magnons in magnetic insulators. The first example is G-type antiferromagnetic perovskite TbFeO_3_, as depicted in Fig. 2A, with four ordered Fe^3+^ moments at the Wyckoff position 4*b* of its MSG *Pn*′*m*a′ (62.448). The Tb^3+^ ions remain paramagnetic until cooled down to 8.5 K (*30*, *31*). These basic inputs dictate a symmetry-enforced fourfold degeneracy at R and S and two twofold degeneracies at each of T, U, X, Y, and Z in the magnon dispersion in Fig. 2B.

An external magnetic field parallel to the moment direction reduces its symmetry group down to $P2_{1}^{\prime }/c^{\prime }$, as does any uniaxial strain in *ab* or *bc* plane. Figure 2C illustrates the effect of a magnetic (Zeeman) field on the magnon spectrum and magnon band representations. Note that each fourfold degeneracy in Fig. 2B is split into two two-dimensional (2D) irreducible representations of $P2_{1}^{\prime }/c^{\prime }$. The magnetic subgroup $P2_{1}^{\prime }/c^{\prime }$ has a ℤ_4_ SI group due to the inversion symmetry (*18*, *24*, *25*), characterized by the number of negative inversion eigenvalues at the eight inversion-symmetric *k-*point modulo 4.

The ℤ_4_ indicator of the higher (or lower) two bands in Fig. 2D must be an odd integer (ν = 1, 3 ∈ ℤ_4_) as long as the four connected bands of the group *Pn*′*m*a′ are split into two 2D irreducible representations of group $P2_{1}^{\prime }/c^{\prime }$. Therefore, the presence of Weyl magnons is robust, independent of either the microscopic spin model or the form of external perturbations. In particular, a magnetic field along the *c* axis creates a pair of Weyl magnons shown in Fig. 2B. A similar analysis applies to all six rare earth perovskites in the top row of Table 1, which share the same MSG and Wyckoff positions of magnetically ordered ions.

**Fig. 3.**
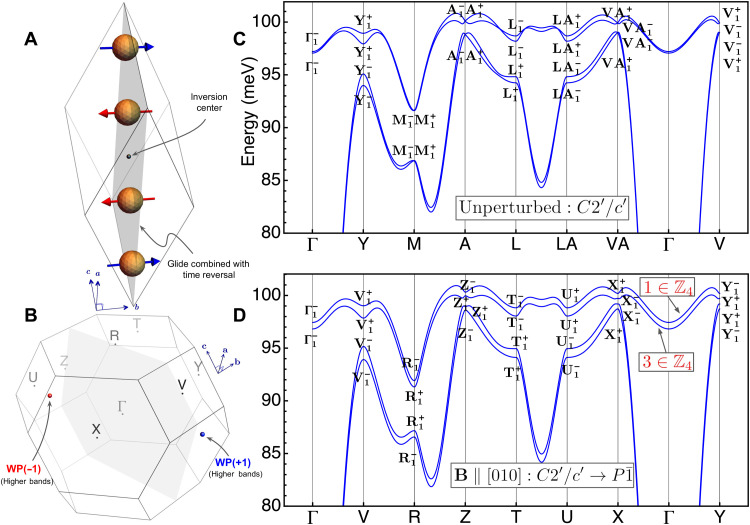
Magnetic field–induced Weyl magnons in α-Fe_2_O_3_. (**A** and **B**) Magnetic unit cell of α-Fe_2_O_3_ (only Fe atoms shown) and the Brillouin zone. (**C**) Unperturbed magnon spectrum with two pairs of bands separated at all high-symmetry momentum. The spectrum is calculated using the first 10 nearest-neighbor Heisenberg interactions obtained from inelastic neutron scattering data, which we supplemented with small anisotropy terms (see the Supplementary Materials). The higher two bands are connected because of several symmetry-protected degeneracies at high-symmetry points. (**D**) Magnon spectrum upon the application of B-field perturbation (2.5 T) gapping out the degeneracies within the higher two bands. The inversion SIs of the newly separated bands (illustrated in red) diagnose Weyl magnons thanks to their odd values. The WPs and their chirality are depicted in (B).

The second example is α-Fe_2_O_3_, an antiferromagnetic insulator with a monoclinic MSG *C*2′/*c*′ (*32*), shown in Fig. 3. The four sublattices of Fe^3+^ ions give rise to four magnon bands, and a generic spin model with dominant *J*_1_-*J*_2_-*J*_3_ Heisenberg terms leads to a gap at all inversion-symmetric *k* points between the two acoustic and two optical bands. If an external magnetic field is additionally applied in the [010] direction, the MSG symmetry is lowered to $P\bar{1}\;(2.4)$, lifting all the symmetry-protected degeneracies within the two optical bands and creating a nontrivial gap. More precisely, each of the higher two bands acquires a nontrivial inversion SI ν = 1, 3 ∈ ℤ_4_, corresponding to bulk Weyl magnons and surface magnon arcs (*27*). In contrast to the first example above, the magnetic interactions in α-Fe_2_O_3_ have been estimated with inelastic neutron scattering, where up to the 10th nearest-neighbor Heisenberg parameters have been fitted to the magnon spectrum (*33*). The magnon spectrum in Fig. 3C is reproduced using the reported Heisenberg terms in addition to other small symmetry-allowed terms to lift accidental degeneracies in the spectrum (see the Supplementary Materials). The perturbed spectrum and its SIs are shown in Fig. 3D, and the Weyl points are illustrated in Fig. 3B.

While we focused on the bulk magnon spectra and their band topology in the main text, their associated topological magnon surface states are discussed and illustrated in section S3. As a brief summary, while the bulk Weyl magnons always correspond to surface magnon arcs, magnon axion insulators in the bulk can exhibit two types of protected surface states. When the Chern number of the bulk magnon bands is nonzero in a 2D plane of the 3D Brillouin zone, the magnon axion insulator hosts chiral surface magnons (*28*). On the other hand, if the magnon Chern number vanishes in any 2D plane of the 3D Brillouin zone, the magnon axion insulator exhibits chiral hinge magnons (*34*).

### Synthetic accessibility of candidates

Because the proposed realization of topological magnons requires the application of magnetic/electric fields and/or mechanical strains along specific crystallographic directions, the preparation of crystals or thin films with macroscopic dimensions is essential. The synthesis of pure, high-quality crystals and thin films of the identified compounds have been well established. For hematite, chemical vapor transport has been used to produce millimeter-by-millimeter scale α-Fe_2_O_3_ (*35*). In the case of LaCrO_3_ thin films, the pseudocubic phase has been grown epitaxially on SrTiO_3_ (001) to use strain to tune magnetic and optical properties (*36*). Furthermore, substitutions in *M_x_*La_1−*x*_CrO_3_ (i.e., *M* = Sr, *x <* 0.25) has been used to control p-type doping and induce in-plane ≈2% compressive to ≈2% tensile strain (*37*). For *R*FeO_3_ (*R* = Nd, Ce, Tb, Sm), high-quality crystals on the order of centimeter (length) and millimeter (diameter) have been grown with floating zone techniques (*38*, *39*).

## DISCUSSION

In summary, in a symmetry-based approach using the theory of topological quantum chemistry and SIs, we carry out an efficient semiautomatic search for topological magnons in all high-transition temperature (*T_c_ >* 300 K) magnetic materials in the BCS database. We identified 12 magnetic insulator candidate materials, which host room-temperature topological magnons induced by external perturbations, including electric/magnetic fields and mechanical strains. They feature Weyl magnons with surface magnon arcs and magnonic axion insulators with either chiral surface or hinge magnon modes (*40*, *41*).

Now, we briefly comment on the limitations of this work. While our symmetry-based approach can reliably predict the presence/absence of a magnon spectrum gap and the associated topological properties, it does not give a quantitative prediction on the size of the magnon spectrum gap that protects the topological magnons. In reality, both the finite temperature and magnon-magnon interactions give rise to a broadening of the magnon spectrum and a finite lifetime of magnons (*42*–*45*). We expect the topological magnons to survive when the magnon spectrum gap is much larger than the broadening. Another effect that exists in real materials but not captured in our approach is disorder. It has been shown that topology is well defined even in the presence of disorders if the disorders on average preserve the symmetry that protects the topology (*46*–*48*). Most of the topological magnons predicted by our search algorithm are diagnosed by the inversion SI; therefore, we expect the topological magnons to be robust in the presence of weak disorders, which obey an average inversion symmetry.

The results of this work pave the road for future material synthesis and experimental discovery of room-temperature topological magnons in magnetic insulators. A complete search through the whole BCS database, irrespective of the magnetic transition temperature, is a natural next step of the current work. Moreover, our search strategy can be naturally applied to other bosonic topological excitations, such as topological phonons (*49*, *50*). In particular, phonons in magnetic materials naturally fit into our search algorithm, where the long-range magnetic orders at low temperatures can be viewed as perturbations to the high-temperature paramagnetic phase. We leave these interesting questions for future works.

## MATERIALS AND METHODS

### Review of SIs

Consider a set of bands that is energetically separated from other bands at all high-symmetry momenta. For each high-symmetry momentum *k*, the integer $n_{k}^{\alpha }$ is defined as the number of times the little-group irreducible representation $\rho_{k}^{\alpha }$ appears in the bands. These integers are well defined thanks to the requirement that the set of bands are isolated from other bands. The collection of these integers for all pairs of distinct high-symmetry momenta and irreducible representations forms an integer-valued “vector” $b=\{n_{k}^{\alpha }\}$ and is dubbed a band structure (*18*). Any band structure ***b*** must satisfy the so-called compatibility relations, which are a set of constraints needed to consistently patch together the representations while maintaining the gap at high-symmetry momenta. The set of all valid band structures is denoted by {BS}.

A special kind of band structures arises from considering localized atomic orbitals with vanishing hopping. In this case, the irreducible representation multiplicities $\left\{n_{k}^{\alpha }\right\}$ trivially satisfy the compatibility relations and thus constitute a band structure. The set {AI} denotes the collection of all such atomic insulators. This set can be obtained by listing all the band structures arising from all pairs of sites and site-symmetry group irreducible representations and by considering all combination thereof.

The topology of a given set of bands can be diagnosed on the basis of symmetry information by considering its band structure ***b***. If ***b*** ∈ {BS} but ***b***∉ {AI}, then it is not possible to adiabatically deform the bands to any atomic insulator; they must be topologically nontrivial. If ***b***∉ {BS} (and thus necessarily ***b***∉ {AI}), the bands violate the compatibility relations, signaling a violation of the gap condition at high-symmetry momenta. Last, nontrivial topology is not ruled out by ***b*** ∈ {AI}; it is just not detectable by SIs.

While the component-wise addition of two band structures corresponds to the direct sum of the representations of two bands, we can formally define the component-wise subtraction as the inverse operation. This allows the components of ***b*** to run negative, and the collections {AI} and {BS} become abelian groups of the same rank in all (magnetic) space groups (*18*, *19*). Here, we do not consider the case of fragile topology (*8*, *18*).

Last, the SI group is defined as the quotient

$$X_{\mathrm{BS}}=\frac{\left\{\mathrm{BS}\right\}}{\left\{\mathrm{AI}\right\}}=Z_{n1}\times Z_{n2}\times \cdots$$ (1)

### Spin-wave Hamiltonian, symmetry, and band representation

For a bilinear exchange Hamiltonian

$$H=\frac{1}{2}\sum_{i,j}\sum_{\alpha ,\beta =x,y,z}J_{i,j}^{\alpha ,\beta }S_{i}^{\alpha }S_{j}^{\beta }$$ (2)

and a magnetic order $\langle S_{i}^{x}\rangle =\langle S_{i}^{y}\rangle =0,\langle S_{i}^{z}\rangle =S_{i}$ (written in a local frame by locally rotating the ordered moments to the *z* axis), one obtains the spin-wave dynamics by expanding *H* around the order for small spin fluctuations ($S_{i}^{x}$ and $S_{i}^{y}$). Omitting an irrelevant constant term and up to quadratic order in $S_{i}^{x}$ and $S_{i}^{y}$, the expansion yields

$$H=\frac{1}{2}\sum_{i,j}\sum_{\alpha ,\beta =x,y}S_{i}^{\alpha }R_{i\alpha ,j\beta }S_{j}^{\beta }$$ (3)

where the real, symmetric matrix *R* is required to be non-negative definite for the stability of the magnetic order. Rescaling the transverse variables by $S_{i}^{\alpha }\to S_{i}^{\alpha }/\sqrt{S_{i}}$, one obtains the commutation relations

$$[S_{i}^{\alpha },S_{j}^{\beta }]=-\delta_{i,j}{\left(\sigma_{y}\right)}^{\alpha ,\beta }$$ (4)

where σ*_y_* is the second Pauli matrix.

The Heisenberg equation of motion yields

$$-i\frac{dS_{i}^{\alpha }}{dt}=\sum_{j,\beta }{\left(Y\cdot R\right)}_{i\alpha ,j\beta }S_{j}$$ (5)

where we define the matrix *Y*_*i*α,*j*β_ = δ_*i*, *j*_(σ*_y_*)^α,β^, and the magnon spectrum is given by the eigenvalues of the non-Hermitian “dynamical matrix” *Y* · *R*.

The spin-wave system inherits all the symmetries of the exchange Hamiltonian as long as they preserve the magnetic order. Under any symmetry *g*, [*O_g_*,*R*] = 0, where *O_g_* ∈ *SO*(2 *N*). Being an imaginary matrix, note that the right-hand side of Eq. 4 acquires a minus sign under the action of an antiunitary symmetry *g*. This corresponds to flipping the handedness of the local spin frame by time reversal. Thus, *O_g_* anti-commutes (commutes) with *Y* for an antiunitary (unitary) symmetry *g*.

A similarity transformation of *Y* · *R*, defined as *H*_f_ = *R*^1/2^(*Y* · *R*)*R*^−1/2^, maps the dynamical matrix to a Hermitian problem (*21*, *22*). Note that *H*_f_ inherits the symmetry of the spin-wave system and its implementation with $H_{f}\to O_{g}H_{f}O_{g}^{†}$ for unitary *g* and $H_{f}\to -O_{g}H_{f}O_{g}^{†}=O_{g}H_{f}^{⋆}O_{g}^{†}$ for antiunitary *g*. Thus, the spin-wave problem and the Hermitian counterpart share the same band topology, and the implications of topological quantum chemistry and SIs carry over to the LSW problem.

In particular, the magnon system is mapped to a spinless (i.e., no spin-orbit coupling) electronic counterpart, and the spin-wave variables $S_{i}^{\alpha =x,y}$ pick up a +1 sign upon a 2π rotation or T^2^ (time-reversal squared). In addition, the “atomic orbitals” (at a given site *i*) from which the band representation can be induced are the site-symmetry group representation of the spin-wave variables $S_{i}^{\alpha =x,y}$. For all site-symmetry groups compatible with a magnetic order, this is always a direct sum of two 1D representations. The BCS MBANDREP tool (*12*, *20*) provides a complete tabulation of induced band representations.

### Symmetry constraints on exchange interactions induced by an electric field or strain

Now that an external magnetic field couples to the spin moment by a Zeeman term in the lowest order, here, we consider perturbations to the spin Hamiltonian induced by an external electric field and mechanical strains.

The electric field couples to the electric polarization operator in a material. Odd powers of spin operators violate time-reversal symmetry, and thus, the polarization operators ${\hat{P}}^{\alpha =x,y,z}$, which are time-reversal invariant, are bilinear in spins at the lowest order and read

$${\hat{P}}^{\alpha }=\frac{1}{2}\sum_{i,j}S_{i}^{T}P_{i,j}^{\alpha }S_{j}$$ (6)

where $S_{i}^{T}=(S_{i}^{x},S_{i}^{y},S_{i}^{z})$ is composed of the spin operators at site *i* and $P_{i,j}^{\alpha }$ are 3 × 3 matrices of exchange coefficients.

$P_{i,j}^{\alpha }$ are further constrained by the space group *G*. This is because the polarization is a vector operator, and under any symmetry *g* = {*O_g_*|*t_g_*} ∈ *G* [where g is composed of a proper/improper rotational part *O_g_* ∈ *O*(3) and a translation *t*], ${\hat{P}}^{\alpha }$ must transform like the components of a vector

$${\hat{P}}^{\alpha }\to O_{g}^{\alpha \alpha^{\prime }}{\hat{P}}^{\alpha^{\prime }}$$ (7)

On the other hand, the transformation of the right-hand side of Eq. 6 is

$$\frac{1}{2}\sum_{i,j}S_{i}^{T}P_{i,j}^{\alpha }S_{j}\to \frac{1}{2}S_{g^{-1}i}^{T}O_{g}^{T}P_{i,j}^{\alpha }O_{g}S_{g^{-1}j}$$ (8)

(Note that in the last equation, the spin operators should transform like a pseudo-vector rather than a vector. However, this distinction should not matter for the bilinear terms.)

Thus, $P_{i,j}^{\alpha }$ must satisfy the constraints

$$O_{g}^{\alpha \alpha^{\prime }}\sum_{i,j}S_{i}^{T}P_{i,j}^{\alpha^{\prime }}S_{j}=\sum_{i,j}S_{g^{-1}i}^{T}O_{g}^{T}P_{i,j}^{\alpha }O_{g}S_{g^{-1}j}\;$$ (9)

In practice, one can impose these constraints for a given exchange path *i* ↔ *j* by considering all the elements *g* ∈ *G* that leave sites *i* and *j* invariant (up to swapping the two sites). These elements impose constraints only on three matrices $P_{i,j}^{\alpha =x,y,z}$. Subsequently, for all other symmetry-related bonds *i*′ ↔ *j*′ (with *i*′ = *h*^−1^*i*, *j*′ = *h*^−1^*j*, *h* ∈ *G*), the corresponding matrices are obtained with

$$P_{i^{\prime },j^{\prime }}^{\alpha }=\sum_{\alpha^{\prime }}O_{h}^{\alpha^{\prime }\alpha }O_{h}^{T}P_{i,j}^{\alpha^{\prime }}O_{h}$$ (10)

Therefore, we have $H(E)=-E\cdot \hat{P}$ as the most general bilinear perturbation induced by an electric field ***E***.

A strain-induced perturbation for a given strain tensor σ^α,β = *x,y,z*^ reads

$$H(\{\sigma^{\alpha ,\beta }\})=\sum_{\alpha \beta }\sigma^{\alpha ,\beta }{\hat{\Sigma }}^{\alpha ,\beta }$$ (11)

where the operators ${\hat{\Sigma }}^{\alpha ,\beta }$ are time-reversal, as well as inversion invariant, and transform like a rank-two tensor under a proper rotation *O_g_* ∈ *SO*(3)

$${\hat{\Sigma }}^{\alpha ,\beta }\to O_{g}^{\alpha \alpha^{\prime }}O_{g}^{\beta \beta^{\prime }}{\hat{\Sigma }}^{\alpha^{\prime },\beta^{\prime }}$$ (12)

and at the bilinear level have the form

$${\hat{\Sigma }}^{\alpha ,\beta }=\frac{1}{2}\sum_{i,j}S_{i}^{T}\Sigma_{i,j}^{\alpha ,\beta }S_{j}$$ (13)

where the 3 × 3 matrices $\Sigma_{i,j}^{\alpha ,\beta }$ determine the exchange coefficients. Analogous to the electric field perturbation above, these matrices must satisfy

$$O_{g}^{\alpha \alpha^{\prime }}O_{g}^{\beta \beta^{\prime }}\sum_{i,j}S_{i}^{T}\sigma_{i,j}^{\alpha^{\prime },\beta^{\prime }}S_{j}=\sum_{i,j}S_{g^{-1}i}^{T}O_{g}^{T}\Sigma_{i,j}^{\alpha ,\beta }O_{g}S_{g^{-1}j}$$ (14)

for any *g* ∈ *G*.

## Supplementary Materials

### This PDF file includes:

Sections S1 to S7 Figs. S1 to S14 Tables S1 to S1663 References
