# Supplementary material for: An efficient material search for room-temperature topological magnons
Source: Sci Adv. 2023 Feb 17;9(7):eade7731. doi: 10.1126/sciadv.ade7731 (PMC9937570; doi:10.1126/sciadv.ade7731)
Supplement: Supplementary file 1 — Sections S1 to S7 Figs. S1 to S14 Tables S1 to S1663 References [file sciadv.ade7731_sm.pdf]

Science Advances
AAAS

Supplementary Materials for

An efficient material search for room-temperature topological magnons

Mohammed J. Karaki *et al.*

Corresponding author: Mohammed J. Karaki, karaki.4@osu.edu; Yuan-Ming Lu, lu.1435@osu.edu

Sci. Adv. **9**, eade7731 (2023)
DOI: 10.1126/sciadv.ade7731

This PDF file includes:

Sections S1 to S7
Figs. S1 to S14
Tables S1 to S1663
References

Section S1. Spin-wave details for topological magnon candidates

In this section we provide details about the spin-wave calculations for the systems mentioned in the main text. For each material, we describe the crystalline/magnetic structure and symmetries, the most generic symmetry-constrained exchange interactions for the first few nearest-neighbor paths, the little group irreducible representation (irrep) content of the induced magnon bands (and thus the symmetry-enforced degeneracies) at the high-symmetry k-points, and the effects of external perturbations on the magnetic space group symmetry as well as on the magnon spectrum, including the SI-based prediction of topological bands.

For each system, we use $\boldsymbol{r}_s$ to denote the position of magnetic sublattice $s = \mathrm{A}, \mathrm{B}, \cdots$, within a structural unit cell. Using 3 integers $\{x_i\}$, we label each lattice site with the coordinates (x_1, x_2, x_3, s), and its position is given by

$$\boldsymbol{R}(x_1, x_2, x_3, s) = \sum_{i=1}^{3} x_i \boldsymbol{a}_i + \boldsymbol{r}_s, \tag{S1}$$

where $\boldsymbol{a}_1$, $\boldsymbol{a}_2$ and $\boldsymbol{a}_3$ are the primitive Bravais translations of the crystal.

A general interaction Hamiltonian bilinear in spins reads

$$H = \frac{1}{2} \sum_{\boldsymbol{R}_1, \boldsymbol{R}_2} \sum_{\alpha, \beta} \mathsf{J}^{\alpha,\beta}_{\boldsymbol{R}_1, \boldsymbol{R}_2} S^{\alpha}_{\boldsymbol{R}_1} S^{\beta}_{\boldsymbol{R}_2}, \tag{S2}$$

where $\mathsf{J}_{\boldsymbol{R}_1,\boldsymbol{R}_2} = [\mathsf{J}_{\boldsymbol{R}_2,\boldsymbol{R}_1}]^T$ is a 3×3 real matrix of exchange couplings. For example, a Heisenberg interaction corresponds to an exchange matrix proportional to the identity. A general exchange matrix J can be decomposed as

$$\mathsf{J} = \begin{pmatrix} J & \Gamma^{xy} + D^z & \Gamma^{xz} - D^y \\ \Gamma^{xy} - D^z & J + J^y & \Gamma^{yz} + D^x \\ \Gamma^{xz} + D^y & \Gamma^{yz} - D^x & J + J^z \end{pmatrix}, \tag{S3}$$

where the parameters $\Gamma^{\alpha,\beta}$ (D^{α}) describe the symmetric (anti-symmetric) exchange. In particular, D^{α} correspond to the Dzyaloshinskii–Moriya interaction, of the form $\boldsymbol{D} \cdot \boldsymbol{S}_1 \times \boldsymbol{S}_2$.

Our conventions for labelling the Brillouin zone k-points as well as the band irreps follow the notation used in the Bilbao Crystallographic Server (BCS) tables ([20], [12]). In this notation, a band irrep label at a given k-point always contains the k-point label. For example, in $P4/mmm$ the irreps at $k = X$ are labelled $X_1^+, X_1^-, X_2^+, \cdots$.

Note that in two magnetic space groups $G_2 \subset G_1$, the label of the same k-point is in general different. To maintain consistency with the BCS tables of little group irreps, we choose to switch our labelling accordingly when we consider symmetry-lowering perturbations. In particular, plots illustrating the effects of external fields on the magnon spectrum are affected by this relabelling.

S1.1 $TbFeO_3$

S1.1.1 The spin model

The crystal structure of $TbFeO_3$ is described by the orthorhombic space group $Pnma$ (62), and the primitive unit cell hosts 4 Fe atoms. In Cartesian coordinates, the primitive lattice vectors are

$$\boldsymbol{a}_1 = (a, 0, 0), \qquad \boldsymbol{a}_2 = (0, b, 0), \qquad \boldsymbol{a}_3 = (0, 0, c). \tag{S4}$$

The four Fe sublattices are coordinated at

$$\begin{aligned} \boldsymbol{r}_{\mathrm{A}} &= (0, 0, c/2), \\ \boldsymbol{r}_{\mathrm{B}} &= (a/2, b/2, 0), \\ \boldsymbol{r}_{\mathrm{C}} &= (0, b/2, c/2), \\ \boldsymbol{r}_{\mathrm{D}} &= (a/2, 0, 0). \end{aligned}$$

The space group $Pnma$ is generated by

- $G_{100}^{\|\boldsymbol{a}_2+\boldsymbol{a}_3}$, glide plane $\boldsymbol{a_1}/4 + y\boldsymbol{a}_2 + z\boldsymbol{a}_3$ with a fractional translation $\boldsymbol{a}_2/2 + \boldsymbol{a}_3/2$. The

Fe atoms transform as

$$(x_1, x_2, x_3, s) \longrightarrow \begin{cases} (-x_1, x_2, 1+x_3, \mathrm{B}), & s = \mathrm{A} \\ (-x_1, 1+x_2, x_3, \mathrm{A}), & s = \mathrm{B} \\ (-x_1, 1+x_2, 1+x_3, \mathrm{D}), & s = \mathrm{C} \\ (-x_1, x_2, x_3, \mathrm{C}), & s = \mathrm{D} \end{cases}$$

- M_{010}, mirror plane $x\boldsymbol{a_1} + \boldsymbol{a}_2/4 + z\boldsymbol{a}_3$:

$$(x_1, x_2, x_3, s) \longrightarrow \begin{cases} (x_1, -x_2, x_3, \mathrm{C}), & s = \mathrm{A} \\ (x_1, -x_2, x_3, \mathrm{D}), & s = \mathrm{B} \\ (x_1, -x_2, x_3, \mathrm{A}), & s = \mathrm{C} \\ (x_1, -x_2, x_3, \mathrm{B}), & s = \mathrm{D} \end{cases}$$

- $G_{001}^{\|\boldsymbol{a}_1}$, glide plane $x\boldsymbol{a}_1 + y\boldsymbol{a}_2 + \boldsymbol{a}_3/4$ with a fractional translation $\boldsymbol{a}_1/2$:

$$(x_1, x_2, x_3, s) \longrightarrow \begin{cases} (x_1, x_2, -x_3, \mathrm{D}), & s = \mathrm{A} \\ (1+x_1, x_2, -x_3, \mathrm{C}), & s = \mathrm{B} \\ (x_1, x_2, -x_3, \mathrm{B}), & s = \mathrm{C} \\ (1+x_1, x_2, -x_3, \mathrm{A}), & s = \mathrm{D} \end{cases}$$

The 1st nearest-neighbor (NN) exchange coupling

$$\mathsf{J}_1 : (0,0,0,\mathrm{A}) \leftrightarrow (0,0,0,\mathrm{C}) \tag{S5}$$

is constrained only by the mirror symmetry M_{010}, and it has the following generic form

$$\mathsf{J}_1 = \begin{pmatrix} J_1 & D_1^z & \Gamma_1^{xz} \\ -D_1^z & J_1 + J_1^y & D_1^x \\ \Gamma_1^{xz} & -D_1^x & J_1 + J_1^z \end{pmatrix} \tag{S6}$$

The 2nd NN exchange coupling is

$$\mathsf{J}_2 : (0,0,0,\mathrm{A}) \leftrightarrow (0,0,0,\mathrm{D}) \tag{S7}$$

Having no symmetry constraints, it is an arbitrary 3×3 matrix,

$$\mathsf{J}_2 = \begin{pmatrix} J_2 & \Gamma_2^{xy} + D_2^z & \Gamma_2^{xz} - D_2^y \\ \Gamma_2^{xy} - D_2^z & J_2 + J_2^y & \Gamma_2^{yz} + D_2^x \\ \Gamma_2^{xz} + D_2^y & \Gamma_2^{yz} - D_2^x & J_2 + J_2^z \end{pmatrix} \tag{S8}$$

The 3rd NN exchange coupling is

$$\mathsf{J}_3 : (0,0,0,\mathrm{D}) \leftrightarrow (0,0,1,\mathrm{D}) \tag{S9}$$

which is constrained only by inversion symmetry, and thus it is a symmetric matrix,

$$\mathsf{J}_3 = \begin{pmatrix} J_3 & \Gamma_3^{xy} & \Gamma_3^{xz} \\ \Gamma_3^{xy} & J_3 + J_3^y & \Gamma_3^{yz} \\ \Gamma_3^{xz} & \Gamma_3^{yz} & J_3 + J_3^z \end{pmatrix} \tag{S10}$$

The 4th NN exchange coupling is

$$\mathsf{J}_4 : (0,0,0,\mathrm{A}) \leftrightarrow (0,0,0,\mathrm{B}) \tag{S11}$$

and is subject to no constraints,

$$\mathsf{J}_4 = \begin{pmatrix} J_4 & \Gamma_4^{xy} + D_4^z & \Gamma_4^{xz} - D_4^y \\ \Gamma_4^{xy} - D_4^z & J_4 + J_4^y & \Gamma_4^{yz} + D_4^x \\ \Gamma_4^{xz} + D_4^y & \Gamma_4^{yz} - D_4^x & J_4 + J_4^z \end{pmatrix} \tag{S12}$$

S1.1.2 Linear spin-wave theory and field-induced magnon topology

At a transition temperature $T_\mathrm{N} = 681\,\mathrm{K}$, the Fe atoms order antiferromagnetically as shown in Fig. S 1. The moments of the A- and B-sublattices are parallel to z-axis, whereas the C- and D-sublattices point in the opposite direction:

$$\langle \boldsymbol{S}(x_i, s)\rangle \propto \begin{cases} (0,0,+1) & s = \text{A or B} \\ (0,0,-1) & s = \text{C or D} \end{cases} \tag{S13}$$

The magnetic order is invariant under the mirror symmetry M_{010} but it breaks both glide symmetries, $G_{100}^{\|\boldsymbol{a}_2+\boldsymbol{a}_3}$ and $G_{001}^{\|\boldsymbol{a}_1}$. However, the combination of time-reversal with $G_{100}^{\|\boldsymbol{a}_2+\boldsymbol{a}_3}$ or $G_{001}^{\|\boldsymbol{a}_1}$ still preserves the magnetic order. Thus, the magnetic space group is $Pn'ma'$ (62.448), with magnetic Bravais translations

$$\boldsymbol{a} = \boldsymbol{a}_1, \qquad \boldsymbol{b} = \boldsymbol{a}_2, \qquad \boldsymbol{c} = \boldsymbol{a}_3, \tag{S14}$$

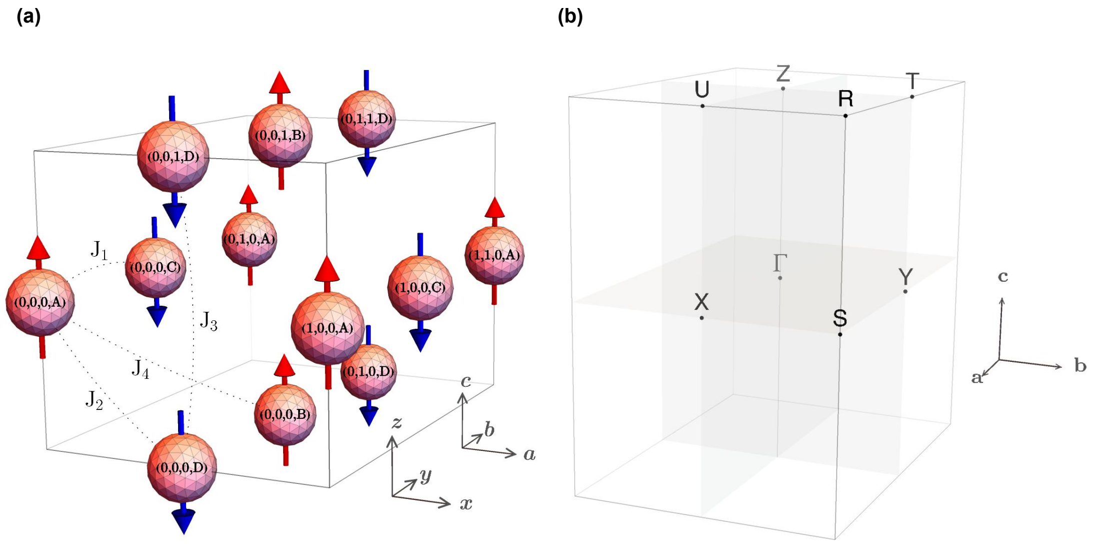

Fig. S 1: **The magnetic structure of $TbFeO_3$. a**, The magnetic unit cell with with four nearest-neighbor exchange paths illustrated. **b**, The magnetic Brillouin zone.

and the Brillouin zone is shown in Fig. S 1.

As mentioned in the main text, to study the field-induced topology of magnon bands, our starting point is identify symmetry-protected degeneracies.

Fe^{3+} ions occupy the $4b$ Wyckoff position of $Pn'ma'$, and the site-symmetry group is $\bar{1}$. The spin-wave variables transform trivially under this group, and therefore they decompose into two copies of the trivial 1D irrep A_g (see Table. S 9 for the spin-wave representation in all magnetic point groups.) Using the MBANDREP tool on the Bilbao Crystallographic Server, one obtains the band representation (Table. S 1).

To study the field-induced topology of magnon bands, we start from protected degeneracies. From Table. S 1, we find that the bands are four-fold degenerate at both R and S. This degeneracy is partially protected by $\tilde{T} \cdot G_{001}^{||\boldsymbol{a}_1}$, a combination of time-reversal $\tilde{T}$ and the glide symmetry $G_{001}^{||\boldsymbol{a}_1}$. This anti-unitary symmetry squares to -1 at R and S, resulting in effective Kramers pairs.

k point	$A_g \uparrow$ MSG $\downarrow$ Little Group
$\Gamma(0,0,0)$	$2\Gamma_1^+(1) \oplus 2\Gamma_2^+(1)$
$R(\frac{1}{2},\frac{1}{2},\frac{1}{2})$	$R_1R_1(4)$
$S(\frac{1}{2},\frac{1}{2},0)$	$S_1S_1(4)$
$T(0,\frac{1}{2},\frac{1}{2})$	$2T_1(2)$
$U(\frac{1}{2},0,\frac{1}{2})$	$U_1^-U_1^-(2) \oplus U_2^-U_2^-(2)$
$X(\frac{1}{2},0,0)$	$X_1^+X_2^-(2) \oplus X_1^-X_2^+(2)$
$Y(0,\frac{1}{2},0)$	$2Y_1(2)$
$Z(0,0,\frac{1}{2})$	$Z_1^+Z_2^-(2) \oplus Z_1^-Z_2^+(2)$

Table. S 1: Band representation induced from the A_g irrep of $\bar{1}$, the site-symmetry group of the $4b$ Wyckoff position in $Pn'ma'$. The magnon band representation in $TbFeO_3$ is induced from two copies of A_g (see Table. S 9.) The integers in parentheses indicate the dimensionality of an irrep. The $\pm$ superscripts indicate the inversion eigenvalue.

Next, we seek to isolate the bands by reducing the magnetic space group symmetry and evaluate the SIs of the separated bands. Using the subgroup tables in the auxiliary supplementary materials, we find that an external magnetic field along the $\boldsymbol{c}$ axis reduces the symmetry

$$Pn'ma' \ (62.448) \xrightarrow{\boldsymbol{B}\|[001]} P2_1'/c' \ (14.79) \tag{S15}$$

into a subgroup with a non-trivial classification $\mathbb{Z}_2 \times \mathbb{Z}_4$, in which $\mathbb{Z}_4$ is the inversion indicator. The monoclinic axes of the subgroup $P2_1'/c'$ are obtained from the orthorhombic axes of $Pn'ma'$ by

$$\begin{pmatrix} \boldsymbol{a} \\ \boldsymbol{b} \\ \boldsymbol{c} \end{pmatrix} \to \begin{pmatrix} & & 1 \\ 1 & & \\ & 1 & -1 \end{pmatrix} \begin{pmatrix} \boldsymbol{a} \\ \boldsymbol{b} \\ \boldsymbol{c} \end{pmatrix} \tag{S16}$$

Fig. 1c-d show the magnon spectrum before and after an applied field, using parameters $J_1 = 1.00$, $J_2 = 0.44$, $J_4 = -0.30$, $J_1^y = -0.19$, $J_1^z = 0.32$, $J_2^y = -0.21$, $\Gamma_2^{xy} = 0.10$, $\Gamma_4^{xy} = 0.05$, $\Gamma_6^{xy} = 0.04$ and magnetic field $h = 0.5$. The separated bands acquire an odd value of $\mathbb{Z}_4$, signaling Weyl magnons in the bulk. This is because the upper two bands started with an even number of inversion-odd eigenvalues at the gapped TRIM points without an external field,

k-label ($Pn'ma'$)	Effect of $\boldsymbol{B} \parallel [001]$ on irreps	Contribution to $\mathbb{Z}_4$ of higher two bands
R	$R_1R_1 \longrightarrow C_1^-C_1^+ \oplus C_1^-C_1^+$	1 (always)
S	$S_1S_1 \longrightarrow D_1^+D_1^+ \oplus D_1^-D_1^-$	0 or 2

Table. S 2: Effect of a magnetic field perturbation along the $\boldsymbol{c}$ axis of $Pn'ma'$ on the four-fold magnon band degeneracies at $k = R$ and S, as well as on the $\mathbb{Z}_4$ inversion SI.

and the field-induced degeneracy splitting (at R and S) can only change this count by an odd number, see Table. S 2.

This conclusion resulted purely from symmetry, and it is valid for generic spin models and any perturbation leading to the same subgroup $P2_1'/c'$, such as a uniaxial strain in the $\boldsymbol{b} - \boldsymbol{c}$ plane.

S1.2 α-Fe_2O_3

S1.2.1 The spin model

The crystal structure of α-Fe_2O_3 is described by the trigonal space group $R\bar{3}c$ (167), and its primitive rhombohedral lattice vectors can be chosen as

$$\begin{pmatrix} \boldsymbol{a}_1 \\ \boldsymbol{a}_2 \\ \boldsymbol{a}_3 \end{pmatrix} = \frac{1}{3} \begin{pmatrix} 2 & 1 & 1 \\ -1 & 1 & 1 \\ -1 & -2 & 1 \end{pmatrix} \begin{pmatrix} \boldsymbol{a} \\ \boldsymbol{b} \\ \boldsymbol{c} \end{pmatrix}, \tag{S17}$$

where

$$\boldsymbol{a} = a(1,0,0), \qquad \boldsymbol{b} = \frac{a}{2}(-1,\sqrt{3},0), \qquad \boldsymbol{c} = c(0,0,1). \tag{S18}$$

are the conventional hexagonal axes. This choice leads to a simple transformation under a 3-fold rotation:

$$\boldsymbol{a}_1 \to \boldsymbol{a}_2 \to \boldsymbol{a}_3 \to \boldsymbol{a}_1. \tag{S19}$$

With $\alpha \approx 0.355322$, the four Fe^{3+} ions are coordinated at

$$\begin{aligned}
\boldsymbol{r}_{\mathrm{A}} &= \alpha(\boldsymbol{a}_1 + \boldsymbol{a}_2 + \boldsymbol{a}_3), \\
\boldsymbol{r}_{\mathrm{B}} &= \left(\frac{1}{2} - \alpha\right)(\boldsymbol{a}_1 + \boldsymbol{a}_2 + \boldsymbol{a}_3), \\
\boldsymbol{r}_{\mathrm{C}} &= (1 - \alpha)(\boldsymbol{a}_1 + \boldsymbol{a}_2 + \boldsymbol{a}_3), \\
\boldsymbol{r}_{\mathrm{D}} &= \left(\frac{1}{2} + \alpha\right)(\boldsymbol{a}_1 + \boldsymbol{a}_2 + \boldsymbol{a}_3).
\end{aligned}$$

The space group $R\bar{3}c$ is generated by

- i: inversion about the cell center $(\boldsymbol{a}_1 + \boldsymbol{a}_2 + \boldsymbol{a}_3)/2$. This swaps $\mathrm{A} \leftrightarrow \mathrm{C}$ and $\mathrm{B} \leftrightarrow \mathrm{D}$ within the unit cell.

- 3_{001}: a three-fold axis along $\boldsymbol{c} = (\boldsymbol{a}_1 + \boldsymbol{a}_2 + \boldsymbol{a}_3)$:

$$(x_1, x_2, x_3, s) \longrightarrow \begin{cases} (x_3, x_1, x_2, \mathrm{A}), & s = \mathrm{A} \\ (x_3, x_1, x_2, \mathrm{B}), & s = \mathrm{B} \\ (x_3, x_1, x_2, \mathrm{C}), & s = \mathrm{C} \\ (x_3, x_1, x_2, \mathrm{D}), & s = \mathrm{D} \end{cases}$$

- $G_{100}^{\|\boldsymbol{c}}$: a glide plane $x(\boldsymbol{a_1} + \boldsymbol{a}_2) + z\boldsymbol{a}_3$, with a fractional translation $(\boldsymbol{a}_1 + \boldsymbol{a}_2 + \boldsymbol{a}_3)/2$:

$$(x_1, x_2, x_3, s) \longrightarrow \begin{cases} (x_2, x_1, x_3, \mathrm{D}), & s = \mathrm{A} \\ (x_2, x_1, x_3, \mathrm{C}), & s = \mathrm{B} \\ (1 + x_2, 1 + x_1, 1 + x_3, \mathrm{B}), & s = \mathrm{C} \\ (1 + x_2, 1 + x_1, 1 + x_3, \mathrm{A}), & s = \mathrm{D} \end{cases}$$

We consider the exchange paths for the first 10 nearest neighbor bonds:

$$\mathsf{J}_1 : (0,0,0,\mathrm{A}) \leftrightarrow (0,0,0,\mathrm{B}) \tag{S20}$$

$$\mathsf{J}_2 : (0,0,0,\mathrm{A}) \leftrightarrow (0,0,-1,\mathrm{C}) \tag{S21}$$

$$\mathsf{J}_3 : (0,0,0,\mathrm{C}) \leftrightarrow (0,0,-1,\mathrm{D}) \tag{S22}$$

$$\mathsf{J}_4 : (0,0,0,\mathrm{A}) \leftrightarrow (0,0,-1,\mathrm{D}) \tag{S23}$$

$$\mathsf{J}_5 : (0,0,0,\mathrm{A}) \leftrightarrow (0,0,0,\mathrm{C}) \tag{S24}$$

$$\mathsf{J}_6 : (0,0,0,\mathrm{A}) \leftrightarrow (0,-1,1,\mathrm{A}) \tag{S25}$$

$$\mathsf{J}_7 : (0,0,0,\mathrm{A}) \leftrightarrow (0,0,-1,\mathrm{A}) \tag{S26}$$

$$\mathsf{J}_8 : (0,0,0,\mathrm{A}) \leftrightarrow (0,-1,1,\mathrm{B}) \tag{S27}$$

$$\mathsf{J}_9 : (0,0,0,\mathrm{A}) \leftrightarrow (-1,-1,1,\mathrm{C}) \tag{S28}$$

$$\mathsf{J}_{10} : (0,0,0,\mathrm{A}) \leftrightarrow (0,-1,-1,\mathrm{C}) \tag{S29}$$

The 1st NN exchange is constrained by 3-fold and 2-fold axes, parallel and perpendicular to the bond axis, respectively. The 2nd NN bond is invariant only under inversion, and the exchange matrix is symmetric. The 3rd NN exchange is constrianed by a 2-fold axis perpendicular to the bond. The 4th NN exchange is subject to no constraints. Finally, the 5th NN bond is constrained by inversion and a 3-fold axis.

The following is the generic form of the first five exchange matrices.

$$\mathsf{J}_1 = \begin{pmatrix} J_1 & +D_1^z & \\ -D_1^z & J_1 & \\ & & J_1 + J_1^z \end{pmatrix} \qquad \mathsf{J}_2 = \begin{pmatrix} J_2 & \Gamma_2^{xy} & \Gamma_2^{xz} \\ \Gamma_2^{xy} & J_2 + J_2^y & \Gamma_2^{yz} \\ \Gamma_2^{xz} & \Gamma_2^{yz} & J_2 + J_2^z \end{pmatrix} \tag{S30}$$

$$\mathsf{J}_3 = \begin{pmatrix} J_3 & +D_3^z & -D_3^y \\ -D_3^z & J_3 + J_3^y & \Gamma_3^{yz} \\ +D_3^y & \Gamma_3^{yz} & J_3 + J_3^z \end{pmatrix} \qquad \mathsf{J}_4 = \begin{pmatrix} J_4 & \Gamma_4^{xy} + D_4^z & \Gamma_4^{xz} - D_4^y \\ \Gamma_4^{xy} - D_4^z & J_4 + J_4^y & \Gamma_4^{yz} + D_4^x \\ \Gamma_4^{xz} + D_4^y & \Gamma_4^{yz} - D_4^x & J_4 + J_4^z \end{pmatrix} \tag{S31}$$

$$\mathsf{J}_5 = \begin{pmatrix} J_5 & & \\ & J_5 & \\ & & J_5 + J_5^z \end{pmatrix} \tag{S32}$$

S1.2.2 Linear spin-wave spectrum and topology

The magnetic order is collinear AFM with magnetic moments along $\boldsymbol{a} = \boldsymbol{a}_1 - \boldsymbol{a}_2 \propto (1, 0, 0)$, with $T_{\mathrm{N}} = 955\,\mathrm{K}$. More precisely,

$$\langle \boldsymbol{S}(x_i, s) \rangle \propto \begin{cases} (+1, 0, 0) & s = \text{A or C} \\ (-1, 0, 0) & s = \text{B or D} \end{cases} \tag{S33}$$

This is illustrated in Fig. S 2.

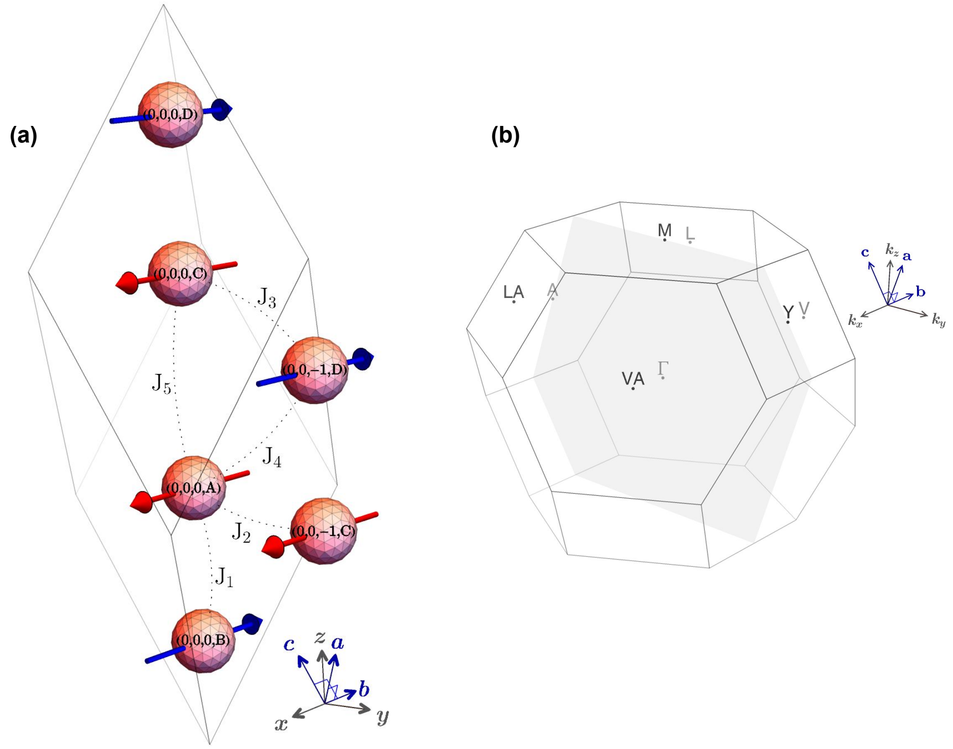

Fig. S 2: **The magnetic structure of Fe_2O_3.** **a**, The magnetic unit cell with with five nearest-neighbor exchange paths illustrated. **b**, The magnetic Brillouin zone.

The magnetic order preserves inversion, an anti-unitary 2-fold axis and an anti-unitary glide. The magnetic space group is $C2'/c'$ (15.89), whose conventional monoclinic axes are

$$\boldsymbol{a}_m = \boldsymbol{a}_1 + \boldsymbol{a}_2, \quad \boldsymbol{b}_m = -\boldsymbol{a}_1 + \boldsymbol{a}_2, \quad \boldsymbol{c}_m = \boldsymbol{a}_3, \tag{S34}$$

with $\boldsymbol{b}_m$ along the 2-fold axis of $C2'/m'$. Here, we use the subscript m to distinguish these magnetic lattice axes from those of the parent hexagonal lattice.

The four magnetic Fe sublattices occupy the $8f$ Wyckoff position of $C2'/c'$, which has a trivial site-symmetry group, and thus the spin-wave variables transform trivially on-site. Table. S 3 summarizes the induced magnon band representation, restricted to the little groups of high-symmetry k-points.

k-point	$A \uparrow$ MSG $\downarrow$ Little Group
$\Gamma(0,0,0)$	$2\Gamma_1^+(1) \oplus 2\Gamma_1^-(1)$
$A(0,0,\frac{1}{2})$	$2A_1^-A_1^+(2)$
$L(\frac{1}{2},\frac{1}{2},\frac{1}{2})$	$2L_1^+(1) \oplus 2L_1^-(1)$
$LA(\frac{1}{2},-\frac{1}{2},\frac{1}{2})$	$2LA_1^-(1) \oplus 2LA_1^+(1)$
$M(0,1,\frac{1}{2})$	$2M_1^-M_1^+(2)$
$V(\frac{1}{2},\frac{1}{2},0)$	$2V_1^+(1) \oplus 2V_1^-(1)$
$VA(\frac{1}{2},-\frac{1}{2},0)$	$2VA_1^+(1) \oplus 2VA_1^-(1)$
$Y(0,1,0)$	$2Y_1^+(1) \oplus 2Y_1^-(1)$

Table. S 3: Band representation induced from the A irrep of 1, the site-symmetry group of the $8f$ Wyckoff position in $C2'/c'$. The magnon band representation in α-Fe_2O_3 is induced from two copies of A (see Table. S 9.)

According to Table. S 3, the highest degeneracy at the inversion-symmetric points is two-fold, and therefore we expect two pairs of bands that are energetically isolated at least at the high-symmetry points, for a generic Hamiltonian. It turns out that 2nd NN Heisenberg term J_2 alone is sufficient open up this gap.

Respectively, J_1 through J_{10} have the following experimental estimates (*33*) in meV units: -1.03, -0.28, 5.12, 4.00, 0.17, -0.09, 0.02, 0.05, 0.02 and 0.00. We supplement this with an on-site anisotropy $J_0^{zz} = 0.12$ meV, a symmetric interaction $\Gamma_3^{yz} = 0.10$ meV and an antisymmetric term $D_3^y = 0.05$ meV to produce the spectrum in Figure 3(c). Finally, the perturbed spectrum in Figure 3(d) corresponds to magnetic field $B = 2.5$ T and $g = 2$.

S1.3 CoF_3

S1.3.1 The spin model

The crystal symmetry is described by the trigonal space group $R\bar{3}c$ (167), and below we work with a rhombohedral primitive cell, and Bravais translations

$$\begin{pmatrix} \boldsymbol{a}_1 \\ \boldsymbol{a}_2 \\ \boldsymbol{a}_3 \end{pmatrix} = \frac{1}{3} \begin{pmatrix} 2 & 1 & 1 \\ -1 & 1 & 1 \\ -1 & -2 & 1 \end{pmatrix} \begin{pmatrix} \boldsymbol{a} \\ \boldsymbol{b} \\ \boldsymbol{c} \end{pmatrix}, \tag{S35}$$

where

$$\boldsymbol{a} = \frac{a}{\sqrt{2}}(0,1,-1), \qquad \boldsymbol{b} = \frac{a}{\sqrt{2}}(-1,0,1), \qquad \boldsymbol{c} = \frac{c}{\sqrt{3}}(1,1,1). \tag{S36}$$

are the conventional hexagonal axes. The two Co atoms are located on the 3-fold axis. Specifically

$$\begin{aligned} \boldsymbol{r}_{\mathrm{A}} &= (0,0,0), \\ \boldsymbol{r}_{\mathrm{B}} &= \frac{1}{2}(\boldsymbol{a}_1 + \boldsymbol{a}_2 + \boldsymbol{a}_3). \end{aligned}$$

We already discussed the generators of $R\bar{3}c$ in Sec. S1.2, and the following describes how they transform Co lattice sites.

- Under cell-centered inversion i,

$$(x_1, x_2, x_3, s) \longrightarrow \begin{cases} (1-x_1, 1-x_2, 1-x_3, \mathrm{A}), & s = \mathrm{A} \\ (-x_1, -x_2, -x_3, \mathrm{B}), & s = \mathrm{B} \end{cases}$$

- Under three-fold axis $3_{\boldsymbol{c}}$,

$$(x_1, x_2, x_3, s) \longrightarrow \begin{cases} (x_3, x_1, x_2, \mathrm{A}), & s = \mathrm{A} \\ (x_3, x_1, x_2, \mathrm{B}), & s = \mathrm{B} \end{cases}$$

- Under glide $G^{\parallel \boldsymbol{c}}_{\perp \boldsymbol{a}}$,

$$(x_1, x_2, x_3, s) \longrightarrow \begin{cases} (x_2, x_1, x_3, \mathrm{B}), & s = \mathrm{A} \\ (1+x_2, 1+x_1, 1+x_3, \mathrm{A}), & s = \mathrm{B} \end{cases}$$

The three nearest-neighbor exchange paths

$$\mathsf{J}_1 : (1,0,0,\mathrm{A}) \leftrightarrow (0,0,0,\mathrm{B}) \tag{S37}$$

$$\mathsf{J}_2 : (1,0,0,\mathrm{A}) \leftrightarrow (0,1,0,\mathrm{A}) \tag{S38}$$

$$\mathsf{J}_3 : (0,0,0,\mathrm{B}) \leftrightarrow (1,0,0,\mathrm{B}) \tag{S39}$$

have the generic form

$$\mathsf{J}_1 = \begin{pmatrix} J_1 & \Gamma_1^{xy} + D_1^z & \Gamma_1^{xz} - D_1^y \\ \Gamma_1^{xy} - D_1^z & J_1 + J_1^y & \Gamma_1^{xy} + D_1^z \\ \Gamma_1^{xz} + D_1^y & \Gamma_1^{xy} - D_1^z & J_1 \end{pmatrix} \tag{S40}$$

$$\mathsf{J}_2 = \begin{pmatrix} J_2 & \Gamma_2^{xy} & \Gamma_2^{xz} \\ \Gamma_2^{xy} & J_2 + J_2^y & \Gamma_2^{yz} \\ \Gamma_2^{xz} & \Gamma_2^{yz} & J_2 + J_2^z \end{pmatrix} \tag{S41}$$

$$\mathsf{J}_3 = \begin{pmatrix} J_3 & \Gamma_3^{xy} & \Gamma_3^{xz} \\ \Gamma_3^{xy} & J_3 + J_3^y & \Gamma_3^{yz} \\ \Gamma_3^{xz} & \Gamma_3^{yz} & J_3 + J_3^z \end{pmatrix} \tag{S42}$$

k point	${}^1E_g \uparrow \text{MSG} \downarrow G_{\boldsymbol{k}}$	${}^1E_g \uparrow \text{MSG} \downarrow G_{\boldsymbol{k}}$
$\Gamma(0,0,0)$	$\Gamma_3^+(2)$	$\Gamma_3^+(2)$
$F(0,\frac{1}{2},1)$	$F_1^+(1) \oplus F_2^+(1)$	$F_1^+(1) \oplus F_2^+(1)$
$L(-\frac{1}{2},\frac{1}{2},\frac{1}{2})$	$L_1(2)$	$L_1(2)$
$T(0,0,\frac{3}{2})$	$T_1(2)$	$T_2(2)$

Table. S 4: Band representation induced from the ${}^1E_g$ and ${}^2E_g$ irreps of $\bar{3}$, the site-symmetry group of the $6b$ Wyckoff position in $R\bar{3}c$. The magnon band representation in CoF_3 is induced from ${}^1E_g \oplus {}^2E_g$ (see Table. S 9.)

Additionally, we consider interactions induced by mechanical strain. With $\sigma_{\alpha,\beta}$ denoting the components of the strain matrix $\sigma = \sigma^T$, the generic form of the 2nd NN Heisenberg interactions

induced by strain reads

$$H' = \sum_{\boldsymbol{x}} \Bigg\{ \tag{S43}$$

$$\left(\vec{S}_{\boldsymbol{x}+\boldsymbol{a}_1,\mathrm{A}} \cdot \vec{S}_{\boldsymbol{x}+\boldsymbol{a}_2,\mathrm{A}} + [\mathrm{A} \leftrightarrow \mathrm{B}]\right)\left(\alpha_1\sigma_{xx} + \alpha_2\sigma_{yy} + \alpha_3\sigma_{zz} + \beta_1\sigma_{xy} + \beta_2\sigma_{yz} + \beta_3\sigma_{zx}\right) \tag{S44}$$

$$+\left(\vec{S}_{\boldsymbol{x}+\boldsymbol{a}_2,\mathrm{A}} \cdot \vec{S}_{\boldsymbol{x}+\boldsymbol{a}_3,\mathrm{A}} + [\mathrm{A} \leftrightarrow \mathrm{B}]\right)\left(\alpha_1\sigma_{yy} + \alpha_2\sigma_{zz} + \alpha_3\sigma_{xx} + \beta_1\sigma_{yz} + \beta_2\sigma_{zx} + \beta_3\sigma_{xy}\right) \tag{S45}$$

$$+\left(\vec{S}_{\boldsymbol{x}+\boldsymbol{a}_3,\mathrm{A}} \cdot \vec{S}_{\boldsymbol{x}+\boldsymbol{a}_1,\mathrm{A}} + [\mathrm{A} \leftrightarrow \mathrm{B}]\right)\left(\alpha_1\sigma_{zz} + \alpha_2\sigma_{xx} + \alpha_3\sigma_{yy} + \beta_1\sigma_{zx} + \beta_2\sigma_{xy} + \beta_3\sigma_{yz}\right)\Bigg\}. \tag{S46}$$

For the strain perturbation we consider below (uniaxial || [110]), the 1NN Heisenberg terms do not lift any degeneracy at the TRIM points, and we do not include their explicit form here.

S1.3.2 Spin-wave spectrum and topology

The AFM order of CoF_3 is illustrated in Fig. S 3a, and it preserves the entire space group. The magnetic space group is thus $R\bar{3}c$ (167.103).

The magnetic atoms occupy the $6b$ Wyckoff position, and the spin-wave variables transform in the ${}^1E_g \oplus {}^2E_g$ representation of the site-symmetry group $\bar{3}$, i.e. two inversion-even irreps with $C_3 = e^{\pm 2\pi/3}$. The band representations induced from ${}^1E_g$ and ${}^2E_g$ are summarized in Table. S 4. Inspecting the irrep dimensions, we find that the two magnon bands are connected at four TRIM points (the k-stars of Γ, L and T). According to the subgroup tables (see the auxiliary supplementary materials), all magnetic subgroups with a non-trivial classification are centrosymmetric, and thus we need only consider magnetic field and strain perturbations to lift these degeneracies. Now we describe how Weyl magnons are induced by a combination of these perturbations.

First, the application of uniaxial strain in the $[110]$ direction breaks down $R\bar{3}c \to C2/c$ (15.85)

with conventional axes (in the b-unique setting)

$$\begin{pmatrix} \boldsymbol{a} \\ \boldsymbol{b} \\ \boldsymbol{c} \end{pmatrix} \to \begin{pmatrix} 1/3 & -1/3 & 2/3 \\ -1 & -1 & 0 \\ 1/3 & -1/3 & -1/3 \end{pmatrix} \begin{pmatrix} \boldsymbol{a} \\ \boldsymbol{b} \\ \boldsymbol{c} \end{pmatrix} \tag{S47}$$

As illustrated in Fig. S 3d, two (out of four) TRIM degeneracies are lifted (at $k = V, V'$). What is the contribution of the new states at $k = V, V'$ to the inversion $\mathbb{Z}_4$ of the higher band? Thanks to the glide symmetry of $C2/c$, the states at V and V' with the same energy have matching inversion eigenvalues, and thus their total contribution to $\mathbb{Z}_4$ must be an even integer, regardless of the details of strain-induced spin interactions.

Finally, if we additionally apply a small magnetic field along $[001]$ of $R\bar{3}c$, the remaining two TRIM degeneracies are lifted, with $+1$ contribution to the higher band (Fig. S 3e). In total, each band is assigned $1, 3 \in \mathbb{Z}_4$, signaling Weyl magnons.

Note that another choice of strain direction ($\perp$ $[110]$) breaks down $R\bar{3}c$ into the same $C2/c$ group, and therefore also leads to Weyl magnons. A different $C2/c$ group can be obtained with strain $\parallel$ $[100]$ or $\perp$ $[100]$, and it turns out a similar argument also predicts Weyl magnons upon an additional $\boldsymbol{B} \parallel [001]$ perturbation.

S1.4 FeF_3

The space group symmetry ($R\bar{3}c$) and the magnetic atom coordinates are identical to CoF_3 in Sec. S1.3. However the magnetic structure (Fig. S 4) is different, having monoclinic $C2'/c'$ (15.89) symmetry. In contrast to CoF_3, note that the 3-fold axis is broken by the magnetic order, leading to a Goldstone mode in bilinear spin models.

The conventional axes of $C2'/c'$ (in the b-unique setting) can be obtained from the hexagonal axes of $R\bar{3}c$ by

$$\begin{pmatrix} \boldsymbol{a} \\ \boldsymbol{b} \\ \boldsymbol{c} \end{pmatrix} \to \begin{pmatrix} 1/3 & 2/3 & 2/3 \\ -1 & 0 & 0 \\ -1/3 & -2/3 & 1/3 \end{pmatrix} \begin{pmatrix} \boldsymbol{a} \\ \boldsymbol{b} \\ \boldsymbol{c} \end{pmatrix} \tag{S48}$$

The magnetic atoms occupy the $4d$ Wyckoff position of $C2'/c'$, with site-symmetry group $\bar{1}$. The spin-wave variables consist of two copies of the even irrep (i.e. $A_g \oplus A_g$) of $\bar{1}$, and the induced band representation is described in Table. S 5. We find two degenerate irreps at the TRIM points $k = M, A$.

k point	$A_g \uparrow$ MSG $\downarrow G_{\boldsymbol{k}}$
$\Gamma(0,0,0)$	$2\Gamma_1^+(1)$
$A(0,0,\frac{1}{2})$	$A_1^- A_1^+(2)$
$L(\frac{1}{2},\frac{1}{2},\frac{1}{2})$	$2L_1^+(1)$
$LA(\frac{1}{2},-\frac{1}{2},\frac{1}{2})$	$2LA_1^-(1)$
$M(0,1,\frac{1}{2})$	$M_1^- M_1^+(2)$
$V(\frac{1}{2},\frac{1}{2},0)$	$V_1^-(1) \oplus V_1^+(1)$
$VA(\frac{1}{2},-\frac{1}{2},0)$	$VA_1^-(1) \oplus VA_1^+(1)$
$Y(0,1,0)$	$2Y_1^+(1)$

Table. S 5: Band representation induced from the A_g irrep of $\bar{1}$, the site-symmetry group of the $4d$ Wyckoff position in $C2'/c'$. The magnon band representation in FeF_3 is induced from two copies of A_g (see Table. S 9.)

Before the system is perturbed, the gapped TRIM points $k = \Gamma, L$ have no contribution to the inversion $\mathbb{Z}_4$ index of the higher band, while each of $k = Y, LA$ contributes $+1$, independently of the spin model (as only one type of inversion parity arises at each of these points). In contrast, the points $k = V$, VA individually contribute wither 0 or $+1$, depending on the model details. However, thanks to the anti-unitary glide c' of $C2'/c'$, the combined contributions of V and VA always add up to an even number. The magnon spectrum and irrep labels are illustrated in Fig. S 4c.

A magnetic field perturbation ($\boldsymbol{B} \parallel [010]$) along the moment directions breaks down $C2'/c'$ into $P\bar{1}$, lifting the two degeneracies with a $+1$ contribution to $\mathbb{Z}_4$. In total, the $\mathbf{Z}_4$ is odd for each band, diagnosing Weyl magnons and Fermi arcs. The band structure after the perturbation is illustrated in Fig. S 4d.

Alternatively, we consider strain-induced Heisenberg interactions. It turns out that only the $A_1^- A_1^+$ degeneracy is lifted. This combined with a subsequent magnetic field perturbation allows to tune a Hamiltonian with $2 \in \mathbb{Z}_4$, indicating a magnon axion insulator.

S1.5 $\mathbf{SrRu_2O_6}$

The structure of $SrRu_2O_6$ has trigonal $P\bar{3}1m$ (162) symmetry. The structural and magnetic unit cells, hosting 2 and 4 Ru atoms respectively, are illustrated in Fig. S 5a.

In Cartesian coordinates, the primitive (hexagonal) translations of the crystal are

$$\boldsymbol{a}_1 = \frac{a}{\sqrt{6}}(1, 1, -2), \qquad \boldsymbol{a}_2 = \frac{a}{\sqrt{6}}(-2, 1, 1), \qquad \boldsymbol{a}_3 = \frac{c}{\sqrt{3}}(1, 1, 1), \tag{S49}$$

and the two Ru atoms in the structural unit cell are positioned at

$$\boldsymbol{r}_\mathrm{A} = \frac{1}{3}\boldsymbol{a}_1 + \frac{2}{3}\boldsymbol{a}_2 + \frac{1}{2}\boldsymbol{a}_3 \tag{S50}$$

$$\boldsymbol{r}_\mathrm{B} = \frac{2}{3}\boldsymbol{a}_1 + \frac{1}{3}\boldsymbol{a}_2 + \frac{1}{2}\boldsymbol{a}_3. \tag{S51}$$

The two sublattices transform under $P\bar{3}1m$ as follows.

- i, bond-centered inversion: $\mathrm{A} \leftrightarrow \mathrm{B}$ within the same cell.
- 3_{001}, three-fold axis along $\boldsymbol{a}_3$:

$$(x_1, x_2, x_3, s) \longrightarrow \begin{cases} (-1 - x_2, -1 + x_1 - x_2, x_3, \mathrm{A}), & s = \mathrm{A} \\ (-1 - x_2, x_1 - x_2, x_3, \mathrm{B}), & s = \mathrm{B} \end{cases}$$

- $M_{1\bar{1}0}$, mirror plane $u(\boldsymbol{a}_1 + \boldsymbol{a}_2) + v\boldsymbol{a}_3$:

$$(x_1, x_2, x_3, s) \longrightarrow \begin{cases} (x_2, x_1, x_3, \mathrm{B}), & s = \mathrm{A} \\ (x_2, x_1, x_3, \mathrm{A}), & s = \mathrm{B} \end{cases}$$

The first NN exchange

$$\mathrm{J}_1 : (0, 0, 0, \mathrm{A}) \leftrightarrow (0, 0, 0, \mathrm{B}) \tag{S52}$$

is constrained by mirror and inversion symmetries to the form

$$\mathsf{J}_1 = \begin{pmatrix} J_1 & \Gamma_1^{xy} & \Gamma_1^{xz} \\ \Gamma_1^{xy} & J_1 + J_1^y & \Gamma_1^{xy} \\ \Gamma_1^{xz} & \Gamma_1^{xy} & J_1 \end{pmatrix}, \tag{S53}$$

while the third NN exchange

$$\mathsf{J}_3 : (0, 0, 0, \mathrm{A}) \leftrightarrow (0, 0, 1, \mathrm{A}) \tag{S54}$$

is constrained by the three-fold axis and a perpendicular two-fold axis to the form

$$\mathsf{J}_3 = \begin{pmatrix} J_3 & J_3^{xy} & J_3^{xz} \\ J_3^{xz} & J_3 & J_3^{xy} \\ J_3^{xy} & J_3^{xz} & J_3 \end{pmatrix} = \begin{pmatrix} J_3 & \Gamma_3 + D_3 & \Gamma_3 - D_3 \\ \Gamma_3 - D_3 & J_3 & \Gamma_3 + D_3 \\ \Gamma_3 + D_3 & \Gamma_3 - D_3 & J_3 \end{pmatrix}. \tag{S55}$$

S1.5.1 Spin-wave spectrum and topology

The magnetic structure (Fig. S 5a) preserves the generators of $P\bar{3}1m$ except $T_{\boldsymbol{a}_3}$, a translation by $\boldsymbol{a}_3$. However, it preserves magnetic translation $\tilde{T}_{\boldsymbol{a}_3} = \tilde{\mathcal{T}} \times T_{\boldsymbol{a}_3}$, a combination of time-reversal $\tilde{\mathcal{T}}$ and $T_{\boldsymbol{a}_3}$. The magnetic space group is therefore $P_c\bar{3}1m$ (162.78), and the unit cell is doubled. The magnetic Bravais translations are given by

$$\begin{pmatrix} \boldsymbol{a} \\ \boldsymbol{b} \\ \boldsymbol{c} \end{pmatrix} = \begin{pmatrix} 1 & & \\ & 1 & \\ & & 2 \end{pmatrix} \begin{pmatrix} \boldsymbol{a}_1 \\ \boldsymbol{a}_2 \\ \boldsymbol{a}_3 \end{pmatrix}. \tag{S56}$$

The magnetic Fe atoms sit on the $4d$ Wyckoff position of $P_c\bar{3}1m$, with site-symmetry group $32'$. Thus the spin-wave variables furnish a direct sum of two irreps (labeled ${}^1E$ and ${}^2E$) with $C_3 = e^{\pm i2\pi/3}$ eigenvalues of the three-fold rotation. Shown in Table. S 6 is the band representations induced from ${}^1E$ and ${}^2E$.

Now we proceed to show that a $\boldsymbol{B} \parallel [001]$ perturbation shall separate the four bands (previously connected by the 4D irrep $A_3^- A_3^+$) at all TRIM points, and furthermore assign a non-trivial SI to the higher pair of bands. The argument can be summarized in the following points.

k point	${}^1E \uparrow \text{MSG} \downarrow G_{\boldsymbol{k}}$	${}^2E \uparrow \text{MSG} \downarrow G_{\boldsymbol{k}}$
$\Gamma(0,0,0)$	$\Gamma_3^+(2) \oplus \Gamma_3^-(2)$	$\Gamma_3^+(2) \oplus \Gamma_3^-(2)$
$A(0,0,\frac{1}{2})$	$A_3^- A_3^+(4)$	$A_3^- A_3^+(4)$
$H(\frac{1}{3},\frac{1}{3},\frac{1}{2})$	$H_1(1) \oplus H_2(1) \oplus H_3(2)$	$H_1(1) \oplus H_2(1) \oplus H_3(2)$
$K(\frac{1}{3},\frac{1}{3},0)$	$K_1(1) \oplus K_2(1) \oplus K_3(2)$	$K_1(1) \oplus K_2(1) \oplus K_3(2)$
$L(\frac{1}{2},0,\frac{1}{2})$	$L_1^+ L_2^-(2) \oplus L_1^- L_2^+(2)$	$L_1^+ L_2^-(2) \oplus L_1^- L_2^+(2)$
$M(\frac{1}{2},0,0)$	$M_1^+(1) \oplus M_1^-(1) \oplus M_2^+(1) \oplus M_2^-(1)$	$M_1^+(1) \oplus M_1^-(1) \oplus M_2^+(1) \oplus M_2^-(1)$

Table. S 6: Band representation induced from the E_1 and E_2 irreps of $32'$, the site-symmetry group of the $4d$ Wyckoff position in $P_c\bar{3}1m$. The magnon band representation in $SrRu_2O_6$ is induced from ${}^1E \oplus {}^2E$ (see Table. S 9.)

- The perturbation $\boldsymbol{B} \parallel [001]$ breaks down $P_c\bar{3}1m$ down into $P\bar{3}1c$ (163.83), and as a result, the 4D irrep into two 2D irreps, each of which containing a single inversion-odd component:

$$A_3^- A_3^+(4) \longrightarrow A_2^- A_2^+(2) \oplus A_3^- A_3^+(2) \tag{S57}$$

- At $k = L$ there exist two copies of the 2D irrep $L_1^- L_1^+$, each containing a single inversion-odd component. Furthermore, the orbit of L (i.e. its k-star) contains two other k-points related by the 3-fold symmetry. This is true both before and after the perturbation.

  Therefore, following the perturbation, the combined contribution of A and L to $\mathbb{Z}_4$ of the higher bands is always

$$1 + 3 = 0 \mod 4. \tag{S58}$$

- The contribution of the remaining 4 TRIM points (Γ and the orbit of M) cannot be fixed by symmetry alone, but requires a concrete spin model. Specifically, we show below that in any spin model with dominant J_1-J_2-J_3 Heisenberg interactions, the combined contribution of Γ and M is constrained to $1, 3, 2 \in \mathbb{Z}_4$, with the latter (i.e. $2 \in \mathbb{Z}_4$) corresponding to the limit of vanishing SOC terms.

Therefore the contribution of the 8 TRIM points shall be $1, 3, 2 \in \mathbb{Z}_4$. The perturbed magnon spectrum is illustrated in Fig. S 6.

However, note that although the higher two bands are separated at all TRIM points following the $\boldsymbol{B}$-field perturbation, they do not satisfy the compatibility relations, which means it is impossible to consistently patch together the two bands while maintaining an energy gap at all high-symmetry lines and planes. In our particular case, this results from a mismatch in the C_3 eigenvalues of the representations at Γ and A within the higher two bands. It follows that the C_3-symmetric line $\Gamma - A$, connecting Γ and A, must host a gapless point to correct for this mismatch.

To eliminate this gapless point, we need to break the C_3 symmetry. This can be achieved by a generic strain perturbation.

Finally, we show by a direct calculation the contribution of Γ and M to $\mathbb{Z}_4$ of the higher bands. The magnon energies at $k = M$ are given by

$$(E_{M_1^+})^2 = \frac{1}{3}\big(4J_1 - 8J_2 + \alpha\big)\big(6J_1 - 24J_2 + 12J_3 + \beta\big) \tag{S59}$$

$$(E_{M_2^+})^2 = \frac{1}{3}\big(12J_1 - 24J_2 + \gamma\big)\big(2J_1 - 8J_2 + 4J_3 + \delta\big) \tag{S60}$$

$$(E_{M_1^-})^2 = \frac{1}{3}\big(2J_1 - 8J_2 + \delta\big)\big(12J_1 - 24J_2 + 12J_3 + \gamma\big) \tag{S61}$$

$$(E_{M_2^-})^2 = \frac{1}{3}\big(6J_1 - 24J_2 + \beta\big)\big(4J_1 - 8J_2 + 4J_3 + \alpha\big) \tag{S62}$$

where $\alpha, \beta, \gamma, \delta$ are SOC terms, given by (up to 2NN bonds)

$$\alpha = -6A + 2\Gamma_1^{xy} + 3\Gamma_1^{xz} - 6\Gamma_2^{xy} - 4\Gamma_2^{xz} + 2J_1^y \tag{S63}$$

$$\beta = -18A + 10\Gamma_1^{xy} + 11\Gamma_1^{xz} - 2\Gamma_2^{xy} - 28\Gamma_2^{xz} + 4J_1^y \tag{S64}$$

$$\gamma = -18A + 14\Gamma_1^{xy} + \Gamma_1^{xz} - 2\Gamma_2^{xy} - 28\Gamma_2^{xz} + 2J_1^y \tag{S65}$$

$$\delta = -6A + 6\Gamma_1^{xy} + \Gamma_1^{xz} - 6\Gamma_2^{xy} - 4\Gamma_2^{xz} \tag{S66}$$

It follows that

$$(E_{M_1^+})^2 - (E_{M_2^-})^2 = \frac{4}{3}J_3(6J_1 + 3\alpha - \beta) \tag{S67}$$

$$= \frac{8}{3}J_3(3J_1 + J_1^y - 2\Gamma_1^{xy} - \Gamma_1^{xz} - 8\Gamma_2^{xy} + 8\Gamma_2^{xz}), \tag{S68}$$

and

$$(E_{M_2^+})^2 - (E_{M_1^-})^2 = \frac{4}{3}(6J_1 - 3\delta + \gamma)J_3 \tag{S69}$$

$$= \frac{8}{3}J_3(3J_1 + J_1^y - 2\Gamma_1^{xy} - \Gamma_1^{xz} + 8\Gamma_2^{xy} - 8\Gamma_2^{xz}). \tag{S70}$$

Furthermore, for the two 2D states at $k = \Gamma$ we find that

$$(E_{\Gamma_3^-})^2 - (E_{\Gamma_3^+})^2 = 8J_3(3J_1 + J_1^y - \Gamma_1^{xy} - \Gamma_1^{xz}), \tag{S71}$$

In the following, we assume the SOC terms are small compared to J_1.

For $J_1 J_3 > 0$, Eqs. S67 and S69 rule out the possibility of having two inversion-odd eigenvalues in the higher two bands. On the other hand, Eq. S71 forces 2D irrep Γ_3^- to be higher than 2D irrep Γ_3^+. Thus, the number of inversion-odd eigenvalues at Γ and the orbit of M is constrained to 2 or 5. Conversely, if $J_1 J_3 < 0$, the total count is either 3 or 6.

S1.6 LaSrFeO$_4$

The structure of LaSrFeO$_4$ is illustrated in Fig. S 7a. The magnetic unit cell has four times the size of the structural cell, with 4 Fe atoms in former and only one in the latter.

The primitive translations of the crystal are

$$\boldsymbol{a}_1 = a(1,0,0), \qquad \boldsymbol{a}_2 = a(0,1,0), \qquad \boldsymbol{a}_3 = \frac{1}{2}(-a,a,c), \tag{S72}$$

and the Fe atom sits at the unit cell corner,

$$\boldsymbol{r}_{\mathrm{A}} = (0,0,0). \tag{S73}$$

The space group $I4/mmm$ (139) transforms Fe lattice sites as follows.

- 4_z, four-fold axis $(0, 0, z)$:

$$(x_1, x_2, x_3, A) \longrightarrow (-x_2, x_1 - x_3, x_3, \mathrm{A}) \tag{S74}$$

- M_z, mirror plane $(x, y, 0)$:

$$(x_1, x_2, x_3, A) \longrightarrow (x_1 - x_3, x_2 + x_3, -x_3, \mathrm{A}) \tag{S75}$$

- M_x, mirror plane $(0, y, z)$:

$$(x_1, x_2, x_3, A) \longrightarrow (-x_1 + x_3, x_2, x_3, \mathrm{A}) \tag{S76}$$

- M_{110}, mirror plane $(x, -x, z)$:

$$(x_1, x_2, x_3, A) \longrightarrow (-x_2, -x_1, x_3, \mathrm{A}) \tag{S77}$$

The first 3 NN exchange paths are

$$\mathsf{J}_1 : (0, 0, 0, \mathrm{A}) \leftrightarrow (0, 1, 0, \mathrm{A}) \tag{S78}$$

$$\mathsf{J}_2 : (0, 0, 0, \mathrm{A}) \leftrightarrow (1, 1, 0, \mathrm{A}) \tag{S79}$$

$$\mathsf{J}_3 : (0, 0, 0, \mathrm{A}) \leftrightarrow (0, 0, 1, \mathrm{A}), \tag{S80}$$

with generic exchange matrices,

$$\mathsf{J}_1 = \begin{pmatrix} J_1 & & \\ & J_1 + J_1^y & \\ & & J_1 + J_1^z \end{pmatrix}, \qquad \mathsf{J}_2 = \begin{pmatrix} J_2 & \Gamma_2^{xy} & \\ \Gamma_2^{xy} & J_2 & \\ & & J_2 + J_2^z \end{pmatrix}, \tag{S81}$$

$$\mathsf{J}_3 = \begin{pmatrix} J_3 & \Gamma_3^{xy} & \Gamma_3^{xz} \\ \Gamma_3^{xy} & J_3 & -\Gamma_3^{xz} \\ \Gamma_3^{xz} & -\Gamma_3^{xz} & J_3 + J_3^z \end{pmatrix}. \tag{S82}$$

k point	$B_g \uparrow \text{MSG} \downarrow G_{\boldsymbol{k}}$
$\Gamma(0,0,0)$	$\Gamma_2^+(1) \oplus \Gamma_3^+(1) \oplus \Gamma_5^+(2)$
$A(\frac{1}{2},\frac{1}{2},\frac{1}{2})$	$A_1^- A_4^-(2) \oplus A_5^-(2)$
$M(\frac{1}{2},\frac{1}{2},0)$	$M_2(2) \oplus M_3(2)$
$R(0,\frac{1}{2},\frac{1}{2})$	$R_1 R_2(4)$
$X(0,\frac{1}{2},0)$	$X_1(2) \oplus X_2(2)$
$Z(0,0,\frac{1}{2})$	$Z_2(2) \oplus Z_4(2)$

Table. S 7: Band representation induced from the B_g irrep of $m'm'm$, the site-symmetry group of the $4d$ Wyckoff position in P_C4_2/nnm. The magnon band representation in $LaSrFeO_4$ is induced from two copies of B_g (see Table. S 9.)

S1.6.1 Spin-wave spectrum and topology

The antiferromagnetic structure of $LaSrFeO_4$ is described by tetragonal P_C4_2/nnm (134.481) symmetry, with a magnetic unit cell illustrated in Fig. S 7a. The magnetic Fe atoms occupy the $4d$ Wyckoff position, and the spin-wave variables transform as two copies of the B_g irrep of the site-symmetry group $m'm'm$ (B_g labels the 1D inversion-even mirror-odd irrep.) Table. S 7 summarized the band representation induced from B_g, indicating a four-fold degeneracy at $k = R$ connecting all magnon bands together.

A combined perturbation of $\boldsymbol{E} \parallel [110]$ and strain $\perp [110]$ reduces $P_C4_2/nnm \longrightarrow C_a2$ (5.17) with monoclinic axes (in the b-unique setting) obtained by

$$\begin{pmatrix} \boldsymbol{a} \\ \boldsymbol{b} \\ \boldsymbol{c} \end{pmatrix} \to \begin{pmatrix} 1 & -1 & \\ 1 & 1 & \\ & & 1 \end{pmatrix} \begin{pmatrix} \boldsymbol{a} \\ \boldsymbol{b} \\ \boldsymbol{c} \end{pmatrix} \tag{S83}$$

and a $\mathbb{Z}_2$ index counting the number of $C_2 = -1$ eigenvalues of 2-fold rotation symmetry at both $k = \Gamma$ and $k = Z$. The nontrivial element $1 \in \mathbb{Z}_2$ corresponds to Weyl points in a plane perpendicular to the 2-fold axis of C_a2.

For the unperturbed system, a spin model with J_1-J_2-J_3 Heisenberg exchange as well as all

symmetry-allowed terms up to 2NN leads to gaps at Γ and Z with

$$\left(E_{Z_4}\right)^2 - \left(E_{Z_2}\right)^2 = \left(E_{\Gamma_5^+}\right)^2 - \left(E_{\Gamma_2^+\oplus\Gamma_3^+}\right)^2 \tag{S84}$$

$$= 8(2J_1 + J_1^y)(J_1^y - 2J_1^z + 2J_2^z). \tag{S85}$$

Thus, the 2D irreps Γ_5^+ and Z_4 belong the higher two bands. Additionally, within C_a2 these irreps break down into

$$\Gamma_5^+ \longrightarrow \Gamma_1(C_2 = +1) \oplus \Gamma_2(C_2 = -1) \tag{S86}$$

$$Z_4 \longrightarrow A_2(C_2 = -1) \oplus A_2(C_2 = -1), \tag{S87}$$

and thus the higher two bands must be nontrivial, following the perturbation.

The nontrivial $\mathbb{Z}_2$ symmetry indicator physically corresponds to Weyl magnons located in lines $k_a = k_b = \pm\pi/2$ in the BZ, for the following reason. First of all, the magnetic translation $\tilde{\mathcal{T}} \equiv T_1 \cdot \mathcal{T}$ satisfies:

$$\tilde{\mathcal{T}}^2 = (C_2 \cdot \tilde{\mathcal{T}})^2 = T_a T_b = e^{i(k_b + k_a)} \tag{S88}$$

Since the 2-fold rotation C_2 is perpendicular to $\hat{z}$-axis, the combined $C_2 \cdot \tilde{\mathcal{T}}$ symmetry plays the role of a PT symmetry in in any one-dimensional manifold $k_a = k_b = k_0,\ \forall\ k_0$. As a result, the polarization in any line $k_a = k_b = k_0,\ \forall\ k_0$ must be quantized with a $\mathbb{Z}_2$ topological invariant (*51*). For a set of trivial bands separated from other bands, as we vary $k_0 \in [-\pi, \pi)$ in the BZ, this $\mathbb{Z}_2$-valued invariant (i.e. polarization) should remain the same: otherwise, the gap must close and Weyl points between this set of bands and other bands must appear.

In the presence of unitary C_2 symmetry for the case of $k_0 = 0, \pi$ with $(C_2 \cdot \tilde{\mathcal{T}})^2 = 1$, the $\mathbb{Z}_2$-valued polarization is exactly given by the aforementioned $\mathbb{Z}_2$ symmetry indicator. Meanwhile, for $k_0 = \pm\pi/2$ lines, the anti-unitary PT symmetry satisfies $(C_2 \cdot \tilde{\mathcal{T}})^2 = -1$ with a Kramers degeneracy, and its polarization must be trivial for any gapped bands, hence a contradiction

with the nontrivial polarization at $k_0 = 0, \pi$. As a result, Weyl points must show up along the $k_a = k_b = \pm\pi/2$ lines.

S1.7 MnTe

S1.7.1 Crystal symmetries and spin model

The MnTe crystal has hexagonal $P6_3/mmc$ (194) symmetry. In Cartesian coordinates, we work with primitive hexagonal translations given by

$$\boldsymbol{a}_1 = \frac{a}{\sqrt{2}}(1, 0, -1), \qquad \boldsymbol{a}_2 = \frac{a}{\sqrt{2}}(-1, 1, 0), \qquad \boldsymbol{a}_3 = \frac{c}{\sqrt{3}}(1, 1, 1). \tag{S89}$$

The magnetic Mn atoms in a crystalline unit cell are

$$\boldsymbol{r}_{\mathrm{A}} = (0, 0, 0) \tag{S90}$$

$$\boldsymbol{r}_{\mathrm{B}} = \frac{1}{2}\boldsymbol{a}_3. \tag{S91}$$

The generators of $P6_3/mmc$ and their action on Mn atoms are as follows.

- 6_3, six-fold screw axis along $w\boldsymbol{a}_3$:

$$(x_1, x_2, x_3, s) \longrightarrow \begin{cases} (x_1 - x_2, x_1, x_3, \mathrm{B}), & s = \mathrm{A} \\ (x_1 - x_2, x_1, 1 + x_3, \mathrm{A}), & s = \mathrm{B} \end{cases}$$

- M_{001}, mirror plane $u\boldsymbol{a}_1 + v\boldsymbol{a}_2 + \frac{1}{4}\boldsymbol{a}_3$:

$$(x_1, x_2, x_3, s) \longrightarrow \begin{cases} (x_1, x_2, -x_3, \mathrm{B}), & s = \mathrm{A} \\ (x_1, x_2, -x_3, \mathrm{A}), & s = \mathrm{B} \end{cases}$$

- M_{100}, mirror plane $u(\boldsymbol{a}_1 + 2\boldsymbol{a}_2) + w\boldsymbol{a}_3$:

$$(x_1, x_2, x_3, s) \longrightarrow \begin{cases} (-x_1 + x_2, x_2, x_3, \mathrm{A}), & s = \mathrm{A} \\ (-x_1 + x_2, x_2, x_3, \mathrm{B}), & s = \mathrm{B} \end{cases}$$

- M_{120}, glide plane $u\boldsymbol{a}_1 + w\boldsymbol{a}_3$ with a fractional translation $\frac{1}{2}\boldsymbol{a}_3$:

$$(x_1, x_2, x_3, s) \longrightarrow \begin{cases} (x_1 - x_2, -x_2, x_3, \mathrm{B}), & s = \mathrm{A} \\ (x_1 - x_2, -x_2, 1 + x_3, \mathrm{A}), & s = \mathrm{B} \end{cases}$$

k point	$B_g \uparrow$ MSG $\downarrow G_{\boldsymbol{k}}$
$\Gamma(0,0,0)$	$\Gamma_2^+(1) \oplus \Gamma_4^+(1)$
$R(1/2,1/2,1/2)$	$R_1(2)$
$S(1/2,1/2,0)$	$S_1^+(1) \oplus S_2^+(1)$
$T(1,0,1/2)$	$T_2(2)$
$Y(1,0,0)$	$Y_2^+(1) \oplus Y_4^+(1)$
$Z(0,0,1/2)$	$Z_2(2)$

Table. S 8: Band representation induced from the B_g irrep of $2/m$, the site-symmetry group of the $4a$ Wyckoff position in $Cmcm$. The magnon band representation in MnTe is induced from two copies of B_g (see Table. S 9.)

The first three NN bonds are given as follows.

$$\mathsf{J}_{1\mathrm{a}} : (0,0,0,\mathrm{A}) \leftrightarrow (0,0,0,\mathrm{B}) \tag{S92}$$

is constrained by a three-fold axis 3_{001}, a perpendicular mirror M_{001} and a parallel mirror M_{100}, leading to the generic form

$$\mathsf{J}_1 = \begin{pmatrix} J_1 & \Gamma_1 & \Gamma_1 \\ \Gamma_1 & J_1 & \Gamma_1 \\ \Gamma_1 & \Gamma_1 & J_1 \end{pmatrix} \tag{S93}$$

$$\mathsf{J}_2 : (0,0,0,\mathrm{A}) \leftrightarrow (1,0,0,\mathrm{A}), \tag{S94}$$

due to inversion symmetry and a perpendicular mirror, reads

$$\mathsf{J}_2 = \begin{pmatrix} J_2 & \Gamma_2^{xy} & \Gamma_2^{xz} \\ \Gamma_2^{xy} & J_2 + J_2^y & \Gamma_2^{xy} \\ \Gamma_2^{xz} & \Gamma_2^{xy} & J_2 \end{pmatrix} \tag{S95}$$

S1.7.2 Spin-wave spectrum and topology

The magnetic moments are aligned antiferromagnetically along the $[110]$ direction of $P6_3/mmc$ (Fig. S 8a), breaking the hexagonal symmetry down into $Cmcm$ (63.457), with conventional

monoclinic axes

$$\begin{pmatrix} \boldsymbol{a} \\ \boldsymbol{b} \\ \boldsymbol{c} \end{pmatrix} = \begin{pmatrix} 1 & 1 & \\ -1 & 1 & \\ & & 1 \end{pmatrix} \begin{pmatrix} \boldsymbol{a}_1 \\ \boldsymbol{a}_2 \\ \boldsymbol{a}_3 \end{pmatrix}, \tag{S96}$$

where $\{\boldsymbol{a}_i\}$ are the hexagonal axes defined in Eq. S89.

The Mn atoms are positioned at the $4a$ Wyckoff position of $Cmcm$, and the magnon band representation is induced from two copies of the B_g irrep of $2/m$, the site-symmetry group at $4a$ (see Table. S 8). The two magnon bands are connected at high-symmetry points $k = R, T, Z$, as shown in Fig. S 8c.

From the subgroup tables (see auxiliary supplementary materials), the only relevant subgroups are centrosymmetric, and thus we only consider magnetic field and strain perturbations. A uniaxial strain $\perp$ $[010]$ breaks down the symmetry $Cmcm \to C2/c$, lifting two degeneracies at $k = L, L'$ in Fig. S 8d. These two k-points are related by glide symmetry, and their inversion eigenvalues are opposite, thus they always contribute $+1$ in total to $\mathbb{Z}_4$. The remaining two gapless TRIM points can be gapped out with $\boldsymbol{B} \parallel [100]$, contributing a multiple of 2 to $\mathbb{Z}_4$ (Fig. S 8e), and thus the SI of either band is odd, signaling Weyl magnons.

Alternatively, a strain-only perturbation can induce topological magnons, as summarized in Fig. S 9. In particular, uniaxial strain $\perp$ $[100]$ reduces $Cmcm \to C2/m$, gapping out all TRIM points. It turns out that within J_1-J_2-J_3 Heisenberg interactions induced by strain, only 3 (out of 4) points are gapped out, and their total contribution to $\mathbb{Z}_4$ is either $+1$ or $+2$ (Fig. S 9b). The remaining point is gapped out by strain-induced SOC terms, and contributes either 0 or $+1$ to $\mathbb{Z}_4$, depending on microscopic details, and thus the result must be either Weyl magnons or magnon axion insulator.

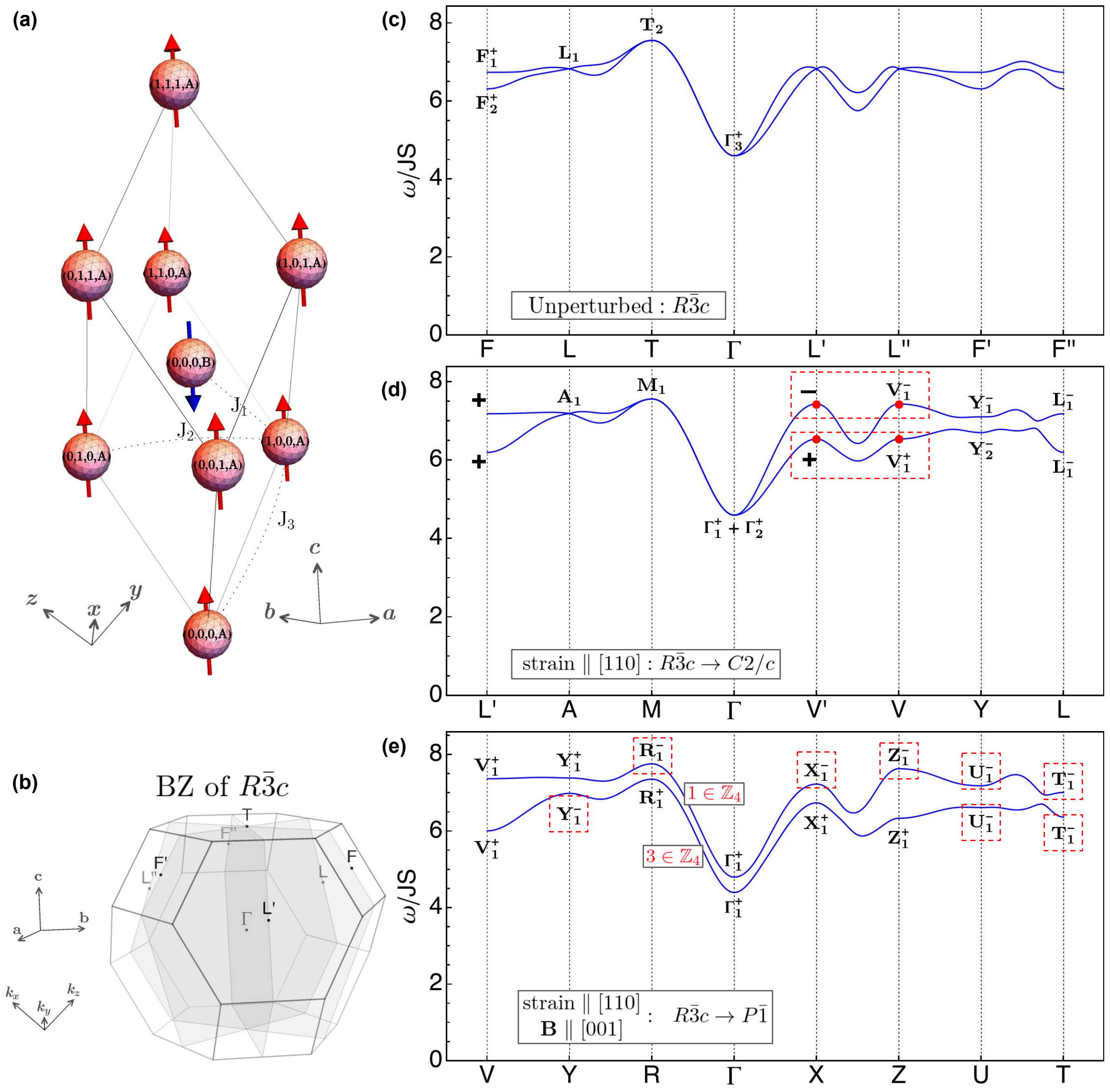

Fig. S 3: **Topological magnons in CoF_3 induced by a combination of strain and magnetic field.** **a**-**b**, The magnetic structure of CoF_3 and the Brillouin zone. **c**, Unperturbed magnon spectrum with $J_1 = 1.00$ and $\Gamma_2^{yz} = -0.26$. **d**, Magnon spectrum in the presence of Heisenberg interactions induced by uni-axial strain in the $[110]$ direction, reducing $R\bar{3}c$ into $C2/c$. At each of V and V' in **d**, there are two non-degenerate states with different contributions to $\mathbb{Z}_4$. However, thanks to the glide symmetry relating k-points V and V', their states show up in pairs of equal energy and identical inversion eigenvalues, rendering their contribution to $\mathbb{Z}_4$ independent of the spin model. **e**, Further applying a magnetic field in the $[001]$ direction gaps out the remaining degeneracies and results in two bands with $1, 3 \in \mathbb{Z}_4$ inversion SI, signaling Weyl magnons. (Perturbation parameters $\sigma = 0.5$, $\beta_3 = 1.2$ and magnetic field $h = 0.2$.)

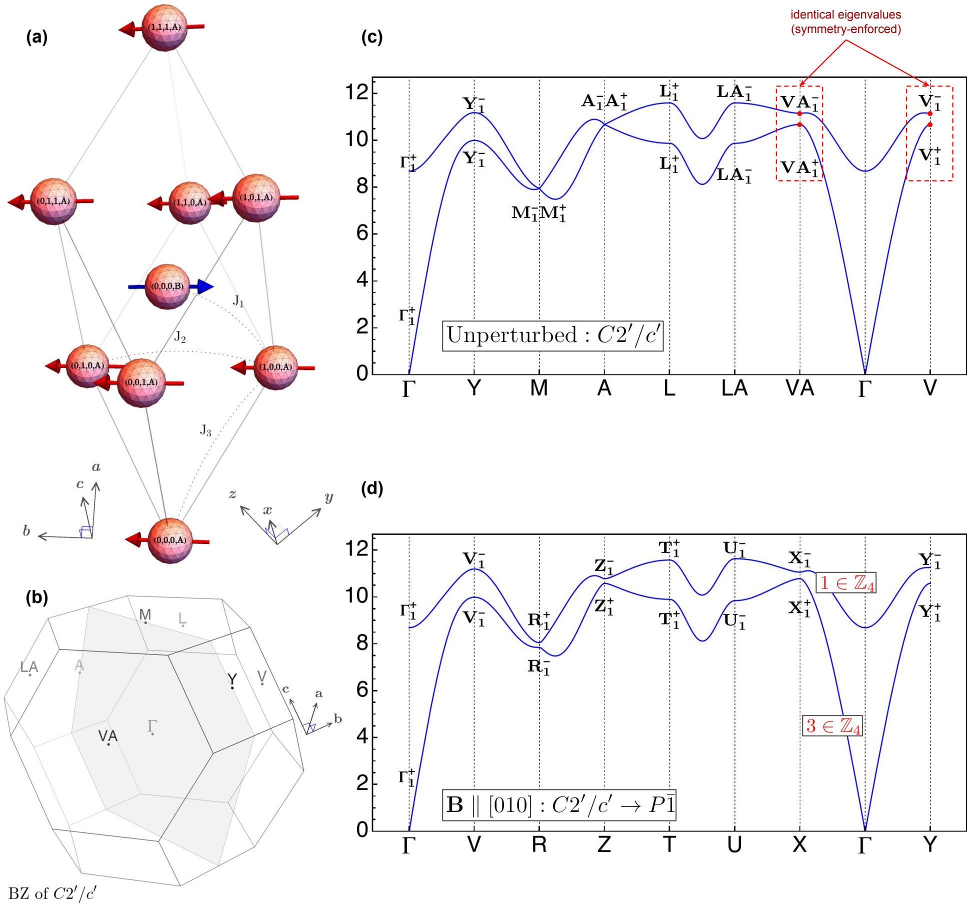

Fig. S 4: **Weyl magnons in FeF_3 induced by a magnetic field. a-b**, The magnetic structure of FeF_3 and the Brillouin zone. **c**, Unperturbed magnon spectrum with $J_1 = 1.00$, $J_2 = -0.40$, $\Gamma_1^{xy} = -0.31$ and $\Gamma_2^{xy} = 0.25$. For any choice of spin model, the total contribution of $k = \Gamma, Y, L, LA$ to $\mathbb{Z}_4$ of the higher band is fixed, as only one parity of inversion arises at each of these points. In contrast, both parities appear at $k = V$ and $k = VA$. However, V and VA are symmetry-related, constraining their total contribution to 0, or 2. **d**, Magnon spectrum after a $\boldsymbol{B} \parallel [010]$ perturbation ($h = 0.1$), leading to $1, 3 \in \mathbb{Z}_4$ inversion SI, signaling Weyl magnons.

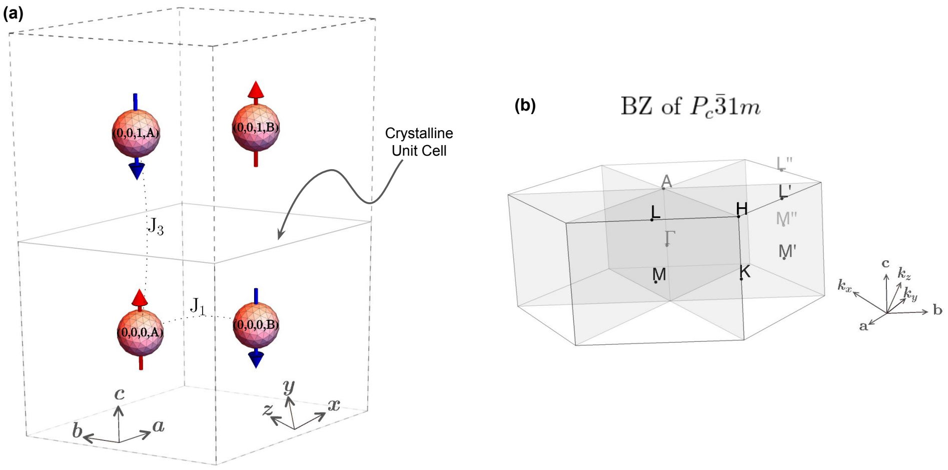

Fig. S 5: **The magnetic structure of $SrRu_2O_6$.** **a**, The magnetic unit cell which is twice as large as the crystalline unit cell. **b**, The magnetic Brillouin zone.

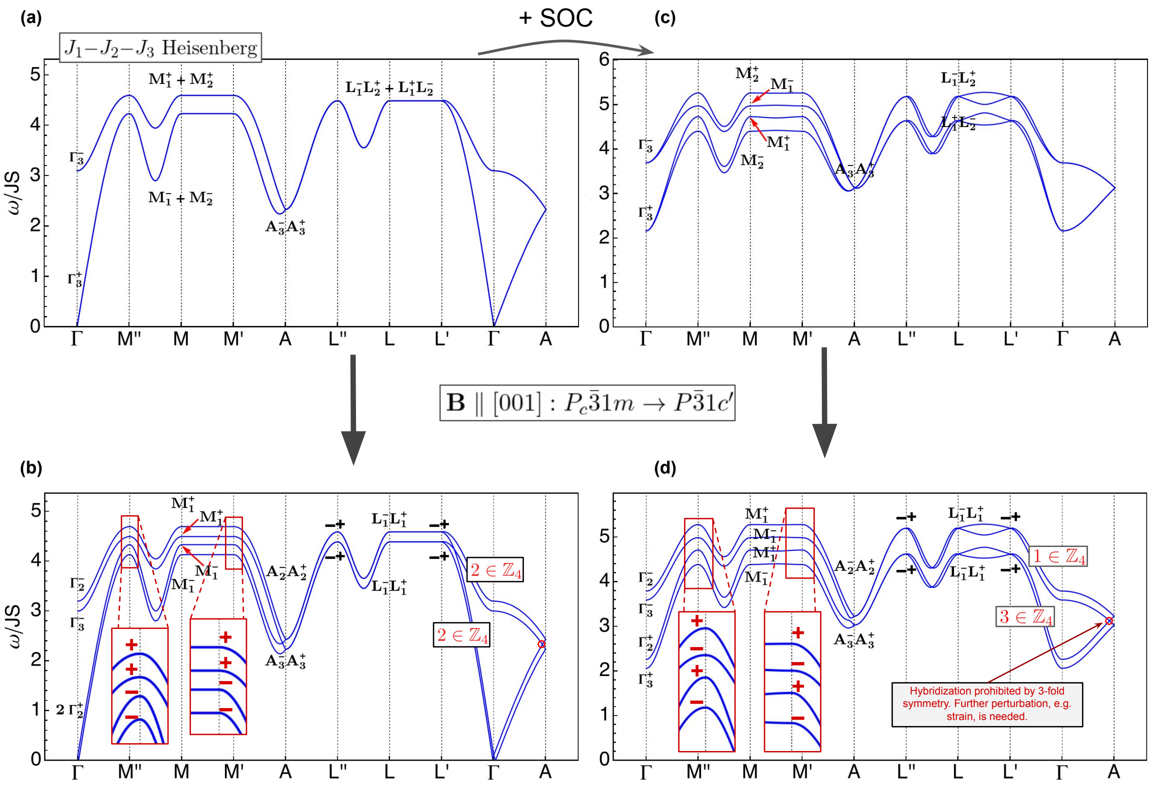

Fig. S 6: **B-field induced topological magnons in $SrRu_2O_6$.** Panels **a** and **c** (**b** and **d**) show the magnon spectrum without (with) a $\boldsymbol{B}$-field perturbation. In **a** and **c** we use a Heisenberg model with $J_1 = 1.00$ and $J_3 = 0.40$.

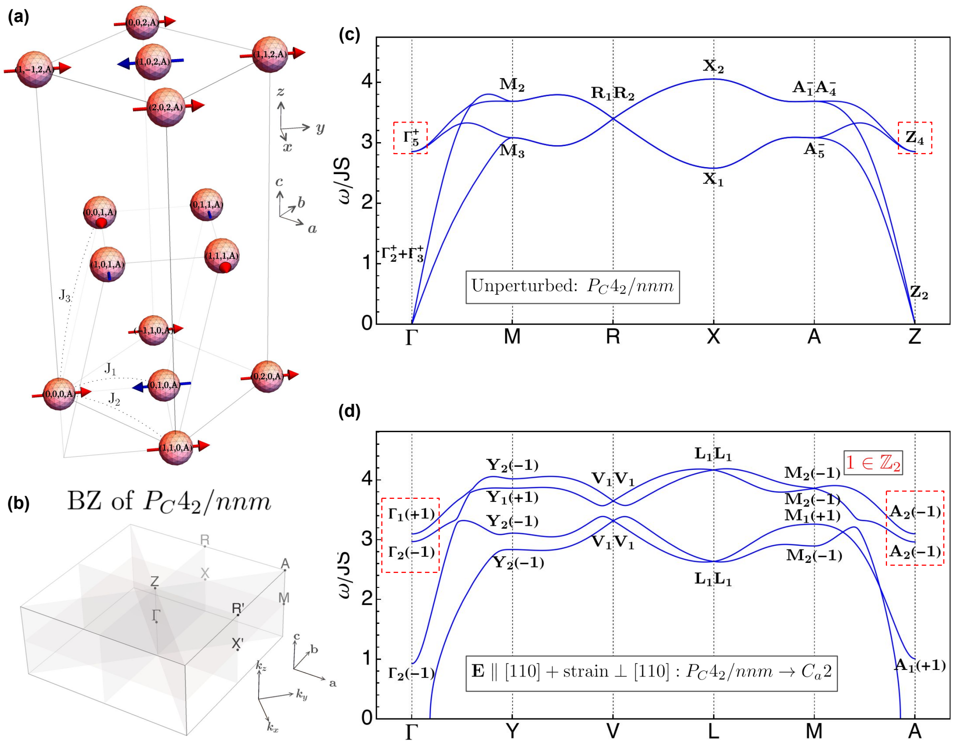

Fig. S 7: **Topological magnon bands in LaSrFeO$_4$. a-b**, The magnetic unit cell and the magnetic Brillouin zone. **c**, Magnon spectrum with $J_1 = 1.00$, $J_3 = 0.36$, $J_1^y = -0.30$ and $J_1^z = -0.45$. Without inter-planar interactions, the states Γ_5^+ and Z_4 are equal in energy. In **d**, Γ_5^+ splits into two states with ± 1 eigenvalues of the 2_{010} symmetry of the magnetic subgroup C_a2, while Z_4 splits into -1 states. These irreps in the subgroup cannot arise from atomic insulators, and they correspond to the nontrivial indicator $1 \in \mathbb{Z}_2$ of C_a2.

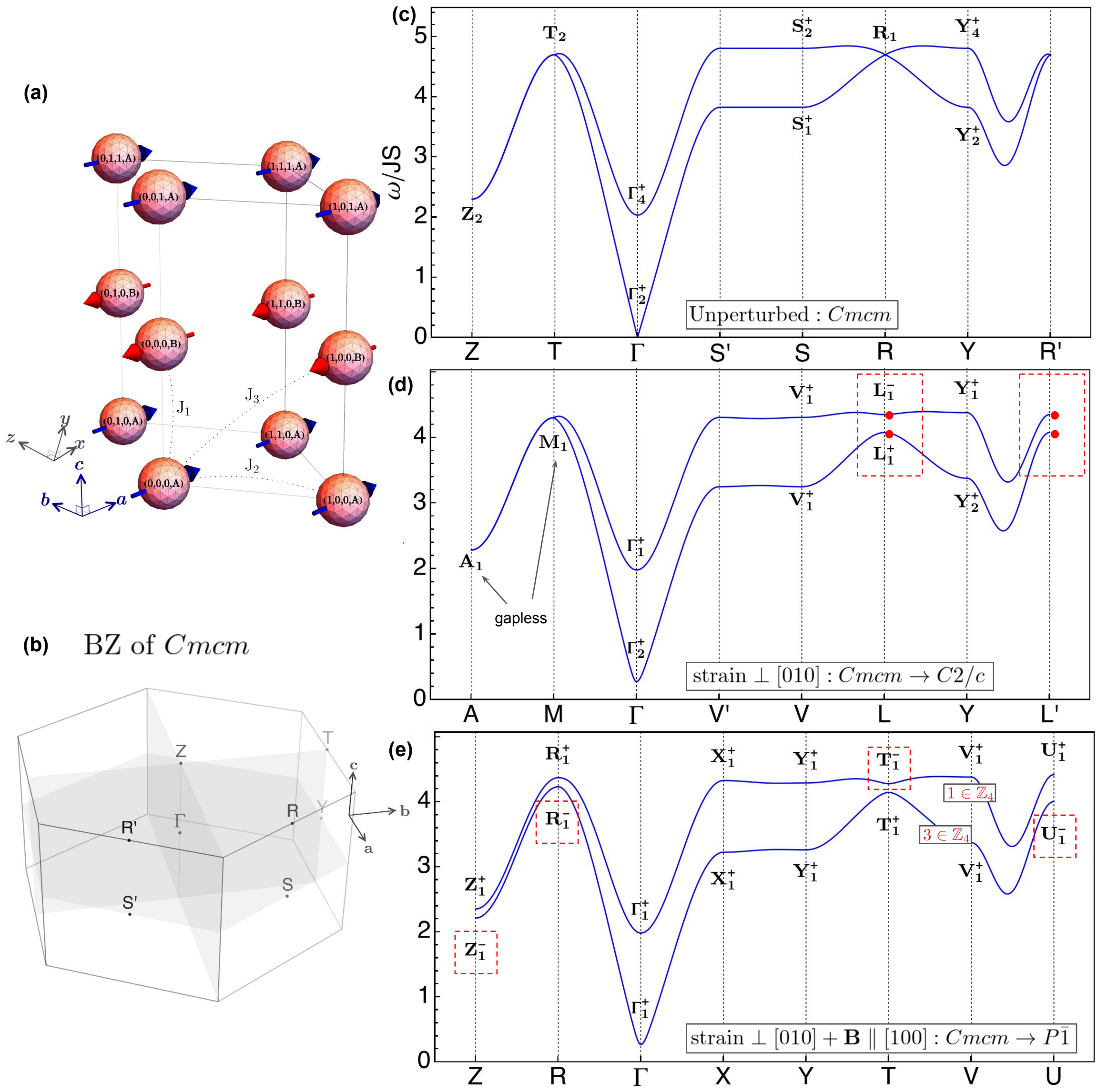

Fig. S 8: **Weyl magnons in MnTe induced by a combination of strain and magnetic field. a-b**, The magnetic structure of MnTe and the Brillouin zone. **c**, Unperturbed magnon spectrum with $J_1 = 1.00$, $\Gamma_1 = -0.15$, $J_2 = -0.30$, $J_3 = 0.22$ and $\Gamma_3^{xy} = -0.05$. **d**, Magnon spectrum perturbed by uniaxial strain $\parallel$ [101] with $\sigma = 0.12$, reducing $Cmcm$ into $C2/c$. We use strain-induced terms $S_2^{xx,J} = .7$, $S_2^{xx,yz} = -0.13$. **e**, Further applying a magnetic field $h = 0.07$ in the [100] direction gaps out the remaining points, and separated bands acquire $1, 3 \in \mathbb{Z}_4$.

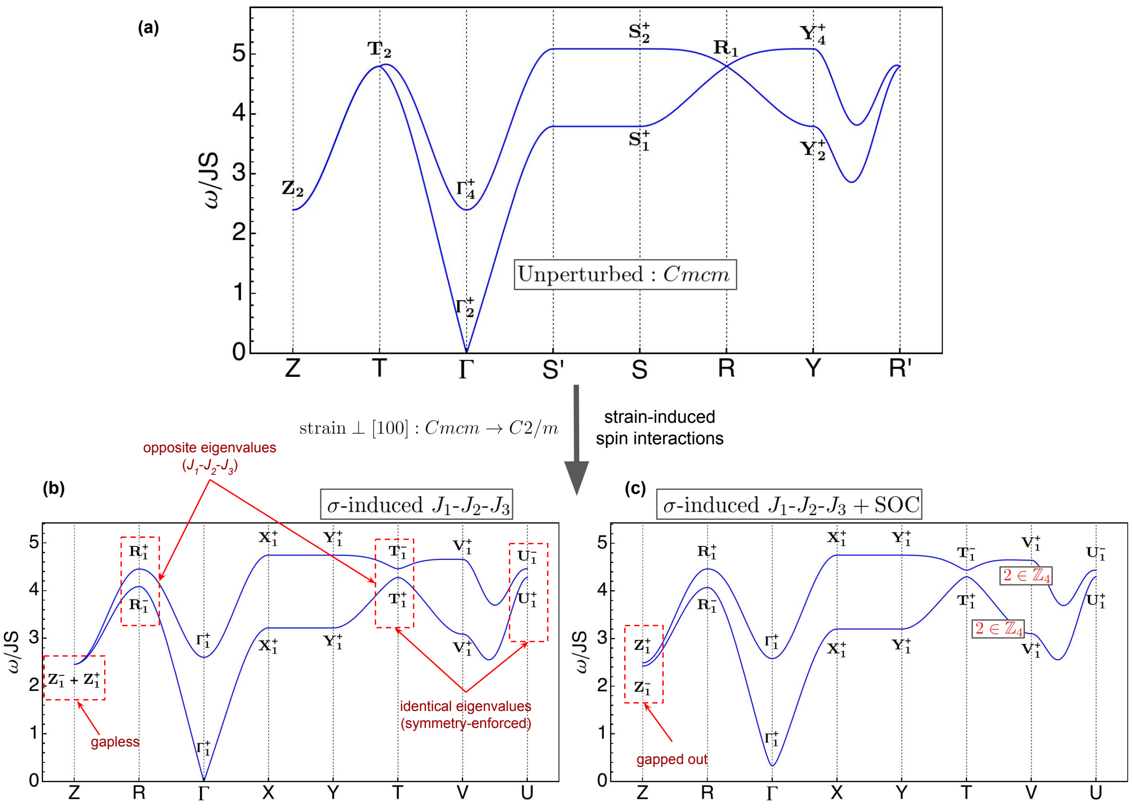

Fig. S 9: **Weyl/magnon axion insulator in MnTe induced by uniaxial strain in the** b**-**c **plane of** $Cmcm$**.** The spin model parameters in **a** are $J_1 = 1.00$, $J_2 = -0.30$, $J_3 = 0.20$, $\Gamma_1^{xy} = -0.23$ and $\Gamma_3^{xy} = -0.05$. In **b-c**, we consider $[011]$ strain $\sigma = 0.20$ with Heisenberg and SOC terms $S_2^{xx,J} = 0.50$, $S_2^{xx,yz} = -0.15$.

Section S2. Holstein-Primakoff boson representation in site-symmetry groups

As discussed in the methods section, the magnon band representation is induced from the representation of the spin-wave variables S_x and S_y (or equivalently, the Holstein-Primakoff boson $b = (S_x + \mathrm{i}S_y)/\sqrt{2S}$ and $b^\dagger$) of the site-symmetry group of a given Wyckoff position. Table. S 9 lists the irreducible representation (irrep) content of the spin-wave variables in all point groups compatible with magnetism.

G	$\rho_{(S_x,S_y)} \downarrow G$	G	$\rho_{(S_x,S_y)} \downarrow G$	G	$\rho_{(S_x,S_y)} \downarrow G$
1	$2A$	4	${}^1E \oplus {}^2E$	6	${}^1E_2 \oplus {}^2E_2$
$\bar{1}$	$2A_g$	$\bar{4}$	${}^1E \oplus {}^2E$	$\bar{6}$	${}^1E'' \oplus {}^2E''$
2	$2B$	$4/m$	${}^1E_g \oplus {}^2E_g$	$6/m$	${}^1E_{2g} \oplus {}^2E_{2g}$
$2'$	$2A$	$42'2'$	${}^1E \oplus {}^2E$	$62'2'$	${}^1E_2 \oplus {}^2E_2$
m	$2A''$	$4m'm'$	${}^1E \oplus {}^2E$	$6m'm'$	${}^1E_2 \oplus {}^2E_2$
m'	$2A$	$\bar{4}2'm'$	${}^1E \oplus {}^2E$	$\bar{6}m'2'$	${}^1E'' \oplus {}^2E''$
$2/m$	$2B_g$	$4/mm'm'$	${}^1E_g \oplus {}^2E_g$	$6/mm'm'$	${}^1E_{2g} \oplus {}^2E_{2g}$
$2'/m'$	$2A_g$	3	${}^1E \oplus {}^2E$		
$2'2'2$	$2B$	$\bar{3}$	${}^1E_g \oplus {}^2E_g$		
$m'm'2$	$2B$	$32'$	${}^1E \oplus {}^2E$		
$m'm2'$	$2A''$	$3m'$	${}^1E \oplus {}^2E$		
$m'm'm$	$2B_g$	$\bar{3}m'$	${}^1E_g \oplus {}^2E_g$		

Table. S 9: Representation of spin-wave variables (S_x, S_y) in all magnetic point groups compatible with magnetism. The notation in Ref. (52) is used for point-group irrep labels.

Section S3. Illustration of magnon surface states

Below, we illustrate the topologically protected magnon surface states in magnetically ordered materials. We focus on three representative cases relevant to the high-T_c topological magnon materials presented in the main text: (1) surface magnon arcs associated with bulk Weyl magnons; (2) surface chiral magnons in magnon axion insulators, where the bulk Chern

number does not vanish; and (3) magnon hinge states in magnon axion insulators, where the bulk Chern number vanishes. We first illustrate the surface magnon arcs that arise due to Weyl points in the bulk magnon spectrum, using $TbFeO_3$ as an example. Next, we discuss two types of topological magnon surface states associated with a magnon axion insulator in the bulk, diagnosed by a $\mathbb{Z}_4$ inversion symmetry indicator $\nu = 2 \bmod 4$. When the Chern number in one 2D plane of the 3D Brillouin zone is nonzero, the magnon axion insulator exhibits chiral surface magnons, which we illustrate using α-Fe_2O_3 as an example. When the Chern number in any 2D plane of the 3D Brillouin zone vanishes, the magnon axion insulator exhibits chiral hinge magnons, which we illustrate using HoRh as an example.

S3.1 Surface magnon arcs

To illustrate the surface magnon arcs that arise in $TbFeO_3$ upon the application of an external magnetic field, we consider a spin model with parameters $J_1^y = 0.8$, $J_1^z = 1.5$, $J_2^y = -0.5$, $\Gamma_2^{xy} = -0.6$, $D_2^z = 0.8$, $J_4^y = -1.0$, $J_4^z = -0.5$, $\Gamma_4^{xy} = -0.7$, and a magnetic field $h_c = 1.0$. Fig. S10(a) shows the magnon spectrum along a line across the Brillouin zone, which connects the two Weyl points in the Brillouin zone. Note that no other states exist in the bulk magnon spectrum at the energy $E = E_{\text{Weyl}}$, indicated with a red dashed line in the figure.

Next, we consider an open boundary in the $\boldsymbol{a}$-direction and compute the surface Green function using the iterative scheme described in Ref. (*53*). The surface spectral function, $\rho_{k_b,k_c}(E = E_{\text{Weyl}}) = -\frac{1}{\pi}\,\mathrm{Im}\,G_{k_b,k_c}(E_{\text{Weyl}} + i0^+)$, is plotted in Fig. S10(b), showing the surface arc connecting the projections of the two Weyl points.

S3.2 Chiral surface magnons in magnon axion insulators

In this section we illustrate the surface chiral magnons with the example of α-Fe_2O_3. With the parameters $J_1 = -0.1, J_2^z = -0.1, J_2 = -1.1, \Gamma_2^{xy} = \Gamma_2^{xz} = \Gamma_2^{yz} = -1$, we find that there's an

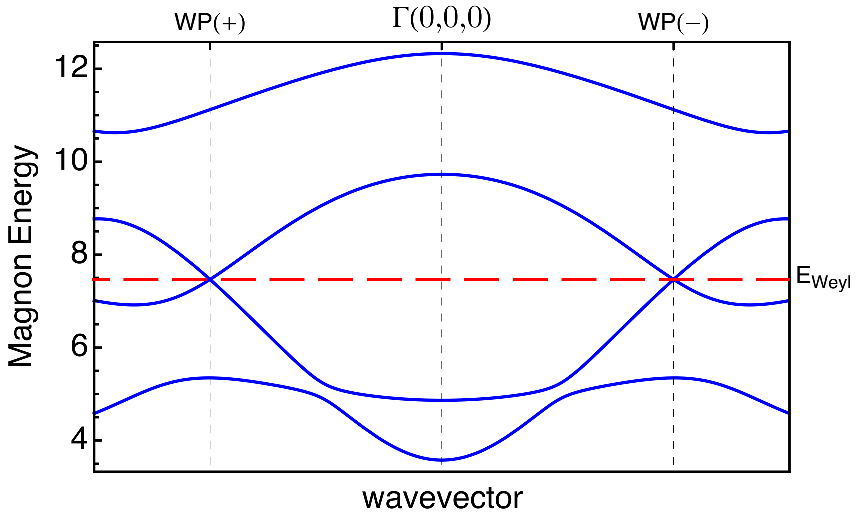

(a)

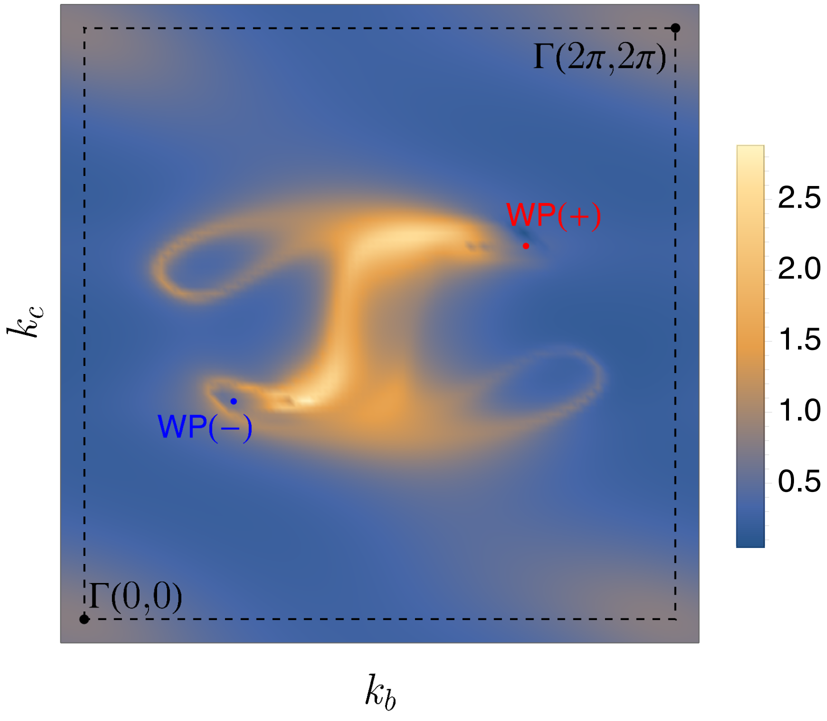

(b)

Fig. S 10: **Weyl points and surface magnon arc in TbFeO$_3$. (a)**, The bulk magnon spectrum along a straight line in the Brillouin zone connecting the two Weyl points. We use the following spin model parameters: $J_1^y = 0.8$, $J_1^z = 1.5$, $J_2^y = -0.5$, $\Gamma_2^{xy} = -0.6$, $D_2^z = 0.8$, $J_4^y = -1.0$, $J_4^z = -0.5$, $\Gamma_4^{xy} = -0.7$, and a magnetic field $h_c = 1.0$. The dashed, red line indicates the energy of the Weyl points, $E = E_{\rm Weyl}$. **(b)**, The spectral function computed at a surface perpendicular to the $\boldsymbol{a}$-direction showing a magnon arc connecting the projections of the Weyl points on the surface.

indirect magnon energy gap between the two upper bands and the two lower bands: there's no states in the energy range $1.41 - 2.06$. In the 2D plane with momentum $k_z = 0$, we find that the total Chern number of the two upper bands is 2. The Chern number for a set of bands isolated from other bands in a 2d plane can be defined (Ref. (*54*)) using H_f, the Hermitian counterpart of the dynamical matrix YR.

Since there's a gap between the upper two magnon bands and the lower two bands, the total Chern number of the two upper bands is 2 for any 2D plane with a fixed momentum k_z.

Let's restrict ourselves to the effective 2D Hamiltonian with $k_z = 0$ as an illustration. We keep the system periodic along $\boldsymbol{a}_1$ direction and make it open along $\boldsymbol{a}_2$ direction. We then plot the magnon spectrum as a function of k_x in Fig. S11. Two pairs of chiral edge states interpolating between the two upper bands and the two lower bands are clearly seen.

S3.3 Hinge magnons in magnon axion insulators

The magnon hinge states observed in this work are different from what was previously discussed in Ref. (*40*) and (*41*). Specifically, in Ref. (*40*), the chiral hinge magnons are protected by a mirror Chern number of the gapped bulk magnons, and therefore are robust only on a hinge that preserves the mirror symmetry. In Ref. (*41*), the magnon hinge states coexist with magnon band crossings in the bulk, in analogy to topological semimetals in the context of electron bands. Moreover, the $\mathbb{Z}_2$ topological protection of the hinge magnons is provided by a 2-fold rotational symmetry C_{2x} in Ref. (*41*). In contrast, the hinge magnons of magnon axion insulators discussed in this work are protected by the inversion symmetry (*34*), different from both Ref. (*40*) and (*41*).

For an axion insulator with a $\mathbb{Z}_4$ inversion symmetry indicator $\nu = 2 \bmod 4$, the reason for protected hinge magnons can be understood as follows (*34*). When its Chern number in any 2D plane in the momentum space vanishes, the wavefunction of an axion insulator can be

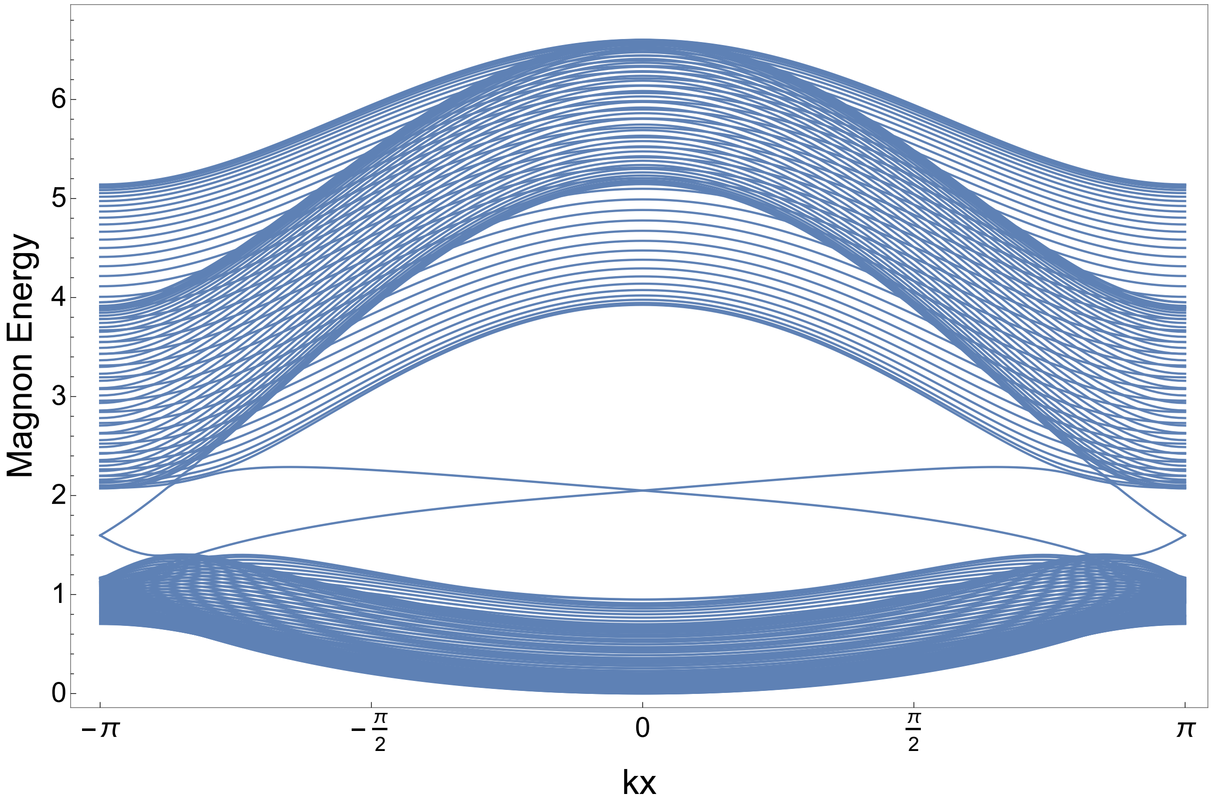

Fig. S 11: **Surface chiral magnons in** $\alpha-$**Fe**$_2$**O**$_3$**.** The parameters we use are $J_1 = -0.1$, $J_2^z = -0.1$, $J_2 = -1.1$, $\Gamma_2^{xy} = \Gamma_2^{xz} = \Gamma_2^{yz} = -1$. We keep the system periodic along $\boldsymbol{a}_1, \boldsymbol{a}_3$ direction and open along $\boldsymbol{a}_2$ direction. We have included 40 unit-cells along $\boldsymbol{a}_2$ direction. In this figure we have plotted the magnon spectrum as a function of k_x with $k_z = 0$ fixed. The bulk spectrum has an indirect energy gap $(1.41 - 2.06)$ between the two lower bands and the two upper bands. With the presence of an edge in the x-direction, two pairs of chiral edge states interpolating between the upper bands and the lower bands are clearly seen, consistent with the fact that Chern number is 2 for the upper bands.

adiabatically tuned into that of a 3D topological insulator, which exhibits Dirac surface states. Now consider a finite system with two open surfaces which join at a hinge. Each of the two surfaces preserve no symmetry, and hence the Dirac surface states should be gapped out by a mass term. Meanwhile, the inversion symmetry guarantees the two surfaces related by inversion have opposite mass terms (*34*). As a result, the hinge is a mass domain wall of the Dirac surface states, giving rise to a 1D chiral hinge mode in 3D axion insulators with a vanishing Hall conductance tensor.

In the following, we illustrate the hinge magnon states using HoRh as an example. We start by first summarizing the results, and subsequently describing more details of the system.

S3.3.1 Hinge magnon modes in HoRh

Below $T_{\mathrm{c}} = 3.2\,\mathrm{K}$, the magnetic moments of the holmium atoms form a non-collinear anti-ferromagnetic order (*55*), as illustrated in Fig. S12. More specifically, the magnetic structure is symmetric under the MSG $P_I a\bar{3}$ (205.36) and the magnetic atoms are located at the $8b$ Wyckoff position. The coordinates and moments of the (magnetic) holmium atoms, belonging to the $8b$ Wyckoff position, are given by (in basis of primitive, cubic Bravais vectors)

$$\begin{aligned}
&\Big\{\Big(0,0,0\Big),(m_x,m_x,m_x)\Big\}, &&\Big\{\Big(\frac{1}{2},\frac{1}{2},0),(m_x,-m_x,-m_x\Big)\Big\},\\
&\Big\{\Big(0,\frac{1}{2},\frac{1}{2}\Big),(-m_x,m_x,-m_x)\Big\}, &&\Big\{\Big(\frac{1}{2},0,\frac{1}{2}\Big),(-m_x,-m_x,m_x)\Big\},\\
&\Big\{\Big(\frac{1}{2},\frac{1}{2},\frac{1}{2}\Big),(-m_x,-m_x,-m_x)\Big\}, &&\Big\{\Big(0,0,\frac{1}{2}),(-m_x,m_x,m_x\Big)\Big\},\\
&\Big\{\Big(\frac{1}{2},0,0\Big),(m_x,-m_x,m_x)\Big\}, &&\Big\{\Big(0,\frac{1}{2},0),(m_x,m_x,-m_x\Big)\Big\}
\end{aligned}$$

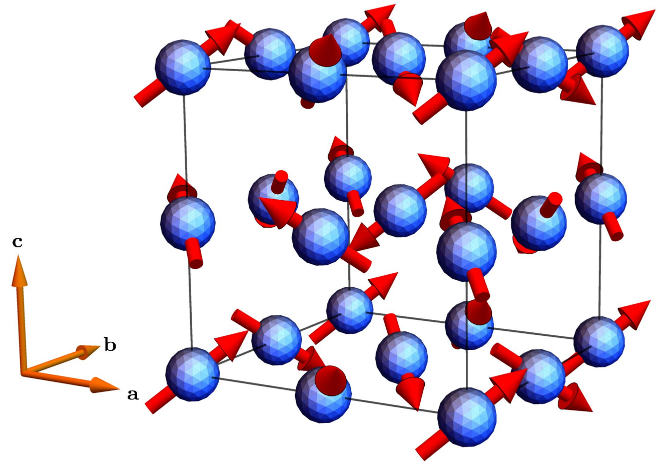

Fig. S 12: **The magnetic order of HoRh below $T_{\mathbf{c}} = 3.2\,\mathrm{K}$.** Only the magnetic atoms (Ho) are shown, and they occupy the $8b$ WP of $P_Ia\bar{3}$ (205.36) with a site symmetry group $\bar{3}$.

The 8 magnon bands have the following band representation:

$$\begin{aligned}({}^1E_g)_{8b} \uparrow P_Ia\bar{3}\ (205.36) = {} & \Gamma_2^+\Gamma_3^+(2) \oplus 2\Gamma_4^+(3) \\ & \oplus R_1^+R_3^-(4) \oplus R_2^-R_2^+(4) \\ & \oplus 2X_1(2) \oplus 2X_2(2) \\ & \oplus 2M_1(2) \oplus 2M_2(2)\end{aligned}$$

where

$$\begin{aligned}\boldsymbol{k}_\Gamma &= (0,0,0), \\ \boldsymbol{k}_R &= (\pi,\pi,\pi), \\ \star\boldsymbol{k}_X &= \{(\pi,0,0),(0,\pi,0),(0,0,\pi)\}, \\ \star\boldsymbol{k}_M &= \{(0,\pi,\pi),(\pi,0,\pi),(\pi,\pi,0)\}.\end{aligned}$$

A crucial property of this band representation is that, at any given TRIM point, all the irreps have the same number of $I = -1$ inversion eigenvalues. For example, at $\boldsymbol{k}_M$, each of $M_1(2)$ and $M_2(2)$ contains one inversion-even and one inversion-odd modes. Additionally, by

simple counting, the highest 4 magnon bands must always have an inversion SI $\nu = 2 \bmod 4$. Therefore, if we energetically separate the highest 4 bands by applying an inversion-preserving perturbation, the result is always a magnon axion insulator, independent of the details of the unperturbed spectrum and the perturbation specifics. Thus, HoRh is a type-I topological magnon candidate.

Next, we consider a system with open boundaries in the $\boldsymbol{a}$- and $\boldsymbol{b}$-directions and we keep it periodic in the $\boldsymbol{c}$-direction. We construct a spin model (see details in the next subsection), as well as an inversion-preserving perturbation that separates the highest four bands and creates a gap both in the bulk and the 2D surfaces. We summarize the resulting spectrum in Fig. S13. This figure illustrates the appearance of two chiral 1D modes localized at two opposite hinges and propagating in opposite directions along the $\boldsymbol{c}$-direction.

S3.3.2 Additional details: the magnon Hamiltonian

Here, we provides details about the Hamiltonian we used to compute the spectrum in Fig. S13. We start by identifying all symmetry-allowed, bilinear interaction terms up to the 3rd nearest-neighbor bonds.

The space group of HoRh is $Pm\bar{3}m$ (221), with a primitive cubic lattice. There is one Ho atom per crystalline unit cell and it is located at the origin, where the site symmetry group is $m\bar{3}m$. We use a triplet of integers, $(x_1 x_2 x_3)$, to label a general Ho site, with a location at $\boldsymbol{R}_{(x_1 x_2 x_3)} = x_1 \boldsymbol{a}_1 + x_2 \boldsymbol{a}_2 + x_3 \boldsymbol{a}_3$. Here $\{\boldsymbol{a}_i\}$ are the primitive translations, given in Cartesian coordinates as $a(1,0,0)$, $a(0,1,0)$, and $a(0,0,1)$ respectively.

The 1NN interactions are specified by two free parameters. For example, the interaction matrix between sites (000) and (100) reads

$$\mathsf{J}_1 = \begin{pmatrix} J_1 + J_1^x & & \\ & J_1 & \\ & & J_1 \end{pmatrix}. \tag{S97}$$

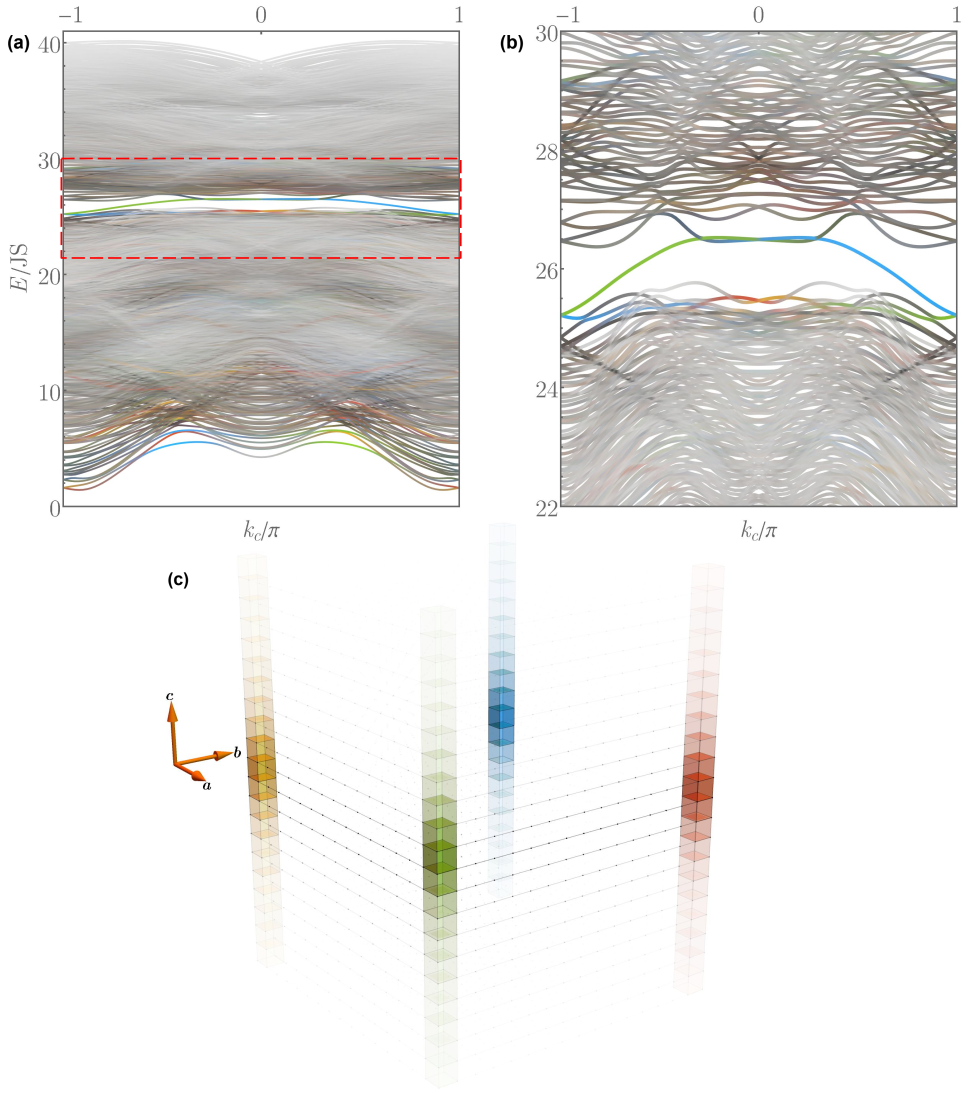

Fig. S 13: **1D chiral hinge magnons in HoRh after the application of mechanical strain in a low-symmetry direction. (a)**, The magnon spectrum upon perturbing the system, using open boundaries in the ***a***- and ***b***-directions and a periodic ***c***-direction. We include 16 crystalline unit cells in each open direction. **(b)**, The same band structure but focusing on states near the gap (see the red, dashed rectangle in (a)). The colors in the spectrum are given by the weights of the wave function in the ***a***-***b*** plane with different regions contributing different colors. Specifically, bulk and surface states tend to be gray and dark gray, respectively, and states localized at a hinge tends towards the corresponding color shown in (c). **(c)**, A schematic of the system with two open boundaries and one periodic, as well as a color code to label hinge modes. In the spectrum in (a) and (b), one can clearly see the existence of in-gap 1D chiral modes localized at opposite hinges.

For the 2NN interaction between sites (000) and (110), the interaction matrix is

$$\mathsf{J}_2 = \begin{pmatrix} J_2 & \Gamma_2 & \\ \Gamma_2 & J_2 & \\ & & J_2 + J_2^z \end{pmatrix} \tag{S98}$$

For the 3NN interaction between sites (000) and (111), we have

$$\mathsf{J}_3 = \begin{pmatrix} J_3 & \Gamma_3 & \Gamma_3 \\ \Gamma_3 & J_3 & \Gamma_3 \\ \Gamma_3 & \Gamma_3 & J_3 \end{pmatrix} \tag{S99}$$

All other 1NN, 2NN and 3NN interactions are related to the bonds above by a symmetry in $Pm\bar{3}m$, and therefore they are all fixed by the same parameters.

Next, we compute the interaction matrices that couple to the strain tensor

$$\sigma = \begin{pmatrix} \sigma^{xx} & \sigma^{xy} & \sigma^{xz} \\ \sigma^{xy} & \sigma^{yy} & \sigma^{yz} \\ \sigma^{xz} & \sigma^{xy} & \sigma^{zz} \end{pmatrix}. \tag{S100}$$

For the 1NN bond $(000) - (100)$, the symmetry allowed exchange matrices are

$$\mathsf{\Sigma}_1 = \begin{pmatrix} \begin{pmatrix} \Sigma_1^{xx,xx} & & \\ & \Sigma_1^{xx,yy} & \\ & & \Sigma_1^{xx,yy} \end{pmatrix} & \begin{pmatrix} & \Sigma_1^{xy,xy} & \\ \Sigma_1^{xy,xy} & & \\ & & \end{pmatrix} & \begin{pmatrix} & & \Sigma_1^{xy,xy} \\ & & \\ \Sigma_1^{xy,xy} & & \end{pmatrix} \\ \begin{pmatrix} & \Sigma_1^{xy,xy} & \\ \Sigma_1^{xy,xy} & & \\ & & \end{pmatrix} & \begin{pmatrix} \Sigma_1^{yy,xx} & & \\ & \Sigma_1^{yy,yy} & \\ & & \Sigma_1^{yy,zz} \end{pmatrix} & \begin{pmatrix} & & \\ & & \Sigma_1^{yz,yz} \\ & \Sigma_1^{yz,yz} & \end{pmatrix} \\ \begin{pmatrix} & & \Sigma_1^{xy,xy} \\ & & \\ \Sigma_1^{xy,xy} & & \end{pmatrix} & \begin{pmatrix} & & \\ & & \Sigma_1^{yz,yz} \\ & \Sigma_1^{yz,yz} & \end{pmatrix} & \begin{pmatrix} \Sigma_1^{yy,xx} & & \\ & \Sigma_1^{yy,zz} & \\ & & \Sigma_1^{yy,yy} \end{pmatrix} \end{pmatrix} \tag{S101}$$

For the 2NN bond $(000) - (110)$, we obtain

$$\mathsf{\Sigma}_2 = \begin{pmatrix} \begin{pmatrix} \Sigma_2^{xx,xx} & \Sigma_2^{xx,xy} & \\ \Sigma_2^{xx,xy} & \Sigma_2^{xx,yy} & \\ & & \Sigma_2^{xx,zz} \end{pmatrix} & \begin{pmatrix} \Sigma_2^{xy,xx} & \Sigma_2^{xy,xy} & \\ \Sigma_2^{xy,xy} & \Sigma_2^{xy,xx} & \\ & & \Sigma_2^{xy,zz} \end{pmatrix} & \begin{pmatrix} & & \Sigma_2^{xz,xz} \\ & & \Sigma_2^{xz,yz} \\ \Sigma_2^{xz,xz} & \Sigma_2^{xz,yz} & \end{pmatrix} \\ \begin{pmatrix} \Sigma_2^{xy,xx} & \Sigma_2^{xy,xy} & \\ \Sigma_2^{xy,xy} & \Sigma_2^{xy,xx} & \\ & & \Sigma_2^{xy,zz} \end{pmatrix} & \begin{pmatrix} \Sigma_2^{xx,yy} & \Sigma_2^{xx,xy} & \\ \Sigma_2^{xx,xy} & \Sigma_2^{xx,xx} & \\ & & \Sigma_2^{xx,zz} \end{pmatrix} & \begin{pmatrix} & & \Sigma_2^{xz,yz} \\ & & \Sigma_2^{xz,xz} \\ \Sigma_2^{xz,yz} & \Sigma_2^{xz,xz} & \end{pmatrix} \\ \begin{pmatrix} & & \Sigma_2^{xz,xz} \\ & & \Sigma_2^{xz,yz} \\ \Sigma_2^{xz,xz} & \Sigma_2^{xz,yz} & \end{pmatrix} & \begin{pmatrix} & & \Sigma_2^{xz,yz} \\ & & \Sigma_2^{xz,xz} \\ \Sigma_2^{xz,yz} & \Sigma_2^{xz,xz} & \end{pmatrix} & \begin{pmatrix} \Sigma_2^{zz,xx} & \Sigma_2^{zz,xy} & \\ \Sigma_2^{zz,xy} & \Sigma_2^{zz,xx} & \\ & & \Sigma_2^{zz,zz} \end{pmatrix} \end{pmatrix} \tag{S102}$$

Finally, for the 3NN bond $(000) - (111)$, we have

$$\Sigma_3 = \begin{pmatrix} \begin{pmatrix} \Sigma_3^{xx,xx} & \Sigma_3^{xx,xy} & \Sigma_3^{xx,xy} \\ \Sigma_3^{xx,xy} & \Sigma_3^{xx,yy} & \Sigma_3^{xx,yz} \\ \Sigma_3^{xx,xy} & \Sigma_3^{xx,yz} & \Sigma_3^{xx,yy} \end{pmatrix} & \begin{pmatrix} \Sigma_3^{xy,xx} & \Sigma_3^{xy,xy} & \Sigma_3^{xy,xz} \\ \Sigma_3^{xy,xy} & \Sigma_3^{xy,xx} & \Sigma_3^{xy,xz} \\ \Sigma_3^{xy,xz} & \Sigma_3^{xy,xz} & \Sigma_3^{xy,zz} \end{pmatrix} & \begin{pmatrix} \Sigma_3^{xy,xx} & \Sigma_3^{xy,xz} & \Sigma_3^{xy,xy} \\ \Sigma_3^{xy,xz} & \Sigma_3^{xy,zz} & \Sigma_3^{xy,xz} \\ \Sigma_3^{xy,xy} & \Sigma_3^{xy,xz} & \Sigma_3^{xy,xx} \end{pmatrix} \\ \begin{pmatrix} \Sigma_3^{xy,xx} & \Sigma_3^{xy,xy} & \Sigma_3^{xy,xz} \\ \Sigma_3^{xy,xy} & \Sigma_3^{xy,xx} & \Sigma_3^{xy,xz} \\ \Sigma_3^{xy,xz} & \Sigma_3^{xy,xz} & \Sigma_3^{xy,zz} \end{pmatrix} & \begin{pmatrix} \Sigma_3^{xx,yy} & \Sigma_3^{xx,xy} & \Sigma_3^{xx,yz} \\ \Sigma_3^{xx,xy} & \Sigma_3^{xx,xx} & \Sigma_3^{xx,xy} \\ \Sigma_3^{xx,yz} & \Sigma_3^{xx,xy} & \Sigma_3^{xx,yy} \end{pmatrix} & \begin{pmatrix} \Sigma_3^{xy,zz} & \Sigma_3^{xy,xz} & \Sigma_3^{xy,xz} \\ \Sigma_3^{xy,xz} & \Sigma_3^{xy,xx} & \Sigma_3^{xy,xy} \\ \Sigma_3^{xy,xz} & \Sigma_3^{xy,xy} & \Sigma_3^{xy,xx} \end{pmatrix} \\ \begin{pmatrix} \Sigma_3^{xy,xx} & \Sigma_3^{xy,xz} & \Sigma_3^{xy,xy} \\ \Sigma_3^{xy,xz} & \Sigma_3^{xy,zz} & \Sigma_3^{xy,xz} \\ \Sigma_3^{xy,xy} & \Sigma_3^{xy,xz} & \Sigma_3^{xy,xx} \end{pmatrix} & \begin{pmatrix} \Sigma_3^{xy,zz} & \Sigma_3^{xy,xz} & \Sigma_3^{xy,xz} \\ \Sigma_3^{xy,xz} & \Sigma_3^{xy,xx} & \Sigma_3^{xy,xy} \\ \Sigma_3^{xy,xz} & \Sigma_3^{xy,xy} & \Sigma_3^{xy,xx} \end{pmatrix} & \begin{pmatrix} \Sigma_3^{xx,yy} & \Sigma_3^{xx,yz} & \Sigma_3^{xx,xy} \\ \Sigma_3^{xx,yz} & \Sigma_3^{xx,yy} & \Sigma_3^{xx,xy} \\ \Sigma_3^{xx,xy} & \Sigma_3^{xx,xy} & \Sigma_3^{xx,xx} \end{pmatrix} \end{pmatrix} \tag{S103}$$

Similar to the unperturbed terms, all other 1NN, 2NN and 3NN interactions induced by strain are related to the bonds above by a symmetry in $Pm\bar{3}m$, and therefore they are also all fixed by the same parameters shown above.

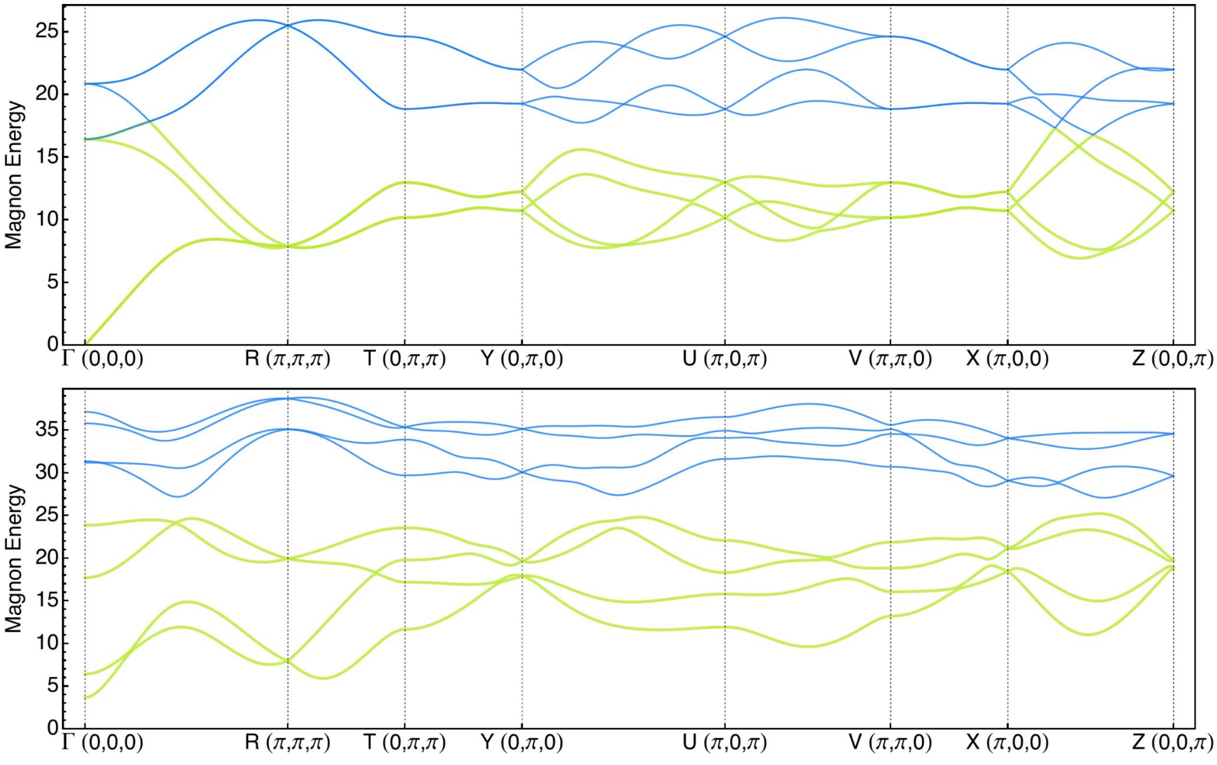

Fig. S 14: **The bulk magnon spectrum of HoRh.** The top (bottom) panel shows the magnon spectrum before (after) perturbing the system with a mechanical strain (see the supplementary text for a complete description of the perturbation specifics).

We included the following parameters in our calculation in this section (both bulk and hinge

calculations):

$$J_1 = 0.40, J_1^x = -0.15, J_2^z = 3.30, \Gamma_2 = 2.20, J3 = 0.50, \Gamma_3 = 0.40,$$

$$\Sigma_1^{xyxy} = 0.5, \Sigma_1^{yyxx} = -0.5, \Sigma_2^{xxxx} = 0.34, \Sigma_2^{xxxy} = -0.94,$$

$$\Sigma_2^{xxyy} = 0.26, \Sigma_2^{xxzz} = 0.27, \Sigma_2^{xyxx} = -0.50, \Sigma_2^{xyxy} = 1.34,$$

$$\Sigma_2^{xyzz} = -0.86, \Sigma_2^{xzxz} = -0.2^0, \Sigma_2^{xzyz} = -0.3^2, \Sigma_2^{zzxx} = 0.06,$$

$$\Sigma_2^{zzxy} = -0.81, \Sigma_3^{xxxx} = -0.30, \Sigma_3^{xxxy} = 0.50, \Sigma_3^{xxyy} = -0.75,$$

$$\Sigma_3^{xyxx} = -0.60, \Sigma_3^{xyxy} = 0.05, \Sigma_3^{xyxz} = -0.25, \Sigma_3^{xyzz} = 0.50,$$

$$\sigma^{xx} = -0.10, \sigma^{xy} = -0.64, \sigma^{xz} = 0.52, \sigma^{yy} = -0.33, \sigma^{yz} = 0.79, \sigma^{zz} = -0.12,$$

in addition to a staggered magnetic field ($h = 0.7$) to stabilize the magnetic structure.

The bulk magnetic structure before and after the perturbation is illustrated in Fig. S14.

Section S4. Realizations of subgroups via external perturbations

For every magnetic space group, the tables in the auxiliary supplementary materials provide a complete list of subgroups that can be obtained by the application of an electric field, a magnetic field, strain or any combination thereof. For each subgroup, the corresponding symmetry indicator group is also listed. The second column in each table lists all possible field directions that realize a subgroup.

These tables can be utilized in two ways to speed up the search:

1. Screening a database of magnetic materials: if the subgroups table of a material's MSG only contains trivial SI groups, the material is dropped from further consideration. There are > 300 such MSGs, all of which are listed in Section S5.. Based on this list, the Bilbao database was scanned to eliminate 381 materials in this category.

2. For other MSGs, the tables can speed up the choice of perturbations to consider. For example, the magnon bands of $BaMn_2P_2$ have degeneracies protected by its MSG symmetry ($I4'/m'm'm$). We would like to lift the degeneracy and evaluate the SIs for the newly isolated bands. There are many perturbations/subgroups to consider, but according to Tab. 10, all subgroups except for one have trivial SI groups: we only need to consider applying a magnetic field in $[001]$ direction.

Table. S 10: Subgroups of $I4'/m'm'm$ (139.536), the magnetic space group of $BaMn_2P_2$.

Subgroup & SI Group	Perturbation
$C2'$ (5.15) $\mathbb{Z}_1$	• $\boldsymbol{B} \parallel [001]$ and strain $\perp [110]$<br>• $\boldsymbol{B} \perp [110]$<br>• $\boldsymbol{E} \parallel [110]$ and strain $\perp [110]$<br>• $\boldsymbol{E} \parallel [110]$ and $\boldsymbol{B} \parallel [001]$
Cm' (8.34) $\mathbb{Z}_1$	• $\boldsymbol{B} \parallel [100]$ and strain $\parallel [110]$<br>• $\boldsymbol{B} \parallel [100]$ and strain $\perp [001]$<br>• $\boldsymbol{B} \parallel [110]$ and strain $\parallel [100]$<br>• $\boldsymbol{B} \parallel [110]$ and strain $\perp [001]$<br>• $\boldsymbol{B} \perp [001]$<br>• $\boldsymbol{E} \parallel [100]$ and strain $\parallel [110]$<br>• $\boldsymbol{E} \parallel [100]$ and strain $\perp [001]$<br>• $\boldsymbol{E} \parallel [100]$ and $\boldsymbol{B} \parallel [110]$<br>• $\boldsymbol{E} \parallel [110]$ and strain $\parallel [100]$<br>• $\boldsymbol{E} \parallel [110]$ and strain $\perp [001]$<br>• $\boldsymbol{E} \parallel [110]$ and $\boldsymbol{B} \parallel [100]$<br>• $\boldsymbol{E} \parallel [110]$ and $\boldsymbol{B} \parallel [110]$<br>• $\boldsymbol{E} \perp [001]$
$Fm'm2'$ (42.221) $\mathbb{Z}_1$	• $\boldsymbol{E} \parallel [110]$
Cm' (8.34) $\mathbb{Z}_1$	• $\boldsymbol{E} \parallel [001]$ and $\boldsymbol{B} \parallel [100]$<br>• $\boldsymbol{E} \parallel [100]$ and $\boldsymbol{B} \parallel [001]$
$C2$ (5.13) $\mathbb{Z}_1$	• $\boldsymbol{B} \parallel [100]$ and strain $\perp [100]$<br>• $\boldsymbol{E} \parallel [100]$ and strain $\perp [100]$

Continued on next page

…continued

$Im'm'2$ (44.232) $\mathbb{Z}_1$	• $\boldsymbol{B} \parallel [100]$<br>• $\boldsymbol{E} \parallel [100]$
Cm (8.32) $\mathbb{Z}_1$	• $\boldsymbol{B} \parallel [110]$ and strain $\perp [110]$<br>• $\boldsymbol{E} \parallel [001]$ and strain $\perp [110]$<br>• $\boldsymbol{E} \parallel [001]$ and $\boldsymbol{B} \parallel [110]$<br>• $\boldsymbol{E} \perp [110]$
$Fm'm2'$ (42.221) $\mathbb{Z}_1$	• $\boldsymbol{B} \parallel [110]$
Cm' (8.34) $\mathbb{Z}_1$	• $\boldsymbol{B} \parallel [001]$ and strain $\perp [100]$<br>• $\boldsymbol{B} \perp [100]$<br>• $\boldsymbol{E} \parallel [001]$ and strain $\perp [100]$<br>• $\boldsymbol{E} \perp [100]$
$C2$ (5.13) $\mathbb{Z}_1$	• $\boldsymbol{B} \parallel [001]$ and strain $\perp [001]$<br>• $\boldsymbol{E} \parallel [001]$ and strain $\perp [001]$<br>• $\boldsymbol{E} \parallel [001]$ and $\boldsymbol{B} \parallel [001]$ and strain $\parallel [110]$
$Fmm2$ (42.219) $\mathbb{Z}_1$	• $\boldsymbol{E} \parallel [001]$ and strain $\parallel [110]$
$F2'2'2$ (22.47) $\mathbb{Z}_1$	• $\boldsymbol{B} \parallel [001]$ and strain $\parallel [110]$
$Im'm'2$ (44.232) $\mathbb{Z}_1$	• $\boldsymbol{B} \parallel [001]$ and strain $\parallel [100]$<br>• $\boldsymbol{E} \parallel [001]$ and strain $\parallel [100]$<br>• $\boldsymbol{E} \parallel [001]$ and $\boldsymbol{B} \parallel [001]$
$I4'm'm$ (107.229) $\mathbb{Z}_1$	• $\boldsymbol{E} \parallel [001]$
$I\bar{4}m'2'$ (119.319) $\mathbb{Z}_2 \times \mathbb{Z}_2 \times \mathbb{Z}_2$	• $\boldsymbol{B} \parallel [001]$
$P\bar{1}'$ (2.6) $\mathbb{Z}_1$	• strain in generic direction
$C2'/m$ (12.60) $\mathbb{Z}_1$	• strain $\perp [110]$
$C2/m'$ (12.61) $\mathbb{Z}_1$	• strain $\perp [100]$
$C2/m'$ (12.61) $\mathbb{Z}_1$	• strain $\perp [001]$
$Fm'mm$ (69.523) $\mathbb{Z}_1$	• strain $\parallel [110]$
$Im'm'm'$ (71.537) $\mathbb{Z}_1$	• strain $\parallel [100]$

S4.1 Notation

- The expression "$\boldsymbol{E} \perp [100]$" is short for "$\boldsymbol{E}$ is perpendicular to $[100]$ *and is not parallel to any other high symmetry direction in the crystal family*."

- "strain" is short for "uniaxial strain."

- For both hexagonal and trigonal space groups, the conventional hexagonal axes are used.

S4.2 Subgroups and their classification

For a complete list of subgroups of any magnetic space group, we refer the reader to the tables in the auxiliary supplementary materials.

Section S5. MSGs which have only trivially classified subgroups

In Table. S 11 we list all magnetic space groups with the property that every subgroup realizable by symmetry-breaking $\boldsymbol{B}/\boldsymbol{E}$-fields and/or mechanical strains has a trivial classification.

Table. S 11: MSGs whose subgroups have only trivial symmetry indicator groups

$P11'$ (1.2)	$Pm'm2'$ (25.59)	$Abm2$ (39.195)	$P4_1$ (76.7)
P_S1 (1.3)	$Pmc2_1$ (26.66)	$Ab'm2'$ (39.197)	$P4_11'$ (76.8)
$P\bar{1}$ (2.4)	$Pmc2_11'$ (26.67)	$Abm'2'$ (39.198)	$P4_1'$ (76.9)
$P\bar{1}'$ (2.6)	$Pm'c2_1'$ (26.68)	$Ab'm'2$ (39.199)	$P4_3$ (78.19)
$P2'$ (3.3)	$Pmc'2_1'$ (26.69)	A_Bbm2 (39.202)	$P4_31'$ (78.20)
P_a2_1 (4.10)	$Pm'c'2_1$ (26.70)	$Ama2$ (40.203)	P_c4_3 (78.22)
P_C2_1 (4.12)	P_bmc2_1 (26.72)	$Ama21'$ (40.204)	P_C4_3 (78.23)
$P2_1$ (4.7)	P_cmc2_1 (26.73)	$Am'a2'$ (40.205)	P_I4_3 (78.24)
$P2_11'$ (4.8)	P_Amc2_1 (26.74)	$Ama'2'$ (40.206)	$I4$ (79.25)
$P2_1'$ (4.9)	P_Bmc2_1 (26.75)	$Am'a'2$ (40.207)	$I4'$ (79.27)
$C2$ (5.13)	P_Cmc2_1 (26.76)	A_ama2 (40.208)	$I4_1$ (80.29)
$C21'$ (5.14)	P_Imc2_1 (26.77)	$Ab'a2'$ (41.213)	$I4_11'$ (80.30)
$C2'$ (5.15)	$Pc'c2'$ (27.80)	$Aba'2'$ (41.214)	$I\bar{4}$ (82.39)
C_c2 (5.16)	$Pm'a2'$ (28.89)	$Ab'a'2$ (41.215)	$I4_1/a'$ (88.84)
C_a2 (5.17)	$Pma'2'$ (28.90)	A_Bba2 (41.218)	$P4_12'2'$ (91.106)
Pm (6.18)	$Pca2_11'$ (29.100)	$Fmm2$ (42.219)	$P4_1'2'2$ (91.107)
$Pm1'$ (6.19)	$Pca'2_1'$ (29.102)	$Fm'm'2$ (42.222)	$P4_12_121'$ (92.112)
Pm' (6.20)	$Pc'a'2_1$ (29.103)	$Fdd2$ (43.224)	$P4_1'2_12'$ (92.113)

Continued on next page

. . . continued

P_bm (6.22)	P_aca2_1 (29.104)	$Fdd21'$ (43.225)	$P4_12'_12'$ (92.114)
P_Cm (6.23)	P_bca2_1 (29.105)	$Fd'd2'$ (43.226)	$P4'_12'_12$ (92.115)
Pc (7.24)	P_cca2_1 (29.106)	$Fd'd'2$ (43.227)	$P_c4_12_12$ (92.116)
$Pc1'$ (7.25)	P_Aca2_1 (29.107)	$Imm2$ (44.229)	$P_I4_12_12$ (92.118)
Pc' (7.26)	P_Bca2_1 (29.108)	$Imm21'$ (44.230)	$P4_32'2'$ (95.138)
P_ac (7.27)	P_Cca2_1 (29.109)	$Im'm'2$ (44.232)	$P4'_32'2$ (95.139)
P_cc (7.28)	P_Ica2_1 (29.110)	I_amm2 (44.234)	$P4_32_12$ (96.143)
P_bc (7.29)	$Pca2_1$ (29.99)	$Iba2$ (45.235)	$P4_32_121'$ (96.144)
P_Cc (7.30)	$Pn'c2'$ (30.113)	$Ib'a2'$ (45.237)	$P4'_32_12'$ (96.145)
Cm (8.32)	$Pnc'2'$ (30.114)	$Ib'a'2$ (45.238)	$P4_32'_12'$ (96.146)
$Cm1'$ (8.33)	$Pmn2_1$ (31.123)	$Ima21'$ (46.242)	$P4'_32'_12$ (96.147)
Cm' (8.34)	$Pmn2_11'$ (31.124)	$Im'a2'$ (46.243)	$P_c4_32_12$ (96.148)
C_cm (8.35)	$Pm'n2'_1$ (31.125)	$Ima'2'$ (46.244)	$P_I4_32_12$ (96.150)
C_am (8.36)	$Pmn'2'_1$ (31.126)	$Im'a'2$ (46.245)	$I4'22'$ (97.153)
Cc (9.37)	$Pm'n'2_1$ (31.127)	I_ama2 (46.247)	$I4'2'2$ (97.155)
$Cc1'$ (9.38)	P_amn2_1 (31.128)	$Pnn'a$ (52.308)	$I4_122$ (98.157)
Cc' (9.39)	P_bmn2_1 (31.129)	$Pmna'$ (53.325)	$I4_1221'$ (98.158)
C_cc (9.40)	P_cmn2_1 (31.130)	$Pc'ca$ (54.339)	$I4'_122'$ (98.159)
$P2'/m$ (10.44)	P_Bmn2_1 (31.132)	$Pb'am$ (55.355)	$I4_12'2'$ (98.160)
$P2'_1/m$ (11.52)	P_Cmn2_1 (31.133)	$Pc'cn$ (56.367)	$I4'm'm$ (107.229)
$P2_1/m'$ (11.53)	P_Imn2_1 (31.134)	$Pbc'm$ (57.380)	$I4_1md$ (109.239)
$C2'/m$ (12.60)	$Pb'a2'$ (32.137)	$Pn'nm$ (58.395)	$I4_1md1'$ (109.240)
$P2'/c$ (13.67)	$Pna2_1$ (33.144)	$Pm'mn$ (59.407)	$I4'_1md'$ (109.242)
$P2'_1/c$ (14.77)	$Pna2_11'$ (33.145)	$Pb'cn$ (60.419)	$I4_1m'd'$ (109.243)
$P2_1/c'$ (14.78)	$Pn'a2'_1$ (33.146)	$Pb'ca$ (61.435)	$I\bar{4}'m'2$ (119.317)
$C2'/c$ (15.87)	$Pna'2'_1$ (33.147)	$Pb'c'a'$ (61.437)	$I\bar{4}'m2'$ (119.318)
$C2/c'$ (15.88)	$Pn'a'2_1$ (33.148)	$Pn'ma$ (62.443)	$I\bar{4}m'2'$ (119.319)
$P2'2'2_1$ (17.9)	P_ana2_1 (33.149)	$Pnm'a$ (62.444)	$I\bar{4}'2'm$ (121.329)
$P2_12'_12'$ (18.19)	P_bna2_1 (33.150)	$Pnma'$ (62.445)	$I\bar{4}'2'd$ (122.335)
$P2_12_12_1$ (19.25)	P_Ana2_1 (33.152)	$Pn'm'a'$ (62.449)	$I\bar{4}'2d'$ (122.336)
$P2_12_12_11'$ (19.26)	P_Bna2_1 (33.153)	$Cm'cm$ (63.459)	$I\bar{4}2'd'$ (122.337)
$P2'_12'_12_1$ (19.27)	P_Cna2_1 (33.154)	$Cmc'm$ (63.460)	$I4_1/a'md$ (141.553)
$P_c2_12_12_1$ (19.28)	P_Ina2_1 (33.155)	$Cm'c'm'$ (63.465)	$I4_1/a'm'd'$ (141.559)
$P_C2_12_12_1$ (19.29)	$Pn'n2'$ (34.158)	$Cmc'a$ (64.472)	$P3_1$ (144.4)
$P_I2_12_12_1$ (19.30)	$Cm'm2'$ (35.167)	$Cmca'$ (64.473)	$P3_11'$ (144.5)
$C222_11'$ (20.32)	$Cmc2_1$ (36.172)	$Cm'mm$ (65.483)	P_c3_1 (144.6)
$C2'2'2_1$ (20.33)	$Cmc2_11'$ (36.173)	$Cc'cm$ (66.493)	$P3_2$ (145.7)
$C22'2'_1$ (20.34)	$Cm'c2'_1$ (36.174)	$Cm'ma$ (67.503)	$P3_21'$ (145.8)
C_c222_1 (20.35)	$Cmc'2'_1$ (36.175)	$Cc'ca$ (68.513)	P_c3_2 (145.9)

Continued on next page

...continued

C_A222_1 (20.37)	$Cm'c'2_1$ (36.176)	$Fm'mm$ (69.523)	$R3$ (146.10)
$F222$ (22.45)	C_cmc2_1 (36.177)	$Fd'dd$ (70.529)	R_I3 (146.12)
$F2221'$ (22.46)	C_Amc2_1 (36.179)	$Im'mm$ (71.535)	$R\bar{3}'$ (148.19)
$F2'2'2$ (22.47)	$Cc'c2'$ (37.182)	$Im'm'm'$ (71.537)	$P3_112$ (151.29)
$I222$ (23.49)	$Amm2$ (38.187)	$Im'ma$ (74.556)	$P3_1121'$ (151.30)
$I2221'$ (23.50)	$Amm21'$ (38.188)	$Imma'$ (74.557)	P_c3_112 (151.32)
$I2_12_12_1$ (24.53)	$Am'm2'$ (38.189)	$Im'm'a'$ (74.560)	$P3_121$ (152.33)
$I2_12_12_11'$ (24.54)	$Amm'2'$ (38.190)	P_c4_1 (76.10)	$P3_1211'$ (152.34)
$I2_1'2_1'2_1$ (24.55)	A_amm2 (38.192)	P_I4_1 (76.12)	$P3_12'1$ (152.35)
P_c3_121 (152.36)	R_I3c (161.72)	$P6_12'2'$ (178.159)	$Fd'\bar{3}'$ (203.28)
$P3_212$ (153.37)	$R\bar{3}'m'$ (166.100)	P_c6_122 (178.160)	$Im'\bar{3}'$ (204.32)
$P3_2121'$ (153.38)	$R\bar{3}'m$ (166.99)	$P6_5221'$ (179.162)	$Pa'\bar{3}'$ (205.35)
$P3_212'$ (153.39)	$R\bar{3}'c$ (167.105)	$P6_5'2'2$ (179.163)	$F4'32'$ (209.50)
P_c3_212 (153.40)	$R\bar{3}'c'$ (167.106)	$P6_5'22'$ (179.164)	$F4_132$ (210.52)
$P3_2211'$ (154.42)	$P6_1$ (169.113)	$P6_52'2'$ (179.165)	$F4_1321'$ (210.53)
$P3_22'1$ (154.43)	$P6_11'$ (169.114)	P_c6_522 (179.166)	$F4_1'32'$ (210.54)
P_c3_221 (154.44)	$P6_1'$ (169.115)	$P6_2'2'2$ (180.169)	$I4'32'$ (211.58)
$R32$ (155.45)	P_c6_1 (169.116)	$P6_2'22'$ (180.170)	$P4_332$ (212.59)
$R321'$ (155.46)	$P6_5$ (170.117)	$P6_4'2'2$ (181.175)	$P4_3321'$ (212.60)
$R32'$ (155.47)	$P6_51'$ (170.118)	$P6_4'22'$ (181.176)	P_I4_332 (212.62)
R_I32 (155.48)	$P6_5'$ (170.119)	$P6_3m'c'$ (186.207)	$P4_132$ (213.63)
$P31m'$ (157.55)	P_c6_5 (170.120)	$F23$ (196.4)	$P4_1321'$ (213.64)
$P3c'1$ (158.59)	$P6_2'$ (171.123)	$F231'$ (196.5)	$P4_1'32'$ (213.65)
$P31c'$ (159.63)	$P6_4'$ (172.127)	$I23$ (197.7)	P_I4_132 (213.66)
$R3m$ (160.65)	$P6_3$ (173.129)	$I231'$ (197.8)	$I4_132$ (214.67)
$R3m1'$ (160.66)	$P6_122$ (178.155)	$P2_131'$ (198.10)	$I4_1321'$ (214.68)
$R3m'$ (160.67)	$P6_1221'$ (178.156)	$P2_13$ (198.9)	$I4_1'32'$ (214.69)
R_I3m (160.68)	$P6_1'2'2$ (178.157)	$I2_13$ (199.12)	$F\bar{4}'3m'$ (216.76)
$R3c$ (161.69)	$P6_1'22'$ (178.158)	$I2_131'$ (199.13)	$Fd'\bar{3}'m'$ (227.132)
$R3c1'$ (161.70)			

Section S6. BCS database materials passing our group-theoretical filters

Listed in Table. S 12 are the magnetic materials in the Bilbao database which passed our filters for magnetic space group and Wyckoff position, as explained in the main text. Entries are

listed in a decreasing order of their transition temperature. A green-colored Wyckoff position label indicates that the induced magnon band representation contains one or more magnon band degeneracies, whereas a black label indicates that the site of the corresponding magnetic atom is incompatible with magnetism (and thus the magnetic moment is $|\mathbf{m}| = \mathbf{0}$.)

Table. S 12: Bilbao database materials that passed our filters for magnetic space group and Wyckoff position.

No.	Compound	SG & MSG	T_c [K]	(WP, $|\boldsymbol{m}|/\mu_B$)	Band Gap [eV]
1	$CsFeO_2$	$Fd\bar{3}m$ (227)<br>$I4_1'/a'm'd$ (141.556)	1055	(**4a**, 3.7)	
2	$RbFeO_2$	$Fd\bar{3}m$ (227)<br>$I4_1'/a'm'd$ (141.556)	1027	(**4a**, 2.75)	1.70 (56)
3	α-Fe_2O_3	$R\bar{3}c$ (167)<br>$C2'/c'$ (15.89)	955	(**8f**, 4.11)	2.2 (57)
4	$MnPt_{0.5}Pd_{0.5}$	$P4/mmm$ (123)<br>C_amma (67.509)	866	(**4g**, 4.09)	
5	$NiFe_2O_4$	$Fd\bar{3}m$ (227)<br>$I4_1/am'd'$ (141.557)	858	(**4a**, 2.41)<br>(**8d**, 3.96)<br>(**8d**, 3.96)	3.7 (58)
6	$CuFeS_2$	$I\bar{4}2d$ (122)<br>$I\bar{4}2d$ (122.333)	823	(**4b**, 3.85)	0.6 (59)
7	$NdFeO_3$	$Pnma$ (62)<br>$Pn'ma'$ (62.448)	760	(**4c**, 0.0)<br>(**4b**, 3.84)	0.183 (60)
8	$TbCr_2Si_2$	$I4/mmm$ (139)<br>$I4'/m'm'm$ (139.536)	758	(2a,0.0)<br>(**4d**, 1.62)	
9	$BaFeO_{2.5}$	$P2_1/c$ (14)<br>P_a2_1/c (14.80)	720	(**8e**, 4.16)<br>(**8e**, 3.22)<br>(**8e**, 2.88)<br>(**8e**, 3.46)<br>(**8e**, 4.08)<br>(**8e**, 3.86)<br>(**8e**, 4.39)	

Continued on next page

. . . continued

No.	Compound	SG & MSG	T_c [K]	(WP, $\|\boldsymbol{m}\|/\mu_B$)	Band Gap [eV]
10	$CeFeO_3$	$Pbnm$ (62)<br>$Pn'ma'$ (62.448)	720	(**4c**, 0.0)<br>(**4b**, 3.85)	1.77 (*61*)
11	$HoCr_2Si_2$	$I4/mmm$ (139)<br>$I4'/m'm'm$ (139.536)	718	(2a,0.0)<br>(**4d**, 1.6)	
12	$HoCr_2Si_2$	$I4/mmm$ (139)<br>$I4'/m'm'm$ (139.536)	718	(**4d**, 1.6)<br>(2a,0.0)	
13	$SrFeO_2F$	$Pm\bar{3}m$ (221)<br>I_c4/mcm (140.550)	710	(**4a**, 4.04)	2.4 (*62*)
14	$La_{0.5}Sr_{0.5}FeO_{2.5}F_{0.5}$	$Pnma$ (062)<br>$Pn'ma'$ (62.448)	700	(**4a**, 3.5)	
15	$ErCr_2Si_2$	$I4/mmm$ (139)<br>$I4'/m'm'm$ (139.536)	692	(2a,0.0)<br>(**4d**, 1.41)	
16	$TbFeO_3$	$Pbnm$ (62)<br>$Pn'ma'$ (62.448)	681	(**4b**, 4.8)<br>(**4c**, 0.0)	1.95 (*63*)
17	$SmFeO_3$	$Pnma$ (62)<br>$Pn'ma'$ (62.448)	670	(**4b**, 2.8)	1.094 (*64*)
18	$EuMn_2Ge_2$	$I4/mmm$ (139)<br>$I4'/m'm'm$ (139.536)	667	(2a,0.0)<br>(**4d**, 0.0)	
19	$Sr_2Fe_{1.9}Cr_{0.1}O_5$	$Icmm$ (74)<br>P_Imna (53.336)	650	(**8i**, 3.9)<br>(**4a**, 3.2)<br>(**4a**, 3.2)	
20	$BaMn_2As_2$	$I4/mmm$ (139)<br>$I4'/m'm'm$ (139.536)	625	(**4d**, 3.88)	0.03 (*65*)
21	$CrSb$	$P6_3/mmc$ (194)<br>$P6_3'/m'm'c$ (194.268)	600	(**2a**, 2.45)	
22	$AlCr_2$	$I4/mmm$ (139)<br>P_A2_1/c (14.83)	598	(**4i**, 0.91)	
23	$Sr_2Cr_3As_2O_2$	$I4/mmm$ (139)<br>P_I4_2/mnm (136.506)	590	(2a,0.0)<br>(**4d**, 1.97)	
24	$SrCr_2As_2$	$I4/mmm$ (139)<br>$I4'/m'm'm$ (139.536)	590	(**4d**, 1.9)	

Continued on next page

...continued

No.	Compound	SG & MSG	T_c [K]	(WP, $\|\boldsymbol{m}\|/\mu_B$)	Band Gap [eV]
25	$BaCr_2As_2$	$I4/mmm$ (139)<br>$I4'/m'm'm$ (139.536)	580	(**4d**, 1.88)	
26	Mn_2As	$P4/nmm$ (129)<br>P_anma (62.450)	573	(**4a**, 3.7)<br>(**4b**, 3.5)	
27	$SrRu_2O_6$	$P\bar{3}1m$ (162)<br>$P_c\bar{3}1m$ (162.78)	565	(**4d**, 1.3)	0.4 (*66*)
28	$SrMnSb_2$	$Pnma$ (62)<br>$Pn'ma'$ (62.448)	565	(**4c**, 0.28)	
29	$SrRu_2O_6$	$P\bar{3}1m$ (162)<br>$P_c\bar{3}1m$ (162.78)	563	(**4d**, 1.42)	
30	$K_yFe_{2-x}Se_2$	$I4/m$ (87)<br>$I4/m'$ (87.78)	560	(4d,0.0)<br>(**16i**, 2.55)	
31	$Rb_yFe_{2-x}Se_2$	$I4/m$ (87)<br>$I4/m'$ (87.78)	560	(4d,0.0)<br>(**16i**, 2.15)	
32	$K_{0.8}Fe_{1.8}Se_2$	$I4/m$ (87)<br>$I4/m'$ (87.78)	559	(4d,0.0)<br>(**16i**, 3.31)	
33	YFe_6Sn_6	$Immm$ (71)<br>P_Innn (48.264)	558	(**4f**, 2.03)<br>(**8k**, 2.03)<br>(**8n**, 2.03)<br>(**16o**, 2.03)	
34	$CaMn_2Ge_2$	$I4/mmm$ (139)<br>$I4'/m'm'm$ (139.536)	546	(**4d**, 2.6)	
35	$CaMn_2Ge_2$	$I4/mmm$ (139)<br>$I4'/m'm'm$ (139.536)	546	(**4d**, 2.67)	
36	$Sr_2Co_2O_5$	$Ima2$ (46)<br>P_Inc2 (30.122)	537	(**4a**, 3.12)<br>(**4b**, 2.88)	1.10 (*67*)
37	NiO	$Fm\bar{3}m$ (225)<br>C_c2/c (15.90)	523	(**4c**, 2.44)	4.0 (*68*)
38	Mn_2FeReO_6	$P2_1/c$ (14)<br>$P2_1'/c'$ (14.79)	520	(**4e**, 0.0)<br>(**2c**, 3.97)<br>(**2b**, 0.17)	

Continued on next page

…continued

No.	Compound	SG & MSG	T_c [K]	(WP, $\|\boldsymbol{m}\|/\mu_B$)	Band Gap [eV]
39	Mn_2FeReO_6	$P2_1/c$ (14)<br>$P2_1'/c'$ (14.79)	520	(**4e**, 0.0)<br>(**2c**, 3.60)<br>(**2b**, 0.15)	
40	Mn_2FeReO_6	$P2_1/c$ (14)<br>$P2_1'/c'$ (14.79)	520	(**4e**, 0.0)<br>(**2c**, 3.48)<br>(**2b**, 0.48)	
41	$ScMn_6Ge_6$	$P6/mmm$ (191)<br>P_c6/mcc (192.252)	516	(**12i**, 2.08)	
42	$ScMn_6Ge_6$	$P6/mmm$ (191)<br>P_c6/mcc (192.252)	516	(**12i**, 1.96)	
43	$Pb_{0.7}Bi_{0.3}Fe_{0.762}W_{0.231}O_3$	$Pm\bar{3}m$ (221)<br>I_c4/mcm (140.550)	504	(**4a**, 1.55)	
44	$TlFe_{1.6}Se_2$	$I4/m$ (87)<br>$I4/m'$ (87.78)	500	(**16i**, 3.14)	
45	$BaCo_2Fe_{16}O_{27}$	$P6_3/mmc$ (194)<br>$P6_3/mm'c'$ (194.270)	490	(**2d**, 3.9)<br>(**4f**, 4.9)<br>(**6g**, 4.1)<br>(**4e**, 4.7)<br>(**4f**, 4.5)<br>(**4f**, 4.2)<br>(**12k**, 4.8)	
46	YFe_6Ge_6	$Cmcm$ (63)<br>P_Bnna (52.318)	486	(**8d**, 1.42)<br>(**8e**, 1.42)<br>(**8g**, 1.42)	
47	YMn_6Ge_6	$P6/mmm$ (191)<br>P_c6/mcc (192.252)	482	(**12i**, 1.82)	
48	$SmFeO_3$	$Pbnm$ (62)<br>$Pn'm'a$ (62.446)	480	(**4b**, 3.69)	
49	$ErMn_2Ge_2$	$I4/mmm$ (139)<br>P_I4/nnc (126.386)	475	(2a,0.0)<br>(**4d**, 2.21)	
50	$YBaCuFeO_5$	$P4/mmm$ (123)<br>F_Smmm (69.526)	475	(**16s**, 0.96)<br>(**16s**, 2.77)	

Continued on next page

...continued

No.	Compound	SG & MSG	T_c [K]	(WP, $\|m\|/\mu_B$)	Band Gap [eV]
51	$YBaCuFeO_5$	$P4/mmm$ (123)<br>C_a2/m (12.64)	475	(**8n**, 0.0)<br>(**8n**, 1.45)	
52	Mn_3Pt	$Pm\bar{3}m$ (221)<br>P_c4_2/mcm (132.456)	475	(**4f**, 3.4)<br>(2d,0.0)	
53	$SrFeO_2$	$P4/mmm$ (123)<br>F_Smmm (69.526)	473	(**8g**, 3.1)	1.2 (*69*)
54	$ErFe_6Ge_6$	$Immm$ (71)<br>P_Innn (48.264)	470	(2a,0.0)<br>(**4h**, 0.0)<br>(2b,0.0)<br>(**4g**, 0.0)<br>(**4f**, 1.35)<br>(**8k**, 1.35)<br>(**8n**, 1.35)<br>(**16o**, 1.35)	
55	$BaFe_{12}O_{19}$	$P6_3/mmc$ (194)<br>$P6_3/mm'c'$ (194.270)	470	(**2a**, 4.1)<br>(**2b**, 4.8)<br>(**4f**, 4.5)<br>(**4f**, 4.2)<br>(**12k**, 4.9)	
56	$BaMn_2Ge_2$	$I4/mmm$ (139)<br>$I4'/m'm'm$ (139.536)	465	(**4d**, 3.6)	
57	$BaMn_2Ge_2$	$I4/mmm$ (139)<br>$I4'/m'm'm$ (139.536)	465	(**4d**, 3.66)	
58	YMn_2Si_2	$I4/mmm$ (139)<br>P_I4/nnc (126.386)	460	(**4d**, 2.4)	
59	$Mn_{2.85}Ga_{1.15}$	$P6_3/mmc$ (194)<br>$P6_3'/m'm'c$ (194.268)	460	(**6h**, 3.32)	
60	CoF_3	$R\bar{3}c$ (167)<br>$R\bar{3}c$ (167.103)	460	(**6b**, 3.21)	0.45 (*70*)

Continued on next page

…continued

No.	Compound	SG & MSG	T_c [K]	(WP, $\|\boldsymbol{m}\|/\mu_B$)	Band Gap [eV]
61	$BaMg_2Fe_{16}O_{27}$	$P6_3/mmc$ (194)<br>$P6_3/mm'c'$ (194.270)	452	(**2d**, 3.8)<br>(**4f**, 4.1)<br>(**6g**, 3.0)<br>(**4e**, 4.1)<br>(**4f**, 4.0)<br>(**4f**, 5.0)<br>(**12k**, 4.6)	
62	$Ni_{1.64}Co_{0.36}Mn_{1.28}Ga_{0.72}$	$I4/mmm$ (139)<br>P_I4_2/mnm (136.506)	450	(2a,0.0)<br>(2b,0.0)<br>(**4d**, 0.97)<br>(**4d**, 0.97)	
63	$LaMn_2Si_2$	$I4/mmm$ (139)<br>$I4'/m'm'm$ (139.536)	450	(**4d**, 2.21)	
64	$Pb_5Fe_3TiO_{11}Cl$	$P4/mmm$ (123)<br>P_Bmma (51.302)	450	(**4l**, 4.05)<br>(**4l**, 3.86)	
65	$YBaCuFeO_5$	$P4mm$ (99)<br>F_Smm2 (42.223)	446	(**8c**, 2.51)<br>(**8c**, 0.0)	
66	$BaMn_2Sb_2$	$I4/mmm$ (139)<br>$I4'/m'm'm$ (139.536)	443	(**4d**, 3.83)	
67	$YBaCuFeO_5$	$P4/mmm$ (123)<br>C_a2/m (12.64)	441	(**8n**, 0.0)<br>(**8n**, 3.03)	
68	$NdMnSi_2$	$Cmcm$ (63)<br>$Cm'cm'$ (63.464)	441	(**4c**, 0.0)<br>(**4c**, 2.06)	
69	$NdMnSi_2$	$Cmcm$ (63)<br>$Cm'cm'$ (63.464)	441	(**4c**, 0.0)<br>(**4c**, 2.48)	
70	$CeCo_2P_2$	$I4/mmm$ (139)<br>P_I4/nnc (126.386)	440	(2a,0.0)<br>(**4d**, 0.94)	
71	$Pb_{0.8}Bi_{0.2}Fe_{0.728}W_{0.264}O_3$	$Pm\bar{3}m$ (221)<br>I_c4/mcm (140.550)	435	(**4a**, 1.4)	
72	$PrMnSi_2$	$Cmcm$ (63)<br>$Cm'cm'$ (63.464)	434	(**4c**, 0.0)<br>(**4c**, 1.93)	

Continued on next page

. . . continued

No.	Compound	SG & MSG	T_c [K]	(WP, $\|\boldsymbol{m}\|/\mu_B$)	Band Gap [eV]
73	$PrMnSi_2$	$Cmcm$ (63)<br>$Cm'cm'$ (63.464)	434	(**4c**, 0.0)<br>(**4c**, 2.06)	
74	$Nd_6Fe_{13}Sn$	$I4/mcm$ (140)<br>P_I4/mcc (124.362)	433	(**8f**, 2.47)<br>(**16l**, 1.94)<br>(**4d**, 2.2)<br>(**16k**, 2.4)<br>(**16l**, 2.2)<br>(**16l**, 2.32)	
75	$Pb_2BaCuFeO_5Br$	$P4/mmm$ (123)<br>I_bmma (74.562)	423	(**8k**, 1.01)	
76	$YBa_2Cu_3O_6$	$P4/mmm$ (123)<br>C_ammm (65.489)	420	(4b,0.0)<br>(**8s**, 0.54)	
77	Mn_3As	$P6_3/mmc$ (194)<br>$Cm'cm'$ (63.464)	420	(**8g**, 4.24)<br>(**4c**, 6.70)	
78	Mn_3As	$P6_3/mmc$ (194)<br>$Cmc'm'$ (63.463)	420	(**8g**, 4.24)<br>(**4c**, 3.0)	
79	Mn_3Sn	$P6_3/mmc$ (194)<br>$Cm'cm'$ (63.464)	420	(**8g**, 2.44)<br>(**4c**, 3.86)	
80	Mn_3Sn	$P6_3/mmc$ (194)<br>$Cmc'm'$ (63.463)	420	(**8g**, 4.24)<br>(**4c**, 3.0)	
81	$Li_{0.5}FeCr_{1.5}O_4$	$Fd\bar{3}m$ (227)<br>$I4_1/am'd'$ (141.557)	417	(**4a**, 3.9)<br>(**8d**, 1.4)	
82	FeGe	$P6/mmm$ (191)<br>P_c6/mcc (192.252)	410	(**6f**, 1.67)	
83	$YBa_2Cu_3O_{6+d}$	$P4/mmm$ (123)<br>C_ammm (65.489)	410	(4b,0.0)<br>(**8s**, 1.0)	
84	YMn_2Si_2	$I4/mmm$ (139)<br>P_I4/nnc (126.386)	410	(**4d**, 2.21)	
85	$NaOsO_3$	$Pnma$ (62)<br>$Pn'ma'$ (62.448)	410	(**4b**, 1.0)	0.1 (71)

Continued on next page

...continued

No.	Compound	SG & MSG	T_c [K]	(WP, $\|\boldsymbol{m}\|/\mu_B$)	Band Gap [eV]
86	U_2N_2As	$P\bar{3}m1$ (164)<br>$P_c\bar{3}c1$ (165.96)	406	(**4d**, 1.6)	
87	$CeMnSi_2$	$Cmcm$ (63)<br>$Cm'cm'$ (63.464)	398	(**4c**, 0.0)<br>(**4c**, 2.18)	
88	$CeMnSi_2$	$Cmcm$ (63)<br>$Cm'cm'$ (63.464)	398	(**4c**, 0.23)<br>(**4c**, 2.24)	
89	YMn_2Ge_2	$I4/mmm$ (139)<br>P_I4/nnc (126.386)	395	(**4d**, 2.95)	
90	FeF_3	$R\bar{3}c$ (167)<br>$C2'/c'$ (15.89)	394	(**4d**, 4.45)	5.96 (72)
91	LiMnAs	$P4/nmm$ (129)<br>P_c4_2/ncm (138.528)	393	(**4b**, 1.59)	0.2–0.57 (73)
92	LiMnAs	$P4/nmm$ (129)<br>P_c4_2/ncm (138.528)	393	(**4b**, 2.59)	
93	LiMnAs	$P4/nmm$ (129)<br>P_c4_2/ncm (138.528)	393	(**4b**, 3.75)	
94	$Pr_6Fe_{13}Sn$	$I4/mcm$ (140)<br>P_Ibcn (60.432)	393	(**8h**, 2.91)<br>(**16k**, 2.73)<br>(**4d**, 2.3)<br>(**8j**, 2.57)<br>(**8j**, 2.57)<br>(**16k**, 2.42)<br>(**16k**, 2.42)<br>(**16k**, 2.54)	
95	Cr_2As	$P4/nmm$ (129)<br>P_anma (62.450)	393	(**4a**, 0.4)<br>(**4b**, 1.34)	
96	Mn_3B_4	$Immm$ (71)<br>P_Innm (58.404)	392	(**2c**, 2.92)<br>(**4g**, 0.44)	
97	$EuMn_2Si_2$	$I4/mmm$ (139)<br>P_I4/nnc (126.386)	391	(2a,0.0)<br>(**4d**, 1.97)	
98	Mn_2AlB_2	$Cmmm$ (65)<br>C_cmcm (63.466)	390	(**8g**, 0.71)	

Continued on next page

…continued

No.	Compound	SG & MSG	T_c [K]	(WP, $\|m\|/\mu_B$)	Band Gap [eV]
99	$BaMn_2Bi_2$	$I4/mmm$ (139)<br>$I4'/m'm'm$ (139.536)	390	(**4d**, 3.83)	
100	$LaMnSi_2$	$Cmcm$ (63)<br>$Cm'cm'$ (63.464)	386	(**4c**, 1.77)	
101	$LaMnSi_2$	$Cmcm$ (63)<br>$Cm'cm'$ (63.464)	386	(**4c**, 2.03)	
102	$LaMnSi_2$	$Cmcm$ (63)<br>$Cm'cm'$ (63.464)	386	(**4c**, 2.07)	
103	$Y_2SrCu_{0.6}Co_{1.4}O_{6.5}$	$Ibam$ (72)<br>$Ib'a'm$ (72.543)	385	(**16k**, 1.66)	
104	$Y_2SrCu_{0.6}Co_{1.4}O_{6.5}$	$Ibam$ (72)<br>$Ib'a'm$ (72.543)	385	(**16k**, 2.18)	
105	$CeMn_2Si_2$	$I4/mmm$ (139)<br>P_I4/nnc (126.386)	384	(2a,0.0)<br>(**4d**, 2.01)	
106	$NdMn_2Si_2$	$I4/mmm$ (139)<br>P_I4/nnc (126.386)	382	(2a,0.0)<br>(**4d**, 1.92)	
107	$LaSrFeO_4$	$I4/mmm$ (139)<br>P_C4_2/nnm (134.481)	380	(**4d**, 4.04)	0.525 (74)
108	$LaSrFeO_4$	$I4/mmm$ (139)<br>P_C4_2/ncm (138.529)	380	(**4d**, 4.04)	
109	$NdMn_2Si_2$	$I4/mmm$ (139)<br>P_I4/nnc (126.386)	380	(2a,0.0)<br>(**4d**, 2.57)	
110	$CeMn_2Si_2$	$I4/mmm$ (139)<br>P_I4/nnc (126.386)	380	(2a,0.0)<br>(**4d**, 1.9)	
111	$LaSrFeO_4$	$I4/mmm$ (139)<br>C_Amca (64.480)	380	(**4a**, 5.37)	
112	$Mn_3Cu_{0.5}Ge_{0.5}N$	$Pm\bar{3}m$ (221)<br>$R\bar{3}m$ (166.97)	380	(**9e**, 2.47)	
113	$LaCrO_3$	$R\bar{3}c$ (167)<br>$R\bar{3}c$ (167.103)	380	(**6b**, 1.67)	

Continued on next page

…continued

No.	Compound	SG & MSG	T_c [K]	(WP, $\|m\|/\mu_B$)	Band Gap [eV]
114	Mn_3Ge	$P6_3/mmc$ (194)<br>$Cm'cm'$ (63.464)	380	(**8g**, 3.42)<br>(**4c**, 2.16)	
115	$CeMn_2Si_2$	$I4/mmm$ (139)<br>P_I4/nnc (126.386)	379	(2a,0.0)<br>(**4d**, 2.3)	
116	$FeSn_2$	$I4/mcm$ (140)<br>P_Ibcn (60.432)	378	(**4a**, 1.15)	
117	$LaCaFeO_4$	$I4/mmm$ (139)<br>P_C4_2/ncm (138.529)	373	(**4d**, 4.61)	
118	FeSn	$P6/mmm$ (191)<br>C_cmcm (63.466)	373	(**4b**, 1.5)<br>(**8e**, 1.5)	
119	$LiErF_4$	$I4_1/a$ (88)<br>P_C2_1/c (14.84)	373	(**4e**, 2.2)	
120	$LaCaFeO_4$	$Cmce$ (64)<br>$Cm'c'a$ (64.474)	373	(**4a**, 3.74)	
121	$PrMn_2Si_2$	$I4/mmm$ (139)<br>P_I4/nnc (126.386)	368	(2a,0.0)<br>(**4d**, 2.03)	
122	U_2N_2P	$P\bar{3}m1$ (164)<br>$P_c\bar{3}c1$ (165.96)	366	(**4d**, 1.7)	
123	$SrNdFeO_4$	$I4/mmm$ (139)<br>C_Accm (66.500)	360	(**8g**, 0.0)<br>(**4b**, 4.11)	
124	CaMnSi	$P4/nmm$ (129)<br>$P4'/n'm'm$ (129.416)	360	(**2a**, 1.96)	
125	CaMnSi	$P4/nmm$ (129)<br>$P4'/n'm'm$ (129.416)	360	(**2a**, 3.27)	
126	$CaFe_5O_7$	$P2_1/m$ (11)<br>$P2_1/m$ (11.50)	360	(**4f**, 2.94)<br>(**4f**, 3.43)<br>(**2a**, 2.01)	
127	NdMnAsO	$P4/nmm$ (129)<br>$P4'/n'm'm$ (129.416)	359	(**2b**, 2.41)<br>(2c,0.0)	
128	$LuMn_6Sn_6$	$P6/mmm$ (191)<br>C_cmcm (63.466)	353	(**16m**, 1.33)<br>(**8l**, 1.33)	

Continued on next page

...continued

No.	Compound	SG & MSG	T_c [K]	(WP, $\|\boldsymbol{m}\|/\mu_B$)	Band Gap [eV]
129	Fe_2As	$P4/nmm$ (129)<br>P_anma (62.450)	353	(**4a**, 0.95)<br>(**4b**, 1.52)	
130	$LaSr_3Fe_3O_9$	$Cmcm$ (63)<br>P_Cbcm (57.391)	350	(**8g**, 3.99)<br>(**4c**, 3.58)	
131	Ca_2FeOsO_6	$P2_1/n$ (14)<br>$P2_1'/c'$ (14.79)	350	(**2c**, 2.58)<br>(**2b**, 1.5)	1.2 (75)
132	$La_{0.75}Bi_{0.25}Fe_{0.5}Cr_{0.5}O_3$	$Pnma$ (62)<br>$Pnma$ (62.441)	350	(**4b**, 3.3)	
133	$PrMn_2Si_2$	$I4/mmm$ (139)<br>P_I4/nnc (126.386)	348	(2a,0.0)<br>(**4d**, 2.48)	
134	$FeBO_3$	$R\bar{3}c$ (167)<br>$C2'/c'$ (15.89)	348	(**4c**, 4.7)	
135	CeMnAsO	$P4/nmm$ (129)<br>$P4'/n'm'm$ (129.416)	347	(2c,0.0)<br>(**2b**, 2.78)	
136	$YbMnSb_2$	$P4/nmm$ (129)<br>$P4'/n'm'm$ (129.416)	345	(**2a**, 3.48)	
137	MnCoGe	$Pnma$ (62)<br>$Pn'm'a$ (62.446)	345	(**4c**, 0.63)<br>(**4c**, 3.54)	
138	$Sr_2Mn_3As_2O_2$	$I4/mmm$ (139)<br>$I4'/m'm'm$ (139.536)	340	(2a,0.0)<br>(**4d**, 3.4)	
139	NdMnAsO	$P4/nmm$ (129)<br>$P4'/n'm'm$ (129.416)	335	(**2a**, 2.35)<br>(2c,0.0)	
140	La_2NiO_4	$Bmeb$ (64)<br>P_Cmna (53.335)	330	(**4a**, 1.6)	
141	Sr_2CoO_3Cl	$P4/nmm$ (129)<br>P_Bccm (49.274)	330	(**4g**, 2.82)	
142	$MnFe_4Si_3$	$P6_3/mcm$ (193)<br>$P6_3/mc'm'$ (193.260)	330	(**4d**, 0.0)<br>(**4d**, 1.0)<br>(**6g**, 1.2)<br>(**6g**, 1.5)	

Continued on next page

... continued

No.	Compound	SG & MSG	T_c [K]	(WP, $\|\boldsymbol{m}\|/\mu_B$)	Band Gap [eV]
143	$MnSn_2$	$I4/mcm$ (140)<br>C_Acca (68.520)	325	(**8f**, 2.36)	
144	MnTe	$P6_3/mmc$ (194)<br>$Cmcm$ (63.457)	323	(**4a**, 6.50)	1.46 (76)
145	Nd_2NiO_4	$Cmce$ (64)<br>P_Cmna (53.335)	320	(**4a**, 1.57)<br>(**8f**, 0.0)	
146	LaMnAsO	$P4/nmm$ (129)<br>$P4'/n'm'm$ (129.416)	317	(**2a**, 3.54)	
147	LaMnAsO	$P4/nmm$ (129)<br>$P4'/n'm'm$ (129.416)	317	(**2a**, 2.43)	
148	La_2CuO_4	$Bmeb$ (64)<br>P_Accn (56.374)	316	(**4a**, 0.42)	1.24 (77)
149	$FeGe_2$	$I4/mcm$ (140)<br>P_Ibcn (60.432)	315	(**4a**, 1.2)	
150	$EuMnBi_2$	$I4/mmm$ (139)<br>$I4'/m'm'm$ (139.536)	315	(4e,0.0)<br>(**4d**, 3.4)	
151	$Ba_2Mn_3Sb_2O_2$	$I4/mmm$ (139)<br>$I4'/m'm'm$ (139.536)	314	(**4d**, 3.44)<br>(2a,0.0)	
152	$Mn_{0.375}Co_{0.375}Fe_{0.25}$	$Fm\bar{3}m$ (225)<br>P_A2_1/c (14.83)	310	(**2a**, 0.66)	
153	$LaCrO_3$	$Pnma$ (62)<br>$Pn'ma'$ (62.448)	310	(**4b**, 0.98)	3.39 (78)
154	$NdCo_2P_2$	$I4/mmm$ (139)<br>P_c4/mcc (124.360)	309	(2b,0.0)<br>(**2c**, 0.74)<br>(**8i**, 0.64)	
155	Mn_5Ge_3	$P6_3/mcm$ (193)<br>$P6_3/mc'm'$ (193.260)	304	(**4d**, 1.96)<br>(**6g**, 3.23)	
156	$MnFe_4Si_3$	$P6_3/mcm$ (193)<br>$P6_3/mc'm'$ (193.260)	302	(**4d**, 0.0)<br>(**4d**, 0.0)<br>(**6g**, 1.4)<br>(**6g**, 1.4)	

Continued on next page

...continued

No.	Compound	SG & MSG	T_c [K]	(WP, $\|\boldsymbol{m}\|/\mu_B$)	Band Gap [eV]
157	$CaMnBi_2$	$P4/nmm$ (129)<br>$P4'/n'm'm$ (129.416)	300	(**2a**, 3.73)	
158	RuO_2	$P4_2/mnm$ (136)<br>$P4_2'/mnm'$ (136.499)	300	(**2a**, 0.05)	
159	$Sr_{0.7}Ho_{0.3}CoO_{2.7}$	$I4/mmm$ (139)<br>$I4'/mmm'$ (139.535)	300	(**8h**, 2.5)<br>(**8f**, 1.5)	
160	$Sr_{0.7}Tb_{0.3}CoO_{2.9}$	$I4/mmm$ (139)<br>$I4'/mmm'$ (139.535)	300	(**8h**, 2.7)<br>(**8f**, 1.4)	
161	$CoFe_3O_5$	$Cmcm$ (63)<br>$Cm'cm'$ (63.464)	300	(**8f**, 2.54)<br>(**4a**, 2.21)<br>(**4c**, 0.0)	
162	$YbMnBi_2$	$P4/nmm$ (129)<br>$P4'/n'm'm$ (129.416)	298	(**2a**, 3.78)	
163	Mn_3GaN	$Pm\bar{3}m$ (221)<br>$R\bar{3}m$ (166.97)	298	(**9e**, 1.17)	
164	$NiTiO_3$	$R\bar{3}$ (148)<br>$P_S\bar{1}$ (2.7)	295	(**4i**, 2.25)<br>(**4i**, 0.0)	
165	CsMnP	$P4/nmm$ (129)<br>P_c4_2/ncm (138.528)	295	(**4b**, 2.86)	
166	CsMnP	$P4/nmm$ (129)<br>P_c4_2/ncm (138.528)	295	(**4b**, 3.55)	
167	CsMnBi	$P4/nmm$ (129)<br>P_c4_2/ncm (138.528)	295	(**4b**, 4.33)	
168	RbMnAs	$P4/nmm$ (129)<br>P_c4_2/ncm (138.528)	295	(**4b**, 4.05)	
169	RbMnP	$P4/nmm$ (129)<br>P_c4_2/ncm (138.528)	295	(**4b**, 3.21)	
170	RbMnP	$P4/nmm$ (129)<br>P_c4_2/ncm (138.528)	295	(**4b**, 3.87)	
171	KMnP	$P4/nmm$ (129)<br>P_c4_2/ncm (138.528)	295	(**4b**, 2.88)	

Continued on next page

…continued

No.	Compound	SG & MSG	T_c [K]	(WP, $\|m\|/\mu_B$)	Band Gap [eV]
172	KMnP	$P4/nmm$ (129)<br>P_c4_2/ncm (138.528)	295	(**4b**, 3.96)	
173	$SrMnBi_2$	$I4/mmm$ (139)<br>$I4'/m'm'm$ (139.536)	295	(**4d**, 3.75)	
174	KMnBi	$P4/nmm$ (129)<br>$P4'/n'm'm$ (129.416)	295	(**2a**, 4.5)	
175	KMnSb	$P4/nmm$ (129)<br>$P4'/n'm'm$ (129.416)	295	(**2a**, 4.1)	
176	KMnAs	$P4/nmm$ (129)<br>P_c4_2/ncm (138.528)	293	(**4b**, 2.93)	
177	KMnAs	$P4/nmm$ (129)<br>P_c4_2/ncm (138.528)	293	(**4b**, 4.03)	
178	RbMnAs	$P4/nmm$ (129)<br>P_c4_2/ncm (138.528)	293	(**4b**, 3.29)	
179	NaMnBi	$P4/nmm$ (129)<br>$P4'/n'm'm$ (129.416)	293	(**2a**, 3.19)	
180	NaMnBi	$P4/nmm$ (129)<br>$P4'/n'm'm$ (129.416)	293	(**2a**, 4.58)	
181	NaMnSb	$P4/nmm$ (129)<br>$P4'/n'm'm$ (129.416)	293	(**2a**, 2.67)	
182	NaMnSb	$P4/nmm$ (129)<br>$P4'/n'm'm$ (129.416)	293	(**2a**, 4.08)	
183	NaMnAs	$P4/nmm$ (129)<br>$P4'/n'm'm$ (129.416)	293	(**2a**, 3.45)	
184	NaMnAs	$P4/nmm$ (129)<br>$P4'/n'm'm$ (129.416)	293	(**2a**, 4.01)	
185	NaMnP	$P4/nmm$ (129)<br>$P4'/n'm'm$ (129.416)	293	(**2a**, 3.21)	
186	NaMnP	$P4/nmm$ (129)<br>$P4'/n'm'm$ (129.416)	293	(**2a**, 3.6)	

Continued on next page

…continued

No.	Compound	SG & MSG	T_c [K]	(WP, $\|\boldsymbol{m}\|/\mu_B$)	Band Gap [eV]
187	NaMnP	$P4/nmm$ (129)<br>$P4'/n'm'm$ (129.416)	293	(**2a**, 3.63)	
188	$Sr_2Cr_3As_2O_2$	$I4/mmm$ (139)<br>P_C2_1/c (14.84)	291	(**4c**, 3.1)<br>(**4e**, 2.19)<br>(**4e**, 2.19)	
189	CoO	$Fm\bar{3}m$ (225)<br>I_c4_1/acd (142.570)	290	(**16e**, 0.99)	
190	CoO	$Fm\bar{3}m$ (225)<br>C_c2/c (15.90)	290	(**4c**, 3.98)	
191	CoO	$Fm\bar{3}m$ (225)<br>C_c2/c (15.90)	290	(**4c**, 0.99)	
192	$Sr_{0.7}Er_{0.3}CoO_{2.8}$	$I4/mmm$ (139)<br>$I4'/mmm'$ (139.535)	290	(**8h**, 2.4)<br>(**8f**, 1.5)	
193	$LaCrO_3$	$Pnma$ (62)<br>$Pnma$ (62.441)	290	(**4b**, 2.51)	
194	$YbMnBi_2$	$P4/nmm$ (129)<br>$P4'/n'm'm$ (129.416)	290	(**2a**, 4.3)	
195	Gd_2CuO_4	$I4/mmm$ (139)<br>C_Accm (66.500)	285	(**4a**, 1.41)	
196	Gd_2CuO_4	$Aeam$ (64)<br>$Cm'ca'$ (64.476)	285	(**4a**, 1.01)	
197	Pr_2CuO_4	$I4/mmm$ (139)<br>P_C4_2/nnm (134.481)	284	(**8i**, 0.08)<br>(**4d**, 0.4)	
198	$BaMnSb_2$	$I4/mmm$ (139)<br>$I4'/m'm'm$ (139.536)	283	(**4d**, 3.95)	
199	Sm_2CuO_4	$I4/mmm$ (139)<br>P_C4_2/ncm (138.529)	280	(**4c**, 0.38)	
200	Sm_2CuO_4	$I4/mmm$ (139)<br>C_Amca (64.480)	280	(**4a**, 0.38)	
201	Nd_2CuO_4	$I4/mmm$ (139)<br>P_C4_2/nnm (134.481)	276	(**4d**, 1.0)	

Continued on next page

...continued

No.	Compound	SG & MSG	T_c [K]	(WP, $\|m\|/\mu_B$)	Band Gap [eV]
202	La_2CoO_4	$Cmce$ (64)<br>P_Cmna (53.335)	275	(**4a**, 2.9)	
203	$KNiF_3$	$Pm\bar{3}m$ (221)<br>I_c4/mcm (140.550)	275	(**4a**, 2.22)	
204	CrN	$Fm\bar{3}m$ (225)<br>P_anma (62.450)	273	(**4b**, 2.40)	
205	Pr_2CuO_4	$I4/mmm$ (139)<br>P_C4_2/nnm (134.481)	270	(**4d**, 0.48)	
206	Pr_2CuO_4	$I4/mmm$ (139)<br>C_Accm (66.500)	270	(**4a**, 0.48)	
207	$Cu_{0.82}Mn_{1.18}As$	$P\bar{6}$ (174)<br>$P\bar{6}'$ (174.135)	270	(**3j**, 3.02)<br>(**3k**, 3.02)<br>(**3k**, 3.02)	
208	$Bi_{0.85}Ca_{0.15}Fe_{0.55}Mn_{0.45}O_3$	$Pnma$ (62)<br>$Pn'm'a$ (62.446)	268	(**4b**, 2.21)<br>(**4b**, 2.21)	
209	Eu_2CuO_4	$I4/mmm$ (139)<br>P_C4_2/ncm (138.529)	265	(**4c**, 0.4)	
210	$BaCrFeAs_2$	$I4/mmm$ (139)<br>$I4'/m'm'm$ (139.536)	265	(**4d**, 1.09)	
211	Mn_3Sn_2	$Pnma$ (62)<br>$Pn'ma'$ (62.448)	262	(**8d**, 0.83)<br>(**4c**, 1.53)	
212	$MnFe_4Si_3$	$P6_3/mcm$ (193)<br>$P6_3/mc'm'$ (193.260)	260	(**4d**, 1.3)<br>(**4d**, 1.3)<br>(**6g**, 2.1)<br>(**6g**, 2.1)	
213	$MnCoGeB_{0.05}$	$P6_3/mmc$ (194)<br>$P6_3/mm'c'$ (194.270)	260	(**2a**, 2.01)<br>(**2d**, 0.72)	
214	$BaFe_2Se_3$	$Pnma$ (62)<br>C_ac (9.41)	256	(**8a**, 2.8)<br>(**8a**, 2.8)<br>(**8a**, 2.8)<br>(**8a**, 2.8)	

Continued on next page

…continued

No.	Compound	SG & MSG	T_c [K]	(WP, $\|\boldsymbol{m}\|/\mu_B$)	Band Gap [eV]
215	$Y_2SrCuFeO_{6.5}$	$Ibam$ (72)<br>$Pc'c'n$ (56.369)	255	(**8e**, 0.0)<br>(**8e**, 0.0)<br>(**8e**, 2.06)<br>(**8e**, 2.06)	
216	Pr_2CuO_4	$I4/mmm$ (139)<br>C_Accm (66.500)	255	(**8g**, 0.08)<br>(**4a**, 0.39)	
217	LaMnSbO	$P4/nmm$ (129)<br>$P4'/n'm'm$ (129.416)	255	(**2b**, 3.45)	
218	$Sr_2CuO_2Cl_2$	$I4/mmm$ (139)<br>C_Amca (64.480)	251	(**4a**, 0.34)	
219	Fe_2PO_5	$Pnma$ (62)<br>$Pnma$ (62.441)	250	(**4a**, 3.89)<br>(**4c**, 4.22)	
220	Nd_2CuO_4	$I4/mmm$ (139)<br>P_C4_2/nnm (134.481)	245	(**8i**, 0.0)<br>(**4c**, 0.4)	
221	Nd_2CuO_4	$I4/mmm$ (139)<br>C_Accm (66.500)	245	(**4a**, 0.39)<br>(**8g**, 0.0)	
222	U_2N_2Se	$P\bar{3}m1$ (164)<br>$P\bar{3}'m'1$ (164.88)	245	(**2d**, 2.3)	
223	$Bi_2Fe_4O_9$	$Pbam$ (55)<br>C_a2/m (12.64)	244	(**8n**, 3.52)<br>(**8n**, 3.52)<br>(**8j**, 3.73)<br>(**8l**, 3.73)	
224	Sr_2IrO_4	$I4_1/acd$ (142)<br>P_Icca (54.352)	240	(**8c**, 0.24)	
225	$YBaCuFeO_5$	$P4/mmm$ (123)<br>C_a2/m (12.64)	240	(**8n**, 0.0)<br>(**8n**, 0.0)<br>(**8n**, 0.0)<br>(**8n**, 0.0)<br>(**8n**, 2.34)<br>(**8n**, 2.34)<br>(**8n**, 2.34)<br>(**8n**, 2.34)	

Continued on next page

. . . continued

No.	Compound	SG & MSG	T_c [K]	(WP, $\|\boldsymbol{m}\|/\mu_B$)	Band Gap [eV]
226	Fe_2WO_6	$Pbcn$ (60)<br>$Pbc'n'$ (60.423)	240	(**4c**, 2.55)<br>(**4c**, 2.15)	
227	Fe_2WO_6	$Pbcn$ (60)<br>$Pbc'n'$ (60.423)	240	(**4c**, 2.80)<br>(**4c**, 1.96)	
228	$Bi_{0.8}La_{0.2}Fe_{0.5}Mn_{0.5}O_3$	$Imma$ (74)<br>$Imm'a'$ (74.559)	240	(**4c**, 2.0)<br>(**4c**, 2.0)	
229	CeMnSbO	$P4/nmm$ (129)<br>$P4'/n'm'm$ (129.416)	240	(2c,0.0)<br>(**2b**, 3.73)	
230	$PrCrO_3$	$Pbnm$ (62)<br>$Pn'ma'$ (62.448)	239	(**4c**, 0.5)<br>(**4b**, 2.46)	
231	MnPtGa	$P6_3/mmc$ (194)<br>$Cm'c'm$ (63.462)	236	(**4a**, 3.1)	
232	Fe_2WO_6	$Pbcn$ (60)<br>$Pbc'n'$ (60.423)	235.7	(**4c**, 3.6)<br>(**4c**, 4.5)	
233	U_2N_2S	$P\bar{3}m1$ (164)<br>$P\bar{3}'m'1$ (164.88)	233	(**2d**, 1.4)	
234	$Ba_2CoO_2Cu_2S_2$	$I4/mmm$ (139)<br>$P_S\bar{1}$ (2.7)	230	(**2a**, 4.45)	
235	$SmNiO_3$	$Pnma$ (62)<br>C_amc2_1 (36.178)	230	(**8a**, 0.0)<br>(**8b**, 0.4)<br>(**16c**, 0.85)	
236	$Pr_{0.5}Sr_{0.5}CoO_3$	$Imma$ (74)<br>$Im'm'a$ (74.558)	230	(**4b**, 1.49)	
237	$SrFe_2Se_2O$	$Pmmn$ (59)<br>$Pm'm'n'$ (59.411)	228	(**4e**, 3.45)	
238	Mn_3Sn_2	$Pnma$ (62)<br>$Pn'ma'$ (62.448)	227	(**8d**, 1.90)<br>(**4c**, 1.79)	
239	$Cd_2Os_2O_7$	$Fd\bar{3}m$ (227)<br>$Fd\bar{3}m'$ (227.131)	227	(**16c**, 1.03)	
240	$Pr_{0.5}Sr_{0.4}Ba_{0.1}CoO_3$	$Imma$ (74)<br>$Im'm'a$ (74.558)	226	(**4b**, 1.40)	

Continued on next page

…continued

No.	Compound	SG & MSG	T_c [K]	(WP, $\|\boldsymbol{m}\|/\mu_B$)	Band Gap [eV]
241	Mn_3As_2	$C2/m$ (12)<br>$C2/c$ (15.85)	225	(**8f**, 3.23)<br>(**8f**, 3.04)<br>(**4e**, 4.48)<br>(**4a**, 1.61)	
242	$La_{0.25}Pr_{0.75}Co_2P_2$	$I4/mmm$ (139)<br>P_c4/mcc (124.360)	225	(2b,0.0)<br>(**2c**, 0.0)<br>(**8i**, 0.85)	
243	Sr_2IrO_4	$I4_1/acd$ (142)<br>P_Icca (54.352)	224	(**8c**, 0.20)	
244	$NdCrO_3$	$Pbnm$ (62)<br>$P2_1/m$ (11.50)	224	(**2e**, 1.3)<br>(**2e**, 1.3)<br>(**2b**, 2.55)<br>(**2c**, 2.55)	
245	BaCoSO	$Cmcm$ (63)<br>P_abcm (57.386)	220	(**8d**, 3.01)	
246	$Sr_2CoO_2Cu_2S_2$	$I4/mmm$ (139)<br>$P_S\bar{1}$ (2.7)	220	(**2a**, 3.80)	
247	$EuNiO_3$	$Pnma$ (62)<br>C_amc2_1 (36.178)	220	(**8a**, 0.0)<br>(**8b**, 0.0)<br>(**16c**, 1.18)	
248	$CeFeO_3$	$Pbnm$ (62)<br>$Pnma$ (62.441)	220	(**4c**, 0.11)<br>(**4b**, 4.17)	
249	$CeFeO_3$	$Pbnm$ (62)<br>$Pnma$ (62.441)	220	(**4c**, 0.0)<br>(**4b**, 4.05)	
250	$BiFe_{0.5}Sc_{0.5}O_3$	$Pnma$ (62)<br>$Pn'm'a$ (62.446)	220	(**8d**, 2.1)<br>(**8d**, 2.1)<br>(**8d**, 2.1)<br>(**8d**, 2.1)	
251	Fe_2TeO_6	$P4_2/mnm$ (136)<br>$P4_2/m'n'm'$ (136.503)	219	(**4e**, 4.19)	

Continued on next page

...continued

No.	Compound	SG & MSG	T_c [K]	(WP, $\|\boldsymbol{m}\|/\mu_B$)	Band Gap [eV]
252	$La(Fe_{0.91}Al_{0.09})_{13}$	$Fm\bar{3}c$ (226)<br>P_I4/mcc (124.362)	218	(**4d**, 1.1)<br>(**16l**, 2.14)<br>(**16l**, 2.14)<br>(**16k**, 2.14)	
253	$SrFe_2S_2O$	$Pmmn$ (59)<br>$Pm'm'n'$ (59.411)	216	(**4e**, 3.31)	
254	USb	$Fm\bar{3}m$ (225)<br>$Pn\bar{3}m'$ (224.113)	213	(**4b**, 2.85)	
255	CuO	$C2/c$ (15)<br>P_a2_1/c (14.80)	213	(**8e**, 0.65)	
256	Mn_3O_4	$Pbcm$ (57)<br>P_cnma (62.452)	210	(**16e**, 3.49)<br>(**8d**, 0.0)	
257	$SrCaFeOsO_6$	$P2_1/n$ (14)<br>$P2_1'/c'$ (14.79)	210	(**2c**, 4.3)<br>(**2b**, 2.25)	
258	USb_2	$P4/nmm$ (129)<br>P_c4/ncc (130.432)	206	(**4c**, 0.94)	
259	UP_2	$P4/nmm$ (129)<br>P_c4/ncc (130.432)	203	(**4c**, 1.0)	
260	$Fe_{0.945}O$	$Fm\bar{3}m$ (225)<br>$R_I\bar{3}c$ (167.108)	200	(**6a**, 3.31)	
261	$Pd_{2.87}Mn_{0.88}$	$I4/mmm$ (139)<br>P_A2_1/c (14.83)	200	(**4i**, 5.19)	
262	$NdNiO_3$	$Pbnm$ (62)<br>C_amc2_1 (36.178)	200	(**8b**, 0.0)<br>(**8a**, 0.0)<br>(**16c**, 0.94)	
263	$NdNiO_3$	$Pbnm$ (62)<br>C_amc2_1 (36.178)	200	(**8b**, 2.1)<br>(**8a**, 0.0)<br>(**16c**, 0.90)	
264	$La_{0.33}Sr_{0.67}FeO_3$	$R\bar{3}c$ (167)<br>$C2/c$ (15.85)	200	(**4a**, 3.26)<br>(**8f**, 3.67)	

Continued on next page

…continued

No.	Compound	SG & MSG	T_c [K]	(WP, $\lvert \boldsymbol{m} \rvert/\mu_B$)	Band Gap [eV]
265	$La_{0.33}Sr_{0.67}FeO_3$	$R\bar{3}c$ (167)<br>$P3_221$ (154.41)	200	(**6c**, 4.16)	
266	U_3As_4	$I\bar{4}3d$ (220)<br>$R3c'$ (161.71)	200	(**18b**, 1.9)	
267	$Mn_6Ni_{16}Si_7$	$Fm\bar{3}m$ (225)<br>C_Amca (64.480)	198	(**8h**, 4.45)<br>(**8g**, 4.45)<br>(**8i**, 0.0)	
268	Mn_3Sn_2	$Pnma$ (62)<br>$P2_1'/c'$ (14.79)	197	(**4e**, 2.96)<br>(**4e**, 2.96)<br>(**4e**, 2.3)	
269	$CuFePO_5$	$Pnma$ (62)<br>$Pnma$ (62.441)	195	(**4a**, 0.95)<br>(**4c**, 4.28)	
270	$Sr_2Ir_{0.92}Sn_{0.08}O_4$	$I4_1/acd$ (142)<br>$Ib'c'a$ (73.551)	195	(**8c**, 0.19)	
271	$Mn_2Fe_{0.8}Mo_{1.2}O_6$	$P2_1/n$ (14)<br>$P2_1/c$ (14.75)	194	(**4e**, 0.0)<br>(**2c**, 2.5)<br>(**2b**, 1.1)<br>(**2c**, 2.5)	
272	NpBi	$Fm\bar{3}m$ (225)<br>$Pn\bar{3}m'$ (224.113)	192.5	(**4b**, 2.42)	
273	$SmCrO_3$	$Pbnm$ (62)<br>$Pn'ma'$ (62.448)	192	(**4b**, 2.71)<br>(**4c**, 0.1)	
274	$SmCrO_3$	$Pnma$ (62)<br>$Pn'ma'$ (62.448)	191	(**4c**, 1.3)<br>(**4b**, 2.79)	
275	$Sr_2CoO_2Ag_2Se_2$	$I4/mmm$ (139)<br>P_C4_2/n (86.73)	190	(**4c**, 3.76)	
276	$TlFeS_2$	$C2/m$ (12)<br>C_c2/m (12.63)	190	(**8h**, 1.75)	
277	Pr_2CuO_4	$I4/mmm$ (139)<br>C_Accm (66.500)	190-250	(**4a**, 0.31)	

Continued on next page

...continued

No.	Compound	SG & MSG	T_c [K]	(WP, $\|\boldsymbol{m}\|/\mu_B$)	Band Gap [eV]
278	K_2NiF_4	$I4/mmm$ (139)<br>C_Amca (64.480)	190	(**4a**, 1.0)	
279	$CaMn_{0.7}Co_{1.3}ReO_6$	$P4_2/n$ (86)<br>$P4_2/n$ (86.67)	188	(**2a**, 2.9)<br>(**2a**, 2.9)<br>(**2b**, 1.3)<br>(**2b**, 1.3)<br>(**4c**, 3.27)<br>(**4c**, 3.27)<br>(**4d**, 0.3)	
280	MnPtGa	$P6_3/mmc$ (194)<br>$Cm'c'm$ (63.462)	186	(**4a**, 2.69)	
281	UBi_2	$P4/nmm$ (129)<br>$P4/n'm'm'$ (129.419)	183	(**2c**, 2.1)	
282	Mn_3ZnN	$Pm\bar{3}m$ (221)<br>$R\bar{3}m$ (166.97)	183	(**9e**, 1.21)	
283	$Ba_2CoO_2Ag_2Se_2$	$I4/mmm$ (139)<br>P_C4_2/n (86.73)	180	(**4c**, 3.97)	
284	$FeCr_2S_4$	$Fd\bar{3}m$ (227)<br>$I4_1/am'd'$ (141.557)	180	(**8c**, 3.72)<br>(**4b**, 2.63)	
285	$FeCr_2S_4$	$Fd\bar{3}m$ (227)<br>$I4_1/am'd'$ (141.557)	180	(**8c**, 3.69)<br>(**4b**, 2.64)	
286	$NdFeO_3$	$Pnma$ (62)<br>$P2_1'/c'$ (14.79)	180	(**4e**, 1.1)<br>(**2b**, 4.14)<br>(**2c**, 4.14)	
287	$CaFe_4Al_8$	$I4/mmm$ (139)<br>$I4'/mmm'$ (139.535)	180	(**8f**, 0.71)	
288	$Bi_4Fe_5O_{13}F$	$P4_2/mbc$ (135)<br>P_C4_2/n (86.73)	178	(**8i**, 2.71)<br>(**8i**, 1.80)<br>(**8j**, 1.98)<br>(**8j**, 1.98)<br>(**8i**, 0.0)	

Continued on next page

...continued

No.	Compound	SG & MSG	T_c [K]	(WP, $\|\boldsymbol{m}\|/\mu_B$)	Band Gap [eV]
289	$Bi_4Fe_5O_{13}F$	$P4_2/mbc$ (135)<br>P_C4_2/n (86.73)	178	(**8i**, 3.22)<br>(**8i**, 2.10)<br>(**8j**, 2.43)<br>(**8j**, 2.43)<br>(**8i**, 0.0)	
290	$Bi_4Fe_5O_{13}F$	$P4_2/mbc$ (135)<br>P_C4_2/n (86.73)	178	(**8i**, 3.56)<br>(**8i**, 2.43)<br>(**8j**, 2.68)<br>(**8j**, 2.68)<br>(**8i**, 0.0)	
291	$Bi_4Fe_5O_{13}F$	$P4_2/mbc$ (135)<br>P_C4_2/n (86.73)	178	(**8i**, 3.58)<br>(**8i**, 2.51)<br>(**8j**, 2.70)<br>(**8j**, 2.70)<br>(**8i**, 0.0)	
292	$Bi_4Fe_5O_{13}F$	$P4_2/mbc$ (135)<br>P_C4_2/n (86.73)	178	(**8i**, 3.72)<br>(**8i**, 2.33)<br>(**8j**, 2.65)<br>(**8j**, 2.65)<br>(**8i**, 0.0)	
293	$Bi_4Fe_5O_{13}F$	$P4_2/mbc$ (135)<br>P_C4_2/n (86.73)	178	(**8i**, 3.82)<br>(**8i**, 2.32)<br>(**8j**, 2.65)<br>(**8j**, 2.65)<br>(**8i**, 0.0)	
294	$Bi_4Fe_5O_{13}F$	$P4_2/mbc$ (135)<br>P_C4_2/n (86.73)	178	(**8i**, 3.83)<br>(**8i**, 2.21)<br>(**8j**, 2.60)<br>(**8j**, 2.60)<br>(**8i**, 0.0)	

Continued on next page

...continued

No.	Compound	SG & MSG	T_c [K]	(WP, $\|\boldsymbol{m}\|/\mu_B$)	Band Gap [eV]
295	$Bi_4Fe_5O_{13}F$	$P4_2/mbc$ (135)<br>P_C4_2/n (86.73)	178	(**8i**, 3.87)<br>(**8i**, 1.88)<br>(**8j**, 2.42)<br>(**8j**, 2.41)<br>(**8i**, 0.40)	
296	$Bi_4Fe_5O_{13}F$	$P4_2/mbc$ (135)<br>P_C4_2/n (86.73)	178	(**8i**, 3.63)<br>(**8i**, 3.10)<br>(**8j**, 0.99)<br>(**8j**, 3.64)<br>(**8i**, 1.01)	
297	$Bi_4Fe_5O_{13}F$	$P4_2/mbc$ (135)<br>P_C4_2/n (86.73)	178	(**8i**, 3.77)<br>(**8i**, 3.13)<br>(**8j**, 1.09)<br>(**8j**, 3.76)<br>(**8i**, 1.08)	
298	$Bi_4Fe_5O_{13}F$	$P4_2/mbc$ (135)<br>P_C4_2/n (86.73)	178	(**8i**, 3.70)<br>(**8i**, 3.06)<br>(**8j**, 1.10)<br>(**8j**, 3.69)<br>(**8i**, 1.15)	
299	$Bi_4Fe_5O_{13}F$	$P4_2/mbc$ (135)<br>P_C4_2/n (86.73)	178	(**8i**, 3.73)<br>(**8i**, 3.20)<br>(**8j**, 1.10)<br>(**8j**, 3.72)<br>(**8i**, 1.31)	
300	$Bi_4Fe_5O_{13}F$	$P4_2/mbc$ (135)<br>P_C4_2/n (86.73)	178	(**8i**, 3.43)<br>(**8i**, 3.55)<br>(**8j**, 2.31)<br>(**8j**, 3.44)<br>(**8i**, 1.68)	

Continued on next page

…continued

No.	Compound	SG & MSG	T_c [K]	(WP, $\|\boldsymbol{m}\|/\mu_B$)	Band Gap [eV]
301	$Bi_4Fe_5O_{13}F$	$P4_2/mbc$ (135)<br>P_C4_2/n (86.73)	178	(**8i**, 3.48)<br>(**8i**, 3.48)<br>(**8j**, 3.13)<br>(**8j**, 3.13)<br>(**8i**, 1.90)	
302	$Bi_4Fe_5O_{13}F$	$P4_2/mbc$ (135)<br>P_C4_2/n (86.73)	178	(**8i**, 3.63)<br>(**8i**, 3.63)<br>(**8j**, 3.18)<br>(**8j**, 3.18)<br>(**8i**, 2.10)	
303	$Bi_4Fe_5O_{13}F$	$P4_2/mbc$ (135)<br>P_C4_2/n (86.73)	178	(**8i**, 3.81)<br>(**8i**, 3.81)<br>(**8j**, 3.32)<br>(**8j**, 3.32)<br>(**8i**, 3.02)	
304	$Bi_4Fe_5O_{13}F$	$P4_2/mbc$ (135)<br>P_C4_2/n (86.73)	178	(**8i**, 3.91)<br>(**8i**, 3.91)<br>(**8j**, 3.33)<br>(**8j**, 3.33)<br>(**8i**, 3.91)	
305	$TaFe_{1+y}Te_3$	$P2_1/m$ (11)<br>P_c2_1/c (14.82)	178	(**4e**, 1.86)<br>(**4e**, 2.25)	
306	$Bi_4Fe_5O_{13}F$	$P4_2/mbc$ (135)<br>P_C4_2/n (86.73)	178	(**8i**, 4.06)<br>(**8i**, 4.06)<br>(**8j**, 3.33)<br>(**8j**, 3.33)<br>(**8i**, 4.06)	
307	$NiFePO_5$	$Pnma$ (62)<br>$Pnma$ (62.441)	178	(**4a**, 2.03)<br>(**4c**, 4.09)	
308	$CsFe_2Se_3$	$Cmcm$ (63)<br>P_c2_1/c (14.82)	175	(**8f**, 1.77)	
309	Ca_2NiOsO_6	$P2_1/n$ (14)<br>$P2_1'/c'$ (14.79)	175	(**2b**, 2.6)<br>(**2c**, 1.41)	

Continued on next page

…continued

No.	Compound	SG & MSG	T_c [K]	(WP, $\|\boldsymbol{m}\|/\mu_B$)	Band Gap [eV]
310	$Ca_2FeMn_{0.5}W_{0.5}O_6$	$P2_1/n$ (14)<br>$P2_1'/c'$ (14.79)	175	(**2b**, 1.77)<br>(**2c**, 1.77)	
311	$CoFePO_5$	$Pnma$ (62)<br>$Pnm'a'$ (62.447)	175	(**4a**, 3.44)<br>(**4c**, 4.22)	
312	$CaFe_2As_2$	$I4/mmm$ (139)<br>C_Amca (64.480)	173	(**8f**, 0.8)	
313	$FeCr_2S_4$	$Fd\bar{3}m$ (227)<br>$I4_1/am'd'$ (141.557)	172	(**4a**, 3.52)<br>(**8d**, 2.72)	
314	$YBaMn_2O_5$	$P4/nmm$ (129)<br>$P4/nm'm'$ (129.417)	165	(**2c**, 2.9)<br>(**2c**, 3.9)	
315	$SrRuO_3$	$Pnma$ (62)<br>$Pn'm'a$ (62.446)	165	(**4b**, 1.63)	
316	$Yb_{0.42}Sc_{0.58}FeO_3$	$P6_3cm$ (185)<br>$P6_3c'm'$ (185.201)	165	(**6c**, 2.63)	
317	Mn_3GaC	$Pm\bar{3}m$ (221)<br>$R_I\bar{3}c$ (167.108)	164	(**18e**, 1.81)	
318	$KCuMnS_2$	$I4/mmm$ (139)<br>P_C4/mmm (123.349)	160	(**8i**, 0.46)	
319	$KCuMnS_2$	$I4/mmm$ (139)<br>C_Ammm (65.490)	160	(**8f**, 0.46)	
320	V_2O_3	$I2/a$ (15)<br>P_C2_1/c (14.84)	160	(**8f**, 1.20)	
321	Ca_2MnGaO_5	$Pnma$ (62)<br>$Pnm'a'$ (62.447)	160	(**4a**, 3.6)	
322	$CaCu_3Fe_2Sb_2O_{12}$	$Pn\bar{3}$ (201)<br>$Pn'n'n$ (48.260)	160	(**2b**, 0.93)<br>(**2d**, 0.93)<br>(**2c**, 0.93)<br>(**4f**, 4.33)	
323	$Fe_3(PO_4)_2(OH)_2$	$P2_1/c$ (14)<br>$P2_1/c$ (14.75)	160	(**2a**, 5.02)<br>(**4e**, 5.02)	

Continued on next page

…continued

No.	Compound	SG & MSG	T_c [K]	(WP, $\|m\|/\mu_B$)	Band Gap [eV]
324	$TbCrO_3$	$Pbnm$ (62)<br>$Pn'm'a$ (62.446)	158	(**4c**, 0.0)<br>(**4b**, 2.55)	
325	$La_{0.5}Ca_{0.5}MnO_3$	$Pnma$ (62)<br>P_a2_1/m (11.55)	155	(**4a**, 2.97)<br>(**4c**, 2.97)<br>(**8f**, 2.57)	
326	$NiPS_3$	$C2/m$ (12)<br>P_C2_1/m (11.57)	155	(**4g**, 1.0)	
327	$LuFeO_3$	$P6_3cm$ (185)<br>$P6_3c'm'$ (185.201)	155	(**6c**, 2.9)	
328	MnS	$Fm\bar{3}m$ (225)<br>C_c2/m (12.63)	152	(**4a**, 4.39)	
329	$CaMn_{1.2}Ni_{0.8}ReO_6$	$P4_2/n$ (86)<br>$P4_2/n$ (86.67)	152	(**2a**, 3.0)<br>(**2a**, 3.0)<br>(**2b**, 1.2)<br>(**2b**, 1.2)<br>(**4c**, 1.2)<br>(**4c**, 1.2)<br>(**4d**, 0.2)	
330	$LiCoF_4$	$P2_1/c$ (14)<br>P_a2_1/c (14.80)	150	(**4a**, 4.46)	
331	$DyCrO_3$	$Pbnm$ (62)<br>$Pn'm'a$ (62.446)	146	(**4b**, 2.6)<br>(**4c**, 0.0)	
332	Mn_3CuN	$Pm\bar{3}m$ (221)<br>$P4/n$ (85.59)	143	(**4e**, 2.85)<br>(**2c**, 0.65)	
333	$BaFe_2As_2$	$I4/mmm$ (139)<br>C_Amca (64.480)	142	(**8f**, 0.87)	
334	NdFeAsO	$Cmme$ (67)<br>I_cbca (73.553)	141	(**8g**, 0.0)<br>(**8b**, 0.25)	
335	$YCrO_3$	$Pbnm$ (62)<br>$Pn'ma'$ (62.448)	141	(**4b**, 2.96)	
336	Mn_3ZnN	$Pm\bar{3}m$ (221)<br>P_Ibcn (60.432)	140	(**8j**, 0.61)<br>(**4b**, 1.03)	

Continued on next page

…continued

No.	Compound	SG & MSG	T_c [K]	(WP, $\|\boldsymbol{m}\|/\mu_B$)	Band Gap [eV]
337	Sr_2FeOsO_6	$I4/m$ (87)<br>P_I4/m (83.50)	140	(**2a**, 1.83)<br>(**2b**, 0.48)	
338	$Pr_{0.5}Sr_{0.5}MnO_3$	$I4/mcm$ (140)<br>C_Amcm (63.468)	140	(**8d**, 3.4)	
339	U_3P_4	$I\bar{4}3d$ (220)<br>$R3c'$ (161.71)	140	(**18b**, 1.47)	
340	$LaMnO_3$	$Pnma$ (62)<br>$Pn'ma'$ (62.448)	139.5	(**4b**, 3.87)	
341	URh_2Si_2	$I4/mmm$ (139)<br>P_I4/mnc (128.410)	137	(**2a**, 1.96)	
342	$Y_3Co_{3.25}Al_{0.75}$	$Cmcm$ (63)<br>$Cm'cm'$ (63.464)	137	(**8f**, 8.77)<br>(**4c**, 5.5)<br>(**8f**, 0.39)<br>(**4c**, 0.18)	
343	$PrNiO_3$	$Pbnm$ (62)<br>C_amc2_1 (36.178)	135	(**8b**, 0.0)<br>(**8a**, 0.0)<br>(**16c**, 0.93)	
344	$Ba_6Co_6ClO_{15.5}$	$P\bar{6}m2$ (187)<br>$P_c\bar{6}c2$ (188.220)	135	(**4h**, 0.53)<br>(**4h**, 0.54)<br>(**4i**, 2.85)	
345	LaFeAsO	$Cmme$ (67)<br>I_cbca (73.553)	135	(**8b**, 0.63)	
346	$NdVO_3$	$Pbnm$ (62)<br>$P2_1'/m'$ (11.54)	135	(**2e**, 0.0)<br>(**2e**, 0.0)<br>(**2b**, 0.92)<br>(**2c**, 0.92)	
347	$NdVO_3$	$Pbnm$ (62)<br>$P2_1'/m'$ (11.54)	135	(**2e**, 0.52)<br>(**2e**, 0.52)<br>(**2b**, 1.06)<br>(**2c**, 1.06)	

Continued on next page

...continued

No.	Compound	SG & MSG	T_c [K]	(WP, $\|\boldsymbol{m}\|/\mu_B$)	Band Gap [eV]
348	Tb_3NiGe_2	$Pnma$ (62)<br>$Pn'ma'$ (62.448)	135	(**4c**, 7.37)<br>(**4c**, 7.37)<br>(**4c**, 7.37)<br>(**4c**, 0.8)	
349	La_2NiIrO_6	$P2_1/n$ (14)<br>$P_S\bar{1}$ (2.7)	134	(**2c**, 3.15)<br>(**2c**, 4.06)	
350	$ErCrO_3$	$Pbnm$ (62)<br>$Pn'ma'$ (62.448)	133	(**4c**, 0.0)<br>(**4b**, 2.45)	
351	$Fe_{0.48}TiSe_2$	$I2/m$ (12)<br>C_c2/c (15.90)	130	(**4c**, 3.05)<br>(**8i**, 0.31)	
352	$Fe_{0.48}TiSe_2$	$I2/m$ (12)<br>C_c2/c (15.90)	130	(**4c**, 3.05)	
353	$TeNiO_3$	$Pnma$ (62)<br>$Pn'm'a$ (62.446)	130	(**4b**, 2.08)	
354	Nd_2NiO_4	$P4_2/ncm$ (138)<br>$P4_2/nc'm'$ (138.525)	130	(**4d**, 1.59)<br>(**8i**, 3.04)	
355	$Ca_2PrCr_2TaO_9$	$Pnma$ (62)<br>$Pn'm'a$ (62.446)	130	(**4b**, 2.0)	
356	$ScMnO_3$	$P6_3cm$ (185)<br>$P6_3c'm'$ (185.201)	129	(**6c**, 3.03)	
357	$FeOHSO_4$	$C2/c$ (15)<br>$C2'/c'$ (15.89)	125	(**4c**, 4.09)	
358	UAsS	$P4/nmm$ (129)<br>$P4/nm'm'$ (129.417)	125	(**2c**, 1.24)	
359	$Mn_4Nb_2O_9$	$P\bar{3}c1$ (165)<br>$P\bar{3}'c'1$ (165.94)	125	(**4d**, 5.9)<br>(**4d**, 5.9)	
360	$La_2Ni_{1.19}Os_{0.81}O_6$	$P2_1/c$ (14)<br>$P2_1'/c'$ (14.79)	125	(**2b**, 1.86)<br>(**2c**, 0.35)	
361	$CaFe_5O_7$	$P2_1/m$ (11)<br>$P2_1'/m'$ (11.54)	125	(**4f**, 3.8)<br>(**4f**, 3.9)<br>(**2a**, 3.5)	

Continued on next page

…continued

No.	Compound	SG & MSG	T_c [K]	(WP, $\|\boldsymbol{m}\|/\mu_B$)	Band Gap [eV]
362	UAs	$Fm\bar{3}m$ (225)<br>P_I4/mnc (128.410)	124	(**2a**, 1.9)	
363	$TmCrO_3$	$Pbnm$ (62)<br>$Pn'ma'$ (62.448)	124	(**4c**, 0.0)<br>(**4b**, 2.58)	
364	UP	$Fm\bar{3}m$ (225)<br>P_I4/mnc (128.410)	122	(**2a**, 1.7)	
365	Ca_2MnReO_6	$P2_1/c$ (14)<br>$P2_1/c$ (14.75)	121	(**2c**, 4.34)<br>(**2b**, 0.21)	
366	$InMnO_3$	$P6_3cm$ (185)<br>P_c31m (157.56)	120	(**6c**, 4.19)<br>(**6c**, 4.19)	
367	$InMnO_3$	$P6_3cm$ (185)<br>P_c31c (159.64)	120	(**6c**, 3.25)<br>(**6c**, 3.25)	
368	MnO	$Fm\bar{3}m$ (225)<br>C_c2/c (15.90)	120	(**4c**, 5.65)	
369	$CoPS_3$	$C2/m$ (12)<br>P_C2_1/m (11.57)	120	(**4g**, 3.18)	
370	$FePS_3$	$C2/m$ (12)<br>C_c2/m (12.63)	120	(**8h**, 4.98)	
371	$YBaMn_2O_{5.5}$	$Icam$ (72)<br>$Ib'a'm$ (72.543)	120	(**8g**, 3.5)<br>(**8g**, 3.7)	
372	Tb_2MnNiO_6	$P2_1/c$ (14)<br>$P2_1'/c'$ (14.79)	120	(**4e**, 0.0)<br>(**2c**, 1.35)<br>(**2b**, 2.03)	
373	Sr_2MnReO_6	$P2_1/c$ (14)<br>$P2_1'/c'$ (14.79)	120	(**2c**, 4.48)<br>(**2b**, 0.92)	
374	$FePSe_3$	$R\bar{3}$ (148)<br>$P_S\bar{1}$ (2.7)	119	(**4i**, 4.9)	
375	Ca_3LiOsO_6	$R\bar{3}c$ (167)<br>$C2'/c'$ (15.89)	117.1	(**4c**, 2.2)	
376	Ca_3LiRuO_6	$R\bar{3}c$ (167)<br>$C2'/c'$ (15.89)	117	(**4d**, 2.8)	

Continued on next page

...continued

No.	Compound	SG & MSG	T_c [K]	(WP, $\|\boldsymbol{m}\|/\mu_B$)	Band Gap [eV]
377	$ThMn_2$	$P6_3/mmc$ (194)<br>$P\bar{6}'2'm$ (189.223)	115	(**6i**, 0.0)<br>(**4h**, 0.0)<br>(2e,0.0)<br>(**6i**, 0.0)<br>(**3f**, 1.6)<br>(**6j**, 1.6)<br>(**3g**, 1.6)<br>(**6k**, 1.6)	
378	$CaIrO_3$	$Cmcm$ (63)<br>$Cm'cm'$ (63.464)	115	(**4a**, 1.0)	
379	$BiCrO_3$	$C2/c$ (15)<br>$C2/c$ (15.85)	114	(**4d**, 2.04)<br>(**4e**, 2.04)	
380	$CaCo_2P_2$	$I4/mmm$ (139)<br>P_Immn (59.416)	113	(**4h**, 0.32)	
381	$ErVO_3$	$Pbnm$ (62)<br>$P2_1'/m'$ (11.54)	112	(**2e**, 0.0)<br>(**2e**, 0.0)<br>(**2b**, 1.19)<br>(**2c**, 1.19)	
382	GdMg	$Pm\bar{3}m$ (221)<br>$C2'/c'$ (15.89)	110	(**4a**, 7.12)	
383	$Sr_2MnGaO_{5.5}$	$P4/mmm$ (123)<br>P_C4/mbm (127.397)	110	(**2c**, 2.1)	
384	$Ba_3CoRu_2O_9$	$P6_3/mmc$ (194)<br>P_Bnma (62.454)	110	(**8f**, 1.16)<br>(**4a**, 2.84)	
385	UPSe	$P4/nmm$ (129)<br>$P4/nm'm'$ (129.417)	110	(**2c**, 1.35)	
386	$Mn_4Ta_2O_9$	$P\bar{3}c1$ (165)<br>$P\bar{3}'c'1$ (165.94)	110	(**4d**, 5.1)<br>(**4d**, 4.8)	
387	Ca_2RuO_4	$Pbca$ (61)<br>$Pbca$ (61.433)	110	(**4a**, 1.00)	
388	Ca_2MnO_4	$I4_1/acd$ (142)<br>$I4_1'/a'cd'$ (142.568)	110	(**8a**, 2.4)	

Continued on next page

. . . continued

No.	Compound	SG & MSG	T_c [K]	(WP, $\|\boldsymbol{m}\|/\mu_B$)	Band Gap [eV]
389	$Ca_2PrCr_2NbO_9$	$Pnma$ (62)<br>$Pn'm'a$ (62.446)	110	(**4b**, 1.8)	
390	$Ba_5Co_5ClO_{13}$	$P6_3/mmc$ (194)<br>$P6_3'/m'm'c$ (194.268)	110	(**4e**, 0.61)<br>(**4f**, 2.21)<br>(**2a**, 0.35)	
391	Mn_3ReO_6	$P2_1/c$ (14)<br>$P_S\bar{1}$ (2.7)	109	(**4i**, 1.10)<br>(**4i**, 1.10)<br>(**2d**, 1.89)<br>(**2c**, 1.89)<br>(**2a**, 0.0)<br>(**2e**, 0.0)	
392	Sr_2CoOsO_6	$I4/m$ (87)<br>C_c2/c (15.90)	108	(**4c**, 1.59)<br>(**4d**, 0.70)	
393	UPd_2Si_2	$I4/mmm$ (139)<br>P_I4/mnc (128.410)	108	(**2a**, 2.4)	
394	Sr_2CoOsO_6	$I2/m$ (12)<br>$P_S\bar{1}$ (2.7)	108	(**2d**, 0.0)<br>(**2a**, 1.56)	
395	Sr_2CoOsO_6	$B2/n$ (15)<br>$C2/c$ (15.85)	108	(**4c**, 1.57)<br>(**4d**, 0.70)	
396	$Ba_3CoIr_2O_9$	$C2/c$ (15)<br>$C2/c$ (15.85)	107	(**4a**, 2.45)<br>(**8f**, 0.57)	
397	$Mn_4Ta_2O_9$	$P\bar{3}c1$ (165)<br>$P\bar{3}'c'1$ (165.94)	106.4	(**4d**, 4.7)<br>(**4d**, 4.8)	
398	$Sr_2F_2Fe_2OS_2$	$I4/mmm$ (139)<br>C_a2/m (12.64)	106	(**16o**, 3.3)	
399	$La_2O_2Fe_2OS_2$	$I4/mmm$ (139)<br>C_a2/m (12.64)	105	(**16o**, 3.3)	
400	$Ba_4Ru_3O_{10}$	$Cmce$ (64)<br>$Cm'ca$ (64.471)	105	(4a,0.0)<br>(**8f**, 1.0)	
401	$LiFe_2F_6$	$P4_2/mnm$ (136)<br>$P4_2'/mnm'$ (136.499)	105	(**4e**, 4.4)	

Continued on next page

. . . continued

No.	Compound	SG & MSG	T_c [K]	(WP, $\|\boldsymbol{m}\|/\mu_B$)	Band Gap [eV]
402	$(Tm_{0.7}Mn_{0.3})MnO_3$	$Pnma$ (62)<br>$Pnm'a'$ (62.447)	104	(**4c**, 5.01)<br>(**4c**, 3.93)<br>(**4b**, 3.2)	
403	$La_8Cu_7O_{19}$	$C2/c$ (15)<br>$P_S\bar{1}$ (2.7)	103	(**4i**, 0.69)<br>(**4i**, 0.69)<br>(**4i**, 0.73)<br>(**4i**, 0.73)<br>(**4i**, 0.73)<br>(**4i**, 0.73)<br>(**4i**, 1.16)<br>(**4i**, 1.13)<br>(**4i**, 1.19)<br>(**4i**, 1.16)<br>(**4i**, 1.32)<br>(**4i**, 1.30)<br>(**4i**, 1.32)<br>(**4i**, 1.33)	
404	Mn_5Si_3	$P6_3/mcm$ (193)<br>P_Cbcn (60.431)	100	(**8e**, 0.0)<br>(**4c**, 0.0)<br>(**8g**, 1.88)	
405	$LaBaMn_2O_5$	$P4/nmm$ (129)<br>$P4/nm'm'$ (129.417)	100	(**2c**, 2.7)<br>(**2c**, 3.2)	
406	$CoFe_3O_5$	$Cmcm$ (63)<br>$P2_1'/m'$ (11.54)	100	(**4f**, 4.34)<br>(**2a**, 3.31)<br>(**2e**, 2.56)	
407	$Fe_{1.5}Mn_{1.5}BO_5$	$Pbam$ (55)<br>$Pbam$ (55.353)	100	(**2a**, 1.7)<br>(**2d**, 2.7)<br>(**4g**, 2.5)<br>(**4h**, 2.7)	
408	$NdCo_2$	$Fd\bar{3}m$ (227)<br>$I4_1/am'd'$ (141.557)	100	(**4b**, 2.43)<br>(**8c**, 0.59)	
409	$FeSO_4F$	$C2/c$ (15)<br>$C2'/c'$ (15.89)	100	(**4d**, 4.32)	

Continued on next page

…continued

No.	Compound	SG & MSG	T_c [K]	(WP, $\lvert \boldsymbol{m} \rvert/\mu_B$)	Band Gap [eV]
410	Mn_5Si_3	$P6_3/mcm$ (193)<br>P_Cbcn (60.431)	99	(**8e**, 0.0)<br>(**4c**, 0.0)<br>(**8g**, 1.48)	
411	Mn_3ReO_6	$P2_1/c$ (14)<br>$P_S\bar{1}$ (2.7)	99	(**4i**, 4.49)<br>(**4i**, 4.49)<br>(**2d**, 1.99)<br>(**2c**, 1.99)<br>(**2a**, 0.98)<br>(**2e**, 0.98)	
412	$CrReO_4$	$C2/m$ (12)<br>C_c2/c (15.90)	98	(**8i**, 1.93)<br>(**8g**, 0.33)	
413	Tb_2Pt	$Pnma$ (62)<br>$P2_1'/m'$ (11.54)	98	(**2e**, 5.57)<br>(**2e**, 5.57)<br>(**2e**, 5.57)<br>(**2e**, 5.57)	
414	FeF_3	$P6/mmm$ (191)<br>$P6_3/m$ (176.143)	97	(**6g**, 4.07)	
415	$YRuO_3$	$Pnma$ (62)<br>$Pn'ma'$ (62.448)	97	(**4b**, 0.33)	
416	alpha-Mn	$I\bar{4}3m$ (217)<br>$P_I\bar{4}2_1c$ (114.282)	95	(**2a**, 2.83)<br>(**8i**, 1.83)<br>(**8i**, 0.74)<br>(**16j**, 0.47)<br>(**8i**, 0.59)<br>(**16j**, 0.66)	
417	$Sr_2Fe_3Se_2O_3$	$Pbam$ (55)<br>C_amc2_1 (36.178)	95	(**8b**, 0.0)<br>(**8a**, 2.78)<br>(**8a**, 2.78)	
418	$Ba_3NiRu_2O_9$	$P6_3/mmc$ (194)<br>$P6_3'/m'm'c$ (194.268)	95	(**2a**, 1.7)<br>(**4f**, 1.5)	
419	$Ho_2Ru_2O_7$	$Fd\bar{3}m$ (227)<br>$I4_1/am'd'$ (141.557)	95	(**8d**, 0.0)<br>(**8c**, 1.19)	

Continued on next page

...continued

No.	Compound	SG & MSG	T_c [K]	(WP, $\lvert \boldsymbol{m} \rvert/\mu_B$)	Band Gap [eV]
420	La_2CoIrO_6	$P2_1/n$ (14)<br>$P2_1/c$ (14.75)	95	(**2c**, 2.41)<br>(**2b**, 0.0)	
421	Mn_2CoReO_6	$P2_1/n$ (14)<br>$P_S\bar{1}$ (2.7)	94	(**4i**, 2.41)<br>(**4i**, 2.41)<br>(**2e**, 2.41)<br>(**2a**, 2.41)<br>(**2c**, 0.80)<br>(**2d**, 0.80)	
422	$FeSn_2$	$I4/mcm$ (140)<br>P_Cbcn (60.431)	93	(**8e**, 1.70)	
423	$InCrO_3$	$Pnma$ (62)<br>$Pnma$ (62.441)	93	(**4b**, 2.5)	
424	$Sr_2Fe_3Se_2O_3$	$Pbam$ (55)<br>C_amc2_1 (36.178)	92	(**8b**, 0.0)<br>(**8a**, 3.01)<br>(**8a**, 3.01)	
425	$Ba_2Ni_2F_7Cl$	$P2_1/m$ (11)<br>P_c2_1/c (14.82)	92	(**4e**, 1.86)<br>(**4e**, 1.57)	
426	$TbRh_2Si_2$	$I4/mmm$ (139)<br>P_I4/mnc (128.410)	92	(**2a**, 8.5)	
427	$TlMnO_3$	$Pm\bar{3}m$ (221)<br>$P_S\bar{1}$ (2.7)	92	(**2d**, 4.82)<br>(**2e**, 4.82)<br>(**2a**, 4.82)<br>(**2c**, 4.82)	
428	$Cu_{1.94}Mn_{1.06}BO_5$	$P2_1/c$ (14)<br>$P2_1'/c'$ (14.79)	92	(**2a**, 0.44)<br>(**2d**, 1.14)<br>(**4e**, 0.93)<br>(**4e**, 2.71)	
429	$PrMnO_3$	$Pbnm$ (62)<br>$Pn'ma'$ (62.448)	91	(**4c**, 0.0)<br>(**4b**, 1.77)	
430	$UGeS$	$P4/nmm$ (129)<br>P_c4/ncc (130.432)	90	(**4c**, 1.26)	

Continued on next page

. . . continued

No.	Compound	SG & MSG	T_c [K]	(WP, $\|\boldsymbol{m}\|/\mu_B$)	Band Gap [eV]
431	Fe_2MnBO_5	$Pbam$ (55)<br>P_bnma (62.451)	90	(4a,0.0)<br>(**4d**, 2.5)<br>(**8g**, 0.0)<br>(**8h**, 1.8)	
432	Sr_3LiRuO_6	$R\bar{3}c$ (167)<br>$C2'/c'$ (15.89)	90	(**4d**, 2.03)	
433	$Er_2Ru_2O_7$	$Fd\bar{3}m$ (227)<br>$I4_1'/am'd$ (141.554)	90	(**8d**, 4.5)<br>(**8c**, 2.0)	
434	$La_2O_2Fe_2OSe_2$	$I4/mmm$ (139)<br>C_a2/m (12.64)	89	(**16o**, 3.5)	
435	$TlCrO_3$	$Pnma$ (62)<br>$Pnma$ (62.441)	89	(**4b**, 2.46)	
436	Na_2NiFeF_7	$Imma$ (74)<br>$Imm'a'$ (74.559)	88	(**4c**, 1.35)<br>(**4b**, 4.93)	
437	Na_2NiFeF_7	$Imma$ (74)<br>$Imm'a'$ (74.559)	88	(**4c**, 0.96)<br>(**4b**, 4.34)	
438	$KMnF_3$	$I4/mcm$ (140)<br>$I4/mcm$ (140.541)	86.8	(**4c**, 1.0)	
439	$MnTe_2$	$Pa\bar{3}$ (205)<br>$Pa\bar{3}$ (205.33)	86.5	(**4a**, 4.27)	
440	$UNiGa_5$	$P4/mmm$ (123)<br>I_c4/mcm (140.550)	86	(**4a**, 0.75)<br>(4b,0.0)	
441	PrFeAsO	$Cmme$ (67)<br>P_Bcca (54.350)	85	(**4g**, 0.0)<br>(**4b**, 0.35)	
442	$Pr_{0.95}K_{0.05}MnO_3$	$Pbnm$ (62)<br>$Pn'ma'$ (62.448)	85	(**4b**, 2.84)<br>(**4c**, 0.0)	
443	$NdMnO_3$	$Pbnm$ (62)<br>$P2_1/m$ (11.50)	85	(**2e**, 0.0)<br>(**2e**, 0.0)<br>(**2b**, 1.71)<br>(**2c**, 1.71)	

Continued on next page

. . . continued

No.	Compound	SG & MSG	T_c [K]	(WP, $\|\boldsymbol{m}\|/\mu_B$)	Band Gap [eV]
444	$YbMnO_3$	$P6_3cm$ (185)<br>$P6_3'c'm$ (185.199)	85	(2a,0.0)<br>(**4b**, 1.15)<br>(**6c**, 4.40)	
445	$YbMnO_3$	$P6_3cm$ (185)<br>$P6_3'c'm$ (185.199)	85	(2a,0.0)<br>(**4b**, 0.81)<br>(**6c**, 4.29)	
446	$YbMnO_3$	$P6_3cm$ (185)<br>$P6_3'c'm$ (185.199)	85	(**6c**, 4.20)	
447	$BaMnFeF_7$	$P2_1/c$ (14)<br>$P2_1'/c'$ (14.79)	84.9	(**4e**, 4.13)<br>(**4e**, 4.16)	
448	$TbCr_{0.5}Mn_{0.5}O_3$	$Pbnm$ (62)<br>$Pn'ma'$ (62.448)	84	(**4c**, 0.0)<br>(**4b**, 0.77)<br>(**4b**, 0.77)	
449	$Ba_2Co_2F_7Cl$	$P2_1/m$ (11)<br>P_a2_1/m (11.55)	83	(**4e**, 3.79)<br>(**4e**, 3.79)	
450	TbMg	$Pm\bar{3}m$ (221)<br>$Pc'cm'$ (49.270)	81	(**2a**, 7.33)	
451	TbMg	$Pm\bar{3}m$ (221)<br>$Pmm'a'$ (51.295)	81	(**2a**, 7.33)	
452	UPd_2Ge_2	$I4/mmm$ (139)<br>P_c4/ncc (130.432)	80	(**4c**, 2.37)<br>(**4c**, 2.23)	
453	VOCl	$Pmmn$ (59)<br>C_a2/c (15.91)	80	(**8f**, 1.3)	
454	La_2NiO_4	$P4_2/ncm$ (138)<br>$Pc'c'n$ (56.369)	80	(**4a**, 1.68)	
455	Mn_2O_3-alpha	$Pbca$ (61)<br>$Pbca$ (61.433)	80	(**4a**, 2.69)<br>(**4b**, 3.10)<br>(**8c**, 3.01)<br>(**8c**, 2.91)<br>(**8c**, 3.54)	

Continued on next page

...continued

No.	Compound	SG & MSG	T_c [K]	(WP, $\|\boldsymbol{m}\|/\mu_B$)	Band Gap [eV]
456	Mn_2O_3-alpha	$Pbca$ (61)<br>$Pbca$ (61.433)	80	(**4a**, 3.05)<br>(**4b**, 3.47)<br>(**8c**, 3.49)<br>(**8c**, 3.26)<br>(**8c**, 4.18)	
457	Tl_2NiMnO_6	$P2_1/c$ (14)<br>$P2_1/c$ (14.75)	80	(**2b**, 1.51)<br>(**2c**, 0.99)	
458	Y_2MnCoO_6	$P2_1/c$ (14)<br>$P2_1'/c'$ (14.79)	80	(**2b**, 2.92)<br>(**2c**, 2.92)	
459	$CsCo_2Se_2$	$I4/mmm$ (139)<br>C_Amcm (63.468)	78	(**8f**, 0.77)	
460	$Mn_3Si_2Te_6$	$P\bar{3}1c$ (163)<br>$C2'/c'$ (15.89)	78	(**8f**, 4.2)<br>(**4e**, 4.2)	
461	$NdMnO_3$	$Pnma$ (62)<br>$Pn'ma'$ (62.448)	78	(**4c**, 0.0)<br>(**4b**, 3.00)	
462	$Mn_3Ti_2Te_6$	$P\bar{3}1c$ (163)<br>$C2'/c'$ (15.89)	78	(**8f**, 4.2)<br>(**4e**, 4.2)	
463	YVO_3	$Pbnm$ (62)<br>$Pn'm'a$ (62.446)	77	(**4b**, 1.72)	
464	$KMnF_3$	$Pnma$ (62)<br>$Pn'ma'$ (62.448)	77	(**4b**, 4.8)	
465	$Sr_2Fe_3S_2O_3$	$Pbam$ (55)<br>P_bnma (62.451)	76	(**4b**, 0.0)<br>(**8g**, 3.21)	
466	$(Ho_{0.8}Mn_{0.2})MnO_3$	$Pnma$ (62)<br>$Pnm'a'$ (62.447)	76	(**4c**, 2.02)<br>(**4c**, 2.02)<br>(**4b**, 2.2)	
467	$Co_6(OH)_3(TeO_3)_4(OH)_{0.9}(H_{20})$	$P6_3mc$ (186)<br>$P6_3'mc'$ (186.206)	75.5	(**12d**, 3.04)	
468	Nd_2CuO_4	$I4/mmm$ (139)<br>C_Amca (64.480)	75	(**8h**, 0.0)<br>(**4a**, 0.39)	

Continued on next page

…continued

No.	Compound	SG & MSG	T_c [K]	(WP, $\|m\|/\mu_B$)	Band Gap [eV]
469	$Fe_{0.25}TiSe_2$	$F2/m$ (12)<br>C_c2/c (15.90)	75	(**8i**, 4.03)	
470	Sr_3NiIrO_6	$R\bar{3}c$ (167)<br>$P\bar{3}c'1$ (165.95)	75	(**2b**, 0.5)<br>(**4d**, 0.25)<br>(**2a**, 1.5)<br>(**4d**, 0.75)	
471	$MnCoGeB_{0.05}$	$P6_3/mmc$ (194)<br>$Cm'c'm$ (63.462)	75	(**4b**, 2.19)<br>(**4c**, 0.76)	
472	$MnPrMnSbO_6$	$P4_2/n$ (86)<br>$P4_2/n$ (86.67)	75	(**2a**, 5.1)<br>(**2b**, 5.1)<br>(**4e**, 2.2)<br>(**4c**, 5.1)	
473	$MnSn_2$	$I4/mcm$ (140)<br>C_cccm (66.498)	74	(**16m**, 2.33)	
474	$NiCr_2O_4$	$I4_1/amd$ (141)<br>$Fd'd'd$ (70.530)	74	(**8b**, 1.64)<br>(**16c**, 1.40)	
475	$TmMn_3O_6$	$Pmmn$ (59)<br>$Pm'm'n$ (59.409)	74	(**2a**, 0.0)<br>(**2a**, 0.0)<br>(**2b**, 1.95)<br>(**2b**, 3.1)<br>(**4c**, 1.89)<br>(**4d**, 1.58)	
476	UGeTe	$I4/mmm$ (139)<br>P_I4/nnc (126.386)	73	(**4e**, 1.5)	
477	Sr_3CoIrO_6	$R\bar{3}c$ (167)<br>$P\bar{3}c'1$ (165.95)	73	(**2a**, 3.6)<br>(**4d**, 1.8)<br>(**2b**, 0.6)<br>(**4d**, 0.3)	
478	$NdMnO_3$	$Pnma$ (62)<br>$Pn'ma'$ (62.448)	73	(**4c**, 0.0)<br>(**4b**, 2.64)	
479	NiF_2	$P4_2/mnm$ (136)<br>$Pnn'm'$ (58.398)	73	(**2a**, 2.00)	

Continued on next page

...continued

No.	Compound	SG & MSG	T_c [K]	(WP, $\|m\|/\mu_B$)	Band Gap [eV]
480	$ScCrO_3$	$Pnma$ (62)<br>$Pnma$ (62.441)	73	(**4b**, 2.7)	
481	$HoMnO_3$	$P6_3cm$ (185)<br>$P6_3c'm'$ (185.201)	72	(**6c**, 2.52)	
482	NdZn	$Pm\bar{3}m$ (221)<br>$P_In\bar{3}n$ (222.103)	70	(**8c**, 2.51)	
483	Sr_2CoOsO_6	$I2/m$ (12)<br>$P_S\bar{1}$ (2.7)	70	(**4i**, 1.81)<br>(**2a**, 2.89)<br>(**2e**, 2.89)	
484	$YMnO_3$	$P6_3cm$ (185)<br>$P6_3cm$ (185.197)	70	(**6c**, 3.75)	
485	$HoMnO_3$	$P6_3cm$ (185)<br>$P6_3'c'm$ (185.199)	70	(2a,0.0)<br>(**4b**, 0.27)<br>(**6c**, 3.17)	
486	Sr_3NaRuO_6	$R\bar{3}c$ (167)<br>$C2'/c'$ (15.89)	70	(**4d**, 1.0)	
487	Tb_2MnNiO_6	$P2_1/c$ (14)<br>$P2_1'/c'$ (14.79)	70	(**4e**, 0.0)<br>(**2c**, 1.74)<br>(**2b**, 2.61)	
488	CuF_2	$P2_1/c$ (14)<br>$P_S\bar{1}$ (2.7)	69	(**2a**, 0.72)<br>(**2e**, 0.72)	
489	$FeTiO_3$	$R\bar{3}$ (148)<br>$R_I\bar{3}$ (148.20)	68	(**12c**, 1.0)<br>(**12c**, 0.0)	
490	Sr_2FeOsO_6	$I4/m$ (87)<br>P_c4/n (85.64)	67	(**4c**, 3.07)<br>(**4c**, 0.68)	
491	$Fe_{1.068}Te$	$P4/nmm$ (129)<br>$P_S\bar{1}$ (2.7)	67	(**4i**, 2.23)<br>(**4i**, 0.0)	
492	Cr_2ReO_6	$P4_2/mnm$ (136)<br>P_a2_1/c (14.80)	67	(**8e**, 1.62)<br>(**4a**, 0.25)	
493	$(Lu_{0.6}Mn_{0.4})MnO_3$	$Pnma$ (62)<br>$Pnm'a'$ (62.447)	67	(**4b**, 3.23)<br>(**4c**, 5.07)	

Continued on next page

. . . continued

No.	Compound	SG & MSG	T_c [K]	(WP, $\|m\|/\mu_B$)	Band Gap [eV]
494	MnF_2	$P4_2/mnm$ (136)<br>$P4_2'/mnm'$ (136.499)	67	(**2a**, 4.6)	
495	$FeWO_4$	$P2/c$ (13)<br>P_a2/c (13.70)	66	(**4f**, 2.19)	
496	TbC_2	$I4/mmm$ (139)<br>P_c222_1 (17.12)	66	(**2d**, 1.6)<br>(**2a**, 7.1)<br>(**4t**, 5.14)	
497	$YMnO_3$	$P6_3cm$ (185)<br>$P6_3'$ (173.131)	66	(**6c**, 3.49)	
498	Fe_2SiO_4	$Pnma$ (62)<br>$Pnma$ (62.441)	65.3	(**4a**, 4.44)<br>(**4c**, 4.4)	
499	$CuMnO_2$	$C2/m$ (12)<br>$P_S\bar{1}$ (2.7)	65	(**2a**, 3.42)	
500	Tb_2BaNiO_5	$Immm$ (71)<br>C_c2/c (15.90)	65	(**4c**, 2.01)<br>(**8i**, 8.02)	
501	$Tb_{0.55}Sr_{0.45}MnO_3$	$Pnma$ (62)<br>$Pnm'a'$ (62.447)	65	(**4c**, 0.0)<br>(**4b**, 1.31)	
502	$Tb_{0.55}Sr_{0.45}MnO_3$	$Pnma$ (62)<br>$Pnm'a'$ (62.447)	65	(**4c**, 0.0)<br>(**4b**, 1.60)	
503	$DyCu$	$Pm\bar{3}m$ (221)<br>$Im\bar{3}m'$ (229.143)	64	(**8c**, 8.62)	
504	$Sr_2Ru_{0.95}Fe_{0.05}O_4$	$I4/mmm$ (139)<br>C_cmcm (63.466)	64	(**4a**, 0.4)<br>(4c,0.0)	
505	$Sr_2Ru_{0.95}Fe_{0.05}O_4$	$I4/mmm$ (139)<br>C_cmca (64.478)	64	(**8g**, 0.28)	
506	$KFe_3(OH)_6(SO_4)_2$	$R\bar{3}m$ (166)<br>$R_I\bar{3}c$ (167.108)	64	(**18e**, 3.11)	
507	$Fe_{1.125}Te$	$P4/nmm$ (129)<br>$P_S\bar{1}$ (2.7)	63	(**4i**, 2.06)<br>(**4i**, 0.0)	
508	$Sr_2MnO_2Ag_{1.5}Se_2$	$I4/mmm$ (139)<br>P_I4/mnc (128.410)	63	(**2a**, 3.99)	

Continued on next page

. . . continued

No.	Compound	SG & MSG	T_c [K]	(WP, $\|\boldsymbol{m}\|/\mu_B$)	Band Gap [eV]
509	UAs	$Fm\bar{3}m$ (225)<br>P_C4_2/nnm (134.481)	62	(**8l**, 2.26)<br>(**8k**, 2.26)	
510	$NiWO_4$	$P2/c$ (13)<br>P_a2/c (13.70)	62	(**4f**, 2.24)	
511	NdMg	$Pm\bar{3}m$ (221)<br>P_c4/mcc (124.360)	61	(**2a**, 2.6)	
512	Tm_2CoMnO_6	$P2_1/c$ (14)<br>$P2_1'/c'$ (14.79)	60	(**4e**, 0.78)<br>(**2c**, 2.56)<br>(**2b**, 2.56)	
513	Tm_2CoMnO_6	$P2_1/c$ (14)<br>$P2_1'/c'$ (14.79)	60	(**4e**, 2.18)<br>(**2c**, 2.83)<br>(**2b**, 2.83)	
514	Ho_2CoMnO_6	$P2_1/c$ (14)<br>$P2_1'/c'$ (14.79)	60	(**4e**, 0.0)<br>(**2c**, 2.92)<br>(**2b**, 2.92)	
515	Tb_2MnNiO_6	$P2_1/c$ (14)<br>$P2_1/c$ (14.75)	60	(**4e**, 0.0)<br>(**2b**, 1.51)<br>(**2b**, 0.0)<br>(**2c**, 0.0)<br>(**2c**, 0.99)	
516	$ErVO_3$	$Pbnm$ (62)<br>$P2_1/c$ (14.75)	60	(**4e**, 8.2)<br>(**2c**, 1.47)<br>(**2d**, 1.47)	
517	$NdMn_{0.8}Fe_{0.2}O_3$	$Pnma$ (62)<br>$Pn'ma'$ (62.448)	59	(**4c**, 0.7)<br>(**4b**, 3.17)<br>(**4b**, 0.0)	
518	$Fe_2Mo_3O_8$	$P6_3mc$ (186)<br>$P6_3'm'c$ (186.205)	59	(**2b**, 4.6)<br>(**2b**, 4.6)	
519	$Ca_2Mn_3O_8$	$C2/m$ (12)<br>$P_S\bar{1}$ (2.7)	58	(**2d**, 2.5)<br>(**4i**, 2.17)	
520	Sr_2CaIrO_6	$P2_1/n$ (14)<br>$P_S\bar{1}$ (2.7)	58	(**2d**, 1.33)<br>(**2c**, 1.33)	

Continued on next page

. . . continued

No.	Compound	SG & MSG	T_c [K]	(WP, $\|\boldsymbol{m}\|/\mu_B$)	Band Gap [eV]
521	Ag_2NiO_2	$C2/m$ (12)<br>C_c2/c (15.90)	56	(**4c**, 0.72)<br>(**8h**, 0.36)	
522	TbPt	$Pnma$ (62)<br>$Pn'm'a$ (62.446)	56	(**4c**, 8.09)	
523	$DyFe_4Ge_2$	$P4_2/mnm$ (136)<br>P_ccc2 (27.82)	55	(**2d**, 4.8)<br>(**2b**, 4.8)<br>(**2c**, 4.8)<br>(**2a**, 4.8)<br>(**4g**, 1.39)<br>(**4g**, 1.33)<br>(**4h**, 1.39)<br>(**4h**, 1.33)<br>(**4g**, 1.39)<br>(**4g**, 1.33)<br>(**4h**, 1.39)<br>(**4h**, 1.33)	
524	UN	$Fm\bar{3}m$ (225)<br>P_I4/mnc (128.410)	55	(**2a**, 0.75)	
525	$NdRh_2Si_2$	$I4/mmm$ (139)<br>P_I4/mnc (128.410)	55	(**2a**, 3.25)<br>(4d,0.0)	
526	$La_2NiO_3F_2$	$Cccm$ (66)<br>P_Amna (53.333)	55	(**4e**, 0.7)	
527	$CrTe_3$	$P2_1/c$ (14)<br>$P_S\bar{1}$ (2.7)	55	(**4i**, 2.47)<br>(**4i**, 2.47)<br>(**4i**, 2.47)<br>(**4i**, 2.47)	
528	$HoMn_{0.9}Fe_{0.1}O_3$	$P6_3cm$ (185)<br>$P6_3'cm'$ (185.200)	55	(**2a**, 3.4)<br>(**4b**, 1.3)<br>(**6c**, 2.79)<br>(**6c**, 2.79)	
529	PrSi	$Pnma$ (62)<br>$Pnm'a'$ (62.447)	54	(**4c**, 2.80)	

Continued on next page

...continued

No.	Compound	SG & MSG	T_c [K]	(WP, $\|m\|/\mu_B$)	Band Gap [eV]
530	$CsCoF_4$	$I\bar{4}c2$ (120)<br>$I\bar{4}'$ (82.41)	54	(**4f**, 3.5)<br>(**8g**, 2.8)<br>(**8g**, 3.5)	
531	Ho_2BaNiO_5	$Immm$ (71)<br>C_c2/c (15.90)	53	(**8i**, 9.06)<br>(**4c**, 1.38)	
532	MnV_2O_4	$I4_1/a$ (88)<br>$I4_1/a$ (88.81)	53	(**4a**, 4.2)<br>(**8d**, 1.29)	
533	$Nd_2NiO_{4.11}$	$P4_2/ncm$ (138)<br>$P4_2/nc'm'$ (138.525)	53	(**8i**, 1.62)<br>(**4d**, 1.14)	
534	$Sr_2Fe_3S_2O_3$	$Pbam$ (55)<br>P_A2_1/c (14.83)	52.5	(**2a**, 3.74)<br>(**2b**, 3.74)<br>(**2d**, 3.74)<br>(**2c**, 3.74)<br>(**8j**, 3.60)<br>(**8j**, 3.60)	
535	$NiBr_2$	$R\bar{3}m$ (166)<br>C_c2/c (15.90)	52	(**4c**, 2.58)	
536	$NiCl_2$	$R\bar{3}m$ (166)<br>C_c2/c (15.90)	52	(**4c**, 2.72)	
537	$Cu_{1.07}Mn_{0.93}O_2$	$C2/m$ (12)<br>$P_S\bar{1}$ (2.7)	52	(**2a**, 2.28)	
538	$CaCo_{1.86}As_2$	$I4/mmm$ (139)<br>P_I4/nnc (126.386)	52	(**4d**, 0.6)	
539	Nd_5Ge_4	$Pnma$ (62)<br>$Pnm'a'$ (62.447)	52	(**8d**, 2.63)<br>(**8d**, 3.04)<br>(**4c**, 2.41)	
540	CaV_2O_4	$P2_1/n11$ (14)<br>P_a2_1/c (14.80)	51-78	(**8e**, 0.95)<br>(**8e**, 1.02)	
541	Sr_2TbIrO_6	$P2_1/n$ (14)<br>$P2_1/c$ (14.75)	51	(**2b**, 6.6)<br>(**2c**, 0.0)	

Continued on next page

. . . continued

No.	Compound	SG & MSG	T_c [K]	(WP, $\|\boldsymbol{m}\|/\mu_B$)	Band Gap [eV]
542	Nd_5Ge_4	$Pnma$ (62)<br>$Pnm'a'$ (62.447)	51	(**4c**, 1.84)<br>(**8d**, 3.17)<br>(**8d**, 2.74)	
543	$Mn_6Ni_{16}Si_7$	$Fm\bar{3}m$ (225)<br>P_A2_1/c (14.83)	50	(**4i**, 4.73)<br>(**4i**, 4.73)<br>(**4g**, 0.65)	
544	$NaFe_3(SO_4)_2(OH)_6$	$R\bar{3}m$ (166)<br>$R_I\bar{3}c$ (167.108)	50	(**18e**, 4.51)	
545	$Fe_{1.05}Te$	$P4/nmm$ (129)<br>P_a2_1/m (11.55)	50	(**4e**, 1.78)<br>(**4e**, 1.78)	
546	$Fe_{1.05}Te$	$P4/nmm$ (129)<br>P_a2_1/m (11.55)	50	(**4e**, 2.54)<br>(**4e**, 0.0)	
547	$BaNi_2V_2O_8$	$R\bar{3}$ (148)<br>$P_S\bar{1}$ (2.7)	50	(**4i**, 1.55)	
548	La_2CuO_4	$Cmce$ (64)<br>$'P_Accn'$ (56.374)	50	(**4a**, 0.17)	
549	$HoMn_{0.95}Fe_{0.05}O_3$	$P6_3cm$ (185)<br>$P6_3'cm'$ (185.200)	50	(**2a**, 3.8)<br>(**4b**, 1.1)<br>(**6c**, 2.97)<br>(**6c**, 2.97)	
550	Bi_2CuO_4	$P4/ncc$ (130)<br>$P4/n'c'c'$ (130.431)	50	(**4c**, 0.93)	
551	Co_2SiO_4	$Pnma$ (62)<br>$Pnma$ (62.441)	49.5	(**4a**, 3.88)<br>(**4c**, 3.64)	
552	$MnSe_2$	$Pa\bar{3}$ (205)<br>$Pbca$ (61.433)	49	(**4a**, 1.0)<br>(**8c**, 1.0)	
553	Co_2SiO_4	$Pnma$ (62)<br>$Pnma$ (62.441)	49	(**4a**, 3.87)<br>(**4c**, 3.35)	
554	Ba_2YbRuO_6	$Fm\bar{3}m$ (225)<br>P_I4/mnc (128.410)	48	(**2b**, 1.0)<br>(**2a**, 2.57)	

Continued on next page

...continued

No.	Compound	SG & MSG	T_c [K]	(WP, $\|m\|/\mu_B$)	Band Gap [eV]
555	CeScGe	$I4/mmm$ (139)<br>C_Amcm (63.468)	48	(**8i**, 0.76)	
556	Nd_2BaNiO_5	$Immm$ (71)<br>C_c2/c (15.90)	48	(**8i**, 2.65)<br>(**4c**, 1.58)	
557	$Fe_2F_5(H_2O)_2$	$Imma$ (74)<br>$Imm'a'$ (74.559)	48	(**4c**, 3.86)<br>(**4a**, 2.8)	
558	$MnLaMnSbO_6$	$P4_2/n$ (86)<br>$P2'/c'$ (13.69)	48	(**2f**, 4.8)<br>(**2f**, 4.8)<br>(**2a**, 4.8)<br>(**2b**, 4.8)	
559	Bi_2CuO_4	$P4/ncc$ (130)<br>$P4/n'c'c'$ (130.431)	47.5	(**4c**, 0.45)	
560	Mn_2SiO_4	$Pnma$ (62)<br>$Pn'm'a$ (62.446)	47.1	(**4a**, 3.84)<br>(**4c**, 4.67)	
561	$TbPt_{0.8}Cu_{0.2}$	$Pnma$ (62)<br>$Pn'm'a$ (62.446)	47	(**4c**, 8.29)	
562	Mn_2GeO_4	$Pnma$ (62)<br>$Pn'm'a$ (62.446)	47	(**4a**, 3.02)<br>(**4c**, 4.5)	
563	NdSi	$Pnma$ (62)<br>$Pn'm'a$ (62.446)	46	(**4c**, 2.59)	
564	La_3OsO_7	$Cmcm$ (63)<br>P_a2_1/m (11.55)	45	(**4a**, 1.69)	
565	La_3OsO_7	$Cmcm$ (63)<br>P_a2_1/m (11.55)	45	(**4a**, 1.70)	
566	$Mn_5(VO_4)_2(OH)_4$	$C2/m$ (12)<br>P_b2/c (13.71)	45	(**4f**, 1.98)<br>(**4f**, 1.98)<br>(**4e**, 2.43)<br>(**4e**, 2.43)<br>(**4f**, 2.48)	
567	$NaMnO_2$	$C2/m$ (12)<br>$P_S\bar{1}$ (2.7)	45	(**2a**, 2.47)	

Continued on next page

...continued

No.	Compound	SG & MSG	T_c [K]	(WP, $\|\boldsymbol{m}\|/\mu_B$)	Band Gap [eV]
568	$HoMn_{0.99}Fe_{0.01}O_3$	$P6_3cm$ (185)<br>$P6_3'cm'$ (185.200)	45	(**2a**, 4.1)<br>(**4b**, 1.5)<br>(**6c**, 3.17)<br>(**6c**, 3.17)	
569	$Mn_2Fe_{0.8}Mo_{1.2}O_6$	$P2_1/n$ (14)<br>$P2_1/c$ (14.75)	45	(**4e**, 2.10)<br>(**2c**, 2.63)<br>(**2b**, 0.8)<br>(**2c**, 2.63)	
570	$HoMnO_3$	$P6_3cm$ (185)<br>$P6_3cm$ (185.197)	44.6	(**6c**, 3.84)	
571	$GdIn_3$	$Pm\bar{3}m$ (221)<br>P_C4/mbm (127.397)	44.15	(**2c**, 1.0)	
572	$GdIn_3$	$Pm\bar{3}m$ (221)<br>P_C4/mbm (127.397)	44	(**2c**, 1.0)	
573	Nd_5Pb_3	$P6_3/mcm$ (193)<br>P_Bnma (62.454)	44	(**8e**, 2.7)<br>(**8g**, 0.0)<br>(**4c**, 2.1)	
574	$Tb_2Fe_2Si_2C$	$C2/m$ (12)<br>C_c2/m (12.63)	44	(**8i**, 8.0)	
575	$LaMn_3V_4O_{12}$	$Im\bar{3}$ (204)<br>$R_I\bar{3}$ (148.20)	44	(**18e**, 4.17)	
576	Sr_2YbRuO_6	$P2_1/n$ (14)<br>$P2_1/c$ (14.75)	44	(**2b**, 0.92)<br>(**2c**, 3.00)	
577	YFe_4Ge_2	$Pnnm$ (58)<br>$Pn'n'm'$ (58.399)	43.5	(**4g**, 0.63)<br>(**4g**, 0.63)	
578	TbB_4	$P4/mbm$ (127)<br>$Pb'a'm'$ (55.359)	43	(**4g**, 7.7)	
579	Ho_3NiGe_2	$Pnma$ (62)<br>$Pnm'a'$ (62.447)	43	(**4c**, 9.08)<br>(**4c**, 9.07)<br>(**4c**, 9.08)<br>(**4c**, 0.0)	

Continued on next page

…continued

No.	Compound	SG & MSG	T_c [K]	(WP, $\|\boldsymbol{m}\|/\mu_B$)	Band Gap [eV]
580	Ba_2TmRuO_6	$Fm\bar{3}m$ (225)<br>P_I4/mnc (128.410)	42	(**2b**, 1.91)<br>(**2a**, 2.13)	
581	GdB_4	$P4/mbm$ (127)<br>$P4/m'b'm'$ (127.395)	42	(**4g**, 7.14)	
582	Mn_2NiReO_6	$P2_1/n$ (14)<br>$P2_1/c$ (14.75)	42	(**4e**, 3.97)<br>(**2b**, 1.24)<br>(**2c**, 0.62)	
583	$MnAl_2O_4$	$Fd\bar{3}m$ (227)<br>$I4_1'/a'm'd$ (141.556)	42.0	(**4a**, 3.71)	
584	$NdCo_2$	$Fd\bar{3}m$ (227)<br>$Imm'a'$ (74.559)	42	(**4e**, 2.38)<br>(**4c**, 0.65)<br>(**4b**, 0.63)	
585	$Co_2Mo_3O_8$	$P6_3mc$ (186)<br>$P6_3'm'c$ (186.205)	42	(**2b**, 3.3)<br>(**2b**, 3.3)	
586	$NdCo_2$	$Fd\bar{3}m$ (227)<br>$C2'/c'$ (15.89)	42	(**4e**, 2.80)<br>(**4b**, 0.73)<br>(**4d**, 0.73)	
587	Cu_4O_3	$I4_1/amd$ (141)<br>$I_c\bar{4}2m$ (121.332)	41	(**16j**, 0.46)<br>(**16k**, 0.46)	
588	$Ba_2Co_9O_{14}$	$R\bar{3}m$ (166)<br>C_c2/m (12.63)	41	(**8i**, 2.88)<br>(**4a**, 1.76)	
589	$Ba_2Co_9O_{14}$	$R\bar{3}m$ (166)<br>C_c2/c (15.90)	41	(**8i**, 3.63)<br>(**4c**, 2.32)	
590	U_2Pd_2Sn	$P4/mbm$ (127)<br>$P4'/m'bm'$ (127.394)	41	(**4h**, 1.89)	
591	Sr_2TbRuO_6	$P2_1/n$ (14)<br>$P2_1/c$ (14.75)	41	(**2b**, 4.98)<br>(**2c**, 2.99)	
592	U_2Pd_2In	$P4/mbm$ (127)<br>$P4'/m'bm'$ (127.394)	40.8	(**4h**, 1.72)	
593	NpTe	$Fm\bar{3}m$ (225)<br>$F_Sd\bar{3}c$ (228.139)	40	(**32b**, 1.40)	

Continued on next page

…continued

No.	Compound	SG & MSG	T_c [K]	(WP, $\|m\|/\mu_B$)	Band Gap [eV]
594	Ce_5TeO_8	$Fd\bar{3}m$ (227)<br>$I4_1/am'd'$ (141.557)	40	(**4a**, 1.94)<br>(**8d**, 2.39)	
595	$(Ho_{0.8}Mn_{0.2})MnO_3$	$Pnma$ (62)<br>$Pnm'a'$ (62.447)	40	(**4c**, 5.86)<br>(**4c**, 3.57)<br>(**4b**, 2.33)	
596	Cr_2F_5	$C2/c$ (15)<br>$C2/c$ (15.85)	40	(**4a**, 2.32)<br>(**4b**, 3.72)	
597	Co_3O_4	$Fd\bar{3}m$ (227)<br>$I4_1'/a'm'd$ (141.556)	40	(**4a**, 3.26)<br>(8d,0.0)	
598	$HoMnO_3$	$P6_3cm$ (185)<br>$P6_3'cm'$ (185.200)	40	(**2a**, 4.17)<br>(**4b**, 1.31)<br>(**6c**, 3.32)	
599	UGeSe	$I4/mmm$ (139)<br>$I4/m'm'm'$ (139.539)	40	(**4e**, 0.26)	
600	U_2Pd_2Sn	$P4/mbm$ (127)<br>$P4'/m'bm'$ (127.394)	40	(**4h**, 2.19)	
601	Sr_2DyRuO_6	$P2_1/n$ (14)<br>$P2_1'/c'$ (14.79)	39.5	(**2c**, 4.92)<br>(**2b**, 1.94)	
602	$BiMn_2O_5$	$Pbam$ (55)<br>C_amc2_1 (36.178)	39	(**16c**, 2.51)<br>(**8b**, 3.22)<br>(**8b**, 3.22)	
603	$CeRh_2Si_2$	$I4/mmm$ (139)<br>C_Amca (64.480)	39	(**4a**, 1.28)	
604	NiS_2	$Pa\bar{3}$ (205)<br>$P_S\bar{1}$ (2.7)	39	(**2a**, 1.26)<br>(**2d**, 0.65)<br>(**2e**, 0.65)<br>(**2c**, 1.26)	
605	$Co_2Mo_3O_8$	$P6_3mc$ (186)<br>$P6_3'm'c$ (186.205)	39	(**2b**, 3.44)<br>(**2b**, 3.35)	
606	CoF_2	$P4_2/mnm$ (136)<br>$P4_2'/mnm'$ (136.499)	39	(**2a**, 2.6)	

Continued on next page

. . . continued

No.	Compound	SG & MSG	T_c [K]	(WP, $\|\boldsymbol{m}\|/\mu_B$)	Band Gap [eV]
607	$Pr_3Ru_4Al_{12}$	$P6_3/mmc$ (194)<br>$Cm'c'm$ (63.462)	39	(**4c**, 3.1)<br>(**8g**, 1.4)	
608	NiS_2	$Pa\bar{3}$ (205)<br>$Pa\bar{3}$ (205.33)	39	(**4a**, 0.98)	
609	$Nd_3Ru_4Al_{12}$	$P6_3/mmc$ (194)<br>$Cm'c'm$ (63.462)	39	(**4c**, 0.95)<br>(**8g**, 2.66)	
610	NpSe	$Fm\bar{3}m$ (225)<br>$F_Sd\bar{3}c$ (228.139)	38	(**32b**, 1.29)	
611	$FeCO_3$	$R\bar{3}c$ (167)<br>$R\bar{3}c$ (167.103)	38	(**6b**, 1.0)	
612	$Tb_{0.6}Y_{0.4}RhIn_5$	$P4/mmm$ (123)<br>C_amma (67.509)	37.6	(**4e**, 8.8)	
613	$NdNi_{0.6}Cu_{0.4}$	$Pnma$ (62)<br>$Pnm'a'$ (62.447)	37.5	(**4c**, 2.59)	
614	$NiSO_4$	$Cmcm$ (63)<br>P_Cbcn (60.431)	37	(**4a**, 2.1)	
615	DyGe	$Cmcm$ (63)<br>C_c2/c (15.90)	37	(**8i**, 9.0)	
616	$SmCrO_3$	$Pbnm$ (62)<br>$Pbn'm'$ (62.446)	37	(**4b**, 2.98)<br>(**4c**, 0.53)	
617	$SmCrO_3$	$Pbnm$ (62)<br>$Pn'm'a$ (62.446)	37	(**4b**, 2.86)<br>(**4c**, 0.41)	
618	$[NH_2(CH_3)_2]_n[Fe^{III}Fe^{II}(HCOO)_6]_n$	$R\bar{3}c$ (167)<br>$R\bar{3}c'$ (167.107)	37	(**18e**, 4.02)<br>(**6b**, 4.1)<br>(**12c**, 4.1)	
619	Li_2MnO_3	$C2/m$ (12)<br>C_c2/m (12.63)	36	(**8h**, 2.61)	
620	$SrNdFeO_4$	$I4/mmm$ (139)<br>C_Amca (64.480)	36	(**8g**, 2.45)<br>(**4a**, 4.23)	
621	CeScGe	$I4/mmm$ (139)<br>$P_S\bar{1}$ (2.7)	36	(**4i**, 1.42)	

Continued on next page

…continued

No.	Compound	SG & MSG	T_c [K]	(WP, $\|m\|/\mu_B$)	Band Gap [eV]
622	$NpRhGa_5$	$P4/mmm$ (123)<br>P_c4/mcc (124.360)	36	(2b,0.0)<br>(**2a**, 0.32)	
623	VCl_2	$P\bar{3}m1$ (164)<br>P_c31c (159.64)	36	(**6c**, 1.19)	
624	$CeRh_2Si_2$	$I4/mmm$ (139)<br>C_Amca (64.480)	36	(**4a**, 1.5)	
625	$NaFeSO_4F$	$C2/c$ (15)<br>P_C2/c (13.74)	36	(**4a**, 4.42)	
626	$RbCoBr_3$	$P6_3/mmc$ (194)<br>$P6_3'/m'cm'$ (193.259)	36	(**2b**, 3.4)<br>(**4d**, 3.4)	
627	U_2Pd_2In	$P4/mbm$ (127)<br>$P4'/m'bm'$ (127.394)	36	(**4h**, 1.40)	
628	Sr_2HoRuO_6	$P2_1/n$ (14)<br>$P2_1/c$ (14.75)	36	(**2b**, 3.02)<br>(**2c**, 2.19)	
629	Sr_2HoRuO_6	$P2_1/n$ (14)<br>$P2_1/c$ (14.75)	36	(**2b**, 6.66)<br>(**2c**, 2.74)	
630	Sr_2TmRuO_6	$P2_1/n$ (14)<br>$P2_1/c$ (14.75)	36	(**2b**, 1.4)<br>(**2c**, 1.5)	
631	Sr_2YbRuO_6	$P112_1/n$ (14)<br>$P2_1/c$ (14.75)	36	(**2b**, 0.54)<br>(**2c**, 2.09)	
632	Mn_3TeO_6	$P2_1/c$ (14)<br>P_a2_1/c (14.80)	35.8	(**4c**, 4.02)<br>(**8e**, 4.69)	
633	$HoNi$	$Pnma$ (62)<br>$Pnm'a'$ (62.447)	35.5	(**4c**, 7.75)	
634	$NdMg$	$Pm\bar{3}m$ (221)<br>P_C4/nbm (125.373)	35	(**4f**, 2.55)	
635	Ba_2LuRuO_6	$Fm\bar{3}m$ (225)<br>C_Amca (64.480)	35	(**4a**, 4.4)	
636	$TbAg_2$	$I4/mmm$ (139)<br>C_Amca (64.480)	35	(**4a**, 8.95)	

Continued on next page

. . . continued

No.	Compound	SG & MSG	T_c [K]	(WP, $\|\boldsymbol{m}\|/\mu_B$)	Band Gap [eV]
637	Sr_2FeWO_6	$P2_1/n$ (14)<br>$P_S\bar{1}$ (2.7)	35	(**2a**, 3.85)<br>(**2c**, 3.85)	
638	Sc_2NiMnO_6	$P2_1/c$ (14)<br>P_a2_1/c (14.80)	35	(**4c**, 1.66)<br>(4b,0.0)	
639	$YMn_3Al_4O_{12}$	$Im\bar{3}$ (204)<br>P_Innm (58.404)	35	(**2b**, 2.92)<br>(**2d**, 2.92)<br>(**2c**, 2.92)	
640	$Cu_3Y(SeO_3)_2O_2Cl$	$Pmmn$ (59)<br>P_cccn (56.373)	35	(**8c**, 0.41)<br>(**4a**, 1.04)	
641	$KOsO_4$	$I4_1/a$ (88)<br>$I4_1'/a'$ (88.85)	35	(**4a**, 0.46)	
642	$(NH_2(CH_3)_2)(FeMn(HCOO)_6)$	$P\bar{3}c1$ (163)<br>$C2'/c'$ (15.89)	35	(**4e**, 6.28)<br>(**4a**, 5.81)	
643	UPt_2Si_2	$P4/nmm$ (129)<br>$P4/n'm'm'$ (129.419)	35	(**2c**, 1.67)	
644	$LiFe(SO_4)_2$	$P2_1/c$ (14)<br>$P2_1/c$ (14.75)	35	(**2a**, 4.33)	
645	$NdMn_2Si_2$	$I4/mmm$ (139)<br>$Pmm'n'$ (59.410)	34	(**2a**, 2.83)<br>(**4f**, 1.60)	
646	$PrMnSi_2$	$Cmcm$ (63)<br>P_Bnna (52.318)	34	(**4c**, 2.35)<br>(**4c**, 2.04)	
647	Ni_2SiO_4	$Pnma$ (62)<br>P_c2_1/c (14.82)	34	(**4c**, 2.03)<br>(**4d**, 2.03)<br>(**4e**, 2.08)<br>(**4e**, 2.08)	
648	Ni_2SiO_4	$Pnma$ (62)<br>P_c2_1/c (14.82)	34	(**4c**, 1.41)<br>(**4d**, 1.41)<br>(**4e**, 1.41)<br>(**4e**, 1.41)	
649	Sr_2HoRuO_6	$P2_1/n$ (14)<br>$P2_1/c$ (14.75)	34	(**2c**, 7.92)<br>(**2b**, 1.80)	

Continued on next page

...continued

No.	Compound	SG & MSG	T_c [K]	(WP, $\|\boldsymbol{m}\|/\mu_B$)	Band Gap [eV]
650	$CoGeO_3$	$C2/c$ (15)<br>P_C2_1/c (14.84)	33.5	(**4e**, 2.69)<br>(**4e**, 4.77)	
651	$CoGeO_3$	$C2/c$ (15)<br>$C2'/c'$ (15.89)	33.5	(**4e**, 2.67)<br>(**4e**, 2.67)<br>(**4e**, 2.67)<br>(**4e**, 4.61)<br>(**4e**, 4.61)<br>(**4e**, 4.61)	
652	Mn_3O_4	$I4_1/amd$ (141)<br>$Pb'c'n$ (60.422)	33	(**8d**, 4.34)<br>(**4c**, 3.64)<br>(**8d**, 3.25)<br>(**4b**, 3.64)	
653	$La_{2.8}Ca_{0.2}OsO_7$	$Cmcm$ (63)<br>P_a2_1/m (11.55)	33	(**4a**, 1.20)	
654	CeC_2	$I4/mmm$ (139)<br>P_I4/mnc (128.410)	33	(**2a**, 1.74)	
655	$NpRhGa_5$	$P4/mmm$ (123)<br>C_cmcm (63.466)	33	(4d,0.0)<br>(**4a**, 0.89)	
656	Er_2BaNiO_5	$Immm$ (71)<br>C_c2/c (15.90)	33	(**8i**, 7.89)<br>(**4c**, 1.53)	
657	$HoMnO_3$	$P6_3cm$ (185)<br>$P6_3'cm'$ (185.200)	33	(**2a**, 4.2)<br>(**4b**, 1.3)<br>(**6c**, 3.01)	
658	$CoGeO_3$	$C2/c$ (15)<br>P_C2_1/c (14.84)	32.5	(**4e**, 2.71)<br>(**4e**, 4.72)	
659	$GeCu_2O_4$	$I4_1/amd$ (141)<br>$I_c\bar{4}2d$ (122.338)	32.2	(**32i**, 0.88)	
660	UPb_3	$Pm\bar{3}m$ (221)<br>P_c4/mcc (124.360)	32	(**2a**, 1.7)	
661	$(NH_2(CH_3)_2)(FeCo(HCOO)_6)$	$P\bar{3}c1$ (163)<br>$C2'/c'$ (15.89)	32	(**4e**, 3.26)<br>(**4a**, 5.80)	

Continued on next page

…continued

No.	Compound	SG & MSG	T_c [K]	(WP, $\|\boldsymbol{m}\|/\mu_B$)	Band Gap [eV]
662	$LuFe_4Ge_2$	$P4_2/mnm$ (136)<br>$Pn'n'm'$ (58.399)	32	(**4g**, 0.44)<br>(**4g**, 0.44)	
663	$MnCO_3$	$R\bar{3}c$ (167)<br>$C2/c$ (15.85)	32	(**4c**, 2.23)	
664	$LaMn_2O_5$	$Pbam$ (55)<br>P_cbam (55.361)	31	(**8f**, 1.61)<br>(**8h**, 2.59)	
665	$Ho(Co_{0.667}Ga_{0.333})_2$	$P6_3/mmc$ (194)<br>$C2'/c'$ (15.89)	31	(**8f**, 6.36)	
666	U_2Pd_2In	$P4/mbm$ (127)<br>$P4'/m'bm'$ (127.394)	31	(**4h**, 1.54)	
667	UO_2	$Fm\bar{3}m$ (225)<br>$Pn\bar{3}m'$ (224.113)	30.8	(**4c**, 1.73)	
668	$NiTe_2O_5$	$Pnma$ (62)<br>$Pnma$ (62.441)	30.5	(**8d**, 2.18)	
669	Fe_2MnBO_5	$Pbam$ (55)<br>$Pb'am'$ (55.358)	30	(**4e**, 1.6)<br>(**2c**, 3.0)<br>(**2d**, 3.0)<br>(**8i**, 1.6)<br>(**4g**, 2.2)<br>(**4h**, 2.2)	
670	$EuSn_2P_2$	$R\bar{3}m$ (166)<br>C_c2/m (12.63)	30	(**4a**, 5.65)	
671	$TbCo_2Si_2$	$I4/mmm$ (139)<br>P_I4/mnc (128.410)	30	(**2a**, 8.8)	
672	UCr_2Si_2	$C2/m$ (12)<br>C_c2/c (15.90)	30	(**4c**, 0.81)<br>(**8g**, 0.0)	
673	Nd_2CuO_4	$I4/mmm$ (139)<br>C_Accm (66.500)	30	(**8g**, 1.30)<br>(**4a**, 0.46)	
674	$Co_3(PO_4)_2$	$P2_1/c$ (14)<br>P_a2_1/c (14.80)	30	(**4a**, 3.76)<br>(**8e**, 3.48)	

Continued on next page

. . . continued

No.	Compound	SG & MSG	T_c [K]	(WP, $\|\boldsymbol{m}\|/\mu_B$)	Band Gap [eV]
675	La_2LiOsO_6	$P2_1/n$ (14)<br>$P_S\bar{1}$ (2.7)	30	(**2a**, 1.80)<br>(**2e**, 1.80)	
676	Sr_2LuRuO_6	$P2_1/n$ (14)<br>$P2_1/c$ (14.75)	30	(**2c**, 3.9)	
677	La_2LiRuO_6	$P2_1/n$ (14)<br>$P2_1/c$ (14.75)	30	(**2c**, 2.20)	
678	$Ni_3B_7O_{13}Br$	$Pca2_1$ (29)<br>$Pc'a2_1'$ (29.101)	30	(**4a**, 3.56)<br>(**4a**, 1.32)<br>(**4a**, 1.40)	
679	$NiCO_3$	$R\bar{3}c$ (167)<br>$C2/c$ (15.85)	30	(**4c**, 2.23)	
680	$Co_4Nb_2O_9$	$P\bar{3}c1$ (165)<br>$P\bar{3}'c'1$ (165.94)	30	(**4c**, 0.0)<br>(**4d**, 3.0)<br>(**4d**, 3.0)	
681	Tb_2Pd_2In	$P4/mbm$ (127)<br>C_amca (64.479)	29.5	(**8e**, 8.7)<br>(**8e**, 8.7)<br>(**16i**, 8.7)	
682	VBr_2	$P\bar{3}m1$ (164)<br>P_c31c (159.64)	29.5	(**6c**, 3.19)	
683	NdC_2	$I4/mmm$ (139)<br>P_I4/mnc (128.410)	29	(**2a**, 2.95)	
684	Sr_2CuTeO_6	$I4/m$ (87)<br>C_c2/c (15.90)	29	(**4c**, 0.69)	
685	$NaCoSO_4F$	$C2/c$ (15)<br>P_C2/c (13.74)	29	(**4a**, 4.06)	
686	Tb_2CoGa_8	$P4/mmm$ (123)<br>I_c4/mcm (140.550)	28.5	(**8g**, 9.6)	
687	La_2CoPtO_6	$P2_1/n$ (14)<br>P_a2_1/c (14.80)	28	(**4c**, 2.81)	
688	U_2Rh_2Sn	$P4/mbm$ (127)<br>P_c4_2/mbc (135.492)	28	(**8g**, 0.53)	

Continued on next page

…continued

No.	Compound	SG & MSG	T_c [K]	(WP, $\|m\|/\mu_B$)	Band Gap [eV]
689	Pr_3CoGe_2	$Pnma$ (62)<br>$Pnm'a'$ (62.447)	28	(**4c**, 3.03)<br>(**4c**, 3.03)<br>(**4c**, 3.03)<br>(**4c**, 0.0)	
690	$TmMn_3O_6$	$Pmmn$ (59)<br>$Pmm'n'$ (59.410)	28	(**2b**, 0.77)<br>(**2b**, 3.7)<br>(**2a**, 2.00)<br>(**2a**, 3.17)<br>(**4c**, 1.91)<br>(**4d**, 1.59)	
691	$Cu_3Bi(SeO_3)_2O_2Br$	$Pmmn$ (59)<br>P_cccn (56.373)	27.4	(**8c**, 0.91)<br>(**4a**, 0.9)	
692	$Na_2Mn_3Se_4$	$C2/m$ (12)<br>$P_S\bar{1}$ (2.7)	27	(**4i**, 3.89)<br>(**4i**, 4.11)<br>(**4i**, 3.25)	
693	$CeRh_2Si_2$	$I4/mmm$ (139)<br>P_Bcca (54.350)	27	(**4g**, 2.39)<br>(**4g**, 0.16)	
694	$CeRu_2Al_{10}$	$Cmcm$ (63)<br>P_Cbcm (57.391)	27	(**4c**, 0.34)	
695	Sr_2FeWO_5N	$I4/m$ (87)<br>$P_S\bar{1}$ (2.7)	27	(**2a**, 2.12)<br>(2f,0.0)	
696	$La_{0.25}Pr_{0.75}Co_2P_2$	$I4/mmm$ (139)<br>C_c2/c (15.90)	27	(4b,0.0)<br>(**4c**, 1.41)<br>(**16j**, 0.8)	
697	HoC_2	$I4/mmm$ (139)<br>P_amma (51.298)	26	(**2b**, 9.75)<br>(**4l**, 6.89)<br>(2a,0.0)	
698	$CoTi_2O_5$	$Cmcm$ (63)<br>P_a2_1/m (11.55)	26	(**4e**, 2.72)	
699	$CeScSi$	$I4/mmm$ (139)<br>C_Amcm (63.468)	26	(**8i**, 0.96)	

Continued on next page

... continued

No.	Compound	SG & MSG	T_c [K]	(WP, $\|\boldsymbol{m}\|/\mu_B$)	Band Gap [eV]
700	Ba_2CoO_4	$P2_1/n$ (14)<br>P_a2_1/c (14.80)	26	(**8e**, 2.69)	
701	$UPtGa_5$	$P4/mmm$ (123)<br>P_c4/mcc (124.360)	26	(2b,0.0)<br>(**2a**, 0.32)	
702	$Dy_2Fe_2Si_2C$	$C2/m$ (12)<br>C_c2/m (12.63)	26	(**8i**, 6.6)<br>(**8i**, 0.0)	
703	$TbMg_3$	$Fm\bar{3}m$ (225)<br>$R_I\bar{3}c$ (167.108)	26	(**6a**, 9.59)	
704	NdMgPb	$I4/mmm$ (139)<br>P_A2/c (13.73)	26	(**4i**, 3.37)	
705	Sr_2YRuO_6	$'P$ (14)<br>$P2_1/c$ (14.75)	26	(**2a**, 2.19)	
706	$Mn_2SeO_3F_2$	$Pnma$ (62)<br>$Pn'ma'$ (62.448)	26	(**8d**, 4.34)	
707	$Fe_2F_5(H_2O)_2$	$Imma$ (74)<br>$C2'/c'$ (15.89)	26	(**4a**, 3.88)<br>(**4c**, 4.91)	
708	$U_2Rh_3Si_5$	$I2/c$ (15)<br>P_C2/c (13.74)	26	(**8f**, 1.81)	
709	$BaMn_2Si_2O_7$	$C2/c$ (15)<br>$C2/c$ (15.85)	26	(**4e**, 3.9)<br>(**4e**, 3.9)<br>(**8f**, 3.9)	
710	$Mn_3B_7O_{13}I$	$Pca2_1$ (29)<br>$Pc'a2_1'$ (29.101)	26	(**4a**, 5.41)<br>(**4a**, 3.8)<br>(**4a**, 3.8)	
711	$HoMnO_3$	$P6_3cm$ (185)<br>$P6_3cm$ (185.197)	25.4	(2a,0.0)<br>(**4b**, 2.87)<br>(**6c**, 3.93)	
712	$CoRh_2O_4$	$Fd\bar{3}m$ (227)<br>$I4_1'/a'm'd$ (141.556)	25.2	(**4a**, 3.11)	
713	$Na_{2.4}Ni_2TeO_6$	$P6_3/mcm$ (193)<br>P_Anma (62.453)	25	(**8e**, 1.47)	

Continued on next page

. . . continued

No.	Compound	SG & MSG	T_c [K]	(WP, $\|m\|/\mu_B$)	Band Gap [eV]
714	U_2Ni_2Sn	$P4/mbm$ (127)<br>P_c4_2/mbc (135.492)	25	(**8g**, 0.87)	
715	Ba_2CoO_4	$P2_1/n$ (14)<br>P_a2_1/c (14.80)	25	(**8e**, 3.43)	
716	$CoNb_3S_6$	$P6_322$ (182)<br>$P_B2_12_12$ (18.22)	25	(**4a**, 2.6)	
717	Ba_2FeWO_6	$I4/m$ (87)<br>$P_S\bar{1}$ (2.7)	25	(**2e**, 3.42)	
718	$Fe_2(C_2O_4)_{3.4}H_2O$	$P\bar{1}$ (2)<br>$P_S\bar{1}$ (2.7)	25	(**4i**, 4.25)	
719	$CoCl_2$	$R\bar{3}m$ (166)<br>C_c2/c (15.90)	25	(**4c**, 3.86)	
720	U_2Rh_2Sn	$P4/mbm$ (127)<br>P_c4_2/mbc (135.492)	25	(**8g**, 0.5)<br>(**8h**, 0.05)	
721	U_2Ni_2Sn	$P4/mbm$ (127)<br>C_cmcm (63.466)	25	(**8g**, 1.41)<br>(**8i**, 1.41)	
722	$LiFeSO_4F$	$P\bar{1}$ (2)<br>$P_S\bar{1}$ (2.7)	25	(**2d**, 3.50)<br>(**2a**, 3.50)	
723	$La_{0.8}Bi_{0.2}Mn_2O_5$	$Pbam$ (55)<br>P_cbam (55.361)	25	(**8f**, 1.58)<br>(**8h**, 1.83)	
724	$Rb_2Fe_2O(AsO_4)_2$	$Pnma$ (62)<br>$Pn'ma'$ (62.448)	25	(**4c**, 3.1)<br>(**4b**, 3.09)	
725	$Rb_2Fe_2O(AsO_4)_2$	$Pnma$ (62)<br>$Pnma$ (62.441)	25	(**4c**, 3.64)<br>(**4b**, 3.18)	
726	$Tm_2Mn_2O_7$	$Fd\bar{3}m$ (227)<br>$I4_1/am'd'$ (141.557)	25	(**8c**, 1.84)<br>(**8d**, 2.31)	
727	$CsMn_2F_6$	$Pnma$ (62)<br>$Pnm'a'$ (62.447)	24.1	(**4b**, 3.25)<br>(**4c**, 0.59)	
728	$DyGe_3$	$Cmcm$ (63)<br>P_a2_1/m (11.55)	24	(**8f**, 8.2)<br>(**4e**, 8.1)	

Continued on next page

. . . continued

No.	Compound	SG & MSG	T_c [K]	(WP, $\|m\|/\mu_B$)	Band Gap [eV]
729	Nd_2BaCoO_5	$Immm$ (71)<br>C_c2/c (15.90)	24	(**8i**, 2.55)<br>(**4c**, 3.07)	
730	$MnBi_2Te_4$	$R\bar{3}m$ (166)<br>$R_I\bar{3}c$ (167.108)	24	(**6a**, 4.7)	
731	$MnBi_2Te_4$	$R\bar{3}m$ (166)<br>$R_I\bar{3}c$ (167.108)	24	(**6a**, 4.04)	
732	$FeCl_2$	$R\bar{3}m$ (166)<br>$R_I\bar{3}c$ (167.108)	24	(**6a**, 4.5)	
733	Sr_2CuWO_6	$I4/m$ (87)<br>$P_S\bar{1}$ (2.7)	24	(**2a**, 0.57)	
734	K_2ReI_6	$P2_1/n$ (14)<br>$P2_1/c$ (14.75)	24	(**2a**, 2.2)	
735	K_2ReI_6	$P2_1/n$ (14)<br>$P2_1/c$ (14.75)	24	(**2a**, 2.2)	
736	$LiFe(MoO_4)_2$	$P\bar{1}$ (2)<br>$P_S\bar{1}$ (2.7)	23.8	(**4i**, 4.43)	
737	$EuSn_2As_2$	$R\bar{3}m$ (166)<br>C_c2/m (12.63)	23.5	(**4a**, 4.5)	
738	NpS	$Fm\bar{3}m$ (225)<br>$F_Sd\bar{3}c$ (228.139)	23	(**32b**, 0.69)	
739	$TbGe_3$	$Cmcm$ (63)<br>P_cnma (62.452)	23	(**16e**, 8.15)<br>(**8d**, 8.3)	
740	Ba_2CoO_4	$P2_1/n$ (14)<br>P_a2_1/c (14.80)	23	(**8e**, 3.20)	
741	NdPt	$Cmcm$ (63)<br>$C2'/c'$ (15.89)	23	(**4e**, 2.34)	
742	DyPt	$Pnma$ (62)<br>$Pn'm'a$ (62.446)	23	(**4c**, 7.10)	
743	$Li_3Fe_2(PO_4)_3$	$P2_1/n$ (14)<br>$P2_1'/c'$ (14.79)	23	(**4e**, 4.7)<br>(**4e**, 4.0)	

Continued on next page

…continued

No.	Compound	SG & MSG	T_c [K]	(WP, $\|\boldsymbol{m}\|/\mu_B$)	Band Gap [eV]
744	UP	$Fm\bar{3}m$ (225)<br>P_C4_2/nnm (134.481)	22.5	(**4c**, 1.90)	
745	$KRuO_4$	$I4_1/a$ (88)<br>$I4_1'/a'$ (88.85)	22.4	(**4a**, 0.57)	
746	$Cu_3Ni_2SbO_6$	$C2/c$ (15)<br>P_C2/c (13.74)	22.3	(**8f**, 1.55)<br>(4c,0.0)	
747	Mn_2ScSbO_6	$P2_1/n$ (14)<br>$P2_1/c$ (14.75)	22.3	(**4e**, 4.54)	
748	$EuMnBi_2$	$I4/mmm$ (139)<br>$P4_2'/m'm'c$ (131.440)	22	(**4i**, 6.4)<br>(**2e**, 3.4)<br>(**2f**, 3.4)	
749	$Ba_3LaRu_2O_9$	$P6_3/mmc$ (194)<br>P_C2_1/c (14.84)	22	(**8f**, 1.43)	
750	$NiTiO_3$	$R\bar{3}$ (148)<br>$P_S\bar{1}$ (2.7)	22	(**4i**, 1.46)	
751	CeScSi	$I4/mmm$ (139)<br>$P_S\bar{1}$ (2.7)	22	(**4i**, 1.50)	
752	$BaMo(PO_4)_2$	$C2/m$ (12)<br>$P_S\bar{1}$ (2.7)	22	(**2d**, 1.37)	
753	$U_{14}Au_{51}$	$P6/m$ (175)<br>$P6/m'$ (175.140)	22	(**6k**, 2.31)<br>(**6j**, 1.32)<br>(**2e**, 0.0)	
754	$U_{14}Au_{51}$	$P6/m$ (175)<br>$P6'/m$ (175.139)	22	(**6k**, 0.5)<br>(**6j**, 1.8)<br>(2e,0.0)	
755	$CsCoCl_3$	$P6_3/mmc$ (194)<br>$P6_3'/m'cm'$ (193.259)	21.5	(**2b**, 2.3)<br>(**4d**, 2.3)	
756	$Tb_2Ni_{1.78}In$	$P/4mbm$ (127)<br>C_amca (64.479)	21	(**8e**, 7.55)<br>(**8e**, 7.55)<br>(**16i**, 7.55)	

Continued on next page

. . . continued

No.	Compound	SG & MSG	T_c [K]	(WP, $\|m\|/\mu_B$)	Band Gap [eV]
757	PrFeAsO	$Cmme$ (67)<br>P_Acc2 (27.85)	21	(**4c**, 1.0)<br>(**4b**, 0.5)	
758	$FeSO_4$	$Cmcm$ (63)<br>P_Cbcn (60.431)	21	(**4a**, 4.1)	
759	$BaMoP_2O_8$	$C2/m$ (12)<br>$P_S\bar{1}$ (2.7)	21	(**2c**, 1.17)	
760	$DyCo_2Si_2$	$I4/mmm$ (139)<br>P_I4/mnc (128.410)	21	(**2a**, 9.5)	
761	$CeOs_{1.84}Ir_{0.16}Al_{10}$	$Cmcm$ (63)<br>P_Anma (62.453)	21	(**4c**, 0.92)	
762	$NdCrTiO_5$	$Pbam$ (55)<br>$Pbam'$ (55.356)	21	(**4g**, 2.89)<br>(**4h**, 2.95)<br>(**4f**, 2.95)	
763	$CsCoCl_3$	$P6_3/mmc$ (194)<br>$P6_3'/m'cm'$ (193.259)	20.82	(**2b**, 2.8)<br>(**4d**, 2.66)	
764	$Tb_2Pd_{2.05}Sn_{0.95}$	$P4/mbm$ (127)<br>P_bnma (62.451)	20.8	(**8h**, 8.69)	
765	$Cs_2Cu_3SnF_{12}$	$P2_1/n$ (14)<br>$P2_1'/c'$ (14.79)	20.2	(**2c**, 0.72)<br>(**4e**, 0.69)	
766	$Ca_3CuNi_2(PO_4)_4$	$C2/c$ (15)<br>C_a2/c (15.91)	20	(**8a**, 0.50)<br>(**8b**, 0.50)<br>(**16g**, 1.80)<br>(**16g**, 1.80)	
767	Ba_2MnTeO_6	$R\bar{3}m$ (166)<br>P_A2_1/c (14.83)	20	(**2a**, 4.04)	
768	$AgNiO_2$	$P6_322$ (182)<br>$P_B2_12_12$ (18.22)	20	(**4b**, 1.55)<br>(**4b**, 0.0)<br>(**4b**, 0.0)	
769	$LiFeGe_2O_6$	$P2_1/c$ (14)<br>P_a2_1/c (14.80)	20	(**8e**, 4.71)	

Continued on next page

. . . continued

No.	Compound	SG & MSG	T_c [K]	(WP, $\|m\|/\mu_B$)	Band Gap [eV]
770	$ErFeCuGe_4O_{12}$	$P4/nbm$ (125)<br>P_c4/nnc (126.384)	20	(**4a**, 7.9)<br>(**8e**, 2.43)	
771	$FeF_3(H_2O)_2H_2O$	$P4/n$ (85)<br>P_b2_1/c (14.81)	20	(**4e**, 3.23)	
772	$KCo_4(PO_4)_3$	$Pnnm$ (58)<br>$Pnn'm'$ (58.398)	20	(**4g**, 2.16)<br>(**4g**, 3.18)<br>(**8h**, 3.14)	
773	Bi_2RuMnO_7	$Fd\bar{3}m$ (227)<br>$Fd'd'd$ (70.530)	20	(**16c**, 1.78)	
774	$EuFe_2As_2$	$I4/mmm$ (139)<br>P_Cbca (61.439)	19	(**4a**, 6.8)<br>(**8e**, 0.98)	
775	Pu_2O_3	$P\bar{3}m1$ (164)<br>C_c2/c (15.90)	19	(**8i**, 0.6)	
776	$BaNi_2As_2O_8$	$R\bar{3}$ (148)<br>$P_S\bar{1}$ (2.7)	19	(**4i**, 2.0)	
777	$CoBr_2$	$P\bar{3}m1$ (164)<br>C_c2/c (15.90)	19	(**4c**, 3.57)	
778	$NaBaFe_2F_9$	$P2_1/c$ (14)<br>$P2_1/c$ (14.75)	19	(**4e**, 3.53)<br>(**4e**, 3.55)	
779	$NH_4Fe_2F_6$	$Pnma$ (62)<br>$Pnma$ (62.441)	19	(**4a**, 4.12)<br>(**4c**, 3.12)	
780	$Fe(N(CN_2))_2$	$Pnnm$ (58)<br>$Pnn'm'$ (58.398)	19	(**2a**, 4.11)	
781	$CsMnF_4$	$P4/nmm$ (129)<br>$Pmm'n'$ (59.410)	18.9	(**4c**, 4.0)	
782	$DyBaCuO_5$	$Pnma$ (62)<br>P_a2_1/c (14.80)	18.5	(**8e**, 0.1)<br>(**8e**, 3.00)<br>(**8e**, 0.55)	
783	$Cu_3Co_2SbO_6$	$C2/c$ (15)<br>P_C2_1/c (14.84)	18.5	(**8f**, 2.4)<br>(4a,0.0)	

Continued on next page

…continued

No.	Compound	SG & MSG	T_c [K]	(WP, $\|m\|/\mu_B$)	Band Gap [eV]
784	$Er(Co_{0.667}Ga_{0.333})_2$	$P6_3/mmc$ (194)<br>$P6_3/mm'c'$ (194.270)	18.5	(**4f**, 6.07)	
785	$CaCoGe_2O_6$	$C2/c$ (15)<br>P_C2_1/c (14.84)	18.1	(**4e**, 3.35)	
786	Na_2IrO_3	$C2/m$ (12)<br>C_c2/m (12.63)	18.1	(**8h**, 0.22)<br>(4c,0.0)	
787	$NpNiGa_5$	$P4/mmm$ (123)<br>$Imm'a'$ (74.559)	18	(**4d**, 0.83)<br>(**4e**, 0.0)	
788	$ErRh_2Si_2$	$I4/mmm$ (139)<br>P_Innm (58.404)	18	(**2a**, 7.7)<br>(**4f**, 0.0)	
789	$PrMn_2O_5$	$Pbam$ (55)<br>P_bnma (62.451)	18	(**8h**, 0.0)<br>(**8f**, 2.62)<br>(**8g**, 0.59)	
790	$Na_{0.5}Li_{0.5}FeGe_2O_6$	$P2_1/c$ (14)<br>P_a2_1/c (14.80)	18	(**8e**, 4.3)	
791	$DyFeWO_6$	$Pna2_1$ (33)<br>C_ac (9.41)	18	(**8a**, 7.73)<br>(**8a**, 7.73)<br>(**8a**, 4.92)<br>(**8a**, 4.92)	
792	Gd_2CuO_4	$Aeam$ (64)<br>P_Accn (56.374)	18	(**4a**, 1.21)	
793	Pb_2MnO_4	$P\bar{4}2_1c$ (114)<br>$P\bar{4}'2_1c'$ (114.278)	18	(**8e**, 2.74)	
794	$CoCO_3$	$R\bar{3}c$ (167)<br>$C2/c$ (15.85)	18	(**4c**, 2.23)	
795	$HoBaCuO_5$	$Pnma$ (62)<br>P_a2_1/c (14.80)	17.5	(**8e**, 0.49)<br>(**8e**, 2.26)<br>(**8e**, 0.44)	
796	URu_2Si_2	$I4/mmm$ (139)<br>P_I4/mnc (128.410)	17.5	(**2a**, 0.04)	

Continued on next page

…continued

No.	Compound	SG & MSG	T_c [K]	(WP, $\|m\|/\mu_B$)	Band Gap [eV]
797	$BiNiO(PO_4)$	$P2_1/n$ (14)<br>P_a2_1/c (14.80)	17.5	(**8e**, 1.99)	
798	$Co_3B_7O_{13}Br$	$Pca2_1$ (29)<br>$Pc'a2_1'$ (29.101)	17.5	(**4a**, 4.7)<br>(**4a**, 4.03)<br>(**4a**, 1.70)	
799	$ZnMnO_3$	$R\bar{3}$ (148)<br>$P_S\bar{1}$ (2.7)	17.4	(**4i**, 2.19)	
800	Co_3TeO_6	$C2/c$ (15)<br>$P_S\bar{1}$ (2.7)	17.4	(**4i**, 3.58)<br>(**4i**, 3.58)<br>(**4i**, 2.19)<br>(**4i**, 2.19)<br>(**4i**, 2.19)<br>(**4i**, 2.19)<br>(**4i**, 0.53)<br>(**4i**, 0.53)<br>(**4i**, 0.53)<br>(**4i**, 0.53)<br>(**4i**, 0.94)<br>(**4i**, 0.94)<br>(**4i**, 0.94)<br>(**4i**, 0.94)<br>(**4i**, 2.16)<br>(**4i**, 2.16)<br>(**4i**, 2.16)<br>(**4i**, 2.16)	
801	NdB_4	$P4/mbm$ (127)<br>$P4/m'$ (83.46)	17.2	(**4j**, 2.09)	
802	Sc_2NiMnO_6	$P2_1/c$ (14)<br>$P_S\bar{1}$ (2.7)	17	(**4i**, 1.66)<br>(**4i**, 1.66)<br>(**4i**, 1.49)<br>(**4i**, 1.49)	
803	Sr_3ZnIrO_6	$R\bar{3}c$ (167)<br>P_C2/c (13.74)	17	(**4a**, 0.88)	

Continued on next page

…continued

No.	Compound	SG & MSG	T_c [K]	(WP, $\|m\|/\mu_B$)	Band Gap [eV]
804	$TbNi_2Ge_2$	$I4/mmm$ (139)<br>P_c4/mcc (124.360)	17	(2b,0.0)<br>(**4h**, 6.22)<br>(**2a**, 8.8)	
805	HFe_2Ge_2	$I4/mmm$ (139)<br>C_Amca (64.480)	17	(**4a**, 6.6)	
806	$U_2Pd_{2.35}Sn_{0.65}$	$P4/mbm$ (127)<br>P_c4/mnc (128.408)	17	(**8g**, 0.9)	
807	$Mn_{0.81}Cu_{0.19}WO_4$	$P2/c$ (13)<br>P_a2/c (13.70)	17	(**4f**, 1.78)	
808	$CuSe_2O_5$	$C2/c$ (15)<br>P_c2_1/c (14.84)	17	(**4c**, 0.51)	
809	Mn_2GeO_4	$Pnma$ (62)<br>$Pnma$ (62.441)	17	(**4a**, 4.09)<br>(**4c**, 4.9)	
810	$ErCrO_3$	$Pbnm$ (62)<br>$P2_1/m$ (11.50)	16.8	(**2e**, 5.2)<br>(**2e**, 5.2)<br>(**2b**, 2.90)<br>(**2c**, 2.90)	
811	HoSi	$Cmcm$ (63)<br>C_a2/c (15.91)	16.5	(**16g**, 8.6)	
812	$EuMn_2P_2$	$P\bar{3}m1$ (164)<br>C_c2/m (12.63)	16.5	(**4a**, 6.98)<br>(**8i**, 0.0)	
813	Nd_2NaOsO_6	$P2_1/n$ (14)<br>$P_S\bar{1}$ (2.7)	16.2	(**4i**, 1.61)<br>(**4i**, 1.61)<br>(**2d**, 0.9)<br>(**2c**, 0.9)	
814	$DyGe_{1.3}$	$P6/mmm$ (191)<br>C_cmcm (63.466)	16	(**4a**, 7.2)	
815	$CuFeO_2$	$R\bar{3}m$ (166)<br>C_c2/c (15.90)	16	(**4c**, 1.0)<br>(**8h**, 1.0)<br>(**8h**, 1.0)	
816	$Co_2C_{10}O_8H_2$	$C2/m$ (12)<br>P_C2_1/m (11.57)	16	(**4h**, 3.77)	

Continued on next page

...continued

No.	Compound	SG & MSG	T_c [K]	(WP, $\|m\|/\mu_B$)	Band Gap [eV]
817	HoPt	$Pnma$ (62)<br>$Pnm'a'$ (62.447)	16	(**4c**, 8.20)	
818	ErPt	$Pnma$ (62)<br>$Pnm'a'$ (62.447)	16	(**4c**, 8.09)	
819	PrScSb	$I4/mmm$ (139)<br>P_I4/mnc (128.410)	16	(**4e**, 2.54)	
820	$RbFe_2F_6$	$Pnma$ (62)<br>$Pnma$ (62.441)	16	(**4c**, 3.99)<br>(**4a**, 4.29)	
821	$Mn(N(CN_2))_2$	$Pnnm$ (58)<br>$Pnn'm'$ (58.398)	16	(**2a**, 5.01)	
822	$(CH_3NH_3)(Co(COOH)_3$	$P2_1/n$ (14)<br>$P2_1'/c'$ (14.79)	15.9	(**2c**, 2.94)<br>(**2b**, 2.94)	
823	$(CH_3NH_3)(Co(COOH)_3$	$Pnma$ (62)<br>$Pn'ma'$ (62.448)	15.9	(**4b**, 2.89)	
824	$CoSO_4$	$Cmcm$ (63)<br>P_Cbcn (60.431)	15.5	(**4a**, 3.29)	
825	$CuFe_2(P_2O_7)_2$	$P2_1/n$ (14)<br>P_a2_1/c (14.80)	15.5	(**4a**, 0.83)<br>(**8e**, 4.88)	
826	$CrCl_3$	$R\bar{3}$ (148)<br>$P_S\bar{1}$ (2.7)	15.5	(**4i**, 3.0)	
827	CeCuSi	$P6_3/mmc$ (194)<br>$Cmc'm'$ (63.463)	15.5	(**4a**, 1.25)	
828	Dy_2CoGa_8	$P4/mmm$ (123)<br>I_c4/mcm (140.550)	15.2	(**8g**, 10.5)	
829	CoV_2O_6	$C2/m$ (12)<br>C_c2/c (15.90)	15	(**4c**, 4.77)	
830	$YBa_2Cu_3O_{6+d}$	$P4/mmm$ (123)<br>F_Smmm (69.526)	15	(**8g**, 0.0)<br>(**16r**, 0.47)	
831	PrC_2	$I4/mmm$ (139)<br>P_I4/mnc (128.410)	15	(**2a**, 1.14)	

Continued on next page

...continued

No.	Compound	SG & MSG	T_c [K]	(WP, $\|m\|/\mu_B$)	Band Gap [eV]
832	$Cu_6(SiO_3)_6(H_2O)_6$	$R\bar{3}$ (148)<br>$R_I\bar{3}$ (148.20)	15	(**36f**, 0.64)	
833	U_2Ni_2In	$P4/mbm$ (127)<br>P_c4/mnc (128.408)	15	(**8h**, 0.84)<br>(**8g**, 0.0)	
834	$Li_{0.31}Na_{0.69}FeGe_2O_6$	$P2_1/c$ (14)<br>P_a2_1/c (14.80)	15	(**8e**, 3.69)	
835	Tm_2BaNiO_5	$Immm$ (71)<br>$P_S\bar{1}$ (2.7)	15	(**4i**, 3.33)<br>(**2a**, 1.14)	
836	$BaNd_{0.9}Y_{0.1}MoO_6$	$I4/m$ (87)<br>P_I4/m (83.50)	15	(**2b**, 0.11)<br>(**2a**, 2.9)	
837	$BiCoO(PO_4)$	$P2_1/n$ (14)<br>P_a2_1/c (14.80)	15	(**8e**, 3.27)	
838	$Li_2Ni(SO_4)_2$	$P2_1/c$ (14)<br>$P2_1/c$ (14.75)	15	(**2a**, 2.29)	
839	PrPt	$Cmcm$ (63)<br>$Cm'c'm$ (63.462)	15	(**4c**, 2.2)	
840	$NdMnO_3$	$Pnma$ (62)<br>$Pn'ma'$ (62.448)	15	(**4c**, 1.06)<br>(**4b**, 2.95)	
841	Sr_2CoTeO_6	$P2_1/n$ (14)<br>$P2_1/c$ (14.75)	15	(**2d**, 2.09)	
842	$Cu_4(OD)_6FBr$	$Pnma$ (62)<br>$Pn'm'a$ (62.446)	15	(**4a**, 0.15)<br>(**8d**, 0.40)<br>(**4c**, 0.53)	
843	Tb_2MnNiO_6	$P2_1/c$ (14)<br>$P2_1'/c'$ (14.79)	15	(**4e**, 1.17)<br>(**2c**, 1.80)<br>(**2b**, 2.71)	
844	$NpCo_2$	$Fd\bar{3}m$ (227)<br>$I4_1'/a'm'd$ (141.556)	15	(**16h**, 0.5)	
845	$DyNiAl_4$	$Cmcm$ (63)<br>P_Anma (62.453)	14.6	(**4c**, 8.1)	

Continued on next page

...continued

No.	Compound	SG & MSG	T_c [K]	(WP, $\|\boldsymbol{m}\|/\mu_B$)	Band Gap [eV]
846	Nd_2NaRuO_6	$P2_1/n$ (14)<br>$P2_1/c$ (14.75)	14.3	(**4e**, 2.24)<br>(**2b**, 1.63)	
847	$[C(ND_2)_3]Co(DCOO)_3$	$Pnna$ (52)<br>$Pn'na'$ (52.312)	14.04	(**4d**, 2.97)	
848	Li_2CoSiO_4	$Pna2_1$ (33)<br>C_ac (9.41)	14	(**8a**, 2.92)<br>(**8a**, 2.92)	
849	PrFeAsO	$Cmme$ (67)<br>P_Bcca (54.350)	14	(**4g**, 0.84)<br>(**4b**, 0.48)	
850	$FeSO_4$	$Pbnm$ (62)<br>P_c2_1/c (14.82)	14	(**4c**, 5.44)<br>(**4d**, 5.44)	
851	U_2Ni_2In	$P4/mbm$ (127)<br>P_c4/mnc (128.408)	14.0	(**8h**, 0.91)<br>(**8g**, 0.0)	
852	$CeIrAl_4Si_2$	$P4/mmm$ (123)<br>P_c4/mcc (124.360)	14	(**2a**, 1.41)	
853	$Li_{0.22}Na_{0.78}FeGe_2O_6$	$C2/c$ (15)<br>P_C2_1/c (14.84)	14	(**4e**, 3.63)	
854	$FeBr_2$	$P\bar{3}m1$ (164)<br>$P_c\bar{3}c1$ (165.96)	14	(**2a**, 3.9)	
855	CoV_2O_6-alpha	$C2/m$ (12)<br>C_c2/c (15.90)	14	(**4c**, 4.82)	
856	NdCoAsO	$P4/nmm$ (129)<br>P_anma (62.450)	14	(**4b**, 1.39)<br>(**4a**, 0.32)	
857	PrAg	$Pm\bar{3}m$ (221)<br>P_Bmna (53.334)	14	(**2c**, 2.12)	
858	PrMgPb	$I4/mmm$ (139)<br>P_A2/c (13.73)	14	(**4i**, 1.80)	
859	U_2Ni_2In	$P4/mbm$ (127)<br>P_c4/mnc (128.408)	14	(**8h**, 0.59)<br>(**8g**, 0.37)	

Continued on next page

...continued

No.	Compound	SG & MSG	T_c [K]	(WP, $\|\boldsymbol{m}\|/\mu_B$)	Band Gap [eV]
860	$Cs_2MnU_3F_{16}$	$P6_3/mmc$ (194)<br>$P6_3/mc'm'$ (193.260)	14	(**6g**, 1.97)<br>(**12j**, 1.97)<br>(**2b**, 3.26)<br>(**4d**, 3.26)	
861	$GdFeZnGe_4O_{12}$	$P4/nbm$ (125)<br>P_bnna (52.315)	13.8	(**4c**, 6.02)<br>(**8f**, 3.84)	
862	HoNi	$Pnma$ (62)<br>$P2_1'/c'$ (14.79)	13.5	(**4e**, 9.61)	
863	$Ca_2Sr_2IrO_6$	$R\bar{3}$ (148)<br>$P_S\bar{1}$ (2.7)	13.1	(**2a**, 0.82)<br>(**2e**, 0.78)	
864	$FeTa_2O_6$	$P4_2/mnm$ (136)<br>I_c4_1/a (88.86)	13	(**16c**, 4.00)	
865	$HoCo_2Si_2$	$I4/mmm$ (139)<br>P_I4/mnc (128.410)	13	(**2a**, 8.1)	
866	$Tb_2Ni_3Si_5$	$Ibam$ (72)<br>P_Ibam (55.364)	13	(**8j**, 7.87)	
867	$TbCu_2Si_2$	$I4/mmm$ (139)<br>$P_S\bar{1}$ (2.7)	13	(**2a**, 8.59)	
868	$NaMnF_4$	$P2_1/c$ (14)<br>P_a2_1/c (14.80)	13	(**4a**, 3.85)	
869	$MnBi_4Te_7$	$P\bar{3}m1$ (164)<br>$P_c\bar{3}c1$ (165.96)	13	(**2a**, 4.01)	
870	$Li_2Ni(WO_4)_2$	$P\bar{1}$ (2)<br>$P_S\bar{1}$ (2.7)	13	(**2a**, 1.91)	
871	$NaFePO_4$	$Pnma$ (62)<br>P_c2_1/c (14.82)	13	(**4c**, 3.88)<br>(**4d**, 3.88)	
872	$NdMn_{0.8}Fe_{0.2}O_3$	$Pnma$ (62)<br>$Pn'ma'$ (62.448)	13	(**4c**, 1.64)<br>(**4b**, 3.05)<br>(**4b**, 0.0)	
873	Ho_2CrSbO_7	$Fd\bar{3}m$ (227)<br>$I4_1/am'd'$ (141.557)	13	(**8c**, 2.72)<br>(**8d**, 10.01)	

Continued on next page

... continued

No.	Compound	SG & MSG	T_c [K]	(WP, $\|m\|/\mu_B$)	Band Gap [eV]
874	$Sr_2Co_{0.9}Mg_{0.1}TeO_6$	$P2_1/n$ (14)<br>$P2_1/c$ (14.75)	13	(**2d**, 1.95)	
875	$NdMnO_3$	$Pnma$ (62)<br>$Pn'ma'$ (62.448)	13	(**4c**, 1.2)<br>(**4b**, 3.22)	
876	$CeCoAl_4$	$Pmma$ (51)<br>C_amca (64.479)	12.8	(**8e**, 1.29)	
877	$GdCu_2Si_2$	$I4/mmm$ (139)<br>C_c2/m (12.63)	12.5	(**4a**, 7.2)	
878	$Tb_2Ir_3Ga_9$	$Cmcm$ (63)<br>$Cm'cm'$ (63.464)	12.5	(**8g**, 8.95)<br>(**8e**, 0.0)<br>(**4a**, 0.0)	
879	$Tb_2Ir_3Ga_9$	$Cmcm$ (63)<br>$Cm'cm'$ (63.464)	12.5	(**8g**, 7.5)<br>(**8e**, 0.0)<br>(**4a**, 0.0)	
880	$GeNi_2O_4$	$Fd\bar{3}m$ (227)<br>C_c2/m (12.63)	12.1	(4c,0.0)<br>(**8e**, 1.30)<br>(**4b**, 1.30)	
881	$AgMnVO_4$	$Pnma$ (62)<br>P_a2_1/m (11.55)	12.1	(**4a**, 4.00)<br>(**4c**, 4.00)	
882	Li_2MnSiO_4	$P2_1/n$ (14)<br>P_a2_1/c (14.80)	12	(**8e**, 4.58)	
883	$Ca_3Co_2O_6$	$R\bar{3}c$ (167)<br>P_C2_1/c (14.84)	12	(**4c**, 0.0)<br>(**4e**, 1.0)	
884	$HoCo_2Si_2$	$I4/mmm$ (139)<br>P_I4/mnc (128.410)	12	(**2a**, 9.85)	
885	Ho_3Ge_4	$Cmcm$ (63)<br>P_Bnna (52.318)	12	(**8f**, 5.73)<br>(**4c**, 0.0)	
886	$Cu(NCS)_2$	$P\bar{1}$ (2)<br>$P_S\bar{1}$ (2.7)	12	(**2a**, 0.58)	
887	Ca_4IrO_6	$R\bar{3}c$ (167)<br>P_C2/c (13.74)	12	(**4a**, 0.41)	

Continued on next page

...continued

No.	Compound	SG & MSG	T_c [K]	(WP, $\|m\|/\mu_B$)	Band Gap [eV]
888	$NiTa_2O_6$	$P4_2/mnm$ (136)<br>P_c2_1/c (14.82)	12	(**4e**, 1.93)<br>(**4e**, 1.93)	
889	$CoSO_4$	$Pbnm$ (62)<br>$Pnma$ (62.441)	12	(**4a**, 3.81)	
890	$ErCo_2Si_2$	$I4/mmm$ (139)<br>P_Innm (58.404)	11	(**2a**, 8.7)	
891	$Li_3Co_2SbO_6$	$C2/m$ (12)<br>C_c2/m (12.63)	11	(**8g**, 2.61)	
892	$TbCu_2Si_2$	$I4/mmm$ (139)<br>$P_S\bar{1}$ (2.7)	11	(**2a**, 8.50)	
893	$DyNi_2B_2C$	$I4/mmm$ (139)<br>C_Amca (64.480)	11	(**4a**, 8.47)	
894	$DyCu_2Si_2$	$I4/mmm$ (139)<br>C_c2/m (12.63)	11	(**4a**, 8.3)	
895	$Co_3Al_2Si_3O_{12}$	$Ia\bar{3}d$ (230)<br>$I4_1/a'cd$ (142.563)	11	(**8b**, 3.45)<br>(**16e**, 3.45)	
896	Ce_2PdGe_3	$P4_2/mmc$ (131)<br>$P4_2'/m'm'c$ (131.440)	10.7	(2c,0.0)<br>(**2f**, 1.78)	
897	Nd_2RhIn_8	$P4/mmm$ (123)<br>I_c4/mcm (140.550)	10.63	(**8g**, 2.53)	
898	$NiTa_2O_6$	$P4_2/mnm$ (136)<br>A_bba2 (41.217)	10.55	(**8c**, 1.6)<br>(**8c**, 1.6)	
899	Ho_2RhIn_8	$P4/mmm$ (123)<br>$Cm'cm'$ (63.464)	10.4	(**4c**, 7.5)<br>(**4c**, 7.5)<br>(**4c**, 7.5)<br>(**4c**, 7.5)	
900	$Au_{72}Al_{14}Tb_{14}$	$Im\bar{3}$ (204)<br>$P_In\bar{3}$ (201.21)	10.4	(**24g**, 7.53)	
901	Ho_2RhIn_8	$P4/mmm$ (123)<br>P_cccm (49.273)	10.4	(**4j**, 6.9)	

Continued on next page

. . . continued

No.	Compound	SG & MSG	T_c [K]	(WP, $\|\boldsymbol{m}\|/\mu_B$)	Band Gap [eV]
902	$MnBi_6Te_{10}$	$R\bar{3}m$ (166)<br>$R_I\bar{3}c$ (167.108)	10.3	(**6a**, 3.8)	
903	VPO_4	$Cmcm$ (63)<br>P_cnma (62.452)	10.3	(**8c**, 0.8)	
904	$ErMn_2Si_2$	$I4/mmm$ (139)<br>$Pm'm'n$ (59.409)	10	(**2a**, 8.9)<br>(**4f**, 2.3)	
905	$ErMn_2Si_2$	$I4/mmm$ (139)<br>$Pm'm'n$ (59.409)	10	(**2a**, 7.3)<br>(**4f**, 2.3)	
906	DyOCl	$P4/nmm$ (129)<br>P_anma (62.450)	10	(**4b**, 10.1)	
907	$TbNi_2Si_2$	$I4/mmm$ (139)<br>C_Amca (64.480)	10	(**4a**, 8.8)	
908	$NdScSiC_{0.5}H_{0.2}$	$I4/mmm$ (139)<br>P_I4/nnc (126.386)	10	(**4e**, 2.73)	
909	$HoCu_2Si_2$	$I4/mmm$ (139)<br>$P_S\bar{1}$ (2.7)	10	(**2a**, 8.81)	
910	$HoGe_{1.5}$	$P6/mmm$ (191)<br>C_cmcm (63.466)	10	(**4a**, 6.2)	
911	$CuFeO_2$	$R\bar{3}m$ (166)<br>C_a2/c (15.91)	10	(**8f**, 4.0)	
912	$EuAs_3$	$C2/m$ (12)<br>C_c2/m (12.63)	10	(**8i**, 5.74)	
913	$BiMnTeO_6$	$P2_1/c$ (14)<br>P_a2_1/c (14.80)	10	(**8e**, 3.64)	
914	$CeAu_2Si_2$	$I4/mmm$ (139)<br>P_I4/mnc (128.410)	10	(**2a**, 1.29)	
915	$CsMn_2F_6$	$Pnma$ (62)<br>$P2_1/c$ (14.75)	10	(**2b**, 3.40)<br>(**2c**, 3.52)<br>(**4e**, 4.12)	
916	$CoAl_2O_4$	$Fd\bar{3}m$ (227)<br>$I4_1'/a'm'd$ (141.556)	10	(**4a**, 1.9)	

Continued on next page

...continued

No.	Compound	SG & MSG	T_c [K]	(WP, $\|\boldsymbol{m}\|/\mu_B$)	Band Gap [eV]
917	$SmCrO_3$	$Pnma$ (62)<br>$Pnma$ (62.441)	10	(**4c**, 0.7)<br>(**4b**, 2.40)	
918	$EuCd_2As_2$	$P\bar{3}m1$ (164)<br>C_c2/m (12.63)	9.5	(**4a**, 1.0)	
919	$Ba(TiO)Cu_4(PO_4)_4$	$P42_12$ (90)<br>$P_c4_22_12$ (94.132)	9.5	(**16g**, 0.79)	
920	FeI_2	$P\bar{3}m1$ (164)<br>$P_S\bar{1}$ (2.7)	9.3	(**4i**, 3.7)	
921	FeI_2	$P\bar{3}m1$ (164)<br>C_c2/c (15.90)	9.3	(**8i**, 3.7)	
922	$TbCuSb_2$	$P4/nmm$ (129)<br>P_a2_1/m (11.55)	9	(**4e**, 6.27)<br>(**4e**, 6.27)	
923	$CeRhAl_4Si_2$	$P4/mmm$ (123)<br>P_c4/mcc (124.360)	9	(**2a**, 1.14)	
924	$NdNiMg_{15}$	$P4/nmm$ (129)<br>P_Bcca (54.350)	9	(**4g**, 2.7)	
925	$NaFeSi_2O_6$	$C2/c$ (15)<br>P_C2_1/c (14.84)	9	(**4e**, 1.82)	
926	Ce_3NIn	$Pm\bar{3}m$ (221)<br>$P_C\bar{4}b2$ (117.305)	9	(**2d**, 1.75)<br>(**4f**, 1.75)	
927	$Li_2FeP_2O_7$	$P2_1/c$ (14)<br>$P2_1/c$ (14.75)	9	(**4e**, 3.54)<br>(**4e**, 3.94)<br>(**4e**, 3.54)	
928	$Ni_3B_7O_{13}Cl$	$Pca2_1$ (29)<br>$Pc'a2_1'$ (29.101)	9	(**4a**, 1.65)<br>(**4a**, 0.79)<br>(**4a**, 0.79)	
929	$[C(ND_2)_3]Mn(DCOO)_3$	$Pnna$ (52)<br>$Pn'n'a$ (52.310)	8.73	(**4d**, 5.04)	
930	$CuSb_2O_6$	$P2_1/n$ (14)<br>P_a2_1/c (14.80)	8.7	(**4a**, 0.51)	

Continued on next page

…continued

No.	Compound	SG & MSG	T_c [K]	(WP, $\|\boldsymbol{m}\|/\mu_B$)	Band Gap [eV]
931	$FeTa_2O_6$	$P4_2/mnm$ (136)<br>I_c4_1/a (88.86)	8.5	(**16c**, 3.69)	
932	$CePd_2Si_2$	$I4/mmm$ (139)<br>C_Accm (66.500)	8.5	(**4b**, 0.66)	
933	Dy_2O_2Se	$P\bar{3}m1$ (164)<br>C_c2/c (15.90)	8.5	(**8i**, 9.0)	
934	Li_2MnTeO_6	$P\bar{3}1c$ (163)<br>$P\bar{3}c1$ (165.91)	8.5	(**6f**, 1.52)	
935	$TbFeO_3$	$Pbnm$ (62)<br>$Pn'm'a$ (62.446)	8.4	(**4b**, 4.8)<br>(**4c**, 3.06)	
936	Li_2CuO_2	$Immm$ (71)<br>P_Innm (58.404)	8.3	(**2c**, 0.96)	
937	$Na_3Co_2SbO_6$	$C2/m$ (12)<br>$P_S\bar{1}$ (2.7)	8.3	(**4i**, 1.79)	
938	$ErMn_2Ge_2$	$I4/mmm$ (139)<br>$Pm'm'n$ (59.409)	8	(**2a**, 7.7)<br>(**4f**, 2.3)	
939	$MnWO_4$	$P2/c$ (13)<br>C_a2/c (15.91)	8	(**16g**, 4.5)	
940	Li_2MnGeO_4	$Pmn2_1$ (31)<br>C_ac (9.41)	8	(**8a**, 4.89)	
941	$HoCo_2Ge_2$	$I4/mmm$ (139)<br>P_I4/mnc (128.410)	8	(4d,0.0)<br>(**2a**, 8.64)	
942	Nd_5Pb_3	$P6_3/mcm$ (193)<br>P_Bnma (62.454)	8	(**8e**, 3.2)<br>(**8g**, 1.1)<br>(**4c**, 1.8)	
943	$HoCu_2Si_2$	$I4/mmm$ (139)<br>C_c2/m (12.63)	8	(**4a**, 6.5)	
944	$NaFeSi_2O_6$	$C2/c$ (15)<br>P_C2_1/c (14.84)	8	(**4e**, 2.65)	
945	$HoCr(BO_3)_2$	$R\bar{3}$ (148)<br>$P_S\bar{1}$ (2.7)	8	(**2a**, 3.92)	

Continued on next page

…continued

No.	Compound	SG & MSG	T_c [K]	(WP, $\|m\|/\mu_B$)	Band Gap [eV]
946	$YCr(BO_3)_2$	$R\bar{3}$ (148)<br>$P_S\bar{1}$ (2.7)	8	(**2a**, 3.25)	
947	$Tb_{0.55}Sr_{0.45}MnO_3$	$Pnma$ (62)<br>$Pnm'a'$ (62.447)	8	(**4c**, 1.48)<br>(**4b**, 0.72)	
948	$ZrMn_2Ge_4O_{12}$	$P4/nbm$ (125)<br>$P4'/nbm'$ (125.367)	8	(**4f**, 4.68)	
949	Er_3Ge_4	$Cmcm$ (63)<br>P_Cbcm (57.391)	7.3	(**8f**, 7.32)<br>(**4c**, 6.37)	
950	$DyGa_3$	$R\bar{3}m$ (166)<br>C_c2/c (15.90)	7.2	(**8i**, 7.37)<br>(**8i**, 7.37)	
951	$Ce_3Ni_2Ge_7$	$Cmmm$ (65)<br>P_Cmmn (59.415)	7.2	(2d,0.0)<br>(**4i**, 1.98)	
952	$Dy_3Ru_4Al_{12}$	$P6_3/mmc$ (194)<br>$P\bar{3}c'1$ (165.95)	7	(**12g**, 8.93)<br>(**6f**, 8.93)	
953	Tb_2O_2Se	$P\bar{3}m1$ (164)<br>C_c2/c (15.90)	7	(**8i**, 6.49)	
954	$Dy_3Ru_4Al_{12}$	$P6_3/mmc$ (194)<br>C_c2/c (15.90)	7	(**16j**, 6.90)<br>(**8i**, 6.90)	
955	$NaMnGe_2O_6$	$C2/c$ (15)<br>$P_S\bar{1}$ (2.7)	7	(**4i**, 2.05)	
956	$RuCl_3$	$C2/m$ (12)<br>P_C2/m (10.49)	7	(**8j**, 0.52)<br>(**4h**, 0.0)	
957	CeMgPb	$I4/mmm$ (139)<br>C_Amma (67.510)	7	(**8h**, 1.38)	
958	$Dy_3Ru_4Al_{12}$	$P6_3/mmc$ (194)<br>C_c2/c (15.90)	7	(**16j**, 0.53)<br>(**8i**, 0.70)	
959	TmNi	$Pnma$ (62)<br>$Pn'm'a$ (62.446)	7	(**4c**, 0.0)<br>(**4c**, 5.89)	
960	$Na_2BaFe(VO_4)_2$	$C2/c$ (15)<br>$C2'/c'$ (15.89)	7	(**4b**, 4.73)	

Continued on next page

...continued

No.	Compound	SG & MSG	T_c [K]	(WP, $\|\boldsymbol{m}\|/\mu_B$)	Band Gap [eV]
961	$Pr_3Ru_4Al_{12}$	$P6_3/mmc$ (194)<br>$C2'/c'$ (15.89)	7	(**4e**, 3.2)<br>(**8f**, 1.47)	
962	$Li_2Co(SO_4)_2$	$P2_1/c$ (14)<br>$P2_1'/c'$ (14.79)	7	(**2a**, 3.33)	
963	$EuMg_2Bi_2$	$P\bar{3}m1$ (164)<br>C_c2/m (12.63)	6.72	(**4a**, 6.61)	
964	$EuMg_2Bi_2$	$P\bar{3}m1$ (164)<br>C_c2/m (12.63)	6.7	(**4a**, 5.3)	
965	$Cu_2(OD)_3Cl$	$P2_1/c$ (14)<br>$P2_1/c$ (14.75)	6.7	(**2b**, 0.61)<br>(**2a**, 0.61)<br>(**4e**, 0.61)	
966	Ho_3Ge_4	$Cmcm$ (63)<br>P_c2_1/c (14.82)	6.6	(**8f**, 8.9)<br>(**4e**, 7.2)	
967	$Mn_5(PO_4)_2(PO_3(OH))_2(HOH)_4$	$C2/c$ (15)<br>$C2'/c'$ (15.89)	6.17	(**4e**, 3.74)<br>(**8f**, 3.39)<br>(**8f**, 3.50)	
968	$Mn_5(PO_4)_2(PO_3(OH))_2(HOH)_4$	$C2/c$ (15)<br>$C2'/c'$ (15.89)	6.17	(**4e**, 3.05)<br>(**8f**, 4.08)<br>(**8f**, 3.81)	
969	$Mn_5(PO_4)_2(PO_3(OH))_2(HOH)_4$	$C2/c$ (15)<br>$C2'/c'$ (15.89)	6.17	(**4e**, 2.69)<br>(**8f**, 2.85)<br>(**8f**, 3.13)	
970	$MnPb_4Sb_6S_{14}$	$P2_1/c$ (14)<br>P_a2_1/c (14.80)	6	(**4a**, 3.2)	
971	$ErCo_2Si_2$	$I4/mmm$ (139)<br>P_A2_1/c (14.83)	6	(**2a**, 6.74)	
972	Tb_2O_2S	$P\bar{3}m1$ (164)<br>C_c2/c (15.90)	6	(**8i**, 7.21)	
973	Dy_3Ge_4	$Cmcm$ (63)<br>P_a2_1/m (11.55)	6	(**8f**, 7.36)<br>(**4e**, 5.7)	

Continued on next page

...continued

No.	Compound	SG & MSG	T_c [K]	(WP, $\|\boldsymbol{m}\|/\mu_B$)	Band Gap [eV]
974	$Dy_2Fe_2Si_2C$	$C2/m$ (12)<br>$P_S\bar{1}$ (2.7)	6	(**4i**, 10.95)<br>(**4i**, 0.0)	
975	Er_2Ni_2In	$Cmmm$ (65)<br>C_amcm (63.467)	6	(**16j**, 7.71)<br>(**16i**, 0.0)	
976	$NH_4FeCl_2(HCOO)$	$C2/c$ (15)<br>P_C2_1/c (14.84)	6	(**4c**, 3.40)	
977	TmPt	$Pnma$ (62)<br>$Pnm'a'$ (62.447)	6	(**4c**, 4.49)	
978	$Er_3Cu_4O_7$	$C2/m$ (12)<br>C_c2/m (12.63)	6	(**4a**, 8.86)<br>(**8i**, 0.0)	
979	$NdNiGe_2$	$Cmcm$ (63)<br>$Cm'c'm$ (63.462)	6	(**4c**, 2.32)	
980	$Cu_4(OH)_6FBr$	$P63/m$ (176)<br>$P2_1'/m'$ (11.54)	6	(**4f**, 0.14)<br>(**4f**, 0.22)<br>(**4f**, 0.17)<br>(**4f**, 0.27)<br>(**4f**, 0.29)<br>(**4f**, 0.19)<br>(**2e**, 0.18)<br>(**2e**, 0.18)<br>(**2e**, 0.18)<br>(**2e**, 0.16)<br>(**2e**, 0.10)<br>(**2e**, 0.16)<br>(**2e**, 0.16)<br>(**2e**, 0.10)<br>(**2e**, 0.16)<br>(**2e**, 0.16)<br>(**2e**, 0.10)<br>(**2e**, 0.16)	
981	$NaCrGe_2O_6$	$C2/c$ (15)<br>$C2'/c'$ (15.89)	6	(**4e**, 1.85)	

Continued on next page

. . . continued

No.	Compound	SG & MSG	T_c [K]	(WP, $\|\boldsymbol{m}\|/\mu_B$)	Band Gap [eV]
982	$Cu_2(OD)_3Cl$	$P2_1/c$ (14)<br>$P2_1/c$ (14.75)	6	(**4e**, 0.58)<br>(**2b**, 0.57)<br>(**2a**, 0.57)	
983	$CeMnCoGe_4O_{12}$	$P4/nbm$ (125)<br>$Pb'an'$ (50.282)	6	(**4f**, 3.13)<br>(**4f**, 3.13)	
984	$Li_2Mn(SO_4)_2$	$P2_1/c$ (14)<br>$P2_1/c$ (14.75)	6	(**2a**, 4.10)	
985	$CeNiGe_3$	$Cmmm$ (65)<br>P_Cmmn (59.415)	5.9	(**4h**, 0.22)	
986	Dy_2O_2S	$P\bar{3}m1$ (164)<br>C_c2/c (15.90)	5.8	(**8i**, 7.2)	
987	$ErMn_2Ge_2$	$I4/mmm$ (139)<br>$Pm'm'n$ (59.409)	5.5	(**2a**, 6.81)<br>(**4f**, 2.34)	
988	HoP	$Fm\bar{3}m$ (225)<br>$C2'/c'$ (15.89)	5.5	(**4d**, 8.8)	
989	Mn_2GeO_4	$Pnma$ (62)<br>$P2_1/c$ (14.75)	5.5	(**2a**, 3.39)<br>(**2b**, 2.95)<br>(**4e**, 4.46)	
990	$Cu_3Mg(OD)_6Br_2$	$P\bar{3}m1$ (164)<br>C_c2/m (12.63)	5.4	(**8e**, 0.83)<br>(**4b**, 0.83)	
991	$Co_4(OH)_2(C_{10}H_{16}O_4)_3$	$P2_1/c$ (14)<br>$P2_1'/c'$ (14.79)	5.4	(**4e**, 2.19)<br>(**4e**, 2.68)	
992	$HoNi_2B_2C$	$I4/mmm$ (139)<br>C_Amca (64.480)	5.2	(**4a**, 9.16)	
993	$HoNi_2B_2C$	$I4/mmm$ (139)<br>C_Amca (64.480)	5.2	(**4a**, 9.16)<br>(**8f**, 0.0)	
994	$KMnF_4$	$P2_1/a$ (14)<br>$P2_1'/c'$ (14.79)	5.2	(**2a**, 2.84)<br>(**2c**, 2.84)	
995	PrCuSi	$P6_3/mmc$ (194)<br>P_Cbcn (60.431)	5.1	(**4a**, 1.14)	

Continued on next page

…continued

No.	Compound	SG & MSG	T_c [K]	(WP, $\|\boldsymbol{m}\|/\mu_B$)	Band Gap [eV]
996	$CePd_2Ge_2$	$I4/mmm$ (139)<br>C_Accm (66.500)	5.1	(**4b**, 1.30)	
997	$FePb_4Sb_6S_{14}$	$P2_1/c$ (14)<br>P_a2_1/c (14.80)	5	(**4a**, 3.21)	
998	$TbCuSb_2$	$P4/nmm$ (129)<br>$P_S\bar{1}$ (2.7)	5	(**4i**, 7.79)	
999	Pr_2Pd_2In	$P4/mbm$ (127)<br>P_bnma (62.451)	5	(**8g**, 1.8)	
1000	$BaCo_2V_2O_8$	$I4_1/acd$ (142)<br>P_Icca (54.352)	5	(**16f**, 2.26)	
1001	$HoNi_2B_2C$	$I4/mmm$ (139)<br>C_Amca (64.480)	5	(**4a**, 8.61)	
1002	$C_8H_{10}Co_2O_{11}$	$P\bar{1}$ (2)<br>$P_S\bar{1}$ (2.7)	5	(**4i**, 3.33)<br>(**2e**, 4.41)<br>(**2c**, 3.53)	
1003	$YbMnO_3$	$P6_3cm$ (185)<br>$P6_3c'm'$ (185.201)	5	(**2a**, 1.47)<br>(**4b**, 1.76)<br>(**6c**, 3.41)	
1004	Ce_4Ge_3	$I\bar{4}3d$ (220)<br>$I\bar{4}2d$ (122.333)	5	(**16e**, 1.55)	
1005	$Ba_3Nb_2NiO_9$	$P\bar{3}m1$ (164)<br>P_c31c (159.64)	4.9	(**6c**, 1.90)	
1006	$NdNi_2B_2C$	$I4/mmm$ (139)<br>C_c2/c (15.90)	4.87	(**4c**, 2.1)	
1007	$Cs_2NiU_3F_{16}$	$P6_3/mmc$ (194)<br>$P6_3/mc'm'$ (193.260)	4.8	(**6g**, 0.94)<br>(**12j**, 0.94)<br>(**2b**, 1.53)<br>(**4d**, 1.53)	
1008	$Ce_2Ni_3Ge_5$	$Ibam$ (72)<br>P_Iccn (56.376)	4.8	(**8j**, 0.4)	

Continued on next page

. . . continued

No.	Compound	SG & MSG	T_c [K]	(WP, $\|m\|/\mu_B$)	Band Gap [eV]
1009	$Ce_2Ni_3Ge_5$	$Ibam$ (72)<br>P_Ibcn (60.432)	4.8	(**8j**, 0.4)	
1010	MnV_2O_6	$Pbcn$ (60)<br>P_a2_1/c (14.80)	4.7	(**8e**, 3.74)	
1011	$CsFeCl_3$	$P6_3/mmc$ (194)<br>$P\bar{6}'2'm$ (189.223)	4.7	(**6i**, 3.16)	
1012	$CsFe(MoO_4)_2$	$P\bar{3}$ (147)<br>P_c3 (143.3)	4.5	(**6d**, 5.17)	
1013	$CeCo_2Ge_4O_{12}$	$P4/nbm$ (125)<br>P_bnna (52.315)	4.5	(**8f**, 2.58)	
1014	$Ce_2Ni_3Ge_5$	$Ibam$ (72)<br>P_Ibcn (60.432)	4.3	(**8j**, 0.47)	
1015	TmAgGe	$P\bar{6}2m$ (189)<br>$P\bar{6}'2m'$ (189.224)	4.2	(**3g**, 6.44)<br>(**3g**, 6.44)<br>(**6k**, 9.10)	
1016	$TlMnF_4$	$I2/a$ (15)<br>P_C2/c (13.74)	4.2	(**4b**, 3.1)	
1017	$NdCu_2$	$Imma$ (74)<br>P_Inma (62.456)	4.1	(**4e**, 1.78)<br>(**8i**, 1.78)<br>(**8i**, 1.78)	
1018	$PrNi_2B_2C$	$I4/mmm$ (139)<br>C_Amca (64.480)	4	(**4a**, 0.80)	
1019	Ho_2O_2Se	$P\bar{3}m1$ (164)<br>P_A2/c (13.73)	4	(**4i**, 9.3)	
1020	$Li_2Fe(SO_4)_2$	$P2_1/c$ (14)<br>P_a2_1/c (14.80)	4	(**4a**, 3.22)	
1021	Na_2NiCrF_7	$Imma$ (74)<br>$Im'm'a$ (74.558)	4	(**4c**, 1.75)<br>(**4b**, 2.22)	
1022	Na_2NiCrF_7	$Imma$ (74)<br>$Im'm'a$ (74.558)	4	(**4c**, 1.64)<br>(**4b**, 1.84)	

Continued on next page

…continued

No.	Compound	SG & MSG	T_c [K]	(WP, $\|m\|/\mu_B$)	Band Gap [eV]
1023	$CuCl(C_4H_4N_2)_2(BF_4)$	$P4/nbm$ (125)<br>P_bnna (52.315)	3.9	(**4b**, 0.76)	
1024	$Li_2CuW_2O_8$	$P\bar{1}$ (2)<br>$P_S\bar{1}$ (2.7)	3.9	(**2d**, 0.65)	
1025	$CuBr(C_4H_4N_2)_2(BF_4)$	$P4/nbm$ (125)<br>P_bnna (52.315)	3.8	(**4b**, 0.6)	
1026	$Ce_3Ni_2Sn_7$	$Cmmm$ (65)<br>P_Cmmn (59.415)	3.8	(2c,0.0)<br>(**4i**, 1.9)	
1027	$Ba_3CoSb_2O_9$	$P6_3/mmc$ (194)<br>$P\bar{6}2m$ (189.221)	3.8	(**6i**, 1.41)	
1028	KTb_3F_{12}	$I4/m$ (87)<br>P_I4_2/m (84.58)	3.6	(2b,0.0)<br>(**4d**, 6.95)	
1029	TmPtIn	$P\bar{6}2m$ (189)<br>A_bbm2 (39.201)	3.5	(**8e**, 5.59)<br>(**4a**, 7.90)<br>(**8e**, 5.59)<br>(**4c**, 7.90)	
1030	$Fe_4Si_2Sn_7O_{16}$	$P\bar{3}m1$ (164)<br>C_c2/m (12.63)	3.5	(4d,0.0)<br>(**8e**, 2.74)	
1031	$Cs_2CoU_3F_{16}$	$P6_3/mmc$ (194)<br>$P6_3/mc'm'$ (193.260)	3.5	(**6g**, 0.91)<br>(**12j**, 0.91)<br>(**2b**, 1.7)<br>(**4d**, 1.7)	
1032	$ZrCo_2Ge_4O_{12}$	$P4/nbm$ (125)<br>$Pb'an'$ (50.282)	3.5	(**4f**, 2.55)	
1033	$CsCoCl_3(D_2O)_2$	$Pcca$ (54)<br>P_bccn (56.372)	3.4	(**8c**, 2.5)	
1034	$Ba_3MnNb_2O_9$	$P\bar{3}m1$ (164)<br>$P31m$ (157.53)	3.4	(**3c**, 6.94)	
1035	ErRh	$Pm\bar{3}m$ (221)<br>P_amma (51.298)	3.3	(**2b**, 6.1)	

Continued on next page

. . . continued

No.	Compound	SG & MSG	T_c [K]	(WP, $\|\boldsymbol{m}\|/\mu_B$)	Band Gap [eV]
1036	HoRh	$Pm\bar{3}m$ (221)<br>$P_Ia\bar{3}$ (205.36)	3.2	(**8b**, 5.19)	
1037	HoRh	$Pm\bar{3}m$ (221)<br>P_C2_1/m (11.57)	3.2	(**4e**, 5.19)	
1038	$NaCeO_2$	$I4_1/amd$ (141)<br>$I4_1'/a'm'd$ (141.556)	3.18	(**4a**, 0.57)	
1039	$ErFe_6Ge_6$	$Immm$ (71)<br>$Pm'm'n$ (59.409)	3	(**2b**, 7.5)<br>(**4e**, 7.5)<br>(**2b**, 7.5)<br>(**4e**, 7.5)<br>(**2a**, 2.0)<br>(**2a**, 2.0)<br>(**4c**, 2.0)<br>(**4d**, 2.0)<br>(**4e**, 2.0)<br>(**4e**, 2.0)<br>(**8g**, 2.0)<br>(**8g**, 2.0)	
1040	$CeIr(In_{0.97}Cd_{0.03})_5$	$P4/mmm$ (123)<br>I_c4/mcm (140.550)	3	(**4a**, 0.47)	
1041	Yb_2O_2S	$P\bar{3}m1$ (164)<br>C_c2/m (12.63)	3	(**8i**, 2.0)	
1042	$Yb_2Pd_2(In_{0.4}Sn_{0.6})$	$P4/mbm$ (127)<br>P_c4/mbm (127.396)	3	(**8h**, 0.80)	
1043	Er_2CoGa_8	$P4/mmm$ (123)<br>P_amma (51.298)	3	(**4m**, 4.71)	
1044	$K_2Mn_3(VO_4)_2CO_3$	$P6_3/m$ (176)<br>$P6_3'/m$ (176.145)	3	(**12i**, 5.0)<br>(**2a**, 3.7)<br>(**2c**, 1.85)<br>(**2d**, 1.85)	
1045	$Ba_2MnSi_2O_7$	$P\bar{4}2_1m$ (113)<br>$P\bar{4}2_1m$ (113.267)	2.95	(**2a**, 4.1)	

Continued on next page

...continued

No.	Compound	SG & MSG	T_c [K]	(WP, $\|\boldsymbol{m}\|/\mu_B$)	Band Gap [eV]
1046	ErNiGe	$Pnma$ (62)<br>P_a2_1/c (14.80)	2.9	(**8e**, 9.04)	
1047	Li_2VOSiO_4	$P4/nmm$ (129)<br>P_Abcm (57.389)	2.8	(**4g**, 0.62)	
1048	TmPdIn	$P\bar{6}2m$ (189)<br>$P_c\bar{6}$ (174.136)	2.7	(**6k**, 0.0)<br>(**6k**, 6.15)<br>(**6k**, 8.69)	
1049	$CsNiF_3$	$P6_3/mmc$ (194)<br>P_Bnnm (58.402)	2.67	(**4a**, 2.25)	
1050	$ErFe_2Si_2$	$I4/mmm$ (139)<br>P_anma (62.450)	2.6	(**4b**, 7.4)	
1051	$Er_2Fe_2Si_2C$	$C2/m$ (12)<br>$P_S\bar{1}$ (2.7)	2.6	(**4i**, 11.16)	
1052	$RbFeCl_3$	$P6_3/mmc$ (194)<br>$P\bar{6}'2m'$ (189.224)	2.55	(**6i**, 1.6)	
1053	$NiSb_2O_6$	$P4_2/mnm$ (136)<br>$P_S\bar{1}$ (2.7)	2.5	(**2a**, 1.55)<br>(**2e**, 1.55)	
1054	$GdVO_4$	$I4_1/amd$ (141)<br>$I4_1'/a'm'd$ (141.556)	2.5	(**4a**, 7.0)	
1055	$Dy_3Al_5O_{12}$	$Ia\bar{3}d$ (230)<br>$Ia\bar{3}d'$ (230.148)	2.49	(**24c**, 9.0)	
1056	CeB_6	$Pm\bar{3}m$ (221)<br>C_amca (64.479)	2.4	(**16k**, 0.28)<br>(**16k**, 0.28)	
1057	CeSbTe	$P4/nmm$ (129)<br>P_c4/ncc (130.432)	2.3	(**4c**, 0.38)	
1058	$BaMnO_3$	$P6_3/mmc$ (194)<br>$P6_3'/m'cm'$ (193.259)	2.3	(**2b**, 3.0)<br>(**4d**, 3.0)	
1059	$SrNd_2O_4$	$Pnam$ (62)<br>P_a2_1/c (14.80)	2.28	(**8e**, 0.29)<br>(**8e**, 1.95)	
1060	$TbPO_4$	$I4_1/amd$ (141)<br>$I4_1'/a'm'd$ (141.556)	2.28	(**4a**, 1.0)	

Continued on next page

...continued

No.	Compound	SG & MSG	T_c [K]	(WP, $\|m\|/\mu_B$)	Band Gap [eV]
1061	$DyCrO_3$	$Pbnm$ (62)<br>$P2_1'/m'$ (11.54)	2.2	(**2a**, 2.7)<br>(**2c**, 2.7)<br>(**2d**, 2.7)<br>(**2b**, 2.7)<br>(**2e**, 8.59)<br>(**2e**, 8.59)<br>(**2e**, 8.59)<br>(**2e**, 8.59)	
1062	NdBiPt	$F\bar{4}3m$ (216)<br>$P_I\bar{4}n2$ (118.314)	2.18	(**2d**, 1.78)	
1063	$MnBr_2$	$P\bar{3}m1$ (164)<br>C_c2/m (12.63)	2.17	(**8i**, 1.0)	
1064	$DyCrO_3$	$Pbnm$ (62)<br>$P2_1'/m'$ (11.54)	2.16	(**2a**, 2.76)<br>(**2c**, 2.76)<br>(**2d**, 2.76)<br>(**2b**, 2.76)<br>(**2e**, 9.68)<br>(**2e**, 9.68)<br>(**2e**, 9.68)<br>(**2e**, 9.68)	
1065	Tm_2CoGa_8	$P4/mmm$ (123)<br>C_ammm (65.489)	2	(**8o**, 2.35)	
1066	NdFeAsO	$Cmme$ (67)<br>P_A2/c (13.73)	1.96	(**4i**, 1.55)<br>(**4h**, 0.9)	
1067	$CdYb_2S_4$	$Fd\bar{3}m$ (227)<br>$I4_1/amd$ (141.551)	1.92	(**8d**, 0.77)	
1068	$BaNd_2ZnO_5$	$I4/mcm$ (140)<br>P_C4/ncc (130.433)	1.80	(**16m**, 1.89)	
1069	$CdYb_2Se_4$	$Fd\bar{3}m$ (227)<br>$I4_1/amd$ (141.551)	1.75	(**8d**, 0.62)	
1070	$BaNd_2O_4$	$Pnam$ (62)<br>P_a2_1/c (14.80)	1.7	(**8e**, 0.0)<br>(**8e**, 2.64)	

Continued on next page

...continued

No.	Compound	SG & MSG	T_c [K]	(WP, $\|\boldsymbol{m}\|/\mu_B$)	Band Gap [eV]
1071	$BaNd_2O_4$	$Pnam$ (62)<br>P_a2_1/c (14.80)	1.7	(**8e**, 2.64)<br>(**8e**, 0.0)	
1072	$Ho_2Ge_2O_7$	$P4_12_12$ (92)<br>$P4_12_12$ (92.111)	1.6	(**8b**, 9.05)	
1073	$Na_3Co(CO_3)_2Cl$	$Fd\bar{3}$ (203)<br>$Fd\bar{3}$ (203.26)	1.5	(**16c**, 1.73)	
1074	$Ho_2Ru_2O_7$	$Fd\bar{3}m$ (227)<br>$I4_1/am'd'$ (141.557)	1.4	(**8d**, 6.29)<br>(**8c**, 1.79)	
1075	$Tb_3Al_5O_{12}$	$Ia\bar{3}d$ (230)<br>$Ia\bar{3}d'$ (230.148)	1.35	(**24c**, 5.7)	
1076	$NdPd_5Al_2$	$I4/mmm$ (139)<br>P_anma (62.450)	1.3	(**4a**, 2.7)	
1077	$Na_2BaMn(VO_4)_2$	$C2/c$ (15)<br>P_C2_1/c (14.84)	1.3	(**4d**, 2.04)	
1078	$NdCoO_3$	$Pbnm$ (62)<br>$Pnma$ (62.441)	1.20	(**4c**, 1.39)	
1079	$Er_2Ti_2O_7$	$Fd\bar{3}m$ (227)<br>$I4_1'/am'd$ (141.554)	1.173	(**8c**, 3.24)	
1080	$Gd_2Sn_2O_7$	$Fd\bar{3}m$ (227)<br>$I4_1'/amd'$ (141.555)	1	(**8d**, 6.08)	
1081	$Gd_2Ti_2O_7$	$Fd\bar{3}m$ (227)<br>$R_I\bar{3}m$ (166.102)	0.97	(6b,0.0)<br>(**18e**, 6.73)	
1082	$Nd_2Sn_2O_7$	$Fd\bar{3}m$ (227)<br>$Fd\bar{3}m'$ (227.131)	0.91	(**16d**, 1.70)	
1083	$YbCo_2Si_2$	$I4/mmm$ (139)<br>I_cbca (73.553)	0.9	(**8g**, 1.41)	
1084	$Tb_2Sn_2O_7$	$Fd\bar{3}m$ (227)<br>$I4_1/am'd'$ (141.557)	0.87	(**8d**, 5.87)	
1085	$Ho_3Al_5O_{12}$	$Ia\bar{3}d$ (230)<br>$Ia\bar{3}d'$ (230.148)	0.85	(**24c**, 5.2)	

Continued on next page

. . . continued

No.	Compound	SG & MSG	T_c [K]	(WP, $\|\boldsymbol{m}\|/\mu_B$)	Band Gap [eV]
1086	$GdPO_4$	$P2_1/n$ (14)<br>P_a2_1/c (14.80)	0.8	(**8e**, 7.61)	
1087	$Er_3Ga_5O_{12}$	$Ia\bar{3}d$ (230)<br>$Ia\bar{3}d'$ (230.148)	0.8	(**24c**, 5.24)	
1088	$Gd_2Ti_2O_7$	$Fd\bar{3}m$ (227)<br>$F_S\bar{4}3m$ (216.77)	0.7	(**96i**, 7.00)<br>(32e,0.0)	
1089	Nd_2O_3	$P\bar{3}m1$ (164)<br>C_c2/m (12.63)	0.55	(**8i**, 1.87)	
1090	$Nd_2Hf_2O_7$	$Fd\bar{3}m$ (227)<br>$Fd\bar{3}m'$ (227.131)	0.55	(**16d**, 0.62)	
1091	$KCeS_2$	$R\bar{3}m$ (166)<br>C_c2/c (15.90)	0.4	(**4c**, 0.41)	
1092	$Nd_2Zr_2O_7$	$Fd\bar{3}m$ (227)<br>$Fd\bar{3}m'$ (227.131)	0.4	(**16d**, 1.26)	
1093	$Er_2Pt_2O_7$	$Fd\bar{3}m$ (227)<br>$I4_1'/amd'$ (141.555)	0.38	(**8d**, 4.80)	
1094	Nd_2ScNbO_7	$Fd\bar{3}m$ (227)<br>$Fd\bar{3}m'$ (227.131)	0.371	(**16d**, 1.12)	
1095	$Dy_3Ga_5O_{12}$	$Ia\bar{3}d$ (230)<br>$Ia\bar{3}d'$ (230.148)	0.37	(**24c**, 3.09)	
1096	$Sm_2Ti_2O_7$	$Fd\bar{3}m$ (227)<br>$Fd\bar{3}m'$ (227.131)	0.35	(**16d**, 0.44)	
1097	$Yb_2Ti_2O_7$	$Fd\bar{3}m$ (227)<br>$I4_1/am'd'$ (141.557)	0.26	(**8d**, 0.89)	
1098	$Tb_3Ga_5O_{12}$	$Ia\bar{3}d$ (230)<br>$Ia\bar{3}d'$ (230.148)	0.25	(**24c**, 2.16)	
1099	$Tb_3Ga_5O_{12}$	$Ia\bar{3}d$ (230)<br>$Ia\bar{3}d'$ (230.148)	0.24	(**24c**, 4.0)	
1100	$KErSe_2$	$R\bar{3}m$ (166)<br>C_c2/m (12.63)	0.2	(**4a**, 3.06)	

Continued on next page

...continued

No.	Compound	SG & MSG	T_c [K]	(WP, $\|\boldsymbol{m}\|/\mu_B$)	Band Gap [eV]
1101	$Ho_3Ga_5O_{12}$	$Ia\bar{3}d$ (230)<br>$Ia\bar{3}d'$ (230.148)	0.15	(**24c**, 3.9)	
1102	$Yb_2Sn_2O_7$	$Fd\bar{3}m$ (227)<br>$I4_1/am'd'$ (141.557)	0.15	(**8d**, 1.04)	
1103	$Er_2Sn_2O_7$	$Fd\bar{3}m$ (227)<br>$I4_1'/amd'$ (141.555)	0.1	(**8d**, 4.38)	
1104	$Fe_{0.7}Mn_{0.3}$	$Fm\bar{3}m$ (225)<br>$Pn\bar{3}m'$ (224.113)	-	(**4b**, 1.55)	
1105	$La_{0.73}Tb_{0.27}Mn_2Si_2$	$I4/mmm$ (139)<br>$Pnnm'$ (58.396)	-	(2a,0.0)<br>(**4g**, 1.39)	
1106	$TbMn_2Si_2$	$I4/mmm$ (139)<br>$Pmm'n'$ (59.410)	-	(**2a**, 8.9)<br>(**4f**, 1.52)	
1107	$Pb_2BaCuFeO_5Cl$	$P4/mmm$ (123)<br>P_C2 (3.6)	-	(**4c**, 2.44)<br>(**4c**, 1.45)	
1108	$Pb_2BaCuFeO_5Br$	$P4/mmm$ (123)<br>P_C2 (3.6)	-	(**4c**, 2.47)<br>(**4c**, 1.30)	
1109	$LaBaFeO_4$	$I4/mmm$ (139)<br>P_C4_2/ncm (138.529)	-	(**4d**, 4.06)	
1110	$Fe(ND_3)_2PO_4$	$P2_1/n$ (14)<br>$P_S\bar{1}$ (2.7)	-	(**4i**, 3.69)<br>(**4i**, 3.69)	
1111	$CoNb_2O_6$	$Pbcn$ (60)<br>P_a2_1/c (14.80)	-	(**8e**, 1.0)	
1112	$NiNb_2O_6$	$Pbcn$ (60)<br>P_b2_1/c (14.81)	-	(**4f**, 1.0)<br>(**4e**, 1.0)	
1113	$ErMn_2Ge_2$	$I4/mmm$ (139)<br>P_I4/nnc (126.386)	-	(2a,0.0)<br>(**4d**, 2.2)	
1114	$ErMn_2Ge_2$	$I4/mmm$ (139)<br>P_I4/nnc (126.386)	-	(2a,0.0)<br>(**4d**, 1.7)	
1115	$ErMn_2Si_2$	$I4/mmm$ (139)<br>P_I4/nnc (126.386)	-	(2a,0.0)<br>(**4d**, 2.24)	

Continued on next page

. . . continued

No.	Compound	SG & MSG	T_c [K]	(WP, $\|\boldsymbol{m}\|/\mu_B$)	Band Gap [eV]
1116	$ErMn_2Si_2$	$I4/mmm$ (139)<br>P_I4/nnc (126.386)	-	(2a,0.0)<br>(**4d**, 2.17)	
1117	BaCoSO	$Cmcm$ (63)<br>P_abcm (57.386)	-	(**8d**, 2.75)	
1118	$Fe_{0.967}Nb_3S_6$	$P6_322$ (182)<br>$P_c2_12_12$ (18.21)	-	(**8c**, 3.8)<br>(**8c**, 0.0)<br>(**4b**, 0.0)<br>(**4a**, 0.0)	
1119	RbMnBi	$P4/nmm$ (129)<br>P_c4_2/ncm (138.528)	-	(**4b**, 4.24)	
1120	Er_2BaNiO_5	$Immm$ (71)<br>C_c2/c (15.90)	-	(**8i**, 7.23)<br>(**4c**, 1.39)	
1121	$Eu_{0.5}Ca_{0.5}Fe_2As_2$	$Fmmm$ (69)<br>C_Amca (64.480)	-	(**4a**, 0.0)<br>(**8f**, 1.1)	
1122	$TbMn_2Si_2$	$I4/mmm$ (139)<br>P_I4/nnc (126.386)	-	(2a,0.0)<br>(**4d**, 1.83)	
1123	$Sr_2CuO_2Cu_2S_2$	$I4/mmm$ (139)<br>$I_c\bar{4}2d$ (122.338)	-	(**8c**, 0.39)	
1124	Y_2BaCuO_5	$Pnma$ (62)<br>P_a2_1/c (14.80)	-	(**8e**, 0.55)	
1125	Ba_2YRuO_6	$Fm\bar{3}m$ (225)<br>C_Amca (64.480)	-	(**4a**, 4.5)	
1126	$NdCeBaCu_{0.9}Co_{1.1}O_7$	$I4/mmm$ (139)<br>C_Amca (64.480)	-	(**8g**, 0.88)	
1127	$NdCeBaCuFeO_7$	$I4/mmm$ (139)<br>C_Accm (66.500)	-	(**8g**, 1.46)	
1128	$Pb_2BaCuFeO_5Cl$	$P4/mmm$ (123)<br>I_bmma (74.562)	-	(**8k**, 0.99)	
1129	$La_2NiO_3F_{1.93}$	$C2/c$ (15)<br>P_C2_1/c (14.84)	-	(**4c**, 1.62)	

Continued on next page

. . . continued

No.	Compound	SG & MSG	T_c [K]	(WP, $\|m\|/\mu_B$)	Band Gap [eV]
1130	Sr_2FeO_3F	$P4/nmm$ (129)<br>$P_C\bar{4}2m$ (111.257)	-	(**4g**, 3.73)	
1131	Sr_2FeO_3F	$P4/nmm$ (129)<br>$I_c\bar{4}2m$ (121.332)	-	(**8g**, 3.73)	
1132	Sr_2FeO_3F	$P4/nmm$ (129)<br>$P_C\bar{4}2_1m$ (113.273)	-	(**4g**, 3.73)	
1133	Ca_2FeO_3Br	$P4/nmm$ (129)<br>$P_C\bar{4}2_1m$ (113.273)	-	(**4g**, 3.48)	
1134	Ca_2FeO_3Cl	$P4/nmm$ (129)<br>$P_C\bar{4}2_1m$ (113.273)	-	(**4g**, 3.64)	
1135	Sr_2FeO_3Br	$P4/nmm$ (129)<br>$P_C\bar{4}2_1m$ (113.273)	-	(**4g**, 3.79)	
1136	Sr_2FeO_3Cl	$P4/nmm$ (129)<br>$P_C\bar{4}2_1m$ (113.273)	-	(**4g**, 3.57)	
1137	$Li_3Ni_2SbO_6$	$C2/m$ (12)<br>$P_S\bar{1}$ (2.7)	-	(**4i**, 1.81)	
1138	Dy_2BaNiO_5	$Immm$ (71)<br>C_c2/c (15.90)	-	(**8i**, 7.70)<br>(**4c**, 1.35)	
1139	$YBa_2Fe_3O_{7.84}$	$Pmmm$ (47)<br>C_a2/m (12.64)	-	(**4h**, 3.80)<br>(**8l**, 3.80)	
1140	$YBa_2Fe_3O_{8.08}$	$P4/mmm$ (123)<br>I_bmma (74.562)	-	(**4d**, 3.47)<br>(**8k**, 3.47)	
1141	Yb_2O_2Se	$P\bar{3}m1$ (164)<br>C_c2/c (15.90)	-	(**8i**, 2.01)	
1142	$Ba_3Fe_3O_7F$	$P2_1/m$ (11)<br>P_a2_1/m (11.55)	-	(**4e**, 3.7)<br>(**4e**, 3.5)<br>(**4e**, 3.8)	
1143	LaCrAsO	$P4/nmm$ (129)<br>P_c4_2/ncm (138.528)	-	(**4b**, 1.56)	
1144	$Mn_3Ni_{20}P_6$	$Fm\bar{3}m$ (225)<br>C_Amca (64.480)	-	(**4a**, 2.4)<br>(**8f**, 0.0)	

Continued on next page

. . . continued

No.	Compound	SG & MSG	T_c [K]	(WP, $\|\boldsymbol{m}\|/\mu_B$)	Band Gap [eV]
1145	$AgFe_3(SO_4)_2(OD)_6$	$R\bar{3}m$ (166)<br>$R_I\bar{3}c$ (167.108)	-	(**18e**, 4.51)	
1146	$CoSO_4$	$Pnma$ (62)<br>$Pnma$ (62.441)	-	(**4a**, 3.21)	
1147	$Ca_2Fe_2O_5$	$Pcmn$ (62)<br>$Pcm'n'$ (62.446)	-	(**4a**, 4.30)<br>(**4c**, 3.72)	
1148	$CeMnSi_2$	$Cmcm$ (63)<br>$Cm'cm'$ (63.464)	-	(**4c**, 0.1)<br>(**4c**, 2.7)	
1149	$Ba_3CoRu_2O_9$	$Cmcm$ (63)<br>P_Bnma (62.454)	-	(**4a**, 2.7)<br>(**8f**, 1.44)	
1150	$MnTa_4S_8$	$P6_3/mmc$ (194)<br>$Cmc'm'$ (63.463)	-	(**4a**, 4.2)	
1151	$MnNb_4S_8$	$P6_3/mmc$ (194)<br>$Cmc'm'$ (63.463)	-	(**4a**, 3.4)	
1152	$CrNb_4S_8$	$P6_3/mmc$ (194)<br>$P6_3'/m'm'c$ (194.268)	-	(**2a**, 1.5)	
1153	$Nd_{0.85}Sr_{0.15}CrO_3$	$Pbnm$ (62)<br>$Pnma$ (62.441)	-	(**4c**, 2.2)<br>(**4b**, 2.57)	
1154	$Nd_{0.9}Sr_{0.1}CrO_3$	$Pbnm$ (62)<br>$Pnma$ (62.441)	-	(**4c**, 2.39)<br>(**4b**, 2.77)	
1155	$Nd_{0.95}Sr_{0.05}CrO_3$	$'P$ (62)<br>$Pnma$ (62.441)	-	(**4c**, 3.0)<br>(**4b**, 3.19)	
1156	$La_{0.875}Ba_{0.125}Mn_{0.875}Ti_{0.125}O_3$	$Pnma$ (62)<br>$Pn'ma'$ (62.448)	-	(**4a**, 3.06)	
1157	$La_{0.90}Ba_{0.10}Mn_{0.90}Ti_{0.10}O_3$	$Pnma$ (62)<br>$Pn'ma'$ (62.448)	-	(**4a**, 3.13)	
1158	$La_{0.95}Ba_{0.05}Mn_{0.95}Ti_{0.05}O_3$	$Pnma$ (62)<br>$Pn'ma'$ (62.448)	-	(**4a**, 3.6)	
1159	$La_{0.95}Ba_{0.05}MnO_3$	$Pnma$ (62)<br>$Pn'ma'$ (62.448)	-	(**4a**, 3.35)	

Continued on next page

…continued

No.	Compound	SG & MSG	T_c [K]	(WP, $\|m\|/\mu_B$)	Band Gap [eV]
1160	$La_{0.95}Ca_{0.05}MnO_3$	$Pnma$ (62)<br>$Pn'ma'$ (62.448)	-	(**4a**, 3.51)	
1161	$LaMnO_3$	$Pnma$ (62)<br>$Pn'ma'$ (62.448)	-	(**4a**, 3.7)	
1162	$Ho_{0.2}Bi_{0.8}FeO_3$	$Pnma$ (62)<br>$Pn'ma'$ (62.448)	-	(**4b**, 4.7)	
1163	$Ho_{0.15}Bi_{0.85}FeO_3$	$Pnma$ (62)<br>$Pn'ma'$ (62.448)	-	(**4b**, 4.7)	
1164	$FeMnO_3$	$Ia\bar{3}$ (206)<br>$Ib'c'a$ (73.551)	-	(**8b**, 2.48)<br>(**8b**, 1.0)<br>(**8c**, 3.97)<br>(**8d**, 3.97)<br>(**8e**, 3.97)<br>(**8c**, 3.01)<br>(**8d**, 3.01)<br>(**8e**, 3.01)	
1165	$K_{1.62}Fe_4O_{6.62}(OH)_{0.38}$	$P\bar{3}1c$ (163)<br>$P\bar{3}1c$ (163.79)	-	(**4f**, 4.18)<br>(**4e**, 3.97)	
1166	$LaMn_2Si_2$	$I4/mmm$ (139)<br>$I4'/m'm'm$ (139.536)	-	(**4d**, 1.2)	
1167	$BaMn_2P_2$	$I4/mmm$ (139)<br>$I4'/m'm'm$ (139.536)	-	(**4d**, 4.2)	
1168	FeF_3	$R\bar{3}c$ (167)<br>$C2'/c'$ (15.89)	-	(**4d**, 2.6)	
1169	$Ca_2Fe_{0.875}Cr_{0.125}GaO_5$	$Pnma$ (62)<br>$Pn'm'a$ (62.446)	-	(**4a**, 3.15)<br>(**4a**, 3.15)<br>(**4c**, 0.93)	
1170	$DyVO_3$	$Pbnm$ (62)<br>$P2_1'/m'$ (11.54)	-	(**2e**, 7.76)<br>(**2e**, 7.76)<br>(**2b**, 1.45)<br>(**2c**, 1.45)	
1171	$YCr_{0.5}Mn_{0.5}O_3$	$Pnma$ (62)<br>$Pn'ma'$ (62.448)	-	(**4a**, 1.95)	

Section S7. Tables of subgroups

Table. S 13: Subgroups of $P1$ (1.1) that can be realized by applying $\boldsymbol{E}/\boldsymbol{B}$ fields and/or uniaxial strains.

Subgroup & SI group	Perturbation
(Only $P1$)	

Table. S 14: Subgroups of $P11'$ (1.2) that can be realized by applying $\boldsymbol{E}/\boldsymbol{B}$ fields and/or uniaxial strains.

Subgroup & SI group	Perturbation
(Only $P1$)	

Table. S 15: Subgroups of P_S1 (1.3) that can be realized by applying $\boldsymbol{E}/\boldsymbol{B}$ fields and/or uniaxial strains.

Subgroup & SI group	Perturbation
(Only $P1$)	

Table. S 16: Subgroups of $P\bar{1}$ (2.4) that can be realized by applying $\boldsymbol{E}/\boldsymbol{B}$ fields and/or uniaxial strains.

Subgroup & SI group	Perturbation
(Only $P1$)	

Table. S 17: Subgroups of $P\bar{1}1'$ (2.5) that can be realized by applying $\boldsymbol{E}/\boldsymbol{B}$ fields and/or uniaxial strains.

Subgroup & SI group	Perturbation
$P11'$ (1.2) $\mathbb{Z}_1$	• $\boldsymbol{E}$ in generic direction
$P\bar{1}$ (2.4) $\mathbb{Z}_2\times\mathbb{Z}_2\times\mathbb{Z}_2\times\mathbb{Z}_4$	• $\boldsymbol{B}$ in generic direction

Table. S 18: Subgroups of $P\bar{1}'$ (2.6) that can be realized by applying $\boldsymbol{E}/\boldsymbol{B}$ fields and/or uniaxial strains.

Subgroup & SI group	Perturbation
(Only $P1$)	

Table. S 19: Subgroups of $P_S\bar{1}$ (2.7) that can be realized by applying $\boldsymbol{E}/\boldsymbol{B}$ fields and/or uniaxial strains.

Subgroup & SI group	Perturbation
P_S1 (1.3) $\mathbb{Z}_1$	• $\boldsymbol{E}$ in generic direction
$P\bar{1}$ (2.4) $\mathbb{Z}_2 \times \mathbb{Z}_2 \times \mathbb{Z}_2 \times \mathbb{Z}_4$	• $\boldsymbol{B}$ in generic direction

Table. S 20: Subgroups of $P2$ (3.1) that can be realized by applying $\boldsymbol{E}/\boldsymbol{B}$ fields and/or uniaxial strains.

Subgroup & SI group	Perturbation
(Only $P1$)	

Table. S 21: Subgroups of $P21'$ (3.2) that can be realized by applying $\boldsymbol{E}/\boldsymbol{B}$ fields and/or uniaxial strains.

Subgroup & SI group	Perturbation
$P11'$ (1.2) $\mathbb{Z}_1$	• strain in generic direction<br>• $\boldsymbol{E}$ in generic direction
$P2'$ (3.3) $\mathbb{Z}_1$	• $\boldsymbol{B} \perp [010]$
$P2$ (3.1) $\mathbb{Z}_2$	• $\boldsymbol{B} \parallel [010]$

Table. S 22: Subgroups of $P2'$ (3.3) that can be realized by applying $\boldsymbol{E}/\boldsymbol{B}$ fields and/or uniaxial strains.

Subgroup & SI group	Perturbation
(Only $P1$)	

Table. S 23: Subgroups of P_a2 (3.4) that can be realized by applying $\boldsymbol{E}/\boldsymbol{B}$ fields and/or uniaxial strains.

Subgroup & SI group	Perturbation
P_S1 (1.3) $\mathbb{Z}_1$	• strain in generic direction<br>• $\boldsymbol{E}$ in generic direction
$P2$ (3.1) $\mathbb{Z}_2$	• $\boldsymbol{B} \parallel [010]$
$P2'$ (3.3) $\mathbb{Z}_1$	• $\boldsymbol{B} \perp [010]$

Table. S 24: Subgroups of P_b2 (3.5) that can be realized by applying $\boldsymbol{E}/\boldsymbol{B}$ fields and/or uniaxial strains.

Subgroup & SI group	Perturbation
P_S1 (1.3) $\mathbb{Z}_1$	• strain in generic direction<br>• $\boldsymbol{E}$ in generic direction
$P2$ (3.1) $\mathbb{Z}_2$	• $\boldsymbol{B} \parallel [010]$
$P2_1'$ (4.9) $\mathbb{Z}_1$	• $\boldsymbol{B} \perp [010]$

Table. S 25: Subgroups of P_C2 (3.6) that can be realized by applying $\boldsymbol{E}/\boldsymbol{B}$ fields and/or uniaxial strains.

Subgroup & SI group	Perturbation
P_S1 (1.3) $\mathbb{Z}_1$	• strain in generic direction<br>• $\boldsymbol{E}$ in generic direction
$P2$ (3.1) $\mathbb{Z}_2$	• $\boldsymbol{B} \parallel [010]$

Continued on next page

...continued

Subgroup & SI group	Perturbation
$P2_1'$ (4.9) $\mathbb{Z}_1$	• $\boldsymbol{B} \perp [010]$

Table. S 26: Subgroups of P_a2_1 (4.10) that can be realized by applying $\boldsymbol{E}/\boldsymbol{B}$ fields and/or uniaxial strains.

Subgroup & SI group	Perturbation
P_S1 (1.3) $\mathbb{Z}_1$	• strain in generic direction<br>• $\boldsymbol{E}$ in generic direction
$P2_1$ (4.7) $\mathbb{Z}_1$	• $\boldsymbol{B} \parallel [010]$
$P2_1'$ (4.9) $\mathbb{Z}_1$	• $\boldsymbol{B} \perp [010]$

Table. S 27: Subgroups of P_b2_1 (4.11) that can be realized by applying $\boldsymbol{E}/\boldsymbol{B}$ fields and/or uniaxial strains.

Subgroup & SI group	Perturbation
P_S1 (1.3) $\mathbb{Z}_1$	• strain in generic direction<br>• $\boldsymbol{E}$ in generic direction
$P2'$ (3.3) $\mathbb{Z}_1$	• $\boldsymbol{B} \perp [010]$
$P2_1$ (4.7) $\mathbb{Z}_1$	• $\boldsymbol{B} \parallel [010]$

Table. S 28: Subgroups of P_C2_1 (4.12) that can be realized by applying $\boldsymbol{E}/\boldsymbol{B}$ fields and/or uniaxial strains.

Subgroup & SI group	Perturbation
P_S1 (1.3) $\mathbb{Z}_1$	• strain in generic direction<br>• $\boldsymbol{E}$ in generic direction
$P2_1$ (4.7) $\mathbb{Z}_1$	• $\boldsymbol{B} \parallel [010]$
$P2'$ (3.3) $\mathbb{Z}_1$	• $\boldsymbol{B} \perp [010]$

Table. S 29: Subgroups of $P2_1$ (4.7) that can be realized by applying $\boldsymbol{E}/\boldsymbol{B}$ fields and/or uniaxial strains.

Subgroup & SI group	Perturbation
(Only $P1$)	

Table. S 30: Subgroups of $P2_11'$ (4.8) that can be realized by applying $\boldsymbol{E}/\boldsymbol{B}$ fields and/or uniaxial strains.

Subgroup & SI group	Perturbation
$P11'$ (1.2) $\mathbb{Z}_1$	• strain in generic direction<br>• $\boldsymbol{E}$ in generic direction
$P2_1'$ (4.9) $\mathbb{Z}_1$	• $\boldsymbol{B} \perp [010]$
$P2_1$ (4.7) $\mathbb{Z}_1$	• $\boldsymbol{B} \parallel [010]$

Table. S 31: Subgroups of $P2_1'$ (4.9) that can be realized by applying $\boldsymbol{E}/\boldsymbol{B}$ fields and/or uniaxial strains.

Subgroup & SI group	Perturbation
(Only $P1$)	

Table. S 32: Subgroups of $C2$ (5.13) that can be realized by applying $\boldsymbol{E}/\boldsymbol{B}$ fields and/or uniaxial strains.

Subgroup & SI group	Perturbation
(Only $P1$)	

Table. S 33: Subgroups of $C21'$ (5.14) that can be realized by applying $\boldsymbol{E}/\boldsymbol{B}$ fields and/or uniaxial strains.

Subgroup & SI group	Perturbation
$P11'$ (1.2) $\mathbb{Z}_1$	• strain in generic direction<br>• $\boldsymbol{E}$ in generic direction
$C2'$ (5.15) $\mathbb{Z}_1$	• $\boldsymbol{B} \perp [010]$
$C2$ (5.13) $\mathbb{Z}_1$	• $\boldsymbol{B} \parallel [010]$

Table. S 34: Subgroups of $C2'$ (5.15) that can be realized by applying $\boldsymbol{E}/\boldsymbol{B}$ fields and/or uniaxial strains.

Subgroup & SI group	Perturbation
(Only $P1$)	

Table. S 35: Subgroups of C_c2 (5.16) that can be realized by applying $\boldsymbol{E}/\boldsymbol{B}$ fields and/or uniaxial strains.

Subgroup & SI group	Perturbation
P_S1 (1.3) $\mathbb{Z}_1$	• strain in generic direction<br>• $\boldsymbol{E}$ in generic direction
$C2$ (5.13) $\mathbb{Z}_1$	• $\boldsymbol{B} \parallel [010]$
$C2'$ (5.15) $\mathbb{Z}_1$	• $\boldsymbol{B} \perp [010]$

Table. S 36: Subgroups of C_a2 (5.17) that can be realized by applying $\boldsymbol{E}/\boldsymbol{B}$ fields and/or uniaxial strains.

Subgroup & SI group	Perturbation
P_S1 (1.3) $\mathbb{Z}_1$	• strain in generic direction<br>• $\boldsymbol{E}$ in generic direction
$C2'$ (5.15) $\mathbb{Z}_1$	• $\boldsymbol{B} \perp [010]$

Continued on next page

. . . continued

Subgroup & SI group	Perturbation
$C2$ (5.13) $\mathbb{Z}_1$	• $\boldsymbol{B} \parallel [010]$

Table. S 37: Subgroups of Pm (6.18) that can be realized by applying $\boldsymbol{E}/\boldsymbol{B}$ fields and/or uniaxial strains.

Subgroup & SI group	Perturbation
(Only $P1$)	

Table. S 38: Subgroups of $Pm1'$ (6.19) that can be realized by applying $\boldsymbol{E}/\boldsymbol{B}$ fields and/or uniaxial strains.

Subgroup & SI group	Perturbation
$P11'$ (1.2) $\mathbb{Z}_1$	• strain in generic direction<br>• $\boldsymbol{E}$ in generic direction
Pm' (6.20) $\mathbb{Z}_1$	• $\boldsymbol{B} \perp [010]$
Pm (6.18) $\mathbb{Z}_1$	• $\boldsymbol{B} \parallel [010]$

Table. S 39: Subgroups of Pm' (6.20) that can be realized by applying $\boldsymbol{E}/\boldsymbol{B}$ fields and/or uniaxial strains.

Subgroup & SI group	Perturbation
(Only $P1$)	

Table. S 40: Subgroups of P_am (6.21) that can be realized by applying $\boldsymbol{E}/\boldsymbol{B}$ fields and/or uniaxial strains.

Subgroup & SI group	Perturbation

Continued on next page

...continued

Subgroup & SI group	Perturbation
Pm (6.18) $\mathbb{Z}_1$	• $\boldsymbol{B} \parallel [010]$
P_S1 (1.3) $\mathbb{Z}_1$	• strain in generic direction<br>• $\boldsymbol{E}$ in generic direction
Pc' (7.26) $\mathbb{Z}_1$	• $\boldsymbol{B} \perp [010]$

Table. S 41: Subgroups of P_bm (6.22) that can be realized by applying $\boldsymbol{E}/\boldsymbol{B}$ fields and/or uniaxial strains.

Subgroup & SI group	Perturbation
P_S1 (1.3) $\mathbb{Z}_1$	• strain in generic direction<br>• $\boldsymbol{E}$ in generic direction
Pm (6.18) $\mathbb{Z}_1$	• $\boldsymbol{B} \parallel [010]$
Pm' (6.20) $\mathbb{Z}_1$	• $\boldsymbol{B} \perp [010]$

Table. S 42: Subgroups of P_Cm (6.23) that can be realized by applying $\boldsymbol{E}/\boldsymbol{B}$ fields and/or uniaxial strains.

Subgroup & SI group	Perturbation
Pm (6.18) $\mathbb{Z}_1$	• $\boldsymbol{B} \parallel [010]$
P_S1 (1.3) $\mathbb{Z}_1$	• strain in generic direction<br>• $\boldsymbol{E}$ in generic direction
Pc' (7.26) $\mathbb{Z}_1$	• $\boldsymbol{B} \perp [010]$

Table. S 43: Subgroups of Pc (7.24) that can be realized by applying $\boldsymbol{E}/\boldsymbol{B}$ fields and/or uniaxial strains.

Subgroup & SI group	Perturbation
(Only $P1$)	

Table. S 44: Subgroups of $Pc1'$ (7.25) that can be realized by applying $\boldsymbol{E}/\boldsymbol{B}$ fields and/or uniaxial strains.

Subgroup & SI group	Perturbation
$P11'$ (1.2) $\mathbb{Z}_1$	• strain in generic direction<br>• $\boldsymbol{E}$ in generic direction
Pc' (7.26) $\mathbb{Z}_1$	• $\boldsymbol{B} \perp [010]$
Pc (7.24) $\mathbb{Z}_1$	• $\boldsymbol{B} \parallel [010]$

Table. S 45: Subgroups of Pc' (7.26) that can be realized by applying $\boldsymbol{E}/\boldsymbol{B}$ fields and/or uniaxial strains.

Subgroup & SI group	Perturbation
(Only $P1$)	

Table. S 46: Subgroups of P_ac (7.27) that can be realized by applying $\boldsymbol{E}/\boldsymbol{B}$ fields and/or uniaxial strains.

Subgroup & SI group	Perturbation
Pc (7.24) $\mathbb{Z}_1$	• $\boldsymbol{B} \parallel [010]$
P_S1 (1.3) $\mathbb{Z}_1$	• strain in generic direction<br>• $\boldsymbol{E}$ in generic direction
Pc' (7.26) $\mathbb{Z}_1$	• $\boldsymbol{B} \perp [010]$

Table. S 47: Subgroups of P_cc (7.28) that can be realized by applying $\boldsymbol{E}/\boldsymbol{B}$ fields and/or uniaxial strains.

Subgroup & SI group	Perturbation
P_S1 (1.3) $\mathbb{Z}_1$	• strain in generic direction<br>• $\boldsymbol{E}$ in generic direction
Pm' (6.20) $\mathbb{Z}_1$	• $\boldsymbol{B} \perp [010]$

Continued on next page

…continued

Subgroup & SI group	Perturbation
Pc (7.24) $\mathbb{Z}_1$	• $\boldsymbol{B} \parallel [010]$

Table. S 48: Subgroups of P_bc (7.29) that can be realized by applying $\boldsymbol{E}/\boldsymbol{B}$ fields and/or uniaxial strains.

Subgroup & SI group	Perturbation
P_S1 (1.3) $\mathbb{Z}_1$	• strain in generic direction<br>• $\boldsymbol{E}$ in generic direction
Pc (7.24) $\mathbb{Z}_1$	• $\boldsymbol{B} \parallel [010]$
Pc' (7.26) $\mathbb{Z}_1$	• $\boldsymbol{B} \perp [010]$

Table. S 49: Subgroups of P_Cc (7.30) that can be realized by applying $\boldsymbol{E}/\boldsymbol{B}$ fields and/or uniaxial strains.

Subgroup & SI group	Perturbation
Pc (7.24) $\mathbb{Z}_1$	• $\boldsymbol{B} \parallel [010]$
P_S1 (1.3) $\mathbb{Z}_1$	• strain in generic direction<br>• $\boldsymbol{E}$ in generic direction
Pc' (7.26) $\mathbb{Z}_1$	• $\boldsymbol{B} \perp [010]$

Table. S 50: Subgroups of P_Ac (7.31) that can be realized by applying $\boldsymbol{E}/\boldsymbol{B}$ fields and/or uniaxial strains.

Subgroup & SI group	Perturbation
P_S1 (1.3) $\mathbb{Z}_1$	• strain in generic direction<br>• $\boldsymbol{E}$ in generic direction
Pc (7.24) $\mathbb{Z}_1$	• $\boldsymbol{B} \parallel [010]$
Pm' (6.20) $\mathbb{Z}_1$	• $\boldsymbol{B} \perp [010]$

Table. S 51: Subgroups of Cm (8.32) that can be realized by applying $\boldsymbol{E}/\boldsymbol{B}$ fields and/or uniaxial strains.

Subgroup & SI group	Perturbation
(Only $P1$)	

Table. S 52: Subgroups of $Cm1'$ (8.33) that can be realized by applying $\boldsymbol{E}/\boldsymbol{B}$ fields and/or uniaxial strains.

Subgroup & SI group	Perturbation
$P11'$ (1.2) $\mathbb{Z}_1$	• strain in generic direction<br>• $\boldsymbol{E}$ in generic direction
Cm' (8.34) $\mathbb{Z}_1$	• $\boldsymbol{B} \perp [010]$
Cm (8.32) $\mathbb{Z}_1$	• $\boldsymbol{B} \parallel [010]$

Table. S 53: Subgroups of Cm' (8.34) that can be realized by applying $\boldsymbol{E}/\boldsymbol{B}$ fields and/or uniaxial strains.

Subgroup & SI group	Perturbation
(Only $P1$)	

Table. S 54: Subgroups of C_cm (8.35) that can be realized by applying $\boldsymbol{E}/\boldsymbol{B}$ fields and/or uniaxial strains.

Subgroup & SI group	Perturbation
P_S1 (1.3) $\mathbb{Z}_1$	• strain in generic direction<br>• $\boldsymbol{E}$ in generic direction
Cm (8.32) $\mathbb{Z}_1$	• $\boldsymbol{B} \parallel [010]$
Cc' (9.39) $\mathbb{Z}_1$	• $\boldsymbol{B} \perp [010]$

Table. S 55: Subgroups of C_am (8.36) that can be realized by applying $\boldsymbol{E}/\boldsymbol{B}$ fields and/or uniaxial strains.

Subgroup & SI group	Perturbation
P_S1 (1.3) $\mathbb{Z}_1$	• strain in generic direction<br>• $\boldsymbol{E}$ in generic direction
Cm' (8.34) $\mathbb{Z}_1$	• $\boldsymbol{B} \perp [010]$
Cm (8.32) $\mathbb{Z}_1$	• $\boldsymbol{B} \parallel [010]$

Table. S 56: Subgroups of Cc (9.37) that can be realized by applying $\boldsymbol{E}/\boldsymbol{B}$ fields and/or uniaxial strains.

Subgroup & SI group	Perturbation
(Only $P1$)	

Table. S 57: Subgroups of $Cc1'$ (9.38) that can be realized by applying $\boldsymbol{E}/\boldsymbol{B}$ fields and/or uniaxial strains.

Subgroup & SI group	Perturbation
$P11'$ (1.2) $\mathbb{Z}_1$	• strain in generic direction<br>• $\boldsymbol{E}$ in generic direction
Cc' (9.39) $\mathbb{Z}_1$	• $\boldsymbol{B} \perp [010]$
Cc (9.37) $\mathbb{Z}_1$	• $\boldsymbol{B} \parallel [010]$

Table. S 58: Subgroups of Cc' (9.39) that can be realized by applying $\boldsymbol{E}/\boldsymbol{B}$ fields and/or uniaxial strains.

Subgroup & SI group	Perturbation
(Only $P1$)	

Table. S 59: Subgroups of $C_c c$ (9.40) that can be realized by applying $\boldsymbol{E}/\boldsymbol{B}$ fields and/or uniaxial strains.

Subgroup & SI group	Perturbation
$P_S 1$ (1.3) $\mathbb{Z}_1$	• strain in generic direction<br>• $\boldsymbol{E}$ in generic direction
Cm' (8.34) $\mathbb{Z}_1$	• $\boldsymbol{B} \perp [010]$
Cc (9.37) $\mathbb{Z}_1$	• $\boldsymbol{B} \parallel [010]$

Table. S 60: Subgroups of $C_a c$ (9.41) that can be realized by applying $\boldsymbol{E}/\boldsymbol{B}$ fields and/or uniaxial strains.

Subgroup & SI group	Perturbation
$P_S 1$ (1.3) $\mathbb{Z}_1$	• strain in generic direction<br>• $\boldsymbol{E}$ in generic direction
Cc' (9.39) $\mathbb{Z}_1$	• $\boldsymbol{B} \perp [010]$
Cc (9.37) $\mathbb{Z}_1$	• $\boldsymbol{B} \parallel [010]$

Table. S 61: Subgroups of $P2/m$ (10.42) that can be realized by applying $\boldsymbol{E}/\boldsymbol{B}$ fields and/or uniaxial strains.

Subgroup & SI group	Perturbation
Pm (6.18) $\mathbb{Z}_1$	• $\boldsymbol{E} \perp [010]$
$P2$ (3.1) $\mathbb{Z}_2$	• $\boldsymbol{E} \parallel [010]$
$P\bar{1}$ (2.4) $\mathbb{Z}_2 \times \mathbb{Z}_2 \times \mathbb{Z}_2 \times \mathbb{Z}_4$	• strain in generic direction<br>• $\boldsymbol{B}$ in generic direction

Table. S 62: Subgroups of $P2/m1'$ (10.43) that can be realized by applying $\boldsymbol{E}/\boldsymbol{B}$ fields and/or uniaxial strains.

Subgroup & SI group	Perturbation

Continued on next page

…continued

Subgroup & SI group	Perturbation
$P11'$ (1.2) $\mathbb{Z}_1$	• $\boldsymbol{E} \parallel [010]$ and strain in generic direction<br>• $\boldsymbol{E} \perp [010]$ and strain in generic direction<br>• $\boldsymbol{E}$ in generic direction
Pm' (6.20) $\mathbb{Z}_1$	• $\boldsymbol{E} \perp [010]$ and $\boldsymbol{B} \perp [010]$
Pm (6.18) $\mathbb{Z}_1$	• $\boldsymbol{E} \perp [010]$ and $\boldsymbol{B} \parallel [010]$
$Pm1'$ (6.19) $\mathbb{Z}_1$	• $\boldsymbol{E} \perp [010]$
$P2'$ (3.3) $\mathbb{Z}_1$	• $\boldsymbol{E} \parallel [010]$ and $\boldsymbol{B} \perp [010]$
$P2$ (3.1) $\mathbb{Z}_2$	• $\boldsymbol{E} \parallel [010]$ and $\boldsymbol{B} \parallel [010]$
$P21'$ (3.2) $\mathbb{Z}_2$	• $\boldsymbol{E} \parallel [010]$
$P\bar{1}$ (2.4) $\mathbb{Z}_2 \times \mathbb{Z}_2 \times \mathbb{Z}_2 \times \mathbb{Z}_4$	• $\boldsymbol{B} \parallel [010]$ and strain in generic direction<br>• $\boldsymbol{B} \perp [010]$ and strain in generic direction<br>• $\boldsymbol{B}$ in generic direction
$P2'/m'$ (10.46) $\mathbb{Z}_2 \times \mathbb{Z}_2 \times \mathbb{Z}_2 \times \mathbb{Z}_4$	• $\boldsymbol{B} \perp [010]$
$P2/m$ (10.42) $\mathbb{Z}_2 \times \mathbb{Z}_2 \times \mathbb{Z}_2$	• $\boldsymbol{B} \parallel [010]$
$P\bar{1}1'$ (2.5) $\mathbb{Z}_2 \times \mathbb{Z}_2 \times \mathbb{Z}_2 \times \mathbb{Z}_4$	• strain in generic direction

Table. S 63: Subgroups of $P2'/m$ (10.44) that can be realized by applying $\boldsymbol{E}/\boldsymbol{B}$ fields and/or uniaxial strains.

Subgroup & SI group	Perturbation
Pm (6.18) $\mathbb{Z}_1$	• $\boldsymbol{B} \parallel [010]$<br>• $\boldsymbol{E} \perp [010]$
$P2'$ (3.3) $\mathbb{Z}_1$	• $\boldsymbol{B} \perp [010]$<br>• $\boldsymbol{E} \parallel [010]$
$P\bar{1}'$ (2.6) $\mathbb{Z}_1$	• strain in generic direction

Table. S 64: Subgroups of $P2/m'$ (10.45) that can be realized by applying $\boldsymbol{E}/\boldsymbol{B}$ fields and/or uniaxial strains.

Subgroup & SI group	Perturbation
Pm' (6.20) $\mathbb{Z}_1$	• $\boldsymbol{B} \perp [010]$<br>• $\boldsymbol{E} \perp [010]$
$P2$ (3.1) $\mathbb{Z}_2$	• $\boldsymbol{B} \parallel [010]$<br>• $\boldsymbol{E} \parallel [010]$
$P\bar{1}'$ (2.6) $\mathbb{Z}_1$	• strain in generic direction

Table. S 65: Subgroups of $P2'/m'$ (10.46) that can be realized by applying $\boldsymbol{E}/\boldsymbol{B}$ fields and/or uniaxial strains.

Subgroup & SI group	Perturbation
Pm' (6.20) $\mathbb{Z}_1$	• $\boldsymbol{E} \perp [010]$
$P2'$ (3.3) $\mathbb{Z}_1$	• $\boldsymbol{E} \parallel [010]$
$P\bar{1}$ (2.4) $\mathbb{Z}_2 \times \mathbb{Z}_2 \times \mathbb{Z}_2 \times \mathbb{Z}_4$	• strain in generic direction<br>• $\boldsymbol{B}$ in generic direction

Table. S 66: Subgroups of P_a2/m (10.47) that can be realized by applying $\boldsymbol{E}/\boldsymbol{B}$ fields and/or uniaxial strains.

Subgroup & SI group	Perturbation
Pm (6.18) $\mathbb{Z}_1$	• $\boldsymbol{E} \perp [010]$ and $\boldsymbol{B} \parallel [010]$
P_S1 (1.3) $\mathbb{Z}_1$	• $\boldsymbol{E} \parallel [010]$ and strain in generic direction<br>• $\boldsymbol{E} \perp [010]$ and strain in generic direction<br>• $\boldsymbol{E}$ in generic direction
Pc' (7.26) $\mathbb{Z}_1$	• $\boldsymbol{E} \perp [010]$ and $\boldsymbol{B} \perp [010]$
P_am (6.21) $\mathbb{Z}_1$	• $\boldsymbol{E} \perp [010]$
$P2$ (3.1) $\mathbb{Z}_2$	• $\boldsymbol{E} \parallel [010]$ and $\boldsymbol{B} \parallel [010]$
$P\bar{1}$ (2.4) $\mathbb{Z}_2 \times \mathbb{Z}_2 \times \mathbb{Z}_2 \times \mathbb{Z}_4$	• $\boldsymbol{B} \parallel [010]$ and strain in generic direction<br>• $\boldsymbol{B} \perp [010]$ and strain in generic direction<br>• $\boldsymbol{B}$ in generic direction

Continued on next page

...continued

Subgroup & SI group	Perturbation
$P2/m$ (10.42) $\mathbb{Z}_2 \times \mathbb{Z}_2 \times \mathbb{Z}_2$	• $\boldsymbol{B} \parallel [010]$
$P2'$ (3.3) $\mathbb{Z}_1$	• $\boldsymbol{E} \parallel [010]$ and $\boldsymbol{B} \perp [010]$
P_a2 (3.4) $\mathbb{Z}_2$	• $\boldsymbol{E} \parallel [010]$
$P2'/c'$ (13.69) $\mathbb{Z}_2 \times \mathbb{Z}_2 \times \mathbb{Z}_4$	• $\boldsymbol{B} \perp [010]$
$P_S\bar{1}$ (2.7) $\mathbb{Z}_2 \times \mathbb{Z}_2 \times \mathbb{Z}_4$	• strain in generic direction

Table. S 67: Subgroups of P_b2/m (10.48) that can be realized by applying $\boldsymbol{E}/\boldsymbol{B}$ fields and/or uniaxial strains.

Subgroup & SI group	Perturbation
P_S1 (1.3) $\mathbb{Z}_1$	• $\boldsymbol{E} \parallel [010]$ and strain in generic direction<br>• $\boldsymbol{E} \perp [010]$ and strain in generic direction<br>• $\boldsymbol{E}$ in generic direction
Pm (6.18) $\mathbb{Z}_1$	• $\boldsymbol{E} \perp [010]$ and $\boldsymbol{B} \parallel [010]$
Pm' (6.20) $\mathbb{Z}_1$	• $\boldsymbol{E} \perp [010]$ and $\boldsymbol{B} \perp [010]$
P_bm (6.22) $\mathbb{Z}_1$	• $\boldsymbol{E} \perp [010]$
$P2$ (3.1) $\mathbb{Z}_2$	• $\boldsymbol{E} \parallel [010]$ and $\boldsymbol{B} \parallel [010]$
$P2_1'$ (4.9) $\mathbb{Z}_1$	• $\boldsymbol{E} \parallel [010]$ and $\boldsymbol{B} \perp [010]$
P_b2 (3.5) $\mathbb{Z}_2$	• $\boldsymbol{E} \parallel [010]$
$P\bar{1}$ (2.4) $\mathbb{Z}_2 \times \mathbb{Z}_2 \times \mathbb{Z}_2 \times \mathbb{Z}_4$	• $\boldsymbol{B} \parallel [010]$ and strain in generic direction<br>• $\boldsymbol{B} \perp [010]$ and strain in generic direction<br>• $\boldsymbol{B}$ in generic direction
$P2/m$ (10.42) $\mathbb{Z}_2 \times \mathbb{Z}_2 \times \mathbb{Z}_2$	• $\boldsymbol{B} \parallel [010]$
$P2_1'/m'$ (11.54) $\mathbb{Z}_2 \times \mathbb{Z}_2 \times \mathbb{Z}_4$	• $\boldsymbol{B} \perp [010]$
$P_S\bar{1}$ (2.7) $\mathbb{Z}_2 \times \mathbb{Z}_2 \times \mathbb{Z}_4$	• strain in generic direction

Table. S 68: Subgroups of P_C2/m (10.49) that can be realized by applying $\boldsymbol{E}/\boldsymbol{B}$ fields and/or uniaxial strains.

Subgroup & SI group	Perturbation
Pm (6.18) $\mathbb{Z}_1$	• $\boldsymbol{E} \perp [010]$ and $\boldsymbol{B} \parallel [010]$
P_S1 (1.3) $\mathbb{Z}_1$	• $\boldsymbol{E} \parallel [010]$ and strain in generic direction<br>• $\boldsymbol{E} \perp [010]$ and strain in generic direction<br>• $\boldsymbol{E}$ in generic direction
Pc' (7.26) $\mathbb{Z}_1$	• $\boldsymbol{E} \perp [010]$ and $\boldsymbol{B} \perp [010]$
P_Cm (6.23) $\mathbb{Z}_1$	• $\boldsymbol{E} \perp [010]$
$P2$ (3.1) $\mathbb{Z}_2$	• $\boldsymbol{E} \parallel [010]$ and $\boldsymbol{B} \parallel [010]$
$P\bar{1}$ (2.4) $\mathbb{Z}_2 \times \mathbb{Z}_2 \times \mathbb{Z}_2 \times \mathbb{Z}_4$	• $\boldsymbol{B} \parallel [010]$ and strain in generic direction<br>• $\boldsymbol{B} \perp [010]$ and strain in generic direction<br>• $\boldsymbol{B}$ in generic direction
$P2/m$ (10.42) $\mathbb{Z}_2 \times \mathbb{Z}_2 \times \mathbb{Z}_2$	• $\boldsymbol{B} \parallel [010]$
$P2_1'$ (4.9) $\mathbb{Z}_1$	• $\boldsymbol{E} \parallel [010]$ and $\boldsymbol{B} \perp [010]$
P_C2 (3.6) $\mathbb{Z}_1$	• $\boldsymbol{E} \parallel [010]$
$P2_1'/c'$ (14.79) $\mathbb{Z}_2 \times \mathbb{Z}_4$	• $\boldsymbol{B} \perp [010]$
$P_S\bar{1}$ (2.7) $\mathbb{Z}_2 \times \mathbb{Z}_2 \times \mathbb{Z}_4$	• strain in generic direction

Table. S 69: Subgroups of $P2_1/m$ (11.50) that can be realized by applying $\boldsymbol{E}/\boldsymbol{B}$ fields and/or uniaxial strains.

Subgroup & SI group	Perturbation
Pm (6.18) $\mathbb{Z}_1$	• $\boldsymbol{E} \perp [010]$
$P2_1$ (4.7) $\mathbb{Z}_1$	• $\boldsymbol{E} \parallel [010]$
$P\bar{1}$ (2.4) $\mathbb{Z}_2 \times \mathbb{Z}_2 \times \mathbb{Z}_2 \times \mathbb{Z}_4$	• strain in generic direction<br>• $\boldsymbol{B}$ in generic direction

Table. S 70: Subgroups of $P2_1/m1'$ (11.51) that can be realized by applying $\boldsymbol{E}/\boldsymbol{B}$ fields and/or uniaxial strains.

Subgroup & SI group	Perturbation
$P11'$ (1.2) $\mathbb{Z}_1$	• $\boldsymbol{E} \parallel [010]$ and strain in generic direction<br>• $\boldsymbol{E} \perp [010]$ and strain in generic direction<br>• $\boldsymbol{E}$ in generic direction
Pm' (6.20) $\mathbb{Z}_1$	• $\boldsymbol{E} \perp [010]$ and $\boldsymbol{B} \perp [010]$
Pm (6.18) $\mathbb{Z}_1$	• $\boldsymbol{E} \perp [010]$ and $\boldsymbol{B} \parallel [010]$
$Pm1'$ (6.19) $\mathbb{Z}_1$	• $\boldsymbol{E} \perp [010]$
$P2_1'$ (4.9) $\mathbb{Z}_1$	• $\boldsymbol{E} \parallel [010]$ and $\boldsymbol{B} \perp [010]$
$P2_1$ (4.7) $\mathbb{Z}_1$	• $\boldsymbol{E} \parallel [010]$ and $\boldsymbol{B} \parallel [010]$
$P2_11'$ (4.8) $\mathbb{Z}_1$	• $\boldsymbol{E} \parallel [010]$
$P\bar{1}$ (2.4) $\mathbb{Z}_2 \times \mathbb{Z}_2 \times \mathbb{Z}_2 \times \mathbb{Z}_4$	• $\boldsymbol{B} \parallel [010]$ and strain in generic direction<br>• $\boldsymbol{B} \perp [010]$ and strain in generic direction<br>• $\boldsymbol{B}$ in generic direction
$P2_1'/m'$ (11.54) $\mathbb{Z}_2 \times \mathbb{Z}_2 \times \mathbb{Z}_4$	• $\boldsymbol{B} \perp [010]$
$P2_1/m$ (11.50) $\mathbb{Z}_2$	• $\boldsymbol{B} \parallel [010]$
$P\bar{1}1'$ (2.5) $\mathbb{Z}_2 \times \mathbb{Z}_2 \times \mathbb{Z}_2 \times \mathbb{Z}_4$	• strain in generic direction

Table. S 71: Subgroups of $P2_1'/m$ (11.52) that can be realized by applying $\boldsymbol{E}/\boldsymbol{B}$ fields and/or uniaxial strains.

Subgroup & SI group	Perturbation
Pm (6.18) $\mathbb{Z}_1$	• $\boldsymbol{B} \parallel [010]$<br>• $\boldsymbol{E} \perp [010]$
$P2_1'$ (4.9) $\mathbb{Z}_1$	• $\boldsymbol{B} \perp [010]$<br>• $\boldsymbol{E} \parallel [010]$
$P\bar{1}'$ (2.6) $\mathbb{Z}_1$	• strain in generic direction

Table. S 72: Subgroups of $P2_1/m'$ (11.53) that can be realized by applying $\boldsymbol{E}/\boldsymbol{B}$ fields and/or uniaxial strains.

Subgroup & SI group	Perturbation
Pm' (6.20) $\mathbb{Z}_1$	• $\boldsymbol{B} \perp [010]$<br>• $\boldsymbol{E} \perp [010]$
$P2_1$ (4.7) $\mathbb{Z}_1$	• $\boldsymbol{B} \parallel [010]$<br>• $\boldsymbol{E} \parallel [010]$
$P\bar{1}'$ (2.6) $\mathbb{Z}_1$	• strain in generic direction

Table. S 73: Subgroups of $P2_1'/m'$ (11.54) that can be realized by applying $\boldsymbol{E}/\boldsymbol{B}$ fields and/or uniaxial strains.

Subgroup & SI group	Perturbation
Pm' (6.20) $\mathbb{Z}_1$	• $\boldsymbol{E} \perp [010]$
$P2_1'$ (4.9) $\mathbb{Z}_1$	• $\boldsymbol{E} \parallel [010]$
$P\bar{1}$ (2.4) $\mathbb{Z}_2 \times \mathbb{Z}_2 \times \mathbb{Z}_2 \times \mathbb{Z}_4$	• strain in generic direction<br>• $\boldsymbol{B}$ in generic direction

Table. S 74: Subgroups of P_a2_1/m (11.55) that can be realized by applying $\boldsymbol{E}/\boldsymbol{B}$ fields and/or uniaxial strains.

Subgroup & SI group	Perturbation
Pm (6.18) $\mathbb{Z}_1$	• $\boldsymbol{E} \perp [010]$ and $\boldsymbol{B} \parallel [010]$
P_S1 (1.3) $\mathbb{Z}_1$	• $\boldsymbol{E} \parallel [010]$ and strain in generic direction<br>• $\boldsymbol{E} \perp [010]$ and strain in generic direction<br>• $\boldsymbol{E}$ in generic direction
Pc' (7.26) $\mathbb{Z}_1$	• $\boldsymbol{E} \perp [010]$ and $\boldsymbol{B} \perp [010]$
P_am (6.21) $\mathbb{Z}_1$	• $\boldsymbol{E} \perp [010]$
$P2_1$ (4.7) $\mathbb{Z}_1$	• $\boldsymbol{E} \parallel [010]$ and $\boldsymbol{B} \parallel [010]$
$P\bar{1}$ (2.4) $\mathbb{Z}_2 \times \mathbb{Z}_2 \times \mathbb{Z}_2 \times \mathbb{Z}_4$	• $\boldsymbol{B} \parallel [010]$ and strain in generic direction<br>• $\boldsymbol{B} \perp [010]$ and strain in generic direction<br>• $\boldsymbol{B}$ in generic direction

Continued on next page

...continued

Subgroup & SI group	Perturbation
$P2_1/m$ (11.50) $\mathbb{Z}_2$	• $\boldsymbol{B} \parallel [010]$
$P2_1'$ (4.9) $\mathbb{Z}_1$	• $\boldsymbol{E} \parallel [010]$ and $\boldsymbol{B} \perp [010]$
P_a2_1 (4.10) $\mathbb{Z}_1$	• $\boldsymbol{E} \parallel [010]$
$P2_1'/c'$ (14.79) $\mathbb{Z}_2 \times \mathbb{Z}_4$	• $\boldsymbol{B} \perp [010]$
$P_S\bar{1}$ (2.7) $\mathbb{Z}_2 \times \mathbb{Z}_2 \times \mathbb{Z}_4$	• strain in generic direction

Table. S 75: Subgroups of P_b2_1/m (11.56) that can be realized by applying $\boldsymbol{E}/\boldsymbol{B}$ fields and/or uniaxial strains.

Subgroup & SI group	Perturbation
P_S1 (1.3) $\mathbb{Z}_1$	• $\boldsymbol{E} \parallel [010]$ and strain in generic direction<br>• $\boldsymbol{E} \perp [010]$ and strain in generic direction<br>• $\boldsymbol{E}$ in generic direction
Pm' (6.20) $\mathbb{Z}_1$	• $\boldsymbol{E} \perp [010]$ and $\boldsymbol{B} \perp [010]$
Pm (6.18) $\mathbb{Z}_1$	• $\boldsymbol{E} \perp [010]$ and $\boldsymbol{B} \parallel [010]$
P_bm (6.22) $\mathbb{Z}_1$	• $\boldsymbol{E} \perp [010]$
$P2'$ (3.3) $\mathbb{Z}_1$	• $\boldsymbol{E} \parallel [010]$ and $\boldsymbol{B} \perp [010]$
$P2_1$ (4.7) $\mathbb{Z}_1$	• $\boldsymbol{E} \parallel [010]$ and $\boldsymbol{B} \parallel [010]$
P_b2_1 (4.11) $\mathbb{Z}_1$	• $\boldsymbol{E} \parallel [010]$
$P\bar{1}$ (2.4) $\mathbb{Z}_2 \times \mathbb{Z}_2 \times \mathbb{Z}_2 \times \mathbb{Z}_4$	• $\boldsymbol{B} \parallel [010]$ and strain in generic direction<br>• $\boldsymbol{B} \perp [010]$ and strain in generic direction<br>• $\boldsymbol{B}$ in generic direction
$P2'/m'$ (10.46) $\mathbb{Z}_2 \times \mathbb{Z}_2 \times \mathbb{Z}_2 \times \mathbb{Z}_4$	• $\boldsymbol{B} \perp [010]$
$P2_1/m$ (11.50) $\mathbb{Z}_2$	• $\boldsymbol{B} \parallel [010]$
$P_S\bar{1}$ (2.7) $\mathbb{Z}_2 \times \mathbb{Z}_2 \times \mathbb{Z}_4$	• strain in generic direction

Table. S 76: Subgroups of P_C2_1/m (11.57) that can be realized by applying $\boldsymbol{E}/\boldsymbol{B}$ fields and/or uniaxial strains.

Subgroup & SI group	Perturbation
Pm (6.18) $\mathbb{Z}_1$	• $\boldsymbol{E} \perp [010]$ and $\boldsymbol{B} \parallel [010]$
P_S1 (1.3) $\mathbb{Z}_1$	• $\boldsymbol{E} \parallel [010]$ and strain in generic direction<br>• $\boldsymbol{E} \perp [010]$ and strain in generic direction<br>• $\boldsymbol{E}$ in generic direction
Pc' (7.26) $\mathbb{Z}_1$	• $\boldsymbol{E} \perp [010]$ and $\boldsymbol{B} \perp [010]$
P_Cm (6.23) $\mathbb{Z}_1$	• $\boldsymbol{E} \perp [010]$
$P2_1$ (4.7) $\mathbb{Z}_1$	• $\boldsymbol{E} \parallel [010]$ and $\boldsymbol{B} \parallel [010]$
$P\bar{1}$ (2.4) $\mathbb{Z}_2 \times \mathbb{Z}_2 \times \mathbb{Z}_2 \times \mathbb{Z}_4$	• $\boldsymbol{B} \parallel [010]$ and strain in generic direction<br>• $\boldsymbol{B} \perp [010]$ and strain in generic direction<br>• $\boldsymbol{B}$ in generic direction
$P2_1/m$ (11.50) $\mathbb{Z}_2$	• $\boldsymbol{B} \parallel [010]$
$P2'$ (3.3) $\mathbb{Z}_1$	• $\boldsymbol{E} \parallel [010]$ and $\boldsymbol{B} \perp [010]$
P_C2_1 (4.12) $\mathbb{Z}_1$	• $\boldsymbol{E} \parallel [010]$
$P2'/c'$ (13.69) $\mathbb{Z}_2 \times \mathbb{Z}_2 \times \mathbb{Z}_4$	• $\boldsymbol{B} \perp [010]$
$P_S\bar{1}$ (2.7) $\mathbb{Z}_2 \times \mathbb{Z}_2 \times \mathbb{Z}_4$	• strain in generic direction

Table. S 77: Subgroups of $C2/m$ (12.58) that can be realized by applying $\boldsymbol{E}/\boldsymbol{B}$ fields and/or uniaxial strains.

Subgroup & SI group	Perturbation
Cm (8.32) $\mathbb{Z}_1$	• $\boldsymbol{E} \perp [010]$
$C2$ (5.13) $\mathbb{Z}_1$	• $\boldsymbol{E} \parallel [010]$
$P\bar{1}$ (2.4) $\mathbb{Z}_2 \times \mathbb{Z}_2 \times \mathbb{Z}_2 \times \mathbb{Z}_4$	• strain in generic direction<br>• $\boldsymbol{B}$ in generic direction

Table. S 78: Subgroups of $C2/m1'$ (12.59) that can be realized by applying $\boldsymbol{E}/\boldsymbol{B}$ fields and/or uniaxial strains.

Subgroup & SI group	Perturbation
$P11'$ (1.2) $\mathbb{Z}_1$	• $\boldsymbol{E} \parallel [010]$ and strain in generic direction<br>• $\boldsymbol{E} \perp [010]$ and strain in generic direction<br>• $\boldsymbol{E}$ in generic direction
Cm' (8.34) $\mathbb{Z}_1$	• $\boldsymbol{E} \perp [010]$ and $\boldsymbol{B} \perp [010]$
Cm (8.32) $\mathbb{Z}_1$	• $\boldsymbol{E} \perp [010]$ and $\boldsymbol{B} \parallel [010]$
$Cm1'$ (8.33) $\mathbb{Z}_1$	• $\boldsymbol{E} \perp [010]$
$C2'$ (5.15) $\mathbb{Z}_1$	• $\boldsymbol{E} \parallel [010]$ and $\boldsymbol{B} \perp [010]$
$C2$ (5.13) $\mathbb{Z}_1$	• $\boldsymbol{E} \parallel [010]$ and $\boldsymbol{B} \parallel [010]$
$C21'$ (5.14) $\mathbb{Z}_1$	• $\boldsymbol{E} \parallel [010]$
$P\bar{1}$ (2.4) $\mathbb{Z}_2 \times \mathbb{Z}_2 \times \mathbb{Z}_2 \times \mathbb{Z}_4$	• $\boldsymbol{B} \parallel [010]$ and strain in generic direction<br>• $\boldsymbol{B} \perp [010]$ and strain in generic direction<br>• $\boldsymbol{B}$ in generic direction
$C2'/m'$ (12.62) $\mathbb{Z}_2 \times \mathbb{Z}_2 \times \mathbb{Z}_4$	• $\boldsymbol{B} \perp [010]$
$C2/m$ (12.58) $\mathbb{Z}_2 \times \mathbb{Z}_2$	• $\boldsymbol{B} \parallel [010]$
$P\bar{1}1'$ (2.5) $\mathbb{Z}_2 \times \mathbb{Z}_2 \times \mathbb{Z}_2 \times \mathbb{Z}_4$	• strain in generic direction

Table. S 79: Subgroups of $C2'/m$ (12.60) that can be realized by applying $\boldsymbol{E}/\boldsymbol{B}$ fields and/or uniaxial strains.

Subgroup & SI group	Perturbation
Cm (8.32) $\mathbb{Z}_1$	• $\boldsymbol{B} \parallel [010]$<br>• $\boldsymbol{E} \perp [010]$
$C2'$ (5.15) $\mathbb{Z}_1$	• $\boldsymbol{B} \perp [010]$<br>• $\boldsymbol{E} \parallel [010]$
$P\bar{1}'$ (2.6) $\mathbb{Z}_1$	• strain in generic direction

Table. S 80: Subgroups of $C2/m'$ (12.61) that can be realized by applying $\boldsymbol{E}/\boldsymbol{B}$ fields and/or uniaxial strains.

Subgroup & SI group	Perturbation
Cm' (8.34) $\mathbb{Z}_1$	• $\boldsymbol{B} \perp [010]$<br>• $\boldsymbol{E} \perp [010]$
$C2$ (5.13) $\mathbb{Z}_1$	• $\boldsymbol{B} \parallel [010]$<br>• $\boldsymbol{E} \parallel [010]$
$P\bar{1}'$ (2.6) $\mathbb{Z}_1$	• strain in generic direction

Table. S 81: Subgroups of $C2'/m'$ (12.62) that can be realized by applying $\boldsymbol{E}/\boldsymbol{B}$ fields and/or uniaxial strains.

Subgroup & SI group	Perturbation
Cm' (8.34) $\mathbb{Z}_1$	• $\boldsymbol{E} \perp [010]$
$C2'$ (5.15) $\mathbb{Z}_1$	• $\boldsymbol{E} \parallel [010]$
$P\bar{1}$ (2.4) $\mathbb{Z}_2 \times \mathbb{Z}_2 \times \mathbb{Z}_2 \times \mathbb{Z}_4$	• strain in generic direction<br>• $\boldsymbol{B}$ in generic direction

Table. S 82: Subgroups of C_c2/m (12.63) that can be realized by applying $\boldsymbol{E}/\boldsymbol{B}$ fields and/or uniaxial strains.

Subgroup & SI group	Perturbation
P_S1 (1.3) $\mathbb{Z}_1$	• $\boldsymbol{E} \parallel [010]$ and strain in generic direction<br>• $\boldsymbol{E} \perp [010]$ and strain in generic direction<br>• $\boldsymbol{E}$ in generic direction
Cm (8.32) $\mathbb{Z}_1$	• $\boldsymbol{E} \perp [010]$ and $\boldsymbol{B} \parallel [010]$
Cc' (9.39) $\mathbb{Z}_1$	• $\boldsymbol{E} \perp [010]$ and $\boldsymbol{B} \perp [010]$
C_cm (8.35) $\mathbb{Z}_1$	• $\boldsymbol{E} \perp [010]$
$C2$ (5.13) $\mathbb{Z}_1$	• $\boldsymbol{E} \parallel [010]$ and $\boldsymbol{B} \parallel [010]$
$C2'$ (5.15) $\mathbb{Z}_1$	• $\boldsymbol{E} \parallel [010]$ and $\boldsymbol{B} \perp [010]$
C_c2 (5.16) $\mathbb{Z}_1$	• $\boldsymbol{E} \parallel [010]$

Continued on next page

…continued

Subgroup & SI group	Perturbation
$P\bar{1}$ (2.4) $\mathbb{Z}_2 \times \mathbb{Z}_2 \times \mathbb{Z}_2 \times \mathbb{Z}_4$	• $\boldsymbol{B} \parallel [010]$ and strain in generic direction<br>• $\boldsymbol{B} \perp [010]$ and strain in generic direction<br>• $\boldsymbol{B}$ in generic direction
$C2/m$ (12.58) $\mathbb{Z}_2 \times \mathbb{Z}_2$	• $\boldsymbol{B} \parallel [010]$
$C2'/c'$ (15.89) $\mathbb{Z}_2 \times \mathbb{Z}_4$	• $\boldsymbol{B} \perp [010]$
$P_S\bar{1}$ (2.7) $\mathbb{Z}_2 \times \mathbb{Z}_2 \times \mathbb{Z}_4$	• strain in generic direction

Table. S 83: Subgroups of C_a2/m (12.64) that can be realized by applying $\boldsymbol{E}/\boldsymbol{B}$ fields and/or uniaxial strains.

Subgroup & SI group	Perturbation
P_S1 (1.3) $\mathbb{Z}_1$	• $\boldsymbol{E} \parallel [010]$ and strain in generic direction<br>• $\boldsymbol{E} \perp [010]$ and strain in generic direction<br>• $\boldsymbol{E}$ in generic direction
Cm' (8.34) $\mathbb{Z}_1$	• $\boldsymbol{E} \perp [010]$ and $\boldsymbol{B} \perp [010]$
Cm (8.32) $\mathbb{Z}_1$	• $\boldsymbol{E} \perp [010]$ and $\boldsymbol{B} \parallel [010]$
C_am (8.36) $\mathbb{Z}_1$	• $\boldsymbol{E} \perp [010]$
$C2'$ (5.15) $\mathbb{Z}_1$	• $\boldsymbol{E} \parallel [010]$ and $\boldsymbol{B} \perp [010]$
$C2$ (5.13) $\mathbb{Z}_1$	• $\boldsymbol{E} \parallel [010]$ and $\boldsymbol{B} \parallel [010]$
C_a2 (5.17) $\mathbb{Z}_2$	• $\boldsymbol{E} \parallel [010]$
$P\bar{1}$ (2.4) $\mathbb{Z}_2 \times \mathbb{Z}_2 \times \mathbb{Z}_2 \times \mathbb{Z}_4$	• $\boldsymbol{B} \parallel [010]$ and strain in generic direction<br>• $\boldsymbol{B} \perp [010]$ and strain in generic direction<br>• $\boldsymbol{B}$ in generic direction
$C2'/m'$ (12.62) $\mathbb{Z}_2 \times \mathbb{Z}_2 \times \mathbb{Z}_4$	• $\boldsymbol{B} \perp [010]$
$C2/m$ (12.58) $\mathbb{Z}_2 \times \mathbb{Z}_2$	• $\boldsymbol{B} \parallel [010]$
$P_S\bar{1}$ (2.7) $\mathbb{Z}_2 \times \mathbb{Z}_2 \times \mathbb{Z}_4$	• strain in generic direction

Table. S 84: Subgroups of $P2/c$ (13.65) that can be realized by applying $\boldsymbol{E}/\boldsymbol{B}$ fields and/or uniaxial strains.

Subgroup & SI group	Perturbation
Pc (7.24) $\mathbb{Z}_1$	• $\boldsymbol{E} \perp [010]$
$P2$ (3.1) $\mathbb{Z}_2$	• $\boldsymbol{E} \parallel [010]$
$P\bar{1}$ (2.4) $\mathbb{Z}_2 \times \mathbb{Z}_2 \times \mathbb{Z}_2 \times \mathbb{Z}_4$	• strain in generic direction<br>• $\boldsymbol{B}$ in generic direction

Table. S 85: Subgroups of $P2/c1'$ (13.66) that can be realized by applying $\boldsymbol{E}/\boldsymbol{B}$ fields and/or uniaxial strains.

Subgroup & SI group	Perturbation
$P11'$ (1.2) $\mathbb{Z}_1$	• $\boldsymbol{E} \parallel [010]$ and strain in generic direction<br>• $\boldsymbol{E} \perp [010]$ and strain in generic direction<br>• $\boldsymbol{E}$ in generic direction
Pc' (7.26) $\mathbb{Z}_1$	• $\boldsymbol{E} \perp [010]$ and $\boldsymbol{B} \perp [010]$
Pc (7.24) $\mathbb{Z}_1$	• $\boldsymbol{E} \perp [010]$ and $\boldsymbol{B} \parallel [010]$
$Pc1'$ (7.25) $\mathbb{Z}_1$	• $\boldsymbol{E} \perp [010]$
$P2'$ (3.3) $\mathbb{Z}_1$	• $\boldsymbol{E} \parallel [010]$ and $\boldsymbol{B} \perp [010]$
$P2$ (3.1) $\mathbb{Z}_2$	• $\boldsymbol{E} \parallel [010]$ and $\boldsymbol{B} \parallel [010]$
$P21'$ (3.2) $\mathbb{Z}_2$	• $\boldsymbol{E} \parallel [010]$
$P\bar{1}$ (2.4) $\mathbb{Z}_2 \times \mathbb{Z}_2 \times \mathbb{Z}_2 \times \mathbb{Z}_4$	• $\boldsymbol{B} \parallel [010]$ and strain in generic direction<br>• $\boldsymbol{B} \perp [010]$ and strain in generic direction<br>• $\boldsymbol{B}$ in generic direction
$P2'/c'$ (13.69) $\mathbb{Z}_2 \times \mathbb{Z}_2 \times \mathbb{Z}_4$	• $\boldsymbol{B} \perp [010]$
$P2/c$ (13.65) $\mathbb{Z}_2 \times \mathbb{Z}_2$	• $\boldsymbol{B} \parallel [010]$
$P\bar{1}1'$ (2.5) $\mathbb{Z}_2 \times \mathbb{Z}_2 \times \mathbb{Z}_2 \times \mathbb{Z}_4$	• strain in generic direction

Table. S 86: Subgroups of $P2'/c$ (13.67) that can be realized by applying $\boldsymbol{E}/\boldsymbol{B}$ fields and/or uniaxial strains.

Subgroup & SI group	Perturbation
Pc (7.24) $\mathbb{Z}_1$	• $\boldsymbol{B} \parallel [010]$<br>• $\boldsymbol{E} \perp [010]$
$P2'$ (3.3) $\mathbb{Z}_1$	• $\boldsymbol{B} \perp [010]$<br>• $\boldsymbol{E} \parallel [010]$
$P\bar{1}'$ (2.6) $\mathbb{Z}_1$	• strain in generic direction

Table. S 87: Subgroups of $P2/c'$ (13.68) that can be realized by applying $\boldsymbol{E}/\boldsymbol{B}$ fields and/or uniaxial strains.

Subgroup & SI group	Perturbation
Pc' (7.26) $\mathbb{Z}_1$	• $\boldsymbol{B} \perp [010]$<br>• $\boldsymbol{E} \perp [010]$
$P2$ (3.1) $\mathbb{Z}_2$	• $\boldsymbol{B} \parallel [010]$<br>• $\boldsymbol{E} \parallel [010]$
$P\bar{1}'$ (2.6) $\mathbb{Z}_1$	• strain in generic direction

Table. S 88: Subgroups of $P2'/c'$ (13.69) that can be realized by applying $\boldsymbol{E}/\boldsymbol{B}$ fields and/or uniaxial strains.

Subgroup & SI group	Perturbation
Pc' (7.26) $\mathbb{Z}_1$	• $\boldsymbol{E} \perp [010]$
$P2'$ (3.3) $\mathbb{Z}_1$	• $\boldsymbol{E} \parallel [010]$
$P\bar{1}$ (2.4) $\mathbb{Z}_2 \times \mathbb{Z}_2 \times \mathbb{Z}_2 \times \mathbb{Z}_4$	• strain in generic direction<br>• $\boldsymbol{B}$ in generic direction

Table. S 89: Subgroups of P_a2/c (13.70) that can be realized by applying $\boldsymbol{E}/\boldsymbol{B}$ fields and/or uniaxial strains.

Subgroup & SI group	Perturbation
Pc (7.24) $\mathbb{Z}_1$	• $\boldsymbol{E} \perp [010]$ and $\boldsymbol{B} \parallel [010]$
P_S1 (1.3) $\mathbb{Z}_1$	• $\boldsymbol{E} \parallel [010]$ and strain in generic direction<br>• $\boldsymbol{E} \perp [010]$ and strain in generic direction<br>• $\boldsymbol{E}$ in generic direction
Pc' (7.26) $\mathbb{Z}_1$	• $\boldsymbol{E} \perp [010]$ and $\boldsymbol{B} \perp [010]$
P_ac (7.27) $\mathbb{Z}_1$	• $\boldsymbol{E} \perp [010]$
$P2$ (3.1) $\mathbb{Z}_2$	• $\boldsymbol{E} \parallel [010]$ and $\boldsymbol{B} \parallel [010]$
$P\bar{1}$ (2.4) $\mathbb{Z}_2 \times \mathbb{Z}_2 \times \mathbb{Z}_2 \times \mathbb{Z}_4$	• $\boldsymbol{B} \parallel [010]$ and strain in generic direction<br>• $\boldsymbol{B} \perp [010]$ and strain in generic direction<br>• $\boldsymbol{B}$ in generic direction
$P2/c$ (13.65) $\mathbb{Z}_2 \times \mathbb{Z}_2$	• $\boldsymbol{B} \parallel [010]$
$P2'$ (3.3) $\mathbb{Z}_1$	• $\boldsymbol{E} \parallel [010]$ and $\boldsymbol{B} \perp [010]$
P_a2 (3.4) $\mathbb{Z}_2$	• $\boldsymbol{E} \parallel [010]$
$P2'/c'$ (13.69) $\mathbb{Z}_2 \times \mathbb{Z}_2 \times \mathbb{Z}_4$	• $\boldsymbol{B} \perp [010]$
$P_S\bar{1}$ (2.7) $\mathbb{Z}_2 \times \mathbb{Z}_2 \times \mathbb{Z}_4$	• strain in generic direction

Table. S 90: Subgroups of P_b2/c (13.71) that can be realized by applying $\boldsymbol{E}/\boldsymbol{B}$ fields and/or uniaxial strains.

Subgroup & SI group	Perturbation
P_S1 (1.3) $\mathbb{Z}_1$	• $\boldsymbol{E} \parallel [010]$ and strain in generic direction<br>• $\boldsymbol{E} \perp [010]$ and strain in generic direction<br>• $\boldsymbol{E}$ in generic direction
Pc (7.24) $\mathbb{Z}_1$	• $\boldsymbol{E} \perp [010]$ and $\boldsymbol{B} \parallel [010]$
Pc' (7.26) $\mathbb{Z}_1$	• $\boldsymbol{E} \perp [010]$ and $\boldsymbol{B} \perp [010]$
P_bc (7.29) $\mathbb{Z}_1$	• $\boldsymbol{E} \perp [010]$
$P2$ (3.1) $\mathbb{Z}_2$	• $\boldsymbol{E} \parallel [010]$ and $\boldsymbol{B} \parallel [010]$
$P2_1'$ (4.9) $\mathbb{Z}_1$	• $\boldsymbol{E} \parallel [010]$ and $\boldsymbol{B} \perp [010]$
P_b2 (3.5) $\mathbb{Z}_2$	• $\boldsymbol{E} \parallel [010]$

Continued on next page

. . . continued

Subgroup & SI group	Perturbation
$P\bar{1}$ (2.4) $\mathbb{Z}_2 \times \mathbb{Z}_2 \times \mathbb{Z}_2 \times \mathbb{Z}_4$	• $\boldsymbol{B} \parallel [010]$ and strain in generic direction<br>• $\boldsymbol{B} \perp [010]$ and strain in generic direction<br>• $\boldsymbol{B}$ in generic direction
$P2/c$ (13.65) $\mathbb{Z}_2 \times \mathbb{Z}_2$	• $\boldsymbol{B} \parallel [010]$
$P2_1'/c'$ (14.79) $\mathbb{Z}_2 \times \mathbb{Z}_4$	• $\boldsymbol{B} \perp [010]$
$P_S\bar{1}$ (2.7) $\mathbb{Z}_2 \times \mathbb{Z}_2 \times \mathbb{Z}_4$	• strain in generic direction

Table. S 91: Subgroups of P_c2/c (13.72) that can be realized by applying $\boldsymbol{E}/\boldsymbol{B}$ fields and/or uniaxial strains.

Subgroup & SI group	Perturbation
P_S1 (1.3) $\mathbb{Z}_1$	• $\boldsymbol{E} \parallel [010]$ and strain in generic direction<br>• $\boldsymbol{E} \perp [010]$ and strain in generic direction<br>• $\boldsymbol{E}$ in generic direction
Pm' (6.20) $\mathbb{Z}_1$	• $\boldsymbol{E} \perp [010]$ and $\boldsymbol{B} \perp [010]$
Pc (7.24) $\mathbb{Z}_1$	• $\boldsymbol{E} \perp [010]$ and $\boldsymbol{B} \parallel [010]$
P_cc (7.28) $\mathbb{Z}_1$	• $\boldsymbol{E} \perp [010]$
$P2'$ (3.3) $\mathbb{Z}_1$	• $\boldsymbol{E} \parallel [010]$ and $\boldsymbol{B} \perp [010]$
$P2$ (3.1) $\mathbb{Z}_2$	• $\boldsymbol{E} \parallel [010]$ and $\boldsymbol{B} \parallel [010]$
P_a2 (3.4) $\mathbb{Z}_2$	• $\boldsymbol{E} \parallel [010]$
$P\bar{1}$ (2.4) $\mathbb{Z}_2 \times \mathbb{Z}_2 \times \mathbb{Z}_2 \times \mathbb{Z}_4$	• $\boldsymbol{B} \parallel [010]$ and strain in generic direction<br>• $\boldsymbol{B} \perp [010]$ and strain in generic direction<br>• $\boldsymbol{B}$ in generic direction
$P2'/m'$ (10.46) $\mathbb{Z}_2 \times \mathbb{Z}_2 \times \mathbb{Z}_2 \times \mathbb{Z}_4$	• $\boldsymbol{B} \perp [010]$
$P2/c$ (13.65) $\mathbb{Z}_2 \times \mathbb{Z}_2$	• $\boldsymbol{B} \parallel [010]$
$P_S\bar{1}$ (2.7) $\mathbb{Z}_2 \times \mathbb{Z}_2 \times \mathbb{Z}_4$	• strain in generic direction

Table. S 92: Subgroups of P_A2/c (13.73) that can be realized by applying $\boldsymbol{E}/\boldsymbol{B}$ fields and/or uniaxial strains.

Subgroup & SI group	Perturbation
P_S1 (1.3) $\mathbb{Z}_1$	• $\boldsymbol{E}$ ∥ [010] and strain in generic direction<br>• $\boldsymbol{E}$ ⊥ [010] and strain in generic direction<br>• $\boldsymbol{E}$ in generic direction
Pc (7.24) $\mathbb{Z}_1$	• $\boldsymbol{E}$ ⊥ [010] and $\boldsymbol{B}$ ∥ [010]
Pm' (6.20) $\mathbb{Z}_1$	• $\boldsymbol{E}$ ⊥ [010] and $\boldsymbol{B}$ ⊥ [010]
P_Ac (7.31) $\mathbb{Z}_1$	• $\boldsymbol{E}$ ⊥ [010]
$P2$ (3.1) $\mathbb{Z}_2$	• $\boldsymbol{E}$ ∥ [010] and $\boldsymbol{B}$ ∥ [010]
$P2_1'$ (4.9) $\mathbb{Z}_1$	• $\boldsymbol{E}$ ∥ [010] and $\boldsymbol{B}$ ⊥ [010]
P_C2 (3.6) $\mathbb{Z}_1$	• $\boldsymbol{E}$ ∥ [010]
$P\bar{1}$ (2.4) $\mathbb{Z}_2 \times \mathbb{Z}_2 \times \mathbb{Z}_2 \times \mathbb{Z}_4$	• $\boldsymbol{B}$ ∥ [010] and strain in generic direction<br>• $\boldsymbol{B}$ ⊥ [010] and strain in generic direction<br>• $\boldsymbol{B}$ in generic direction
$P2/c$ (13.65) $\mathbb{Z}_2 \times \mathbb{Z}_2$	• $\boldsymbol{B}$ ∥ [010]
$P2_1'/m'$ (11.54) $\mathbb{Z}_2 \times \mathbb{Z}_2 \times \mathbb{Z}_4$	• $\boldsymbol{B}$ ⊥ [010]
$P_S\bar{1}$ (2.7) $\mathbb{Z}_2 \times \mathbb{Z}_2 \times \mathbb{Z}_4$	• strain in generic direction

Table. S 93: Subgroups of P_C2/c (13.74) that can be realized by applying $\boldsymbol{E}/\boldsymbol{B}$ fields and/or uniaxial strains.

Subgroup & SI group	Perturbation
Pc (7.24) $\mathbb{Z}_1$	• $\boldsymbol{E}$ ⊥ [010] and $\boldsymbol{B}$ ∥ [010]
P_S1 (1.3) $\mathbb{Z}_1$	• $\boldsymbol{E}$ ∥ [010] and strain in generic direction<br>• $\boldsymbol{E}$ ⊥ [010] and strain in generic direction<br>• $\boldsymbol{E}$ in generic direction
Pc' (7.26) $\mathbb{Z}_1$	• $\boldsymbol{E}$ ⊥ [010] and $\boldsymbol{B}$ ⊥ [010]
P_Cc (7.30) $\mathbb{Z}_1$	• $\boldsymbol{E}$ ⊥ [010]
$P2$ (3.1) $\mathbb{Z}_2$	• $\boldsymbol{E}$ ∥ [010] and $\boldsymbol{B}$ ∥ [010]
$P\bar{1}$ (2.4) $\mathbb{Z}_2 \times \mathbb{Z}_2 \times \mathbb{Z}_2 \times \mathbb{Z}_4$	• $\boldsymbol{B}$ ∥ [010] and strain in generic direction<br>• $\boldsymbol{B}$ ⊥ [010] and strain in generic direction<br>• $\boldsymbol{B}$ in generic direction

Continued on next page

...continued

Subgroup & SI group	Perturbation
$P2/c$ (13.65) $\mathbb{Z}_2 \times \mathbb{Z}_2$	• $\boldsymbol{B} \parallel [010]$
$P2_1'$ (4.9) $\mathbb{Z}_1$	• $\boldsymbol{E} \parallel [010]$ and $\boldsymbol{B} \perp [010]$
P_C2 (3.6) $\mathbb{Z}_1$	• $\boldsymbol{E} \parallel [010]$
$P2_1'/c'$ (14.79) $\mathbb{Z}_2 \times \mathbb{Z}_4$	• $\boldsymbol{B} \perp [010]$
$P_S\bar{1}$ (2.7) $\mathbb{Z}_2 \times \mathbb{Z}_2 \times \mathbb{Z}_4$	• strain in generic direction

Table. S 94: Subgroups of $P2_1/c$ (14.75) that can be realized by applying $\boldsymbol{E}/\boldsymbol{B}$ fields and/or uniaxial strains.

Subgroup & SI group	Perturbation
Pc (7.24) $\mathbb{Z}_1$	• $\boldsymbol{E} \perp [010]$
$P2_1$ (4.7) $\mathbb{Z}_1$	• $\boldsymbol{E} \parallel [010]$
$P\bar{1}$ (2.4) $\mathbb{Z}_2 \times \mathbb{Z}_2 \times \mathbb{Z}_2 \times \mathbb{Z}_4$	• strain in generic direction<br>• $\boldsymbol{B}$ in generic direction

Table. S 95: Subgroups of $P2_1/c1'$ (14.76) that can be realized by applying $\boldsymbol{E}/\boldsymbol{B}$ fields and/or uniaxial strains.

Subgroup & SI group	Perturbation
$P11'$ (1.2) $\mathbb{Z}_1$	• $\boldsymbol{E} \parallel [010]$ and strain in generic direction<br>• $\boldsymbol{E} \perp [010]$ and strain in generic direction<br>• $\boldsymbol{E}$ in generic direction
Pc' (7.26) $\mathbb{Z}_1$	• $\boldsymbol{E} \perp [010]$ and $\boldsymbol{B} \perp [010]$
Pc (7.24) $\mathbb{Z}_1$	• $\boldsymbol{E} \perp [010]$ and $\boldsymbol{B} \parallel [010]$
$Pc1'$ (7.25) $\mathbb{Z}_1$	• $\boldsymbol{E} \perp [010]$
$P2_1'$ (4.9) $\mathbb{Z}_1$	• $\boldsymbol{E} \parallel [010]$ and $\boldsymbol{B} \perp [010]$
$P2_1$ (4.7) $\mathbb{Z}_1$	• $\boldsymbol{E} \parallel [010]$ and $\boldsymbol{B} \parallel [010]$
$P2_11'$ (4.8) $\mathbb{Z}_1$	• $\boldsymbol{E} \parallel [010]$

Continued on next page

...continued

Subgroup & SI group	Perturbation
$P\bar{1}$ (2.4) $\mathbb{Z}_2 \times \mathbb{Z}_2 \times \mathbb{Z}_2 \times \mathbb{Z}_4$	• $\boldsymbol{B} \parallel [010]$ and strain in generic direction<br>• $\boldsymbol{B} \perp [010]$ and strain in generic direction<br>• $\boldsymbol{B}$ in generic direction
$P2_1'/c'$ (14.79) $\mathbb{Z}_2 \times \mathbb{Z}_4$	• $\boldsymbol{B} \perp [010]$
$P2_1/c$ (14.75) $\mathbb{Z}_2$	• $\boldsymbol{B} \parallel [010]$
$P\bar{1}1'$ (2.5) $\mathbb{Z}_2 \times \mathbb{Z}_2 \times \mathbb{Z}_2 \times \mathbb{Z}_4$	• strain in generic direction

Table. S 96: Subgroups of $P2_1'/c$ (14.77) that can be realized by applying $\boldsymbol{E}/\boldsymbol{B}$ fields and/or uniaxial strains.

Subgroup & SI group	Perturbation
Pc (7.24) $\mathbb{Z}_1$	• $\boldsymbol{B} \parallel [010]$<br>• $\boldsymbol{E} \perp [010]$
$P2_1'$ (4.9) $\mathbb{Z}_1$	• $\boldsymbol{B} \perp [010]$<br>• $\boldsymbol{E} \parallel [010]$
$P\bar{1}'$ (2.6) $\mathbb{Z}_1$	• strain in generic direction

Table. S 97: Subgroups of $P2_1/c'$ (14.78) that can be realized by applying $\boldsymbol{E}/\boldsymbol{B}$ fields and/or uniaxial strains.

Subgroup & SI group	Perturbation
Pc' (7.26) $\mathbb{Z}_1$	• $\boldsymbol{B} \perp [010]$<br>• $\boldsymbol{E} \perp [010]$
$P2_1$ (4.7) $\mathbb{Z}_1$	• $\boldsymbol{B} \parallel [010]$<br>• $\boldsymbol{E} \parallel [010]$
$P\bar{1}'$ (2.6) $\mathbb{Z}_1$	• strain in generic direction

Table. S 98: Subgroups of $P2_1'/c'$ (14.79) that can be realized by applying $\boldsymbol{E}/\boldsymbol{B}$ fields and/or uniaxial strains.

Subgroup & SI group	Perturbation
Pc' (7.26) $\mathbb{Z}_1$	• $\boldsymbol{E} \perp [010]$
$P2_1'$ (4.9) $\mathbb{Z}_1$	• $\boldsymbol{E} \parallel [010]$
$P\bar{1}$ (2.4) $\mathbb{Z}_2 \times \mathbb{Z}_2 \times \mathbb{Z}_2 \times \mathbb{Z}_4$	• strain in generic direction<br>• $\boldsymbol{B}$ in generic direction

Table. S 99: Subgroups of P_a2_1/c (14.80) that can be realized by applying $\boldsymbol{E}/\boldsymbol{B}$ fields and/or uniaxial strains.

Subgroup & SI group	Perturbation
Pc (7.24) $\mathbb{Z}_1$	• $\boldsymbol{E} \perp [010]$ and $\boldsymbol{B} \parallel [010]$
P_S1 (1.3) $\mathbb{Z}_1$	• $\boldsymbol{E} \parallel [010]$ and strain in generic direction<br>• $\boldsymbol{E} \perp [010]$ and strain in generic direction<br>• $\boldsymbol{E}$ in generic direction
Pc' (7.26) $\mathbb{Z}_1$	• $\boldsymbol{E} \perp [010]$ and $\boldsymbol{B} \perp [010]$
P_ac (7.27) $\mathbb{Z}_1$	• $\boldsymbol{E} \perp [010]$
$P2_1$ (4.7) $\mathbb{Z}_1$	• $\boldsymbol{E} \parallel [010]$ and $\boldsymbol{B} \parallel [010]$
$P\bar{1}$ (2.4) $\mathbb{Z}_2 \times \mathbb{Z}_2 \times \mathbb{Z}_2 \times \mathbb{Z}_4$	• $\boldsymbol{B} \parallel [010]$ and strain in generic direction<br>• $\boldsymbol{B} \perp [010]$ and strain in generic direction<br>• $\boldsymbol{B}$ in generic direction
$P2_1/c$ (14.75) $\mathbb{Z}_2$	• $\boldsymbol{B} \parallel [010]$
$P2_1'$ (4.9) $\mathbb{Z}_1$	• $\boldsymbol{E} \parallel [010]$ and $\boldsymbol{B} \perp [010]$
P_a2_1 (4.10) $\mathbb{Z}_1$	• $\boldsymbol{E} \parallel [010]$
$P2_1'/c'$ (14.79) $\mathbb{Z}_2 \times \mathbb{Z}_4$	• $\boldsymbol{B} \perp [010]$
$P_S\bar{1}$ (2.7) $\mathbb{Z}_2 \times \mathbb{Z}_2 \times \mathbb{Z}_4$	• strain in generic direction

Table. S 100: Subgroups of P_b2_1/c (14.81) that can be realized by applying $\boldsymbol{E}/\boldsymbol{B}$ fields and/or uniaxial strains.

Subgroup & SI group	Perturbation
P_S1 (1.3) $\mathbb{Z}_1$	• $\boldsymbol{E} \parallel [010]$ and strain in generic direction<br>• $\boldsymbol{E} \perp [010]$ and strain in generic direction<br>• $\boldsymbol{E}$ in generic direction
Pc' (7.26) $\mathbb{Z}_1$	• $\boldsymbol{E} \perp [010]$ and $\boldsymbol{B} \perp [010]$
Pc (7.24) $\mathbb{Z}_1$	• $\boldsymbol{E} \perp [010]$ and $\boldsymbol{B} \parallel [010]$
P_bc (7.29) $\mathbb{Z}_1$	• $\boldsymbol{E} \perp [010]$
$P2'$ (3.3) $\mathbb{Z}_1$	• $\boldsymbol{E} \parallel [010]$ and $\boldsymbol{B} \perp [010]$
$P2_1$ (4.7) $\mathbb{Z}_1$	• $\boldsymbol{E} \parallel [010]$ and $\boldsymbol{B} \parallel [010]$
P_b2_1 (4.11) $\mathbb{Z}_1$	• $\boldsymbol{E} \parallel [010]$
$P\bar{1}$ (2.4) $\mathbb{Z}_2 \times \mathbb{Z}_2 \times \mathbb{Z}_2 \times \mathbb{Z}_4$	• $\boldsymbol{B} \parallel [010]$ and strain in generic direction<br>• $\boldsymbol{B} \perp [010]$ and strain in generic direction<br>• $\boldsymbol{B}$ in generic direction
$P2'/c'$ (13.69) $\mathbb{Z}_2 \times \mathbb{Z}_2 \times \mathbb{Z}_4$	• $\boldsymbol{B} \perp [010]$
$P2_1/c$ (14.75) $\mathbb{Z}_2$	• $\boldsymbol{B} \parallel [010]$
$P_S\bar{1}$ (2.7) $\mathbb{Z}_2 \times \mathbb{Z}_2 \times \mathbb{Z}_4$	• strain in generic direction

Table. S 101: Subgroups of P_c2_1/c (14.82) that can be realized by applying $\boldsymbol{E}/\boldsymbol{B}$ fields and/or uniaxial strains.

Subgroup & SI group	Perturbation
P_S1 (1.3) $\mathbb{Z}_1$	• $\boldsymbol{E} \parallel [010]$ and strain in generic direction<br>• $\boldsymbol{E} \perp [010]$ and strain in generic direction<br>• $\boldsymbol{E}$ in generic direction
Pm' (6.20) $\mathbb{Z}_1$	• $\boldsymbol{E} \perp [010]$ and $\boldsymbol{B} \perp [010]$
Pc (7.24) $\mathbb{Z}_1$	• $\boldsymbol{E} \perp [010]$ and $\boldsymbol{B} \parallel [010]$
P_cc (7.28) $\mathbb{Z}_1$	• $\boldsymbol{E} \perp [010]$
$P2_1'$ (4.9) $\mathbb{Z}_1$	• $\boldsymbol{E} \parallel [010]$ and $\boldsymbol{B} \perp [010]$
$P2_1$ (4.7) $\mathbb{Z}_1$	• $\boldsymbol{E} \parallel [010]$ and $\boldsymbol{B} \parallel [010]$
P_a2_1 (4.10) $\mathbb{Z}_1$	• $\boldsymbol{E} \parallel [010]$

Continued on next page

. . . continued

Subgroup & SI group	Perturbation
$P\bar{1}$ (2.4) $\mathbb{Z}_2 \times \mathbb{Z}_2 \times \mathbb{Z}_2 \times \mathbb{Z}_4$	• $\boldsymbol{B} \parallel [010]$ and strain in generic direction<br>• $\boldsymbol{B} \perp [010]$ and strain in generic direction<br>• $\boldsymbol{B}$ in generic direction
$P2_1'/m'$ (11.54) $\mathbb{Z}_2 \times \mathbb{Z}_2 \times \mathbb{Z}_4$	• $\boldsymbol{B} \perp [010]$
$P2_1/c$ (14.75) $\mathbb{Z}_2$	• $\boldsymbol{B} \parallel [010]$
$P_S\bar{1}$ (2.7) $\mathbb{Z}_2 \times \mathbb{Z}_2 \times \mathbb{Z}_4$	• strain in generic direction

Table. S 102: Subgroups of P_A2_1/c (14.83) that can be realized by applying $\boldsymbol{E}/\boldsymbol{B}$ fields and/or uniaxial strains.

Subgroup & SI group	Perturbation
P_S1 (1.3) $\mathbb{Z}_1$	• $\boldsymbol{E} \parallel [010]$ and strain in generic direction<br>• $\boldsymbol{E} \perp [010]$ and strain in generic direction<br>• $\boldsymbol{E}$ in generic direction
Pm' (6.20) $\mathbb{Z}_1$	• $\boldsymbol{E} \perp [010]$ and $\boldsymbol{B} \perp [010]$
Pc (7.24) $\mathbb{Z}_1$	• $\boldsymbol{E} \perp [010]$ and $\boldsymbol{B} \parallel [010]$
P_Ac (7.31) $\mathbb{Z}_1$	• $\boldsymbol{E} \perp [010]$
$P2'$ (3.3) $\mathbb{Z}_1$	• $\boldsymbol{E} \parallel [010]$ and $\boldsymbol{B} \perp [010]$
$P2_1$ (4.7) $\mathbb{Z}_1$	• $\boldsymbol{E} \parallel [010]$ and $\boldsymbol{B} \parallel [010]$
P_C2_1 (4.12) $\mathbb{Z}_1$	• $\boldsymbol{E} \parallel [010]$
$P\bar{1}$ (2.4) $\mathbb{Z}_2 \times \mathbb{Z}_2 \times \mathbb{Z}_2 \times \mathbb{Z}_4$	• $\boldsymbol{B} \parallel [010]$ and strain in generic direction<br>• $\boldsymbol{B} \perp [010]$ and strain in generic direction<br>• $\boldsymbol{B}$ in generic direction
$P2'/m'$ (10.46) $\mathbb{Z}_2 \times \mathbb{Z}_2 \times \mathbb{Z}_2 \times \mathbb{Z}_4$	• $\boldsymbol{B} \perp [010]$
$P2_1/c$ (14.75) $\mathbb{Z}_2$	• $\boldsymbol{B} \parallel [010]$
$P_S\bar{1}$ (2.7) $\mathbb{Z}_2 \times \mathbb{Z}_2 \times \mathbb{Z}_4$	• strain in generic direction

Table. S 103: Subgroups of $P_C 2_1/c$ (14.84) that can be realized by applying $\boldsymbol{E}/\boldsymbol{B}$ fields and/or uniaxial strains.

Subgroup & SI group	Perturbation
Pc (7.24) $\mathbb{Z}_1$	• $\boldsymbol{E} \perp [010]$ and $\boldsymbol{B} \parallel [010]$
P_S1 (1.3) $\mathbb{Z}_1$	• $\boldsymbol{E} \parallel [010]$ and strain in generic direction<br>• $\boldsymbol{E} \perp [010]$ and strain in generic direction<br>• $\boldsymbol{E}$ in generic direction
Pc' (7.26) $\mathbb{Z}_1$	• $\boldsymbol{E} \perp [010]$ and $\boldsymbol{B} \perp [010]$
P_Cc (7.30) $\mathbb{Z}_1$	• $\boldsymbol{E} \perp [010]$
$P2_1$ (4.7) $\mathbb{Z}_1$	• $\boldsymbol{E} \parallel [010]$ and $\boldsymbol{B} \parallel [010]$
$P\bar{1}$ (2.4) $\mathbb{Z}_2 \times \mathbb{Z}_2 \times \mathbb{Z}_2 \times \mathbb{Z}_4$	• $\boldsymbol{B} \parallel [010]$ and strain in generic direction<br>• $\boldsymbol{B} \perp [010]$ and strain in generic direction<br>• $\boldsymbol{B}$ in generic direction
$P2_1/c$ (14.75) $\mathbb{Z}_2$	• $\boldsymbol{B} \parallel [010]$
$P2'$ (3.3) $\mathbb{Z}_1$	• $\boldsymbol{E} \parallel [010]$ and $\boldsymbol{B} \perp [010]$
P_C2_1 (4.12) $\mathbb{Z}_1$	• $\boldsymbol{E} \parallel [010]$
$P2'/c'$ (13.69) $\mathbb{Z}_2 \times \mathbb{Z}_2 \times \mathbb{Z}_4$	• $\boldsymbol{B} \perp [010]$
$P_S\bar{1}$ (2.7) $\mathbb{Z}_2 \times \mathbb{Z}_2 \times \mathbb{Z}_4$	• strain in generic direction

Table. S 104: Subgroups of $C2/c$ (15.85) that can be realized by applying $\boldsymbol{E}/\boldsymbol{B}$ fields and/or uniaxial strains.

Subgroup & SI group	Perturbation
Cc (9.37) $\mathbb{Z}_1$	• $\boldsymbol{E} \perp [010]$
$C2$ (5.13) $\mathbb{Z}_1$	• $\boldsymbol{E} \parallel [010]$
$P\bar{1}$ (2.4) $\mathbb{Z}_2 \times \mathbb{Z}_2 \times \mathbb{Z}_2 \times \mathbb{Z}_4$	• strain in generic direction<br>• $\boldsymbol{B}$ in generic direction

Table. S 105: Subgroups of $C2/c1'$ (15.86) that can be realized by applying $\boldsymbol{E}/\boldsymbol{B}$ fields and/or uniaxial strains.

Subgroup & SI group	Perturbation
$P11'$ (1.2) $\mathbb{Z}_1$	• $\boldsymbol{E} \parallel [010]$ and strain in generic direction<br>• $\boldsymbol{E} \perp [010]$ and strain in generic direction<br>• $\boldsymbol{E}$ in generic direction
Cc' (9.39) $\mathbb{Z}_1$	• $\boldsymbol{E} \perp [010]$ and $\boldsymbol{B} \perp [010]$
Cc (9.37) $\mathbb{Z}_1$	• $\boldsymbol{E} \perp [010]$ and $\boldsymbol{B} \parallel [010]$
$Cc1'$ (9.38) $\mathbb{Z}_1$	• $\boldsymbol{E} \perp [010]$
$C2'$ (5.15) $\mathbb{Z}_1$	• $\boldsymbol{E} \parallel [010]$ and $\boldsymbol{B} \perp [010]$
$C2$ (5.13) $\mathbb{Z}_1$	• $\boldsymbol{E} \parallel [010]$ and $\boldsymbol{B} \parallel [010]$
$C21'$ (5.14) $\mathbb{Z}_1$	• $\boldsymbol{E} \parallel [010]$
$P\bar{1}$ (2.4) $\mathbb{Z}_2 \times \mathbb{Z}_2 \times \mathbb{Z}_2 \times \mathbb{Z}_4$	• $\boldsymbol{B} \parallel [010]$ and strain in generic direction<br>• $\boldsymbol{B} \perp [010]$ and strain in generic direction<br>• $\boldsymbol{B}$ in generic direction
$C2'/c'$ (15.89) $\mathbb{Z}_2 \times \mathbb{Z}_4$	• $\boldsymbol{B} \perp [010]$
$C2/c$ (15.85) $\mathbb{Z}_2 \times \mathbb{Z}_2$	• $\boldsymbol{B} \parallel [010]$
$P\bar{1}1'$ (2.5) $\mathbb{Z}_2 \times \mathbb{Z}_2 \times \mathbb{Z}_2 \times \mathbb{Z}_4$	• strain in generic direction

Table. S 106: Subgroups of $C2'/c$ (15.87) that can be realized by applying $\boldsymbol{E}/\boldsymbol{B}$ fields and/or uniaxial strains.

Subgroup & SI group	Perturbation
Cc (9.37) $\mathbb{Z}_1$	• $\boldsymbol{B} \parallel [010]$<br>• $\boldsymbol{E} \perp [010]$
$C2'$ (5.15) $\mathbb{Z}_1$	• $\boldsymbol{B} \perp [010]$<br>• $\boldsymbol{E} \parallel [010]$
$P\bar{1}'$ (2.6) $\mathbb{Z}_1$	• strain in generic direction

Table. S 107: Subgroups of $C2/c'$ (15.88) that can be realized by applying $\boldsymbol{E}/\boldsymbol{B}$ fields and/or uniaxial strains.

Subgroup & SI group	Perturbation
Cc' (9.39) $\mathbb{Z}_1$	• $\boldsymbol{B} \perp [010]$<br>• $\boldsymbol{E} \perp [010]$
$C2$ (5.13) $\mathbb{Z}_1$	• $\boldsymbol{B} \parallel [010]$<br>• $\boldsymbol{E} \parallel [010]$
$P\bar{1}'$ (2.6) $\mathbb{Z}_1$	• strain in generic direction

Table. S 108: Subgroups of $C2'/c'$ (15.89) that can be realized by applying $\boldsymbol{E}/\boldsymbol{B}$ fields and/or uniaxial strains.

Subgroup & SI group	Perturbation
Cc' (9.39) $\mathbb{Z}_1$	• $\boldsymbol{E} \perp [010]$
$C2'$ (5.15) $\mathbb{Z}_1$	• $\boldsymbol{E} \parallel [010]$
$P\bar{1}$ (2.4) $\mathbb{Z}_2 \times \mathbb{Z}_2 \times \mathbb{Z}_2 \times \mathbb{Z}_4$	• strain in generic direction<br>• $\boldsymbol{B}$ in generic direction

Table. S 109: Subgroups of C_c2/c (15.90) that can be realized by applying $\boldsymbol{E}/\boldsymbol{B}$ fields and/or uniaxial strains.

Subgroup & SI group	Perturbation
P_S1 (1.3) $\mathbb{Z}_1$	• $\boldsymbol{E} \parallel [010]$ and strain in generic direction<br>• $\boldsymbol{E} \perp [010]$ and strain in generic direction<br>• $\boldsymbol{E}$ in generic direction
Cm' (8.34) $\mathbb{Z}_1$	• $\boldsymbol{E} \perp [010]$ and $\boldsymbol{B} \perp [010]$
Cc (9.37) $\mathbb{Z}_1$	• $\boldsymbol{E} \perp [010]$ and $\boldsymbol{B} \parallel [010]$
C_cc (9.40) $\mathbb{Z}_1$	• $\boldsymbol{E} \perp [010]$
$C2'$ (5.15) $\mathbb{Z}_1$	• $\boldsymbol{E} \parallel [010]$ and $\boldsymbol{B} \perp [010]$
$C2$ (5.13) $\mathbb{Z}_1$	• $\boldsymbol{E} \parallel [010]$ and $\boldsymbol{B} \parallel [010]$
C_c2 (5.16) $\mathbb{Z}_1$	• $\boldsymbol{E} \parallel [010]$

Continued on next page

...continued

Subgroup & SI group	Perturbation
$P\bar{1}$ (2.4) $\mathbb{Z}_2 \times \mathbb{Z}_2 \times \mathbb{Z}_2 \times \mathbb{Z}_4$	• $\boldsymbol{B} \parallel [010]$ and strain in generic direction<br>• $\boldsymbol{B} \perp [010]$ and strain in generic direction<br>• $\boldsymbol{B}$ in generic direction
$C2'/m'$ (12.62) $\mathbb{Z}_2 \times \mathbb{Z}_2 \times \mathbb{Z}_4$	• $\boldsymbol{B} \perp [010]$
$C2/c$ (15.85) $\mathbb{Z}_2 \times \mathbb{Z}_2$	• $\boldsymbol{B} \parallel [010]$
$P_S\bar{1}$ (2.7) $\mathbb{Z}_2 \times \mathbb{Z}_2 \times \mathbb{Z}_4$	• strain in generic direction

Table. S 110: Subgroups of C_a2/c (15.91) that can be realized by applying $\boldsymbol{E}/\boldsymbol{B}$ fields and/or uniaxial strains.

Subgroup & SI group	Perturbation
P_S1 (1.3) $\mathbb{Z}_1$	• $\boldsymbol{E} \parallel [010]$ and strain in generic direction<br>• $\boldsymbol{E} \perp [010]$ and strain in generic direction<br>• $\boldsymbol{E}$ in generic direction
Cc' (9.39) $\mathbb{Z}_1$	• $\boldsymbol{E} \perp [010]$ and $\boldsymbol{B} \perp [010]$
Cc (9.37) $\mathbb{Z}_1$	• $\boldsymbol{E} \perp [010]$ and $\boldsymbol{B} \parallel [010]$
C_ac (9.41) $\mathbb{Z}_2$	• $\boldsymbol{E} \perp [010]$
$C2'$ (5.15) $\mathbb{Z}_1$	• $\boldsymbol{E} \parallel [010]$ and $\boldsymbol{B} \perp [010]$
$C2$ (5.13) $\mathbb{Z}_1$	• $\boldsymbol{E} \parallel [010]$ and $\boldsymbol{B} \parallel [010]$
C_a2 (5.17) $\mathbb{Z}_2$	• $\boldsymbol{E} \parallel [010]$
$P\bar{1}$ (2.4) $\mathbb{Z}_2 \times \mathbb{Z}_2 \times \mathbb{Z}_2 \times \mathbb{Z}_4$	• $\boldsymbol{B} \parallel [010]$ and strain in generic direction<br>• $\boldsymbol{B} \perp [010]$ and strain in generic direction<br>• $\boldsymbol{B}$ in generic direction
$C2'/c'$ (15.89) $\mathbb{Z}_2 \times \mathbb{Z}_4$	• $\boldsymbol{B} \perp [010]$
$C2/c$ (15.85) $\mathbb{Z}_2 \times \mathbb{Z}_2$	• $\boldsymbol{B} \parallel [010]$
$P_S\bar{1}$ (2.7) $\mathbb{Z}_2 \times \mathbb{Z}_2 \times \mathbb{Z}_4$	• strain in generic direction

Table. S 111: Subgroups of $P222$ (16.1) that can be realized by applying $\boldsymbol{E}/\boldsymbol{B}$ fields and/or uniaxial strains.

Subgroup & SI group	Perturbation
$P2$ (3.1) $\mathbb{Z}_2$	• strain $\perp$ [100]<br>• $\boldsymbol{B} \parallel$ [100]<br>• $\boldsymbol{E} \parallel$ [100]
$P2$ (3.1) $\mathbb{Z}_2$	• strain $\perp$ [010]<br>• $\boldsymbol{B} \parallel$ [010]<br>• $\boldsymbol{E} \parallel$ [010]
$P2$ (3.1) $\mathbb{Z}_2$	• strain $\perp$ [001]<br>• $\boldsymbol{B} \parallel$ [001]<br>• $\boldsymbol{E} \parallel$ [001]

Table. S 112: Subgroups of $P2221'$ (16.2) that can be realized by applying $\boldsymbol{E}/\boldsymbol{B}$ fields and/or uniaxial strains.

Subgroup & SI group	Perturbation
$P11'$ (1.2) $\mathbb{Z}_1$	• strain in generic direction<br>• $\boldsymbol{E} \parallel$ [100] and strain $\perp$ [010]<br>• $\boldsymbol{E} \parallel$ [100] and strain $\perp$ [001]<br>• $\boldsymbol{E} \parallel$ [010] and strain $\perp$ [100]<br>• $\boldsymbol{E} \parallel$ [010] and strain $\perp$ [001]<br>• $\boldsymbol{E} \parallel$ [001] and strain $\perp$ [100]<br>• $\boldsymbol{E} \parallel$ [001] and strain $\perp$ [010]<br>• $\boldsymbol{E}$ in generic direction
$P2'$ (3.3) $\mathbb{Z}_1$	• $\boldsymbol{B} \parallel$ [010] and strain $\perp$ [100]<br>• $\boldsymbol{B} \parallel$ [001] and strain $\perp$ [100]<br>• $\boldsymbol{B} \perp$ [100]<br>• $\boldsymbol{E} \parallel$ [100] and $\boldsymbol{B} \parallel$ [010]<br>• $\boldsymbol{E} \parallel$ [100] and $\boldsymbol{B} \parallel$ [001]
$P2$ (3.1) $\mathbb{Z}_2$	• $\boldsymbol{B} \parallel$ [100] and strain $\perp$ [100]<br>• $\boldsymbol{E} \parallel$ [100] and $\boldsymbol{B} \parallel$ [100]
$P21'$ (3.2) $\mathbb{Z}_2$	• strain $\perp$ [100]<br>• $\boldsymbol{E} \parallel$ [100]

Continued on next page

...continued

Subgroup & SI group	Perturbation
$P2'$ (3.3) $\mathbb{Z}_1$	• $\boldsymbol{B}$ ∥ [100] and strain ⊥ [010]<br>• $\boldsymbol{B}$ ∥ [001] and strain ⊥ [010]<br>• $\boldsymbol{B}$ ⊥ [010]<br>• $\boldsymbol{E}$ ∥ [010] and $\boldsymbol{B}$ ∥ [100]<br>• $\boldsymbol{E}$ ∥ [010] and $\boldsymbol{B}$ ∥ [001]
$P2$ (3.1) $\mathbb{Z}_2$	• $\boldsymbol{B}$ ∥ [010] and strain ⊥ [010]<br>• $\boldsymbol{E}$ ∥ [010] and $\boldsymbol{B}$ ∥ [010]
$P21'$ (3.2) $\mathbb{Z}_2$	• strain ⊥ [010]<br>• $\boldsymbol{E}$ ∥ [010]
$P2'$ (3.3) $\mathbb{Z}_1$	• $\boldsymbol{B}$ ∥ [100] and strain ⊥ [001]<br>• $\boldsymbol{B}$ ∥ [010] and strain ⊥ [001]<br>• $\boldsymbol{B}$ ⊥ [001]<br>• $\boldsymbol{E}$ ∥ [001] and $\boldsymbol{B}$ ∥ [100]<br>• $\boldsymbol{E}$ ∥ [001] and $\boldsymbol{B}$ ∥ [010]
$P2'2'2$ (16.3) $\mathbb{Z}_2$	• $\boldsymbol{B}$ ∥ [100]
$P2'2'2$ (16.3) $\mathbb{Z}_2$	• $\boldsymbol{B}$ ∥ [010]
$P2$ (3.1) $\mathbb{Z}_2$	• $\boldsymbol{B}$ ∥ [001] and strain ⊥ [001]<br>• $\boldsymbol{E}$ ∥ [001] and $\boldsymbol{B}$ ∥ [001]
$P2'2'2$ (16.3) $\mathbb{Z}_2$	• $\boldsymbol{B}$ ∥ [001]
$P21'$ (3.2) $\mathbb{Z}_2$	• strain ⊥ [001]<br>• $\boldsymbol{E}$ ∥ [001]

Table. S 113: Subgroups of $P2'2'2$ (16.3) that can be realized by applying $\boldsymbol{E}/\boldsymbol{B}$ fields and/or uniaxial strains.

Subgroup & SI group	Perturbation
$P2'$ (3.3) $\mathbb{Z}_1$	• strain ⊥ [100]<br>• ($\boldsymbol{B}$ ∥ [010] or $\boldsymbol{B}$ ⊥ [100])<br>• $\boldsymbol{E}$ ∥ [100]
$P2'$ (3.3) $\mathbb{Z}_1$	• strain ⊥ [010]<br>• ($\boldsymbol{B}$ ∥ [100] or $\boldsymbol{B}$ ⊥ [010])<br>• $\boldsymbol{E}$ ∥ [010]
$P2$ (3.1) $\mathbb{Z}_2$	• strain ⊥ [001]<br>• $\boldsymbol{E}$ ∥ [001]

Table. S 114: Subgroups of P_a222 (16.4) that can be realized by applying $\boldsymbol{E}/\boldsymbol{B}$ fields and/or uniaxial strains.

Subgroup & SI group	Perturbation
$P2$ (3.1) $\mathbb{Z}_2$	• $\boldsymbol{B} \parallel [100]$ and strain $\perp [100]$<br>• $\boldsymbol{E} \parallel [100]$ and $\boldsymbol{B} \parallel [100]$
P_S1 (1.3) $\mathbb{Z}_1$	• strain in generic direction<br>• $\boldsymbol{E} \parallel [100]$ and strain $\perp [010]$<br>• $\boldsymbol{E} \parallel [100]$ and strain $\perp [001]$<br>• $\boldsymbol{E} \parallel [010]$ and strain $\perp [100]$<br>• $\boldsymbol{E} \parallel [010]$ and strain $\perp [001]$<br>• $\boldsymbol{E} \parallel [001]$ and strain $\perp [100]$<br>• $\boldsymbol{E} \parallel [001]$ and strain $\perp [010]$<br>• $\boldsymbol{E}$ in generic direction
$P2_1'$ (4.9) $\mathbb{Z}_1$	• $\boldsymbol{B} \parallel [010]$ and strain $\perp [100]$<br>• $\boldsymbol{B} \parallel [001]$ and strain $\perp [100]$<br>• $\boldsymbol{B} \perp [100]$<br>• $\boldsymbol{E} \parallel [100]$ and $\boldsymbol{B} \parallel [010]$<br>• $\boldsymbol{E} \parallel [100]$ and $\boldsymbol{B} \parallel [001]$
P_b2 (3.5) $\mathbb{Z}_2$	• strain $\perp [100]$<br>• $\boldsymbol{E} \parallel [100]$
$P2$ (3.1) $\mathbb{Z}_2$	• $\boldsymbol{B} \parallel [010]$ and strain $\perp [010]$<br>• $\boldsymbol{E} \parallel [010]$ and $\boldsymbol{B} \parallel [010]$
$P2$ (3.1) $\mathbb{Z}_2$	• $\boldsymbol{B} \parallel [001]$ and strain $\perp [001]$<br>• $\boldsymbol{E} \parallel [001]$ and $\boldsymbol{B} \parallel [001]$
$P2'$ (3.3) $\mathbb{Z}_1$	• $\boldsymbol{B} \parallel [100]$ and strain $\perp [010]$<br>• $\boldsymbol{B} \parallel [001]$ and strain $\perp [010]$<br>• $\boldsymbol{B} \perp [010]$<br>• $\boldsymbol{E} \parallel [010]$ and $\boldsymbol{B} \parallel [100]$<br>• $\boldsymbol{E} \parallel [010]$ and $\boldsymbol{B} \parallel [001]$
P_a2 (3.4) $\mathbb{Z}_2$	• strain $\perp [010]$<br>• $\boldsymbol{E} \parallel [010]$
$P22'2_1'$ (17.10) $\mathbb{Z}_2$	• $\boldsymbol{B} \parallel [001]$
$P2'$ (3.3) $\mathbb{Z}_1$	• $\boldsymbol{B} \parallel [100]$ and strain $\perp [001]$<br>• $\boldsymbol{B} \parallel [010]$ and strain $\perp [001]$<br>• $\boldsymbol{B} \perp [001]$<br>• $\boldsymbol{E} \parallel [001]$ and $\boldsymbol{B} \parallel [100]$<br>• $\boldsymbol{E} \parallel [001]$ and $\boldsymbol{B} \parallel [010]$
$P22'2_1'$ (17.10) $\mathbb{Z}_2$	• $\boldsymbol{B} \parallel [010]$

Continued on next page

. . . continued

Subgroup & SI group	Perturbation
P_a2 (3.4) $\mathbb{Z}_2$	• strain $\perp$ [001]<br>• $\boldsymbol{E}$ $\parallel$ [001]
$P2'2'2$ (16.3) $\mathbb{Z}_2$	• $\boldsymbol{B}$ $\parallel$ [100]

Table. S 115: Subgroups of P_C222 (16.5) that can be realized by applying $\boldsymbol{E}/\boldsymbol{B}$ fields and/or uniaxial strains.

Subgroup & SI group	Perturbation
$P2$ (3.1) $\mathbb{Z}_2$	• $\boldsymbol{B}$ $\parallel$ [100] and strain $\perp$ [100]<br>• $\boldsymbol{E}$ $\parallel$ [100] and $\boldsymbol{B}$ $\parallel$ [100]
P_S1 (1.3) $\mathbb{Z}_1$	• strain in generic direction<br>• $\boldsymbol{E}$ $\parallel$ [100] and strain $\perp$ [010]<br>• $\boldsymbol{E}$ $\parallel$ [100] and strain $\perp$ [001]<br>• $\boldsymbol{E}$ $\parallel$ [010] and strain $\perp$ [100]<br>• $\boldsymbol{E}$ $\parallel$ [010] and strain $\perp$ [001]<br>• $\boldsymbol{E}$ $\parallel$ [001] and strain $\perp$ [100]<br>• $\boldsymbol{E}$ $\parallel$ [001] and strain $\perp$ [010]<br>• $\boldsymbol{E}$ in generic direction
$P2_1'$ (4.9) $\mathbb{Z}_1$	• $\boldsymbol{B}$ $\parallel$ [010] and strain $\perp$ [100]<br>• $\boldsymbol{B}$ $\parallel$ [001] and strain $\perp$ [100]<br>• $\boldsymbol{B}$ $\perp$ [100]<br>• $\boldsymbol{E}$ $\parallel$ [100] and $\boldsymbol{B}$ $\parallel$ [010]<br>• $\boldsymbol{E}$ $\parallel$ [100] and $\boldsymbol{B}$ $\parallel$ [001]
P_C2 (3.6) $\mathbb{Z}_1$	• strain $\perp$ [100]<br>• $\boldsymbol{E}$ $\parallel$ [100]
$P2$ (3.1) $\mathbb{Z}_2$	• $\boldsymbol{B}$ $\parallel$ [010] and strain $\perp$ [010]<br>• $\boldsymbol{E}$ $\parallel$ [010] and $\boldsymbol{B}$ $\parallel$ [010]
$P2$ (3.1) $\mathbb{Z}_2$	• $\boldsymbol{B}$ $\parallel$ [001] and strain $\perp$ [001]<br>• $\boldsymbol{E}$ $\parallel$ [001] and $\boldsymbol{B}$ $\parallel$ [001]
$P2_1'$ (4.9) $\mathbb{Z}_1$	• $\boldsymbol{B}$ $\parallel$ [100] and strain $\perp$ [010]<br>• $\boldsymbol{B}$ $\parallel$ [001] and strain $\perp$ [010]<br>• $\boldsymbol{B}$ $\perp$ [010]<br>• $\boldsymbol{E}$ $\parallel$ [010] and $\boldsymbol{B}$ $\parallel$ [100]<br>• $\boldsymbol{E}$ $\parallel$ [010] and $\boldsymbol{B}$ $\parallel$ [001]

Continued on next page

...continued

Subgroup & SI group	Perturbation
P_C2 (3.6) $\mathbb{Z}_1$	• strain $\perp$ [010]<br>• $\boldsymbol{E}$ $\parallel$ [010]
$P2_1'2_1'2$ (18.18) $\mathbb{Z}_2$	• $\boldsymbol{B}$ $\parallel$ [001]
$P2'$ (3.3) $\mathbb{Z}_1$	• $\boldsymbol{B}$ $\parallel$ [100] and strain $\perp$ [001]<br>• $\boldsymbol{B}$ $\parallel$ [010] and strain $\perp$ [001]<br>• $\boldsymbol{B}$ $\perp$ [001]<br>• $\boldsymbol{E}$ $\parallel$ [001] and $\boldsymbol{B}$ $\parallel$ [100]<br>• $\boldsymbol{E}$ $\parallel$ [001] and $\boldsymbol{B}$ $\parallel$ [010]
$P22'2_1'$ (17.10) $\mathbb{Z}_2$	• $\boldsymbol{B}$ $\parallel$ [010]
P_a2 (3.4) $\mathbb{Z}_2$	• strain $\perp$ [001]<br>• $\boldsymbol{E}$ $\parallel$ [001]
$P22'2_1'$ (17.10) $\mathbb{Z}_2$	• $\boldsymbol{B}$ $\parallel$ [100]

Table. S 116: Subgroups of P_I222 (16.6) that can be realized by applying $\boldsymbol{E}/\boldsymbol{B}$ fields and/or uniaxial strains.

Subgroup & SI group	Perturbation
$P2$ (3.1) $\mathbb{Z}_2$	• $\boldsymbol{B}$ $\parallel$ [100] and strain $\perp$ [100]<br>• $\boldsymbol{E}$ $\parallel$ [100] and $\boldsymbol{B}$ $\parallel$ [100]
P_S1 (1.3) $\mathbb{Z}_1$	• strain in generic direction<br>• $\boldsymbol{E}$ $\parallel$ [100] and strain $\perp$ [010]<br>• $\boldsymbol{E}$ $\parallel$ [100] and strain $\perp$ [001]<br>• $\boldsymbol{E}$ $\parallel$ [010] and strain $\perp$ [100]<br>• $\boldsymbol{E}$ $\parallel$ [010] and strain $\perp$ [001]<br>• $\boldsymbol{E}$ $\parallel$ [001] and strain $\perp$ [100]<br>• $\boldsymbol{E}$ $\parallel$ [001] and strain $\perp$ [010]<br>• $\boldsymbol{E}$ in generic direction
$P2_1'$ (4.9) $\mathbb{Z}_1$	• $\boldsymbol{B}$ $\parallel$ [010] and strain $\perp$ [100]<br>• $\boldsymbol{B}$ $\parallel$ [001] and strain $\perp$ [100]<br>• $\boldsymbol{B}$ $\perp$ [100]<br>• $\boldsymbol{E}$ $\parallel$ [100] and $\boldsymbol{B}$ $\parallel$ [010]<br>• $\boldsymbol{E}$ $\parallel$ [100] and $\boldsymbol{B}$ $\parallel$ [001]
P_C2 (3.6) $\mathbb{Z}_1$	• strain $\perp$ [100]<br>• $\boldsymbol{E}$ $\parallel$ [100]

Continued on next page

…continued

Subgroup & SI group	Perturbation
$P2$ (3.1) $\mathbb{Z}_2$	• $\boldsymbol{B} \parallel [010]$ and strain $\perp [010]$<br>• $\boldsymbol{E} \parallel [010]$ and $\boldsymbol{B} \parallel [010]$
$P2$ (3.1) $\mathbb{Z}_2$	• $\boldsymbol{B} \parallel [001]$ and strain $\perp [001]$<br>• $\boldsymbol{E} \parallel [001]$ and $\boldsymbol{B} \parallel [001]$
$P2_1'$ (4.9) $\mathbb{Z}_1$	• $\boldsymbol{B} \parallel [100]$ and strain $\perp [010]$<br>• $\boldsymbol{B} \parallel [001]$ and strain $\perp [010]$<br>• $\boldsymbol{B} \perp [010]$<br>• $\boldsymbol{E} \parallel [010]$ and $\boldsymbol{B} \parallel [100]$<br>• $\boldsymbol{E} \parallel [010]$ and $\boldsymbol{B} \parallel [001]$
P_C2 (3.6) $\mathbb{Z}_1$	• strain $\perp [010]$<br>• $\boldsymbol{E} \parallel [010]$
$P2_1'2_1'2$ (18.18) $\mathbb{Z}_2$	• $\boldsymbol{B} \parallel [001]$
$P2_1'$ (4.9) $\mathbb{Z}_1$	• $\boldsymbol{B} \parallel [100]$ and strain $\perp [001]$<br>• $\boldsymbol{B} \parallel [010]$ and strain $\perp [001]$<br>• $\boldsymbol{B} \perp [001]$<br>• $\boldsymbol{E} \parallel [001]$ and $\boldsymbol{B} \parallel [100]$<br>• $\boldsymbol{E} \parallel [001]$ and $\boldsymbol{B} \parallel [010]$
$P2_1'2_1'2$ (18.18) $\mathbb{Z}_2$	• $\boldsymbol{B} \parallel [010]$
P_C2 (3.6) $\mathbb{Z}_1$	• strain $\perp [001]$<br>• $\boldsymbol{E} \parallel [001]$
$P2_1'2_1'2$ (18.18) $\mathbb{Z}_2$	• $\boldsymbol{B} \parallel [100]$

Table. S 117: Subgroups of $P22'2_1'$ (17.10) that can be realized by applying $\boldsymbol{E}/\boldsymbol{B}$ fields and/or uniaxial strains.

Subgroup & SI group	Perturbation
$P2$ (3.1) $\mathbb{Z}_2$	• strain $\perp [100]$<br>• $\boldsymbol{E} \parallel [100]$
$P2'$ (3.3) $\mathbb{Z}_1$	• strain $\perp [010]$<br>• ($\boldsymbol{B} \parallel [001]$ or $\boldsymbol{B} \perp [010]$)<br>• $\boldsymbol{E} \parallel [010]$
$P2_1'$ (4.9) $\mathbb{Z}_1$	• strain $\perp [001]$<br>• ($\boldsymbol{B} \parallel [010]$ or $\boldsymbol{B} \perp [001]$)<br>• $\boldsymbol{E} \parallel [001]$

Table. S 118: Subgroups of P_a222_1 (17.11) that can be realized by applying $\boldsymbol{E}/\boldsymbol{B}$ fields and/or uniaxial strains.

Subgroup & SI group	Perturbation
$P2$ (3.1) $\mathbb{Z}_2$	• $\boldsymbol{B} \parallel [100]$ and strain $\perp [100]$<br>• $\boldsymbol{E} \parallel [100]$ and $\boldsymbol{B} \parallel [100]$
P_S1 (1.3) $\mathbb{Z}_1$	• strain in generic direction<br>• $\boldsymbol{E} \parallel [100]$ and strain $\perp [010]$<br>• $\boldsymbol{E} \parallel [100]$ and strain $\perp [001]$<br>• $\boldsymbol{E} \parallel [010]$ and strain $\perp [100]$<br>• $\boldsymbol{E} \parallel [010]$ and strain $\perp [001]$<br>• $\boldsymbol{E} \parallel [001]$ and strain $\perp [100]$<br>• $\boldsymbol{E} \parallel [001]$ and strain $\perp [010]$<br>• $\boldsymbol{E}$ in generic direction
$P2_1'$ (4.9) $\mathbb{Z}_1$	• $\boldsymbol{B} \parallel [010]$ and strain $\perp [100]$<br>• $\boldsymbol{B} \parallel [001]$ and strain $\perp [100]$<br>• $\boldsymbol{B} \perp [100]$<br>• $\boldsymbol{E} \parallel [100]$ and $\boldsymbol{B} \parallel [010]$<br>• $\boldsymbol{E} \parallel [100]$ and $\boldsymbol{B} \parallel [001]$
P_b2 (3.5) $\mathbb{Z}_2$	• strain $\perp [100]$<br>• $\boldsymbol{E} \parallel [100]$
$P2$ (3.1) $\mathbb{Z}_2$	• $\boldsymbol{B} \parallel [010]$ and strain $\perp [010]$<br>• $\boldsymbol{E} \parallel [010]$ and $\boldsymbol{B} \parallel [010]$
$P2_1$ (4.7) $\mathbb{Z}_1$	• $\boldsymbol{B} \parallel [001]$ and strain $\perp [001]$<br>• $\boldsymbol{E} \parallel [001]$ and $\boldsymbol{B} \parallel [001]$
$P2'$ (3.3) $\mathbb{Z}_1$	• $\boldsymbol{B} \parallel [100]$ and strain $\perp [010]$<br>• $\boldsymbol{B} \parallel [001]$ and strain $\perp [010]$<br>• $\boldsymbol{B} \perp [010]$<br>• $\boldsymbol{E} \parallel [010]$ and $\boldsymbol{B} \parallel [100]$<br>• $\boldsymbol{E} \parallel [010]$ and $\boldsymbol{B} \parallel [001]$
P_a2 (3.4) $\mathbb{Z}_2$	• strain $\perp [010]$<br>• $\boldsymbol{E} \parallel [010]$
$P2_12_1'2'$ (18.19) $\mathbb{Z}_1$	• $\boldsymbol{B} \parallel [001]$
$P2_1'$ (4.9) $\mathbb{Z}_1$	• $\boldsymbol{B} \parallel [100]$ and strain $\perp [001]$<br>• $\boldsymbol{B} \parallel [010]$ and strain $\perp [001]$<br>• $\boldsymbol{B} \perp [001]$<br>• $\boldsymbol{E} \parallel [001]$ and $\boldsymbol{B} \parallel [100]$<br>• $\boldsymbol{E} \parallel [001]$ and $\boldsymbol{B} \parallel [010]$
$P2_1'2_1'2$ (18.18) $\mathbb{Z}_2$	• $\boldsymbol{B} \parallel [010]$

Continued on next page

…continued

Subgroup & SI group	Perturbation
P_a2_1 (4.10) $\mathbb{Z}_1$	• strain $\perp$ [001]<br>• $\boldsymbol{E}$ $\parallel$ [001]
$P22'2'_1$ (17.10) $\mathbb{Z}_2$	• $\boldsymbol{B}$ $\parallel$ [100]

Table. S 119: Subgroups of P_c222_1 (17.12) that can be realized by applying $\boldsymbol{E}/\boldsymbol{B}$ fields and/or uniaxial strains.

Subgroup & SI group	Perturbation
P_S1 (1.3) $\mathbb{Z}_1$	• strain in generic direction<br>• $\boldsymbol{E}$ $\parallel$ [100] and strain $\perp$ [010]<br>• $\boldsymbol{E}$ $\parallel$ [100] and strain $\perp$ [001]<br>• $\boldsymbol{E}$ $\parallel$ [010] and strain $\perp$ [100]<br>• $\boldsymbol{E}$ $\parallel$ [010] and strain $\perp$ [001]<br>• $\boldsymbol{E}$ $\parallel$ [001] and strain $\perp$ [100]<br>• $\boldsymbol{E}$ $\parallel$ [001] and strain $\perp$ [010]<br>• $\boldsymbol{E}$ in generic direction
$P2$ (3.1) $\mathbb{Z}_2$	• $\boldsymbol{B}$ $\parallel$ [100] and strain $\perp$ [100]<br>• $\boldsymbol{E}$ $\parallel$ [100] and $\boldsymbol{B}$ $\parallel$ [100]
$P2'$ (3.3) $\mathbb{Z}_1$	• $\boldsymbol{B}$ $\parallel$ [010] and strain $\perp$ [100]<br>• $\boldsymbol{B}$ $\parallel$ [001] and strain $\perp$ [100]<br>• $\boldsymbol{B}$ $\perp$ [100]<br>• $\boldsymbol{E}$ $\parallel$ [100] and $\boldsymbol{B}$ $\parallel$ [010]<br>• $\boldsymbol{E}$ $\parallel$ [100] and $\boldsymbol{B}$ $\parallel$ [001]
P_a2 (3.4) $\mathbb{Z}_2$	• strain $\perp$ [100]<br>• $\boldsymbol{E}$ $\parallel$ [100]
$P2'$ (3.3) $\mathbb{Z}_1$	• $\boldsymbol{B}$ $\parallel$ [100] and strain $\perp$ [010]<br>• $\boldsymbol{B}$ $\parallel$ [001] and strain $\perp$ [010]<br>• $\boldsymbol{B}$ $\perp$ [010]<br>• $\boldsymbol{E}$ $\parallel$ [010] and $\boldsymbol{B}$ $\parallel$ [100]<br>• $\boldsymbol{E}$ $\parallel$ [010] and $\boldsymbol{B}$ $\parallel$ [001]
$P2$ (3.1) $\mathbb{Z}_2$	• $\boldsymbol{B}$ $\parallel$ [010] and strain $\perp$ [010]<br>• $\boldsymbol{E}$ $\parallel$ [010] and $\boldsymbol{B}$ $\parallel$ [010]
P_a2 (3.4) $\mathbb{Z}_2$	• strain $\perp$ [010]<br>• $\boldsymbol{E}$ $\parallel$ [010]

Continued on next page

…continued

Subgroup & SI group	Perturbation
$P2'$ (3.3) $\mathbb{Z}_1$	• $\boldsymbol{B} \parallel [100]$ and strain $\perp [001]$<br>• $\boldsymbol{B} \parallel [010]$ and strain $\perp [001]$<br>• $\boldsymbol{B} \perp [001]$<br>• $\boldsymbol{E} \parallel [001]$ and $\boldsymbol{B} \parallel [100]$<br>• $\boldsymbol{E} \parallel [001]$ and $\boldsymbol{B} \parallel [010]$
$P2'2'2$ (16.3) $\mathbb{Z}_2$	• $\boldsymbol{B} \parallel [100]$
$P2'2'2$ (16.3) $\mathbb{Z}_2$	• $\boldsymbol{B} \parallel [010]$
$P2_1$ (4.7) $\mathbb{Z}_1$	• $\boldsymbol{B} \parallel [001]$ and strain $\perp [001]$<br>• $\boldsymbol{E} \parallel [001]$ and $\boldsymbol{B} \parallel [001]$
$P2'2'2_1$ (17.9) $\mathbb{Z}_1$	• $\boldsymbol{B} \parallel [001]$
P_b2_1 (4.11) $\mathbb{Z}_1$	• strain $\perp [001]$<br>• $\boldsymbol{E} \parallel [001]$

Table. S 120: Subgroups of P_B222_1 (17.13) that can be realized by applying $\boldsymbol{E}/\boldsymbol{B}$ fields and/or uniaxial strains.

Subgroup & SI group	Perturbation
$P2$ (3.1) $\mathbb{Z}_2$	• $\boldsymbol{B} \parallel [100]$ and strain $\perp [100]$<br>• $\boldsymbol{E} \parallel [100]$ and $\boldsymbol{B} \parallel [100]$
P_S1 (1.3) $\mathbb{Z}_1$	• strain in generic direction<br>• $\boldsymbol{E} \parallel [100]$ and strain $\perp [010]$<br>• $\boldsymbol{E} \parallel [100]$ and strain $\perp [001]$<br>• $\boldsymbol{E} \parallel [010]$ and strain $\perp [100]$<br>• $\boldsymbol{E} \parallel [010]$ and strain $\perp [001]$<br>• $\boldsymbol{E} \parallel [001]$ and strain $\perp [100]$<br>• $\boldsymbol{E} \parallel [001]$ and strain $\perp [010]$<br>• $\boldsymbol{E}$ in generic direction
$P2_1'$ (4.9) $\mathbb{Z}_1$	• $\boldsymbol{B} \parallel [010]$ and strain $\perp [100]$<br>• $\boldsymbol{B} \parallel [001]$ and strain $\perp [100]$<br>• $\boldsymbol{B} \perp [100]$<br>• $\boldsymbol{E} \parallel [100]$ and $\boldsymbol{B} \parallel [010]$<br>• $\boldsymbol{E} \parallel [100]$ and $\boldsymbol{B} \parallel [001]$
P_C2 (3.6) $\mathbb{Z}_1$	• strain $\perp [100]$<br>• $\boldsymbol{E} \parallel [100]$

Continued on next page

. . . continued

Subgroup & SI group	Perturbation
$P2$ (3.1) $\mathbb{Z}_2$	• $\boldsymbol{B} \parallel [010]$ and strain $\perp [010]$<br>• $\boldsymbol{E} \parallel [010]$ and $\boldsymbol{B} \parallel [010]$
$P2_1$ (4.7) $\mathbb{Z}_1$	• $\boldsymbol{B} \parallel [001]$ and strain $\perp [001]$<br>• $\boldsymbol{E} \parallel [001]$ and $\boldsymbol{B} \parallel [001]$
$P2'$ (3.3) $\mathbb{Z}_1$	• $\boldsymbol{B} \parallel [100]$ and strain $\perp [010]$<br>• $\boldsymbol{B} \parallel [001]$ and strain $\perp [010]$<br>• $\boldsymbol{B} \perp [010]$<br>• $\boldsymbol{E} \parallel [010]$ and $\boldsymbol{B} \parallel [100]$<br>• $\boldsymbol{E} \parallel [010]$ and $\boldsymbol{B} \parallel [001]$
P_a2 (3.4) $\mathbb{Z}_2$	• strain $\perp [010]$<br>• $\boldsymbol{E} \parallel [010]$
$P2_12_1'2'$ (18.19) $\mathbb{Z}_1$	• $\boldsymbol{B} \parallel [001]$
$P2'$ (3.3) $\mathbb{Z}_1$	• $\boldsymbol{B} \parallel [100]$ and strain $\perp [001]$<br>• $\boldsymbol{B} \parallel [010]$ and strain $\perp [001]$<br>• $\boldsymbol{B} \perp [001]$<br>• $\boldsymbol{E} \parallel [001]$ and $\boldsymbol{B} \parallel [100]$<br>• $\boldsymbol{E} \parallel [001]$ and $\boldsymbol{B} \parallel [010]$
$P22'2_1'$ (17.10) $\mathbb{Z}_2$	• $\boldsymbol{B} \parallel [010]$
P_C2_1 (4.12) $\mathbb{Z}_1$	• strain $\perp [001]$<br>• $\boldsymbol{E} \parallel [001]$
$P2'2'2$ (16.3) $\mathbb{Z}_2$	• $\boldsymbol{B} \parallel [100]$

Table. S 121: Subgroups of P_C222_1 (17.14) that can be realized by applying $\boldsymbol{E}/\boldsymbol{B}$ fields and/or uniaxial strains.

Subgroup & SI group	Perturbation
$P2$ (3.1) $\mathbb{Z}_2$	• $\boldsymbol{B} \parallel [100]$ and strain $\perp [100]$<br>• $\boldsymbol{E} \parallel [100]$ and $\boldsymbol{B} \parallel [100]$

Continued on next page

...continued

Subgroup & SI group	Perturbation
P_S1 (1.3) $\mathbb{Z}_1$	• strain in generic direction<br>• $\boldsymbol{E} \parallel [100]$ and strain $\perp [010]$<br>• $\boldsymbol{E} \parallel [100]$ and strain $\perp [001]$<br>• $\boldsymbol{E} \parallel [010]$ and strain $\perp [100]$<br>• $\boldsymbol{E} \parallel [010]$ and strain $\perp [001]$<br>• $\boldsymbol{E} \parallel [001]$ and strain $\perp [100]$<br>• $\boldsymbol{E} \parallel [001]$ and strain $\perp [010]$<br>• $\boldsymbol{E}$ in generic direction
$P2_1'$ (4.9) $\mathbb{Z}_1$	• $\boldsymbol{B} \parallel [010]$ and strain $\perp [100]$<br>• $\boldsymbol{B} \parallel [001]$ and strain $\perp [100]$<br>• $\boldsymbol{B} \perp [100]$<br>• $\boldsymbol{E} \parallel [100]$ and $\boldsymbol{B} \parallel [010]$<br>• $\boldsymbol{E} \parallel [100]$ and $\boldsymbol{B} \parallel [001]$
P_C2 (3.6) $\mathbb{Z}_1$	• strain $\perp [100]$<br>• $\boldsymbol{E} \parallel [100]$
$P2$ (3.1) $\mathbb{Z}_2$	• $\boldsymbol{B} \parallel [010]$ and strain $\perp [010]$<br>• $\boldsymbol{E} \parallel [010]$ and $\boldsymbol{B} \parallel [010]$
$P2_1$ (4.7) $\mathbb{Z}_1$	• $\boldsymbol{B} \parallel [001]$ and strain $\perp [001]$<br>• $\boldsymbol{E} \parallel [001]$ and $\boldsymbol{B} \parallel [001]$
$P2_1'$ (4.9) $\mathbb{Z}_1$	• $\boldsymbol{B} \parallel [100]$ and strain $\perp [010]$<br>• $\boldsymbol{B} \parallel [001]$ and strain $\perp [010]$<br>• $\boldsymbol{B} \perp [010]$<br>• $\boldsymbol{E} \parallel [010]$ and $\boldsymbol{B} \parallel [100]$<br>• $\boldsymbol{E} \parallel [010]$ and $\boldsymbol{B} \parallel [001]$
P_C2 (3.6) $\mathbb{Z}_1$	• strain $\perp [010]$<br>• $\boldsymbol{E} \parallel [010]$
$P2_1'2_1'2_1$ (19.27) $\mathbb{Z}_1$	• $\boldsymbol{B} \parallel [001]$
$P2_1'$ (4.9) $\mathbb{Z}_1$	• $\boldsymbol{B} \parallel [100]$ and strain $\perp [001]$<br>• $\boldsymbol{B} \parallel [010]$ and strain $\perp [001]$<br>• $\boldsymbol{B} \perp [001]$<br>• $\boldsymbol{E} \parallel [001]$ and $\boldsymbol{B} \parallel [100]$<br>• $\boldsymbol{E} \parallel [001]$ and $\boldsymbol{B} \parallel [010]$
$P2_1'2_1'2$ (18.18) $\mathbb{Z}_2$	• $\boldsymbol{B} \parallel [010]$
P_a2_1 (4.10) $\mathbb{Z}_1$	• strain $\perp [001]$<br>• $\boldsymbol{E} \parallel [001]$
$P2_1'2_1'2$ (18.18) $\mathbb{Z}_2$	• $\boldsymbol{B} \parallel [100]$

Table. S 122: Subgroups of $P_I 222_1$ (17.15) that can be realized by applying $\boldsymbol{E}/\boldsymbol{B}$ fields and/or uniaxial strains.

Subgroup & SI group	Perturbation
$P2$ (3.1) $\mathbb{Z}_2$	• $\boldsymbol{B} \parallel [100]$ and strain $\perp [100]$<br>• $\boldsymbol{E} \parallel [100]$ and $\boldsymbol{B} \parallel [100]$
P_S1 (1.3) $\mathbb{Z}_1$	• strain in generic direction<br>• $\boldsymbol{E} \parallel [100]$ and strain $\perp [010]$<br>• $\boldsymbol{E} \parallel [100]$ and strain $\perp [001]$<br>• $\boldsymbol{E} \parallel [010]$ and strain $\perp [100]$<br>• $\boldsymbol{E} \parallel [010]$ and strain $\perp [001]$<br>• $\boldsymbol{E} \parallel [001]$ and strain $\perp [100]$<br>• $\boldsymbol{E} \parallel [001]$ and strain $\perp [010]$<br>• $\boldsymbol{E}$ in generic direction
$P2_1'$ (4.9) $\mathbb{Z}_1$	• $\boldsymbol{B} \parallel [010]$ and strain $\perp [100]$<br>• $\boldsymbol{B} \parallel [001]$ and strain $\perp [100]$<br>• $\boldsymbol{B} \perp [100]$<br>• $\boldsymbol{E} \parallel [100]$ and $\boldsymbol{B} \parallel [010]$<br>• $\boldsymbol{E} \parallel [100]$ and $\boldsymbol{B} \parallel [001]$
P_C2 (3.6) $\mathbb{Z}_1$	• strain $\perp [100]$<br>• $\boldsymbol{E} \parallel [100]$
$P2$ (3.1) $\mathbb{Z}_2$	• $\boldsymbol{B} \parallel [010]$ and strain $\perp [010]$<br>• $\boldsymbol{E} \parallel [010]$ and $\boldsymbol{B} \parallel [010]$
$P2_1$ (4.7) $\mathbb{Z}_1$	• $\boldsymbol{B} \parallel [001]$ and strain $\perp [001]$<br>• $\boldsymbol{E} \parallel [001]$ and $\boldsymbol{B} \parallel [001]$
$P2_1'$ (4.9) $\mathbb{Z}_1$	• $\boldsymbol{B} \parallel [100]$ and strain $\perp [010]$<br>• $\boldsymbol{B} \parallel [001]$ and strain $\perp [010]$<br>• $\boldsymbol{B} \perp [010]$<br>• $\boldsymbol{E} \parallel [010]$ and $\boldsymbol{B} \parallel [100]$<br>• $\boldsymbol{E} \parallel [010]$ and $\boldsymbol{B} \parallel [001]$
P_C2 (3.6) $\mathbb{Z}_1$	• strain $\perp [010]$<br>• $\boldsymbol{E} \parallel [010]$
$P2_1'2_1'2_1$ (19.27) $\mathbb{Z}_1$	• $\boldsymbol{B} \parallel [001]$

Continued on next page

. . . continued

Subgroup & SI group	Perturbation
$P2'$ (3.3) $\mathbb{Z}_1$	• $\boldsymbol{B}$ ∥ [100] and strain ⊥ [001]<br>• $\boldsymbol{B}$ ∥ [010] and strain ⊥ [001]<br>• $\boldsymbol{B}$ ⊥ [001]<br>• $\boldsymbol{E}$ ∥ [001] and $\boldsymbol{B}$ ∥ [100]<br>• $\boldsymbol{E}$ ∥ [001] and $\boldsymbol{B}$ ∥ [010]
$P22'2_1'$ (17.10) $\mathbb{Z}_2$	• $\boldsymbol{B}$ ∥ [010]
P_C2_1 (4.12) $\mathbb{Z}_1$	• strain ⊥ [001]<br>• $\boldsymbol{E}$ ∥ [001]
$P22'2_1'$ (17.10) $\mathbb{Z}_2$	• $\boldsymbol{B}$ ∥ [100]

Table. S 123: Subgroups of $P222_1$ (17.7) that can be realized by applying $\boldsymbol{E}/\boldsymbol{B}$ fields and/or uniaxial strains.

Subgroup & SI group	Perturbation
$P2$ (3.1) $\mathbb{Z}_2$	• strain ⊥ [100]<br>• $\boldsymbol{B}$ ∥ [100]<br>• $\boldsymbol{E}$ ∥ [100]
$P2$ (3.1) $\mathbb{Z}_2$	• strain ⊥ [010]<br>• $\boldsymbol{B}$ ∥ [010]<br>• $\boldsymbol{E}$ ∥ [010]
$P2_1$ (4.7) $\mathbb{Z}_1$	• strain ⊥ [001]<br>• $\boldsymbol{B}$ ∥ [001]<br>• $\boldsymbol{E}$ ∥ [001]

Table. S 124: Subgroups of $P222_11'$ (17.8) that can be realized by applying $\boldsymbol{E}/\boldsymbol{B}$ fields and/or uniaxial strains.

Subgroup & SI group	Perturbation

Continued on next page

. . . continued

Subgroup & SI group	Perturbation
$P11'$ (1.2) $\mathbb{Z}_1$	• strain in generic direction<br>• $\boldsymbol{E}$ || [100] and strain $\perp$ [010]<br>• $\boldsymbol{E}$ || [100] and strain $\perp$ [001]<br>• $\boldsymbol{E}$ || [010] and strain $\perp$ [100]<br>• $\boldsymbol{E}$ || [010] and strain $\perp$ [001]<br>• $\boldsymbol{E}$ || [001] and strain $\perp$ [100]<br>• $\boldsymbol{E}$ || [001] and strain $\perp$ [010]<br>• $\boldsymbol{E}$ in generic direction
$P2'$ (3.3) $\mathbb{Z}_1$	• $\boldsymbol{B}$ || [010] and strain $\perp$ [100]<br>• $\boldsymbol{B}$ || [001] and strain $\perp$ [100]<br>• $\boldsymbol{B}$ $\perp$ [100]<br>• $\boldsymbol{E}$ || [100] and $\boldsymbol{B}$ || [010]<br>• $\boldsymbol{E}$ || [100] and $\boldsymbol{B}$ || [001]
$P2$ (3.1) $\mathbb{Z}_2$	• $\boldsymbol{B}$ || [100] and strain $\perp$ [100]<br>• $\boldsymbol{E}$ || [100] and $\boldsymbol{B}$ || [100]
$P21'$ (3.2) $\mathbb{Z}_2$	• strain $\perp$ [100]<br>• $\boldsymbol{E}$ || [100]
$P2'$ (3.3) $\mathbb{Z}_1$	• $\boldsymbol{B}$ || [100] and strain $\perp$ [010]<br>• $\boldsymbol{B}$ || [001] and strain $\perp$ [010]<br>• $\boldsymbol{B}$ $\perp$ [010]<br>• $\boldsymbol{E}$ || [010] and $\boldsymbol{B}$ || [100]<br>• $\boldsymbol{E}$ || [010] and $\boldsymbol{B}$ || [001]
$P2$ (3.1) $\mathbb{Z}_2$	• $\boldsymbol{B}$ || [010] and strain $\perp$ [010]<br>• $\boldsymbol{E}$ || [010] and $\boldsymbol{B}$ || [010]
$P21'$ (3.2) $\mathbb{Z}_2$	• strain $\perp$ [010]<br>• $\boldsymbol{E}$ || [010]
$P2_1'$ (4.9) $\mathbb{Z}_1$	• $\boldsymbol{B}$ || [100] and strain $\perp$ [001]<br>• $\boldsymbol{B}$ || [010] and strain $\perp$ [001]<br>• $\boldsymbol{B}$ $\perp$ [001]<br>• $\boldsymbol{E}$ || [001] and $\boldsymbol{B}$ || [100]<br>• $\boldsymbol{E}$ || [001] and $\boldsymbol{B}$ || [010]
$P22'2_1'$ (17.10) $\mathbb{Z}_2$	• $\boldsymbol{B}$ || [100]
$P22'2_1'$ (17.10) $\mathbb{Z}_2$	• $\boldsymbol{B}$ || [010]
$P2_1$ (4.7) $\mathbb{Z}_1$	• $\boldsymbol{B}$ || [001] and strain $\perp$ [001]<br>• $\boldsymbol{E}$ || [001] and $\boldsymbol{B}$ || [001]
$P2'2'2_1$ (17.9) $\mathbb{Z}_1$	• $\boldsymbol{B}$ || [001]

Continued on next page

...continued

Subgroup & SI group	Perturbation
$P2_11'$ (4.8) $\mathbb{Z}_1$	• strain $\perp$ [001]<br>• $\boldsymbol{E} \parallel$ [001]

Table. S 125: Subgroups of $P2'2'2_1$ (17.9) that can be realized by applying $\boldsymbol{E}/\boldsymbol{B}$ fields and/or uniaxial strains.

Subgroup & SI group	Perturbation
$P2'$ (3.3) $\mathbb{Z}_1$	• strain $\perp$ [100]<br>• ($\boldsymbol{B} \parallel$ [010] or $\boldsymbol{B} \perp$ [100])<br>• $\boldsymbol{E} \parallel$ [100]
$P2'$ (3.3) $\mathbb{Z}_1$	• strain $\perp$ [010]<br>• ($\boldsymbol{B} \parallel$ [100] or $\boldsymbol{B} \perp$ [010])<br>• $\boldsymbol{E} \parallel$ [010]
$P2_1$ (4.7) $\mathbb{Z}_1$	• strain $\perp$ [001]<br>• $\boldsymbol{E} \parallel$ [001]

Table. S 126: Subgroups of $P2_12_12$ (18.16) that can be realized by applying $\boldsymbol{E}/\boldsymbol{B}$ fields and/or uniaxial strains.

Subgroup & SI group	Perturbation
$P2_1$ (4.7) $\mathbb{Z}_1$	• strain $\perp$ [100]<br>• $\boldsymbol{B} \parallel$ [100]<br>• $\boldsymbol{E} \parallel$ [100]
$P2$ (3.1) $\mathbb{Z}_2$	• strain $\perp$ [001]<br>• $\boldsymbol{B} \parallel$ [001]<br>• $\boldsymbol{E} \parallel$ [001]
$P2_1$ (4.7) $\mathbb{Z}_1$	• strain $\perp$ [010]<br>• $\boldsymbol{B} \parallel$ [010]<br>• $\boldsymbol{E} \parallel$ [010]

Table. S 127: Subgroups of $P2_12_121'$ (18.17) that can be realized by applying $\boldsymbol{E}/\boldsymbol{B}$ fields and/or uniaxial strains.

Subgroup & SI group	Perturbation
$P11'$ (1.2) $\mathbb{Z}_1$	• strain in generic direction<br>• $\boldsymbol{E} \parallel [100]$ and strain $\perp [010]$<br>• $\boldsymbol{E} \parallel [100]$ and strain $\perp [001]$<br>• $\boldsymbol{E} \parallel [010]$ and strain $\perp [100]$<br>• $\boldsymbol{E} \parallel [010]$ and strain $\perp [001]$<br>• $\boldsymbol{E} \parallel [001]$ and strain $\perp [100]$<br>• $\boldsymbol{E} \parallel [001]$ and strain $\perp [010]$<br>• $\boldsymbol{E}$ in generic direction
$P2_1'$ (4.9) $\mathbb{Z}_1$	• $\boldsymbol{B} \parallel [010]$ and strain $\perp [100]$<br>• $\boldsymbol{B} \parallel [001]$ and strain $\perp [100]$<br>• $\boldsymbol{B} \perp [100]$<br>• $\boldsymbol{E} \parallel [100]$ and $\boldsymbol{B} \parallel [010]$<br>• $\boldsymbol{E} \parallel [100]$ and $\boldsymbol{B} \parallel [001]$
$P2_1$ (4.7) $\mathbb{Z}_1$	• $\boldsymbol{B} \parallel [100]$ and strain $\perp [100]$<br>• $\boldsymbol{E} \parallel [100]$ and $\boldsymbol{B} \parallel [100]$
$P2_11'$ (4.8) $\mathbb{Z}_1$	• strain $\perp [100]$<br>• $\boldsymbol{E} \parallel [100]$
$P2'$ (3.3) $\mathbb{Z}_1$	• $\boldsymbol{B} \parallel [100]$ and strain $\perp [001]$<br>• $\boldsymbol{B} \parallel [010]$ and strain $\perp [001]$<br>• $\boldsymbol{B} \perp [001]$<br>• $\boldsymbol{E} \parallel [001]$ and $\boldsymbol{B} \parallel [100]$<br>• $\boldsymbol{E} \parallel [001]$ and $\boldsymbol{B} \parallel [010]$
$P2$ (3.1) $\mathbb{Z}_2$	• $\boldsymbol{B} \parallel [001]$ and strain $\perp [001]$<br>• $\boldsymbol{E} \parallel [001]$ and $\boldsymbol{B} \parallel [001]$
$P21'$ (3.2) $\mathbb{Z}_2$	• strain $\perp [001]$<br>• $\boldsymbol{E} \parallel [001]$
$P2_1'$ (4.9) $\mathbb{Z}_1$	• $\boldsymbol{B} \parallel [100]$ and strain $\perp [010]$<br>• $\boldsymbol{B} \parallel [001]$ and strain $\perp [010]$<br>• $\boldsymbol{B} \perp [010]$<br>• $\boldsymbol{E} \parallel [010]$ and $\boldsymbol{B} \parallel [100]$<br>• $\boldsymbol{E} \parallel [010]$ and $\boldsymbol{B} \parallel [001]$
$P2_12_1'2'$ (18.19) $\mathbb{Z}_1$	• $\boldsymbol{B} \parallel [100]$
$P2_1'2_1'2$ (18.18) $\mathbb{Z}_2$	• $\boldsymbol{B} \parallel [001]$
$P2_1$ (4.7) $\mathbb{Z}_1$	• $\boldsymbol{B} \parallel [010]$ and strain $\perp [010]$<br>• $\boldsymbol{E} \parallel [010]$ and $\boldsymbol{B} \parallel [010]$

Continued on next page

...continued

Subgroup & SI group	Perturbation
$P2_12_1'2'$ (18.19) $\mathbb{Z}_1$	• $\boldsymbol{B} \parallel [010]$
$P2_11'$ (4.8) $\mathbb{Z}_1$	• strain $\perp [010]$<br>• $\boldsymbol{E} \parallel [010]$

Table. S 128: Subgroups of $P2_1'2_1'2$ (18.18) that can be realized by applying $\boldsymbol{E}/\boldsymbol{B}$ fields and/or uniaxial strains.

Subgroup & SI group	Perturbation
$P2_1'$ (4.9) $\mathbb{Z}_1$	• strain $\perp [100]$<br>• ($\boldsymbol{B} \parallel [010]$ or $\boldsymbol{B} \perp [100]$)<br>• $\boldsymbol{E} \parallel [100]$
$P2$ (3.1) $\mathbb{Z}_2$	• strain $\perp [001]$<br>• $\boldsymbol{E} \parallel [001]$
$P2_1'$ (4.9) $\mathbb{Z}_1$	• strain $\perp [010]$<br>• ($\boldsymbol{B} \parallel [100]$ or $\boldsymbol{B} \perp [010]$)<br>• $\boldsymbol{E} \parallel [010]$

Table. S 129: Subgroups of $P2_12_1'2'$ (18.19) that can be realized by applying $\boldsymbol{E}/\boldsymbol{B}$ fields and/or uniaxial strains.

Subgroup & SI group	Perturbation
$P2_1$ (4.7) $\mathbb{Z}_1$	• strain $\perp [100]$<br>• $\boldsymbol{E} \parallel [100]$
$P2'$ (3.3) $\mathbb{Z}_1$	• strain $\perp [001]$<br>• ($\boldsymbol{B} \parallel [010]$ or $\boldsymbol{B} \perp [001]$)<br>• $\boldsymbol{E} \parallel [001]$
$P2_1'$ (4.9) $\mathbb{Z}_1$	• strain $\perp [010]$<br>• ($\boldsymbol{B} \parallel [001]$ or $\boldsymbol{B} \perp [010]$)<br>• $\boldsymbol{E} \parallel [010]$

Table. S 130: Subgroups of $P_b2_12_12$ (18.20) that can be realized by applying $\boldsymbol{E}/\boldsymbol{B}$ fields and/or uniaxial strains.

Subgroup & SI group	Perturbation
P_S1 (1.3) $\mathbb{Z}_1$	• strain in generic direction<br>• $\boldsymbol{E} \parallel [100]$ and strain $\perp [010]$<br>• $\boldsymbol{E} \parallel [100]$ and strain $\perp [001]$<br>• $\boldsymbol{E} \parallel [010]$ and strain $\perp [100]$<br>• $\boldsymbol{E} \parallel [010]$ and strain $\perp [001]$<br>• $\boldsymbol{E} \parallel [001]$ and strain $\perp [100]$<br>• $\boldsymbol{E} \parallel [001]$ and strain $\perp [010]$<br>• $\boldsymbol{E}$ in generic direction
$P2_1'$ (4.9) $\mathbb{Z}_1$	• $\boldsymbol{B} \parallel [010]$ and strain $\perp [100]$<br>• $\boldsymbol{B} \parallel [001]$ and strain $\perp [100]$<br>• $\boldsymbol{B} \perp [100]$<br>• $\boldsymbol{E} \parallel [100]$ and $\boldsymbol{B} \parallel [010]$<br>• $\boldsymbol{E} \parallel [100]$ and $\boldsymbol{B} \parallel [001]$
$P2_1$ (4.7) $\mathbb{Z}_1$	• $\boldsymbol{B} \parallel [100]$ and strain $\perp [100]$<br>• $\boldsymbol{E} \parallel [100]$ and $\boldsymbol{B} \parallel [100]$
P_a2_1 (4.10) $\mathbb{Z}_1$	• strain $\perp [100]$<br>• $\boldsymbol{E} \parallel [100]$
$P2$ (3.1) $\mathbb{Z}_2$	• $\boldsymbol{B} \parallel [001]$ and strain $\perp [001]$<br>• $\boldsymbol{E} \parallel [001]$ and $\boldsymbol{B} \parallel [001]$
$P2'$ (3.3) $\mathbb{Z}_1$	• $\boldsymbol{B} \parallel [100]$ and strain $\perp [001]$<br>• $\boldsymbol{B} \parallel [010]$ and strain $\perp [001]$<br>• $\boldsymbol{B} \perp [001]$<br>• $\boldsymbol{E} \parallel [001]$ and $\boldsymbol{B} \parallel [100]$<br>• $\boldsymbol{E} \parallel [001]$ and $\boldsymbol{B} \parallel [010]$
P_a2 (3.4) $\mathbb{Z}_2$	• strain $\perp [001]$<br>• $\boldsymbol{E} \parallel [001]$
$P2'$ (3.3) $\mathbb{Z}_1$	• $\boldsymbol{B} \parallel [100]$ and strain $\perp [010]$<br>• $\boldsymbol{B} \parallel [001]$ and strain $\perp [010]$<br>• $\boldsymbol{B} \perp [010]$<br>• $\boldsymbol{E} \parallel [010]$ and $\boldsymbol{B} \parallel [100]$<br>• $\boldsymbol{E} \parallel [010]$ and $\boldsymbol{B} \parallel [001]$
$P22'2_1'$ (17.10) $\mathbb{Z}_2$	• $\boldsymbol{B} \parallel [001]$
$P2'2'2_1$ (17.9) $\mathbb{Z}_1$	• $\boldsymbol{B} \parallel [100]$
$P2_1$ (4.7) $\mathbb{Z}_1$	• $\boldsymbol{B} \parallel [010]$ and strain $\perp [010]$<br>• $\boldsymbol{E} \parallel [010]$ and $\boldsymbol{B} \parallel [010]$

Continued on next page

...continued

Subgroup & SI group	Perturbation
$P2_12_1'2'$ (18.19) $\mathbb{Z}_1$	• $\boldsymbol{B}$ $\parallel$ [010]
P_b2_1 (4.11) $\mathbb{Z}_1$	• strain $\perp$ [010]<br>• $\boldsymbol{E}$ $\parallel$ [010]

Table. S 131: Subgroups of $P_c2_12_12$ (18.21) that can be realized by applying $\boldsymbol{E}/\boldsymbol{B}$ fields and/or uniaxial strains.

Subgroup & SI group	Perturbation
P_S1 (1.3) $\mathbb{Z}_1$	• strain in generic direction<br>• $\boldsymbol{E}$ $\parallel$ [100] and strain $\perp$ [010]<br>• $\boldsymbol{E}$ $\parallel$ [100] and strain $\perp$ [001]<br>• $\boldsymbol{E}$ $\parallel$ [010] and strain $\perp$ [100]<br>• $\boldsymbol{E}$ $\parallel$ [010] and strain $\perp$ [001]<br>• $\boldsymbol{E}$ $\parallel$ [001] and strain $\perp$ [100]<br>• $\boldsymbol{E}$ $\parallel$ [001] and strain $\perp$ [010]<br>• $\boldsymbol{E}$ in generic direction
$P2_1$ (4.7) $\mathbb{Z}_1$	• $\boldsymbol{B}$ $\parallel$ [100] and strain $\perp$ [100]<br>• $\boldsymbol{E}$ $\parallel$ [100] and $\boldsymbol{B}$ $\parallel$ [100]
$P2_1'$ (4.9) $\mathbb{Z}_1$	• $\boldsymbol{B}$ $\parallel$ [010] and strain $\perp$ [100]<br>• $\boldsymbol{B}$ $\parallel$ [001] and strain $\perp$ [100]<br>• $\boldsymbol{B}$ $\perp$ [100]<br>• $\boldsymbol{E}$ $\parallel$ [100] and $\boldsymbol{B}$ $\parallel$ [010]<br>• $\boldsymbol{E}$ $\parallel$ [100] and $\boldsymbol{B}$ $\parallel$ [001]
P_a2_1 (4.10) $\mathbb{Z}_1$	• strain $\perp$ [100]<br>• $\boldsymbol{E}$ $\parallel$ [100]
$P2$ (3.1) $\mathbb{Z}_2$	• $\boldsymbol{B}$ $\parallel$ [001] and strain $\perp$ [001]<br>• $\boldsymbol{E}$ $\parallel$ [001] and $\boldsymbol{B}$ $\parallel$ [001]
$P2_1'$ (4.9) $\mathbb{Z}_1$	• $\boldsymbol{B}$ $\parallel$ [100] and strain $\perp$ [001]<br>• $\boldsymbol{B}$ $\parallel$ [010] and strain $\perp$ [001]<br>• $\boldsymbol{B}$ $\perp$ [001]<br>• $\boldsymbol{E}$ $\parallel$ [001] and $\boldsymbol{B}$ $\parallel$ [100]<br>• $\boldsymbol{E}$ $\parallel$ [001] and $\boldsymbol{B}$ $\parallel$ [010]
P_b2 (3.5) $\mathbb{Z}_2$	• strain $\perp$ [001]<br>• $\boldsymbol{E}$ $\parallel$ [001]

Continued on next page

…continued

Subgroup & SI group	Perturbation
$P2_1$ (4.7) $\mathbb{Z}_1$	• $\boldsymbol{B}$ ∥ [010] and strain ⊥ [010]<br>• $\boldsymbol{E}$ ∥ [010] and $\boldsymbol{B}$ ∥ [010]
$P2_1'2_1'2_1$ (19.27) $\mathbb{Z}_1$	• $\boldsymbol{B}$ ∥ [010]
$P2_1'$ (4.9) $\mathbb{Z}_1$	• $\boldsymbol{B}$ ∥ [100] and strain ⊥ [010]<br>• $\boldsymbol{B}$ ∥ [001] and strain ⊥ [010]<br>• $\boldsymbol{B}$ ⊥ [010]<br>• $\boldsymbol{E}$ ∥ [010] and $\boldsymbol{B}$ ∥ [100]<br>• $\boldsymbol{E}$ ∥ [010] and $\boldsymbol{B}$ ∥ [001]
$P2_1'2_1'2$ (18.18) $\mathbb{Z}_2$	• $\boldsymbol{B}$ ∥ [001]
$P2_1'2_1'2_1$ (19.27) $\mathbb{Z}_1$	• $\boldsymbol{B}$ ∥ [100]
P_a2_1 (4.10) $\mathbb{Z}_1$	• strain ⊥ [010]<br>• $\boldsymbol{E}$ ∥ [010]

Table. S 132: Subgroups of $P_B2_12_12$ (18.22) that can be realized by applying $\boldsymbol{E}/\boldsymbol{B}$ fields and/or uniaxial strains.

Subgroup & SI group	Perturbation
$P2'$ (3.3) $\mathbb{Z}_1$	• $\boldsymbol{B}$ ∥ [010] and strain ⊥ [100]<br>• $\boldsymbol{B}$ ∥ [001] and strain ⊥ [100]<br>• $\boldsymbol{B}$ ⊥ [100]<br>• $\boldsymbol{E}$ ∥ [100] and $\boldsymbol{B}$ ∥ [010]<br>• $\boldsymbol{E}$ ∥ [100] and $\boldsymbol{B}$ ∥ [001]
P_S1 (1.3) $\mathbb{Z}_1$	• strain in generic direction<br>• $\boldsymbol{E}$ ∥ [100] and strain ⊥ [010]<br>• $\boldsymbol{E}$ ∥ [100] and strain ⊥ [001]<br>• $\boldsymbol{E}$ ∥ [010] and strain ⊥ [100]<br>• $\boldsymbol{E}$ ∥ [010] and strain ⊥ [001]<br>• $\boldsymbol{E}$ ∥ [001] and strain ⊥ [100]<br>• $\boldsymbol{E}$ ∥ [001] and strain ⊥ [010]<br>• $\boldsymbol{E}$ in generic direction
$P2_1$ (4.7) $\mathbb{Z}_1$	• $\boldsymbol{B}$ ∥ [100] and strain ⊥ [100]<br>• $\boldsymbol{E}$ ∥ [100] and $\boldsymbol{B}$ ∥ [100]
P_C2_1 (4.12) $\mathbb{Z}_1$	• strain ⊥ [100]<br>• $\boldsymbol{E}$ ∥ [100]

Continued on next page

...continued

Subgroup & SI group	Perturbation
$P2_1'$ (4.9) $\mathbb{Z}_1$	• $\boldsymbol{B}$ ∥ [100] and strain ⊥ [010]<br>• $\boldsymbol{B}$ ∥ [001] and strain ⊥ [010]<br>• $\boldsymbol{B}$ ⊥ [010]<br>• $\boldsymbol{E}$ ∥ [010] and $\boldsymbol{B}$ ∥ [100]<br>• $\boldsymbol{E}$ ∥ [010] and $\boldsymbol{B}$ ∥ [001]
$P2$ (3.1) $\mathbb{Z}_2$	• $\boldsymbol{B}$ ∥ [001] and strain ⊥ [001]<br>• $\boldsymbol{E}$ ∥ [001] and $\boldsymbol{B}$ ∥ [001]
$P22'2_1'$ (17.10) $\mathbb{Z}_2$	• $\boldsymbol{B}$ ∥ [001]
$P2_1$ (4.7) $\mathbb{Z}_1$	• $\boldsymbol{B}$ ∥ [010] and strain ⊥ [010]<br>• $\boldsymbol{E}$ ∥ [010] and $\boldsymbol{B}$ ∥ [010]
P_a2_1 (4.10) $\mathbb{Z}_1$	• strain ⊥ [010]<br>• $\boldsymbol{E}$ ∥ [010]
$P2_1'$ (4.9) $\mathbb{Z}_1$	• $\boldsymbol{B}$ ∥ [100] and strain ⊥ [001]<br>• $\boldsymbol{B}$ ∥ [010] and strain ⊥ [001]<br>• $\boldsymbol{B}$ ⊥ [001]<br>• $\boldsymbol{E}$ ∥ [001] and $\boldsymbol{B}$ ∥ [100]<br>• $\boldsymbol{E}$ ∥ [001] and $\boldsymbol{B}$ ∥ [010]
$P2_1'2_1'2_1$ (19.27) $\mathbb{Z}_1$	• $\boldsymbol{B}$ ∥ [100]
P_C2 (3.6) $\mathbb{Z}_1$	• strain ⊥ [001]<br>• $\boldsymbol{E}$ ∥ [001]
$P2_12_1'2'$ (18.19) $\mathbb{Z}_1$	• $\boldsymbol{B}$ ∥ [010]

Table. S 133: Subgroups of $P_C2_12_12$ (18.23) that can be realized by applying $\boldsymbol{E}/\boldsymbol{B}$ fields and/or uniaxial strains.

Subgroup & SI group	Perturbation
$P2'$ (3.3) $\mathbb{Z}_1$	• $\boldsymbol{B}$ ∥ [010] and strain ⊥ [100]<br>• $\boldsymbol{B}$ ∥ [001] and strain ⊥ [100]<br>• $\boldsymbol{B}$ ⊥ [100]<br>• $\boldsymbol{E}$ ∥ [100] and $\boldsymbol{B}$ ∥ [010]<br>• $\boldsymbol{E}$ ∥ [100] and $\boldsymbol{B}$ ∥ [001]

Continued on next page

. . . continued

Subgroup & SI group	Perturbation
P_S1 (1.3) $\mathbb{Z}_1$	• strain in generic direction<br>• $\boldsymbol{E} \parallel [100]$ and strain $\perp [010]$<br>• $\boldsymbol{E} \parallel [100]$ and strain $\perp [001]$<br>• $\boldsymbol{E} \parallel [010]$ and strain $\perp [100]$<br>• $\boldsymbol{E} \parallel [010]$ and strain $\perp [001]$<br>• $\boldsymbol{E} \parallel [001]$ and strain $\perp [100]$<br>• $\boldsymbol{E} \parallel [001]$ and strain $\perp [010]$<br>• $\boldsymbol{E}$ in generic direction
$P2_1$ (4.7) $\mathbb{Z}_1$	• $\boldsymbol{B} \parallel [100]$ and strain $\perp [100]$<br>• $\boldsymbol{E} \parallel [100]$ and $\boldsymbol{B} \parallel [100]$
P_C2_1 (4.12) $\mathbb{Z}_1$	• strain $\perp [100]$<br>• $\boldsymbol{E} \parallel [100]$
$P2'$ (3.3) $\mathbb{Z}_1$	• $\boldsymbol{B} \parallel [100]$ and strain $\perp [010]$<br>• $\boldsymbol{B} \parallel [001]$ and strain $\perp [010]$<br>• $\boldsymbol{B} \perp [010]$<br>• $\boldsymbol{E} \parallel [010]$ and $\boldsymbol{B} \parallel [100]$<br>• $\boldsymbol{E} \parallel [010]$ and $\boldsymbol{B} \parallel [001]$
$P2$ (3.1) $\mathbb{Z}_2$	• $\boldsymbol{B} \parallel [001]$ and strain $\perp [001]$<br>• $\boldsymbol{E} \parallel [001]$ and $\boldsymbol{B} \parallel [001]$
$P2'2'2$ (16.3) $\mathbb{Z}_2$	• $\boldsymbol{B} \parallel [001]$
$P2_1$ (4.7) $\mathbb{Z}_1$	• $\boldsymbol{B} \parallel [010]$ and strain $\perp [010]$<br>• $\boldsymbol{E} \parallel [010]$ and $\boldsymbol{B} \parallel [010]$
P_C2_1 (4.12) $\mathbb{Z}_1$	• strain $\perp [010]$<br>• $\boldsymbol{E} \parallel [010]$
$P2'$ (3.3) $\mathbb{Z}_1$	• $\boldsymbol{B} \parallel [100]$ and strain $\perp [001]$<br>• $\boldsymbol{B} \parallel [010]$ and strain $\perp [001]$<br>• $\boldsymbol{B} \perp [001]$<br>• $\boldsymbol{E} \parallel [001]$ and $\boldsymbol{B} \parallel [100]$<br>• $\boldsymbol{E} \parallel [001]$ and $\boldsymbol{B} \parallel [010]$
$P2'2'2_1$ (17.9) $\mathbb{Z}_1$	• $\boldsymbol{B} \parallel [100]$
P_a2 (3.4) $\mathbb{Z}_2$	• strain $\perp [001]$<br>• $\boldsymbol{E} \parallel [001]$
$P2'2'2_1$ (17.9) $\mathbb{Z}_1$	• $\boldsymbol{B} \parallel [010]$

Table. S 134: Subgroups of $P_I2_12_12$ (18.24) that can be realized by applying $\boldsymbol{E}/\boldsymbol{B}$ fields and/or uniaxial strains.

Subgroup & SI group	Perturbation
$P2'$ (3.3) $\mathbb{Z}_1$	• $\boldsymbol{B} \parallel [010]$ and strain $\perp [100]$<br>• $\boldsymbol{B} \parallel [001]$ and strain $\perp [100]$<br>• $\boldsymbol{B} \perp [100]$<br>• $\boldsymbol{E} \parallel [100]$ and $\boldsymbol{B} \parallel [010]$<br>• $\boldsymbol{E} \parallel [100]$ and $\boldsymbol{B} \parallel [001]$
P_S1 (1.3) $\mathbb{Z}_1$	• strain in generic direction<br>• $\boldsymbol{E} \parallel [100]$ and strain $\perp [010]$<br>• $\boldsymbol{E} \parallel [100]$ and strain $\perp [001]$<br>• $\boldsymbol{E} \parallel [010]$ and strain $\perp [100]$<br>• $\boldsymbol{E} \parallel [010]$ and strain $\perp [001]$<br>• $\boldsymbol{E} \parallel [001]$ and strain $\perp [100]$<br>• $\boldsymbol{E} \parallel [001]$ and strain $\perp [010]$<br>• $\boldsymbol{E}$ in generic direction
$P2_1$ (4.7) $\mathbb{Z}_1$	• $\boldsymbol{B} \parallel [100]$ and strain $\perp [100]$<br>• $\boldsymbol{E} \parallel [100]$ and $\boldsymbol{B} \parallel [100]$
P_C2_1 (4.12) $\mathbb{Z}_1$	• strain $\perp [100]$<br>• $\boldsymbol{E} \parallel [100]$
$P2'$ (3.3) $\mathbb{Z}_1$	• $\boldsymbol{B} \parallel [100]$ and strain $\perp [010]$<br>• $\boldsymbol{B} \parallel [001]$ and strain $\perp [010]$<br>• $\boldsymbol{B} \perp [010]$<br>• $\boldsymbol{E} \parallel [010]$ and $\boldsymbol{B} \parallel [100]$<br>• $\boldsymbol{E} \parallel [010]$ and $\boldsymbol{B} \parallel [001]$
$P2$ (3.1) $\mathbb{Z}_2$	• $\boldsymbol{B} \parallel [001]$ and strain $\perp [001]$<br>• $\boldsymbol{E} \parallel [001]$ and $\boldsymbol{B} \parallel [001]$
$P2'2'2$ (16.3) $\mathbb{Z}_2$	• $\boldsymbol{B} \parallel [001]$
$P2_1$ (4.7) $\mathbb{Z}_1$	• $\boldsymbol{B} \parallel [010]$ and strain $\perp [010]$<br>• $\boldsymbol{E} \parallel [010]$ and $\boldsymbol{B} \parallel [010]$
P_C2_1 (4.12) $\mathbb{Z}_1$	• strain $\perp [010]$<br>• $\boldsymbol{E} \parallel [010]$
$P2_1'$ (4.9) $\mathbb{Z}_1$	• $\boldsymbol{B} \parallel [100]$ and strain $\perp [001]$<br>• $\boldsymbol{B} \parallel [010]$ and strain $\perp [001]$<br>• $\boldsymbol{B} \perp [001]$<br>• $\boldsymbol{E} \parallel [001]$ and $\boldsymbol{B} \parallel [100]$<br>• $\boldsymbol{E} \parallel [001]$ and $\boldsymbol{B} \parallel [010]$
$P2_12_1'2'$ (18.19) $\mathbb{Z}_1$	• $\boldsymbol{B} \parallel [100]$

Continued on next page

. . . continued

Subgroup & SI group	Perturbation
P_C2 (3.6) $\mathbb{Z}_1$	• strain ⊥ [001]<br>• $\boldsymbol{E}$ ∥ [001]
$P2_12_1'2'$ (18.19) $\mathbb{Z}_1$	• $\boldsymbol{B}$ ∥ [010]

Table. S 135: Subgroups of $P2_12_12_1$ (19.25) that can be realized by applying $\boldsymbol{E}/\boldsymbol{B}$ fields and/or uniaxial strains.

Subgroup & SI group	Perturbation
$P2_1$ (4.7) $\mathbb{Z}_1$	• strain ⊥ [100]<br>• $\boldsymbol{B}$ ∥ [100]<br>• $\boldsymbol{E}$ ∥ [100]
$P2_1$ (4.7) $\mathbb{Z}_1$	• strain ⊥ [010]<br>• $\boldsymbol{B}$ ∥ [010]<br>• $\boldsymbol{E}$ ∥ [010]
$P2_1$ (4.7) $\mathbb{Z}_1$	• strain ⊥ [001]<br>• $\boldsymbol{B}$ ∥ [001]<br>• $\boldsymbol{E}$ ∥ [001]

Table. S 136: Subgroups of $P2_12_12_11'$ (19.26) that can be realized by applying $\boldsymbol{E}/\boldsymbol{B}$ fields and/or uniaxial strains.

Subgroup & SI group	Perturbation
$P11'$ (1.2) $\mathbb{Z}_1$	• strain in generic direction<br>• $\boldsymbol{E}$ ∥ [100] and strain ⊥ [010]<br>• $\boldsymbol{E}$ ∥ [100] and strain ⊥ [001]<br>• $\boldsymbol{E}$ ∥ [010] and strain ⊥ [100]<br>• $\boldsymbol{E}$ ∥ [010] and strain ⊥ [001]<br>• $\boldsymbol{E}$ ∥ [001] and strain ⊥ [100]<br>• $\boldsymbol{E}$ ∥ [001] and strain ⊥ [010]<br>• $\boldsymbol{E}$ in generic direction

Continued on next page

...continued

Subgroup & SI group	Perturbation
$P2_1'$ (4.9) $\mathbb{Z}_1$	• $\boldsymbol{B}\parallel[010]$ and strain $\perp[100]$<br>• $\boldsymbol{B}\parallel[001]$ and strain $\perp[100]$<br>• $\boldsymbol{B}\perp[100]$<br>• $\boldsymbol{E}\parallel[100]$ and $\boldsymbol{B}\parallel[010]$<br>• $\boldsymbol{E}\parallel[100]$ and $\boldsymbol{B}\parallel[001]$
$P2_1$ (4.7) $\mathbb{Z}_1$	• $\boldsymbol{B}\parallel[100]$ and strain $\perp[100]$<br>• $\boldsymbol{E}\parallel[100]$ and $\boldsymbol{B}\parallel[100]$
$P2_11'$ (4.8) $\mathbb{Z}_1$	• strain $\perp[100]$<br>• $\boldsymbol{E}\parallel[100]$
$P2_1'$ (4.9) $\mathbb{Z}_1$	• $\boldsymbol{B}\parallel[100]$ and strain $\perp[010]$<br>• $\boldsymbol{B}\parallel[001]$ and strain $\perp[010]$<br>• $\boldsymbol{B}\perp[010]$<br>• $\boldsymbol{E}\parallel[010]$ and $\boldsymbol{B}\parallel[100]$<br>• $\boldsymbol{E}\parallel[010]$ and $\boldsymbol{B}\parallel[001]$
$P2_1$ (4.7) $\mathbb{Z}_1$	• $\boldsymbol{B}\parallel[010]$ and strain $\perp[010]$<br>• $\boldsymbol{E}\parallel[010]$ and $\boldsymbol{B}\parallel[010]$
$P2_11'$ (4.8) $\mathbb{Z}_1$	• strain $\perp[010]$<br>• $\boldsymbol{E}\parallel[010]$
$P2_1'$ (4.9) $\mathbb{Z}_1$	• $\boldsymbol{B}\parallel[100]$ and strain $\perp[001]$<br>• $\boldsymbol{B}\parallel[010]$ and strain $\perp[001]$<br>• $\boldsymbol{B}\perp[001]$<br>• $\boldsymbol{E}\parallel[001]$ and $\boldsymbol{B}\parallel[100]$<br>• $\boldsymbol{E}\parallel[001]$ and $\boldsymbol{B}\parallel[010]$
$P2_1'2_1'2_1$ (19.27) $\mathbb{Z}_1$	• $\boldsymbol{B}\parallel[100]$
$P2_1'2_1'2_1$ (19.27) $\mathbb{Z}_1$	• $\boldsymbol{B}\parallel[010]$
$P2_1$ (4.7) $\mathbb{Z}_1$	• $\boldsymbol{B}\parallel[001]$ and strain $\perp[001]$<br>• $\boldsymbol{E}\parallel[001]$ and $\boldsymbol{B}\parallel[001]$
$P2_1'2_1'2_1$ (19.27) $\mathbb{Z}_1$	• $\boldsymbol{B}\parallel[001]$
$P2_11'$ (4.8) $\mathbb{Z}_1$	• strain $\perp[001]$<br>• $\boldsymbol{E}\parallel[001]$

Table. S 137: Subgroups of $P2_1'2_1'2_1$ (19.27) that can be realized by applying $\boldsymbol{E}/\boldsymbol{B}$ fields and/or uniaxial strains.

Subgroup & SI group	Perturbation
$P2_1'$ (4.9) $\mathbb{Z}_1$	• strain $\perp$ [100]<br>• ($\boldsymbol{B}$ ∥ [010] or $\boldsymbol{B}$ $\perp$ [100])<br>• $\boldsymbol{E}$ ∥ [100]
$P2_1'$ (4.9) $\mathbb{Z}_1$	• strain $\perp$ [010]<br>• ($\boldsymbol{B}$ ∥ [100] or $\boldsymbol{B}$ $\perp$ [010])<br>• $\boldsymbol{E}$ ∥ [010]
$P2_1$ (4.7) $\mathbb{Z}_1$	• strain $\perp$ [001]<br>• $\boldsymbol{E}$ ∥ [001]

Table. S 138: Subgroups of $P_c2_12_12_1$ (19.28) that can be realized by applying $\boldsymbol{E}/\boldsymbol{B}$ fields and/or uniaxial strains.

Subgroup & SI group	Perturbation
P_S1 (1.3) $\mathbb{Z}_1$	• strain in generic direction<br>• $\boldsymbol{E}$ ∥ [100] and strain $\perp$ [010]<br>• $\boldsymbol{E}$ ∥ [100] and strain $\perp$ [001]<br>• $\boldsymbol{E}$ ∥ [010] and strain $\perp$ [100]<br>• $\boldsymbol{E}$ ∥ [010] and strain $\perp$ [001]<br>• $\boldsymbol{E}$ ∥ [001] and strain $\perp$ [100]<br>• $\boldsymbol{E}$ ∥ [001] and strain $\perp$ [010]<br>• $\boldsymbol{E}$ in generic direction
$P2_1$ (4.7) $\mathbb{Z}_1$	• $\boldsymbol{B}$ ∥ [100] and strain $\perp$ [100]<br>• $\boldsymbol{E}$ ∥ [100] and $\boldsymbol{B}$ ∥ [100]
$P2_1'$ (4.9) $\mathbb{Z}_1$	• $\boldsymbol{B}$ ∥ [010] and strain $\perp$ [100]<br>• $\boldsymbol{B}$ ∥ [001] and strain $\perp$ [100]<br>• $\boldsymbol{B}$ $\perp$ [100]<br>• $\boldsymbol{E}$ ∥ [100] and $\boldsymbol{B}$ ∥ [010]<br>• $\boldsymbol{E}$ ∥ [100] and $\boldsymbol{B}$ ∥ [001]
P_a2_1 (4.10) $\mathbb{Z}_1$	• strain $\perp$ [100]<br>• $\boldsymbol{E}$ ∥ [100]

Continued on next page

. . . continued

Subgroup & SI group	Perturbation
$P2_1'$ (4.9) $\mathbb{Z}_1$	• $\boldsymbol{B}$ $\parallel$ [100] and strain $\perp$ [010]<br>• $\boldsymbol{B}$ $\parallel$ [001] and strain $\perp$ [010]<br>• $\boldsymbol{B}$ $\perp$ [010]<br>• $\boldsymbol{E}$ $\parallel$ [010] and $\boldsymbol{B}$ $\parallel$ [100]<br>• $\boldsymbol{E}$ $\parallel$ [010] and $\boldsymbol{B}$ $\parallel$ [001]
$P2_1$ (4.7) $\mathbb{Z}_1$	• $\boldsymbol{B}$ $\parallel$ [010] and strain $\perp$ [010]<br>• $\boldsymbol{E}$ $\parallel$ [010] and $\boldsymbol{B}$ $\parallel$ [010]
P_a2_1 (4.10) $\mathbb{Z}_1$	• strain $\perp$ [010]<br>• $\boldsymbol{E}$ $\parallel$ [010]
$P2'$ (3.3) $\mathbb{Z}_1$	• $\boldsymbol{B}$ $\parallel$ [100] and strain $\perp$ [001]<br>• $\boldsymbol{B}$ $\parallel$ [010] and strain $\perp$ [001]<br>• $\boldsymbol{B}$ $\perp$ [001]<br>• $\boldsymbol{E}$ $\parallel$ [001] and $\boldsymbol{B}$ $\parallel$ [100]<br>• $\boldsymbol{E}$ $\parallel$ [001] and $\boldsymbol{B}$ $\parallel$ [010]
$P2_12_1'2'$ (18.19) $\mathbb{Z}_1$	• $\boldsymbol{B}$ $\parallel$ [100]
$P2_12_1'2'$ (18.19) $\mathbb{Z}_1$	• $\boldsymbol{B}$ $\parallel$ [010]
$P2_1$ (4.7) $\mathbb{Z}_1$	• $\boldsymbol{B}$ $\parallel$ [001] and strain $\perp$ [001]<br>• $\boldsymbol{E}$ $\parallel$ [001] and $\boldsymbol{B}$ $\parallel$ [001]
$P2_1'2_1'2_1$ (19.27) $\mathbb{Z}_1$	• $\boldsymbol{B}$ $\parallel$ [001]
P_b2_1 (4.11) $\mathbb{Z}_1$	• strain $\perp$ [001]<br>• $\boldsymbol{E}$ $\parallel$ [001]

Table. S 139: Subgroups of $P_C2_12_12_1$ (19.29) that can be realized by applying $\boldsymbol{E}/\boldsymbol{B}$ fields and/or uniaxial strains.

Subgroup & SI group	Perturbation
$P2'$ (3.3) $\mathbb{Z}_1$	• $\boldsymbol{B}$ $\parallel$ [010] and strain $\perp$ [100]<br>• $\boldsymbol{B}$ $\parallel$ [001] and strain $\perp$ [100]<br>• $\boldsymbol{B}$ $\perp$ [100]<br>• $\boldsymbol{E}$ $\parallel$ [100] and $\boldsymbol{B}$ $\parallel$ [010]<br>• $\boldsymbol{E}$ $\parallel$ [100] and $\boldsymbol{B}$ $\parallel$ [001]

Continued on next page

...continued

Subgroup & SI group	Perturbation
P_S1 (1.3) $\mathbb{Z}_1$	• strain in generic direction<br>• $\boldsymbol{E} \parallel [100]$ and strain $\perp [010]$<br>• $\boldsymbol{E} \parallel [100]$ and strain $\perp [001]$<br>• $\boldsymbol{E} \parallel [010]$ and strain $\perp [100]$<br>• $\boldsymbol{E} \parallel [010]$ and strain $\perp [001]$<br>• $\boldsymbol{E} \parallel [001]$ and strain $\perp [100]$<br>• $\boldsymbol{E} \parallel [001]$ and strain $\perp [010]$<br>• $\boldsymbol{E}$ in generic direction
$P2_1$ (4.7) $\mathbb{Z}_1$	• $\boldsymbol{B} \parallel [100]$ and strain $\perp [100]$<br>• $\boldsymbol{E} \parallel [100]$ and $\boldsymbol{B} \parallel [100]$
P_C2_1 (4.12) $\mathbb{Z}_1$	• strain $\perp [100]$<br>• $\boldsymbol{E} \parallel [100]$
$P2_1$ (4.7) $\mathbb{Z}_1$	• $\boldsymbol{B} \parallel [010]$ and strain $\perp [010]$<br>• $\boldsymbol{E} \parallel [010]$ and $\boldsymbol{B} \parallel [010]$
$P2_1'$ (4.9) $\mathbb{Z}_1$	• $\boldsymbol{B} \parallel [100]$ and strain $\perp [001]$<br>• $\boldsymbol{B} \parallel [010]$ and strain $\perp [001]$<br>• $\boldsymbol{B} \perp [001]$<br>• $\boldsymbol{E} \parallel [001]$ and $\boldsymbol{B} \parallel [100]$<br>• $\boldsymbol{E} \parallel [001]$ and $\boldsymbol{B} \parallel [010]$
$P2_12_1'2'$ (18.19) $\mathbb{Z}_1$	• $\boldsymbol{B} \parallel [010]$
$P2'$ (3.3) $\mathbb{Z}_1$	• $\boldsymbol{B} \parallel [100]$ and strain $\perp [010]$<br>• $\boldsymbol{B} \parallel [001]$ and strain $\perp [010]$<br>• $\boldsymbol{B} \perp [010]$<br>• $\boldsymbol{E} \parallel [010]$ and $\boldsymbol{B} \parallel [100]$<br>• $\boldsymbol{E} \parallel [010]$ and $\boldsymbol{B} \parallel [001]$
P_C2_1 (4.12) $\mathbb{Z}_1$	• strain $\perp [010]$<br>• $\boldsymbol{E} \parallel [010]$
$P2_12_1'2'$ (18.19) $\mathbb{Z}_1$	• $\boldsymbol{B} \parallel [100]$
$P2_1$ (4.7) $\mathbb{Z}_1$	• $\boldsymbol{B} \parallel [001]$ and strain $\perp [001]$<br>• $\boldsymbol{E} \parallel [001]$ and $\boldsymbol{B} \parallel [001]$
P_a2_1 (4.10) $\mathbb{Z}_1$	• strain $\perp [001]$<br>• $\boldsymbol{E} \parallel [001]$
$P2'2'2_1$ (17.9) $\mathbb{Z}_1$	• $\boldsymbol{B} \parallel [001]$

Table. S 140: Subgroups of $P_I 2_1 2_1 2_1$ (19.30) that can be realized by applying $\boldsymbol{E}/\boldsymbol{B}$ fields and/or uniaxial strains.

Subgroup & SI group	Perturbation
$P2'$ (3.3) $\mathbb{Z}_1$	• $\boldsymbol{B} \parallel [010]$ and strain $\perp [100]$<br>• $\boldsymbol{B} \parallel [001]$ and strain $\perp [100]$<br>• $\boldsymbol{B} \perp [100]$<br>• $\boldsymbol{E} \parallel [100]$ and $\boldsymbol{B} \parallel [010]$<br>• $\boldsymbol{E} \parallel [100]$ and $\boldsymbol{B} \parallel [001]$
P_S1 (1.3) $\mathbb{Z}_1$	• strain in generic direction<br>• $\boldsymbol{E} \parallel [100]$ and strain $\perp [010]$<br>• $\boldsymbol{E} \parallel [100]$ and strain $\perp [001]$<br>• $\boldsymbol{E} \parallel [010]$ and strain $\perp [100]$<br>• $\boldsymbol{E} \parallel [010]$ and strain $\perp [001]$<br>• $\boldsymbol{E} \parallel [001]$ and strain $\perp [100]$<br>• $\boldsymbol{E} \parallel [001]$ and strain $\perp [010]$<br>• $\boldsymbol{E}$ in generic direction
$P2_1$ (4.7) $\mathbb{Z}_1$	• $\boldsymbol{B} \parallel [100]$ and strain $\perp [100]$<br>• $\boldsymbol{E} \parallel [100]$ and $\boldsymbol{B} \parallel [100]$
P_C2_1 (4.12) $\mathbb{Z}_1$	• strain $\perp [100]$<br>• $\boldsymbol{E} \parallel [100]$
$P2_1$ (4.7) $\mathbb{Z}_1$	• $\boldsymbol{B} \parallel [010]$ and strain $\perp [010]$<br>• $\boldsymbol{E} \parallel [010]$ and $\boldsymbol{B} \parallel [010]$
$P2'$ (3.3) $\mathbb{Z}_1$	• $\boldsymbol{B} \parallel [100]$ and strain $\perp [001]$<br>• $\boldsymbol{B} \parallel [010]$ and strain $\perp [001]$<br>• $\boldsymbol{B} \perp [001]$<br>• $\boldsymbol{E} \parallel [001]$ and $\boldsymbol{B} \parallel [100]$<br>• $\boldsymbol{E} \parallel [001]$ and $\boldsymbol{B} \parallel [010]$
$P2'2'2_1$ (17.9) $\mathbb{Z}_1$	• $\boldsymbol{B} \parallel [010]$
$P2'$ (3.3) $\mathbb{Z}_1$	• $\boldsymbol{B} \parallel [100]$ and strain $\perp [010]$<br>• $\boldsymbol{B} \parallel [001]$ and strain $\perp [010]$<br>• $\boldsymbol{B} \perp [010]$<br>• $\boldsymbol{E} \parallel [010]$ and $\boldsymbol{B} \parallel [100]$<br>• $\boldsymbol{E} \parallel [010]$ and $\boldsymbol{B} \parallel [001]$
P_C2_1 (4.12) $\mathbb{Z}_1$	• strain $\perp [010]$<br>• $\boldsymbol{E} \parallel [010]$
$P2'2'2_1$ (17.9) $\mathbb{Z}_1$	• $\boldsymbol{B} \parallel [100]$
$P2_1$ (4.7) $\mathbb{Z}_1$	• $\boldsymbol{B} \parallel [001]$ and strain $\perp [001]$<br>• $\boldsymbol{E} \parallel [001]$ and $\boldsymbol{B} \parallel [001]$

Continued on next page

...continued

Subgroup & SI group	Perturbation
P_C2_1 (4.12) $\mathbb{Z}_1$	• strain $\perp$ $[001]$<br>• $\boldsymbol{E}$ $\parallel$ $[001]$
$P2'2'2_1$ (17.9) $\mathbb{Z}_1$	• $\boldsymbol{B}$ $\parallel$ $[001]$

Table. S 141: Subgroups of $C222_1$ (20.31) that can be realized by applying $\boldsymbol{E}/\boldsymbol{B}$ fields and/or uniaxial strains.

Subgroup & SI group	Perturbation
$C2$ (5.13) $\mathbb{Z}_1$	• strain $\perp$ $[100]$<br>• $\boldsymbol{B}$ $\parallel$ $[100]$<br>• $\boldsymbol{E}$ $\parallel$ $[100]$
$C2$ (5.13) $\mathbb{Z}_1$	• strain $\perp$ $[010]$<br>• $\boldsymbol{B}$ $\parallel$ $[010]$<br>• $\boldsymbol{E}$ $\parallel$ $[010]$
$P2_1$ (4.7) $\mathbb{Z}_1$	• strain $\perp$ $[001]$<br>• $\boldsymbol{B}$ $\parallel$ $[001]$<br>• $\boldsymbol{E}$ $\parallel$ $[001]$

Table. S 142: Subgroups of $C222_11'$ (20.32) that can be realized by applying $\boldsymbol{E}/\boldsymbol{B}$ fields and/or uniaxial strains.

Subgroup & SI group	Perturbation
$P11'$ (1.2) $\mathbb{Z}_1$	• strain in generic direction<br>• $\boldsymbol{E}$ $\parallel$ $[100]$ and strain $\perp$ $[010]$<br>• $\boldsymbol{E}$ $\parallel$ $[100]$ and strain $\perp$ $[001]$<br>• $\boldsymbol{E}$ $\parallel$ $[010]$ and strain $\perp$ $[100]$<br>• $\boldsymbol{E}$ $\parallel$ $[010]$ and strain $\perp$ $[001]$<br>• $\boldsymbol{E}$ $\parallel$ $[001]$ and strain $\perp$ $[100]$<br>• $\boldsymbol{E}$ $\parallel$ $[001]$ and strain $\perp$ $[010]$<br>• $\boldsymbol{E}$ in generic direction

Continued on next page

...continued

Subgroup & SI group	Perturbation
$C2'$ (5.15) $\mathbb{Z}_1$	• $\boldsymbol{B} \parallel [010]$ and strain $\perp [100]$<br>• $\boldsymbol{B} \parallel [001]$ and strain $\perp [100]$<br>• $\boldsymbol{B} \perp [100]$<br>• $\boldsymbol{E} \parallel [100]$ and $\boldsymbol{B} \parallel [010]$<br>• $\boldsymbol{E} \parallel [100]$ and $\boldsymbol{B} \parallel [001]$
$C2$ (5.13) $\mathbb{Z}_1$	• $\boldsymbol{B} \parallel [100]$ and strain $\perp [100]$<br>• $\boldsymbol{E} \parallel [100]$ and $\boldsymbol{B} \parallel [100]$
$C21'$ (5.14) $\mathbb{Z}_1$	• strain $\perp [100]$<br>• $\boldsymbol{E} \parallel [100]$
$C2'$ (5.15) $\mathbb{Z}_1$	• $\boldsymbol{B} \parallel [100]$ and strain $\perp [010]$<br>• $\boldsymbol{B} \parallel [001]$ and strain $\perp [010]$<br>• $\boldsymbol{B} \perp [010]$<br>• $\boldsymbol{E} \parallel [010]$ and $\boldsymbol{B} \parallel [100]$<br>• $\boldsymbol{E} \parallel [010]$ and $\boldsymbol{B} \parallel [001]$
$C2$ (5.13) $\mathbb{Z}_1$	• $\boldsymbol{B} \parallel [010]$ and strain $\perp [010]$<br>• $\boldsymbol{E} \parallel [010]$ and $\boldsymbol{B} \parallel [010]$
$C21'$ (5.14) $\mathbb{Z}_1$	• strain $\perp [010]$<br>• $\boldsymbol{E} \parallel [010]$
$P2_1'$ (4.9) $\mathbb{Z}_1$	• $\boldsymbol{B} \parallel [100]$ and strain $\perp [001]$<br>• $\boldsymbol{B} \parallel [010]$ and strain $\perp [001]$<br>• $\boldsymbol{B} \perp [001]$<br>• $\boldsymbol{E} \parallel [001]$ and $\boldsymbol{B} \parallel [100]$<br>• $\boldsymbol{E} \parallel [001]$ and $\boldsymbol{B} \parallel [010]$
$C22'2_1'$ (20.34) $\mathbb{Z}_1$	• $\boldsymbol{B} \parallel [100]$
$C22'2_1'$ (20.34) $\mathbb{Z}_1$	• $\boldsymbol{B} \parallel [010]$
$P2_1$ (4.7) $\mathbb{Z}_1$	• $\boldsymbol{B} \parallel [001]$ and strain $\perp [001]$<br>• $\boldsymbol{E} \parallel [001]$ and $\boldsymbol{B} \parallel [001]$
$C2'2'2_1$ (20.33) $\mathbb{Z}_1$	• $\boldsymbol{B} \parallel [001]$
$P2_11'$ (4.8) $\mathbb{Z}_1$	• strain $\perp [001]$<br>• $\boldsymbol{E} \parallel [001]$

Table. S 143: Subgroups of $C2'2'2_1$ (20.33) that can be realized by applying $\boldsymbol{E}/\boldsymbol{B}$ fields and/or uniaxial strains.

Subgroup & SI group	Perturbation
$C2'$ (5.15) $\mathbb{Z}_1$	• strain $\perp$ [100]<br>• ($\boldsymbol{B} \parallel$ [010] or $\boldsymbol{B} \perp$ [100])<br>• $\boldsymbol{E} \parallel$ [100]
$C2'$ (5.15) $\mathbb{Z}_1$	• strain $\perp$ [010]<br>• ($\boldsymbol{B} \parallel$ [100] or $\boldsymbol{B} \perp$ [010])<br>• $\boldsymbol{E} \parallel$ [010]
$P2_1$ (4.7) $\mathbb{Z}_1$	• strain $\perp$ [001]<br>• $\boldsymbol{E} \parallel$ [001]

Table. S 144: Subgroups of $C22'2'_1$ (20.34) that can be realized by applying $\boldsymbol{E}/\boldsymbol{B}$ fields and/or uniaxial strains.

Subgroup & SI group	Perturbation
$C2$ (5.13) $\mathbb{Z}_1$	• strain $\perp$ [100]<br>• $\boldsymbol{E} \parallel$ [100]
$C2'$ (5.15) $\mathbb{Z}_1$	• strain $\perp$ [010]<br>• ($\boldsymbol{B} \parallel$ [001] or $\boldsymbol{B} \perp$ [010])<br>• $\boldsymbol{E} \parallel$ [010]
$P2'_1$ (4.9) $\mathbb{Z}_1$	• strain $\perp$ [001]<br>• ($\boldsymbol{B} \parallel$ [010] or $\boldsymbol{B} \perp$ [001])<br>• $\boldsymbol{E} \parallel$ [001]

Table. S 145: Subgroups of C_c222_1 (20.35) that can be realized by applying $\boldsymbol{E}/\boldsymbol{B}$ fields and/or uniaxial strains.

Subgroup & SI group	Perturbation

Continued on next page

. . . continued

Subgroup & SI group	Perturbation
P_S1 (1.3) $\mathbb{Z}_1$	• strain in generic direction<br>• $\boldsymbol{E}$ || [100] and strain ⊥ [010]<br>• $\boldsymbol{E}$ || [100] and strain ⊥ [001]<br>• $\boldsymbol{E}$ || [010] and strain ⊥ [100]<br>• $\boldsymbol{E}$ || [010] and strain ⊥ [001]<br>• $\boldsymbol{E}$ || [001] and strain ⊥ [100]<br>• $\boldsymbol{E}$ || [001] and strain ⊥ [010]<br>• $\boldsymbol{E}$ in generic direction
$C2$ (5.13) $\mathbb{Z}_1$	• $\boldsymbol{B}$ || [100] and strain ⊥ [100]<br>• $\boldsymbol{E}$ || [100] and $\boldsymbol{B}$ || [100]
$C2'$ (5.15) $\mathbb{Z}_1$	• $\boldsymbol{B}$ || [010] and strain ⊥ [100]<br>• $\boldsymbol{B}$ || [001] and strain ⊥ [100]<br>• $\boldsymbol{B}$ ⊥ [100]<br>• $\boldsymbol{E}$ || [100] and $\boldsymbol{B}$ || [010]<br>• $\boldsymbol{E}$ || [100] and $\boldsymbol{B}$ || [001]
C_c2 (5.16) $\mathbb{Z}_1$	• strain ⊥ [100]<br>• $\boldsymbol{E}$ || [100]
$C2'$ (5.15) $\mathbb{Z}_1$	• $\boldsymbol{B}$ || [100] and strain ⊥ [010]<br>• $\boldsymbol{B}$ || [001] and strain ⊥ [010]<br>• $\boldsymbol{B}$ ⊥ [010]<br>• $\boldsymbol{E}$ || [010] and $\boldsymbol{B}$ || [100]<br>• $\boldsymbol{E}$ || [010] and $\boldsymbol{B}$ || [001]
$C2$ (5.13) $\mathbb{Z}_1$	• $\boldsymbol{B}$ || [010] and strain ⊥ [010]<br>• $\boldsymbol{E}$ || [010] and $\boldsymbol{B}$ || [010]
C_c2 (5.16) $\mathbb{Z}_1$	• strain ⊥ [010]<br>• $\boldsymbol{E}$ || [010]
$P2'$ (3.3) $\mathbb{Z}_1$	• $\boldsymbol{B}$ || [100] and strain ⊥ [001]<br>• $\boldsymbol{B}$ || [010] and strain ⊥ [001]<br>• $\boldsymbol{B}$ ⊥ [001]<br>• $\boldsymbol{E}$ || [001] and $\boldsymbol{B}$ || [100]<br>• $\boldsymbol{E}$ || [001] and $\boldsymbol{B}$ || [010]
$C22'2'$ (21.41) $\mathbb{Z}_1$	• $\boldsymbol{B}$ || [100]
$C22'2'$ (21.41) $\mathbb{Z}_1$	• $\boldsymbol{B}$ || [010]
$P2_1$ (4.7) $\mathbb{Z}_1$	• $\boldsymbol{B}$ || [001] and strain ⊥ [001]<br>• $\boldsymbol{E}$ || [001] and $\boldsymbol{B}$ || [001]
$C2'2'2_1$ (20.33) $\mathbb{Z}_1$	• $\boldsymbol{B}$ || [001]

Continued on next page

...continued

Subgroup & SI group	Perturbation
P_b2_1 (4.11) $\mathbb{Z}_1$	• strain $\perp$ [001]<br>• $\boldsymbol{E} \parallel$ [001]

Table. S 146: Subgroups of C_a222_1 (20.36) that can be realized by applying $\boldsymbol{E}/\boldsymbol{B}$ fields and/or uniaxial strains.

Subgroup & SI group	Perturbation
P_S1 (1.3) $\mathbb{Z}_1$	• strain in generic direction<br>• $\boldsymbol{E} \parallel$ [100] and strain $\perp$ [010]<br>• $\boldsymbol{E} \parallel$ [100] and strain $\perp$ [001]<br>• $\boldsymbol{E} \parallel$ [010] and strain $\perp$ [100]<br>• $\boldsymbol{E} \parallel$ [010] and strain $\perp$ [001]<br>• $\boldsymbol{E} \parallel$ [001] and strain $\perp$ [100]<br>• $\boldsymbol{E} \parallel$ [001] and strain $\perp$ [010]<br>• $\boldsymbol{E}$ in generic direction
$C2'$ (5.15) $\mathbb{Z}_1$	• $\boldsymbol{B} \parallel$ [010] and strain $\perp$ [100]<br>• $\boldsymbol{B} \parallel$ [001] and strain $\perp$ [100]<br>• $\boldsymbol{B} \perp$ [100]<br>• $\boldsymbol{E} \parallel$ [100] and $\boldsymbol{B} \parallel$ [010]<br>• $\boldsymbol{E} \parallel$ [100] and $\boldsymbol{B} \parallel$ [001]
$C2$ (5.13) $\mathbb{Z}_1$	• $\boldsymbol{B} \parallel$ [100] and strain $\perp$ [100]<br>• $\boldsymbol{E} \parallel$ [100] and $\boldsymbol{B} \parallel$ [100]
C_a2 (5.17) $\mathbb{Z}_2$	• strain $\perp$ [100]<br>• $\boldsymbol{E} \parallel$ [100]
$C2'$ (5.15) $\mathbb{Z}_1$	• $\boldsymbol{B} \parallel$ [100] and strain $\perp$ [010]<br>• $\boldsymbol{B} \parallel$ [001] and strain $\perp$ [010]<br>• $\boldsymbol{B} \perp$ [010]<br>• $\boldsymbol{E} \parallel$ [010] and $\boldsymbol{B} \parallel$ [100]<br>• $\boldsymbol{E} \parallel$ [010] and $\boldsymbol{B} \parallel$ [001]
$C2$ (5.13) $\mathbb{Z}_1$	• $\boldsymbol{B} \parallel$ [010] and strain $\perp$ [010]<br>• $\boldsymbol{E} \parallel$ [010] and $\boldsymbol{B} \parallel$ [010]
C_a2 (5.17) $\mathbb{Z}_2$	• strain $\perp$ [010]<br>• $\boldsymbol{E} \parallel$ [010]

Continued on next page

...continued

Subgroup & SI group	Perturbation
$P2_1'$ (4.9) $\mathbb{Z}_1$	• $\boldsymbol{B} \parallel [100]$ and strain $\perp [001]$<br>• $\boldsymbol{B} \parallel [010]$ and strain $\perp [001]$<br>• $\boldsymbol{B} \perp [001]$<br>• $\boldsymbol{E} \parallel [001]$ and $\boldsymbol{B} \parallel [100]$<br>• $\boldsymbol{E} \parallel [001]$ and $\boldsymbol{B} \parallel [010]$
$C22'2_1'$ (20.34) $\mathbb{Z}_1$	• $\boldsymbol{B} \parallel [100]$
$C22'2_1'$ (20.34) $\mathbb{Z}_1$	• $\boldsymbol{B} \parallel [010]$
$P2_1$ (4.7) $\mathbb{Z}_1$	• $\boldsymbol{B} \parallel [001]$ and strain $\perp [001]$<br>• $\boldsymbol{E} \parallel [001]$ and $\boldsymbol{B} \parallel [001]$
$C2'2'2_1$ (20.33) $\mathbb{Z}_1$	• $\boldsymbol{B} \parallel [001]$
P_a2_1 (4.10) $\mathbb{Z}_1$	• strain $\perp [001]$<br>• $\boldsymbol{E} \parallel [001]$

Table. S 147: Subgroups of C_A222_1 (20.37) that can be realized by applying $\boldsymbol{E}/\boldsymbol{B}$ fields and/or uniaxial strains.

Subgroup & SI group	Perturbation
P_S1 (1.3) $\mathbb{Z}_1$	• strain in generic direction<br>• $\boldsymbol{E} \parallel [100]$ and strain $\perp [010]$<br>• $\boldsymbol{E} \parallel [100]$ and strain $\perp [001]$<br>• $\boldsymbol{E} \parallel [010]$ and strain $\perp [100]$<br>• $\boldsymbol{E} \parallel [010]$ and strain $\perp [001]$<br>• $\boldsymbol{E} \parallel [001]$ and strain $\perp [100]$<br>• $\boldsymbol{E} \parallel [001]$ and strain $\perp [010]$<br>• $\boldsymbol{E}$ in generic direction
$C2'$ (5.15) $\mathbb{Z}_1$	• $\boldsymbol{B} \parallel [010]$ and strain $\perp [100]$<br>• $\boldsymbol{B} \parallel [001]$ and strain $\perp [100]$<br>• $\boldsymbol{B} \perp [100]$<br>• $\boldsymbol{E} \parallel [100]$ and $\boldsymbol{B} \parallel [010]$<br>• $\boldsymbol{E} \parallel [100]$ and $\boldsymbol{B} \parallel [001]$
$C2$ (5.13) $\mathbb{Z}_1$	• $\boldsymbol{B} \parallel [100]$ and strain $\perp [100]$<br>• $\boldsymbol{E} \parallel [100]$ and $\boldsymbol{B} \parallel [100]$
C_c2 (5.16) $\mathbb{Z}_1$	• strain $\perp [100]$<br>• $\boldsymbol{E} \parallel [100]$

Continued on next page

...continued

Subgroup & SI group	Perturbation
$C2'$ (5.15) $\mathbb{Z}_1$	• $\boldsymbol{B} \parallel [100]$ and strain $\perp [010]$<br>• $\boldsymbol{B} \parallel [001]$ and strain $\perp [010]$<br>• $\boldsymbol{B} \perp [010]$<br>• $\boldsymbol{E} \parallel [010]$ and $\boldsymbol{B} \parallel [100]$<br>• $\boldsymbol{E} \parallel [010]$ and $\boldsymbol{B} \parallel [001]$
$C2$ (5.13) $\mathbb{Z}_1$	• $\boldsymbol{B} \parallel [010]$ and strain $\perp [010]$<br>• $\boldsymbol{E} \parallel [010]$ and $\boldsymbol{B} \parallel [010]$
C_c2 (5.16) $\mathbb{Z}_1$	• strain $\perp [010]$<br>• $\boldsymbol{E} \parallel [010]$
$P2'$ (3.3) $\mathbb{Z}_1$	• $\boldsymbol{B} \parallel [100]$ and strain $\perp [001]$<br>• $\boldsymbol{B} \parallel [010]$ and strain $\perp [001]$<br>• $\boldsymbol{B} \perp [001]$<br>• $\boldsymbol{E} \parallel [001]$ and $\boldsymbol{B} \parallel [100]$<br>• $\boldsymbol{E} \parallel [001]$ and $\boldsymbol{B} \parallel [010]$
$C22'2'$ (21.41) $\mathbb{Z}_1$	• $\boldsymbol{B} \parallel [100]$
$C22'2'$ (21.41) $\mathbb{Z}_1$	• $\boldsymbol{B} \parallel [010]$
$P2_1$ (4.7) $\mathbb{Z}_1$	• $\boldsymbol{B} \parallel [001]$ and strain $\perp [001]$<br>• $\boldsymbol{E} \parallel [001]$ and $\boldsymbol{B} \parallel [001]$
$C2'2'2_1$ (20.33) $\mathbb{Z}_1$	• $\boldsymbol{B} \parallel [001]$
P_C2_1 (4.12) $\mathbb{Z}_1$	• strain $\perp [001]$<br>• $\boldsymbol{E} \parallel [001]$

Table. S 148: Subgroups of $C222$ (21.38) that can be realized by applying $\boldsymbol{E}/\boldsymbol{B}$ fields and/or uniaxial strains.

Subgroup & SI group	Perturbation
$C2$ (5.13) $\mathbb{Z}_1$	• strain $\perp [100]$<br>• $\boldsymbol{B} \parallel [100]$<br>• $\boldsymbol{E} \parallel [100]$
$C2$ (5.13) $\mathbb{Z}_1$	• strain $\perp [010]$<br>• $\boldsymbol{B} \parallel [010]$<br>• $\boldsymbol{E} \parallel [010]$

Continued on next page

. . . continued

Subgroup & SI group	Perturbation
$P2$ (3.1) $\mathbb{Z}_2$	• strain $\perp$ [001]<br>• $\boldsymbol{B} \parallel$ [001]<br>• $\boldsymbol{E} \parallel$ [001]

Table. S 149: Subgroups of $C2221'$ (21.39) that can be realized by applying $\boldsymbol{E}/\boldsymbol{B}$ fields and/or uniaxial strains.

Subgroup & SI group	Perturbation
$P11'$ (1.2) $\mathbb{Z}_1$	• strain in generic direction<br>• $\boldsymbol{E} \parallel$ [100] and strain $\perp$ [010]<br>• $\boldsymbol{E} \parallel$ [100] and strain $\perp$ [001]<br>• $\boldsymbol{E} \parallel$ [010] and strain $\perp$ [100]<br>• $\boldsymbol{E} \parallel$ [010] and strain $\perp$ [001]<br>• $\boldsymbol{E} \parallel$ [001] and strain $\perp$ [100]<br>• $\boldsymbol{E} \parallel$ [001] and strain $\perp$ [010]<br>• $\boldsymbol{E}$ in generic direction
$C2'$ (5.15) $\mathbb{Z}_1$	• $\boldsymbol{B} \parallel$ [010] and strain $\perp$ [100]<br>• $\boldsymbol{B} \parallel$ [001] and strain $\perp$ [100]<br>• $\boldsymbol{B} \perp$ [100]<br>• $\boldsymbol{E} \parallel$ [100] and $\boldsymbol{B} \parallel$ [010]<br>• $\boldsymbol{E} \parallel$ [100] and $\boldsymbol{B} \parallel$ [001]
$C2$ (5.13) $\mathbb{Z}_1$	• $\boldsymbol{B} \parallel$ [100] and strain $\perp$ [100]<br>• $\boldsymbol{E} \parallel$ [100] and $\boldsymbol{B} \parallel$ [100]
$C21'$ (5.14) $\mathbb{Z}_1$	• strain $\perp$ [100]<br>• $\boldsymbol{E} \parallel$ [100]
$C2'$ (5.15) $\mathbb{Z}_1$	• $\boldsymbol{B} \parallel$ [100] and strain $\perp$ [010]<br>• $\boldsymbol{B} \parallel$ [001] and strain $\perp$ [010]<br>• $\boldsymbol{B} \perp$ [010]<br>• $\boldsymbol{E} \parallel$ [010] and $\boldsymbol{B} \parallel$ [100]<br>• $\boldsymbol{E} \parallel$ [010] and $\boldsymbol{B} \parallel$ [001]
$C2$ (5.13) $\mathbb{Z}_1$	• $\boldsymbol{B} \parallel$ [010] and strain $\perp$ [010]<br>• $\boldsymbol{E} \parallel$ [010] and $\boldsymbol{B} \parallel$ [010]
$C21'$ (5.14) $\mathbb{Z}_1$	• strain $\perp$ [010]<br>• $\boldsymbol{E} \parallel$ [010]

Continued on next page

...continued

Subgroup & SI group	Perturbation
$P2'$ (3.3) $\mathbb{Z}_1$	• $\boldsymbol{B}$ ∥ [100] and strain ⊥ [001]<br>• $\boldsymbol{B}$ ∥ [010] and strain ⊥ [001]<br>• $\boldsymbol{B}$ ⊥ [001]<br>• $\boldsymbol{E}$ ∥ [001] and $\boldsymbol{B}$ ∥ [100]<br>• $\boldsymbol{E}$ ∥ [001] and $\boldsymbol{B}$ ∥ [010]
$C22'2'$ (21.41) $\mathbb{Z}_1$	• $\boldsymbol{B}$ ∥ [100]
$C22'2'$ (21.41) $\mathbb{Z}_1$	• $\boldsymbol{B}$ ∥ [010]
$P2$ (3.1) $\mathbb{Z}_2$	• $\boldsymbol{B}$ ∥ [001] and strain ⊥ [001]<br>• $\boldsymbol{E}$ ∥ [001] and $\boldsymbol{B}$ ∥ [001]
$C2'2'2$ (21.40) $\mathbb{Z}_2$	• $\boldsymbol{B}$ ∥ [001]
$P21'$ (3.2) $\mathbb{Z}_2$	• strain ⊥ [001]<br>• $\boldsymbol{E}$ ∥ [001]

Table. S 150: Subgroups of $C2'2'2$ (21.40) that can be realized by applying $\boldsymbol{E}/\boldsymbol{B}$ fields and/or uniaxial strains.

Subgroup & SI group	Perturbation
$C2'$ (5.15) $\mathbb{Z}_1$	• strain ⊥ [100]<br>• ($\boldsymbol{B}$ ∥ [010] or $\boldsymbol{B}$ ⊥ [100])<br>• $\boldsymbol{E}$ ∥ [100]
$C2'$ (5.15) $\mathbb{Z}_1$	• strain ⊥ [010]<br>• ($\boldsymbol{B}$ ∥ [100] or $\boldsymbol{B}$ ⊥ [010])<br>• $\boldsymbol{E}$ ∥ [010]
$P2$ (3.1) $\mathbb{Z}_2$	• strain ⊥ [001]<br>• $\boldsymbol{E}$ ∥ [001]

Table. S 151: Subgroups of $C22'2'$ (21.41) that can be realized by applying $\boldsymbol{E}/\boldsymbol{B}$ fields and/or uniaxial strains.

Subgroup & SI group	Perturbation

Continued on next page

. . . continued

Subgroup & SI group	Perturbation
$C2$ (5.13) $\mathbb{Z}_1$	• strain $\perp$ [100]<br>• $\boldsymbol{E}$ $\parallel$ [100]
$C2'$ (5.15) $\mathbb{Z}_1$	• strain $\perp$ [010]<br>• ($\boldsymbol{B}$ $\parallel$ [001] or $\boldsymbol{B}$ $\perp$ [010])<br>• $\boldsymbol{E}$ $\parallel$ [010]
$P2'$ (3.3) $\mathbb{Z}_1$	• strain $\perp$ [001]<br>• ($\boldsymbol{B}$ $\parallel$ [010] or $\boldsymbol{B}$ $\perp$ [001])<br>• $\boldsymbol{E}$ $\parallel$ [001]

Table. S 152: Subgroups of C_c222 (21.42) that can be realized by applying $\boldsymbol{E}/\boldsymbol{B}$ fields and/or uniaxial strains.

Subgroup & SI group	Perturbation
P_S1 (1.3) $\mathbb{Z}_1$	• strain in generic direction<br>• $\boldsymbol{E}$ $\parallel$ [100] and strain $\perp$ [010]<br>• $\boldsymbol{E}$ $\parallel$ [100] and strain $\perp$ [001]<br>• $\boldsymbol{E}$ $\parallel$ [010] and strain $\perp$ [100]<br>• $\boldsymbol{E}$ $\parallel$ [010] and strain $\perp$ [001]<br>• $\boldsymbol{E}$ $\parallel$ [001] and strain $\perp$ [100]<br>• $\boldsymbol{E}$ $\parallel$ [001] and strain $\perp$ [010]<br>• $\boldsymbol{E}$ in generic direction
$C2$ (5.13) $\mathbb{Z}_1$	• $\boldsymbol{B}$ $\parallel$ [100] and strain $\perp$ [100]<br>• $\boldsymbol{E}$ $\parallel$ [100] and $\boldsymbol{B}$ $\parallel$ [100]
$C2'$ (5.15) $\mathbb{Z}_1$	• $\boldsymbol{B}$ $\parallel$ [010] and strain $\perp$ [100]<br>• $\boldsymbol{B}$ $\parallel$ [001] and strain $\perp$ [100]<br>• $\boldsymbol{B}$ $\perp$ [100]<br>• $\boldsymbol{E}$ $\parallel$ [100] and $\boldsymbol{B}$ $\parallel$ [010]<br>• $\boldsymbol{E}$ $\parallel$ [100] and $\boldsymbol{B}$ $\parallel$ [001]
C_c2 (5.16) $\mathbb{Z}_1$	• strain $\perp$ [100]<br>• $\boldsymbol{E}$ $\parallel$ [100]
$C2$ (5.13) $\mathbb{Z}_1$	• $\boldsymbol{B}$ $\parallel$ [010] and strain $\perp$ [010]<br>• $\boldsymbol{E}$ $\parallel$ [010] and $\boldsymbol{B}$ $\parallel$ [010]

Continued on next page

...continued

Subgroup & SI group	Perturbation
$C2'$ (5.15) $\mathbb{Z}_1$	• $\boldsymbol{B} \parallel [100]$ and strain $\perp [010]$<br>• $\boldsymbol{B} \parallel [001]$ and strain $\perp [010]$<br>• $\boldsymbol{B} \perp [010]$<br>• $\boldsymbol{E} \parallel [010]$ and $\boldsymbol{B} \parallel [100]$<br>• $\boldsymbol{E} \parallel [010]$ and $\boldsymbol{B} \parallel [001]$
C_c2 (5.16) $\mathbb{Z}_1$	• strain $\perp [010]$<br>• $\boldsymbol{E} \parallel [010]$
$P2$ (3.1) $\mathbb{Z}_2$	• $\boldsymbol{B} \parallel [001]$ and strain $\perp [001]$<br>• $\boldsymbol{E} \parallel [001]$ and $\boldsymbol{B} \parallel [001]$
$C2'2'2$ (21.40) $\mathbb{Z}_2$	• $\boldsymbol{B} \parallel [001]$
$P2_1'$ (4.9) $\mathbb{Z}_1$	• $\boldsymbol{B} \parallel [100]$ and strain $\perp [001]$<br>• $\boldsymbol{B} \parallel [010]$ and strain $\perp [001]$<br>• $\boldsymbol{B} \perp [001]$<br>• $\boldsymbol{E} \parallel [001]$ and $\boldsymbol{B} \parallel [100]$<br>• $\boldsymbol{E} \parallel [001]$ and $\boldsymbol{B} \parallel [010]$
$C22'2_1'$ (20.34) $\mathbb{Z}_1$	• $\boldsymbol{B} \parallel [010]$
$C22'2_1'$ (20.34) $\mathbb{Z}_1$	• $\boldsymbol{B} \parallel [100]$
P_b2 (3.5) $\mathbb{Z}_2$	• strain $\perp [001]$<br>• $\boldsymbol{E} \parallel [001]$

Table. S 153: Subgroups of C_a222 (21.43) that can be realized by applying $\boldsymbol{E}/\boldsymbol{B}$ fields and/or uniaxial strains.

Subgroup & SI group	Perturbation
P_S1 (1.3) $\mathbb{Z}_1$	• strain in generic direction<br>• $\boldsymbol{E} \parallel [100]$ and strain $\perp [010]$<br>• $\boldsymbol{E} \parallel [100]$ and strain $\perp [001]$<br>• $\boldsymbol{E} \parallel [010]$ and strain $\perp [100]$<br>• $\boldsymbol{E} \parallel [010]$ and strain $\perp [001]$<br>• $\boldsymbol{E} \parallel [001]$ and strain $\perp [100]$<br>• $\boldsymbol{E} \parallel [001]$ and strain $\perp [010]$<br>• $\boldsymbol{E}$ in generic direction

Continued on next page

...continued

Subgroup & SI group	Perturbation
$C2'$ (5.15) $\mathbb{Z}_1$	• $\boldsymbol{B} \parallel [010]$ and strain $\perp [100]$<br>• $\boldsymbol{B} \parallel [001]$ and strain $\perp [100]$<br>• $\boldsymbol{B} \perp [100]$<br>• $\boldsymbol{E} \parallel [100]$ and $\boldsymbol{B} \parallel [010]$<br>• $\boldsymbol{E} \parallel [100]$ and $\boldsymbol{B} \parallel [001]$
$C2$ (5.13) $\mathbb{Z}_1$	• $\boldsymbol{B} \parallel [100]$ and strain $\perp [100]$<br>• $\boldsymbol{E} \parallel [100]$ and $\boldsymbol{B} \parallel [100]$
C_a2 (5.17) $\mathbb{Z}_2$	• strain $\perp [100]$<br>• $\boldsymbol{E} \parallel [100]$
$C2'$ (5.15) $\mathbb{Z}_1$	• $\boldsymbol{B} \parallel [100]$ and strain $\perp [010]$<br>• $\boldsymbol{B} \parallel [001]$ and strain $\perp [010]$<br>• $\boldsymbol{B} \perp [010]$<br>• $\boldsymbol{E} \parallel [010]$ and $\boldsymbol{B} \parallel [100]$<br>• $\boldsymbol{E} \parallel [010]$ and $\boldsymbol{B} \parallel [001]$
$C2$ (5.13) $\mathbb{Z}_1$	• $\boldsymbol{B} \parallel [010]$ and strain $\perp [010]$<br>• $\boldsymbol{E} \parallel [010]$ and $\boldsymbol{B} \parallel [010]$
C_a2 (5.17) $\mathbb{Z}_2$	• strain $\perp [010]$<br>• $\boldsymbol{E} \parallel [010]$
$P2'$ (3.3) $\mathbb{Z}_1$	• $\boldsymbol{B} \parallel [100]$ and strain $\perp [001]$<br>• $\boldsymbol{B} \parallel [010]$ and strain $\perp [001]$<br>• $\boldsymbol{B} \perp [001]$<br>• $\boldsymbol{E} \parallel [001]$ and $\boldsymbol{B} \parallel [100]$<br>• $\boldsymbol{E} \parallel [001]$ and $\boldsymbol{B} \parallel [010]$
$C22'2'$ (21.41) $\mathbb{Z}_1$	• $\boldsymbol{B} \parallel [100]$
$C22'2'$ (21.41) $\mathbb{Z}_1$	• $\boldsymbol{B} \parallel [010]$
$P2$ (3.1) $\mathbb{Z}_2$	• $\boldsymbol{B} \parallel [001]$ and strain $\perp [001]$<br>• $\boldsymbol{E} \parallel [001]$ and $\boldsymbol{B} \parallel [001]$
$C2'2'2$ (21.40) $\mathbb{Z}_2$	• $\boldsymbol{B} \parallel [001]$
P_a2 (3.4) $\mathbb{Z}_2$	• strain $\perp [001]$<br>• $\boldsymbol{E} \parallel [001]$

Table. S 154: Subgroups of C_A222 (21.44) that can be realized by applying $\boldsymbol{E}/\boldsymbol{B}$ fields and/or uniaxial strains.

Subgroup & SI group	Perturbation
P_S1 (1.3) $\mathbb{Z}_1$	• strain in generic direction<br>• $\boldsymbol{E} \parallel [100]$ and strain $\perp [010]$<br>• $\boldsymbol{E} \parallel [100]$ and strain $\perp [001]$<br>• $\boldsymbol{E} \parallel [010]$ and strain $\perp [100]$<br>• $\boldsymbol{E} \parallel [010]$ and strain $\perp [001]$<br>• $\boldsymbol{E} \parallel [001]$ and strain $\perp [100]$<br>• $\boldsymbol{E} \parallel [001]$ and strain $\perp [010]$<br>• $\boldsymbol{E}$ in generic direction
$C2'$ (5.15) $\mathbb{Z}_1$	• $\boldsymbol{B} \parallel [010]$ and strain $\perp [100]$<br>• $\boldsymbol{B} \parallel [001]$ and strain $\perp [100]$<br>• $\boldsymbol{B} \perp [100]$<br>• $\boldsymbol{E} \parallel [100]$ and $\boldsymbol{B} \parallel [010]$<br>• $\boldsymbol{E} \parallel [100]$ and $\boldsymbol{B} \parallel [001]$
$C2$ (5.13) $\mathbb{Z}_1$	• $\boldsymbol{B} \parallel [100]$ and strain $\perp [100]$<br>• $\boldsymbol{E} \parallel [100]$ and $\boldsymbol{B} \parallel [100]$
C_c2 (5.16) $\mathbb{Z}_1$	• strain $\perp [100]$<br>• $\boldsymbol{E} \parallel [100]$
$C2'$ (5.15) $\mathbb{Z}_1$	• $\boldsymbol{B} \parallel [100]$ and strain $\perp [010]$<br>• $\boldsymbol{B} \parallel [001]$ and strain $\perp [010]$<br>• $\boldsymbol{B} \perp [010]$<br>• $\boldsymbol{E} \parallel [010]$ and $\boldsymbol{B} \parallel [100]$<br>• $\boldsymbol{E} \parallel [010]$ and $\boldsymbol{B} \parallel [001]$
$C2$ (5.13) $\mathbb{Z}_1$	• $\boldsymbol{B} \parallel [010]$ and strain $\perp [010]$<br>• $\boldsymbol{E} \parallel [010]$ and $\boldsymbol{B} \parallel [010]$
C_c2 (5.16) $\mathbb{Z}_1$	• strain $\perp [010]$<br>• $\boldsymbol{E} \parallel [010]$
$P2_1'$ (4.9) $\mathbb{Z}_1$	• $\boldsymbol{B} \parallel [100]$ and strain $\perp [001]$<br>• $\boldsymbol{B} \parallel [010]$ and strain $\perp [001]$<br>• $\boldsymbol{B} \perp [001]$<br>• $\boldsymbol{E} \parallel [001]$ and $\boldsymbol{B} \parallel [100]$<br>• $\boldsymbol{E} \parallel [001]$ and $\boldsymbol{B} \parallel [010]$
$C22'2_1'$ (20.34) $\mathbb{Z}_1$	• $\boldsymbol{B} \parallel [100]$
$C22'2_1'$ (20.34) $\mathbb{Z}_1$	• $\boldsymbol{B} \parallel [010]$
$P2$ (3.1) $\mathbb{Z}_2$	• $\boldsymbol{B} \parallel [001]$ and strain $\perp [001]$<br>• $\boldsymbol{E} \parallel [001]$ and $\boldsymbol{B} \parallel [001]$

Continued on next page

...continued

Subgroup & SI group	Perturbation
$C2'2'2$ (21.40) $\mathbb{Z}_2$	• $\boldsymbol{B} \parallel [001]$
P_C2 (3.6) $\mathbb{Z}_1$	• strain $\perp [001]$<br>• $\boldsymbol{E} \parallel [001]$

Table. S 155: Subgroups of $F222$ (22.45) that can be realized by applying $\boldsymbol{E}/\boldsymbol{B}$ fields and/or uniaxial strains.

Subgroup & SI group	Perturbation
$C2$ (5.13) $\mathbb{Z}_1$	• strain $\perp [100]$<br>• $\boldsymbol{B} \parallel [100]$<br>• $\boldsymbol{E} \parallel [100]$
$C2$ (5.13) $\mathbb{Z}_1$	• strain $\perp [010]$<br>• $\boldsymbol{B} \parallel [010]$<br>• $\boldsymbol{E} \parallel [010]$
$C2$ (5.13) $\mathbb{Z}_1$	• strain $\perp [001]$<br>• $\boldsymbol{B} \parallel [001]$<br>• $\boldsymbol{E} \parallel [001]$

Table. S 156: Subgroups of $F2221'$ (22.46) that can be realized by applying $\boldsymbol{E}/\boldsymbol{B}$ fields and/or uniaxial strains.

Subgroup & SI group	Perturbation
$P11'$ (1.2) $\mathbb{Z}_1$	• strain in generic direction<br>• $\boldsymbol{E} \parallel [100]$ and strain $\perp [010]$<br>• $\boldsymbol{E} \parallel [100]$ and strain $\perp [001]$<br>• $\boldsymbol{E} \parallel [010]$ and strain $\perp [100]$<br>• $\boldsymbol{E} \parallel [010]$ and strain $\perp [001]$<br>• $\boldsymbol{E} \parallel [001]$ and strain $\perp [100]$<br>• $\boldsymbol{E} \parallel [001]$ and strain $\perp [010]$<br>• $\boldsymbol{E}$ in generic direction

Continued on next page

...continued

Subgroup & SI group	Perturbation
$C2'$ (5.15) $\mathbb{Z}_1$	• $\boldsymbol{B} \parallel [010]$ and strain $\perp [100]$<br>• $\boldsymbol{B} \parallel [001]$ and strain $\perp [100]$<br>• $\boldsymbol{B} \perp [100]$<br>• $\boldsymbol{E} \parallel [100]$ and $\boldsymbol{B} \parallel [010]$<br>• $\boldsymbol{E} \parallel [100]$ and $\boldsymbol{B} \parallel [001]$
$C2$ (5.13) $\mathbb{Z}_1$	• $\boldsymbol{B} \parallel [100]$ and strain $\perp [100]$<br>• $\boldsymbol{E} \parallel [100]$ and $\boldsymbol{B} \parallel [100]$
$C21'$ (5.14) $\mathbb{Z}_1$	• strain $\perp [100]$<br>• $\boldsymbol{E} \parallel [100]$
$C2'$ (5.15) $\mathbb{Z}_1$	• $\boldsymbol{B} \parallel [100]$ and strain $\perp [010]$<br>• $\boldsymbol{B} \parallel [001]$ and strain $\perp [010]$<br>• $\boldsymbol{B} \perp [010]$<br>• $\boldsymbol{E} \parallel [010]$ and $\boldsymbol{B} \parallel [100]$<br>• $\boldsymbol{E} \parallel [010]$ and $\boldsymbol{B} \parallel [001]$
$C2$ (5.13) $\mathbb{Z}_1$	• $\boldsymbol{B} \parallel [010]$ and strain $\perp [010]$<br>• $\boldsymbol{E} \parallel [010]$ and $\boldsymbol{B} \parallel [010]$
$C21'$ (5.14) $\mathbb{Z}_1$	• strain $\perp [010]$<br>• $\boldsymbol{E} \parallel [010]$
$C2'$ (5.15) $\mathbb{Z}_1$	• $\boldsymbol{B} \parallel [100]$ and strain $\perp [001]$<br>• $\boldsymbol{B} \parallel [010]$ and strain $\perp [001]$<br>• $\boldsymbol{B} \perp [001]$<br>• $\boldsymbol{E} \parallel [001]$ and $\boldsymbol{B} \parallel [100]$<br>• $\boldsymbol{E} \parallel [001]$ and $\boldsymbol{B} \parallel [010]$
$F2'2'2$ (22.47) $\mathbb{Z}_1$	• $\boldsymbol{B} \parallel [100]$
$F2'2'2$ (22.47) $\mathbb{Z}_1$	• $\boldsymbol{B} \parallel [010]$
$C2$ (5.13) $\mathbb{Z}_1$	• $\boldsymbol{B} \parallel [001]$ and strain $\perp [001]$<br>• $\boldsymbol{E} \parallel [001]$ and $\boldsymbol{B} \parallel [001]$
$F2'2'2$ (22.47) $\mathbb{Z}_1$	• $\boldsymbol{B} \parallel [001]$
$C21'$ (5.14) $\mathbb{Z}_1$	• strain $\perp [001]$<br>• $\boldsymbol{E} \parallel [001]$

Table. S 157: Subgroups of $F2'2'2$ (22.47) that can be realized by applying $\boldsymbol{E}/\boldsymbol{B}$ fields and/or uniaxial strains.

Subgroup & SI group	Perturbation
$C2'$ (5.15) $\mathbb{Z}_1$	• strain $\perp$ [100]<br>• ($\boldsymbol{B}$ $\parallel$ [010] or $\boldsymbol{B}$ $\perp$ [100])<br>• $\boldsymbol{E}$ $\parallel$ [100]
$C2'$ (5.15) $\mathbb{Z}_1$	• strain $\perp$ [010]<br>• ($\boldsymbol{B}$ $\parallel$ [100] or $\boldsymbol{B}$ $\perp$ [010])<br>• $\boldsymbol{E}$ $\parallel$ [010]
$C2$ (5.13) $\mathbb{Z}_1$	• strain $\perp$ [001]<br>• $\boldsymbol{E}$ $\parallel$ [001]

Table. S 158: Subgroups of F_S222 (22.48) that can be realized by applying $\boldsymbol{E}/\boldsymbol{B}$ fields and/or uniaxial strains.

Subgroup & SI group	Perturbation
P_S1 (1.3) $\mathbb{Z}_1$	• strain in generic direction<br>• $\boldsymbol{E}$ $\parallel$ [100] and strain $\perp$ [010]<br>• $\boldsymbol{E}$ $\parallel$ [100] and strain $\perp$ [001]<br>• $\boldsymbol{E}$ $\parallel$ [010] and strain $\perp$ [100]<br>• $\boldsymbol{E}$ $\parallel$ [010] and strain $\perp$ [001]<br>• $\boldsymbol{E}$ $\parallel$ [001] and strain $\perp$ [100]<br>• $\boldsymbol{E}$ $\parallel$ [001] and strain $\perp$ [010]<br>• $\boldsymbol{E}$ in generic direction
$C2$ (5.13) $\mathbb{Z}_1$	• $\boldsymbol{B}$ $\parallel$ [100] and strain $\perp$ [100]<br>• $\boldsymbol{E}$ $\parallel$ [100] and $\boldsymbol{B}$ $\parallel$ [100]
$C2'$ (5.15) $\mathbb{Z}_1$	• $\boldsymbol{B}$ $\parallel$ [010] and strain $\perp$ [100]<br>• $\boldsymbol{B}$ $\parallel$ [001] and strain $\perp$ [100]<br>• $\boldsymbol{B}$ $\perp$ [100]<br>• $\boldsymbol{E}$ $\parallel$ [100] and $\boldsymbol{B}$ $\parallel$ [010]<br>• $\boldsymbol{E}$ $\parallel$ [100] and $\boldsymbol{B}$ $\parallel$ [001]
C_a2 (5.17) $\mathbb{Z}_2$	• strain $\perp$ [100]<br>• $\boldsymbol{E}$ $\parallel$ [100]
$C2$ (5.13) $\mathbb{Z}_1$	• $\boldsymbol{B}$ $\parallel$ [010] and strain $\perp$ [010]<br>• $\boldsymbol{E}$ $\parallel$ [010] and $\boldsymbol{B}$ $\parallel$ [010]

Continued on next page

…continued

Subgroup & SI group	Perturbation
$C2'$ (5.15) $\mathbb{Z}_1$	• $\boldsymbol{B} \parallel [100]$ and strain $\perp [010]$<br>• $\boldsymbol{B} \parallel [001]$ and strain $\perp [010]$<br>• $\boldsymbol{B} \perp [010]$<br>• $\boldsymbol{E} \parallel [010]$ and $\boldsymbol{B} \parallel [100]$<br>• $\boldsymbol{E} \parallel [010]$ and $\boldsymbol{B} \parallel [001]$
C_a2 (5.17) $\mathbb{Z}_2$	• strain $\perp [010]$<br>• $\boldsymbol{E} \parallel [010]$
$C2$ (5.13) $\mathbb{Z}_1$	• $\boldsymbol{B} \parallel [001]$ and strain $\perp [001]$<br>• $\boldsymbol{E} \parallel [001]$ and $\boldsymbol{B} \parallel [001]$
$F2'2'2$ (22.47) $\mathbb{Z}_1$	• $\boldsymbol{B} \parallel [001]$
$C2'$ (5.15) $\mathbb{Z}_1$	• $\boldsymbol{B} \parallel [100]$ and strain $\perp [001]$<br>• $\boldsymbol{B} \parallel [010]$ and strain $\perp [001]$<br>• $\boldsymbol{B} \perp [001]$<br>• $\boldsymbol{E} \parallel [001]$ and $\boldsymbol{B} \parallel [100]$<br>• $\boldsymbol{E} \parallel [001]$ and $\boldsymbol{B} \parallel [010]$
$F2'2'2$ (22.47) $\mathbb{Z}_1$	• $\boldsymbol{B} \parallel [010]$
$F2'2'2$ (22.47) $\mathbb{Z}_1$	• $\boldsymbol{B} \parallel [100]$
C_a2 (5.17) $\mathbb{Z}_2$	• strain $\perp [001]$<br>• $\boldsymbol{E} \parallel [001]$

Table. S 159: Subgroups of $I222$ (23.49) that can be realized by applying $\boldsymbol{E}/\boldsymbol{B}$ fields and/or uniaxial strains.

Subgroup & SI group	Perturbation
$C2$ (5.13) $\mathbb{Z}_1$	• strain $\perp [100]$<br>• $\boldsymbol{B} \parallel [100]$<br>• $\boldsymbol{E} \parallel [100]$
$C2$ (5.13) $\mathbb{Z}_1$	• strain $\perp [010]$<br>• $\boldsymbol{B} \parallel [010]$<br>• $\boldsymbol{E} \parallel [010]$
$C2$ (5.13) $\mathbb{Z}_1$	• strain $\perp [001]$<br>• $\boldsymbol{B} \parallel [001]$<br>• $\boldsymbol{E} \parallel [001]$

Table. S 160: Subgroups of $I2221'$ (23.50) that can be realized by applying $\boldsymbol{E}/\boldsymbol{B}$ fields and/or uniaxial strains.

Subgroup & SI group	Perturbation
$P11'$ (1.2) $\mathbb{Z}_1$	• strain in generic direction<br>• $\boldsymbol{E}$ ∥ [100] and strain ⊥ [010]<br>• $\boldsymbol{E}$ ∥ [100] and strain ⊥ [001]<br>• $\boldsymbol{E}$ ∥ [010] and strain ⊥ [100]<br>• $\boldsymbol{E}$ ∥ [010] and strain ⊥ [001]<br>• $\boldsymbol{E}$ ∥ [001] and strain ⊥ [100]<br>• $\boldsymbol{E}$ ∥ [001] and strain ⊥ [010]<br>• $\boldsymbol{E}$ in generic direction
$C2'$ (5.15) $\mathbb{Z}_1$	• $\boldsymbol{B}$ ∥ [010] and strain ⊥ [100]<br>• $\boldsymbol{B}$ ∥ [001] and strain ⊥ [100]<br>• $\boldsymbol{B}$ ⊥ [100]<br>• $\boldsymbol{E}$ ∥ [100] and $\boldsymbol{B}$ ∥ [010]<br>• $\boldsymbol{E}$ ∥ [100] and $\boldsymbol{B}$ ∥ [001]
$C2$ (5.13) $\mathbb{Z}_1$	• $\boldsymbol{B}$ ∥ [100] and strain ⊥ [100]<br>• $\boldsymbol{E}$ ∥ [100] and $\boldsymbol{B}$ ∥ [100]
$C21'$ (5.14) $\mathbb{Z}_1$	• strain ⊥ [100]<br>• $\boldsymbol{E}$ ∥ [100]
$C2'$ (5.15) $\mathbb{Z}_1$	• $\boldsymbol{B}$ ∥ [100] and strain ⊥ [010]<br>• $\boldsymbol{B}$ ∥ [001] and strain ⊥ [010]<br>• $\boldsymbol{B}$ ⊥ [010]<br>• $\boldsymbol{E}$ ∥ [010] and $\boldsymbol{B}$ ∥ [100]<br>• $\boldsymbol{E}$ ∥ [010] and $\boldsymbol{B}$ ∥ [001]
$C2$ (5.13) $\mathbb{Z}_1$	• $\boldsymbol{B}$ ∥ [010] and strain ⊥ [010]<br>• $\boldsymbol{E}$ ∥ [010] and $\boldsymbol{B}$ ∥ [010]
$C21'$ (5.14) $\mathbb{Z}_1$	• strain ⊥ [010]<br>• $\boldsymbol{E}$ ∥ [010]
$C2'$ (5.15) $\mathbb{Z}_1$	• $\boldsymbol{B}$ ∥ [100] and strain ⊥ [001]<br>• $\boldsymbol{B}$ ∥ [010] and strain ⊥ [001]<br>• $\boldsymbol{B}$ ⊥ [001]<br>• $\boldsymbol{E}$ ∥ [001] and $\boldsymbol{B}$ ∥ [100]<br>• $\boldsymbol{E}$ ∥ [001] and $\boldsymbol{B}$ ∥ [010]
$I2'2'2$ (23.51) $\mathbb{Z}_1$	• $\boldsymbol{B}$ ∥ [100]
$I2'2'2$ (23.51) $\mathbb{Z}_1$	• $\boldsymbol{B}$ ∥ [010]
$C2$ (5.13) $\mathbb{Z}_1$	• $\boldsymbol{B}$ ∥ [001] and strain ⊥ [001]<br>• $\boldsymbol{E}$ ∥ [001] and $\boldsymbol{B}$ ∥ [001]

Continued on next page

...continued

Subgroup & SI group	Perturbation
$I2'2'2$ (23.51) $\mathbb{Z}_1$	• $\boldsymbol{B} \parallel [001]$
$C21'$ (5.14) $\mathbb{Z}_1$	• strain $\perp [001]$<br>• $\boldsymbol{E} \parallel [001]$

Table. S 161: Subgroups of $I2'2'2$ (23.51) that can be realized by applying $\boldsymbol{E}/\boldsymbol{B}$ fields and/or uniaxial strains.

Subgroup & SI group	Perturbation
$C2'$ (5.15) $\mathbb{Z}_1$	• strain $\perp [100]$<br>• ($\boldsymbol{B} \parallel [010]$ or $\boldsymbol{B} \perp [100]$)<br>• $\boldsymbol{E} \parallel [100]$
$C2'$ (5.15) $\mathbb{Z}_1$	• strain $\perp [010]$<br>• ($\boldsymbol{B} \parallel [100]$ or $\boldsymbol{B} \perp [010]$)<br>• $\boldsymbol{E} \parallel [010]$
$C2$ (5.13) $\mathbb{Z}_1$	• strain $\perp [001]$<br>• $\boldsymbol{E} \parallel [001]$

Table. S 162: Subgroups of I_c222 (23.52) that can be realized by applying $\boldsymbol{E}/\boldsymbol{B}$ fields and/or uniaxial strains.

Subgroup & SI group	Perturbation
P_S1 (1.3) $\mathbb{Z}_1$	• strain in generic direction<br>• $\boldsymbol{E} \parallel [100]$ and strain $\perp [010]$<br>• $\boldsymbol{E} \parallel [100]$ and strain $\perp [001]$<br>• $\boldsymbol{E} \parallel [010]$ and strain $\perp [100]$<br>• $\boldsymbol{E} \parallel [010]$ and strain $\perp [001]$<br>• $\boldsymbol{E} \parallel [001]$ and strain $\perp [100]$<br>• $\boldsymbol{E} \parallel [001]$ and strain $\perp [010]$<br>• $\boldsymbol{E}$ in generic direction

Continued on next page

…continued

Subgroup & SI group	Perturbation
$C2'$ (5.15) $\mathbb{Z}_1$	• $\boldsymbol{B}$ ∥ [010] and strain ⊥ [100]<br>• $\boldsymbol{B}$ ∥ [001] and strain ⊥ [100]<br>• $\boldsymbol{B}$ ⊥ [100]<br>• $\boldsymbol{E}$ ∥ [100] and $\boldsymbol{B}$ ∥ [010]<br>• $\boldsymbol{E}$ ∥ [100] and $\boldsymbol{B}$ ∥ [001]
$C2$ (5.13) $\mathbb{Z}_1$	• $\boldsymbol{B}$ ∥ [100] and strain ⊥ [100]<br>• $\boldsymbol{E}$ ∥ [100] and $\boldsymbol{B}$ ∥ [100]
C_c2 (5.16) $\mathbb{Z}_1$	• strain ⊥ [100]<br>• $\boldsymbol{E}$ ∥ [100]
$C2'$ (5.15) $\mathbb{Z}_1$	• $\boldsymbol{B}$ ∥ [100] and strain ⊥ [010]<br>• $\boldsymbol{B}$ ∥ [001] and strain ⊥ [010]<br>• $\boldsymbol{B}$ ⊥ [010]<br>• $\boldsymbol{E}$ ∥ [010] and $\boldsymbol{B}$ ∥ [100]<br>• $\boldsymbol{E}$ ∥ [010] and $\boldsymbol{B}$ ∥ [001]
$C2$ (5.13) $\mathbb{Z}_1$	• $\boldsymbol{B}$ ∥ [010] and strain ⊥ [010]<br>• $\boldsymbol{E}$ ∥ [010] and $\boldsymbol{B}$ ∥ [010]
C_c2 (5.16) $\mathbb{Z}_1$	• strain ⊥ [010]<br>• $\boldsymbol{E}$ ∥ [010]
$C2'$ (5.15) $\mathbb{Z}_1$	• $\boldsymbol{B}$ ∥ [100] and strain ⊥ [001]<br>• $\boldsymbol{B}$ ∥ [010] and strain ⊥ [001]<br>• $\boldsymbol{B}$ ⊥ [001]<br>• $\boldsymbol{E}$ ∥ [001] and $\boldsymbol{B}$ ∥ [100]<br>• $\boldsymbol{E}$ ∥ [001] and $\boldsymbol{B}$ ∥ [010]
$I2_1'2_1'2_1$ (24.55) $\mathbb{Z}_1$	• $\boldsymbol{B}$ ∥ [100]
$I2_1'2_1'2_1$ (24.55) $\mathbb{Z}_1$	• $\boldsymbol{B}$ ∥ [010]
$C2$ (5.13) $\mathbb{Z}_1$	• $\boldsymbol{B}$ ∥ [001] and strain ⊥ [001]<br>• $\boldsymbol{E}$ ∥ [001] and $\boldsymbol{B}$ ∥ [001]
$I2'2'2$ (23.51) $\mathbb{Z}_1$	• $\boldsymbol{B}$ ∥ [001]
C_a2 (5.17) $\mathbb{Z}_2$	• strain ⊥ [001]<br>• $\boldsymbol{E}$ ∥ [001]

Table. S 163: Subgroups of $I2_12_12_1$ (24.53) that can be realized by applying $\boldsymbol{E}/\boldsymbol{B}$ fields and/or uniaxial strains.

Subgroup & SI group	Perturbation
$C2$ (5.13) $\mathbb{Z}_1$	• strain $\perp$ [100]<br>• $\boldsymbol{B}$ $\parallel$ [100]<br>• $\boldsymbol{E}$ $\parallel$ [100]
$C2$ (5.13) $\mathbb{Z}_1$	• strain $\perp$ [010]<br>• $\boldsymbol{B}$ $\parallel$ [010]<br>• $\boldsymbol{E}$ $\parallel$ [010]
$C2$ (5.13) $\mathbb{Z}_1$	• strain $\perp$ [001]<br>• $\boldsymbol{B}$ $\parallel$ [001]<br>• $\boldsymbol{E}$ $\parallel$ [001]

Table. S 164: Subgroups of $I2_12_12_11'$ (24.54) that can be realized by applying $\boldsymbol{E}/\boldsymbol{B}$ fields and/or uniaxial strains.

Subgroup & SI group	Perturbation
$P11'$ (1.2) $\mathbb{Z}_1$	• strain in generic direction<br>• $\boldsymbol{E}$ $\parallel$ [100] and strain $\perp$ [010]<br>• $\boldsymbol{E}$ $\parallel$ [100] and strain $\perp$ [001]<br>• $\boldsymbol{E}$ $\parallel$ [010] and strain $\perp$ [100]<br>• $\boldsymbol{E}$ $\parallel$ [010] and strain $\perp$ [001]<br>• $\boldsymbol{E}$ $\parallel$ [001] and strain $\perp$ [100]<br>• $\boldsymbol{E}$ $\parallel$ [001] and strain $\perp$ [010]<br>• $\boldsymbol{E}$ in generic direction
$C2'$ (5.15) $\mathbb{Z}_1$	• $\boldsymbol{B}$ $\parallel$ [010] and strain $\perp$ [100]<br>• $\boldsymbol{B}$ $\parallel$ [001] and strain $\perp$ [100]<br>• $\boldsymbol{B}$ $\perp$ [100]<br>• $\boldsymbol{E}$ $\parallel$ [100] and $\boldsymbol{B}$ $\parallel$ [010]<br>• $\boldsymbol{E}$ $\parallel$ [100] and $\boldsymbol{B}$ $\parallel$ [001]
$C2$ (5.13) $\mathbb{Z}_1$	• $\boldsymbol{B}$ $\parallel$ [100] and strain $\perp$ [100]<br>• $\boldsymbol{E}$ $\parallel$ [100] and $\boldsymbol{B}$ $\parallel$ [100]
$C21'$ (5.14) $\mathbb{Z}_1$	• strain $\perp$ [100]<br>• $\boldsymbol{E}$ $\parallel$ [100]

Continued on next page

. . . continued

Subgroup & SI group	Perturbation
$C2'$ (5.15) $\mathbb{Z}_1$	• $\boldsymbol{B}$ ∥ [100] and strain ⊥ [010]<br>• $\boldsymbol{B}$ ∥ [001] and strain ⊥ [010]<br>• $\boldsymbol{B}$ ⊥ [010]<br>• $\boldsymbol{E}$ ∥ [010] and $\boldsymbol{B}$ ∥ [100]<br>• $\boldsymbol{E}$ ∥ [010] and $\boldsymbol{B}$ ∥ [001]
$C2$ (5.13) $\mathbb{Z}_1$	• $\boldsymbol{B}$ ∥ [010] and strain ⊥ [010]<br>• $\boldsymbol{E}$ ∥ [010] and $\boldsymbol{B}$ ∥ [010]
$C21'$ (5.14) $\mathbb{Z}_1$	• strain ⊥ [010]<br>• $\boldsymbol{E}$ ∥ [010]
$C2'$ (5.15) $\mathbb{Z}_1$	• $\boldsymbol{B}$ ∥ [100] and strain ⊥ [001]<br>• $\boldsymbol{B}$ ∥ [010] and strain ⊥ [001]<br>• $\boldsymbol{B}$ ⊥ [001]<br>• $\boldsymbol{E}$ ∥ [001] and $\boldsymbol{B}$ ∥ [100]<br>• $\boldsymbol{E}$ ∥ [001] and $\boldsymbol{B}$ ∥ [010]
$I2_1'2_1'2_1$ (24.55) $\mathbb{Z}_1$	• $\boldsymbol{B}$ ∥ [100]
$I2_1'2_1'2_1$ (24.55) $\mathbb{Z}_1$	• $\boldsymbol{B}$ ∥ [010]
$C2$ (5.13) $\mathbb{Z}_1$	• $\boldsymbol{B}$ ∥ [001] and strain ⊥ [001]<br>• $\boldsymbol{E}$ ∥ [001] and $\boldsymbol{B}$ ∥ [001]
$I2_1'2_1'2_1$ (24.55) $\mathbb{Z}_1$	• $\boldsymbol{B}$ ∥ [001]
$C21'$ (5.14) $\mathbb{Z}_1$	• strain ⊥ [001]<br>• $\boldsymbol{E}$ ∥ [001]

Table. S 165: Subgroups of $I2_1'2_1'2_1$ (24.55) that can be realized by applying $\boldsymbol{E}/\boldsymbol{B}$ fields and/or uniaxial strains.

Subgroup & SI group	Perturbation
$C2'$ (5.15) $\mathbb{Z}_1$	• strain ⊥ [100]<br>• ($\boldsymbol{B}$ ∥ [010] or $\boldsymbol{B}$ ⊥ [100])<br>• $\boldsymbol{E}$ ∥ [100]
$C2'$ (5.15) $\mathbb{Z}_1$	• strain ⊥ [010]<br>• ($\boldsymbol{B}$ ∥ [100] or $\boldsymbol{B}$ ⊥ [010])<br>• $\boldsymbol{E}$ ∥ [010]
$C2$ (5.13) $\mathbb{Z}_1$	• strain ⊥ [001]<br>• $\boldsymbol{E}$ ∥ [001]

Table. S 166: Subgroups of $I_c2_12_12_1$ (24.56) that can be realized by applying $\boldsymbol{E}/\boldsymbol{B}$ fields and/or uniaxial strains.

Subgroup & SI group	Perturbation
P_S1 (1.3) $\mathbb{Z}_1$	• strain in generic direction<br>• $\boldsymbol{E} \parallel [100]$ and strain $\perp [010]$<br>• $\boldsymbol{E} \parallel [100]$ and strain $\perp [001]$<br>• $\boldsymbol{E} \parallel [010]$ and strain $\perp [100]$<br>• $\boldsymbol{E} \parallel [010]$ and strain $\perp [001]$<br>• $\boldsymbol{E} \parallel [001]$ and strain $\perp [100]$<br>• $\boldsymbol{E} \parallel [001]$ and strain $\perp [010]$<br>• $\boldsymbol{E}$ in generic direction
$C2$ (5.13) $\mathbb{Z}_1$	• $\boldsymbol{B} \parallel [100]$ and strain $\perp [100]$<br>• $\boldsymbol{E} \parallel [100]$ and $\boldsymbol{B} \parallel [100]$
$C2'$ (5.15) $\mathbb{Z}_1$	• $\boldsymbol{B} \parallel [010]$ and strain $\perp [100]$<br>• $\boldsymbol{B} \parallel [001]$ and strain $\perp [100]$<br>• $\boldsymbol{B} \perp [100]$<br>• $\boldsymbol{E} \parallel [100]$ and $\boldsymbol{B} \parallel [010]$<br>• $\boldsymbol{E} \parallel [100]$ and $\boldsymbol{B} \parallel [001]$
C_c2 (5.16) $\mathbb{Z}_1$	• strain $\perp [100]$<br>• $\boldsymbol{E} \parallel [100]$
$C2$ (5.13) $\mathbb{Z}_1$	• $\boldsymbol{B} \parallel [010]$ and strain $\perp [010]$<br>• $\boldsymbol{E} \parallel [010]$ and $\boldsymbol{B} \parallel [010]$
$C2'$ (5.15) $\mathbb{Z}_1$	• $\boldsymbol{B} \parallel [100]$ and strain $\perp [010]$<br>• $\boldsymbol{B} \parallel [001]$ and strain $\perp [010]$<br>• $\boldsymbol{B} \perp [010]$<br>• $\boldsymbol{E} \parallel [010]$ and $\boldsymbol{B} \parallel [100]$<br>• $\boldsymbol{E} \parallel [010]$ and $\boldsymbol{B} \parallel [001]$
C_c2 (5.16) $\mathbb{Z}_1$	• strain $\perp [010]$<br>• $\boldsymbol{E} \parallel [010]$
$C2'$ (5.15) $\mathbb{Z}_1$	• $\boldsymbol{B} \parallel [100]$ and strain $\perp [001]$<br>• $\boldsymbol{B} \parallel [010]$ and strain $\perp [001]$<br>• $\boldsymbol{B} \perp [001]$<br>• $\boldsymbol{E} \parallel [001]$ and $\boldsymbol{B} \parallel [100]$<br>• $\boldsymbol{E} \parallel [001]$ and $\boldsymbol{B} \parallel [010]$
$I2'2'2$ (23.51) $\mathbb{Z}_1$	• $\boldsymbol{B} \parallel [010]$
$I2'2'2$ (23.51) $\mathbb{Z}_1$	• $\boldsymbol{B} \parallel [100]$
$C2$ (5.13) $\mathbb{Z}_1$	• $\boldsymbol{B} \parallel [001]$ and strain $\perp [001]$<br>• $\boldsymbol{E} \parallel [001]$ and $\boldsymbol{B} \parallel [001]$

Continued on next page

...continued

Subgroup & SI group	Perturbation
$I2_1'2_1'2_1$ (24.55) $\mathbb{Z}_1$	• $\boldsymbol{B} \parallel [001]$
C_a2 (5.17) $\mathbb{Z}_2$	• strain $\perp [001]$<br>• $\boldsymbol{E} \parallel [001]$

Table. S 167: Subgroups of $Pmm2$ (25.57) that can be realized by applying $\boldsymbol{E}/\boldsymbol{B}$ fields and/or uniaxial strains.

Subgroup & SI group	Perturbation
Pm (6.18) $\mathbb{Z}_1$	• strain $\perp [010]$<br>• $\boldsymbol{B} \parallel [010]$<br>• ($\boldsymbol{E} \parallel [100]$ or $\boldsymbol{E} \perp [010]$)
Pm (6.18) $\mathbb{Z}_1$	• strain $\perp [100]$<br>• $\boldsymbol{B} \parallel [100]$<br>• ($\boldsymbol{E} \parallel [010]$ or $\boldsymbol{E} \perp [100]$)
$P2$ (3.1) $\mathbb{Z}_2$	• strain $\perp [001]$<br>• $\boldsymbol{B} \parallel [001]$

Table. S 168: Subgroups of $Pmm21'$ (25.58) that can be realized by applying $\boldsymbol{E}/\boldsymbol{B}$ fields and/or uniaxial strains.

Subgroup & SI group	Perturbation
$P11'$ (1.2) $\mathbb{Z}_1$	• strain in generic direction<br>• ($\boldsymbol{E} \parallel [100]$ or $\boldsymbol{E} \perp [010]$) and strain $\perp [100]$<br>• ($\boldsymbol{E} \parallel [100]$ or $\boldsymbol{E} \perp [010]$) and strain $\perp [001]$<br>• ($\boldsymbol{E} \parallel [010]$ or $\boldsymbol{E} \perp [100]$) and strain $\perp [010]$<br>• ($\boldsymbol{E} \parallel [010]$ or $\boldsymbol{E} \perp [100]$) and strain $\perp [001]$<br>• $\boldsymbol{E}$ in generic direction
Pm' (6.20) $\mathbb{Z}_1$	• $\boldsymbol{B} \parallel [100]$ and strain $\perp [010]$<br>• $\boldsymbol{B} \parallel [001]$ and strain $\perp [010]$<br>• $\boldsymbol{B} \perp [010]$<br>• ($\boldsymbol{E} \parallel [100]$ or $\boldsymbol{E} \perp [010]$) and $\boldsymbol{B} \parallel [100]$<br>• ($\boldsymbol{E} \parallel [100]$ or $\boldsymbol{E} \perp [010]$) and $\boldsymbol{B} \parallel [001]$

Continued on next page

. . . continued

Subgroup & SI group	Perturbation
Pm (6.18) $\mathbb{Z}_1$	• $\boldsymbol{B}$ ∥ [010] and strain ⊥ [010]<br>• ($\boldsymbol{E}$ ∥ [100] or $\boldsymbol{E}$ ⊥ [010]) and $\boldsymbol{B}$ ∥ [010]
$Pm1'$ (6.19) $\mathbb{Z}_1$	• strain ⊥ [010]<br>• ($\boldsymbol{E}$ ∥ [100] or $\boldsymbol{E}$ ⊥ [010])
Pm' (6.20) $\mathbb{Z}_1$	• $\boldsymbol{B}$ ∥ [010] and strain ⊥ [100]<br>• $\boldsymbol{B}$ ∥ [001] and strain ⊥ [100]<br>• $\boldsymbol{B}$ ⊥ [100]<br>• ($\boldsymbol{E}$ ∥ [010] or $\boldsymbol{E}$ ⊥ [100]) and $\boldsymbol{B}$ ∥ [010]<br>• ($\boldsymbol{E}$ ∥ [010] or $\boldsymbol{E}$ ⊥ [100]) and $\boldsymbol{B}$ ∥ [001]
Pm (6.18) $\mathbb{Z}_1$	• $\boldsymbol{B}$ ∥ [100] and strain ⊥ [100]<br>• ($\boldsymbol{E}$ ∥ [010] or $\boldsymbol{E}$ ⊥ [100]) and $\boldsymbol{B}$ ∥ [100]
$Pm1'$ (6.19) $\mathbb{Z}_1$	• strain ⊥ [100]<br>• ($\boldsymbol{E}$ ∥ [010] or $\boldsymbol{E}$ ⊥ [100])
$P2'$ (3.3) $\mathbb{Z}_1$	• $\boldsymbol{B}$ ∥ [100] and strain ⊥ [001]<br>• $\boldsymbol{B}$ ∥ [010] and strain ⊥ [001]<br>• $\boldsymbol{B}$ ⊥ [001]
$Pm'm2'$ (25.59) $\mathbb{Z}_1$	• $\boldsymbol{B}$ ∥ [010]
$Pm'm2'$ (25.59) $\mathbb{Z}_1$	• $\boldsymbol{B}$ ∥ [100]
$P2$ (3.1) $\mathbb{Z}_2$	• $\boldsymbol{B}$ ∥ [001] and strain ⊥ [001]
$Pm'm'2$ (25.60) $\mathbb{Z}_2$	• $\boldsymbol{B}$ ∥ [001]
$P21'$ (3.2) $\mathbb{Z}_2$	• strain ⊥ [001]

Table. S 169: Subgroups of $Pm'm2'$ (25.59) that can be realized by applying $\boldsymbol{E}/\boldsymbol{B}$ fields and/or uniaxial strains.

Subgroup & SI group	Perturbation
Pm (6.18) $\mathbb{Z}_1$	• strain ⊥ [010]<br>• ($\boldsymbol{E}$ ∥ [100] or $\boldsymbol{E}$ ⊥ [010])
Pm' (6.20) $\mathbb{Z}_1$	• strain ⊥ [100]<br>• ($\boldsymbol{B}$ ∥ [001] or $\boldsymbol{B}$ ⊥ [100])<br>• ($\boldsymbol{E}$ ∥ [010] or $\boldsymbol{E}$ ⊥ [100])
$P2'$ (3.3) $\mathbb{Z}_1$	• strain ⊥ [001]<br>• ($\boldsymbol{B}$ ∥ [100] or $\boldsymbol{B}$ ⊥ [001])

Table. S 170: Subgroups of $Pm'm'2$ (25.60) that can be realized by applying $\boldsymbol{E}/\boldsymbol{B}$ fields and/or uniaxial strains.

Subgroup & SI group	Perturbation
Pm' (6.20) $\mathbb{Z}_1$	• strain $\perp$ [010]<br>• ($\boldsymbol{B} \parallel$ [100] or $\boldsymbol{B} \perp$ [010])<br>• ($\boldsymbol{E} \parallel$ [100] or $\boldsymbol{E} \perp$ [010])
Pm' (6.20) $\mathbb{Z}_1$	• strain $\perp$ [100]<br>• ($\boldsymbol{B} \parallel$ [010] or $\boldsymbol{B} \perp$ [100])<br>• ($\boldsymbol{E} \parallel$ [010] or $\boldsymbol{E} \perp$ [100])
$P2$ (3.1) $\mathbb{Z}_2$	• strain $\perp$ [001]

Table. S 171: Subgroups of P_cmm2 (25.61) that can be realized by applying $\boldsymbol{E}/\boldsymbol{B}$ fields and/or uniaxial strains.

Subgroup & SI group	Perturbation
P_S1 (1.3) $\mathbb{Z}_1$	• strain in generic direction<br>• ($\boldsymbol{E} \parallel$ [100] or $\boldsymbol{E} \perp$ [010]) and strain $\perp$ [100]<br>• ($\boldsymbol{E} \parallel$ [100] or $\boldsymbol{E} \perp$ [010]) and strain $\perp$ [001]<br>• ($\boldsymbol{E} \parallel$ [010] or $\boldsymbol{E} \perp$ [100]) and strain $\perp$ [010]<br>• ($\boldsymbol{E} \parallel$ [010] or $\boldsymbol{E} \perp$ [100]) and strain $\perp$ [001]<br>• $\boldsymbol{E}$ in generic direction
Pm (6.18) $\mathbb{Z}_1$	• $\boldsymbol{B} \parallel$ [010] and strain $\perp$ [010]<br>• ($\boldsymbol{E} \parallel$ [100] or $\boldsymbol{E} \perp$ [010]) and $\boldsymbol{B} \parallel$ [010]
Pc' (7.26) $\mathbb{Z}_1$	• $\boldsymbol{B} \parallel$ [100] and strain $\perp$ [010]<br>• $\boldsymbol{B} \parallel$ [001] and strain $\perp$ [010]<br>• $\boldsymbol{B} \perp$ [010]<br>• ($\boldsymbol{E} \parallel$ [100] or $\boldsymbol{E} \perp$ [010]) and $\boldsymbol{B} \parallel$ [100]<br>• ($\boldsymbol{E} \parallel$ [100] or $\boldsymbol{E} \perp$ [010]) and $\boldsymbol{B} \parallel$ [001]
P_am (6.21) $\mathbb{Z}_1$	• strain $\perp$ [010]<br>• ($\boldsymbol{E} \parallel$ [100] or $\boldsymbol{E} \perp$ [010])
Pm (6.18) $\mathbb{Z}_1$	• $\boldsymbol{B} \parallel$ [100] and strain $\perp$ [100]<br>• ($\boldsymbol{E} \parallel$ [010] or $\boldsymbol{E} \perp$ [100]) and $\boldsymbol{B} \parallel$ [100]

Continued on next page

…continued

Subgroup & SI group	Perturbation
Pc' (7.26) $\mathbb{Z}_1$	• $\boldsymbol{B}$ ∥ [010] and strain ⊥ [100]<br>• $\boldsymbol{B}$ ∥ [001] and strain ⊥ [100]<br>• $\boldsymbol{B}$ ⊥ [100]<br>• ($\boldsymbol{E}$ ∥ [010] or $\boldsymbol{E}$ ⊥ [100]) and $\boldsymbol{B}$ ∥ [010]<br>• ($\boldsymbol{E}$ ∥ [010] or $\boldsymbol{E}$ ⊥ [100]) and $\boldsymbol{B}$ ∥ [001]
P_am (6.21) $\mathbb{Z}_1$	• strain ⊥ [100]<br>• ($\boldsymbol{E}$ ∥ [010] or $\boldsymbol{E}$ ⊥ [100])
$P2$ (3.1) $\mathbb{Z}_2$	• $\boldsymbol{B}$ ∥ [001] and strain ⊥ [001]
$Pc'c'2$ (27.81) $\mathbb{Z}_2$	• $\boldsymbol{B}$ ∥ [001]
$P2_1'$ (4.9) $\mathbb{Z}_1$	• $\boldsymbol{B}$ ∥ [100] and strain ⊥ [001]<br>• $\boldsymbol{B}$ ∥ [010] and strain ⊥ [001]<br>• $\boldsymbol{B}$ ⊥ [001]
$Pmc'2_1'$ (26.69) $\mathbb{Z}_1$	• $\boldsymbol{B}$ ∥ [100]
$Pmc'2_1'$ (26.69) $\mathbb{Z}_1$	• $\boldsymbol{B}$ ∥ [010]
P_b2 (3.5) $\mathbb{Z}_2$	• strain ⊥ [001]

Table. S 172: Subgroups of P_amm2 (25.62) that can be realized by applying $\boldsymbol{E}/\boldsymbol{B}$ fields and/or uniaxial strains.

Subgroup & SI group	Perturbation
Pm (6.18) $\mathbb{Z}_1$	• $\boldsymbol{B}$ ∥ [010] and strain ⊥ [010]<br>• ($\boldsymbol{E}$ ∥ [100] or $\boldsymbol{E}$ ⊥ [010]) and $\boldsymbol{B}$ ∥ [010]
P_S1 (1.3) $\mathbb{Z}_1$	• strain in generic direction<br>• ($\boldsymbol{E}$ ∥ [100] or $\boldsymbol{E}$ ⊥ [010]) and strain ⊥ [100]<br>• ($\boldsymbol{E}$ ∥ [100] or $\boldsymbol{E}$ ⊥ [010]) and strain ⊥ [001]<br>• ($\boldsymbol{E}$ ∥ [010] or $\boldsymbol{E}$ ⊥ [100]) and strain ⊥ [010]<br>• ($\boldsymbol{E}$ ∥ [010] or $\boldsymbol{E}$ ⊥ [100]) and strain ⊥ [001]<br>• $\boldsymbol{E}$ in generic direction
Pc' (7.26) $\mathbb{Z}_1$	• $\boldsymbol{B}$ ∥ [100] and strain ⊥ [010]<br>• $\boldsymbol{B}$ ∥ [001] and strain ⊥ [010]<br>• $\boldsymbol{B}$ ⊥ [010]<br>• ($\boldsymbol{E}$ ∥ [100] or $\boldsymbol{E}$ ⊥ [010]) and $\boldsymbol{B}$ ∥ [100]<br>• ($\boldsymbol{E}$ ∥ [100] or $\boldsymbol{E}$ ⊥ [010]) and $\boldsymbol{B}$ ∥ [001]

Continued on next page

…continued

Subgroup & SI group	Perturbation
P_am (6.21) $\mathbb{Z}_1$	• strain $\perp$ [010]<br>• ($\boldsymbol{E}$ $\parallel$ [100] or $\boldsymbol{E}$ $\perp$ [010])
Pm (6.18) $\mathbb{Z}_1$	• $\boldsymbol{B}$ $\parallel$ [100] and strain $\perp$ [100]<br>• ($\boldsymbol{E}$ $\parallel$ [010] or $\boldsymbol{E}$ $\perp$ [100]) and $\boldsymbol{B}$ $\parallel$ [100]
$P2$ (3.1) $\mathbb{Z}_2$	• $\boldsymbol{B}$ $\parallel$ [001] and strain $\perp$ [001]
Pm' (6.20) $\mathbb{Z}_1$	• $\boldsymbol{B}$ $\parallel$ [010] and strain $\perp$ [100]<br>• $\boldsymbol{B}$ $\parallel$ [001] and strain $\perp$ [100]<br>• $\boldsymbol{B}$ $\perp$ [100]<br>• ($\boldsymbol{E}$ $\parallel$ [010] or $\boldsymbol{E}$ $\perp$ [100]) and $\boldsymbol{B}$ $\parallel$ [010]<br>• ($\boldsymbol{E}$ $\parallel$ [010] or $\boldsymbol{E}$ $\perp$ [100]) and $\boldsymbol{B}$ $\parallel$ [001]
P_bm (6.22) $\mathbb{Z}_1$	• strain $\perp$ [100]<br>• ($\boldsymbol{E}$ $\parallel$ [010] or $\boldsymbol{E}$ $\perp$ [100])
$Pm'a'2$ (28.91) $\mathbb{Z}_2$	• $\boldsymbol{B}$ $\parallel$ [001]
$P2'$ (3.3) $\mathbb{Z}_1$	• $\boldsymbol{B}$ $\parallel$ [100] and strain $\perp$ [001]<br>• $\boldsymbol{B}$ $\parallel$ [010] and strain $\perp$ [001]<br>• $\boldsymbol{B}$ $\perp$ [001]
$Pma'2'$ (28.90) $\mathbb{Z}_1$	• $\boldsymbol{B}$ $\parallel$ [100]
P_a2 (3.4) $\mathbb{Z}_2$	• strain $\perp$ [001]
$Pm'm2'$ (25.59) $\mathbb{Z}_1$	• $\boldsymbol{B}$ $\parallel$ [010]

Table. S 173: Subgroups of P_Cmm2 (25.63) that can be realized by applying $\boldsymbol{E}/\boldsymbol{B}$ fields and/or uniaxial strains.

Subgroup & SI group	Perturbation
Pm (6.18) $\mathbb{Z}_1$	• $\boldsymbol{B}$ $\parallel$ [010] and strain $\perp$ [010]<br>• ($\boldsymbol{E}$ $\parallel$ [100] or $\boldsymbol{E}$ $\perp$ [010]) and $\boldsymbol{B}$ $\parallel$ [010]
P_S1 (1.3) $\mathbb{Z}_1$	• strain in generic direction<br>• ($\boldsymbol{E}$ $\parallel$ [100] or $\boldsymbol{E}$ $\perp$ [010]) and strain $\perp$ [100]<br>• ($\boldsymbol{E}$ $\parallel$ [100] or $\boldsymbol{E}$ $\perp$ [010]) and strain $\perp$ [001]<br>• ($\boldsymbol{E}$ $\parallel$ [010] or $\boldsymbol{E}$ $\perp$ [100]) and strain $\perp$ [010]<br>• ($\boldsymbol{E}$ $\parallel$ [010] or $\boldsymbol{E}$ $\perp$ [100]) and strain $\perp$ [001]<br>• $\boldsymbol{E}$ in generic direction

Continued on next page

...continued

Subgroup & SI group	Perturbation
Pc' (7.26) $\mathbb{Z}_1$	• $\boldsymbol{B} \parallel [100]$ and strain $\perp [010]$<br>• $\boldsymbol{B} \parallel [001]$ and strain $\perp [010]$<br>• $\boldsymbol{B} \perp [010]$<br>• ($\boldsymbol{E} \parallel [100]$ or $\boldsymbol{E} \perp [010]$) and $\boldsymbol{B} \parallel [100]$<br>• ($\boldsymbol{E} \parallel [100]$ or $\boldsymbol{E} \perp [010]$) and $\boldsymbol{B} \parallel [001]$
P_Cm (6.23) $\mathbb{Z}_1$	• strain $\perp [010]$<br>• ($\boldsymbol{E} \parallel [100]$ or $\boldsymbol{E} \perp [010]$)
Pm (6.18) $\mathbb{Z}_1$	• $\boldsymbol{B} \parallel [100]$ and strain $\perp [100]$<br>• ($\boldsymbol{E} \parallel [010]$ or $\boldsymbol{E} \perp [100]$) and $\boldsymbol{B} \parallel [100]$
$P2$ (3.1) $\mathbb{Z}_2$	• $\boldsymbol{B} \parallel [001]$ and strain $\perp [001]$
Pc' (7.26) $\mathbb{Z}_1$	• $\boldsymbol{B} \parallel [010]$ and strain $\perp [100]$<br>• $\boldsymbol{B} \parallel [001]$ and strain $\perp [100]$<br>• $\boldsymbol{B} \perp [100]$<br>• ($\boldsymbol{E} \parallel [010]$ or $\boldsymbol{E} \perp [100]$) and $\boldsymbol{B} \parallel [010]$<br>• ($\boldsymbol{E} \parallel [010]$ or $\boldsymbol{E} \perp [100]$) and $\boldsymbol{B} \parallel [001]$
P_Cm (6.23) $\mathbb{Z}_1$	• strain $\perp [100]$<br>• ($\boldsymbol{E} \parallel [010]$ or $\boldsymbol{E} \perp [100]$)
$Pb'a'2$ (32.138) $\mathbb{Z}_2$	• $\boldsymbol{B} \parallel [001]$
$P2'$ (3.3) $\mathbb{Z}_1$	• $\boldsymbol{B} \parallel [100]$ and strain $\perp [001]$<br>• $\boldsymbol{B} \parallel [010]$ and strain $\perp [001]$<br>• $\boldsymbol{B} \perp [001]$
$Pma'2'$ (28.90) $\mathbb{Z}_1$	• $\boldsymbol{B} \parallel [100]$
P_a2 (3.4) $\mathbb{Z}_2$	• strain $\perp [001]$
$Pma'2'$ (28.90) $\mathbb{Z}_1$	• $\boldsymbol{B} \parallel [010]$

Table. S 174: Subgroups of P_Amm2 (25.64) that can be realized by applying $\boldsymbol{E}/\boldsymbol{B}$ fields and/or uniaxial strains.

Subgroup & SI group	Perturbation

Continued on next page

. . . continued

Subgroup & SI group	Perturbation
P_S1 (1.3) $\mathbb{Z}_1$	• strain in generic direction<br>• ($\boldsymbol{E} \parallel [100]$ or $\boldsymbol{E} \perp [010]$) and strain $\perp [100]$<br>• ($\boldsymbol{E} \parallel [100]$ or $\boldsymbol{E} \perp [010]$) and strain $\perp [001]$<br>• ($\boldsymbol{E} \parallel [010]$ or $\boldsymbol{E} \perp [100]$) and strain $\perp [010]$<br>• ($\boldsymbol{E} \parallel [010]$ or $\boldsymbol{E} \perp [100]$) and strain $\perp [001]$<br>• $\boldsymbol{E}$ in generic direction
Pm (6.18) $\mathbb{Z}_1$	• $\boldsymbol{B} \parallel [010]$ and strain $\perp [010]$<br>• ($\boldsymbol{E} \parallel [100]$ or $\boldsymbol{E} \perp [010]$) and $\boldsymbol{B} \parallel [010]$
Pc' (7.26) $\mathbb{Z}_1$	• $\boldsymbol{B} \parallel [100]$ and strain $\perp [010]$<br>• $\boldsymbol{B} \parallel [001]$ and strain $\perp [010]$<br>• $\boldsymbol{B} \perp [010]$<br>• ($\boldsymbol{E} \parallel [100]$ or $\boldsymbol{E} \perp [010]$) and $\boldsymbol{B} \parallel [100]$<br>• ($\boldsymbol{E} \parallel [100]$ or $\boldsymbol{E} \perp [010]$) and $\boldsymbol{B} \parallel [001]$
P_Cm (6.23) $\mathbb{Z}_1$	• strain $\perp [010]$<br>• ($\boldsymbol{E} \parallel [100]$ or $\boldsymbol{E} \perp [010]$)
Pm (6.18) $\mathbb{Z}_1$	• $\boldsymbol{B} \parallel [100]$ and strain $\perp [100]$<br>• ($\boldsymbol{E} \parallel [010]$ or $\boldsymbol{E} \perp [100]$) and $\boldsymbol{B} \parallel [100]$
Pc' (7.26) $\mathbb{Z}_1$	• $\boldsymbol{B} \parallel [010]$ and strain $\perp [100]$<br>• $\boldsymbol{B} \parallel [001]$ and strain $\perp [100]$<br>• $\boldsymbol{B} \perp [100]$<br>• ($\boldsymbol{E} \parallel [010]$ or $\boldsymbol{E} \perp [100]$) and $\boldsymbol{B} \parallel [010]$<br>• ($\boldsymbol{E} \parallel [010]$ or $\boldsymbol{E} \perp [100]$) and $\boldsymbol{B} \parallel [001]$
P_am (6.21) $\mathbb{Z}_1$	• strain $\perp [100]$<br>• ($\boldsymbol{E} \parallel [010]$ or $\boldsymbol{E} \perp [100]$)
$P2$ (3.1) $\mathbb{Z}_2$	• $\boldsymbol{B} \parallel [001]$ and strain $\perp [001]$
$Pn'c'2$ (30.115) $\mathbb{Z}_1$	• $\boldsymbol{B} \parallel [001]$
$P2_1'$ (4.9) $\mathbb{Z}_1$	• $\boldsymbol{B} \parallel [100]$ and strain $\perp [001]$<br>• $\boldsymbol{B} \parallel [010]$ and strain $\perp [001]$<br>• $\boldsymbol{B} \perp [001]$
$Pmc'2_1'$ (26.69) $\mathbb{Z}_1$	• $\boldsymbol{B} \parallel [100]$
$Pmn'2_1'$ (31.126) $\mathbb{Z}_1$	• $\boldsymbol{B} \parallel [010]$
P_C2 (3.6) $\mathbb{Z}_1$	• strain $\perp [001]$

Table. S 175: Subgroups of $P_{I}mm2$ (25.65) that can be realized by applying $\boldsymbol{E}/\boldsymbol{B}$ fields and/or uniaxial strains.

Subgroup & SI group	Perturbation
Pm (6.18) $\mathbb{Z}_1$	• $\boldsymbol{B} \parallel [010]$ and strain $\perp [010]$<br>• ($\boldsymbol{E} \parallel [100]$ or $\boldsymbol{E} \perp [010]$) and $\boldsymbol{B} \parallel [010]$
P_S1 (1.3) $\mathbb{Z}_1$	• strain in generic direction<br>• ($\boldsymbol{E} \parallel [100]$ or $\boldsymbol{E} \perp [010]$) and strain $\perp [100]$<br>• ($\boldsymbol{E} \parallel [100]$ or $\boldsymbol{E} \perp [010]$) and strain $\perp [001]$<br>• ($\boldsymbol{E} \parallel [010]$ or $\boldsymbol{E} \perp [100]$) and strain $\perp [010]$<br>• ($\boldsymbol{E} \parallel [010]$ or $\boldsymbol{E} \perp [100]$) and strain $\perp [001]$<br>• $\boldsymbol{E}$ in generic direction
Pc' (7.26) $\mathbb{Z}_1$	• $\boldsymbol{B} \parallel [100]$ and strain $\perp [010]$<br>• $\boldsymbol{B} \parallel [001]$ and strain $\perp [010]$<br>• $\boldsymbol{B} \perp [010]$<br>• ($\boldsymbol{E} \parallel [100]$ or $\boldsymbol{E} \perp [010]$) and $\boldsymbol{B} \parallel [100]$<br>• ($\boldsymbol{E} \parallel [100]$ or $\boldsymbol{E} \perp [010]$) and $\boldsymbol{B} \parallel [001]$
P_Cm (6.23) $\mathbb{Z}_1$	• strain $\perp [010]$<br>• ($\boldsymbol{E} \parallel [100]$ or $\boldsymbol{E} \perp [010]$)
Pm (6.18) $\mathbb{Z}_1$	• $\boldsymbol{B} \parallel [100]$ and strain $\perp [100]$<br>• ($\boldsymbol{E} \parallel [010]$ or $\boldsymbol{E} \perp [100]$) and $\boldsymbol{B} \parallel [100]$
$P2$ (3.1) $\mathbb{Z}_2$	• $\boldsymbol{B} \parallel [001]$ and strain $\perp [001]$
Pc' (7.26) $\mathbb{Z}_1$	• $\boldsymbol{B} \parallel [010]$ and strain $\perp [100]$<br>• $\boldsymbol{B} \parallel [001]$ and strain $\perp [100]$<br>• $\boldsymbol{B} \perp [100]$<br>• ($\boldsymbol{E} \parallel [010]$ or $\boldsymbol{E} \perp [100]$) and $\boldsymbol{B} \parallel [010]$<br>• ($\boldsymbol{E} \parallel [010]$ or $\boldsymbol{E} \perp [100]$) and $\boldsymbol{B} \parallel [001]$
P_Cm (6.23) $\mathbb{Z}_1$	• strain $\perp [100]$<br>• ($\boldsymbol{E} \parallel [010]$ or $\boldsymbol{E} \perp [100]$)
$Pn'n'2$ (34.159) $\mathbb{Z}_1$	• $\boldsymbol{B} \parallel [001]$
$P2_1'$ (4.9) $\mathbb{Z}_1$	• $\boldsymbol{B} \parallel [100]$ and strain $\perp [001]$<br>• $\boldsymbol{B} \parallel [010]$ and strain $\perp [001]$<br>• $\boldsymbol{B} \perp [001]$
$Pmn'2_1'$ (31.126) $\mathbb{Z}_1$	• $\boldsymbol{B} \parallel [100]$
P_C2 (3.6) $\mathbb{Z}_1$	• strain $\perp [001]$
$Pmn'2_1'$ (31.126) $\mathbb{Z}_1$	• $\boldsymbol{B} \parallel [010]$

Table. S 176: Subgroups of $Pmc2_1$ (26.66) that can be realized by applying $\boldsymbol{E}/\boldsymbol{B}$ fields and/or uniaxial strains.

Subgroup & SI group	Perturbation
Pc (7.24) $\mathbb{Z}_1$	• strain $\perp$ [010]<br>• $\boldsymbol{B} \parallel$ [010]<br>• ($\boldsymbol{E} \parallel$ [100] or $\boldsymbol{E} \perp$ [010])
Pm (6.18) $\mathbb{Z}_1$	• strain $\perp$ [100]<br>• $\boldsymbol{B} \parallel$ [100]<br>• ($\boldsymbol{E} \parallel$ [010] or $\boldsymbol{E} \perp$ [100])
$P2_1$ (4.7) $\mathbb{Z}_1$	• strain $\perp$ [001]<br>• $\boldsymbol{B} \parallel$ [001]

Table. S 177: Subgroups of $Pmc2_11'$ (26.67) that can be realized by applying $\boldsymbol{E}/\boldsymbol{B}$ fields and/or uniaxial strains.

Subgroup & SI group	Perturbation
$P11'$ (1.2) $\mathbb{Z}_1$	• strain in generic direction<br>• ($\boldsymbol{E} \parallel$ [100] or $\boldsymbol{E} \perp$ [010]) and strain $\perp$ [100]<br>• ($\boldsymbol{E} \parallel$ [100] or $\boldsymbol{E} \perp$ [010]) and strain $\perp$ [001]<br>• ($\boldsymbol{E} \parallel$ [010] or $\boldsymbol{E} \perp$ [100]) and strain $\perp$ [010]<br>• ($\boldsymbol{E} \parallel$ [010] or $\boldsymbol{E} \perp$ [100]) and strain $\perp$ [001]<br>• $\boldsymbol{E}$ in generic direction
Pc' (7.26) $\mathbb{Z}_1$	• $\boldsymbol{B} \parallel$ [100] and strain $\perp$ [010]<br>• $\boldsymbol{B} \parallel$ [001] and strain $\perp$ [010]<br>• $\boldsymbol{B} \perp$ [010]<br>• ($\boldsymbol{E} \parallel$ [100] or $\boldsymbol{E} \perp$ [010]) and $\boldsymbol{B} \parallel$ [100]<br>• ($\boldsymbol{E} \parallel$ [100] or $\boldsymbol{E} \perp$ [010]) and $\boldsymbol{B} \parallel$ [001]
Pc (7.24) $\mathbb{Z}_1$	• $\boldsymbol{B} \parallel$ [010] and strain $\perp$ [010]<br>• ($\boldsymbol{E} \parallel$ [100] or $\boldsymbol{E} \perp$ [010]) and $\boldsymbol{B} \parallel$ [010]
$Pc1'$ (7.25) $\mathbb{Z}_1$	• strain $\perp$ [010]<br>• ($\boldsymbol{E} \parallel$ [100] or $\boldsymbol{E} \perp$ [010])
Pm' (6.20) $\mathbb{Z}_1$	• $\boldsymbol{B} \parallel$ [010] and strain $\perp$ [100]<br>• $\boldsymbol{B} \parallel$ [001] and strain $\perp$ [100]<br>• $\boldsymbol{B} \perp$ [100]<br>• ($\boldsymbol{E} \parallel$ [010] or $\boldsymbol{E} \perp$ [100]) and $\boldsymbol{B} \parallel$ [010]<br>• ($\boldsymbol{E} \parallel$ [010] or $\boldsymbol{E} \perp$ [100]) and $\boldsymbol{B} \parallel$ [001]

Continued on next page

...continued

Subgroup & SI group	Perturbation
Pm (6.18) $\mathbb{Z}_1$	• $\boldsymbol{B}$ ∥ [100] and strain ⊥ [100]<br>• ($\boldsymbol{E}$ ∥ [010] or $\boldsymbol{E}$ ⊥ [100]) and $\boldsymbol{B}$ ∥ [100]
$Pm1'$ (6.19) $\mathbb{Z}_1$	• strain ⊥ [100]<br>• ($\boldsymbol{E}$ ∥ [010] or $\boldsymbol{E}$ ⊥ [100])
$P2_1'$ (4.9) $\mathbb{Z}_1$	• $\boldsymbol{B}$ ∥ [100] and strain ⊥ [001]<br>• $\boldsymbol{B}$ ∥ [010] and strain ⊥ [001]<br>• $\boldsymbol{B}$ ⊥ [001]
$Pm'c2_1'$ (26.68) $\mathbb{Z}_1$	• $\boldsymbol{B}$ ∥ [010]
$Pmc'2_1'$ (26.69) $\mathbb{Z}_1$	• $\boldsymbol{B}$ ∥ [100]
$P2_1$ (4.7) $\mathbb{Z}_1$	• $\boldsymbol{B}$ ∥ [001] and strain ⊥ [001]
$Pm'c'2_1$ (26.70) $\mathbb{Z}_1$	• $\boldsymbol{B}$ ∥ [001]
$P2_11'$ (4.8) $\mathbb{Z}_1$	• strain ⊥ [001]

Table. S 178: Subgroups of $Pm'c2_1'$ (26.68) that can be realized by applying $\boldsymbol{E}/\boldsymbol{B}$ fields and/or uniaxial strains.

Subgroup & SI group	Perturbation
Pc (7.24) $\mathbb{Z}_1$	• strain ⊥ [010]<br>• ($\boldsymbol{E}$ ∥ [100] or $\boldsymbol{E}$ ⊥ [010])
Pm' (6.20) $\mathbb{Z}_1$	• strain ⊥ [100]<br>• ($\boldsymbol{B}$ ∥ [001] or $\boldsymbol{B}$ ⊥ [100])<br>• ($\boldsymbol{E}$ ∥ [010] or $\boldsymbol{E}$ ⊥ [100])
$P2_1'$ (4.9) $\mathbb{Z}_1$	• strain ⊥ [001]<br>• ($\boldsymbol{B}$ ∥ [100] or $\boldsymbol{B}$ ⊥ [001])

Table. S 179: Subgroups of $Pmc'2_1'$ (26.69) that can be realized by applying $\boldsymbol{E}/\boldsymbol{B}$ fields and/or uniaxial strains.

Subgroup & SI group	Perturbation

Continued on next page

...continued

Subgroup & SI group	Perturbation
Pc' (7.26) $\mathbb{Z}_1$	• strain $\perp$ [010]<br>• ($\boldsymbol{B}$ $\parallel$ [001] or $\boldsymbol{B}$ $\perp$ [010])<br>• ($\boldsymbol{E}$ $\parallel$ [100] or $\boldsymbol{E}$ $\perp$ [010])
Pm (6.18) $\mathbb{Z}_1$	• strain $\perp$ [100]<br>• ($\boldsymbol{E}$ $\parallel$ [010] or $\boldsymbol{E}$ $\perp$ [100])
$P2_1'$ (4.9) $\mathbb{Z}_1$	• strain $\perp$ [001]<br>• ($\boldsymbol{B}$ $\parallel$ [010] or $\boldsymbol{B}$ $\perp$ [001])

Table. S 180: Subgroups of $Pm'c'2_1$ (26.70) that can be realized by applying $\boldsymbol{E}/\boldsymbol{B}$ fields and/or uniaxial strains.

Subgroup & SI group	Perturbation
Pc' (7.26) $\mathbb{Z}_1$	• strain $\perp$ [010]<br>• ($\boldsymbol{B}$ $\parallel$ [100] or $\boldsymbol{B}$ $\perp$ [010])<br>• ($\boldsymbol{E}$ $\parallel$ [100] or $\boldsymbol{E}$ $\perp$ [010])
Pm' (6.20) $\mathbb{Z}_1$	• strain $\perp$ [100]<br>• ($\boldsymbol{B}$ $\parallel$ [010] or $\boldsymbol{B}$ $\perp$ [100])<br>• ($\boldsymbol{E}$ $\parallel$ [010] or $\boldsymbol{E}$ $\perp$ [100])
$P2_1$ (4.7) $\mathbb{Z}_1$	• strain $\perp$ [001]

Table. S 181: Subgroups of P_amc2_1 (26.71) that can be realized by applying $\boldsymbol{E}/\boldsymbol{B}$ fields and/or uniaxial strains.

Subgroup & SI group	Perturbation
Pc (7.24) $\mathbb{Z}_1$	• $\boldsymbol{B}$ $\parallel$ [010] and strain $\perp$ [010]<br>• ($\boldsymbol{E}$ $\parallel$ [100] or $\boldsymbol{E}$ $\perp$ [010]) and $\boldsymbol{B}$ $\parallel$ [010]
P_S1 (1.3) $\mathbb{Z}_1$	• strain in generic direction<br>• ($\boldsymbol{E}$ $\parallel$ [100] or $\boldsymbol{E}$ $\perp$ [010]) and strain $\perp$ [100]<br>• ($\boldsymbol{E}$ $\parallel$ [100] or $\boldsymbol{E}$ $\perp$ [010]) and strain $\perp$ [001]<br>• ($\boldsymbol{E}$ $\parallel$ [010] or $\boldsymbol{E}$ $\perp$ [100]) and strain $\perp$ [010]<br>• ($\boldsymbol{E}$ $\parallel$ [010] or $\boldsymbol{E}$ $\perp$ [100]) and strain $\perp$ [001]<br>• $\boldsymbol{E}$ in generic direction

Continued on next page

...continued

Subgroup & SI group	Perturbation
Pc' (7.26) $\mathbb{Z}_1$	• $\boldsymbol{B} \parallel [100]$ and strain $\perp [010]$<br>• $\boldsymbol{B} \parallel [001]$ and strain $\perp [010]$<br>• $\boldsymbol{B} \perp [010]$<br>• ($\boldsymbol{E} \parallel [100]$ or $\boldsymbol{E} \perp [010]$) and $\boldsymbol{B} \parallel [100]$<br>• ($\boldsymbol{E} \parallel [100]$ or $\boldsymbol{E} \perp [010]$) and $\boldsymbol{B} \parallel [001]$
P_ac (7.27) $\mathbb{Z}_1$	• strain $\perp [010]$<br>• ($\boldsymbol{E} \parallel [100]$ or $\boldsymbol{E} \perp [010]$)
Pm (6.18) $\mathbb{Z}_1$	• $\boldsymbol{B} \parallel [100]$ and strain $\perp [100]$<br>• ($\boldsymbol{E} \parallel [010]$ or $\boldsymbol{E} \perp [100]$) and $\boldsymbol{B} \parallel [100]$
$P2_1$ (4.7) $\mathbb{Z}_1$	• $\boldsymbol{B} \parallel [001]$ and strain $\perp [001]$
Pm' (6.20) $\mathbb{Z}_1$	• $\boldsymbol{B} \parallel [010]$ and strain $\perp [100]$<br>• $\boldsymbol{B} \parallel [001]$ and strain $\perp [100]$<br>• $\boldsymbol{B} \perp [100]$<br>• ($\boldsymbol{E} \parallel [010]$ or $\boldsymbol{E} \perp [100]$) and $\boldsymbol{B} \parallel [010]$<br>• ($\boldsymbol{E} \parallel [010]$ or $\boldsymbol{E} \perp [100]$) and $\boldsymbol{B} \parallel [001]$
P_bm (6.22) $\mathbb{Z}_1$	• strain $\perp [100]$<br>• ($\boldsymbol{E} \parallel [010]$ or $\boldsymbol{E} \perp [100]$)
$Pm'n'2_1$ (31.127) $\mathbb{Z}_1$	• $\boldsymbol{B} \parallel [001]$
$P2_1'$ (4.9) $\mathbb{Z}_1$	• $\boldsymbol{B} \parallel [100]$ and strain $\perp [001]$<br>• $\boldsymbol{B} \parallel [010]$ and strain $\perp [001]$<br>• $\boldsymbol{B} \perp [001]$
$Pmn'2_1'$ (31.126) $\mathbb{Z}_1$	• $\boldsymbol{B} \parallel [100]$
P_a2_1 (4.10) $\mathbb{Z}_1$	• strain $\perp [001]$
$Pm'c2_1'$ (26.68) $\mathbb{Z}_1$	• $\boldsymbol{B} \parallel [010]$

Table. S 182: Subgroups of P_bmc2_1 (26.72) that can be realized by applying $\boldsymbol{E}/\boldsymbol{B}$ fields and/or uniaxial strains.

Subgroup & SI group	Perturbation

Continued on next page

...continued

Subgroup & SI group	Perturbation
P_S1 (1.3) $\mathbb{Z}_1$	• strain in generic direction<br>• ($\boldsymbol{E} \parallel [100]$ or $\boldsymbol{E} \perp [010]$) and strain $\perp [100]$<br>• ($\boldsymbol{E} \parallel [100]$ or $\boldsymbol{E} \perp [010]$) and strain $\perp [001]$<br>• ($\boldsymbol{E} \parallel [010]$ or $\boldsymbol{E} \perp [100]$) and strain $\perp [010]$<br>• ($\boldsymbol{E} \parallel [010]$ or $\boldsymbol{E} \perp [100]$) and strain $\perp [001]$<br>• $\boldsymbol{E}$ in generic direction
Pc (7.24) $\mathbb{Z}_1$	• $\boldsymbol{B} \parallel [010]$ and strain $\perp [010]$<br>• ($\boldsymbol{E} \parallel [100]$ or $\boldsymbol{E} \perp [010]$) and $\boldsymbol{B} \parallel [010]$
Pc' (7.26) $\mathbb{Z}_1$	• $\boldsymbol{B} \parallel [100]$ and strain $\perp [010]$<br>• $\boldsymbol{B} \parallel [001]$ and strain $\perp [010]$<br>• $\boldsymbol{B} \perp [010]$<br>• ($\boldsymbol{E} \parallel [100]$ or $\boldsymbol{E} \perp [010]$) and $\boldsymbol{B} \parallel [100]$<br>• ($\boldsymbol{E} \parallel [100]$ or $\boldsymbol{E} \perp [010]$) and $\boldsymbol{B} \parallel [001]$
P_bc (7.29) $\mathbb{Z}_1$	• strain $\perp [010]$<br>• ($\boldsymbol{E} \parallel [100]$ or $\boldsymbol{E} \perp [010]$)
Pm (6.18) $\mathbb{Z}_1$	• $\boldsymbol{B} \parallel [100]$ and strain $\perp [100]$<br>• ($\boldsymbol{E} \parallel [010]$ or $\boldsymbol{E} \perp [100]$) and $\boldsymbol{B} \parallel [100]$
Pc' (7.26) $\mathbb{Z}_1$	• $\boldsymbol{B} \parallel [010]$ and strain $\perp [100]$<br>• $\boldsymbol{B} \parallel [001]$ and strain $\perp [100]$<br>• $\boldsymbol{B} \perp [100]$<br>• ($\boldsymbol{E} \parallel [010]$ or $\boldsymbol{E} \perp [100]$) and $\boldsymbol{B} \parallel [010]$<br>• ($\boldsymbol{E} \parallel [010]$ or $\boldsymbol{E} \perp [100]$) and $\boldsymbol{B} \parallel [001]$
P_am (6.21) $\mathbb{Z}_1$	• strain $\perp [100]$<br>• ($\boldsymbol{E} \parallel [010]$ or $\boldsymbol{E} \perp [100]$)
$P2_1$ (4.7) $\mathbb{Z}_1$	• $\boldsymbol{B} \parallel [001]$ and strain $\perp [001]$
$Pc'a'2_1$ (29.103) $\mathbb{Z}_1$	• $\boldsymbol{B} \parallel [001]$
$P2_1'$ (4.9) $\mathbb{Z}_1$	• $\boldsymbol{B} \parallel [100]$ and strain $\perp [001]$<br>• $\boldsymbol{B} \parallel [010]$ and strain $\perp [001]$<br>• $\boldsymbol{B} \perp [001]$
$Pmc'2_1'$ (26.69) $\mathbb{Z}_1$	• $\boldsymbol{B} \parallel [100]$
$Pca'2_1'$ (29.102) $\mathbb{Z}_1$	• $\boldsymbol{B} \parallel [010]$
P_a2_1 (4.10) $\mathbb{Z}_1$	• strain $\perp [001]$

Table. S 183: Subgroups of P_cmc2_1 (26.73) that can be realized by applying $\boldsymbol{E}/\boldsymbol{B}$ fields and/or uniaxial strains.

Subgroup & SI group	Perturbation
P_S1 (1.3) $\mathbb{Z}_1$	• strain in generic direction<br>• ($\boldsymbol{E} \parallel [100]$ or $\boldsymbol{E} \perp [010]$) and strain $\perp [100]$<br>• ($\boldsymbol{E} \parallel [100]$ or $\boldsymbol{E} \perp [010]$) and strain $\perp [001]$<br>• ($\boldsymbol{E} \parallel [010]$ or $\boldsymbol{E} \perp [100]$) and strain $\perp [010]$<br>• ($\boldsymbol{E} \parallel [010]$ or $\boldsymbol{E} \perp [100]$) and strain $\perp [001]$<br>• $\boldsymbol{E}$ in generic direction
Pm' (6.20) $\mathbb{Z}_1$	• $\boldsymbol{B} \parallel [100]$ and strain $\perp [010]$<br>• $\boldsymbol{B} \parallel [001]$ and strain $\perp [010]$<br>• $\boldsymbol{B} \perp [010]$<br>• ($\boldsymbol{E} \parallel [100]$ or $\boldsymbol{E} \perp [010]$) and $\boldsymbol{B} \parallel [100]$<br>• ($\boldsymbol{E} \parallel [100]$ or $\boldsymbol{E} \perp [010]$) and $\boldsymbol{B} \parallel [001]$
Pc (7.24) $\mathbb{Z}_1$	• $\boldsymbol{B} \parallel [010]$ and strain $\perp [010]$<br>• ($\boldsymbol{E} \parallel [100]$ or $\boldsymbol{E} \perp [010]$) and $\boldsymbol{B} \parallel [010]$
P_cc (7.28) $\mathbb{Z}_1$	• strain $\perp [010]$<br>• ($\boldsymbol{E} \parallel [100]$ or $\boldsymbol{E} \perp [010]$)
Pm (6.18) $\mathbb{Z}_1$	• $\boldsymbol{B} \parallel [100]$ and strain $\perp [100]$<br>• ($\boldsymbol{E} \parallel [010]$ or $\boldsymbol{E} \perp [100]$) and $\boldsymbol{B} \parallel [100]$
Pc' (7.26) $\mathbb{Z}_1$	• $\boldsymbol{B} \parallel [010]$ and strain $\perp [100]$<br>• $\boldsymbol{B} \parallel [001]$ and strain $\perp [100]$<br>• $\boldsymbol{B} \perp [100]$<br>• ($\boldsymbol{E} \parallel [010]$ or $\boldsymbol{E} \perp [100]$) and $\boldsymbol{B} \parallel [010]$<br>• ($\boldsymbol{E} \parallel [010]$ or $\boldsymbol{E} \perp [100]$) and $\boldsymbol{B} \parallel [001]$
P_am (6.21) $\mathbb{Z}_1$	• strain $\perp [100]$<br>• ($\boldsymbol{E} \parallel [010]$ or $\boldsymbol{E} \perp [100]$)
$P2'$ (3.3) $\mathbb{Z}_1$	• $\boldsymbol{B} \parallel [100]$ and strain $\perp [001]$<br>• $\boldsymbol{B} \parallel [010]$ and strain $\perp [001]$<br>• $\boldsymbol{B} \perp [001]$
$Pm'm2'$ (25.59) $\mathbb{Z}_1$	• $\boldsymbol{B} \parallel [100]$
$Pc'c2'$ (27.80) $\mathbb{Z}_1$	• $\boldsymbol{B} \parallel [010]$
$P2_1$ (4.7) $\mathbb{Z}_1$	• $\boldsymbol{B} \parallel [001]$ and strain $\perp [001]$
$Pm'c'2_1$ (26.70) $\mathbb{Z}_1$	• $\boldsymbol{B} \parallel [001]$
P_b2_1 (4.11) $\mathbb{Z}_1$	• strain $\perp [001]$

Table. S 184: Subgroups of $P_A mc2_1$ (26.74) that can be realized by applying $\boldsymbol{E}/\boldsymbol{B}$ fields and/or uniaxial strains.

Subgroup & SI group	Perturbation
P_S1 (1.3) $\mathbb{Z}_1$	• strain in generic direction<br>• ($\boldsymbol{E} \parallel [100]$ or $\boldsymbol{E} \perp [010]$) and strain $\perp [100]$<br>• ($\boldsymbol{E} \parallel [100]$ or $\boldsymbol{E} \perp [010]$) and strain $\perp [001]$<br>• ($\boldsymbol{E} \parallel [010]$ or $\boldsymbol{E} \perp [100]$) and strain $\perp [010]$<br>• ($\boldsymbol{E} \parallel [010]$ or $\boldsymbol{E} \perp [100]$) and strain $\perp [001]$<br>• $\boldsymbol{E}$ in generic direction
Pc (7.24) $\mathbb{Z}_1$	• $\boldsymbol{B} \parallel [010]$ and strain $\perp [010]$<br>• ($\boldsymbol{E} \parallel [100]$ or $\boldsymbol{E} \perp [010]$) and $\boldsymbol{B} \parallel [010]$
Pm' (6.20) $\mathbb{Z}_1$	• $\boldsymbol{B} \parallel [100]$ and strain $\perp [010]$<br>• $\boldsymbol{B} \parallel [001]$ and strain $\perp [010]$<br>• $\boldsymbol{B} \perp [010]$<br>• ($\boldsymbol{E} \parallel [100]$ or $\boldsymbol{E} \perp [010]$) and $\boldsymbol{B} \parallel [100]$<br>• ($\boldsymbol{E} \parallel [100]$ or $\boldsymbol{E} \perp [010]$) and $\boldsymbol{B} \parallel [001]$
P_Ac (7.31) $\mathbb{Z}_1$	• strain $\perp [010]$<br>• ($\boldsymbol{E} \parallel [100]$ or $\boldsymbol{E} \perp [010]$)
Pm (6.18) $\mathbb{Z}_1$	• $\boldsymbol{B} \parallel [100]$ and strain $\perp [100]$<br>• ($\boldsymbol{E} \parallel [010]$ or $\boldsymbol{E} \perp [100]$) and $\boldsymbol{B} \parallel [100]$
Pc' (7.26) $\mathbb{Z}_1$	• $\boldsymbol{B} \parallel [010]$ and strain $\perp [100]$<br>• $\boldsymbol{B} \parallel [001]$ and strain $\perp [100]$<br>• $\boldsymbol{B} \perp [100]$<br>• ($\boldsymbol{E} \parallel [010]$ or $\boldsymbol{E} \perp [100]$) and $\boldsymbol{B} \parallel [010]$<br>• ($\boldsymbol{E} \parallel [010]$ or $\boldsymbol{E} \perp [100]$) and $\boldsymbol{B} \parallel [001]$
P_am (6.21) $\mathbb{Z}_1$	• strain $\perp [100]$<br>• ($\boldsymbol{E} \parallel [010]$ or $\boldsymbol{E} \perp [100]$)
$P2_1$ (4.7) $\mathbb{Z}_1$	• $\boldsymbol{B} \parallel [001]$ and strain $\perp [001]$
$Pm'n'2_1$ (31.127) $\mathbb{Z}_1$	• $\boldsymbol{B} \parallel [001]$
$P2'$ (3.3) $\mathbb{Z}_1$	• $\boldsymbol{B} \parallel [100]$ and strain $\perp [001]$<br>• $\boldsymbol{B} \parallel [010]$ and strain $\perp [001]$<br>• $\boldsymbol{B} \perp [001]$
$Pm'm2'$ (25.59) $\mathbb{Z}_1$	• $\boldsymbol{B} \parallel [100]$
$Pn'c2'$ (30.113) $\mathbb{Z}_1$	• $\boldsymbol{B} \parallel [010]$
P_C2_1 (4.12) $\mathbb{Z}_1$	• strain $\perp [001]$

Table. S 185: Subgroups of P_Bmc2_1 (26.75) that can be realized by applying $\boldsymbol{E}/\boldsymbol{B}$ fields and/or uniaxial strains.

Subgroup & SI group	Perturbation
Pc (7.24) $\mathbb{Z}_1$	• $\boldsymbol{B}$ ∥ [010] and strain ⊥ [010]<br>• ($\boldsymbol{E}$ ∥ [100] or $\boldsymbol{E}$ ⊥ [010]) and $\boldsymbol{B}$ ∥ [010]
P_S1 (1.3) $\mathbb{Z}_1$	• strain in generic direction<br>• ($\boldsymbol{E}$ ∥ [100] or $\boldsymbol{E}$ ⊥ [010]) and strain ⊥ [100]<br>• ($\boldsymbol{E}$ ∥ [100] or $\boldsymbol{E}$ ⊥ [010]) and strain ⊥ [001]<br>• ($\boldsymbol{E}$ ∥ [010] or $\boldsymbol{E}$ ⊥ [100]) and strain ⊥ [010]<br>• ($\boldsymbol{E}$ ∥ [010] or $\boldsymbol{E}$ ⊥ [100]) and strain ⊥ [001]<br>• $\boldsymbol{E}$ in generic direction
Pc' (7.26) $\mathbb{Z}_1$	• $\boldsymbol{B}$ ∥ [100] and strain ⊥ [010]<br>• $\boldsymbol{B}$ ∥ [001] and strain ⊥ [010]<br>• $\boldsymbol{B}$ ⊥ [010]<br>• ($\boldsymbol{E}$ ∥ [100] or $\boldsymbol{E}$ ⊥ [010]) and $\boldsymbol{B}$ ∥ [100]<br>• ($\boldsymbol{E}$ ∥ [100] or $\boldsymbol{E}$ ⊥ [010]) and $\boldsymbol{B}$ ∥ [001]
P_ac (7.27) $\mathbb{Z}_1$	• strain ⊥ [010]<br>• ($\boldsymbol{E}$ ∥ [100] or $\boldsymbol{E}$ ⊥ [010])
Pm (6.18) $\mathbb{Z}_1$	• $\boldsymbol{B}$ ∥ [100] and strain ⊥ [100]<br>• ($\boldsymbol{E}$ ∥ [010] or $\boldsymbol{E}$ ⊥ [100]) and $\boldsymbol{B}$ ∥ [100]
$P2_1$ (4.7) $\mathbb{Z}_1$	• $\boldsymbol{B}$ ∥ [001] and strain ⊥ [001]
Pc' (7.26) $\mathbb{Z}_1$	• $\boldsymbol{B}$ ∥ [010] and strain ⊥ [100]<br>• $\boldsymbol{B}$ ∥ [001] and strain ⊥ [100]<br>• $\boldsymbol{B}$ ⊥ [100]<br>• ($\boldsymbol{E}$ ∥ [010] or $\boldsymbol{E}$ ⊥ [100]) and $\boldsymbol{B}$ ∥ [010]<br>• ($\boldsymbol{E}$ ∥ [010] or $\boldsymbol{E}$ ⊥ [100]) and $\boldsymbol{B}$ ∥ [001]
P_Cm (6.23) $\mathbb{Z}_1$	• strain ⊥ [100]<br>• ($\boldsymbol{E}$ ∥ [010] or $\boldsymbol{E}$ ⊥ [100])
$Pc'a'2_1$ (29.103) $\mathbb{Z}_1$	• $\boldsymbol{B}$ ∥ [001]
$P2'$ (3.3) $\mathbb{Z}_1$	• $\boldsymbol{B}$ ∥ [100] and strain ⊥ [001]<br>• $\boldsymbol{B}$ ∥ [010] and strain ⊥ [001]<br>• $\boldsymbol{B}$ ⊥ [001]
$Pma'2'$ (28.90) $\mathbb{Z}_1$	• $\boldsymbol{B}$ ∥ [100]
P_C2_1 (4.12) $\mathbb{Z}_1$	• strain ⊥ [001]
$Pc'c2'$ (27.80) $\mathbb{Z}_1$	• $\boldsymbol{B}$ ∥ [010]

Table. S 186: Subgroups of P_Cmc2_1 (26.76) that can be realized by applying $\boldsymbol{E}/\boldsymbol{B}$ fields and/or uniaxial strains.

Subgroup & SI group	Perturbation
Pc (7.24) $\mathbb{Z}_1$	• $\boldsymbol{B} \parallel [010]$ and strain $\perp [010]$<br>• ($\boldsymbol{E} \parallel [100]$ or $\boldsymbol{E} \perp [010]$) and $\boldsymbol{B} \parallel [010]$
P_S1 (1.3) $\mathbb{Z}_1$	• strain in generic direction<br>• ($\boldsymbol{E} \parallel [100]$ or $\boldsymbol{E} \perp [010]$) and strain $\perp [100]$<br>• ($\boldsymbol{E} \parallel [100]$ or $\boldsymbol{E} \perp [010]$) and strain $\perp [001]$<br>• ($\boldsymbol{E} \parallel [010]$ or $\boldsymbol{E} \perp [100]$) and strain $\perp [010]$<br>• ($\boldsymbol{E} \parallel [010]$ or $\boldsymbol{E} \perp [100]$) and strain $\perp [001]$<br>• $\boldsymbol{E}$ in generic direction
Pc' (7.26) $\mathbb{Z}_1$	• $\boldsymbol{B} \parallel [100]$ and strain $\perp [010]$<br>• $\boldsymbol{B} \parallel [001]$ and strain $\perp [010]$<br>• $\boldsymbol{B} \perp [010]$<br>• ($\boldsymbol{E} \parallel [100]$ or $\boldsymbol{E} \perp [010]$) and $\boldsymbol{B} \parallel [100]$<br>• ($\boldsymbol{E} \parallel [100]$ or $\boldsymbol{E} \perp [010]$) and $\boldsymbol{B} \parallel [001]$
P_Cc (7.30) $\mathbb{Z}_1$	• strain $\perp [010]$<br>• ($\boldsymbol{E} \parallel [100]$ or $\boldsymbol{E} \perp [010]$)
Pm (6.18) $\mathbb{Z}_1$	• $\boldsymbol{B} \parallel [100]$ and strain $\perp [100]$<br>• ($\boldsymbol{E} \parallel [010]$ or $\boldsymbol{E} \perp [100]$) and $\boldsymbol{B} \parallel [100]$
$P2_1$ (4.7) $\mathbb{Z}_1$	• $\boldsymbol{B} \parallel [001]$ and strain $\perp [001]$
Pc' (7.26) $\mathbb{Z}_1$	• $\boldsymbol{B} \parallel [010]$ and strain $\perp [100]$<br>• $\boldsymbol{B} \parallel [001]$ and strain $\perp [100]$<br>• $\boldsymbol{B} \perp [100]$<br>• ($\boldsymbol{E} \parallel [010]$ or $\boldsymbol{E} \perp [100]$) and $\boldsymbol{B} \parallel [010]$<br>• ($\boldsymbol{E} \parallel [010]$ or $\boldsymbol{E} \perp [100]$) and $\boldsymbol{B} \parallel [001]$
P_Cm (6.23) $\mathbb{Z}_1$	• strain $\perp [100]$<br>• ($\boldsymbol{E} \parallel [010]$ or $\boldsymbol{E} \perp [100]$)
$Pn'a'2_1$ (33.148) $\mathbb{Z}_1$	• $\boldsymbol{B} \parallel [001]$
$P2_1'$ (4.9) $\mathbb{Z}_1$	• $\boldsymbol{B} \parallel [100]$ and strain $\perp [001]$<br>• $\boldsymbol{B} \parallel [010]$ and strain $\perp [001]$<br>• $\boldsymbol{B} \perp [001]$
$Pmn'2_1'$ (31.126) $\mathbb{Z}_1$	• $\boldsymbol{B} \parallel [100]$
P_a2_1 (4.10) $\mathbb{Z}_1$	• strain $\perp [001]$
$Pca'2_1'$ (29.102) $\mathbb{Z}_1$	• $\boldsymbol{B} \parallel [010]$

Table. S 187: Subgroups of P_Imc2_1 (26.77) that can be realized by applying $\boldsymbol{E}/\boldsymbol{B}$ fields and/or uniaxial strains.

Subgroup & SI group	Perturbation
Pc (7.24) $\mathbb{Z}_1$	• $\boldsymbol{B} \parallel [010]$ and strain $\perp [010]$<br>• ($\boldsymbol{E} \parallel [100]$ or $\boldsymbol{E} \perp [010]$) and $\boldsymbol{B} \parallel [010]$
P_S1 (1.3) $\mathbb{Z}_1$	• strain in generic direction<br>• ($\boldsymbol{E} \parallel [100]$ or $\boldsymbol{E} \perp [010]$) and strain $\perp [100]$<br>• ($\boldsymbol{E} \parallel [100]$ or $\boldsymbol{E} \perp [010]$) and strain $\perp [001]$<br>• ($\boldsymbol{E} \parallel [010]$ or $\boldsymbol{E} \perp [100]$) and strain $\perp [010]$<br>• ($\boldsymbol{E} \parallel [010]$ or $\boldsymbol{E} \perp [100]$) and strain $\perp [001]$<br>• $\boldsymbol{E}$ in generic direction
Pc' (7.26) $\mathbb{Z}_1$	• $\boldsymbol{B} \parallel [100]$ and strain $\perp [010]$<br>• $\boldsymbol{B} \parallel [001]$ and strain $\perp [010]$<br>• $\boldsymbol{B} \perp [010]$<br>• ($\boldsymbol{E} \parallel [100]$ or $\boldsymbol{E} \perp [010]$) and $\boldsymbol{B} \parallel [100]$<br>• ($\boldsymbol{E} \parallel [100]$ or $\boldsymbol{E} \perp [010]$) and $\boldsymbol{B} \parallel [001]$
P_Cc (7.30) $\mathbb{Z}_1$	• strain $\perp [010]$<br>• ($\boldsymbol{E} \parallel [100]$ or $\boldsymbol{E} \perp [010]$)
Pm (6.18) $\mathbb{Z}_1$	• $\boldsymbol{B} \parallel [100]$ and strain $\perp [100]$<br>• ($\boldsymbol{E} \parallel [010]$ or $\boldsymbol{E} \perp [100]$) and $\boldsymbol{B} \parallel [100]$
$P2_1$ (4.7) $\mathbb{Z}_1$	• $\boldsymbol{B} \parallel [001]$ and strain $\perp [001]$
Pc' (7.26) $\mathbb{Z}_1$	• $\boldsymbol{B} \parallel [010]$ and strain $\perp [100]$<br>• $\boldsymbol{B} \parallel [001]$ and strain $\perp [100]$<br>• $\boldsymbol{B} \perp [100]$<br>• ($\boldsymbol{E} \parallel [010]$ or $\boldsymbol{E} \perp [100]$) and $\boldsymbol{B} \parallel [010]$<br>• ($\boldsymbol{E} \parallel [010]$ or $\boldsymbol{E} \perp [100]$) and $\boldsymbol{B} \parallel [001]$
P_Cm (6.23) $\mathbb{Z}_1$	• strain $\perp [100]$<br>• ($\boldsymbol{E} \parallel [010]$ or $\boldsymbol{E} \perp [100]$)
$Pn'a'2_1$ (33.148) $\mathbb{Z}_1$	• $\boldsymbol{B} \parallel [001]$
$P2'$ (3.3) $\mathbb{Z}_1$	• $\boldsymbol{B} \parallel [100]$ and strain $\perp [001]$<br>• $\boldsymbol{B} \parallel [010]$ and strain $\perp [001]$<br>• $\boldsymbol{B} \perp [001]$
$Pma'2'$ (28.90) $\mathbb{Z}_1$	• $\boldsymbol{B} \parallel [100]$
P_C2_1 (4.12) $\mathbb{Z}_1$	• strain $\perp [001]$
$Pn'c2'$ (30.113) $\mathbb{Z}_1$	• $\boldsymbol{B} \parallel [010]$

Table. S 188: Subgroups of $Pcc2$ (27.78) that can be realized by applying $\boldsymbol{E}/\boldsymbol{B}$ fields and/or uniaxial strains.

Subgroup & SI group	Perturbation
Pc (7.24) $\mathbb{Z}_1$	• strain $\perp$ [010]<br>• $\boldsymbol{B} \parallel$ [010]<br>• ($\boldsymbol{E} \parallel$ [100] or $\boldsymbol{E} \perp$ [010])
Pc (7.24) $\mathbb{Z}_1$	• strain $\perp$ [100]<br>• $\boldsymbol{B} \parallel$ [100]<br>• ($\boldsymbol{E} \parallel$ [010] or $\boldsymbol{E} \perp$ [100])
$P2$ (3.1) $\mathbb{Z}_2$	• strain $\perp$ [001]<br>• $\boldsymbol{B} \parallel$ [001]

Table. S 189: Subgroups of $Pcc21'$ (27.79) that can be realized by applying $\boldsymbol{E}/\boldsymbol{B}$ fields and/or uniaxial strains.

Subgroup & SI group	Perturbation
$P11'$ (1.2) $\mathbb{Z}_1$	• strain in generic direction<br>• ($\boldsymbol{E} \parallel$ [100] or $\boldsymbol{E} \perp$ [010]) and strain $\perp$ [100]<br>• ($\boldsymbol{E} \parallel$ [100] or $\boldsymbol{E} \perp$ [010]) and strain $\perp$ [001]<br>• ($\boldsymbol{E} \parallel$ [010] or $\boldsymbol{E} \perp$ [100]) and strain $\perp$ [010]<br>• ($\boldsymbol{E} \parallel$ [010] or $\boldsymbol{E} \perp$ [100]) and strain $\perp$ [001]<br>• $\boldsymbol{E}$ in generic direction
Pc' (7.26) $\mathbb{Z}_1$	• $\boldsymbol{B} \parallel$ [100] and strain $\perp$ [010]<br>• $\boldsymbol{B} \parallel$ [001] and strain $\perp$ [010]<br>• $\boldsymbol{B} \perp$ [010]<br>• ($\boldsymbol{E} \parallel$ [100] or $\boldsymbol{E} \perp$ [010]) and $\boldsymbol{B} \parallel$ [100]<br>• ($\boldsymbol{E} \parallel$ [100] or $\boldsymbol{E} \perp$ [010]) and $\boldsymbol{B} \parallel$ [001]
Pc (7.24) $\mathbb{Z}_1$	• $\boldsymbol{B} \parallel$ [010] and strain $\perp$ [010]<br>• ($\boldsymbol{E} \parallel$ [100] or $\boldsymbol{E} \perp$ [010]) and $\boldsymbol{B} \parallel$ [010]
$Pc1'$ (7.25) $\mathbb{Z}_1$	• strain $\perp$ [010]<br>• ($\boldsymbol{E} \parallel$ [100] or $\boldsymbol{E} \perp$ [010])
Pc' (7.26) $\mathbb{Z}_1$	• $\boldsymbol{B} \parallel$ [010] and strain $\perp$ [100]<br>• $\boldsymbol{B} \parallel$ [001] and strain $\perp$ [100]<br>• $\boldsymbol{B} \perp$ [100]<br>• ($\boldsymbol{E} \parallel$ [010] or $\boldsymbol{E} \perp$ [100]) and $\boldsymbol{B} \parallel$ [010]<br>• ($\boldsymbol{E} \parallel$ [010] or $\boldsymbol{E} \perp$ [100]) and $\boldsymbol{B} \parallel$ [001]

Continued on next page

...continued

Subgroup & SI group	Perturbation
Pc (7.24) $\mathbb{Z}_1$	• $\boldsymbol{B}$ ∥ [100] and strain ⊥ [100]<br>• ($\boldsymbol{E}$ ∥ [010] or $\boldsymbol{E}$ ⊥ [100]) and $\boldsymbol{B}$ ∥ [100]
$Pc1'$ (7.25) $\mathbb{Z}_1$	• strain ⊥ [100]<br>• ($\boldsymbol{E}$ ∥ [010] or $\boldsymbol{E}$ ⊥ [100])
$P2'$ (3.3) $\mathbb{Z}_1$	• $\boldsymbol{B}$ ∥ [100] and strain ⊥ [001]<br>• $\boldsymbol{B}$ ∥ [010] and strain ⊥ [001]<br>• $\boldsymbol{B}$ ⊥ [001]
$Pc'c2'$ (27.80) $\mathbb{Z}_1$	• $\boldsymbol{B}$ ∥ [010]
$Pc'c2'$ (27.80) $\mathbb{Z}_1$	• $\boldsymbol{B}$ ∥ [100]
$P2$ (3.1) $\mathbb{Z}_2$	• $\boldsymbol{B}$ ∥ [001] and strain ⊥ [001]
$Pc'c'2$ (27.81) $\mathbb{Z}_2$	• $\boldsymbol{B}$ ∥ [001]
$P21'$ (3.2) $\mathbb{Z}_2$	• strain ⊥ [001]

Table. S 190: Subgroups of $Pc'c2'$ (27.80) that can be realized by applying $\boldsymbol{E}/\boldsymbol{B}$ fields and/or uniaxial strains.

Subgroup & SI group	Perturbation
Pc (7.24) $\mathbb{Z}_1$	• strain ⊥ [010]<br>• ($\boldsymbol{E}$ ∥ [100] or $\boldsymbol{E}$ ⊥ [010])
Pc' (7.26) $\mathbb{Z}_1$	• strain ⊥ [100]<br>• ($\boldsymbol{B}$ ∥ [001] or $\boldsymbol{B}$ ⊥ [100])<br>• ($\boldsymbol{E}$ ∥ [010] or $\boldsymbol{E}$ ⊥ [100])
$P2'$ (3.3) $\mathbb{Z}_1$	• strain ⊥ [001]<br>• ($\boldsymbol{B}$ ∥ [100] or $\boldsymbol{B}$ ⊥ [001])

Table. S 191: Subgroups of $Pc'c'2$ (27.81) that can be realized by applying $\boldsymbol{E}/\boldsymbol{B}$ fields and/or uniaxial strains.

Subgroup & SI group	Perturbation

Continued on next page

. . . continued

Subgroup & SI group	Perturbation
Pc' (7.26) $\mathbb{Z}_1$	• strain $\perp$ [010]<br>• ($\boldsymbol{B}$ $\parallel$ [100] or $\boldsymbol{B}$ $\perp$ [010])<br>• ($\boldsymbol{E}$ $\parallel$ [100] or $\boldsymbol{E}$ $\perp$ [010])
Pc' (7.26) $\mathbb{Z}_1$	• strain $\perp$ [100]<br>• ($\boldsymbol{B}$ $\parallel$ [010] or $\boldsymbol{B}$ $\perp$ [100])<br>• ($\boldsymbol{E}$ $\parallel$ [010] or $\boldsymbol{E}$ $\perp$ [100])
$P2$ (3.1) $\mathbb{Z}_2$	• strain $\perp$ [001]

Table. S 192: Subgroups of $P_c cc2$ (27.82) that can be realized by applying $\boldsymbol{E}/\boldsymbol{B}$ fields and/or uniaxial strains.

Subgroup & SI group	Perturbation
P_S1 (1.3) $\mathbb{Z}_1$	• strain in generic direction<br>• ($\boldsymbol{E}$ $\parallel$ [100] or $\boldsymbol{E}$ $\perp$ [010]) and strain $\perp$ [100]<br>• ($\boldsymbol{E}$ $\parallel$ [100] or $\boldsymbol{E}$ $\perp$ [010]) and strain $\perp$ [001]<br>• ($\boldsymbol{E}$ $\parallel$ [010] or $\boldsymbol{E}$ $\perp$ [100]) and strain $\perp$ [010]<br>• ($\boldsymbol{E}$ $\parallel$ [010] or $\boldsymbol{E}$ $\perp$ [100]) and strain $\perp$ [001]<br>• $\boldsymbol{E}$ in generic direction
Pm' (6.20) $\mathbb{Z}_1$	• $\boldsymbol{B}$ $\parallel$ [100] and strain $\perp$ [010]<br>• $\boldsymbol{B}$ $\parallel$ [001] and strain $\perp$ [010]<br>• $\boldsymbol{B}$ $\perp$ [010]<br>• ($\boldsymbol{E}$ $\parallel$ [100] or $\boldsymbol{E}$ $\perp$ [010]) and $\boldsymbol{B}$ $\parallel$ [100]<br>• ($\boldsymbol{E}$ $\parallel$ [100] or $\boldsymbol{E}$ $\perp$ [010]) and $\boldsymbol{B}$ $\parallel$ [001]
Pc (7.24) $\mathbb{Z}_1$	• $\boldsymbol{B}$ $\parallel$ [010] and strain $\perp$ [010]<br>• ($\boldsymbol{E}$ $\parallel$ [100] or $\boldsymbol{E}$ $\perp$ [010]) and $\boldsymbol{B}$ $\parallel$ [010]
P_cc (7.28) $\mathbb{Z}_1$	• strain $\perp$ [010]<br>• ($\boldsymbol{E}$ $\parallel$ [100] or $\boldsymbol{E}$ $\perp$ [010])
Pm' (6.20) $\mathbb{Z}_1$	• $\boldsymbol{B}$ $\parallel$ [010] and strain $\perp$ [100]<br>• $\boldsymbol{B}$ $\parallel$ [001] and strain $\perp$ [100]<br>• $\boldsymbol{B}$ $\perp$ [100]<br>• ($\boldsymbol{E}$ $\parallel$ [010] or $\boldsymbol{E}$ $\perp$ [100]) and $\boldsymbol{B}$ $\parallel$ [010]<br>• ($\boldsymbol{E}$ $\parallel$ [010] or $\boldsymbol{E}$ $\perp$ [100]) and $\boldsymbol{B}$ $\parallel$ [001]
Pc (7.24) $\mathbb{Z}_1$	• $\boldsymbol{B}$ $\parallel$ [100] and strain $\perp$ [100]<br>• ($\boldsymbol{E}$ $\parallel$ [010] or $\boldsymbol{E}$ $\perp$ [100]) and $\boldsymbol{B}$ $\parallel$ [100]

Continued on next page

...continued

Subgroup & SI group	Perturbation
$P_c c$ (7.28) $\mathbb{Z}_1$	• strain ⊥ [100]<br>• ($\boldsymbol{E}$ ∥ [010] or $\boldsymbol{E}$ ⊥ [100])
$P2$ (3.1) $\mathbb{Z}_2$	• $\boldsymbol{B}$ ∥ [001] and strain ⊥ [001]
$Pm'm'2$ (25.60) $\mathbb{Z}_2$	• $\boldsymbol{B}$ ∥ [001]
$P2_1'$ (4.9) $\mathbb{Z}_1$	• $\boldsymbol{B}$ ∥ [100] and strain ⊥ [001]<br>• $\boldsymbol{B}$ ∥ [010] and strain ⊥ [001]<br>• $\boldsymbol{B}$ ⊥ [001]
$Pm'c2_1'$ (26.68) $\mathbb{Z}_1$	• $\boldsymbol{B}$ ∥ [010]
$Pm'c2_1'$ (26.68) $\mathbb{Z}_1$	• $\boldsymbol{B}$ ∥ [100]
P_b2 (3.5) $\mathbb{Z}_2$	• strain ⊥ [001]

Table. S 193: Subgroups of P_acc2 (27.83) that can be realized by applying $\boldsymbol{E}/\boldsymbol{B}$ fields and/or uniaxial strains.

Subgroup & SI group	Perturbation
Pc (7.24) $\mathbb{Z}_1$	• $\boldsymbol{B}$ ∥ [010] and strain ⊥ [010]<br>• ($\boldsymbol{E}$ ∥ [100] or $\boldsymbol{E}$ ⊥ [010]) and $\boldsymbol{B}$ ∥ [010]
P_S1 (1.3) $\mathbb{Z}_1$	• strain in generic direction<br>• ($\boldsymbol{E}$ ∥ [100] or $\boldsymbol{E}$ ⊥ [010]) and strain ⊥ [100]<br>• ($\boldsymbol{E}$ ∥ [100] or $\boldsymbol{E}$ ⊥ [010]) and strain ⊥ [001]<br>• ($\boldsymbol{E}$ ∥ [010] or $\boldsymbol{E}$ ⊥ [100]) and strain ⊥ [010]<br>• ($\boldsymbol{E}$ ∥ [010] or $\boldsymbol{E}$ ⊥ [100]) and strain ⊥ [001]<br>• $\boldsymbol{E}$ in generic direction
Pc' (7.26) $\mathbb{Z}_1$	• $\boldsymbol{B}$ ∥ [100] and strain ⊥ [010]<br>• $\boldsymbol{B}$ ∥ [001] and strain ⊥ [010]<br>• $\boldsymbol{B}$ ⊥ [010]<br>• ($\boldsymbol{E}$ ∥ [100] or $\boldsymbol{E}$ ⊥ [010]) and $\boldsymbol{B}$ ∥ [100]<br>• ($\boldsymbol{E}$ ∥ [100] or $\boldsymbol{E}$ ⊥ [010]) and $\boldsymbol{B}$ ∥ [001]
P_ac (7.27) $\mathbb{Z}_1$	• strain ⊥ [010]<br>• ($\boldsymbol{E}$ ∥ [100] or $\boldsymbol{E}$ ⊥ [010])
Pc (7.24) $\mathbb{Z}_1$	• $\boldsymbol{B}$ ∥ [100] and strain ⊥ [100]<br>• ($\boldsymbol{E}$ ∥ [010] or $\boldsymbol{E}$ ⊥ [100]) and $\boldsymbol{B}$ ∥ [100]
$P2$ (3.1) $\mathbb{Z}_2$	• $\boldsymbol{B}$ ∥ [001] and strain ⊥ [001]

Continued on next page

. . . continued

Subgroup & SI group	Perturbation
Pc' (7.26) $\mathbb{Z}_1$	• $\boldsymbol{B} \parallel [010]$ and strain $\perp [100]$<br>• $\boldsymbol{B} \parallel [001]$ and strain $\perp [100]$<br>• $\boldsymbol{B} \perp [100]$<br>• ($\boldsymbol{E} \parallel [010]$ or $\boldsymbol{E} \perp [100]$) and $\boldsymbol{B} \parallel [010]$<br>• ($\boldsymbol{E} \parallel [010]$ or $\boldsymbol{E} \perp [100]$) and $\boldsymbol{B} \parallel [001]$
P_bc (7.29) $\mathbb{Z}_1$	• strain $\perp [100]$<br>• ($\boldsymbol{E} \parallel [010]$ or $\boldsymbol{E} \perp [100]$)
$Pn'c'2$ (30.115) $\mathbb{Z}_1$	• $\boldsymbol{B} \parallel [001]$
$P2'$ (3.3) $\mathbb{Z}_1$	• $\boldsymbol{B} \parallel [100]$ and strain $\perp [001]$<br>• $\boldsymbol{B} \parallel [010]$ and strain $\perp [001]$<br>• $\boldsymbol{B} \perp [001]$
$Pn'c2'$ (30.113) $\mathbb{Z}_1$	• $\boldsymbol{B} \parallel [100]$
P_a2 (3.4) $\mathbb{Z}_2$	• strain $\perp [001]$
$Pc'c2'$ (27.80) $\mathbb{Z}_1$	• $\boldsymbol{B} \parallel [010]$

Table. S 194: Subgroups of P_Ccc2 (27.84) that can be realized by applying $\boldsymbol{E}/\boldsymbol{B}$ fields and/or uniaxial strains.

Subgroup & SI group	Perturbation
Pc (7.24) $\mathbb{Z}_1$	• $\boldsymbol{B} \parallel [010]$ and strain $\perp [010]$<br>• ($\boldsymbol{E} \parallel [100]$ or $\boldsymbol{E} \perp [010]$) and $\boldsymbol{B} \parallel [010]$
P_S1 (1.3) $\mathbb{Z}_1$	• strain in generic direction<br>• ($\boldsymbol{E} \parallel [100]$ or $\boldsymbol{E} \perp [010]$) and strain $\perp [100]$<br>• ($\boldsymbol{E} \parallel [100]$ or $\boldsymbol{E} \perp [010]$) and strain $\perp [001]$<br>• ($\boldsymbol{E} \parallel [010]$ or $\boldsymbol{E} \perp [100]$) and strain $\perp [010]$<br>• ($\boldsymbol{E} \parallel [010]$ or $\boldsymbol{E} \perp [100]$) and strain $\perp [001]$<br>• $\boldsymbol{E}$ in generic direction
Pc' (7.26) $\mathbb{Z}_1$	• $\boldsymbol{B} \parallel [100]$ and strain $\perp [010]$<br>• $\boldsymbol{B} \parallel [001]$ and strain $\perp [010]$<br>• $\boldsymbol{B} \perp [010]$<br>• ($\boldsymbol{E} \parallel [100]$ or $\boldsymbol{E} \perp [010]$) and $\boldsymbol{B} \parallel [100]$<br>• ($\boldsymbol{E} \parallel [100]$ or $\boldsymbol{E} \perp [010]$) and $\boldsymbol{B} \parallel [001]$
P_Cc (7.30) $\mathbb{Z}_1$	• strain $\perp [010]$<br>• ($\boldsymbol{E} \parallel [100]$ or $\boldsymbol{E} \perp [010]$)

Continued on next page

...continued

Subgroup & SI group	Perturbation
Pc (7.24) $\mathbb{Z}_1$	• $\boldsymbol{B} \parallel [100]$ and strain $\perp [100]$<br>• ($\boldsymbol{E} \parallel [010]$ or $\boldsymbol{E} \perp [100]$) and $\boldsymbol{B} \parallel [100]$
$P2$ (3.1) $\mathbb{Z}_2$	• $\boldsymbol{B} \parallel [001]$ and strain $\perp [001]$
Pc' (7.26) $\mathbb{Z}_1$	• $\boldsymbol{B} \parallel [010]$ and strain $\perp [100]$<br>• $\boldsymbol{B} \parallel [001]$ and strain $\perp [100]$<br>• $\boldsymbol{B} \perp [100]$<br>• ($\boldsymbol{E} \parallel [010]$ or $\boldsymbol{E} \perp [100]$) and $\boldsymbol{B} \parallel [010]$<br>• ($\boldsymbol{E} \parallel [010]$ or $\boldsymbol{E} \perp [100]$) and $\boldsymbol{B} \parallel [001]$
P_Cc (7.30) $\mathbb{Z}_1$	• strain $\perp [100]$<br>• ($\boldsymbol{E} \parallel [010]$ or $\boldsymbol{E} \perp [100]$)
$Pn'n'2$ (34.159) $\mathbb{Z}_1$	• $\boldsymbol{B} \parallel [001]$
$P2'$ (3.3) $\mathbb{Z}_1$	• $\boldsymbol{B} \parallel [100]$ and strain $\perp [001]$<br>• $\boldsymbol{B} \parallel [010]$ and strain $\perp [001]$<br>• $\boldsymbol{B} \perp [001]$
$Pn'c2'$ (30.113) $\mathbb{Z}_1$	• $\boldsymbol{B} \parallel [100]$
P_a2 (3.4) $\mathbb{Z}_2$	• strain $\perp [001]$
$Pn'c2'$ (30.113) $\mathbb{Z}_1$	• $\boldsymbol{B} \parallel [010]$

Table. S 195: Subgroups of P_Acc2 (27.85) that can be realized by applying $\boldsymbol{E}/\boldsymbol{B}$ fields and/or uniaxial strains.

Subgroup & SI group	Perturbation
P_S1 (1.3) $\mathbb{Z}_1$	• strain in generic direction<br>• ($\boldsymbol{E} \parallel [100]$ or $\boldsymbol{E} \perp [010]$) and strain $\perp [100]$<br>• ($\boldsymbol{E} \parallel [100]$ or $\boldsymbol{E} \perp [010]$) and strain $\perp [001]$<br>• ($\boldsymbol{E} \parallel [010]$ or $\boldsymbol{E} \perp [100]$) and strain $\perp [010]$<br>• ($\boldsymbol{E} \parallel [010]$ or $\boldsymbol{E} \perp [100]$) and strain $\perp [001]$<br>• $\boldsymbol{E}$ in generic direction
Pc (7.24) $\mathbb{Z}_1$	• $\boldsymbol{B} \parallel [010]$ and strain $\perp [010]$<br>• ($\boldsymbol{E} \parallel [100]$ or $\boldsymbol{E} \perp [010]$) and $\boldsymbol{B} \parallel [010]$

Continued on next page

. . . continued

Subgroup & SI group	Perturbation
Pm' (6.20) $\mathbb{Z}_1$	• $\boldsymbol{B} \parallel [100]$ and strain $\perp [010]$<br>• $\boldsymbol{B} \parallel [001]$ and strain $\perp [010]$<br>• $\boldsymbol{B} \perp [010]$<br>• ($\boldsymbol{E} \parallel [100]$ or $\boldsymbol{E} \perp [010]$) and $\boldsymbol{B} \parallel [100]$<br>• ($\boldsymbol{E} \parallel [100]$ or $\boldsymbol{E} \perp [010]$) and $\boldsymbol{B} \parallel [001]$
P_Ac (7.31) $\mathbb{Z}_1$	• strain $\perp [010]$<br>• ($\boldsymbol{E} \parallel [100]$ or $\boldsymbol{E} \perp [010]$)
Pc (7.24) $\mathbb{Z}_1$	• $\boldsymbol{B} \parallel [100]$ and strain $\perp [100]$<br>• ($\boldsymbol{E} \parallel [010]$ or $\boldsymbol{E} \perp [100]$) and $\boldsymbol{B} \parallel [100]$
Pc' (7.26) $\mathbb{Z}_1$	• $\boldsymbol{B} \parallel [010]$ and strain $\perp [100]$<br>• $\boldsymbol{B} \parallel [001]$ and strain $\perp [100]$<br>• $\boldsymbol{B} \perp [100]$<br>• ($\boldsymbol{E} \parallel [010]$ or $\boldsymbol{E} \perp [100]$) and $\boldsymbol{B} \parallel [010]$<br>• ($\boldsymbol{E} \parallel [010]$ or $\boldsymbol{E} \perp [100]$) and $\boldsymbol{B} \parallel [001]$
P_ac (7.27) $\mathbb{Z}_1$	• strain $\perp [100]$<br>• ($\boldsymbol{E} \parallel [010]$ or $\boldsymbol{E} \perp [100]$)
$P2$ (3.1) $\mathbb{Z}_2$	• $\boldsymbol{B} \parallel [001]$ and strain $\perp [001]$
$Pm'a'2$ (28.91) $\mathbb{Z}_2$	• $\boldsymbol{B} \parallel [001]$
$P2_1'$ (4.9) $\mathbb{Z}_1$	• $\boldsymbol{B} \parallel [100]$ and strain $\perp [001]$<br>• $\boldsymbol{B} \parallel [010]$ and strain $\perp [001]$<br>• $\boldsymbol{B} \perp [001]$
$Pm'c2_1'$ (26.68) $\mathbb{Z}_1$	• $\boldsymbol{B} \parallel [100]$
$Pca'2_1'$ (29.102) $\mathbb{Z}_1$	• $\boldsymbol{B} \parallel [010]$
P_C2 (3.6) $\mathbb{Z}_1$	• strain $\perp [001]$

Table. S 196: Subgroups of P_Icc2 (27.86) that can be realized by applying $\boldsymbol{E}/\boldsymbol{B}$ fields and/or uniaxial strains.

Subgroup & SI group	Perturbation
Pc (7.24) $\mathbb{Z}_1$	• $\boldsymbol{B} \parallel [010]$ and strain $\perp [010]$<br>• ($\boldsymbol{E} \parallel [100]$ or $\boldsymbol{E} \perp [010]$) and $\boldsymbol{B} \parallel [010]$

Continued on next page

. . . continued

Subgroup & SI group	Perturbation
P_S1 (1.3) $\mathbb{Z}_1$	• strain in generic direction<br>• ($\boldsymbol{E} \parallel [100]$ or $\boldsymbol{E} \perp [010]$) and strain $\perp [100]$<br>• ($\boldsymbol{E} \parallel [100]$ or $\boldsymbol{E} \perp [010]$) and strain $\perp [001]$<br>• ($\boldsymbol{E} \parallel [010]$ or $\boldsymbol{E} \perp [100]$) and strain $\perp [010]$<br>• ($\boldsymbol{E} \parallel [010]$ or $\boldsymbol{E} \perp [100]$) and strain $\perp [001]$<br>• $\boldsymbol{E}$ in generic direction
Pc' (7.26) $\mathbb{Z}_1$	• $\boldsymbol{B} \parallel [100]$ and strain $\perp [010]$<br>• $\boldsymbol{B} \parallel [001]$ and strain $\perp [010]$<br>• $\boldsymbol{B} \perp [010]$<br>• ($\boldsymbol{E} \parallel [100]$ or $\boldsymbol{E} \perp [010]$) and $\boldsymbol{B} \parallel [100]$<br>• ($\boldsymbol{E} \parallel [100]$ or $\boldsymbol{E} \perp [010]$) and $\boldsymbol{B} \parallel [001]$
P_Cc (7.30) $\mathbb{Z}_1$	• strain $\perp [010]$<br>• ($\boldsymbol{E} \parallel [100]$ or $\boldsymbol{E} \perp [010]$)
Pc (7.24) $\mathbb{Z}_1$	• $\boldsymbol{B} \parallel [100]$ and strain $\perp [100]$<br>• ($\boldsymbol{E} \parallel [010]$ or $\boldsymbol{E} \perp [100]$) and $\boldsymbol{B} \parallel [100]$
$P2$ (3.1) $\mathbb{Z}_2$	• $\boldsymbol{B} \parallel [001]$ and strain $\perp [001]$
Pc' (7.26) $\mathbb{Z}_1$	• $\boldsymbol{B} \parallel [010]$ and strain $\perp [100]$<br>• $\boldsymbol{B} \parallel [001]$ and strain $\perp [100]$<br>• $\boldsymbol{B} \perp [100]$<br>• ($\boldsymbol{E} \parallel [010]$ or $\boldsymbol{E} \perp [100]$) and $\boldsymbol{B} \parallel [010]$<br>• ($\boldsymbol{E} \parallel [010]$ or $\boldsymbol{E} \perp [100]$) and $\boldsymbol{B} \parallel [001]$
P_Cc (7.30) $\mathbb{Z}_1$	• strain $\perp [100]$<br>• ($\boldsymbol{E} \parallel [010]$ or $\boldsymbol{E} \perp [100]$)
$Pb'a'2$ (32.138) $\mathbb{Z}_2$	• $\boldsymbol{B} \parallel [001]$
$P2_1'$ (4.9) $\mathbb{Z}_1$	• $\boldsymbol{B} \parallel [100]$ and strain $\perp [001]$<br>• $\boldsymbol{B} \parallel [010]$ and strain $\perp [001]$<br>• $\boldsymbol{B} \perp [001]$
$Pca'2_1'$ (29.102) $\mathbb{Z}_1$	• $\boldsymbol{B} \parallel [100]$
P_C2 (3.6) $\mathbb{Z}_1$	• strain $\perp [001]$
$Pca'2_1'$ (29.102) $\mathbb{Z}_1$	• $\boldsymbol{B} \parallel [010]$

Table. S 197: Subgroups of $Pma2$ (28.87) that can be realized by applying $\boldsymbol{E}/\boldsymbol{B}$ fields and/or uniaxial strains.

Subgroup & SI group	Perturbation
Pc (7.24) $\mathbb{Z}_1$	• strain $\perp$ [010]<br>• $\boldsymbol{B}$ $\parallel$ [010]<br>• ($\boldsymbol{E}$ $\parallel$ [100] or $\boldsymbol{E}$ $\perp$ [010])
$P2$ (3.1) $\mathbb{Z}_2$	• strain $\perp$ [001]<br>• $\boldsymbol{B}$ $\parallel$ [001]
Pm (6.18) $\mathbb{Z}_1$	• strain $\perp$ [100]<br>• $\boldsymbol{B}$ $\parallel$ [100]<br>• ($\boldsymbol{E}$ $\parallel$ [010] or $\boldsymbol{E}$ $\perp$ [100])

Table. S 198: Subgroups of $Pma21'$ (28.88) that can be realized by applying $\boldsymbol{E}/\boldsymbol{B}$ fields and/or uniaxial strains.

Subgroup & SI group	Perturbation
$P11'$ (1.2) $\mathbb{Z}_1$	• strain in generic direction<br>• ($\boldsymbol{E}$ $\parallel$ [100] or $\boldsymbol{E}$ $\perp$ [010]) and strain $\perp$ [100]<br>• ($\boldsymbol{E}$ $\parallel$ [100] or $\boldsymbol{E}$ $\perp$ [010]) and strain $\perp$ [001]<br>• ($\boldsymbol{E}$ $\parallel$ [010] or $\boldsymbol{E}$ $\perp$ [100]) and strain $\perp$ [010]<br>• ($\boldsymbol{E}$ $\parallel$ [010] or $\boldsymbol{E}$ $\perp$ [100]) and strain $\perp$ [001]<br>• $\boldsymbol{E}$ in generic direction
Pc' (7.26) $\mathbb{Z}_1$	• $\boldsymbol{B}$ $\parallel$ [100] and strain $\perp$ [010]<br>• $\boldsymbol{B}$ $\parallel$ [001] and strain $\perp$ [010]<br>• $\boldsymbol{B}$ $\perp$ [010]<br>• ($\boldsymbol{E}$ $\parallel$ [100] or $\boldsymbol{E}$ $\perp$ [010]) and $\boldsymbol{B}$ $\parallel$ [100]<br>• ($\boldsymbol{E}$ $\parallel$ [100] or $\boldsymbol{E}$ $\perp$ [010]) and $\boldsymbol{B}$ $\parallel$ [001]
Pc (7.24) $\mathbb{Z}_1$	• $\boldsymbol{B}$ $\parallel$ [010] and strain $\perp$ [010]<br>• ($\boldsymbol{E}$ $\parallel$ [100] or $\boldsymbol{E}$ $\perp$ [010]) and $\boldsymbol{B}$ $\parallel$ [010]
$Pc1'$ (7.25) $\mathbb{Z}_1$	• strain $\perp$ [010]<br>• ($\boldsymbol{E}$ $\parallel$ [100] or $\boldsymbol{E}$ $\perp$ [010])
$P2'$ (3.3) $\mathbb{Z}_1$	• $\boldsymbol{B}$ $\parallel$ [100] and strain $\perp$ [001]<br>• $\boldsymbol{B}$ $\parallel$ [010] and strain $\perp$ [001]<br>• $\boldsymbol{B}$ $\perp$ [001]
$P2$ (3.1) $\mathbb{Z}_2$	• $\boldsymbol{B}$ $\parallel$ [001] and strain $\perp$ [001]

Continued on next page

. . . continued

Subgroup & SI group	Perturbation
$P21'$ (3.2) $\mathbb{Z}_2$	• strain $\perp$ [001]
Pm' (6.20) $\mathbb{Z}_1$	• $\boldsymbol{B}$ $\parallel$ [010] and strain $\perp$ [100]<br>• $\boldsymbol{B}$ $\parallel$ [001] and strain $\perp$ [100]<br>• $\boldsymbol{B}$ $\perp$ [100]<br>• ($\boldsymbol{E}$ $\parallel$ [010] or $\boldsymbol{E}$ $\perp$ [100]) and $\boldsymbol{B}$ $\parallel$ [010]<br>• ($\boldsymbol{E}$ $\parallel$ [010] or $\boldsymbol{E}$ $\perp$ [100]) and $\boldsymbol{B}$ $\parallel$ [001]
$Pm'a2'$ (28.89) $\mathbb{Z}_1$	• $\boldsymbol{B}$ $\parallel$ [010]
$Pm'a'2$ (28.91) $\mathbb{Z}_2$	• $\boldsymbol{B}$ $\parallel$ [001]
Pm (6.18) $\mathbb{Z}_1$	• $\boldsymbol{B}$ $\parallel$ [100] and strain $\perp$ [100]<br>• ($\boldsymbol{E}$ $\parallel$ [010] or $\boldsymbol{E}$ $\perp$ [100]) and $\boldsymbol{B}$ $\parallel$ [100]
$Pma'2'$ (28.90) $\mathbb{Z}_1$	• $\boldsymbol{B}$ $\parallel$ [100]
$Pm1'$ (6.19) $\mathbb{Z}_1$	• strain $\perp$ [100]<br>• ($\boldsymbol{E}$ $\parallel$ [010] or $\boldsymbol{E}$ $\perp$ [100])

Table. S 199: Subgroups of $Pm'a2'$ (28.89) that can be realized by applying $\boldsymbol{E}/\boldsymbol{B}$ fields and/or uniaxial strains.

Subgroup & SI group	Perturbation
Pc (7.24) $\mathbb{Z}_1$	• strain $\perp$ [010]<br>• ($\boldsymbol{E}$ $\parallel$ [100] or $\boldsymbol{E}$ $\perp$ [010])
$P2'$ (3.3) $\mathbb{Z}_1$	• strain $\perp$ [001]<br>• ($\boldsymbol{B}$ $\parallel$ [100] or $\boldsymbol{B}$ $\perp$ [001])
Pm' (6.20) $\mathbb{Z}_1$	• strain $\perp$ [100]<br>• ($\boldsymbol{B}$ $\parallel$ [001] or $\boldsymbol{B}$ $\perp$ [100])<br>• ($\boldsymbol{E}$ $\parallel$ [010] or $\boldsymbol{E}$ $\perp$ [100])

Table. S 200: Subgroups of $Pma'2'$ (28.90) that can be realized by applying $\boldsymbol{E}/\boldsymbol{B}$ fields and/or uniaxial strains.

Subgroup & SI group	Perturbation

Continued on next page

...continued

Subgroup & SI group	Perturbation
Pc' (7.26) $\mathbb{Z}_1$	• strain $\perp$ $[010]$<br>• ($\boldsymbol{B}$ $\parallel$ $[001]$ or $\boldsymbol{B}$ $\perp$ $[010]$)<br>• ($\boldsymbol{E}$ $\parallel$ $[100]$ or $\boldsymbol{E}$ $\perp$ $[010]$)
$P2'$ (3.3) $\mathbb{Z}_1$	• strain $\perp$ $[001]$<br>• ($\boldsymbol{B}$ $\parallel$ $[010]$ or $\boldsymbol{B}$ $\perp$ $[001]$)
Pm (6.18) $\mathbb{Z}_1$	• strain $\perp$ $[100]$<br>• ($\boldsymbol{E}$ $\parallel$ $[010]$ or $\boldsymbol{E}$ $\perp$ $[100]$)

Table. S 201: Subgroups of $Pm'a'2$ (28.91) that can be realized by applying $\boldsymbol{E}/\boldsymbol{B}$ fields and/or uniaxial strains.

Subgroup & SI group	Perturbation
Pc' (7.26) $\mathbb{Z}_1$	• strain $\perp$ $[010]$<br>• ($\boldsymbol{B}$ $\parallel$ $[100]$ or $\boldsymbol{B}$ $\perp$ $[010]$)<br>• ($\boldsymbol{E}$ $\parallel$ $[100]$ or $\boldsymbol{E}$ $\perp$ $[010]$)
$P2$ (3.1) $\mathbb{Z}_2$	• strain $\perp$ $[001]$
Pm' (6.20) $\mathbb{Z}_1$	• strain $\perp$ $[100]$<br>• ($\boldsymbol{B}$ $\parallel$ $[010]$ or $\boldsymbol{B}$ $\perp$ $[100]$)<br>• ($\boldsymbol{E}$ $\parallel$ $[010]$ or $\boldsymbol{E}$ $\perp$ $[100]$)

Table. S 202: Subgroups of P_ama2 (28.92) that can be realized by applying $\boldsymbol{E}/\boldsymbol{B}$ fields and/or uniaxial strains.

Subgroup & SI group	Perturbation
Pm' (6.20) $\mathbb{Z}_1$	• $\boldsymbol{B}$ $\parallel$ $[100]$ and strain $\perp$ $[010]$<br>• $\boldsymbol{B}$ $\parallel$ $[001]$ and strain $\perp$ $[010]$<br>• $\boldsymbol{B}$ $\perp$ $[010]$<br>• ($\boldsymbol{E}$ $\parallel$ $[100]$ or $\boldsymbol{E}$ $\perp$ $[010]$) and $\boldsymbol{B}$ $\parallel$ $[100]$<br>• ($\boldsymbol{E}$ $\parallel$ $[100]$ or $\boldsymbol{E}$ $\perp$ $[010]$) and $\boldsymbol{B}$ $\parallel$ $[001]$

Continued on next page

...continued

Subgroup & SI group	Perturbation
P_S1 (1.3) $\mathbb{Z}_1$	• strain in generic direction<br>• ($\boldsymbol{E} \parallel [100]$ or $\boldsymbol{E} \perp [010]$) and strain $\perp [100]$<br>• ($\boldsymbol{E} \parallel [100]$ or $\boldsymbol{E} \perp [010]$) and strain $\perp [001]$<br>• ($\boldsymbol{E} \parallel [010]$ or $\boldsymbol{E} \perp [100]$) and strain $\perp [010]$<br>• ($\boldsymbol{E} \parallel [010]$ or $\boldsymbol{E} \perp [100]$) and strain $\perp [001]$<br>• $\boldsymbol{E}$ in generic direction
Pc (7.24) $\mathbb{Z}_1$	• $\boldsymbol{B} \parallel [010]$ and strain $\perp [010]$<br>• ($\boldsymbol{E} \parallel [100]$ or $\boldsymbol{E} \perp [010]$) and $\boldsymbol{B} \parallel [010]$
P_cc (7.28) $\mathbb{Z}_1$	• strain $\perp [010]$<br>• ($\boldsymbol{E} \parallel [100]$ or $\boldsymbol{E} \perp [010]$)
Pm' (6.20) $\mathbb{Z}_1$	• $\boldsymbol{B} \parallel [010]$ and strain $\perp [100]$<br>• $\boldsymbol{B} \parallel [001]$ and strain $\perp [100]$<br>• $\boldsymbol{B} \perp [100]$<br>• ($\boldsymbol{E} \parallel [010]$ or $\boldsymbol{E} \perp [100]$) and $\boldsymbol{B} \parallel [010]$<br>• ($\boldsymbol{E} \parallel [010]$ or $\boldsymbol{E} \perp [100]$) and $\boldsymbol{B} \parallel [001]$
$P2$ (3.1) $\mathbb{Z}_2$	• $\boldsymbol{B} \parallel [001]$ and strain $\perp [001]$
$Pm'm'2$ (25.60) $\mathbb{Z}_2$	• $\boldsymbol{B} \parallel [001]$
Pm (6.18) $\mathbb{Z}_1$	• $\boldsymbol{B} \parallel [100]$ and strain $\perp [100]$<br>• ($\boldsymbol{E} \parallel [010]$ or $\boldsymbol{E} \perp [100]$) and $\boldsymbol{B} \parallel [100]$
P_bm (6.22) $\mathbb{Z}_1$	• strain $\perp [100]$<br>• ($\boldsymbol{E} \parallel [010]$ or $\boldsymbol{E} \perp [100]$)
$P2'$ (3.3) $\mathbb{Z}_1$	• $\boldsymbol{B} \parallel [100]$ and strain $\perp [001]$<br>• $\boldsymbol{B} \parallel [010]$ and strain $\perp [001]$<br>• $\boldsymbol{B} \perp [001]$
$Pm'a2'$ (28.89) $\mathbb{Z}_1$	• $\boldsymbol{B} \parallel [010]$
P_a2 (3.4) $\mathbb{Z}_2$	• strain $\perp [001]$
$Pm'm2'$ (25.59) $\mathbb{Z}_1$	• $\boldsymbol{B} \parallel [100]$

Table. S 203: Subgroups of P_bma2 (28.93) that can be realized by applying $\boldsymbol{E}/\boldsymbol{B}$ fields and/or uniaxial strains.

Subgroup & SI group	Perturbation

Continued on next page

. . . continued

Subgroup & SI group	Perturbation
P_S1 (1.3) $\mathbb{Z}_1$	• strain in generic direction<br>• ($\boldsymbol{E} \parallel [100]$ or $\boldsymbol{E} \perp [010]$) and strain $\perp [100]$<br>• ($\boldsymbol{E} \parallel [100]$ or $\boldsymbol{E} \perp [010]$) and strain $\perp [001]$<br>• ($\boldsymbol{E} \parallel [010]$ or $\boldsymbol{E} \perp [100]$) and strain $\perp [010]$<br>• ($\boldsymbol{E} \parallel [010]$ or $\boldsymbol{E} \perp [100]$) and strain $\perp [001]$<br>• $\boldsymbol{E}$ in generic direction
Pc (7.24) $\mathbb{Z}_1$	• $\boldsymbol{B} \parallel [010]$ and strain $\perp [010]$<br>• ($\boldsymbol{E} \parallel [100]$ or $\boldsymbol{E} \perp [010]$) and $\boldsymbol{B} \parallel [010]$
Pc' (7.26) $\mathbb{Z}_1$	• $\boldsymbol{B} \parallel [100]$ and strain $\perp [010]$<br>• $\boldsymbol{B} \parallel [001]$ and strain $\perp [010]$<br>• $\boldsymbol{B} \perp [010]$<br>• ($\boldsymbol{E} \parallel [100]$ or $\boldsymbol{E} \perp [010]$) and $\boldsymbol{B} \parallel [100]$<br>• ($\boldsymbol{E} \parallel [100]$ or $\boldsymbol{E} \perp [010]$) and $\boldsymbol{B} \parallel [001]$
P_bc (7.29) $\mathbb{Z}_1$	• strain $\perp [010]$<br>• ($\boldsymbol{E} \parallel [100]$ or $\boldsymbol{E} \perp [010]$)
$P2$ (3.1) $\mathbb{Z}_2$	• $\boldsymbol{B} \parallel [001]$ and strain $\perp [001]$
$P2'$ (3.3) $\mathbb{Z}_1$	• $\boldsymbol{B} \parallel [100]$ and strain $\perp [001]$<br>• $\boldsymbol{B} \parallel [010]$ and strain $\perp [001]$<br>• $\boldsymbol{B} \perp [001]$
P_a2 (3.4) $\mathbb{Z}_2$	• strain $\perp [001]$
Pm (6.18) $\mathbb{Z}_1$	• $\boldsymbol{B} \parallel [100]$ and strain $\perp [100]$<br>• ($\boldsymbol{E} \parallel [010]$ or $\boldsymbol{E} \perp [100]$) and $\boldsymbol{B} \parallel [100]$
$Pma'2'$ (28.90) $\mathbb{Z}_1$	• $\boldsymbol{B} \parallel [100]$
Pc' (7.26) $\mathbb{Z}_1$	• $\boldsymbol{B} \parallel [010]$ and strain $\perp [100]$<br>• $\boldsymbol{B} \parallel [001]$ and strain $\perp [100]$<br>• $\boldsymbol{B} \perp [100]$<br>• ($\boldsymbol{E} \parallel [010]$ or $\boldsymbol{E} \perp [100]$) and $\boldsymbol{B} \parallel [010]$<br>• ($\boldsymbol{E} \parallel [010]$ or $\boldsymbol{E} \perp [100]$) and $\boldsymbol{B} \parallel [001]$
$Pb'a'2$ (32.138) $\mathbb{Z}_2$	• $\boldsymbol{B} \parallel [001]$
$Pb'a2'$ (32.137) $\mathbb{Z}_1$	• $\boldsymbol{B} \parallel [010]$
P_am (6.21) $\mathbb{Z}_1$	• strain $\perp [100]$<br>• ($\boldsymbol{E} \parallel [010]$ or $\boldsymbol{E} \perp [100]$)

Table. S 204: Subgroups of P_cma2 (28.94) that can be realized by applying $\boldsymbol{E}/\boldsymbol{B}$ fields and/or uniaxial strains.

Subgroup & SI group	Perturbation
P_S1 (1.3) $\mathbb{Z}_1$	• strain in generic direction<br>• ($\boldsymbol{E} \parallel [100]$ or $\boldsymbol{E} \perp [010]$) and strain $\perp [100]$<br>• ($\boldsymbol{E} \parallel [100]$ or $\boldsymbol{E} \perp [010]$) and strain $\perp [001]$<br>• ($\boldsymbol{E} \parallel [010]$ or $\boldsymbol{E} \perp [100]$) and strain $\perp [010]$<br>• ($\boldsymbol{E} \parallel [010]$ or $\boldsymbol{E} \perp [100]$) and strain $\perp [001]$<br>• $\boldsymbol{E}$ in generic direction
Pc (7.24) $\mathbb{Z}_1$	• $\boldsymbol{B} \parallel [010]$ and strain $\perp [010]$<br>• ($\boldsymbol{E} \parallel [100]$ or $\boldsymbol{E} \perp [010]$) and $\boldsymbol{B} \parallel [010]$
Pc' (7.26) $\mathbb{Z}_1$	• $\boldsymbol{B} \parallel [100]$ and strain $\perp [010]$<br>• $\boldsymbol{B} \parallel [001]$ and strain $\perp [010]$<br>• $\boldsymbol{B} \perp [010]$<br>• ($\boldsymbol{E} \parallel [100]$ or $\boldsymbol{E} \perp [010]$) and $\boldsymbol{B} \parallel [100]$<br>• ($\boldsymbol{E} \parallel [100]$ or $\boldsymbol{E} \perp [010]$) and $\boldsymbol{B} \parallel [001]$
P_ac (7.27) $\mathbb{Z}_1$	• strain $\perp [010]$<br>• ($\boldsymbol{E} \parallel [100]$ or $\boldsymbol{E} \perp [010]$)
$P2$ (3.1) $\mathbb{Z}_2$	• $\boldsymbol{B} \parallel [001]$ and strain $\perp [001]$
$P2_1'$ (4.9) $\mathbb{Z}_1$	• $\boldsymbol{B} \parallel [100]$ and strain $\perp [001]$<br>• $\boldsymbol{B} \parallel [010]$ and strain $\perp [001]$<br>• $\boldsymbol{B} \perp [001]$
P_b2 (3.5) $\mathbb{Z}_2$	• strain $\perp [001]$
Pm (6.18) $\mathbb{Z}_1$	• $\boldsymbol{B} \parallel [100]$ and strain $\perp [100]$<br>• ($\boldsymbol{E} \parallel [010]$ or $\boldsymbol{E} \perp [100]$) and $\boldsymbol{B} \parallel [100]$
$Pmn'2_1'$ (31.126) $\mathbb{Z}_1$	• $\boldsymbol{B} \parallel [100]$
Pc' (7.26) $\mathbb{Z}_1$	• $\boldsymbol{B} \parallel [010]$ and strain $\perp [100]$<br>• $\boldsymbol{B} \parallel [001]$ and strain $\perp [100]$<br>• $\boldsymbol{B} \perp [100]$<br>• ($\boldsymbol{E} \parallel [010]$ or $\boldsymbol{E} \perp [100]$) and $\boldsymbol{B} \parallel [010]$<br>• ($\boldsymbol{E} \parallel [010]$ or $\boldsymbol{E} \perp [100]$) and $\boldsymbol{B} \parallel [001]$
$Pn'c'2$ (30.115) $\mathbb{Z}_1$	• $\boldsymbol{B} \parallel [001]$
$Pc'a2_1'$ (29.101) $\mathbb{Z}_1$	• $\boldsymbol{B} \parallel [010]$
P_am (6.21) $\mathbb{Z}_1$	• strain $\perp [100]$<br>• ($\boldsymbol{E} \parallel [010]$ or $\boldsymbol{E} \perp [100]$)

Table. S 205: Subgroups of $P_{A}ma2$ (28.95) that can be realized by applying $\boldsymbol{E}/\boldsymbol{B}$ fields and/or uniaxial strains.

Subgroup & SI group	Perturbation
P_S1 (1.3) $\mathbb{Z}_1$	• strain in generic direction<br>• ($\boldsymbol{E} \parallel [100]$ or $\boldsymbol{E} \perp [010]$) and strain $\perp [100]$<br>• ($\boldsymbol{E} \parallel [100]$ or $\boldsymbol{E} \perp [010]$) and strain $\perp [001]$<br>• ($\boldsymbol{E} \parallel [010]$ or $\boldsymbol{E} \perp [100]$) and strain $\perp [010]$<br>• ($\boldsymbol{E} \parallel [010]$ or $\boldsymbol{E} \perp [100]$) and strain $\perp [001]$<br>• $\boldsymbol{E}$ in generic direction
Pc (7.24) $\mathbb{Z}_1$	• $\boldsymbol{B} \parallel [010]$ and strain $\perp [010]$<br>• ($\boldsymbol{E} \parallel [100]$ or $\boldsymbol{E} \perp [010]$) and $\boldsymbol{B} \parallel [010]$
Pc' (7.26) $\mathbb{Z}_1$	• $\boldsymbol{B} \parallel [100]$ and strain $\perp [010]$<br>• $\boldsymbol{B} \parallel [001]$ and strain $\perp [010]$<br>• $\boldsymbol{B} \perp [010]$<br>• ($\boldsymbol{E} \parallel [100]$ or $\boldsymbol{E} \perp [010]$) and $\boldsymbol{B} \parallel [100]$<br>• ($\boldsymbol{E} \parallel [100]$ or $\boldsymbol{E} \perp [010]$) and $\boldsymbol{B} \parallel [001]$
P_Cc (7.30) $\mathbb{Z}_1$	• strain $\perp [010]$<br>• ($\boldsymbol{E} \parallel [100]$ or $\boldsymbol{E} \perp [010]$)
$P2$ (3.1) $\mathbb{Z}_2$	• $\boldsymbol{B} \parallel [001]$ and strain $\perp [001]$
$P2_1'$ (4.9) $\mathbb{Z}_1$	• $\boldsymbol{B} \parallel [100]$ and strain $\perp [001]$<br>• $\boldsymbol{B} \parallel [010]$ and strain $\perp [001]$<br>• $\boldsymbol{B} \perp [001]$
P_C2 (3.6) $\mathbb{Z}_1$	• strain $\perp [001]$
Pm (6.18) $\mathbb{Z}_1$	• $\boldsymbol{B} \parallel [100]$ and strain $\perp [100]$<br>• ($\boldsymbol{E} \parallel [010]$ or $\boldsymbol{E} \perp [100]$) and $\boldsymbol{B} \parallel [100]$
$Pmn'2_1'$ (31.126) $\mathbb{Z}_1$	• $\boldsymbol{B} \parallel [100]$
Pc' (7.26) $\mathbb{Z}_1$	• $\boldsymbol{B} \parallel [010]$ and strain $\perp [100]$<br>• $\boldsymbol{B} \parallel [001]$ and strain $\perp [100]$<br>• $\boldsymbol{B} \perp [100]$<br>• ($\boldsymbol{E} \parallel [010]$ or $\boldsymbol{E} \perp [100]$) and $\boldsymbol{B} \parallel [010]$<br>• ($\boldsymbol{E} \parallel [010]$ or $\boldsymbol{E} \perp [100]$) and $\boldsymbol{B} \parallel [001]$
$Pn'n'2$ (34.159) $\mathbb{Z}_1$	• $\boldsymbol{B} \parallel [001]$
$Pn'a2_1'$ (33.146) $\mathbb{Z}_1$	• $\boldsymbol{B} \parallel [010]$
P_am (6.21) $\mathbb{Z}_1$	• strain $\perp [100]$<br>• ($\boldsymbol{E} \parallel [010]$ or $\boldsymbol{E} \perp [100]$)

Table. S 206: Subgroups of P_Bma2 (28.96) that can be realized by applying $\boldsymbol{E}/\boldsymbol{B}$ fields and/or uniaxial strains.

Subgroup & SI group	Perturbation
Pc' (7.26) $\mathbb{Z}_1$	• $\boldsymbol{B} \parallel [100]$ and strain $\perp [010]$<br>• $\boldsymbol{B} \parallel [001]$ and strain $\perp [010]$<br>• $\boldsymbol{B} \perp [010]$<br>• ($\boldsymbol{E} \parallel [100]$ or $\boldsymbol{E} \perp [010]$) and $\boldsymbol{B} \parallel [100]$<br>• ($\boldsymbol{E} \parallel [100]$ or $\boldsymbol{E} \perp [010]$) and $\boldsymbol{B} \parallel [001]$
P_S1 (1.3) $\mathbb{Z}_1$	• strain in generic direction<br>• ($\boldsymbol{E} \parallel [100]$ or $\boldsymbol{E} \perp [010]$) and strain $\perp [100]$<br>• ($\boldsymbol{E} \parallel [100]$ or $\boldsymbol{E} \perp [010]$) and strain $\perp [001]$<br>• ($\boldsymbol{E} \parallel [010]$ or $\boldsymbol{E} \perp [100]$) and strain $\perp [010]$<br>• ($\boldsymbol{E} \parallel [010]$ or $\boldsymbol{E} \perp [100]$) and strain $\perp [001]$<br>• $\boldsymbol{E}$ in generic direction
Pc (7.24) $\mathbb{Z}_1$	• $\boldsymbol{B} \parallel [010]$ and strain $\perp [010]$<br>• ($\boldsymbol{E} \parallel [100]$ or $\boldsymbol{E} \perp [010]$) and $\boldsymbol{B} \parallel [010]$
P_ac (7.27) $\mathbb{Z}_1$	• strain $\perp [010]$<br>• ($\boldsymbol{E} \parallel [100]$ or $\boldsymbol{E} \perp [010]$)
Pc' (7.26) $\mathbb{Z}_1$	• $\boldsymbol{B} \parallel [010]$ and strain $\perp [100]$<br>• $\boldsymbol{B} \parallel [001]$ and strain $\perp [100]$<br>• $\boldsymbol{B} \perp [100]$<br>• ($\boldsymbol{E} \parallel [010]$ or $\boldsymbol{E} \perp [100]$) and $\boldsymbol{B} \parallel [010]$<br>• ($\boldsymbol{E} \parallel [010]$ or $\boldsymbol{E} \perp [100]$) and $\boldsymbol{B} \parallel [001]$
$P2$ (3.1) $\mathbb{Z}_2$	• $\boldsymbol{B} \parallel [001]$ and strain $\perp [001]$
$Pc'c'2$ (27.81) $\mathbb{Z}_2$	• $\boldsymbol{B} \parallel [001]$
Pm (6.18) $\mathbb{Z}_1$	• $\boldsymbol{B} \parallel [100]$ and strain $\perp [100]$<br>• ($\boldsymbol{E} \parallel [010]$ or $\boldsymbol{E} \perp [100]$) and $\boldsymbol{B} \parallel [100]$
P_Cm (6.23) $\mathbb{Z}_1$	• strain $\perp [100]$<br>• ($\boldsymbol{E} \parallel [010]$ or $\boldsymbol{E} \perp [100]$)
$P2_1'$ (4.9) $\mathbb{Z}_1$	• $\boldsymbol{B} \parallel [100]$ and strain $\perp [001]$<br>• $\boldsymbol{B} \parallel [010]$ and strain $\perp [001]$<br>• $\boldsymbol{B} \perp [001]$
$Pc'a2_1'$ (29.101) $\mathbb{Z}_1$	• $\boldsymbol{B} \parallel [010]$
P_C2 (3.6) $\mathbb{Z}_1$	• strain $\perp [001]$
$Pmc'2_1'$ (26.69) $\mathbb{Z}_1$	• $\boldsymbol{B} \parallel [100]$

Table. S 207: Subgroups of $P_{C}ma2$ (28.97) that can be realized by applying $\boldsymbol{E}/\boldsymbol{B}$ fields and/or uniaxial strains.

Subgroup & SI group	Perturbation
Pm' (6.20) $\mathbb{Z}_1$	• $\boldsymbol{B} \parallel [100]$ and strain $\perp [010]$<br>• $\boldsymbol{B} \parallel [001]$ and strain $\perp [010]$<br>• $\boldsymbol{B} \perp [010]$<br>• ($\boldsymbol{E} \parallel [100]$ or $\boldsymbol{E} \perp [010]$) and $\boldsymbol{B} \parallel [100]$<br>• ($\boldsymbol{E} \parallel [100]$ or $\boldsymbol{E} \perp [010]$) and $\boldsymbol{B} \parallel [001]$
P_S1 (1.3) $\mathbb{Z}_1$	• strain in generic direction<br>• ($\boldsymbol{E} \parallel [100]$ or $\boldsymbol{E} \perp [010]$) and strain $\perp [100]$<br>• ($\boldsymbol{E} \parallel [100]$ or $\boldsymbol{E} \perp [010]$) and strain $\perp [001]$<br>• ($\boldsymbol{E} \parallel [010]$ or $\boldsymbol{E} \perp [100]$) and strain $\perp [010]$<br>• ($\boldsymbol{E} \parallel [010]$ or $\boldsymbol{E} \perp [100]$) and strain $\perp [001]$<br>• $\boldsymbol{E}$ in generic direction
Pc (7.24) $\mathbb{Z}_1$	• $\boldsymbol{B} \parallel [010]$ and strain $\perp [010]$<br>• ($\boldsymbol{E} \parallel [100]$ or $\boldsymbol{E} \perp [010]$) and $\boldsymbol{B} \parallel [010]$
P_Ac (7.31) $\mathbb{Z}_1$	• strain $\perp [010]$<br>• ($\boldsymbol{E} \parallel [100]$ or $\boldsymbol{E} \perp [010]$)
Pc' (7.26) $\mathbb{Z}_1$	• $\boldsymbol{B} \parallel [010]$ and strain $\perp [100]$<br>• $\boldsymbol{B} \parallel [001]$ and strain $\perp [100]$<br>• $\boldsymbol{B} \perp [100]$<br>• ($\boldsymbol{E} \parallel [010]$ or $\boldsymbol{E} \perp [100]$) and $\boldsymbol{B} \parallel [010]$<br>• ($\boldsymbol{E} \parallel [010]$ or $\boldsymbol{E} \perp [100]$) and $\boldsymbol{B} \parallel [001]$
$P2$ (3.1) $\mathbb{Z}_2$	• $\boldsymbol{B} \parallel [001]$ and strain $\perp [001]$
$Pm'a'2$ (28.91) $\mathbb{Z}_2$	• $\boldsymbol{B} \parallel [001]$
Pm (6.18) $\mathbb{Z}_1$	• $\boldsymbol{B} \parallel [100]$ and strain $\perp [100]$<br>• ($\boldsymbol{E} \parallel [010]$ or $\boldsymbol{E} \perp [100]$) and $\boldsymbol{B} \parallel [100]$
P_Cm (6.23) $\mathbb{Z}_1$	• strain $\perp [100]$<br>• ($\boldsymbol{E} \parallel [010]$ or $\boldsymbol{E} \perp [100]$)
$P2'$ (3.3) $\mathbb{Z}_1$	• $\boldsymbol{B} \parallel [100]$ and strain $\perp [001]$<br>• $\boldsymbol{B} \parallel [010]$ and strain $\perp [001]$<br>• $\boldsymbol{B} \perp [001]$
$Pb'a2'$ (32.137) $\mathbb{Z}_1$	• $\boldsymbol{B} \parallel [010]$
P_a2 (3.4) $\mathbb{Z}_2$	• strain $\perp [001]$
$Pm'm2'$ (25.59) $\mathbb{Z}_1$	• $\boldsymbol{B} \parallel [100]$

Table. S 208: Subgroups of P_Ima2 (28.98) that can be realized by applying $\boldsymbol{E}/\boldsymbol{B}$ fields and/or uniaxial strains.

Subgroup & SI group	Perturbation
Pc' (7.26) $\mathbb{Z}_1$	• $\boldsymbol{B} \parallel [100]$ and strain $\perp [010]$<br>• $\boldsymbol{B} \parallel [001]$ and strain $\perp [010]$<br>• $\boldsymbol{B} \perp [010]$<br>• ($\boldsymbol{E} \parallel [100]$ or $\boldsymbol{E} \perp [010]$) and $\boldsymbol{B} \parallel [100]$<br>• ($\boldsymbol{E} \parallel [100]$ or $\boldsymbol{E} \perp [010]$) and $\boldsymbol{B} \parallel [001]$
P_S1 (1.3) $\mathbb{Z}_1$	• strain in generic direction<br>• ($\boldsymbol{E} \parallel [100]$ or $\boldsymbol{E} \perp [010]$) and strain $\perp [100]$<br>• ($\boldsymbol{E} \parallel [100]$ or $\boldsymbol{E} \perp [010]$) and strain $\perp [001]$<br>• ($\boldsymbol{E} \parallel [010]$ or $\boldsymbol{E} \perp [100]$) and strain $\perp [010]$<br>• ($\boldsymbol{E} \parallel [010]$ or $\boldsymbol{E} \perp [100]$) and strain $\perp [001]$<br>• $\boldsymbol{E}$ in generic direction
Pc (7.24) $\mathbb{Z}_1$	• $\boldsymbol{B} \parallel [010]$ and strain $\perp [010]$<br>• ($\boldsymbol{E} \parallel [100]$ or $\boldsymbol{E} \perp [010]$) and $\boldsymbol{B} \parallel [010]$
P_Cc (7.30) $\mathbb{Z}_1$	• strain $\perp [010]$<br>• ($\boldsymbol{E} \parallel [100]$ or $\boldsymbol{E} \perp [010]$)
Pc' (7.26) $\mathbb{Z}_1$	• $\boldsymbol{B} \parallel [010]$ and strain $\perp [100]$<br>• $\boldsymbol{B} \parallel [001]$ and strain $\perp [100]$<br>• $\boldsymbol{B} \perp [100]$<br>• ($\boldsymbol{E} \parallel [010]$ or $\boldsymbol{E} \perp [100]$) and $\boldsymbol{B} \parallel [010]$<br>• ($\boldsymbol{E} \parallel [010]$ or $\boldsymbol{E} \perp [100]$) and $\boldsymbol{B} \parallel [001]$
$P2$ (3.1) $\mathbb{Z}_2$	• $\boldsymbol{B} \parallel [001]$ and strain $\perp [001]$
$Pn'c'2$ (30.115) $\mathbb{Z}_1$	• $\boldsymbol{B} \parallel [001]$
Pm (6.18) $\mathbb{Z}_1$	• $\boldsymbol{B} \parallel [100]$ and strain $\perp [100]$<br>• ($\boldsymbol{E} \parallel [010]$ or $\boldsymbol{E} \perp [100]$) and $\boldsymbol{B} \parallel [100]$
P_Cm (6.23) $\mathbb{Z}_1$	• strain $\perp [100]$<br>• ($\boldsymbol{E} \parallel [010]$ or $\boldsymbol{E} \perp [100]$)
$P2_1'$ (4.9) $\mathbb{Z}_1$	• $\boldsymbol{B} \parallel [100]$ and strain $\perp [001]$<br>• $\boldsymbol{B} \parallel [010]$ and strain $\perp [001]$<br>• $\boldsymbol{B} \perp [001]$
$Pn'a2_1'$ (33.146) $\mathbb{Z}_1$	• $\boldsymbol{B} \parallel [010]$
P_C2 (3.6) $\mathbb{Z}_1$	• strain $\perp [001]$
$Pmc'2_1'$ (26.69) $\mathbb{Z}_1$	• $\boldsymbol{B} \parallel [100]$

Table. S 209: Subgroups of $Pca2_11'$ (29.100) that can be realized by applying $\boldsymbol{E}/\boldsymbol{B}$ fields and/or uniaxial strains.

Subgroup & SI group	Perturbation
$P11'$ (1.2) $\mathbb{Z}_1$	• strain in generic direction<br>• ($\boldsymbol{E} \parallel [100]$ or $\boldsymbol{E} \perp [010]$) and strain $\perp [100]$<br>• ($\boldsymbol{E} \parallel [100]$ or $\boldsymbol{E} \perp [010]$) and strain $\perp [001]$<br>• ($\boldsymbol{E} \parallel [010]$ or $\boldsymbol{E} \perp [100]$) and strain $\perp [010]$<br>• ($\boldsymbol{E} \parallel [010]$ or $\boldsymbol{E} \perp [100]$) and strain $\perp [001]$<br>• $\boldsymbol{E}$ in generic direction
Pc' (7.26) $\mathbb{Z}_1$	• $\boldsymbol{B} \parallel [100]$ and strain $\perp [010]$<br>• $\boldsymbol{B} \parallel [001]$ and strain $\perp [010]$<br>• $\boldsymbol{B} \perp [010]$<br>• ($\boldsymbol{E} \parallel [100]$ or $\boldsymbol{E} \perp [010]$) and $\boldsymbol{B} \parallel [100]$<br>• ($\boldsymbol{E} \parallel [100]$ or $\boldsymbol{E} \perp [010]$) and $\boldsymbol{B} \parallel [001]$
Pc (7.24) $\mathbb{Z}_1$	• $\boldsymbol{B} \parallel [010]$ and strain $\perp [010]$<br>• ($\boldsymbol{E} \parallel [100]$ or $\boldsymbol{E} \perp [010]$) and $\boldsymbol{B} \parallel [010]$
$Pc1'$ (7.25) $\mathbb{Z}_1$	• strain $\perp [010]$<br>• ($\boldsymbol{E} \parallel [100]$ or $\boldsymbol{E} \perp [010]$)
$P2_1'$ (4.9) $\mathbb{Z}_1$	• $\boldsymbol{B} \parallel [100]$ and strain $\perp [001]$<br>• $\boldsymbol{B} \parallel [010]$ and strain $\perp [001]$<br>• $\boldsymbol{B} \perp [001]$
$P2_1$ (4.7) $\mathbb{Z}_1$	• $\boldsymbol{B} \parallel [001]$ and strain $\perp [001]$
$P2_11'$ (4.8) $\mathbb{Z}_1$	• strain $\perp [001]$
Pc' (7.26) $\mathbb{Z}_1$	• $\boldsymbol{B} \parallel [010]$ and strain $\perp [100]$<br>• $\boldsymbol{B} \parallel [001]$ and strain $\perp [100]$<br>• $\boldsymbol{B} \perp [100]$<br>• ($\boldsymbol{E} \parallel [010]$ or $\boldsymbol{E} \perp [100]$) and $\boldsymbol{B} \parallel [010]$<br>• ($\boldsymbol{E} \parallel [010]$ or $\boldsymbol{E} \perp [100]$) and $\boldsymbol{B} \parallel [001]$
$Pc'a2_1'$ (29.101) $\mathbb{Z}_1$	• $\boldsymbol{B} \parallel [010]$
$Pc'a'2_1$ (29.103) $\mathbb{Z}_1$	• $\boldsymbol{B} \parallel [001]$
Pc (7.24) $\mathbb{Z}_1$	• $\boldsymbol{B} \parallel [100]$ and strain $\perp [100]$<br>• ($\boldsymbol{E} \parallel [010]$ or $\boldsymbol{E} \perp [100]$) and $\boldsymbol{B} \parallel [100]$
$Pca'2_1'$ (29.102) $\mathbb{Z}_1$	• $\boldsymbol{B} \parallel [100]$
$Pc1'$ (7.25) $\mathbb{Z}_1$	• strain $\perp [100]$<br>• ($\boldsymbol{E} \parallel [010]$ or $\boldsymbol{E} \perp [100]$)

Table. S 210: Subgroups of $Pc'a2_1'$ (29.101) that can be realized by applying $\boldsymbol{E}/\boldsymbol{B}$ fields and/or uniaxial strains.

Subgroup & SI group	Perturbation
Pc (7.24) $\mathbb{Z}_1$	• strain $\perp$ [010]<br>• ($\boldsymbol{E} \parallel$ [100] or $\boldsymbol{E} \perp$ [010])
$P2_1'$ (4.9) $\mathbb{Z}_1$	• strain $\perp$ [001]<br>• ($\boldsymbol{B} \parallel$ [100] or $\boldsymbol{B} \perp$ [001])
Pc' (7.26) $\mathbb{Z}_1$	• strain $\perp$ [100]<br>• ($\boldsymbol{B} \parallel$ [001] or $\boldsymbol{B} \perp$ [100])<br>• ($\boldsymbol{E} \parallel$ [010] or $\boldsymbol{E} \perp$ [100])

Table. S 211: Subgroups of $Pca'2_1'$ (29.102) that can be realized by applying $\boldsymbol{E}/\boldsymbol{B}$ fields and/or uniaxial strains.

Subgroup & SI group	Perturbation
Pc' (7.26) $\mathbb{Z}_1$	• strain $\perp$ [010]<br>• ($\boldsymbol{B} \parallel$ [001] or $\boldsymbol{B} \perp$ [010])<br>• ($\boldsymbol{E} \parallel$ [100] or $\boldsymbol{E} \perp$ [010])
$P2_1'$ (4.9) $\mathbb{Z}_1$	• strain $\perp$ [001]<br>• ($\boldsymbol{B} \parallel$ [010] or $\boldsymbol{B} \perp$ [001])
Pc (7.24) $\mathbb{Z}_1$	• strain $\perp$ [100]<br>• ($\boldsymbol{E} \parallel$ [010] or $\boldsymbol{E} \perp$ [100])

Table. S 212: Subgroups of $Pc'a'2_1$ (29.103) that can be realized by applying $\boldsymbol{E}/\boldsymbol{B}$ fields and/or uniaxial strains.

Subgroup & SI group	Perturbation
Pc' (7.26) $\mathbb{Z}_1$	• strain $\perp$ [010]<br>• ($\boldsymbol{B} \parallel$ [100] or $\boldsymbol{B} \perp$ [010])<br>• ($\boldsymbol{E} \parallel$ [100] or $\boldsymbol{E} \perp$ [010])
$P2_1$ (4.7) $\mathbb{Z}_1$	• strain $\perp$ [001]

Continued on next page

...continued

Subgroup & SI group	Perturbation
Pc' (7.26) $\mathbb{Z}_1$	• strain $\perp$ [100]<br>• ($\boldsymbol{B} \parallel$ [010] or $\boldsymbol{B} \perp$ [100])<br>• ($\boldsymbol{E} \parallel$ [010] or $\boldsymbol{E} \perp$ [100])

Table. S 213: Subgroups of P_aca2_1 (29.104) that can be realized by applying $\boldsymbol{E}/\boldsymbol{B}$ fields and/or uniaxial strains.

Subgroup & SI group	Perturbation
Pm' (6.20) $\mathbb{Z}_1$	• $\boldsymbol{B} \parallel$ [100] and strain $\perp$ [010]<br>• $\boldsymbol{B} \parallel$ [001] and strain $\perp$ [010]<br>• $\boldsymbol{B} \perp$ [010]<br>• ($\boldsymbol{E} \parallel$ [100] or $\boldsymbol{E} \perp$ [010]) and $\boldsymbol{B} \parallel$ [100]<br>• ($\boldsymbol{E} \parallel$ [100] or $\boldsymbol{E} \perp$ [010]) and $\boldsymbol{B} \parallel$ [001]
P_S1 (1.3) $\mathbb{Z}_1$	• strain in generic direction<br>• ($\boldsymbol{E} \parallel$ [100] or $\boldsymbol{E} \perp$ [010]) and strain $\perp$ [100]<br>• ($\boldsymbol{E} \parallel$ [100] or $\boldsymbol{E} \perp$ [010]) and strain $\perp$ [001]<br>• ($\boldsymbol{E} \parallel$ [010] or $\boldsymbol{E} \perp$ [100]) and strain $\perp$ [010]<br>• ($\boldsymbol{E} \parallel$ [010] or $\boldsymbol{E} \perp$ [100]) and strain $\perp$ [001]<br>• $\boldsymbol{E}$ in generic direction
Pc (7.24) $\mathbb{Z}_1$	• $\boldsymbol{B} \parallel$ [010] and strain $\perp$ [010]<br>• ($\boldsymbol{E} \parallel$ [100] or $\boldsymbol{E} \perp$ [010]) and $\boldsymbol{B} \parallel$ [010]
P_cc (7.28) $\mathbb{Z}_1$	• strain $\perp$ [010]<br>• ($\boldsymbol{E} \parallel$ [100] or $\boldsymbol{E} \perp$ [010])
Pc' (7.26) $\mathbb{Z}_1$	• $\boldsymbol{B} \parallel$ [010] and strain $\perp$ [100]<br>• $\boldsymbol{B} \parallel$ [001] and strain $\perp$ [100]<br>• $\boldsymbol{B} \perp$ [100]<br>• ($\boldsymbol{E} \parallel$ [010] or $\boldsymbol{E} \perp$ [100]) and $\boldsymbol{B} \parallel$ [010]<br>• ($\boldsymbol{E} \parallel$ [010] or $\boldsymbol{E} \perp$ [100]) and $\boldsymbol{B} \parallel$ [001]
$P2_1$ (4.7) $\mathbb{Z}_1$	• $\boldsymbol{B} \parallel$ [001] and strain $\perp$ [001]
$Pm'c'2_1$ (26.70) $\mathbb{Z}_1$	• $\boldsymbol{B} \parallel$ [001]
Pc (7.24) $\mathbb{Z}_1$	• $\boldsymbol{B} \parallel$ [100] and strain $\perp$ [100]<br>• ($\boldsymbol{E} \parallel$ [010] or $\boldsymbol{E} \perp$ [100]) and $\boldsymbol{B} \parallel$ [100]
P_bc (7.29) $\mathbb{Z}_1$	• strain $\perp$ [100]<br>• ($\boldsymbol{E} \parallel$ [010] or $\boldsymbol{E} \perp$ [100])

Continued on next page

. . . continued

Subgroup & SI group	Perturbation
$P2_1'$ (4.9) $\mathbb{Z}_1$	• $\boldsymbol{B} \parallel [100]$ and strain $\perp [001]$<br>• $\boldsymbol{B} \parallel [010]$ and strain $\perp [001]$<br>• $\boldsymbol{B} \perp [001]$
$Pc'a2_1'$ (29.101) $\mathbb{Z}_1$	• $\boldsymbol{B} \parallel [010]$
P_a2_1 (4.10) $\mathbb{Z}_1$	• strain $\perp [001]$
$Pm'c2_1'$ (26.68) $\mathbb{Z}_1$	• $\boldsymbol{B} \parallel [100]$

Table. S 214: Subgroups of P_bca2_1 (29.105) that can be realized by applying $\boldsymbol{E}/\boldsymbol{B}$ fields and/or uniaxial strains.

Subgroup & SI group	Perturbation
P_S1 (1.3) $\mathbb{Z}_1$	• strain in generic direction<br>• ($\boldsymbol{E} \parallel [100]$ or $\boldsymbol{E} \perp [010]$) and strain $\perp [100]$<br>• ($\boldsymbol{E} \parallel [100]$ or $\boldsymbol{E} \perp [010]$) and strain $\perp [001]$<br>• ($\boldsymbol{E} \parallel [010]$ or $\boldsymbol{E} \perp [100]$) and strain $\perp [010]$<br>• ($\boldsymbol{E} \parallel [010]$ or $\boldsymbol{E} \perp [100]$) and strain $\perp [001]$<br>• $\boldsymbol{E}$ in generic direction
Pc (7.24) $\mathbb{Z}_1$	• $\boldsymbol{B} \parallel [010]$ and strain $\perp [010]$<br>• ($\boldsymbol{E} \parallel [100]$ or $\boldsymbol{E} \perp [010]$) and $\boldsymbol{B} \parallel [010]$
Pc' (7.26) $\mathbb{Z}_1$	• $\boldsymbol{B} \parallel [100]$ and strain $\perp [010]$<br>• $\boldsymbol{B} \parallel [001]$ and strain $\perp [010]$<br>• $\boldsymbol{B} \perp [010]$<br>• ($\boldsymbol{E} \parallel [100]$ or $\boldsymbol{E} \perp [010]$) and $\boldsymbol{B} \parallel [100]$<br>• ($\boldsymbol{E} \parallel [100]$ or $\boldsymbol{E} \perp [010]$) and $\boldsymbol{B} \parallel [001]$
P_bc (7.29) $\mathbb{Z}_1$	• strain $\perp [010]$<br>• ($\boldsymbol{E} \parallel [100]$ or $\boldsymbol{E} \perp [010]$)
$P2_1$ (4.7) $\mathbb{Z}_1$	• $\boldsymbol{B} \parallel [001]$ and strain $\perp [001]$
$P2_1'$ (4.9) $\mathbb{Z}_1$	• $\boldsymbol{B} \parallel [100]$ and strain $\perp [001]$<br>• $\boldsymbol{B} \parallel [010]$ and strain $\perp [001]$<br>• $\boldsymbol{B} \perp [001]$
P_a2_1 (4.10) $\mathbb{Z}_1$	• strain $\perp [001]$
Pc (7.24) $\mathbb{Z}_1$	• $\boldsymbol{B} \parallel [100]$ and strain $\perp [100]$<br>• ($\boldsymbol{E} \parallel [010]$ or $\boldsymbol{E} \perp [100]$) and $\boldsymbol{B} \parallel [100]$

Continued on next page

. . . continued

Subgroup & SI group	Perturbation
$Pca'2_1'$ (29.102) $\mathbb{Z}_1$	• $\boldsymbol{B} \parallel [100]$
Pc' (7.26) $\mathbb{Z}_1$	• $\boldsymbol{B} \parallel [010]$ and strain $\perp [100]$<br>• $\boldsymbol{B} \parallel [001]$ and strain $\perp [100]$<br>• $\boldsymbol{B} \perp [100]$<br>• ($\boldsymbol{E} \parallel [010]$ or $\boldsymbol{E} \perp [100]$) and $\boldsymbol{B} \parallel [010]$<br>• ($\boldsymbol{E} \parallel [010]$ or $\boldsymbol{E} \perp [100]$) and $\boldsymbol{B} \parallel [001]$
$Pn'a'2_1$ (33.148) $\mathbb{Z}_1$	• $\boldsymbol{B} \parallel [001]$
$Pn'a2_1'$ (33.146) $\mathbb{Z}_1$	• $\boldsymbol{B} \parallel [010]$
P_ac (7.27) $\mathbb{Z}_1$	• strain $\perp [100]$<br>• ($\boldsymbol{E} \parallel [010]$ or $\boldsymbol{E} \perp [100]$)

Table. S 215: Subgroups of P_cca2_1 (29.106) that can be realized by applying $\boldsymbol{E}/\boldsymbol{B}$ fields and/or uniaxial strains.

Subgroup & SI group	Perturbation
P_S1 (1.3) $\mathbb{Z}_1$	• strain in generic direction<br>• ($\boldsymbol{E} \parallel [100]$ or $\boldsymbol{E} \perp [010]$) and strain $\perp [100]$<br>• ($\boldsymbol{E} \parallel [100]$ or $\boldsymbol{E} \perp [010]$) and strain $\perp [001]$<br>• ($\boldsymbol{E} \parallel [010]$ or $\boldsymbol{E} \perp [100]$) and strain $\perp [010]$<br>• ($\boldsymbol{E} \parallel [010]$ or $\boldsymbol{E} \perp [100]$) and strain $\perp [001]$<br>• $\boldsymbol{E}$ in generic direction
Pc (7.24) $\mathbb{Z}_1$	• $\boldsymbol{B} \parallel [010]$ and strain $\perp [010]$<br>• ($\boldsymbol{E} \parallel [100]$ or $\boldsymbol{E} \perp [010]$) and $\boldsymbol{B} \parallel [010]$
Pc' (7.26) $\mathbb{Z}_1$	• $\boldsymbol{B} \parallel [100]$ and strain $\perp [010]$<br>• $\boldsymbol{B} \parallel [001]$ and strain $\perp [010]$<br>• $\boldsymbol{B} \perp [010]$<br>• ($\boldsymbol{E} \parallel [100]$ or $\boldsymbol{E} \perp [010]$) and $\boldsymbol{B} \parallel [100]$<br>• ($\boldsymbol{E} \parallel [100]$ or $\boldsymbol{E} \perp [010]$) and $\boldsymbol{B} \parallel [001]$
P_ac (7.27) $\mathbb{Z}_1$	• strain $\perp [010]$<br>• ($\boldsymbol{E} \parallel [100]$ or $\boldsymbol{E} \perp [010]$)
$P2'$ (3.3) $\mathbb{Z}_1$	• $\boldsymbol{B} \parallel [100]$ and strain $\perp [001]$<br>• $\boldsymbol{B} \parallel [010]$ and strain $\perp [001]$<br>• $\boldsymbol{B} \perp [001]$
$P2_1$ (4.7) $\mathbb{Z}_1$	• $\boldsymbol{B} \parallel [001]$ and strain $\perp [001]$

Continued on next page

...continued

Subgroup & SI group	Perturbation
P_b2_1 (4.11) $\mathbb{Z}_1$	• strain $\perp$ [001]
Pm' (6.20) $\mathbb{Z}_1$	• $\boldsymbol{B}$ $\parallel$ [010] and strain $\perp$ [100]<br>• $\boldsymbol{B}$ $\parallel$ [001] and strain $\perp$ [100]<br>• $\boldsymbol{B}$ $\perp$ [100]<br>• ($\boldsymbol{E}$ $\parallel$ [010] or $\boldsymbol{E}$ $\perp$ [100]) and $\boldsymbol{B}$ $\parallel$ [010]<br>• ($\boldsymbol{E}$ $\parallel$ [010] or $\boldsymbol{E}$ $\perp$ [100]) and $\boldsymbol{B}$ $\parallel$ [001]
$Pm'a2'$ (28.89) $\mathbb{Z}_1$	• $\boldsymbol{B}$ $\parallel$ [010]
$Pm'n'2_1$ (31.127) $\mathbb{Z}_1$	• $\boldsymbol{B}$ $\parallel$ [001]
Pc (7.24) $\mathbb{Z}_1$	• $\boldsymbol{B}$ $\parallel$ [100] and strain $\perp$ [100]<br>• ($\boldsymbol{E}$ $\parallel$ [010] or $\boldsymbol{E}$ $\perp$ [100]) and $\boldsymbol{B}$ $\parallel$ [100]
$Pn'c2'$ (30.113) $\mathbb{Z}_1$	• $\boldsymbol{B}$ $\parallel$ [100]
P_cc (7.28) $\mathbb{Z}_1$	• strain $\perp$ [100]<br>• ($\boldsymbol{E}$ $\parallel$ [010] or $\boldsymbol{E}$ $\perp$ [100])

Table. S 216: Subgroups of P_Aca2_1 (29.107) that can be realized by applying $\boldsymbol{E}/\boldsymbol{B}$ fields and/or uniaxial strains.

Subgroup & SI group	Perturbation
P_S1 (1.3) $\mathbb{Z}_1$	• strain in generic direction<br>• ($\boldsymbol{E}$ $\parallel$ [100] or $\boldsymbol{E}$ $\perp$ [010]) and strain $\perp$ [100]<br>• ($\boldsymbol{E}$ $\parallel$ [100] or $\boldsymbol{E}$ $\perp$ [010]) and strain $\perp$ [001]<br>• ($\boldsymbol{E}$ $\parallel$ [010] or $\boldsymbol{E}$ $\perp$ [100]) and strain $\perp$ [010]<br>• ($\boldsymbol{E}$ $\parallel$ [010] or $\boldsymbol{E}$ $\perp$ [100]) and strain $\perp$ [001]<br>• $\boldsymbol{E}$ in generic direction
Pc (7.24) $\mathbb{Z}_1$	• $\boldsymbol{B}$ $\parallel$ [010] and strain $\perp$ [010]<br>• ($\boldsymbol{E}$ $\parallel$ [100] or $\boldsymbol{E}$ $\perp$ [010]) and $\boldsymbol{B}$ $\parallel$ [010]
Pc' (7.26) $\mathbb{Z}_1$	• $\boldsymbol{B}$ $\parallel$ [100] and strain $\perp$ [010]<br>• $\boldsymbol{B}$ $\parallel$ [001] and strain $\perp$ [010]<br>• $\boldsymbol{B}$ $\perp$ [010]<br>• ($\boldsymbol{E}$ $\parallel$ [100] or $\boldsymbol{E}$ $\perp$ [010]) and $\boldsymbol{B}$ $\parallel$ [100]<br>• ($\boldsymbol{E}$ $\parallel$ [100] or $\boldsymbol{E}$ $\perp$ [010]) and $\boldsymbol{B}$ $\parallel$ [001]
P_Cc (7.30) $\mathbb{Z}_1$	• strain $\perp$ [010]<br>• ($\boldsymbol{E}$ $\parallel$ [100] or $\boldsymbol{E}$ $\perp$ [010])

Continued on next page

. . . continued

Subgroup & SI group	Perturbation
$P2_1$ (4.7) $\mathbb{Z}_1$	• $\boldsymbol{B}$ ∥ [001] and strain ⊥ [001]
$P2'$ (3.3) $\mathbb{Z}_1$	• $\boldsymbol{B}$ ∥ [100] and strain ⊥ [001]<br>• $\boldsymbol{B}$ ∥ [010] and strain ⊥ [001]<br>• $\boldsymbol{B}$ ⊥ [001]
P_C2_1 (4.12) $\mathbb{Z}_1$	• strain ⊥ [001]
Pc (7.24) $\mathbb{Z}_1$	• $\boldsymbol{B}$ ∥ [100] and strain ⊥ [100]<br>• ($\boldsymbol{E}$ ∥ [010] or $\boldsymbol{E}$ ⊥ [100]) and $\boldsymbol{B}$ ∥ [100]
$Pn'c2'$ (30.113) $\mathbb{Z}_1$	• $\boldsymbol{B}$ ∥ [100]
Pc' (7.26) $\mathbb{Z}_1$	• $\boldsymbol{B}$ ∥ [010] and strain ⊥ [100]<br>• $\boldsymbol{B}$ ∥ [001] and strain ⊥ [100]<br>• $\boldsymbol{B}$ ⊥ [100]<br>• ($\boldsymbol{E}$ ∥ [010] or $\boldsymbol{E}$ ⊥ [100]) and $\boldsymbol{B}$ ∥ [010]<br>• ($\boldsymbol{E}$ ∥ [010] or $\boldsymbol{E}$ ⊥ [100]) and $\boldsymbol{B}$ ∥ [001]
$Pn'a'2_1$ (33.148) $\mathbb{Z}_1$	• $\boldsymbol{B}$ ∥ [001]
$Pb'a2'$ (32.137) $\mathbb{Z}_1$	• $\boldsymbol{B}$ ∥ [010]
P_ac (7.27) $\mathbb{Z}_1$	• strain ⊥ [100]<br>• ($\boldsymbol{E}$ ∥ [010] or $\boldsymbol{E}$ ⊥ [100])

Table. S 217: Subgroups of P_Bca2_1 (29.108) that can be realized by applying $\boldsymbol{E}/\boldsymbol{B}$ fields and/or uniaxial strains.

Subgroup & SI group	Perturbation
Pc' (7.26) $\mathbb{Z}_1$	• $\boldsymbol{B}$ ∥ [100] and strain ⊥ [010]<br>• $\boldsymbol{B}$ ∥ [001] and strain ⊥ [010]<br>• $\boldsymbol{B}$ ⊥ [010]<br>• ($\boldsymbol{E}$ ∥ [100] or $\boldsymbol{E}$ ⊥ [010]) and $\boldsymbol{B}$ ∥ [100]<br>• ($\boldsymbol{E}$ ∥ [100] or $\boldsymbol{E}$ ⊥ [010]) and $\boldsymbol{B}$ ∥ [001]
P_S1 (1.3) $\mathbb{Z}_1$	• strain in generic direction<br>• ($\boldsymbol{E}$ ∥ [100] or $\boldsymbol{E}$ ⊥ [010]) and strain ⊥ [100]<br>• ($\boldsymbol{E}$ ∥ [100] or $\boldsymbol{E}$ ⊥ [010]) and strain ⊥ [001]<br>• ($\boldsymbol{E}$ ∥ [010] or $\boldsymbol{E}$ ⊥ [100]) and strain ⊥ [010]<br>• ($\boldsymbol{E}$ ∥ [010] or $\boldsymbol{E}$ ⊥ [100]) and strain ⊥ [001]<br>• $\boldsymbol{E}$ in generic direction

Continued on next page

...continued

Subgroup & SI group	Perturbation
Pc (7.24) $\mathbb{Z}_1$	• $\boldsymbol{B}$ ∥ [010] and strain ⊥ [010]<br>• ($\boldsymbol{E}$ ∥ [100] or $\boldsymbol{E}$ ⊥ [010]) and $\boldsymbol{B}$ ∥ [010]
P_ac (7.27) $\mathbb{Z}_1$	• strain ⊥ [010]<br>• ($\boldsymbol{E}$ ∥ [100] or $\boldsymbol{E}$ ⊥ [010])
Pm' (6.20) $\mathbb{Z}_1$	• $\boldsymbol{B}$ ∥ [010] and strain ⊥ [100]<br>• $\boldsymbol{B}$ ∥ [001] and strain ⊥ [100]<br>• $\boldsymbol{B}$ ⊥ [100]<br>• ($\boldsymbol{E}$ ∥ [010] or $\boldsymbol{E}$ ⊥ [100]) and $\boldsymbol{B}$ ∥ [010]<br>• ($\boldsymbol{E}$ ∥ [010] or $\boldsymbol{E}$ ⊥ [100]) and $\boldsymbol{B}$ ∥ [001]
$P2_1$ (4.7) $\mathbb{Z}_1$	• $\boldsymbol{B}$ ∥ [001] and strain ⊥ [001]
$Pm'c'2_1$ (26.70) $\mathbb{Z}_1$	• $\boldsymbol{B}$ ∥ [001]
Pc (7.24) $\mathbb{Z}_1$	• $\boldsymbol{B}$ ∥ [100] and strain ⊥ [100]<br>• ($\boldsymbol{E}$ ∥ [010] or $\boldsymbol{E}$ ⊥ [100]) and $\boldsymbol{B}$ ∥ [100]
P_Ac (7.31) $\mathbb{Z}_1$	• strain ⊥ [100]<br>• ($\boldsymbol{E}$ ∥ [010] or $\boldsymbol{E}$ ⊥ [100])
$P2'$ (3.3) $\mathbb{Z}_1$	• $\boldsymbol{B}$ ∥ [100] and strain ⊥ [001]<br>• $\boldsymbol{B}$ ∥ [010] and strain ⊥ [001]<br>• $\boldsymbol{B}$ ⊥ [001]
$Pm'a2'$ (28.89) $\mathbb{Z}_1$	• $\boldsymbol{B}$ ∥ [010]
P_C2_1 (4.12) $\mathbb{Z}_1$	• strain ⊥ [001]
$Pc'c2'$ (27.80) $\mathbb{Z}_1$	• $\boldsymbol{B}$ ∥ [100]

Table. S 218: Subgroups of P_Cca2_1 (29.109) that can be realized by applying $\boldsymbol{E}/\boldsymbol{B}$ fields and/or uniaxial strains.

Subgroup & SI group	Perturbation
Pm' (6.20) $\mathbb{Z}_1$	• $\boldsymbol{B}$ ∥ [100] and strain ⊥ [010]<br>• $\boldsymbol{B}$ ∥ [001] and strain ⊥ [010]<br>• $\boldsymbol{B}$ ⊥ [010]<br>• ($\boldsymbol{E}$ ∥ [100] or $\boldsymbol{E}$ ⊥ [010]) and $\boldsymbol{B}$ ∥ [100]<br>• ($\boldsymbol{E}$ ∥ [100] or $\boldsymbol{E}$ ⊥ [010]) and $\boldsymbol{B}$ ∥ [001]

Continued on next page

...continued

Subgroup & SI group	Perturbation
P_S1 (1.3) $\mathbb{Z}_1$	• strain in generic direction<br>• ($\boldsymbol{E} \parallel [100]$ or $\boldsymbol{E} \perp [010]$) and strain $\perp [100]$<br>• ($\boldsymbol{E} \parallel [100]$ or $\boldsymbol{E} \perp [010]$) and strain $\perp [001]$<br>• ($\boldsymbol{E} \parallel [010]$ or $\boldsymbol{E} \perp [100]$) and strain $\perp [010]$<br>• ($\boldsymbol{E} \parallel [010]$ or $\boldsymbol{E} \perp [100]$) and strain $\perp [001]$<br>• $\boldsymbol{E}$ in generic direction
Pc (7.24) $\mathbb{Z}_1$	• $\boldsymbol{B} \parallel [010]$ and strain $\perp [010]$<br>• ($\boldsymbol{E} \parallel [100]$ or $\boldsymbol{E} \perp [010]$) and $\boldsymbol{B} \parallel [010]$
P_Ac (7.31) $\mathbb{Z}_1$	• strain $\perp [010]$<br>• ($\boldsymbol{E} \parallel [100]$ or $\boldsymbol{E} \perp [010]$)
Pc' (7.26) $\mathbb{Z}_1$	• $\boldsymbol{B} \parallel [010]$ and strain $\perp [100]$<br>• $\boldsymbol{B} \parallel [001]$ and strain $\perp [100]$<br>• $\boldsymbol{B} \perp [100]$<br>• ($\boldsymbol{E} \parallel [010]$ or $\boldsymbol{E} \perp [100]$) and $\boldsymbol{B} \parallel [010]$<br>• ($\boldsymbol{E} \parallel [010]$ or $\boldsymbol{E} \perp [100]$) and $\boldsymbol{B} \parallel [001]$
$P2_1$ (4.7) $\mathbb{Z}_1$	• $\boldsymbol{B} \parallel [001]$ and strain $\perp [001]$
$Pm'n'2_1$ (31.127) $\mathbb{Z}_1$	• $\boldsymbol{B} \parallel [001]$
Pc (7.24) $\mathbb{Z}_1$	• $\boldsymbol{B} \parallel [100]$ and strain $\perp [100]$<br>• ($\boldsymbol{E} \parallel [010]$ or $\boldsymbol{E} \perp [100]$) and $\boldsymbol{B} \parallel [100]$
P_Cc (7.30) $\mathbb{Z}_1$	• strain $\perp [100]$<br>• ($\boldsymbol{E} \parallel [010]$ or $\boldsymbol{E} \perp [100]$)
$P2_1'$ (4.9) $\mathbb{Z}_1$	• $\boldsymbol{B} \parallel [100]$ and strain $\perp [001]$<br>• $\boldsymbol{B} \parallel [010]$ and strain $\perp [001]$<br>• $\boldsymbol{B} \perp [001]$
$Pn'a2_1'$ (33.146) $\mathbb{Z}_1$	• $\boldsymbol{B} \parallel [010]$
P_a2_1 (4.10) $\mathbb{Z}_1$	• strain $\perp [001]$
$Pm'c2_1'$ (26.68) $\mathbb{Z}_1$	• $\boldsymbol{B} \parallel [100]$

Table. S 219: Subgroups of P_Ica2_1 (29.110) that can be realized by applying $\boldsymbol{E}/\boldsymbol{B}$ fields and/or uniaxial strains.

Subgroup & SI group	Perturbation

Continued on next page

... continued

Subgroup & SI group	Perturbation
Pc' (7.26) $\mathbb{Z}_1$	• $\boldsymbol{B} \parallel [100]$ and strain $\perp [010]$<br>• $\boldsymbol{B} \parallel [001]$ and strain $\perp [010]$<br>• $\boldsymbol{B} \perp [010]$<br>• ($\boldsymbol{E} \parallel [100]$ or $\boldsymbol{E} \perp [010]$) and $\boldsymbol{B} \parallel [100]$<br>• ($\boldsymbol{E} \parallel [100]$ or $\boldsymbol{E} \perp [010]$) and $\boldsymbol{B} \parallel [001]$
P_S1 (1.3) $\mathbb{Z}_1$	• strain in generic direction<br>• ($\boldsymbol{E} \parallel [100]$ or $\boldsymbol{E} \perp [010]$) and strain $\perp [100]$<br>• ($\boldsymbol{E} \parallel [100]$ or $\boldsymbol{E} \perp [010]$) and strain $\perp [001]$<br>• ($\boldsymbol{E} \parallel [010]$ or $\boldsymbol{E} \perp [100]$) and strain $\perp [010]$<br>• ($\boldsymbol{E} \parallel [010]$ or $\boldsymbol{E} \perp [100]$) and strain $\perp [001]$<br>• $\boldsymbol{E}$ in generic direction
Pc (7.24) $\mathbb{Z}_1$	• $\boldsymbol{B} \parallel [010]$ and strain $\perp [010]$<br>• ($\boldsymbol{E} \parallel [100]$ or $\boldsymbol{E} \perp [010]$) and $\boldsymbol{B} \parallel [010]$
P_Cc (7.30) $\mathbb{Z}_1$	• strain $\perp [010]$<br>• ($\boldsymbol{E} \parallel [100]$ or $\boldsymbol{E} \perp [010]$)
Pc' (7.26) $\mathbb{Z}_1$	• $\boldsymbol{B} \parallel [010]$ and strain $\perp [100]$<br>• $\boldsymbol{B} \parallel [001]$ and strain $\perp [100]$<br>• $\boldsymbol{B} \perp [100]$<br>• ($\boldsymbol{E} \parallel [010]$ or $\boldsymbol{E} \perp [100]$) and $\boldsymbol{B} \parallel [010]$<br>• ($\boldsymbol{E} \parallel [010]$ or $\boldsymbol{E} \perp [100]$) and $\boldsymbol{B} \parallel [001]$
$P2_1$ (4.7) $\mathbb{Z}_1$	• $\boldsymbol{B} \parallel [001]$ and strain $\perp [001]$
$Pc'a'2_1$ (29.103) $\mathbb{Z}_1$	• $\boldsymbol{B} \parallel [001]$
Pc (7.24) $\mathbb{Z}_1$	• $\boldsymbol{B} \parallel [100]$ and strain $\perp [100]$<br>• ($\boldsymbol{E} \parallel [010]$ or $\boldsymbol{E} \perp [100]$) and $\boldsymbol{B} \parallel [100]$
P_Cc (7.30) $\mathbb{Z}_1$	• strain $\perp [100]$<br>• ($\boldsymbol{E} \parallel [010]$ or $\boldsymbol{E} \perp [100]$)
$P2'$ (3.3) $\mathbb{Z}_1$	• $\boldsymbol{B} \parallel [100]$ and strain $\perp [001]$<br>• $\boldsymbol{B} \parallel [010]$ and strain $\perp [001]$<br>• $\boldsymbol{B} \perp [001]$
$Pb'a2'$ (32.137) $\mathbb{Z}_1$	• $\boldsymbol{B} \parallel [010]$
P_C2_1 (4.12) $\mathbb{Z}_1$	• strain $\perp [001]$
$Pc'c2'$ (27.80) $\mathbb{Z}_1$	• $\boldsymbol{B} \parallel [100]$

Table. S 220: Subgroups of $Pca2_1$ (29.99) that can be realized by applying $\boldsymbol{E}/\boldsymbol{B}$ fields and/or uniaxial strains.

Subgroup & SI group	Perturbation
Pc (7.24) $\mathbb{Z}_1$	• strain $\perp$ [010]<br>• $\boldsymbol{B} \parallel$ [010]<br>• ($\boldsymbol{E} \parallel$ [100] or $\boldsymbol{E} \perp$ [010])
$P2_1$ (4.7) $\mathbb{Z}_1$	• strain $\perp$ [001]<br>• $\boldsymbol{B} \parallel$ [001]
Pc (7.24) $\mathbb{Z}_1$	• strain $\perp$ [100]<br>• $\boldsymbol{B} \parallel$ [100]<br>• ($\boldsymbol{E} \parallel$ [010] or $\boldsymbol{E} \perp$ [100])

Table. S 221: Subgroups of $Pnc2$ (30.111) that can be realized by applying $\boldsymbol{E}/\boldsymbol{B}$ fields and/or uniaxial strains.

Subgroup & SI group	Perturbation
Pc (7.24) $\mathbb{Z}_1$	• strain $\perp$ [010]<br>• $\boldsymbol{B} \parallel$ [010]<br>• ($\boldsymbol{E} \parallel$ [100] or $\boldsymbol{E} \perp$ [010])
Pc (7.24) $\mathbb{Z}_1$	• strain $\perp$ [100]<br>• $\boldsymbol{B} \parallel$ [100]<br>• ($\boldsymbol{E} \parallel$ [010] or $\boldsymbol{E} \perp$ [100])
$P2$ (3.1) $\mathbb{Z}_2$	• strain $\perp$ [001]<br>• $\boldsymbol{B} \parallel$ [001]

Table. S 222: Subgroups of $Pnc21'$ (30.112) that can be realized by applying $\boldsymbol{E}/\boldsymbol{B}$ fields and/or uniaxial strains.

Subgroup & SI group	Perturbation

Continued on next page

. . . continued

Subgroup & SI group	Perturbation
$P11'$ (1.2) $\mathbb{Z}_1$	• strain in generic direction<br>• ($\boldsymbol{E}$ ∥ [100] or $\boldsymbol{E} \perp$ [010]) and strain ⊥ [100]<br>• ($\boldsymbol{E}$ ∥ [100] or $\boldsymbol{E} \perp$ [010]) and strain ⊥ [001]<br>• ($\boldsymbol{E}$ ∥ [010] or $\boldsymbol{E} \perp$ [100]) and strain ⊥ [010]<br>• ($\boldsymbol{E}$ ∥ [010] or $\boldsymbol{E} \perp$ [100]) and strain ⊥ [001]<br>• $\boldsymbol{E}$ in generic direction
Pc' (7.26) $\mathbb{Z}_1$	• $\boldsymbol{B}$ ∥ [100] and strain ⊥ [010]<br>• $\boldsymbol{B}$ ∥ [001] and strain ⊥ [010]<br>• $\boldsymbol{B} \perp$ [010]<br>• ($\boldsymbol{E}$ ∥ [100] or $\boldsymbol{E} \perp$ [010]) and $\boldsymbol{B}$ ∥ [100]<br>• ($\boldsymbol{E}$ ∥ [100] or $\boldsymbol{E} \perp$ [010]) and $\boldsymbol{B}$ ∥ [001]
Pc (7.24) $\mathbb{Z}_1$	• $\boldsymbol{B}$ ∥ [010] and strain ⊥ [010]<br>• ($\boldsymbol{E}$ ∥ [100] or $\boldsymbol{E} \perp$ [010]) and $\boldsymbol{B}$ ∥ [010]
$Pc1'$ (7.25) $\mathbb{Z}_1$	• strain ⊥ [010]<br>• ($\boldsymbol{E}$ ∥ [100] or $\boldsymbol{E} \perp$ [010])
Pc' (7.26) $\mathbb{Z}_1$	• $\boldsymbol{B}$ ∥ [010] and strain ⊥ [100]<br>• $\boldsymbol{B}$ ∥ [001] and strain ⊥ [100]<br>• $\boldsymbol{B} \perp$ [100]<br>• ($\boldsymbol{E}$ ∥ [010] or $\boldsymbol{E} \perp$ [100]) and $\boldsymbol{B}$ ∥ [010]<br>• ($\boldsymbol{E}$ ∥ [010] or $\boldsymbol{E} \perp$ [100]) and $\boldsymbol{B}$ ∥ [001]
Pc (7.24) $\mathbb{Z}_1$	• $\boldsymbol{B}$ ∥ [100] and strain ⊥ [100]<br>• ($\boldsymbol{E}$ ∥ [010] or $\boldsymbol{E} \perp$ [100]) and $\boldsymbol{B}$ ∥ [100]
$Pc1'$ (7.25) $\mathbb{Z}_1$	• strain ⊥ [100]<br>• ($\boldsymbol{E}$ ∥ [010] or $\boldsymbol{E} \perp$ [100])
$P2'$ (3.3) $\mathbb{Z}_1$	• $\boldsymbol{B}$ ∥ [100] and strain ⊥ [001]<br>• $\boldsymbol{B}$ ∥ [010] and strain ⊥ [001]<br>• $\boldsymbol{B} \perp$ [001]
$Pn'c2'$ (30.113) $\mathbb{Z}_1$	• $\boldsymbol{B}$ ∥ [010]
$Pnc'2'$ (30.114) $\mathbb{Z}_1$	• $\boldsymbol{B}$ ∥ [100]
$P2$ (3.1) $\mathbb{Z}_2$	• $\boldsymbol{B}$ ∥ [001] and strain ⊥ [001]
$Pn'c'2$ (30.115) $\mathbb{Z}_1$	• $\boldsymbol{B}$ ∥ [001]
$P21'$ (3.2) $\mathbb{Z}_2$	• strain ⊥ [001]

Table. S 223: Subgroups of $Pn'c2'$ (30.113) that can be realized by applying $\boldsymbol{E}/\boldsymbol{B}$ fields and/or uniaxial strains.

Subgroup & SI group	Perturbation
Pc (7.24) $\mathbb{Z}_1$	• strain $\perp$ [010]<br>• ($\boldsymbol{E} \parallel$ [100] or $\boldsymbol{E} \perp$ [010])
Pc' (7.26) $\mathbb{Z}_1$	• strain $\perp$ [100]<br>• ($\boldsymbol{B} \parallel$ [001] or $\boldsymbol{B} \perp$ [100])<br>• ($\boldsymbol{E} \parallel$ [010] or $\boldsymbol{E} \perp$ [100])
$P2'$ (3.3) $\mathbb{Z}_1$	• strain $\perp$ [001]<br>• ($\boldsymbol{B} \parallel$ [100] or $\boldsymbol{B} \perp$ [001])

Table. S 224: Subgroups of $Pnc'2'$ (30.114) that can be realized by applying $\boldsymbol{E}/\boldsymbol{B}$ fields and/or uniaxial strains.

Subgroup & SI group	Perturbation
Pc' (7.26) $\mathbb{Z}_1$	• strain $\perp$ [010]<br>• ($\boldsymbol{B} \parallel$ [001] or $\boldsymbol{B} \perp$ [010])<br>• ($\boldsymbol{E} \parallel$ [100] or $\boldsymbol{E} \perp$ [010])
Pc (7.24) $\mathbb{Z}_1$	• strain $\perp$ [100]<br>• ($\boldsymbol{E} \parallel$ [010] or $\boldsymbol{E} \perp$ [100])
$P2'$ (3.3) $\mathbb{Z}_1$	• strain $\perp$ [001]<br>• ($\boldsymbol{B} \parallel$ [010] or $\boldsymbol{B} \perp$ [001])

Table. S 225: Subgroups of $Pn'c'2$ (30.115) that can be realized by applying $\boldsymbol{E}/\boldsymbol{B}$ fields and/or uniaxial strains.

Subgroup & SI group	Perturbation
Pc' (7.26) $\mathbb{Z}_1$	• strain $\perp$ [010]<br>• ($\boldsymbol{B} \parallel$ [100] or $\boldsymbol{B} \perp$ [010])<br>• ($\boldsymbol{E} \parallel$ [100] or $\boldsymbol{E} \perp$ [010])
Pc' (7.26) $\mathbb{Z}_1$	• strain $\perp$ [100]<br>• ($\boldsymbol{B} \parallel$ [010] or $\boldsymbol{B} \perp$ [100])<br>• ($\boldsymbol{E} \parallel$ [010] or $\boldsymbol{E} \perp$ [100])

Continued on next page

. . . continued

Subgroup & SI group	Perturbation
$P2$ (3.1) $\mathbb{Z}_2$	• strain $\perp$ [001]

Table. S 226: Subgroups of P_anc2 (30.116) that can be realized by applying $\boldsymbol{E}/\boldsymbol{B}$ fields and/or uniaxial strains.

Subgroup & SI group	Perturbation
Pc (7.24) $\mathbb{Z}_1$	• $\boldsymbol{B} \parallel$ [010] and strain $\perp$ [010]<br>• ($\boldsymbol{E} \parallel$ [100] or $\boldsymbol{E} \perp$ [010]) and $\boldsymbol{B} \parallel$ [010]
P_S1 (1.3) $\mathbb{Z}_1$	• strain in generic direction<br>• ($\boldsymbol{E} \parallel$ [100] or $\boldsymbol{E} \perp$ [010]) and strain $\perp$ [100]<br>• ($\boldsymbol{E} \parallel$ [100] or $\boldsymbol{E} \perp$ [010]) and strain $\perp$ [001]<br>• ($\boldsymbol{E} \parallel$ [010] or $\boldsymbol{E} \perp$ [100]) and strain $\perp$ [010]<br>• ($\boldsymbol{E} \parallel$ [010] or $\boldsymbol{E} \perp$ [100]) and strain $\perp$ [001]<br>• $\boldsymbol{E}$ in generic direction
Pc' (7.26) $\mathbb{Z}_1$	• $\boldsymbol{B} \parallel$ [100] and strain $\perp$ [010]<br>• $\boldsymbol{B} \parallel$ [001] and strain $\perp$ [010]<br>• $\boldsymbol{B} \perp$ [010]<br>• ($\boldsymbol{E} \parallel$ [100] or $\boldsymbol{E} \perp$ [010]) and $\boldsymbol{B} \parallel$ [100]<br>• ($\boldsymbol{E} \parallel$ [100] or $\boldsymbol{E} \perp$ [010]) and $\boldsymbol{B} \parallel$ [001]
P_ac (7.27) $\mathbb{Z}_1$	• strain $\perp$ [010]<br>• ($\boldsymbol{E} \parallel$ [100] or $\boldsymbol{E} \perp$ [010])
Pc (7.24) $\mathbb{Z}_1$	• $\boldsymbol{B} \parallel$ [100] and strain $\perp$ [100]<br>• ($\boldsymbol{E} \parallel$ [010] or $\boldsymbol{E} \perp$ [100]) and $\boldsymbol{B} \parallel$ [100]
$P2$ (3.1) $\mathbb{Z}_2$	• $\boldsymbol{B} \parallel$ [001] and strain $\perp$ [001]
Pc' (7.26) $\mathbb{Z}_1$	• $\boldsymbol{B} \parallel$ [010] and strain $\perp$ [100]<br>• $\boldsymbol{B} \parallel$ [001] and strain $\perp$ [100]<br>• $\boldsymbol{B} \perp$ [100]<br>• ($\boldsymbol{E} \parallel$ [010] or $\boldsymbol{E} \perp$ [100]) and $\boldsymbol{B} \parallel$ [010]<br>• ($\boldsymbol{E} \parallel$ [010] or $\boldsymbol{E} \perp$ [100]) and $\boldsymbol{B} \parallel$ [001]
P_bc (7.29) $\mathbb{Z}_1$	• strain $\perp$ [100]<br>• ($\boldsymbol{E} \parallel$ [010] or $\boldsymbol{E} \perp$ [100])
$Pn'n'2$ (34.159) $\mathbb{Z}_1$	• $\boldsymbol{B} \parallel$ [001]

Continued on next page

. . . continued

Subgroup & SI group	Perturbation
$P2'$ (3.3) $\mathbb{Z}_1$	• $\boldsymbol{B} \parallel [100]$ and strain $\perp [001]$<br>• $\boldsymbol{B} \parallel [010]$ and strain $\perp [001]$<br>• $\boldsymbol{B} \perp [001]$
$Pn'n2'$ (34.158) $\mathbb{Z}_1$	• $\boldsymbol{B} \parallel [100]$
P_a2 (3.4) $\mathbb{Z}_2$	• strain $\perp [001]$
$Pn'c2'$ (30.113) $\mathbb{Z}_1$	• $\boldsymbol{B} \parallel [010]$

Table. S 227: Subgroups of P_bnc2 (30.117) that can be realized by applying $\boldsymbol{E}/\boldsymbol{B}$ fields and/or uniaxial strains.

Subgroup & SI group	Perturbation
P_S1 (1.3) $\mathbb{Z}_1$	• strain in generic direction<br>• ($\boldsymbol{E} \parallel [100]$ or $\boldsymbol{E} \perp [010]$) and strain $\perp [100]$<br>• ($\boldsymbol{E} \parallel [100]$ or $\boldsymbol{E} \perp [010]$) and strain $\perp [001]$<br>• ($\boldsymbol{E} \parallel [010]$ or $\boldsymbol{E} \perp [100]$) and strain $\perp [010]$<br>• ($\boldsymbol{E} \parallel [010]$ or $\boldsymbol{E} \perp [100]$) and strain $\perp [001]$<br>• $\boldsymbol{E}$ in generic direction
Pc' (7.26) $\mathbb{Z}_1$	• $\boldsymbol{B} \parallel [100]$ and strain $\perp [010]$<br>• $\boldsymbol{B} \parallel [001]$ and strain $\perp [010]$<br>• $\boldsymbol{B} \perp [010]$<br>• ($\boldsymbol{E} \parallel [100]$ or $\boldsymbol{E} \perp [010]$) and $\boldsymbol{B} \parallel [100]$<br>• ($\boldsymbol{E} \parallel [100]$ or $\boldsymbol{E} \perp [010]$) and $\boldsymbol{B} \parallel [001]$
Pc (7.24) $\mathbb{Z}_1$	• $\boldsymbol{B} \parallel [010]$ and strain $\perp [010]$<br>• ($\boldsymbol{E} \parallel [100]$ or $\boldsymbol{E} \perp [010]$) and $\boldsymbol{B} \parallel [010]$
P_bc (7.29) $\mathbb{Z}_1$	• strain $\perp [010]$<br>• ($\boldsymbol{E} \parallel [100]$ or $\boldsymbol{E} \perp [010]$)
Pc' (7.26) $\mathbb{Z}_1$	• $\boldsymbol{B} \parallel [010]$ and strain $\perp [100]$<br>• $\boldsymbol{B} \parallel [001]$ and strain $\perp [100]$<br>• $\boldsymbol{B} \perp [100]$<br>• ($\boldsymbol{E} \parallel [010]$ or $\boldsymbol{E} \perp [100]$) and $\boldsymbol{B} \parallel [010]$<br>• ($\boldsymbol{E} \parallel [010]$ or $\boldsymbol{E} \perp [100]$) and $\boldsymbol{B} \parallel [001]$
Pc (7.24) $\mathbb{Z}_1$	• $\boldsymbol{B} \parallel [100]$ and strain $\perp [100]$<br>• ($\boldsymbol{E} \parallel [010]$ or $\boldsymbol{E} \perp [100]$) and $\boldsymbol{B} \parallel [100]$

Continued on next page

. . . continued

Subgroup & SI group	Perturbation
P_ac (7.27) $\mathbb{Z}_1$	• strain $\perp$ [100]<br>• ($\boldsymbol{E}$ $\parallel$ [010] or $\boldsymbol{E}$ $\perp$ [100])
$P2$ (3.1) $\mathbb{Z}_2$	• $\boldsymbol{B}$ $\parallel$ [001] and strain $\perp$ [001]
$Pc'c'2$ (27.81) $\mathbb{Z}_2$	• $\boldsymbol{B}$ $\parallel$ [001]
$P2'$ (3.3) $\mathbb{Z}_1$	• $\boldsymbol{B}$ $\parallel$ [100] and strain $\perp$ [001]<br>• $\boldsymbol{B}$ $\parallel$ [010] and strain $\perp$ [001]<br>• $\boldsymbol{B}$ $\perp$ [001]
$Pc'c2'$ (27.80) $\mathbb{Z}_1$	• $\boldsymbol{B}$ $\parallel$ [010]
$Pnc'2'$ (30.114) $\mathbb{Z}_1$	• $\boldsymbol{B}$ $\parallel$ [100]
P_a2 (3.4) $\mathbb{Z}_2$	• strain $\perp$ [001]

Table. S 228: Subgroups of P_cnc2 (30.118) that can be realized by applying $\boldsymbol{E}/\boldsymbol{B}$ fields and/or uniaxial strains.

Subgroup & SI group	Perturbation
P_S1 (1.3) $\mathbb{Z}_1$	• strain in generic direction<br>• ($\boldsymbol{E}$ $\parallel$ [100] or $\boldsymbol{E}$ $\perp$ [010]) and strain $\perp$ [100]<br>• ($\boldsymbol{E}$ $\parallel$ [100] or $\boldsymbol{E}$ $\perp$ [010]) and strain $\perp$ [001]<br>• ($\boldsymbol{E}$ $\parallel$ [010] or $\boldsymbol{E}$ $\perp$ [100]) and strain $\perp$ [010]<br>• ($\boldsymbol{E}$ $\parallel$ [010] or $\boldsymbol{E}$ $\perp$ [100]) and strain $\perp$ [001]<br>• $\boldsymbol{E}$ in generic direction
Pm' (6.20) $\mathbb{Z}_1$	• $\boldsymbol{B}$ $\parallel$ [100] and strain $\perp$ [010]<br>• $\boldsymbol{B}$ $\parallel$ [001] and strain $\perp$ [010]<br>• $\boldsymbol{B}$ $\perp$ [010]<br>• ($\boldsymbol{E}$ $\parallel$ [100] or $\boldsymbol{E}$ $\perp$ [010]) and $\boldsymbol{B}$ $\parallel$ [100]<br>• ($\boldsymbol{E}$ $\parallel$ [100] or $\boldsymbol{E}$ $\perp$ [010]) and $\boldsymbol{B}$ $\parallel$ [001]
Pc (7.24) $\mathbb{Z}_1$	• $\boldsymbol{B}$ $\parallel$ [010] and strain $\perp$ [010]<br>• ($\boldsymbol{E}$ $\parallel$ [100] or $\boldsymbol{E}$ $\perp$ [010]) and $\boldsymbol{B}$ $\parallel$ [010]
P_cc (7.28) $\mathbb{Z}_1$	• strain $\perp$ [010]<br>• ($\boldsymbol{E}$ $\parallel$ [100] or $\boldsymbol{E}$ $\perp$ [010])

Continued on next page

...continued

Subgroup & SI group	Perturbation
Pc' (7.26) $\mathbb{Z}_1$	• $\boldsymbol{B}$ ∥ [010] and strain ⊥ [100]<br>• $\boldsymbol{B}$ ∥ [001] and strain ⊥ [100]<br>• $\boldsymbol{B}$ ⊥ [100]<br>• ($\boldsymbol{E}$ ∥ [010] or $\boldsymbol{E}$ ⊥ [100]) and $\boldsymbol{B}$ ∥ [010]<br>• ($\boldsymbol{E}$ ∥ [010] or $\boldsymbol{E}$ ⊥ [100]) and $\boldsymbol{B}$ ∥ [001]
Pc (7.24) $\mathbb{Z}_1$	• $\boldsymbol{B}$ ∥ [100] and strain ⊥ [100]<br>• ($\boldsymbol{E}$ ∥ [010] or $\boldsymbol{E}$ ⊥ [100]) and $\boldsymbol{B}$ ∥ [100]
P_ac (7.27) $\mathbb{Z}_1$	• strain ⊥ [100]<br>• ($\boldsymbol{E}$ ∥ [010] or $\boldsymbol{E}$ ⊥ [100])
$P2$ (3.1) $\mathbb{Z}_2$	• $\boldsymbol{B}$ ∥ [001] and strain ⊥ [001]
$Pm'a'2$ (28.91) $\mathbb{Z}_2$	• $\boldsymbol{B}$ ∥ [001]
$P2_1'$ (4.9) $\mathbb{Z}_1$	• $\boldsymbol{B}$ ∥ [100] and strain ⊥ [001]<br>• $\boldsymbol{B}$ ∥ [010] and strain ⊥ [001]<br>• $\boldsymbol{B}$ ⊥ [001]
$Pca'2_1'$ (29.102) $\mathbb{Z}_1$	• $\boldsymbol{B}$ ∥ [010]
$Pm'n2_1'$ (31.125) $\mathbb{Z}_1$	• $\boldsymbol{B}$ ∥ [100]
P_b2 (3.5) $\mathbb{Z}_2$	• strain ⊥ [001]

Table. S 229: Subgroups of P_Anc2 (30.119) that can be realized by applying $\boldsymbol{E}/\boldsymbol{B}$ fields and/or uniaxial strains.

Subgroup & SI group	Perturbation
P_S1 (1.3) $\mathbb{Z}_1$	• strain in generic direction<br>• ($\boldsymbol{E}$ ∥ [100] or $\boldsymbol{E}$ ⊥ [010]) and strain ⊥ [100]<br>• ($\boldsymbol{E}$ ∥ [100] or $\boldsymbol{E}$ ⊥ [010]) and strain ⊥ [001]<br>• ($\boldsymbol{E}$ ∥ [010] or $\boldsymbol{E}$ ⊥ [100]) and strain ⊥ [010]<br>• ($\boldsymbol{E}$ ∥ [010] or $\boldsymbol{E}$ ⊥ [100]) and strain ⊥ [001]<br>• $\boldsymbol{E}$ in generic direction
Pm' (6.20) $\mathbb{Z}_1$	• $\boldsymbol{B}$ ∥ [100] and strain ⊥ [010]<br>• $\boldsymbol{B}$ ∥ [001] and strain ⊥ [010]<br>• $\boldsymbol{B}$ ⊥ [010]<br>• ($\boldsymbol{E}$ ∥ [100] or $\boldsymbol{E}$ ⊥ [010]) and $\boldsymbol{B}$ ∥ [100]<br>• ($\boldsymbol{E}$ ∥ [100] or $\boldsymbol{E}$ ⊥ [010]) and $\boldsymbol{B}$ ∥ [001]

Continued on next page

...continued

Subgroup & SI group	Perturbation
Pc (7.24) $\mathbb{Z}_1$	• $\boldsymbol{B}$ ∥ [010] and strain ⊥ [010]<br>• ($\boldsymbol{E}$ ∥ [100] or $\boldsymbol{E}$ ⊥ [010]) and $\boldsymbol{B}$ ∥ [010]
P_Ac (7.31) $\mathbb{Z}_1$	• strain ⊥ [010]<br>• ($\boldsymbol{E}$ ∥ [100] or $\boldsymbol{E}$ ⊥ [010])
Pm' (6.20) $\mathbb{Z}_1$	• $\boldsymbol{B}$ ∥ [010] and strain ⊥ [100]<br>• $\boldsymbol{B}$ ∥ [001] and strain ⊥ [100]<br>• $\boldsymbol{B}$ ⊥ [100]<br>• ($\boldsymbol{E}$ ∥ [010] or $\boldsymbol{E}$ ⊥ [100]) and $\boldsymbol{B}$ ∥ [010]<br>• ($\boldsymbol{E}$ ∥ [010] or $\boldsymbol{E}$ ⊥ [100]) and $\boldsymbol{B}$ ∥ [001]
Pc (7.24) $\mathbb{Z}_1$	• $\boldsymbol{B}$ ∥ [100] and strain ⊥ [100]<br>• ($\boldsymbol{E}$ ∥ [010] or $\boldsymbol{E}$ ⊥ [100]) and $\boldsymbol{B}$ ∥ [100]
P_cc (7.28) $\mathbb{Z}_1$	• strain ⊥ [100]<br>• ($\boldsymbol{E}$ ∥ [010] or $\boldsymbol{E}$ ⊥ [100])
$P2$ (3.1) $\mathbb{Z}_2$	• $\boldsymbol{B}$ ∥ [001] and strain ⊥ [001]
$Pm'm'2$ (25.60) $\mathbb{Z}_2$	• $\boldsymbol{B}$ ∥ [001]
$P2_1'$ (4.9) $\mathbb{Z}_1$	• $\boldsymbol{B}$ ∥ [100] and strain ⊥ [001]<br>• $\boldsymbol{B}$ ∥ [010] and strain ⊥ [001]<br>• $\boldsymbol{B}$ ⊥ [001]
$Pm'c2_1'$ (26.68) $\mathbb{Z}_1$	• $\boldsymbol{B}$ ∥ [010]
$Pm'n2_1'$ (31.125) $\mathbb{Z}_1$	• $\boldsymbol{B}$ ∥ [100]
P_C2 (3.6) $\mathbb{Z}_1$	• strain ⊥ [001]

Table. S 230: Subgroups of P_Bnc2 (30.120) that can be realized by applying $\boldsymbol{E}/\boldsymbol{B}$ fields and/or uniaxial strains.

Subgroup & SI group	Perturbation
Pc (7.24) $\mathbb{Z}_1$	• $\boldsymbol{B}$ ∥ [010] and strain ⊥ [010]<br>• ($\boldsymbol{E}$ ∥ [100] or $\boldsymbol{E}$ ⊥ [010]) and $\boldsymbol{B}$ ∥ [010]

Continued on next page

. . . continued

Subgroup & SI group	Perturbation
P_S1 (1.3) $\mathbb{Z}_1$	• strain in generic direction<br>• ($\boldsymbol{E} \parallel [100]$ or $\boldsymbol{E} \perp [010]$) and strain $\perp [100]$<br>• ($\boldsymbol{E} \parallel [100]$ or $\boldsymbol{E} \perp [010]$) and strain $\perp [001]$<br>• ($\boldsymbol{E} \parallel [010]$ or $\boldsymbol{E} \perp [100]$) and strain $\perp [010]$<br>• ($\boldsymbol{E} \parallel [010]$ or $\boldsymbol{E} \perp [100]$) and strain $\perp [001]$<br>• $\boldsymbol{E}$ in generic direction
Pc' (7.26) $\mathbb{Z}_1$	• $\boldsymbol{B} \parallel [100]$ and strain $\perp [010]$<br>• $\boldsymbol{B} \parallel [001]$ and strain $\perp [010]$<br>• $\boldsymbol{B} \perp [010]$<br>• ($\boldsymbol{E} \parallel [100]$ or $\boldsymbol{E} \perp [010]$) and $\boldsymbol{B} \parallel [100]$<br>• ($\boldsymbol{E} \parallel [100]$ or $\boldsymbol{E} \perp [010]$) and $\boldsymbol{B} \parallel [001]$
P_ac (7.27) $\mathbb{Z}_1$	• strain $\perp [010]$<br>• ($\boldsymbol{E} \parallel [100]$ or $\boldsymbol{E} \perp [010]$)
Pc (7.24) $\mathbb{Z}_1$	• $\boldsymbol{B} \parallel [100]$ and strain $\perp [100]$<br>• ($\boldsymbol{E} \parallel [010]$ or $\boldsymbol{E} \perp [100]$) and $\boldsymbol{B} \parallel [100]$
$P2$ (3.1) $\mathbb{Z}_2$	• $\boldsymbol{B} \parallel [001]$ and strain $\perp [001]$
Pc' (7.26) $\mathbb{Z}_1$	• $\boldsymbol{B} \parallel [010]$ and strain $\perp [100]$<br>• $\boldsymbol{B} \parallel [001]$ and strain $\perp [100]$<br>• $\boldsymbol{B} \perp [100]$<br>• ($\boldsymbol{E} \parallel [010]$ or $\boldsymbol{E} \perp [100]$) and $\boldsymbol{B} \parallel [010]$<br>• ($\boldsymbol{E} \parallel [010]$ or $\boldsymbol{E} \perp [100]$) and $\boldsymbol{B} \parallel [001]$
P_Cc (7.30) $\mathbb{Z}_1$	• strain $\perp [100]$<br>• ($\boldsymbol{E} \parallel [010]$ or $\boldsymbol{E} \perp [100]$)
$Pb'a'2$ (32.138) $\mathbb{Z}_2$	• $\boldsymbol{B} \parallel [001]$
$P2_1'$ (4.9) $\mathbb{Z}_1$	• $\boldsymbol{B} \parallel [100]$ and strain $\perp [001]$<br>• $\boldsymbol{B} \parallel [010]$ and strain $\perp [001]$<br>• $\boldsymbol{B} \perp [001]$
$Pna'2_1'$ (33.147) $\mathbb{Z}_1$	• $\boldsymbol{B} \parallel [100]$
P_C2 (3.6) $\mathbb{Z}_1$	• strain $\perp [001]$
$Pca'2_1'$ (29.102) $\mathbb{Z}_1$	• $\boldsymbol{B} \parallel [010]$

Table. S 231: Subgroups of P_Cnc2 (30.121) that can be realized by applying $\boldsymbol{E}/\boldsymbol{B}$ fields and/or uniaxial strains.

Subgroup & SI group	Perturbation
Pc (7.24) $\mathbb{Z}_1$	• $\boldsymbol{B} \parallel [010]$ and strain $\perp [010]$<br>• ($\boldsymbol{E} \parallel [100]$ or $\boldsymbol{E} \perp [010]$) and $\boldsymbol{B} \parallel [010]$
P_S1 (1.3) $\mathbb{Z}_1$	• strain in generic direction<br>• ($\boldsymbol{E} \parallel [100]$ or $\boldsymbol{E} \perp [010]$) and strain $\perp [100]$<br>• ($\boldsymbol{E} \parallel [100]$ or $\boldsymbol{E} \perp [010]$) and strain $\perp [001]$<br>• ($\boldsymbol{E} \parallel [010]$ or $\boldsymbol{E} \perp [100]$) and strain $\perp [010]$<br>• ($\boldsymbol{E} \parallel [010]$ or $\boldsymbol{E} \perp [100]$) and strain $\perp [001]$<br>• $\boldsymbol{E}$ in generic direction
Pc' (7.26) $\mathbb{Z}_1$	• $\boldsymbol{B} \parallel [100]$ and strain $\perp [010]$<br>• $\boldsymbol{B} \parallel [001]$ and strain $\perp [010]$<br>• $\boldsymbol{B} \perp [010]$<br>• ($\boldsymbol{E} \parallel [100]$ or $\boldsymbol{E} \perp [010]$) and $\boldsymbol{B} \parallel [100]$<br>• ($\boldsymbol{E} \parallel [100]$ or $\boldsymbol{E} \perp [010]$) and $\boldsymbol{B} \parallel [001]$
P_Cc (7.30) $\mathbb{Z}_1$	• strain $\perp [010]$<br>• ($\boldsymbol{E} \parallel [100]$ or $\boldsymbol{E} \perp [010]$)
Pc (7.24) $\mathbb{Z}_1$	• $\boldsymbol{B} \parallel [100]$ and strain $\perp [100]$<br>• ($\boldsymbol{E} \parallel [010]$ or $\boldsymbol{E} \perp [100]$) and $\boldsymbol{B} \parallel [100]$
$P2$ (3.1) $\mathbb{Z}_2$	• $\boldsymbol{B} \parallel [001]$ and strain $\perp [001]$
Pc' (7.26) $\mathbb{Z}_1$	• $\boldsymbol{B} \parallel [010]$ and strain $\perp [100]$<br>• $\boldsymbol{B} \parallel [001]$ and strain $\perp [100]$<br>• $\boldsymbol{B} \perp [100]$<br>• ($\boldsymbol{E} \parallel [010]$ or $\boldsymbol{E} \perp [100]$) and $\boldsymbol{B} \parallel [010]$<br>• ($\boldsymbol{E} \parallel [010]$ or $\boldsymbol{E} \perp [100]$) and $\boldsymbol{B} \parallel [001]$
P_Cc (7.30) $\mathbb{Z}_1$	• strain $\perp [100]$<br>• ($\boldsymbol{E} \parallel [010]$ or $\boldsymbol{E} \perp [100]$)
$Pn'c'2$ (30.115) $\mathbb{Z}_1$	• $\boldsymbol{B} \parallel [001]$
$P2'$ (3.3) $\mathbb{Z}_1$	• $\boldsymbol{B} \parallel [100]$ and strain $\perp [001]$<br>• $\boldsymbol{B} \parallel [010]$ and strain $\perp [001]$<br>• $\boldsymbol{B} \perp [001]$
$Pn'n2'$ (34.158) $\mathbb{Z}_1$	• $\boldsymbol{B} \parallel [100]$
P_a2 (3.4) $\mathbb{Z}_2$	• strain $\perp [001]$
$Pc'c2'$ (27.80) $\mathbb{Z}_1$	• $\boldsymbol{B} \parallel [010]$

Table. S 232: Subgroups of P_Inc2 (30.122) that can be realized by applying $\boldsymbol{E}/\boldsymbol{B}$ fields and/or uniaxial strains.

Subgroup & SI group	Perturbation
Pc (7.24) $\mathbb{Z}_1$	• $\boldsymbol{B} \parallel [010]$ and strain $\perp [010]$<br>• ($\boldsymbol{E} \parallel [100]$ or $\boldsymbol{E} \perp [010]$) and $\boldsymbol{B} \parallel [010]$
P_S1 (1.3) $\mathbb{Z}_1$	• strain in generic direction<br>• ($\boldsymbol{E} \parallel [100]$ or $\boldsymbol{E} \perp [010]$) and strain $\perp [100]$<br>• ($\boldsymbol{E} \parallel [100]$ or $\boldsymbol{E} \perp [010]$) and strain $\perp [001]$<br>• ($\boldsymbol{E} \parallel [010]$ or $\boldsymbol{E} \perp [100]$) and strain $\perp [010]$<br>• ($\boldsymbol{E} \parallel [010]$ or $\boldsymbol{E} \perp [100]$) and strain $\perp [001]$<br>• $\boldsymbol{E}$ in generic direction
Pc' (7.26) $\mathbb{Z}_1$	• $\boldsymbol{B} \parallel [100]$ and strain $\perp [010]$<br>• $\boldsymbol{B} \parallel [001]$ and strain $\perp [010]$<br>• $\boldsymbol{B} \perp [010]$<br>• ($\boldsymbol{E} \parallel [100]$ or $\boldsymbol{E} \perp [010]$) and $\boldsymbol{B} \parallel [100]$<br>• ($\boldsymbol{E} \parallel [100]$ or $\boldsymbol{E} \perp [010]$) and $\boldsymbol{B} \parallel [001]$
P_Cc (7.30) $\mathbb{Z}_1$	• strain $\perp [010]$<br>• ($\boldsymbol{E} \parallel [100]$ or $\boldsymbol{E} \perp [010]$)
Pc (7.24) $\mathbb{Z}_1$	• $\boldsymbol{B} \parallel [100]$ and strain $\perp [100]$<br>• ($\boldsymbol{E} \parallel [010]$ or $\boldsymbol{E} \perp [100]$) and $\boldsymbol{B} \parallel [100]$
$P2$ (3.1) $\mathbb{Z}_2$	• $\boldsymbol{B} \parallel [001]$ and strain $\perp [001]$
Pm' (6.20) $\mathbb{Z}_1$	• $\boldsymbol{B} \parallel [010]$ and strain $\perp [100]$<br>• $\boldsymbol{B} \parallel [001]$ and strain $\perp [100]$<br>• $\boldsymbol{B} \perp [100]$<br>• ($\boldsymbol{E} \parallel [010]$ or $\boldsymbol{E} \perp [100]$) and $\boldsymbol{B} \parallel [010]$<br>• ($\boldsymbol{E} \parallel [010]$ or $\boldsymbol{E} \perp [100]$) and $\boldsymbol{B} \parallel [001]$
P_Ac (7.31) $\mathbb{Z}_1$	• strain $\perp [100]$<br>• ($\boldsymbol{E} \parallel [010]$ or $\boldsymbol{E} \perp [100]$)
$Pm'a'2$ (28.91) $\mathbb{Z}_2$	• $\boldsymbol{B} \parallel [001]$
$P2_1'$ (4.9) $\mathbb{Z}_1$	• $\boldsymbol{B} \parallel [100]$ and strain $\perp [001]$<br>• $\boldsymbol{B} \parallel [010]$ and strain $\perp [001]$<br>• $\boldsymbol{B} \perp [001]$
$Pna'2_1'$ (33.147) $\mathbb{Z}_1$	• $\boldsymbol{B} \parallel [100]$
P_C2 (3.6) $\mathbb{Z}_1$	• strain $\perp [001]$
$Pm'c2_1'$ (26.68) $\mathbb{Z}_1$	• $\boldsymbol{B} \parallel [010]$

Table. S 233: Subgroups of $Pmn2_1$ (31.123) that can be realized by applying $\boldsymbol{E}/\boldsymbol{B}$ fields and/or uniaxial strains.

Subgroup & SI group	Perturbation
Pc (7.24) $\mathbb{Z}_1$	• strain $\perp$ [010]<br>• $\boldsymbol{B}$ $\parallel$ [010]<br>• ($\boldsymbol{E}$ $\parallel$ [100] or $\boldsymbol{E}$ $\perp$ [010])
Pm (6.18) $\mathbb{Z}_1$	• strain $\perp$ [100]<br>• $\boldsymbol{B}$ $\parallel$ [100]<br>• ($\boldsymbol{E}$ $\parallel$ [010] or $\boldsymbol{E}$ $\perp$ [100])
$P2_1$ (4.7) $\mathbb{Z}_1$	• strain $\perp$ [001]<br>• $\boldsymbol{B}$ $\parallel$ [001]

Table. S 234: Subgroups of $Pmn2_11'$ (31.124) that can be realized by applying $\boldsymbol{E}/\boldsymbol{B}$ fields and/or uniaxial strains.

Subgroup & SI group	Perturbation
$P11'$ (1.2) $\mathbb{Z}_1$	• strain in generic direction<br>• ($\boldsymbol{E}$ $\parallel$ [100] or $\boldsymbol{E}$ $\perp$ [010]) and strain $\perp$ [100]<br>• ($\boldsymbol{E}$ $\parallel$ [100] or $\boldsymbol{E}$ $\perp$ [010]) and strain $\perp$ [001]<br>• ($\boldsymbol{E}$ $\parallel$ [010] or $\boldsymbol{E}$ $\perp$ [100]) and strain $\perp$ [010]<br>• ($\boldsymbol{E}$ $\parallel$ [010] or $\boldsymbol{E}$ $\perp$ [100]) and strain $\perp$ [001]<br>• $\boldsymbol{E}$ in generic direction
Pc' (7.26) $\mathbb{Z}_1$	• $\boldsymbol{B}$ $\parallel$ [100] and strain $\perp$ [010]<br>• $\boldsymbol{B}$ $\parallel$ [001] and strain $\perp$ [010]<br>• $\boldsymbol{B}$ $\perp$ [010]<br>• ($\boldsymbol{E}$ $\parallel$ [100] or $\boldsymbol{E}$ $\perp$ [010]) and $\boldsymbol{B}$ $\parallel$ [100]<br>• ($\boldsymbol{E}$ $\parallel$ [100] or $\boldsymbol{E}$ $\perp$ [010]) and $\boldsymbol{B}$ $\parallel$ [001]
Pc (7.24) $\mathbb{Z}_1$	• $\boldsymbol{B}$ $\parallel$ [010] and strain $\perp$ [010]<br>• ($\boldsymbol{E}$ $\parallel$ [100] or $\boldsymbol{E}$ $\perp$ [010]) and $\boldsymbol{B}$ $\parallel$ [010]
$Pc1'$ (7.25) $\mathbb{Z}_1$	• strain $\perp$ [010]<br>• ($\boldsymbol{E}$ $\parallel$ [100] or $\boldsymbol{E}$ $\perp$ [010])
Pm' (6.20) $\mathbb{Z}_1$	• $\boldsymbol{B}$ $\parallel$ [010] and strain $\perp$ [100]<br>• $\boldsymbol{B}$ $\parallel$ [001] and strain $\perp$ [100]<br>• $\boldsymbol{B}$ $\perp$ [100]<br>• ($\boldsymbol{E}$ $\parallel$ [010] or $\boldsymbol{E}$ $\perp$ [100]) and $\boldsymbol{B}$ $\parallel$ [010]<br>• ($\boldsymbol{E}$ $\parallel$ [010] or $\boldsymbol{E}$ $\perp$ [100]) and $\boldsymbol{B}$ $\parallel$ [001]

Continued on next page

. . . continued

Subgroup & SI group	Perturbation
Pm (6.18) $\mathbb{Z}_1$	• $\boldsymbol{B}$ ∥ [100] and strain ⊥ [100]<br>• ($\boldsymbol{E}$ ∥ [010] or $\boldsymbol{E}$ ⊥ [100]) and $\boldsymbol{B}$ ∥ [100]
$Pm1'$ (6.19) $\mathbb{Z}_1$	• strain ⊥ [100]<br>• ($\boldsymbol{E}$ ∥ [010] or $\boldsymbol{E}$ ⊥ [100])
$P2_1'$ (4.9) $\mathbb{Z}_1$	• $\boldsymbol{B}$ ∥ [100] and strain ⊥ [001]<br>• $\boldsymbol{B}$ ∥ [010] and strain ⊥ [001]<br>• $\boldsymbol{B}$ ⊥ [001]
$Pm'n2_1'$ (31.125) $\mathbb{Z}_1$	• $\boldsymbol{B}$ ∥ [010]
$Pmn'2_1'$ (31.126) $\mathbb{Z}_1$	• $\boldsymbol{B}$ ∥ [100]
$P2_1$ (4.7) $\mathbb{Z}_1$	• $\boldsymbol{B}$ ∥ [001] and strain ⊥ [001]
$Pm'n'2_1$ (31.127) $\mathbb{Z}_1$	• $\boldsymbol{B}$ ∥ [001]
$P2_11'$ (4.8) $\mathbb{Z}_1$	• strain ⊥ [001]

Table. S 235: Subgroups of $Pm'n2_1'$ (31.125) that can be realized by applying $\boldsymbol{E}/\boldsymbol{B}$ fields and/or uniaxial strains.

Subgroup & SI group	Perturbation
Pc (7.24) $\mathbb{Z}_1$	• strain ⊥ [010]<br>• ($\boldsymbol{E}$ ∥ [100] or $\boldsymbol{E}$ ⊥ [010])
Pm' (6.20) $\mathbb{Z}_1$	• strain ⊥ [100]<br>• ($\boldsymbol{B}$ ∥ [001] or $\boldsymbol{B}$ ⊥ [100])<br>• ($\boldsymbol{E}$ ∥ [010] or $\boldsymbol{E}$ ⊥ [100])
$P2_1'$ (4.9) $\mathbb{Z}_1$	• strain ⊥ [001]<br>• ($\boldsymbol{B}$ ∥ [100] or $\boldsymbol{B}$ ⊥ [001])

Table. S 236: Subgroups of $Pmn'2_1'$ (31.126) that can be realized by applying $\boldsymbol{E}/\boldsymbol{B}$ fields and/or uniaxial strains.

Subgroup & SI group	Perturbation

Continued on next page

…continued

Subgroup & SI group	Perturbation
Pc' (7.26) $\mathbb{Z}_1$	• strain $\perp$ $[010]$<br>• ($\boldsymbol{B}$ $\parallel$ $[001]$ or $\boldsymbol{B}$ $\perp$ $[010]$)<br>• ($\boldsymbol{E}$ $\parallel$ $[100]$ or $\boldsymbol{E}$ $\perp$ $[010]$)
Pm (6.18) $\mathbb{Z}_1$	• strain $\perp$ $[100]$<br>• ($\boldsymbol{E}$ $\parallel$ $[010]$ or $\boldsymbol{E}$ $\perp$ $[100]$)
$P2_1'$ (4.9) $\mathbb{Z}_1$	• strain $\perp$ $[001]$<br>• ($\boldsymbol{B}$ $\parallel$ $[010]$ or $\boldsymbol{B}$ $\perp$ $[001]$)

Table. S 237: Subgroups of $Pm'n'2_1$ (31.127) that can be realized by applying $\boldsymbol{E}/\boldsymbol{B}$ fields and/or uniaxial strains.

Subgroup & SI group	Perturbation
Pc' (7.26) $\mathbb{Z}_1$	• strain $\perp$ $[010]$<br>• ($\boldsymbol{B}$ $\parallel$ $[100]$ or $\boldsymbol{B}$ $\perp$ $[010]$)<br>• ($\boldsymbol{E}$ $\parallel$ $[100]$ or $\boldsymbol{E}$ $\perp$ $[010]$)
Pm' (6.20) $\mathbb{Z}_1$	• strain $\perp$ $[100]$<br>• ($\boldsymbol{B}$ $\parallel$ $[010]$ or $\boldsymbol{B}$ $\perp$ $[100]$)<br>• ($\boldsymbol{E}$ $\parallel$ $[010]$ or $\boldsymbol{E}$ $\perp$ $[100]$)
$P2_1$ (4.7) $\mathbb{Z}_1$	• strain $\perp$ $[001]$

Table. S 238: Subgroups of P_amn2_1 (31.128) that can be realized by applying $\boldsymbol{E}/\boldsymbol{B}$ fields and/or uniaxial strains.

Subgroup & SI group	Perturbation
Pc' (7.26) $\mathbb{Z}_1$	• $\boldsymbol{B}$ $\parallel$ $[100]$ and strain $\perp$ $[010]$<br>• $\boldsymbol{B}$ $\parallel$ $[001]$ and strain $\perp$ $[010]$<br>• $\boldsymbol{B}$ $\perp$ $[010]$<br>• ($\boldsymbol{E}$ $\parallel$ $[100]$ or $\boldsymbol{E}$ $\perp$ $[010]$) and $\boldsymbol{B}$ $\parallel$ $[100]$<br>• ($\boldsymbol{E}$ $\parallel$ $[100]$ or $\boldsymbol{E}$ $\perp$ $[010]$) and $\boldsymbol{B}$ $\parallel$ $[001]$

Continued on next page

...continued

Subgroup & SI group	Perturbation
P_S1 (1.3) $\mathbb{Z}_1$	• strain in generic direction<br>• ($\boldsymbol{E} \parallel [100]$ or $\boldsymbol{E} \perp [010]$) and strain $\perp [100]$<br>• ($\boldsymbol{E} \parallel [100]$ or $\boldsymbol{E} \perp [010]$) and strain $\perp [001]$<br>• ($\boldsymbol{E} \parallel [010]$ or $\boldsymbol{E} \perp [100]$) and strain $\perp [010]$<br>• ($\boldsymbol{E} \parallel [010]$ or $\boldsymbol{E} \perp [100]$) and strain $\perp [001]$<br>• $\boldsymbol{E}$ in generic direction
Pc (7.24) $\mathbb{Z}_1$	• $\boldsymbol{B} \parallel [010]$ and strain $\perp [010]$<br>• ($\boldsymbol{E} \parallel [100]$ or $\boldsymbol{E} \perp [010]$) and $\boldsymbol{B} \parallel [010]$
P_ac (7.27) $\mathbb{Z}_1$	• strain $\perp [010]$<br>• ($\boldsymbol{E} \parallel [100]$ or $\boldsymbol{E} \perp [010]$)
Pm (6.18) $\mathbb{Z}_1$	• $\boldsymbol{B} \parallel [100]$ and strain $\perp [100]$<br>• ($\boldsymbol{E} \parallel [010]$ or $\boldsymbol{E} \perp [100]$) and $\boldsymbol{B} \parallel [100]$
$P2_1'$ (4.9) $\mathbb{Z}_1$	• $\boldsymbol{B} \parallel [100]$ and strain $\perp [001]$<br>• $\boldsymbol{B} \parallel [010]$ and strain $\perp [001]$<br>• $\boldsymbol{B} \perp [001]$
$Pmc'2_1'$ (26.69) $\mathbb{Z}_1$	• $\boldsymbol{B} \parallel [100]$
Pm' (6.20) $\mathbb{Z}_1$	• $\boldsymbol{B} \parallel [010]$ and strain $\perp [100]$<br>• $\boldsymbol{B} \parallel [001]$ and strain $\perp [100]$<br>• $\boldsymbol{B} \perp [100]$<br>• ($\boldsymbol{E} \parallel [010]$ or $\boldsymbol{E} \perp [100]$) and $\boldsymbol{B} \parallel [010]$<br>• ($\boldsymbol{E} \parallel [010]$ or $\boldsymbol{E} \perp [100]$) and $\boldsymbol{B} \parallel [001]$
P_bm (6.22) $\mathbb{Z}_1$	• strain $\perp [100]$<br>• ($\boldsymbol{E} \parallel [010]$ or $\boldsymbol{E} \perp [100]$)
$Pm'n2_1'$ (31.125) $\mathbb{Z}_1$	• $\boldsymbol{B} \parallel [010]$
$P2_1$ (4.7) $\mathbb{Z}_1$	• $\boldsymbol{B} \parallel [001]$ and strain $\perp [001]$
P_a2_1 (4.10) $\mathbb{Z}_1$	• strain $\perp [001]$
$Pm'c'2_1$ (26.70) $\mathbb{Z}_1$	• $\boldsymbol{B} \parallel [001]$

Table. S 239: Subgroups of P_bmn2_1 (31.129) that can be realized by applying $\boldsymbol{E}/\boldsymbol{B}$ fields and/or uniaxial strains.

Subgroup & SI group	Perturbation

Continued on next page

...continued

Subgroup & SI group	Perturbation
P_S1 (1.3) $\mathbb{Z}_1$	• strain in generic direction<br>• ($\boldsymbol{E} \parallel [100]$ or $\boldsymbol{E} \perp [010]$) and strain $\perp [100]$<br>• ($\boldsymbol{E} \parallel [100]$ or $\boldsymbol{E} \perp [010]$) and strain $\perp [001]$<br>• ($\boldsymbol{E} \parallel [010]$ or $\boldsymbol{E} \perp [100]$) and strain $\perp [010]$<br>• ($\boldsymbol{E} \parallel [010]$ or $\boldsymbol{E} \perp [100]$) and strain $\perp [001]$<br>• $\boldsymbol{E}$ in generic direction
Pc (7.24) $\mathbb{Z}_1$	• $\boldsymbol{B} \parallel [010]$ and strain $\perp [010]$<br>• ($\boldsymbol{E} \parallel [100]$ or $\boldsymbol{E} \perp [010]$) and $\boldsymbol{B} \parallel [010]$
Pc' (7.26) $\mathbb{Z}_1$	• $\boldsymbol{B} \parallel [100]$ and strain $\perp [010]$<br>• $\boldsymbol{B} \parallel [001]$ and strain $\perp [010]$<br>• $\boldsymbol{B} \perp [010]$<br>• ($\boldsymbol{E} \parallel [100]$ or $\boldsymbol{E} \perp [010]$) and $\boldsymbol{B} \parallel [100]$<br>• ($\boldsymbol{E} \parallel [100]$ or $\boldsymbol{E} \perp [010]$) and $\boldsymbol{B} \parallel [001]$
P_bc (7.29) $\mathbb{Z}_1$	• strain $\perp [010]$<br>• ($\boldsymbol{E} \parallel [100]$ or $\boldsymbol{E} \perp [010]$)
Pm (6.18) $\mathbb{Z}_1$	• $\boldsymbol{B} \parallel [100]$ and strain $\perp [100]$<br>• ($\boldsymbol{E} \parallel [010]$ or $\boldsymbol{E} \perp [100]$) and $\boldsymbol{B} \parallel [100]$
Pc' (7.26) $\mathbb{Z}_1$	• $\boldsymbol{B} \parallel [010]$ and strain $\perp [100]$<br>• $\boldsymbol{B} \parallel [001]$ and strain $\perp [100]$<br>• $\boldsymbol{B} \perp [100]$<br>• ($\boldsymbol{E} \parallel [010]$ or $\boldsymbol{E} \perp [100]$) and $\boldsymbol{B} \parallel [010]$<br>• ($\boldsymbol{E} \parallel [010]$ or $\boldsymbol{E} \perp [100]$) and $\boldsymbol{B} \parallel [001]$
P_am (6.21) $\mathbb{Z}_1$	• strain $\perp [100]$<br>• ($\boldsymbol{E} \parallel [010]$ or $\boldsymbol{E} \perp [100]$)
$P2_1$ (4.7) $\mathbb{Z}_1$	• $\boldsymbol{B} \parallel [001]$ and strain $\perp [001]$
$Pn'a'2_1$ (33.148) $\mathbb{Z}_1$	• $\boldsymbol{B} \parallel [001]$
$P2_1'$ (4.9) $\mathbb{Z}_1$	• $\boldsymbol{B} \parallel [100]$ and strain $\perp [001]$<br>• $\boldsymbol{B} \parallel [010]$ and strain $\perp [001]$<br>• $\boldsymbol{B} \perp [001]$
$Pmn'2_1'$ (31.126) $\mathbb{Z}_1$	• $\boldsymbol{B} \parallel [100]$
$Pna'2_1'$ (33.147) $\mathbb{Z}_1$	• $\boldsymbol{B} \parallel [010]$
P_a2_1 (4.10) $\mathbb{Z}_1$	• strain $\perp [001]$

Table. S 240: Subgroups of P_cmn2_1 (31.130) that can be realized by applying $\boldsymbol{E}/\boldsymbol{B}$ fields and/or uniaxial strains.

Subgroup & SI group	Perturbation
P_S1 (1.3) $\mathbb{Z}_1$	• strain in generic direction<br>• ($\boldsymbol{E} \parallel [100]$ or $\boldsymbol{E} \perp [010]$) and strain $\perp [100]$<br>• ($\boldsymbol{E} \parallel [100]$ or $\boldsymbol{E} \perp [010]$) and strain $\perp [001]$<br>• ($\boldsymbol{E} \parallel [010]$ or $\boldsymbol{E} \perp [100]$) and strain $\perp [010]$<br>• ($\boldsymbol{E} \parallel [010]$ or $\boldsymbol{E} \perp [100]$) and strain $\perp [001]$<br>• $\boldsymbol{E}$ in generic direction
Pc' (7.26) $\mathbb{Z}_1$	• $\boldsymbol{B} \parallel [100]$ and strain $\perp [010]$<br>• $\boldsymbol{B} \parallel [001]$ and strain $\perp [010]$<br>• $\boldsymbol{B} \perp [010]$<br>• ($\boldsymbol{E} \parallel [100]$ or $\boldsymbol{E} \perp [010]$) and $\boldsymbol{B} \parallel [100]$<br>• ($\boldsymbol{E} \parallel [100]$ or $\boldsymbol{E} \perp [010]$) and $\boldsymbol{B} \parallel [001]$
Pc (7.24) $\mathbb{Z}_1$	• $\boldsymbol{B} \parallel [010]$ and strain $\perp [010]$<br>• ($\boldsymbol{E} \parallel [100]$ or $\boldsymbol{E} \perp [010]$) and $\boldsymbol{B} \parallel [010]$
P_ac (7.27) $\mathbb{Z}_1$	• strain $\perp [010]$<br>• ($\boldsymbol{E} \parallel [100]$ or $\boldsymbol{E} \perp [010]$)
Pm (6.18) $\mathbb{Z}_1$	• $\boldsymbol{B} \parallel [100]$ and strain $\perp [100]$<br>• ($\boldsymbol{E} \parallel [010]$ or $\boldsymbol{E} \perp [100]$) and $\boldsymbol{B} \parallel [100]$
Pc' (7.26) $\mathbb{Z}_1$	• $\boldsymbol{B} \parallel [010]$ and strain $\perp [100]$<br>• $\boldsymbol{B} \parallel [001]$ and strain $\perp [100]$<br>• $\boldsymbol{B} \perp [100]$<br>• ($\boldsymbol{E} \parallel [010]$ or $\boldsymbol{E} \perp [100]$) and $\boldsymbol{B} \parallel [010]$<br>• ($\boldsymbol{E} \parallel [010]$ or $\boldsymbol{E} \perp [100]$) and $\boldsymbol{B} \parallel [001]$
P_am (6.21) $\mathbb{Z}_1$	• strain $\perp [100]$<br>• ($\boldsymbol{E} \parallel [010]$ or $\boldsymbol{E} \perp [100]$)
$P2'$ (3.3) $\mathbb{Z}_1$	• $\boldsymbol{B} \parallel [100]$ and strain $\perp [001]$<br>• $\boldsymbol{B} \parallel [010]$ and strain $\perp [001]$<br>• $\boldsymbol{B} \perp [001]$
$Pma'2'$ (28.90) $\mathbb{Z}_1$	• $\boldsymbol{B} \parallel [100]$
$Pnc'2'$ (30.114) $\mathbb{Z}_1$	• $\boldsymbol{B} \parallel [010]$
$P2_1$ (4.7) $\mathbb{Z}_1$	• $\boldsymbol{B} \parallel [001]$ and strain $\perp [001]$
$Pc'a'2_1$ (29.103) $\mathbb{Z}_1$	• $\boldsymbol{B} \parallel [001]$
P_b2_1 (4.11) $\mathbb{Z}_1$	• strain $\perp [001]$

Table. S 241: Subgroups of $P_{A}mn2_1$ (31.131) that can be realized by applying $\boldsymbol{E}/\boldsymbol{B}$ fields and/or uniaxial strains.

Subgroup & SI group	Perturbation
P_S1 (1.3) $\mathbb{Z}_1$	• strain in generic direction<br>• ($\boldsymbol{E} \parallel [100]$ or $\boldsymbol{E} \perp [010]$) and strain $\perp [100]$<br>• ($\boldsymbol{E} \parallel [100]$ or $\boldsymbol{E} \perp [010]$) and strain $\perp [001]$<br>• ($\boldsymbol{E} \parallel [010]$ or $\boldsymbol{E} \perp [100]$) and strain $\perp [010]$<br>• ($\boldsymbol{E} \parallel [010]$ or $\boldsymbol{E} \perp [100]$) and strain $\perp [001]$<br>• $\boldsymbol{E}$ in generic direction
Pc (7.24) $\mathbb{Z}_1$	• $\boldsymbol{B} \parallel [010]$ and strain $\perp [010]$<br>• ($\boldsymbol{E} \parallel [100]$ or $\boldsymbol{E} \perp [010]$) and $\boldsymbol{B} \parallel [010]$
Pc' (7.26) $\mathbb{Z}_1$	• $\boldsymbol{B} \parallel [100]$ and strain $\perp [010]$<br>• $\boldsymbol{B} \parallel [001]$ and strain $\perp [010]$<br>• $\boldsymbol{B} \perp [010]$<br>• ($\boldsymbol{E} \parallel [100]$ or $\boldsymbol{E} \perp [010]$) and $\boldsymbol{B} \parallel [100]$<br>• ($\boldsymbol{E} \parallel [100]$ or $\boldsymbol{E} \perp [010]$) and $\boldsymbol{B} \parallel [001]$
P_Cc (7.30) $\mathbb{Z}_1$	• strain $\perp [010]$<br>• ($\boldsymbol{E} \parallel [100]$ or $\boldsymbol{E} \perp [010]$)
Pm (6.18) $\mathbb{Z}_1$	• $\boldsymbol{B} \parallel [100]$ and strain $\perp [100]$<br>• ($\boldsymbol{E} \parallel [010]$ or $\boldsymbol{E} \perp [100]$) and $\boldsymbol{B} \parallel [100]$
Pc' (7.26) $\mathbb{Z}_1$	• $\boldsymbol{B} \parallel [010]$ and strain $\perp [100]$<br>• $\boldsymbol{B} \parallel [001]$ and strain $\perp [100]$<br>• $\boldsymbol{B} \perp [100]$<br>• ($\boldsymbol{E} \parallel [010]$ or $\boldsymbol{E} \perp [100]$) and $\boldsymbol{B} \parallel [010]$<br>• ($\boldsymbol{E} \parallel [010]$ or $\boldsymbol{E} \perp [100]$) and $\boldsymbol{B} \parallel [001]$
P_am (6.21) $\mathbb{Z}_1$	• strain $\perp [100]$<br>• ($\boldsymbol{E} \parallel [010]$ or $\boldsymbol{E} \perp [100]$)
$P2_1$ (4.7) $\mathbb{Z}_1$	• $\boldsymbol{B} \parallel [001]$ and strain $\perp [001]$
$Pn'a'2_1$ (33.148) $\mathbb{Z}_1$	• $\boldsymbol{B} \parallel [001]$
$P2'$ (3.3) $\mathbb{Z}_1$	• $\boldsymbol{B} \parallel [100]$ and strain $\perp [001]$<br>• $\boldsymbol{B} \parallel [010]$ and strain $\perp [001]$<br>• $\boldsymbol{B} \perp [001]$
$Pma'2'$ (28.90) $\mathbb{Z}_1$	• $\boldsymbol{B} \parallel [100]$
$Pn'n2'$ (34.158) $\mathbb{Z}_1$	• $\boldsymbol{B} \parallel [010]$
P_C2_1 (4.12) $\mathbb{Z}_1$	• strain $\perp [001]$

Table. S 242: Subgroups of P_Bmn2_1 (31.132) that can be realized by applying $\boldsymbol{E}/\boldsymbol{B}$ fields and/or uniaxial strains.

Subgroup & SI group	Perturbation
Pm' (6.20) $\mathbb{Z}_1$	• $\boldsymbol{B} \parallel [100]$ and strain $\perp [010]$<br>• $\boldsymbol{B} \parallel [001]$ and strain $\perp [010]$<br>• $\boldsymbol{B} \perp [010]$<br>• ($\boldsymbol{E} \parallel [100]$ or $\boldsymbol{E} \perp [010]$) and $\boldsymbol{B} \parallel [100]$<br>• ($\boldsymbol{E} \parallel [100]$ or $\boldsymbol{E} \perp [010]$) and $\boldsymbol{B} \parallel [001]$
P_S1 (1.3) $\mathbb{Z}_1$	• strain in generic direction<br>• ($\boldsymbol{E} \parallel [100]$ or $\boldsymbol{E} \perp [010]$) and strain $\perp [100]$<br>• ($\boldsymbol{E} \parallel [100]$ or $\boldsymbol{E} \perp [010]$) and strain $\perp [001]$<br>• ($\boldsymbol{E} \parallel [010]$ or $\boldsymbol{E} \perp [100]$) and strain $\perp [010]$<br>• ($\boldsymbol{E} \parallel [010]$ or $\boldsymbol{E} \perp [100]$) and strain $\perp [001]$<br>• $\boldsymbol{E}$ in generic direction
Pc (7.24) $\mathbb{Z}_1$	• $\boldsymbol{B} \parallel [010]$ and strain $\perp [010]$<br>• ($\boldsymbol{E} \parallel [100]$ or $\boldsymbol{E} \perp [010]$) and $\boldsymbol{B} \parallel [010]$
P_cc (7.28) $\mathbb{Z}_1$	• strain $\perp [010]$<br>• ($\boldsymbol{E} \parallel [100]$ or $\boldsymbol{E} \perp [010]$)
Pm (6.18) $\mathbb{Z}_1$	• $\boldsymbol{B} \parallel [100]$ and strain $\perp [100]$<br>• ($\boldsymbol{E} \parallel [010]$ or $\boldsymbol{E} \perp [100]$) and $\boldsymbol{B} \parallel [100]$
$P2'$ (3.3) $\mathbb{Z}_1$	• $\boldsymbol{B} \parallel [100]$ and strain $\perp [001]$<br>• $\boldsymbol{B} \parallel [010]$ and strain $\perp [001]$<br>• $\boldsymbol{B} \perp [001]$
$Pm'm2'$ (25.59) $\mathbb{Z}_1$	• $\boldsymbol{B} \parallel [100]$
Pc' (7.26) $\mathbb{Z}_1$	• $\boldsymbol{B} \parallel [010]$ and strain $\perp [100]$<br>• $\boldsymbol{B} \parallel [001]$ and strain $\perp [100]$<br>• $\boldsymbol{B} \perp [100]$<br>• ($\boldsymbol{E} \parallel [010]$ or $\boldsymbol{E} \perp [100]$) and $\boldsymbol{B} \parallel [010]$<br>• ($\boldsymbol{E} \parallel [010]$ or $\boldsymbol{E} \perp [100]$) and $\boldsymbol{B} \parallel [001]$
P_Cm (6.23) $\mathbb{Z}_1$	• strain $\perp [100]$<br>• ($\boldsymbol{E} \parallel [010]$ or $\boldsymbol{E} \perp [100]$)
$Pnc'2'$ (30.114) $\mathbb{Z}_1$	• $\boldsymbol{B} \parallel [010]$
$P2_1$ (4.7) $\mathbb{Z}_1$	• $\boldsymbol{B} \parallel [001]$ and strain $\perp [001]$
P_C2_1 (4.12) $\mathbb{Z}_1$	• strain $\perp [001]$
$Pm'c'2_1$ (26.70) $\mathbb{Z}_1$	• $\boldsymbol{B} \parallel [001]$

Table. S 243: Subgroups of $P_{C}mn2_1$ (31.133) that can be realized by applying $\boldsymbol{E}/\boldsymbol{B}$ fields and/or uniaxial strains.

Subgroup & SI group	Perturbation
Pc' (7.26) $\mathbb{Z}_1$	• $\boldsymbol{B}$ ∥ [100] and strain ⊥ [010]<br>• $\boldsymbol{B}$ ∥ [001] and strain ⊥ [010]<br>• $\boldsymbol{B}$ ⊥ [010]<br>• ($\boldsymbol{E}$ ∥ [100] or $\boldsymbol{E}$ ⊥ [010]) and $\boldsymbol{B}$ ∥ [100]<br>• ($\boldsymbol{E}$ ∥ [100] or $\boldsymbol{E}$ ⊥ [010]) and $\boldsymbol{B}$ ∥ [001]
$P_{S}1$ (1.3) $\mathbb{Z}_1$	• strain in generic direction<br>• ($\boldsymbol{E}$ ∥ [100] or $\boldsymbol{E}$ ⊥ [010]) and strain ⊥ [100]<br>• ($\boldsymbol{E}$ ∥ [100] or $\boldsymbol{E}$ ⊥ [010]) and strain ⊥ [001]<br>• ($\boldsymbol{E}$ ∥ [010] or $\boldsymbol{E}$ ⊥ [100]) and strain ⊥ [010]<br>• ($\boldsymbol{E}$ ∥ [010] or $\boldsymbol{E}$ ⊥ [100]) and strain ⊥ [001]<br>• $\boldsymbol{E}$ in generic direction
Pc (7.24) $\mathbb{Z}_1$	• $\boldsymbol{B}$ ∥ [010] and strain ⊥ [010]<br>• ($\boldsymbol{E}$ ∥ [100] or $\boldsymbol{E}$ ⊥ [010]) and $\boldsymbol{B}$ ∥ [010]
$P_{C}c$ (7.30) $\mathbb{Z}_1$	• strain ⊥ [010]<br>• ($\boldsymbol{E}$ ∥ [100] or $\boldsymbol{E}$ ⊥ [010])
Pm (6.18) $\mathbb{Z}_1$	• $\boldsymbol{B}$ ∥ [100] and strain ⊥ [100]<br>• ($\boldsymbol{E}$ ∥ [010] or $\boldsymbol{E}$ ⊥ [100]) and $\boldsymbol{B}$ ∥ [100]
$P2_1'$ (4.9) $\mathbb{Z}_1$	• $\boldsymbol{B}$ ∥ [100] and strain ⊥ [001]<br>• $\boldsymbol{B}$ ∥ [010] and strain ⊥ [001]<br>• $\boldsymbol{B}$ ⊥ [001]
$Pmc'2_1'$ (26.69) $\mathbb{Z}_1$	• $\boldsymbol{B}$ ∥ [100]
Pc' (7.26) $\mathbb{Z}_1$	• $\boldsymbol{B}$ ∥ [010] and strain ⊥ [100]<br>• $\boldsymbol{B}$ ∥ [001] and strain ⊥ [100]<br>• $\boldsymbol{B}$ ⊥ [100]<br>• ($\boldsymbol{E}$ ∥ [010] or $\boldsymbol{E}$ ⊥ [100]) and $\boldsymbol{B}$ ∥ [010]<br>• ($\boldsymbol{E}$ ∥ [010] or $\boldsymbol{E}$ ⊥ [100]) and $\boldsymbol{B}$ ∥ [001]
$P_{C}m$ (6.23) $\mathbb{Z}_1$	• strain ⊥ [100]<br>• ($\boldsymbol{E}$ ∥ [010] or $\boldsymbol{E}$ ⊥ [100])
$Pna'2_1'$ (33.147) $\mathbb{Z}_1$	• $\boldsymbol{B}$ ∥ [010]
$P2_1$ (4.7) $\mathbb{Z}_1$	• $\boldsymbol{B}$ ∥ [001] and strain ⊥ [001]
$P_{a}2_1$ (4.10) $\mathbb{Z}_1$	• strain ⊥ [001]
$Pc'a'2_1$ (29.103) $\mathbb{Z}_1$	• $\boldsymbol{B}$ ∥ [001]

Table. S 244: Subgroups of P_Imn2_1 (31.134) that can be realized by applying $\boldsymbol{E}/\boldsymbol{B}$ fields and/or uniaxial strains.

Subgroup & SI group	Perturbation
Pm' (6.20) $\mathbb{Z}_1$	• $\boldsymbol{B} \parallel [100]$ and strain $\perp [010]$<br>• $\boldsymbol{B} \parallel [001]$ and strain $\perp [010]$<br>• $\boldsymbol{B} \perp [010]$<br>• ($\boldsymbol{E} \parallel [100]$ or $\boldsymbol{E} \perp [010]$) and $\boldsymbol{B} \parallel [100]$<br>• ($\boldsymbol{E} \parallel [100]$ or $\boldsymbol{E} \perp [010]$) and $\boldsymbol{B} \parallel [001]$
P_S1 (1.3) $\mathbb{Z}_1$	• strain in generic direction<br>• ($\boldsymbol{E} \parallel [100]$ or $\boldsymbol{E} \perp [010]$) and strain $\perp [100]$<br>• ($\boldsymbol{E} \parallel [100]$ or $\boldsymbol{E} \perp [010]$) and strain $\perp [001]$<br>• ($\boldsymbol{E} \parallel [010]$ or $\boldsymbol{E} \perp [100]$) and strain $\perp [010]$<br>• ($\boldsymbol{E} \parallel [010]$ or $\boldsymbol{E} \perp [100]$) and strain $\perp [001]$<br>• $\boldsymbol{E}$ in generic direction
Pc (7.24) $\mathbb{Z}_1$	• $\boldsymbol{B} \parallel [010]$ and strain $\perp [010]$<br>• ($\boldsymbol{E} \parallel [100]$ or $\boldsymbol{E} \perp [010]$) and $\boldsymbol{B} \parallel [010]$
P_Ac (7.31) $\mathbb{Z}_1$	• strain $\perp [010]$<br>• ($\boldsymbol{E} \parallel [100]$ or $\boldsymbol{E} \perp [010]$)
Pm (6.18) $\mathbb{Z}_1$	• $\boldsymbol{B} \parallel [100]$ and strain $\perp [100]$<br>• ($\boldsymbol{E} \parallel [010]$ or $\boldsymbol{E} \perp [100]$) and $\boldsymbol{B} \parallel [100]$
$P2'$ (3.3) $\mathbb{Z}_1$	• $\boldsymbol{B} \parallel [100]$ and strain $\perp [001]$<br>• $\boldsymbol{B} \parallel [010]$ and strain $\perp [001]$<br>• $\boldsymbol{B} \perp [001]$
$Pm'm2'$ (25.59) $\mathbb{Z}_1$	• $\boldsymbol{B} \parallel [100]$
Pc' (7.26) $\mathbb{Z}_1$	• $\boldsymbol{B} \parallel [010]$ and strain $\perp [100]$<br>• $\boldsymbol{B} \parallel [001]$ and strain $\perp [100]$<br>• $\boldsymbol{B} \perp [100]$<br>• ($\boldsymbol{E} \parallel [010]$ or $\boldsymbol{E} \perp [100]$) and $\boldsymbol{B} \parallel [010]$<br>• ($\boldsymbol{E} \parallel [010]$ or $\boldsymbol{E} \perp [100]$) and $\boldsymbol{B} \parallel [001]$
P_Cm (6.23) $\mathbb{Z}_1$	• strain $\perp [100]$<br>• ($\boldsymbol{E} \parallel [010]$ or $\boldsymbol{E} \perp [100]$)
$Pn'n2'$ (34.158) $\mathbb{Z}_1$	• $\boldsymbol{B} \parallel [010]$
$P2_1$ (4.7) $\mathbb{Z}_1$	• $\boldsymbol{B} \parallel [001]$ and strain $\perp [001]$
P_C2_1 (4.12) $\mathbb{Z}_1$	• strain $\perp [001]$
$Pm'n'2_1$ (31.127) $\mathbb{Z}_1$	• $\boldsymbol{B} \parallel [001]$

Table. S 245: Subgroups of $Pba2$ (32.135) that can be realized by applying $\boldsymbol{E}/\boldsymbol{B}$ fields and/or uniaxial strains.

Subgroup & SI group	Perturbation
Pc (7.24) $\mathbb{Z}_1$	• strain $\perp$ [010]<br>• $\boldsymbol{B} \parallel$ [010]<br>• ($\boldsymbol{E} \parallel$ [100] or $\boldsymbol{E} \perp$ [010])
$P2$ (3.1) $\mathbb{Z}_2$	• strain $\perp$ [001]<br>• $\boldsymbol{B} \parallel$ [001]
Pc (7.24) $\mathbb{Z}_1$	• strain $\perp$ [100]<br>• $\boldsymbol{B} \parallel$ [100]<br>• ($\boldsymbol{E} \parallel$ [010] or $\boldsymbol{E} \perp$ [100])

Table. S 246: Subgroups of $Pba21'$ (32.136) that can be realized by applying $\boldsymbol{E}/\boldsymbol{B}$ fields and/or uniaxial strains.

Subgroup & SI group	Perturbation
$P11'$ (1.2) $\mathbb{Z}_1$	• strain in generic direction<br>• ($\boldsymbol{E} \parallel$ [100] or $\boldsymbol{E} \perp$ [010]) and strain $\perp$ [100]<br>• ($\boldsymbol{E} \parallel$ [100] or $\boldsymbol{E} \perp$ [010]) and strain $\perp$ [001]<br>• ($\boldsymbol{E} \parallel$ [010] or $\boldsymbol{E} \perp$ [100]) and strain $\perp$ [010]<br>• ($\boldsymbol{E} \parallel$ [010] or $\boldsymbol{E} \perp$ [100]) and strain $\perp$ [001]<br>• $\boldsymbol{E}$ in generic direction
Pc' (7.26) $\mathbb{Z}_1$	• $\boldsymbol{B} \parallel$ [100] and strain $\perp$ [010]<br>• $\boldsymbol{B} \parallel$ [001] and strain $\perp$ [010]<br>• $\boldsymbol{B} \perp$ [010]<br>• ($\boldsymbol{E} \parallel$ [100] or $\boldsymbol{E} \perp$ [010]) and $\boldsymbol{B} \parallel$ [100]<br>• ($\boldsymbol{E} \parallel$ [100] or $\boldsymbol{E} \perp$ [010]) and $\boldsymbol{B} \parallel$ [001]
Pc (7.24) $\mathbb{Z}_1$	• $\boldsymbol{B} \parallel$ [010] and strain $\perp$ [010]<br>• ($\boldsymbol{E} \parallel$ [100] or $\boldsymbol{E} \perp$ [010]) and $\boldsymbol{B} \parallel$ [010]
$Pc1'$ (7.25) $\mathbb{Z}_1$	• strain $\perp$ [010]<br>• ($\boldsymbol{E} \parallel$ [100] or $\boldsymbol{E} \perp$ [010])
$P2'$ (3.3) $\mathbb{Z}_1$	• $\boldsymbol{B} \parallel$ [100] and strain $\perp$ [001]<br>• $\boldsymbol{B} \parallel$ [010] and strain $\perp$ [001]<br>• $\boldsymbol{B} \perp$ [001]
$P2$ (3.1) $\mathbb{Z}_2$	• $\boldsymbol{B} \parallel$ [001] and strain $\perp$ [001]

Continued on next page

...continued

Subgroup & SI group	Perturbation
$P21'$ (3.2) $\mathbb{Z}_2$	• strain $\perp$ [001]
Pc' (7.26) $\mathbb{Z}_1$	• $\boldsymbol{B}$ $\parallel$ [010] and strain $\perp$ [100]<br>• $\boldsymbol{B}$ $\parallel$ [001] and strain $\perp$ [100]<br>• $\boldsymbol{B}$ $\perp$ [100]<br>• ($\boldsymbol{E}$ $\parallel$ [010] or $\boldsymbol{E}$ $\perp$ [100]) and $\boldsymbol{B}$ $\parallel$ [010]<br>• ($\boldsymbol{E}$ $\parallel$ [010] or $\boldsymbol{E}$ $\perp$ [100]) and $\boldsymbol{B}$ $\parallel$ [001]
$Pb'a2'$ (32.137) $\mathbb{Z}_1$	• $\boldsymbol{B}$ $\parallel$ [010]
$Pb'a'2$ (32.138) $\mathbb{Z}_2$	• $\boldsymbol{B}$ $\parallel$ [001]
Pc (7.24) $\mathbb{Z}_1$	• $\boldsymbol{B}$ $\parallel$ [100] and strain $\perp$ [100]<br>• ($\boldsymbol{E}$ $\parallel$ [010] or $\boldsymbol{E}$ $\perp$ [100]) and $\boldsymbol{B}$ $\parallel$ [100]
$Pb'a2'$ (32.137) $\mathbb{Z}_1$	• $\boldsymbol{B}$ $\parallel$ [100]
$Pc1'$ (7.25) $\mathbb{Z}_1$	• strain $\perp$ [100]<br>• ($\boldsymbol{E}$ $\parallel$ [010] or $\boldsymbol{E}$ $\perp$ [100])

Table. S 247: Subgroups of $Pb'a2'$ (32.137) that can be realized by applying $\boldsymbol{E}/\boldsymbol{B}$ fields and/or uniaxial strains.

Subgroup & SI group	Perturbation
Pc (7.24) $\mathbb{Z}_1$	• strain $\perp$ [010]<br>• ($\boldsymbol{E}$ $\parallel$ [100] or $\boldsymbol{E}$ $\perp$ [010])
$P2'$ (3.3) $\mathbb{Z}_1$	• strain $\perp$ [001]<br>• ($\boldsymbol{B}$ $\parallel$ [100] or $\boldsymbol{B}$ $\perp$ [001])
Pc' (7.26) $\mathbb{Z}_1$	• strain $\perp$ [100]<br>• ($\boldsymbol{B}$ $\parallel$ [001] or $\boldsymbol{B}$ $\perp$ [100])<br>• ($\boldsymbol{E}$ $\parallel$ [010] or $\boldsymbol{E}$ $\perp$ [100])

Table. S 248: Subgroups of $Pb'a'2$ (32.138) that can be realized by applying $\boldsymbol{E}/\boldsymbol{B}$ fields and/or uniaxial strains.

Subgroup & SI group	Perturbation

Continued on next page

...continued

Subgroup & SI group	Perturbation
Pc' (7.26) $\mathbb{Z}_1$	• strain $\perp$ [010]<br>• ($\boldsymbol{B} \parallel$ [100] or $\boldsymbol{B} \perp$ [010])<br>• ($\boldsymbol{E} \parallel$ [100] or $\boldsymbol{E} \perp$ [010])
$P2$ (3.1) $\mathbb{Z}_2$	• strain $\perp$ [001]
Pc' (7.26) $\mathbb{Z}_1$	• strain $\perp$ [100]<br>• ($\boldsymbol{B} \parallel$ [010] or $\boldsymbol{B} \perp$ [100])<br>• ($\boldsymbol{E} \parallel$ [010] or $\boldsymbol{E} \perp$ [100])

Table. S 249: Subgroups of P_cba2 (32.139) that can be realized by applying $\boldsymbol{E}/\boldsymbol{B}$ fields and/or uniaxial strains.

Subgroup & SI group	Perturbation
P_S1 (1.3) $\mathbb{Z}_1$	• strain in generic direction<br>• ($\boldsymbol{E} \parallel$ [100] or $\boldsymbol{E} \perp$ [010]) and strain $\perp$ [100]<br>• ($\boldsymbol{E} \parallel$ [100] or $\boldsymbol{E} \perp$ [010]) and strain $\perp$ [001]<br>• ($\boldsymbol{E} \parallel$ [010] or $\boldsymbol{E} \perp$ [100]) and strain $\perp$ [010]<br>• ($\boldsymbol{E} \parallel$ [010] or $\boldsymbol{E} \perp$ [100]) and strain $\perp$ [001]<br>• $\boldsymbol{E}$ in generic direction
Pc (7.24) $\mathbb{Z}_1$	• $\boldsymbol{B} \parallel$ [010] and strain $\perp$ [010]<br>• ($\boldsymbol{E} \parallel$ [100] or $\boldsymbol{E} \perp$ [010]) and $\boldsymbol{B} \parallel$ [010]
Pc' (7.26) $\mathbb{Z}_1$	• $\boldsymbol{B} \parallel$ [100] and strain $\perp$ [010]<br>• $\boldsymbol{B} \parallel$ [001] and strain $\perp$ [010]<br>• $\boldsymbol{B} \perp$ [010]<br>• ($\boldsymbol{E} \parallel$ [100] or $\boldsymbol{E} \perp$ [010]) and $\boldsymbol{B} \parallel$ [100]<br>• ($\boldsymbol{E} \parallel$ [100] or $\boldsymbol{E} \perp$ [010]) and $\boldsymbol{B} \parallel$ [001]
P_ac (7.27) $\mathbb{Z}_1$	• strain $\perp$ [010]<br>• ($\boldsymbol{E} \parallel$ [100] or $\boldsymbol{E} \perp$ [010])
$P2$ (3.1) $\mathbb{Z}_2$	• $\boldsymbol{B} \parallel$ [001] and strain $\perp$ [001]
$P2_1'$ (4.9) $\mathbb{Z}_1$	• $\boldsymbol{B} \parallel$ [100] and strain $\perp$ [001]<br>• $\boldsymbol{B} \parallel$ [010] and strain $\perp$ [001]<br>• $\boldsymbol{B} \perp$ [001]
P_b2 (3.5) $\mathbb{Z}_2$	• strain $\perp$ [001]
Pc (7.24) $\mathbb{Z}_1$	• $\boldsymbol{B} \parallel$ [100] and strain $\perp$ [100]<br>• ($\boldsymbol{E} \parallel$ [010] or $\boldsymbol{E} \perp$ [100]) and $\boldsymbol{B} \parallel$ [100]

Continued on next page

...continued

Subgroup & SI group	Perturbation
$Pn'a2_1'$ (33.146) $\mathbb{Z}_1$	• $\boldsymbol{B} \parallel [100]$
Pc' (7.26) $\mathbb{Z}_1$	• $\boldsymbol{B} \parallel [010]$ and strain $\perp [100]$<br>• $\boldsymbol{B} \parallel [001]$ and strain $\perp [100]$<br>• $\boldsymbol{B} \perp [100]$<br>• ($\boldsymbol{E} \parallel [010]$ or $\boldsymbol{E} \perp [100]$) and $\boldsymbol{B} \parallel [010]$<br>• ($\boldsymbol{E} \parallel [010]$ or $\boldsymbol{E} \perp [100]$) and $\boldsymbol{B} \parallel [001]$
$Pn'n'2$ (34.159) $\mathbb{Z}_1$	• $\boldsymbol{B} \parallel [001]$
$Pn'a2_1'$ (33.146) $\mathbb{Z}_1$	• $\boldsymbol{B} \parallel [010]$
P_ac (7.27) $\mathbb{Z}_1$	• strain $\perp [100]$<br>• ($\boldsymbol{E} \parallel [010]$ or $\boldsymbol{E} \perp [100]$)

Table. S 250: Subgroups of P_bba2 (32.140) that can be realized by applying $\boldsymbol{E}/\boldsymbol{B}$ fields and/or uniaxial strains.

Subgroup & SI group	Perturbation
P_S1 (1.3) $\mathbb{Z}_1$	• strain in generic direction<br>• ($\boldsymbol{E} \parallel [100]$ or $\boldsymbol{E} \perp [010]$) and strain $\perp [100]$<br>• ($\boldsymbol{E} \parallel [100]$ or $\boldsymbol{E} \perp [010]$) and strain $\perp [001]$<br>• ($\boldsymbol{E} \parallel [010]$ or $\boldsymbol{E} \perp [100]$) and strain $\perp [010]$<br>• ($\boldsymbol{E} \parallel [010]$ or $\boldsymbol{E} \perp [100]$) and strain $\perp [001]$<br>• $\boldsymbol{E}$ in generic direction
Pc' (7.26) $\mathbb{Z}_1$	• $\boldsymbol{B} \parallel [100]$ and strain $\perp [010]$<br>• $\boldsymbol{B} \parallel [001]$ and strain $\perp [010]$<br>• $\boldsymbol{B} \perp [010]$<br>• ($\boldsymbol{E} \parallel [100]$ or $\boldsymbol{E} \perp [010]$) and $\boldsymbol{B} \parallel [100]$<br>• ($\boldsymbol{E} \parallel [100]$ or $\boldsymbol{E} \perp [010]$) and $\boldsymbol{B} \parallel [001]$
Pc (7.24) $\mathbb{Z}_1$	• $\boldsymbol{B} \parallel [010]$ and strain $\perp [010]$<br>• ($\boldsymbol{E} \parallel [100]$ or $\boldsymbol{E} \perp [010]$) and $\boldsymbol{B} \parallel [010]$
P_bc (7.29) $\mathbb{Z}_1$	• strain $\perp [010]$<br>• ($\boldsymbol{E} \parallel [100]$ or $\boldsymbol{E} \perp [010]$)
$P2$ (3.1) $\mathbb{Z}_2$	• $\boldsymbol{B} \parallel [001]$ and strain $\perp [001]$
$P2'$ (3.3) $\mathbb{Z}_1$	• $\boldsymbol{B} \parallel [100]$ and strain $\perp [001]$<br>• $\boldsymbol{B} \parallel [010]$ and strain $\perp [001]$<br>• $\boldsymbol{B} \perp [001]$

Continued on next page

. . . continued

Subgroup & SI group	Perturbation
P_a2 (3.4) $\mathbb{Z}_2$	• strain $\perp$ [001]
Pm' (6.20) $\mathbb{Z}_1$	• $\boldsymbol{B}$ $\parallel$ [010] and strain $\perp$ [100]<br>• $\boldsymbol{B}$ $\parallel$ [001] and strain $\perp$ [100]<br>• $\boldsymbol{B}$ $\perp$ [100]<br>• ($\boldsymbol{E}$ $\parallel$ [010] or $\boldsymbol{E}$ $\perp$ [100]) and $\boldsymbol{B}$ $\parallel$ [010]<br>• ($\boldsymbol{E}$ $\parallel$ [010] or $\boldsymbol{E}$ $\perp$ [100]) and $\boldsymbol{B}$ $\parallel$ [001]
$Pm'a'2$ (28.91) $\mathbb{Z}_2$	• $\boldsymbol{B}$ $\parallel$ [001]
$Pm'a2'$ (28.89) $\mathbb{Z}_1$	• $\boldsymbol{B}$ $\parallel$ [010]
Pc (7.24) $\mathbb{Z}_1$	• $\boldsymbol{B}$ $\parallel$ [100] and strain $\perp$ [100]<br>• ($\boldsymbol{E}$ $\parallel$ [010] or $\boldsymbol{E}$ $\perp$ [100]) and $\boldsymbol{B}$ $\parallel$ [100]
$Pb'a2'$ (32.137) $\mathbb{Z}_1$	• $\boldsymbol{B}$ $\parallel$ [100]
P_cc (7.28) $\mathbb{Z}_1$	• strain $\perp$ [100]<br>• ($\boldsymbol{E}$ $\parallel$ [010] or $\boldsymbol{E}$ $\perp$ [100])

Table. S 251: Subgroups of P_Cba2 (32.141) that can be realized by applying $\boldsymbol{E}/\boldsymbol{B}$ fields and/or uniaxial strains.

Subgroup & SI group	Perturbation
Pm' (6.20) $\mathbb{Z}_1$	• $\boldsymbol{B}$ $\parallel$ [100] and strain $\perp$ [010]<br>• $\boldsymbol{B}$ $\parallel$ [001] and strain $\perp$ [010]<br>• $\boldsymbol{B}$ $\perp$ [010]<br>• ($\boldsymbol{E}$ $\parallel$ [100] or $\boldsymbol{E}$ $\perp$ [010]) and $\boldsymbol{B}$ $\parallel$ [100]<br>• ($\boldsymbol{E}$ $\parallel$ [100] or $\boldsymbol{E}$ $\perp$ [010]) and $\boldsymbol{B}$ $\parallel$ [001]
P_S1 (1.3) $\mathbb{Z}_1$	• strain in generic direction<br>• ($\boldsymbol{E}$ $\parallel$ [100] or $\boldsymbol{E}$ $\perp$ [010]) and strain $\perp$ [100]<br>• ($\boldsymbol{E}$ $\parallel$ [100] or $\boldsymbol{E}$ $\perp$ [010]) and strain $\perp$ [001]<br>• ($\boldsymbol{E}$ $\parallel$ [010] or $\boldsymbol{E}$ $\perp$ [100]) and strain $\perp$ [010]<br>• ($\boldsymbol{E}$ $\parallel$ [010] or $\boldsymbol{E}$ $\perp$ [100]) and strain $\perp$ [001]<br>• $\boldsymbol{E}$ in generic direction
Pc (7.24) $\mathbb{Z}_1$	• $\boldsymbol{B}$ $\parallel$ [010] and strain $\perp$ [010]<br>• ($\boldsymbol{E}$ $\parallel$ [100] or $\boldsymbol{E}$ $\perp$ [010]) and $\boldsymbol{B}$ $\parallel$ [010]
P_Ac (7.31) $\mathbb{Z}_1$	• strain $\perp$ [010]<br>• ($\boldsymbol{E}$ $\parallel$ [100] or $\boldsymbol{E}$ $\perp$ [010])

Continued on next page

. . . continued

Subgroup & SI group	Perturbation
Pm' (6.20) $\mathbb{Z}_1$	• $\boldsymbol{B} \parallel [010]$ and strain $\perp [100]$<br>• $\boldsymbol{B} \parallel [001]$ and strain $\perp [100]$<br>• $\boldsymbol{B} \perp [100]$<br>• ($\boldsymbol{E} \parallel [010]$ or $\boldsymbol{E} \perp [100]$) and $\boldsymbol{B} \parallel [010]$<br>• ($\boldsymbol{E} \parallel [010]$ or $\boldsymbol{E} \perp [100]$) and $\boldsymbol{B} \parallel [001]$
$P2$ (3.1) $\mathbb{Z}_2$	• $\boldsymbol{B} \parallel [001]$ and strain $\perp [001]$
$Pm'm'2$ (25.60) $\mathbb{Z}_2$	• $\boldsymbol{B} \parallel [001]$
Pc (7.24) $\mathbb{Z}_1$	• $\boldsymbol{B} \parallel [100]$ and strain $\perp [100]$<br>• ($\boldsymbol{E} \parallel [010]$ or $\boldsymbol{E} \perp [100]$) and $\boldsymbol{B} \parallel [100]$
P_Ac (7.31) $\mathbb{Z}_1$	• strain $\perp [100]$<br>• ($\boldsymbol{E} \parallel [010]$ or $\boldsymbol{E} \perp [100]$)
$P2'$ (3.3) $\mathbb{Z}_1$	• $\boldsymbol{B} \parallel [100]$ and strain $\perp [001]$<br>• $\boldsymbol{B} \parallel [010]$ and strain $\perp [001]$<br>• $\boldsymbol{B} \perp [001]$
$Pm'a2'$ (28.89) $\mathbb{Z}_1$	• $\boldsymbol{B} \parallel [010]$
P_a2 (3.4) $\mathbb{Z}_2$	• strain $\perp [001]$
$Pm'a2'$ (28.89) $\mathbb{Z}_1$	• $\boldsymbol{B} \parallel [100]$

Table. S 252: Subgroups of P_Aba2 (32.142) that can be realized by applying $\boldsymbol{E}/\boldsymbol{B}$ fields and/or uniaxial strains.

Subgroup & SI group	Perturbation
P_S1 (1.3) $\mathbb{Z}_1$	• strain in generic direction<br>• ($\boldsymbol{E} \parallel [100]$ or $\boldsymbol{E} \perp [010]$) and strain $\perp [100]$<br>• ($\boldsymbol{E} \parallel [100]$ or $\boldsymbol{E} \perp [010]$) and strain $\perp [001]$<br>• ($\boldsymbol{E} \parallel [010]$ or $\boldsymbol{E} \perp [100]$) and strain $\perp [010]$<br>• ($\boldsymbol{E} \parallel [010]$ or $\boldsymbol{E} \perp [100]$) and strain $\perp [001]$<br>• $\boldsymbol{E}$ in generic direction
Pc' (7.26) $\mathbb{Z}_1$	• $\boldsymbol{B} \parallel [100]$ and strain $\perp [010]$<br>• $\boldsymbol{B} \parallel [001]$ and strain $\perp [010]$<br>• $\boldsymbol{B} \perp [010]$<br>• ($\boldsymbol{E} \parallel [100]$ or $\boldsymbol{E} \perp [010]$) and $\boldsymbol{B} \parallel [100]$<br>• ($\boldsymbol{E} \parallel [100]$ or $\boldsymbol{E} \perp [010]$) and $\boldsymbol{B} \parallel [001]$

Continued on next page

...continued

Subgroup & SI group	Perturbation
Pc (7.24) $\mathbb{Z}_1$	• $\boldsymbol{B}$ ∥ [010] and strain ⊥ [010]<br>• ($\boldsymbol{E}$ ∥ [100] or $\boldsymbol{E}$ ⊥ [010]) and $\boldsymbol{B}$ ∥ [010]
P_Cc (7.30) $\mathbb{Z}_1$	• strain ⊥ [010]<br>• ($\boldsymbol{E}$ ∥ [100] or $\boldsymbol{E}$ ⊥ [010])
$P2$ (3.1) $\mathbb{Z}_2$	• $\boldsymbol{B}$ ∥ [001] and strain ⊥ [001]
$P2_1'$ (4.9) $\mathbb{Z}_1$	• $\boldsymbol{B}$ ∥ [100] and strain ⊥ [001]<br>• $\boldsymbol{B}$ ∥ [010] and strain ⊥ [001]<br>• $\boldsymbol{B}$ ⊥ [001]
P_C2 (3.6) $\mathbb{Z}_1$	• strain ⊥ [001]
Pc' (7.26) $\mathbb{Z}_1$	• $\boldsymbol{B}$ ∥ [010] and strain ⊥ [100]<br>• $\boldsymbol{B}$ ∥ [001] and strain ⊥ [100]<br>• $\boldsymbol{B}$ ⊥ [100]<br>• ($\boldsymbol{E}$ ∥ [010] or $\boldsymbol{E}$ ⊥ [100]) and $\boldsymbol{B}$ ∥ [010]<br>• ($\boldsymbol{E}$ ∥ [010] or $\boldsymbol{E}$ ⊥ [100]) and $\boldsymbol{B}$ ∥ [001]
$Pn'c'2$ (30.115) $\mathbb{Z}_1$	• $\boldsymbol{B}$ ∥ [001]
$Pc'a2_1'$ (29.101) $\mathbb{Z}_1$	• $\boldsymbol{B}$ ∥ [010]
Pc (7.24) $\mathbb{Z}_1$	• $\boldsymbol{B}$ ∥ [100] and strain ⊥ [100]<br>• ($\boldsymbol{E}$ ∥ [010] or $\boldsymbol{E}$ ⊥ [100]) and $\boldsymbol{B}$ ∥ [100]
$Pn'a2_1'$ (33.146) $\mathbb{Z}_1$	• $\boldsymbol{B}$ ∥ [100]
P_ac (7.27) $\mathbb{Z}_1$	• strain ⊥ [100]<br>• ($\boldsymbol{E}$ ∥ [010] or $\boldsymbol{E}$ ⊥ [100])

Table. S 253: Subgroups of P_Iba2 (32.143) that can be realized by applying $\boldsymbol{E}/\boldsymbol{B}$ fields and/or uniaxial strains.

Subgroup & SI group	Perturbation
Pc' (7.26) $\mathbb{Z}_1$	• $\boldsymbol{B}$ ∥ [100] and strain ⊥ [010]<br>• $\boldsymbol{B}$ ∥ [001] and strain ⊥ [010]<br>• $\boldsymbol{B}$ ⊥ [010]<br>• ($\boldsymbol{E}$ ∥ [100] or $\boldsymbol{E}$ ⊥ [010]) and $\boldsymbol{B}$ ∥ [100]<br>• ($\boldsymbol{E}$ ∥ [100] or $\boldsymbol{E}$ ⊥ [010]) and $\boldsymbol{B}$ ∥ [001]

Continued on next page

…continued

Subgroup & SI group	Perturbation
P_S1 (1.3) $\mathbb{Z}_1$	• strain in generic direction<br>• ($\boldsymbol{E} \parallel [100]$ or $\boldsymbol{E} \perp [010]$) and strain $\perp [100]$<br>• ($\boldsymbol{E} \parallel [100]$ or $\boldsymbol{E} \perp [010]$) and strain $\perp [001]$<br>• ($\boldsymbol{E} \parallel [010]$ or $\boldsymbol{E} \perp [100]$) and strain $\perp [010]$<br>• ($\boldsymbol{E} \parallel [010]$ or $\boldsymbol{E} \perp [100]$) and strain $\perp [001]$<br>• $\boldsymbol{E}$ in generic direction
Pc (7.24) $\mathbb{Z}_1$	• $\boldsymbol{B} \parallel [010]$ and strain $\perp [010]$<br>• ($\boldsymbol{E} \parallel [100]$ or $\boldsymbol{E} \perp [010]$) and $\boldsymbol{B} \parallel [010]$
P_Cc (7.30) $\mathbb{Z}_1$	• strain $\perp [010]$<br>• ($\boldsymbol{E} \parallel [100]$ or $\boldsymbol{E} \perp [010]$)
Pc' (7.26) $\mathbb{Z}_1$	• $\boldsymbol{B} \parallel [010]$ and strain $\perp [100]$<br>• $\boldsymbol{B} \parallel [001]$ and strain $\perp [100]$<br>• $\boldsymbol{B} \perp [100]$<br>• ($\boldsymbol{E} \parallel [010]$ or $\boldsymbol{E} \perp [100]$) and $\boldsymbol{B} \parallel [010]$<br>• ($\boldsymbol{E} \parallel [010]$ or $\boldsymbol{E} \perp [100]$) and $\boldsymbol{B} \parallel [001]$
$P2$ (3.1) $\mathbb{Z}_2$	• $\boldsymbol{B} \parallel [001]$ and strain $\perp [001]$
$Pc'c'2$ (27.81) $\mathbb{Z}_2$	• $\boldsymbol{B} \parallel [001]$
Pc (7.24) $\mathbb{Z}_1$	• $\boldsymbol{B} \parallel [100]$ and strain $\perp [100]$<br>• ($\boldsymbol{E} \parallel [010]$ or $\boldsymbol{E} \perp [100]$) and $\boldsymbol{B} \parallel [100]$
P_Cc (7.30) $\mathbb{Z}_1$	• strain $\perp [100]$<br>• ($\boldsymbol{E} \parallel [010]$ or $\boldsymbol{E} \perp [100]$)
$P2_1'$ (4.9) $\mathbb{Z}_1$	• $\boldsymbol{B} \parallel [100]$ and strain $\perp [001]$<br>• $\boldsymbol{B} \parallel [010]$ and strain $\perp [001]$<br>• $\boldsymbol{B} \perp [001]$
$Pc'a2_1'$ (29.101) $\mathbb{Z}_1$	• $\boldsymbol{B} \parallel [010]$
P_C2 (3.6) $\mathbb{Z}_1$	• strain $\perp [001]$
$Pc'a2_1'$ (29.101) $\mathbb{Z}_1$	• $\boldsymbol{B} \parallel [100]$

Table. S 254: Subgroups of $Pna2_1$ (33.144) that can be realized by applying $\boldsymbol{E}/\boldsymbol{B}$ fields and/or uniaxial strains.

Subgroup & SI group	Perturbation

Continued on next page

...continued

Subgroup & SI group	Perturbation
Pc (7.24) $\mathbb{Z}_1$	• strain $\perp$ [010]<br>• $\boldsymbol{B} \parallel$ [010]<br>• ($\boldsymbol{E} \parallel$ [100] or $\boldsymbol{E} \perp$ [010])
$P2_1$ (4.7) $\mathbb{Z}_1$	• strain $\perp$ [001]<br>• $\boldsymbol{B} \parallel$ [001]
Pc (7.24) $\mathbb{Z}_1$	• strain $\perp$ [100]<br>• $\boldsymbol{B} \parallel$ [100]<br>• ($\boldsymbol{E} \parallel$ [010] or $\boldsymbol{E} \perp$ [100])

Table. S 255: Subgroups of $Pna2_11'$ (33.145) that can be realized by applying $\boldsymbol{E}/\boldsymbol{B}$ fields and/or uniaxial strains.

Subgroup & SI group	Perturbation
$P11'$ (1.2) $\mathbb{Z}_1$	• strain in generic direction<br>• ($\boldsymbol{E} \parallel$ [100] or $\boldsymbol{E} \perp$ [010]) and strain $\perp$ [100]<br>• ($\boldsymbol{E} \parallel$ [100] or $\boldsymbol{E} \perp$ [010]) and strain $\perp$ [001]<br>• ($\boldsymbol{E} \parallel$ [010] or $\boldsymbol{E} \perp$ [100]) and strain $\perp$ [010]<br>• ($\boldsymbol{E} \parallel$ [010] or $\boldsymbol{E} \perp$ [100]) and strain $\perp$ [001]<br>• $\boldsymbol{E}$ in generic direction
Pc' (7.26) $\mathbb{Z}_1$	• $\boldsymbol{B} \parallel$ [100] and strain $\perp$ [010]<br>• $\boldsymbol{B} \parallel$ [001] and strain $\perp$ [010]<br>• $\boldsymbol{B} \perp$ [010]<br>• ($\boldsymbol{E} \parallel$ [100] or $\boldsymbol{E} \perp$ [010]) and $\boldsymbol{B} \parallel$ [100]<br>• ($\boldsymbol{E} \parallel$ [100] or $\boldsymbol{E} \perp$ [010]) and $\boldsymbol{B} \parallel$ [001]
Pc (7.24) $\mathbb{Z}_1$	• $\boldsymbol{B} \parallel$ [010] and strain $\perp$ [010]<br>• ($\boldsymbol{E} \parallel$ [100] or $\boldsymbol{E} \perp$ [010]) and $\boldsymbol{B} \parallel$ [010]
$Pc1'$ (7.25) $\mathbb{Z}_1$	• strain $\perp$ [010]<br>• ($\boldsymbol{E} \parallel$ [100] or $\boldsymbol{E} \perp$ [010])
$P2_1'$ (4.9) $\mathbb{Z}_1$	• $\boldsymbol{B} \parallel$ [100] and strain $\perp$ [001]<br>• $\boldsymbol{B} \parallel$ [010] and strain $\perp$ [001]<br>• $\boldsymbol{B} \perp$ [001]
$P2_1$ (4.7) $\mathbb{Z}_1$	• $\boldsymbol{B} \parallel$ [001] and strain $\perp$ [001]
$P2_11'$ (4.8) $\mathbb{Z}_1$	• strain $\perp$ [001]

Continued on next page

. . . continued

Subgroup & SI group	Perturbation
Pc' (7.26) $\mathbb{Z}_1$	• $\boldsymbol{B} \parallel [010]$ and strain $\perp [100]$<br>• $\boldsymbol{B} \parallel [001]$ and strain $\perp [100]$<br>• $\boldsymbol{B} \perp [100]$<br>• ($\boldsymbol{E} \parallel [010]$ or $\boldsymbol{E} \perp [100]$) and $\boldsymbol{B} \parallel [010]$<br>• ($\boldsymbol{E} \parallel [010]$ or $\boldsymbol{E} \perp [100]$) and $\boldsymbol{B} \parallel [001]$
$Pn'a2_1'$ (33.146) $\mathbb{Z}_1$	• $\boldsymbol{B} \parallel [010]$
$Pn'a'2_1$ (33.148) $\mathbb{Z}_1$	• $\boldsymbol{B} \parallel [001]$
Pc (7.24) $\mathbb{Z}_1$	• $\boldsymbol{B} \parallel [100]$ and strain $\perp [100]$<br>• ($\boldsymbol{E} \parallel [010]$ or $\boldsymbol{E} \perp [100]$) and $\boldsymbol{B} \parallel [100]$
$Pna'2_1'$ (33.147) $\mathbb{Z}_1$	• $\boldsymbol{B} \parallel [100]$
$Pc1'$ (7.25) $\mathbb{Z}_1$	• strain $\perp [100]$<br>• ($\boldsymbol{E} \parallel [010]$ or $\boldsymbol{E} \perp [100]$)

Table. S 256: Subgroups of $Pn'a2_1'$ (33.146) that can be realized by applying $\boldsymbol{E}/\boldsymbol{B}$ fields and/or uniaxial strains.

Subgroup & SI group	Perturbation
Pc (7.24) $\mathbb{Z}_1$	• strain $\perp [010]$<br>• ($\boldsymbol{E} \parallel [100]$ or $\boldsymbol{E} \perp [010]$)
$P2_1'$ (4.9) $\mathbb{Z}_1$	• strain $\perp [001]$<br>• ($\boldsymbol{B} \parallel [100]$ or $\boldsymbol{B} \perp [001]$)
Pc' (7.26) $\mathbb{Z}_1$	• strain $\perp [100]$<br>• ($\boldsymbol{B} \parallel [001]$ or $\boldsymbol{B} \perp [100]$)<br>• ($\boldsymbol{E} \parallel [010]$ or $\boldsymbol{E} \perp [100]$)

Table. S 257: Subgroups of $Pna'2_1'$ (33.147) that can be realized by applying $\boldsymbol{E}/\boldsymbol{B}$ fields and/or uniaxial strains.

Subgroup & SI group	Perturbation

Continued on next page

...continued

Subgroup & SI group	Perturbation
Pc' (7.26) $\mathbb{Z}_1$	• strain $\perp$ [010]<br>• ($\boldsymbol{B} \parallel$ [001] or $\boldsymbol{B} \perp$ [010])<br>• ($\boldsymbol{E} \parallel$ [100] or $\boldsymbol{E} \perp$ [010])
$P2_1'$ (4.9) $\mathbb{Z}_1$	• strain $\perp$ [001]<br>• ($\boldsymbol{B} \parallel$ [010] or $\boldsymbol{B} \perp$ [001])
Pc (7.24) $\mathbb{Z}_1$	• strain $\perp$ [100]<br>• ($\boldsymbol{E} \parallel$ [010] or $\boldsymbol{E} \perp$ [100])

Table. S 258: Subgroups of $Pn'a'2_1$ (33.148) that can be realized by applying $\boldsymbol{E}/\boldsymbol{B}$ fields and/or uniaxial strains.

Subgroup & SI group	Perturbation
Pc' (7.26) $\mathbb{Z}_1$	• strain $\perp$ [010]<br>• ($\boldsymbol{B} \parallel$ [100] or $\boldsymbol{B} \perp$ [010])<br>• ($\boldsymbol{E} \parallel$ [100] or $\boldsymbol{E} \perp$ [010])
$P2_1$ (4.7) $\mathbb{Z}_1$	• strain $\perp$ [001]
Pc' (7.26) $\mathbb{Z}_1$	• strain $\perp$ [100]<br>• ($\boldsymbol{B} \parallel$ [010] or $\boldsymbol{B} \perp$ [100])<br>• ($\boldsymbol{E} \parallel$ [010] or $\boldsymbol{E} \perp$ [100])

Table. S 259: Subgroups of P_ana2_1 (33.149) that can be realized by applying $\boldsymbol{E}/\boldsymbol{B}$ fields and/or uniaxial strains.

Subgroup & SI group	Perturbation
Pm' (6.20) $\mathbb{Z}_1$	• $\boldsymbol{B} \parallel$ [100] and strain $\perp$ [010]<br>• $\boldsymbol{B} \parallel$ [001] and strain $\perp$ [010]<br>• $\boldsymbol{B} \perp$ [010]<br>• ($\boldsymbol{E} \parallel$ [100] or $\boldsymbol{E} \perp$ [010]) and $\boldsymbol{B} \parallel$ [100]<br>• ($\boldsymbol{E} \parallel$ [100] or $\boldsymbol{E} \perp$ [010]) and $\boldsymbol{B} \parallel$ [001]

Continued on next page

...continued

Subgroup & SI group	Perturbation
P_S1 (1.3) $\mathbb{Z}_1$	• strain in generic direction<br>• ($\boldsymbol{E} \parallel [100]$ or $\boldsymbol{E} \perp [010]$) and strain $\perp [100]$<br>• ($\boldsymbol{E} \parallel [100]$ or $\boldsymbol{E} \perp [010]$) and strain $\perp [001]$<br>• ($\boldsymbol{E} \parallel [010]$ or $\boldsymbol{E} \perp [100]$) and strain $\perp [010]$<br>• ($\boldsymbol{E} \parallel [010]$ or $\boldsymbol{E} \perp [100]$) and strain $\perp [001]$<br>• $\boldsymbol{E}$ in generic direction
Pc (7.24) $\mathbb{Z}_1$	• $\boldsymbol{B} \parallel [010]$ and strain $\perp [010]$<br>• ($\boldsymbol{E} \parallel [100]$ or $\boldsymbol{E} \perp [010]$) and $\boldsymbol{B} \parallel [010]$
P_cc (7.28) $\mathbb{Z}_1$	• strain $\perp [010]$<br>• ($\boldsymbol{E} \parallel [100]$ or $\boldsymbol{E} \perp [010]$)
Pc' (7.26) $\mathbb{Z}_1$	• $\boldsymbol{B} \parallel [010]$ and strain $\perp [100]$<br>• $\boldsymbol{B} \parallel [001]$ and strain $\perp [100]$<br>• $\boldsymbol{B} \perp [100]$<br>• ($\boldsymbol{E} \parallel [010]$ or $\boldsymbol{E} \perp [100]$) and $\boldsymbol{B} \parallel [010]$<br>• ($\boldsymbol{E} \parallel [010]$ or $\boldsymbol{E} \perp [100]$) and $\boldsymbol{B} \parallel [001]$
$P2_1$ (4.7) $\mathbb{Z}_1$	• $\boldsymbol{B} \parallel [001]$ and strain $\perp [001]$
$Pm'n'2_1$ (31.127) $\mathbb{Z}_1$	• $\boldsymbol{B} \parallel [001]$
Pc (7.24) $\mathbb{Z}_1$	• $\boldsymbol{B} \parallel [100]$ and strain $\perp [100]$<br>• ($\boldsymbol{E} \parallel [010]$ or $\boldsymbol{E} \perp [100]$) and $\boldsymbol{B} \parallel [100]$
P_bc (7.29) $\mathbb{Z}_1$	• strain $\perp [100]$<br>• ($\boldsymbol{E} \parallel [010]$ or $\boldsymbol{E} \perp [100]$)
$P2_1'$ (4.9) $\mathbb{Z}_1$	• $\boldsymbol{B} \parallel [100]$ and strain $\perp [001]$<br>• $\boldsymbol{B} \parallel [010]$ and strain $\perp [001]$<br>• $\boldsymbol{B} \perp [001]$
$Pn'a2_1'$ (33.146) $\mathbb{Z}_1$	• $\boldsymbol{B} \parallel [010]$
P_a2_1 (4.10) $\mathbb{Z}_1$	• strain $\perp [001]$
$Pm'n2_1'$ (31.125) $\mathbb{Z}_1$	• $\boldsymbol{B} \parallel [100]$

Table. S 260: Subgroups of P_bna2_1 (33.150) that can be realized by applying $\boldsymbol{E}/\boldsymbol{B}$ fields and/or uniaxial strains.

Subgroup & SI group	Perturbation

Continued on next page

... continued

Subgroup & SI group	Perturbation
P_S1 (1.3) $\mathbb{Z}_1$	• strain in generic direction<br>• ($\boldsymbol{E} \parallel [100]$ or $\boldsymbol{E} \perp [010]$) and strain $\perp [100]$<br>• ($\boldsymbol{E} \parallel [100]$ or $\boldsymbol{E} \perp [010]$) and strain $\perp [001]$<br>• ($\boldsymbol{E} \parallel [010]$ or $\boldsymbol{E} \perp [100]$) and strain $\perp [010]$<br>• ($\boldsymbol{E} \parallel [010]$ or $\boldsymbol{E} \perp [100]$) and strain $\perp [001]$<br>• $\boldsymbol{E}$ in generic direction
Pc' (7.26) $\mathbb{Z}_1$	• $\boldsymbol{B} \parallel [100]$ and strain $\perp [010]$<br>• $\boldsymbol{B} \parallel [001]$ and strain $\perp [010]$<br>• $\boldsymbol{B} \perp [010]$<br>• ($\boldsymbol{E} \parallel [100]$ or $\boldsymbol{E} \perp [010]$) and $\boldsymbol{B} \parallel [100]$<br>• ($\boldsymbol{E} \parallel [100]$ or $\boldsymbol{E} \perp [010]$) and $\boldsymbol{B} \parallel [001]$
Pc (7.24) $\mathbb{Z}_1$	• $\boldsymbol{B} \parallel [010]$ and strain $\perp [010]$<br>• ($\boldsymbol{E} \parallel [100]$ or $\boldsymbol{E} \perp [010]$) and $\boldsymbol{B} \parallel [010]$
P_bc (7.29) $\mathbb{Z}_1$	• strain $\perp [010]$<br>• ($\boldsymbol{E} \parallel [100]$ or $\boldsymbol{E} \perp [010]$)
$P2_1$ (4.7) $\mathbb{Z}_1$	• $\boldsymbol{B} \parallel [001]$ and strain $\perp [001]$
$P2_1'$ (4.9) $\mathbb{Z}_1$	• $\boldsymbol{B} \parallel [100]$ and strain $\perp [001]$<br>• $\boldsymbol{B} \parallel [010]$ and strain $\perp [001]$<br>• $\boldsymbol{B} \perp [001]$
P_a2_1 (4.10) $\mathbb{Z}_1$	• strain $\perp [001]$
Pc' (7.26) $\mathbb{Z}_1$	• $\boldsymbol{B} \parallel [010]$ and strain $\perp [100]$<br>• $\boldsymbol{B} \parallel [001]$ and strain $\perp [100]$<br>• $\boldsymbol{B} \perp [100]$<br>• ($\boldsymbol{E} \parallel [010]$ or $\boldsymbol{E} \perp [100]$) and $\boldsymbol{B} \parallel [010]$<br>• ($\boldsymbol{E} \parallel [010]$ or $\boldsymbol{E} \perp [100]$) and $\boldsymbol{B} \parallel [001]$
$Pc'a'2_1$ (29.103) $\mathbb{Z}_1$	• $\boldsymbol{B} \parallel [001]$
$Pc'a2_1'$ (29.101) $\mathbb{Z}_1$	• $\boldsymbol{B} \parallel [010]$
Pc (7.24) $\mathbb{Z}_1$	• $\boldsymbol{B} \parallel [100]$ and strain $\perp [100]$<br>• ($\boldsymbol{E} \parallel [010]$ or $\boldsymbol{E} \perp [100]$) and $\boldsymbol{B} \parallel [100]$
$Pna'2_1'$ (33.147) $\mathbb{Z}_1$	• $\boldsymbol{B} \parallel [100]$
P_ac (7.27) $\mathbb{Z}_1$	• strain $\perp [100]$<br>• ($\boldsymbol{E} \parallel [010]$ or $\boldsymbol{E} \perp [100]$)

Table. S 261: Subgroups of P_cna2_1 (33.151) that can be realized by applying $\boldsymbol{E}/\boldsymbol{B}$ fields and/or uniaxial strains.

Subgroup & SI group	Perturbation
P_S1 (1.3) $\mathbb{Z}_1$	• strain in generic direction<br>• ($\boldsymbol{E} \parallel [100]$ or $\boldsymbol{E} \perp [010]$) and strain $\perp [100]$<br>• ($\boldsymbol{E} \parallel [100]$ or $\boldsymbol{E} \perp [010]$) and strain $\perp [001]$<br>• ($\boldsymbol{E} \parallel [010]$ or $\boldsymbol{E} \perp [100]$) and strain $\perp [010]$<br>• ($\boldsymbol{E} \parallel [010]$ or $\boldsymbol{E} \perp [100]$) and strain $\perp [001]$<br>• $\boldsymbol{E}$ in generic direction
Pc (7.24) $\mathbb{Z}_1$	• $\boldsymbol{B} \parallel [010]$ and strain $\perp [010]$<br>• ($\boldsymbol{E} \parallel [100]$ or $\boldsymbol{E} \perp [010]$) and $\boldsymbol{B} \parallel [010]$
Pc' (7.26) $\mathbb{Z}_1$	• $\boldsymbol{B} \parallel [100]$ and strain $\perp [010]$<br>• $\boldsymbol{B} \parallel [001]$ and strain $\perp [010]$<br>• $\boldsymbol{B} \perp [010]$<br>• ($\boldsymbol{E} \parallel [100]$ or $\boldsymbol{E} \perp [010]$) and $\boldsymbol{B} \parallel [100]$<br>• ($\boldsymbol{E} \parallel [100]$ or $\boldsymbol{E} \perp [010]$) and $\boldsymbol{B} \parallel [001]$
P_ac (7.27) $\mathbb{Z}_1$	• strain $\perp [010]$<br>• ($\boldsymbol{E} \parallel [100]$ or $\boldsymbol{E} \perp [010]$)
$P2'$ (3.3) $\mathbb{Z}_1$	• $\boldsymbol{B} \parallel [100]$ and strain $\perp [001]$<br>• $\boldsymbol{B} \parallel [010]$ and strain $\perp [001]$<br>• $\boldsymbol{B} \perp [001]$
$P2_1$ (4.7) $\mathbb{Z}_1$	• $\boldsymbol{B} \parallel [001]$ and strain $\perp [001]$
P_b2_1 (4.11) $\mathbb{Z}_1$	• strain $\perp [001]$
Pc' (7.26) $\mathbb{Z}_1$	• $\boldsymbol{B} \parallel [010]$ and strain $\perp [100]$<br>• $\boldsymbol{B} \parallel [001]$ and strain $\perp [100]$<br>• $\boldsymbol{B} \perp [100]$<br>• ($\boldsymbol{E} \parallel [010]$ or $\boldsymbol{E} \perp [100]$) and $\boldsymbol{B} \parallel [010]$<br>• ($\boldsymbol{E} \parallel [010]$ or $\boldsymbol{E} \perp [100]$) and $\boldsymbol{B} \parallel [001]$
$Pb'a2'$ (32.137) $\mathbb{Z}_1$	• $\boldsymbol{B} \parallel [010]$
$Pn'a'2_1$ (33.148) $\mathbb{Z}_1$	• $\boldsymbol{B} \parallel [001]$
Pc (7.24) $\mathbb{Z}_1$	• $\boldsymbol{B} \parallel [100]$ and strain $\perp [100]$<br>• ($\boldsymbol{E} \parallel [010]$ or $\boldsymbol{E} \perp [100]$) and $\boldsymbol{B} \parallel [100]$
$Pn'n2'$ (34.158) $\mathbb{Z}_1$	• $\boldsymbol{B} \parallel [100]$
P_ac (7.27) $\mathbb{Z}_1$	• strain $\perp [100]$<br>• ($\boldsymbol{E} \parallel [010]$ or $\boldsymbol{E} \perp [100]$)

Table. S 262: Subgroups of P_Ana2_1 (33.152) that can be realized by applying $\boldsymbol{E}/\boldsymbol{B}$ fields and/or uniaxial strains.

Subgroup & SI group	Perturbation
P_S1 (1.3) $\mathbb{Z}_1$	• strain in generic direction<br>• ($\boldsymbol{E} \parallel [100]$ or $\boldsymbol{E} \perp [010]$) and strain $\perp [100]$<br>• ($\boldsymbol{E} \parallel [100]$ or $\boldsymbol{E} \perp [010]$) and strain $\perp [001]$<br>• ($\boldsymbol{E} \parallel [010]$ or $\boldsymbol{E} \perp [100]$) and strain $\perp [010]$<br>• ($\boldsymbol{E} \parallel [010]$ or $\boldsymbol{E} \perp [100]$) and strain $\perp [001]$<br>• $\boldsymbol{E}$ in generic direction
Pc' (7.26) $\mathbb{Z}_1$	• $\boldsymbol{B} \parallel [100]$ and strain $\perp [010]$<br>• $\boldsymbol{B} \parallel [001]$ and strain $\perp [010]$<br>• $\boldsymbol{B} \perp [010]$<br>• ($\boldsymbol{E} \parallel [100]$ or $\boldsymbol{E} \perp [010]$) and $\boldsymbol{B} \parallel [100]$<br>• ($\boldsymbol{E} \parallel [100]$ or $\boldsymbol{E} \perp [010]$) and $\boldsymbol{B} \parallel [001]$
Pc (7.24) $\mathbb{Z}_1$	• $\boldsymbol{B} \parallel [010]$ and strain $\perp [010]$<br>• ($\boldsymbol{E} \parallel [100]$ or $\boldsymbol{E} \perp [010]$) and $\boldsymbol{B} \parallel [010]$
P_Cc (7.30) $\mathbb{Z}_1$	• strain $\perp [010]$<br>• ($\boldsymbol{E} \parallel [100]$ or $\boldsymbol{E} \perp [010]$)
$P2_1$ (4.7) $\mathbb{Z}_1$	• $\boldsymbol{B} \parallel [001]$ and strain $\perp [001]$
$P2'$ (3.3) $\mathbb{Z}_1$	• $\boldsymbol{B} \parallel [100]$ and strain $\perp [001]$<br>• $\boldsymbol{B} \parallel [010]$ and strain $\perp [001]$<br>• $\boldsymbol{B} \perp [001]$
P_C2_1 (4.12) $\mathbb{Z}_1$	• strain $\perp [001]$
Pm' (6.20) $\mathbb{Z}_1$	• $\boldsymbol{B} \parallel [010]$ and strain $\perp [100]$<br>• $\boldsymbol{B} \parallel [001]$ and strain $\perp [100]$<br>• $\boldsymbol{B} \perp [100]$<br>• ($\boldsymbol{E} \parallel [010]$ or $\boldsymbol{E} \perp [100]$) and $\boldsymbol{B} \parallel [010]$<br>• ($\boldsymbol{E} \parallel [010]$ or $\boldsymbol{E} \perp [100]$) and $\boldsymbol{B} \parallel [001]$
$Pm'n'2_1$ (31.127) $\mathbb{Z}_1$	• $\boldsymbol{B} \parallel [001]$
$Pm'a2'$ (28.89) $\mathbb{Z}_1$	• $\boldsymbol{B} \parallel [010]$
Pc (7.24) $\mathbb{Z}_1$	• $\boldsymbol{B} \parallel [100]$ and strain $\perp [100]$<br>• ($\boldsymbol{E} \parallel [010]$ or $\boldsymbol{E} \perp [100]$) and $\boldsymbol{B} \parallel [100]$
$Pn'n2'$ (34.158) $\mathbb{Z}_1$	• $\boldsymbol{B} \parallel [100]$
P_cc (7.28) $\mathbb{Z}_1$	• strain $\perp [100]$<br>• ($\boldsymbol{E} \parallel [010]$ or $\boldsymbol{E} \perp [100]$)

Table. S 263: Subgroups of P_Bna2_1 (33.153) that can be realized by applying $\boldsymbol{E}/\boldsymbol{B}$ fields and/or uniaxial strains.

Subgroup & SI group	Perturbation
Pc' (7.26) $\mathbb{Z}_1$	• $\boldsymbol{B}$ ∥ [100] and strain ⊥ [010]<br>• $\boldsymbol{B}$ ∥ [001] and strain ⊥ [010]<br>• $\boldsymbol{B}$ ⊥ [010]<br>• ($\boldsymbol{E}$ ∥ [100] or $\boldsymbol{E}$ ⊥ [010]) and $\boldsymbol{B}$ ∥ [100]<br>• ($\boldsymbol{E}$ ∥ [100] or $\boldsymbol{E}$ ⊥ [010]) and $\boldsymbol{B}$ ∥ [001]
P_S1 (1.3) $\mathbb{Z}_1$	• strain in generic direction<br>• ($\boldsymbol{E}$ ∥ [100] or $\boldsymbol{E}$ ⊥ [010]) and strain ⊥ [100]<br>• ($\boldsymbol{E}$ ∥ [100] or $\boldsymbol{E}$ ⊥ [010]) and strain ⊥ [001]<br>• ($\boldsymbol{E}$ ∥ [010] or $\boldsymbol{E}$ ⊥ [100]) and strain ⊥ [010]<br>• ($\boldsymbol{E}$ ∥ [010] or $\boldsymbol{E}$ ⊥ [100]) and strain ⊥ [001]<br>• $\boldsymbol{E}$ in generic direction
Pc (7.24) $\mathbb{Z}_1$	• $\boldsymbol{B}$ ∥ [010] and strain ⊥ [010]<br>• ($\boldsymbol{E}$ ∥ [100] or $\boldsymbol{E}$ ⊥ [010]) and $\boldsymbol{B}$ ∥ [010]
P_ac (7.27) $\mathbb{Z}_1$	• strain ⊥ [010]<br>• ($\boldsymbol{E}$ ∥ [100] or $\boldsymbol{E}$ ⊥ [010])
Pc' (7.26) $\mathbb{Z}_1$	• $\boldsymbol{B}$ ∥ [010] and strain ⊥ [100]<br>• $\boldsymbol{B}$ ∥ [001] and strain ⊥ [100]<br>• $\boldsymbol{B}$ ⊥ [100]<br>• ($\boldsymbol{E}$ ∥ [010] or $\boldsymbol{E}$ ⊥ [100]) and $\boldsymbol{B}$ ∥ [010]<br>• ($\boldsymbol{E}$ ∥ [010] or $\boldsymbol{E}$ ⊥ [100]) and $\boldsymbol{B}$ ∥ [001]
$P2_1$ (4.7) $\mathbb{Z}_1$	• $\boldsymbol{B}$ ∥ [001] and strain ⊥ [001]
$Pc'a'2_1$ (29.103) $\mathbb{Z}_1$	• $\boldsymbol{B}$ ∥ [001]
Pc (7.24) $\mathbb{Z}_1$	• $\boldsymbol{B}$ ∥ [100] and strain ⊥ [100]<br>• ($\boldsymbol{E}$ ∥ [010] or $\boldsymbol{E}$ ⊥ [100]) and $\boldsymbol{B}$ ∥ [100]
P_Cc (7.30) $\mathbb{Z}_1$	• strain ⊥ [100]<br>• ($\boldsymbol{E}$ ∥ [010] or $\boldsymbol{E}$ ⊥ [100])
$P2'$ (3.3) $\mathbb{Z}_1$	• $\boldsymbol{B}$ ∥ [100] and strain ⊥ [001]<br>• $\boldsymbol{B}$ ∥ [010] and strain ⊥ [001]<br>• $\boldsymbol{B}$ ⊥ [001]
$Pb'a2'$ (32.137) $\mathbb{Z}_1$	• $\boldsymbol{B}$ ∥ [010]
P_C2_1 (4.12) $\mathbb{Z}_1$	• strain ⊥ [001]
$Pnc'2'$ (30.114) $\mathbb{Z}_1$	• $\boldsymbol{B}$ ∥ [100]

Table. S 264: Subgroups of P_Cna2_1 (33.154) that can be realized by applying $\boldsymbol{E}/\boldsymbol{B}$ fields and/or uniaxial strains.

Subgroup & SI group	Perturbation
Pm' (6.20) $\mathbb{Z}_1$	• $\boldsymbol{B}$ ∥ [100] and strain ⊥ [010]<br>• $\boldsymbol{B}$ ∥ [001] and strain ⊥ [010]<br>• $\boldsymbol{B}$ ⊥ [010]<br>• ($\boldsymbol{E}$ ∥ [100] or $\boldsymbol{E}$ ⊥ [010]) and $\boldsymbol{B}$ ∥ [100]<br>• ($\boldsymbol{E}$ ∥ [100] or $\boldsymbol{E}$ ⊥ [010]) and $\boldsymbol{B}$ ∥ [001]
P_S1 (1.3) $\mathbb{Z}_1$	• strain in generic direction<br>• ($\boldsymbol{E}$ ∥ [100] or $\boldsymbol{E}$ ⊥ [010]) and strain ⊥ [100]<br>• ($\boldsymbol{E}$ ∥ [100] or $\boldsymbol{E}$ ⊥ [010]) and strain ⊥ [001]<br>• ($\boldsymbol{E}$ ∥ [010] or $\boldsymbol{E}$ ⊥ [100]) and strain ⊥ [010]<br>• ($\boldsymbol{E}$ ∥ [010] or $\boldsymbol{E}$ ⊥ [100]) and strain ⊥ [001]<br>• $\boldsymbol{E}$ in generic direction
Pc (7.24) $\mathbb{Z}_1$	• $\boldsymbol{B}$ ∥ [010] and strain ⊥ [010]<br>• ($\boldsymbol{E}$ ∥ [100] or $\boldsymbol{E}$ ⊥ [010]) and $\boldsymbol{B}$ ∥ [010]
P_Ac (7.31) $\mathbb{Z}_1$	• strain ⊥ [010]<br>• ($\boldsymbol{E}$ ∥ [100] or $\boldsymbol{E}$ ⊥ [010])
Pc' (7.26) $\mathbb{Z}_1$	• $\boldsymbol{B}$ ∥ [010] and strain ⊥ [100]<br>• $\boldsymbol{B}$ ∥ [001] and strain ⊥ [100]<br>• $\boldsymbol{B}$ ⊥ [100]<br>• ($\boldsymbol{E}$ ∥ [010] or $\boldsymbol{E}$ ⊥ [100]) and $\boldsymbol{B}$ ∥ [010]<br>• ($\boldsymbol{E}$ ∥ [010] or $\boldsymbol{E}$ ⊥ [100]) and $\boldsymbol{B}$ ∥ [001]
$P2_1$ (4.7) $\mathbb{Z}_1$	• $\boldsymbol{B}$ ∥ [001] and strain ⊥ [001]
$Pm'c'2_1$ (26.70) $\mathbb{Z}_1$	• $\boldsymbol{B}$ ∥ [001]
Pc (7.24) $\mathbb{Z}_1$	• $\boldsymbol{B}$ ∥ [100] and strain ⊥ [100]<br>• ($\boldsymbol{E}$ ∥ [010] or $\boldsymbol{E}$ ⊥ [100]) and $\boldsymbol{B}$ ∥ [100]
P_Cc (7.30) $\mathbb{Z}_1$	• strain ⊥ [100]<br>• ($\boldsymbol{E}$ ∥ [010] or $\boldsymbol{E}$ ⊥ [100])
$P2_1'$ (4.9) $\mathbb{Z}_1$	• $\boldsymbol{B}$ ∥ [100] and strain ⊥ [001]<br>• $\boldsymbol{B}$ ∥ [010] and strain ⊥ [001]<br>• $\boldsymbol{B}$ ⊥ [001]
$Pc'a2_1'$ (29.101) $\mathbb{Z}_1$	• $\boldsymbol{B}$ ∥ [010]
P_a2_1 (4.10) $\mathbb{Z}_1$	• strain ⊥ [001]
$Pm'n2_1'$ (31.125) $\mathbb{Z}_1$	• $\boldsymbol{B}$ ∥ [100]

Table. S 265: Subgroups of P_Ina2_1 (33.155) that can be realized by applying $\boldsymbol{E}/\boldsymbol{B}$ fields and/or uniaxial strains.

Subgroup & SI group	Perturbation
Pc' (7.26) $\mathbb{Z}_1$	• $\boldsymbol{B} \parallel [100]$ and strain $\perp [010]$<br>• $\boldsymbol{B} \parallel [001]$ and strain $\perp [010]$<br>• $\boldsymbol{B} \perp [010]$<br>• ($\boldsymbol{E} \parallel [100]$ or $\boldsymbol{E} \perp [010]$) and $\boldsymbol{B} \parallel [100]$<br>• ($\boldsymbol{E} \parallel [100]$ or $\boldsymbol{E} \perp [010]$) and $\boldsymbol{B} \parallel [001]$
P_S1 (1.3) $\mathbb{Z}_1$	• strain in generic direction<br>• ($\boldsymbol{E} \parallel [100]$ or $\boldsymbol{E} \perp [010]$) and strain $\perp [100]$<br>• ($\boldsymbol{E} \parallel [100]$ or $\boldsymbol{E} \perp [010]$) and strain $\perp [001]$<br>• ($\boldsymbol{E} \parallel [010]$ or $\boldsymbol{E} \perp [100]$) and strain $\perp [010]$<br>• ($\boldsymbol{E} \parallel [010]$ or $\boldsymbol{E} \perp [100]$) and strain $\perp [001]$<br>• $\boldsymbol{E}$ in generic direction
Pc (7.24) $\mathbb{Z}_1$	• $\boldsymbol{B} \parallel [010]$ and strain $\perp [010]$<br>• ($\boldsymbol{E} \parallel [100]$ or $\boldsymbol{E} \perp [010]$) and $\boldsymbol{B} \parallel [010]$
P_Cc (7.30) $\mathbb{Z}_1$	• strain $\perp [010]$<br>• ($\boldsymbol{E} \parallel [100]$ or $\boldsymbol{E} \perp [010]$)
Pm' (6.20) $\mathbb{Z}_1$	• $\boldsymbol{B} \parallel [010]$ and strain $\perp [100]$<br>• $\boldsymbol{B} \parallel [001]$ and strain $\perp [100]$<br>• $\boldsymbol{B} \perp [100]$<br>• ($\boldsymbol{E} \parallel [010]$ or $\boldsymbol{E} \perp [100]$) and $\boldsymbol{B} \parallel [010]$<br>• ($\boldsymbol{E} \parallel [010]$ or $\boldsymbol{E} \perp [100]$) and $\boldsymbol{B} \parallel [001]$
$P2_1$ (4.7) $\mathbb{Z}_1$	• $\boldsymbol{B} \parallel [001]$ and strain $\perp [001]$
$Pm'c'2_1$ (26.70) $\mathbb{Z}_1$	• $\boldsymbol{B} \parallel [001]$
Pc (7.24) $\mathbb{Z}_1$	• $\boldsymbol{B} \parallel [100]$ and strain $\perp [100]$<br>• ($\boldsymbol{E} \parallel [010]$ or $\boldsymbol{E} \perp [100]$) and $\boldsymbol{B} \parallel [100]$
P_Ac (7.31) $\mathbb{Z}_1$	• strain $\perp [100]$<br>• ($\boldsymbol{E} \parallel [010]$ or $\boldsymbol{E} \perp [100]$)
$P2'$ (3.3) $\mathbb{Z}_1$	• $\boldsymbol{B} \parallel [100]$ and strain $\perp [001]$<br>• $\boldsymbol{B} \parallel [010]$ and strain $\perp [001]$<br>• $\boldsymbol{B} \perp [001]$
$Pm'a2'$ (28.89) $\mathbb{Z}_1$	• $\boldsymbol{B} \parallel [010]$
P_C2_1 (4.12) $\mathbb{Z}_1$	• strain $\perp [001]$
$Pnc'2'$ (30.114) $\mathbb{Z}_1$	• $\boldsymbol{B} \parallel [100]$

Table. S 266: Subgroups of $Pnn2$ (34.156) that can be realized by applying $\boldsymbol{E}/\boldsymbol{B}$ fields and/or uniaxial strains.

Subgroup & SI group	Perturbation
Pc (7.24) $\mathbb{Z}_1$	• strain ⊥ [010]<br>• $\boldsymbol{B}$ ∥ [010]<br>• ($\boldsymbol{E}$ ∥ [100] or $\boldsymbol{E}$ ⊥ [010])
$P2$ (3.1) $\mathbb{Z}_2$	• strain ⊥ [001]<br>• $\boldsymbol{B}$ ∥ [001]
Pc (7.24) $\mathbb{Z}_1$	• strain ⊥ [100]<br>• $\boldsymbol{B}$ ∥ [100]<br>• ($\boldsymbol{E}$ ∥ [010] or $\boldsymbol{E}$ ⊥ [100])

Table. S 267: Subgroups of $Pnn21'$ (34.157) that can be realized by applying $\boldsymbol{E}/\boldsymbol{B}$ fields and/or uniaxial strains.

Subgroup & SI group	Perturbation
$P11'$ (1.2) $\mathbb{Z}_1$	• strain in generic direction<br>• ($\boldsymbol{E}$ ∥ [100] or $\boldsymbol{E}$ ⊥ [010]) and strain ⊥ [100]<br>• ($\boldsymbol{E}$ ∥ [100] or $\boldsymbol{E}$ ⊥ [010]) and strain ⊥ [001]<br>• ($\boldsymbol{E}$ ∥ [010] or $\boldsymbol{E}$ ⊥ [100]) and strain ⊥ [010]<br>• ($\boldsymbol{E}$ ∥ [010] or $\boldsymbol{E}$ ⊥ [100]) and strain ⊥ [001]<br>• $\boldsymbol{E}$ in generic direction
Pc' (7.26) $\mathbb{Z}_1$	• $\boldsymbol{B}$ ∥ [100] and strain ⊥ [010]<br>• $\boldsymbol{B}$ ∥ [001] and strain ⊥ [010]<br>• $\boldsymbol{B}$ ⊥ [010]<br>• ($\boldsymbol{E}$ ∥ [100] or $\boldsymbol{E}$ ⊥ [010]) and $\boldsymbol{B}$ ∥ [100]<br>• ($\boldsymbol{E}$ ∥ [100] or $\boldsymbol{E}$ ⊥ [010]) and $\boldsymbol{B}$ ∥ [001]
Pc (7.24) $\mathbb{Z}_1$	• $\boldsymbol{B}$ ∥ [010] and strain ⊥ [010]<br>• ($\boldsymbol{E}$ ∥ [100] or $\boldsymbol{E}$ ⊥ [010]) and $\boldsymbol{B}$ ∥ [010]
$Pc1'$ (7.25) $\mathbb{Z}_1$	• strain ⊥ [010]<br>• ($\boldsymbol{E}$ ∥ [100] or $\boldsymbol{E}$ ⊥ [010])
$P2'$ (3.3) $\mathbb{Z}_1$	• $\boldsymbol{B}$ ∥ [100] and strain ⊥ [001]<br>• $\boldsymbol{B}$ ∥ [010] and strain ⊥ [001]<br>• $\boldsymbol{B}$ ⊥ [001]
$P2$ (3.1) $\mathbb{Z}_2$	• $\boldsymbol{B}$ ∥ [001] and strain ⊥ [001]

Continued on next page

...continued

Subgroup & SI group	Perturbation
$P21'$ (3.2) $\mathbb{Z}_2$	• strain $\perp$ [001]
Pc' (7.26) $\mathbb{Z}_1$	• $\boldsymbol{B}$ $\parallel$ [010] and strain $\perp$ [100]<br>• $\boldsymbol{B}$ $\parallel$ [001] and strain $\perp$ [100]<br>• $\boldsymbol{B}$ $\perp$ [100]<br>• ($\boldsymbol{E}$ $\parallel$ [010] or $\boldsymbol{E}$ $\perp$ [100]) and $\boldsymbol{B}$ $\parallel$ [010]<br>• ($\boldsymbol{E}$ $\parallel$ [010] or $\boldsymbol{E}$ $\perp$ [100]) and $\boldsymbol{B}$ $\parallel$ [001]
$Pn'n2'$ (34.158) $\mathbb{Z}_1$	• $\boldsymbol{B}$ $\parallel$ [010]
$Pn'n'2$ (34.159) $\mathbb{Z}_1$	• $\boldsymbol{B}$ $\parallel$ [001]
Pc (7.24) $\mathbb{Z}_1$	• $\boldsymbol{B}$ $\parallel$ [100] and strain $\perp$ [100]<br>• ($\boldsymbol{E}$ $\parallel$ [010] or $\boldsymbol{E}$ $\perp$ [100]) and $\boldsymbol{B}$ $\parallel$ [100]
$Pn'n2'$ (34.158) $\mathbb{Z}_1$	• $\boldsymbol{B}$ $\parallel$ [100]
$Pc1'$ (7.25) $\mathbb{Z}_1$	• strain $\perp$ [100]<br>• ($\boldsymbol{E}$ $\parallel$ [010] or $\boldsymbol{E}$ $\perp$ [100])

Table. S 268: Subgroups of $Pn'n2'$ (34.158) that can be realized by applying $\boldsymbol{E}/\boldsymbol{B}$ fields and/or uniaxial strains.

Subgroup & SI group	Perturbation
Pc (7.24) $\mathbb{Z}_1$	• strain $\perp$ [010]<br>• ($\boldsymbol{E}$ $\parallel$ [100] or $\boldsymbol{E}$ $\perp$ [010])
$P2'$ (3.3) $\mathbb{Z}_1$	• strain $\perp$ [001]<br>• ($\boldsymbol{B}$ $\parallel$ [100] or $\boldsymbol{B}$ $\perp$ [001])
Pc' (7.26) $\mathbb{Z}_1$	• strain $\perp$ [100]<br>• ($\boldsymbol{B}$ $\parallel$ [001] or $\boldsymbol{B}$ $\perp$ [100])<br>• ($\boldsymbol{E}$ $\parallel$ [010] or $\boldsymbol{E}$ $\perp$ [100])

Table. S 269: Subgroups of $Pn'n'2$ (34.159) that can be realized by applying $\boldsymbol{E}/\boldsymbol{B}$ fields and/or uniaxial strains.

Subgroup & SI group	Perturbation

Continued on next page

...continued

Subgroup & SI group	Perturbation
Pc' (7.26) $\mathbb{Z}_1$	• strain ⊥ [010]<br>• ($\boldsymbol{B}$ ‖ [100] or $\boldsymbol{B}$ ⊥ [010])<br>• ($\boldsymbol{E}$ ‖ [100] or $\boldsymbol{E}$ ⊥ [010])
$P2$ (3.1) $\mathbb{Z}_2$	• strain ⊥ [001]
Pc' (7.26) $\mathbb{Z}_1$	• strain ⊥ [100]<br>• ($\boldsymbol{B}$ ‖ [010] or $\boldsymbol{B}$ ⊥ [100])<br>• ($\boldsymbol{E}$ ‖ [010] or $\boldsymbol{E}$ ⊥ [100])

Table. S 270: Subgroups of P_ann2 (34.160) that can be realized by applying $\boldsymbol{E}/\boldsymbol{B}$ fields and/or uniaxial strains.

Subgroup & SI group	Perturbation
Pc' (7.26) $\mathbb{Z}_1$	• $\boldsymbol{B}$ ‖ [100] and strain ⊥ [010]<br>• $\boldsymbol{B}$ ‖ [001] and strain ⊥ [010]<br>• $\boldsymbol{B}$ ⊥ [010]<br>• ($\boldsymbol{E}$ ‖ [100] or $\boldsymbol{E}$ ⊥ [010]) and $\boldsymbol{B}$ ‖ [100]<br>• ($\boldsymbol{E}$ ‖ [100] or $\boldsymbol{E}$ ⊥ [010]) and $\boldsymbol{B}$ ‖ [001]
P_S1 (1.3) $\mathbb{Z}_1$	• strain in generic direction<br>• ($\boldsymbol{E}$ ‖ [100] or $\boldsymbol{E}$ ⊥ [010]) and strain ⊥ [100]<br>• ($\boldsymbol{E}$ ‖ [100] or $\boldsymbol{E}$ ⊥ [010]) and strain ⊥ [001]<br>• ($\boldsymbol{E}$ ‖ [010] or $\boldsymbol{E}$ ⊥ [100]) and strain ⊥ [010]<br>• ($\boldsymbol{E}$ ‖ [010] or $\boldsymbol{E}$ ⊥ [100]) and strain ⊥ [001]<br>• $\boldsymbol{E}$ in generic direction
Pc (7.24) $\mathbb{Z}_1$	• $\boldsymbol{B}$ ‖ [010] and strain ⊥ [010]<br>• ($\boldsymbol{E}$ ‖ [100] or $\boldsymbol{E}$ ⊥ [010]) and $\boldsymbol{B}$ ‖ [010]
P_ac (7.27) $\mathbb{Z}_1$	• strain ⊥ [010]<br>• ($\boldsymbol{E}$ ‖ [100] or $\boldsymbol{E}$ ⊥ [010])
Pc' (7.26) $\mathbb{Z}_1$	• $\boldsymbol{B}$ ‖ [010] and strain ⊥ [100]<br>• $\boldsymbol{B}$ ‖ [001] and strain ⊥ [100]<br>• $\boldsymbol{B}$ ⊥ [100]<br>• ($\boldsymbol{E}$ ‖ [010] or $\boldsymbol{E}$ ⊥ [100]) and $\boldsymbol{B}$ ‖ [010]<br>• ($\boldsymbol{E}$ ‖ [010] or $\boldsymbol{E}$ ⊥ [100]) and $\boldsymbol{B}$ ‖ [001]
$P2$ (3.1) $\mathbb{Z}_2$	• $\boldsymbol{B}$ ‖ [001] and strain ⊥ [001]
$Pn'c'2$ (30.115) $\mathbb{Z}_1$	• $\boldsymbol{B}$ ‖ [001]

Continued on next page

...continued

Subgroup & SI group	Perturbation
Pc (7.24) $\mathbb{Z}_1$	• $\boldsymbol{B}$ ∥ [100] and strain ⊥ [100]<br>• ($\boldsymbol{E}$ ∥ [010] or $\boldsymbol{E}$ ⊥ [100]) and $\boldsymbol{B}$ ∥ [100]
P_bc (7.29) $\mathbb{Z}_1$	• strain ⊥ [100]<br>• ($\boldsymbol{E}$ ∥ [010] or $\boldsymbol{E}$ ⊥ [100])
$P2'$ (3.3) $\mathbb{Z}_1$	• $\boldsymbol{B}$ ∥ [100] and strain ⊥ [001]<br>• $\boldsymbol{B}$ ∥ [010] and strain ⊥ [001]<br>• $\boldsymbol{B}$ ⊥ [001]
$Pn'n2'$ (34.158) $\mathbb{Z}_1$	• $\boldsymbol{B}$ ∥ [010]
P_a2 (3.4) $\mathbb{Z}_2$	• strain ⊥ [001]
$Pnc'2'$ (30.114) $\mathbb{Z}_1$	• $\boldsymbol{B}$ ∥ [100]

Table. S 271: Subgroups of P_cnn2 (34.161) that can be realized by applying $\boldsymbol{E}/\boldsymbol{B}$ fields and/or uniaxial strains.

Subgroup & SI group	Perturbation
P_S1 (1.3) $\mathbb{Z}_1$	• strain in generic direction<br>• ($\boldsymbol{E}$ ∥ [100] or $\boldsymbol{E}$ ⊥ [010]) and strain ⊥ [100]<br>• ($\boldsymbol{E}$ ∥ [100] or $\boldsymbol{E}$ ⊥ [010]) and strain ⊥ [001]<br>• ($\boldsymbol{E}$ ∥ [010] or $\boldsymbol{E}$ ⊥ [100]) and strain ⊥ [010]<br>• ($\boldsymbol{E}$ ∥ [010] or $\boldsymbol{E}$ ⊥ [100]) and strain ⊥ [001]<br>• $\boldsymbol{E}$ in generic direction
Pc' (7.26) $\mathbb{Z}_1$	• $\boldsymbol{B}$ ∥ [100] and strain ⊥ [010]<br>• $\boldsymbol{B}$ ∥ [001] and strain ⊥ [010]<br>• $\boldsymbol{B}$ ⊥ [010]<br>• ($\boldsymbol{E}$ ∥ [100] or $\boldsymbol{E}$ ⊥ [010]) and $\boldsymbol{B}$ ∥ [100]<br>• ($\boldsymbol{E}$ ∥ [100] or $\boldsymbol{E}$ ⊥ [010]) and $\boldsymbol{B}$ ∥ [001]
Pc (7.24) $\mathbb{Z}_1$	• $\boldsymbol{B}$ ∥ [010] and strain ⊥ [010]<br>• ($\boldsymbol{E}$ ∥ [100] or $\boldsymbol{E}$ ⊥ [010]) and $\boldsymbol{B}$ ∥ [010]
P_ac (7.27) $\mathbb{Z}_1$	• strain ⊥ [010]<br>• ($\boldsymbol{E}$ ∥ [100] or $\boldsymbol{E}$ ⊥ [010])
$P2$ (3.1) $\mathbb{Z}_2$	• $\boldsymbol{B}$ ∥ [001] and strain ⊥ [001]

Continued on next page

...continued

Subgroup & SI group	Perturbation
$P2_1'$ (4.9) $\mathbb{Z}_1$	• $\boldsymbol{B} \parallel [100]$ and strain $\perp [001]$<br>• $\boldsymbol{B} \parallel [010]$ and strain $\perp [001]$<br>• $\boldsymbol{B} \perp [001]$
P_b2 (3.5) $\mathbb{Z}_2$	• strain $\perp [001]$
Pc' (7.26) $\mathbb{Z}_1$	• $\boldsymbol{B} \parallel [010]$ and strain $\perp [100]$<br>• $\boldsymbol{B} \parallel [001]$ and strain $\perp [100]$<br>• $\boldsymbol{B} \perp [100]$<br>• ($\boldsymbol{E} \parallel [010]$ or $\boldsymbol{E} \perp [100]$) and $\boldsymbol{B} \parallel [010]$<br>• ($\boldsymbol{E} \parallel [010]$ or $\boldsymbol{E} \perp [100]$) and $\boldsymbol{B} \parallel [001]$
$Pb'a'2$ (32.138) $\mathbb{Z}_2$	• $\boldsymbol{B} \parallel [001]$
$Pna'2_1'$ (33.147) $\mathbb{Z}_1$	• $\boldsymbol{B} \parallel [010]$
Pc (7.24) $\mathbb{Z}_1$	• $\boldsymbol{B} \parallel [100]$ and strain $\perp [100]$<br>• ($\boldsymbol{E} \parallel [010]$ or $\boldsymbol{E} \perp [100]$) and $\boldsymbol{B} \parallel [100]$
$Pna'2_1'$ (33.147) $\mathbb{Z}_1$	• $\boldsymbol{B} \parallel [100]$
P_ac (7.27) $\mathbb{Z}_1$	• strain $\perp [100]$<br>• ($\boldsymbol{E} \parallel [010]$ or $\boldsymbol{E} \perp [100]$)

Table. S 272: Subgroups of P_Ann2 (34.162) that can be realized by applying $\boldsymbol{E}/\boldsymbol{B}$ fields and/or uniaxial strains.

Subgroup & SI group	Perturbation
P_S1 (1.3) $\mathbb{Z}_1$	• strain in generic direction<br>• ($\boldsymbol{E} \parallel [100]$ or $\boldsymbol{E} \perp [010]$) and strain $\perp [100]$<br>• ($\boldsymbol{E} \parallel [100]$ or $\boldsymbol{E} \perp [010]$) and strain $\perp [001]$<br>• ($\boldsymbol{E} \parallel [010]$ or $\boldsymbol{E} \perp [100]$) and strain $\perp [010]$<br>• ($\boldsymbol{E} \parallel [010]$ or $\boldsymbol{E} \perp [100]$) and strain $\perp [001]$<br>• $\boldsymbol{E}$ in generic direction
Pc' (7.26) $\mathbb{Z}_1$	• $\boldsymbol{B} \parallel [100]$ and strain $\perp [010]$<br>• $\boldsymbol{B} \parallel [001]$ and strain $\perp [010]$<br>• $\boldsymbol{B} \perp [010]$<br>• ($\boldsymbol{E} \parallel [100]$ or $\boldsymbol{E} \perp [010]$) and $\boldsymbol{B} \parallel [100]$<br>• ($\boldsymbol{E} \parallel [100]$ or $\boldsymbol{E} \perp [010]$) and $\boldsymbol{B} \parallel [001]$
Pc (7.24) $\mathbb{Z}_1$	• $\boldsymbol{B} \parallel [010]$ and strain $\perp [010]$<br>• ($\boldsymbol{E} \parallel [100]$ or $\boldsymbol{E} \perp [010]$) and $\boldsymbol{B} \parallel [010]$

Continued on next page

. . . continued

Subgroup & SI group	Perturbation
P_Cc (7.30) $\mathbb{Z}_1$	• strain ⊥ [010]<br>• ($\boldsymbol{E}$ ∥ [100] or $\boldsymbol{E}$ ⊥ [010])
$P2$ (3.1) $\mathbb{Z}_2$	• $\boldsymbol{B}$ ∥ [001] and strain ⊥ [001]
$P2_1'$ (4.9) $\mathbb{Z}_1$	• $\boldsymbol{B}$ ∥ [100] and strain ⊥ [001]<br>• $\boldsymbol{B}$ ∥ [010] and strain ⊥ [001]<br>• $\boldsymbol{B}$ ⊥ [001]
P_C2 (3.6) $\mathbb{Z}_1$	• strain ⊥ [001]
Pm' (6.20) $\mathbb{Z}_1$	• $\boldsymbol{B}$ ∥ [010] and strain ⊥ [100]<br>• $\boldsymbol{B}$ ∥ [001] and strain ⊥ [100]<br>• $\boldsymbol{B}$ ⊥ [100]<br>• ($\boldsymbol{E}$ ∥ [010] or $\boldsymbol{E}$ ⊥ [100]) and $\boldsymbol{B}$ ∥ [010]<br>• ($\boldsymbol{E}$ ∥ [010] or $\boldsymbol{E}$ ⊥ [100]) and $\boldsymbol{B}$ ∥ [001]
$Pm'a'2$ (28.91) $\mathbb{Z}_2$	• $\boldsymbol{B}$ ∥ [001]
$Pm'n2_1'$ (31.125) $\mathbb{Z}_1$	• $\boldsymbol{B}$ ∥ [010]
Pc (7.24) $\mathbb{Z}_1$	• $\boldsymbol{B}$ ∥ [100] and strain ⊥ [100]<br>• ($\boldsymbol{E}$ ∥ [010] or $\boldsymbol{E}$ ⊥ [100]) and $\boldsymbol{B}$ ∥ [100]
$Pna'2_1'$ (33.147) $\mathbb{Z}_1$	• $\boldsymbol{B}$ ∥ [100]
P_cc (7.28) $\mathbb{Z}_1$	• strain ⊥ [100]<br>• ($\boldsymbol{E}$ ∥ [010] or $\boldsymbol{E}$ ⊥ [100])

Table. S 273: Subgroups of P_Cnn2 (34.163) that can be realized by applying $\boldsymbol{E}/\boldsymbol{B}$ fields and/or uniaxial strains.

Subgroup & SI group	Perturbation
Pc' (7.26) $\mathbb{Z}_1$	• $\boldsymbol{B}$ ∥ [100] and strain ⊥ [010]<br>• $\boldsymbol{B}$ ∥ [001] and strain ⊥ [010]<br>• $\boldsymbol{B}$ ⊥ [010]<br>• ($\boldsymbol{E}$ ∥ [100] or $\boldsymbol{E}$ ⊥ [010]) and $\boldsymbol{B}$ ∥ [100]<br>• ($\boldsymbol{E}$ ∥ [100] or $\boldsymbol{E}$ ⊥ [010]) and $\boldsymbol{B}$ ∥ [001]

Continued on next page

...continued

Subgroup & SI group	Perturbation
P_S1 (1.3) $\mathbb{Z}_1$	• strain in generic direction<br>• ($\boldsymbol{E} \parallel [100]$ or $\boldsymbol{E} \perp [010]$) and strain $\perp [100]$<br>• ($\boldsymbol{E} \parallel [100]$ or $\boldsymbol{E} \perp [010]$) and strain $\perp [001]$<br>• ($\boldsymbol{E} \parallel [010]$ or $\boldsymbol{E} \perp [100]$) and strain $\perp [010]$<br>• ($\boldsymbol{E} \parallel [010]$ or $\boldsymbol{E} \perp [100]$) and strain $\perp [001]$<br>• $\boldsymbol{E}$ in generic direction
Pc (7.24) $\mathbb{Z}_1$	• $\boldsymbol{B} \parallel [010]$ and strain $\perp [010]$<br>• ($\boldsymbol{E} \parallel [100]$ or $\boldsymbol{E} \perp [010]$) and $\boldsymbol{B} \parallel [010]$
P_Cc (7.30) $\mathbb{Z}_1$	• strain $\perp [010]$<br>• ($\boldsymbol{E} \parallel [100]$ or $\boldsymbol{E} \perp [010]$)
Pc' (7.26) $\mathbb{Z}_1$	• $\boldsymbol{B} \parallel [010]$ and strain $\perp [100]$<br>• $\boldsymbol{B} \parallel [001]$ and strain $\perp [100]$<br>• $\boldsymbol{B} \perp [100]$<br>• ($\boldsymbol{E} \parallel [010]$ or $\boldsymbol{E} \perp [100]$) and $\boldsymbol{B} \parallel [010]$<br>• ($\boldsymbol{E} \parallel [010]$ or $\boldsymbol{E} \perp [100]$) and $\boldsymbol{B} \parallel [001]$
$P2$ (3.1) $\mathbb{Z}_2$	• $\boldsymbol{B} \parallel [001]$ and strain $\perp [001]$
$Pc'c'2$ (27.81) $\mathbb{Z}_2$	• $\boldsymbol{B} \parallel [001]$
Pc (7.24) $\mathbb{Z}_1$	• $\boldsymbol{B} \parallel [100]$ and strain $\perp [100]$<br>• ($\boldsymbol{E} \parallel [010]$ or $\boldsymbol{E} \perp [100]$) and $\boldsymbol{B} \parallel [100]$
P_Cc (7.30) $\mathbb{Z}_1$	• strain $\perp [100]$<br>• ($\boldsymbol{E} \parallel [010]$ or $\boldsymbol{E} \perp [100]$)
$P2'$ (3.3) $\mathbb{Z}_1$	• $\boldsymbol{B} \parallel [100]$ and strain $\perp [001]$<br>• $\boldsymbol{B} \parallel [010]$ and strain $\perp [001]$<br>• $\boldsymbol{B} \perp [001]$
$Pnc'2'$ (30.114) $\mathbb{Z}_1$	• $\boldsymbol{B} \parallel [010]$
P_a2 (3.4) $\mathbb{Z}_2$	• strain $\perp [001]$
$Pnc'2'$ (30.114) $\mathbb{Z}_1$	• $\boldsymbol{B} \parallel [100]$

Table. S 274: Subgroups of P_Inn2 (34.164) that can be realized by applying $\boldsymbol{E}/\boldsymbol{B}$ fields and/or uniaxial strains.

Subgroup & SI group	Perturbation

Continued on next page

... continued

Subgroup & SI group	Perturbation
Pm' (6.20) $\mathbb{Z}_1$	• $\boldsymbol{B} \parallel [100]$ and strain $\perp [010]$<br>• $\boldsymbol{B} \parallel [001]$ and strain $\perp [010]$<br>• $\boldsymbol{B} \perp [010]$<br>• ($\boldsymbol{E} \parallel [100]$ or $\boldsymbol{E} \perp [010]$) and $\boldsymbol{B} \parallel [100]$<br>• ($\boldsymbol{E} \parallel [100]$ or $\boldsymbol{E} \perp [010]$) and $\boldsymbol{B} \parallel [001]$
P_S1 (1.3) $\mathbb{Z}_1$	• strain in generic direction<br>• ($\boldsymbol{E} \parallel [100]$ or $\boldsymbol{E} \perp [010]$) and strain $\perp [100]$<br>• ($\boldsymbol{E} \parallel [100]$ or $\boldsymbol{E} \perp [010]$) and strain $\perp [001]$<br>• ($\boldsymbol{E} \parallel [010]$ or $\boldsymbol{E} \perp [100]$) and strain $\perp [010]$<br>• ($\boldsymbol{E} \parallel [010]$ or $\boldsymbol{E} \perp [100]$) and strain $\perp [001]$<br>• $\boldsymbol{E}$ in generic direction
Pc (7.24) $\mathbb{Z}_1$	• $\boldsymbol{B} \parallel [010]$ and strain $\perp [010]$<br>• ($\boldsymbol{E} \parallel [100]$ or $\boldsymbol{E} \perp [010]$) and $\boldsymbol{B} \parallel [010]$
P_Ac (7.31) $\mathbb{Z}_1$	• strain $\perp [010]$<br>• ($\boldsymbol{E} \parallel [100]$ or $\boldsymbol{E} \perp [010]$)
Pm' (6.20) $\mathbb{Z}_1$	• $\boldsymbol{B} \parallel [010]$ and strain $\perp [100]$<br>• $\boldsymbol{B} \parallel [001]$ and strain $\perp [100]$<br>• $\boldsymbol{B} \perp [100]$<br>• ($\boldsymbol{E} \parallel [010]$ or $\boldsymbol{E} \perp [100]$) and $\boldsymbol{B} \parallel [010]$<br>• ($\boldsymbol{E} \parallel [010]$ or $\boldsymbol{E} \perp [100]$) and $\boldsymbol{B} \parallel [001]$
$P2$ (3.1) $\mathbb{Z}_2$	• $\boldsymbol{B} \parallel [001]$ and strain $\perp [001]$
$Pm'm'2$ (25.60) $\mathbb{Z}_2$	• $\boldsymbol{B} \parallel [001]$
Pc (7.24) $\mathbb{Z}_1$	• $\boldsymbol{B} \parallel [100]$ and strain $\perp [100]$<br>• ($\boldsymbol{E} \parallel [010]$ or $\boldsymbol{E} \perp [100]$) and $\boldsymbol{B} \parallel [100]$
P_Ac (7.31) $\mathbb{Z}_1$	• strain $\perp [100]$<br>• ($\boldsymbol{E} \parallel [010]$ or $\boldsymbol{E} \perp [100]$)
$P2_1'$ (4.9) $\mathbb{Z}_1$	• $\boldsymbol{B} \parallel [100]$ and strain $\perp [001]$<br>• $\boldsymbol{B} \parallel [010]$ and strain $\perp [001]$<br>• $\boldsymbol{B} \perp [001]$
$Pm'n2_1'$ (31.125) $\mathbb{Z}_1$	• $\boldsymbol{B} \parallel [010]$
P_C2 (3.6) $\mathbb{Z}_1$	• strain $\perp [001]$
$Pm'n2_1'$ (31.125) $\mathbb{Z}_1$	• $\boldsymbol{B} \parallel [100]$

Table. S 275: Subgroups of $Cmm2$ (35.165) that can be realized by applying $\boldsymbol{E}/\boldsymbol{B}$ fields and/or uniaxial strains.

Subgroup & SI group	Perturbation
Cm (8.32) $\mathbb{Z}_1$	• strain $\perp$ [010]<br>• $\boldsymbol{B} \parallel$ [010]<br>• ($\boldsymbol{E} \parallel$ [100] or $\boldsymbol{E} \perp$ [010])
Cm (8.32) $\mathbb{Z}_1$	• strain $\perp$ [100]<br>• $\boldsymbol{B} \parallel$ [100]<br>• ($\boldsymbol{E} \parallel$ [010] or $\boldsymbol{E} \perp$ [100])
$P2$ (3.1) $\mathbb{Z}_2$	• strain $\perp$ [001]<br>• $\boldsymbol{B} \parallel$ [001]

Table. S 276: Subgroups of $Cmm21'$ (35.166) that can be realized by applying $\boldsymbol{E}/\boldsymbol{B}$ fields and/or uniaxial strains.

Subgroup & SI group	Perturbation
$P11'$ (1.2) $\mathbb{Z}_1$	• strain in generic direction<br>• ($\boldsymbol{E} \parallel$ [100] or $\boldsymbol{E} \perp$ [010]) and strain $\perp$ [100]<br>• ($\boldsymbol{E} \parallel$ [100] or $\boldsymbol{E} \perp$ [010]) and strain $\perp$ [001]<br>• ($\boldsymbol{E} \parallel$ [010] or $\boldsymbol{E} \perp$ [100]) and strain $\perp$ [010]<br>• ($\boldsymbol{E} \parallel$ [010] or $\boldsymbol{E} \perp$ [100]) and strain $\perp$ [001]<br>• $\boldsymbol{E}$ in generic direction
Cm' (8.34) $\mathbb{Z}_1$	• $\boldsymbol{B} \parallel$ [100] and strain $\perp$ [010]<br>• $\boldsymbol{B} \parallel$ [001] and strain $\perp$ [010]<br>• $\boldsymbol{B} \perp$ [010]<br>• ($\boldsymbol{E} \parallel$ [100] or $\boldsymbol{E} \perp$ [010]) and $\boldsymbol{B} \parallel$ [100]<br>• ($\boldsymbol{E} \parallel$ [100] or $\boldsymbol{E} \perp$ [010]) and $\boldsymbol{B} \parallel$ [001]
Cm (8.32) $\mathbb{Z}_1$	• $\boldsymbol{B} \parallel$ [010] and strain $\perp$ [010]<br>• ($\boldsymbol{E} \parallel$ [100] or $\boldsymbol{E} \perp$ [010]) and $\boldsymbol{B} \parallel$ [010]
$Cm1'$ (8.33) $\mathbb{Z}_1$	• strain $\perp$ [010]<br>• ($\boldsymbol{E} \parallel$ [100] or $\boldsymbol{E} \perp$ [010])
Cm' (8.34) $\mathbb{Z}_1$	• $\boldsymbol{B} \parallel$ [010] and strain $\perp$ [100]<br>• $\boldsymbol{B} \parallel$ [001] and strain $\perp$ [100]<br>• $\boldsymbol{B} \perp$ [100]<br>• ($\boldsymbol{E} \parallel$ [010] or $\boldsymbol{E} \perp$ [100]) and $\boldsymbol{B} \parallel$ [010]<br>• ($\boldsymbol{E} \parallel$ [010] or $\boldsymbol{E} \perp$ [100]) and $\boldsymbol{B} \parallel$ [001]

Continued on next page

. . . continued

Subgroup & SI group	Perturbation
Cm (8.32) $\mathbb{Z}_1$	• $\boldsymbol{B}$ ∥ [100] and strain ⊥ [100]<br>• ($\boldsymbol{E}$ ∥ [010] or $\boldsymbol{E}$ ⊥ [100]) and $\boldsymbol{B}$ ∥ [100]
$Cm1'$ (8.33) $\mathbb{Z}_1$	• strain ⊥ [100]<br>• ($\boldsymbol{E}$ ∥ [010] or $\boldsymbol{E}$ ⊥ [100])
$P2'$ (3.3) $\mathbb{Z}_1$	• $\boldsymbol{B}$ ∥ [100] and strain ⊥ [001]<br>• $\boldsymbol{B}$ ∥ [010] and strain ⊥ [001]<br>• $\boldsymbol{B}$ ⊥ [001]
$Cm'm2'$ (35.167) $\mathbb{Z}_1$	• $\boldsymbol{B}$ ∥ [010]
$Cm'm2'$ (35.167) $\mathbb{Z}_1$	• $\boldsymbol{B}$ ∥ [100]
$P2$ (3.1) $\mathbb{Z}_2$	• $\boldsymbol{B}$ ∥ [001] and strain ⊥ [001]
$Cm'm'2$ (35.168) $\mathbb{Z}_2$	• $\boldsymbol{B}$ ∥ [001]
$P21'$ (3.2) $\mathbb{Z}_2$	• strain ⊥ [001]

Table. S 277: Subgroups of $Cm'm2'$ (35.167) that can be realized by applying $\boldsymbol{E}/\boldsymbol{B}$ fields and/or uniaxial strains.

Subgroup & SI group	Perturbation
Cm (8.32) $\mathbb{Z}_1$	• strain ⊥ [010]<br>• ($\boldsymbol{E}$ ∥ [100] or $\boldsymbol{E}$ ⊥ [010])
Cm' (8.34) $\mathbb{Z}_1$	• strain ⊥ [100]<br>• ($\boldsymbol{B}$ ∥ [001] or $\boldsymbol{B}$ ⊥ [100])<br>• ($\boldsymbol{E}$ ∥ [010] or $\boldsymbol{E}$ ⊥ [100])
$P2'$ (3.3) $\mathbb{Z}_1$	• strain ⊥ [001]<br>• ($\boldsymbol{B}$ ∥ [100] or $\boldsymbol{B}$ ⊥ [001])

Table. S 278: Subgroups of $Cm'm'2$ (35.168) that can be realized by applying $\boldsymbol{E}/\boldsymbol{B}$ fields and/or uniaxial strains.

Subgroup & SI group	Perturbation

Continued on next page

...continued

Subgroup & SI group	Perturbation
Cm' (8.34) $\mathbb{Z}_1$	• strain $\perp$ [010]<br>• ($\boldsymbol{B}$ $\parallel$ [100] or $\boldsymbol{B}$ $\perp$ [010])<br>• ($\boldsymbol{E}$ $\parallel$ [100] or $\boldsymbol{E}$ $\perp$ [010])
Cm' (8.34) $\mathbb{Z}_1$	• strain $\perp$ [100]<br>• ($\boldsymbol{B}$ $\parallel$ [010] or $\boldsymbol{B}$ $\perp$ [100])<br>• ($\boldsymbol{E}$ $\parallel$ [010] or $\boldsymbol{E}$ $\perp$ [100])
$P2$ (3.1) $\mathbb{Z}_2$	• strain $\perp$ [001]

Table. S 279: Subgroups of C_cmm2 (35.169) that can be realized by applying $\boldsymbol{E}/\boldsymbol{B}$ fields and/or uniaxial strains.

Subgroup & SI group	Perturbation
P_S1 (1.3) $\mathbb{Z}_1$	• strain in generic direction<br>• ($\boldsymbol{E}$ $\parallel$ [100] or $\boldsymbol{E}$ $\perp$ [010]) and strain $\perp$ [100]<br>• ($\boldsymbol{E}$ $\parallel$ [100] or $\boldsymbol{E}$ $\perp$ [010]) and strain $\perp$ [001]<br>• ($\boldsymbol{E}$ $\parallel$ [010] or $\boldsymbol{E}$ $\perp$ [100]) and strain $\perp$ [010]<br>• ($\boldsymbol{E}$ $\parallel$ [010] or $\boldsymbol{E}$ $\perp$ [100]) and strain $\perp$ [001]<br>• $\boldsymbol{E}$ in generic direction
Cm (8.32) $\mathbb{Z}_1$	• $\boldsymbol{B}$ $\parallel$ [010] and strain $\perp$ [010]<br>• ($\boldsymbol{E}$ $\parallel$ [100] or $\boldsymbol{E}$ $\perp$ [010]) and $\boldsymbol{B}$ $\parallel$ [010]
Cc' (9.39) $\mathbb{Z}_1$	• $\boldsymbol{B}$ $\parallel$ [100] and strain $\perp$ [010]<br>• $\boldsymbol{B}$ $\parallel$ [001] and strain $\perp$ [010]<br>• $\boldsymbol{B}$ $\perp$ [010]<br>• ($\boldsymbol{E}$ $\parallel$ [100] or $\boldsymbol{E}$ $\perp$ [010]) and $\boldsymbol{B}$ $\parallel$ [100]<br>• ($\boldsymbol{E}$ $\parallel$ [100] or $\boldsymbol{E}$ $\perp$ [010]) and $\boldsymbol{B}$ $\parallel$ [001]
C_cm (8.35) $\mathbb{Z}_1$	• strain $\perp$ [010]<br>• ($\boldsymbol{E}$ $\parallel$ [100] or $\boldsymbol{E}$ $\perp$ [010])
Cm (8.32) $\mathbb{Z}_1$	• $\boldsymbol{B}$ $\parallel$ [100] and strain $\perp$ [100]<br>• ($\boldsymbol{E}$ $\parallel$ [010] or $\boldsymbol{E}$ $\perp$ [100]) and $\boldsymbol{B}$ $\parallel$ [100]
Cc' (9.39) $\mathbb{Z}_1$	• $\boldsymbol{B}$ $\parallel$ [010] and strain $\perp$ [100]<br>• $\boldsymbol{B}$ $\parallel$ [001] and strain $\perp$ [100]<br>• $\boldsymbol{B}$ $\perp$ [100]<br>• ($\boldsymbol{E}$ $\parallel$ [010] or $\boldsymbol{E}$ $\perp$ [100]) and $\boldsymbol{B}$ $\parallel$ [010]<br>• ($\boldsymbol{E}$ $\parallel$ [010] or $\boldsymbol{E}$ $\perp$ [100]) and $\boldsymbol{B}$ $\parallel$ [001]

Continued on next page

. . . continued

Subgroup & SI group	Perturbation
C_cm (8.35) $\mathbb{Z}_1$	• strain ⊥ [100]<br>• ($\boldsymbol{E}$ ∥ [010] or $\boldsymbol{E}$ ⊥ [100])
$P2$ (3.1) $\mathbb{Z}_2$	• $\boldsymbol{B}$ ∥ [001] and strain ⊥ [001]
$Cc'c'2$ (37.183) $\mathbb{Z}_2$	• $\boldsymbol{B}$ ∥ [001]
$P2_1'$ (4.9) $\mathbb{Z}_1$	• $\boldsymbol{B}$ ∥ [100] and strain ⊥ [001]<br>• $\boldsymbol{B}$ ∥ [010] and strain ⊥ [001]<br>• $\boldsymbol{B}$ ⊥ [001]
$Cmc'2_1'$ (36.175) $\mathbb{Z}_1$	• $\boldsymbol{B}$ ∥ [100]
$Cmc'2_1'$ (36.175) $\mathbb{Z}_1$	• $\boldsymbol{B}$ ∥ [010]
P_b2 (3.5) $\mathbb{Z}_2$	• strain ⊥ [001]

Table. S 280: Subgroups of C_amm2 (35.170) that can be realized by applying $\boldsymbol{E}/\boldsymbol{B}$ fields and/or uniaxial strains.

Subgroup & SI group	Perturbation
P_S1 (1.3) $\mathbb{Z}_1$	• strain in generic direction<br>• ($\boldsymbol{E}$ ∥ [100] or $\boldsymbol{E}$ ⊥ [010]) and strain ⊥ [100]<br>• ($\boldsymbol{E}$ ∥ [100] or $\boldsymbol{E}$ ⊥ [010]) and strain ⊥ [001]<br>• ($\boldsymbol{E}$ ∥ [010] or $\boldsymbol{E}$ ⊥ [100]) and strain ⊥ [010]<br>• ($\boldsymbol{E}$ ∥ [010] or $\boldsymbol{E}$ ⊥ [100]) and strain ⊥ [001]<br>• $\boldsymbol{E}$ in generic direction
Cm' (8.34) $\mathbb{Z}_1$	• $\boldsymbol{B}$ ∥ [100] and strain ⊥ [010]<br>• $\boldsymbol{B}$ ∥ [001] and strain ⊥ [010]<br>• $\boldsymbol{B}$ ⊥ [010]<br>• ($\boldsymbol{E}$ ∥ [100] or $\boldsymbol{E}$ ⊥ [010]) and $\boldsymbol{B}$ ∥ [100]<br>• ($\boldsymbol{E}$ ∥ [100] or $\boldsymbol{E}$ ⊥ [010]) and $\boldsymbol{B}$ ∥ [001]
Cm (8.32) $\mathbb{Z}_1$	• $\boldsymbol{B}$ ∥ [010] and strain ⊥ [010]<br>• ($\boldsymbol{E}$ ∥ [100] or $\boldsymbol{E}$ ⊥ [010]) and $\boldsymbol{B}$ ∥ [010]
C_am (8.36) $\mathbb{Z}_1$	• strain ⊥ [010]<br>• ($\boldsymbol{E}$ ∥ [100] or $\boldsymbol{E}$ ⊥ [010])

Continued on next page

...continued

Subgroup & SI group	Perturbation
Cm' (8.34) $\mathbb{Z}_1$	• $\boldsymbol{B}$ ∥ [010] and strain ⊥ [100]<br>• $\boldsymbol{B}$ ∥ [001] and strain ⊥ [100]<br>• $\boldsymbol{B}$ ⊥ [100]<br>• ($\boldsymbol{E}$ ∥ [010] or $\boldsymbol{E}$ ⊥ [100]) and $\boldsymbol{B}$ ∥ [010]<br>• ($\boldsymbol{E}$ ∥ [010] or $\boldsymbol{E}$ ⊥ [100]) and $\boldsymbol{B}$ ∥ [001]
Cm (8.32) $\mathbb{Z}_1$	• $\boldsymbol{B}$ ∥ [100] and strain ⊥ [100]<br>• ($\boldsymbol{E}$ ∥ [010] or $\boldsymbol{E}$ ⊥ [100]) and $\boldsymbol{B}$ ∥ [100]
C_am (8.36) $\mathbb{Z}_1$	• strain ⊥ [100]<br>• ($\boldsymbol{E}$ ∥ [010] or $\boldsymbol{E}$ ⊥ [100])
$P2'$ (3.3) $\mathbb{Z}_1$	• $\boldsymbol{B}$ ∥ [100] and strain ⊥ [001]<br>• $\boldsymbol{B}$ ∥ [010] and strain ⊥ [001]<br>• $\boldsymbol{B}$ ⊥ [001]
$Cm'm2'$ (35.167) $\mathbb{Z}_1$	• $\boldsymbol{B}$ ∥ [010]
$Cm'm2'$ (35.167) $\mathbb{Z}_1$	• $\boldsymbol{B}$ ∥ [100]
$P2$ (3.1) $\mathbb{Z}_2$	• $\boldsymbol{B}$ ∥ [001] and strain ⊥ [001]
$Cm'm'2$ (35.168) $\mathbb{Z}_2$	• $\boldsymbol{B}$ ∥ [001]
P_a2 (3.4) $\mathbb{Z}_2$	• strain ⊥ [001]

Table. S 281: Subgroups of C_Amm2 (35.171) that can be realized by applying $\boldsymbol{E}/\boldsymbol{B}$ fields and/or uniaxial strains.

Subgroup & SI group	Perturbation
P_S1 (1.3) $\mathbb{Z}_1$	• strain in generic direction<br>• ($\boldsymbol{E}$ ∥ [100] or $\boldsymbol{E}$ ⊥ [010]) and strain ⊥ [100]<br>• ($\boldsymbol{E}$ ∥ [100] or $\boldsymbol{E}$ ⊥ [010]) and strain ⊥ [001]<br>• ($\boldsymbol{E}$ ∥ [010] or $\boldsymbol{E}$ ⊥ [100]) and strain ⊥ [010]<br>• ($\boldsymbol{E}$ ∥ [010] or $\boldsymbol{E}$ ⊥ [100]) and strain ⊥ [001]<br>• $\boldsymbol{E}$ in generic direction
Cc' (9.39) $\mathbb{Z}_1$	• $\boldsymbol{B}$ ∥ [100] and strain ⊥ [010]<br>• $\boldsymbol{B}$ ∥ [001] and strain ⊥ [010]<br>• $\boldsymbol{B}$ ⊥ [010]<br>• ($\boldsymbol{E}$ ∥ [100] or $\boldsymbol{E}$ ⊥ [010]) and $\boldsymbol{B}$ ∥ [100]<br>• ($\boldsymbol{E}$ ∥ [100] or $\boldsymbol{E}$ ⊥ [010]) and $\boldsymbol{B}$ ∥ [001]

Continued on next page

...continued

Subgroup & SI group	Perturbation
Cm (8.32) $\mathbb{Z}_1$	• $\boldsymbol{B}$ ∥ [010] and strain ⊥ [010]<br>• ($\boldsymbol{E}$ ∥ [100] or $\boldsymbol{E}$ ⊥ [010]) and $\boldsymbol{B}$ ∥ [010]
C_cm (8.35) $\mathbb{Z}_1$	• strain ⊥ [010]<br>• ($\boldsymbol{E}$ ∥ [100] or $\boldsymbol{E}$ ⊥ [010])
Cc' (9.39) $\mathbb{Z}_1$	• $\boldsymbol{B}$ ∥ [010] and strain ⊥ [100]<br>• $\boldsymbol{B}$ ∥ [001] and strain ⊥ [100]<br>• $\boldsymbol{B}$ ⊥ [100]<br>• ($\boldsymbol{E}$ ∥ [010] or $\boldsymbol{E}$ ⊥ [100]) and $\boldsymbol{B}$ ∥ [010]<br>• ($\boldsymbol{E}$ ∥ [010] or $\boldsymbol{E}$ ⊥ [100]) and $\boldsymbol{B}$ ∥ [001]
Cm (8.32) $\mathbb{Z}_1$	• $\boldsymbol{B}$ ∥ [100] and strain ⊥ [100]<br>• ($\boldsymbol{E}$ ∥ [010] or $\boldsymbol{E}$ ⊥ [100]) and $\boldsymbol{B}$ ∥ [100]
C_cm (8.35) $\mathbb{Z}_1$	• strain ⊥ [100]<br>• ($\boldsymbol{E}$ ∥ [010] or $\boldsymbol{E}$ ⊥ [100])
$P2_1'$ (4.9) $\mathbb{Z}_1$	• $\boldsymbol{B}$ ∥ [100] and strain ⊥ [001]<br>• $\boldsymbol{B}$ ∥ [010] and strain ⊥ [001]<br>• $\boldsymbol{B}$ ⊥ [001]
$Cmc'2_1'$ (36.175) $\mathbb{Z}_1$	• $\boldsymbol{B}$ ∥ [010]
$Cmc'2_1'$ (36.175) $\mathbb{Z}_1$	• $\boldsymbol{B}$ ∥ [100]
$P2$ (3.1) $\mathbb{Z}_2$	• $\boldsymbol{B}$ ∥ [001] and strain ⊥ [001]
$Cc'c'2$ (37.183) $\mathbb{Z}_2$	• $\boldsymbol{B}$ ∥ [001]
P_C2 (3.6) $\mathbb{Z}_1$	• strain ⊥ [001]

Table. S 282: Subgroups of $Cmc2_1$ (36.172) that can be realized by applying $\boldsymbol{E}/\boldsymbol{B}$ fields and/or uniaxial strains.

Subgroup & SI group	Perturbation
Cc (9.37) $\mathbb{Z}_1$	• strain ⊥ [010]<br>• $\boldsymbol{B}$ ∥ [010]<br>• ($\boldsymbol{E}$ ∥ [100] or $\boldsymbol{E}$ ⊥ [010])
Cm (8.32) $\mathbb{Z}_1$	• strain ⊥ [100]<br>• $\boldsymbol{B}$ ∥ [100]<br>• ($\boldsymbol{E}$ ∥ [010] or $\boldsymbol{E}$ ⊥ [100])

Continued on next page

. . . continued

Subgroup & SI group	Perturbation
$P2_1$ (4.7) $\mathbb{Z}_1$	• strain $\perp$ [001]<br>• $\boldsymbol{B}$ $\parallel$ [001]

Table. S 283: Subgroups of $Cmc2_11'$ (36.173) that can be realized by applying $\boldsymbol{E}/\boldsymbol{B}$ fields and/or uniaxial strains.

Subgroup & SI group	Perturbation
$P11'$ (1.2) $\mathbb{Z}_1$	• strain in generic direction<br>• ($\boldsymbol{E}$ $\parallel$ [100] or $\boldsymbol{E}$ $\perp$ [010]) and strain $\perp$ [100]<br>• ($\boldsymbol{E}$ $\parallel$ [100] or $\boldsymbol{E}$ $\perp$ [010]) and strain $\perp$ [001]<br>• ($\boldsymbol{E}$ $\parallel$ [010] or $\boldsymbol{E}$ $\perp$ [100]) and strain $\perp$ [010]<br>• ($\boldsymbol{E}$ $\parallel$ [010] or $\boldsymbol{E}$ $\perp$ [100]) and strain $\perp$ [001]<br>• $\boldsymbol{E}$ in generic direction
Cc' (9.39) $\mathbb{Z}_1$	• $\boldsymbol{B}$ $\parallel$ [100] and strain $\perp$ [010]<br>• $\boldsymbol{B}$ $\parallel$ [001] and strain $\perp$ [010]<br>• $\boldsymbol{B}$ $\perp$ [010]<br>• ($\boldsymbol{E}$ $\parallel$ [100] or $\boldsymbol{E}$ $\perp$ [010]) and $\boldsymbol{B}$ $\parallel$ [100]<br>• ($\boldsymbol{E}$ $\parallel$ [100] or $\boldsymbol{E}$ $\perp$ [010]) and $\boldsymbol{B}$ $\parallel$ [001]
Cc (9.37) $\mathbb{Z}_1$	• $\boldsymbol{B}$ $\parallel$ [010] and strain $\perp$ [010]<br>• ($\boldsymbol{E}$ $\parallel$ [100] or $\boldsymbol{E}$ $\perp$ [010]) and $\boldsymbol{B}$ $\parallel$ [010]
$Cc1'$ (9.38) $\mathbb{Z}_1$	• strain $\perp$ [010]<br>• ($\boldsymbol{E}$ $\parallel$ [100] or $\boldsymbol{E}$ $\perp$ [010])
Cm' (8.34) $\mathbb{Z}_1$	• $\boldsymbol{B}$ $\parallel$ [010] and strain $\perp$ [100]<br>• $\boldsymbol{B}$ $\parallel$ [001] and strain $\perp$ [100]<br>• $\boldsymbol{B}$ $\perp$ [100]<br>• ($\boldsymbol{E}$ $\parallel$ [010] or $\boldsymbol{E}$ $\perp$ [100]) and $\boldsymbol{B}$ $\parallel$ [010]<br>• ($\boldsymbol{E}$ $\parallel$ [010] or $\boldsymbol{E}$ $\perp$ [100]) and $\boldsymbol{B}$ $\parallel$ [001]
Cm (8.32) $\mathbb{Z}_1$	• $\boldsymbol{B}$ $\parallel$ [100] and strain $\perp$ [100]<br>• ($\boldsymbol{E}$ $\parallel$ [010] or $\boldsymbol{E}$ $\perp$ [100]) and $\boldsymbol{B}$ $\parallel$ [100]
$Cm1'$ (8.33) $\mathbb{Z}_1$	• strain $\perp$ [100]<br>• ($\boldsymbol{E}$ $\parallel$ [010] or $\boldsymbol{E}$ $\perp$ [100])
$P2_1'$ (4.9) $\mathbb{Z}_1$	• $\boldsymbol{B}$ $\parallel$ [100] and strain $\perp$ [001]<br>• $\boldsymbol{B}$ $\parallel$ [010] and strain $\perp$ [001]<br>• $\boldsymbol{B}$ $\perp$ [001]

Continued on next page

. . . continued

Subgroup & SI group	Perturbation
$Cm'c2_1'$ (36.174) $\mathbb{Z}_1$	• $\boldsymbol{B}$ ∥ [010]
$Cmc'2_1'$ (36.175) $\mathbb{Z}_1$	• $\boldsymbol{B}$ ∥ [100]
$P2_1$ (4.7) $\mathbb{Z}_1$	• $\boldsymbol{B}$ ∥ [001] and strain ⊥ [001]
$Cm'c'2_1$ (36.176) $\mathbb{Z}_1$	• $\boldsymbol{B}$ ∥ [001]
$P2_11'$ (4.8) $\mathbb{Z}_1$	• strain ⊥ [001]

Table. S 284: Subgroups of $Cm'c2_1'$ (36.174) that can be realized by applying $\boldsymbol{E}/\boldsymbol{B}$ fields and/or uniaxial strains.

Subgroup & SI group	Perturbation
Cc (9.37) $\mathbb{Z}_1$	• strain ⊥ [010]<br>• ($\boldsymbol{E}$ ∥ [100] or $\boldsymbol{E}$ ⊥ [010])
Cm' (8.34) $\mathbb{Z}_1$	• strain ⊥ [100]<br>• ($\boldsymbol{B}$ ∥ [001] or $\boldsymbol{B}$ ⊥ [100])<br>• ($\boldsymbol{E}$ ∥ [010] or $\boldsymbol{E}$ ⊥ [100])
$P2_1'$ (4.9) $\mathbb{Z}_1$	• strain ⊥ [001]<br>• ($\boldsymbol{B}$ ∥ [100] or $\boldsymbol{B}$ ⊥ [001])

Table. S 285: Subgroups of $Cmc'2_1'$ (36.175) that can be realized by applying $\boldsymbol{E}/\boldsymbol{B}$ fields and/or uniaxial strains.

Subgroup & SI group	Perturbation
Cc' (9.39) $\mathbb{Z}_1$	• strain ⊥ [010]<br>• ($\boldsymbol{B}$ ∥ [001] or $\boldsymbol{B}$ ⊥ [010])<br>• ($\boldsymbol{E}$ ∥ [100] or $\boldsymbol{E}$ ⊥ [010])
Cm (8.32) $\mathbb{Z}_1$	• strain ⊥ [100]<br>• ($\boldsymbol{E}$ ∥ [010] or $\boldsymbol{E}$ ⊥ [100])
$P2_1'$ (4.9) $\mathbb{Z}_1$	• strain ⊥ [001]<br>• ($\boldsymbol{B}$ ∥ [010] or $\boldsymbol{B}$ ⊥ [001])

Table. S 286: Subgroups of $Cm'c'2_1$ (36.176) that can be realized by applying $\boldsymbol{E}/\boldsymbol{B}$ fields and/or uniaxial strains.

Subgroup & SI group	Perturbation
Cc' (9.39) $\mathbb{Z}_1$	• strain $\perp$ [010]<br>• ($\boldsymbol{B} \parallel$ [100] or $\boldsymbol{B} \perp$ [010])<br>• ($\boldsymbol{E} \parallel$ [100] or $\boldsymbol{E} \perp$ [010])
Cm' (8.34) $\mathbb{Z}_1$	• strain $\perp$ [100]<br>• ($\boldsymbol{B} \parallel$ [010] or $\boldsymbol{B} \perp$ [100])<br>• ($\boldsymbol{E} \parallel$ [010] or $\boldsymbol{E} \perp$ [100])
$P2_1$ (4.7) $\mathbb{Z}_1$	• strain $\perp$ [001]

Table. S 287: Subgroups of C_cmc2_1 (36.177) that can be realized by applying $\boldsymbol{E}/\boldsymbol{B}$ fields and/or uniaxial strains.

Subgroup & SI group	Perturbation
P_S1 (1.3) $\mathbb{Z}_1$	• strain in generic direction<br>• ($\boldsymbol{E} \parallel$ [100] or $\boldsymbol{E} \perp$ [010]) and strain $\perp$ [100]<br>• ($\boldsymbol{E} \parallel$ [100] or $\boldsymbol{E} \perp$ [010]) and strain $\perp$ [001]<br>• ($\boldsymbol{E} \parallel$ [010] or $\boldsymbol{E} \perp$ [100]) and strain $\perp$ [010]<br>• ($\boldsymbol{E} \parallel$ [010] or $\boldsymbol{E} \perp$ [100]) and strain $\perp$ [001]<br>• $\boldsymbol{E}$ in generic direction
Cm' (8.34) $\mathbb{Z}_1$	• $\boldsymbol{B} \parallel$ [100] and strain $\perp$ [010]<br>• $\boldsymbol{B} \parallel$ [001] and strain $\perp$ [010]<br>• $\boldsymbol{B} \perp$ [010]<br>• ($\boldsymbol{E} \parallel$ [100] or $\boldsymbol{E} \perp$ [010]) and $\boldsymbol{B} \parallel$ [100]<br>• ($\boldsymbol{E} \parallel$ [100] or $\boldsymbol{E} \perp$ [010]) and $\boldsymbol{B} \parallel$ [001]
Cc (9.37) $\mathbb{Z}_1$	• $\boldsymbol{B} \parallel$ [010] and strain $\perp$ [010]<br>• ($\boldsymbol{E} \parallel$ [100] or $\boldsymbol{E} \perp$ [010]) and $\boldsymbol{B} \parallel$ [010]
C_cc (9.40) $\mathbb{Z}_1$	• strain $\perp$ [010]<br>• ($\boldsymbol{E} \parallel$ [100] or $\boldsymbol{E} \perp$ [010])
Cm (8.32) $\mathbb{Z}_1$	• $\boldsymbol{B} \parallel$ [100] and strain $\perp$ [100]<br>• ($\boldsymbol{E} \parallel$ [010] or $\boldsymbol{E} \perp$ [100]) and $\boldsymbol{B} \parallel$ [100]

Continued on next page

...continued

Subgroup & SI group	Perturbation
Cc' (9.39) $\mathbb{Z}_1$	• $\boldsymbol{B}$ ∥ [010] and strain ⊥ [100]<br>• $\boldsymbol{B}$ ∥ [001] and strain ⊥ [100]<br>• $\boldsymbol{B}$ ⊥ [100]<br>• ($\boldsymbol{E}$ ∥ [010] or $\boldsymbol{E}$ ⊥ [100]) and $\boldsymbol{B}$ ∥ [010]<br>• ($\boldsymbol{E}$ ∥ [010] or $\boldsymbol{E}$ ⊥ [100]) and $\boldsymbol{B}$ ∥ [001]
C_cm (8.35) $\mathbb{Z}_1$	• strain ⊥ [100]<br>• ($\boldsymbol{E}$ ∥ [010] or $\boldsymbol{E}$ ⊥ [100])
$P2'$ (3.3) $\mathbb{Z}_1$	• $\boldsymbol{B}$ ∥ [100] and strain ⊥ [001]<br>• $\boldsymbol{B}$ ∥ [010] and strain ⊥ [001]<br>• $\boldsymbol{B}$ ⊥ [001]
$Cm'm2'$ (35.167) $\mathbb{Z}_1$	• $\boldsymbol{B}$ ∥ [100]
$Cc'c2'$ (37.182) $\mathbb{Z}_1$	• $\boldsymbol{B}$ ∥ [010]
$P2_1$ (4.7) $\mathbb{Z}_1$	• $\boldsymbol{B}$ ∥ [001] and strain ⊥ [001]
$Cm'c'2_1$ (36.176) $\mathbb{Z}_1$	• $\boldsymbol{B}$ ∥ [001]
P_b2_1 (4.11) $\mathbb{Z}_1$	• strain ⊥ [001]

Table. S 288: Subgroups of C_amc2_1 (36.178) that can be realized by applying $\boldsymbol{E}/\boldsymbol{B}$ fields and/or uniaxial strains.

Subgroup & SI group	Perturbation
P_S1 (1.3) $\mathbb{Z}_1$	• strain in generic direction<br>• ($\boldsymbol{E}$ ∥ [100] or $\boldsymbol{E}$ ⊥ [010]) and strain ⊥ [100]<br>• ($\boldsymbol{E}$ ∥ [100] or $\boldsymbol{E}$ ⊥ [010]) and strain ⊥ [001]<br>• ($\boldsymbol{E}$ ∥ [010] or $\boldsymbol{E}$ ⊥ [100]) and strain ⊥ [010]<br>• ($\boldsymbol{E}$ ∥ [010] or $\boldsymbol{E}$ ⊥ [100]) and strain ⊥ [001]<br>• $\boldsymbol{E}$ in generic direction
Cc' (9.39) $\mathbb{Z}_1$	• $\boldsymbol{B}$ ∥ [100] and strain ⊥ [010]<br>• $\boldsymbol{B}$ ∥ [001] and strain ⊥ [010]<br>• $\boldsymbol{B}$ ⊥ [010]<br>• ($\boldsymbol{E}$ ∥ [100] or $\boldsymbol{E}$ ⊥ [010]) and $\boldsymbol{B}$ ∥ [100]<br>• ($\boldsymbol{E}$ ∥ [100] or $\boldsymbol{E}$ ⊥ [010]) and $\boldsymbol{B}$ ∥ [001]
Cc (9.37) $\mathbb{Z}_1$	• $\boldsymbol{B}$ ∥ [010] and strain ⊥ [010]<br>• ($\boldsymbol{E}$ ∥ [100] or $\boldsymbol{E}$ ⊥ [010]) and $\boldsymbol{B}$ ∥ [010]

Continued on next page

...continued

Subgroup & SI group	Perturbation
C_ac (9.41) $\mathbb{Z}_2$	• strain $\perp$ [010]<br>• ($\boldsymbol{E} \parallel$ [100] or $\boldsymbol{E} \perp$ [010])
Cm' (8.34) $\mathbb{Z}_1$	• $\boldsymbol{B} \parallel$ [010] and strain $\perp$ [100]<br>• $\boldsymbol{B} \parallel$ [001] and strain $\perp$ [100]<br>• $\boldsymbol{B} \perp$ [100]<br>• ($\boldsymbol{E} \parallel$ [010] or $\boldsymbol{E} \perp$ [100]) and $\boldsymbol{B} \parallel$ [010]<br>• ($\boldsymbol{E} \parallel$ [010] or $\boldsymbol{E} \perp$ [100]) and $\boldsymbol{B} \parallel$ [001]
Cm (8.32) $\mathbb{Z}_1$	• $\boldsymbol{B} \parallel$ [100] and strain $\perp$ [100]<br>• ($\boldsymbol{E} \parallel$ [010] or $\boldsymbol{E} \perp$ [100]) and $\boldsymbol{B} \parallel$ [100]
C_am (8.36) $\mathbb{Z}_1$	• strain $\perp$ [100]<br>• ($\boldsymbol{E} \parallel$ [010] or $\boldsymbol{E} \perp$ [100])
$P2_1'$ (4.9) $\mathbb{Z}_1$	• $\boldsymbol{B} \parallel$ [100] and strain $\perp$ [001]<br>• $\boldsymbol{B} \parallel$ [010] and strain $\perp$ [001]<br>• $\boldsymbol{B} \perp$ [001]
$Cm'c2_1'$ (36.174) $\mathbb{Z}_1$	• $\boldsymbol{B} \parallel$ [010]
$Cmc'2_1'$ (36.175) $\mathbb{Z}_1$	• $\boldsymbol{B} \parallel$ [100]
$P2_1$ (4.7) $\mathbb{Z}_1$	• $\boldsymbol{B} \parallel$ [001] and strain $\perp$ [001]
$Cm'c'2_1$ (36.176) $\mathbb{Z}_1$	• $\boldsymbol{B} \parallel$ [001]
P_a2_1 (4.10) $\mathbb{Z}_1$	• strain $\perp$ [001]

Table. S 289: Subgroups of C_Amc2_1 (36.179) that can be realized by applying $\boldsymbol{E}/\boldsymbol{B}$ fields and/or uniaxial strains.

Subgroup & SI group	Perturbation
P_S1 (1.3) $\mathbb{Z}_1$	• strain in generic direction<br>• ($\boldsymbol{E} \parallel$ [100] or $\boldsymbol{E} \perp$ [010]) and strain $\perp$ [100]<br>• ($\boldsymbol{E} \parallel$ [100] or $\boldsymbol{E} \perp$ [010]) and strain $\perp$ [001]<br>• ($\boldsymbol{E} \parallel$ [010] or $\boldsymbol{E} \perp$ [100]) and strain $\perp$ [010]<br>• ($\boldsymbol{E} \parallel$ [010] or $\boldsymbol{E} \perp$ [100]) and strain $\perp$ [001]<br>• $\boldsymbol{E}$ in generic direction

Continued on next page

...continued

Subgroup & SI group	Perturbation
Cm' (8.34) $\mathbb{Z}_1$	• $\boldsymbol{B}$ ∥ [100] and strain ⊥ [010]<br>• $\boldsymbol{B}$ ∥ [001] and strain ⊥ [010]<br>• $\boldsymbol{B}$ ⊥ [010]<br>• ($\boldsymbol{E}$ ∥ [100] or $\boldsymbol{E}$ ⊥ [010]) and $\boldsymbol{B}$ ∥ [100]<br>• ($\boldsymbol{E}$ ∥ [100] or $\boldsymbol{E}$ ⊥ [010]) and $\boldsymbol{B}$ ∥ [001]
Cc (9.37) $\mathbb{Z}_1$	• $\boldsymbol{B}$ ∥ [010] and strain ⊥ [010]<br>• ($\boldsymbol{E}$ ∥ [100] or $\boldsymbol{E}$ ⊥ [010]) and $\boldsymbol{B}$ ∥ [010]
C_cc (9.40) $\mathbb{Z}_1$	• strain ⊥ [010]<br>• ($\boldsymbol{E}$ ∥ [100] or $\boldsymbol{E}$ ⊥ [010])
Cc' (9.39) $\mathbb{Z}_1$	• $\boldsymbol{B}$ ∥ [010] and strain ⊥ [100]<br>• $\boldsymbol{B}$ ∥ [001] and strain ⊥ [100]<br>• $\boldsymbol{B}$ ⊥ [100]<br>• ($\boldsymbol{E}$ ∥ [010] or $\boldsymbol{E}$ ⊥ [100]) and $\boldsymbol{B}$ ∥ [010]<br>• ($\boldsymbol{E}$ ∥ [010] or $\boldsymbol{E}$ ⊥ [100]) and $\boldsymbol{B}$ ∥ [001]
Cm (8.32) $\mathbb{Z}_1$	• $\boldsymbol{B}$ ∥ [100] and strain ⊥ [100]<br>• ($\boldsymbol{E}$ ∥ [010] or $\boldsymbol{E}$ ⊥ [100]) and $\boldsymbol{B}$ ∥ [100]
C_cm (8.35) $\mathbb{Z}_1$	• strain ⊥ [100]<br>• ($\boldsymbol{E}$ ∥ [010] or $\boldsymbol{E}$ ⊥ [100])
$P2'$ (3.3) $\mathbb{Z}_1$	• $\boldsymbol{B}$ ∥ [100] and strain ⊥ [001]<br>• $\boldsymbol{B}$ ∥ [010] and strain ⊥ [001]<br>• $\boldsymbol{B}$ ⊥ [001]
$Cc'c2'$ (37.182) $\mathbb{Z}_1$	• $\boldsymbol{B}$ ∥ [010]
$Cm'm2'$ (35.167) $\mathbb{Z}_1$	• $\boldsymbol{B}$ ∥ [100]
$P2_1$ (4.7) $\mathbb{Z}_1$	• $\boldsymbol{B}$ ∥ [001] and strain ⊥ [001]
$Cm'c'2_1$ (36.176) $\mathbb{Z}_1$	• $\boldsymbol{B}$ ∥ [001]
P_C2_1 (4.12) $\mathbb{Z}_1$	• strain ⊥ [001]

Table. S 290: Subgroups of $Ccc2$ (37.180) that can be realized by applying $\boldsymbol{E}/\boldsymbol{B}$ fields and/or uniaxial strains.

Subgroup & SI group	Perturbation

Continued on next page

...continued

Subgroup & SI group	Perturbation
Cc (9.37) $\mathbb{Z}_1$	• strain $\perp$ [010]<br>• $\boldsymbol{B} \parallel$ [010]<br>• ($\boldsymbol{E} \parallel$ [100] or $\boldsymbol{E} \perp$ [010])
Cc (9.37) $\mathbb{Z}_1$	• strain $\perp$ [100]<br>• $\boldsymbol{B} \parallel$ [100]<br>• ($\boldsymbol{E} \parallel$ [010] or $\boldsymbol{E} \perp$ [100])
$P2$ (3.1) $\mathbb{Z}_2$	• strain $\perp$ [001]<br>• $\boldsymbol{B} \parallel$ [001]

Table. S 291: Subgroups of $Ccc21'$ (37.181) that can be realized by applying $\boldsymbol{E}/\boldsymbol{B}$ fields and/or uniaxial strains.

Subgroup & SI group	Perturbation
$P11'$ (1.2) $\mathbb{Z}_1$	• strain in generic direction<br>• ($\boldsymbol{E} \parallel$ [100] or $\boldsymbol{E} \perp$ [010]) and strain $\perp$ [100]<br>• ($\boldsymbol{E} \parallel$ [100] or $\boldsymbol{E} \perp$ [010]) and strain $\perp$ [001]<br>• ($\boldsymbol{E} \parallel$ [010] or $\boldsymbol{E} \perp$ [100]) and strain $\perp$ [010]<br>• ($\boldsymbol{E} \parallel$ [010] or $\boldsymbol{E} \perp$ [100]) and strain $\perp$ [001]<br>• $\boldsymbol{E}$ in generic direction
Cc' (9.39) $\mathbb{Z}_1$	• $\boldsymbol{B} \parallel$ [100] and strain $\perp$ [010]<br>• $\boldsymbol{B} \parallel$ [001] and strain $\perp$ [010]<br>• $\boldsymbol{B} \perp$ [010]<br>• ($\boldsymbol{E} \parallel$ [100] or $\boldsymbol{E} \perp$ [010]) and $\boldsymbol{B} \parallel$ [100]<br>• ($\boldsymbol{E} \parallel$ [100] or $\boldsymbol{E} \perp$ [010]) and $\boldsymbol{B} \parallel$ [001]
Cc (9.37) $\mathbb{Z}_1$	• $\boldsymbol{B} \parallel$ [010] and strain $\perp$ [010]<br>• ($\boldsymbol{E} \parallel$ [100] or $\boldsymbol{E} \perp$ [010]) and $\boldsymbol{B} \parallel$ [010]
$Cc1'$ (9.38) $\mathbb{Z}_1$	• strain $\perp$ [010]<br>• ($\boldsymbol{E} \parallel$ [100] or $\boldsymbol{E} \perp$ [010])
Cc' (9.39) $\mathbb{Z}_1$	• $\boldsymbol{B} \parallel$ [010] and strain $\perp$ [100]<br>• $\boldsymbol{B} \parallel$ [001] and strain $\perp$ [100]<br>• $\boldsymbol{B} \perp$ [100]<br>• ($\boldsymbol{E} \parallel$ [010] or $\boldsymbol{E} \perp$ [100]) and $\boldsymbol{B} \parallel$ [010]<br>• ($\boldsymbol{E} \parallel$ [010] or $\boldsymbol{E} \perp$ [100]) and $\boldsymbol{B} \parallel$ [001]
Cc (9.37) $\mathbb{Z}_1$	• $\boldsymbol{B} \parallel$ [100] and strain $\perp$ [100]<br>• ($\boldsymbol{E} \parallel$ [010] or $\boldsymbol{E} \perp$ [100]) and $\boldsymbol{B} \parallel$ [100]

Continued on next page

...continued

Subgroup & SI group	Perturbation
$Cc1'$ (9.38) $\mathbb{Z}_1$	• strain $\perp$ $[100]$<br>• ($\boldsymbol{E}$ $\parallel$ $[010]$ or $\boldsymbol{E}$ $\perp$ $[100]$)
$P2'$ (3.3) $\mathbb{Z}_1$	• $\boldsymbol{B}$ $\parallel$ $[100]$ and strain $\perp$ $[001]$<br>• $\boldsymbol{B}$ $\parallel$ $[010]$ and strain $\perp$ $[001]$<br>• $\boldsymbol{B}$ $\perp$ $[001]$
$Cc'c2'$ (37.182) $\mathbb{Z}_1$	• $\boldsymbol{B}$ $\parallel$ $[010]$
$Cc'c2'$ (37.182) $\mathbb{Z}_1$	• $\boldsymbol{B}$ $\parallel$ $[100]$
$P2$ (3.1) $\mathbb{Z}_2$	• $\boldsymbol{B}$ $\parallel$ $[001]$ and strain $\perp$ $[001]$
$Cc'c'2$ (37.183) $\mathbb{Z}_2$	• $\boldsymbol{B}$ $\parallel$ $[001]$
$P21'$ (3.2) $\mathbb{Z}_2$	• strain $\perp$ $[001]$

Table. S 292: Subgroups of $Cc'c2'$ (37.182) that can be realized by applying $\boldsymbol{E}/\boldsymbol{B}$ fields and/or uniaxial strains.

Subgroup & SI group	Perturbation
Cc (9.37) $\mathbb{Z}_1$	• strain $\perp$ $[010]$<br>• ($\boldsymbol{E}$ $\parallel$ $[100]$ or $\boldsymbol{E}$ $\perp$ $[010]$)
Cc' (9.39) $\mathbb{Z}_1$	• strain $\perp$ $[100]$<br>• ($\boldsymbol{B}$ $\parallel$ $[001]$ or $\boldsymbol{B}$ $\perp$ $[100]$)<br>• ($\boldsymbol{E}$ $\parallel$ $[010]$ or $\boldsymbol{E}$ $\perp$ $[100]$)
$P2'$ (3.3) $\mathbb{Z}_1$	• strain $\perp$ $[001]$<br>• ($\boldsymbol{B}$ $\parallel$ $[100]$ or $\boldsymbol{B}$ $\perp$ $[001]$)

Table. S 293: Subgroups of $Cc'c'2$ (37.183) that can be realized by applying $\boldsymbol{E}/\boldsymbol{B}$ fields and/or uniaxial strains.

Subgroup & SI group	Perturbation
Cc' (9.39) $\mathbb{Z}_1$	• strain $\perp$ $[010]$<br>• ($\boldsymbol{B}$ $\parallel$ $[100]$ or $\boldsymbol{B}$ $\perp$ $[010]$)<br>• ($\boldsymbol{E}$ $\parallel$ $[100]$ or $\boldsymbol{E}$ $\perp$ $[010]$)

Continued on next page

...continued

Subgroup & SI group	Perturbation
Cc' (9.39) $\mathbb{Z}_1$	• strain $\perp$ [100]<br>• ($\boldsymbol{B}$ $\parallel$ [010] or $\boldsymbol{B}$ $\perp$ [100])<br>• ($\boldsymbol{E}$ $\parallel$ [010] or $\boldsymbol{E}$ $\perp$ [100])
$P2$ (3.1) $\mathbb{Z}_2$	• strain $\perp$ [001]

Table. S 294: Subgroups of $C_c cc2$ (37.184) that can be realized by applying $\boldsymbol{E}/\boldsymbol{B}$ fields and/or uniaxial strains.

Subgroup & SI group	Perturbation
P_S1 (1.3) $\mathbb{Z}_1$	• strain in generic direction<br>• ($\boldsymbol{E}$ $\parallel$ [100] or $\boldsymbol{E}$ $\perp$ [010]) and strain $\perp$ [100]<br>• ($\boldsymbol{E}$ $\parallel$ [100] or $\boldsymbol{E}$ $\perp$ [010]) and strain $\perp$ [001]<br>• ($\boldsymbol{E}$ $\parallel$ [010] or $\boldsymbol{E}$ $\perp$ [100]) and strain $\perp$ [010]<br>• ($\boldsymbol{E}$ $\parallel$ [010] or $\boldsymbol{E}$ $\perp$ [100]) and strain $\perp$ [001]<br>• $\boldsymbol{E}$ in generic direction
Cm' (8.34) $\mathbb{Z}_1$	• $\boldsymbol{B}$ $\parallel$ [100] and strain $\perp$ [010]<br>• $\boldsymbol{B}$ $\parallel$ [001] and strain $\perp$ [010]<br>• $\boldsymbol{B}$ $\perp$ [010]<br>• ($\boldsymbol{E}$ $\parallel$ [100] or $\boldsymbol{E}$ $\perp$ [010]) and $\boldsymbol{B}$ $\parallel$ [100]<br>• ($\boldsymbol{E}$ $\parallel$ [100] or $\boldsymbol{E}$ $\perp$ [010]) and $\boldsymbol{B}$ $\parallel$ [001]
Cc (9.37) $\mathbb{Z}_1$	• $\boldsymbol{B}$ $\parallel$ [010] and strain $\perp$ [010]<br>• ($\boldsymbol{E}$ $\parallel$ [100] or $\boldsymbol{E}$ $\perp$ [010]) and $\boldsymbol{B}$ $\parallel$ [010]
C_cc (9.40) $\mathbb{Z}_1$	• strain $\perp$ [010]<br>• ($\boldsymbol{E}$ $\parallel$ [100] or $\boldsymbol{E}$ $\perp$ [010])
Cm' (8.34) $\mathbb{Z}_1$	• $\boldsymbol{B}$ $\parallel$ [010] and strain $\perp$ [100]<br>• $\boldsymbol{B}$ $\parallel$ [001] and strain $\perp$ [100]<br>• $\boldsymbol{B}$ $\perp$ [100]<br>• ($\boldsymbol{E}$ $\parallel$ [010] or $\boldsymbol{E}$ $\perp$ [100]) and $\boldsymbol{B}$ $\parallel$ [010]<br>• ($\boldsymbol{E}$ $\parallel$ [010] or $\boldsymbol{E}$ $\perp$ [100]) and $\boldsymbol{B}$ $\parallel$ [001]
Cc (9.37) $\mathbb{Z}_1$	• $\boldsymbol{B}$ $\parallel$ [100] and strain $\perp$ [100]<br>• ($\boldsymbol{E}$ $\parallel$ [010] or $\boldsymbol{E}$ $\perp$ [100]) and $\boldsymbol{B}$ $\parallel$ [100]
C_cc (9.40) $\mathbb{Z}_1$	• strain $\perp$ [100]<br>• ($\boldsymbol{E}$ $\parallel$ [010] or $\boldsymbol{E}$ $\perp$ [100])
$P2$ (3.1) $\mathbb{Z}_2$	• $\boldsymbol{B}$ $\parallel$ [001] and strain $\perp$ [001]

Continued on next page

...continued

Subgroup & SI group	Perturbation
$Cm'm'2$ (35.168) $\mathbb{Z}_2$	• $\boldsymbol{B} \parallel [001]$
$P2_1'$ (4.9) $\mathbb{Z}_1$	• $\boldsymbol{B} \parallel [100]$ and strain $\perp [001]$<br>• $\boldsymbol{B} \parallel [010]$ and strain $\perp [001]$<br>• $\boldsymbol{B} \perp [001]$
$Cm'c2_1'$ (36.174) $\mathbb{Z}_1$	• $\boldsymbol{B} \parallel [010]$
$Cm'c2_1'$ (36.174) $\mathbb{Z}_1$	• $\boldsymbol{B} \parallel [100]$
P_b2 (3.5) $\mathbb{Z}_2$	• strain $\perp [001]$

Table. S 295: Subgroups of C_acc2 (37.185) that can be realized by applying $\boldsymbol{E}/\boldsymbol{B}$ fields and/or uniaxial strains.

Subgroup & SI group	Perturbation
P_S1 (1.3) $\mathbb{Z}_1$	• strain in generic direction<br>• ($\boldsymbol{E} \parallel [100]$ or $\boldsymbol{E} \perp [010]$) and strain $\perp [100]$<br>• ($\boldsymbol{E} \parallel [100]$ or $\boldsymbol{E} \perp [010]$) and strain $\perp [001]$<br>• ($\boldsymbol{E} \parallel [010]$ or $\boldsymbol{E} \perp [100]$) and strain $\perp [010]$<br>• ($\boldsymbol{E} \parallel [010]$ or $\boldsymbol{E} \perp [100]$) and strain $\perp [001]$<br>• $\boldsymbol{E}$ in generic direction
Cc' (9.39) $\mathbb{Z}_1$	• $\boldsymbol{B} \parallel [100]$ and strain $\perp [010]$<br>• $\boldsymbol{B} \parallel [001]$ and strain $\perp [010]$<br>• $\boldsymbol{B} \perp [010]$<br>• ($\boldsymbol{E} \parallel [100]$ or $\boldsymbol{E} \perp [010]$) and $\boldsymbol{B} \parallel [100]$<br>• ($\boldsymbol{E} \parallel [100]$ or $\boldsymbol{E} \perp [010]$) and $\boldsymbol{B} \parallel [001]$
Cc (9.37) $\mathbb{Z}_1$	• $\boldsymbol{B} \parallel [010]$ and strain $\perp [010]$<br>• ($\boldsymbol{E} \parallel [100]$ or $\boldsymbol{E} \perp [010]$) and $\boldsymbol{B} \parallel [010]$
C_ac (9.41) $\mathbb{Z}_2$	• strain $\perp [010]$<br>• ($\boldsymbol{E} \parallel [100]$ or $\boldsymbol{E} \perp [010]$)
Cc' (9.39) $\mathbb{Z}_1$	• $\boldsymbol{B} \parallel [010]$ and strain $\perp [100]$<br>• $\boldsymbol{B} \parallel [001]$ and strain $\perp [100]$<br>• $\boldsymbol{B} \perp [100]$<br>• ($\boldsymbol{E} \parallel [010]$ or $\boldsymbol{E} \perp [100]$) and $\boldsymbol{B} \parallel [010]$<br>• ($\boldsymbol{E} \parallel [010]$ or $\boldsymbol{E} \perp [100]$) and $\boldsymbol{B} \parallel [001]$
Cc (9.37) $\mathbb{Z}_1$	• $\boldsymbol{B} \parallel [100]$ and strain $\perp [100]$<br>• ($\boldsymbol{E} \parallel [010]$ or $\boldsymbol{E} \perp [100]$) and $\boldsymbol{B} \parallel [100]$

Continued on next page

. . . continued

Subgroup & SI group	Perturbation
C_ac (9.41) $\mathbb{Z}_2$	• strain ⊥ [100]<br>• ($\boldsymbol{E}$ ∥ [010] or $\boldsymbol{E}$ ⊥ [100])
$P2'$ (3.3) $\mathbb{Z}_1$	• $\boldsymbol{B}$ ∥ [100] and strain ⊥ [001]<br>• $\boldsymbol{B}$ ∥ [010] and strain ⊥ [001]<br>• $\boldsymbol{B}$ ⊥ [001]
$Cc'c2'$ (37.182) $\mathbb{Z}_1$	• $\boldsymbol{B}$ ∥ [010]
$Cc'c2'$ (37.182) $\mathbb{Z}_1$	• $\boldsymbol{B}$ ∥ [100]
$P2$ (3.1) $\mathbb{Z}_2$	• $\boldsymbol{B}$ ∥ [001] and strain ⊥ [001]
$Cc'c'2$ (37.183) $\mathbb{Z}_2$	• $\boldsymbol{B}$ ∥ [001]
P_a2 (3.4) $\mathbb{Z}_2$	• strain ⊥ [001]

Table. S 296: Subgroups of C_Acc2 (37.186) that can be realized by applying $\boldsymbol{E}/\boldsymbol{B}$ fields and/or uniaxial strains.

Subgroup & SI group	Perturbation
P_S1 (1.3) $\mathbb{Z}_1$	• strain in generic direction<br>• ($\boldsymbol{E}$ ∥ [100] or $\boldsymbol{E}$ ⊥ [010]) and strain ⊥ [100]<br>• ($\boldsymbol{E}$ ∥ [100] or $\boldsymbol{E}$ ⊥ [010]) and strain ⊥ [001]<br>• ($\boldsymbol{E}$ ∥ [010] or $\boldsymbol{E}$ ⊥ [100]) and strain ⊥ [010]<br>• ($\boldsymbol{E}$ ∥ [010] or $\boldsymbol{E}$ ⊥ [100]) and strain ⊥ [001]<br>• $\boldsymbol{E}$ in generic direction
Cm' (8.34) $\mathbb{Z}_1$	• $\boldsymbol{B}$ ∥ [100] and strain ⊥ [010]<br>• $\boldsymbol{B}$ ∥ [001] and strain ⊥ [010]<br>• $\boldsymbol{B}$ ⊥ [010]<br>• ($\boldsymbol{E}$ ∥ [100] or $\boldsymbol{E}$ ⊥ [010]) and $\boldsymbol{B}$ ∥ [100]<br>• ($\boldsymbol{E}$ ∥ [100] or $\boldsymbol{E}$ ⊥ [010]) and $\boldsymbol{B}$ ∥ [001]
Cc (9.37) $\mathbb{Z}_1$	• $\boldsymbol{B}$ ∥ [010] and strain ⊥ [010]<br>• ($\boldsymbol{E}$ ∥ [100] or $\boldsymbol{E}$ ⊥ [010]) and $\boldsymbol{B}$ ∥ [010]
C_cc (9.40) $\mathbb{Z}_1$	• strain ⊥ [010]<br>• ($\boldsymbol{E}$ ∥ [100] or $\boldsymbol{E}$ ⊥ [010])

Continued on next page

. . . continued

Subgroup & SI group	Perturbation
Cm' (8.34) $\mathbb{Z}_1$	• $\boldsymbol{B}$ ∥ [010] and strain ⊥ [100]<br>• $\boldsymbol{B}$ ∥ [001] and strain ⊥ [100]<br>• $\boldsymbol{B}$ ⊥ [100]<br>• ($\boldsymbol{E}$ ∥ [010] or $\boldsymbol{E}$ ⊥ [100]) and $\boldsymbol{B}$ ∥ [010]<br>• ($\boldsymbol{E}$ ∥ [010] or $\boldsymbol{E}$ ⊥ [100]) and $\boldsymbol{B}$ ∥ [001]
Cc (9.37) $\mathbb{Z}_1$	• $\boldsymbol{B}$ ∥ [100] and strain ⊥ [100]<br>• ($\boldsymbol{E}$ ∥ [010] or $\boldsymbol{E}$ ⊥ [100]) and $\boldsymbol{B}$ ∥ [100]
C_cc (9.40) $\mathbb{Z}_1$	• strain ⊥ [100]<br>• ($\boldsymbol{E}$ ∥ [010] or $\boldsymbol{E}$ ⊥ [100])
$P2_1'$ (4.9) $\mathbb{Z}_1$	• $\boldsymbol{B}$ ∥ [100] and strain ⊥ [001]<br>• $\boldsymbol{B}$ ∥ [010] and strain ⊥ [001]<br>• $\boldsymbol{B}$ ⊥ [001]
$Cm'c2_1'$ (36.174) $\mathbb{Z}_1$	• $\boldsymbol{B}$ ∥ [010]
$Cm'c2_1'$ (36.174) $\mathbb{Z}_1$	• $\boldsymbol{B}$ ∥ [100]
$P2$ (3.1) $\mathbb{Z}_2$	• $\boldsymbol{B}$ ∥ [001] and strain ⊥ [001]
$Cm'm'2$ (35.168) $\mathbb{Z}_2$	• $\boldsymbol{B}$ ∥ [001]
P_C2 (3.6) $\mathbb{Z}_1$	• strain ⊥ [001]

Table. S 297: Subgroups of $Amm2$ (38.187) that can be realized by applying $\boldsymbol{E}/\boldsymbol{B}$ fields and/or uniaxial strains.

Subgroup & SI group	Perturbation
Cm (8.32) $\mathbb{Z}_1$	• strain ⊥ [010]<br>• $\boldsymbol{B}$ ∥ [010]<br>• ($\boldsymbol{E}$ ∥ [100] or $\boldsymbol{E}$ ⊥ [010])
Pm (6.18) $\mathbb{Z}_1$	• strain ⊥ [100]<br>• $\boldsymbol{B}$ ∥ [100]<br>• ($\boldsymbol{E}$ ∥ [010] or $\boldsymbol{E}$ ⊥ [100])
$C2$ (5.13) $\mathbb{Z}_1$	• strain ⊥ [001]<br>• $\boldsymbol{B}$ ∥ [001]

Table. S 298: Subgroups of $Amm21'$ (38.188) that can be realized by applying $\boldsymbol{E}/\boldsymbol{B}$ fields and/or uniaxial strains.

Subgroup & SI group	Perturbation
$P11'$ (1.2) $\mathbb{Z}_1$	• strain in generic direction<br>• ($\boldsymbol{E} \parallel [100]$ or $\boldsymbol{E} \perp [010]$) and strain $\perp [100]$<br>• ($\boldsymbol{E} \parallel [100]$ or $\boldsymbol{E} \perp [010]$) and strain $\perp [001]$<br>• ($\boldsymbol{E} \parallel [010]$ or $\boldsymbol{E} \perp [100]$) and strain $\perp [010]$<br>• ($\boldsymbol{E} \parallel [010]$ or $\boldsymbol{E} \perp [100]$) and strain $\perp [001]$<br>• $\boldsymbol{E}$ in generic direction
Cm' (8.34) $\mathbb{Z}_1$	• $\boldsymbol{B} \parallel [100]$ and strain $\perp [010]$<br>• $\boldsymbol{B} \parallel [001]$ and strain $\perp [010]$<br>• $\boldsymbol{B} \perp [010]$<br>• ($\boldsymbol{E} \parallel [100]$ or $\boldsymbol{E} \perp [010]$) and $\boldsymbol{B} \parallel [100]$<br>• ($\boldsymbol{E} \parallel [100]$ or $\boldsymbol{E} \perp [010]$) and $\boldsymbol{B} \parallel [001]$
Cm (8.32) $\mathbb{Z}_1$	• $\boldsymbol{B} \parallel [010]$ and strain $\perp [010]$<br>• ($\boldsymbol{E} \parallel [100]$ or $\boldsymbol{E} \perp [010]$) and $\boldsymbol{B} \parallel [010]$
$Cm1'$ (8.33) $\mathbb{Z}_1$	• strain $\perp [010]$<br>• ($\boldsymbol{E} \parallel [100]$ or $\boldsymbol{E} \perp [010]$)
Pm' (6.20) $\mathbb{Z}_1$	• $\boldsymbol{B} \parallel [010]$ and strain $\perp [100]$<br>• $\boldsymbol{B} \parallel [001]$ and strain $\perp [100]$<br>• $\boldsymbol{B} \perp [100]$<br>• ($\boldsymbol{E} \parallel [010]$ or $\boldsymbol{E} \perp [100]$) and $\boldsymbol{B} \parallel [010]$<br>• ($\boldsymbol{E} \parallel [010]$ or $\boldsymbol{E} \perp [100]$) and $\boldsymbol{B} \parallel [001]$
Pm (6.18) $\mathbb{Z}_1$	• $\boldsymbol{B} \parallel [100]$ and strain $\perp [100]$<br>• ($\boldsymbol{E} \parallel [010]$ or $\boldsymbol{E} \perp [100]$) and $\boldsymbol{B} \parallel [100]$
$Pm1'$ (6.19) $\mathbb{Z}_1$	• strain $\perp [100]$<br>• ($\boldsymbol{E} \parallel [010]$ or $\boldsymbol{E} \perp [100]$)
$C2'$ (5.15) $\mathbb{Z}_1$	• $\boldsymbol{B} \parallel [100]$ and strain $\perp [001]$<br>• $\boldsymbol{B} \parallel [010]$ and strain $\perp [001]$<br>• $\boldsymbol{B} \perp [001]$
$Am'm2'$ (38.189) $\mathbb{Z}_1$	• $\boldsymbol{B} \parallel [010]$
$Amm'2'$ (38.190) $\mathbb{Z}_1$	• $\boldsymbol{B} \parallel [100]$
$C2$ (5.13) $\mathbb{Z}_1$	• $\boldsymbol{B} \parallel [001]$ and strain $\perp [001]$
$Am'm'2$ (38.191) $\mathbb{Z}_1$	• $\boldsymbol{B} \parallel [001]$
$C21'$ (5.14) $\mathbb{Z}_1$	• strain $\perp [001]$

Table. S 299: Subgroups of $Am'm2'$ (38.189) that can be realized by applying $\boldsymbol{E}/\boldsymbol{B}$ fields and/or uniaxial strains.

Subgroup & SI group	Perturbation
Cm (8.32) $\mathbb{Z}_1$	• strain $\perp$ [010]<br>• ($\boldsymbol{E} \parallel$ [100] or $\boldsymbol{E} \perp$ [010])
Pm' (6.20) $\mathbb{Z}_1$	• strain $\perp$ [100]<br>• ($\boldsymbol{B} \parallel$ [001] or $\boldsymbol{B} \perp$ [100])<br>• ($\boldsymbol{E} \parallel$ [010] or $\boldsymbol{E} \perp$ [100])
$C2'$ (5.15) $\mathbb{Z}_1$	• strain $\perp$ [001]<br>• ($\boldsymbol{B} \parallel$ [100] or $\boldsymbol{B} \perp$ [001])

Table. S 300: Subgroups of $Amm'2'$ (38.190) that can be realized by applying $\boldsymbol{E}/\boldsymbol{B}$ fields and/or uniaxial strains.

Subgroup & SI group	Perturbation
Cm' (8.34) $\mathbb{Z}_1$	• strain $\perp$ [010]<br>• ($\boldsymbol{B} \parallel$ [001] or $\boldsymbol{B} \perp$ [010])<br>• ($\boldsymbol{E} \parallel$ [100] or $\boldsymbol{E} \perp$ [010])
Pm (6.18) $\mathbb{Z}_1$	• strain $\perp$ [100]<br>• ($\boldsymbol{E} \parallel$ [010] or $\boldsymbol{E} \perp$ [100])
$C2'$ (5.15) $\mathbb{Z}_1$	• strain $\perp$ [001]<br>• ($\boldsymbol{B} \parallel$ [010] or $\boldsymbol{B} \perp$ [001])

Table. S 301: Subgroups of $Am'm'2$ (38.191) that can be realized by applying $\boldsymbol{E}/\boldsymbol{B}$ fields and/or uniaxial strains.

Subgroup & SI group	Perturbation
Cm' (8.34) $\mathbb{Z}_1$	• strain $\perp$ [010]<br>• ($\boldsymbol{B} \parallel$ [100] or $\boldsymbol{B} \perp$ [010])<br>• ($\boldsymbol{E} \parallel$ [100] or $\boldsymbol{E} \perp$ [010])
Pm' (6.20) $\mathbb{Z}_1$	• strain $\perp$ [100]<br>• ($\boldsymbol{B} \parallel$ [010] or $\boldsymbol{B} \perp$ [100])<br>• ($\boldsymbol{E} \parallel$ [010] or $\boldsymbol{E} \perp$ [100])

Continued on next page

...continued

Subgroup & SI group	Perturbation
$C2$ (5.13) $\mathbb{Z}_1$	• strain $\perp$ [001]

Table. S 302: Subgroups of A_amm2 (38.192) that can be realized by applying $\boldsymbol{E}/\boldsymbol{B}$ fields and/or uniaxial strains.

Subgroup & SI group	Perturbation
Cm (8.32) $\mathbb{Z}_1$	• $\boldsymbol{B} \parallel$ [010] and strain $\perp$ [010]<br>• ($\boldsymbol{E} \parallel$ [100] or $\boldsymbol{E} \perp$ [010]) and $\boldsymbol{B} \parallel$ [010]
P_S1 (1.3) $\mathbb{Z}_1$	• strain in generic direction<br>• ($\boldsymbol{E} \parallel$ [100] or $\boldsymbol{E} \perp$ [010]) and strain $\perp$ [100]<br>• ($\boldsymbol{E} \parallel$ [100] or $\boldsymbol{E} \perp$ [010]) and strain $\perp$ [001]<br>• ($\boldsymbol{E} \parallel$ [010] or $\boldsymbol{E} \perp$ [100]) and strain $\perp$ [010]<br>• ($\boldsymbol{E} \parallel$ [010] or $\boldsymbol{E} \perp$ [100]) and strain $\perp$ [001]<br>• $\boldsymbol{E}$ in generic direction
Cc' (9.39) $\mathbb{Z}_1$	• $\boldsymbol{B} \parallel$ [100] and strain $\perp$ [010]<br>• $\boldsymbol{B} \parallel$ [001] and strain $\perp$ [010]<br>• $\boldsymbol{B} \perp$ [010]<br>• ($\boldsymbol{E} \parallel$ [100] or $\boldsymbol{E} \perp$ [010]) and $\boldsymbol{B} \parallel$ [100]<br>• ($\boldsymbol{E} \parallel$ [100] or $\boldsymbol{E} \perp$ [010]) and $\boldsymbol{B} \parallel$ [001]
C_cm (8.35) $\mathbb{Z}_1$	• strain $\perp$ [010]<br>• ($\boldsymbol{E} \parallel$ [100] or $\boldsymbol{E} \perp$ [010])
Pm (6.18) $\mathbb{Z}_1$	• $\boldsymbol{B} \parallel$ [100] and strain $\perp$ [100]<br>• ($\boldsymbol{E} \parallel$ [010] or $\boldsymbol{E} \perp$ [100]) and $\boldsymbol{B} \parallel$ [100]
$C2$ (5.13) $\mathbb{Z}_1$	• $\boldsymbol{B} \parallel$ [001] and strain $\perp$ [001]
Pm' (6.20) $\mathbb{Z}_1$	• $\boldsymbol{B} \parallel$ [010] and strain $\perp$ [100]<br>• $\boldsymbol{B} \parallel$ [001] and strain $\perp$ [100]<br>• $\boldsymbol{B} \perp$ [100]<br>• ($\boldsymbol{E} \parallel$ [010] or $\boldsymbol{E} \perp$ [100]) and $\boldsymbol{B} \parallel$ [010]<br>• ($\boldsymbol{E} \parallel$ [010] or $\boldsymbol{E} \perp$ [100]) and $\boldsymbol{B} \parallel$ [001]
P_bm (6.22) $\mathbb{Z}_1$	• strain $\perp$ [100]<br>• ($\boldsymbol{E} \parallel$ [010] or $\boldsymbol{E} \perp$ [100])
$Am'a'2$ (40.207) $\mathbb{Z}_1$	• $\boldsymbol{B} \parallel$ [001]

Continued on next page

. . . continued

Subgroup & SI group	Perturbation
$C2'$ (5.15) $\mathbb{Z}_1$	• $\boldsymbol{B}$ ∥ [100] and strain ⊥ [001]<br>• $\boldsymbol{B}$ ∥ [010] and strain ⊥ [001]<br>• $\boldsymbol{B}$ ⊥ [001]
$Ama'2'$ (40.206) $\mathbb{Z}_1$	• $\boldsymbol{B}$ ∥ [100]
C_c2 (5.16) $\mathbb{Z}_1$	• strain ⊥ [001]
$Am'm2'$ (38.189) $\mathbb{Z}_1$	• $\boldsymbol{B}$ ∥ [010]

Table. S 303: Subgroups of A_bmm2 (38.193) that can be realized by applying $\boldsymbol{E}/\boldsymbol{B}$ fields and/or uniaxial strains.

Subgroup & SI group	Perturbation
P_S1 (1.3) $\mathbb{Z}_1$	• strain in generic direction<br>• ($\boldsymbol{E}$ ∥ [100] or $\boldsymbol{E}$ ⊥ [010]) and strain ⊥ [100]<br>• ($\boldsymbol{E}$ ∥ [100] or $\boldsymbol{E}$ ⊥ [010]) and strain ⊥ [001]<br>• ($\boldsymbol{E}$ ∥ [010] or $\boldsymbol{E}$ ⊥ [100]) and strain ⊥ [010]<br>• ($\boldsymbol{E}$ ∥ [010] or $\boldsymbol{E}$ ⊥ [100]) and strain ⊥ [001]<br>• $\boldsymbol{E}$ in generic direction
Cm' (8.34) $\mathbb{Z}_1$	• $\boldsymbol{B}$ ∥ [100] and strain ⊥ [010]<br>• $\boldsymbol{B}$ ∥ [001] and strain ⊥ [010]<br>• $\boldsymbol{B}$ ⊥ [010]<br>• ($\boldsymbol{E}$ ∥ [100] or $\boldsymbol{E}$ ⊥ [010]) and $\boldsymbol{B}$ ∥ [100]<br>• ($\boldsymbol{E}$ ∥ [100] or $\boldsymbol{E}$ ⊥ [010]) and $\boldsymbol{B}$ ∥ [001]
Cm (8.32) $\mathbb{Z}_1$	• $\boldsymbol{B}$ ∥ [010] and strain ⊥ [010]<br>• ($\boldsymbol{E}$ ∥ [100] or $\boldsymbol{E}$ ⊥ [010]) and $\boldsymbol{B}$ ∥ [010]
C_am (8.36) $\mathbb{Z}_1$	• strain ⊥ [010]<br>• ($\boldsymbol{E}$ ∥ [100] or $\boldsymbol{E}$ ⊥ [010])
Pc' (7.26) $\mathbb{Z}_1$	• $\boldsymbol{B}$ ∥ [010] and strain ⊥ [100]<br>• $\boldsymbol{B}$ ∥ [001] and strain ⊥ [100]<br>• $\boldsymbol{B}$ ⊥ [100]<br>• ($\boldsymbol{E}$ ∥ [010] or $\boldsymbol{E}$ ⊥ [100]) and $\boldsymbol{B}$ ∥ [010]<br>• ($\boldsymbol{E}$ ∥ [010] or $\boldsymbol{E}$ ⊥ [100]) and $\boldsymbol{B}$ ∥ [001]
Pm (6.18) $\mathbb{Z}_1$	• $\boldsymbol{B}$ ∥ [100] and strain ⊥ [100]<br>• ($\boldsymbol{E}$ ∥ [010] or $\boldsymbol{E}$ ⊥ [100]) and $\boldsymbol{B}$ ∥ [100]

Continued on next page

...continued

Subgroup & SI group	Perturbation
P_am (6.21) $\mathbb{Z}_1$	• strain $\perp$ [100]<br>• ($\boldsymbol{E}$ $\parallel$ [010] or $\boldsymbol{E}$ $\perp$ [100])
$C2'$ (5.15) $\mathbb{Z}_1$	• $\boldsymbol{B}$ $\parallel$ [100] and strain $\perp$ [001]<br>• $\boldsymbol{B}$ $\parallel$ [010] and strain $\perp$ [001]<br>• $\boldsymbol{B}$ $\perp$ [001]
$Ab'm2'$ (39.197) $\mathbb{Z}_1$	• $\boldsymbol{B}$ $\parallel$ [010]
$Amm'2'$ (38.190) $\mathbb{Z}_1$	• $\boldsymbol{B}$ $\parallel$ [100]
$C2$ (5.13) $\mathbb{Z}_1$	• $\boldsymbol{B}$ $\parallel$ [001] and strain $\perp$ [001]
$Ab'm'2$ (39.199) $\mathbb{Z}_2$	• $\boldsymbol{B}$ $\parallel$ [001]
C_a2 (5.17) $\mathbb{Z}_2$	• strain $\perp$ [001]

Table. S 304: Subgroups of A_Bmm2 (38.194) that can be realized by applying $\boldsymbol{E}/\boldsymbol{B}$ fields and/or uniaxial strains.

Subgroup & SI group	Perturbation
Cm (8.32) $\mathbb{Z}_1$	• $\boldsymbol{B}$ $\parallel$ [010] and strain $\perp$ [010]<br>• ($\boldsymbol{E}$ $\parallel$ [100] or $\boldsymbol{E}$ $\perp$ [010]) and $\boldsymbol{B}$ $\parallel$ [010]
P_S1 (1.3) $\mathbb{Z}_1$	• strain in generic direction<br>• ($\boldsymbol{E}$ $\parallel$ [100] or $\boldsymbol{E}$ $\perp$ [010]) and strain $\perp$ [100]<br>• ($\boldsymbol{E}$ $\parallel$ [100] or $\boldsymbol{E}$ $\perp$ [010]) and strain $\perp$ [001]<br>• ($\boldsymbol{E}$ $\parallel$ [010] or $\boldsymbol{E}$ $\perp$ [100]) and strain $\perp$ [010]<br>• ($\boldsymbol{E}$ $\parallel$ [010] or $\boldsymbol{E}$ $\perp$ [100]) and strain $\perp$ [001]<br>• $\boldsymbol{E}$ in generic direction
Cc' (9.39) $\mathbb{Z}_1$	• $\boldsymbol{B}$ $\parallel$ [100] and strain $\perp$ [010]<br>• $\boldsymbol{B}$ $\parallel$ [001] and strain $\perp$ [010]<br>• $\boldsymbol{B}$ $\perp$ [010]<br>• ($\boldsymbol{E}$ $\parallel$ [100] or $\boldsymbol{E}$ $\perp$ [010]) and $\boldsymbol{B}$ $\parallel$ [100]<br>• ($\boldsymbol{E}$ $\parallel$ [100] or $\boldsymbol{E}$ $\perp$ [010]) and $\boldsymbol{B}$ $\parallel$ [001]
C_cm (8.35) $\mathbb{Z}_1$	• strain $\perp$ [010]<br>• ($\boldsymbol{E}$ $\parallel$ [100] or $\boldsymbol{E}$ $\perp$ [010])
Pm (6.18) $\mathbb{Z}_1$	• $\boldsymbol{B}$ $\parallel$ [100] and strain $\perp$ [100]<br>• ($\boldsymbol{E}$ $\parallel$ [010] or $\boldsymbol{E}$ $\perp$ [100]) and $\boldsymbol{B}$ $\parallel$ [100]
$C2$ (5.13) $\mathbb{Z}_1$	• $\boldsymbol{B}$ $\parallel$ [001] and strain $\perp$ [001]

Continued on next page

…continued

Subgroup & SI group	Perturbation
Pc' (7.26) $\mathbb{Z}_1$	• $\boldsymbol{B} \parallel [010]$ and strain $\perp [100]$<br>• $\boldsymbol{B} \parallel [001]$ and strain $\perp [100]$<br>• $\boldsymbol{B} \perp [100]$<br>• ($\boldsymbol{E} \parallel [010]$ or $\boldsymbol{E} \perp [100]$) and $\boldsymbol{B} \parallel [010]$<br>• ($\boldsymbol{E} \parallel [010]$ or $\boldsymbol{E} \perp [100]$) and $\boldsymbol{B} \parallel [001]$
P_Cm (6.23) $\mathbb{Z}_1$	• strain $\perp [100]$<br>• ($\boldsymbol{E} \parallel [010]$ or $\boldsymbol{E} \perp [100]$)
$Ab'a'2$ (41.215) $\mathbb{Z}_2$	• $\boldsymbol{B} \parallel [001]$
$C2'$ (5.15) $\mathbb{Z}_1$	• $\boldsymbol{B} \parallel [100]$ and strain $\perp [001]$<br>• $\boldsymbol{B} \parallel [010]$ and strain $\perp [001]$<br>• $\boldsymbol{B} \perp [001]$
$Ama'2'$ (40.206) $\mathbb{Z}_1$	• $\boldsymbol{B} \parallel [100]$
C_c2 (5.16) $\mathbb{Z}_1$	• strain $\perp [001]$
$Ab'm2'$ (39.197) $\mathbb{Z}_1$	• $\boldsymbol{B} \parallel [010]$

Table. S 305: Subgroups of $Abm2$ (39.195) that can be realized by applying $\boldsymbol{E}/\boldsymbol{B}$ fields and/or uniaxial strains.

Subgroup & SI group	Perturbation
Cm (8.32) $\mathbb{Z}_1$	• strain $\perp [010]$<br>• $\boldsymbol{B} \parallel [010]$<br>• ($\boldsymbol{E} \parallel [100]$ or $\boldsymbol{E} \perp [010]$)
Pc (7.24) $\mathbb{Z}_1$	• strain $\perp [100]$<br>• $\boldsymbol{B} \parallel [100]$<br>• ($\boldsymbol{E} \parallel [010]$ or $\boldsymbol{E} \perp [100]$)
$C2$ (5.13) $\mathbb{Z}_1$	• strain $\perp [001]$<br>• $\boldsymbol{B} \parallel [001]$

Table. S 306: Subgroups of $Abm21'$ (39.196) that can be realized by applying $\boldsymbol{E}/\boldsymbol{B}$ fields and/or uniaxial strains.

Subgroup & SI group	Perturbation
$P11'$ (1.2) $\mathbb{Z}_1$	• strain in generic direction<br>• ($\boldsymbol{E} \parallel [100]$ or $\boldsymbol{E} \perp [010]$) and strain $\perp [100]$<br>• ($\boldsymbol{E} \parallel [100]$ or $\boldsymbol{E} \perp [010]$) and strain $\perp [001]$<br>• ($\boldsymbol{E} \parallel [010]$ or $\boldsymbol{E} \perp [100]$) and strain $\perp [010]$<br>• ($\boldsymbol{E} \parallel [010]$ or $\boldsymbol{E} \perp [100]$) and strain $\perp [001]$<br>• $\boldsymbol{E}$ in generic direction
Cm' (8.34) $\mathbb{Z}_1$	• $\boldsymbol{B} \parallel [100]$ and strain $\perp [010]$<br>• $\boldsymbol{B} \parallel [001]$ and strain $\perp [010]$<br>• $\boldsymbol{B} \perp [010]$<br>• ($\boldsymbol{E} \parallel [100]$ or $\boldsymbol{E} \perp [010]$) and $\boldsymbol{B} \parallel [100]$<br>• ($\boldsymbol{E} \parallel [100]$ or $\boldsymbol{E} \perp [010]$) and $\boldsymbol{B} \parallel [001]$
Cm (8.32) $\mathbb{Z}_1$	• $\boldsymbol{B} \parallel [010]$ and strain $\perp [010]$<br>• ($\boldsymbol{E} \parallel [100]$ or $\boldsymbol{E} \perp [010]$) and $\boldsymbol{B} \parallel [010]$
$Cm1'$ (8.33) $\mathbb{Z}_1$	• strain $\perp [010]$<br>• ($\boldsymbol{E} \parallel [100]$ or $\boldsymbol{E} \perp [010]$)
Pc' (7.26) $\mathbb{Z}_1$	• $\boldsymbol{B} \parallel [010]$ and strain $\perp [100]$<br>• $\boldsymbol{B} \parallel [001]$ and strain $\perp [100]$<br>• $\boldsymbol{B} \perp [100]$<br>• ($\boldsymbol{E} \parallel [010]$ or $\boldsymbol{E} \perp [100]$) and $\boldsymbol{B} \parallel [010]$<br>• ($\boldsymbol{E} \parallel [010]$ or $\boldsymbol{E} \perp [100]$) and $\boldsymbol{B} \parallel [001]$
Pc (7.24) $\mathbb{Z}_1$	• $\boldsymbol{B} \parallel [100]$ and strain $\perp [100]$<br>• ($\boldsymbol{E} \parallel [010]$ or $\boldsymbol{E} \perp [100]$) and $\boldsymbol{B} \parallel [100]$
$Pc1'$ (7.25) $\mathbb{Z}_1$	• strain $\perp [100]$<br>• ($\boldsymbol{E} \parallel [010]$ or $\boldsymbol{E} \perp [100]$)
$C2'$ (5.15) $\mathbb{Z}_1$	• $\boldsymbol{B} \parallel [100]$ and strain $\perp [001]$<br>• $\boldsymbol{B} \parallel [010]$ and strain $\perp [001]$<br>• $\boldsymbol{B} \perp [001]$
$Ab'm2'$ (39.197) $\mathbb{Z}_1$	• $\boldsymbol{B} \parallel [010]$
$Abm'2'$ (39.198) $\mathbb{Z}_1$	• $\boldsymbol{B} \parallel [100]$
$C2$ (5.13) $\mathbb{Z}_1$	• $\boldsymbol{B} \parallel [001]$ and strain $\perp [001]$
$Ab'm'2$ (39.199) $\mathbb{Z}_2$	• $\boldsymbol{B} \parallel [001]$
$C21'$ (5.14) $\mathbb{Z}_1$	• strain $\perp [001]$

Table. S 307: Subgroups of $Ab'm2'$ (39.197) that can be realized by applying $\boldsymbol{E}/\boldsymbol{B}$ fields and/or uniaxial strains.

Subgroup & SI group	Perturbation
Cm (8.32) $\mathbb{Z}_1$	• strain $\perp$ [010]<br>• ($\boldsymbol{E} \parallel$ [100] or $\boldsymbol{E} \perp$ [010])
Pc' (7.26) $\mathbb{Z}_1$	• strain $\perp$ [100]<br>• ($\boldsymbol{B} \parallel$ [001] or $\boldsymbol{B} \perp$ [100])<br>• ($\boldsymbol{E} \parallel$ [010] or $\boldsymbol{E} \perp$ [100])
$C2'$ (5.15) $\mathbb{Z}_1$	• strain $\perp$ [001]<br>• ($\boldsymbol{B} \parallel$ [100] or $\boldsymbol{B} \perp$ [001])

Table. S 308: Subgroups of $Abm'2'$ (39.198) that can be realized by applying $\boldsymbol{E}/\boldsymbol{B}$ fields and/or uniaxial strains.

Subgroup & SI group	Perturbation
Cm' (8.34) $\mathbb{Z}_1$	• strain $\perp$ [010]<br>• ($\boldsymbol{B} \parallel$ [001] or $\boldsymbol{B} \perp$ [010])<br>• ($\boldsymbol{E} \parallel$ [100] or $\boldsymbol{E} \perp$ [010])
Pc (7.24) $\mathbb{Z}_1$	• strain $\perp$ [100]<br>• ($\boldsymbol{E} \parallel$ [010] or $\boldsymbol{E} \perp$ [100])
$C2'$ (5.15) $\mathbb{Z}_1$	• strain $\perp$ [001]<br>• ($\boldsymbol{B} \parallel$ [010] or $\boldsymbol{B} \perp$ [001])

Table. S 309: Subgroups of $Ab'm'2$ (39.199) that can be realized by applying $\boldsymbol{E}/\boldsymbol{B}$ fields and/or uniaxial strains.

Subgroup & SI group	Perturbation
Cm' (8.34) $\mathbb{Z}_1$	• strain $\perp$ [010]<br>• ($\boldsymbol{B} \parallel$ [100] or $\boldsymbol{B} \perp$ [010])<br>• ($\boldsymbol{E} \parallel$ [100] or $\boldsymbol{E} \perp$ [010])
Pc' (7.26) $\mathbb{Z}_1$	• strain $\perp$ [100]<br>• ($\boldsymbol{B} \parallel$ [010] or $\boldsymbol{B} \perp$ [100])<br>• ($\boldsymbol{E} \parallel$ [010] or $\boldsymbol{E} \perp$ [100])

Continued on next page

. . . continued

Subgroup & SI group	Perturbation
$C2$ (5.13) $\mathbb{Z}_1$	• strain $\perp$ [001]

Table. S 310: Subgroups of A_abm2 (39.200) that can be realized by applying $\boldsymbol{E}/\boldsymbol{B}$ fields and/or uniaxial strains.

Subgroup & SI group	Perturbation
Cm (8.32) $\mathbb{Z}_1$	• $\boldsymbol{B} \parallel$ [010] and strain $\perp$ [010]<br>• ($\boldsymbol{E} \parallel$ [100] or $\boldsymbol{E} \perp$ [010]) and $\boldsymbol{B} \parallel$ [010]
P_S1 (1.3) $\mathbb{Z}_1$	• strain in generic direction<br>• ($\boldsymbol{E} \parallel$ [100] or $\boldsymbol{E} \perp$ [010]) and strain $\perp$ [100]<br>• ($\boldsymbol{E} \parallel$ [100] or $\boldsymbol{E} \perp$ [010]) and strain $\perp$ [001]<br>• ($\boldsymbol{E} \parallel$ [010] or $\boldsymbol{E} \perp$ [100]) and strain $\perp$ [010]<br>• ($\boldsymbol{E} \parallel$ [010] or $\boldsymbol{E} \perp$ [100]) and strain $\perp$ [001]<br>• $\boldsymbol{E}$ in generic direction
Cc' (9.39) $\mathbb{Z}_1$	• $\boldsymbol{B} \parallel$ [100] and strain $\perp$ [010]<br>• $\boldsymbol{B} \parallel$ [001] and strain $\perp$ [010]<br>• $\boldsymbol{B} \perp$ [010]<br>• ($\boldsymbol{E} \parallel$ [100] or $\boldsymbol{E} \perp$ [010]) and $\boldsymbol{B} \parallel$ [100]<br>• ($\boldsymbol{E} \parallel$ [100] or $\boldsymbol{E} \perp$ [010]) and $\boldsymbol{B} \parallel$ [001]
C_cm (8.35) $\mathbb{Z}_1$	• strain $\perp$ [010]<br>• ($\boldsymbol{E} \parallel$ [100] or $\boldsymbol{E} \perp$ [010])
Pc (7.24) $\mathbb{Z}_1$	• $\boldsymbol{B} \parallel$ [100] and strain $\perp$ [100]<br>• ($\boldsymbol{E} \parallel$ [010] or $\boldsymbol{E} \perp$ [100]) and $\boldsymbol{B} \parallel$ [100]
$C2$ (5.13) $\mathbb{Z}_1$	• $\boldsymbol{B} \parallel$ [001] and strain $\perp$ [001]
Pc' (7.26) $\mathbb{Z}_1$	• $\boldsymbol{B} \parallel$ [010] and strain $\perp$ [100]<br>• $\boldsymbol{B} \parallel$ [001] and strain $\perp$ [100]<br>• $\boldsymbol{B} \perp$ [100]<br>• ($\boldsymbol{E} \parallel$ [010] or $\boldsymbol{E} \perp$ [100]) and $\boldsymbol{B} \parallel$ [010]<br>• ($\boldsymbol{E} \parallel$ [010] or $\boldsymbol{E} \perp$ [100]) and $\boldsymbol{B} \parallel$ [001]
P_bc (7.29) $\mathbb{Z}_1$	• strain $\perp$ [100]<br>• ($\boldsymbol{E} \parallel$ [010] or $\boldsymbol{E} \perp$ [100])
$Ab'a'2$ (41.215) $\mathbb{Z}_2$	• $\boldsymbol{B} \parallel$ [001]

Continued on next page

. . . continued

Subgroup & SI group	Perturbation
$C2'$ (5.15) $\mathbb{Z}_1$	• $\boldsymbol{B} \parallel [100]$ and strain $\perp [001]$<br>• $\boldsymbol{B} \parallel [010]$ and strain $\perp [001]$<br>• $\boldsymbol{B} \perp [001]$
$Aba'2'$ (41.214) $\mathbb{Z}_1$	• $\boldsymbol{B} \parallel [100]$
C_c2 (5.16) $\mathbb{Z}_1$	• strain $\perp [001]$
$Ab'm2'$ (39.197) $\mathbb{Z}_1$	• $\boldsymbol{B} \parallel [010]$

Table. S 311: Subgroups of A_bbm2 (39.201) that can be realized by applying $\boldsymbol{E}/\boldsymbol{B}$ fields and/or uniaxial strains.

Subgroup & SI group	Perturbation
P_S1 (1.3) $\mathbb{Z}_1$	• strain in generic direction<br>• ($\boldsymbol{E} \parallel [100]$ or $\boldsymbol{E} \perp [010]$) and strain $\perp [100]$<br>• ($\boldsymbol{E} \parallel [100]$ or $\boldsymbol{E} \perp [010]$) and strain $\perp [001]$<br>• ($\boldsymbol{E} \parallel [010]$ or $\boldsymbol{E} \perp [100]$) and strain $\perp [010]$<br>• ($\boldsymbol{E} \parallel [010]$ or $\boldsymbol{E} \perp [100]$) and strain $\perp [001]$<br>• $\boldsymbol{E}$ in generic direction
Cm (8.32) $\mathbb{Z}_1$	• $\boldsymbol{B} \parallel [010]$ and strain $\perp [010]$<br>• ($\boldsymbol{E} \parallel [100]$ or $\boldsymbol{E} \perp [010]$) and $\boldsymbol{B} \parallel [010]$
Cm' (8.34) $\mathbb{Z}_1$	• $\boldsymbol{B} \parallel [100]$ and strain $\perp [010]$<br>• $\boldsymbol{B} \parallel [001]$ and strain $\perp [010]$<br>• $\boldsymbol{B} \perp [010]$<br>• ($\boldsymbol{E} \parallel [100]$ or $\boldsymbol{E} \perp [010]$) and $\boldsymbol{B} \parallel [100]$<br>• ($\boldsymbol{E} \parallel [100]$ or $\boldsymbol{E} \perp [010]$) and $\boldsymbol{B} \parallel [001]$
C_am (8.36) $\mathbb{Z}_1$	• strain $\perp [010]$<br>• ($\boldsymbol{E} \parallel [100]$ or $\boldsymbol{E} \perp [010]$)
Pc (7.24) $\mathbb{Z}_1$	• $\boldsymbol{B} \parallel [100]$ and strain $\perp [100]$<br>• ($\boldsymbol{E} \parallel [010]$ or $\boldsymbol{E} \perp [100]$) and $\boldsymbol{B} \parallel [100]$
Pm' (6.20) $\mathbb{Z}_1$	• $\boldsymbol{B} \parallel [010]$ and strain $\perp [100]$<br>• $\boldsymbol{B} \parallel [001]$ and strain $\perp [100]$<br>• $\boldsymbol{B} \perp [100]$<br>• ($\boldsymbol{E} \parallel [010]$ or $\boldsymbol{E} \perp [100]$) and $\boldsymbol{B} \parallel [010]$<br>• ($\boldsymbol{E} \parallel [010]$ or $\boldsymbol{E} \perp [100]$) and $\boldsymbol{B} \parallel [001]$

Continued on next page

...continued

Subgroup & SI group	Perturbation
$P_c c$ (7.28) $\mathbb{Z}_1$	• strain $\perp$ [100]<br>• ($\boldsymbol{E} \parallel$ [010] or $\boldsymbol{E} \perp$ [100])
$C2'$ (5.15) $\mathbb{Z}_1$	• $\boldsymbol{B} \parallel$ [100] and strain $\perp$ [001]<br>• $\boldsymbol{B} \parallel$ [010] and strain $\perp$ [001]<br>• $\boldsymbol{B} \perp$ [001]
$Abm'2'$ (39.198) $\mathbb{Z}_1$	• $\boldsymbol{B} \parallel$ [100]
$Am'm2'$ (38.189) $\mathbb{Z}_1$	• $\boldsymbol{B} \parallel$ [010]
$C2$ (5.13) $\mathbb{Z}_1$	• $\boldsymbol{B} \parallel$ [001] and strain $\perp$ [001]
$Am'm'2$ (38.191) $\mathbb{Z}_1$	• $\boldsymbol{B} \parallel$ [001]
$C_a 2$ (5.17) $\mathbb{Z}_2$	• strain $\perp$ [001]

Table. S 312: Subgroups of $A_B bm2$ (39.202) that can be realized by applying $\boldsymbol{E}/\boldsymbol{B}$ fields and/or uniaxial strains.

Subgroup & SI group	Perturbation
Cm (8.32) $\mathbb{Z}_1$	• $\boldsymbol{B} \parallel$ [010] and strain $\perp$ [010]<br>• ($\boldsymbol{E} \parallel$ [100] or $\boldsymbol{E} \perp$ [010]) and $\boldsymbol{B} \parallel$ [010]
$P_S 1$ (1.3) $\mathbb{Z}_1$	• strain in generic direction<br>• ($\boldsymbol{E} \parallel$ [100] or $\boldsymbol{E} \perp$ [010]) and strain $\perp$ [100]<br>• ($\boldsymbol{E} \parallel$ [100] or $\boldsymbol{E} \perp$ [010]) and strain $\perp$ [001]<br>• ($\boldsymbol{E} \parallel$ [010] or $\boldsymbol{E} \perp$ [100]) and strain $\perp$ [010]<br>• ($\boldsymbol{E} \parallel$ [010] or $\boldsymbol{E} \perp$ [100]) and strain $\perp$ [001]<br>• $\boldsymbol{E}$ in generic direction
Cc' (9.39) $\mathbb{Z}_1$	• $\boldsymbol{B} \parallel$ [100] and strain $\perp$ [010]<br>• $\boldsymbol{B} \parallel$ [001] and strain $\perp$ [010]<br>• $\boldsymbol{B} \perp$ [010]<br>• ($\boldsymbol{E} \parallel$ [100] or $\boldsymbol{E} \perp$ [010]) and $\boldsymbol{B} \parallel$ [100]<br>• ($\boldsymbol{E} \parallel$ [100] or $\boldsymbol{E} \perp$ [010]) and $\boldsymbol{B} \parallel$ [001]
$C_c m$ (8.35) $\mathbb{Z}_1$	• strain $\perp$ [010]<br>• ($\boldsymbol{E} \parallel$ [100] or $\boldsymbol{E} \perp$ [010])
Pc (7.24) $\mathbb{Z}_1$	• $\boldsymbol{B} \parallel$ [100] and strain $\perp$ [100]<br>• ($\boldsymbol{E} \parallel$ [010] or $\boldsymbol{E} \perp$ [100]) and $\boldsymbol{B} \parallel$ [100]
$C2$ (5.13) $\mathbb{Z}_1$	• $\boldsymbol{B} \parallel$ [001] and strain $\perp$ [001]

Continued on next page

...continued

Subgroup & SI group	Perturbation
Pm' (6.20) $\mathbb{Z}_1$	• $\boldsymbol{B}$ ∥ [010] and strain ⊥ [100]<br>• $\boldsymbol{B}$ ∥ [001] and strain ⊥ [100]<br>• $\boldsymbol{B}$ ⊥ [100]<br>• ($\boldsymbol{E}$ ∥ [010] or $\boldsymbol{E}$ ⊥ [100]) and $\boldsymbol{B}$ ∥ [010]<br>• ($\boldsymbol{E}$ ∥ [010] or $\boldsymbol{E}$ ⊥ [100]) and $\boldsymbol{B}$ ∥ [001]
P_Ac (7.31) $\mathbb{Z}_1$	• strain ⊥ [100]<br>• ($\boldsymbol{E}$ ∥ [010] or $\boldsymbol{E}$ ⊥ [100])
$Am'a'2$ (40.207) $\mathbb{Z}_1$	• $\boldsymbol{B}$ ∥ [001]
$C2'$ (5.15) $\mathbb{Z}_1$	• $\boldsymbol{B}$ ∥ [100] and strain ⊥ [001]<br>• $\boldsymbol{B}$ ∥ [010] and strain ⊥ [001]<br>• $\boldsymbol{B}$ ⊥ [001]
$Aba'2'$ (41.214) $\mathbb{Z}_1$	• $\boldsymbol{B}$ ∥ [100]
C_c2 (5.16) $\mathbb{Z}_1$	• strain ⊥ [001]
$Am'm2'$ (38.189) $\mathbb{Z}_1$	• $\boldsymbol{B}$ ∥ [010]

Table. S 313: Subgroups of $Ama2$ (40.203) that can be realized by applying $\boldsymbol{E}/\boldsymbol{B}$ fields and/or uniaxial strains.

Subgroup & SI group	Perturbation
Cc (9.37) $\mathbb{Z}_1$	• strain ⊥ [010]<br>• $\boldsymbol{B}$ ∥ [010]<br>• ($\boldsymbol{E}$ ∥ [100] or $\boldsymbol{E}$ ⊥ [010])
$C2$ (5.13) $\mathbb{Z}_1$	• strain ⊥ [001]<br>• $\boldsymbol{B}$ ∥ [001]
Pm (6.18) $\mathbb{Z}_1$	• strain ⊥ [100]<br>• $\boldsymbol{B}$ ∥ [100]<br>• ($\boldsymbol{E}$ ∥ [010] or $\boldsymbol{E}$ ⊥ [100])

Table. S 314: Subgroups of $Ama21'$ (40.204) that can be realized by applying $\boldsymbol{E}/\boldsymbol{B}$ fields and/or uniaxial strains.

Subgroup & SI group	Perturbation
$P11'$ (1.2) $\mathbb{Z}_1$	• strain in generic direction<br>• ($\boldsymbol{E} \parallel [100]$ or $\boldsymbol{E} \perp [010]$) and strain $\perp [100]$<br>• ($\boldsymbol{E} \parallel [100]$ or $\boldsymbol{E} \perp [010]$) and strain $\perp [001]$<br>• ($\boldsymbol{E} \parallel [010]$ or $\boldsymbol{E} \perp [100]$) and strain $\perp [010]$<br>• ($\boldsymbol{E} \parallel [010]$ or $\boldsymbol{E} \perp [100]$) and strain $\perp [001]$<br>• $\boldsymbol{E}$ in generic direction
Cc' (9.39) $\mathbb{Z}_1$	• $\boldsymbol{B} \parallel [100]$ and strain $\perp [010]$<br>• $\boldsymbol{B} \parallel [001]$ and strain $\perp [010]$<br>• $\boldsymbol{B} \perp [010]$<br>• ($\boldsymbol{E} \parallel [100]$ or $\boldsymbol{E} \perp [010]$) and $\boldsymbol{B} \parallel [100]$<br>• ($\boldsymbol{E} \parallel [100]$ or $\boldsymbol{E} \perp [010]$) and $\boldsymbol{B} \parallel [001]$
Cc (9.37) $\mathbb{Z}_1$	• $\boldsymbol{B} \parallel [010]$ and strain $\perp [010]$<br>• ($\boldsymbol{E} \parallel [100]$ or $\boldsymbol{E} \perp [010]$) and $\boldsymbol{B} \parallel [010]$
$Cc1'$ (9.38) $\mathbb{Z}_1$	• strain $\perp [010]$<br>• ($\boldsymbol{E} \parallel [100]$ or $\boldsymbol{E} \perp [010]$)
$C2'$ (5.15) $\mathbb{Z}_1$	• $\boldsymbol{B} \parallel [100]$ and strain $\perp [001]$<br>• $\boldsymbol{B} \parallel [010]$ and strain $\perp [001]$<br>• $\boldsymbol{B} \perp [001]$
$C2$ (5.13) $\mathbb{Z}_1$	• $\boldsymbol{B} \parallel [001]$ and strain $\perp [001]$
$C21'$ (5.14) $\mathbb{Z}_1$	• strain $\perp [001]$
Pm' (6.20) $\mathbb{Z}_1$	• $\boldsymbol{B} \parallel [010]$ and strain $\perp [100]$<br>• $\boldsymbol{B} \parallel [001]$ and strain $\perp [100]$<br>• $\boldsymbol{B} \perp [100]$<br>• ($\boldsymbol{E} \parallel [010]$ or $\boldsymbol{E} \perp [100]$) and $\boldsymbol{B} \parallel [010]$<br>• ($\boldsymbol{E} \parallel [010]$ or $\boldsymbol{E} \perp [100]$) and $\boldsymbol{B} \parallel [001]$
$Am'a2'$ (40.205) $\mathbb{Z}_1$	• $\boldsymbol{B} \parallel [010]$
$Am'a'2$ (40.207) $\mathbb{Z}_1$	• $\boldsymbol{B} \parallel [001]$
Pm (6.18) $\mathbb{Z}_1$	• $\boldsymbol{B} \parallel [100]$ and strain $\perp [100]$<br>• ($\boldsymbol{E} \parallel [010]$ or $\boldsymbol{E} \perp [100]$) and $\boldsymbol{B} \parallel [100]$
$Ama'2'$ (40.206) $\mathbb{Z}_1$	• $\boldsymbol{B} \parallel [100]$
$Pm1'$ (6.19) $\mathbb{Z}_1$	• strain $\perp [100]$<br>• ($\boldsymbol{E} \parallel [010]$ or $\boldsymbol{E} \perp [100]$)

Table. S 315: Subgroups of $Am'a2'$ (40.205) that can be realized by applying $\boldsymbol{E}/\boldsymbol{B}$ fields and/or uniaxial strains.

Subgroup & SI group	Perturbation
Cc (9.37) $\mathbb{Z}_1$	• strain $\perp$ [010]<br>• ($\boldsymbol{E} \parallel$ [100] or $\boldsymbol{E} \perp$ [010])
$C2'$ (5.15) $\mathbb{Z}_1$	• strain $\perp$ [001]<br>• ($\boldsymbol{B} \parallel$ [100] or $\boldsymbol{B} \perp$ [001])
Pm' (6.20) $\mathbb{Z}_1$	• strain $\perp$ [100]<br>• ($\boldsymbol{B} \parallel$ [001] or $\boldsymbol{B} \perp$ [100])<br>• ($\boldsymbol{E} \parallel$ [010] or $\boldsymbol{E} \perp$ [100])

Table. S 316: Subgroups of $Ama'2'$ (40.206) that can be realized by applying $\boldsymbol{E}/\boldsymbol{B}$ fields and/or uniaxial strains.

Subgroup & SI group	Perturbation
Cc' (9.39) $\mathbb{Z}_1$	• strain $\perp$ [010]<br>• ($\boldsymbol{B} \parallel$ [001] or $\boldsymbol{B} \perp$ [010])<br>• ($\boldsymbol{E} \parallel$ [100] or $\boldsymbol{E} \perp$ [010])
$C2'$ (5.15) $\mathbb{Z}_1$	• strain $\perp$ [001]<br>• ($\boldsymbol{B} \parallel$ [010] or $\boldsymbol{B} \perp$ [001])
Pm (6.18) $\mathbb{Z}_1$	• strain $\perp$ [100]<br>• ($\boldsymbol{E} \parallel$ [010] or $\boldsymbol{E} \perp$ [100])

Table. S 317: Subgroups of $Am'a'2$ (40.207) that can be realized by applying $\boldsymbol{E}/\boldsymbol{B}$ fields and/or uniaxial strains.

Subgroup & SI group	Perturbation
Cc' (9.39) $\mathbb{Z}_1$	• strain $\perp$ [010]<br>• ($\boldsymbol{B} \parallel$ [100] or $\boldsymbol{B} \perp$ [010])<br>• ($\boldsymbol{E} \parallel$ [100] or $\boldsymbol{E} \perp$ [010])
$C2$ (5.13) $\mathbb{Z}_1$	• strain $\perp$ [001]

Continued on next page

...continued

Subgroup & SI group	Perturbation
Pm' (6.20) $\mathbb{Z}_1$	• strain $\perp$ [100]<br>• ($\boldsymbol{B} \parallel$ [010] or $\boldsymbol{B} \perp$ [100])<br>• ($\boldsymbol{E} \parallel$ [010] or $\boldsymbol{E} \perp$ [100])

Table. S 318: Subgroups of A_ama2 (40.208) that can be realized by applying $\boldsymbol{E}/\boldsymbol{B}$ fields and/or uniaxial strains.

Subgroup & SI group	Perturbation
Cm' (8.34) $\mathbb{Z}_1$	• $\boldsymbol{B} \parallel$ [100] and strain $\perp$ [010]<br>• $\boldsymbol{B} \parallel$ [001] and strain $\perp$ [010]<br>• $\boldsymbol{B} \perp$ [010]<br>• ($\boldsymbol{E} \parallel$ [100] or $\boldsymbol{E} \perp$ [010]) and $\boldsymbol{B} \parallel$ [100]<br>• ($\boldsymbol{E} \parallel$ [100] or $\boldsymbol{E} \perp$ [010]) and $\boldsymbol{B} \parallel$ [001]
P_S1 (1.3) $\mathbb{Z}_1$	• strain in generic direction<br>• ($\boldsymbol{E} \parallel$ [100] or $\boldsymbol{E} \perp$ [010]) and strain $\perp$ [100]<br>• ($\boldsymbol{E} \parallel$ [100] or $\boldsymbol{E} \perp$ [010]) and strain $\perp$ [001]<br>• ($\boldsymbol{E} \parallel$ [010] or $\boldsymbol{E} \perp$ [100]) and strain $\perp$ [010]<br>• ($\boldsymbol{E} \parallel$ [010] or $\boldsymbol{E} \perp$ [100]) and strain $\perp$ [001]<br>• $\boldsymbol{E}$ in generic direction
Cc (9.37) $\mathbb{Z}_1$	• $\boldsymbol{B} \parallel$ [010] and strain $\perp$ [010]<br>• ($\boldsymbol{E} \parallel$ [100] or $\boldsymbol{E} \perp$ [010]) and $\boldsymbol{B} \parallel$ [010]
C_cc (9.40) $\mathbb{Z}_1$	• strain $\perp$ [010]<br>• ($\boldsymbol{E} \parallel$ [100] or $\boldsymbol{E} \perp$ [010])
Pm' (6.20) $\mathbb{Z}_1$	• $\boldsymbol{B} \parallel$ [010] and strain $\perp$ [100]<br>• $\boldsymbol{B} \parallel$ [001] and strain $\perp$ [100]<br>• $\boldsymbol{B} \perp$ [100]<br>• ($\boldsymbol{E} \parallel$ [010] or $\boldsymbol{E} \perp$ [100]) and $\boldsymbol{B} \parallel$ [010]<br>• ($\boldsymbol{E} \parallel$ [010] or $\boldsymbol{E} \perp$ [100]) and $\boldsymbol{B} \parallel$ [001]
$C2$ (5.13) $\mathbb{Z}_1$	• $\boldsymbol{B} \parallel$ [001] and strain $\perp$ [001]
$Am'm'2$ (38.191) $\mathbb{Z}_1$	• $\boldsymbol{B} \parallel$ [001]
Pm (6.18) $\mathbb{Z}_1$	• $\boldsymbol{B} \parallel$ [100] and strain $\perp$ [100]<br>• ($\boldsymbol{E} \parallel$ [010] or $\boldsymbol{E} \perp$ [100]) and $\boldsymbol{B} \parallel$ [100]
P_bm (6.22) $\mathbb{Z}_1$	• strain $\perp$ [100]<br>• ($\boldsymbol{E} \parallel$ [010] or $\boldsymbol{E} \perp$ [100])

Continued on next page

. . . continued

Subgroup & SI group	Perturbation
$C2'$ (5.15) $\mathbb{Z}_1$	• $\boldsymbol{B}$ ∥ [100] and strain ⊥ [001]<br>• $\boldsymbol{B}$ ∥ [010] and strain ⊥ [001]<br>• $\boldsymbol{B}$ ⊥ [001]
$Am'a2'$ (40.205) $\mathbb{Z}_1$	• $\boldsymbol{B}$ ∥ [010]
C_c2 (5.16) $\mathbb{Z}_1$	• strain ⊥ [001]
$Amm'2'$ (38.190) $\mathbb{Z}_1$	• $\boldsymbol{B}$ ∥ [100]

Table. S 319: Subgroups of A_bma2 (40.209) that can be realized by applying $\boldsymbol{E}/\boldsymbol{B}$ fields and/or uniaxial strains.

Subgroup & SI group	Perturbation
P_S1 (1.3) $\mathbb{Z}_1$	• strain in generic direction<br>• ($\boldsymbol{E}$ ∥ [100] or $\boldsymbol{E}$ ⊥ [010]) and strain ⊥ [100]<br>• ($\boldsymbol{E}$ ∥ [100] or $\boldsymbol{E}$ ⊥ [010]) and strain ⊥ [001]<br>• ($\boldsymbol{E}$ ∥ [010] or $\boldsymbol{E}$ ⊥ [100]) and strain ⊥ [010]<br>• ($\boldsymbol{E}$ ∥ [010] or $\boldsymbol{E}$ ⊥ [100]) and strain ⊥ [001]<br>• $\boldsymbol{E}$ in generic direction
Cc' (9.39) $\mathbb{Z}_1$	• $\boldsymbol{B}$ ∥ [100] and strain ⊥ [010]<br>• $\boldsymbol{B}$ ∥ [001] and strain ⊥ [010]<br>• $\boldsymbol{B}$ ⊥ [010]<br>• ($\boldsymbol{E}$ ∥ [100] or $\boldsymbol{E}$ ⊥ [010]) and $\boldsymbol{B}$ ∥ [100]<br>• ($\boldsymbol{E}$ ∥ [100] or $\boldsymbol{E}$ ⊥ [010]) and $\boldsymbol{B}$ ∥ [001]
Cc (9.37) $\mathbb{Z}_1$	• $\boldsymbol{B}$ ∥ [010] and strain ⊥ [010]<br>• ($\boldsymbol{E}$ ∥ [100] or $\boldsymbol{E}$ ⊥ [010]) and $\boldsymbol{B}$ ∥ [010]
C_ac (9.41) $\mathbb{Z}_2$	• strain ⊥ [010]<br>• ($\boldsymbol{E}$ ∥ [100] or $\boldsymbol{E}$ ⊥ [010])
$C2'$ (5.15) $\mathbb{Z}_1$	• $\boldsymbol{B}$ ∥ [100] and strain ⊥ [001]<br>• $\boldsymbol{B}$ ∥ [010] and strain ⊥ [001]<br>• $\boldsymbol{B}$ ⊥ [001]
$C2$ (5.13) $\mathbb{Z}_1$	• $\boldsymbol{B}$ ∥ [001] and strain ⊥ [001]
C_a2 (5.17) $\mathbb{Z}_2$	• strain ⊥ [001]

Continued on next page

...continued

Subgroup & SI group	Perturbation
Pc' (7.26) $\mathbb{Z}_1$	• $\boldsymbol{B}$ ∥ [010] and strain ⊥ [100]<br>• $\boldsymbol{B}$ ∥ [001] and strain ⊥ [100]<br>• $\boldsymbol{B}$ ⊥ [100]<br>• ($\boldsymbol{E}$ ∥ [010] or $\boldsymbol{E}$ ⊥ [100]) and $\boldsymbol{B}$ ∥ [010]<br>• ($\boldsymbol{E}$ ∥ [010] or $\boldsymbol{E}$ ⊥ [100]) and $\boldsymbol{B}$ ∥ [001]
$Ab'a2'$ (41.213) $\mathbb{Z}_1$	• $\boldsymbol{B}$ ∥ [010]
$Ab'a'2$ (41.215) $\mathbb{Z}_2$	• $\boldsymbol{B}$ ∥ [001]
Pm (6.18) $\mathbb{Z}_1$	• $\boldsymbol{B}$ ∥ [100] and strain ⊥ [100]<br>• ($\boldsymbol{E}$ ∥ [010] or $\boldsymbol{E}$ ⊥ [100]) and $\boldsymbol{B}$ ∥ [100]
$Ama'2'$ (40.206) $\mathbb{Z}_1$	• $\boldsymbol{B}$ ∥ [100]
P_am (6.21) $\mathbb{Z}_1$	• strain ⊥ [100]<br>• ($\boldsymbol{E}$ ∥ [010] or $\boldsymbol{E}$ ⊥ [100])

Table. S 320: Subgroups of A_Bma2 (40.210) that can be realized by applying $\boldsymbol{E}/\boldsymbol{B}$ fields and/or uniaxial strains.

Subgroup & SI group	Perturbation
Cm' (8.34) $\mathbb{Z}_1$	• $\boldsymbol{B}$ ∥ [100] and strain ⊥ [010]<br>• $\boldsymbol{B}$ ∥ [001] and strain ⊥ [010]<br>• $\boldsymbol{B}$ ⊥ [010]<br>• ($\boldsymbol{E}$ ∥ [100] or $\boldsymbol{E}$ ⊥ [010]) and $\boldsymbol{B}$ ∥ [100]<br>• ($\boldsymbol{E}$ ∥ [100] or $\boldsymbol{E}$ ⊥ [010]) and $\boldsymbol{B}$ ∥ [001]
P_S1 (1.3) $\mathbb{Z}_1$	• strain in generic direction<br>• ($\boldsymbol{E}$ ∥ [100] or $\boldsymbol{E}$ ⊥ [010]) and strain ⊥ [100]<br>• ($\boldsymbol{E}$ ∥ [100] or $\boldsymbol{E}$ ⊥ [010]) and strain ⊥ [001]<br>• ($\boldsymbol{E}$ ∥ [010] or $\boldsymbol{E}$ ⊥ [100]) and strain ⊥ [010]<br>• ($\boldsymbol{E}$ ∥ [010] or $\boldsymbol{E}$ ⊥ [100]) and strain ⊥ [001]<br>• $\boldsymbol{E}$ in generic direction
Cc (9.37) $\mathbb{Z}_1$	• $\boldsymbol{B}$ ∥ [010] and strain ⊥ [010]<br>• ($\boldsymbol{E}$ ∥ [100] or $\boldsymbol{E}$ ⊥ [010]) and $\boldsymbol{B}$ ∥ [010]
C_cc (9.40) $\mathbb{Z}_1$	• strain ⊥ [010]<br>• ($\boldsymbol{E}$ ∥ [100] or $\boldsymbol{E}$ ⊥ [010])

Continued on next page

…continued

Subgroup & SI group	Perturbation
Pc' (7.26) $\mathbb{Z}_1$	• $\boldsymbol{B}$ ∥ [010] and strain ⊥ [100]<br>• $\boldsymbol{B}$ ∥ [001] and strain ⊥ [100]<br>• $\boldsymbol{B}$ ⊥ [100]<br>• ($\boldsymbol{E}$ ∥ [010] or $\boldsymbol{E}$ ⊥ [100]) and $\boldsymbol{B}$ ∥ [010]<br>• ($\boldsymbol{E}$ ∥ [010] or $\boldsymbol{E}$ ⊥ [100]) and $\boldsymbol{B}$ ∥ [001]
$C2$ (5.13) $\mathbb{Z}_1$	• $\boldsymbol{B}$ ∥ [001] and strain ⊥ [001]
$Ab'm'2$ (39.199) $\mathbb{Z}_2$	• $\boldsymbol{B}$ ∥ [001]
Pm (6.18) $\mathbb{Z}_1$	• $\boldsymbol{B}$ ∥ [100] and strain ⊥ [100]<br>• ($\boldsymbol{E}$ ∥ [010] or $\boldsymbol{E}$ ⊥ [100]) and $\boldsymbol{B}$ ∥ [100]
P_Cm (6.23) $\mathbb{Z}_1$	• strain ⊥ [100]<br>• ($\boldsymbol{E}$ ∥ [010] or $\boldsymbol{E}$ ⊥ [100])
$C2'$ (5.15) $\mathbb{Z}_1$	• $\boldsymbol{B}$ ∥ [100] and strain ⊥ [001]<br>• $\boldsymbol{B}$ ∥ [010] and strain ⊥ [001]<br>• $\boldsymbol{B}$ ⊥ [001]
$Ab'a2'$ (41.213) $\mathbb{Z}_1$	• $\boldsymbol{B}$ ∥ [010]
C_c2 (5.16) $\mathbb{Z}_1$	• strain ⊥ [001]
$Amm'2'$ (38.190) $\mathbb{Z}_1$	• $\boldsymbol{B}$ ∥ [100]

Table. S 321: Subgroups of $Aba2$ (41.211) that can be realized by applying $\boldsymbol{E}/\boldsymbol{B}$ fields and/or uniaxial strains.

Subgroup & SI group	Perturbation
Cc (9.37) $\mathbb{Z}_1$	• strain ⊥ [010]<br>• $\boldsymbol{B}$ ∥ [010]<br>• ($\boldsymbol{E}$ ∥ [100] or $\boldsymbol{E}$ ⊥ [010])
$C2$ (5.13) $\mathbb{Z}_1$	• strain ⊥ [001]<br>• $\boldsymbol{B}$ ∥ [001]
Pc (7.24) $\mathbb{Z}_1$	• strain ⊥ [100]<br>• $\boldsymbol{B}$ ∥ [100]<br>• ($\boldsymbol{E}$ ∥ [010] or $\boldsymbol{E}$ ⊥ [100])

Table. S 322: Subgroups of $Aba21'$ (41.212) that can be realized by applying $\boldsymbol{E}/\boldsymbol{B}$ fields and/or uniaxial strains.

Subgroup & SI group	Perturbation
$P11'$ (1.2) $\mathbb{Z}_1$	• strain in generic direction<br>• ($\boldsymbol{E} \parallel [100]$ or $\boldsymbol{E} \perp [010]$) and strain $\perp [100]$<br>• ($\boldsymbol{E} \parallel [100]$ or $\boldsymbol{E} \perp [010]$) and strain $\perp [001]$<br>• ($\boldsymbol{E} \parallel [010]$ or $\boldsymbol{E} \perp [100]$) and strain $\perp [010]$<br>• ($\boldsymbol{E} \parallel [010]$ or $\boldsymbol{E} \perp [100]$) and strain $\perp [001]$<br>• $\boldsymbol{E}$ in generic direction
Cc' (9.39) $\mathbb{Z}_1$	• $\boldsymbol{B} \parallel [100]$ and strain $\perp [010]$<br>• $\boldsymbol{B} \parallel [001]$ and strain $\perp [010]$<br>• $\boldsymbol{B} \perp [010]$<br>• ($\boldsymbol{E} \parallel [100]$ or $\boldsymbol{E} \perp [010]$) and $\boldsymbol{B} \parallel [100]$<br>• ($\boldsymbol{E} \parallel [100]$ or $\boldsymbol{E} \perp [010]$) and $\boldsymbol{B} \parallel [001]$
Cc (9.37) $\mathbb{Z}_1$	• $\boldsymbol{B} \parallel [010]$ and strain $\perp [010]$<br>• ($\boldsymbol{E} \parallel [100]$ or $\boldsymbol{E} \perp [010]$) and $\boldsymbol{B} \parallel [010]$
$Cc1'$ (9.38) $\mathbb{Z}_1$	• strain $\perp [010]$<br>• ($\boldsymbol{E} \parallel [100]$ or $\boldsymbol{E} \perp [010]$)
$C2'$ (5.15) $\mathbb{Z}_1$	• $\boldsymbol{B} \parallel [100]$ and strain $\perp [001]$<br>• $\boldsymbol{B} \parallel [010]$ and strain $\perp [001]$<br>• $\boldsymbol{B} \perp [001]$
$C2$ (5.13) $\mathbb{Z}_1$	• $\boldsymbol{B} \parallel [001]$ and strain $\perp [001]$
$C21'$ (5.14) $\mathbb{Z}_1$	• strain $\perp [001]$
Pc' (7.26) $\mathbb{Z}_1$	• $\boldsymbol{B} \parallel [010]$ and strain $\perp [100]$<br>• $\boldsymbol{B} \parallel [001]$ and strain $\perp [100]$<br>• $\boldsymbol{B} \perp [100]$<br>• ($\boldsymbol{E} \parallel [010]$ or $\boldsymbol{E} \perp [100]$) and $\boldsymbol{B} \parallel [010]$<br>• ($\boldsymbol{E} \parallel [010]$ or $\boldsymbol{E} \perp [100]$) and $\boldsymbol{B} \parallel [001]$
$Ab'a2'$ (41.213) $\mathbb{Z}_1$	• $\boldsymbol{B} \parallel [010]$
$Ab'a'2$ (41.215) $\mathbb{Z}_2$	• $\boldsymbol{B} \parallel [001]$
Pc (7.24) $\mathbb{Z}_1$	• $\boldsymbol{B} \parallel [100]$ and strain $\perp [100]$<br>• ($\boldsymbol{E} \parallel [010]$ or $\boldsymbol{E} \perp [100]$) and $\boldsymbol{B} \parallel [100]$
$Aba'2'$ (41.214) $\mathbb{Z}_1$	• $\boldsymbol{B} \parallel [100]$
$Pc1'$ (7.25) $\mathbb{Z}_1$	• strain $\perp [100]$<br>• ($\boldsymbol{E} \parallel [010]$ or $\boldsymbol{E} \perp [100]$)

Table. S 323: Subgroups of $Ab'a2'$ (41.213) that can be realized by applying $\boldsymbol{E}/\boldsymbol{B}$ fields and/or uniaxial strains.

Subgroup & SI group	Perturbation
Cc (9.37) $\mathbb{Z}_1$	• strain $\perp$ $[010]$<br>• ($\boldsymbol{E}$ $\parallel$ $[100]$ or $\boldsymbol{E}$ $\perp$ $[010]$)
$C2'$ (5.15) $\mathbb{Z}_1$	• strain $\perp$ $[001]$<br>• ($\boldsymbol{B}$ $\parallel$ $[100]$ or $\boldsymbol{B}$ $\perp$ $[001]$)
Pc' (7.26) $\mathbb{Z}_1$	• strain $\perp$ $[100]$<br>• ($\boldsymbol{B}$ $\parallel$ $[001]$ or $\boldsymbol{B}$ $\perp$ $[100]$)<br>• ($\boldsymbol{E}$ $\parallel$ $[010]$ or $\boldsymbol{E}$ $\perp$ $[100]$)

Table. S 324: Subgroups of $Aba'2'$ (41.214) that can be realized by applying $\boldsymbol{E}/\boldsymbol{B}$ fields and/or uniaxial strains.

Subgroup & SI group	Perturbation
Cc' (9.39) $\mathbb{Z}_1$	• strain $\perp$ $[010]$<br>• ($\boldsymbol{B}$ $\parallel$ $[001]$ or $\boldsymbol{B}$ $\perp$ $[010]$)<br>• ($\boldsymbol{E}$ $\parallel$ $[100]$ or $\boldsymbol{E}$ $\perp$ $[010]$)
$C2'$ (5.15) $\mathbb{Z}_1$	• strain $\perp$ $[001]$<br>• ($\boldsymbol{B}$ $\parallel$ $[010]$ or $\boldsymbol{B}$ $\perp$ $[001]$)
Pc (7.24) $\mathbb{Z}_1$	• strain $\perp$ $[100]$<br>• ($\boldsymbol{E}$ $\parallel$ $[010]$ or $\boldsymbol{E}$ $\perp$ $[100]$)

Table. S 325: Subgroups of $Ab'a'2$ (41.215) that can be realized by applying $\boldsymbol{E}/\boldsymbol{B}$ fields and/or uniaxial strains.

Subgroup & SI group	Perturbation
Cc' (9.39) $\mathbb{Z}_1$	• strain $\perp$ $[010]$<br>• ($\boldsymbol{B}$ $\parallel$ $[100]$ or $\boldsymbol{B}$ $\perp$ $[010]$)<br>• ($\boldsymbol{E}$ $\parallel$ $[100]$ or $\boldsymbol{E}$ $\perp$ $[010]$)
$C2$ (5.13) $\mathbb{Z}_1$	• strain $\perp$ $[001]$

Continued on next page

...continued

Subgroup & SI group	Perturbation
Pc' (7.26) $\mathbb{Z}_1$	• strain $\perp$ [100]<br>• ($\boldsymbol{B}$ $\parallel$ [010] or $\boldsymbol{B}$ $\perp$ [100])<br>• ($\boldsymbol{E}$ $\parallel$ [010] or $\boldsymbol{E}$ $\perp$ [100])

Table. S 326: Subgroups of A_aba2 (41.216) that can be realized by applying $\boldsymbol{E}/\boldsymbol{B}$ fields and/or uniaxial strains.

Subgroup & SI group	Perturbation
Cm' (8.34) $\mathbb{Z}_1$	• $\boldsymbol{B}$ $\parallel$ [100] and strain $\perp$ [010]<br>• $\boldsymbol{B}$ $\parallel$ [001] and strain $\perp$ [010]<br>• $\boldsymbol{B}$ $\perp$ [010]<br>• ($\boldsymbol{E}$ $\parallel$ [100] or $\boldsymbol{E}$ $\perp$ [010]) and $\boldsymbol{B}$ $\parallel$ [100]<br>• ($\boldsymbol{E}$ $\parallel$ [100] or $\boldsymbol{E}$ $\perp$ [010]) and $\boldsymbol{B}$ $\parallel$ [001]
P_S1 (1.3) $\mathbb{Z}_1$	• strain in generic direction<br>• ($\boldsymbol{E}$ $\parallel$ [100] or $\boldsymbol{E}$ $\perp$ [010]) and strain $\perp$ [100]<br>• ($\boldsymbol{E}$ $\parallel$ [100] or $\boldsymbol{E}$ $\perp$ [010]) and strain $\perp$ [001]<br>• ($\boldsymbol{E}$ $\parallel$ [010] or $\boldsymbol{E}$ $\perp$ [100]) and strain $\perp$ [010]<br>• ($\boldsymbol{E}$ $\parallel$ [010] or $\boldsymbol{E}$ $\perp$ [100]) and strain $\perp$ [001]<br>• $\boldsymbol{E}$ in generic direction
Cc (9.37) $\mathbb{Z}_1$	• $\boldsymbol{B}$ $\parallel$ [010] and strain $\perp$ [010]<br>• ($\boldsymbol{E}$ $\parallel$ [100] or $\boldsymbol{E}$ $\perp$ [010]) and $\boldsymbol{B}$ $\parallel$ [010]
C_cc (9.40) $\mathbb{Z}_1$	• strain $\perp$ [010]<br>• ($\boldsymbol{E}$ $\parallel$ [100] or $\boldsymbol{E}$ $\perp$ [010])
Pc' (7.26) $\mathbb{Z}_1$	• $\boldsymbol{B}$ $\parallel$ [010] and strain $\perp$ [100]<br>• $\boldsymbol{B}$ $\parallel$ [001] and strain $\perp$ [100]<br>• $\boldsymbol{B}$ $\perp$ [100]<br>• ($\boldsymbol{E}$ $\parallel$ [010] or $\boldsymbol{E}$ $\perp$ [100]) and $\boldsymbol{B}$ $\parallel$ [010]<br>• ($\boldsymbol{E}$ $\parallel$ [010] or $\boldsymbol{E}$ $\perp$ [100]) and $\boldsymbol{B}$ $\parallel$ [001]
$C2$ (5.13) $\mathbb{Z}_1$	• $\boldsymbol{B}$ $\parallel$ [001] and strain $\perp$ [001]
$Ab'm'2$ (39.199) $\mathbb{Z}_2$	• $\boldsymbol{B}$ $\parallel$ [001]
Pc (7.24) $\mathbb{Z}_1$	• $\boldsymbol{B}$ $\parallel$ [100] and strain $\perp$ [100]<br>• ($\boldsymbol{E}$ $\parallel$ [010] or $\boldsymbol{E}$ $\perp$ [100]) and $\boldsymbol{B}$ $\parallel$ [100]
P_bc (7.29) $\mathbb{Z}_1$	• strain $\perp$ [100]<br>• ($\boldsymbol{E}$ $\parallel$ [010] or $\boldsymbol{E}$ $\perp$ [100])

Continued on next page

. . . continued

Subgroup & SI group	Perturbation
$C2'$ (5.15) $\mathbb{Z}_1$	• $\boldsymbol{B} \parallel [100]$ and strain $\perp [001]$<br>• $\boldsymbol{B} \parallel [010]$ and strain $\perp [001]$<br>• $\boldsymbol{B} \perp [001]$
$Ab'a2'$ (41.213) $\mathbb{Z}_1$	• $\boldsymbol{B} \parallel [010]$
C_c2 (5.16) $\mathbb{Z}_1$	• strain $\perp [001]$
$Abm'2'$ (39.198) $\mathbb{Z}_1$	• $\boldsymbol{B} \parallel [100]$

Table. S 327: Subgroups of A_bba2 (41.217) that can be realized by applying $\boldsymbol{E}/\boldsymbol{B}$ fields and/or uniaxial strains.

Subgroup & SI group	Perturbation
P_S1 (1.3) $\mathbb{Z}_1$	• strain in generic direction<br>• ($\boldsymbol{E} \parallel [100]$ or $\boldsymbol{E} \perp [010]$) and strain $\perp [100]$<br>• ($\boldsymbol{E} \parallel [100]$ or $\boldsymbol{E} \perp [010]$) and strain $\perp [001]$<br>• ($\boldsymbol{E} \parallel [010]$ or $\boldsymbol{E} \perp [100]$) and strain $\perp [010]$<br>• ($\boldsymbol{E} \parallel [010]$ or $\boldsymbol{E} \perp [100]$) and strain $\perp [001]$<br>• $\boldsymbol{E}$ in generic direction
Cc (9.37) $\mathbb{Z}_1$	• $\boldsymbol{B} \parallel [010]$ and strain $\perp [010]$<br>• ($\boldsymbol{E} \parallel [100]$ or $\boldsymbol{E} \perp [010]$) and $\boldsymbol{B} \parallel [010]$
Cc' (9.39) $\mathbb{Z}_1$	• $\boldsymbol{B} \parallel [100]$ and strain $\perp [010]$<br>• $\boldsymbol{B} \parallel [001]$ and strain $\perp [010]$<br>• $\boldsymbol{B} \perp [010]$<br>• ($\boldsymbol{E} \parallel [100]$ or $\boldsymbol{E} \perp [010]$) and $\boldsymbol{B} \parallel [100]$<br>• ($\boldsymbol{E} \parallel [100]$ or $\boldsymbol{E} \perp [010]$) and $\boldsymbol{B} \parallel [001]$
C_ac (9.41) $\mathbb{Z}_2$	• strain $\perp [010]$<br>• ($\boldsymbol{E} \parallel [100]$ or $\boldsymbol{E} \perp [010]$)
$C2'$ (5.15) $\mathbb{Z}_1$	• $\boldsymbol{B} \parallel [100]$ and strain $\perp [001]$<br>• $\boldsymbol{B} \parallel [010]$ and strain $\perp [001]$<br>• $\boldsymbol{B} \perp [001]$
$C2$ (5.13) $\mathbb{Z}_1$	• $\boldsymbol{B} \parallel [001]$ and strain $\perp [001]$
C_a2 (5.17) $\mathbb{Z}_2$	• strain $\perp [001]$
Pc (7.24) $\mathbb{Z}_1$	• $\boldsymbol{B} \parallel [100]$ and strain $\perp [100]$<br>• ($\boldsymbol{E} \parallel [010]$ or $\boldsymbol{E} \perp [100]$) and $\boldsymbol{B} \parallel [100]$

Continued on next page

...continued

Subgroup & SI group	Perturbation
$Aba'2'$ (41.214) $\mathbb{Z}_1$	• $\boldsymbol{B}$ ∥ [100]
Pm' (6.20) $\mathbb{Z}_1$	• $\boldsymbol{B}$ ∥ [010] and strain ⊥ [100]<br>• $\boldsymbol{B}$ ∥ [001] and strain ⊥ [100]<br>• $\boldsymbol{B}$ ⊥ [100]<br>• ($\boldsymbol{E}$ ∥ [010] or $\boldsymbol{E}$ ⊥ [100]) and $\boldsymbol{B}$ ∥ [010]<br>• ($\boldsymbol{E}$ ∥ [010] or $\boldsymbol{E}$ ⊥ [100]) and $\boldsymbol{B}$ ∥ [001]
$Am'a2'$ (40.205) $\mathbb{Z}_1$	• $\boldsymbol{B}$ ∥ [010]
$Am'a'2$ (40.207) $\mathbb{Z}_1$	• $\boldsymbol{B}$ ∥ [001]
P_cc (7.28) $\mathbb{Z}_1$	• strain ⊥ [100]<br>• ($\boldsymbol{E}$ ∥ [010] or $\boldsymbol{E}$ ⊥ [100])

Table. S 328: Subgroups of A_Bba2 (41.218) that can be realized by applying $\boldsymbol{E}/\boldsymbol{B}$ fields and/or uniaxial strains.

Subgroup & SI group	Perturbation
Cm' (8.34) $\mathbb{Z}_1$	• $\boldsymbol{B}$ ∥ [100] and strain ⊥ [010]<br>• $\boldsymbol{B}$ ∥ [001] and strain ⊥ [010]<br>• $\boldsymbol{B}$ ⊥ [010]<br>• ($\boldsymbol{E}$ ∥ [100] or $\boldsymbol{E}$ ⊥ [010]) and $\boldsymbol{B}$ ∥ [100]<br>• ($\boldsymbol{E}$ ∥ [100] or $\boldsymbol{E}$ ⊥ [010]) and $\boldsymbol{B}$ ∥ [001]
P_S1 (1.3) $\mathbb{Z}_1$	• strain in generic direction<br>• ($\boldsymbol{E}$ ∥ [100] or $\boldsymbol{E}$ ⊥ [010]) and strain ⊥ [100]<br>• ($\boldsymbol{E}$ ∥ [100] or $\boldsymbol{E}$ ⊥ [010]) and strain ⊥ [001]<br>• ($\boldsymbol{E}$ ∥ [010] or $\boldsymbol{E}$ ⊥ [100]) and strain ⊥ [010]<br>• ($\boldsymbol{E}$ ∥ [010] or $\boldsymbol{E}$ ⊥ [100]) and strain ⊥ [001]<br>• $\boldsymbol{E}$ in generic direction
Cc (9.37) $\mathbb{Z}_1$	• $\boldsymbol{B}$ ∥ [010] and strain ⊥ [010]<br>• ($\boldsymbol{E}$ ∥ [100] or $\boldsymbol{E}$ ⊥ [010]) and $\boldsymbol{B}$ ∥ [010]
C_cc (9.40) $\mathbb{Z}_1$	• strain ⊥ [010]<br>• ($\boldsymbol{E}$ ∥ [100] or $\boldsymbol{E}$ ⊥ [010])

Continued on next page

...continued

Subgroup & SI group	Perturbation
Pm' (6.20) $\mathbb{Z}_1$	• $\boldsymbol{B} \parallel [010]$ and strain $\perp [100]$<br>• $\boldsymbol{B} \parallel [001]$ and strain $\perp [100]$<br>• $\boldsymbol{B} \perp [100]$<br>• ($\boldsymbol{E} \parallel [010]$ or $\boldsymbol{E} \perp [100]$) and $\boldsymbol{B} \parallel [010]$<br>• ($\boldsymbol{E} \parallel [010]$ or $\boldsymbol{E} \perp [100]$) and $\boldsymbol{B} \parallel [001]$
$C2$ (5.13) $\mathbb{Z}_1$	• $\boldsymbol{B} \parallel [001]$ and strain $\perp [001]$
$Am'm'2$ (38.191) $\mathbb{Z}_1$	• $\boldsymbol{B} \parallel [001]$
Pc (7.24) $\mathbb{Z}_1$	• $\boldsymbol{B} \parallel [100]$ and strain $\perp [100]$<br>• ($\boldsymbol{E} \parallel [010]$ or $\boldsymbol{E} \perp [100]$) and $\boldsymbol{B} \parallel [100]$
P_Ac (7.31) $\mathbb{Z}_1$	• strain $\perp [100]$<br>• ($\boldsymbol{E} \parallel [010]$ or $\boldsymbol{E} \perp [100]$)
$C2'$ (5.15) $\mathbb{Z}_1$	• $\boldsymbol{B} \parallel [100]$ and strain $\perp [001]$<br>• $\boldsymbol{B} \parallel [010]$ and strain $\perp [001]$<br>• $\boldsymbol{B} \perp [001]$
$Am'a2'$ (40.205) $\mathbb{Z}_1$	• $\boldsymbol{B} \parallel [010]$
C_c2 (5.16) $\mathbb{Z}_1$	• strain $\perp [001]$
$Abm'2'$ (39.198) $\mathbb{Z}_1$	• $\boldsymbol{B} \parallel [100]$

Table. S 329: Subgroups of $Fmm2$ (42.219) that can be realized by applying $\boldsymbol{E}/\boldsymbol{B}$ fields and/or uniaxial strains.

Subgroup & SI group	Perturbation
Cm (8.32) $\mathbb{Z}_1$	• strain $\perp [010]$<br>• $\boldsymbol{B} \parallel [010]$<br>• ($\boldsymbol{E} \parallel [100]$ or $\boldsymbol{E} \perp [010]$)
Cm (8.32) $\mathbb{Z}_1$	• strain $\perp [100]$<br>• $\boldsymbol{B} \parallel [100]$<br>• ($\boldsymbol{E} \parallel [010]$ or $\boldsymbol{E} \perp [100]$)
$C2$ (5.13) $\mathbb{Z}_1$	• strain $\perp [001]$<br>• $\boldsymbol{B} \parallel [001]$

Table. S 330: Subgroups of $Fmm21'$ (42.220) that can be realized by applying $\boldsymbol{E}/\boldsymbol{B}$ fields and/or uniaxial strains.

Subgroup & SI group	Perturbation
$P11'$ (1.2) $\mathbb{Z}_1$	• strain in generic direction<br>• ($\boldsymbol{E}$ ∥ [100] or $\boldsymbol{E}$ ⊥ [010]) and strain ⊥ [100]<br>• ($\boldsymbol{E}$ ∥ [100] or $\boldsymbol{E}$ ⊥ [010]) and strain ⊥ [001]<br>• ($\boldsymbol{E}$ ∥ [010] or $\boldsymbol{E}$ ⊥ [100]) and strain ⊥ [010]<br>• ($\boldsymbol{E}$ ∥ [010] or $\boldsymbol{E}$ ⊥ [100]) and strain ⊥ [001]<br>• $\boldsymbol{E}$ in generic direction
Cm' (8.34) $\mathbb{Z}_1$	• $\boldsymbol{B}$ ∥ [100] and strain ⊥ [010]<br>• $\boldsymbol{B}$ ∥ [001] and strain ⊥ [010]<br>• $\boldsymbol{B}$ ⊥ [010]<br>• ($\boldsymbol{E}$ ∥ [100] or $\boldsymbol{E}$ ⊥ [010]) and $\boldsymbol{B}$ ∥ [100]<br>• ($\boldsymbol{E}$ ∥ [100] or $\boldsymbol{E}$ ⊥ [010]) and $\boldsymbol{B}$ ∥ [001]
Cm (8.32) $\mathbb{Z}_1$	• $\boldsymbol{B}$ ∥ [010] and strain ⊥ [010]<br>• ($\boldsymbol{E}$ ∥ [100] or $\boldsymbol{E}$ ⊥ [010]) and $\boldsymbol{B}$ ∥ [010]
$Cm1'$ (8.33) $\mathbb{Z}_1$	• strain ⊥ [010]<br>• ($\boldsymbol{E}$ ∥ [100] or $\boldsymbol{E}$ ⊥ [010])
Cm' (8.34) $\mathbb{Z}_1$	• $\boldsymbol{B}$ ∥ [010] and strain ⊥ [100]<br>• $\boldsymbol{B}$ ∥ [001] and strain ⊥ [100]<br>• $\boldsymbol{B}$ ⊥ [100]<br>• ($\boldsymbol{E}$ ∥ [010] or $\boldsymbol{E}$ ⊥ [100]) and $\boldsymbol{B}$ ∥ [010]<br>• ($\boldsymbol{E}$ ∥ [010] or $\boldsymbol{E}$ ⊥ [100]) and $\boldsymbol{B}$ ∥ [001]
Cm (8.32) $\mathbb{Z}_1$	• $\boldsymbol{B}$ ∥ [100] and strain ⊥ [100]<br>• ($\boldsymbol{E}$ ∥ [010] or $\boldsymbol{E}$ ⊥ [100]) and $\boldsymbol{B}$ ∥ [100]
$Cm1'$ (8.33) $\mathbb{Z}_1$	• strain ⊥ [100]<br>• ($\boldsymbol{E}$ ∥ [010] or $\boldsymbol{E}$ ⊥ [100])
$C2'$ (5.15) $\mathbb{Z}_1$	• $\boldsymbol{B}$ ∥ [100] and strain ⊥ [001]<br>• $\boldsymbol{B}$ ∥ [010] and strain ⊥ [001]<br>• $\boldsymbol{B}$ ⊥ [001]
$Fm'm2'$ (42.221) $\mathbb{Z}_1$	• $\boldsymbol{B}$ ∥ [010]
$Fm'm2'$ (42.221) $\mathbb{Z}_1$	• $\boldsymbol{B}$ ∥ [100]
$C2$ (5.13) $\mathbb{Z}_1$	• $\boldsymbol{B}$ ∥ [001] and strain ⊥ [001]
$Fm'm'2$ (42.222) $\mathbb{Z}_2$	• $\boldsymbol{B}$ ∥ [001]
$C21'$ (5.14) $\mathbb{Z}_1$	• strain ⊥ [001]

Table. S 331: Subgroups of $Fm'm2'$ (42.221) that can be realized by applying $\boldsymbol{E}/\boldsymbol{B}$ fields and/or uniaxial strains.

Subgroup & SI group	Perturbation
Cm (8.32) $\mathbb{Z}_1$	• strain $\perp$ [010]<br>• ($\boldsymbol{E} \parallel$ [100] or $\boldsymbol{E} \perp$ [010])
Cm' (8.34) $\mathbb{Z}_1$	• strain $\perp$ [100]<br>• ($\boldsymbol{B} \parallel$ [001] or $\boldsymbol{B} \perp$ [100])<br>• ($\boldsymbol{E} \parallel$ [010] or $\boldsymbol{E} \perp$ [100])
$C2'$ (5.15) $\mathbb{Z}_1$	• strain $\perp$ [001]<br>• ($\boldsymbol{B} \parallel$ [100] or $\boldsymbol{B} \perp$ [001])

Table. S 332: Subgroups of $Fm'm'2$ (42.222) that can be realized by applying $\boldsymbol{E}/\boldsymbol{B}$ fields and/or uniaxial strains.

Subgroup & SI group	Perturbation
Cm' (8.34) $\mathbb{Z}_1$	• strain $\perp$ [010]<br>• ($\boldsymbol{B} \parallel$ [100] or $\boldsymbol{B} \perp$ [010])<br>• ($\boldsymbol{E} \parallel$ [100] or $\boldsymbol{E} \perp$ [010])
Cm' (8.34) $\mathbb{Z}_1$	• strain $\perp$ [100]<br>• ($\boldsymbol{B} \parallel$ [010] or $\boldsymbol{B} \perp$ [100])<br>• ($\boldsymbol{E} \parallel$ [010] or $\boldsymbol{E} \perp$ [100])
$C2$ (5.13) $\mathbb{Z}_1$	• strain $\perp$ [001]

Table. S 333: Subgroups of F_Smm2 (42.223) that can be realized by applying $\boldsymbol{E}/\boldsymbol{B}$ fields and/or uniaxial strains.

Subgroup & SI group	Perturbation
P_S1 (1.3) $\mathbb{Z}_1$	• strain in generic direction<br>• ($\boldsymbol{E} \parallel$ [100] or $\boldsymbol{E} \perp$ [010]) and strain $\perp$ [100]<br>• ($\boldsymbol{E} \parallel$ [100] or $\boldsymbol{E} \perp$ [010]) and strain $\perp$ [001]<br>• ($\boldsymbol{E} \parallel$ [010] or $\boldsymbol{E} \perp$ [100]) and strain $\perp$ [010]<br>• ($\boldsymbol{E} \parallel$ [010] or $\boldsymbol{E} \perp$ [100]) and strain $\perp$ [001]<br>• $\boldsymbol{E}$ in generic direction

Continued on next page

. . . continued

Subgroup & SI group	Perturbation
Cm (8.32) $\mathbb{Z}_1$	• $\boldsymbol{B}$ ∥ [010] and strain ⊥ [010]<br>• ($\boldsymbol{E}$ ∥ [100] or $\boldsymbol{E}$ ⊥ [010]) and $\boldsymbol{B}$ ∥ [010]
Cm' (8.34) $\mathbb{Z}_1$	• $\boldsymbol{B}$ ∥ [100] and strain ⊥ [010]<br>• $\boldsymbol{B}$ ∥ [001] and strain ⊥ [010]<br>• $\boldsymbol{B}$ ⊥ [010]<br>• ($\boldsymbol{E}$ ∥ [100] or $\boldsymbol{E}$ ⊥ [010]) and $\boldsymbol{B}$ ∥ [100]<br>• ($\boldsymbol{E}$ ∥ [100] or $\boldsymbol{E}$ ⊥ [010]) and $\boldsymbol{B}$ ∥ [001]
C_am (8.36) $\mathbb{Z}_1$	• strain ⊥ [010]<br>• ($\boldsymbol{E}$ ∥ [100] or $\boldsymbol{E}$ ⊥ [010])
Cm (8.32) $\mathbb{Z}_1$	• $\boldsymbol{B}$ ∥ [100] and strain ⊥ [100]<br>• ($\boldsymbol{E}$ ∥ [010] or $\boldsymbol{E}$ ⊥ [100]) and $\boldsymbol{B}$ ∥ [100]
Cm' (8.34) $\mathbb{Z}_1$	• $\boldsymbol{B}$ ∥ [010] and strain ⊥ [100]<br>• $\boldsymbol{B}$ ∥ [001] and strain ⊥ [100]<br>• $\boldsymbol{B}$ ⊥ [100]<br>• ($\boldsymbol{E}$ ∥ [010] or $\boldsymbol{E}$ ⊥ [100]) and $\boldsymbol{B}$ ∥ [010]<br>• ($\boldsymbol{E}$ ∥ [010] or $\boldsymbol{E}$ ⊥ [100]) and $\boldsymbol{B}$ ∥ [001]
C_am (8.36) $\mathbb{Z}_1$	• strain ⊥ [100]<br>• ($\boldsymbol{E}$ ∥ [010] or $\boldsymbol{E}$ ⊥ [100])
$C2$ (5.13) $\mathbb{Z}_1$	• $\boldsymbol{B}$ ∥ [001] and strain ⊥ [001]
$Fm'm'2$ (42.222) $\mathbb{Z}_2$	• $\boldsymbol{B}$ ∥ [001]
$C2'$ (5.15) $\mathbb{Z}_1$	• $\boldsymbol{B}$ ∥ [100] and strain ⊥ [001]<br>• $\boldsymbol{B}$ ∥ [010] and strain ⊥ [001]<br>• $\boldsymbol{B}$ ⊥ [001]
$Fm'm2'$ (42.221) $\mathbb{Z}_1$	• $\boldsymbol{B}$ ∥ [100]
$Fm'm2'$ (42.221) $\mathbb{Z}_1$	• $\boldsymbol{B}$ ∥ [010]
C_a2 (5.17) $\mathbb{Z}_2$	• strain ⊥ [001]

Table. S 334: Subgroups of $Fdd2$ (43.224) that can be realized by applying $\boldsymbol{E}/\boldsymbol{B}$ fields and/or uniaxial strains.

Subgroup & SI group	Perturbation

Continued on next page

...continued

Subgroup & SI group	Perturbation
Cc (9.37) $\mathbb{Z}_1$	• strain $\perp$ [010]<br>• $\boldsymbol{B}$ $\parallel$ [010]<br>• ($\boldsymbol{E}$ $\parallel$ [100] or $\boldsymbol{E}$ $\perp$ [010])
Cc (9.37) $\mathbb{Z}_1$	• strain $\perp$ [100]<br>• $\boldsymbol{B}$ $\parallel$ [100]<br>• ($\boldsymbol{E}$ $\parallel$ [010] or $\boldsymbol{E}$ $\perp$ [100])
$C2$ (5.13) $\mathbb{Z}_1$	• strain $\perp$ [001]<br>• $\boldsymbol{B}$ $\parallel$ [001]

Table. S 335: Subgroups of $Fdd21'$ (43.225) that can be realized by applying $\boldsymbol{E}/\boldsymbol{B}$ fields and/or uniaxial strains.

Subgroup & SI group	Perturbation
Cc' (9.39) $\mathbb{Z}_1$	• $\boldsymbol{B}$ $\parallel$ [100] and strain $\perp$ [010]<br>• $\boldsymbol{B}$ $\parallel$ [001] and strain $\perp$ [010]<br>• $\boldsymbol{B}$ $\perp$ [010]<br>• ($\boldsymbol{E}$ $\parallel$ [100] or $\boldsymbol{E}$ $\perp$ [010]) and $\boldsymbol{B}$ $\parallel$ [100]<br>• ($\boldsymbol{E}$ $\parallel$ [100] or $\boldsymbol{E}$ $\perp$ [010]) and $\boldsymbol{B}$ $\parallel$ [001]
Cc (9.37) $\mathbb{Z}_1$	• $\boldsymbol{B}$ $\parallel$ [010] and strain $\perp$ [010]<br>• ($\boldsymbol{E}$ $\parallel$ [100] or $\boldsymbol{E}$ $\perp$ [010]) and $\boldsymbol{B}$ $\parallel$ [010]
$P11'$ (1.2) $\mathbb{Z}_1$	• strain in generic direction<br>• ($\boldsymbol{E}$ $\parallel$ [100] or $\boldsymbol{E}$ $\perp$ [010]) and strain $\perp$ [100]<br>• ($\boldsymbol{E}$ $\parallel$ [100] or $\boldsymbol{E}$ $\perp$ [010]) and strain $\perp$ [001]<br>• ($\boldsymbol{E}$ $\parallel$ [010] or $\boldsymbol{E}$ $\perp$ [100]) and strain $\perp$ [010]<br>• ($\boldsymbol{E}$ $\parallel$ [010] or $\boldsymbol{E}$ $\perp$ [100]) and strain $\perp$ [001]<br>• $\boldsymbol{E}$ in generic direction
$Cc1'$ (9.38) $\mathbb{Z}_1$	• strain $\perp$ [010]<br>• ($\boldsymbol{E}$ $\parallel$ [100] or $\boldsymbol{E}$ $\perp$ [010])
Cc' (9.39) $\mathbb{Z}_1$	• $\boldsymbol{B}$ $\parallel$ [010] and strain $\perp$ [100]<br>• $\boldsymbol{B}$ $\parallel$ [001] and strain $\perp$ [100]<br>• $\boldsymbol{B}$ $\perp$ [100]<br>• ($\boldsymbol{E}$ $\parallel$ [010] or $\boldsymbol{E}$ $\perp$ [100]) and $\boldsymbol{B}$ $\parallel$ [010]<br>• ($\boldsymbol{E}$ $\parallel$ [010] or $\boldsymbol{E}$ $\perp$ [100]) and $\boldsymbol{B}$ $\parallel$ [001]
Cc (9.37) $\mathbb{Z}_1$	• $\boldsymbol{B}$ $\parallel$ [100] and strain $\perp$ [100]<br>• ($\boldsymbol{E}$ $\parallel$ [010] or $\boldsymbol{E}$ $\perp$ [100]) and $\boldsymbol{B}$ $\parallel$ [100]

Continued on next page

…continued

Subgroup & SI group	Perturbation
$Cc1'$ (9.38) $\mathbb{Z}_1$	• strain $\perp$ $[100]$<br>• ($\boldsymbol{E}$ $\parallel$ $[010]$ or $\boldsymbol{E}$ $\perp$ $[100]$)
$C2'$ (5.15) $\mathbb{Z}_1$	• $\boldsymbol{B}$ $\parallel$ $[100]$ and strain $\perp$ $[001]$<br>• $\boldsymbol{B}$ $\parallel$ $[010]$ and strain $\perp$ $[001]$<br>• $\boldsymbol{B}$ $\perp$ $[001]$
$Fd'd2'$ (43.226) $\mathbb{Z}_1$	• $\boldsymbol{B}$ $\parallel$ $[010]$
$Fd'd2'$ (43.226) $\mathbb{Z}_1$	• $\boldsymbol{B}$ $\parallel$ $[100]$
$C2$ (5.13) $\mathbb{Z}_1$	• $\boldsymbol{B}$ $\parallel$ $[001]$ and strain $\perp$ $[001]$
$Fd'd'2$ (43.227) $\mathbb{Z}_1$	• $\boldsymbol{B}$ $\parallel$ $[001]$
$C21'$ (5.14) $\mathbb{Z}_1$	• strain $\perp$ $[001]$

Table. S 336: Subgroups of $Fd'd2'$ (43.226) that can be realized by applying $\boldsymbol{E}/\boldsymbol{B}$ fields and/or uniaxial strains.

Subgroup & SI group	Perturbation
Cc (9.37) $\mathbb{Z}_1$	• strain $\perp$ $[010]$<br>• ($\boldsymbol{E}$ $\parallel$ $[100]$ or $\boldsymbol{E}$ $\perp$ $[010]$)
Cc' (9.39) $\mathbb{Z}_1$	• strain $\perp$ $[100]$<br>• ($\boldsymbol{B}$ $\parallel$ $[001]$ or $\boldsymbol{B}$ $\perp$ $[100]$)<br>• ($\boldsymbol{E}$ $\parallel$ $[010]$ or $\boldsymbol{E}$ $\perp$ $[100]$)
$C2'$ (5.15) $\mathbb{Z}_1$	• strain $\perp$ $[001]$<br>• ($\boldsymbol{B}$ $\parallel$ $[100]$ or $\boldsymbol{B}$ $\perp$ $[001]$)

Table. S 337: Subgroups of $Fd'd'2$ (43.227) that can be realized by applying $\boldsymbol{E}/\boldsymbol{B}$ fields and/or uniaxial strains.

Subgroup & SI group	Perturbation
Cc' (9.39) $\mathbb{Z}_1$	• strain $\perp$ $[010]$<br>• ($\boldsymbol{B}$ $\parallel$ $[100]$ or $\boldsymbol{B}$ $\perp$ $[010]$)<br>• ($\boldsymbol{E}$ $\parallel$ $[100]$ or $\boldsymbol{E}$ $\perp$ $[010]$)

Continued on next page

...continued

Subgroup & SI group	Perturbation
Cc' (9.39) $\mathbb{Z}_1$	• strain $\perp$ [100]<br>• ($\boldsymbol{B} \parallel$ [010] or $\boldsymbol{B} \perp$ [100])<br>• ($\boldsymbol{E} \parallel$ [010] or $\boldsymbol{E} \perp$ [100])
$C2$ (5.13) $\mathbb{Z}_1$	• strain $\perp$ [001]

Table. S 338: Subgroups of F_Sdd2 (43.228) that can be realized by applying $\boldsymbol{E}/\boldsymbol{B}$ fields and/or uniaxial strains.

Subgroup & SI group	Perturbation
Cc' (9.39) $\mathbb{Z}_1$	• $\boldsymbol{B} \parallel$ [100] and strain $\perp$ [010]<br>• $\boldsymbol{B} \parallel$ [001] and strain $\perp$ [010]<br>• $\boldsymbol{B} \perp$ [010]<br>• ($\boldsymbol{E} \parallel$ [100] or $\boldsymbol{E} \perp$ [010]) and $\boldsymbol{B} \parallel$ [100]<br>• ($\boldsymbol{E} \parallel$ [100] or $\boldsymbol{E} \perp$ [010]) and $\boldsymbol{B} \parallel$ [001]
Cc (9.37) $\mathbb{Z}_1$	• $\boldsymbol{B} \parallel$ [010] and strain $\perp$ [010]<br>• ($\boldsymbol{E} \parallel$ [100] or $\boldsymbol{E} \perp$ [010]) and $\boldsymbol{B} \parallel$ [010]
P_S1 (1.3) $\mathbb{Z}_1$	• strain in generic direction<br>• ($\boldsymbol{E} \parallel$ [100] or $\boldsymbol{E} \perp$ [010]) and strain $\perp$ [100]<br>• ($\boldsymbol{E} \parallel$ [100] or $\boldsymbol{E} \perp$ [010]) and strain $\perp$ [001]<br>• ($\boldsymbol{E} \parallel$ [010] or $\boldsymbol{E} \perp$ [100]) and strain $\perp$ [010]<br>• ($\boldsymbol{E} \parallel$ [010] or $\boldsymbol{E} \perp$ [100]) and strain $\perp$ [001]<br>• $\boldsymbol{E}$ in generic direction
C_ac (9.41) $\mathbb{Z}_2$	• strain $\perp$ [010]<br>• ($\boldsymbol{E} \parallel$ [100] or $\boldsymbol{E} \perp$ [010])
Cc' (9.39) $\mathbb{Z}_1$	• $\boldsymbol{B} \parallel$ [010] and strain $\perp$ [100]<br>• $\boldsymbol{B} \parallel$ [001] and strain $\perp$ [100]<br>• $\boldsymbol{B} \perp$ [100]<br>• ($\boldsymbol{E} \parallel$ [010] or $\boldsymbol{E} \perp$ [100]) and $\boldsymbol{B} \parallel$ [010]<br>• ($\boldsymbol{E} \parallel$ [010] or $\boldsymbol{E} \perp$ [100]) and $\boldsymbol{B} \parallel$ [001]
Cc (9.37) $\mathbb{Z}_1$	• $\boldsymbol{B} \parallel$ [100] and strain $\perp$ [100]<br>• ($\boldsymbol{E} \parallel$ [010] or $\boldsymbol{E} \perp$ [100]) and $\boldsymbol{B} \parallel$ [100]
C_ac (9.41) $\mathbb{Z}_2$	• strain $\perp$ [100]<br>• ($\boldsymbol{E} \parallel$ [010] or $\boldsymbol{E} \perp$ [100])
$C2$ (5.13) $\mathbb{Z}_1$	• $\boldsymbol{B} \parallel$ [001] and strain $\perp$ [001]

Continued on next page

. . . continued

Subgroup & SI group	Perturbation
$Fd'd'2$ (43.227) $\mathbb{Z}_1$	• $\boldsymbol{B}$ ∥ [001]
$C2'$ (5.15) $\mathbb{Z}_1$	• $\boldsymbol{B}$ ∥ [100] and strain ⊥ [001]<br>• $\boldsymbol{B}$ ∥ [010] and strain ⊥ [001]<br>• $\boldsymbol{B}$ ⊥ [001]
$Fd'd2'$ (43.226) $\mathbb{Z}_1$	• $\boldsymbol{B}$ ∥ [010]
$Fd'd2'$ (43.226) $\mathbb{Z}_1$	• $\boldsymbol{B}$ ∥ [100]
C_a2 (5.17) $\mathbb{Z}_2$	• strain ⊥ [001]

Table. S 339: Subgroups of $Imm2$ (44.229) that can be realized by applying $\boldsymbol{E}/\boldsymbol{B}$ fields and/or uniaxial strains.

Subgroup & SI group	Perturbation
Cm (8.32) $\mathbb{Z}_1$	• strain ⊥ [010]<br>• $\boldsymbol{B}$ ∥ [010]<br>• ($\boldsymbol{E}$ ∥ [100] or $\boldsymbol{E}$ ⊥ [010])
Cm (8.32) $\mathbb{Z}_1$	• strain ⊥ [100]<br>• $\boldsymbol{B}$ ∥ [100]<br>• ($\boldsymbol{E}$ ∥ [010] or $\boldsymbol{E}$ ⊥ [100])
$C2$ (5.13) $\mathbb{Z}_1$	• strain ⊥ [001]<br>• $\boldsymbol{B}$ ∥ [001]

Table. S 340: Subgroups of $Imm21'$ (44.230) that can be realized by applying $\boldsymbol{E}/\boldsymbol{B}$ fields and/or uniaxial strains.

Subgroup & SI group	Perturbation
$P11'$ (1.2) $\mathbb{Z}_1$	• strain in generic direction<br>• ($\boldsymbol{E}$ ∥ [100] or $\boldsymbol{E}$ ⊥ [010]) and strain ⊥ [100]<br>• ($\boldsymbol{E}$ ∥ [100] or $\boldsymbol{E}$ ⊥ [010]) and strain ⊥ [001]<br>• ($\boldsymbol{E}$ ∥ [010] or $\boldsymbol{E}$ ⊥ [100]) and strain ⊥ [010]<br>• ($\boldsymbol{E}$ ∥ [010] or $\boldsymbol{E}$ ⊥ [100]) and strain ⊥ [001]<br>• $\boldsymbol{E}$ in generic direction

Continued on next page

...continued

Subgroup & SI group	Perturbation
Cm' (8.34) $\mathbb{Z}_1$	• $\boldsymbol{B} \parallel [100]$ and strain $\perp [010]$<br>• $\boldsymbol{B} \parallel [001]$ and strain $\perp [010]$<br>• $\boldsymbol{B} \perp [010]$<br>• ($\boldsymbol{E} \parallel [100]$ or $\boldsymbol{E} \perp [010]$) and $\boldsymbol{B} \parallel [100]$<br>• ($\boldsymbol{E} \parallel [100]$ or $\boldsymbol{E} \perp [010]$) and $\boldsymbol{B} \parallel [001]$
Cm (8.32) $\mathbb{Z}_1$	• $\boldsymbol{B} \parallel [010]$ and strain $\perp [010]$<br>• ($\boldsymbol{E} \parallel [100]$ or $\boldsymbol{E} \perp [010]$) and $\boldsymbol{B} \parallel [010]$
$Cm1'$ (8.33) $\mathbb{Z}_1$	• strain $\perp [010]$<br>• ($\boldsymbol{E} \parallel [100]$ or $\boldsymbol{E} \perp [010]$)
Cm' (8.34) $\mathbb{Z}_1$	• $\boldsymbol{B} \parallel [010]$ and strain $\perp [100]$<br>• $\boldsymbol{B} \parallel [001]$ and strain $\perp [100]$<br>• $\boldsymbol{B} \perp [100]$<br>• ($\boldsymbol{E} \parallel [010]$ or $\boldsymbol{E} \perp [100]$) and $\boldsymbol{B} \parallel [010]$<br>• ($\boldsymbol{E} \parallel [010]$ or $\boldsymbol{E} \perp [100]$) and $\boldsymbol{B} \parallel [001]$
Cm (8.32) $\mathbb{Z}_1$	• $\boldsymbol{B} \parallel [100]$ and strain $\perp [100]$<br>• ($\boldsymbol{E} \parallel [010]$ or $\boldsymbol{E} \perp [100]$) and $\boldsymbol{B} \parallel [100]$
$Cm1'$ (8.33) $\mathbb{Z}_1$	• strain $\perp [100]$<br>• ($\boldsymbol{E} \parallel [010]$ or $\boldsymbol{E} \perp [100]$)
$C2'$ (5.15) $\mathbb{Z}_1$	• $\boldsymbol{B} \parallel [100]$ and strain $\perp [001]$<br>• $\boldsymbol{B} \parallel [010]$ and strain $\perp [001]$<br>• $\boldsymbol{B} \perp [001]$
$Im'm2'$ (44.231) $\mathbb{Z}_1$	• $\boldsymbol{B} \parallel [010]$
$Im'm2'$ (44.231) $\mathbb{Z}_1$	• $\boldsymbol{B} \parallel [100]$
$C2$ (5.13) $\mathbb{Z}_1$	• $\boldsymbol{B} \parallel [001]$ and strain $\perp [001]$
$Im'm'2$ (44.232) $\mathbb{Z}_1$	• $\boldsymbol{B} \parallel [001]$
$C21'$ (5.14) $\mathbb{Z}_1$	• strain $\perp [001]$

Table. S 341: Subgroups of $Im'm2'$ (44.231) that can be realized by applying $\boldsymbol{E}/\boldsymbol{B}$ fields and/or uniaxial strains.

Subgroup & SI group	Perturbation
Cm (8.32) $\mathbb{Z}_1$	• strain $\perp [010]$<br>• ($\boldsymbol{E} \parallel [100]$ or $\boldsymbol{E} \perp [010]$)

Continued on next page

...continued

Subgroup & SI group	Perturbation
Cm' (8.34) $\mathbb{Z}_1$	• strain $\perp$ [100]<br>• ($\boldsymbol{B}$ ∥ [001] or $\boldsymbol{B} \perp$ [100])<br>• ($\boldsymbol{E}$ ∥ [010] or $\boldsymbol{E} \perp$ [100])
$C2'$ (5.15) $\mathbb{Z}_1$	• strain $\perp$ [001]<br>• ($\boldsymbol{B}$ ∥ [100] or $\boldsymbol{B} \perp$ [001])

Table. S 342: Subgroups of $Im'm'2$ (44.232) that can be realized by applying $\boldsymbol{E}/\boldsymbol{B}$ fields and/or uniaxial strains.

Subgroup & SI group	Perturbation
Cm' (8.34) $\mathbb{Z}_1$	• strain $\perp$ [010]<br>• ($\boldsymbol{B}$ ∥ [100] or $\boldsymbol{B} \perp$ [010])<br>• ($\boldsymbol{E}$ ∥ [100] or $\boldsymbol{E} \perp$ [010])
Cm' (8.34) $\mathbb{Z}_1$	• strain $\perp$ [100]<br>• ($\boldsymbol{B}$ ∥ [010] or $\boldsymbol{B} \perp$ [100])<br>• ($\boldsymbol{E}$ ∥ [010] or $\boldsymbol{E} \perp$ [100])
$C2$ (5.13) $\mathbb{Z}_1$	• strain $\perp$ [001]

Table. S 343: Subgroups of I_cmm2 (44.233) that can be realized by applying $\boldsymbol{E}/\boldsymbol{B}$ fields and/or uniaxial strains.

Subgroup & SI group	Perturbation
P_S1 (1.3) $\mathbb{Z}_1$	• strain in generic direction<br>• ($\boldsymbol{E}$ ∥ [100] or $\boldsymbol{E} \perp$ [010]) and strain $\perp$ [100]<br>• ($\boldsymbol{E}$ ∥ [100] or $\boldsymbol{E} \perp$ [010]) and strain $\perp$ [001]<br>• ($\boldsymbol{E}$ ∥ [010] or $\boldsymbol{E} \perp$ [100]) and strain $\perp$ [010]<br>• ($\boldsymbol{E}$ ∥ [010] or $\boldsymbol{E} \perp$ [100]) and strain $\perp$ [001]<br>• $\boldsymbol{E}$ in generic direction
Cc' (9.39) $\mathbb{Z}_1$	• $\boldsymbol{B}$ ∥ [100] and strain $\perp$ [010]<br>• $\boldsymbol{B}$ ∥ [001] and strain $\perp$ [010]<br>• $\boldsymbol{B} \perp$ [010]<br>• ($\boldsymbol{E}$ ∥ [100] or $\boldsymbol{E} \perp$ [010]) and $\boldsymbol{B}$ ∥ [100]<br>• ($\boldsymbol{E}$ ∥ [100] or $\boldsymbol{E} \perp$ [010]) and $\boldsymbol{B}$ ∥ [001]

Continued on next page

...continued

Subgroup & SI group	Perturbation
Cm (8.32) $\mathbb{Z}_1$	• $\boldsymbol{B} \parallel [010]$ and strain $\perp [010]$<br>• ($\boldsymbol{E} \parallel [100]$ or $\boldsymbol{E} \perp [010]$) and $\boldsymbol{B} \parallel [010]$
C_cm (8.35) $\mathbb{Z}_1$	• strain $\perp [010]$<br>• ($\boldsymbol{E} \parallel [100]$ or $\boldsymbol{E} \perp [010]$)
Cc' (9.39) $\mathbb{Z}_1$	• $\boldsymbol{B} \parallel [010]$ and strain $\perp [100]$<br>• $\boldsymbol{B} \parallel [001]$ and strain $\perp [100]$<br>• $\boldsymbol{B} \perp [100]$<br>• ($\boldsymbol{E} \parallel [010]$ or $\boldsymbol{E} \perp [100]$) and $\boldsymbol{B} \parallel [010]$<br>• ($\boldsymbol{E} \parallel [010]$ or $\boldsymbol{E} \perp [100]$) and $\boldsymbol{B} \parallel [001]$
Cm (8.32) $\mathbb{Z}_1$	• $\boldsymbol{B} \parallel [100]$ and strain $\perp [100]$<br>• ($\boldsymbol{E} \parallel [010]$ or $\boldsymbol{E} \perp [100]$) and $\boldsymbol{B} \parallel [100]$
C_cm (8.35) $\mathbb{Z}_1$	• strain $\perp [100]$<br>• ($\boldsymbol{E} \parallel [010]$ or $\boldsymbol{E} \perp [100]$)
$C2'$ (5.15) $\mathbb{Z}_1$	• $\boldsymbol{B} \parallel [100]$ and strain $\perp [001]$<br>• $\boldsymbol{B} \parallel [010]$ and strain $\perp [001]$<br>• $\boldsymbol{B} \perp [001]$
$Ima'2'$ (46.244) $\mathbb{Z}_1$	• $\boldsymbol{B} \parallel [010]$
$Ima'2'$ (46.244) $\mathbb{Z}_1$	• $\boldsymbol{B} \parallel [100]$
$C2$ (5.13) $\mathbb{Z}_1$	• $\boldsymbol{B} \parallel [001]$ and strain $\perp [001]$
$Ib'a'2$ (45.238) $\mathbb{Z}_2$	• $\boldsymbol{B} \parallel [001]$
C_a2 (5.17) $\mathbb{Z}_2$	• strain $\perp [001]$

Table. S 344: Subgroups of I_amm2 (44.234) that can be realized by applying $\boldsymbol{E}/\boldsymbol{B}$ fields and/or uniaxial strains.

Subgroup & SI group	Perturbation
P_S1 (1.3) $\mathbb{Z}_1$	• strain in generic direction<br>• ($\boldsymbol{E} \parallel [100]$ or $\boldsymbol{E} \perp [010]$) and strain $\perp [100]$<br>• ($\boldsymbol{E} \parallel [100]$ or $\boldsymbol{E} \perp [010]$) and strain $\perp [001]$<br>• ($\boldsymbol{E} \parallel [010]$ or $\boldsymbol{E} \perp [100]$) and strain $\perp [010]$<br>• ($\boldsymbol{E} \parallel [010]$ or $\boldsymbol{E} \perp [100]$) and strain $\perp [001]$<br>• $\boldsymbol{E}$ in generic direction

Continued on next page

. . . continued

Subgroup & SI group	Perturbation
Cc' (9.39) $\mathbb{Z}_1$	• $\boldsymbol{B} \parallel [100]$ and strain $\perp [010]$<br>• $\boldsymbol{B} \parallel [001]$ and strain $\perp [010]$<br>• $\boldsymbol{B} \perp [010]$<br>• ($\boldsymbol{E} \parallel [100]$ or $\boldsymbol{E} \perp [010]$) and $\boldsymbol{B} \parallel [100]$<br>• ($\boldsymbol{E} \parallel [100]$ or $\boldsymbol{E} \perp [010]$) and $\boldsymbol{B} \parallel [001]$
Cm (8.32) $\mathbb{Z}_1$	• $\boldsymbol{B} \parallel [010]$ and strain $\perp [010]$<br>• ($\boldsymbol{E} \parallel [100]$ or $\boldsymbol{E} \perp [010]$) and $\boldsymbol{B} \parallel [010]$
C_cm (8.35) $\mathbb{Z}_1$	• strain $\perp [010]$<br>• ($\boldsymbol{E} \parallel [100]$ or $\boldsymbol{E} \perp [010]$)
Cm' (8.34) $\mathbb{Z}_1$	• $\boldsymbol{B} \parallel [010]$ and strain $\perp [100]$<br>• $\boldsymbol{B} \parallel [001]$ and strain $\perp [100]$<br>• $\boldsymbol{B} \perp [100]$<br>• ($\boldsymbol{E} \parallel [010]$ or $\boldsymbol{E} \perp [100]$) and $\boldsymbol{B} \parallel [010]$<br>• ($\boldsymbol{E} \parallel [010]$ or $\boldsymbol{E} \perp [100]$) and $\boldsymbol{B} \parallel [001]$
Cm (8.32) $\mathbb{Z}_1$	• $\boldsymbol{B} \parallel [100]$ and strain $\perp [100]$<br>• ($\boldsymbol{E} \parallel [010]$ or $\boldsymbol{E} \perp [100]$) and $\boldsymbol{B} \parallel [100]$
C_am (8.36) $\mathbb{Z}_1$	• strain $\perp [100]$<br>• ($\boldsymbol{E} \parallel [010]$ or $\boldsymbol{E} \perp [100]$)
$C2'$ (5.15) $\mathbb{Z}_1$	• $\boldsymbol{B} \parallel [100]$ and strain $\perp [001]$<br>• $\boldsymbol{B} \parallel [010]$ and strain $\perp [001]$<br>• $\boldsymbol{B} \perp [001]$
$Im'm2'$ (44.231) $\mathbb{Z}_1$	• $\boldsymbol{B} \parallel [010]$
$Ima'2'$ (46.244) $\mathbb{Z}_1$	• $\boldsymbol{B} \parallel [100]$
$C2$ (5.13) $\mathbb{Z}_1$	• $\boldsymbol{B} \parallel [001]$ and strain $\perp [001]$
$Im'a'2$ (46.245) $\mathbb{Z}_1$	• $\boldsymbol{B} \parallel [001]$
C_c2 (5.16) $\mathbb{Z}_1$	• strain $\perp [001]$

Table. S 345: Subgroups of $Iba2$ (45.235) that can be realized by applying $\boldsymbol{E}/\boldsymbol{B}$ fields and/or uniaxial strains.

Subgroup & SI group	Perturbation

Continued on next page

...continued

Subgroup & SI group	Perturbation
Cc (9.37) $\mathbb{Z}_1$	• strain $\perp$ $[010]$<br>• $\boldsymbol{B}$ $\parallel$ $[010]$<br>• ($\boldsymbol{E}$ $\parallel$ $[100]$ or $\boldsymbol{E}$ $\perp$ $[010]$)
Cc (9.37) $\mathbb{Z}_1$	• strain $\perp$ $[100]$<br>• $\boldsymbol{B}$ $\parallel$ $[100]$<br>• ($\boldsymbol{E}$ $\parallel$ $[010]$ or $\boldsymbol{E}$ $\perp$ $[100]$)
$C2$ (5.13) $\mathbb{Z}_1$	• strain $\perp$ $[001]$<br>• $\boldsymbol{B}$ $\parallel$ $[001]$

Table. S 346: Subgroups of $Iba21'$ (45.236) that can be realized by applying $\boldsymbol{E}/\boldsymbol{B}$ fields and/or uniaxial strains.

Subgroup & SI group	Perturbation
$P11'$ (1.2) $\mathbb{Z}_1$	• strain in generic direction<br>• ($\boldsymbol{E}$ $\parallel$ $[100]$ or $\boldsymbol{E}$ $\perp$ $[010]$) and strain $\perp$ $[100]$<br>• ($\boldsymbol{E}$ $\parallel$ $[100]$ or $\boldsymbol{E}$ $\perp$ $[010]$) and strain $\perp$ $[001]$<br>• ($\boldsymbol{E}$ $\parallel$ $[010]$ or $\boldsymbol{E}$ $\perp$ $[100]$) and strain $\perp$ $[010]$<br>• ($\boldsymbol{E}$ $\parallel$ $[010]$ or $\boldsymbol{E}$ $\perp$ $[100]$) and strain $\perp$ $[001]$<br>• $\boldsymbol{E}$ in generic direction
Cc' (9.39) $\mathbb{Z}_1$	• $\boldsymbol{B}$ $\parallel$ $[100]$ and strain $\perp$ $[010]$<br>• $\boldsymbol{B}$ $\parallel$ $[001]$ and strain $\perp$ $[010]$<br>• $\boldsymbol{B}$ $\perp$ $[010]$<br>• ($\boldsymbol{E}$ $\parallel$ $[100]$ or $\boldsymbol{E}$ $\perp$ $[010]$) and $\boldsymbol{B}$ $\parallel$ $[100]$<br>• ($\boldsymbol{E}$ $\parallel$ $[100]$ or $\boldsymbol{E}$ $\perp$ $[010]$) and $\boldsymbol{B}$ $\parallel$ $[001]$
Cc (9.37) $\mathbb{Z}_1$	• $\boldsymbol{B}$ $\parallel$ $[010]$ and strain $\perp$ $[010]$<br>• ($\boldsymbol{E}$ $\parallel$ $[100]$ or $\boldsymbol{E}$ $\perp$ $[010]$) and $\boldsymbol{B}$ $\parallel$ $[010]$
$Cc1'$ (9.38) $\mathbb{Z}_1$	• strain $\perp$ $[010]$<br>• ($\boldsymbol{E}$ $\parallel$ $[100]$ or $\boldsymbol{E}$ $\perp$ $[010]$)
Cc' (9.39) $\mathbb{Z}_1$	• $\boldsymbol{B}$ $\parallel$ $[010]$ and strain $\perp$ $[100]$<br>• $\boldsymbol{B}$ $\parallel$ $[001]$ and strain $\perp$ $[100]$<br>• $\boldsymbol{B}$ $\perp$ $[100]$<br>• ($\boldsymbol{E}$ $\parallel$ $[010]$ or $\boldsymbol{E}$ $\perp$ $[100]$) and $\boldsymbol{B}$ $\parallel$ $[010]$<br>• ($\boldsymbol{E}$ $\parallel$ $[010]$ or $\boldsymbol{E}$ $\perp$ $[100]$) and $\boldsymbol{B}$ $\parallel$ $[001]$
Cc (9.37) $\mathbb{Z}_1$	• $\boldsymbol{B}$ $\parallel$ $[100]$ and strain $\perp$ $[100]$<br>• ($\boldsymbol{E}$ $\parallel$ $[010]$ or $\boldsymbol{E}$ $\perp$ $[100]$) and $\boldsymbol{B}$ $\parallel$ $[100]$

Continued on next page

...continued

Subgroup & SI group	Perturbation
$Cc1'$ (9.38) $\mathbb{Z}_1$	• strain $\perp$ $[100]$<br>• ($\boldsymbol{E}$ $\parallel$ $[010]$ or $\boldsymbol{E}$ $\perp$ $[100]$)
$C2'$ (5.15) $\mathbb{Z}_1$	• $\boldsymbol{B}$ $\parallel$ $[100]$ and strain $\perp$ $[001]$<br>• $\boldsymbol{B}$ $\parallel$ $[010]$ and strain $\perp$ $[001]$<br>• $\boldsymbol{B}$ $\perp$ $[001]$
$Ib'a2'$ (45.237) $\mathbb{Z}_1$	• $\boldsymbol{B}$ $\parallel$ $[010]$
$Ib'a2'$ (45.237) $\mathbb{Z}_1$	• $\boldsymbol{B}$ $\parallel$ $[100]$
$C2$ (5.13) $\mathbb{Z}_1$	• $\boldsymbol{B}$ $\parallel$ $[001]$ and strain $\perp$ $[001]$
$Ib'a'2$ (45.238) $\mathbb{Z}_2$	• $\boldsymbol{B}$ $\parallel$ $[001]$
$C21'$ (5.14) $\mathbb{Z}_1$	• strain $\perp$ $[001]$

Table. S 347: Subgroups of $Ib'a2'$ (45.237) that can be realized by applying $\boldsymbol{E}/\boldsymbol{B}$ fields and/or uniaxial strains.

Subgroup & SI group	Perturbation
Cc (9.37) $\mathbb{Z}_1$	• strain $\perp$ $[010]$<br>• ($\boldsymbol{E}$ $\parallel$ $[100]$ or $\boldsymbol{E}$ $\perp$ $[010]$)
Cc' (9.39) $\mathbb{Z}_1$	• strain $\perp$ $[100]$<br>• ($\boldsymbol{B}$ $\parallel$ $[001]$ or $\boldsymbol{B}$ $\perp$ $[100]$)<br>• ($\boldsymbol{E}$ $\parallel$ $[010]$ or $\boldsymbol{E}$ $\perp$ $[100]$)
$C2'$ (5.15) $\mathbb{Z}_1$	• strain $\perp$ $[001]$<br>• ($\boldsymbol{B}$ $\parallel$ $[100]$ or $\boldsymbol{B}$ $\perp$ $[001]$)

Table. S 348: Subgroups of $Ib'a'2$ (45.238) that can be realized by applying $\boldsymbol{E}/\boldsymbol{B}$ fields and/or uniaxial strains.

Subgroup & SI group	Perturbation
Cc' (9.39) $\mathbb{Z}_1$	• strain $\perp$ $[010]$<br>• ($\boldsymbol{B}$ $\parallel$ $[100]$ or $\boldsymbol{B}$ $\perp$ $[010]$)<br>• ($\boldsymbol{E}$ $\parallel$ $[100]$ or $\boldsymbol{E}$ $\perp$ $[010]$)

Continued on next page

. . . continued

Subgroup & SI group	Perturbation
Cc' (9.39) $\mathbb{Z}_1$	• strain $\perp$ [100]<br>• ($\boldsymbol{B} \parallel$ [010] or $\boldsymbol{B} \perp$ [100])<br>• ($\boldsymbol{E} \parallel$ [010] or $\boldsymbol{E} \perp$ [100])
$C2$ (5.13) $\mathbb{Z}_1$	• strain $\perp$ [001]

Table. S 349: Subgroups of I_cba2 (45.239) that can be realized by applying $\boldsymbol{E}/\boldsymbol{B}$ fields and/or uniaxial strains.

Subgroup & SI group	Perturbation
P_S1 (1.3) $\mathbb{Z}_1$	• strain in generic direction<br>• ($\boldsymbol{E} \parallel$ [100] or $\boldsymbol{E} \perp$ [010]) and strain $\perp$ [100]<br>• ($\boldsymbol{E} \parallel$ [100] or $\boldsymbol{E} \perp$ [010]) and strain $\perp$ [001]<br>• ($\boldsymbol{E} \parallel$ [010] or $\boldsymbol{E} \perp$ [100]) and strain $\perp$ [010]<br>• ($\boldsymbol{E} \parallel$ [010] or $\boldsymbol{E} \perp$ [100]) and strain $\perp$ [001]<br>• $\boldsymbol{E}$ in generic direction
Cc (9.37) $\mathbb{Z}_1$	• $\boldsymbol{B} \parallel$ [010] and strain $\perp$ [010]<br>• ($\boldsymbol{E} \parallel$ [100] or $\boldsymbol{E} \perp$ [010]) and $\boldsymbol{B} \parallel$ [010]
Cm' (8.34) $\mathbb{Z}_1$	• $\boldsymbol{B} \parallel$ [100] and strain $\perp$ [010]<br>• $\boldsymbol{B} \parallel$ [001] and strain $\perp$ [010]<br>• $\boldsymbol{B} \perp$ [010]<br>• ($\boldsymbol{E} \parallel$ [100] or $\boldsymbol{E} \perp$ [010]) and $\boldsymbol{B} \parallel$ [100]<br>• ($\boldsymbol{E} \parallel$ [100] or $\boldsymbol{E} \perp$ [010]) and $\boldsymbol{B} \parallel$ [001]
C_cc (9.40) $\mathbb{Z}_1$	• strain $\perp$ [010]<br>• ($\boldsymbol{E} \parallel$ [100] or $\boldsymbol{E} \perp$ [010])
Cc (9.37) $\mathbb{Z}_1$	• $\boldsymbol{B} \parallel$ [100] and strain $\perp$ [100]<br>• ($\boldsymbol{E} \parallel$ [010] or $\boldsymbol{E} \perp$ [100]) and $\boldsymbol{B} \parallel$ [100]
Cm' (8.34) $\mathbb{Z}_1$	• $\boldsymbol{B} \parallel$ [010] and strain $\perp$ [100]<br>• $\boldsymbol{B} \parallel$ [001] and strain $\perp$ [100]<br>• $\boldsymbol{B} \perp$ [100]<br>• ($\boldsymbol{E} \parallel$ [010] or $\boldsymbol{E} \perp$ [100]) and $\boldsymbol{B} \parallel$ [010]<br>• ($\boldsymbol{E} \parallel$ [010] or $\boldsymbol{E} \perp$ [100]) and $\boldsymbol{B} \parallel$ [001]
C_cc (9.40) $\mathbb{Z}_1$	• strain $\perp$ [100]<br>• ($\boldsymbol{E} \parallel$ [010] or $\boldsymbol{E} \perp$ [100])

Continued on next page

...continued

Subgroup & SI group	Perturbation
$C2'$ (5.15) $\mathbb{Z}_1$	• $\boldsymbol{B}$ ∥ [100] and strain ⊥ [001]<br>• $\boldsymbol{B}$ ∥ [010] and strain ⊥ [001]<br>• $\boldsymbol{B}$ ⊥ [001]
$Im'a2'$ (46.243) $\mathbb{Z}_1$	• $\boldsymbol{B}$ ∥ [100]
$Im'a2'$ (46.243) $\mathbb{Z}_1$	• $\boldsymbol{B}$ ∥ [010]
$C2$ (5.13) $\mathbb{Z}_1$	• $\boldsymbol{B}$ ∥ [001] and strain ⊥ [001]
$Im'm'2$ (44.232) $\mathbb{Z}_1$	• $\boldsymbol{B}$ ∥ [001]
C_a2 (5.17) $\mathbb{Z}_2$	• strain ⊥ [001]

Table. S 350: Subgroups of I_aba2 (45.240) that can be realized by applying $\boldsymbol{E}/\boldsymbol{B}$ fields and/or uniaxial strains.

Subgroup & SI group	Perturbation
P_S1 (1.3) $\mathbb{Z}_1$	• strain in generic direction<br>• ($\boldsymbol{E}$ ∥ [100] or $\boldsymbol{E}$ ⊥ [010]) and strain ⊥ [100]<br>• ($\boldsymbol{E}$ ∥ [100] or $\boldsymbol{E}$ ⊥ [010]) and strain ⊥ [001]<br>• ($\boldsymbol{E}$ ∥ [010] or $\boldsymbol{E}$ ⊥ [100]) and strain ⊥ [010]<br>• ($\boldsymbol{E}$ ∥ [010] or $\boldsymbol{E}$ ⊥ [100]) and strain ⊥ [001]<br>• $\boldsymbol{E}$ in generic direction
Cm' (8.34) $\mathbb{Z}_1$	• $\boldsymbol{B}$ ∥ [100] and strain ⊥ [010]<br>• $\boldsymbol{B}$ ∥ [001] and strain ⊥ [010]<br>• $\boldsymbol{B}$ ⊥ [010]<br>• ($\boldsymbol{E}$ ∥ [100] or $\boldsymbol{E}$ ⊥ [010]) and $\boldsymbol{B}$ ∥ [100]<br>• ($\boldsymbol{E}$ ∥ [100] or $\boldsymbol{E}$ ⊥ [010]) and $\boldsymbol{B}$ ∥ [001]
Cc (9.37) $\mathbb{Z}_1$	• $\boldsymbol{B}$ ∥ [010] and strain ⊥ [010]<br>• ($\boldsymbol{E}$ ∥ [100] or $\boldsymbol{E}$ ⊥ [010]) and $\boldsymbol{B}$ ∥ [010]
C_cc (9.40) $\mathbb{Z}_1$	• strain ⊥ [010]<br>• ($\boldsymbol{E}$ ∥ [100] or $\boldsymbol{E}$ ⊥ [010])
Cc' (9.39) $\mathbb{Z}_1$	• $\boldsymbol{B}$ ∥ [010] and strain ⊥ [100]<br>• $\boldsymbol{B}$ ∥ [001] and strain ⊥ [100]<br>• $\boldsymbol{B}$ ⊥ [100]<br>• ($\boldsymbol{E}$ ∥ [010] or $\boldsymbol{E}$ ⊥ [100]) and $\boldsymbol{B}$ ∥ [010]<br>• ($\boldsymbol{E}$ ∥ [010] or $\boldsymbol{E}$ ⊥ [100]) and $\boldsymbol{B}$ ∥ [001]

Continued on next page

. . . continued

Subgroup & SI group	Perturbation
Cc (9.37) $\mathbb{Z}_1$	• $\boldsymbol{B} \parallel [100]$ and strain $\perp [100]$<br>• ($\boldsymbol{E} \parallel [010]$ or $\boldsymbol{E} \perp [100]$) and $\boldsymbol{B} \parallel [100]$
C_ac (9.41) $\mathbb{Z}_2$	• strain $\perp [100]$<br>• ($\boldsymbol{E} \parallel [010]$ or $\boldsymbol{E} \perp [100]$)
$C2'$ (5.15) $\mathbb{Z}_1$	• $\boldsymbol{B} \parallel [100]$ and strain $\perp [001]$<br>• $\boldsymbol{B} \parallel [010]$ and strain $\perp [001]$<br>• $\boldsymbol{B} \perp [001]$
$Ib'a2'$ (45.237) $\mathbb{Z}_1$	• $\boldsymbol{B} \parallel [010]$
$Im'a2'$ (46.243) $\mathbb{Z}_1$	• $\boldsymbol{B} \parallel [100]$
$C2$ (5.13) $\mathbb{Z}_1$	• $\boldsymbol{B} \parallel [001]$ and strain $\perp [001]$
$Im'a'2$ (46.245) $\mathbb{Z}_1$	• $\boldsymbol{B} \parallel [001]$
C_c2 (5.16) $\mathbb{Z}_1$	• strain $\perp [001]$

Table. S 351: Subgroups of $Ima2$ (46.241) that can be realized by applying $\boldsymbol{E}/\boldsymbol{B}$ fields and/or uniaxial strains.

Subgroup & SI group	Perturbation
Cc (9.37) $\mathbb{Z}_1$	• strain $\perp [010]$<br>• $\boldsymbol{B} \parallel [010]$<br>• ($\boldsymbol{E} \parallel [100]$ or $\boldsymbol{E} \perp [010]$)
Cm (8.32) $\mathbb{Z}_1$	• strain $\perp [100]$<br>• $\boldsymbol{B} \parallel [100]$<br>• ($\boldsymbol{E} \parallel [010]$ or $\boldsymbol{E} \perp [100]$)
$C2$ (5.13) $\mathbb{Z}_1$	• strain $\perp [001]$<br>• $\boldsymbol{B} \parallel [001]$

Table. S 352: Subgroups of $Ima21'$ (46.242) that can be realized by applying $\boldsymbol{E}/\boldsymbol{B}$ fields and/or uniaxial strains.

Subgroup & SI group	Perturbation

Continued on next page

...continued

Subgroup & SI group	Perturbation
$P11'$ (1.2) $\mathbb{Z}_1$	• strain in generic direction<br>• ($\boldsymbol{E} \parallel [100]$ or $\boldsymbol{E} \perp [010]$) and strain $\perp [100]$<br>• ($\boldsymbol{E} \parallel [100]$ or $\boldsymbol{E} \perp [010]$) and strain $\perp [001]$<br>• ($\boldsymbol{E} \parallel [010]$ or $\boldsymbol{E} \perp [100]$) and strain $\perp [010]$<br>• ($\boldsymbol{E} \parallel [010]$ or $\boldsymbol{E} \perp [100]$) and strain $\perp [001]$<br>• $\boldsymbol{E}$ in generic direction
Cc' (9.39) $\mathbb{Z}_1$	• $\boldsymbol{B} \parallel [100]$ and strain $\perp [010]$<br>• $\boldsymbol{B} \parallel [001]$ and strain $\perp [010]$<br>• $\boldsymbol{B} \perp [010]$<br>• ($\boldsymbol{E} \parallel [100]$ or $\boldsymbol{E} \perp [010]$) and $\boldsymbol{B} \parallel [100]$<br>• ($\boldsymbol{E} \parallel [100]$ or $\boldsymbol{E} \perp [010]$) and $\boldsymbol{B} \parallel [001]$
Cc (9.37) $\mathbb{Z}_1$	• $\boldsymbol{B} \parallel [010]$ and strain $\perp [010]$<br>• ($\boldsymbol{E} \parallel [100]$ or $\boldsymbol{E} \perp [010]$) and $\boldsymbol{B} \parallel [010]$
$Cc1'$ (9.38) $\mathbb{Z}_1$	• strain $\perp [010]$<br>• ($\boldsymbol{E} \parallel [100]$ or $\boldsymbol{E} \perp [010]$)
Cm' (8.34) $\mathbb{Z}_1$	• $\boldsymbol{B} \parallel [010]$ and strain $\perp [100]$<br>• $\boldsymbol{B} \parallel [001]$ and strain $\perp [100]$<br>• $\boldsymbol{B} \perp [100]$<br>• ($\boldsymbol{E} \parallel [010]$ or $\boldsymbol{E} \perp [100]$) and $\boldsymbol{B} \parallel [010]$<br>• ($\boldsymbol{E} \parallel [010]$ or $\boldsymbol{E} \perp [100]$) and $\boldsymbol{B} \parallel [001]$
Cm (8.32) $\mathbb{Z}_1$	• $\boldsymbol{B} \parallel [100]$ and strain $\perp [100]$<br>• ($\boldsymbol{E} \parallel [010]$ or $\boldsymbol{E} \perp [100]$) and $\boldsymbol{B} \parallel [100]$
$Cm1'$ (8.33) $\mathbb{Z}_1$	• strain $\perp [100]$<br>• ($\boldsymbol{E} \parallel [010]$ or $\boldsymbol{E} \perp [100]$)
$C2'$ (5.15) $\mathbb{Z}_1$	• $\boldsymbol{B} \parallel [100]$ and strain $\perp [001]$<br>• $\boldsymbol{B} \parallel [010]$ and strain $\perp [001]$<br>• $\boldsymbol{B} \perp [001]$
$Im'a2'$ (46.243) $\mathbb{Z}_1$	• $\boldsymbol{B} \parallel [010]$
$Ima'2'$ (46.244) $\mathbb{Z}_1$	• $\boldsymbol{B} \parallel [100]$
$C2$ (5.13) $\mathbb{Z}_1$	• $\boldsymbol{B} \parallel [001]$ and strain $\perp [001]$
$Im'a'2$ (46.245) $\mathbb{Z}_1$	• $\boldsymbol{B} \parallel [001]$
$C21'$ (5.14) $\mathbb{Z}_1$	• strain $\perp [001]$

Table. S 353: Subgroups of $Im'a2'$ (46.243) that can be realized by applying $\boldsymbol{E}/\boldsymbol{B}$ fields and/or uniaxial strains.

Subgroup & SI group	Perturbation
Cc (9.37) $\mathbb{Z}_1$	• strain $\perp$ [010]<br>• ($\boldsymbol{E} \parallel$ [100] or $\boldsymbol{E} \perp$ [010])
Cm' (8.34) $\mathbb{Z}_1$	• strain $\perp$ [100]<br>• ($\boldsymbol{B} \parallel$ [001] or $\boldsymbol{B} \perp$ [100])<br>• ($\boldsymbol{E} \parallel$ [010] or $\boldsymbol{E} \perp$ [100])
$C2'$ (5.15) $\mathbb{Z}_1$	• strain $\perp$ [001]<br>• ($\boldsymbol{B} \parallel$ [100] or $\boldsymbol{B} \perp$ [001])

Table. S 354: Subgroups of $Ima'2'$ (46.244) that can be realized by applying $\boldsymbol{E}/\boldsymbol{B}$ fields and/or uniaxial strains.

Subgroup & SI group	Perturbation
Cc' (9.39) $\mathbb{Z}_1$	• strain $\perp$ [010]<br>• ($\boldsymbol{B} \parallel$ [001] or $\boldsymbol{B} \perp$ [010])<br>• ($\boldsymbol{E} \parallel$ [100] or $\boldsymbol{E} \perp$ [010])
Cm (8.32) $\mathbb{Z}_1$	• strain $\perp$ [100]<br>• ($\boldsymbol{E} \parallel$ [010] or $\boldsymbol{E} \perp$ [100])
$C2'$ (5.15) $\mathbb{Z}_1$	• strain $\perp$ [001]<br>• ($\boldsymbol{B} \parallel$ [010] or $\boldsymbol{B} \perp$ [001])

Table. S 355: Subgroups of $Im'a'2$ (46.245) that can be realized by applying $\boldsymbol{E}/\boldsymbol{B}$ fields and/or uniaxial strains.

Subgroup & SI group	Perturbation
Cc' (9.39) $\mathbb{Z}_1$	• strain $\perp$ [010]<br>• ($\boldsymbol{B} \parallel$ [100] or $\boldsymbol{B} \perp$ [010])<br>• ($\boldsymbol{E} \parallel$ [100] or $\boldsymbol{E} \perp$ [010])
Cm' (8.34) $\mathbb{Z}_1$	• strain $\perp$ [100]<br>• ($\boldsymbol{B} \parallel$ [010] or $\boldsymbol{B} \perp$ [100])<br>• ($\boldsymbol{E} \parallel$ [010] or $\boldsymbol{E} \perp$ [100])

Continued on next page

...continued

Subgroup & SI group	Perturbation
$C2$ (5.13) $\mathbb{Z}_1$	• strain $\perp$ [001]

Table. S 356: Subgroups of I_cma2 (46.246) that can be realized by applying $\boldsymbol{E}/\boldsymbol{B}$ fields and/or uniaxial strains.

Subgroup & SI group	Perturbation
P_S1 (1.3) $\mathbb{Z}_1$	• strain in generic direction<br>• ($\boldsymbol{E} \parallel$ [100] or $\boldsymbol{E} \perp$ [010]) and strain $\perp$ [100]<br>• ($\boldsymbol{E} \parallel$ [100] or $\boldsymbol{E} \perp$ [010]) and strain $\perp$ [001]<br>• ($\boldsymbol{E} \parallel$ [010] or $\boldsymbol{E} \perp$ [100]) and strain $\perp$ [010]<br>• ($\boldsymbol{E} \parallel$ [010] or $\boldsymbol{E} \perp$ [100]) and strain $\perp$ [001]<br>• $\boldsymbol{E}$ in generic direction
Cc (9.37) $\mathbb{Z}_1$	• $\boldsymbol{B} \parallel$ [010] and strain $\perp$ [010]<br>• ($\boldsymbol{E} \parallel$ [100] or $\boldsymbol{E} \perp$ [010]) and $\boldsymbol{B} \parallel$ [010]
Cm' (8.34) $\mathbb{Z}_1$	• $\boldsymbol{B} \parallel$ [100] and strain $\perp$ [010]<br>• $\boldsymbol{B} \parallel$ [001] and strain $\perp$ [010]<br>• $\boldsymbol{B} \perp$ [010]<br>• ($\boldsymbol{E} \parallel$ [100] or $\boldsymbol{E} \perp$ [010]) and $\boldsymbol{B} \parallel$ [100]<br>• ($\boldsymbol{E} \parallel$ [100] or $\boldsymbol{E} \perp$ [010]) and $\boldsymbol{B} \parallel$ [001]
C_cc (9.40) $\mathbb{Z}_1$	• strain $\perp$ [010]<br>• ($\boldsymbol{E} \parallel$ [100] or $\boldsymbol{E} \perp$ [010])
Cm (8.32) $\mathbb{Z}_1$	• $\boldsymbol{B} \parallel$ [100] and strain $\perp$ [100]<br>• ($\boldsymbol{E} \parallel$ [010] or $\boldsymbol{E} \perp$ [100]) and $\boldsymbol{B} \parallel$ [100]
Cc' (9.39) $\mathbb{Z}_1$	• $\boldsymbol{B} \parallel$ [010] and strain $\perp$ [100]<br>• $\boldsymbol{B} \parallel$ [001] and strain $\perp$ [100]<br>• $\boldsymbol{B} \perp$ [100]<br>• ($\boldsymbol{E} \parallel$ [010] or $\boldsymbol{E} \perp$ [100]) and $\boldsymbol{B} \parallel$ [010]<br>• ($\boldsymbol{E} \parallel$ [010] or $\boldsymbol{E} \perp$ [100]) and $\boldsymbol{B} \parallel$ [001]
C_cm (8.35) $\mathbb{Z}_1$	• strain $\perp$ [100]<br>• ($\boldsymbol{E} \parallel$ [010] or $\boldsymbol{E} \perp$ [100])
$C2'$ (5.15) $\mathbb{Z}_1$	• $\boldsymbol{B} \parallel$ [100] and strain $\perp$ [001]<br>• $\boldsymbol{B} \parallel$ [010] and strain $\perp$ [001]<br>• $\boldsymbol{B} \perp$ [001]
$Im'm2'$ (44.231) $\mathbb{Z}_1$	• $\boldsymbol{B} \parallel$ [100]

Continued on next page

...continued

Subgroup & SI group	Perturbation
$Ib'a2'$ (45.237) $\mathbb{Z}_1$	• $\boldsymbol{B} \parallel [010]$
$C2$ (5.13) $\mathbb{Z}_1$	• $\boldsymbol{B} \parallel [001]$ and strain $\perp [001]$
$Im'a'2$ (46.245) $\mathbb{Z}_1$	• $\boldsymbol{B} \parallel [001]$
C_a2 (5.17) $\mathbb{Z}_2$	• strain $\perp [001]$

Table. S 357: Subgroups of I_ama2 (46.247) that can be realized by applying $\boldsymbol{E}/\boldsymbol{B}$ fields and/or uniaxial strains.

Subgroup & SI group	Perturbation
P_S1 (1.3) $\mathbb{Z}_1$	• strain in generic direction<br>• ($\boldsymbol{E} \parallel [100]$ or $\boldsymbol{E} \perp [010]$) and strain $\perp [100]$<br>• ($\boldsymbol{E} \parallel [100]$ or $\boldsymbol{E} \perp [010]$) and strain $\perp [001]$<br>• ($\boldsymbol{E} \parallel [010]$ or $\boldsymbol{E} \perp [100]$) and strain $\perp [010]$<br>• ($\boldsymbol{E} \parallel [010]$ or $\boldsymbol{E} \perp [100]$) and strain $\perp [001]$<br>• $\boldsymbol{E}$ in generic direction
Cc (9.37) $\mathbb{Z}_1$	• $\boldsymbol{B} \parallel [010]$ and strain $\perp [010]$<br>• ($\boldsymbol{E} \parallel [100]$ or $\boldsymbol{E} \perp [010]$) and $\boldsymbol{B} \parallel [010]$
Cm' (8.34) $\mathbb{Z}_1$	• $\boldsymbol{B} \parallel [100]$ and strain $\perp [010]$<br>• $\boldsymbol{B} \parallel [001]$ and strain $\perp [010]$<br>• $\boldsymbol{B} \perp [010]$<br>• ($\boldsymbol{E} \parallel [100]$ or $\boldsymbol{E} \perp [010]$) and $\boldsymbol{B} \parallel [100]$<br>• ($\boldsymbol{E} \parallel [100]$ or $\boldsymbol{E} \perp [010]$) and $\boldsymbol{B} \parallel [001]$
C_cc (9.40) $\mathbb{Z}_1$	• strain $\perp [010]$<br>• ($\boldsymbol{E} \parallel [100]$ or $\boldsymbol{E} \perp [010]$)
Cm (8.32) $\mathbb{Z}_1$	• $\boldsymbol{B} \parallel [100]$ and strain $\perp [100]$<br>• ($\boldsymbol{E} \parallel [010]$ or $\boldsymbol{E} \perp [100]$) and $\boldsymbol{B} \parallel [100]$
Cm' (8.34) $\mathbb{Z}_1$	• $\boldsymbol{B} \parallel [010]$ and strain $\perp [100]$<br>• $\boldsymbol{B} \parallel [001]$ and strain $\perp [100]$<br>• $\boldsymbol{B} \perp [100]$<br>• ($\boldsymbol{E} \parallel [010]$ or $\boldsymbol{E} \perp [100]$) and $\boldsymbol{B} \parallel [010]$<br>• ($\boldsymbol{E} \parallel [010]$ or $\boldsymbol{E} \perp [100]$) and $\boldsymbol{B} \parallel [001]$
C_am (8.36) $\mathbb{Z}_1$	• strain $\perp [100]$<br>• ($\boldsymbol{E} \parallel [010]$ or $\boldsymbol{E} \perp [100]$)

Continued on next page

…continued

Subgroup & SI group	Perturbation
$C2'$ (5.15) $\mathbb{Z}_1$	• $\boldsymbol{B}$ ∥ [100] and strain ⊥ [001]<br>• $\boldsymbol{B}$ ∥ [010] and strain ⊥ [001]<br>• $\boldsymbol{B}$ ⊥ [001]
$Im'm2'$ (44.231) $\mathbb{Z}_1$	• $\boldsymbol{B}$ ∥ [100]
$Im'a2'$ (46.243) $\mathbb{Z}_1$	• $\boldsymbol{B}$ ∥ [010]
$C2$ (5.13) $\mathbb{Z}_1$	• $\boldsymbol{B}$ ∥ [001] and strain ⊥ [001]
$Im'm'2$ (44.232) $\mathbb{Z}_1$	• $\boldsymbol{B}$ ∥ [001]
C_c2 (5.16) $\mathbb{Z}_1$	• strain ⊥ [001]

Table. S 358: Subgroups of I_bma2 (46.248) that can be realized by applying $\boldsymbol{E}/\boldsymbol{B}$ fields and/or uniaxial strains.

Subgroup & SI group	Perturbation
P_S1 (1.3) $\mathbb{Z}_1$	• strain in generic direction<br>• ($\boldsymbol{E}$ ∥ [100] or $\boldsymbol{E}$ ⊥ [010]) and strain ⊥ [100]<br>• ($\boldsymbol{E}$ ∥ [100] or $\boldsymbol{E}$ ⊥ [010]) and strain ⊥ [001]<br>• ($\boldsymbol{E}$ ∥ [010] or $\boldsymbol{E}$ ⊥ [100]) and strain ⊥ [010]<br>• ($\boldsymbol{E}$ ∥ [010] or $\boldsymbol{E}$ ⊥ [100]) and strain ⊥ [001]<br>• $\boldsymbol{E}$ in generic direction
Cc (9.37) $\mathbb{Z}_1$	• $\boldsymbol{B}$ ∥ [010] and strain ⊥ [010]<br>• ($\boldsymbol{E}$ ∥ [100] or $\boldsymbol{E}$ ⊥ [010]) and $\boldsymbol{B}$ ∥ [010]
Cc' (9.39) $\mathbb{Z}_1$	• $\boldsymbol{B}$ ∥ [100] and strain ⊥ [010]<br>• $\boldsymbol{B}$ ∥ [001] and strain ⊥ [010]<br>• $\boldsymbol{B}$ ⊥ [010]<br>• ($\boldsymbol{E}$ ∥ [100] or $\boldsymbol{E}$ ⊥ [010]) and $\boldsymbol{B}$ ∥ [100]<br>• ($\boldsymbol{E}$ ∥ [100] or $\boldsymbol{E}$ ⊥ [010]) and $\boldsymbol{B}$ ∥ [001]
C_ac (9.41) $\mathbb{Z}_2$	• strain ⊥ [010]<br>• ($\boldsymbol{E}$ ∥ [100] or $\boldsymbol{E}$ ⊥ [010])
Cm (8.32) $\mathbb{Z}_1$	• $\boldsymbol{B}$ ∥ [100] and strain ⊥ [100]<br>• ($\boldsymbol{E}$ ∥ [010] or $\boldsymbol{E}$ ⊥ [100]) and $\boldsymbol{B}$ ∥ [100]

Continued on next page

...continued

Subgroup & SI group	Perturbation
Cc' (9.39) $\mathbb{Z}_1$	• $\boldsymbol{B} \parallel [010]$ and strain $\perp [100]$<br>• $\boldsymbol{B} \parallel [001]$ and strain $\perp [100]$<br>• $\boldsymbol{B} \perp [100]$<br>• ($\boldsymbol{E} \parallel [010]$ or $\boldsymbol{E} \perp [100]$) and $\boldsymbol{B} \parallel [010]$<br>• ($\boldsymbol{E} \parallel [010]$ or $\boldsymbol{E} \perp [100]$) and $\boldsymbol{B} \parallel [001]$
C_cm (8.35) $\mathbb{Z}_1$	• strain $\perp [100]$<br>• ($\boldsymbol{E} \parallel [010]$ or $\boldsymbol{E} \perp [100]$)
$C2'$ (5.15) $\mathbb{Z}_1$	• $\boldsymbol{B} \parallel [100]$ and strain $\perp [001]$<br>• $\boldsymbol{B} \parallel [010]$ and strain $\perp [001]$<br>• $\boldsymbol{B} \perp [001]$
$Ima'2'$ (46.244) $\mathbb{Z}_1$	• $\boldsymbol{B} \parallel [100]$
$Ib'a2'$ (45.237) $\mathbb{Z}_1$	• $\boldsymbol{B} \parallel [010]$
$C2$ (5.13) $\mathbb{Z}_1$	• $\boldsymbol{B} \parallel [001]$ and strain $\perp [001]$
$Ib'a'2$ (45.238) $\mathbb{Z}_2$	• $\boldsymbol{B} \parallel [001]$
C_c2 (5.16) $\mathbb{Z}_1$	• strain $\perp [001]$

Table. S 359: Subgroups of $Pmmm$ (47.249) that can be realized by applying $\boldsymbol{E}/\boldsymbol{B}$ fields and/or uniaxial strains.

Subgroup & SI group	Perturbation
Pm (6.18) $\mathbb{Z}_1$	• $\boldsymbol{E} \parallel [100]$ and strain $\perp [001]$<br>• $\boldsymbol{E} \parallel [100]$ and $\boldsymbol{B} \parallel [001]$<br>• $\boldsymbol{E} \parallel [010]$ and strain $\perp [001]$<br>• $\boldsymbol{E} \parallel [010]$ and $\boldsymbol{B} \parallel [001]$<br>• $\boldsymbol{E} \perp [001]$
Pm (6.18) $\mathbb{Z}_1$	• $\boldsymbol{E} \parallel [100]$ and strain $\perp [010]$<br>• $\boldsymbol{E} \parallel [100]$ and $\boldsymbol{B} \parallel [010]$<br>• $\boldsymbol{E} \parallel [001]$ and strain $\perp [010]$<br>• $\boldsymbol{E} \parallel [001]$ and $\boldsymbol{B} \parallel [010]$<br>• $\boldsymbol{E} \perp [010]$
$P2$ (3.1) $\mathbb{Z}_2$	• $\boldsymbol{E} \parallel [100]$ and strain $\perp [100]$<br>• $\boldsymbol{E} \parallel [100]$ and $\boldsymbol{B} \parallel [100]$
$Pmm2$ (25.57) $\mathbb{Z}_1$	• $\boldsymbol{E} \parallel [100]$

Continued on next page

…continued

Subgroup & SI group	Perturbation
Pm (6.18) $\mathbb{Z}_1$	• $\boldsymbol{E}$ ∥ [010] and strain ⊥ [100]<br>• $\boldsymbol{E}$ ∥ [010] and $\boldsymbol{B}$ ∥ [100]<br>• $\boldsymbol{E}$ ∥ [001] and strain ⊥ [100]<br>• $\boldsymbol{E}$ ∥ [001] and $\boldsymbol{B}$ ∥ [100]<br>• $\boldsymbol{E}$ ⊥ [100]
$P2$ (3.1) $\mathbb{Z}_2$	• $\boldsymbol{E}$ ∥ [010] and strain ⊥ [010]<br>• $\boldsymbol{E}$ ∥ [010] and $\boldsymbol{B}$ ∥ [010]
$Pmm2$ (25.57) $\mathbb{Z}_1$	• $\boldsymbol{E}$ ∥ [010]
$P2$ (3.1) $\mathbb{Z}_2$	• $\boldsymbol{E}$ ∥ [001] and strain ⊥ [001]<br>• $\boldsymbol{E}$ ∥ [001] and $\boldsymbol{B}$ ∥ [001]
$Pmm2$ (25.57) $\mathbb{Z}_1$	• $\boldsymbol{E}$ ∥ [001]
$P\bar{1}$ (2.4) $\mathbb{Z}_2 \times \mathbb{Z}_2 \times \mathbb{Z}_2 \times \mathbb{Z}_4$	• strain in generic direction<br>• $\boldsymbol{B}$ ∥ [100] and strain ⊥ [010]<br>• $\boldsymbol{B}$ ∥ [100] and strain ⊥ [001]<br>• $\boldsymbol{B}$ ∥ [010] and strain ⊥ [100]<br>• $\boldsymbol{B}$ ∥ [010] and strain ⊥ [001]<br>• $\boldsymbol{B}$ ∥ [001] and strain ⊥ [100]<br>• $\boldsymbol{B}$ ∥ [001] and strain ⊥ [010]<br>• $\boldsymbol{B}$ in generic direction
$P2/m$ (10.42) $\mathbb{Z}_2 \times \mathbb{Z}_2 \times \mathbb{Z}_2$	• strain ⊥ [100]<br>• $\boldsymbol{B}$ ∥ [100]
$P2/m$ (10.42) $\mathbb{Z}_2 \times \mathbb{Z}_2 \times \mathbb{Z}_2$	• strain ⊥ [010]<br>• $\boldsymbol{B}$ ∥ [010]
$P2/m$ (10.42) $\mathbb{Z}_2 \times \mathbb{Z}_2 \times \mathbb{Z}_2$	• strain ⊥ [001]<br>• $\boldsymbol{B}$ ∥ [001]

Table. S 360: Subgroups of $Pmmm1'$ (47.250) that can be realized by applying $\boldsymbol{E}/\boldsymbol{B}$ fields and/or uniaxial strains.

Subgroup & SI group	Perturbation

Continued on next page

. . . continued

Subgroup & SI group	Perturbation
$P11'$ (1.2) $\mathbb{Z}_1$	• $\boldsymbol{E} \parallel [100]$ and strain in generic direction<br>• $\boldsymbol{E} \parallel [010]$ and strain in generic direction<br>• $\boldsymbol{E} \parallel [001]$ and strain in generic direction<br>• $\boldsymbol{E} \perp [100]$ and strain $\perp [010]$<br>• $\boldsymbol{E} \perp [100]$ and strain $\perp [001]$<br>• $\boldsymbol{E} \perp [100]$ and strain in generic direction<br>• $\boldsymbol{E} \perp [010]$ and strain $\perp [100]$<br>• $\boldsymbol{E} \perp [010]$ and strain $\perp [001]$<br>• $\boldsymbol{E} \perp [010]$ and strain in generic direction<br>• $\boldsymbol{E} \perp [001]$ and strain $\perp [100]$<br>• $\boldsymbol{E} \perp [001]$ and strain $\perp [010]$<br>• $\boldsymbol{E} \perp [001]$ and strain in generic direction<br>• $\boldsymbol{E}$ in generic direction
Pm' (6.20) $\mathbb{Z}_1$	• $\boldsymbol{E} \parallel [100]$ and $\boldsymbol{B} \parallel [100]$ and strain $\perp [001]$<br>• $\boldsymbol{E} \parallel [100]$ and $\boldsymbol{B} \parallel [010]$ and strain $\perp [001]$<br>• $\boldsymbol{E} \parallel [100]$ and $\boldsymbol{B} \perp [001]$<br>• $\boldsymbol{E} \parallel [010]$ and $\boldsymbol{B} \parallel [100]$ and strain $\perp [001]$<br>• $\boldsymbol{E} \parallel [010]$ and $\boldsymbol{B} \parallel [010]$ and strain $\perp [001]$<br>• $\boldsymbol{E} \parallel [010]$ and $\boldsymbol{B} \perp [001]$<br>• $\boldsymbol{E} \perp [001]$ and $\boldsymbol{B} \parallel [100]$<br>• $\boldsymbol{E} \perp [001]$ and $\boldsymbol{B} \parallel [010]$<br>• $\boldsymbol{E} \perp [001]$ and $\boldsymbol{B} \perp [001]$
Pm (6.18) $\mathbb{Z}_1$	• $\boldsymbol{E} \parallel [100]$ and $\boldsymbol{B} \parallel [001]$ and strain $\perp [001]$<br>• $\boldsymbol{E} \parallel [010]$ and $\boldsymbol{B} \parallel [001]$ and strain $\perp [001]$<br>• $\boldsymbol{E} \perp [001]$ and $\boldsymbol{B} \parallel [001]$
$Pm1'$ (6.19) $\mathbb{Z}_1$	• $\boldsymbol{E} \parallel [100]$ and strain $\perp [001]$<br>• $\boldsymbol{E} \parallel [010]$ and strain $\perp [001]$<br>• $\boldsymbol{E} \perp [001]$
Pm' (6.20) $\mathbb{Z}_1$	• $\boldsymbol{E} \parallel [100]$ and $\boldsymbol{B} \parallel [100]$ and strain $\perp [010]$<br>• $\boldsymbol{E} \parallel [100]$ and $\boldsymbol{B} \parallel [001]$ and strain $\perp [010]$<br>• $\boldsymbol{E} \parallel [100]$ and $\boldsymbol{B} \perp [010]$<br>• $\boldsymbol{E} \parallel [001]$ and $\boldsymbol{B} \parallel [100]$ and strain $\perp [010]$<br>• $\boldsymbol{E} \parallel [001]$ and $\boldsymbol{B} \parallel [001]$ and strain $\perp [010]$<br>• $\boldsymbol{E} \parallel [001]$ and $\boldsymbol{B} \perp [010]$<br>• $\boldsymbol{E} \perp [010]$ and $\boldsymbol{B} \parallel [100]$<br>• $\boldsymbol{E} \perp [010]$ and $\boldsymbol{B} \parallel [001]$<br>• $\boldsymbol{E} \perp [010]$ and $\boldsymbol{B} \perp [010]$

Continued on next page

…continued

Subgroup & SI group	Perturbation
Pm (6.18) $\mathbb{Z}_1$	• $\boldsymbol{E} \parallel [100]$ and $\boldsymbol{B} \parallel [010]$ and strain $\perp [010]$<br>• $\boldsymbol{E} \parallel [001]$ and $\boldsymbol{B} \parallel [010]$ and strain $\perp [010]$<br>• $\boldsymbol{E} \perp [010]$ and $\boldsymbol{B} \parallel [010]$
$Pm1'$ (6.19) $\mathbb{Z}_1$	• $\boldsymbol{E} \parallel [100]$ and strain $\perp [010]$<br>• $\boldsymbol{E} \parallel [001]$ and strain $\perp [010]$<br>• $\boldsymbol{E} \perp [010]$
$P2'$ (3.3) $\mathbb{Z}_1$	• $\boldsymbol{E} \parallel [100]$ and $\boldsymbol{B} \parallel [010]$ and strain $\perp [100]$<br>• $\boldsymbol{E} \parallel [100]$ and $\boldsymbol{B} \parallel [001]$ and strain $\perp [100]$<br>• $\boldsymbol{E} \parallel [100]$ and $\boldsymbol{B} \perp [100]$
$Pm'm2'$ (25.59) $\mathbb{Z}_1$	• $\boldsymbol{E} \parallel [100]$ and $\boldsymbol{B} \parallel [001]$
$Pm'm2'$ (25.59) $\mathbb{Z}_1$	• $\boldsymbol{E} \parallel [100]$ and $\boldsymbol{B} \parallel [010]$
$P2$ (3.1) $\mathbb{Z}_2$	• $\boldsymbol{E} \parallel [100]$ and $\boldsymbol{B} \parallel [100]$ and strain $\perp [100]$
$Pm'm'2$ (25.60) $\mathbb{Z}_2$	• $\boldsymbol{E} \parallel [100]$ and $\boldsymbol{B} \parallel [100]$
$P21'$ (3.2) $\mathbb{Z}_2$	• $\boldsymbol{E} \parallel [100]$ and strain $\perp [100]$
$Pmm21'$ (25.58) $\mathbb{Z}_1$	• $\boldsymbol{E} \parallel [100]$
Pm' (6.20) $\mathbb{Z}_1$	• $\boldsymbol{E} \parallel [010]$ and $\boldsymbol{B} \parallel [010]$ and strain $\perp [100]$<br>• $\boldsymbol{E} \parallel [010]$ and $\boldsymbol{B} \parallel [001]$ and strain $\perp [100]$<br>• $\boldsymbol{E} \parallel [010]$ and $\boldsymbol{B} \perp [100]$<br>• $\boldsymbol{E} \parallel [001]$ and $\boldsymbol{B} \parallel [010]$ and strain $\perp [100]$<br>• $\boldsymbol{E} \parallel [001]$ and $\boldsymbol{B} \parallel [001]$ and strain $\perp [100]$<br>• $\boldsymbol{E} \parallel [001]$ and $\boldsymbol{B} \perp [100]$<br>• $\boldsymbol{E} \perp [100]$ and $\boldsymbol{B} \parallel [010]$<br>• $\boldsymbol{E} \perp [100]$ and $\boldsymbol{B} \parallel [001]$<br>• $\boldsymbol{E} \perp [100]$ and $\boldsymbol{B} \perp [100]$
Pm (6.18) $\mathbb{Z}_1$	• $\boldsymbol{E} \parallel [010]$ and $\boldsymbol{B} \parallel [100]$ and strain $\perp [100]$<br>• $\boldsymbol{E} \parallel [001]$ and $\boldsymbol{B} \parallel [100]$ and strain $\perp [100]$<br>• $\boldsymbol{E} \perp [100]$ and $\boldsymbol{B} \parallel [100]$
$Pm1'$ (6.19) $\mathbb{Z}_1$	• $\boldsymbol{E} \parallel [010]$ and strain $\perp [100]$<br>• $\boldsymbol{E} \parallel [001]$ and strain $\perp [100]$<br>• $\boldsymbol{E} \perp [100]$
$P2'$ (3.3) $\mathbb{Z}_1$	• $\boldsymbol{E} \parallel [010]$ and $\boldsymbol{B} \parallel [100]$ and strain $\perp [010]$<br>• $\boldsymbol{E} \parallel [010]$ and $\boldsymbol{B} \parallel [001]$ and strain $\perp [010]$<br>• $\boldsymbol{E} \parallel [010]$ and $\boldsymbol{B} \perp [010]$
$Pm'm2'$ (25.59) $\mathbb{Z}_1$	• $\boldsymbol{E} \parallel [010]$ and $\boldsymbol{B} \parallel [001]$
$Pm'm2'$ (25.59) $\mathbb{Z}_1$	• $\boldsymbol{E} \parallel [010]$ and $\boldsymbol{B} \parallel [100]$

Continued on next page

...continued

Subgroup & SI group	Perturbation
$P2$ (3.1) $\mathbb{Z}_2$	• $\boldsymbol{E} \parallel [010]$ and $\boldsymbol{B} \parallel [010]$ and strain $\perp [010]$
$Pm'm'2$ (25.60) $\mathbb{Z}_2$	• $\boldsymbol{E} \parallel [010]$ and $\boldsymbol{B} \parallel [010]$
$P21'$ (3.2) $\mathbb{Z}_2$	• $\boldsymbol{E} \parallel [010]$ and strain $\perp [010]$
$Pmm21'$ (25.58) $\mathbb{Z}_1$	• $\boldsymbol{E} \parallel [010]$
$P2'$ (3.3) $\mathbb{Z}_1$	• $\boldsymbol{E} \parallel [001]$ and $\boldsymbol{B} \parallel [100]$ and strain $\perp [001]$<br>• $\boldsymbol{E} \parallel [001]$ and $\boldsymbol{B} \parallel [010]$ and strain $\perp [001]$<br>• $\boldsymbol{E} \parallel [001]$ and $\boldsymbol{B} \perp [001]$
$Pm'm2'$ (25.59) $\mathbb{Z}_1$	• $\boldsymbol{E} \parallel [001]$ and $\boldsymbol{B} \parallel [010]$
$Pm'm2'$ (25.59) $\mathbb{Z}_1$	• $\boldsymbol{E} \parallel [001]$ and $\boldsymbol{B} \parallel [100]$
$P2$ (3.1) $\mathbb{Z}_2$	• $\boldsymbol{E} \parallel [001]$ and $\boldsymbol{B} \parallel [001]$ and strain $\perp [001]$
$Pm'm'2$ (25.60) $\mathbb{Z}_2$	• $\boldsymbol{E} \parallel [001]$ and $\boldsymbol{B} \parallel [001]$
$P21'$ (3.2) $\mathbb{Z}_2$	• $\boldsymbol{E} \parallel [001]$ and strain $\perp [001]$
$Pmm21'$ (25.58) $\mathbb{Z}_1$	• $\boldsymbol{E} \parallel [001]$
$P\bar{1}$ (2.4) $\mathbb{Z}_2 \times \mathbb{Z}_2 \times \mathbb{Z}_2 \times \mathbb{Z}_4$	• $\boldsymbol{B} \parallel [100]$ and strain in generic direction<br>• $\boldsymbol{B} \parallel [010]$ and strain in generic direction<br>• $\boldsymbol{B} \parallel [001]$ and strain in generic direction<br>• $\boldsymbol{B} \perp [100]$ and strain $\perp [010]$<br>• $\boldsymbol{B} \perp [100]$ and strain $\perp [001]$<br>• $\boldsymbol{B} \perp [100]$ and strain in generic direction<br>• $\boldsymbol{B} \perp [010]$ and strain $\perp [100]$<br>• $\boldsymbol{B} \perp [010]$ and strain $\perp [001]$<br>• $\boldsymbol{B} \perp [010]$ and strain in generic direction<br>• $\boldsymbol{B} \perp [001]$ and strain $\perp [100]$<br>• $\boldsymbol{B} \perp [001]$ and strain $\perp [010]$<br>• $\boldsymbol{B} \perp [001]$ and strain in generic direction<br>• $\boldsymbol{B}$ in generic direction
$P2'/m'$ (10.46) $\mathbb{Z}_2 \times \mathbb{Z}_2 \times \mathbb{Z}_2 \times \mathbb{Z}_4$	• $\boldsymbol{B} \parallel [010]$ and strain $\perp [100]$<br>• $\boldsymbol{B} \parallel [001]$ and strain $\perp [100]$<br>• $\boldsymbol{B} \perp [100]$
$P2/m$ (10.42) $\mathbb{Z}_2 \times \mathbb{Z}_2 \times \mathbb{Z}_2$	• $\boldsymbol{B} \parallel [100]$ and strain $\perp [100]$
$P2'/m'$ (10.46) $\mathbb{Z}_2 \times \mathbb{Z}_2 \times \mathbb{Z}_2 \times \mathbb{Z}_4$	• $\boldsymbol{B} \parallel [100]$ and strain $\perp [010]$<br>• $\boldsymbol{B} \parallel [001]$ and strain $\perp [010]$<br>• $\boldsymbol{B} \perp [010]$
$P2/m$ (10.42) $\mathbb{Z}_2 \times \mathbb{Z}_2 \times \mathbb{Z}_2$	• $\boldsymbol{B} \parallel [010]$ and strain $\perp [010]$

Continued on next page

. . . continued

Subgroup & SI group	Perturbation
$P2'/m'$ (10.46) $\mathbb{Z}_2 \times \mathbb{Z}_2 \times \mathbb{Z}_2 \times \mathbb{Z}_4$	• $\boldsymbol{B} \parallel [100]$ and strain $\perp [001]$<br>• $\boldsymbol{B} \parallel [010]$ and strain $\perp [001]$<br>• $\boldsymbol{B} \perp [001]$
$Pm'm'm$ (47.252) $\mathbb{Z}_2 \times \mathbb{Z}_2 \times \mathbb{Z}_2$	• $\boldsymbol{B} \parallel [100]$
$Pm'm'm$ (47.252) $\mathbb{Z}_2 \times \mathbb{Z}_2 \times \mathbb{Z}_2$	• $\boldsymbol{B} \parallel [010]$
$P2/m$ (10.42) $\mathbb{Z}_2 \times \mathbb{Z}_2 \times \mathbb{Z}_2$	• $\boldsymbol{B} \parallel [001]$ and strain $\perp [001]$
$Pm'm'm$ (47.252) $\mathbb{Z}_2 \times \mathbb{Z}_2 \times \mathbb{Z}_2$	• $\boldsymbol{B} \parallel [001]$
$P\bar{1}1'$ (2.5) $\mathbb{Z}_2 \times \mathbb{Z}_2 \times \mathbb{Z}_2 \times \mathbb{Z}_4$	• strain in generic direction
$P2/m1'$ (10.43) $\mathbb{Z}_2 \times \mathbb{Z}_2 \times \mathbb{Z}_2$	• strain $\perp [100]$
$P2/m1'$ (10.43) $\mathbb{Z}_2 \times \mathbb{Z}_2 \times \mathbb{Z}_2$	• strain $\perp [010]$
$P2/m1'$ (10.43) $\mathbb{Z}_2 \times \mathbb{Z}_2 \times \mathbb{Z}_2$	• strain $\perp [001]$

Table. S 361: Subgroups of $Pm'mm$ (47.251) that can be realized by applying $\boldsymbol{E}/\boldsymbol{B}$ fields and/or uniaxial strains.

Subgroup & SI group	Perturbation
Pm (6.18) $\mathbb{Z}_1$	• $\boldsymbol{B} \parallel [001]$ and strain $\perp [001]$<br>• $\boldsymbol{E} \parallel [100]$ and strain $\perp [001]$<br>• $\boldsymbol{E} \parallel [100]$ and $\boldsymbol{B} \parallel [001]$<br>• $\boldsymbol{E} \parallel [010]$ and strain $\perp [001]$<br>• $\boldsymbol{E} \perp [001]$
Pm (6.18) $\mathbb{Z}_1$	• $\boldsymbol{B} \parallel [010]$ and strain $\perp [010]$<br>• $\boldsymbol{E} \parallel [100]$ and strain $\perp [010]$<br>• $\boldsymbol{E} \parallel [100]$ and $\boldsymbol{B} \parallel [010]$<br>• $\boldsymbol{E} \parallel [001]$ and strain $\perp [010]$<br>• $\boldsymbol{E} \perp [010]$
$P2$ (3.1) $\mathbb{Z}_2$	• $\boldsymbol{B} \parallel [100]$ and strain $\perp [100]$<br>• $\boldsymbol{E} \parallel [100]$ and strain $\perp [100]$<br>• $\boldsymbol{E} \parallel [100]$ and $\boldsymbol{B} \parallel [100]$
$Pmm2$ (25.57) $\mathbb{Z}_1$	• $\boldsymbol{E} \parallel [100]$

Continued on next page

…continued

Subgroup & SI group	Perturbation
Pm' (6.20) $\mathbb{Z}_1$	• $\boldsymbol{B}$ ∥ [010] and strain ⊥ [100]<br>• $\boldsymbol{B}$ ∥ [001] and strain ⊥ [100]<br>• $\boldsymbol{B}$ ⊥ [100]<br>• $\boldsymbol{E}$ ∥ [010] and strain ⊥ [100]<br>• $\boldsymbol{E}$ ∥ [010] and $\boldsymbol{B}$ ∥ [010]<br>• $\boldsymbol{E}$ ∥ [001] and strain ⊥ [100]<br>• $\boldsymbol{E}$ ∥ [001] and $\boldsymbol{B}$ ∥ [001]<br>• $\boldsymbol{E}$ ⊥ [100]
$P2'$ (3.3) $\mathbb{Z}_1$	• $\boldsymbol{B}$ ∥ [100] and strain ⊥ [010]<br>• $\boldsymbol{B}$ ∥ [001] and strain ⊥ [010]<br>• $\boldsymbol{B}$ ⊥ [010]<br>• $\boldsymbol{E}$ ∥ [010] and strain ⊥ [010]<br>• $\boldsymbol{E}$ ∥ [010] and $\boldsymbol{B}$ ∥ [100]
$Pm'm2'$ (25.59) $\mathbb{Z}_1$	• $\boldsymbol{B}$ ∥ [001]<br>• $\boldsymbol{E}$ ∥ [010]
$P2'$ (3.3) $\mathbb{Z}_1$	• $\boldsymbol{B}$ ∥ [100] and strain ⊥ [001]<br>• $\boldsymbol{B}$ ∥ [010] and strain ⊥ [001]<br>• $\boldsymbol{B}$ ⊥ [001]<br>• $\boldsymbol{E}$ ∥ [001] and strain ⊥ [001]<br>• $\boldsymbol{E}$ ∥ [001] and $\boldsymbol{B}$ ∥ [100]
$Pm'm2'$ (25.59) $\mathbb{Z}_1$	• $\boldsymbol{B}$ ∥ [010]<br>• $\boldsymbol{E}$ ∥ [001]
$P2'2'2$ (16.3) $\mathbb{Z}_2$	• $\boldsymbol{B}$ ∥ [100]
$P\bar{1}'$ (2.6) $\mathbb{Z}_1$	• strain in generic direction
$P2/m'$ (10.45) $\mathbb{Z}_2$	• strain ⊥ [100]
$P2'/m$ (10.44) $\mathbb{Z}_1$	• strain ⊥ [010]
$P2'/m$ (10.44) $\mathbb{Z}_1$	• strain ⊥ [001]

Table. S 362: Subgroups of $Pm'm'm$ (47.252) that can be realized by applying $\boldsymbol{E}/\boldsymbol{B}$ fields and/or uniaxial strains.

Subgroup & SI group	Perturbation

Continued on next page

. . . continued

Subgroup & SI group	Perturbation
Pm (6.18) $\mathbb{Z}_1$	• $\boldsymbol{E}$ ∥ [100] and strain ⊥ [001]<br>• $\boldsymbol{E}$ ∥ [010] and strain ⊥ [001]<br>• $\boldsymbol{E}$ ⊥ [001]
Pm' (6.20) $\mathbb{Z}_1$	• $\boldsymbol{E}$ ∥ [100] and strain ⊥ [010]<br>• $\boldsymbol{E}$ ∥ [100] and ($\boldsymbol{B}$ ∥ [100] or $\boldsymbol{B}$ ⊥ [010])<br>• $\boldsymbol{E}$ ∥ [001] and strain ⊥ [010]<br>• $\boldsymbol{E}$ ∥ [001] and ($\boldsymbol{B}$ ∥ [100] or $\boldsymbol{B}$ ⊥ [010])<br>• $\boldsymbol{E}$ ⊥ [010]
$P2'$ (3.3) $\mathbb{Z}_1$	• $\boldsymbol{E}$ ∥ [100] and strain ⊥ [100]<br>• $\boldsymbol{E}$ ∥ [100] and ($\boldsymbol{B}$ ∥ [010] or $\boldsymbol{B}$ ⊥ [100])
$Pm'm2'$ (25.59) $\mathbb{Z}_1$	• $\boldsymbol{E}$ ∥ [100]
Pm' (6.20) $\mathbb{Z}_1$	• $\boldsymbol{E}$ ∥ [010] and strain ⊥ [100]<br>• $\boldsymbol{E}$ ∥ [010] and ($\boldsymbol{B}$ ∥ [010] or $\boldsymbol{B}$ ⊥ [100])<br>• $\boldsymbol{E}$ ∥ [001] and strain ⊥ [100]<br>• $\boldsymbol{E}$ ∥ [001] and ($\boldsymbol{B}$ ∥ [010] or $\boldsymbol{B}$ ⊥ [100])<br>• $\boldsymbol{E}$ ⊥ [100]
$P2'$ (3.3) $\mathbb{Z}_1$	• $\boldsymbol{E}$ ∥ [010] and strain ⊥ [010]<br>• $\boldsymbol{E}$ ∥ [010] and ($\boldsymbol{B}$ ∥ [100] or $\boldsymbol{B}$ ⊥ [010])
$Pm'm2'$ (25.59) $\mathbb{Z}_1$	• $\boldsymbol{E}$ ∥ [010]
$P2$ (3.1) $\mathbb{Z}_2$	• $\boldsymbol{E}$ ∥ [001] and strain ⊥ [001]
$Pm'm'2$ (25.60) $\mathbb{Z}_2$	• $\boldsymbol{E}$ ∥ [001]
$P\bar{1}$ (2.4) $\mathbb{Z}_2 \times \mathbb{Z}_2 \times \mathbb{Z}_2 \times \mathbb{Z}_4$	• strain in generic direction<br>• ($\boldsymbol{B}$ ∥ [100] or $\boldsymbol{B}$ ⊥ [010]) and strain ⊥ [100]<br>• ($\boldsymbol{B}$ ∥ [100] or $\boldsymbol{B}$ ⊥ [010]) and strain ⊥ [001]<br>• ($\boldsymbol{B}$ ∥ [010] or $\boldsymbol{B}$ ⊥ [100]) and strain ⊥ [010]<br>• ($\boldsymbol{B}$ ∥ [010] or $\boldsymbol{B}$ ⊥ [100]) and strain ⊥ [001]<br>• $\boldsymbol{B}$ in generic direction
$P2'/m'$ (10.46) $\mathbb{Z}_2 \times \mathbb{Z}_2 \times \mathbb{Z}_2 \times \mathbb{Z}_4$	• strain ⊥ [100]<br>• ($\boldsymbol{B}$ ∥ [010] or $\boldsymbol{B}$ ⊥ [100])
$P2'/m'$ (10.46) $\mathbb{Z}_2 \times \mathbb{Z}_2 \times \mathbb{Z}_2 \times \mathbb{Z}_4$	• strain ⊥ [010]<br>• ($\boldsymbol{B}$ ∥ [100] or $\boldsymbol{B}$ ⊥ [010])
$P2/m$ (10.42) $\mathbb{Z}_2 \times \mathbb{Z}_2 \times \mathbb{Z}_2$	• strain ⊥ [001]

Table. S 363: Subgroups of $Pm'm'm'$ (47.253) that can be realized by applying $\boldsymbol{E}/\boldsymbol{B}$ fields and/or uniaxial strains.

Subgroup & SI group	Perturbation
Pm' (6.20) $\mathbb{Z}_1$	• $\boldsymbol{B}$ ∥ [100] and strain ⊥ [001]<br>• $\boldsymbol{B}$ ∥ [010] and strain ⊥ [001]<br>• $\boldsymbol{B}$ ⊥ [001]<br>• $\boldsymbol{E}$ ∥ [100] and strain ⊥ [001]<br>• $\boldsymbol{E}$ ∥ [100] and $\boldsymbol{B}$ ∥ [010]<br>• $\boldsymbol{E}$ ∥ [010] and strain ⊥ [001]<br>• $\boldsymbol{E}$ ∥ [010] and $\boldsymbol{B}$ ∥ [100]<br>• $\boldsymbol{E}$ ⊥ [001]
Pm' (6.20) $\mathbb{Z}_1$	• $\boldsymbol{B}$ ∥ [100] and strain ⊥ [010]<br>• $\boldsymbol{B}$ ∥ [001] and strain ⊥ [010]<br>• $\boldsymbol{B}$ ⊥ [010]<br>• $\boldsymbol{E}$ ∥ [100] and strain ⊥ [010]<br>• $\boldsymbol{E}$ ∥ [100] and $\boldsymbol{B}$ ∥ [001]<br>• $\boldsymbol{E}$ ∥ [001] and strain ⊥ [010]<br>• $\boldsymbol{E}$ ∥ [001] and $\boldsymbol{B}$ ∥ [100]<br>• $\boldsymbol{E}$ ⊥ [010]
$P2$ (3.1) $\mathbb{Z}_2$	• $\boldsymbol{B}$ ∥ [100] and strain ⊥ [100]<br>• $\boldsymbol{E}$ ∥ [100] and strain ⊥ [100]
$Pm'm'2$ (25.60) $\mathbb{Z}_2$	• $\boldsymbol{B}$ ∥ [100]<br>• $\boldsymbol{E}$ ∥ [100]
Pm' (6.20) $\mathbb{Z}_1$	• $\boldsymbol{B}$ ∥ [010] and strain ⊥ [100]<br>• $\boldsymbol{B}$ ∥ [001] and strain ⊥ [100]<br>• $\boldsymbol{B}$ ⊥ [100]<br>• $\boldsymbol{E}$ ∥ [010] and strain ⊥ [100]<br>• $\boldsymbol{E}$ ∥ [010] and $\boldsymbol{B}$ ∥ [001]<br>• $\boldsymbol{E}$ ∥ [001] and strain ⊥ [100]<br>• $\boldsymbol{E}$ ∥ [001] and $\boldsymbol{B}$ ∥ [010]<br>• $\boldsymbol{E}$ ⊥ [100]
$P2$ (3.1) $\mathbb{Z}_2$	• $\boldsymbol{B}$ ∥ [010] and strain ⊥ [010]<br>• $\boldsymbol{E}$ ∥ [010] and strain ⊥ [010]
$Pm'm'2$ (25.60) $\mathbb{Z}_2$	• $\boldsymbol{B}$ ∥ [010]<br>• $\boldsymbol{E}$ ∥ [010]
$P2$ (3.1) $\mathbb{Z}_2$	• $\boldsymbol{B}$ ∥ [001] and strain ⊥ [001]<br>• $\boldsymbol{E}$ ∥ [001] and strain ⊥ [001]
$Pm'm'2$ (25.60) $\mathbb{Z}_2$	• $\boldsymbol{B}$ ∥ [001]<br>• $\boldsymbol{E}$ ∥ [001]

Continued on next page

...continued

Subgroup & SI group	Perturbation
$P\bar{1}'$ (2.6) $\mathbb{Z}_1$	• strain in generic direction
$P2/m'$ (10.45) $\mathbb{Z}_2$	• strain $\perp$ [100]
$P2/m'$ (10.45) $\mathbb{Z}_2$	• strain $\perp$ [010]
$P2/m'$ (10.45) $\mathbb{Z}_2$	• strain $\perp$ [001]

Table. S 364: Subgroups of P_ammm (47.254) that can be realized by applying $\boldsymbol{E}/\boldsymbol{B}$ fields and/or uniaxial strains.

Subgroup & SI group	Perturbation
Pm (6.18) $\mathbb{Z}_1$	• $\boldsymbol{E} \parallel$ [100] and $\boldsymbol{B} \parallel$ [001] and strain $\perp$ [001]<br>• $\boldsymbol{E} \parallel$ [010] and $\boldsymbol{B} \parallel$ [001] and strain $\perp$ [001]<br>• $\boldsymbol{E} \perp$ [001] and $\boldsymbol{B} \parallel$ [001]
Pm (6.18) $\mathbb{Z}_1$	• $\boldsymbol{E} \parallel$ [100] and $\boldsymbol{B} \parallel$ [010] and strain $\perp$ [010]<br>• $\boldsymbol{E} \parallel$ [001] and $\boldsymbol{B} \parallel$ [010] and strain $\perp$ [010]<br>• $\boldsymbol{E} \perp$ [010] and $\boldsymbol{B} \parallel$ [010]
$P2$ (3.1) $\mathbb{Z}_2$	• $\boldsymbol{E} \parallel$ [100] and $\boldsymbol{B} \parallel$ [100] and strain $\perp$ [100]
P_S1 (1.3) $\mathbb{Z}_1$	• $\boldsymbol{E} \parallel$ [100] and strain in generic direction<br>• $\boldsymbol{E} \parallel$ [010] and strain in generic direction<br>• $\boldsymbol{E} \parallel$ [001] and strain in generic direction<br>• $\boldsymbol{E} \perp$ [100] and strain $\perp$ [010]<br>• $\boldsymbol{E} \perp$ [100] and strain $\perp$ [001]<br>• $\boldsymbol{E} \perp$ [100] and strain in generic direction<br>• $\boldsymbol{E} \perp$ [010] and strain $\perp$ [100]<br>• $\boldsymbol{E} \perp$ [010] and strain $\perp$ [001]<br>• $\boldsymbol{E} \perp$ [010] and strain in generic direction<br>• $\boldsymbol{E} \perp$ [001] and strain $\perp$ [100]<br>• $\boldsymbol{E} \perp$ [001] and strain $\perp$ [010]<br>• $\boldsymbol{E} \perp$ [001] and strain in generic direction<br>• $\boldsymbol{E}$ in generic direction

Continued on next page

...continued

Subgroup & SI group	Perturbation
Pc' (7.26) $\mathbb{Z}_1$	• $\boldsymbol{E}$ || [100] and $\boldsymbol{B}$ || [100] and strain $\perp$ [001]<br>• $\boldsymbol{E}$ || [100] and $\boldsymbol{B}$ || [010] and strain $\perp$ [001]<br>• $\boldsymbol{E}$ || [100] and $\boldsymbol{B}$ $\perp$ [001]<br>• $\boldsymbol{E}$ || [010] and $\boldsymbol{B}$ || [100] and strain $\perp$ [001]<br>• $\boldsymbol{E}$ || [010] and $\boldsymbol{B}$ || [010] and strain $\perp$ [001]<br>• $\boldsymbol{E}$ || [010] and $\boldsymbol{B}$ $\perp$ [001]<br>• $\boldsymbol{E}$ $\perp$ [001] and $\boldsymbol{B}$ || [100]<br>• $\boldsymbol{E}$ $\perp$ [001] and $\boldsymbol{B}$ || [010]<br>• $\boldsymbol{E}$ $\perp$ [001] and $\boldsymbol{B}$ $\perp$ [001]
P_am (6.21) $\mathbb{Z}_1$	• $\boldsymbol{E}$ || [100] and strain $\perp$ [001]<br>• $\boldsymbol{E}$ || [010] and strain $\perp$ [001]<br>• $\boldsymbol{E}$ $\perp$ [001]
Pc' (7.26) $\mathbb{Z}_1$	• $\boldsymbol{E}$ || [100] and $\boldsymbol{B}$ || [100] and strain $\perp$ [010]<br>• $\boldsymbol{E}$ || [100] and $\boldsymbol{B}$ || [001] and strain $\perp$ [010]<br>• $\boldsymbol{E}$ || [100] and $\boldsymbol{B}$ $\perp$ [010]<br>• $\boldsymbol{E}$ || [001] and $\boldsymbol{B}$ || [100] and strain $\perp$ [010]<br>• $\boldsymbol{E}$ || [001] and $\boldsymbol{B}$ || [001] and strain $\perp$ [010]<br>• $\boldsymbol{E}$ || [001] and $\boldsymbol{B}$ $\perp$ [010]<br>• $\boldsymbol{E}$ $\perp$ [010] and $\boldsymbol{B}$ || [100]<br>• $\boldsymbol{E}$ $\perp$ [010] and $\boldsymbol{B}$ || [001]<br>• $\boldsymbol{E}$ $\perp$ [010] and $\boldsymbol{B}$ $\perp$ [010]
P_am (6.21) $\mathbb{Z}_1$	• $\boldsymbol{E}$ || [100] and strain $\perp$ [010]<br>• $\boldsymbol{E}$ || [001] and strain $\perp$ [010]<br>• $\boldsymbol{E}$ $\perp$ [010]
$Pc'c'2$ (27.81) $\mathbb{Z}_2$	• $\boldsymbol{E}$ || [100] and $\boldsymbol{B}$ || [100]
$P2_1'$ (4.9) $\mathbb{Z}_1$	• $\boldsymbol{E}$ || [100] and $\boldsymbol{B}$ || [010] and strain $\perp$ [100]<br>• $\boldsymbol{E}$ || [100] and $\boldsymbol{B}$ || [001] and strain $\perp$ [100]<br>• $\boldsymbol{E}$ || [100] and $\boldsymbol{B}$ $\perp$ [100]
P_b2 (3.5) $\mathbb{Z}_2$	• $\boldsymbol{E}$ || [100] and strain $\perp$ [100]
$Pmc'2_1'$ (26.69) $\mathbb{Z}_1$	• $\boldsymbol{E}$ || [100] and $\boldsymbol{B}$ || [010]
$Pmc'2_1'$ (26.69) $\mathbb{Z}_1$	• $\boldsymbol{E}$ || [100] and $\boldsymbol{B}$ || [001]
P_cmm2 (25.61) $\mathbb{Z}_1$	• $\boldsymbol{E}$ || [100]
Pm (6.18) $\mathbb{Z}_1$	• $\boldsymbol{E}$ || [010] and $\boldsymbol{B}$ || [100] and strain $\perp$ [100]<br>• $\boldsymbol{E}$ || [001] and $\boldsymbol{B}$ || [100] and strain $\perp$ [100]<br>• $\boldsymbol{E}$ $\perp$ [100] and $\boldsymbol{B}$ || [100]
$P2$ (3.1) $\mathbb{Z}_2$	• $\boldsymbol{E}$ || [010] and $\boldsymbol{B}$ || [010] and strain $\perp$ [010]

Continued on next page

. . . continued

Subgroup & SI group	Perturbation
$P2$ (3.1) $\mathbb{Z}_2$	• $\boldsymbol{E}$ ∥ [001] and $\boldsymbol{B}$ ∥ [001] and strain ⊥ [001]
$P\bar{1}$ (2.4) $\mathbb{Z}_2 \times \mathbb{Z}_2 \times \mathbb{Z}_2 \times \mathbb{Z}_4$	• $\boldsymbol{B}$ ∥ [100] and strain in generic direction<br>• $\boldsymbol{B}$ ∥ [010] and strain in generic direction<br>• $\boldsymbol{B}$ ∥ [001] and strain in generic direction<br>• $\boldsymbol{B}$ ⊥ [100] and strain ⊥ [010]<br>• $\boldsymbol{B}$ ⊥ [100] and strain ⊥ [001]<br>• $\boldsymbol{B}$ ⊥ [100] and strain in generic direction<br>• $\boldsymbol{B}$ ⊥ [010] and strain ⊥ [100]<br>• $\boldsymbol{B}$ ⊥ [010] and strain ⊥ [001]<br>• $\boldsymbol{B}$ ⊥ [010] and strain in generic direction<br>• $\boldsymbol{B}$ ⊥ [001] and strain ⊥ [100]<br>• $\boldsymbol{B}$ ⊥ [001] and strain ⊥ [010]<br>• $\boldsymbol{B}$ ⊥ [001] and strain in generic direction<br>• $\boldsymbol{B}$ in generic direction
$P2/m$ (10.42) $\mathbb{Z}_2 \times \mathbb{Z}_2 \times \mathbb{Z}_2$	• $\boldsymbol{B}$ ∥ [100] and strain ⊥ [100]
$P2/m$ (10.42) $\mathbb{Z}_2 \times \mathbb{Z}_2 \times \mathbb{Z}_2$	• $\boldsymbol{B}$ ∥ [010] and strain ⊥ [010]
$P2/m$ (10.42) $\mathbb{Z}_2 \times \mathbb{Z}_2 \times \mathbb{Z}_2$	• $\boldsymbol{B}$ ∥ [001] and strain ⊥ [001]
Pm' (6.20) $\mathbb{Z}_1$	• $\boldsymbol{E}$ ∥ [010] and $\boldsymbol{B}$ ∥ [010] and strain ⊥ [100]<br>• $\boldsymbol{E}$ ∥ [010] and $\boldsymbol{B}$ ∥ [001] and strain ⊥ [100]<br>• $\boldsymbol{E}$ ∥ [010] and $\boldsymbol{B}$ ⊥ [100]<br>• $\boldsymbol{E}$ ∥ [001] and $\boldsymbol{B}$ ∥ [010] and strain ⊥ [100]<br>• $\boldsymbol{E}$ ∥ [001] and $\boldsymbol{B}$ ∥ [001] and strain ⊥ [100]<br>• $\boldsymbol{E}$ ∥ [001] and $\boldsymbol{B}$ ⊥ [100]<br>• $\boldsymbol{E}$ ⊥ [100] and $\boldsymbol{B}$ ∥ [010]<br>• $\boldsymbol{E}$ ⊥ [100] and $\boldsymbol{B}$ ∥ [001]<br>• $\boldsymbol{E}$ ⊥ [100] and $\boldsymbol{B}$ ⊥ [100]
P_bm (6.22) $\mathbb{Z}_1$	• $\boldsymbol{E}$ ∥ [010] and strain ⊥ [100]<br>• $\boldsymbol{E}$ ∥ [001] and strain ⊥ [100]<br>• $\boldsymbol{E}$ ⊥ [100]
$Pm'a'2$ (28.91) $\mathbb{Z}_2$	• $\boldsymbol{E}$ ∥ [010] and $\boldsymbol{B}$ ∥ [010]
$Pm'a'2$ (28.91) $\mathbb{Z}_2$	• $\boldsymbol{E}$ ∥ [001] and $\boldsymbol{B}$ ∥ [001]
$P2_1'/m'$ (11.54) $\mathbb{Z}_2 \times \mathbb{Z}_2 \times \mathbb{Z}_4$	• $\boldsymbol{B}$ ∥ [010] and strain ⊥ [100]<br>• $\boldsymbol{B}$ ∥ [001] and strain ⊥ [100]<br>• $\boldsymbol{B}$ ⊥ [100]

Continued on next page

. . . continued

Subgroup & SI group	Perturbation
$P2'$ (3.3) $\mathbb{Z}_1$	• $\boldsymbol{E} \parallel [010]$ and $\boldsymbol{B} \parallel [100]$ and strain $\perp [010]$<br>• $\boldsymbol{E} \parallel [010]$ and $\boldsymbol{B} \parallel [001]$ and strain $\perp [010]$<br>• $\boldsymbol{E} \parallel [010]$ and $\boldsymbol{B} \perp [010]$
$Pma'2'$ (28.90) $\mathbb{Z}_1$	• $\boldsymbol{E} \parallel [010]$ and $\boldsymbol{B} \parallel [100]$
P_a2 (3.4) $\mathbb{Z}_2$	• $\boldsymbol{E} \parallel [010]$ and strain $\perp [010]$
$P2'/c'$ (13.69) $\mathbb{Z}_2 \times \mathbb{Z}_2 \times \mathbb{Z}_4$	• $\boldsymbol{B} \parallel [100]$ and strain $\perp [010]$<br>• $\boldsymbol{B} \parallel [001]$ and strain $\perp [010]$<br>• $\boldsymbol{B} \perp [010]$
$Pm'm2'$ (25.59) $\mathbb{Z}_1$	• $\boldsymbol{E} \parallel [010]$ and $\boldsymbol{B} \parallel [001]$
P_amm2 (25.62) $\mathbb{Z}_1$	• $\boldsymbol{E} \parallel [010]$
$Pm'ma'$ (51.296) $\mathbb{Z}_2 \times \mathbb{Z}_2 \times \mathbb{Z}_2$	• $\boldsymbol{B} \parallel [001]$
$P2'$ (3.3) $\mathbb{Z}_1$	• $\boldsymbol{E} \parallel [001]$ and $\boldsymbol{B} \parallel [100]$ and strain $\perp [001]$<br>• $\boldsymbol{E} \parallel [001]$ and $\boldsymbol{B} \parallel [010]$ and strain $\perp [001]$<br>• $\boldsymbol{E} \parallel [001]$ and $\boldsymbol{B} \perp [001]$
$Pma'2'$ (28.90) $\mathbb{Z}_1$	• $\boldsymbol{E} \parallel [001]$ and $\boldsymbol{B} \parallel [100]$
P_a2 (3.4) $\mathbb{Z}_2$	• $\boldsymbol{E} \parallel [001]$ and strain $\perp [001]$
$P2'/c'$ (13.69) $\mathbb{Z}_2 \times \mathbb{Z}_2 \times \mathbb{Z}_4$	• $\boldsymbol{B} \parallel [100]$ and strain $\perp [001]$<br>• $\boldsymbol{B} \parallel [010]$ and strain $\perp [001]$<br>• $\boldsymbol{B} \perp [001]$
$Pm'm2'$ (25.59) $\mathbb{Z}_1$	• $\boldsymbol{E} \parallel [001]$ and $\boldsymbol{B} \parallel [010]$
P_amm2 (25.62) $\mathbb{Z}_1$	• $\boldsymbol{E} \parallel [001]$
$Pm'ma'$ (51.296) $\mathbb{Z}_2 \times \mathbb{Z}_2 \times \mathbb{Z}_2$	• $\boldsymbol{B} \parallel [010]$
$Pc'c'm$ (49.269) $\mathbb{Z}_2 \times \mathbb{Z}_2$	• $\boldsymbol{B} \parallel [100]$
$P_S\bar{1}$ (2.7) $\mathbb{Z}_2 \times \mathbb{Z}_2 \times \mathbb{Z}_4$	• strain in generic direction
P_b2/m (10.48) $\mathbb{Z}_2 \times \mathbb{Z}_2$	• strain $\perp [100]$
P_a2/m (10.47) $\mathbb{Z}_2 \times \mathbb{Z}_2 \times \mathbb{Z}_2$	• strain $\perp [010]$
P_a2/m (10.47) $\mathbb{Z}_2 \times \mathbb{Z}_2 \times \mathbb{Z}_2$	• strain $\perp [001]$

Table. S 365: Subgroups of P_Cmmm (47.255) that can be realized by applying $\boldsymbol{E}/\boldsymbol{B}$ fields and/or uniaxial strains.

Subgroup & SI group	Perturbation
Pm (6.18) $\mathbb{Z}_1$	• $\boldsymbol{E} \parallel [100]$ and $\boldsymbol{B} \parallel [001]$ and strain $\perp [001]$<br>• $\boldsymbol{E} \parallel [010]$ and $\boldsymbol{B} \parallel [001]$ and strain $\perp [001]$<br>• $\boldsymbol{E} \perp [001]$ and $\boldsymbol{B} \parallel [001]$
Pm (6.18) $\mathbb{Z}_1$	• $\boldsymbol{E} \parallel [100]$ and $\boldsymbol{B} \parallel [010]$ and strain $\perp [010]$<br>• $\boldsymbol{E} \parallel [001]$ and $\boldsymbol{B} \parallel [010]$ and strain $\perp [010]$<br>• $\boldsymbol{E} \perp [010]$ and $\boldsymbol{B} \parallel [010]$
$P2$ (3.1) $\mathbb{Z}_2$	• $\boldsymbol{E} \parallel [100]$ and $\boldsymbol{B} \parallel [100]$ and strain $\perp [100]$
P_S1 (1.3) $\mathbb{Z}_1$	• $\boldsymbol{E} \parallel [100]$ and strain in generic direction<br>• $\boldsymbol{E} \parallel [010]$ and strain in generic direction<br>• $\boldsymbol{E} \parallel [001]$ and strain in generic direction<br>• $\boldsymbol{E} \perp [100]$ and strain $\perp [010]$<br>• $\boldsymbol{E} \perp [100]$ and strain $\perp [001]$<br>• $\boldsymbol{E} \perp [100]$ and strain in generic direction<br>• $\boldsymbol{E} \perp [010]$ and strain $\perp [100]$<br>• $\boldsymbol{E} \perp [010]$ and strain $\perp [001]$<br>• $\boldsymbol{E} \perp [010]$ and strain in generic direction<br>• $\boldsymbol{E} \perp [001]$ and strain $\perp [100]$<br>• $\boldsymbol{E} \perp [001]$ and strain $\perp [010]$<br>• $\boldsymbol{E} \perp [001]$ and strain in generic direction<br>• $\boldsymbol{E}$ in generic direction
Pc' (7.26) $\mathbb{Z}_1$	• $\boldsymbol{E} \parallel [100]$ and $\boldsymbol{B} \parallel [100]$ and strain $\perp [001]$<br>• $\boldsymbol{E} \parallel [100]$ and $\boldsymbol{B} \parallel [010]$ and strain $\perp [001]$<br>• $\boldsymbol{E} \parallel [100]$ and $\boldsymbol{B} \perp [001]$<br>• $\boldsymbol{E} \parallel [010]$ and $\boldsymbol{B} \parallel [100]$ and strain $\perp [001]$<br>• $\boldsymbol{E} \parallel [010]$ and $\boldsymbol{B} \parallel [010]$ and strain $\perp [001]$<br>• $\boldsymbol{E} \parallel [010]$ and $\boldsymbol{B} \perp [001]$<br>• $\boldsymbol{E} \perp [001]$ and $\boldsymbol{B} \parallel [100]$<br>• $\boldsymbol{E} \perp [001]$ and $\boldsymbol{B} \parallel [010]$<br>• $\boldsymbol{E} \perp [001]$ and $\boldsymbol{B} \perp [001]$
P_am (6.21) $\mathbb{Z}_1$	• $\boldsymbol{E} \parallel [100]$ and strain $\perp [001]$<br>• $\boldsymbol{E} \parallel [010]$ and strain $\perp [001]$<br>• $\boldsymbol{E} \perp [001]$

Continued on next page

...continued

Subgroup & SI group	Perturbation
Pc' (7.26) $\mathbb{Z}_1$	• $\boldsymbol{E}$ ∥ [100] and $\boldsymbol{B}$ ∥ [100] and strain ⊥ [010]<br>• $\boldsymbol{E}$ ∥ [100] and $\boldsymbol{B}$ ∥ [001] and strain ⊥ [010]<br>• $\boldsymbol{E}$ ∥ [100] and $\boldsymbol{B}$ ⊥ [010]<br>• $\boldsymbol{E}$ ∥ [001] and $\boldsymbol{B}$ ∥ [100] and strain ⊥ [010]<br>• $\boldsymbol{E}$ ∥ [001] and $\boldsymbol{B}$ ∥ [001] and strain ⊥ [010]<br>• $\boldsymbol{E}$ ∥ [001] and $\boldsymbol{B}$ ⊥ [010]<br>• $\boldsymbol{E}$ ⊥ [010] and $\boldsymbol{B}$ ∥ [100]<br>• $\boldsymbol{E}$ ⊥ [010] and $\boldsymbol{B}$ ∥ [001]<br>• $\boldsymbol{E}$ ⊥ [010] and $\boldsymbol{B}$ ⊥ [010]
P_Cm (6.23) $\mathbb{Z}_1$	• $\boldsymbol{E}$ ∥ [100] and strain ⊥ [010]<br>• $\boldsymbol{E}$ ∥ [001] and strain ⊥ [010]<br>• $\boldsymbol{E}$ ⊥ [010]
$Pn'c'2$ (30.115) $\mathbb{Z}_1$	• $\boldsymbol{E}$ ∥ [100] and $\boldsymbol{B}$ ∥ [100]
$P2_1'$ (4.9) $\mathbb{Z}_1$	• $\boldsymbol{E}$ ∥ [100] and $\boldsymbol{B}$ ∥ [010] and strain ⊥ [100]<br>• $\boldsymbol{E}$ ∥ [100] and $\boldsymbol{B}$ ∥ [001] and strain ⊥ [100]<br>• $\boldsymbol{E}$ ∥ [100] and $\boldsymbol{B}$ ⊥ [100]
P_C2 (3.6) $\mathbb{Z}_1$	• $\boldsymbol{E}$ ∥ [100] and strain ⊥ [100]
$Pmn'2_1'$ (31.126) $\mathbb{Z}_1$	• $\boldsymbol{E}$ ∥ [100] and $\boldsymbol{B}$ ∥ [010]
$Pmc'2_1'$ (26.69) $\mathbb{Z}_1$	• $\boldsymbol{E}$ ∥ [100] and $\boldsymbol{B}$ ∥ [001]
P_Amm2 (25.64) $\mathbb{Z}_1$	• $\boldsymbol{E}$ ∥ [100]
Pm (6.18) $\mathbb{Z}_1$	• $\boldsymbol{E}$ ∥ [010] and $\boldsymbol{B}$ ∥ [100] and strain ⊥ [100]<br>• $\boldsymbol{E}$ ∥ [001] and $\boldsymbol{B}$ ∥ [100] and strain ⊥ [100]<br>• $\boldsymbol{E}$ ⊥ [100] and $\boldsymbol{B}$ ∥ [100]
$P2$ (3.1) $\mathbb{Z}_2$	• $\boldsymbol{E}$ ∥ [010] and $\boldsymbol{B}$ ∥ [010] and strain ⊥ [010]
$P2$ (3.1) $\mathbb{Z}_2$	• $\boldsymbol{E}$ ∥ [001] and $\boldsymbol{B}$ ∥ [001] and strain ⊥ [001]

Continued on next page

...continued

Subgroup & SI group	Perturbation
$P\bar{1}$ (2.4) $\mathbb{Z}_2 \times \mathbb{Z}_2 \times \mathbb{Z}_2 \times \mathbb{Z}_4$	• $\boldsymbol{B} \parallel [100]$ and strain in generic direction<br>• $\boldsymbol{B} \parallel [010]$ and strain in generic direction<br>• $\boldsymbol{B} \parallel [001]$ and strain in generic direction<br>• $\boldsymbol{B} \perp [100]$ and strain $\perp [010]$<br>• $\boldsymbol{B} \perp [100]$ and strain $\perp [001]$<br>• $\boldsymbol{B} \perp [100]$ and strain in generic direction<br>• $\boldsymbol{B} \perp [010]$ and strain $\perp [100]$<br>• $\boldsymbol{B} \perp [010]$ and strain $\perp [001]$<br>• $\boldsymbol{B} \perp [010]$ and strain in generic direction<br>• $\boldsymbol{B} \perp [001]$ and strain $\perp [100]$<br>• $\boldsymbol{B} \perp [001]$ and strain $\perp [010]$<br>• $\boldsymbol{B} \perp [001]$ and strain in generic direction<br>• $\boldsymbol{B}$ in generic direction
$P2/m$ (10.42) $\mathbb{Z}_2 \times \mathbb{Z}_2 \times \mathbb{Z}_2$	• $\boldsymbol{B} \parallel [100]$ and strain $\perp [100]$
$P2/m$ (10.42) $\mathbb{Z}_2 \times \mathbb{Z}_2 \times \mathbb{Z}_2$	• $\boldsymbol{B} \parallel [010]$ and strain $\perp [010]$
$P2/m$ (10.42) $\mathbb{Z}_2 \times \mathbb{Z}_2 \times \mathbb{Z}_2$	• $\boldsymbol{B} \parallel [001]$ and strain $\perp [001]$
Pc' (7.26) $\mathbb{Z}_1$	• $\boldsymbol{E} \parallel [010]$ and $\boldsymbol{B} \parallel [010]$ and strain $\perp [100]$<br>• $\boldsymbol{E} \parallel [010]$ and $\boldsymbol{B} \parallel [001]$ and strain $\perp [100]$<br>• $\boldsymbol{E} \parallel [010]$ and $\boldsymbol{B} \perp [100]$<br>• $\boldsymbol{E} \parallel [001]$ and $\boldsymbol{B} \parallel [010]$ and strain $\perp [100]$<br>• $\boldsymbol{E} \parallel [001]$ and $\boldsymbol{B} \parallel [001]$ and strain $\perp [100]$<br>• $\boldsymbol{E} \parallel [001]$ and $\boldsymbol{B} \perp [100]$<br>• $\boldsymbol{E} \perp [100]$ and $\boldsymbol{B} \parallel [010]$<br>• $\boldsymbol{E} \perp [100]$ and $\boldsymbol{B} \parallel [001]$<br>• $\boldsymbol{E} \perp [100]$ and $\boldsymbol{B} \perp [100]$
P_Cm (6.23) $\mathbb{Z}_1$	• $\boldsymbol{E} \parallel [010]$ and strain $\perp [100]$<br>• $\boldsymbol{E} \parallel [001]$ and strain $\perp [100]$<br>• $\boldsymbol{E} \perp [100]$
$Pn'c'2$ (30.115) $\mathbb{Z}_1$	• $\boldsymbol{E} \parallel [010]$ and $\boldsymbol{B} \parallel [010]$
$Pb'a'2$ (32.138) $\mathbb{Z}_2$	• $\boldsymbol{E} \parallel [001]$ and $\boldsymbol{B} \parallel [001]$
$P2_1'/c'$ (14.79) $\mathbb{Z}_2 \times \mathbb{Z}_4$	• $\boldsymbol{B} \parallel [010]$ and strain $\perp [100]$<br>• $\boldsymbol{B} \parallel [001]$ and strain $\perp [100]$<br>• $\boldsymbol{B} \perp [100]$
$P2_1'$ (4.9) $\mathbb{Z}_1$	• $\boldsymbol{E} \parallel [010]$ and $\boldsymbol{B} \parallel [100]$ and strain $\perp [010]$<br>• $\boldsymbol{E} \parallel [010]$ and $\boldsymbol{B} \parallel [001]$ and strain $\perp [010]$<br>• $\boldsymbol{E} \parallel [010]$ and $\boldsymbol{B} \perp [010]$

Continued on next page

...continued

Subgroup & SI group	Perturbation
$Pmn'2'_1$ (31.126) $\mathbb{Z}_1$	• $\boldsymbol{E}$ ∥ [010] and $\boldsymbol{B}$ ∥ [100]
P_C2 (3.6) $\mathbb{Z}_1$	• $\boldsymbol{E}$ ∥ [010] and strain ⊥ [010]
$P2'_1/c'$ (14.79) $\mathbb{Z}_2 \times \mathbb{Z}_4$	• $\boldsymbol{B}$ ∥ [100] and strain ⊥ [010]<br>• $\boldsymbol{B}$ ∥ [001] and strain ⊥ [010]<br>• $\boldsymbol{B}$ ⊥ [010]
$Pmc'2'_1$ (26.69) $\mathbb{Z}_1$	• $\boldsymbol{E}$ ∥ [010] and $\boldsymbol{B}$ ∥ [001]
P_Amm2 (25.64) $\mathbb{Z}_1$	• $\boldsymbol{E}$ ∥ [010]
$Pb'a'm$ (55.357) $\mathbb{Z}_2 \times \mathbb{Z}_2 \times \mathbb{Z}_2$	• $\boldsymbol{B}$ ∥ [001]
$P2'$ (3.3) $\mathbb{Z}_1$	• $\boldsymbol{E}$ ∥ [001] and $\boldsymbol{B}$ ∥ [100] and strain ⊥ [001]<br>• $\boldsymbol{E}$ ∥ [001] and $\boldsymbol{B}$ ∥ [010] and strain ⊥ [001]<br>• $\boldsymbol{E}$ ∥ [001] and $\boldsymbol{B}$ ⊥ [001]
$Pma'2'$ (28.90) $\mathbb{Z}_1$	• $\boldsymbol{E}$ ∥ [001] and $\boldsymbol{B}$ ∥ [100]
P_a2 (3.4) $\mathbb{Z}_2$	• $\boldsymbol{E}$ ∥ [001] and strain ⊥ [001]
$P2'/c'$ (13.69) $\mathbb{Z}_2 \times \mathbb{Z}_2 \times \mathbb{Z}_4$	• $\boldsymbol{B}$ ∥ [100] and strain ⊥ [001]<br>• $\boldsymbol{B}$ ∥ [010] and strain ⊥ [001]<br>• $\boldsymbol{B}$ ⊥ [001]
$Pma'2'$ (28.90) $\mathbb{Z}_1$	• $\boldsymbol{E}$ ∥ [001] and $\boldsymbol{B}$ ∥ [010]
P_Cmm2 (25.63) $\mathbb{Z}_1$	• $\boldsymbol{E}$ ∥ [001]
$Pmn'a'$ (53.327) $\mathbb{Z}_2 \times \mathbb{Z}_2$	• $\boldsymbol{B}$ ∥ [010]
$Pmn'a'$ (53.327) $\mathbb{Z}_2 \times \mathbb{Z}_2$	• $\boldsymbol{B}$ ∥ [100]
$P_S\bar{1}$ (2.7) $\mathbb{Z}_2 \times \mathbb{Z}_2 \times \mathbb{Z}_4$	• strain in generic direction
P_C2/m (10.49) $\mathbb{Z}_2 \times \mathbb{Z}_2$	• strain ⊥ [100]
P_C2/m (10.49) $\mathbb{Z}_2 \times \mathbb{Z}_2$	• strain ⊥ [010]
P_a2/m (10.47) $\mathbb{Z}_2 \times \mathbb{Z}_2 \times \mathbb{Z}_2$	• strain ⊥ [001]

Table. S 366: Subgroups of P_Immm (47.256) that can be realized by applying $\boldsymbol{E}/\boldsymbol{B}$ fields and/or uniaxial strains.

Subgroup & SI group	Perturbation

Continued on next page

…continued

Subgroup & SI group	Perturbation
Pm (6.18) $\mathbb{Z}_1$	• $\boldsymbol{E}$ ∥ [100] and $\boldsymbol{B}$ ∥ [001] and strain ⊥ [001]<br>• $\boldsymbol{E}$ ∥ [010] and $\boldsymbol{B}$ ∥ [001] and strain ⊥ [001]<br>• $\boldsymbol{E}$ ⊥ [001] and $\boldsymbol{B}$ ∥ [001]
Pm (6.18) $\mathbb{Z}_1$	• $\boldsymbol{E}$ ∥ [100] and $\boldsymbol{B}$ ∥ [010] and strain ⊥ [010]<br>• $\boldsymbol{E}$ ∥ [001] and $\boldsymbol{B}$ ∥ [010] and strain ⊥ [010]<br>• $\boldsymbol{E}$ ⊥ [010] and $\boldsymbol{B}$ ∥ [010]
$P2$ (3.1) $\mathbb{Z}_2$	• $\boldsymbol{E}$ ∥ [100] and $\boldsymbol{B}$ ∥ [100] and strain ⊥ [100]
P_S1 (1.3) $\mathbb{Z}_1$	• $\boldsymbol{E}$ ∥ [100] and strain in generic direction<br>• $\boldsymbol{E}$ ∥ [010] and strain in generic direction<br>• $\boldsymbol{E}$ ∥ [001] and strain in generic direction<br>• $\boldsymbol{E}$ ⊥ [100] and strain ⊥ [010]<br>• $\boldsymbol{E}$ ⊥ [100] and strain ⊥ [001]<br>• $\boldsymbol{E}$ ⊥ [100] and strain in generic direction<br>• $\boldsymbol{E}$ ⊥ [010] and strain ⊥ [100]<br>• $\boldsymbol{E}$ ⊥ [010] and strain ⊥ [001]<br>• $\boldsymbol{E}$ ⊥ [010] and strain in generic direction<br>• $\boldsymbol{E}$ ⊥ [001] and strain ⊥ [100]<br>• $\boldsymbol{E}$ ⊥ [001] and strain ⊥ [010]<br>• $\boldsymbol{E}$ ⊥ [001] and strain in generic direction<br>• $\boldsymbol{E}$ in generic direction
Pc' (7.26) $\mathbb{Z}_1$	• $\boldsymbol{E}$ ∥ [100] and $\boldsymbol{B}$ ∥ [100] and strain ⊥ [001]<br>• $\boldsymbol{E}$ ∥ [100] and $\boldsymbol{B}$ ∥ [010] and strain ⊥ [001]<br>• $\boldsymbol{E}$ ∥ [100] and $\boldsymbol{B}$ ⊥ [001]<br>• $\boldsymbol{E}$ ∥ [010] and $\boldsymbol{B}$ ∥ [100] and strain ⊥ [001]<br>• $\boldsymbol{E}$ ∥ [010] and $\boldsymbol{B}$ ∥ [010] and strain ⊥ [001]<br>• $\boldsymbol{E}$ ∥ [010] and $\boldsymbol{B}$ ⊥ [001]<br>• $\boldsymbol{E}$ ⊥ [001] and $\boldsymbol{B}$ ∥ [100]<br>• $\boldsymbol{E}$ ⊥ [001] and $\boldsymbol{B}$ ∥ [010]<br>• $\boldsymbol{E}$ ⊥ [001] and $\boldsymbol{B}$ ⊥ [001]
P_Cm (6.23) $\mathbb{Z}_1$	• $\boldsymbol{E}$ ∥ [100] and strain ⊥ [001]<br>• $\boldsymbol{E}$ ∥ [010] and strain ⊥ [001]<br>• $\boldsymbol{E}$ ⊥ [001]

Continued on next page

...continued

Subgroup & SI group	Perturbation
Pc' (7.26) $\mathbb{Z}_1$	• $\boldsymbol{E}$ ∥ [100] and $\boldsymbol{B}$ ∥ [100] and strain ⊥ [010]<br>• $\boldsymbol{E}$ ∥ [100] and $\boldsymbol{B}$ ∥ [001] and strain ⊥ [010]<br>• $\boldsymbol{E}$ ∥ [100] and $\boldsymbol{B}$ ⊥ [010]<br>• $\boldsymbol{E}$ ∥ [001] and $\boldsymbol{B}$ ∥ [100] and strain ⊥ [010]<br>• $\boldsymbol{E}$ ∥ [001] and $\boldsymbol{B}$ ∥ [001] and strain ⊥ [010]<br>• $\boldsymbol{E}$ ∥ [001] and $\boldsymbol{B}$ ⊥ [010]<br>• $\boldsymbol{E}$ ⊥ [010] and $\boldsymbol{B}$ ∥ [100]<br>• $\boldsymbol{E}$ ⊥ [010] and $\boldsymbol{B}$ ∥ [001]<br>• $\boldsymbol{E}$ ⊥ [010] and $\boldsymbol{B}$ ⊥ [010]
P_Cm (6.23) $\mathbb{Z}_1$	• $\boldsymbol{E}$ ∥ [100] and strain ⊥ [010]<br>• $\boldsymbol{E}$ ∥ [001] and strain ⊥ [010]<br>• $\boldsymbol{E}$ ⊥ [010]
$Pn'n'2$ (34.159) $\mathbb{Z}_1$	• $\boldsymbol{E}$ ∥ [100] and $\boldsymbol{B}$ ∥ [100]
$P2_1'$ (4.9) $\mathbb{Z}_1$	• $\boldsymbol{E}$ ∥ [100] and $\boldsymbol{B}$ ∥ [010] and strain ⊥ [100]<br>• $\boldsymbol{E}$ ∥ [100] and $\boldsymbol{B}$ ∥ [001] and strain ⊥ [100]<br>• $\boldsymbol{E}$ ∥ [100] and $\boldsymbol{B}$ ⊥ [100]
P_C2 (3.6) $\mathbb{Z}_1$	• $\boldsymbol{E}$ ∥ [100] and strain ⊥ [100]
$Pmn'2_1'$ (31.126) $\mathbb{Z}_1$	• $\boldsymbol{E}$ ∥ [100] and $\boldsymbol{B}$ ∥ [010]
$Pmn'2_1'$ (31.126) $\mathbb{Z}_1$	• $\boldsymbol{E}$ ∥ [100] and $\boldsymbol{B}$ ∥ [001]
P_Imm2 (25.65) $\mathbb{Z}_1$	• $\boldsymbol{E}$ ∥ [100]
Pm (6.18) $\mathbb{Z}_1$	• $\boldsymbol{E}$ ∥ [010] and $\boldsymbol{B}$ ∥ [100] and strain ⊥ [100]<br>• $\boldsymbol{E}$ ∥ [001] and $\boldsymbol{B}$ ∥ [100] and strain ⊥ [100]<br>• $\boldsymbol{E}$ ⊥ [100] and $\boldsymbol{B}$ ∥ [100]
$P2$ (3.1) $\mathbb{Z}_2$	• $\boldsymbol{E}$ ∥ [010] and $\boldsymbol{B}$ ∥ [010] and strain ⊥ [010]
$P2$ (3.1) $\mathbb{Z}_2$	• $\boldsymbol{E}$ ∥ [001] and $\boldsymbol{B}$ ∥ [001] and strain ⊥ [001]

Continued on next page

...continued

Subgroup & SI group	Perturbation
$P\bar{1}$ (2.4) $\mathbb{Z}_2 \times \mathbb{Z}_2 \times \mathbb{Z}_2 \times \mathbb{Z}_4$	• $\boldsymbol{B} \parallel [100]$ and strain in generic direction<br>• $\boldsymbol{B} \parallel [010]$ and strain in generic direction<br>• $\boldsymbol{B} \parallel [001]$ and strain in generic direction<br>• $\boldsymbol{B} \perp [100]$ and strain $\perp [010]$<br>• $\boldsymbol{B} \perp [100]$ and strain $\perp [001]$<br>• $\boldsymbol{B} \perp [100]$ and strain in generic direction<br>• $\boldsymbol{B} \perp [010]$ and strain $\perp [100]$<br>• $\boldsymbol{B} \perp [010]$ and strain $\perp [001]$<br>• $\boldsymbol{B} \perp [010]$ and strain in generic direction<br>• $\boldsymbol{B} \perp [001]$ and strain $\perp [100]$<br>• $\boldsymbol{B} \perp [001]$ and strain $\perp [010]$<br>• $\boldsymbol{B} \perp [001]$ and strain in generic direction<br>• $\boldsymbol{B}$ in generic direction
$P2/m$ (10.42) $\mathbb{Z}_2 \times \mathbb{Z}_2 \times \mathbb{Z}_2$	• $\boldsymbol{B} \parallel [100]$ and strain $\perp [100]$
$P2/m$ (10.42) $\mathbb{Z}_2 \times \mathbb{Z}_2 \times \mathbb{Z}_2$	• $\boldsymbol{B} \parallel [010]$ and strain $\perp [010]$
$P2/m$ (10.42) $\mathbb{Z}_2 \times \mathbb{Z}_2 \times \mathbb{Z}_2$	• $\boldsymbol{B} \parallel [001]$ and strain $\perp [001]$
Pc' (7.26) $\mathbb{Z}_1$	• $\boldsymbol{E} \parallel [010]$ and $\boldsymbol{B} \parallel [010]$ and strain $\perp [100]$<br>• $\boldsymbol{E} \parallel [010]$ and $\boldsymbol{B} \parallel [001]$ and strain $\perp [100]$<br>• $\boldsymbol{E} \parallel [010]$ and $\boldsymbol{B} \perp [100]$<br>• $\boldsymbol{E} \parallel [001]$ and $\boldsymbol{B} \parallel [010]$ and strain $\perp [100]$<br>• $\boldsymbol{E} \parallel [001]$ and $\boldsymbol{B} \parallel [001]$ and strain $\perp [100]$<br>• $\boldsymbol{E} \parallel [001]$ and $\boldsymbol{B} \perp [100]$<br>• $\boldsymbol{E} \perp [100]$ and $\boldsymbol{B} \parallel [010]$<br>• $\boldsymbol{E} \perp [100]$ and $\boldsymbol{B} \parallel [001]$<br>• $\boldsymbol{E} \perp [100]$ and $\boldsymbol{B} \perp [100]$
P_Cm (6.23) $\mathbb{Z}_1$	• $\boldsymbol{E} \parallel [010]$ and strain $\perp [100]$<br>• $\boldsymbol{E} \parallel [001]$ and strain $\perp [100]$<br>• $\boldsymbol{E} \perp [100]$
$Pn'n'2$ (34.159) $\mathbb{Z}_1$	• $\boldsymbol{E} \parallel [010]$ and $\boldsymbol{B} \parallel [010]$
$Pn'n'2$ (34.159) $\mathbb{Z}_1$	• $\boldsymbol{E} \parallel [001]$ and $\boldsymbol{B} \parallel [001]$
$P2_1'/c'$ (14.79) $\mathbb{Z}_2 \times \mathbb{Z}_4$	• $\boldsymbol{B} \parallel [010]$ and strain $\perp [100]$<br>• $\boldsymbol{B} \parallel [001]$ and strain $\perp [100]$<br>• $\boldsymbol{B} \perp [100]$
$P2_1'$ (4.9) $\mathbb{Z}_1$	• $\boldsymbol{E} \parallel [010]$ and $\boldsymbol{B} \parallel [100]$ and strain $\perp [010]$<br>• $\boldsymbol{E} \parallel [010]$ and $\boldsymbol{B} \parallel [001]$ and strain $\perp [010]$<br>• $\boldsymbol{E} \parallel [010]$ and $\boldsymbol{B} \perp [010]$

Continued on next page

...continued

Subgroup & SI group	Perturbation
$Pmn'2_1'$ (31.126) $\mathbb{Z}_1$	• $\boldsymbol{E}$ ∥ [010] and $\boldsymbol{B}$ ∥ [100]
P_C2 (3.6) $\mathbb{Z}_1$	• $\boldsymbol{E}$ ∥ [010] and strain ⊥ [010]
$P2_1'/c'$ (14.79) $\mathbb{Z}_2\times\mathbb{Z}_4$	• $\boldsymbol{B}$ ∥ [100] and strain ⊥ [010]<br>• $\boldsymbol{B}$ ∥ [001] and strain ⊥ [010]<br>• $\boldsymbol{B}$ ⊥ [010]
$Pmn'2_1'$ (31.126) $\mathbb{Z}_1$	• $\boldsymbol{E}$ ∥ [010] and $\boldsymbol{B}$ ∥ [001]
P_Imm2 (25.65) $\mathbb{Z}_1$	• $\boldsymbol{E}$ ∥ [010]
$Pn'n'm$ (58.397) $\mathbb{Z}_2\times\mathbb{Z}_2$	• $\boldsymbol{B}$ ∥ [001]
$P2_1'$ (4.9) $\mathbb{Z}_1$	• $\boldsymbol{E}$ ∥ [001] and $\boldsymbol{B}$ ∥ [100] and strain ⊥ [001]<br>• $\boldsymbol{E}$ ∥ [001] and $\boldsymbol{B}$ ∥ [010] and strain ⊥ [001]<br>• $\boldsymbol{E}$ ∥ [001] and $\boldsymbol{B}$ ⊥ [001]
$Pmn'2_1'$ (31.126) $\mathbb{Z}_1$	• $\boldsymbol{E}$ ∥ [001] and $\boldsymbol{B}$ ∥ [100]
P_C2 (3.6) $\mathbb{Z}_1$	• $\boldsymbol{E}$ ∥ [001] and strain ⊥ [001]
$P2_1'/c'$ (14.79) $\mathbb{Z}_2\times\mathbb{Z}_4$	• $\boldsymbol{B}$ ∥ [100] and strain ⊥ [001]<br>• $\boldsymbol{B}$ ∥ [010] and strain ⊥ [001]<br>• $\boldsymbol{B}$ ⊥ [001]
$Pmn'2_1'$ (31.126) $\mathbb{Z}_1$	• $\boldsymbol{E}$ ∥ [001] and $\boldsymbol{B}$ ∥ [010]
P_Imm2 (25.65) $\mathbb{Z}_1$	• $\boldsymbol{E}$ ∥ [001]
$Pn'n'm$ (58.397) $\mathbb{Z}_2\times\mathbb{Z}_2$	• $\boldsymbol{B}$ ∥ [010]
$Pn'n'm$ (58.397) $\mathbb{Z}_2\times\mathbb{Z}_2$	• $\boldsymbol{B}$ ∥ [100]
$P_S\bar{1}$ (2.7) $\mathbb{Z}_2\times\mathbb{Z}_2\times\mathbb{Z}_4$	• strain in generic direction
P_C2/m (10.49) $\mathbb{Z}_2\times\mathbb{Z}_2$	• strain ⊥ [100]
P_C2/m (10.49) $\mathbb{Z}_2\times\mathbb{Z}_2$	• strain ⊥ [010]
P_C2/m (10.49) $\mathbb{Z}_2\times\mathbb{Z}_2$	• strain ⊥ [001]

Table. S 367: Subgroups of $Pnnn$ (48.257) that can be realized by applying $\boldsymbol{E}/\boldsymbol{B}$ fields and/or uniaxial strains.

Subgroup & SI group	Perturbation

Continued on next page

...continued

Subgroup & SI group	Perturbation
$P2$ (3.1) $\mathbb{Z}_2$	• $\boldsymbol{E} \parallel [100]$ and strain $\perp [100]$<br>• $\boldsymbol{E} \parallel [100]$ and $\boldsymbol{B} \parallel [100]$
Pc (7.24) $\mathbb{Z}_1$	• $\boldsymbol{E} \parallel [100]$ and strain $\perp [001]$<br>• $\boldsymbol{E} \parallel [100]$ and $\boldsymbol{B} \parallel [001]$<br>• $\boldsymbol{E} \parallel [010]$ and strain $\perp [001]$<br>• $\boldsymbol{E} \parallel [010]$ and $\boldsymbol{B} \parallel [001]$<br>• $\boldsymbol{E} \perp [001]$
Pc (7.24) $\mathbb{Z}_1$	• $\boldsymbol{E} \parallel [100]$ and strain $\perp [010]$<br>• $\boldsymbol{E} \parallel [100]$ and $\boldsymbol{B} \parallel [010]$<br>• $\boldsymbol{E} \parallel [001]$ and strain $\perp [010]$<br>• $\boldsymbol{E} \parallel [001]$ and $\boldsymbol{B} \parallel [010]$<br>• $\boldsymbol{E} \perp [010]$
$Pnn2$ (34.156) $\mathbb{Z}_1$	• $\boldsymbol{E} \parallel [100]$
Pc (7.24) $\mathbb{Z}_1$	• $\boldsymbol{E} \parallel [010]$ and strain $\perp [100]$<br>• $\boldsymbol{E} \parallel [010]$ and $\boldsymbol{B} \parallel [100]$<br>• $\boldsymbol{E} \parallel [001]$ and strain $\perp [100]$<br>• $\boldsymbol{E} \parallel [001]$ and $\boldsymbol{B} \parallel [100]$<br>• $\boldsymbol{E} \perp [100]$
$P\bar{1}$ (2.4) $\mathbb{Z}_2 \times \mathbb{Z}_2 \times \mathbb{Z}_2 \times \mathbb{Z}_4$	• strain in generic direction<br>• $\boldsymbol{B} \parallel [100]$ and strain $\perp [010]$<br>• $\boldsymbol{B} \parallel [100]$ and strain $\perp [001]$<br>• $\boldsymbol{B} \parallel [010]$ and strain $\perp [100]$<br>• $\boldsymbol{B} \parallel [010]$ and strain $\perp [001]$<br>• $\boldsymbol{B} \parallel [001]$ and strain $\perp [100]$<br>• $\boldsymbol{B} \parallel [001]$ and strain $\perp [010]$<br>• $\boldsymbol{B}$ in generic direction
$P2/c$ (13.65) $\mathbb{Z}_2 \times \mathbb{Z}_2$	• strain $\perp [100]$<br>• $\boldsymbol{B} \parallel [100]$
$P2$ (3.1) $\mathbb{Z}_2$	• $\boldsymbol{E} \parallel [010]$ and strain $\perp [010]$<br>• $\boldsymbol{E} \parallel [010]$ and $\boldsymbol{B} \parallel [010]$
$Pnn2$ (34.156) $\mathbb{Z}_1$	• $\boldsymbol{E} \parallel [010]$
$P2/c$ (13.65) $\mathbb{Z}_2 \times \mathbb{Z}_2$	• strain $\perp [010]$<br>• $\boldsymbol{B} \parallel [010]$
$P2$ (3.1) $\mathbb{Z}_2$	• $\boldsymbol{E} \parallel [001]$ and strain $\perp [001]$<br>• $\boldsymbol{E} \parallel [001]$ and $\boldsymbol{B} \parallel [001]$
$Pnn2$ (34.156) $\mathbb{Z}_1$	• $\boldsymbol{E} \parallel [001]$

Continued on next page

...continued

Subgroup & SI group	Perturbation
$P2/c$ (13.65) $\mathbb{Z}_2 \times \mathbb{Z}_2$	• strain $\perp$ [001]<br>• $\boldsymbol{B} \parallel$ [001]

Table. S 368: Subgroups of $Pnnn1'$ (48.258) that can be realized by applying $\boldsymbol{E}/\boldsymbol{B}$ fields and/or uniaxial strains.

Subgroup & SI group	Perturbation
$P11'$ (1.2) $\mathbb{Z}_1$	• $\boldsymbol{E} \parallel$ [100] and strain in generic direction<br>• $\boldsymbol{E} \parallel$ [010] and strain in generic direction<br>• $\boldsymbol{E} \parallel$ [001] and strain in generic direction<br>• $\boldsymbol{E} \perp$ [100] and strain $\perp$ [010]<br>• $\boldsymbol{E} \perp$ [100] and strain $\perp$ [001]<br>• $\boldsymbol{E} \perp$ [100] and strain in generic direction<br>• $\boldsymbol{E} \perp$ [010] and strain $\perp$ [100]<br>• $\boldsymbol{E} \perp$ [010] and strain $\perp$ [001]<br>• $\boldsymbol{E} \perp$ [010] and strain in generic direction<br>• $\boldsymbol{E} \perp$ [001] and strain $\perp$ [100]<br>• $\boldsymbol{E} \perp$ [001] and strain $\perp$ [010]<br>• $\boldsymbol{E} \perp$ [001] and strain in generic direction<br>• $\boldsymbol{E}$ in generic direction
$P2'$ (3.3) $\mathbb{Z}_1$	• $\boldsymbol{E} \parallel$ [100] and $\boldsymbol{B} \parallel$ [010] and strain $\perp$ [100]<br>• $\boldsymbol{E} \parallel$ [100] and $\boldsymbol{B} \parallel$ [001] and strain $\perp$ [100]<br>• $\boldsymbol{E} \parallel$ [100] and $\boldsymbol{B} \perp$ [100]
$P2$ (3.1) $\mathbb{Z}_2$	• $\boldsymbol{E} \parallel$ [100] and $\boldsymbol{B} \parallel$ [100] and strain $\perp$ [100]
$P21'$ (3.2) $\mathbb{Z}_2$	• $\boldsymbol{E} \parallel$ [100] and strain $\perp$ [100]
Pc' (7.26) $\mathbb{Z}_1$	• $\boldsymbol{E} \parallel$ [100] and $\boldsymbol{B} \parallel$ [100] and strain $\perp$ [001]<br>• $\boldsymbol{E} \parallel$ [100] and $\boldsymbol{B} \parallel$ [010] and strain $\perp$ [001]<br>• $\boldsymbol{E} \parallel$ [100] and $\boldsymbol{B} \perp$ [001]<br>• $\boldsymbol{E} \parallel$ [010] and $\boldsymbol{B} \parallel$ [100] and strain $\perp$ [001]<br>• $\boldsymbol{E} \parallel$ [010] and $\boldsymbol{B} \parallel$ [010] and strain $\perp$ [001]<br>• $\boldsymbol{E} \parallel$ [010] and $\boldsymbol{B} \perp$ [001]<br>• $\boldsymbol{E} \perp$ [001] and $\boldsymbol{B} \parallel$ [100]<br>• $\boldsymbol{E} \perp$ [001] and $\boldsymbol{B} \parallel$ [010]<br>• $\boldsymbol{E} \perp$ [001] and $\boldsymbol{B} \perp$ [001]

Continued on next page

...continued

Subgroup & SI group	Perturbation
Pc (7.24) $\mathbb{Z}_1$	• $\boldsymbol{E}$ ∥ [100] and $\boldsymbol{B}$ ∥ [001] and strain ⊥ [001]<br>• $\boldsymbol{E}$ ∥ [010] and $\boldsymbol{B}$ ∥ [001] and strain ⊥ [001]<br>• $\boldsymbol{E}$ ⊥ [001] and $\boldsymbol{B}$ ∥ [001]
$Pc1'$ (7.25) $\mathbb{Z}_1$	• $\boldsymbol{E}$ ∥ [100] and strain ⊥ [001]<br>• $\boldsymbol{E}$ ∥ [010] and strain ⊥ [001]<br>• $\boldsymbol{E}$ ⊥ [001]
Pc' (7.26) $\mathbb{Z}_1$	• $\boldsymbol{E}$ ∥ [100] and $\boldsymbol{B}$ ∥ [100] and strain ⊥ [010]<br>• $\boldsymbol{E}$ ∥ [100] and $\boldsymbol{B}$ ∥ [001] and strain ⊥ [010]<br>• $\boldsymbol{E}$ ∥ [100] and $\boldsymbol{B}$ ⊥ [010]<br>• $\boldsymbol{E}$ ∥ [001] and $\boldsymbol{B}$ ∥ [100] and strain ⊥ [010]<br>• $\boldsymbol{E}$ ∥ [001] and $\boldsymbol{B}$ ∥ [001] and strain ⊥ [010]<br>• $\boldsymbol{E}$ ∥ [001] and $\boldsymbol{B}$ ⊥ [010]<br>• $\boldsymbol{E}$ ⊥ [010] and $\boldsymbol{B}$ ∥ [100]<br>• $\boldsymbol{E}$ ⊥ [010] and $\boldsymbol{B}$ ∥ [001]<br>• $\boldsymbol{E}$ ⊥ [010] and $\boldsymbol{B}$ ⊥ [010]
$Pn'n'2$ (34.159) $\mathbb{Z}_1$	• $\boldsymbol{E}$ ∥ [100] and $\boldsymbol{B}$ ∥ [100]
$Pn'n2'$ (34.158) $\mathbb{Z}_1$	• $\boldsymbol{E}$ ∥ [100] and $\boldsymbol{B}$ ∥ [001]
Pc (7.24) $\mathbb{Z}_1$	• $\boldsymbol{E}$ ∥ [100] and $\boldsymbol{B}$ ∥ [010] and strain ⊥ [010]<br>• $\boldsymbol{E}$ ∥ [001] and $\boldsymbol{B}$ ∥ [010] and strain ⊥ [010]<br>• $\boldsymbol{E}$ ⊥ [010] and $\boldsymbol{B}$ ∥ [010]
$Pn'n2'$ (34.158) $\mathbb{Z}_1$	• $\boldsymbol{E}$ ∥ [100] and $\boldsymbol{B}$ ∥ [010]
$Pc1'$ (7.25) $\mathbb{Z}_1$	• $\boldsymbol{E}$ ∥ [100] and strain ⊥ [010]<br>• $\boldsymbol{E}$ ∥ [001] and strain ⊥ [010]<br>• $\boldsymbol{E}$ ⊥ [010]
$Pnn21'$ (34.157) $\mathbb{Z}_1$	• $\boldsymbol{E}$ ∥ [100]
Pc' (7.26) $\mathbb{Z}_1$	• $\boldsymbol{E}$ ∥ [010] and $\boldsymbol{B}$ ∥ [010] and strain ⊥ [100]<br>• $\boldsymbol{E}$ ∥ [010] and $\boldsymbol{B}$ ∥ [001] and strain ⊥ [100]<br>• $\boldsymbol{E}$ ∥ [010] and $\boldsymbol{B}$ ⊥ [100]<br>• $\boldsymbol{E}$ ∥ [001] and $\boldsymbol{B}$ ∥ [010] and strain ⊥ [100]<br>• $\boldsymbol{E}$ ∥ [001] and $\boldsymbol{B}$ ∥ [001] and strain ⊥ [100]<br>• $\boldsymbol{E}$ ∥ [001] and $\boldsymbol{B}$ ⊥ [100]<br>• $\boldsymbol{E}$ ⊥ [100] and $\boldsymbol{B}$ ∥ [010]<br>• $\boldsymbol{E}$ ⊥ [100] and $\boldsymbol{B}$ ∥ [001]<br>• $\boldsymbol{E}$ ⊥ [100] and $\boldsymbol{B}$ ⊥ [100]

Continued on next page

. . . continued

Subgroup & SI group	Perturbation
Pc (7.24) $\mathbb{Z}_1$	• $\boldsymbol{E} \parallel [010]$ and $\boldsymbol{B} \parallel [100]$ and strain $\perp [100]$<br>• $\boldsymbol{E} \parallel [001]$ and $\boldsymbol{B} \parallel [100]$ and strain $\perp [100]$<br>• $\boldsymbol{E} \perp [100]$ and $\boldsymbol{B} \parallel [100]$
$Pc1'$ (7.25) $\mathbb{Z}_1$	• $\boldsymbol{E} \parallel [010]$ and strain $\perp [100]$<br>• $\boldsymbol{E} \parallel [001]$ and strain $\perp [100]$<br>• $\boldsymbol{E} \perp [100]$
$P\bar{1}$ (2.4) $\mathbb{Z}_2 \times \mathbb{Z}_2 \times \mathbb{Z}_2 \times \mathbb{Z}_4$	• $\boldsymbol{B} \parallel [100]$ and strain in generic direction<br>• $\boldsymbol{B} \parallel [010]$ and strain in generic direction<br>• $\boldsymbol{B} \parallel [001]$ and strain in generic direction<br>• $\boldsymbol{B} \perp [100]$ and strain $\perp [010]$<br>• $\boldsymbol{B} \perp [100]$ and strain $\perp [001]$<br>• $\boldsymbol{B} \perp [100]$ and strain in generic direction<br>• $\boldsymbol{B} \perp [010]$ and strain $\perp [100]$<br>• $\boldsymbol{B} \perp [010]$ and strain $\perp [001]$<br>• $\boldsymbol{B} \perp [010]$ and strain in generic direction<br>• $\boldsymbol{B} \perp [001]$ and strain $\perp [100]$<br>• $\boldsymbol{B} \perp [001]$ and strain $\perp [010]$<br>• $\boldsymbol{B} \perp [001]$ and strain in generic direction<br>• $\boldsymbol{B}$ in generic direction
$P2'/c'$ (13.69) $\mathbb{Z}_2 \times \mathbb{Z}_2 \times \mathbb{Z}_4$	• $\boldsymbol{B} \parallel [010]$ and strain $\perp [100]$<br>• $\boldsymbol{B} \parallel [001]$ and strain $\perp [100]$<br>• $\boldsymbol{B} \perp [100]$
$P2/c$ (13.65) $\mathbb{Z}_2 \times \mathbb{Z}_2$	• $\boldsymbol{B} \parallel [100]$ and strain $\perp [100]$
$P\bar{1}1'$ (2.5) $\mathbb{Z}_2 \times \mathbb{Z}_2 \times \mathbb{Z}_2 \times \mathbb{Z}_4$	• strain in generic direction
$P2/c1'$ (13.66) $\mathbb{Z}_2 \times \mathbb{Z}_2$	• strain $\perp [100]$
$P2'$ (3.3) $\mathbb{Z}_1$	• $\boldsymbol{E} \parallel [010]$ and $\boldsymbol{B} \parallel [100]$ and strain $\perp [010]$<br>• $\boldsymbol{E} \parallel [010]$ and $\boldsymbol{B} \parallel [001]$ and strain $\perp [010]$<br>• $\boldsymbol{E} \parallel [010]$ and $\boldsymbol{B} \perp [010]$
$Pn'n2'$ (34.158) $\mathbb{Z}_1$	• $\boldsymbol{E} \parallel [010]$ and $\boldsymbol{B} \parallel [001]$
$Pn'n2'$ (34.158) $\mathbb{Z}_1$	• $\boldsymbol{E} \parallel [010]$ and $\boldsymbol{B} \parallel [100]$
$P2'/c'$ (13.69) $\mathbb{Z}_2 \times \mathbb{Z}_2 \times \mathbb{Z}_4$	• $\boldsymbol{B} \parallel [100]$ and strain $\perp [010]$<br>• $\boldsymbol{B} \parallel [001]$ and strain $\perp [010]$<br>• $\boldsymbol{B} \perp [010]$
$P2$ (3.1) $\mathbb{Z}_2$	• $\boldsymbol{E} \parallel [010]$ and $\boldsymbol{B} \parallel [010]$ and strain $\perp [010]$
$Pn'n'2$ (34.159) $\mathbb{Z}_1$	• $\boldsymbol{E} \parallel [010]$ and $\boldsymbol{B} \parallel [010]$

Continued on next page

...continued

Subgroup & SI group	Perturbation
$P2/c$ (13.65) $\mathbb{Z}_2 \times \mathbb{Z}_2$	• $\boldsymbol{B} \parallel [010]$ and strain $\perp [010]$
$P21'$ (3.2) $\mathbb{Z}_2$	• $\boldsymbol{E} \parallel [010]$ and strain $\perp [010]$
$Pnn21'$ (34.157) $\mathbb{Z}_1$	• $\boldsymbol{E} \parallel [010]$
$P2/c1'$ (13.66) $\mathbb{Z}_2 \times \mathbb{Z}_2$	• strain $\perp [010]$
$P2'$ (3.3) $\mathbb{Z}_1$	• $\boldsymbol{E} \parallel [001]$ and $\boldsymbol{B} \parallel [100]$ and strain $\perp [001]$<br>• $\boldsymbol{E} \parallel [001]$ and $\boldsymbol{B} \parallel [010]$ and strain $\perp [001]$<br>• $\boldsymbol{E} \parallel [001]$ and $\boldsymbol{B} \perp [001]$
$Pn'n2'$ (34.158) $\mathbb{Z}_1$	• $\boldsymbol{E} \parallel [001]$ and $\boldsymbol{B} \parallel [010]$
$Pn'n2'$ (34.158) $\mathbb{Z}_1$	• $\boldsymbol{E} \parallel [001]$ and $\boldsymbol{B} \parallel [100]$
$P2'/c'$ (13.69) $\mathbb{Z}_2 \times \mathbb{Z}_2 \times \mathbb{Z}_4$	• $\boldsymbol{B} \parallel [100]$ and strain $\perp [001]$<br>• $\boldsymbol{B} \parallel [010]$ and strain $\perp [001]$<br>• $\boldsymbol{B} \perp [001]$
$Pn'n'n$ (48.260) $\mathbb{Z}_2$	• $\boldsymbol{B} \parallel [100]$
$Pn'n'n$ (48.260) $\mathbb{Z}_2$	• $\boldsymbol{B} \parallel [010]$
$P2$ (3.1) $\mathbb{Z}_2$	• $\boldsymbol{E} \parallel [001]$ and $\boldsymbol{B} \parallel [001]$ and strain $\perp [001]$
$Pn'n'2$ (34.159) $\mathbb{Z}_1$	• $\boldsymbol{E} \parallel [001]$ and $\boldsymbol{B} \parallel [001]$
$P2/c$ (13.65) $\mathbb{Z}_2 \times \mathbb{Z}_2$	• $\boldsymbol{B} \parallel [001]$ and strain $\perp [001]$
$Pn'n'n$ (48.260) $\mathbb{Z}_2$	• $\boldsymbol{B} \parallel [001]$
$P21'$ (3.2) $\mathbb{Z}_2$	• $\boldsymbol{E} \parallel [001]$ and strain $\perp [001]$
$Pnn21'$ (34.157) $\mathbb{Z}_1$	• $\boldsymbol{E} \parallel [001]$
$P2/c1'$ (13.66) $\mathbb{Z}_2 \times \mathbb{Z}_2$	• strain $\perp [001]$

Table. S 369: Subgroups of $Pn'nn$ (48.259) that can be realized by applying $\boldsymbol{E}/\boldsymbol{B}$ fields and/or uniaxial strains.

Subgroup & SI group	Perturbation
$P2$ (3.1) $\mathbb{Z}_2$	• $\boldsymbol{B} \parallel [100]$ and strain $\perp [100]$<br>• $\boldsymbol{E} \parallel [100]$ and strain $\perp [100]$<br>• $\boldsymbol{E} \parallel [100]$ and $\boldsymbol{B} \parallel [100]$

Continued on next page

...continued

Subgroup & SI group	Perturbation
Pc (7.24) $\mathbb{Z}_1$	• $\boldsymbol{B} \parallel [001]$ and strain $\perp [001]$<br>• $\boldsymbol{E} \parallel [100]$ and strain $\perp [001]$<br>• $\boldsymbol{E} \parallel [100]$ and $\boldsymbol{B} \parallel [001]$<br>• $\boldsymbol{E} \parallel [010]$ and strain $\perp [001]$<br>• $\boldsymbol{E} \perp [001]$
Pc (7.24) $\mathbb{Z}_1$	• $\boldsymbol{B} \parallel [010]$ and strain $\perp [010]$<br>• $\boldsymbol{E} \parallel [100]$ and strain $\perp [010]$<br>• $\boldsymbol{E} \parallel [100]$ and $\boldsymbol{B} \parallel [010]$<br>• $\boldsymbol{E} \parallel [001]$ and strain $\perp [010]$<br>• $\boldsymbol{E} \perp [010]$
$Pnn2$ (34.156) $\mathbb{Z}_1$	• $\boldsymbol{E} \parallel [100]$
Pc' (7.26) $\mathbb{Z}_1$	• $\boldsymbol{B} \parallel [010]$ and strain $\perp [100]$<br>• $\boldsymbol{B} \parallel [001]$ and strain $\perp [100]$<br>• $\boldsymbol{B} \perp [100]$<br>• $\boldsymbol{E} \parallel [010]$ and strain $\perp [100]$<br>• $\boldsymbol{E} \parallel [010]$ and $\boldsymbol{B} \parallel [010]$<br>• $\boldsymbol{E} \parallel [001]$ and strain $\perp [100]$<br>• $\boldsymbol{E} \parallel [001]$ and $\boldsymbol{B} \parallel [001]$<br>• $\boldsymbol{E} \perp [100]$
$P\bar{1}'$ (2.6) $\mathbb{Z}_1$	• strain in generic direction
$P2/c'$ (13.68) $\mathbb{Z}_2$	• strain $\perp [100]$
$P2'$ (3.3) $\mathbb{Z}_1$	• $\boldsymbol{B} \parallel [100]$ and strain $\perp [010]$<br>• $\boldsymbol{B} \parallel [001]$ and strain $\perp [010]$<br>• $\boldsymbol{B} \perp [010]$<br>• $\boldsymbol{E} \parallel [010]$ and strain $\perp [010]$<br>• $\boldsymbol{E} \parallel [010]$ and $\boldsymbol{B} \parallel [100]$
$Pn'n2'$ (34.158) $\mathbb{Z}_1$	• $\boldsymbol{B} \parallel [001]$<br>• $\boldsymbol{E} \parallel [010]$
$P2'/c$ (13.67) $\mathbb{Z}_1$	• strain $\perp [010]$
$P2'$ (3.3) $\mathbb{Z}_1$	• $\boldsymbol{B} \parallel [100]$ and strain $\perp [001]$<br>• $\boldsymbol{B} \parallel [010]$ and strain $\perp [001]$<br>• $\boldsymbol{B} \perp [001]$<br>• $\boldsymbol{E} \parallel [001]$ and strain $\perp [001]$<br>• $\boldsymbol{E} \parallel [001]$ and $\boldsymbol{B} \parallel [100]$
$Pn'n2'$ (34.158) $\mathbb{Z}_1$	• $\boldsymbol{B} \parallel [010]$<br>• $\boldsymbol{E} \parallel [001]$

Continued on next page

...continued

Subgroup & SI group	Perturbation
$P2'/c$ (13.67) $\mathbb{Z}_1$	• strain $\perp$ [001]
$P2'2'2$ (16.3) $\mathbb{Z}_2$	• $\boldsymbol{B} \parallel$ [100]

Table. S 370: Subgroups of $Pn'n'n$ (48.260) that can be realized by applying $\boldsymbol{E}/\boldsymbol{B}$ fields and/or uniaxial strains.

Subgroup & SI group	Perturbation
$P2'$ (3.3) $\mathbb{Z}_1$	• $\boldsymbol{E} \parallel$ [100] and strain $\perp$ [100]<br>• $\boldsymbol{E} \parallel$ [100] and ($\boldsymbol{B} \parallel$ [010] or $\boldsymbol{B} \perp$ [100])
Pc (7.24) $\mathbb{Z}_1$	• $\boldsymbol{E} \parallel$ [100] and strain $\perp$ [001]<br>• $\boldsymbol{E} \parallel$ [010] and strain $\perp$ [001]<br>• $\boldsymbol{E} \perp$ [001]
Pc' (7.26) $\mathbb{Z}_1$	• $\boldsymbol{E} \parallel$ [100] and strain $\perp$ [010]<br>• $\boldsymbol{E} \parallel$ [100] and ($\boldsymbol{B} \parallel$ [100] or $\boldsymbol{B} \perp$ [010])<br>• $\boldsymbol{E} \parallel$ [001] and strain $\perp$ [010]<br>• $\boldsymbol{E} \parallel$ [001] and ($\boldsymbol{B} \parallel$ [100] or $\boldsymbol{B} \perp$ [010])<br>• $\boldsymbol{E} \perp$ [010]
$Pn'n2'$ (34.158) $\mathbb{Z}_1$	• $\boldsymbol{E} \parallel$ [100]
Pc' (7.26) $\mathbb{Z}_1$	• $\boldsymbol{E} \parallel$ [010] and strain $\perp$ [100]<br>• $\boldsymbol{E} \parallel$ [010] and ($\boldsymbol{B} \parallel$ [010] or $\boldsymbol{B} \perp$ [100])<br>• $\boldsymbol{E} \parallel$ [001] and strain $\perp$ [100]<br>• $\boldsymbol{E} \parallel$ [001] and ($\boldsymbol{B} \parallel$ [010] or $\boldsymbol{B} \perp$ [100])<br>• $\boldsymbol{E} \perp$ [100]
$P\bar{1}$ (2.4) $\mathbb{Z}_2 \times \mathbb{Z}_2 \times \mathbb{Z}_2 \times \mathbb{Z}_4$	• strain in generic direction<br>• ($\boldsymbol{B} \parallel$ [100] or $\boldsymbol{B} \perp$ [010]) and strain $\perp$ [100]<br>• ($\boldsymbol{B} \parallel$ [100] or $\boldsymbol{B} \perp$ [010]) and strain $\perp$ [001]<br>• ($\boldsymbol{B} \parallel$ [010] or $\boldsymbol{B} \perp$ [100]) and strain $\perp$ [010]<br>• ($\boldsymbol{B} \parallel$ [010] or $\boldsymbol{B} \perp$ [100]) and strain $\perp$ [001]<br>• $\boldsymbol{B}$ in generic direction
$P2'/c'$ (13.69) $\mathbb{Z}_2 \times \mathbb{Z}_2 \times \mathbb{Z}_4$	• strain $\perp$ [100]<br>• ($\boldsymbol{B} \parallel$ [010] or $\boldsymbol{B} \perp$ [100])
$P2'$ (3.3) $\mathbb{Z}_1$	• $\boldsymbol{E} \parallel$ [010] and strain $\perp$ [010]<br>• $\boldsymbol{E} \parallel$ [010] and ($\boldsymbol{B} \parallel$ [100] or $\boldsymbol{B} \perp$ [010])
$Pn'n2'$ (34.158) $\mathbb{Z}_1$	• $\boldsymbol{E} \parallel$ [010]

Continued on next page

...continued

Subgroup & SI group	Perturbation
$P2'/c'$ (13.69) $\mathbb{Z}_2 \times \mathbb{Z}_2 \times \mathbb{Z}_4$	• strain $\perp$ [010]<br>• ($\boldsymbol{B} \parallel$ [100] or $\boldsymbol{B} \perp$ [010])
$P2$ (3.1) $\mathbb{Z}_2$	• $\boldsymbol{E} \parallel$ [001] and strain $\perp$ [001]
$Pn'n'2$ (34.159) $\mathbb{Z}_1$	• $\boldsymbol{E} \parallel$ [001]
$P2/c$ (13.65) $\mathbb{Z}_2 \times \mathbb{Z}_2$	• strain $\perp$ [001]

Table. S 371: Subgroups of $Pn'n'n'$ (48.261) that can be realized by applying $\boldsymbol{E}/\boldsymbol{B}$ fields and/or uniaxial strains.

Subgroup & SI group	Perturbation
$P2$ (3.1) $\mathbb{Z}_2$	• $\boldsymbol{B} \parallel$ [100] and strain $\perp$ [100]<br>• $\boldsymbol{E} \parallel$ [100] and strain $\perp$ [100]
Pc' (7.26) $\mathbb{Z}_1$	• $\boldsymbol{B} \parallel$ [100] and strain $\perp$ [001]<br>• $\boldsymbol{B} \parallel$ [010] and strain $\perp$ [001]<br>• $\boldsymbol{B} \perp$ [001]<br>• $\boldsymbol{E} \parallel$ [100] and strain $\perp$ [001]<br>• $\boldsymbol{E} \parallel$ [100] and $\boldsymbol{B} \parallel$ [010]<br>• $\boldsymbol{E} \parallel$ [010] and strain $\perp$ [001]<br>• $\boldsymbol{E} \parallel$ [010] and $\boldsymbol{B} \parallel$ [100]<br>• $\boldsymbol{E} \perp$ [001]
Pc' (7.26) $\mathbb{Z}_1$	• $\boldsymbol{B} \parallel$ [100] and strain $\perp$ [010]<br>• $\boldsymbol{B} \parallel$ [001] and strain $\perp$ [010]<br>• $\boldsymbol{B} \perp$ [010]<br>• $\boldsymbol{E} \parallel$ [100] and strain $\perp$ [010]<br>• $\boldsymbol{E} \parallel$ [100] and $\boldsymbol{B} \parallel$ [001]<br>• $\boldsymbol{E} \parallel$ [001] and strain $\perp$ [010]<br>• $\boldsymbol{E} \parallel$ [001] and $\boldsymbol{B} \parallel$ [100]<br>• $\boldsymbol{E} \perp$ [010]
$Pn'n'2$ (34.159) $\mathbb{Z}_1$	• $\boldsymbol{B} \parallel$ [100]<br>• $\boldsymbol{E} \parallel$ [100]

Continued on next page

...continued

Subgroup & SI group	Perturbation
Pc' (7.26) $\mathbb{Z}_1$	• $\boldsymbol{B}$ ∥ [010] and strain ⊥ [100]<br>• $\boldsymbol{B}$ ∥ [001] and strain ⊥ [100]<br>• $\boldsymbol{B}$ ⊥ [100]<br>• $\boldsymbol{E}$ ∥ [010] and strain ⊥ [100]<br>• $\boldsymbol{E}$ ∥ [010] and $\boldsymbol{B}$ ∥ [001]<br>• $\boldsymbol{E}$ ∥ [001] and strain ⊥ [100]<br>• $\boldsymbol{E}$ ∥ [001] and $\boldsymbol{B}$ ∥ [010]<br>• $\boldsymbol{E}$ ⊥ [100]
$P\bar{1}'$ (2.6) $\mathbb{Z}_1$	• strain in generic direction
$P2/c'$ (13.68) $\mathbb{Z}_2$	• strain ⊥ [100]
$P2$ (3.1) $\mathbb{Z}_2$	• $\boldsymbol{B}$ ∥ [010] and strain ⊥ [010]<br>• $\boldsymbol{E}$ ∥ [010] and strain ⊥ [010]
$Pn'n'2$ (34.159) $\mathbb{Z}_1$	• $\boldsymbol{B}$ ∥ [010]<br>• $\boldsymbol{E}$ ∥ [010]
$P2/c'$ (13.68) $\mathbb{Z}_2$	• strain ⊥ [010]
$P2$ (3.1) $\mathbb{Z}_2$	• $\boldsymbol{B}$ ∥ [001] and strain ⊥ [001]<br>• $\boldsymbol{E}$ ∥ [001] and strain ⊥ [001]
$Pn'n'2$ (34.159) $\mathbb{Z}_1$	• $\boldsymbol{B}$ ∥ [001]<br>• $\boldsymbol{E}$ ∥ [001]
$P2/c'$ (13.68) $\mathbb{Z}_2$	• strain ⊥ [001]

Table. S 372: Subgroups of P_cnnn (48.262) that can be realized by applying $\boldsymbol{E}/\boldsymbol{B}$ fields and/or uniaxial strains.

Subgroup & SI group	Perturbation

Continued on next page

...continued

Subgroup & SI group	Perturbation
P_S1 (1.3) $\mathbb{Z}_1$	• $\boldsymbol{E} \parallel [100]$ and strain in generic direction<br>• $\boldsymbol{E} \parallel [010]$ and strain in generic direction<br>• $\boldsymbol{E} \parallel [001]$ and strain in generic direction<br>• $\boldsymbol{E} \perp [100]$ and strain $\perp [010]$<br>• $\boldsymbol{E} \perp [100]$ and strain $\perp [001]$<br>• $\boldsymbol{E} \perp [100]$ and strain in generic direction<br>• $\boldsymbol{E} \perp [010]$ and strain $\perp [100]$<br>• $\boldsymbol{E} \perp [010]$ and strain $\perp [001]$<br>• $\boldsymbol{E} \perp [010]$ and strain in generic direction<br>• $\boldsymbol{E} \perp [001]$ and strain $\perp [100]$<br>• $\boldsymbol{E} \perp [001]$ and strain $\perp [010]$<br>• $\boldsymbol{E} \perp [001]$ and strain in generic direction<br>• $\boldsymbol{E}$ in generic direction
$P2'$ (3.3) $\mathbb{Z}_1$	• $\boldsymbol{E} \parallel [100]$ and $\boldsymbol{B} \parallel [010]$ and strain $\perp [100]$<br>• $\boldsymbol{E} \parallel [100]$ and $\boldsymbol{B} \parallel [001]$ and strain $\perp [100]$<br>• $\boldsymbol{E} \parallel [100]$ and $\boldsymbol{B} \perp [100]$
$P2$ (3.1) $\mathbb{Z}_2$	• $\boldsymbol{E} \parallel [100]$ and $\boldsymbol{B} \parallel [100]$ and strain $\perp [100]$
P_a2 (3.4) $\mathbb{Z}_2$	• $\boldsymbol{E} \parallel [100]$ and strain $\perp [100]$
Pc (7.24) $\mathbb{Z}_1$	• $\boldsymbol{E} \parallel [100]$ and $\boldsymbol{B} \parallel [001]$ and strain $\perp [001]$<br>• $\boldsymbol{E} \parallel [010]$ and $\boldsymbol{B} \parallel [001]$ and strain $\perp [001]$<br>• $\boldsymbol{E} \perp [001]$ and $\boldsymbol{B} \parallel [001]$
Pc' (7.26) $\mathbb{Z}_1$	• $\boldsymbol{E} \parallel [100]$ and $\boldsymbol{B} \parallel [100]$ and strain $\perp [001]$<br>• $\boldsymbol{E} \parallel [100]$ and $\boldsymbol{B} \parallel [010]$ and strain $\perp [001]$<br>• $\boldsymbol{E} \parallel [100]$ and $\boldsymbol{B} \perp [001]$<br>• $\boldsymbol{E} \parallel [010]$ and $\boldsymbol{B} \parallel [100]$ and strain $\perp [001]$<br>• $\boldsymbol{E} \parallel [010]$ and $\boldsymbol{B} \parallel [010]$ and strain $\perp [001]$<br>• $\boldsymbol{E} \parallel [010]$ and $\boldsymbol{B} \perp [001]$<br>• $\boldsymbol{E} \perp [001]$ and $\boldsymbol{B} \parallel [100]$<br>• $\boldsymbol{E} \perp [001]$ and $\boldsymbol{B} \parallel [010]$<br>• $\boldsymbol{E} \perp [001]$ and $\boldsymbol{B} \perp [001]$
P_bc (7.29) $\mathbb{Z}_1$	• $\boldsymbol{E} \parallel [100]$ and strain $\perp [001]$<br>• $\boldsymbol{E} \parallel [010]$ and strain $\perp [001]$<br>• $\boldsymbol{E} \perp [001]$

Continued on next page

...continued

Subgroup & SI group	Perturbation
Pc' (7.26) $\mathbb{Z}_1$	• $\boldsymbol{E} \parallel$ [100] and $\boldsymbol{B} \parallel$ [100] and strain $\perp$ [010]<br>• $\boldsymbol{E} \parallel$ [100] and $\boldsymbol{B} \parallel$ [001] and strain $\perp$ [010]<br>• $\boldsymbol{E} \parallel$ [100] and $\boldsymbol{B} \perp$ [010]<br>• $\boldsymbol{E} \parallel$ [001] and $\boldsymbol{B} \parallel$ [100] and strain $\perp$ [010]<br>• $\boldsymbol{E} \parallel$ [001] and $\boldsymbol{B} \parallel$ [001] and strain $\perp$ [010]<br>• $\boldsymbol{E} \parallel$ [001] and $\boldsymbol{B} \perp$ [010]<br>• $\boldsymbol{E} \perp$ [010] and $\boldsymbol{B} \parallel$ [100]<br>• $\boldsymbol{E} \perp$ [010] and $\boldsymbol{B} \parallel$ [001]<br>• $\boldsymbol{E} \perp$ [010] and $\boldsymbol{B} \perp$ [010]
$Pnc'2'$ (30.114) $\mathbb{Z}_1$	• $\boldsymbol{E} \parallel$ [100] and $\boldsymbol{B} \parallel$ [001]
$Pn'c'2$ (30.115) $\mathbb{Z}_1$	• $\boldsymbol{E} \parallel$ [100] and $\boldsymbol{B} \parallel$ [100]
Pc (7.24) $\mathbb{Z}_1$	• $\boldsymbol{E} \parallel$ [100] and $\boldsymbol{B} \parallel$ [010] and strain $\perp$ [010]<br>• $\boldsymbol{E} \parallel$ [001] and $\boldsymbol{B} \parallel$ [010] and strain $\perp$ [010]<br>• $\boldsymbol{E} \perp$ [010] and $\boldsymbol{B} \parallel$ [010]
$Pn'n2'$ (34.158) $\mathbb{Z}_1$	• $\boldsymbol{E} \parallel$ [100] and $\boldsymbol{B} \parallel$ [010]
P_ac (7.27) $\mathbb{Z}_1$	• $\boldsymbol{E} \parallel$ [100] and strain $\perp$ [010]<br>• $\boldsymbol{E} \parallel$ [001] and strain $\perp$ [010]<br>• $\boldsymbol{E} \perp$ [010]
P_ann2 (34.160) $\mathbb{Z}_1$	• $\boldsymbol{E} \parallel$ [100]
Pc' (7.26) $\mathbb{Z}_1$	• $\boldsymbol{E} \parallel$ [010] and $\boldsymbol{B} \parallel$ [010] and strain $\perp$ [100]<br>• $\boldsymbol{E} \parallel$ [010] and $\boldsymbol{B} \parallel$ [001] and strain $\perp$ [100]<br>• $\boldsymbol{E} \parallel$ [010] and $\boldsymbol{B} \perp$ [100]<br>• $\boldsymbol{E} \parallel$ [001] and $\boldsymbol{B} \parallel$ [010] and strain $\perp$ [100]<br>• $\boldsymbol{E} \parallel$ [001] and $\boldsymbol{B} \parallel$ [001] and strain $\perp$ [100]<br>• $\boldsymbol{E} \parallel$ [001] and $\boldsymbol{B} \perp$ [100]<br>• $\boldsymbol{E} \perp$ [100] and $\boldsymbol{B} \parallel$ [010]<br>• $\boldsymbol{E} \perp$ [100] and $\boldsymbol{B} \parallel$ [001]<br>• $\boldsymbol{E} \perp$ [100] and $\boldsymbol{B} \perp$ [100]
Pc (7.24) $\mathbb{Z}_1$	• $\boldsymbol{E} \parallel$ [010] and $\boldsymbol{B} \parallel$ [100] and strain $\perp$ [100]<br>• $\boldsymbol{E} \parallel$ [001] and $\boldsymbol{B} \parallel$ [100] and strain $\perp$ [100]<br>• $\boldsymbol{E} \perp$ [100] and $\boldsymbol{B} \parallel$ [100]
P_ac (7.27) $\mathbb{Z}_1$	• $\boldsymbol{E} \parallel$ [010] and strain $\perp$ [100]<br>• $\boldsymbol{E} \parallel$ [001] and strain $\perp$ [100]<br>• $\boldsymbol{E} \perp$ [100]

Continued on next page

…continued

Subgroup & SI group	Perturbation
$P\bar{1}$ (2.4) $\mathbb{Z}_2 \times \mathbb{Z}_2 \times \mathbb{Z}_2 \times \mathbb{Z}_4$	• $\boldsymbol{B} \parallel [100]$ and strain in generic direction<br>• $\boldsymbol{B} \parallel [010]$ and strain in generic direction<br>• $\boldsymbol{B} \parallel [001]$ and strain in generic direction<br>• $\boldsymbol{B} \perp [100]$ and strain $\perp [010]$<br>• $\boldsymbol{B} \perp [100]$ and strain $\perp [001]$<br>• $\boldsymbol{B} \perp [100]$ and strain in generic direction<br>• $\boldsymbol{B} \perp [010]$ and strain $\perp [100]$<br>• $\boldsymbol{B} \perp [010]$ and strain $\perp [001]$<br>• $\boldsymbol{B} \perp [010]$ and strain in generic direction<br>• $\boldsymbol{B} \perp [001]$ and strain $\perp [100]$<br>• $\boldsymbol{B} \perp [001]$ and strain $\perp [010]$<br>• $\boldsymbol{B} \perp [001]$ and strain in generic direction<br>• $\boldsymbol{B}$ in generic direction
$P2'/c'$ (13.69) $\mathbb{Z}_2 \times \mathbb{Z}_2 \times \mathbb{Z}_4$	• $\boldsymbol{B} \parallel [010]$ and strain $\perp [100]$<br>• $\boldsymbol{B} \parallel [001]$ and strain $\perp [100]$<br>• $\boldsymbol{B} \perp [100]$
$P2/c$ (13.65) $\mathbb{Z}_2 \times \mathbb{Z}_2$	• $\boldsymbol{B} \parallel [100]$ and strain $\perp [100]$
$P_S\bar{1}$ (2.7) $\mathbb{Z}_2 \times \mathbb{Z}_2 \times \mathbb{Z}_4$	• strain in generic direction
P_a2/c (13.70) $\mathbb{Z}_2 \times \mathbb{Z}_2$	• strain $\perp [100]$
$P2'$ (3.3) $\mathbb{Z}_1$	• $\boldsymbol{E} \parallel [010]$ and $\boldsymbol{B} \parallel [100]$ and strain $\perp [010]$<br>• $\boldsymbol{E} \parallel [010]$ and $\boldsymbol{B} \parallel [001]$ and strain $\perp [010]$<br>• $\boldsymbol{E} \parallel [010]$ and $\boldsymbol{B} \perp [010]$
$Pnc'2'$ (30.114) $\mathbb{Z}_1$	• $\boldsymbol{E} \parallel [010]$ and $\boldsymbol{B} \parallel [001]$
$Pn'n2'$ (34.158) $\mathbb{Z}_1$	• $\boldsymbol{E} \parallel [010]$ and $\boldsymbol{B} \parallel [100]$
$P2'/c'$ (13.69) $\mathbb{Z}_2 \times \mathbb{Z}_2 \times \mathbb{Z}_4$	• $\boldsymbol{B} \parallel [100]$ and strain $\perp [010]$<br>• $\boldsymbol{B} \parallel [001]$ and strain $\perp [010]$<br>• $\boldsymbol{B} \perp [010]$
$P2$ (3.1) $\mathbb{Z}_2$	• $\boldsymbol{E} \parallel [010]$ and $\boldsymbol{B} \parallel [010]$ and strain $\perp [010]$
$Pn'c'2$ (30.115) $\mathbb{Z}_1$	• $\boldsymbol{E} \parallel [010]$ and $\boldsymbol{B} \parallel [010]$
$P2/c$ (13.65) $\mathbb{Z}_2 \times \mathbb{Z}_2$	• $\boldsymbol{B} \parallel [010]$ and strain $\perp [010]$
P_a2 (3.4) $\mathbb{Z}_2$	• $\boldsymbol{E} \parallel [010]$ and strain $\perp [010]$
P_ann2 (34.160) $\mathbb{Z}_1$	• $\boldsymbol{E} \parallel [010]$
P_a2/c (13.70) $\mathbb{Z}_2 \times \mathbb{Z}_2$	• strain $\perp [010]$
$P2$ (3.1) $\mathbb{Z}_2$	• $\boldsymbol{E} \parallel [001]$ and $\boldsymbol{B} \parallel [001]$ and strain $\perp [001]$

Continued on next page

...continued

Subgroup & SI group	Perturbation
$Pb'a'2$ (32.138) $\mathbb{Z}_2$	• $\boldsymbol{E}$ ∥ [001] and $\boldsymbol{B}$ ∥ [001]
$P2/c$ (13.65) $\mathbb{Z}_2 \times \mathbb{Z}_2$	• $\boldsymbol{B}$ ∥ [001] and strain ⊥ [001]
$Pb'a'n$ (50.281) $\mathbb{Z}_2 \times \mathbb{Z}_2$	• $\boldsymbol{B}$ ∥ [001]
$P2_1'$ (4.9) $\mathbb{Z}_1$	• $\boldsymbol{E}$ ∥ [001] and $\boldsymbol{B}$ ∥ [100] and strain ⊥ [001]<br>• $\boldsymbol{E}$ ∥ [001] and $\boldsymbol{B}$ ∥ [010] and strain ⊥ [001]<br>• $\boldsymbol{E}$ ∥ [001] and $\boldsymbol{B}$ ⊥ [001]
$Pna'2_1'$ (33.147) $\mathbb{Z}_1$	• $\boldsymbol{E}$ ∥ [001] and $\boldsymbol{B}$ ∥ [010]
$Pna'2_1'$ (33.147) $\mathbb{Z}_1$	• $\boldsymbol{E}$ ∥ [001] and $\boldsymbol{B}$ ∥ [100]
$P2_1'/c'$ (14.79) $\mathbb{Z}_2 \times \mathbb{Z}_4$	• $\boldsymbol{B}$ ∥ [100] and strain ⊥ [001]<br>• $\boldsymbol{B}$ ∥ [010] and strain ⊥ [001]<br>• $\boldsymbol{B}$ ⊥ [001]
$Pnn'a'$ (52.311) $\mathbb{Z}_2$	• $\boldsymbol{B}$ ∥ [100]
$Pnn'a'$ (52.311) $\mathbb{Z}_2$	• $\boldsymbol{B}$ ∥ [010]
P_b2 (3.5) $\mathbb{Z}_2$	• $\boldsymbol{E}$ ∥ [001] and strain ⊥ [001]
P_cnn2 (34.161) $\mathbb{Z}_2$	• $\boldsymbol{E}$ ∥ [001]
P_b2/c (13.71) $\mathbb{Z}_2 \times \mathbb{Z}_2$	• strain ⊥ [001]

Table. S 373: Subgroups of P_Cnnn (48.263) that can be realized by applying $\boldsymbol{E}/\boldsymbol{B}$ fields and/or uniaxial strains.

Subgroup & SI group	Perturbation
Pm' (6.20) $\mathbb{Z}_1$	• $\boldsymbol{E}$ ∥ [100] and $\boldsymbol{B}$ ∥ [100] and strain ⊥ [001]<br>• $\boldsymbol{E}$ ∥ [100] and $\boldsymbol{B}$ ∥ [010] and strain ⊥ [001]<br>• $\boldsymbol{E}$ ∥ [100] and $\boldsymbol{B}$ ⊥ [001]<br>• $\boldsymbol{E}$ ∥ [010] and $\boldsymbol{B}$ ∥ [100] and strain ⊥ [001]<br>• $\boldsymbol{E}$ ∥ [010] and $\boldsymbol{B}$ ∥ [010] and strain ⊥ [001]<br>• $\boldsymbol{E}$ ∥ [010] and $\boldsymbol{B}$ ⊥ [001]<br>• $\boldsymbol{E}$ ⊥ [001] and $\boldsymbol{B}$ ∥ [100]<br>• $\boldsymbol{E}$ ⊥ [001] and $\boldsymbol{B}$ ∥ [010]<br>• $\boldsymbol{E}$ ⊥ [001] and $\boldsymbol{B}$ ⊥ [001]

Continued on next page

...continued

Subgroup & SI group	Perturbation
Pc' (7.26) $\mathbb{Z}_1$	• $\boldsymbol{E}$ ∥ [100] and $\boldsymbol{B}$ ∥ [100] and strain ⊥ [010]<br>• $\boldsymbol{E}$ ∥ [100] and $\boldsymbol{B}$ ∥ [001] and strain ⊥ [010]<br>• $\boldsymbol{E}$ ∥ [100] and $\boldsymbol{B}$ ⊥ [010]<br>• $\boldsymbol{E}$ ∥ [001] and $\boldsymbol{B}$ ∥ [100] and strain ⊥ [010]<br>• $\boldsymbol{E}$ ∥ [001] and $\boldsymbol{B}$ ∥ [001] and strain ⊥ [010]<br>• $\boldsymbol{E}$ ∥ [001] and $\boldsymbol{B}$ ⊥ [010]<br>• $\boldsymbol{E}$ ⊥ [010] and $\boldsymbol{B}$ ∥ [100]<br>• $\boldsymbol{E}$ ⊥ [010] and $\boldsymbol{B}$ ∥ [001]<br>• $\boldsymbol{E}$ ⊥ [010] and $\boldsymbol{B}$ ⊥ [010]
$P2$ (3.1) $\mathbb{Z}_2$	• $\boldsymbol{E}$ ∥ [100] and $\boldsymbol{B}$ ∥ [100] and strain ⊥ [100]
$Pm'a'2$ (28.91) $\mathbb{Z}_2$	• $\boldsymbol{E}$ ∥ [100] and $\boldsymbol{B}$ ∥ [100]
P_S1 (1.3) $\mathbb{Z}_1$	• $\boldsymbol{E}$ ∥ [100] and strain in generic direction<br>• $\boldsymbol{E}$ ∥ [010] and strain in generic direction<br>• $\boldsymbol{E}$ ∥ [001] and strain in generic direction<br>• $\boldsymbol{E}$ ⊥ [100] and strain ⊥ [010]<br>• $\boldsymbol{E}$ ⊥ [100] and strain ⊥ [001]<br>• $\boldsymbol{E}$ ⊥ [100] and strain in generic direction<br>• $\boldsymbol{E}$ ⊥ [010] and strain ⊥ [100]<br>• $\boldsymbol{E}$ ⊥ [010] and strain ⊥ [001]<br>• $\boldsymbol{E}$ ⊥ [010] and strain in generic direction<br>• $\boldsymbol{E}$ ⊥ [001] and strain ⊥ [100]<br>• $\boldsymbol{E}$ ⊥ [001] and strain ⊥ [010]<br>• $\boldsymbol{E}$ ⊥ [001] and strain in generic direction<br>• $\boldsymbol{E}$ in generic direction
Pc (7.24) $\mathbb{Z}_1$	• $\boldsymbol{E}$ ∥ [100] and $\boldsymbol{B}$ ∥ [001] and strain ⊥ [001]<br>• $\boldsymbol{E}$ ∥ [010] and $\boldsymbol{B}$ ∥ [001] and strain ⊥ [001]<br>• $\boldsymbol{E}$ ⊥ [001] and $\boldsymbol{B}$ ∥ [001]
P_cc (7.28) $\mathbb{Z}_1$	• $\boldsymbol{E}$ ∥ [100] and strain ⊥ [001]<br>• $\boldsymbol{E}$ ∥ [010] and strain ⊥ [001]<br>• $\boldsymbol{E}$ ⊥ [001]
Pc (7.24) $\mathbb{Z}_1$	• $\boldsymbol{E}$ ∥ [100] and $\boldsymbol{B}$ ∥ [010] and strain ⊥ [010]<br>• $\boldsymbol{E}$ ∥ [001] and $\boldsymbol{B}$ ∥ [010] and strain ⊥ [010]<br>• $\boldsymbol{E}$ ⊥ [010] and $\boldsymbol{B}$ ∥ [010]
P_Cc (7.30) $\mathbb{Z}_1$	• $\boldsymbol{E}$ ∥ [100] and strain ⊥ [010]<br>• $\boldsymbol{E}$ ∥ [001] and strain ⊥ [010]<br>• $\boldsymbol{E}$ ⊥ [010]

Continued on next page

…continued

Subgroup & SI group	Perturbation
$P2_1'$ (4.9) $\mathbb{Z}_1$	• $\boldsymbol{E} \parallel [100]$ and $\boldsymbol{B} \parallel [010]$ and strain $\perp [100]$<br>• $\boldsymbol{E} \parallel [100]$ and $\boldsymbol{B} \parallel [001]$ and strain $\perp [100]$<br>• $\boldsymbol{E} \parallel [100]$ and $\boldsymbol{B} \perp [100]$
P_C2 (3.6) $\mathbb{Z}_1$	• $\boldsymbol{E} \parallel [100]$ and strain $\perp [100]$
$Pna'2_1'$ (33.147) $\mathbb{Z}_1$	• $\boldsymbol{E} \parallel [100]$ and $\boldsymbol{B} \parallel [001]$
$Pm'n2_1'$ (31.125) $\mathbb{Z}_1$	• $\boldsymbol{E} \parallel [100]$ and $\boldsymbol{B} \parallel [010]$
P_Ann2 (34.162) $\mathbb{Z}_1$	• $\boldsymbol{E} \parallel [100]$
Pc (7.24) $\mathbb{Z}_1$	• $\boldsymbol{E} \parallel [010]$ and $\boldsymbol{B} \parallel [100]$ and strain $\perp [100]$<br>• $\boldsymbol{E} \parallel [001]$ and $\boldsymbol{B} \parallel [100]$ and strain $\perp [100]$<br>• $\boldsymbol{E} \perp [100]$ and $\boldsymbol{B} \parallel [100]$
$P2_1'$ (4.9) $\mathbb{Z}_1$	• $\boldsymbol{E} \parallel [010]$ and $\boldsymbol{B} \parallel [100]$ and strain $\perp [010]$<br>• $\boldsymbol{E} \parallel [010]$ and $\boldsymbol{B} \parallel [001]$ and strain $\perp [010]$<br>• $\boldsymbol{E} \parallel [010]$ and $\boldsymbol{B} \perp [010]$
$Pm'n2_1'$ (31.125) $\mathbb{Z}_1$	• $\boldsymbol{E} \parallel [010]$ and $\boldsymbol{B} \parallel [100]$
$P2'$ (3.3) $\mathbb{Z}_1$	• $\boldsymbol{E} \parallel [001]$ and $\boldsymbol{B} \parallel [100]$ and strain $\perp [001]$<br>• $\boldsymbol{E} \parallel [001]$ and $\boldsymbol{B} \parallel [010]$ and strain $\perp [001]$<br>• $\boldsymbol{E} \parallel [001]$ and $\boldsymbol{B} \perp [001]$
$Pnc'2'$ (30.114) $\mathbb{Z}_1$	• $\boldsymbol{E} \parallel [001]$ and $\boldsymbol{B} \parallel [100]$
$P\bar{1}$ (2.4) $\mathbb{Z}_2 \times \mathbb{Z}_2 \times \mathbb{Z}_2 \times \mathbb{Z}_4$	• $\boldsymbol{B} \parallel [100]$ and strain in generic direction<br>• $\boldsymbol{B} \parallel [010]$ and strain in generic direction<br>• $\boldsymbol{B} \parallel [001]$ and strain in generic direction<br>• $\boldsymbol{B} \perp [100]$ and strain $\perp [010]$<br>• $\boldsymbol{B} \perp [100]$ and strain $\perp [001]$<br>• $\boldsymbol{B} \perp [100]$ and strain in generic direction<br>• $\boldsymbol{B} \perp [010]$ and strain $\perp [100]$<br>• $\boldsymbol{B} \perp [010]$ and strain $\perp [001]$<br>• $\boldsymbol{B} \perp [010]$ and strain in generic direction<br>• $\boldsymbol{B} \perp [001]$ and strain $\perp [100]$<br>• $\boldsymbol{B} \perp [001]$ and strain $\perp [010]$<br>• $\boldsymbol{B} \perp [001]$ and strain in generic direction<br>• $\boldsymbol{B}$ in generic direction
$P2/c$ (13.65) $\mathbb{Z}_2 \times \mathbb{Z}_2$	• $\boldsymbol{B} \parallel [100]$ and strain $\perp [100]$
$P2_1'/c'$ (14.79) $\mathbb{Z}_2 \times \mathbb{Z}_4$	• $\boldsymbol{B} \parallel [100]$ and strain $\perp [010]$<br>• $\boldsymbol{B} \parallel [001]$ and strain $\perp [010]$<br>• $\boldsymbol{B} \perp [010]$

Continued on next page

...continued

Subgroup & SI group	Perturbation
$P2'/m'$ (10.46) $\mathbb{Z}_2 \times \mathbb{Z}_2 \times \mathbb{Z}_2 \times \mathbb{Z}_4$	• $\boldsymbol{B} \parallel [100]$ and strain $\perp [001]$<br>• $\boldsymbol{B} \parallel [010]$ and strain $\perp [001]$<br>• $\boldsymbol{B} \perp [001]$
$Pm'na'$ (53.328) $\mathbb{Z}_2 \times \mathbb{Z}_2$	• $\boldsymbol{B} \parallel [100]$
Pc' (7.26) $\mathbb{Z}_1$	• $\boldsymbol{E} \parallel [010]$ and $\boldsymbol{B} \parallel [010]$ and strain $\perp [100]$<br>• $\boldsymbol{E} \parallel [010]$ and $\boldsymbol{B} \parallel [001]$ and strain $\perp [100]$<br>• $\boldsymbol{E} \parallel [010]$ and $\boldsymbol{B} \perp [100]$<br>• $\boldsymbol{E} \parallel [001]$ and $\boldsymbol{B} \parallel [010]$ and strain $\perp [100]$<br>• $\boldsymbol{E} \parallel [001]$ and $\boldsymbol{B} \parallel [001]$ and strain $\perp [100]$<br>• $\boldsymbol{E} \parallel [001]$ and $\boldsymbol{B} \perp [100]$<br>• $\boldsymbol{E} \perp [100]$ and $\boldsymbol{B} \parallel [010]$<br>• $\boldsymbol{E} \perp [100]$ and $\boldsymbol{B} \parallel [001]$<br>• $\boldsymbol{E} \perp [100]$ and $\boldsymbol{B} \perp [100]$
P_Cc (7.30) $\mathbb{Z}_1$	• $\boldsymbol{E} \parallel [010]$ and strain $\perp [100]$<br>• $\boldsymbol{E} \parallel [001]$ and strain $\perp [100]$<br>• $\boldsymbol{E} \perp [100]$
$Pna'2_1'$ (33.147) $\mathbb{Z}_1$	• $\boldsymbol{E} \parallel [010]$ and $\boldsymbol{B} \parallel [001]$
$Pnc'2'$ (30.114) $\mathbb{Z}_1$	• $\boldsymbol{E} \parallel [001]$ and $\boldsymbol{B} \parallel [010]$
$P2_1'/c'$ (14.79) $\mathbb{Z}_2 \times \mathbb{Z}_4$	• $\boldsymbol{B} \parallel [010]$ and strain $\perp [100]$<br>• $\boldsymbol{B} \parallel [001]$ and strain $\perp [100]$<br>• $\boldsymbol{B} \perp [100]$
$P2$ (3.1) $\mathbb{Z}_2$	• $\boldsymbol{E} \parallel [010]$ and $\boldsymbol{B} \parallel [010]$ and strain $\perp [010]$
P_C2 (3.6) $\mathbb{Z}_1$	• $\boldsymbol{E} \parallel [010]$ and strain $\perp [010]$
$P2/c$ (13.65) $\mathbb{Z}_2 \times \mathbb{Z}_2$	• $\boldsymbol{B} \parallel [010]$ and strain $\perp [010]$
$Pm'a'2$ (28.91) $\mathbb{Z}_2$	• $\boldsymbol{E} \parallel [010]$ and $\boldsymbol{B} \parallel [010]$
P_Ann2 (34.162) $\mathbb{Z}_1$	• $\boldsymbol{E} \parallel [010]$
$Pm'na'$ (53.328) $\mathbb{Z}_2 \times \mathbb{Z}_2$	• $\boldsymbol{B} \parallel [010]$
$P2$ (3.1) $\mathbb{Z}_2$	• $\boldsymbol{E} \parallel [001]$ and $\boldsymbol{B} \parallel [001]$ and strain $\perp [001]$
P_a2 (3.4) $\mathbb{Z}_2$	• $\boldsymbol{E} \parallel [001]$ and strain $\perp [001]$
$P2/c$ (13.65) $\mathbb{Z}_2 \times \mathbb{Z}_2$	• $\boldsymbol{B} \parallel [001]$ and strain $\perp [001]$
$Pc'c'2$ (27.81) $\mathbb{Z}_2$	• $\boldsymbol{E} \parallel [001]$ and $\boldsymbol{B} \parallel [001]$
P_Cnn2 (34.163) $\mathbb{Z}_2$	• $\boldsymbol{E} \parallel [001]$
$Pc'c'n$ (56.369) $\mathbb{Z}_2 \times \mathbb{Z}_2$	• $\boldsymbol{B} \parallel [001]$

Continued on next page

. . . continued

Subgroup & SI group	Perturbation
$P_S\bar{1}$ (2.7) $\mathbb{Z}_2 \times \mathbb{Z}_2 \times \mathbb{Z}_4$	• strain in generic direction
P_C2/c (13.74) $\mathbb{Z}_2$	• strain $\perp$ [100]
P_C2/c (13.74) $\mathbb{Z}_2$	• strain $\perp$ [010]
P_c2/c (13.72) $\mathbb{Z}_2 \times \mathbb{Z}_2$	• strain $\perp$ [001]

Table. S 374: Subgroups of P_Innn (48.264) that can be realized by applying $\boldsymbol{E}/\boldsymbol{B}$ fields and/or uniaxial strains.

Subgroup & SI group	Perturbation
Pm' (6.20) $\mathbb{Z}_1$	• $\boldsymbol{E}$ || [100] and $\boldsymbol{B}$ || [100] and strain $\perp$ [001]<br>• $\boldsymbol{E}$ || [100] and $\boldsymbol{B}$ || [010] and strain $\perp$ [001]<br>• $\boldsymbol{E}$ || [100] and $\boldsymbol{B}$ $\perp$ [001]<br>• $\boldsymbol{E}$ || [010] and $\boldsymbol{B}$ || [100] and strain $\perp$ [001]<br>• $\boldsymbol{E}$ || [010] and $\boldsymbol{B}$ || [010] and strain $\perp$ [001]<br>• $\boldsymbol{E}$ || [010] and $\boldsymbol{B}$ $\perp$ [001]<br>• $\boldsymbol{E}$ $\perp$ [001] and $\boldsymbol{B}$ || [100]<br>• $\boldsymbol{E}$ $\perp$ [001] and $\boldsymbol{B}$ || [010]<br>• $\boldsymbol{E}$ $\perp$ [001] and $\boldsymbol{B}$ $\perp$ [001]
Pm' (6.20) $\mathbb{Z}_1$	• $\boldsymbol{E}$ || [100] and $\boldsymbol{B}$ || [100] and strain $\perp$ [010]<br>• $\boldsymbol{E}$ || [100] and $\boldsymbol{B}$ || [001] and strain $\perp$ [010]<br>• $\boldsymbol{E}$ || [100] and $\boldsymbol{B}$ $\perp$ [010]<br>• $\boldsymbol{E}$ || [001] and $\boldsymbol{B}$ || [100] and strain $\perp$ [010]<br>• $\boldsymbol{E}$ || [001] and $\boldsymbol{B}$ || [001] and strain $\perp$ [010]<br>• $\boldsymbol{E}$ || [001] and $\boldsymbol{B}$ $\perp$ [010]<br>• $\boldsymbol{E}$ $\perp$ [010] and $\boldsymbol{B}$ || [100]<br>• $\boldsymbol{E}$ $\perp$ [010] and $\boldsymbol{B}$ || [001]<br>• $\boldsymbol{E}$ $\perp$ [010] and $\boldsymbol{B}$ $\perp$ [010]
$P2$ (3.1) $\mathbb{Z}_2$	• $\boldsymbol{E}$ || [100] and $\boldsymbol{B}$ || [100] and strain $\perp$ [100]
$Pm'm'2$ (25.60) $\mathbb{Z}_2$	• $\boldsymbol{E}$ || [100] and $\boldsymbol{B}$ || [100]

Continued on next page

. . . continued

Subgroup & SI group	Perturbation
P_S1 (1.3) $\mathbb{Z}_1$	• $\boldsymbol{E}$ ∥ [100] and strain in generic direction<br>• $\boldsymbol{E}$ ∥ [010] and strain in generic direction<br>• $\boldsymbol{E}$ ∥ [001] and strain in generic direction<br>• $\boldsymbol{E} \perp$ [100] and strain $\perp$ [010]<br>• $\boldsymbol{E} \perp$ [100] and strain $\perp$ [001]<br>• $\boldsymbol{E} \perp$ [100] and strain in generic direction<br>• $\boldsymbol{E} \perp$ [010] and strain $\perp$ [100]<br>• $\boldsymbol{E} \perp$ [010] and strain $\perp$ [001]<br>• $\boldsymbol{E} \perp$ [010] and strain in generic direction<br>• $\boldsymbol{E} \perp$ [001] and strain $\perp$ [100]<br>• $\boldsymbol{E} \perp$ [001] and strain $\perp$ [010]<br>• $\boldsymbol{E} \perp$ [001] and strain in generic direction<br>• $\boldsymbol{E}$ in generic direction
Pc (7.24) $\mathbb{Z}_1$	• $\boldsymbol{E}$ ∥ [100] and $\boldsymbol{B}$ ∥ [001] and strain $\perp$ [001]<br>• $\boldsymbol{E}$ ∥ [010] and $\boldsymbol{B}$ ∥ [001] and strain $\perp$ [001]<br>• $\boldsymbol{E} \perp$ [001] and $\boldsymbol{B}$ ∥ [001]
P_Ac (7.31) $\mathbb{Z}_1$	• $\boldsymbol{E}$ ∥ [100] and strain $\perp$ [001]<br>• $\boldsymbol{E}$ ∥ [010] and strain $\perp$ [001]<br>• $\boldsymbol{E} \perp$ [001]
Pc (7.24) $\mathbb{Z}_1$	• $\boldsymbol{E}$ ∥ [100] and $\boldsymbol{B}$ ∥ [010] and strain $\perp$ [010]<br>• $\boldsymbol{E}$ ∥ [001] and $\boldsymbol{B}$ ∥ [010] and strain $\perp$ [010]<br>• $\boldsymbol{E} \perp$ [010] and $\boldsymbol{B}$ ∥ [010]
P_Ac (7.31) $\mathbb{Z}_1$	• $\boldsymbol{E}$ ∥ [100] and strain $\perp$ [010]<br>• $\boldsymbol{E}$ ∥ [001] and strain $\perp$ [010]<br>• $\boldsymbol{E} \perp$ [010]
$P2_1'$ (4.9) $\mathbb{Z}_1$	• $\boldsymbol{E}$ ∥ [100] and $\boldsymbol{B}$ ∥ [010] and strain $\perp$ [100]<br>• $\boldsymbol{E}$ ∥ [100] and $\boldsymbol{B}$ ∥ [001] and strain $\perp$ [100]<br>• $\boldsymbol{E}$ ∥ [100] and $\boldsymbol{B} \perp$ [100]
P_C2 (3.6) $\mathbb{Z}_1$	• $\boldsymbol{E}$ ∥ [100] and strain $\perp$ [100]
$Pm'n2_1'$ (31.125) $\mathbb{Z}_1$	• $\boldsymbol{E}$ ∥ [100] and $\boldsymbol{B}$ ∥ [001]
$Pm'n2_1'$ (31.125) $\mathbb{Z}_1$	• $\boldsymbol{E}$ ∥ [100] and $\boldsymbol{B}$ ∥ [010]
P_Inn2 (34.164) $\mathbb{Z}_1$	• $\boldsymbol{E}$ ∥ [100]
Pc (7.24) $\mathbb{Z}_1$	• $\boldsymbol{E}$ ∥ [010] and $\boldsymbol{B}$ ∥ [100] and strain $\perp$ [100]<br>• $\boldsymbol{E}$ ∥ [001] and $\boldsymbol{B}$ ∥ [100] and strain $\perp$ [100]<br>• $\boldsymbol{E} \perp$ [100] and $\boldsymbol{B}$ ∥ [100]

Continued on next page

. . . continued

Subgroup & SI group	Perturbation
$P2_1'$ (4.9) $\mathbb{Z}_1$	• $\boldsymbol{E} \parallel [010]$ and $\boldsymbol{B} \parallel [100]$ and strain $\perp [010]$<br>• $\boldsymbol{E} \parallel [010]$ and $\boldsymbol{B} \parallel [001]$ and strain $\perp [010]$<br>• $\boldsymbol{E} \parallel [010]$ and $\boldsymbol{B} \perp [010]$
$Pm'n2_1'$ (31.125) $\mathbb{Z}_1$	• $\boldsymbol{E} \parallel [010]$ and $\boldsymbol{B} \parallel [100]$
$P2_1'$ (4.9) $\mathbb{Z}_1$	• $\boldsymbol{E} \parallel [001]$ and $\boldsymbol{B} \parallel [100]$ and strain $\perp [001]$<br>• $\boldsymbol{E} \parallel [001]$ and $\boldsymbol{B} \parallel [010]$ and strain $\perp [001]$<br>• $\boldsymbol{E} \parallel [001]$ and $\boldsymbol{B} \perp [001]$
$Pm'n2_1'$ (31.125) $\mathbb{Z}_1$	• $\boldsymbol{E} \parallel [001]$ and $\boldsymbol{B} \parallel [100]$
$P\bar{1}$ (2.4) $\mathbb{Z}_2 \times \mathbb{Z}_2 \times \mathbb{Z}_2 \times \mathbb{Z}_4$	• $\boldsymbol{B} \parallel [100]$ and strain in generic direction<br>• $\boldsymbol{B} \parallel [010]$ and strain in generic direction<br>• $\boldsymbol{B} \parallel [001]$ and strain in generic direction<br>• $\boldsymbol{B} \perp [100]$ and strain $\perp [010]$<br>• $\boldsymbol{B} \perp [100]$ and strain $\perp [001]$<br>• $\boldsymbol{B} \perp [100]$ and strain in generic direction<br>• $\boldsymbol{B} \perp [010]$ and strain $\perp [100]$<br>• $\boldsymbol{B} \perp [010]$ and strain $\perp [001]$<br>• $\boldsymbol{B} \perp [010]$ and strain in generic direction<br>• $\boldsymbol{B} \perp [001]$ and strain $\perp [100]$<br>• $\boldsymbol{B} \perp [001]$ and strain $\perp [010]$<br>• $\boldsymbol{B} \perp [001]$ and strain in generic direction<br>• $\boldsymbol{B}$ in generic direction
$P2/c$ (13.65) $\mathbb{Z}_2 \times \mathbb{Z}_2$	• $\boldsymbol{B} \parallel [100]$ and strain $\perp [100]$
$P2_1'/m'$ (11.54) $\mathbb{Z}_2 \times \mathbb{Z}_2 \times \mathbb{Z}_4$	• $\boldsymbol{B} \parallel [100]$ and strain $\perp [010]$<br>• $\boldsymbol{B} \parallel [001]$ and strain $\perp [010]$<br>• $\boldsymbol{B} \perp [010]$
$P2_1'/m'$ (11.54) $\mathbb{Z}_2 \times \mathbb{Z}_2 \times \mathbb{Z}_4$	• $\boldsymbol{B} \parallel [100]$ and strain $\perp [001]$<br>• $\boldsymbol{B} \parallel [010]$ and strain $\perp [001]$<br>• $\boldsymbol{B} \perp [001]$
$Pm'm'n$ (59.409) $\mathbb{Z}_2 \times \mathbb{Z}_2$	• $\boldsymbol{B} \parallel [100]$

Continued on next page

. . . continued

Subgroup & SI group	Perturbation
Pm' (6.20) $\mathbb{Z}_1$	• $\boldsymbol{E} \parallel [010]$ and $\boldsymbol{B} \parallel [010]$ and strain $\perp [100]$<br>• $\boldsymbol{E} \parallel [010]$ and $\boldsymbol{B} \parallel [001]$ and strain $\perp [100]$<br>• $\boldsymbol{E} \parallel [010]$ and $\boldsymbol{B} \perp [100]$<br>• $\boldsymbol{E} \parallel [001]$ and $\boldsymbol{B} \parallel [010]$ and strain $\perp [100]$<br>• $\boldsymbol{E} \parallel [001]$ and $\boldsymbol{B} \parallel [001]$ and strain $\perp [100]$<br>• $\boldsymbol{E} \parallel [001]$ and $\boldsymbol{B} \perp [100]$<br>• $\boldsymbol{E} \perp [100]$ and $\boldsymbol{B} \parallel [010]$<br>• $\boldsymbol{E} \perp [100]$ and $\boldsymbol{B} \parallel [001]$<br>• $\boldsymbol{E} \perp [100]$ and $\boldsymbol{B} \perp [100]$
P_Ac (7.31) $\mathbb{Z}_1$	• $\boldsymbol{E} \parallel [010]$ and strain $\perp [100]$<br>• $\boldsymbol{E} \parallel [001]$ and strain $\perp [100]$<br>• $\boldsymbol{E} \perp [100]$
$Pm'n2_1'$ (31.125) $\mathbb{Z}_1$	• $\boldsymbol{E} \parallel [010]$ and $\boldsymbol{B} \parallel [001]$
$Pm'n2_1'$ (31.125) $\mathbb{Z}_1$	• $\boldsymbol{E} \parallel [001]$ and $\boldsymbol{B} \parallel [010]$
$P2_1'/m'$ (11.54) $\mathbb{Z}_2 \times \mathbb{Z}_2 \times \mathbb{Z}_4$	• $\boldsymbol{B} \parallel [010]$ and strain $\perp [100]$<br>• $\boldsymbol{B} \parallel [001]$ and strain $\perp [100]$<br>• $\boldsymbol{B} \perp [100]$
$P2$ (3.1) $\mathbb{Z}_2$	• $\boldsymbol{E} \parallel [010]$ and $\boldsymbol{B} \parallel [010]$ and strain $\perp [010]$
P_C2 (3.6) $\mathbb{Z}_1$	• $\boldsymbol{E} \parallel [010]$ and strain $\perp [010]$
$P2/c$ (13.65) $\mathbb{Z}_2 \times \mathbb{Z}_2$	• $\boldsymbol{B} \parallel [010]$ and strain $\perp [010]$
$Pm'm'2$ (25.60) $\mathbb{Z}_2$	• $\boldsymbol{E} \parallel [010]$ and $\boldsymbol{B} \parallel [010]$
P_Inn2 (34.164) $\mathbb{Z}_1$	• $\boldsymbol{E} \parallel [010]$
$Pm'm'n$ (59.409) $\mathbb{Z}_2 \times \mathbb{Z}_2$	• $\boldsymbol{B} \parallel [010]$
$P2$ (3.1) $\mathbb{Z}_2$	• $\boldsymbol{E} \parallel [001]$ and $\boldsymbol{B} \parallel [001]$ and strain $\perp [001]$
P_C2 (3.6) $\mathbb{Z}_1$	• $\boldsymbol{E} \parallel [001]$ and strain $\perp [001]$
$P2/c$ (13.65) $\mathbb{Z}_2 \times \mathbb{Z}_2$	• $\boldsymbol{B} \parallel [001]$ and strain $\perp [001]$
$Pm'm'2$ (25.60) $\mathbb{Z}_2$	• $\boldsymbol{E} \parallel [001]$ and $\boldsymbol{B} \parallel [001]$
P_Inn2 (34.164) $\mathbb{Z}_1$	• $\boldsymbol{E} \parallel [001]$
$Pm'm'n$ (59.409) $\mathbb{Z}_2 \times \mathbb{Z}_2$	• $\boldsymbol{B} \parallel [001]$
$P_S\bar{1}$ (2.7) $\mathbb{Z}_2 \times \mathbb{Z}_2 \times \mathbb{Z}_4$	• strain in generic direction
P_A2/c (13.73) $\mathbb{Z}_2$	• strain $\perp [100]$
P_A2/c (13.73) $\mathbb{Z}_2$	• strain $\perp [010]$
P_A2/c (13.73) $\mathbb{Z}_2$	• strain $\perp [001]$

Table. S 375: Subgroups of $Pccm$ (49.265) that can be realized by applying $\boldsymbol{E}/\boldsymbol{B}$ fields and/or uniaxial strains.

Subgroup & SI group	Perturbation
Pm (6.18) $\mathbb{Z}_1$	• $\boldsymbol{E} \parallel [100]$ and strain $\perp [001]$<br>• $\boldsymbol{E} \parallel [100]$ and $\boldsymbol{B} \parallel [001]$<br>• $\boldsymbol{E} \parallel [010]$ and strain $\perp [001]$<br>• $\boldsymbol{E} \parallel [010]$ and $\boldsymbol{B} \parallel [001]$<br>• $\boldsymbol{E} \perp [001]$
Pc (7.24) $\mathbb{Z}_1$	• $\boldsymbol{E} \parallel [100]$ and strain $\perp [010]$<br>• $\boldsymbol{E} \parallel [100]$ and $\boldsymbol{B} \parallel [010]$<br>• $\boldsymbol{E} \parallel [001]$ and strain $\perp [010]$<br>• $\boldsymbol{E} \parallel [001]$ and $\boldsymbol{B} \parallel [010]$<br>• $\boldsymbol{E} \perp [010]$
$P2$ (3.1) $\mathbb{Z}_2$	• $\boldsymbol{E} \parallel [100]$ and strain $\perp [100]$<br>• $\boldsymbol{E} \parallel [100]$ and $\boldsymbol{B} \parallel [100]$
$Pma2$ (28.87) $\mathbb{Z}_1$	• $\boldsymbol{E} \parallel [100]$
Pc (7.24) $\mathbb{Z}_1$	• $\boldsymbol{E} \parallel [010]$ and strain $\perp [100]$<br>• $\boldsymbol{E} \parallel [010]$ and $\boldsymbol{B} \parallel [100]$<br>• $\boldsymbol{E} \parallel [001]$ and strain $\perp [100]$<br>• $\boldsymbol{E} \parallel [001]$ and $\boldsymbol{B} \parallel [100]$<br>• $\boldsymbol{E} \perp [100]$
$P2$ (3.1) $\mathbb{Z}_2$	• $\boldsymbol{E} \parallel [010]$ and strain $\perp [010]$<br>• $\boldsymbol{E} \parallel [010]$ and $\boldsymbol{B} \parallel [010]$
$Pma2$ (28.87) $\mathbb{Z}_1$	• $\boldsymbol{E} \parallel [010]$
$P2$ (3.1) $\mathbb{Z}_2$	• $\boldsymbol{E} \parallel [001]$ and strain $\perp [001]$<br>• $\boldsymbol{E} \parallel [001]$ and $\boldsymbol{B} \parallel [001]$
$Pcc2$ (27.78) $\mathbb{Z}_2$	• $\boldsymbol{E} \parallel [001]$
$P\bar{1}$ (2.4) $\mathbb{Z}_2 \times \mathbb{Z}_2 \times \mathbb{Z}_2 \times \mathbb{Z}_4$	• strain in generic direction<br>• $\boldsymbol{B} \parallel [100]$ and strain $\perp [010]$<br>• $\boldsymbol{B} \parallel [100]$ and strain $\perp [001]$<br>• $\boldsymbol{B} \parallel [010]$ and strain $\perp [100]$<br>• $\boldsymbol{B} \parallel [010]$ and strain $\perp [001]$<br>• $\boldsymbol{B} \parallel [001]$ and strain $\perp [100]$<br>• $\boldsymbol{B} \parallel [001]$ and strain $\perp [010]$<br>• $\boldsymbol{B}$ in generic direction

Continued on next page

…continued

Subgroup & SI group	Perturbation
$P2/c$ (13.65) $\mathbb{Z}_2\times\mathbb{Z}_2$	• strain $\perp$ [100]<br>• $\boldsymbol{B}$ $\parallel$ [100]
$P2/c$ (13.65) $\mathbb{Z}_2\times\mathbb{Z}_2$	• strain $\perp$ [010]<br>• $\boldsymbol{B}$ $\parallel$ [010]
$P2/m$ (10.42) $\mathbb{Z}_2\times\mathbb{Z}_2\times\mathbb{Z}_2$	• strain $\perp$ [001]<br>• $\boldsymbol{B}$ $\parallel$ [001]

Table. S 376: Subgroups of $Pccm1'$ (49.266) that can be realized by applying $\boldsymbol{E}/\boldsymbol{B}$ fields and/or uniaxial strains.

Subgroup & SI group	Perturbation
$P11'$ (1.2) $\mathbb{Z}_1$	• $\boldsymbol{E}$ $\parallel$ [100] and strain in generic direction<br>• $\boldsymbol{E}$ $\parallel$ [010] and strain in generic direction<br>• $\boldsymbol{E}$ $\parallel$ [001] and strain in generic direction<br>• $\boldsymbol{E}$ $\perp$ [100] and strain $\perp$ [010]<br>• $\boldsymbol{E}$ $\perp$ [100] and strain $\perp$ [001]<br>• $\boldsymbol{E}$ $\perp$ [100] and strain in generic direction<br>• $\boldsymbol{E}$ $\perp$ [010] and strain $\perp$ [100]<br>• $\boldsymbol{E}$ $\perp$ [010] and strain $\perp$ [001]<br>• $\boldsymbol{E}$ $\perp$ [010] and strain in generic direction<br>• $\boldsymbol{E}$ $\perp$ [001] and strain $\perp$ [100]<br>• $\boldsymbol{E}$ $\perp$ [001] and strain $\perp$ [010]<br>• $\boldsymbol{E}$ $\perp$ [001] and strain in generic direction<br>• $\boldsymbol{E}$ in generic direction
Pm' (6.20) $\mathbb{Z}_1$	• $\boldsymbol{E}$ $\parallel$ [100] and $\boldsymbol{B}$ $\parallel$ [100] and strain $\perp$ [001]<br>• $\boldsymbol{E}$ $\parallel$ [100] and $\boldsymbol{B}$ $\parallel$ [010] and strain $\perp$ [001]<br>• $\boldsymbol{E}$ $\parallel$ [100] and $\boldsymbol{B}$ $\perp$ [001]<br>• $\boldsymbol{E}$ $\parallel$ [010] and $\boldsymbol{B}$ $\parallel$ [100] and strain $\perp$ [001]<br>• $\boldsymbol{E}$ $\parallel$ [010] and $\boldsymbol{B}$ $\parallel$ [010] and strain $\perp$ [001]<br>• $\boldsymbol{E}$ $\parallel$ [010] and $\boldsymbol{B}$ $\perp$ [001]<br>• $\boldsymbol{E}$ $\perp$ [001] and $\boldsymbol{B}$ $\parallel$ [100]<br>• $\boldsymbol{E}$ $\perp$ [001] and $\boldsymbol{B}$ $\parallel$ [010]<br>• $\boldsymbol{E}$ $\perp$ [001] and $\boldsymbol{B}$ $\perp$ [001]
Pm (6.18) $\mathbb{Z}_1$	• $\boldsymbol{E}$ $\parallel$ [100] and $\boldsymbol{B}$ $\parallel$ [001] and strain $\perp$ [001]<br>• $\boldsymbol{E}$ $\parallel$ [010] and $\boldsymbol{B}$ $\parallel$ [001] and strain $\perp$ [001]<br>• $\boldsymbol{E}$ $\perp$ [001] and $\boldsymbol{B}$ $\parallel$ [001]

Continued on next page

…continued

Subgroup & SI group	Perturbation
$Pm1'$ (6.19) $\mathbb{Z}_1$	• $\boldsymbol{E}$ ∥ [100] and strain ⊥ [001]<br>• $\boldsymbol{E}$ ∥ [010] and strain ⊥ [001]<br>• $\boldsymbol{E}$ ⊥ [001]
Pc' (7.26) $\mathbb{Z}_1$	• $\boldsymbol{E}$ ∥ [100] and $\boldsymbol{B}$ ∥ [100] and strain ⊥ [010]<br>• $\boldsymbol{E}$ ∥ [100] and $\boldsymbol{B}$ ∥ [001] and strain ⊥ [010]<br>• $\boldsymbol{E}$ ∥ [100] and $\boldsymbol{B}$ ⊥ [010]<br>• $\boldsymbol{E}$ ∥ [001] and $\boldsymbol{B}$ ∥ [100] and strain ⊥ [010]<br>• $\boldsymbol{E}$ ∥ [001] and $\boldsymbol{B}$ ∥ [001] and strain ⊥ [010]<br>• $\boldsymbol{E}$ ∥ [001] and $\boldsymbol{B}$ ⊥ [010]<br>• $\boldsymbol{E}$ ⊥ [010] and $\boldsymbol{B}$ ∥ [100]<br>• $\boldsymbol{E}$ ⊥ [010] and $\boldsymbol{B}$ ∥ [001]<br>• $\boldsymbol{E}$ ⊥ [010] and $\boldsymbol{B}$ ⊥ [010]
Pc (7.24) $\mathbb{Z}_1$	• $\boldsymbol{E}$ ∥ [100] and $\boldsymbol{B}$ ∥ [010] and strain ⊥ [010]<br>• $\boldsymbol{E}$ ∥ [001] and $\boldsymbol{B}$ ∥ [010] and strain ⊥ [010]<br>• $\boldsymbol{E}$ ⊥ [010] and $\boldsymbol{B}$ ∥ [010]
$Pc1'$ (7.25) $\mathbb{Z}_1$	• $\boldsymbol{E}$ ∥ [100] and strain ⊥ [010]<br>• $\boldsymbol{E}$ ∥ [001] and strain ⊥ [010]<br>• $\boldsymbol{E}$ ⊥ [010]
$P2'$ (3.3) $\mathbb{Z}_1$	• $\boldsymbol{E}$ ∥ [100] and $\boldsymbol{B}$ ∥ [010] and strain ⊥ [100]<br>• $\boldsymbol{E}$ ∥ [100] and $\boldsymbol{B}$ ∥ [001] and strain ⊥ [100]<br>• $\boldsymbol{E}$ ∥ [100] and $\boldsymbol{B}$ ⊥ [100]
$Pma'2'$ (28.90) $\mathbb{Z}_1$	• $\boldsymbol{E}$ ∥ [100] and $\boldsymbol{B}$ ∥ [001]
$Pm'a2'$ (28.89) $\mathbb{Z}_1$	• $\boldsymbol{E}$ ∥ [100] and $\boldsymbol{B}$ ∥ [010]
$P2$ (3.1) $\mathbb{Z}_2$	• $\boldsymbol{E}$ ∥ [100] and $\boldsymbol{B}$ ∥ [100] and strain ⊥ [100]
$Pm'a'2$ (28.91) $\mathbb{Z}_2$	• $\boldsymbol{E}$ ∥ [100] and $\boldsymbol{B}$ ∥ [100]
$P21'$ (3.2) $\mathbb{Z}_2$	• $\boldsymbol{E}$ ∥ [100] and strain ⊥ [100]
$Pma21'$ (28.88) $\mathbb{Z}_1$	• $\boldsymbol{E}$ ∥ [100]
Pc' (7.26) $\mathbb{Z}_1$	• $\boldsymbol{E}$ ∥ [010] and $\boldsymbol{B}$ ∥ [010] and strain ⊥ [100]<br>• $\boldsymbol{E}$ ∥ [010] and $\boldsymbol{B}$ ∥ [001] and strain ⊥ [100]<br>• $\boldsymbol{E}$ ∥ [010] and $\boldsymbol{B}$ ⊥ [100]<br>• $\boldsymbol{E}$ ∥ [001] and $\boldsymbol{B}$ ∥ [010] and strain ⊥ [100]<br>• $\boldsymbol{E}$ ∥ [001] and $\boldsymbol{B}$ ∥ [001] and strain ⊥ [100]<br>• $\boldsymbol{E}$ ∥ [001] and $\boldsymbol{B}$ ⊥ [100]<br>• $\boldsymbol{E}$ ⊥ [100] and $\boldsymbol{B}$ ∥ [010]<br>• $\boldsymbol{E}$ ⊥ [100] and $\boldsymbol{B}$ ∥ [001]<br>• $\boldsymbol{E}$ ⊥ [100] and $\boldsymbol{B}$ ⊥ [100]

Continued on next page

. . . continued

Subgroup & SI group	Perturbation
Pc (7.24) $\mathbb{Z}_1$	• $\boldsymbol{E} \parallel [010]$ and $\boldsymbol{B} \parallel [100]$ and strain $\perp [100]$<br>• $\boldsymbol{E} \parallel [001]$ and $\boldsymbol{B} \parallel [100]$ and strain $\perp [100]$<br>• $\boldsymbol{E} \perp [100]$ and $\boldsymbol{B} \parallel [100]$
$Pc1'$ (7.25) $\mathbb{Z}_1$	• $\boldsymbol{E} \parallel [010]$ and strain $\perp [100]$<br>• $\boldsymbol{E} \parallel [001]$ and strain $\perp [100]$<br>• $\boldsymbol{E} \perp [100]$
$P2'$ (3.3) $\mathbb{Z}_1$	• $\boldsymbol{E} \parallel [010]$ and $\boldsymbol{B} \parallel [100]$ and strain $\perp [010]$<br>• $\boldsymbol{E} \parallel [010]$ and $\boldsymbol{B} \parallel [001]$ and strain $\perp [010]$<br>• $\boldsymbol{E} \parallel [010]$ and $\boldsymbol{B} \perp [010]$
$Pma'2'$ (28.90) $\mathbb{Z}_1$	• $\boldsymbol{E} \parallel [010]$ and $\boldsymbol{B} \parallel [001]$
$Pm'a2'$ (28.89) $\mathbb{Z}_1$	• $\boldsymbol{E} \parallel [010]$ and $\boldsymbol{B} \parallel [100]$
$P2$ (3.1) $\mathbb{Z}_2$	• $\boldsymbol{E} \parallel [010]$ and $\boldsymbol{B} \parallel [010]$ and strain $\perp [010]$
$Pm'a'2$ (28.91) $\mathbb{Z}_2$	• $\boldsymbol{E} \parallel [010]$ and $\boldsymbol{B} \parallel [010]$
$P21'$ (3.2) $\mathbb{Z}_2$	• $\boldsymbol{E} \parallel [010]$ and strain $\perp [010]$
$Pma21'$ (28.88) $\mathbb{Z}_1$	• $\boldsymbol{E} \parallel [010]$
$P2'$ (3.3) $\mathbb{Z}_1$	• $\boldsymbol{E} \parallel [001]$ and $\boldsymbol{B} \parallel [100]$ and strain $\perp [001]$<br>• $\boldsymbol{E} \parallel [001]$ and $\boldsymbol{B} \parallel [010]$ and strain $\perp [001]$<br>• $\boldsymbol{E} \parallel [001]$ and $\boldsymbol{B} \perp [001]$
$Pc'c2'$ (27.80) $\mathbb{Z}_1$	• $\boldsymbol{E} \parallel [001]$ and $\boldsymbol{B} \parallel [010]$
$Pc'c2'$ (27.80) $\mathbb{Z}_1$	• $\boldsymbol{E} \parallel [001]$ and $\boldsymbol{B} \parallel [100]$
$P2$ (3.1) $\mathbb{Z}_2$	• $\boldsymbol{E} \parallel [001]$ and $\boldsymbol{B} \parallel [001]$ and strain $\perp [001]$
$Pc'c'2$ (27.81) $\mathbb{Z}_2$	• $\boldsymbol{E} \parallel [001]$ and $\boldsymbol{B} \parallel [001]$
$P21'$ (3.2) $\mathbb{Z}_2$	• $\boldsymbol{E} \parallel [001]$ and strain $\perp [001]$
$Pcc21'$ (27.79) $\mathbb{Z}_2$	• $\boldsymbol{E} \parallel [001]$

Continued on next page

...continued

Subgroup & SI group	Perturbation
$P\bar{1}$ (2.4) $\mathbb{Z}_2 \times \mathbb{Z}_2 \times \mathbb{Z}_2 \times \mathbb{Z}_4$	• $\boldsymbol{B} \parallel [100]$ and strain in generic direction<br>• $\boldsymbol{B} \parallel [010]$ and strain in generic direction<br>• $\boldsymbol{B} \parallel [001]$ and strain in generic direction<br>• $\boldsymbol{B} \perp [100]$ and strain $\perp [010]$<br>• $\boldsymbol{B} \perp [100]$ and strain $\perp [001]$<br>• $\boldsymbol{B} \perp [100]$ and strain in generic direction<br>• $\boldsymbol{B} \perp [010]$ and strain $\perp [100]$<br>• $\boldsymbol{B} \perp [010]$ and strain $\perp [001]$<br>• $\boldsymbol{B} \perp [010]$ and strain in generic direction<br>• $\boldsymbol{B} \perp [001]$ and strain $\perp [100]$<br>• $\boldsymbol{B} \perp [001]$ and strain $\perp [010]$<br>• $\boldsymbol{B} \perp [001]$ and strain in generic direction<br>• $\boldsymbol{B}$ in generic direction
$P2'/c'$ (13.69) $\mathbb{Z}_2 \times \mathbb{Z}_2 \times \mathbb{Z}_4$	• $\boldsymbol{B} \parallel [010]$ and strain $\perp [100]$<br>• $\boldsymbol{B} \parallel [001]$ and strain $\perp [100]$<br>• $\boldsymbol{B} \perp [100]$
$P2/c$ (13.65) $\mathbb{Z}_2 \times \mathbb{Z}_2$	• $\boldsymbol{B} \parallel [100]$ and strain $\perp [100]$
$P2'/c'$ (13.69) $\mathbb{Z}_2 \times \mathbb{Z}_2 \times \mathbb{Z}_4$	• $\boldsymbol{B} \parallel [100]$ and strain $\perp [010]$<br>• $\boldsymbol{B} \parallel [001]$ and strain $\perp [010]$<br>• $\boldsymbol{B} \perp [010]$
$P2/c$ (13.65) $\mathbb{Z}_2 \times \mathbb{Z}_2$	• $\boldsymbol{B} \parallel [010]$ and strain $\perp [010]$
$P2'/m'$ (10.46) $\mathbb{Z}_2 \times \mathbb{Z}_2 \times \mathbb{Z}_2 \times \mathbb{Z}_4$	• $\boldsymbol{B} \parallel [100]$ and strain $\perp [001]$<br>• $\boldsymbol{B} \parallel [010]$ and strain $\perp [001]$<br>• $\boldsymbol{B} \perp [001]$
$Pc'cm'$ (49.270) $\mathbb{Z}_2 \times \mathbb{Z}_2$	• $\boldsymbol{B} \parallel [100]$
$Pc'cm'$ (49.270) $\mathbb{Z}_2 \times \mathbb{Z}_2$	• $\boldsymbol{B} \parallel [010]$
$P2/m$ (10.42) $\mathbb{Z}_2 \times \mathbb{Z}_2 \times \mathbb{Z}_2$	• $\boldsymbol{B} \parallel [001]$ and strain $\perp [001]$
$Pc'c'm$ (49.269) $\mathbb{Z}_2 \times \mathbb{Z}_2$	• $\boldsymbol{B} \parallel [001]$
$P\bar{1}1'$ (2.5) $\mathbb{Z}_2 \times \mathbb{Z}_2 \times \mathbb{Z}_2 \times \mathbb{Z}_4$	• strain in generic direction
$P2/c1'$ (13.66) $\mathbb{Z}_2 \times \mathbb{Z}_2$	• strain $\perp [100]$
$P2/c1'$ (13.66) $\mathbb{Z}_2 \times \mathbb{Z}_2$	• strain $\perp [010]$
$P2/m1'$ (10.43) $\mathbb{Z}_2 \times \mathbb{Z}_2 \times \mathbb{Z}_2$	• strain $\perp [001]$

Table. S 377: Subgroups of $Pc'cm$ (49.267) that can be realized by applying $\boldsymbol{E}/\boldsymbol{B}$ fields and/or uniaxial strains.

Subgroup & SI group	Perturbation
Pm (6.18) $\mathbb{Z}_1$	• $\boldsymbol{B}$ ‖ [001] and strain ⊥ [001]<br>• $\boldsymbol{E}$ ‖ [100] and strain ⊥ [001]<br>• $\boldsymbol{E}$ ‖ [100] and $\boldsymbol{B}$ ‖ [001]<br>• $\boldsymbol{E}$ ‖ [010] and strain ⊥ [001]<br>• $\boldsymbol{E}$ ⊥ [001]
Pc (7.24) $\mathbb{Z}_1$	• $\boldsymbol{B}$ ‖ [010] and strain ⊥ [010]<br>• $\boldsymbol{E}$ ‖ [100] and strain ⊥ [010]<br>• $\boldsymbol{E}$ ‖ [100] and $\boldsymbol{B}$ ‖ [010]<br>• $\boldsymbol{E}$ ‖ [001] and strain ⊥ [010]<br>• $\boldsymbol{E}$ ⊥ [010]
$P2$ (3.1) $\mathbb{Z}_2$	• $\boldsymbol{B}$ ‖ [100] and strain ⊥ [100]<br>• $\boldsymbol{E}$ ‖ [100] and strain ⊥ [100]<br>• $\boldsymbol{E}$ ‖ [100] and $\boldsymbol{B}$ ‖ [100]
$Pma2$ (28.87) $\mathbb{Z}_1$	• $\boldsymbol{E}$ ‖ [100]
Pc' (7.26) $\mathbb{Z}_1$	• $\boldsymbol{B}$ ‖ [010] and strain ⊥ [100]<br>• $\boldsymbol{B}$ ‖ [001] and strain ⊥ [100]<br>• $\boldsymbol{B}$ ⊥ [100]<br>• $\boldsymbol{E}$ ‖ [010] and strain ⊥ [100]<br>• $\boldsymbol{E}$ ‖ [010] and $\boldsymbol{B}$ ‖ [010]<br>• $\boldsymbol{E}$ ‖ [001] and strain ⊥ [100]<br>• $\boldsymbol{E}$ ‖ [001] and $\boldsymbol{B}$ ‖ [001]<br>• $\boldsymbol{E}$ ⊥ [100]
$P2'$ (3.3) $\mathbb{Z}_1$	• $\boldsymbol{B}$ ‖ [100] and strain ⊥ [010]<br>• $\boldsymbol{B}$ ‖ [001] and strain ⊥ [010]<br>• $\boldsymbol{B}$ ⊥ [010]<br>• $\boldsymbol{E}$ ‖ [010] and strain ⊥ [010]<br>• $\boldsymbol{E}$ ‖ [010] and $\boldsymbol{B}$ ‖ [100]
$Pma'2'$ (28.90) $\mathbb{Z}_1$	• $\boldsymbol{B}$ ‖ [001]<br>• $\boldsymbol{E}$ ‖ [010]
$P2'$ (3.3) $\mathbb{Z}_1$	• $\boldsymbol{B}$ ‖ [100] and strain ⊥ [001]<br>• $\boldsymbol{B}$ ‖ [010] and strain ⊥ [001]<br>• $\boldsymbol{B}$ ⊥ [001]<br>• $\boldsymbol{E}$ ‖ [001] and strain ⊥ [001]<br>• $\boldsymbol{E}$ ‖ [001] and $\boldsymbol{B}$ ‖ [100]
$Pc'c2'$ (27.80) $\mathbb{Z}_1$	• $\boldsymbol{B}$ ‖ [010]<br>• $\boldsymbol{E}$ ‖ [001]

Continued on next page

...continued

Subgroup & SI group	Perturbation
$P2'2'2$ (16.3) $\mathbb{Z}_2$	• $\boldsymbol{B} \parallel [100]$
$P\bar{1}'$ (2.6) $\mathbb{Z}_1$	• strain in generic direction
$P2/c'$ (13.68) $\mathbb{Z}_2$	• strain $\perp [100]$
$P2'/c$ (13.67) $\mathbb{Z}_1$	• strain $\perp [010]$
$P2'/m$ (10.44) $\mathbb{Z}_1$	• strain $\perp [001]$

Table. S 378: Subgroups of $Pccm'$ (49.268) that can be realized by applying $\boldsymbol{E}/\boldsymbol{B}$ fields and/or uniaxial strains.

Subgroup & SI group	Perturbation
Pm' (6.20) $\mathbb{Z}_1$	• $\boldsymbol{B} \parallel [100]$ and strain $\perp [001]$<br>• $\boldsymbol{B} \parallel [010]$ and strain $\perp [001]$<br>• $\boldsymbol{B} \perp [001]$<br>• $\boldsymbol{E} \parallel [100]$ and strain $\perp [001]$<br>• $\boldsymbol{E} \parallel [100]$ and $\boldsymbol{B} \parallel [100]$<br>• $\boldsymbol{E} \parallel [010]$ and strain $\perp [001]$<br>• $\boldsymbol{E} \parallel [010]$ and $\boldsymbol{B} \parallel [010]$<br>• $\boldsymbol{E} \perp [001]$
Pc (7.24) $\mathbb{Z}_1$	• $\boldsymbol{B} \parallel [010]$ and strain $\perp [010]$<br>• $\boldsymbol{E} \parallel [100]$ and strain $\perp [010]$<br>• $\boldsymbol{E} \parallel [001]$ and strain $\perp [010]$<br>• $\boldsymbol{E} \parallel [001]$ and $\boldsymbol{B} \parallel [010]$<br>• $\boldsymbol{E} \perp [010]$
$P2'$ (3.3) $\mathbb{Z}_1$	• $\boldsymbol{B} \parallel [010]$ and strain $\perp [100]$<br>• $\boldsymbol{B} \parallel [001]$ and strain $\perp [100]$<br>• $\boldsymbol{B} \perp [100]$<br>• $\boldsymbol{E} \parallel [100]$ and strain $\perp [100]$<br>• $\boldsymbol{E} \parallel [100]$ and $\boldsymbol{B} \parallel [001]$
$Pm'a2'$ (28.89) $\mathbb{Z}_1$	• $\boldsymbol{B} \parallel [010]$<br>• $\boldsymbol{E} \parallel [100]$

Continued on next page

...continued

Subgroup & SI group	Perturbation
Pc (7.24) $\mathbb{Z}_1$	• $\boldsymbol{B} \parallel [100]$ and strain $\perp [100]$<br>• $\boldsymbol{E} \parallel [010]$ and strain $\perp [100]$<br>• $\boldsymbol{E} \parallel [001]$ and strain $\perp [100]$<br>• $\boldsymbol{E} \parallel [001]$ and $\boldsymbol{B} \parallel [100]$<br>• $\boldsymbol{E} \perp [100]$
$P2'$ (3.3) $\mathbb{Z}_1$	• $\boldsymbol{B} \parallel [100]$ and strain $\perp [010]$<br>• $\boldsymbol{B} \parallel [001]$ and strain $\perp [010]$<br>• $\boldsymbol{B} \perp [010]$<br>• $\boldsymbol{E} \parallel [010]$ and strain $\perp [010]$<br>• $\boldsymbol{E} \parallel [010]$ and $\boldsymbol{B} \parallel [001]$
$Pm'a2'$ (28.89) $\mathbb{Z}_1$	• $\boldsymbol{B} \parallel [100]$<br>• $\boldsymbol{E} \parallel [010]$
$P2$ (3.1) $\mathbb{Z}_2$	• $\boldsymbol{B} \parallel [001]$ and strain $\perp [001]$<br>• $\boldsymbol{E} \parallel [001]$ and strain $\perp [001]$<br>• $\boldsymbol{E} \parallel [001]$ and $\boldsymbol{B} \parallel [001]$
$Pcc2$ (27.78) $\mathbb{Z}_2$	• $\boldsymbol{E} \parallel [001]$
$P2'2'2$ (16.3) $\mathbb{Z}_2$	• $\boldsymbol{B} \parallel [001]$
$P\bar{1}'$ (2.6) $\mathbb{Z}_1$	• strain in generic direction
$P2'/c$ (13.67) $\mathbb{Z}_1$	• strain $\perp [100]$
$P2'/c$ (13.67) $\mathbb{Z}_1$	• strain $\perp [010]$
$P2/m'$ (10.45) $\mathbb{Z}_2$	• strain $\perp [001]$

Table. S 379: Subgroups of $Pc'c'm$ (49.269) that can be realized by applying $\boldsymbol{E}/\boldsymbol{B}$ fields and/or uniaxial strains.

Subgroup & SI group	Perturbation
Pm (6.18) $\mathbb{Z}_1$	• $\boldsymbol{E} \parallel [100]$ and strain $\perp [001]$<br>• $\boldsymbol{E} \parallel [010]$ and strain $\perp [001]$<br>• $\boldsymbol{E} \perp [001]$

Continued on next page

…continued

Subgroup & SI group	Perturbation
Pc' (7.26) $\mathbb{Z}_1$	• $\boldsymbol{E} \parallel [100]$ and strain $\perp [010]$<br>• $\boldsymbol{E} \parallel [100]$ and ($\boldsymbol{B} \parallel [100]$ or $\boldsymbol{B} \perp [010]$)<br>• $\boldsymbol{E} \parallel [001]$ and strain $\perp [010]$<br>• $\boldsymbol{E} \parallel [001]$ and ($\boldsymbol{B} \parallel [100]$ or $\boldsymbol{B} \perp [010]$)<br>• $\boldsymbol{E} \perp [010]$
$P2'$ (3.3) $\mathbb{Z}_1$	• $\boldsymbol{E} \parallel [100]$ and strain $\perp [100]$<br>• $\boldsymbol{E} \parallel [100]$ and ($\boldsymbol{B} \parallel [010]$ or $\boldsymbol{B} \perp [100]$)
$Pma'2'$ (28.90) $\mathbb{Z}_1$	• $\boldsymbol{E} \parallel [100]$
Pc' (7.26) $\mathbb{Z}_1$	• $\boldsymbol{E} \parallel [010]$ and strain $\perp [100]$<br>• $\boldsymbol{E} \parallel [010]$ and ($\boldsymbol{B} \parallel [010]$ or $\boldsymbol{B} \perp [100]$)<br>• $\boldsymbol{E} \parallel [001]$ and strain $\perp [100]$<br>• $\boldsymbol{E} \parallel [001]$ and ($\boldsymbol{B} \parallel [010]$ or $\boldsymbol{B} \perp [100]$)<br>• $\boldsymbol{E} \perp [100]$
$P2'$ (3.3) $\mathbb{Z}_1$	• $\boldsymbol{E} \parallel [010]$ and strain $\perp [010]$<br>• $\boldsymbol{E} \parallel [010]$ and ($\boldsymbol{B} \parallel [100]$ or $\boldsymbol{B} \perp [010]$)
$Pma'2'$ (28.90) $\mathbb{Z}_1$	• $\boldsymbol{E} \parallel [010]$
$P2$ (3.1) $\mathbb{Z}_2$	• $\boldsymbol{E} \parallel [001]$ and strain $\perp [001]$
$Pc'c'2$ (27.81) $\mathbb{Z}_2$	• $\boldsymbol{E} \parallel [001]$
$P\bar{1}$ (2.4) $\mathbb{Z}_2 \times \mathbb{Z}_2 \times \mathbb{Z}_2 \times \mathbb{Z}_4$	• strain in generic direction<br>• ($\boldsymbol{B} \parallel [100]$ or $\boldsymbol{B} \perp [010]$) and strain $\perp [100]$<br>• ($\boldsymbol{B} \parallel [100]$ or $\boldsymbol{B} \perp [010]$) and strain $\perp [001]$<br>• ($\boldsymbol{B} \parallel [010]$ or $\boldsymbol{B} \perp [100]$) and strain $\perp [010]$<br>• ($\boldsymbol{B} \parallel [010]$ or $\boldsymbol{B} \perp [100]$) and strain $\perp [001]$<br>• $\boldsymbol{B}$ in generic direction
$P2'/c'$ (13.69) $\mathbb{Z}_2 \times \mathbb{Z}_2 \times \mathbb{Z}_4$	• strain $\perp [100]$<br>• ($\boldsymbol{B} \parallel [010]$ or $\boldsymbol{B} \perp [100]$)
$P2'/c'$ (13.69) $\mathbb{Z}_2 \times \mathbb{Z}_2 \times \mathbb{Z}_4$	• strain $\perp [010]$<br>• ($\boldsymbol{B} \parallel [100]$ or $\boldsymbol{B} \perp [010]$)
$P2/m$ (10.42) $\mathbb{Z}_2 \times \mathbb{Z}_2 \times \mathbb{Z}_2$	• strain $\perp [001]$

Table. S 380: Subgroups of $Pc'cm'$ (49.270) that can be realized by applying $\boldsymbol{E}/\boldsymbol{B}$ fields and/or uniaxial strains.

Subgroup & SI group	Perturbation
Pm' (6.20) $\mathbb{Z}_1$	• $\boldsymbol{E} \parallel [100]$ and strain $\perp [001]$<br>• $\boldsymbol{E} \parallel [100]$ and ($\boldsymbol{B} \parallel [100]$ or $\boldsymbol{B} \perp [001]$)<br>• $\boldsymbol{E} \parallel [010]$ and strain $\perp [001]$<br>• $\boldsymbol{E} \parallel [010]$ and ($\boldsymbol{B} \parallel [100]$ or $\boldsymbol{B} \perp [001]$)<br>• $\boldsymbol{E} \perp [001]$
Pc (7.24) $\mathbb{Z}_1$	• $\boldsymbol{E} \parallel [100]$ and strain $\perp [010]$<br>• $\boldsymbol{E} \parallel [001]$ and strain $\perp [010]$<br>• $\boldsymbol{E} \perp [010]$
$P2'$ (3.3) $\mathbb{Z}_1$	• $\boldsymbol{E} \parallel [100]$ and strain $\perp [100]$<br>• $\boldsymbol{E} \parallel [100]$ and ($\boldsymbol{B} \parallel [001]$ or $\boldsymbol{B} \perp [100]$)
$Pm'a2'$ (28.89) $\mathbb{Z}_1$	• $\boldsymbol{E} \parallel [100]$
Pc' (7.26) $\mathbb{Z}_1$	• $\boldsymbol{E} \parallel [010]$ and strain $\perp [100]$<br>• $\boldsymbol{E} \parallel [010]$ and ($\boldsymbol{B} \parallel [001]$ or $\boldsymbol{B} \perp [100]$)<br>• $\boldsymbol{E} \parallel [001]$ and strain $\perp [100]$<br>• $\boldsymbol{E} \parallel [001]$ and ($\boldsymbol{B} \parallel [001]$ or $\boldsymbol{B} \perp [100]$)<br>• $\boldsymbol{E} \perp [100]$
$P2$ (3.1) $\mathbb{Z}_2$	• $\boldsymbol{E} \parallel [010]$ and strain $\perp [010]$
$Pm'a'2$ (28.91) $\mathbb{Z}_2$	• $\boldsymbol{E} \parallel [010]$
$P2'$ (3.3) $\mathbb{Z}_1$	• $\boldsymbol{E} \parallel [001]$ and strain $\perp [001]$<br>• $\boldsymbol{E} \parallel [001]$ and ($\boldsymbol{B} \parallel [100]$ or $\boldsymbol{B} \perp [001]$)
$Pc'c2'$ (27.80) $\mathbb{Z}_1$	• $\boldsymbol{E} \parallel [001]$
$P\bar{1}$ (2.4) $\mathbb{Z}_2 \times \mathbb{Z}_2 \times \mathbb{Z}_2 \times \mathbb{Z}_4$	• strain in generic direction<br>• ($\boldsymbol{B} \parallel [100]$ or $\boldsymbol{B} \perp [001]$) and strain $\perp [100]$<br>• ($\boldsymbol{B} \parallel [100]$ or $\boldsymbol{B} \perp [001]$) and strain $\perp [010]$<br>• ($\boldsymbol{B} \parallel [001]$ or $\boldsymbol{B} \perp [100]$) and strain $\perp [010]$<br>• ($\boldsymbol{B} \parallel [001]$ or $\boldsymbol{B} \perp [100]$) and strain $\perp [001]$<br>• $\boldsymbol{B}$ in generic direction
$P2'/c'$ (13.69) $\mathbb{Z}_2 \times \mathbb{Z}_2 \times \mathbb{Z}_4$	• strain $\perp [100]$<br>• ($\boldsymbol{B} \parallel [001]$ or $\boldsymbol{B} \perp [100]$)
$P2/c$ (13.65) $\mathbb{Z}_2 \times \mathbb{Z}_2$	• strain $\perp [010]$
$P2'/m'$ (10.46) $\mathbb{Z}_2 \times \mathbb{Z}_2 \times \mathbb{Z}_2 \times \mathbb{Z}_4$	• strain $\perp [001]$<br>• ($\boldsymbol{B} \parallel [100]$ or $\boldsymbol{B} \perp [001]$)

Table. S 381: Subgroups of $Pc'c'm'$ (49.271) that can be realized by applying $\boldsymbol{E}/\boldsymbol{B}$ fields and/or uniaxial strains.

Subgroup & SI group	Perturbation
Pm' (6.20) $\mathbb{Z}_1$	• $\boldsymbol{B} \parallel [100]$ and strain $\perp [001]$<br>• $\boldsymbol{B} \parallel [010]$ and strain $\perp [001]$<br>• $\boldsymbol{B} \perp [001]$<br>• $\boldsymbol{E} \parallel [100]$ and strain $\perp [001]$<br>• $\boldsymbol{E} \parallel [100]$ and $\boldsymbol{B} \parallel [010]$<br>• $\boldsymbol{E} \parallel [010]$ and strain $\perp [001]$<br>• $\boldsymbol{E} \parallel [010]$ and $\boldsymbol{B} \parallel [100]$<br>• $\boldsymbol{E} \perp [001]$
Pc' (7.26) $\mathbb{Z}_1$	• $\boldsymbol{B} \parallel [100]$ and strain $\perp [010]$<br>• $\boldsymbol{B} \parallel [001]$ and strain $\perp [010]$<br>• $\boldsymbol{B} \perp [010]$<br>• $\boldsymbol{E} \parallel [100]$ and strain $\perp [010]$<br>• $\boldsymbol{E} \parallel [100]$ and $\boldsymbol{B} \parallel [001]$<br>• $\boldsymbol{E} \parallel [001]$ and strain $\perp [010]$<br>• $\boldsymbol{E} \parallel [001]$ and $\boldsymbol{B} \parallel [100]$<br>• $\boldsymbol{E} \perp [010]$
$P2$ (3.1) $\mathbb{Z}_2$	• $\boldsymbol{B} \parallel [100]$ and strain $\perp [100]$<br>• $\boldsymbol{E} \parallel [100]$ and strain $\perp [100]$
$Pm'a'2$ (28.91) $\mathbb{Z}_2$	• $\boldsymbol{B} \parallel [100]$<br>• $\boldsymbol{E} \parallel [100]$
Pc' (7.26) $\mathbb{Z}_1$	• $\boldsymbol{B} \parallel [010]$ and strain $\perp [100]$<br>• $\boldsymbol{B} \parallel [001]$ and strain $\perp [100]$<br>• $\boldsymbol{B} \perp [100]$<br>• $\boldsymbol{E} \parallel [010]$ and strain $\perp [100]$<br>• $\boldsymbol{E} \parallel [010]$ and $\boldsymbol{B} \parallel [001]$<br>• $\boldsymbol{E} \parallel [001]$ and strain $\perp [100]$<br>• $\boldsymbol{E} \parallel [001]$ and $\boldsymbol{B} \parallel [010]$<br>• $\boldsymbol{E} \perp [100]$
$P2$ (3.1) $\mathbb{Z}_2$	• $\boldsymbol{B} \parallel [010]$ and strain $\perp [010]$<br>• $\boldsymbol{E} \parallel [010]$ and strain $\perp [010]$
$Pm'a'2$ (28.91) $\mathbb{Z}_2$	• $\boldsymbol{B} \parallel [010]$<br>• $\boldsymbol{E} \parallel [010]$
$P2$ (3.1) $\mathbb{Z}_2$	• $\boldsymbol{B} \parallel [001]$ and strain $\perp [001]$<br>• $\boldsymbol{E} \parallel [001]$ and strain $\perp [001]$
$Pc'c'2$ (27.81) $\mathbb{Z}_2$	• $\boldsymbol{B} \parallel [001]$<br>• $\boldsymbol{E} \parallel [001]$

Continued on next page

...continued

Subgroup & SI group	Perturbation
$P\bar{1}'$ (2.6) $\mathbb{Z}_1$	• strain in generic direction
$P2/c'$ (13.68) $\mathbb{Z}_2$	• strain $\perp$ [100]
$P2/c'$ (13.68) $\mathbb{Z}_2$	• strain $\perp$ [010]
$P2/m'$ (10.45) $\mathbb{Z}_2$	• strain $\perp$ [001]

Table. S 382: Subgroups of P_accm (49.272) that can be realized by applying $\boldsymbol{E}/\boldsymbol{B}$ fields and/or uniaxial strains.

Subgroup & SI group	Perturbation
Pm (6.18) $\mathbb{Z}_1$	• $\boldsymbol{E}$ || [100] and $\boldsymbol{B}$ || [001] and strain $\perp$ [001]<br>• $\boldsymbol{E}$ || [010] and $\boldsymbol{B}$ || [001] and strain $\perp$ [001]<br>• $\boldsymbol{E}$ $\perp$ [001] and $\boldsymbol{B}$ || [001]
Pc (7.24) $\mathbb{Z}_1$	• $\boldsymbol{E}$ || [100] and $\boldsymbol{B}$ || [010] and strain $\perp$ [010]<br>• $\boldsymbol{E}$ || [001] and $\boldsymbol{B}$ || [010] and strain $\perp$ [010]<br>• $\boldsymbol{E}$ $\perp$ [010] and $\boldsymbol{B}$ || [010]
$P2$ (3.1) $\mathbb{Z}_2$	• $\boldsymbol{E}$ || [100] and $\boldsymbol{B}$ || [100] and strain $\perp$ [100]
P_S1 (1.3) $\mathbb{Z}_1$	• $\boldsymbol{E}$ || [100] and strain in generic direction<br>• $\boldsymbol{E}$ || [010] and strain in generic direction<br>• $\boldsymbol{E}$ || [001] and strain in generic direction<br>• $\boldsymbol{E}$ $\perp$ [100] and strain $\perp$ [010]<br>• $\boldsymbol{E}$ $\perp$ [100] and strain $\perp$ [001]<br>• $\boldsymbol{E}$ $\perp$ [100] and strain in generic direction<br>• $\boldsymbol{E}$ $\perp$ [010] and strain $\perp$ [100]<br>• $\boldsymbol{E}$ $\perp$ [010] and strain $\perp$ [001]<br>• $\boldsymbol{E}$ $\perp$ [010] and strain in generic direction<br>• $\boldsymbol{E}$ $\perp$ [001] and strain $\perp$ [100]<br>• $\boldsymbol{E}$ $\perp$ [001] and strain $\perp$ [010]<br>• $\boldsymbol{E}$ $\perp$ [001] and strain in generic direction<br>• $\boldsymbol{E}$ in generic direction

Continued on next page

…continued

Subgroup & SI group	Perturbation
Pc' (7.26) $\mathbb{Z}_1$	• $\boldsymbol{E} \parallel [100]$ and $\boldsymbol{B} \parallel [100]$ and strain $\perp [001]$<br>• $\boldsymbol{E} \parallel [100]$ and $\boldsymbol{B} \parallel [010]$ and strain $\perp [001]$<br>• $\boldsymbol{E} \parallel [100]$ and $\boldsymbol{B} \perp [001]$<br>• $\boldsymbol{E} \parallel [010]$ and $\boldsymbol{B} \parallel [100]$ and strain $\perp [001]$<br>• $\boldsymbol{E} \parallel [010]$ and $\boldsymbol{B} \parallel [010]$ and strain $\perp [001]$<br>• $\boldsymbol{E} \parallel [010]$ and $\boldsymbol{B} \perp [001]$<br>• $\boldsymbol{E} \perp [001]$ and $\boldsymbol{B} \parallel [100]$<br>• $\boldsymbol{E} \perp [001]$ and $\boldsymbol{B} \parallel [010]$<br>• $\boldsymbol{E} \perp [001]$ and $\boldsymbol{B} \perp [001]$
P_am (6.21) $\mathbb{Z}_1$	• $\boldsymbol{E} \parallel [100]$ and strain $\perp [001]$<br>• $\boldsymbol{E} \parallel [010]$ and strain $\perp [001]$<br>• $\boldsymbol{E} \perp [001]$
Pc' (7.26) $\mathbb{Z}_1$	• $\boldsymbol{E} \parallel [100]$ and $\boldsymbol{B} \parallel [100]$ and strain $\perp [010]$<br>• $\boldsymbol{E} \parallel [100]$ and $\boldsymbol{B} \parallel [001]$ and strain $\perp [010]$<br>• $\boldsymbol{E} \parallel [100]$ and $\boldsymbol{B} \perp [010]$<br>• $\boldsymbol{E} \parallel [001]$ and $\boldsymbol{B} \parallel [100]$ and strain $\perp [010]$<br>• $\boldsymbol{E} \parallel [001]$ and $\boldsymbol{B} \parallel [001]$ and strain $\perp [010]$<br>• $\boldsymbol{E} \parallel [001]$ and $\boldsymbol{B} \perp [010]$<br>• $\boldsymbol{E} \perp [010]$ and $\boldsymbol{B} \parallel [100]$<br>• $\boldsymbol{E} \perp [010]$ and $\boldsymbol{B} \parallel [001]$<br>• $\boldsymbol{E} \perp [010]$ and $\boldsymbol{B} \perp [010]$
P_ac (7.27) $\mathbb{Z}_1$	• $\boldsymbol{E} \parallel [100]$ and strain $\perp [010]$<br>• $\boldsymbol{E} \parallel [001]$ and strain $\perp [010]$<br>• $\boldsymbol{E} \perp [010]$
$Pn'c'2$ (30.115) $\mathbb{Z}_1$	• $\boldsymbol{E} \parallel [100]$ and $\boldsymbol{B} \parallel [100]$
$P2_1'$ (4.9) $\mathbb{Z}_1$	• $\boldsymbol{E} \parallel [100]$ and $\boldsymbol{B} \parallel [010]$ and strain $\perp [100]$<br>• $\boldsymbol{E} \parallel [100]$ and $\boldsymbol{B} \parallel [001]$ and strain $\perp [100]$<br>• $\boldsymbol{E} \parallel [100]$ and $\boldsymbol{B} \perp [100]$
P_b2 (3.5) $\mathbb{Z}_2$	• $\boldsymbol{E} \parallel [100]$ and strain $\perp [100]$
$Pc'a2_1'$ (29.101) $\mathbb{Z}_1$	• $\boldsymbol{E} \parallel [100]$ and $\boldsymbol{B} \parallel [010]$
$Pmn'2_1'$ (31.126) $\mathbb{Z}_1$	• $\boldsymbol{E} \parallel [100]$ and $\boldsymbol{B} \parallel [001]$
P_cma2 (28.94) $\mathbb{Z}_1$	• $\boldsymbol{E} \parallel [100]$
Pc (7.24) $\mathbb{Z}_1$	• $\boldsymbol{E} \parallel [010]$ and $\boldsymbol{B} \parallel [100]$ and strain $\perp [100]$<br>• $\boldsymbol{E} \parallel [001]$ and $\boldsymbol{B} \parallel [100]$ and strain $\perp [100]$<br>• $\boldsymbol{E} \perp [100]$ and $\boldsymbol{B} \parallel [100]$
$P2$ (3.1) $\mathbb{Z}_2$	• $\boldsymbol{E} \parallel [010]$ and $\boldsymbol{B} \parallel [010]$ and strain $\perp [010]$

Continued on next page

…continued

Subgroup & SI group	Perturbation
$P2$ (3.1) $\mathbb{Z}_2$	• $\boldsymbol{E} \parallel [001]$ and $\boldsymbol{B} \parallel [001]$ and strain $\perp [001]$
$P\bar{1}$ (2.4) $\mathbb{Z}_2 \times \mathbb{Z}_2 \times \mathbb{Z}_2 \times \mathbb{Z}_4$	• $\boldsymbol{B} \parallel [100]$ and strain in generic direction<br>• $\boldsymbol{B} \parallel [010]$ and strain in generic direction<br>• $\boldsymbol{B} \parallel [001]$ and strain in generic direction<br>• $\boldsymbol{B} \perp [100]$ and strain $\perp [010]$<br>• $\boldsymbol{B} \perp [100]$ and strain $\perp [001]$<br>• $\boldsymbol{B} \perp [100]$ and strain in generic direction<br>• $\boldsymbol{B} \perp [010]$ and strain $\perp [100]$<br>• $\boldsymbol{B} \perp [010]$ and strain $\perp [001]$<br>• $\boldsymbol{B} \perp [010]$ and strain in generic direction<br>• $\boldsymbol{B} \perp [001]$ and strain $\perp [100]$<br>• $\boldsymbol{B} \perp [001]$ and strain $\perp [010]$<br>• $\boldsymbol{B} \perp [001]$ and strain in generic direction<br>• $\boldsymbol{B}$ in generic direction
$P2/c$ (13.65) $\mathbb{Z}_2 \times \mathbb{Z}_2$	• $\boldsymbol{B} \parallel [100]$ and strain $\perp [100]$
$P2/c$ (13.65) $\mathbb{Z}_2 \times \mathbb{Z}_2$	• $\boldsymbol{B} \parallel [010]$ and strain $\perp [010]$
$P2/m$ (10.42) $\mathbb{Z}_2 \times \mathbb{Z}_2 \times \mathbb{Z}_2$	• $\boldsymbol{B} \parallel [001]$ and strain $\perp [001]$
Pc' (7.26) $\mathbb{Z}_1$	• $\boldsymbol{E} \parallel [010]$ and $\boldsymbol{B} \parallel [010]$ and strain $\perp [100]$<br>• $\boldsymbol{E} \parallel [010]$ and $\boldsymbol{B} \parallel [001]$ and strain $\perp [100]$<br>• $\boldsymbol{E} \parallel [010]$ and $\boldsymbol{B} \perp [100]$<br>• $\boldsymbol{E} \parallel [001]$ and $\boldsymbol{B} \parallel [010]$ and strain $\perp [100]$<br>• $\boldsymbol{E} \parallel [001]$ and $\boldsymbol{B} \parallel [001]$ and strain $\perp [100]$<br>• $\boldsymbol{E} \parallel [001]$ and $\boldsymbol{B} \perp [100]$<br>• $\boldsymbol{E} \perp [100]$ and $\boldsymbol{B} \parallel [010]$<br>• $\boldsymbol{E} \perp [100]$ and $\boldsymbol{B} \parallel [001]$<br>• $\boldsymbol{E} \perp [100]$ and $\boldsymbol{B} \perp [100]$
P_bc (7.29) $\mathbb{Z}_1$	• $\boldsymbol{E} \parallel [010]$ and strain $\perp [100]$<br>• $\boldsymbol{E} \parallel [001]$ and strain $\perp [100]$<br>• $\boldsymbol{E} \perp [100]$
$Pb'a'2$ (32.138) $\mathbb{Z}_2$	• $\boldsymbol{E} \parallel [010]$ and $\boldsymbol{B} \parallel [010]$
$Pn'c'2$ (30.115) $\mathbb{Z}_1$	• $\boldsymbol{E} \parallel [001]$ and $\boldsymbol{B} \parallel [001]$
$P2_1'/c'$ (14.79) $\mathbb{Z}_2 \times \mathbb{Z}_4$	• $\boldsymbol{B} \parallel [010]$ and strain $\perp [100]$<br>• $\boldsymbol{B} \parallel [001]$ and strain $\perp [100]$<br>• $\boldsymbol{B} \perp [100]$

Continued on next page

...continued

Subgroup & SI group	Perturbation
$P2'$ (3.3) $\mathbb{Z}_1$	• $\boldsymbol{E} \parallel [010]$ and $\boldsymbol{B} \parallel [100]$ and strain $\perp [010]$<br>• $\boldsymbol{E} \parallel [010]$ and $\boldsymbol{B} \parallel [001]$ and strain $\perp [010]$<br>• $\boldsymbol{E} \parallel [010]$ and $\boldsymbol{B} \perp [010]$
$Pb'a2'$ (32.137) $\mathbb{Z}_1$	• $\boldsymbol{E} \parallel [010]$ and $\boldsymbol{B} \parallel [100]$
P_a2 (3.4) $\mathbb{Z}_2$	• $\boldsymbol{E} \parallel [010]$ and strain $\perp [010]$
$P2'/c'$ (13.69) $\mathbb{Z}_2 \times \mathbb{Z}_2 \times \mathbb{Z}_4$	• $\boldsymbol{B} \parallel [100]$ and strain $\perp [010]$<br>• $\boldsymbol{B} \parallel [001]$ and strain $\perp [010]$<br>• $\boldsymbol{B} \perp [010]$
$Pma'2'$ (28.90) $\mathbb{Z}_1$	• $\boldsymbol{E} \parallel [010]$ and $\boldsymbol{B} \parallel [001]$
P_bma2 (28.93) $\mathbb{Z}_1$	• $\boldsymbol{E} \parallel [010]$
$Pmn'a'$ (53.327) $\mathbb{Z}_2 \times \mathbb{Z}_2$	• $\boldsymbol{B} \parallel [001]$
$P2'$ (3.3) $\mathbb{Z}_1$	• $\boldsymbol{E} \parallel [001]$ and $\boldsymbol{B} \parallel [100]$ and strain $\perp [001]$<br>• $\boldsymbol{E} \parallel [001]$ and $\boldsymbol{B} \parallel [010]$ and strain $\perp [001]$<br>• $\boldsymbol{E} \parallel [001]$ and $\boldsymbol{B} \perp [001]$
$Pn'c2'$ (30.113) $\mathbb{Z}_1$	• $\boldsymbol{E} \parallel [001]$ and $\boldsymbol{B} \parallel [100]$
P_a2 (3.4) $\mathbb{Z}_2$	• $\boldsymbol{E} \parallel [001]$ and strain $\perp [001]$
$P2'/c'$ (13.69) $\mathbb{Z}_2 \times \mathbb{Z}_2 \times \mathbb{Z}_4$	• $\boldsymbol{B} \parallel [100]$ and strain $\perp [001]$<br>• $\boldsymbol{B} \parallel [010]$ and strain $\perp [001]$<br>• $\boldsymbol{B} \perp [001]$
$Pc'c2'$ (27.80) $\mathbb{Z}_1$	• $\boldsymbol{E} \parallel [001]$ and $\boldsymbol{B} \parallel [010]$
P_acc2 (27.83) $\mathbb{Z}_2$	• $\boldsymbol{E} \parallel [001]$
$Pc'ca'$ (54.344) $\mathbb{Z}_2 \times \mathbb{Z}_2$	• $\boldsymbol{B} \parallel [010]$
$Pb'an'$ (50.282) $\mathbb{Z}_2$	• $\boldsymbol{B} \parallel [100]$
$P_S\bar{1}$ (2.7) $\mathbb{Z}_2 \times \mathbb{Z}_2 \times \mathbb{Z}_4$	• strain in generic direction
P_b2/c (13.71) $\mathbb{Z}_2 \times \mathbb{Z}_2$	• strain $\perp [100]$
P_a2/c (13.70) $\mathbb{Z}_2 \times \mathbb{Z}_2$	• strain $\perp [010]$
P_a2/m (10.47) $\mathbb{Z}_2 \times \mathbb{Z}_2 \times \mathbb{Z}_2$	• strain $\perp [001]$

Table. S 383: Subgroups of $P_{c}ccm$ (49.273) that can be realized by applying $\boldsymbol{E}/\boldsymbol{B}$ fields and/or uniaxial strains.

Subgroup & SI group	Perturbation
P_S1 (1.3) $\mathbb{Z}_1$	• $\boldsymbol{E}$ ∥ [100] and strain in generic direction<br>• $\boldsymbol{E}$ ∥ [010] and strain in generic direction<br>• $\boldsymbol{E}$ ∥ [001] and strain in generic direction<br>• $\boldsymbol{E}$ ⊥ [100] and strain ⊥ [010]<br>• $\boldsymbol{E}$ ⊥ [100] and strain ⊥ [001]<br>• $\boldsymbol{E}$ ⊥ [100] and strain in generic direction<br>• $\boldsymbol{E}$ ⊥ [010] and strain ⊥ [100]<br>• $\boldsymbol{E}$ ⊥ [010] and strain ⊥ [001]<br>• $\boldsymbol{E}$ ⊥ [010] and strain in generic direction<br>• $\boldsymbol{E}$ ⊥ [001] and strain ⊥ [100]<br>• $\boldsymbol{E}$ ⊥ [001] and strain ⊥ [010]<br>• $\boldsymbol{E}$ ⊥ [001] and strain in generic direction<br>• $\boldsymbol{E}$ in generic direction
Pm (6.18) $\mathbb{Z}_1$	• $\boldsymbol{E}$ ∥ [100] and $\boldsymbol{B}$ ∥ [001] and strain ⊥ [001]<br>• $\boldsymbol{E}$ ∥ [010] and $\boldsymbol{B}$ ∥ [001] and strain ⊥ [001]<br>• $\boldsymbol{E}$ ⊥ [001] and $\boldsymbol{B}$ ∥ [001]
Pm' (6.20) $\mathbb{Z}_1$	• $\boldsymbol{E}$ ∥ [100] and $\boldsymbol{B}$ ∥ [100] and strain ⊥ [001]<br>• $\boldsymbol{E}$ ∥ [100] and $\boldsymbol{B}$ ∥ [010] and strain ⊥ [001]<br>• $\boldsymbol{E}$ ∥ [100] and $\boldsymbol{B}$ ⊥ [001]<br>• $\boldsymbol{E}$ ∥ [010] and $\boldsymbol{B}$ ∥ [100] and strain ⊥ [001]<br>• $\boldsymbol{E}$ ∥ [010] and $\boldsymbol{B}$ ∥ [010] and strain ⊥ [001]<br>• $\boldsymbol{E}$ ∥ [010] and $\boldsymbol{B}$ ⊥ [001]<br>• $\boldsymbol{E}$ ⊥ [001] and $\boldsymbol{B}$ ∥ [100]<br>• $\boldsymbol{E}$ ⊥ [001] and $\boldsymbol{B}$ ∥ [010]<br>• $\boldsymbol{E}$ ⊥ [001] and $\boldsymbol{B}$ ⊥ [001]
P_bm (6.22) $\mathbb{Z}_1$	• $\boldsymbol{E}$ ∥ [100] and strain ⊥ [001]<br>• $\boldsymbol{E}$ ∥ [010] and strain ⊥ [001]<br>• $\boldsymbol{E}$ ⊥ [001]
Pm' (6.20) $\mathbb{Z}_1$	• $\boldsymbol{E}$ ∥ [100] and $\boldsymbol{B}$ ∥ [100] and strain ⊥ [010]<br>• $\boldsymbol{E}$ ∥ [100] and $\boldsymbol{B}$ ∥ [001] and strain ⊥ [010]<br>• $\boldsymbol{E}$ ∥ [100] and $\boldsymbol{B}$ ⊥ [010]<br>• $\boldsymbol{E}$ ∥ [001] and $\boldsymbol{B}$ ∥ [100] and strain ⊥ [010]<br>• $\boldsymbol{E}$ ∥ [001] and $\boldsymbol{B}$ ∥ [001] and strain ⊥ [010]<br>• $\boldsymbol{E}$ ∥ [001] and $\boldsymbol{B}$ ⊥ [010]<br>• $\boldsymbol{E}$ ⊥ [010] and $\boldsymbol{B}$ ∥ [100]<br>• $\boldsymbol{E}$ ⊥ [010] and $\boldsymbol{B}$ ∥ [001]<br>• $\boldsymbol{E}$ ⊥ [010] and $\boldsymbol{B}$ ⊥ [010]

Continued on next page

. . . continued

Subgroup & SI group	Perturbation
Pc (7.24) $\mathbb{Z}_1$	• $\boldsymbol{E}$ ∥ [100] and $\boldsymbol{B}$ ∥ [010] and strain ⊥ [010]<br>• $\boldsymbol{E}$ ∥ [001] and $\boldsymbol{B}$ ∥ [010] and strain ⊥ [010]<br>• $\boldsymbol{E}$ ⊥ [010] and $\boldsymbol{B}$ ∥ [010]
P_cc (7.28) $\mathbb{Z}_1$	• $\boldsymbol{E}$ ∥ [100] and strain ⊥ [010]<br>• $\boldsymbol{E}$ ∥ [001] and strain ⊥ [010]<br>• $\boldsymbol{E}$ ⊥ [010]
$P2'$ (3.3) $\mathbb{Z}_1$	• $\boldsymbol{E}$ ∥ [100] and $\boldsymbol{B}$ ∥ [010] and strain ⊥ [100]<br>• $\boldsymbol{E}$ ∥ [100] and $\boldsymbol{B}$ ∥ [001] and strain ⊥ [100]<br>• $\boldsymbol{E}$ ∥ [100] and $\boldsymbol{B}$ ⊥ [100]
$Pm'm2'$ (25.59) $\mathbb{Z}_1$	• $\boldsymbol{E}$ ∥ [100] and $\boldsymbol{B}$ ∥ [001]
$Pm'a2'$ (28.89) $\mathbb{Z}_1$	• $\boldsymbol{E}$ ∥ [100] and $\boldsymbol{B}$ ∥ [010]
$P2$ (3.1) $\mathbb{Z}_2$	• $\boldsymbol{E}$ ∥ [100] and $\boldsymbol{B}$ ∥ [100] and strain ⊥ [100]
$Pm'm'2$ (25.60) $\mathbb{Z}_2$	• $\boldsymbol{E}$ ∥ [100] and $\boldsymbol{B}$ ∥ [100]
P_a2 (3.4) $\mathbb{Z}_2$	• $\boldsymbol{E}$ ∥ [100] and strain ⊥ [100]
P_ama2 (28.92) $\mathbb{Z}_1$	• $\boldsymbol{E}$ ∥ [100]
Pm' (6.20) $\mathbb{Z}_1$	• $\boldsymbol{E}$ ∥ [010] and $\boldsymbol{B}$ ∥ [010] and strain ⊥ [100]<br>• $\boldsymbol{E}$ ∥ [010] and $\boldsymbol{B}$ ∥ [001] and strain ⊥ [100]<br>• $\boldsymbol{E}$ ∥ [010] and $\boldsymbol{B}$ ⊥ [100]<br>• $\boldsymbol{E}$ ∥ [001] and $\boldsymbol{B}$ ∥ [010] and strain ⊥ [100]<br>• $\boldsymbol{E}$ ∥ [001] and $\boldsymbol{B}$ ∥ [001] and strain ⊥ [100]<br>• $\boldsymbol{E}$ ∥ [001] and $\boldsymbol{B}$ ⊥ [100]<br>• $\boldsymbol{E}$ ⊥ [100] and $\boldsymbol{B}$ ∥ [010]<br>• $\boldsymbol{E}$ ⊥ [100] and $\boldsymbol{B}$ ∥ [001]<br>• $\boldsymbol{E}$ ⊥ [100] and $\boldsymbol{B}$ ⊥ [100]
Pc (7.24) $\mathbb{Z}_1$	• $\boldsymbol{E}$ ∥ [010] and $\boldsymbol{B}$ ∥ [100] and strain ⊥ [100]<br>• $\boldsymbol{E}$ ∥ [001] and $\boldsymbol{B}$ ∥ [100] and strain ⊥ [100]<br>• $\boldsymbol{E}$ ⊥ [100] and $\boldsymbol{B}$ ∥ [100]
P_cc (7.28) $\mathbb{Z}_1$	• $\boldsymbol{E}$ ∥ [010] and strain ⊥ [100]<br>• $\boldsymbol{E}$ ∥ [001] and strain ⊥ [100]<br>• $\boldsymbol{E}$ ⊥ [100]
$P2'$ (3.3) $\mathbb{Z}_1$	• $\boldsymbol{E}$ ∥ [010] and $\boldsymbol{B}$ ∥ [100] and strain ⊥ [010]<br>• $\boldsymbol{E}$ ∥ [010] and $\boldsymbol{B}$ ∥ [001] and strain ⊥ [010]<br>• $\boldsymbol{E}$ ∥ [010] and $\boldsymbol{B}$ ⊥ [010]
$Pm'm2'$ (25.59) $\mathbb{Z}_1$	• $\boldsymbol{E}$ ∥ [010] and $\boldsymbol{B}$ ∥ [001]
$Pm'a2'$ (28.89) $\mathbb{Z}_1$	• $\boldsymbol{E}$ ∥ [010] and $\boldsymbol{B}$ ∥ [100]

Continued on next page

...continued

Subgroup & SI group	Perturbation
$P2$ (3.1) $\mathbb{Z}_2$	• $\boldsymbol{E} \parallel [010]$ and $\boldsymbol{B} \parallel [010]$ and strain $\perp [010]$
$Pm'm'2$ (25.60) $\mathbb{Z}_2$	• $\boldsymbol{E} \parallel [010]$ and $\boldsymbol{B} \parallel [010]$
P_a2 (3.4) $\mathbb{Z}_2$	• $\boldsymbol{E} \parallel [010]$ and strain $\perp [010]$
P_ama2 (28.92) $\mathbb{Z}_1$	• $\boldsymbol{E} \parallel [010]$
$P2$ (3.1) $\mathbb{Z}_2$	• $\boldsymbol{E} \parallel [001]$ and $\boldsymbol{B} \parallel [001]$ and strain $\perp [001]$
$Pm'm'2$ (25.60) $\mathbb{Z}_2$	• $\boldsymbol{E} \parallel [001]$ and $\boldsymbol{B} \parallel [001]$
$P2_1'$ (4.9) $\mathbb{Z}_1$	• $\boldsymbol{E} \parallel [001]$ and $\boldsymbol{B} \parallel [100]$ and strain $\perp [001]$<br>• $\boldsymbol{E} \parallel [001]$ and $\boldsymbol{B} \parallel [010]$ and strain $\perp [001]$<br>• $\boldsymbol{E} \parallel [001]$ and $\boldsymbol{B} \perp [001]$
$Pm'c2_1'$ (26.68) $\mathbb{Z}_1$	• $\boldsymbol{E} \parallel [001]$ and $\boldsymbol{B} \parallel [010]$
$Pm'c2_1'$ (26.68) $\mathbb{Z}_1$	• $\boldsymbol{E} \parallel [001]$ and $\boldsymbol{B} \parallel [100]$
P_b2 (3.5) $\mathbb{Z}_2$	• $\boldsymbol{E} \parallel [001]$ and strain $\perp [001]$
P_ccc2 (27.82) $\mathbb{Z}_2$	• $\boldsymbol{E} \parallel [001]$
$P\bar{1}$ (2.4) $\mathbb{Z}_2 \times \mathbb{Z}_2 \times \mathbb{Z}_2 \times \mathbb{Z}_4$	• $\boldsymbol{B} \parallel [100]$ and strain in generic direction<br>• $\boldsymbol{B} \parallel [010]$ and strain in generic direction<br>• $\boldsymbol{B} \parallel [001]$ and strain in generic direction<br>• $\boldsymbol{B} \perp [100]$ and strain $\perp [010]$<br>• $\boldsymbol{B} \perp [100]$ and strain $\perp [001]$<br>• $\boldsymbol{B} \perp [100]$ and strain in generic direction<br>• $\boldsymbol{B} \perp [010]$ and strain $\perp [100]$<br>• $\boldsymbol{B} \perp [010]$ and strain $\perp [001]$<br>• $\boldsymbol{B} \perp [010]$ and strain in generic direction<br>• $\boldsymbol{B} \perp [001]$ and strain $\perp [100]$<br>• $\boldsymbol{B} \perp [001]$ and strain $\perp [010]$<br>• $\boldsymbol{B} \perp [001]$ and strain in generic direction<br>• $\boldsymbol{B}$ in generic direction
$P2'/m'$ (10.46) $\mathbb{Z}_2 \times \mathbb{Z}_2 \times \mathbb{Z}_2 \times \mathbb{Z}_4$	• $\boldsymbol{B} \parallel [010]$ and strain $\perp [100]$<br>• $\boldsymbol{B} \parallel [001]$ and strain $\perp [100]$<br>• $\boldsymbol{B} \perp [100]$
$P2/c$ (13.65) $\mathbb{Z}_2 \times \mathbb{Z}_2$	• $\boldsymbol{B} \parallel [100]$ and strain $\perp [100]$
$P2'/m'$ (10.46) $\mathbb{Z}_2 \times \mathbb{Z}_2 \times \mathbb{Z}_2 \times \mathbb{Z}_4$	• $\boldsymbol{B} \parallel [100]$ and strain $\perp [010]$<br>• $\boldsymbol{B} \parallel [001]$ and strain $\perp [010]$<br>• $\boldsymbol{B} \perp [010]$
$P2/c$ (13.65) $\mathbb{Z}_2 \times \mathbb{Z}_2$	• $\boldsymbol{B} \parallel [010]$ and strain $\perp [010]$

Continued on next page

...continued

Subgroup & SI group	Perturbation
$P2/m$ (10.42) $\mathbb{Z}_2 \times \mathbb{Z}_2 \times \mathbb{Z}_2$	• $\boldsymbol{B}$ ∥ [001] and strain ⊥ [001]
$Pm'm'm$ (47.252) $\mathbb{Z}_2 \times \mathbb{Z}_2 \times \mathbb{Z}_2$	• $\boldsymbol{B}$ ∥ [001]
$P2_1'/m'$ (11.54) $\mathbb{Z}_2 \times \mathbb{Z}_2 \times \mathbb{Z}_4$	• $\boldsymbol{B}$ ∥ [100] and strain ⊥ [001]<br>• $\boldsymbol{B}$ ∥ [010] and strain ⊥ [001]<br>• $\boldsymbol{B}$ ⊥ [001]
$Pm'm'a$ (51.294) $\mathbb{Z}_2 \times \mathbb{Z}_2$	• $\boldsymbol{B}$ ∥ [100]
$Pm'm'a$ (51.294) $\mathbb{Z}_2 \times \mathbb{Z}_2$	• $\boldsymbol{B}$ ∥ [010]
$P_S\bar{1}$ (2.7) $\mathbb{Z}_2 \times \mathbb{Z}_2 \times \mathbb{Z}_4$	• strain in generic direction
P_c2/c (13.72) $\mathbb{Z}_2 \times \mathbb{Z}_2$	• strain ⊥ [100]
P_c2/c (13.72) $\mathbb{Z}_2 \times \mathbb{Z}_2$	• strain ⊥ [010]
P_b2/m (10.48) $\mathbb{Z}_2 \times \mathbb{Z}_2$	• strain ⊥ [001]

Table. S 384: Subgroups of P_Bccm (49.274) that can be realized by applying $\boldsymbol{E}/\boldsymbol{B}$ fields and/or uniaxial strains.

Subgroup & SI group	Perturbation
Pm (6.18) $\mathbb{Z}_1$	• $\boldsymbol{E}$ ∥ [100] and $\boldsymbol{B}$ ∥ [001] and strain ⊥ [001]<br>• $\boldsymbol{E}$ ∥ [010] and $\boldsymbol{B}$ ∥ [001] and strain ⊥ [001]<br>• $\boldsymbol{E}$ ⊥ [001] and $\boldsymbol{B}$ ∥ [001]
Pc (7.24) $\mathbb{Z}_1$	• $\boldsymbol{E}$ ∥ [100] and $\boldsymbol{B}$ ∥ [010] and strain ⊥ [010]<br>• $\boldsymbol{E}$ ∥ [001] and $\boldsymbol{B}$ ∥ [010] and strain ⊥ [010]<br>• $\boldsymbol{E}$ ⊥ [010] and $\boldsymbol{B}$ ∥ [010]
$P2$ (3.1) $\mathbb{Z}_2$	• $\boldsymbol{E}$ ∥ [100] and $\boldsymbol{B}$ ∥ [100] and strain ⊥ [100]

Continued on next page

...continued

Subgroup & SI group	Perturbation
P_S1 (1.3) $\mathbb{Z}_1$	• $\boldsymbol{E}$ ∥ [100] and strain in generic direction<br>• $\boldsymbol{E}$ ∥ [010] and strain in generic direction<br>• $\boldsymbol{E}$ ∥ [001] and strain in generic direction<br>• $\boldsymbol{E} \perp$ [100] and strain $\perp$ [010]<br>• $\boldsymbol{E} \perp$ [100] and strain $\perp$ [001]<br>• $\boldsymbol{E} \perp$ [100] and strain in generic direction<br>• $\boldsymbol{E} \perp$ [010] and strain $\perp$ [100]<br>• $\boldsymbol{E} \perp$ [010] and strain $\perp$ [001]<br>• $\boldsymbol{E} \perp$ [010] and strain in generic direction<br>• $\boldsymbol{E} \perp$ [001] and strain $\perp$ [100]<br>• $\boldsymbol{E} \perp$ [001] and strain $\perp$ [010]<br>• $\boldsymbol{E} \perp$ [001] and strain in generic direction<br>• $\boldsymbol{E}$ in generic direction
Pc' (7.26) $\mathbb{Z}_1$	• $\boldsymbol{E}$ ∥ [100] and $\boldsymbol{B}$ ∥ [100] and strain $\perp$ [001]<br>• $\boldsymbol{E}$ ∥ [100] and $\boldsymbol{B}$ ∥ [010] and strain $\perp$ [001]<br>• $\boldsymbol{E}$ ∥ [100] and $\boldsymbol{B} \perp$ [001]<br>• $\boldsymbol{E}$ ∥ [010] and $\boldsymbol{B}$ ∥ [100] and strain $\perp$ [001]<br>• $\boldsymbol{E}$ ∥ [010] and $\boldsymbol{B}$ ∥ [010] and strain $\perp$ [001]<br>• $\boldsymbol{E}$ ∥ [010] and $\boldsymbol{B} \perp$ [001]<br>• $\boldsymbol{E} \perp$ [001] and $\boldsymbol{B}$ ∥ [100]<br>• $\boldsymbol{E} \perp$ [001] and $\boldsymbol{B}$ ∥ [010]<br>• $\boldsymbol{E} \perp$ [001] and $\boldsymbol{B} \perp$ [001]
P_Cm (6.23) $\mathbb{Z}_1$	• $\boldsymbol{E}$ ∥ [100] and strain $\perp$ [001]<br>• $\boldsymbol{E}$ ∥ [010] and strain $\perp$ [001]<br>• $\boldsymbol{E} \perp$ [001]
Pc' (7.26) $\mathbb{Z}_1$	• $\boldsymbol{E}$ ∥ [100] and $\boldsymbol{B}$ ∥ [100] and strain $\perp$ [010]<br>• $\boldsymbol{E}$ ∥ [100] and $\boldsymbol{B}$ ∥ [001] and strain $\perp$ [010]<br>• $\boldsymbol{E}$ ∥ [100] and $\boldsymbol{B} \perp$ [010]<br>• $\boldsymbol{E}$ ∥ [001] and $\boldsymbol{B}$ ∥ [100] and strain $\perp$ [010]<br>• $\boldsymbol{E}$ ∥ [001] and $\boldsymbol{B}$ ∥ [001] and strain $\perp$ [010]<br>• $\boldsymbol{E}$ ∥ [001] and $\boldsymbol{B} \perp$ [010]<br>• $\boldsymbol{E} \perp$ [010] and $\boldsymbol{B}$ ∥ [100]<br>• $\boldsymbol{E} \perp$ [010] and $\boldsymbol{B}$ ∥ [001]<br>• $\boldsymbol{E} \perp$ [010] and $\boldsymbol{B} \perp$ [010]
P_ac (7.27) $\mathbb{Z}_1$	• $\boldsymbol{E}$ ∥ [100] and strain $\perp$ [010]<br>• $\boldsymbol{E}$ ∥ [001] and strain $\perp$ [010]<br>• $\boldsymbol{E} \perp$ [010]
$Pc'c'2$ (27.81) $\mathbb{Z}_2$	• $\boldsymbol{E}$ ∥ [100] and $\boldsymbol{B}$ ∥ [100]

Continued on next page

...continued

Subgroup & SI group	Perturbation
$P2_1'$ (4.9) $\mathbb{Z}_1$	• $\boldsymbol{E} \parallel [100]$ and $\boldsymbol{B} \parallel [010]$ and strain $\perp [100]$<br>• $\boldsymbol{E} \parallel [100]$ and $\boldsymbol{B} \parallel [001]$ and strain $\perp [100]$<br>• $\boldsymbol{E} \parallel [100]$ and $\boldsymbol{B} \perp [100]$
P_C2 (3.6) $\mathbb{Z}_1$	• $\boldsymbol{E} \parallel [100]$ and strain $\perp [100]$
$Pc'a2_1'$ (29.101) $\mathbb{Z}_1$	• $\boldsymbol{E} \parallel [100]$ and $\boldsymbol{B} \parallel [010]$
$Pmc'2_1'$ (26.69) $\mathbb{Z}_1$	• $\boldsymbol{E} \parallel [100]$ and $\boldsymbol{B} \parallel [001]$
P_Bma2 (28.96) $\mathbb{Z}_1$	• $\boldsymbol{E} \parallel [100]$
Pc (7.24) $\mathbb{Z}_1$	• $\boldsymbol{E} \parallel [010]$ and $\boldsymbol{B} \parallel [100]$ and strain $\perp [100]$<br>• $\boldsymbol{E} \parallel [001]$ and $\boldsymbol{B} \parallel [100]$ and strain $\perp [100]$<br>• $\boldsymbol{E} \perp [100]$ and $\boldsymbol{B} \parallel [100]$
$P2$ (3.1) $\mathbb{Z}_2$	• $\boldsymbol{E} \parallel [010]$ and $\boldsymbol{B} \parallel [010]$ and strain $\perp [010]$
$P2$ (3.1) $\mathbb{Z}_2$	• $\boldsymbol{E} \parallel [001]$ and $\boldsymbol{B} \parallel [001]$ and strain $\perp [001]$
$P\bar{1}$ (2.4) $\mathbb{Z}_2 \times \mathbb{Z}_2 \times \mathbb{Z}_2 \times \mathbb{Z}_4$	• $\boldsymbol{B} \parallel [100]$ and strain in generic direction<br>• $\boldsymbol{B} \parallel [010]$ and strain in generic direction<br>• $\boldsymbol{B} \parallel [001]$ and strain in generic direction<br>• $\boldsymbol{B} \perp [100]$ and strain $\perp [010]$<br>• $\boldsymbol{B} \perp [100]$ and strain $\perp [001]$<br>• $\boldsymbol{B} \perp [100]$ and strain in generic direction<br>• $\boldsymbol{B} \perp [010]$ and strain $\perp [100]$<br>• $\boldsymbol{B} \perp [010]$ and strain $\perp [001]$<br>• $\boldsymbol{B} \perp [010]$ and strain in generic direction<br>• $\boldsymbol{B} \perp [001]$ and strain $\perp [100]$<br>• $\boldsymbol{B} \perp [001]$ and strain $\perp [010]$<br>• $\boldsymbol{B} \perp [001]$ and strain in generic direction<br>• $\boldsymbol{B}$ in generic direction
$P2/c$ (13.65) $\mathbb{Z}_2 \times \mathbb{Z}_2$	• $\boldsymbol{B} \parallel [100]$ and strain $\perp [100]$
$P2/c$ (13.65) $\mathbb{Z}_2 \times \mathbb{Z}_2$	• $\boldsymbol{B} \parallel [010]$ and strain $\perp [010]$
$P2/m$ (10.42) $\mathbb{Z}_2 \times \mathbb{Z}_2 \times \mathbb{Z}_2$	• $\boldsymbol{B} \parallel [001]$ and strain $\perp [001]$

Continued on next page

...continued

Subgroup & SI group	Perturbation
Pm' (6.20) $\mathbb{Z}_1$	• $\boldsymbol{E}$ ∥ [010] and $\boldsymbol{B}$ ∥ [010] and strain ⊥ [100]<br>• $\boldsymbol{E}$ ∥ [010] and $\boldsymbol{B}$ ∥ [001] and strain ⊥ [100]<br>• $\boldsymbol{E}$ ∥ [010] and $\boldsymbol{B}$ ⊥ [100]<br>• $\boldsymbol{E}$ ∥ [001] and $\boldsymbol{B}$ ∥ [010] and strain ⊥ [100]<br>• $\boldsymbol{E}$ ∥ [001] and $\boldsymbol{B}$ ∥ [001] and strain ⊥ [100]<br>• $\boldsymbol{E}$ ∥ [001] and $\boldsymbol{B}$ ⊥ [100]<br>• $\boldsymbol{E}$ ⊥ [100] and $\boldsymbol{B}$ ∥ [010]<br>• $\boldsymbol{E}$ ⊥ [100] and $\boldsymbol{B}$ ∥ [001]<br>• $\boldsymbol{E}$ ⊥ [100] and $\boldsymbol{B}$ ⊥ [100]
P_Ac (7.31) $\mathbb{Z}_1$	• $\boldsymbol{E}$ ∥ [010] and strain ⊥ [100]<br>• $\boldsymbol{E}$ ∥ [001] and strain ⊥ [100]<br>• $\boldsymbol{E}$ ⊥ [100]
$Pm'a'2$ (28.91) $\mathbb{Z}_2$	• $\boldsymbol{E}$ ∥ [010] and $\boldsymbol{B}$ ∥ [010]
$Pm'a'2$ (28.91) $\mathbb{Z}_2$	• $\boldsymbol{E}$ ∥ [001] and $\boldsymbol{B}$ ∥ [001]
$P2_1'/m'$ (11.54) $\mathbb{Z}_2 \times \mathbb{Z}_2 \times \mathbb{Z}_4$	• $\boldsymbol{B}$ ∥ [010] and strain ⊥ [100]<br>• $\boldsymbol{B}$ ∥ [001] and strain ⊥ [100]<br>• $\boldsymbol{B}$ ⊥ [100]
$P2'$ (3.3) $\mathbb{Z}_1$	• $\boldsymbol{E}$ ∥ [010] and $\boldsymbol{B}$ ∥ [100] and strain ⊥ [010]<br>• $\boldsymbol{E}$ ∥ [010] and $\boldsymbol{B}$ ∥ [001] and strain ⊥ [010]<br>• $\boldsymbol{E}$ ∥ [010] and $\boldsymbol{B}$ ⊥ [010]
$Pb'a2'$ (32.137) $\mathbb{Z}_1$	• $\boldsymbol{E}$ ∥ [010] and $\boldsymbol{B}$ ∥ [100]
P_a2 (3.4) $\mathbb{Z}_2$	• $\boldsymbol{E}$ ∥ [010] and strain ⊥ [010]
$P2'/c'$ (13.69) $\mathbb{Z}_2 \times \mathbb{Z}_2 \times \mathbb{Z}_4$	• $\boldsymbol{B}$ ∥ [100] and strain ⊥ [010]<br>• $\boldsymbol{B}$ ∥ [001] and strain ⊥ [010]<br>• $\boldsymbol{B}$ ⊥ [010]
$Pm'm2'$ (25.59) $\mathbb{Z}_1$	• $\boldsymbol{E}$ ∥ [010] and $\boldsymbol{B}$ ∥ [001]
P_Cma2 (28.97) $\mathbb{Z}_1$	• $\boldsymbol{E}$ ∥ [010]
$Pm'ma'$ (51.296) $\mathbb{Z}_2 \times \mathbb{Z}_2 \times \mathbb{Z}_2$	• $\boldsymbol{B}$ ∥ [001]
$P2_1'$ (4.9) $\mathbb{Z}_1$	• $\boldsymbol{E}$ ∥ [001] and $\boldsymbol{B}$ ∥ [100] and strain ⊥ [001]<br>• $\boldsymbol{E}$ ∥ [001] and $\boldsymbol{B}$ ∥ [010] and strain ⊥ [001]<br>• $\boldsymbol{E}$ ∥ [001] and $\boldsymbol{B}$ ⊥ [001]
$Pca'2_1'$ (29.102) $\mathbb{Z}_1$	• $\boldsymbol{E}$ ∥ [001] and $\boldsymbol{B}$ ∥ [100]
P_C2 (3.6) $\mathbb{Z}_1$	• $\boldsymbol{E}$ ∥ [001] and strain ⊥ [001]

Continued on next page

. . . continued

Subgroup & SI group	Perturbation
$P2_1'/c'$ (14.79) $\mathbb{Z}_2 \times \mathbb{Z}_4$	• $\boldsymbol{B} \parallel [100]$ and strain $\perp [001]$<br>• $\boldsymbol{B} \parallel [010]$ and strain $\perp [001]$<br>• $\boldsymbol{B} \perp [001]$
$Pm'c2_1'$ (26.68) $\mathbb{Z}_1$	• $\boldsymbol{E} \parallel [001]$ and $\boldsymbol{B} \parallel [010]$
P_Acc2 (27.85) $\mathbb{Z}_2$	• $\boldsymbol{E} \parallel [001]$
$Pbc'm'$ (57.383) $\mathbb{Z}_2 \times \mathbb{Z}_2$	• $\boldsymbol{B} \parallel [010]$
$Pc'c'a$ (54.342) $\mathbb{Z}_2 \times \mathbb{Z}_2$	• $\boldsymbol{B} \parallel [100]$
$P_S\bar{1}$ (2.7) $\mathbb{Z}_2 \times \mathbb{Z}_2 \times \mathbb{Z}_4$	• strain in generic direction
P_A2/c (13.73) $\mathbb{Z}_2$	• strain $\perp [100]$
P_a2/c (13.70) $\mathbb{Z}_2 \times \mathbb{Z}_2$	• strain $\perp [010]$
P_C2/m (10.49) $\mathbb{Z}_2 \times \mathbb{Z}_2$	• strain $\perp [001]$

Table. S 385: Subgroups of P_Cccm (49.275) that can be realized by applying $\boldsymbol{E}/\boldsymbol{B}$ fields and/or uniaxial strains.

Subgroup & SI group	Perturbation
Pm (6.18) $\mathbb{Z}_1$	• $\boldsymbol{E} \parallel [100]$ and $\boldsymbol{B} \parallel [001]$ and strain $\perp [001]$<br>• $\boldsymbol{E} \parallel [010]$ and $\boldsymbol{B} \parallel [001]$ and strain $\perp [001]$<br>• $\boldsymbol{E} \perp [001]$ and $\boldsymbol{B} \parallel [001]$
Pc (7.24) $\mathbb{Z}_1$	• $\boldsymbol{E} \parallel [100]$ and $\boldsymbol{B} \parallel [010]$ and strain $\perp [010]$<br>• $\boldsymbol{E} \parallel [001]$ and $\boldsymbol{B} \parallel [010]$ and strain $\perp [010]$<br>• $\boldsymbol{E} \perp [010]$ and $\boldsymbol{B} \parallel [010]$
$P2$ (3.1) $\mathbb{Z}_2$	• $\boldsymbol{E} \parallel [100]$ and $\boldsymbol{B} \parallel [100]$ and strain $\perp [100]$

Continued on next page

...continued

Subgroup & SI group	Perturbation
P_S1 (1.3) $\mathbb{Z}_1$	• $\boldsymbol{E}$ ∥ [100] and strain in generic direction<br>• $\boldsymbol{E}$ ∥ [010] and strain in generic direction<br>• $\boldsymbol{E}$ ∥ [001] and strain in generic direction<br>• $\boldsymbol{E} \perp$ [100] and strain $\perp$ [010]<br>• $\boldsymbol{E} \perp$ [100] and strain $\perp$ [001]<br>• $\boldsymbol{E} \perp$ [100] and strain in generic direction<br>• $\boldsymbol{E} \perp$ [010] and strain $\perp$ [100]<br>• $\boldsymbol{E} \perp$ [010] and strain $\perp$ [001]<br>• $\boldsymbol{E} \perp$ [010] and strain in generic direction<br>• $\boldsymbol{E} \perp$ [001] and strain $\perp$ [100]<br>• $\boldsymbol{E} \perp$ [001] and strain $\perp$ [010]<br>• $\boldsymbol{E} \perp$ [001] and strain in generic direction<br>• $\boldsymbol{E}$ in generic direction
Pc' (7.26) $\mathbb{Z}_1$	• $\boldsymbol{E}$ ∥ [100] and $\boldsymbol{B}$ ∥ [100] and strain $\perp$ [001]<br>• $\boldsymbol{E}$ ∥ [100] and $\boldsymbol{B}$ ∥ [010] and strain $\perp$ [001]<br>• $\boldsymbol{E}$ ∥ [100] and $\boldsymbol{B} \perp$ [001]<br>• $\boldsymbol{E}$ ∥ [010] and $\boldsymbol{B}$ ∥ [100] and strain $\perp$ [001]<br>• $\boldsymbol{E}$ ∥ [010] and $\boldsymbol{B}$ ∥ [010] and strain $\perp$ [001]<br>• $\boldsymbol{E}$ ∥ [010] and $\boldsymbol{B} \perp$ [001]<br>• $\boldsymbol{E} \perp$ [001] and $\boldsymbol{B}$ ∥ [100]<br>• $\boldsymbol{E} \perp$ [001] and $\boldsymbol{B}$ ∥ [010]<br>• $\boldsymbol{E} \perp$ [001] and $\boldsymbol{B} \perp$ [001]
P_am (6.21) $\mathbb{Z}_1$	• $\boldsymbol{E}$ ∥ [100] and strain $\perp$ [001]<br>• $\boldsymbol{E}$ ∥ [010] and strain $\perp$ [001]<br>• $\boldsymbol{E} \perp$ [001]
Pc' (7.26) $\mathbb{Z}_1$	• $\boldsymbol{E}$ ∥ [100] and $\boldsymbol{B}$ ∥ [100] and strain $\perp$ [010]<br>• $\boldsymbol{E}$ ∥ [100] and $\boldsymbol{B}$ ∥ [001] and strain $\perp$ [010]<br>• $\boldsymbol{E}$ ∥ [100] and $\boldsymbol{B} \perp$ [010]<br>• $\boldsymbol{E}$ ∥ [001] and $\boldsymbol{B}$ ∥ [100] and strain $\perp$ [010]<br>• $\boldsymbol{E}$ ∥ [001] and $\boldsymbol{B}$ ∥ [001] and strain $\perp$ [010]<br>• $\boldsymbol{E}$ ∥ [001] and $\boldsymbol{B} \perp$ [010]<br>• $\boldsymbol{E} \perp$ [010] and $\boldsymbol{B}$ ∥ [100]<br>• $\boldsymbol{E} \perp$ [010] and $\boldsymbol{B}$ ∥ [001]<br>• $\boldsymbol{E} \perp$ [010] and $\boldsymbol{B} \perp$ [010]
P_Cc (7.30) $\mathbb{Z}_1$	• $\boldsymbol{E}$ ∥ [100] and strain $\perp$ [010]<br>• $\boldsymbol{E}$ ∥ [001] and strain $\perp$ [010]<br>• $\boldsymbol{E} \perp$ [010]
$Pn'n'2$ (34.159) $\mathbb{Z}_1$	• $\boldsymbol{E}$ ∥ [100] and $\boldsymbol{B}$ ∥ [100]

Continued on next page

...continued

Subgroup & SI group	Perturbation
$P2_1'$ (4.9) $\mathbb{Z}_1$	• $\boldsymbol{E} \parallel [100]$ and $\boldsymbol{B} \parallel [010]$ and strain $\perp [100]$<br>• $\boldsymbol{E} \parallel [100]$ and $\boldsymbol{B} \parallel [001]$ and strain $\perp [100]$<br>• $\boldsymbol{E} \parallel [100]$ and $\boldsymbol{B} \perp [100]$
P_C2 (3.6) $\mathbb{Z}_1$	• $\boldsymbol{E} \parallel [100]$ and strain $\perp [100]$
$Pn'a2_1'$ (33.146) $\mathbb{Z}_1$	• $\boldsymbol{E} \parallel [100]$ and $\boldsymbol{B} \parallel [010]$
$Pmn'2_1'$ (31.126) $\mathbb{Z}_1$	• $\boldsymbol{E} \parallel [100]$ and $\boldsymbol{B} \parallel [001]$
P_Ama2 (28.95) $\mathbb{Z}_1$	• $\boldsymbol{E} \parallel [100]$
Pc (7.24) $\mathbb{Z}_1$	• $\boldsymbol{E} \parallel [010]$ and $\boldsymbol{B} \parallel [100]$ and strain $\perp [100]$<br>• $\boldsymbol{E} \parallel [001]$ and $\boldsymbol{B} \parallel [100]$ and strain $\perp [100]$<br>• $\boldsymbol{E} \perp [100]$ and $\boldsymbol{B} \parallel [100]$
$P2$ (3.1) $\mathbb{Z}_2$	• $\boldsymbol{E} \parallel [010]$ and $\boldsymbol{B} \parallel [010]$ and strain $\perp [010]$
$P2$ (3.1) $\mathbb{Z}_2$	• $\boldsymbol{E} \parallel [001]$ and $\boldsymbol{B} \parallel [001]$ and strain $\perp [001]$
$P\bar{1}$ (2.4) $\mathbb{Z}_2 \times \mathbb{Z}_2 \times \mathbb{Z}_2 \times \mathbb{Z}_4$	• $\boldsymbol{B} \parallel [100]$ and strain in generic direction<br>• $\boldsymbol{B} \parallel [010]$ and strain in generic direction<br>• $\boldsymbol{B} \parallel [001]$ and strain in generic direction<br>• $\boldsymbol{B} \perp [100]$ and strain $\perp [010]$<br>• $\boldsymbol{B} \perp [100]$ and strain $\perp [001]$<br>• $\boldsymbol{B} \perp [100]$ and strain in generic direction<br>• $\boldsymbol{B} \perp [010]$ and strain $\perp [100]$<br>• $\boldsymbol{B} \perp [010]$ and strain $\perp [001]$<br>• $\boldsymbol{B} \perp [010]$ and strain in generic direction<br>• $\boldsymbol{B} \perp [001]$ and strain $\perp [100]$<br>• $\boldsymbol{B} \perp [001]$ and strain $\perp [010]$<br>• $\boldsymbol{B} \perp [001]$ and strain in generic direction<br>• $\boldsymbol{B}$ in generic direction
$P2/c$ (13.65) $\mathbb{Z}_2 \times \mathbb{Z}_2$	• $\boldsymbol{B} \parallel [100]$ and strain $\perp [100]$
$P2/c$ (13.65) $\mathbb{Z}_2 \times \mathbb{Z}_2$	• $\boldsymbol{B} \parallel [010]$ and strain $\perp [010]$
$P2/m$ (10.42) $\mathbb{Z}_2 \times \mathbb{Z}_2 \times \mathbb{Z}_2$	• $\boldsymbol{B} \parallel [001]$ and strain $\perp [001]$

Continued on next page

...continued

Subgroup & SI group	Perturbation
Pc' (7.26) $\mathbb{Z}_1$	• $\boldsymbol{E}$ ∥ [010] and $\boldsymbol{B}$ ∥ [010] and strain ⊥ [100]<br>• $\boldsymbol{E}$ ∥ [010] and $\boldsymbol{B}$ ∥ [001] and strain ⊥ [100]<br>• $\boldsymbol{E}$ ∥ [010] and $\boldsymbol{B}$ ⊥ [100]<br>• $\boldsymbol{E}$ ∥ [001] and $\boldsymbol{B}$ ∥ [010] and strain ⊥ [100]<br>• $\boldsymbol{E}$ ∥ [001] and $\boldsymbol{B}$ ∥ [001] and strain ⊥ [100]<br>• $\boldsymbol{E}$ ∥ [001] and $\boldsymbol{B}$ ⊥ [100]<br>• $\boldsymbol{E}$ ⊥ [100] and $\boldsymbol{B}$ ∥ [010]<br>• $\boldsymbol{E}$ ⊥ [100] and $\boldsymbol{B}$ ∥ [001]<br>• $\boldsymbol{E}$ ⊥ [100] and $\boldsymbol{B}$ ⊥ [100]
P_Cc (7.30) $\mathbb{Z}_1$	• $\boldsymbol{E}$ ∥ [010] and strain ⊥ [100]<br>• $\boldsymbol{E}$ ∥ [001] and strain ⊥ [100]<br>• $\boldsymbol{E}$ ⊥ [100]
$Pn'n'2$ (34.159) $\mathbb{Z}_1$	• $\boldsymbol{E}$ ∥ [010] and $\boldsymbol{B}$ ∥ [010]
$Pn'n'2$ (34.159) $\mathbb{Z}_1$	• $\boldsymbol{E}$ ∥ [001] and $\boldsymbol{B}$ ∥ [001]
$P2_1'/c'$ (14.79) $\mathbb{Z}_2 \times \mathbb{Z}_4$	• $\boldsymbol{B}$ ∥ [010] and strain ⊥ [100]<br>• $\boldsymbol{B}$ ∥ [001] and strain ⊥ [100]<br>• $\boldsymbol{B}$ ⊥ [100]
$P2_1'$ (4.9) $\mathbb{Z}_1$	• $\boldsymbol{E}$ ∥ [010] and $\boldsymbol{B}$ ∥ [100] and strain ⊥ [010]<br>• $\boldsymbol{E}$ ∥ [010] and $\boldsymbol{B}$ ∥ [001] and strain ⊥ [010]<br>• $\boldsymbol{E}$ ∥ [010] and $\boldsymbol{B}$ ⊥ [010]
$Pn'a2_1'$ (33.146) $\mathbb{Z}_1$	• $\boldsymbol{E}$ ∥ [010] and $\boldsymbol{B}$ ∥ [100]
P_C2 (3.6) $\mathbb{Z}_1$	• $\boldsymbol{E}$ ∥ [010] and strain ⊥ [010]
$P2_1'/c'$ (14.79) $\mathbb{Z}_2 \times \mathbb{Z}_4$	• $\boldsymbol{B}$ ∥ [100] and strain ⊥ [010]<br>• $\boldsymbol{B}$ ∥ [001] and strain ⊥ [010]<br>• $\boldsymbol{B}$ ⊥ [010]
$Pmn'2_1'$ (31.126) $\mathbb{Z}_1$	• $\boldsymbol{E}$ ∥ [010] and $\boldsymbol{B}$ ∥ [001]
P_Ama2 (28.95) $\mathbb{Z}_1$	• $\boldsymbol{E}$ ∥ [010]
$Pn'n'm$ (58.397) $\mathbb{Z}_2 \times \mathbb{Z}_2$	• $\boldsymbol{B}$ ∥ [001]
$P2'$ (3.3) $\mathbb{Z}_1$	• $\boldsymbol{E}$ ∥ [001] and $\boldsymbol{B}$ ∥ [100] and strain ⊥ [001]<br>• $\boldsymbol{E}$ ∥ [001] and $\boldsymbol{B}$ ∥ [010] and strain ⊥ [001]<br>• $\boldsymbol{E}$ ∥ [001] and $\boldsymbol{B}$ ⊥ [001]
$Pn'c2'$ (30.113) $\mathbb{Z}_1$	• $\boldsymbol{E}$ ∥ [001] and $\boldsymbol{B}$ ∥ [100]
P_a2 (3.4) $\mathbb{Z}_2$	• $\boldsymbol{E}$ ∥ [001] and strain ⊥ [001]

Continued on next page

...continued

Subgroup & SI group	Perturbation
$P2'/c'$ (13.69) $\mathbb{Z}_2 \times \mathbb{Z}_2 \times \mathbb{Z}_4$	• $\boldsymbol{B} \parallel [100]$ and strain $\perp [001]$<br>• $\boldsymbol{B} \parallel [010]$ and strain $\perp [001]$<br>• $\boldsymbol{B} \perp [001]$
$Pn'c2'$ (30.113) $\mathbb{Z}_1$	• $\boldsymbol{E} \parallel [001]$ and $\boldsymbol{B} \parallel [010]$
P_Ccc2 (27.84) $\mathbb{Z}_2$	• $\boldsymbol{E} \parallel [001]$
$Pn'n'a$ (52.310) $\mathbb{Z}_2$	• $\boldsymbol{B} \parallel [010]$
$Pn'n'a$ (52.310) $\mathbb{Z}_2$	• $\boldsymbol{B} \parallel [100]$
$P_S\bar{1}$ (2.7) $\mathbb{Z}_2 \times \mathbb{Z}_2 \times \mathbb{Z}_4$	• strain in generic direction
P_C2/c (13.74) $\mathbb{Z}_2$	• strain $\perp [100]$
P_C2/c (13.74) $\mathbb{Z}_2$	• strain $\perp [010]$
P_a2/m (10.47) $\mathbb{Z}_2 \times \mathbb{Z}_2 \times \mathbb{Z}_2$	• strain $\perp [001]$

Table. S 386: Subgroups of P_Iccm (49.276) that can be realized by applying $\boldsymbol{E}/\boldsymbol{B}$ fields and/or uniaxial strains.

Subgroup & SI group	Perturbation
Pm (6.18) $\mathbb{Z}_1$	• $\boldsymbol{E} \parallel [100]$ and $\boldsymbol{B} \parallel [001]$ and strain $\perp [001]$<br>• $\boldsymbol{E} \parallel [010]$ and $\boldsymbol{B} \parallel [001]$ and strain $\perp [001]$<br>• $\boldsymbol{E} \perp [001]$ and $\boldsymbol{B} \parallel [001]$
Pc (7.24) $\mathbb{Z}_1$	• $\boldsymbol{E} \parallel [100]$ and $\boldsymbol{B} \parallel [010]$ and strain $\perp [010]$<br>• $\boldsymbol{E} \parallel [001]$ and $\boldsymbol{B} \parallel [010]$ and strain $\perp [010]$<br>• $\boldsymbol{E} \perp [010]$ and $\boldsymbol{B} \parallel [010]$
$P2$ (3.1) $\mathbb{Z}_2$	• $\boldsymbol{E} \parallel [100]$ and $\boldsymbol{B} \parallel [100]$ and strain $\perp [100]$

Continued on next page

…continued

Subgroup & SI group	Perturbation
P_S1 (1.3) $\mathbb{Z}_1$	• $\boldsymbol{E}$ ∥ [100] and strain in generic direction<br>• $\boldsymbol{E}$ ∥ [010] and strain in generic direction<br>• $\boldsymbol{E}$ ∥ [001] and strain in generic direction<br>• $\boldsymbol{E} \perp$ [100] and strain $\perp$ [010]<br>• $\boldsymbol{E} \perp$ [100] and strain $\perp$ [001]<br>• $\boldsymbol{E} \perp$ [100] and strain in generic direction<br>• $\boldsymbol{E} \perp$ [010] and strain $\perp$ [100]<br>• $\boldsymbol{E} \perp$ [010] and strain $\perp$ [001]<br>• $\boldsymbol{E} \perp$ [010] and strain in generic direction<br>• $\boldsymbol{E} \perp$ [001] and strain $\perp$ [100]<br>• $\boldsymbol{E} \perp$ [001] and strain $\perp$ [010]<br>• $\boldsymbol{E} \perp$ [001] and strain in generic direction<br>• $\boldsymbol{E}$ in generic direction
Pc' (7.26) $\mathbb{Z}_1$	• $\boldsymbol{E}$ ∥ [100] and $\boldsymbol{B}$ ∥ [100] and strain $\perp$ [001]<br>• $\boldsymbol{E}$ ∥ [100] and $\boldsymbol{B}$ ∥ [010] and strain $\perp$ [001]<br>• $\boldsymbol{E}$ ∥ [100] and $\boldsymbol{B} \perp$ [001]<br>• $\boldsymbol{E}$ ∥ [010] and $\boldsymbol{B}$ ∥ [100] and strain $\perp$ [001]<br>• $\boldsymbol{E}$ ∥ [010] and $\boldsymbol{B}$ ∥ [010] and strain $\perp$ [001]<br>• $\boldsymbol{E}$ ∥ [010] and $\boldsymbol{B} \perp$ [001]<br>• $\boldsymbol{E} \perp$ [001] and $\boldsymbol{B}$ ∥ [100]<br>• $\boldsymbol{E} \perp$ [001] and $\boldsymbol{B}$ ∥ [010]<br>• $\boldsymbol{E} \perp$ [001] and $\boldsymbol{B} \perp$ [001]
P_Cm (6.23) $\mathbb{Z}_1$	• $\boldsymbol{E}$ ∥ [100] and strain $\perp$ [001]<br>• $\boldsymbol{E}$ ∥ [010] and strain $\perp$ [001]<br>• $\boldsymbol{E} \perp$ [001]
Pc' (7.26) $\mathbb{Z}_1$	• $\boldsymbol{E}$ ∥ [100] and $\boldsymbol{B}$ ∥ [100] and strain $\perp$ [010]<br>• $\boldsymbol{E}$ ∥ [100] and $\boldsymbol{B}$ ∥ [001] and strain $\perp$ [010]<br>• $\boldsymbol{E}$ ∥ [100] and $\boldsymbol{B} \perp$ [010]<br>• $\boldsymbol{E}$ ∥ [001] and $\boldsymbol{B}$ ∥ [100] and strain $\perp$ [010]<br>• $\boldsymbol{E}$ ∥ [001] and $\boldsymbol{B}$ ∥ [001] and strain $\perp$ [010]<br>• $\boldsymbol{E}$ ∥ [001] and $\boldsymbol{B} \perp$ [010]<br>• $\boldsymbol{E} \perp$ [010] and $\boldsymbol{B}$ ∥ [100]<br>• $\boldsymbol{E} \perp$ [010] and $\boldsymbol{B}$ ∥ [001]<br>• $\boldsymbol{E} \perp$ [010] and $\boldsymbol{B} \perp$ [010]
P_Cc (7.30) $\mathbb{Z}_1$	• $\boldsymbol{E}$ ∥ [100] and strain $\perp$ [010]<br>• $\boldsymbol{E}$ ∥ [001] and strain $\perp$ [010]<br>• $\boldsymbol{E} \perp$ [010]
$Pn'c'2$ (30.115) $\mathbb{Z}_1$	• $\boldsymbol{E}$ ∥ [100] and $\boldsymbol{B}$ ∥ [100]

Continued on next page

. . . continued

Subgroup & SI group	Perturbation
$P2_1'$ (4.9) $\mathbb{Z}_1$	• $\boldsymbol{E} \parallel [100]$ and $\boldsymbol{B} \parallel [010]$ and strain $\perp [100]$<br>• $\boldsymbol{E} \parallel [100]$ and $\boldsymbol{B} \parallel [001]$ and strain $\perp [100]$<br>• $\boldsymbol{E} \parallel [100]$ and $\boldsymbol{B} \perp [100]$
P_C2 (3.6) $\mathbb{Z}_1$	• $\boldsymbol{E} \parallel [100]$ and strain $\perp [100]$
$Pn'a2_1'$ (33.146) $\mathbb{Z}_1$	• $\boldsymbol{E} \parallel [100]$ and $\boldsymbol{B} \parallel [010]$
$Pmc'2_1'$ (26.69) $\mathbb{Z}_1$	• $\boldsymbol{E} \parallel [100]$ and $\boldsymbol{B} \parallel [001]$
P_Ima2 (28.98) $\mathbb{Z}_1$	• $\boldsymbol{E} \parallel [100]$
Pc (7.24) $\mathbb{Z}_1$	• $\boldsymbol{E} \parallel [010]$ and $\boldsymbol{B} \parallel [100]$ and strain $\perp [100]$<br>• $\boldsymbol{E} \parallel [001]$ and $\boldsymbol{B} \parallel [100]$ and strain $\perp [100]$<br>• $\boldsymbol{E} \perp [100]$ and $\boldsymbol{B} \parallel [100]$
$P2$ (3.1) $\mathbb{Z}_2$	• $\boldsymbol{E} \parallel [010]$ and $\boldsymbol{B} \parallel [010]$ and strain $\perp [010]$
$P2$ (3.1) $\mathbb{Z}_2$	• $\boldsymbol{E} \parallel [001]$ and $\boldsymbol{B} \parallel [001]$ and strain $\perp [001]$
$P\bar{1}$ (2.4) $\mathbb{Z}_2 \times \mathbb{Z}_2 \times \mathbb{Z}_2 \times \mathbb{Z}_4$	• $\boldsymbol{B} \parallel [100]$ and strain in generic direction<br>• $\boldsymbol{B} \parallel [010]$ and strain in generic direction<br>• $\boldsymbol{B} \parallel [001]$ and strain in generic direction<br>• $\boldsymbol{B} \perp [100]$ and strain $\perp [010]$<br>• $\boldsymbol{B} \perp [100]$ and strain $\perp [001]$<br>• $\boldsymbol{B} \perp [100]$ and strain in generic direction<br>• $\boldsymbol{B} \perp [010]$ and strain $\perp [100]$<br>• $\boldsymbol{B} \perp [010]$ and strain $\perp [001]$<br>• $\boldsymbol{B} \perp [010]$ and strain in generic direction<br>• $\boldsymbol{B} \perp [001]$ and strain $\perp [100]$<br>• $\boldsymbol{B} \perp [001]$ and strain $\perp [010]$<br>• $\boldsymbol{B} \perp [001]$ and strain in generic direction<br>• $\boldsymbol{B}$ in generic direction
$P2/c$ (13.65) $\mathbb{Z}_2 \times \mathbb{Z}_2$	• $\boldsymbol{B} \parallel [100]$ and strain $\perp [100]$
$P2/c$ (13.65) $\mathbb{Z}_2 \times \mathbb{Z}_2$	• $\boldsymbol{B} \parallel [010]$ and strain $\perp [010]$
$P2/m$ (10.42) $\mathbb{Z}_2 \times \mathbb{Z}_2 \times \mathbb{Z}_2$	• $\boldsymbol{B} \parallel [001]$ and strain $\perp [001]$

Continued on next page

...continued

Subgroup & SI group	Perturbation
Pc' (7.26) $\mathbb{Z}_1$	• $\boldsymbol{E}$ ∥ [010] and $\boldsymbol{B}$ ∥ [010] and strain ⊥ [100]<br>• $\boldsymbol{E}$ ∥ [010] and $\boldsymbol{B}$ ∥ [001] and strain ⊥ [100]<br>• $\boldsymbol{E}$ ∥ [010] and $\boldsymbol{B}$ ⊥ [100]<br>• $\boldsymbol{E}$ ∥ [001] and $\boldsymbol{B}$ ∥ [010] and strain ⊥ [100]<br>• $\boldsymbol{E}$ ∥ [001] and $\boldsymbol{B}$ ∥ [001] and strain ⊥ [100]<br>• $\boldsymbol{E}$ ∥ [001] and $\boldsymbol{B}$ ⊥ [100]<br>• $\boldsymbol{E}$ ⊥ [100] and $\boldsymbol{B}$ ∥ [010]<br>• $\boldsymbol{E}$ ⊥ [100] and $\boldsymbol{B}$ ∥ [001]<br>• $\boldsymbol{E}$ ⊥ [100] and $\boldsymbol{B}$ ⊥ [100]
P_Cc (7.30) $\mathbb{Z}_1$	• $\boldsymbol{E}$ ∥ [010] and strain ⊥ [100]<br>• $\boldsymbol{E}$ ∥ [001] and strain ⊥ [100]<br>• $\boldsymbol{E}$ ⊥ [100]
$Pn'c'2$ (30.115) $\mathbb{Z}_1$	• $\boldsymbol{E}$ ∥ [010] and $\boldsymbol{B}$ ∥ [010]
$Pb'a'2$ (32.138) $\mathbb{Z}_2$	• $\boldsymbol{E}$ ∥ [001] and $\boldsymbol{B}$ ∥ [001]
$P2_1'/c'$ (14.79) $\mathbb{Z}_2 \times \mathbb{Z}_4$	• $\boldsymbol{B}$ ∥ [010] and strain ⊥ [100]<br>• $\boldsymbol{B}$ ∥ [001] and strain ⊥ [100]<br>• $\boldsymbol{B}$ ⊥ [100]
$P2_1'$ (4.9) $\mathbb{Z}_1$	• $\boldsymbol{E}$ ∥ [010] and $\boldsymbol{B}$ ∥ [100] and strain ⊥ [010]<br>• $\boldsymbol{E}$ ∥ [010] and $\boldsymbol{B}$ ∥ [001] and strain ⊥ [010]<br>• $\boldsymbol{E}$ ∥ [010] and $\boldsymbol{B}$ ⊥ [010]
$Pn'a2_1'$ (33.146) $\mathbb{Z}_1$	• $\boldsymbol{E}$ ∥ [010] and $\boldsymbol{B}$ ∥ [100]
P_C2 (3.6) $\mathbb{Z}_1$	• $\boldsymbol{E}$ ∥ [010] and strain ⊥ [010]
$P2_1'/c'$ (14.79) $\mathbb{Z}_2 \times \mathbb{Z}_4$	• $\boldsymbol{B}$ ∥ [100] and strain ⊥ [010]<br>• $\boldsymbol{B}$ ∥ [001] and strain ⊥ [010]<br>• $\boldsymbol{B}$ ⊥ [010]
$Pmc'2_1'$ (26.69) $\mathbb{Z}_1$	• $\boldsymbol{E}$ ∥ [010] and $\boldsymbol{B}$ ∥ [001]
P_Ima2 (28.98) $\mathbb{Z}_1$	• $\boldsymbol{E}$ ∥ [010]
$Pb'a'm$ (55.357) $\mathbb{Z}_2 \times \mathbb{Z}_2 \times \mathbb{Z}_2$	• $\boldsymbol{B}$ ∥ [001]
$P2_1'$ (4.9) $\mathbb{Z}_1$	• $\boldsymbol{E}$ ∥ [001] and $\boldsymbol{B}$ ∥ [100] and strain ⊥ [001]<br>• $\boldsymbol{E}$ ∥ [001] and $\boldsymbol{B}$ ∥ [010] and strain ⊥ [001]<br>• $\boldsymbol{E}$ ∥ [001] and $\boldsymbol{B}$ ⊥ [001]
$Pca'2_1'$ (29.102) $\mathbb{Z}_1$	• $\boldsymbol{E}$ ∥ [001] and $\boldsymbol{B}$ ∥ [100]
P_C2 (3.6) $\mathbb{Z}_1$	• $\boldsymbol{E}$ ∥ [001] and strain ⊥ [001]

Continued on next page

...continued

Subgroup & SI group	Perturbation
$P2_1'/c'$ (14.79) $\mathbb{Z}_2\times\mathbb{Z}_4$	• $\boldsymbol{B}\parallel[100]$ and strain $\perp[001]$<br>• $\boldsymbol{B}\parallel[010]$ and strain $\perp[001]$<br>• $\boldsymbol{B}\perp[001]$
$Pca'2_1'$ (29.102) $\mathbb{Z}_1$	• $\boldsymbol{E}\parallel[001]$ and $\boldsymbol{B}\parallel[010]$
P_Icc2 (27.86) $\mathbb{Z}_2$	• $\boldsymbol{E}\parallel[001]$
$Pb'cn'$ (60.424) $\mathbb{Z}_2\times\mathbb{Z}_2$	• $\boldsymbol{B}\parallel[010]$
$Pb'cn'$ (60.424) $\mathbb{Z}_2\times\mathbb{Z}_2$	• $\boldsymbol{B}\parallel[100]$
$P_S\bar{1}$ (2.7) $\mathbb{Z}_2\times\mathbb{Z}_2\times\mathbb{Z}_4$	• strain in generic direction
P_C2/c (13.74) $\mathbb{Z}_2$	• strain $\perp[100]$
P_C2/c (13.74) $\mathbb{Z}_2$	• strain $\perp[010]$
P_C2/m (10.49) $\mathbb{Z}_2\times\mathbb{Z}_2$	• strain $\perp[001]$

Table. S 387: Subgroups of $Pban$ (50.277) that can be realized by applying $\boldsymbol{E}/\boldsymbol{B}$ fields and/or uniaxial strains.

Subgroup & SI group	Perturbation
$P2$ (3.1) $\mathbb{Z}_2$	• $\boldsymbol{E}\parallel[100]$ and strain $\perp[100]$<br>• $\boldsymbol{E}\parallel[100]$ and $\boldsymbol{B}\parallel[100]$
Pc (7.24) $\mathbb{Z}_1$	• $\boldsymbol{E}\parallel[100]$ and strain $\perp[001]$<br>• $\boldsymbol{E}\parallel[100]$ and $\boldsymbol{B}\parallel[001]$<br>• $\boldsymbol{E}\parallel[010]$ and strain $\perp[001]$<br>• $\boldsymbol{E}\parallel[010]$ and $\boldsymbol{B}\parallel[001]$<br>• $\boldsymbol{E}\perp[001]$
Pc (7.24) $\mathbb{Z}_1$	• $\boldsymbol{E}\parallel[100]$ and strain $\perp[010]$<br>• $\boldsymbol{E}\parallel[100]$ and $\boldsymbol{B}\parallel[010]$<br>• $\boldsymbol{E}\parallel[001]$ and strain $\perp[010]$<br>• $\boldsymbol{E}\parallel[001]$ and $\boldsymbol{B}\parallel[010]$<br>• $\boldsymbol{E}\perp[010]$
$Pnc2$ (30.111) $\mathbb{Z}_1$	• $\boldsymbol{E}\parallel[100]$

Continued on next page

...continued

Subgroup & SI group	Perturbation
Pc (7.24) $\mathbb{Z}_1$	• $\boldsymbol{E}$ ∥ [010] and strain ⊥ [100]<br>• $\boldsymbol{E}$ ∥ [010] and $\boldsymbol{B}$ ∥ [100]<br>• $\boldsymbol{E}$ ∥ [001] and strain ⊥ [100]<br>• $\boldsymbol{E}$ ∥ [001] and $\boldsymbol{B}$ ∥ [100]<br>• $\boldsymbol{E}$ ⊥ [100]
$P\bar{1}$ (2.4) $\mathbb{Z}_2 \times \mathbb{Z}_2 \times \mathbb{Z}_2 \times \mathbb{Z}_4$	• strain in generic direction<br>• $\boldsymbol{B}$ ∥ [100] and strain ⊥ [010]<br>• $\boldsymbol{B}$ ∥ [100] and strain ⊥ [001]<br>• $\boldsymbol{B}$ ∥ [010] and strain ⊥ [100]<br>• $\boldsymbol{B}$ ∥ [010] and strain ⊥ [001]<br>• $\boldsymbol{B}$ ∥ [001] and strain ⊥ [100]<br>• $\boldsymbol{B}$ ∥ [001] and strain ⊥ [010]<br>• $\boldsymbol{B}$ in generic direction
$P2/c$ (13.65) $\mathbb{Z}_2 \times \mathbb{Z}_2$	• strain ⊥ [100]<br>• $\boldsymbol{B}$ ∥ [100]
$P2$ (3.1) $\mathbb{Z}_2$	• $\boldsymbol{E}$ ∥ [010] and strain ⊥ [010]<br>• $\boldsymbol{E}$ ∥ [010] and $\boldsymbol{B}$ ∥ [010]
$Pnc2$ (30.111) $\mathbb{Z}_1$	• $\boldsymbol{E}$ ∥ [010]
$P2/c$ (13.65) $\mathbb{Z}_2 \times \mathbb{Z}_2$	• strain ⊥ [010]<br>• $\boldsymbol{B}$ ∥ [010]
$P2$ (3.1) $\mathbb{Z}_2$	• $\boldsymbol{E}$ ∥ [001] and strain ⊥ [001]<br>• $\boldsymbol{E}$ ∥ [001] and $\boldsymbol{B}$ ∥ [001]
$Pba2$ (32.135) $\mathbb{Z}_1$	• $\boldsymbol{E}$ ∥ [001]
$P2/c$ (13.65) $\mathbb{Z}_2 \times \mathbb{Z}_2$	• strain ⊥ [001]<br>• $\boldsymbol{B}$ ∥ [001]

Table. S 388: Subgroups of $Pban1'$ (50.278) that can be realized by applying $\boldsymbol{E}/\boldsymbol{B}$ fields and/or uniaxial strains.

Subgroup & SI group	Perturbation

Continued on next page

. . . continued

Subgroup & SI group	Perturbation
$P11'$ (1.2) $\mathbb{Z}_1$	• $\boldsymbol{E} \parallel [100]$ and strain in generic direction<br>• $\boldsymbol{E} \parallel [010]$ and strain in generic direction<br>• $\boldsymbol{E} \parallel [001]$ and strain in generic direction<br>• $\boldsymbol{E} \perp [100]$ and strain $\perp [010]$<br>• $\boldsymbol{E} \perp [100]$ and strain $\perp [001]$<br>• $\boldsymbol{E} \perp [100]$ and strain in generic direction<br>• $\boldsymbol{E} \perp [010]$ and strain $\perp [100]$<br>• $\boldsymbol{E} \perp [010]$ and strain $\perp [001]$<br>• $\boldsymbol{E} \perp [010]$ and strain in generic direction<br>• $\boldsymbol{E} \perp [001]$ and strain $\perp [100]$<br>• $\boldsymbol{E} \perp [001]$ and strain $\perp [010]$<br>• $\boldsymbol{E} \perp [001]$ and strain in generic direction<br>• $\boldsymbol{E}$ in generic direction
$P2'$ (3.3) $\mathbb{Z}_1$	• $\boldsymbol{E} \parallel [100]$ and $\boldsymbol{B} \parallel [010]$ and strain $\perp [100]$<br>• $\boldsymbol{E} \parallel [100]$ and $\boldsymbol{B} \parallel [001]$ and strain $\perp [100]$<br>• $\boldsymbol{E} \parallel [100]$ and $\boldsymbol{B} \perp [100]$
$P2$ (3.1) $\mathbb{Z}_2$	• $\boldsymbol{E} \parallel [100]$ and $\boldsymbol{B} \parallel [100]$ and strain $\perp [100]$
$P21'$ (3.2) $\mathbb{Z}_2$	• $\boldsymbol{E} \parallel [100]$ and strain $\perp [100]$
Pc' (7.26) $\mathbb{Z}_1$	• $\boldsymbol{E} \parallel [100]$ and $\boldsymbol{B} \parallel [100]$ and strain $\perp [001]$<br>• $\boldsymbol{E} \parallel [100]$ and $\boldsymbol{B} \parallel [010]$ and strain $\perp [001]$<br>• $\boldsymbol{E} \parallel [100]$ and $\boldsymbol{B} \perp [001]$<br>• $\boldsymbol{E} \parallel [010]$ and $\boldsymbol{B} \parallel [100]$ and strain $\perp [001]$<br>• $\boldsymbol{E} \parallel [010]$ and $\boldsymbol{B} \parallel [010]$ and strain $\perp [001]$<br>• $\boldsymbol{E} \parallel [010]$ and $\boldsymbol{B} \perp [001]$<br>• $\boldsymbol{E} \perp [001]$ and $\boldsymbol{B} \parallel [100]$<br>• $\boldsymbol{E} \perp [001]$ and $\boldsymbol{B} \parallel [010]$<br>• $\boldsymbol{E} \perp [001]$ and $\boldsymbol{B} \perp [001]$
Pc (7.24) $\mathbb{Z}_1$	• $\boldsymbol{E} \parallel [100]$ and $\boldsymbol{B} \parallel [001]$ and strain $\perp [001]$<br>• $\boldsymbol{E} \parallel [010]$ and $\boldsymbol{B} \parallel [001]$ and strain $\perp [001]$<br>• $\boldsymbol{E} \perp [001]$ and $\boldsymbol{B} \parallel [001]$
$Pc1'$ (7.25) $\mathbb{Z}_1$	• $\boldsymbol{E} \parallel [100]$ and strain $\perp [001]$<br>• $\boldsymbol{E} \parallel [010]$ and strain $\perp [001]$<br>• $\boldsymbol{E} \perp [001]$

Continued on next page

. . . continued

Subgroup & SI group	Perturbation
Pc' (7.26) $\mathbb{Z}_1$	• $\boldsymbol{E}$ ∥ [100] and $\boldsymbol{B}$ ∥ [100] and strain ⊥ [010]<br>• $\boldsymbol{E}$ ∥ [100] and $\boldsymbol{B}$ ∥ [001] and strain ⊥ [010]<br>• $\boldsymbol{E}$ ∥ [100] and $\boldsymbol{B}$ ⊥ [010]<br>• $\boldsymbol{E}$ ∥ [001] and $\boldsymbol{B}$ ∥ [100] and strain ⊥ [010]<br>• $\boldsymbol{E}$ ∥ [001] and $\boldsymbol{B}$ ∥ [001] and strain ⊥ [010]<br>• $\boldsymbol{E}$ ∥ [001] and $\boldsymbol{B}$ ⊥ [010]<br>• $\boldsymbol{E}$ ⊥ [010] and $\boldsymbol{B}$ ∥ [100]<br>• $\boldsymbol{E}$ ⊥ [010] and $\boldsymbol{B}$ ∥ [001]<br>• $\boldsymbol{E}$ ⊥ [010] and $\boldsymbol{B}$ ⊥ [010]
$Pn'c'2$ (30.115) $\mathbb{Z}_1$	• $\boldsymbol{E}$ ∥ [100] and $\boldsymbol{B}$ ∥ [100]
$Pnc'2'$ (30.114) $\mathbb{Z}_1$	• $\boldsymbol{E}$ ∥ [100] and $\boldsymbol{B}$ ∥ [001]
Pc (7.24) $\mathbb{Z}_1$	• $\boldsymbol{E}$ ∥ [100] and $\boldsymbol{B}$ ∥ [010] and strain ⊥ [010]<br>• $\boldsymbol{E}$ ∥ [001] and $\boldsymbol{B}$ ∥ [010] and strain ⊥ [010]<br>• $\boldsymbol{E}$ ⊥ [010] and $\boldsymbol{B}$ ∥ [010]
$Pn'c2'$ (30.113) $\mathbb{Z}_1$	• $\boldsymbol{E}$ ∥ [100] and $\boldsymbol{B}$ ∥ [010]
$Pc1'$ (7.25) $\mathbb{Z}_1$	• $\boldsymbol{E}$ ∥ [100] and strain ⊥ [010]<br>• $\boldsymbol{E}$ ∥ [001] and strain ⊥ [010]<br>• $\boldsymbol{E}$ ⊥ [010]
$Pnc21'$ (30.112) $\mathbb{Z}_1$	• $\boldsymbol{E}$ ∥ [100]
Pc' (7.26) $\mathbb{Z}_1$	• $\boldsymbol{E}$ ∥ [010] and $\boldsymbol{B}$ ∥ [010] and strain ⊥ [100]<br>• $\boldsymbol{E}$ ∥ [010] and $\boldsymbol{B}$ ∥ [001] and strain ⊥ [100]<br>• $\boldsymbol{E}$ ∥ [010] and $\boldsymbol{B}$ ⊥ [100]<br>• $\boldsymbol{E}$ ∥ [001] and $\boldsymbol{B}$ ∥ [010] and strain ⊥ [100]<br>• $\boldsymbol{E}$ ∥ [001] and $\boldsymbol{B}$ ∥ [001] and strain ⊥ [100]<br>• $\boldsymbol{E}$ ∥ [001] and $\boldsymbol{B}$ ⊥ [100]<br>• $\boldsymbol{E}$ ⊥ [100] and $\boldsymbol{B}$ ∥ [010]<br>• $\boldsymbol{E}$ ⊥ [100] and $\boldsymbol{B}$ ∥ [001]<br>• $\boldsymbol{E}$ ⊥ [100] and $\boldsymbol{B}$ ⊥ [100]
Pc (7.24) $\mathbb{Z}_1$	• $\boldsymbol{E}$ ∥ [010] and $\boldsymbol{B}$ ∥ [100] and strain ⊥ [100]<br>• $\boldsymbol{E}$ ∥ [001] and $\boldsymbol{B}$ ∥ [100] and strain ⊥ [100]<br>• $\boldsymbol{E}$ ⊥ [100] and $\boldsymbol{B}$ ∥ [100]
$Pc1'$ (7.25) $\mathbb{Z}_1$	• $\boldsymbol{E}$ ∥ [010] and strain ⊥ [100]<br>• $\boldsymbol{E}$ ∥ [001] and strain ⊥ [100]<br>• $\boldsymbol{E}$ ⊥ [100]

Continued on next page

. . . continued

Subgroup & SI group	Perturbation
$P\bar{1}$ (2.4) $\mathbb{Z}_2 \times \mathbb{Z}_2 \times \mathbb{Z}_2 \times \mathbb{Z}_4$	• $\boldsymbol{B} \parallel [100]$ and strain in generic direction<br>• $\boldsymbol{B} \parallel [010]$ and strain in generic direction<br>• $\boldsymbol{B} \parallel [001]$ and strain in generic direction<br>• $\boldsymbol{B} \perp [100]$ and strain $\perp [010]$<br>• $\boldsymbol{B} \perp [100]$ and strain $\perp [001]$<br>• $\boldsymbol{B} \perp [100]$ and strain in generic direction<br>• $\boldsymbol{B} \perp [010]$ and strain $\perp [100]$<br>• $\boldsymbol{B} \perp [010]$ and strain $\perp [001]$<br>• $\boldsymbol{B} \perp [010]$ and strain in generic direction<br>• $\boldsymbol{B} \perp [001]$ and strain $\perp [100]$<br>• $\boldsymbol{B} \perp [001]$ and strain $\perp [010]$<br>• $\boldsymbol{B} \perp [001]$ and strain in generic direction<br>• $\boldsymbol{B}$ in generic direction
$P2'/c'$ (13.69) $\mathbb{Z}_2 \times \mathbb{Z}_2 \times \mathbb{Z}_4$	• $\boldsymbol{B} \parallel [010]$ and strain $\perp [100]$<br>• $\boldsymbol{B} \parallel [001]$ and strain $\perp [100]$<br>• $\boldsymbol{B} \perp [100]$
$P2/c$ (13.65) $\mathbb{Z}_2 \times \mathbb{Z}_2$	• $\boldsymbol{B} \parallel [100]$ and strain $\perp [100]$
$P\bar{1}1'$ (2.5) $\mathbb{Z}_2 \times \mathbb{Z}_2 \times \mathbb{Z}_2 \times \mathbb{Z}_4$	• strain in generic direction
$P2/c1'$ (13.66) $\mathbb{Z}_2 \times \mathbb{Z}_2$	• strain $\perp [100]$
$P2'$ (3.3) $\mathbb{Z}_1$	• $\boldsymbol{E} \parallel [010]$ and $\boldsymbol{B} \parallel [100]$ and strain $\perp [010]$<br>• $\boldsymbol{E} \parallel [010]$ and $\boldsymbol{B} \parallel [001]$ and strain $\perp [010]$<br>• $\boldsymbol{E} \parallel [010]$ and $\boldsymbol{B} \perp [010]$
$Pnc'2'$ (30.114) $\mathbb{Z}_1$	• $\boldsymbol{E} \parallel [010]$ and $\boldsymbol{B} \parallel [001]$
$Pn'c2'$ (30.113) $\mathbb{Z}_1$	• $\boldsymbol{E} \parallel [010]$ and $\boldsymbol{B} \parallel [100]$
$P2'/c'$ (13.69) $\mathbb{Z}_2 \times \mathbb{Z}_2 \times \mathbb{Z}_4$	• $\boldsymbol{B} \parallel [100]$ and strain $\perp [010]$<br>• $\boldsymbol{B} \parallel [001]$ and strain $\perp [010]$<br>• $\boldsymbol{B} \perp [010]$
$P2$ (3.1) $\mathbb{Z}_2$	• $\boldsymbol{E} \parallel [010]$ and $\boldsymbol{B} \parallel [010]$ and strain $\perp [010]$
$Pn'c'2$ (30.115) $\mathbb{Z}_1$	• $\boldsymbol{E} \parallel [010]$ and $\boldsymbol{B} \parallel [010]$
$P2/c$ (13.65) $\mathbb{Z}_2 \times \mathbb{Z}_2$	• $\boldsymbol{B} \parallel [010]$ and strain $\perp [010]$
$P21'$ (3.2) $\mathbb{Z}_2$	• $\boldsymbol{E} \parallel [010]$ and strain $\perp [010]$
$Pnc21'$ (30.112) $\mathbb{Z}_1$	• $\boldsymbol{E} \parallel [010]$
$P2/c1'$ (13.66) $\mathbb{Z}_2 \times \mathbb{Z}_2$	• strain $\perp [010]$

Continued on next page

...continued

Subgroup & SI group	Perturbation
$P2'$ (3.3) $\mathbb{Z}_1$	• $\boldsymbol{E}$ ∥ [001] and $\boldsymbol{B}$ ∥ [100] and strain ⊥ [001]<br>• $\boldsymbol{E}$ ∥ [001] and $\boldsymbol{B}$ ∥ [010] and strain ⊥ [001]<br>• $\boldsymbol{E}$ ∥ [001] and $\boldsymbol{B}$ ⊥ [001]
$Pb'a2'$ (32.137) $\mathbb{Z}_1$	• $\boldsymbol{E}$ ∥ [001] and $\boldsymbol{B}$ ∥ [010]
$Pb'a2'$ (32.137) $\mathbb{Z}_1$	• $\boldsymbol{E}$ ∥ [001] and $\boldsymbol{B}$ ∥ [100]
$P2'/c'$ (13.69) $\mathbb{Z}_2 \times \mathbb{Z}_2 \times \mathbb{Z}_4$	• $\boldsymbol{B}$ ∥ [100] and strain ⊥ [001]<br>• $\boldsymbol{B}$ ∥ [010] and strain ⊥ [001]<br>• $\boldsymbol{B}$ ⊥ [001]
$Pb'an'$ (50.282) $\mathbb{Z}_2$	• $\boldsymbol{B}$ ∥ [100]
$Pb'an'$ (50.282) $\mathbb{Z}_2$	• $\boldsymbol{B}$ ∥ [010]
$P2$ (3.1) $\mathbb{Z}_2$	• $\boldsymbol{E}$ ∥ [001] and $\boldsymbol{B}$ ∥ [001] and strain ⊥ [001]
$Pb'a'2$ (32.138) $\mathbb{Z}_2$	• $\boldsymbol{E}$ ∥ [001] and $\boldsymbol{B}$ ∥ [001]
$P2/c$ (13.65) $\mathbb{Z}_2 \times \mathbb{Z}_2$	• $\boldsymbol{B}$ ∥ [001] and strain ⊥ [001]
$Pb'a'n$ (50.281) $\mathbb{Z}_2 \times \mathbb{Z}_2$	• $\boldsymbol{B}$ ∥ [001]
$P21'$ (3.2) $\mathbb{Z}_2$	• $\boldsymbol{E}$ ∥ [001] and strain ⊥ [001]
$Pba21'$ (32.136) $\mathbb{Z}_1$	• $\boldsymbol{E}$ ∥ [001]
$P2/c1'$ (13.66) $\mathbb{Z}_2 \times \mathbb{Z}_2$	• strain ⊥ [001]

Table. S 389: Subgroups of $Pb'an$ (50.279) that can be realized by applying $\boldsymbol{E}/\boldsymbol{B}$ fields and/or uniaxial strains.

Subgroup & SI group	Perturbation
$P2$ (3.1) $\mathbb{Z}_2$	• $\boldsymbol{B}$ ∥ [100] and strain ⊥ [100]<br>• $\boldsymbol{E}$ ∥ [100] and strain ⊥ [100]<br>• $\boldsymbol{E}$ ∥ [100] and $\boldsymbol{B}$ ∥ [100]
Pc (7.24) $\mathbb{Z}_1$	• $\boldsymbol{B}$ ∥ [001] and strain ⊥ [001]<br>• $\boldsymbol{E}$ ∥ [100] and strain ⊥ [001]<br>• $\boldsymbol{E}$ ∥ [100] and $\boldsymbol{B}$ ∥ [001]<br>• $\boldsymbol{E}$ ∥ [010] and strain ⊥ [001]<br>• $\boldsymbol{E}$ ⊥ [001]

Continued on next page

...continued

Subgroup & SI group	Perturbation
Pc (7.24) $\mathbb{Z}_1$	• $\boldsymbol{B} \parallel [010]$ and strain $\perp [010]$<br>• $\boldsymbol{E} \parallel [100]$ and strain $\perp [010]$<br>• $\boldsymbol{E} \parallel [100]$ and $\boldsymbol{B} \parallel [010]$<br>• $\boldsymbol{E} \parallel [001]$ and strain $\perp [010]$<br>• $\boldsymbol{E} \perp [010]$
$Pnc2$ (30.111) $\mathbb{Z}_1$	• $\boldsymbol{E} \parallel [100]$
Pc' (7.26) $\mathbb{Z}_1$	• $\boldsymbol{B} \parallel [010]$ and strain $\perp [100]$<br>• $\boldsymbol{B} \parallel [001]$ and strain $\perp [100]$<br>• $\boldsymbol{B} \perp [100]$<br>• $\boldsymbol{E} \parallel [010]$ and strain $\perp [100]$<br>• $\boldsymbol{E} \parallel [010]$ and $\boldsymbol{B} \parallel [010]$<br>• $\boldsymbol{E} \parallel [001]$ and strain $\perp [100]$<br>• $\boldsymbol{E} \parallel [001]$ and $\boldsymbol{B} \parallel [001]$<br>• $\boldsymbol{E} \perp [100]$
$P\bar{1}'$ (2.6) $\mathbb{Z}_1$	• strain in generic direction
$P2/c'$ (13.68) $\mathbb{Z}_2$	• strain $\perp [100]$
$P2'$ (3.3) $\mathbb{Z}_1$	• $\boldsymbol{B} \parallel [100]$ and strain $\perp [010]$<br>• $\boldsymbol{B} \parallel [001]$ and strain $\perp [010]$<br>• $\boldsymbol{B} \perp [010]$<br>• $\boldsymbol{E} \parallel [010]$ and strain $\perp [010]$<br>• $\boldsymbol{E} \parallel [010]$ and $\boldsymbol{B} \parallel [100]$
$Pnc'2'$ (30.114) $\mathbb{Z}_1$	• $\boldsymbol{B} \parallel [001]$<br>• $\boldsymbol{E} \parallel [010]$
$P2'/c$ (13.67) $\mathbb{Z}_1$	• strain $\perp [010]$
$P2'$ (3.3) $\mathbb{Z}_1$	• $\boldsymbol{B} \parallel [100]$ and strain $\perp [001]$<br>• $\boldsymbol{B} \parallel [010]$ and strain $\perp [001]$<br>• $\boldsymbol{B} \perp [001]$<br>• $\boldsymbol{E} \parallel [001]$ and strain $\perp [001]$<br>• $\boldsymbol{E} \parallel [001]$ and $\boldsymbol{B} \parallel [100]$
$Pb'a2'$ (32.137) $\mathbb{Z}_1$	• $\boldsymbol{B} \parallel [010]$<br>• $\boldsymbol{E} \parallel [001]$
$P2'/c$ (13.67) $\mathbb{Z}_1$	• strain $\perp [001]$
$P2'2'2$ (16.3) $\mathbb{Z}_2$	• $\boldsymbol{B} \parallel [100]$

Table. S 390: Subgroups of $Pban'$ (50.280) that can be realized by applying $\boldsymbol{E}/\boldsymbol{B}$ fields and/or uniaxial strains.

Subgroup & SI group	Perturbation
$P2'$ (3.3) $\mathbb{Z}_1$	• $\boldsymbol{B} \parallel [010]$ and strain $\perp [100]$<br>• $\boldsymbol{B} \parallel [001]$ and strain $\perp [100]$<br>• $\boldsymbol{B} \perp [100]$<br>• $\boldsymbol{E} \parallel [100]$ and strain $\perp [100]$<br>• $\boldsymbol{E} \parallel [100]$ and $\boldsymbol{B} \parallel [001]$
Pc' (7.26) $\mathbb{Z}_1$	• $\boldsymbol{B} \parallel [100]$ and strain $\perp [001]$<br>• $\boldsymbol{B} \parallel [010]$ and strain $\perp [001]$<br>• $\boldsymbol{B} \perp [001]$<br>• $\boldsymbol{E} \parallel [100]$ and strain $\perp [001]$<br>• $\boldsymbol{E} \parallel [100]$ and $\boldsymbol{B} \parallel [100]$<br>• $\boldsymbol{E} \parallel [010]$ and strain $\perp [001]$<br>• $\boldsymbol{E} \parallel [010]$ and $\boldsymbol{B} \parallel [010]$<br>• $\boldsymbol{E} \perp [001]$
Pc (7.24) $\mathbb{Z}_1$	• $\boldsymbol{B} \parallel [010]$ and strain $\perp [010]$<br>• $\boldsymbol{E} \parallel [100]$ and strain $\perp [010]$<br>• $\boldsymbol{E} \parallel [001]$ and strain $\perp [010]$<br>• $\boldsymbol{E} \parallel [001]$ and $\boldsymbol{B} \parallel [010]$<br>• $\boldsymbol{E} \perp [010]$
$Pn'c2'$ (30.113) $\mathbb{Z}_1$	• $\boldsymbol{B} \parallel [010]$<br>• $\boldsymbol{E} \parallel [100]$
Pc (7.24) $\mathbb{Z}_1$	• $\boldsymbol{B} \parallel [100]$ and strain $\perp [100]$<br>• $\boldsymbol{E} \parallel [010]$ and strain $\perp [100]$<br>• $\boldsymbol{E} \parallel [001]$ and strain $\perp [100]$<br>• $\boldsymbol{E} \parallel [001]$ and $\boldsymbol{B} \parallel [100]$<br>• $\boldsymbol{E} \perp [100]$
$P\bar{1}'$ (2.6) $\mathbb{Z}_1$	• strain in generic direction
$P2'/c$ (13.67) $\mathbb{Z}_1$	• strain $\perp [100]$
$P2'$ (3.3) $\mathbb{Z}_1$	• $\boldsymbol{B} \parallel [100]$ and strain $\perp [010]$<br>• $\boldsymbol{B} \parallel [001]$ and strain $\perp [010]$<br>• $\boldsymbol{B} \perp [010]$<br>• $\boldsymbol{E} \parallel [010]$ and strain $\perp [010]$<br>• $\boldsymbol{E} \parallel [010]$ and $\boldsymbol{B} \parallel [001]$
$Pn'c2'$ (30.113) $\mathbb{Z}_1$	• $\boldsymbol{B} \parallel [100]$<br>• $\boldsymbol{E} \parallel [010]$
$P2'/c$ (13.67) $\mathbb{Z}_1$	• strain $\perp [010]$

Continued on next page

...continued

Subgroup & SI group	Perturbation
$P2$ (3.1) $\mathbb{Z}_2$	• $\boldsymbol{B} \parallel [001]$ and strain $\perp [001]$<br>• $\boldsymbol{E} \parallel [001]$ and strain $\perp [001]$<br>• $\boldsymbol{E} \parallel [001]$ and $\boldsymbol{B} \parallel [001]$
$Pba2$ (32.135) $\mathbb{Z}_1$	• $\boldsymbol{E} \parallel [001]$
$P2/c'$ (13.68) $\mathbb{Z}_2$	• strain $\perp [001]$
$P2'2'2$ (16.3) $\mathbb{Z}_2$	• $\boldsymbol{B} \parallel [001]$

Table. S 391: Subgroups of $Pb'a'n$ (50.281) that can be realized by applying $\boldsymbol{E}/\boldsymbol{B}$ fields and/or uniaxial strains.

Subgroup & SI group	Perturbation
$P2'$ (3.3) $\mathbb{Z}_1$	• $\boldsymbol{E} \parallel [100]$ and strain $\perp [100]$<br>• $\boldsymbol{E} \parallel [100]$ and ($\boldsymbol{B} \parallel [010]$ or $\boldsymbol{B} \perp [100]$)
Pc (7.24) $\mathbb{Z}_1$	• $\boldsymbol{E} \parallel [100]$ and strain $\perp [001]$<br>• $\boldsymbol{E} \parallel [010]$ and strain $\perp [001]$<br>• $\boldsymbol{E} \perp [001]$
Pc' (7.26) $\mathbb{Z}_1$	• $\boldsymbol{E} \parallel [100]$ and strain $\perp [010]$<br>• $\boldsymbol{E} \parallel [100]$ and ($\boldsymbol{B} \parallel [100]$ or $\boldsymbol{B} \perp [010]$)<br>• $\boldsymbol{E} \parallel [001]$ and strain $\perp [010]$<br>• $\boldsymbol{E} \parallel [001]$ and ($\boldsymbol{B} \parallel [100]$ or $\boldsymbol{B} \perp [010]$)<br>• $\boldsymbol{E} \perp [010]$
$Pnc'2'$ (30.114) $\mathbb{Z}_1$	• $\boldsymbol{E} \parallel [100]$
Pc' (7.26) $\mathbb{Z}_1$	• $\boldsymbol{E} \parallel [010]$ and strain $\perp [100]$<br>• $\boldsymbol{E} \parallel [010]$ and ($\boldsymbol{B} \parallel [010]$ or $\boldsymbol{B} \perp [100]$)<br>• $\boldsymbol{E} \parallel [001]$ and strain $\perp [100]$<br>• $\boldsymbol{E} \parallel [001]$ and ($\boldsymbol{B} \parallel [010]$ or $\boldsymbol{B} \perp [100]$)<br>• $\boldsymbol{E} \perp [100]$
$P\bar{1}$ (2.4) $\mathbb{Z}_2 \times \mathbb{Z}_2 \times \mathbb{Z}_2 \times \mathbb{Z}_4$	• strain in generic direction<br>• ($\boldsymbol{B} \parallel [100]$ or $\boldsymbol{B} \perp [010]$) and strain $\perp [100]$<br>• ($\boldsymbol{B} \parallel [100]$ or $\boldsymbol{B} \perp [010]$) and strain $\perp [001]$<br>• ($\boldsymbol{B} \parallel [010]$ or $\boldsymbol{B} \perp [100]$) and strain $\perp [010]$<br>• ($\boldsymbol{B} \parallel [010]$ or $\boldsymbol{B} \perp [100]$) and strain $\perp [001]$<br>• $\boldsymbol{B}$ in generic direction

Continued on next page

...continued

Subgroup & SI group	Perturbation
$P2'/c'$ (13.69) $\mathbb{Z}_2 \times \mathbb{Z}_2 \times \mathbb{Z}_4$	• strain $\perp$ [100]<br>• ($\boldsymbol{B} \parallel$ [010] or $\boldsymbol{B} \perp$ [100])
$P2'$ (3.3) $\mathbb{Z}_1$	• $\boldsymbol{E} \parallel$ [010] and strain $\perp$ [010]<br>• $\boldsymbol{E} \parallel$ [010] and ($\boldsymbol{B} \parallel$ [100] or $\boldsymbol{B} \perp$ [010])
$Pnc'2'$ (30.114) $\mathbb{Z}_1$	• $\boldsymbol{E} \parallel$ [010]
$P2'/c'$ (13.69) $\mathbb{Z}_2 \times \mathbb{Z}_2 \times \mathbb{Z}_4$	• strain $\perp$ [010]<br>• ($\boldsymbol{B} \parallel$ [100] or $\boldsymbol{B} \perp$ [010])
$P2$ (3.1) $\mathbb{Z}_2$	• $\boldsymbol{E} \parallel$ [001] and strain $\perp$ [001]
$Pb'a'2$ (32.138) $\mathbb{Z}_2$	• $\boldsymbol{E} \parallel$ [001]
$P2/c$ (13.65) $\mathbb{Z}_2 \times \mathbb{Z}_2$	• strain $\perp$ [001]

Table. S 392: Subgroups of $Pb'an'$ (50.282) that can be realized by applying $\boldsymbol{E}/\boldsymbol{B}$ fields and/or uniaxial strains.

Subgroup & SI group	Perturbation
$P2'$ (3.3) $\mathbb{Z}_1$	• $\boldsymbol{E} \parallel$ [100] and strain $\perp$ [100]<br>• $\boldsymbol{E} \parallel$ [100] and ($\boldsymbol{B} \parallel$ [001] or $\boldsymbol{B} \perp$ [100])
Pc' (7.26) $\mathbb{Z}_1$	• $\boldsymbol{E} \parallel$ [100] and strain $\perp$ [001]<br>• $\boldsymbol{E} \parallel$ [100] and ($\boldsymbol{B} \parallel$ [100] or $\boldsymbol{B} \perp$ [001])<br>• $\boldsymbol{E} \parallel$ [010] and strain $\perp$ [001]<br>• $\boldsymbol{E} \parallel$ [010] and ($\boldsymbol{B} \parallel$ [100] or $\boldsymbol{B} \perp$ [001])<br>• $\boldsymbol{E} \perp$ [001]
Pc (7.24) $\mathbb{Z}_1$	• $\boldsymbol{E} \parallel$ [100] and strain $\perp$ [010]<br>• $\boldsymbol{E} \parallel$ [001] and strain $\perp$ [010]<br>• $\boldsymbol{E} \perp$ [010]
$Pn'c2'$ (30.113) $\mathbb{Z}_1$	• $\boldsymbol{E} \parallel$ [100]
Pc' (7.26) $\mathbb{Z}_1$	• $\boldsymbol{E} \parallel$ [010] and strain $\perp$ [100]<br>• $\boldsymbol{E} \parallel$ [010] and ($\boldsymbol{B} \parallel$ [001] or $\boldsymbol{B} \perp$ [100])<br>• $\boldsymbol{E} \parallel$ [001] and strain $\perp$ [100]<br>• $\boldsymbol{E} \parallel$ [001] and ($\boldsymbol{B} \parallel$ [001] or $\boldsymbol{B} \perp$ [100])<br>• $\boldsymbol{E} \perp$ [100]

Continued on next page

...continued

Subgroup & SI group	Perturbation
$P\bar{1}$ (2.4) $\mathbb{Z}_2\times\mathbb{Z}_2\times\mathbb{Z}_2\times\mathbb{Z}_4$	• strain in generic direction<br>• ($\boldsymbol{B}\parallel[100]$ or $\boldsymbol{B}\perp[001]$) and strain $\perp[100]$<br>• ($\boldsymbol{B}\parallel[100]$ or $\boldsymbol{B}\perp[001]$) and strain $\perp[010]$<br>• ($\boldsymbol{B}\parallel[001]$ or $\boldsymbol{B}\perp[100]$) and strain $\perp[010]$<br>• ($\boldsymbol{B}\parallel[001]$ or $\boldsymbol{B}\perp[100]$) and strain $\perp[001]$<br>• $\boldsymbol{B}$ in generic direction
$P2'/c'$ (13.69) $\mathbb{Z}_2\times\mathbb{Z}_2\times\mathbb{Z}_4$	• strain $\perp[100]$<br>• ($\boldsymbol{B}\parallel[001]$ or $\boldsymbol{B}\perp[100]$)
$P2$ (3.1) $\mathbb{Z}_2$	• $\boldsymbol{E}\parallel[010]$ and strain $\perp[010]$
$Pn'c'2$ (30.115) $\mathbb{Z}_1$	• $\boldsymbol{E}\parallel[010]$
$P2/c$ (13.65) $\mathbb{Z}_2\times\mathbb{Z}_2$	• strain $\perp[010]$
$P2'$ (3.3) $\mathbb{Z}_1$	• $\boldsymbol{E}\parallel[001]$ and strain $\perp[001]$<br>• $\boldsymbol{E}\parallel[001]$ and ($\boldsymbol{B}\parallel[100]$ or $\boldsymbol{B}\perp[001]$)
$Pb'a2'$ (32.137) $\mathbb{Z}_1$	• $\boldsymbol{E}\parallel[001]$
$P2'/c'$ (13.69) $\mathbb{Z}_2\times\mathbb{Z}_2\times\mathbb{Z}_4$	• strain $\perp[001]$<br>• ($\boldsymbol{B}\parallel[100]$ or $\boldsymbol{B}\perp[001]$)

Table. S 393: Subgroups of $Pb'a'n'$ (50.283) that can be realized by applying $\boldsymbol{E}/\boldsymbol{B}$ fields and/or uniaxial strains.

Subgroup & SI group	Perturbation
$P2$ (3.1) $\mathbb{Z}_2$	• $\boldsymbol{B}\parallel[100]$ and strain $\perp[100]$<br>• $\boldsymbol{E}\parallel[100]$ and strain $\perp[100]$
Pc' (7.26) $\mathbb{Z}_1$	• $\boldsymbol{B}\parallel[100]$ and strain $\perp[001]$<br>• $\boldsymbol{B}\parallel[010]$ and strain $\perp[001]$<br>• $\boldsymbol{B}\perp[001]$<br>• $\boldsymbol{E}\parallel[100]$ and strain $\perp[001]$<br>• $\boldsymbol{E}\parallel[100]$ and $\boldsymbol{B}\parallel[010]$<br>• $\boldsymbol{E}\parallel[010]$ and strain $\perp[001]$<br>• $\boldsymbol{E}\parallel[010]$ and $\boldsymbol{B}\parallel[100]$<br>• $\boldsymbol{E}\perp[001]$

Continued on next page

…continued

Subgroup & SI group	Perturbation
Pc' (7.26) $\mathbb{Z}_1$	• $\boldsymbol{B} \parallel [100]$ and strain $\perp [010]$<br>• $\boldsymbol{B} \parallel [001]$ and strain $\perp [010]$<br>• $\boldsymbol{B} \perp [010]$<br>• $\boldsymbol{E} \parallel [100]$ and strain $\perp [010]$<br>• $\boldsymbol{E} \parallel [100]$ and $\boldsymbol{B} \parallel [001]$<br>• $\boldsymbol{E} \parallel [001]$ and strain $\perp [010]$<br>• $\boldsymbol{E} \parallel [001]$ and $\boldsymbol{B} \parallel [100]$<br>• $\boldsymbol{E} \perp [010]$
$Pn'c'2$ (30.115) $\mathbb{Z}_1$	• $\boldsymbol{B} \parallel [100]$<br>• $\boldsymbol{E} \parallel [100]$
Pc' (7.26) $\mathbb{Z}_1$	• $\boldsymbol{B} \parallel [010]$ and strain $\perp [100]$<br>• $\boldsymbol{B} \parallel [001]$ and strain $\perp [100]$<br>• $\boldsymbol{B} \perp [100]$<br>• $\boldsymbol{E} \parallel [010]$ and strain $\perp [100]$<br>• $\boldsymbol{E} \parallel [010]$ and $\boldsymbol{B} \parallel [001]$<br>• $\boldsymbol{E} \parallel [001]$ and strain $\perp [100]$<br>• $\boldsymbol{E} \parallel [001]$ and $\boldsymbol{B} \parallel [010]$<br>• $\boldsymbol{E} \perp [100]$
$P\bar{1}'$ (2.6) $\mathbb{Z}_1$	• strain in generic direction
$P2/c'$ (13.68) $\mathbb{Z}_2$	• strain $\perp [100]$
$P2$ (3.1) $\mathbb{Z}_2$	• $\boldsymbol{B} \parallel [010]$ and strain $\perp [010]$<br>• $\boldsymbol{E} \parallel [010]$ and strain $\perp [010]$
$Pn'c'2$ (30.115) $\mathbb{Z}_1$	• $\boldsymbol{B} \parallel [010]$<br>• $\boldsymbol{E} \parallel [010]$
$P2/c'$ (13.68) $\mathbb{Z}_2$	• strain $\perp [010]$
$P2$ (3.1) $\mathbb{Z}_2$	• $\boldsymbol{B} \parallel [001]$ and strain $\perp [001]$<br>• $\boldsymbol{E} \parallel [001]$ and strain $\perp [001]$
$Pb'a'2$ (32.138) $\mathbb{Z}_2$	• $\boldsymbol{B} \parallel [001]$<br>• $\boldsymbol{E} \parallel [001]$
$P2/c'$ (13.68) $\mathbb{Z}_2$	• strain $\perp [001]$

Table. S 394: Subgroups of P_aban (50.284) that can be realized by applying $\boldsymbol{E}/\boldsymbol{B}$ fields and/or uniaxial strains.

Subgroup & SI group	Perturbation
Pc' (7.26) $\mathbb{Z}_1$	• $\boldsymbol{E}$ ∥ [100] and $\boldsymbol{B}$ ∥ [100] and strain ⊥ [001]<br>• $\boldsymbol{E}$ ∥ [100] and $\boldsymbol{B}$ ∥ [010] and strain ⊥ [001]<br>• $\boldsymbol{E}$ ∥ [100] and $\boldsymbol{B}$ ⊥ [001]<br>• $\boldsymbol{E}$ ∥ [010] and $\boldsymbol{B}$ ∥ [100] and strain ⊥ [001]<br>• $\boldsymbol{E}$ ∥ [010] and $\boldsymbol{B}$ ∥ [010] and strain ⊥ [001]<br>• $\boldsymbol{E}$ ∥ [010] and $\boldsymbol{B}$ ⊥ [001]<br>• $\boldsymbol{E}$ ⊥ [001] and $\boldsymbol{B}$ ∥ [100]<br>• $\boldsymbol{E}$ ⊥ [001] and $\boldsymbol{B}$ ∥ [010]<br>• $\boldsymbol{E}$ ⊥ [001] and $\boldsymbol{B}$ ⊥ [001]
Pm' (6.20) $\mathbb{Z}_1$	• $\boldsymbol{E}$ ∥ [100] and $\boldsymbol{B}$ ∥ [100] and strain ⊥ [010]<br>• $\boldsymbol{E}$ ∥ [100] and $\boldsymbol{B}$ ∥ [001] and strain ⊥ [010]<br>• $\boldsymbol{E}$ ∥ [100] and $\boldsymbol{B}$ ⊥ [010]<br>• $\boldsymbol{E}$ ∥ [001] and $\boldsymbol{B}$ ∥ [100] and strain ⊥ [010]<br>• $\boldsymbol{E}$ ∥ [001] and $\boldsymbol{B}$ ∥ [001] and strain ⊥ [010]<br>• $\boldsymbol{E}$ ∥ [001] and $\boldsymbol{B}$ ⊥ [010]<br>• $\boldsymbol{E}$ ⊥ [010] and $\boldsymbol{B}$ ∥ [100]<br>• $\boldsymbol{E}$ ⊥ [010] and $\boldsymbol{B}$ ∥ [001]<br>• $\boldsymbol{E}$ ⊥ [010] and $\boldsymbol{B}$ ⊥ [010]
$P2$ (3.1) $\mathbb{Z}_2$	• $\boldsymbol{E}$ ∥ [100] and $\boldsymbol{B}$ ∥ [100] and strain ⊥ [100]
$Pm'a'2$ (28.91) $\mathbb{Z}_2$	• $\boldsymbol{E}$ ∥ [100] and $\boldsymbol{B}$ ∥ [100]
P_S1 (1.3) $\mathbb{Z}_1$	• $\boldsymbol{E}$ ∥ [100] and strain in generic direction<br>• $\boldsymbol{E}$ ∥ [010] and strain in generic direction<br>• $\boldsymbol{E}$ ∥ [001] and strain in generic direction<br>• $\boldsymbol{E}$ ⊥ [100] and strain ⊥ [010]<br>• $\boldsymbol{E}$ ⊥ [100] and strain ⊥ [001]<br>• $\boldsymbol{E}$ ⊥ [100] and strain in generic direction<br>• $\boldsymbol{E}$ ⊥ [010] and strain ⊥ [100]<br>• $\boldsymbol{E}$ ⊥ [010] and strain ⊥ [001]<br>• $\boldsymbol{E}$ ⊥ [010] and strain in generic direction<br>• $\boldsymbol{E}$ ⊥ [001] and strain ⊥ [100]<br>• $\boldsymbol{E}$ ⊥ [001] and strain ⊥ [010]<br>• $\boldsymbol{E}$ ⊥ [001] and strain in generic direction<br>• $\boldsymbol{E}$ in generic direction
Pc (7.24) $\mathbb{Z}_1$	• $\boldsymbol{E}$ ∥ [100] and $\boldsymbol{B}$ ∥ [001] and strain ⊥ [001]<br>• $\boldsymbol{E}$ ∥ [010] and $\boldsymbol{B}$ ∥ [001] and strain ⊥ [001]<br>• $\boldsymbol{E}$ ⊥ [001] and $\boldsymbol{B}$ ∥ [001]

Continued on next page

...continued

Subgroup & SI group	Perturbation
P_ac (7.27) $\mathbb{Z}_1$	• $\boldsymbol{E} \parallel [100]$ and strain $\perp [001]$<br>• $\boldsymbol{E} \parallel [010]$ and strain $\perp [001]$<br>• $\boldsymbol{E} \perp [001]$
Pc (7.24) $\mathbb{Z}_1$	• $\boldsymbol{E} \parallel [100]$ and $\boldsymbol{B} \parallel [010]$ and strain $\perp [010]$<br>• $\boldsymbol{E} \parallel [001]$ and $\boldsymbol{B} \parallel [010]$ and strain $\perp [010]$<br>• $\boldsymbol{E} \perp [010]$ and $\boldsymbol{B} \parallel [010]$
P_cc (7.28) $\mathbb{Z}_1$	• $\boldsymbol{E} \parallel [100]$ and strain $\perp [010]$<br>• $\boldsymbol{E} \parallel [001]$ and strain $\perp [010]$<br>• $\boldsymbol{E} \perp [010]$
$P2_1'$ (4.9) $\mathbb{Z}_1$	• $\boldsymbol{E} \parallel [100]$ and $\boldsymbol{B} \parallel [010]$ and strain $\perp [100]$<br>• $\boldsymbol{E} \parallel [100]$ and $\boldsymbol{B} \parallel [001]$ and strain $\perp [100]$<br>• $\boldsymbol{E} \parallel [100]$ and $\boldsymbol{B} \perp [100]$
P_b2 (3.5) $\mathbb{Z}_2$	• $\boldsymbol{E} \parallel [100]$ and strain $\perp [100]$
$Pm'n2_1'$ (31.125) $\mathbb{Z}_1$	• $\boldsymbol{E} \parallel [100]$ and $\boldsymbol{B} \parallel [001]$
$Pca'2_1'$ (29.102) $\mathbb{Z}_1$	• $\boldsymbol{E} \parallel [100]$ and $\boldsymbol{B} \parallel [010]$
P_cnc2 (30.118) $\mathbb{Z}_2$	• $\boldsymbol{E} \parallel [100]$
$P2'$ (3.3) $\mathbb{Z}_1$	• $\boldsymbol{E} \parallel [010]$ and $\boldsymbol{B} \parallel [100]$ and strain $\perp [010]$<br>• $\boldsymbol{E} \parallel [010]$ and $\boldsymbol{B} \parallel [001]$ and strain $\perp [010]$<br>• $\boldsymbol{E} \parallel [010]$ and $\boldsymbol{B} \perp [010]$
Pc (7.24) $\mathbb{Z}_1$	• $\boldsymbol{E} \parallel [010]$ and $\boldsymbol{B} \parallel [100]$ and strain $\perp [100]$<br>• $\boldsymbol{E} \parallel [001]$ and $\boldsymbol{B} \parallel [100]$ and strain $\perp [100]$<br>• $\boldsymbol{E} \perp [100]$ and $\boldsymbol{B} \parallel [100]$
$Pc'c2'$ (27.80) $\mathbb{Z}_1$	• $\boldsymbol{E} \parallel [010]$ and $\boldsymbol{B} \parallel [100]$
$P\bar{1}$ (2.4) $\mathbb{Z}_2 \times \mathbb{Z}_2 \times \mathbb{Z}_2 \times \mathbb{Z}_4$	• $\boldsymbol{B} \parallel [100]$ and strain in generic direction<br>• $\boldsymbol{B} \parallel [010]$ and strain in generic direction<br>• $\boldsymbol{B} \parallel [001]$ and strain in generic direction<br>• $\boldsymbol{B} \perp [100]$ and strain $\perp [010]$<br>• $\boldsymbol{B} \perp [100]$ and strain $\perp [001]$<br>• $\boldsymbol{B} \perp [100]$ and strain in generic direction<br>• $\boldsymbol{B} \perp [010]$ and strain $\perp [100]$<br>• $\boldsymbol{B} \perp [010]$ and strain $\perp [001]$<br>• $\boldsymbol{B} \perp [010]$ and strain in generic direction<br>• $\boldsymbol{B} \perp [001]$ and strain $\perp [100]$<br>• $\boldsymbol{B} \perp [001]$ and strain $\perp [010]$<br>• $\boldsymbol{B} \perp [001]$ and strain in generic direction<br>• $\boldsymbol{B}$ in generic direction

Continued on next page

...continued

Subgroup & SI group	Perturbation
$P2'/m'$ (10.46) $\mathbb{Z}_2 \times \mathbb{Z}_2 \times \mathbb{Z}_2 \times \mathbb{Z}_4$	• $\boldsymbol{B}$ ∥ [100] and strain ⊥ [010]<br>• $\boldsymbol{B}$ ∥ [001] and strain ⊥ [010]<br>• $\boldsymbol{B}$ ⊥ [010]
$P2/c$ (13.65) $\mathbb{Z}_2 \times \mathbb{Z}_2$	• $\boldsymbol{B}$ ∥ [100] and strain ⊥ [100]
$P2'$ (3.3) $\mathbb{Z}_1$	• $\boldsymbol{E}$ ∥ [001] and $\boldsymbol{B}$ ∥ [100] and strain ⊥ [001]<br>• $\boldsymbol{E}$ ∥ [001] and $\boldsymbol{B}$ ∥ [010] and strain ⊥ [001]<br>• $\boldsymbol{E}$ ∥ [001] and $\boldsymbol{B}$ ⊥ [001]
$Pm'a2'$ (28.89) $\mathbb{Z}_1$	• $\boldsymbol{E}$ ∥ [001] and $\boldsymbol{B}$ ∥ [100]
$P2'/c'$ (13.69) $\mathbb{Z}_2 \times \mathbb{Z}_2 \times \mathbb{Z}_4$	• $\boldsymbol{B}$ ∥ [100] and strain ⊥ [001]<br>• $\boldsymbol{B}$ ∥ [010] and strain ⊥ [001]<br>• $\boldsymbol{B}$ ⊥ [001]
$Pc'cm'$ (49.270) $\mathbb{Z}_2 \times \mathbb{Z}_2$	• $\boldsymbol{B}$ ∥ [100]
$P2$ (3.1) $\mathbb{Z}_2$	• $\boldsymbol{E}$ ∥ [010] and $\boldsymbol{B}$ ∥ [010] and strain ⊥ [010]
P_a2 (3.4) $\mathbb{Z}_2$	• $\boldsymbol{E}$ ∥ [010] and strain ⊥ [010]
$P2/c$ (13.65) $\mathbb{Z}_2 \times \mathbb{Z}_2$	• $\boldsymbol{B}$ ∥ [010] and strain ⊥ [010]
Pc' (7.26) $\mathbb{Z}_1$	• $\boldsymbol{E}$ ∥ [010] and $\boldsymbol{B}$ ∥ [010] and strain ⊥ [100]<br>• $\boldsymbol{E}$ ∥ [010] and $\boldsymbol{B}$ ∥ [001] and strain ⊥ [100]<br>• $\boldsymbol{E}$ ∥ [010] and $\boldsymbol{B}$ ⊥ [100]<br>• $\boldsymbol{E}$ ∥ [001] and $\boldsymbol{B}$ ∥ [010] and strain ⊥ [100]<br>• $\boldsymbol{E}$ ∥ [001] and $\boldsymbol{B}$ ∥ [001] and strain ⊥ [100]<br>• $\boldsymbol{E}$ ∥ [001] and $\boldsymbol{B}$ ⊥ [100]<br>• $\boldsymbol{E}$ ⊥ [100] and $\boldsymbol{B}$ ∥ [010]<br>• $\boldsymbol{E}$ ⊥ [100] and $\boldsymbol{B}$ ∥ [001]<br>• $\boldsymbol{E}$ ⊥ [100] and $\boldsymbol{B}$ ⊥ [100]
$Pnc'2'$ (30.114) $\mathbb{Z}_1$	• $\boldsymbol{E}$ ∥ [010] and $\boldsymbol{B}$ ∥ [001]
P_bc (7.29) $\mathbb{Z}_1$	• $\boldsymbol{E}$ ∥ [010] and strain ⊥ [100]<br>• $\boldsymbol{E}$ ∥ [001] and strain ⊥ [100]<br>• $\boldsymbol{E}$ ⊥ [100]
$P2_1'/c'$ (14.79) $\mathbb{Z}_2 \times \mathbb{Z}_4$	• $\boldsymbol{B}$ ∥ [010] and strain ⊥ [100]<br>• $\boldsymbol{B}$ ∥ [001] and strain ⊥ [100]<br>• $\boldsymbol{B}$ ⊥ [100]
$Pb'a2'$ (32.137) $\mathbb{Z}_1$	• $\boldsymbol{E}$ ∥ [001] and $\boldsymbol{B}$ ∥ [010]
$Pc'c'2$ (27.81) $\mathbb{Z}_2$	• $\boldsymbol{E}$ ∥ [010] and $\boldsymbol{B}$ ∥ [010]
P_bnc2 (30.117) $\mathbb{Z}_2$	• $\boldsymbol{E}$ ∥ [010]

Continued on next page

...continued

Subgroup & SI group	Perturbation
$Pc'c'a$ (54.342) $\mathbb{Z}_2 \times \mathbb{Z}_2$	• $\boldsymbol{B} \parallel [010]$
$P_S\bar{1}$ (2.7) $\mathbb{Z}_2 \times \mathbb{Z}_2 \times \mathbb{Z}_4$	• strain in generic direction
P_c2/c (13.72) $\mathbb{Z}_2 \times \mathbb{Z}_2$	• strain $\perp$ [010]
P_b2/c (13.71) $\mathbb{Z}_2 \times \mathbb{Z}_2$	• strain $\perp$ [100]
$P2$ (3.1) $\mathbb{Z}_2$	• $\boldsymbol{E} \parallel [001]$ and $\boldsymbol{B} \parallel [001]$ and strain $\perp$ [001]
$P2/c$ (13.65) $\mathbb{Z}_2 \times \mathbb{Z}_2$	• $\boldsymbol{B} \parallel [001]$ and strain $\perp$ [001]
P_a2 (3.4) $\mathbb{Z}_2$	• $\boldsymbol{E} \parallel [001]$ and strain $\perp$ [001]
$Pm'a'2$ (28.91) $\mathbb{Z}_2$	• $\boldsymbol{E} \parallel [001]$ and $\boldsymbol{B} \parallel [001]$
$Pm'na'$ (53.328) $\mathbb{Z}_2 \times \mathbb{Z}_2$	• $\boldsymbol{B} \parallel [001]$
P_bba2 (32.140) $\mathbb{Z}_1$	• $\boldsymbol{E} \parallel [001]$
P_a2/c (13.70) $\mathbb{Z}_2 \times \mathbb{Z}_2$	• strain $\perp$ [001]

Table. S 395: Subgroups of P_cban (50.285) that can be realized by applying $\boldsymbol{E}/\boldsymbol{B}$ fields and/or uniaxial strains.

Subgroup & SI group	Perturbation
P_S1 (1.3) $\mathbb{Z}_1$	• $\boldsymbol{E} \parallel [100]$ and strain in generic direction<br>• $\boldsymbol{E} \parallel [010]$ and strain in generic direction<br>• $\boldsymbol{E} \parallel [001]$ and strain in generic direction<br>• $\boldsymbol{E} \perp [100]$ and strain $\perp$ [010]<br>• $\boldsymbol{E} \perp [100]$ and strain $\perp$ [001]<br>• $\boldsymbol{E} \perp [100]$ and strain in generic direction<br>• $\boldsymbol{E} \perp [010]$ and strain $\perp$ [100]<br>• $\boldsymbol{E} \perp [010]$ and strain $\perp$ [001]<br>• $\boldsymbol{E} \perp [010]$ and strain in generic direction<br>• $\boldsymbol{E} \perp [001]$ and strain $\perp$ [100]<br>• $\boldsymbol{E} \perp [001]$ and strain $\perp$ [010]<br>• $\boldsymbol{E} \perp [001]$ and strain in generic direction<br>• $\boldsymbol{E}$ in generic direction
$P2$ (3.1) $\mathbb{Z}_2$	• $\boldsymbol{E} \parallel [100]$ and $\boldsymbol{B} \parallel [100]$ and strain $\perp$ [100]

Continued on next page

. . . continued

Subgroup & SI group	Perturbation
$P2'$ (3.3) $\mathbb{Z}_1$	• $\boldsymbol{E} \parallel [100]$ and $\boldsymbol{B} \parallel [010]$ and strain $\perp [100]$<br>• $\boldsymbol{E} \parallel [100]$ and $\boldsymbol{B} \parallel [001]$ and strain $\perp [100]$<br>• $\boldsymbol{E} \parallel [100]$ and $\boldsymbol{B} \perp [100]$
P_a2 (3.4) $\mathbb{Z}_2$	• $\boldsymbol{E} \parallel [100]$ and strain $\perp [100]$
Pc (7.24) $\mathbb{Z}_1$	• $\boldsymbol{E} \parallel [100]$ and $\boldsymbol{B} \parallel [001]$ and strain $\perp [001]$<br>• $\boldsymbol{E} \parallel [010]$ and $\boldsymbol{B} \parallel [001]$ and strain $\perp [001]$<br>• $\boldsymbol{E} \perp [001]$ and $\boldsymbol{B} \parallel [001]$
Pc' (7.26) $\mathbb{Z}_1$	• $\boldsymbol{E} \parallel [100]$ and $\boldsymbol{B} \parallel [100]$ and strain $\perp [001]$<br>• $\boldsymbol{E} \parallel [100]$ and $\boldsymbol{B} \parallel [010]$ and strain $\perp [001]$<br>• $\boldsymbol{E} \parallel [100]$ and $\boldsymbol{B} \perp [001]$<br>• $\boldsymbol{E} \parallel [010]$ and $\boldsymbol{B} \parallel [100]$ and strain $\perp [001]$<br>• $\boldsymbol{E} \parallel [010]$ and $\boldsymbol{B} \parallel [010]$ and strain $\perp [001]$<br>• $\boldsymbol{E} \parallel [010]$ and $\boldsymbol{B} \perp [001]$<br>• $\boldsymbol{E} \perp [001]$ and $\boldsymbol{B} \parallel [100]$<br>• $\boldsymbol{E} \perp [001]$ and $\boldsymbol{B} \parallel [010]$<br>• $\boldsymbol{E} \perp [001]$ and $\boldsymbol{B} \perp [001]$
P_bc (7.29) $\mathbb{Z}_1$	• $\boldsymbol{E} \parallel [100]$ and strain $\perp [001]$<br>• $\boldsymbol{E} \parallel [010]$ and strain $\perp [001]$<br>• $\boldsymbol{E} \perp [001]$
Pc (7.24) $\mathbb{Z}_1$	• $\boldsymbol{E} \parallel [100]$ and $\boldsymbol{B} \parallel [010]$ and strain $\perp [010]$<br>• $\boldsymbol{E} \parallel [001]$ and $\boldsymbol{B} \parallel [010]$ and strain $\perp [010]$<br>• $\boldsymbol{E} \perp [010]$ and $\boldsymbol{B} \parallel [010]$
$Pn'c2'$ (30.113) $\mathbb{Z}_1$	• $\boldsymbol{E} \parallel [100]$ and $\boldsymbol{B} \parallel [010]$
Pc' (7.26) $\mathbb{Z}_1$	• $\boldsymbol{E} \parallel [100]$ and $\boldsymbol{B} \parallel [100]$ and strain $\perp [010]$<br>• $\boldsymbol{E} \parallel [100]$ and $\boldsymbol{B} \parallel [001]$ and strain $\perp [010]$<br>• $\boldsymbol{E} \parallel [100]$ and $\boldsymbol{B} \perp [010]$<br>• $\boldsymbol{E} \parallel [001]$ and $\boldsymbol{B} \parallel [100]$ and strain $\perp [010]$<br>• $\boldsymbol{E} \parallel [001]$ and $\boldsymbol{B} \parallel [001]$ and strain $\perp [010]$<br>• $\boldsymbol{E} \parallel [001]$ and $\boldsymbol{B} \perp [010]$<br>• $\boldsymbol{E} \perp [010]$ and $\boldsymbol{B} \parallel [100]$<br>• $\boldsymbol{E} \perp [010]$ and $\boldsymbol{B} \parallel [001]$<br>• $\boldsymbol{E} \perp [010]$ and $\boldsymbol{B} \perp [010]$
$Pn'n2'$ (34.158) $\mathbb{Z}_1$	• $\boldsymbol{E} \parallel [100]$ and $\boldsymbol{B} \parallel [001]$
$Pn'n'2$ (34.159) $\mathbb{Z}_1$	• $\boldsymbol{E} \parallel [100]$ and $\boldsymbol{B} \parallel [100]$

Continued on next page

...continued

Subgroup & SI group	Perturbation
P_ac (7.27) $\mathbb{Z}_1$	• $\boldsymbol{E} \parallel [100]$ and strain $\perp [010]$<br>• $\boldsymbol{E} \parallel [001]$ and strain $\perp [010]$<br>• $\boldsymbol{E} \perp [010]$
P_anc2 (30.116) $\mathbb{Z}_1$	• $\boldsymbol{E} \parallel [100]$
Pc (7.24) $\mathbb{Z}_1$	• $\boldsymbol{E} \parallel [010]$ and $\boldsymbol{B} \parallel [100]$ and strain $\perp [100]$<br>• $\boldsymbol{E} \parallel [001]$ and $\boldsymbol{B} \parallel [100]$ and strain $\perp [100]$<br>• $\boldsymbol{E} \perp [100]$ and $\boldsymbol{B} \parallel [100]$
Pc' (7.26) $\mathbb{Z}_1$	• $\boldsymbol{E} \parallel [010]$ and $\boldsymbol{B} \parallel [010]$ and strain $\perp [100]$<br>• $\boldsymbol{E} \parallel [010]$ and $\boldsymbol{B} \parallel [001]$ and strain $\perp [100]$<br>• $\boldsymbol{E} \parallel [010]$ and $\boldsymbol{B} \perp [100]$<br>• $\boldsymbol{E} \parallel [001]$ and $\boldsymbol{B} \parallel [010]$ and strain $\perp [100]$<br>• $\boldsymbol{E} \parallel [001]$ and $\boldsymbol{B} \parallel [001]$ and strain $\perp [100]$<br>• $\boldsymbol{E} \parallel [001]$ and $\boldsymbol{B} \perp [100]$<br>• $\boldsymbol{E} \perp [100]$ and $\boldsymbol{B} \parallel [010]$<br>• $\boldsymbol{E} \perp [100]$ and $\boldsymbol{B} \parallel [001]$<br>• $\boldsymbol{E} \perp [100]$ and $\boldsymbol{B} \perp [100]$
P_ac (7.27) $\mathbb{Z}_1$	• $\boldsymbol{E} \parallel [010]$ and strain $\perp [100]$<br>• $\boldsymbol{E} \parallel [001]$ and strain $\perp [100]$<br>• $\boldsymbol{E} \perp [100]$
$P\bar{1}$ (2.4) $\mathbb{Z}_2 \times \mathbb{Z}_2 \times \mathbb{Z}_2 \times \mathbb{Z}_4$	• $\boldsymbol{B} \parallel [100]$ and strain in generic direction<br>• $\boldsymbol{B} \parallel [010]$ and strain in generic direction<br>• $\boldsymbol{B} \parallel [001]$ and strain in generic direction<br>• $\boldsymbol{B} \perp [100]$ and strain $\perp [010]$<br>• $\boldsymbol{B} \perp [100]$ and strain $\perp [001]$<br>• $\boldsymbol{B} \perp [100]$ and strain in generic direction<br>• $\boldsymbol{B} \perp [010]$ and strain $\perp [100]$<br>• $\boldsymbol{B} \perp [010]$ and strain $\perp [001]$<br>• $\boldsymbol{B} \perp [010]$ and strain in generic direction<br>• $\boldsymbol{B} \perp [001]$ and strain $\perp [100]$<br>• $\boldsymbol{B} \perp [001]$ and strain $\perp [010]$<br>• $\boldsymbol{B} \perp [001]$ and strain in generic direction<br>• $\boldsymbol{B}$ in generic direction
$P2/c$ (13.65) $\mathbb{Z}_2 \times \mathbb{Z}_2$	• $\boldsymbol{B} \parallel [100]$ and strain $\perp [100]$
$P2'/c'$ (13.69) $\mathbb{Z}_2 \times \mathbb{Z}_2 \times \mathbb{Z}_4$	• $\boldsymbol{B} \parallel [010]$ and strain $\perp [100]$<br>• $\boldsymbol{B} \parallel [001]$ and strain $\perp [100]$<br>• $\boldsymbol{B} \perp [100]$
$P_S\bar{1}$ (2.7) $\mathbb{Z}_2 \times \mathbb{Z}_2 \times \mathbb{Z}_4$	• strain in generic direction

Continued on next page

...continued

Subgroup & SI group	Perturbation
P_a2/c (13.70) $\mathbb{Z}_2 \times \mathbb{Z}_2$	• strain $\perp$ [100]
$P2$ (3.1) $\mathbb{Z}_2$	• $\boldsymbol{E} \parallel$ [010] and $\boldsymbol{B} \parallel$ [010] and strain $\perp$ [010]
$Pn'n'2$ (34.159) $\mathbb{Z}_1$	• $\boldsymbol{E} \parallel$ [010] and $\boldsymbol{B} \parallel$ [010]
$P2/c$ (13.65) $\mathbb{Z}_2 \times \mathbb{Z}_2$	• $\boldsymbol{B} \parallel$ [010] and strain $\perp$ [010]
$P2'$ (3.3) $\mathbb{Z}_1$	• $\boldsymbol{E} \parallel$ [010] and $\boldsymbol{B} \parallel$ [100] and strain $\perp$ [010]<br>• $\boldsymbol{E} \parallel$ [010] and $\boldsymbol{B} \parallel$ [001] and strain $\perp$ [010]<br>• $\boldsymbol{E} \parallel$ [010] and $\boldsymbol{B} \perp$ [010]
$Pn'c2'$ (30.113) $\mathbb{Z}_1$	• $\boldsymbol{E} \parallel$ [010] and $\boldsymbol{B} \parallel$ [100]
$Pn'n2'$ (34.158) $\mathbb{Z}_1$	• $\boldsymbol{E} \parallel$ [010] and $\boldsymbol{B} \parallel$ [001]
$P2'/c'$ (13.69) $\mathbb{Z}_2 \times \mathbb{Z}_2 \times \mathbb{Z}_4$	• $\boldsymbol{B} \parallel$ [100] and strain $\perp$ [010]<br>• $\boldsymbol{B} \parallel$ [001] and strain $\perp$ [010]<br>• $\boldsymbol{B} \perp$ [010]
P_a2 (3.4) $\mathbb{Z}_2$	• $\boldsymbol{E} \parallel$ [010] and strain $\perp$ [010]
P_anc2 (30.116) $\mathbb{Z}_1$	• $\boldsymbol{E} \parallel$ [010]
P_a2/c (13.70) $\mathbb{Z}_2 \times \mathbb{Z}_2$	• strain $\perp$ [010]
$P2$ (3.1) $\mathbb{Z}_2$	• $\boldsymbol{E} \parallel$ [001] and $\boldsymbol{B} \parallel$ [001] and strain $\perp$ [001]
$Pn'n'2$ (34.159) $\mathbb{Z}_1$	• $\boldsymbol{E} \parallel$ [001] and $\boldsymbol{B} \parallel$ [001]
$P2/c$ (13.65) $\mathbb{Z}_2 \times \mathbb{Z}_2$	• $\boldsymbol{B} \parallel$ [001] and strain $\perp$ [001]
$Pn'n'n$ (48.260) $\mathbb{Z}_2$	• $\boldsymbol{B} \parallel$ [001]
$P2_1'$ (4.9) $\mathbb{Z}_1$	• $\boldsymbol{E} \parallel$ [001] and $\boldsymbol{B} \parallel$ [100] and strain $\perp$ [001]<br>• $\boldsymbol{E} \parallel$ [001] and $\boldsymbol{B} \parallel$ [010] and strain $\perp$ [001]<br>• $\boldsymbol{E} \parallel$ [001] and $\boldsymbol{B} \perp$ [001]
$Pn'a2_1'$ (33.146) $\mathbb{Z}_1$	• $\boldsymbol{E} \parallel$ [001] and $\boldsymbol{B} \parallel$ [100]
$Pn'a2_1'$ (33.146) $\mathbb{Z}_1$	• $\boldsymbol{E} \parallel$ [001] and $\boldsymbol{B} \parallel$ [010]
$P2_1'/c'$ (14.79) $\mathbb{Z}_2 \times \mathbb{Z}_4$	• $\boldsymbol{B} \parallel$ [100] and strain $\perp$ [001]<br>• $\boldsymbol{B} \parallel$ [010] and strain $\perp$ [001]<br>• $\boldsymbol{B} \perp$ [001]
$Pn'n'a$ (52.310) $\mathbb{Z}_2$	• $\boldsymbol{B} \parallel$ [010]
$Pn'n'a$ (52.310) $\mathbb{Z}_2$	• $\boldsymbol{B} \parallel$ [100]
P_b2 (3.5) $\mathbb{Z}_2$	• $\boldsymbol{E} \parallel$ [001] and strain $\perp$ [001]
P_cba2 (32.139) $\mathbb{Z}_2$	• $\boldsymbol{E} \parallel$ [001]

Continued on next page

...continued

Subgroup & SI group	Perturbation
P_b2/c (13.71) $\mathbb{Z}_2 \times \mathbb{Z}_2$	• strain $\perp$ [001]

Table. S 396: Subgroups of P_Aban (50.286) that can be realized by applying $\boldsymbol{E}/\boldsymbol{B}$ fields and/or uniaxial strains.

Subgroup & SI group	Perturbation
P_S1 (1.3) $\mathbb{Z}_1$	• $\boldsymbol{E}$ $\parallel$ [100] and strain in generic direction<br>• $\boldsymbol{E}$ $\parallel$ [010] and strain in generic direction<br>• $\boldsymbol{E}$ $\parallel$ [001] and strain in generic direction<br>• $\boldsymbol{E}$ $\perp$ [100] and strain $\perp$ [010]<br>• $\boldsymbol{E}$ $\perp$ [100] and strain $\perp$ [001]<br>• $\boldsymbol{E}$ $\perp$ [100] and strain in generic direction<br>• $\boldsymbol{E}$ $\perp$ [010] and strain $\perp$ [100]<br>• $\boldsymbol{E}$ $\perp$ [010] and strain $\perp$ [001]<br>• $\boldsymbol{E}$ $\perp$ [010] and strain in generic direction<br>• $\boldsymbol{E}$ $\perp$ [001] and strain $\perp$ [100]<br>• $\boldsymbol{E}$ $\perp$ [001] and strain $\perp$ [010]<br>• $\boldsymbol{E}$ $\perp$ [001] and strain in generic direction<br>• $\boldsymbol{E}$ in generic direction
$P2'$ (3.3) $\mathbb{Z}_1$	• $\boldsymbol{E}$ $\parallel$ [100] and $\boldsymbol{B}$ $\parallel$ [010] and strain $\perp$ [100]<br>• $\boldsymbol{E}$ $\parallel$ [100] and $\boldsymbol{B}$ $\parallel$ [001] and strain $\perp$ [100]<br>• $\boldsymbol{E}$ $\parallel$ [100] and $\boldsymbol{B}$ $\perp$ [100]
$P2$ (3.1) $\mathbb{Z}_2$	• $\boldsymbol{E}$ $\parallel$ [100] and $\boldsymbol{B}$ $\parallel$ [100] and strain $\perp$ [100]
P_a2 (3.4) $\mathbb{Z}_2$	• $\boldsymbol{E}$ $\parallel$ [100] and strain $\perp$ [100]
Pc' (7.26) $\mathbb{Z}_1$	• $\boldsymbol{E}$ $\parallel$ [100] and $\boldsymbol{B}$ $\parallel$ [100] and strain $\perp$ [001]<br>• $\boldsymbol{E}$ $\parallel$ [100] and $\boldsymbol{B}$ $\parallel$ [010] and strain $\perp$ [001]<br>• $\boldsymbol{E}$ $\parallel$ [100] and $\boldsymbol{B}$ $\perp$ [001]<br>• $\boldsymbol{E}$ $\parallel$ [010] and $\boldsymbol{B}$ $\parallel$ [100] and strain $\perp$ [001]<br>• $\boldsymbol{E}$ $\parallel$ [010] and $\boldsymbol{B}$ $\parallel$ [010] and strain $\perp$ [001]<br>• $\boldsymbol{E}$ $\parallel$ [010] and $\boldsymbol{B}$ $\perp$ [001]<br>• $\boldsymbol{E}$ $\perp$ [001] and $\boldsymbol{B}$ $\parallel$ [100]<br>• $\boldsymbol{E}$ $\perp$ [001] and $\boldsymbol{B}$ $\parallel$ [010]<br>• $\boldsymbol{E}$ $\perp$ [001] and $\boldsymbol{B}$ $\perp$ [001]

Continued on next page

...continued

Subgroup & SI group	Perturbation
Pc (7.24) $\mathbb{Z}_1$	• $\boldsymbol{E}$ ∥ [100] and $\boldsymbol{B}$ ∥ [001] and strain ⊥ [001]<br>• $\boldsymbol{E}$ ∥ [010] and $\boldsymbol{B}$ ∥ [001] and strain ⊥ [001]<br>• $\boldsymbol{E}$ ⊥ [001] and $\boldsymbol{B}$ ∥ [001]
P_Cc (7.30) $\mathbb{Z}_1$	• $\boldsymbol{E}$ ∥ [100] and strain ⊥ [001]<br>• $\boldsymbol{E}$ ∥ [010] and strain ⊥ [001]<br>• $\boldsymbol{E}$ ⊥ [001]
Pc (7.24) $\mathbb{Z}_1$	• $\boldsymbol{E}$ ∥ [100] and $\boldsymbol{B}$ ∥ [010] and strain ⊥ [010]<br>• $\boldsymbol{E}$ ∥ [001] and $\boldsymbol{B}$ ∥ [010] and strain ⊥ [010]<br>• $\boldsymbol{E}$ ⊥ [010] and $\boldsymbol{B}$ ∥ [010]
$Pc'c2'$ (27.80) $\mathbb{Z}_1$	• $\boldsymbol{E}$ ∥ [100] and $\boldsymbol{B}$ ∥ [010]
Pc' (7.26) $\mathbb{Z}_1$	• $\boldsymbol{E}$ ∥ [100] and $\boldsymbol{B}$ ∥ [100] and strain ⊥ [010]<br>• $\boldsymbol{E}$ ∥ [100] and $\boldsymbol{B}$ ∥ [001] and strain ⊥ [010]<br>• $\boldsymbol{E}$ ∥ [100] and $\boldsymbol{B}$ ⊥ [010]<br>• $\boldsymbol{E}$ ∥ [001] and $\boldsymbol{B}$ ∥ [100] and strain ⊥ [010]<br>• $\boldsymbol{E}$ ∥ [001] and $\boldsymbol{B}$ ∥ [001] and strain ⊥ [010]<br>• $\boldsymbol{E}$ ∥ [001] and $\boldsymbol{B}$ ⊥ [010]<br>• $\boldsymbol{E}$ ⊥ [010] and $\boldsymbol{B}$ ∥ [100]<br>• $\boldsymbol{E}$ ⊥ [010] and $\boldsymbol{B}$ ∥ [001]<br>• $\boldsymbol{E}$ ⊥ [010] and $\boldsymbol{B}$ ⊥ [010]
$Pn'c'2$ (30.115) $\mathbb{Z}_1$	• $\boldsymbol{E}$ ∥ [100] and $\boldsymbol{B}$ ∥ [100]
$Pn'n2'$ (34.158) $\mathbb{Z}_1$	• $\boldsymbol{E}$ ∥ [100] and $\boldsymbol{B}$ ∥ [001]
P_Cc (7.30) $\mathbb{Z}_1$	• $\boldsymbol{E}$ ∥ [100] and strain ⊥ [010]<br>• $\boldsymbol{E}$ ∥ [001] and strain ⊥ [010]<br>• $\boldsymbol{E}$ ⊥ [010]
P_Cnc2 (30.121) $\mathbb{Z}_1$	• $\boldsymbol{E}$ ∥ [100]
Pc' (7.26) $\mathbb{Z}_1$	• $\boldsymbol{E}$ ∥ [010] and $\boldsymbol{B}$ ∥ [010] and strain ⊥ [100]<br>• $\boldsymbol{E}$ ∥ [010] and $\boldsymbol{B}$ ∥ [001] and strain ⊥ [100]<br>• $\boldsymbol{E}$ ∥ [010] and $\boldsymbol{B}$ ⊥ [100]<br>• $\boldsymbol{E}$ ∥ [001] and $\boldsymbol{B}$ ∥ [010] and strain ⊥ [100]<br>• $\boldsymbol{E}$ ∥ [001] and $\boldsymbol{B}$ ∥ [001] and strain ⊥ [100]<br>• $\boldsymbol{E}$ ∥ [001] and $\boldsymbol{B}$ ⊥ [100]<br>• $\boldsymbol{E}$ ⊥ [100] and $\boldsymbol{B}$ ∥ [010]<br>• $\boldsymbol{E}$ ⊥ [100] and $\boldsymbol{B}$ ∥ [001]<br>• $\boldsymbol{E}$ ⊥ [100] and $\boldsymbol{B}$ ⊥ [100]

Continued on next page

...continued

Subgroup & SI group	Perturbation
Pc (7.24) $\mathbb{Z}_1$	• $\boldsymbol{E} \parallel [010]$ and $\boldsymbol{B} \parallel [100]$ and strain $\perp [100]$<br>• $\boldsymbol{E} \parallel [001]$ and $\boldsymbol{B} \parallel [100]$ and strain $\perp [100]$<br>• $\boldsymbol{E} \perp [100]$ and $\boldsymbol{B} \parallel [100]$
P_ac (7.27) $\mathbb{Z}_1$	• $\boldsymbol{E} \parallel [010]$ and strain $\perp [100]$<br>• $\boldsymbol{E} \parallel [001]$ and strain $\perp [100]$<br>• $\boldsymbol{E} \perp [100]$
$P\bar{1}$ (2.4) $\mathbb{Z}_2 \times \mathbb{Z}_2 \times \mathbb{Z}_2 \times \mathbb{Z}_4$	• $\boldsymbol{B} \parallel [100]$ and strain in generic direction<br>• $\boldsymbol{B} \parallel [010]$ and strain in generic direction<br>• $\boldsymbol{B} \parallel [001]$ and strain in generic direction<br>• $\boldsymbol{B} \perp [100]$ and strain $\perp [010]$<br>• $\boldsymbol{B} \perp [100]$ and strain $\perp [001]$<br>• $\boldsymbol{B} \perp [100]$ and strain in generic direction<br>• $\boldsymbol{B} \perp [010]$ and strain $\perp [100]$<br>• $\boldsymbol{B} \perp [010]$ and strain $\perp [001]$<br>• $\boldsymbol{B} \perp [010]$ and strain in generic direction<br>• $\boldsymbol{B} \perp [001]$ and strain $\perp [100]$<br>• $\boldsymbol{B} \perp [001]$ and strain $\perp [010]$<br>• $\boldsymbol{B} \perp [001]$ and strain in generic direction<br>• $\boldsymbol{B}$ in generic direction
$P2'/c'$ (13.69) $\mathbb{Z}_2 \times \mathbb{Z}_2 \times \mathbb{Z}_4$	• $\boldsymbol{B} \parallel [010]$ and strain $\perp [100]$<br>• $\boldsymbol{B} \parallel [001]$ and strain $\perp [100]$<br>• $\boldsymbol{B} \perp [100]$
$P2/c$ (13.65) $\mathbb{Z}_2 \times \mathbb{Z}_2$	• $\boldsymbol{B} \parallel [100]$ and strain $\perp [100]$
$P_S\bar{1}$ (2.7) $\mathbb{Z}_2 \times \mathbb{Z}_2 \times \mathbb{Z}_4$	• strain in generic direction
P_a2/c (13.70) $\mathbb{Z}_2 \times \mathbb{Z}_2$	• strain $\perp [100]$
$P2$ (3.1) $\mathbb{Z}_2$	• $\boldsymbol{E} \parallel [010]$ and $\boldsymbol{B} \parallel [010]$ and strain $\perp [010]$
$Pb'a'2$ (32.138) $\mathbb{Z}_2$	• $\boldsymbol{E} \parallel [010]$ and $\boldsymbol{B} \parallel [010]$
$P2/c$ (13.65) $\mathbb{Z}_2 \times \mathbb{Z}_2$	• $\boldsymbol{B} \parallel [010]$ and strain $\perp [010]$
$P2_1'$ (4.9) $\mathbb{Z}_1$	• $\boldsymbol{E} \parallel [010]$ and $\boldsymbol{B} \parallel [100]$ and strain $\perp [010]$<br>• $\boldsymbol{E} \parallel [010]$ and $\boldsymbol{B} \parallel [001]$ and strain $\perp [010]$<br>• $\boldsymbol{E} \parallel [010]$ and $\boldsymbol{B} \perp [010]$
$Pna'2_1'$ (33.147) $\mathbb{Z}_1$	• $\boldsymbol{E} \parallel [010]$ and $\boldsymbol{B} \parallel [001]$
$Pca'2_1'$ (29.102) $\mathbb{Z}_1$	• $\boldsymbol{E} \parallel [010]$ and $\boldsymbol{B} \parallel [100]$

Continued on next page

...continued

Subgroup & SI group	Perturbation
$P2_1'/c'$ (14.79) $\mathbb{Z}_2 \times \mathbb{Z}_4$	• $\boldsymbol{B}$ ∥ [100] and strain ⊥ [010]<br>• $\boldsymbol{B}$ ∥ [001] and strain ⊥ [010]<br>• $\boldsymbol{B}$ ⊥ [010]
P_C2 (3.6) $\mathbb{Z}_1$	• $\boldsymbol{E}$ ∥ [010] and strain ⊥ [010]
P_Bnc2 (30.120) $\mathbb{Z}_2$	• $\boldsymbol{E}$ ∥ [010]
P_C2/c (13.74) $\mathbb{Z}_2$	• strain ⊥ [010]
$P2_1'$ (4.9) $\mathbb{Z}_1$	• $\boldsymbol{E}$ ∥ [001] and $\boldsymbol{B}$ ∥ [100] and strain ⊥ [001]<br>• $\boldsymbol{E}$ ∥ [001] and $\boldsymbol{B}$ ∥ [010] and strain ⊥ [001]<br>• $\boldsymbol{E}$ ∥ [001] and $\boldsymbol{B}$ ⊥ [001]
$Pc'a2_1'$ (29.101) $\mathbb{Z}_1$	• $\boldsymbol{E}$ ∥ [001] and $\boldsymbol{B}$ ∥ [010]
$Pn'a2_1'$ (33.146) $\mathbb{Z}_1$	• $\boldsymbol{E}$ ∥ [001] and $\boldsymbol{B}$ ∥ [100]
$P2_1'/c'$ (14.79) $\mathbb{Z}_2 \times \mathbb{Z}_4$	• $\boldsymbol{B}$ ∥ [100] and strain ⊥ [001]<br>• $\boldsymbol{B}$ ∥ [010] and strain ⊥ [001]<br>• $\boldsymbol{B}$ ⊥ [001]
$Pc'ca'$ (54.344) $\mathbb{Z}_2 \times \mathbb{Z}_2$	• $\boldsymbol{B}$ ∥ [010]
$Pb'cn'$ (60.424) $\mathbb{Z}_2 \times \mathbb{Z}_2$	• $\boldsymbol{B}$ ∥ [100]
$P2$ (3.1) $\mathbb{Z}_2$	• $\boldsymbol{E}$ ∥ [001] and $\boldsymbol{B}$ ∥ [001] and strain ⊥ [001]
$Pn'c'2$ (30.115) $\mathbb{Z}_1$	• $\boldsymbol{E}$ ∥ [001] and $\boldsymbol{B}$ ∥ [001]
$P2/c$ (13.65) $\mathbb{Z}_2 \times \mathbb{Z}_2$	• $\boldsymbol{B}$ ∥ [001] and strain ⊥ [001]
$Pnn'a'$ (52.311) $\mathbb{Z}_2$	• $\boldsymbol{B}$ ∥ [001]
P_C2 (3.6) $\mathbb{Z}_1$	• $\boldsymbol{E}$ ∥ [001] and strain ⊥ [001]
P_Aba2 (32.142) $\mathbb{Z}_1$	• $\boldsymbol{E}$ ∥ [001]
P_C2/c (13.74) $\mathbb{Z}_2$	• strain ⊥ [001]

Table. S 397: Subgroups of P_Cban (50.287) that can be realized by applying $\boldsymbol{E}/\boldsymbol{B}$ fields and/or uniaxial strains.

Subgroup & SI group	Perturbation

Continued on next page

Subgroup & SI group	Perturbation
Pm' (6.20) $\mathbb{Z}_1$	• $\boldsymbol{E} \parallel [100]$ and $\boldsymbol{B} \parallel [100]$ and strain $\perp [001]$<br>• $\boldsymbol{E} \parallel [100]$ and $\boldsymbol{B} \parallel [010]$ and strain $\perp [001]$<br>• $\boldsymbol{E} \parallel [100]$ and $\boldsymbol{B} \perp [001]$<br>• $\boldsymbol{E} \parallel [010]$ and $\boldsymbol{B} \parallel [100]$ and strain $\perp [001]$<br>• $\boldsymbol{E} \parallel [010]$ and $\boldsymbol{B} \parallel [010]$ and strain $\perp [001]$<br>• $\boldsymbol{E} \parallel [010]$ and $\boldsymbol{B} \perp [001]$<br>• $\boldsymbol{E} \perp [001]$ and $\boldsymbol{B} \parallel [100]$<br>• $\boldsymbol{E} \perp [001]$ and $\boldsymbol{B} \parallel [010]$<br>• $\boldsymbol{E} \perp [001]$ and $\boldsymbol{B} \perp [001]$
Pm' (6.20) $\mathbb{Z}_1$	• $\boldsymbol{E} \parallel [100]$ and $\boldsymbol{B} \parallel [100]$ and strain $\perp [010]$<br>• $\boldsymbol{E} \parallel [100]$ and $\boldsymbol{B} \parallel [001]$ and strain $\perp [010]$<br>• $\boldsymbol{E} \parallel [100]$ and $\boldsymbol{B} \perp [010]$<br>• $\boldsymbol{E} \parallel [001]$ and $\boldsymbol{B} \parallel [100]$ and strain $\perp [010]$<br>• $\boldsymbol{E} \parallel [001]$ and $\boldsymbol{B} \parallel [001]$ and strain $\perp [010]$<br>• $\boldsymbol{E} \parallel [001]$ and $\boldsymbol{B} \perp [010]$<br>• $\boldsymbol{E} \perp [010]$ and $\boldsymbol{B} \parallel [100]$<br>• $\boldsymbol{E} \perp [010]$ and $\boldsymbol{B} \parallel [001]$<br>• $\boldsymbol{E} \perp [010]$ and $\boldsymbol{B} \perp [010]$
$P2$ (3.1) $\mathbb{Z}_2$	• $\boldsymbol{E} \parallel [100]$ and $\boldsymbol{B} \parallel [100]$ and strain $\perp [100]$
$Pm'm'2$ (25.60) $\mathbb{Z}_2$	• $\boldsymbol{E} \parallel [100]$ and $\boldsymbol{B} \parallel [100]$
P_S1 (1.3) $\mathbb{Z}_1$	• $\boldsymbol{E} \parallel [100]$ and strain in generic direction<br>• $\boldsymbol{E} \parallel [010]$ and strain in generic direction<br>• $\boldsymbol{E} \parallel [001]$ and strain in generic direction<br>• $\boldsymbol{E} \perp [100]$ and strain $\perp [010]$<br>• $\boldsymbol{E} \perp [100]$ and strain $\perp [001]$<br>• $\boldsymbol{E} \perp [100]$ and strain in generic direction<br>• $\boldsymbol{E} \perp [010]$ and strain $\perp [100]$<br>• $\boldsymbol{E} \perp [010]$ and strain $\perp [001]$<br>• $\boldsymbol{E} \perp [010]$ and strain in generic direction<br>• $\boldsymbol{E} \perp [001]$ and strain $\perp [100]$<br>• $\boldsymbol{E} \perp [001]$ and strain $\perp [010]$<br>• $\boldsymbol{E} \perp [001]$ and strain in generic direction<br>• $\boldsymbol{E}$ in generic direction
Pc (7.24) $\mathbb{Z}_1$	• $\boldsymbol{E} \parallel [100]$ and $\boldsymbol{B} \parallel [001]$ and strain $\perp [001]$<br>• $\boldsymbol{E} \parallel [010]$ and $\boldsymbol{B} \parallel [001]$ and strain $\perp [001]$<br>• $\boldsymbol{E} \perp [001]$ and $\boldsymbol{B} \parallel [001]$

Continued on next page

. . . continued

Subgroup & SI group	Perturbation
P_cc (7.28) $\mathbb{Z}_1$	• $\boldsymbol{E} \parallel [100]$ and strain $\perp [001]$<br>• $\boldsymbol{E} \parallel [010]$ and strain $\perp [001]$<br>• $\boldsymbol{E} \perp [001]$
Pc (7.24) $\mathbb{Z}_1$	• $\boldsymbol{E} \parallel [100]$ and $\boldsymbol{B} \parallel [010]$ and strain $\perp [010]$<br>• $\boldsymbol{E} \parallel [001]$ and $\boldsymbol{B} \parallel [010]$ and strain $\perp [010]$<br>• $\boldsymbol{E} \perp [010]$ and $\boldsymbol{B} \parallel [010]$
P_Ac (7.31) $\mathbb{Z}_1$	• $\boldsymbol{E} \parallel [100]$ and strain $\perp [010]$<br>• $\boldsymbol{E} \parallel [001]$ and strain $\perp [010]$<br>• $\boldsymbol{E} \perp [010]$
$P2_1'$ (4.9) $\mathbb{Z}_1$	• $\boldsymbol{E} \parallel [100]$ and $\boldsymbol{B} \parallel [010]$ and strain $\perp [100]$<br>• $\boldsymbol{E} \parallel [100]$ and $\boldsymbol{B} \parallel [001]$ and strain $\perp [100]$<br>• $\boldsymbol{E} \parallel [100]$ and $\boldsymbol{B} \perp [100]$
P_C2 (3.6) $\mathbb{Z}_1$	• $\boldsymbol{E} \parallel [100]$ and strain $\perp [100]$
$Pm'n2_1'$ (31.125) $\mathbb{Z}_1$	• $\boldsymbol{E} \parallel [100]$ and $\boldsymbol{B} \parallel [001]$
$Pm'c2_1'$ (26.68) $\mathbb{Z}_1$	• $\boldsymbol{E} \parallel [100]$ and $\boldsymbol{B} \parallel [010]$
P_Anc2 (30.119) $\mathbb{Z}_1$	• $\boldsymbol{E} \parallel [100]$
Pc (7.24) $\mathbb{Z}_1$	• $\boldsymbol{E} \parallel [010]$ and $\boldsymbol{B} \parallel [100]$ and strain $\perp [100]$<br>• $\boldsymbol{E} \parallel [001]$ and $\boldsymbol{B} \parallel [100]$ and strain $\perp [100]$<br>• $\boldsymbol{E} \perp [100]$ and $\boldsymbol{B} \parallel [100]$
$P2_1'$ (4.9) $\mathbb{Z}_1$	• $\boldsymbol{E} \parallel [010]$ and $\boldsymbol{B} \parallel [100]$ and strain $\perp [010]$<br>• $\boldsymbol{E} \parallel [010]$ and $\boldsymbol{B} \parallel [001]$ and strain $\perp [010]$<br>• $\boldsymbol{E} \parallel [010]$ and $\boldsymbol{B} \perp [010]$
$Pm'c2_1'$ (26.68) $\mathbb{Z}_1$	• $\boldsymbol{E} \parallel [010]$ and $\boldsymbol{B} \parallel [100]$
$P2'$ (3.3) $\mathbb{Z}_1$	• $\boldsymbol{E} \parallel [001]$ and $\boldsymbol{B} \parallel [100]$ and strain $\perp [001]$<br>• $\boldsymbol{E} \parallel [001]$ and $\boldsymbol{B} \parallel [010]$ and strain $\perp [001]$<br>• $\boldsymbol{E} \parallel [001]$ and $\boldsymbol{B} \perp [001]$
$Pm'a2'$ (28.89) $\mathbb{Z}_1$	• $\boldsymbol{E} \parallel [001]$ and $\boldsymbol{B} \parallel [100]$

Continued on next page

...continued

Subgroup & SI group	Perturbation
$P\bar{1}$ (2.4) $\mathbb{Z}_2 \times \mathbb{Z}_2 \times \mathbb{Z}_2 \times \mathbb{Z}_4$	• $\boldsymbol{B} \parallel [100]$ and strain in generic direction<br>• $\boldsymbol{B} \parallel [010]$ and strain in generic direction<br>• $\boldsymbol{B} \parallel [001]$ and strain in generic direction<br>• $\boldsymbol{B} \perp [100]$ and strain $\perp [010]$<br>• $\boldsymbol{B} \perp [100]$ and strain $\perp [001]$<br>• $\boldsymbol{B} \perp [100]$ and strain in generic direction<br>• $\boldsymbol{B} \perp [010]$ and strain $\perp [100]$<br>• $\boldsymbol{B} \perp [010]$ and strain $\perp [001]$<br>• $\boldsymbol{B} \perp [010]$ and strain in generic direction<br>• $\boldsymbol{B} \perp [001]$ and strain $\perp [100]$<br>• $\boldsymbol{B} \perp [001]$ and strain $\perp [010]$<br>• $\boldsymbol{B} \perp [001]$ and strain in generic direction<br>• $\boldsymbol{B}$ in generic direction
$P2/c$ (13.65) $\mathbb{Z}_2 \times \mathbb{Z}_2$	• $\boldsymbol{B} \parallel [100]$ and strain $\perp [100]$
$P2_1'/m'$ (11.54) $\mathbb{Z}_2 \times \mathbb{Z}_2 \times \mathbb{Z}_4$	• $\boldsymbol{B} \parallel [100]$ and strain $\perp [010]$<br>• $\boldsymbol{B} \parallel [001]$ and strain $\perp [010]$<br>• $\boldsymbol{B} \perp [010]$
$P2'/m'$ (10.46) $\mathbb{Z}_2 \times \mathbb{Z}_2 \times \mathbb{Z}_2 \times \mathbb{Z}_4$	• $\boldsymbol{B} \parallel [100]$ and strain $\perp [001]$<br>• $\boldsymbol{B} \parallel [010]$ and strain $\perp [001]$<br>• $\boldsymbol{B} \perp [001]$
$Pm'm'a$ (51.294) $\mathbb{Z}_2 \times \mathbb{Z}_2$	• $\boldsymbol{B} \parallel [100]$
Pm' (6.20) $\mathbb{Z}_1$	• $\boldsymbol{E} \parallel [010]$ and $\boldsymbol{B} \parallel [010]$ and strain $\perp [100]$<br>• $\boldsymbol{E} \parallel [010]$ and $\boldsymbol{B} \parallel [001]$ and strain $\perp [100]$<br>• $\boldsymbol{E} \parallel [010]$ and $\boldsymbol{B} \perp [100]$<br>• $\boldsymbol{E} \parallel [001]$ and $\boldsymbol{B} \parallel [010]$ and strain $\perp [100]$<br>• $\boldsymbol{E} \parallel [001]$ and $\boldsymbol{B} \parallel [001]$ and strain $\perp [100]$<br>• $\boldsymbol{E} \parallel [001]$ and $\boldsymbol{B} \perp [100]$<br>• $\boldsymbol{E} \perp [100]$ and $\boldsymbol{B} \parallel [010]$<br>• $\boldsymbol{E} \perp [100]$ and $\boldsymbol{B} \parallel [001]$<br>• $\boldsymbol{E} \perp [100]$ and $\boldsymbol{B} \perp [100]$
P_Ac (7.31) $\mathbb{Z}_1$	• $\boldsymbol{E} \parallel [010]$ and strain $\perp [100]$<br>• $\boldsymbol{E} \parallel [001]$ and strain $\perp [100]$<br>• $\boldsymbol{E} \perp [100]$
$Pm'n2_1'$ (31.125) $\mathbb{Z}_1$	• $\boldsymbol{E} \parallel [010]$ and $\boldsymbol{B} \parallel [001]$
$Pm'a2'$ (28.89) $\mathbb{Z}_1$	• $\boldsymbol{E} \parallel [001]$ and $\boldsymbol{B} \parallel [010]$

Continued on next page

. . . continued

Subgroup & SI group	Perturbation
$P2_1'/m'$ (11.54) $\mathbb{Z}_2\times\mathbb{Z}_2\times\mathbb{Z}_4$	• $\boldsymbol{B}$ ∥ [010] and strain ⊥ [100]<br>• $\boldsymbol{B}$ ∥ [001] and strain ⊥ [100]<br>• $\boldsymbol{B}$ ⊥ [100]
$P2$ (3.1) $\mathbb{Z}_2$	• $\boldsymbol{E}$ ∥ [010] and $\boldsymbol{B}$ ∥ [010] and strain ⊥ [010]
P_C2 (3.6) $\mathbb{Z}_1$	• $\boldsymbol{E}$ ∥ [010] and strain ⊥ [010]
$P2/c$ (13.65) $\mathbb{Z}_2\times\mathbb{Z}_2$	• $\boldsymbol{B}$ ∥ [010] and strain ⊥ [010]
$Pm'm'2$ (25.60) $\mathbb{Z}_2$	• $\boldsymbol{E}$ ∥ [010] and $\boldsymbol{B}$ ∥ [010]
P_Anc2 (30.119) $\mathbb{Z}_1$	• $\boldsymbol{E}$ ∥ [010]
$Pm'm'a$ (51.294) $\mathbb{Z}_2\times\mathbb{Z}_2$	• $\boldsymbol{B}$ ∥ [010]
$P2$ (3.1) $\mathbb{Z}_2$	• $\boldsymbol{E}$ ∥ [001] and $\boldsymbol{B}$ ∥ [001] and strain ⊥ [001]
P_a2 (3.4) $\mathbb{Z}_2$	• $\boldsymbol{E}$ ∥ [001] and strain ⊥ [001]
$P2/c$ (13.65) $\mathbb{Z}_2\times\mathbb{Z}_2$	• $\boldsymbol{B}$ ∥ [001] and strain ⊥ [001]
$Pm'm'2$ (25.60) $\mathbb{Z}_2$	• $\boldsymbol{E}$ ∥ [001] and $\boldsymbol{B}$ ∥ [001]
P_Cba2 (32.141) $\mathbb{Z}_1$	• $\boldsymbol{E}$ ∥ [001]
$Pm'm'n$ (59.409) $\mathbb{Z}_2\times\mathbb{Z}_2$	• $\boldsymbol{B}$ ∥ [001]
$P_S\bar{1}$ (2.7) $\mathbb{Z}_2\times\mathbb{Z}_2\times\mathbb{Z}_4$	• strain in generic direction
P_A2/c (13.73) $\mathbb{Z}_2$	• strain ⊥ [100]
P_A2/c (13.73) $\mathbb{Z}_2$	• strain ⊥ [010]
P_c2/c (13.72) $\mathbb{Z}_2\times\mathbb{Z}_2$	• strain ⊥ [001]

Table. S 398: Subgroups of P_Iban (50.288) that can be realized by applying $\boldsymbol{E}/\boldsymbol{B}$ fields and/or uniaxial strains.

Subgroup & SI group	Perturbation

Continued on next page

. . . continued

Subgroup & SI group	Perturbation
Pm' (6.20) $\mathbb{Z}_1$	• $\boldsymbol{E}$ || [100] and $\boldsymbol{B}$ || [100] and strain ⊥ [001]<br>• $\boldsymbol{E}$ || [100] and $\boldsymbol{B}$ || [010] and strain ⊥ [001]<br>• $\boldsymbol{E}$ || [100] and $\boldsymbol{B}$ ⊥ [001]<br>• $\boldsymbol{E}$ || [010] and $\boldsymbol{B}$ || [100] and strain ⊥ [001]<br>• $\boldsymbol{E}$ || [010] and $\boldsymbol{B}$ || [010] and strain ⊥ [001]<br>• $\boldsymbol{E}$ || [010] and $\boldsymbol{B}$ ⊥ [001]<br>• $\boldsymbol{E}$ ⊥ [001] and $\boldsymbol{B}$ || [100]<br>• $\boldsymbol{E}$ ⊥ [001] and $\boldsymbol{B}$ || [010]<br>• $\boldsymbol{E}$ ⊥ [001] and $\boldsymbol{B}$ ⊥ [001]
Pc' (7.26) $\mathbb{Z}_1$	• $\boldsymbol{E}$ || [100] and $\boldsymbol{B}$ || [100] and strain ⊥ [010]<br>• $\boldsymbol{E}$ || [100] and $\boldsymbol{B}$ || [001] and strain ⊥ [010]<br>• $\boldsymbol{E}$ || [100] and $\boldsymbol{B}$ ⊥ [010]<br>• $\boldsymbol{E}$ || [001] and $\boldsymbol{B}$ || [100] and strain ⊥ [010]<br>• $\boldsymbol{E}$ || [001] and $\boldsymbol{B}$ || [001] and strain ⊥ [010]<br>• $\boldsymbol{E}$ || [001] and $\boldsymbol{B}$ ⊥ [010]<br>• $\boldsymbol{E}$ ⊥ [010] and $\boldsymbol{B}$ || [100]<br>• $\boldsymbol{E}$ ⊥ [010] and $\boldsymbol{B}$ || [001]<br>• $\boldsymbol{E}$ ⊥ [010] and $\boldsymbol{B}$ ⊥ [010]
$P2$ (3.1) $\mathbb{Z}_2$	• $\boldsymbol{E}$ || [100] and $\boldsymbol{B}$ || [100] and strain ⊥ [100]
$Pm'a'2$ (28.91) $\mathbb{Z}_2$	• $\boldsymbol{E}$ || [100] and $\boldsymbol{B}$ || [100]
P_S1 (1.3) $\mathbb{Z}_1$	• $\boldsymbol{E}$ || [100] and strain in generic direction<br>• $\boldsymbol{E}$ || [010] and strain in generic direction<br>• $\boldsymbol{E}$ || [001] and strain in generic direction<br>• $\boldsymbol{E}$ ⊥ [100] and strain ⊥ [010]<br>• $\boldsymbol{E}$ ⊥ [100] and strain ⊥ [001]<br>• $\boldsymbol{E}$ ⊥ [100] and strain in generic direction<br>• $\boldsymbol{E}$ ⊥ [010] and strain ⊥ [100]<br>• $\boldsymbol{E}$ ⊥ [010] and strain ⊥ [001]<br>• $\boldsymbol{E}$ ⊥ [010] and strain in generic direction<br>• $\boldsymbol{E}$ ⊥ [001] and strain ⊥ [100]<br>• $\boldsymbol{E}$ ⊥ [001] and strain ⊥ [010]<br>• $\boldsymbol{E}$ ⊥ [001] and strain in generic direction<br>• $\boldsymbol{E}$ in generic direction
Pc (7.24) $\mathbb{Z}_1$	• $\boldsymbol{E}$ || [100] and $\boldsymbol{B}$ || [001] and strain ⊥ [001]<br>• $\boldsymbol{E}$ || [010] and $\boldsymbol{B}$ || [001] and strain ⊥ [001]<br>• $\boldsymbol{E}$ ⊥ [001] and $\boldsymbol{B}$ || [001]

Continued on next page

...continued

Subgroup & SI group	Perturbation
$P_A c$ (7.31) $\mathbb{Z}_1$	• $\boldsymbol{E}$ || [100] and strain $\perp$ [001]<br>• $\boldsymbol{E}$ || [010] and strain $\perp$ [001]<br>• $\boldsymbol{E} \perp$ [001]
Pc (7.24) $\mathbb{Z}_1$	• $\boldsymbol{E}$ || [100] and $\boldsymbol{B}$ || [010] and strain $\perp$ [010]<br>• $\boldsymbol{E}$ || [001] and $\boldsymbol{B}$ || [010] and strain $\perp$ [010]<br>• $\boldsymbol{E} \perp$ [010] and $\boldsymbol{B}$ || [010]
$P_C c$ (7.30) $\mathbb{Z}_1$	• $\boldsymbol{E}$ || [100] and strain $\perp$ [010]<br>• $\boldsymbol{E}$ || [001] and strain $\perp$ [010]<br>• $\boldsymbol{E} \perp$ [010]
$P2_1'$ (4.9) $\mathbb{Z}_1$	• $\boldsymbol{E}$ || [100] and $\boldsymbol{B}$ || [010] and strain $\perp$ [100]<br>• $\boldsymbol{E}$ || [100] and $\boldsymbol{B}$ || [001] and strain $\perp$ [100]<br>• $\boldsymbol{E}$ || [100] and $\boldsymbol{B} \perp$ [100]
$P_C 2$ (3.6) $\mathbb{Z}_1$	• $\boldsymbol{E}$ || [100] and strain $\perp$ [100]
$Pna'2_1'$ (33.147) $\mathbb{Z}_1$	• $\boldsymbol{E}$ || [100] and $\boldsymbol{B}$ || [001]
$Pm'c2_1'$ (26.68) $\mathbb{Z}_1$	• $\boldsymbol{E}$ || [100] and $\boldsymbol{B}$ || [010]
$P_I nc2$ (30.122) $\mathbb{Z}_1$	• $\boldsymbol{E}$ || [100]
Pc (7.24) $\mathbb{Z}_1$	• $\boldsymbol{E}$ || [010] and $\boldsymbol{B}$ || [100] and strain $\perp$ [100]<br>• $\boldsymbol{E}$ || [001] and $\boldsymbol{B}$ || [100] and strain $\perp$ [100]<br>• $\boldsymbol{E} \perp$ [100] and $\boldsymbol{B}$ || [100]
$P2_1'$ (4.9) $\mathbb{Z}_1$	• $\boldsymbol{E}$ || [010] and $\boldsymbol{B}$ || [100] and strain $\perp$ [010]<br>• $\boldsymbol{E}$ || [010] and $\boldsymbol{B}$ || [001] and strain $\perp$ [010]<br>• $\boldsymbol{E}$ || [010] and $\boldsymbol{B} \perp$ [010]
$Pm'c2_1'$ (26.68) $\mathbb{Z}_1$	• $\boldsymbol{E}$ || [010] and $\boldsymbol{B}$ || [100]
$P2_1'$ (4.9) $\mathbb{Z}_1$	• $\boldsymbol{E}$ || [001] and $\boldsymbol{B}$ || [100] and strain $\perp$ [001]<br>• $\boldsymbol{E}$ || [001] and $\boldsymbol{B}$ || [010] and strain $\perp$ [001]<br>• $\boldsymbol{E}$ || [001] and $\boldsymbol{B} \perp$ [001]
$Pc'a2_1'$ (29.101) $\mathbb{Z}_1$	• $\boldsymbol{E}$ || [001] and $\boldsymbol{B}$ || [100]

Continued on next page

...continued

Subgroup & SI group	Perturbation
$P\bar{1}$ (2.4) $\mathbb{Z}_2 \times \mathbb{Z}_2 \times \mathbb{Z}_2 \times \mathbb{Z}_4$	• $\boldsymbol{B} \parallel [100]$ and strain in generic direction<br>• $\boldsymbol{B} \parallel [010]$ and strain in generic direction<br>• $\boldsymbol{B} \parallel [001]$ and strain in generic direction<br>• $\boldsymbol{B} \perp [100]$ and strain $\perp [010]$<br>• $\boldsymbol{B} \perp [100]$ and strain $\perp [001]$<br>• $\boldsymbol{B} \perp [100]$ and strain in generic direction<br>• $\boldsymbol{B} \perp [010]$ and strain $\perp [100]$<br>• $\boldsymbol{B} \perp [010]$ and strain $\perp [001]$<br>• $\boldsymbol{B} \perp [010]$ and strain in generic direction<br>• $\boldsymbol{B} \perp [001]$ and strain $\perp [100]$<br>• $\boldsymbol{B} \perp [001]$ and strain $\perp [010]$<br>• $\boldsymbol{B} \perp [001]$ and strain in generic direction<br>• $\boldsymbol{B}$ in generic direction
$P2/c$ (13.65) $\mathbb{Z}_2 \times \mathbb{Z}_2$	• $\boldsymbol{B} \parallel [100]$ and strain $\perp [100]$
$P2_1'/c'$ (14.79) $\mathbb{Z}_2 \times \mathbb{Z}_4$	• $\boldsymbol{B} \parallel [100]$ and strain $\perp [010]$<br>• $\boldsymbol{B} \parallel [001]$ and strain $\perp [010]$<br>• $\boldsymbol{B} \perp [010]$
$P2_1'/m'$ (11.54) $\mathbb{Z}_2 \times \mathbb{Z}_2 \times \mathbb{Z}_4$	• $\boldsymbol{B} \parallel [100]$ and strain $\perp [001]$<br>• $\boldsymbol{B} \parallel [010]$ and strain $\perp [001]$<br>• $\boldsymbol{B} \perp [001]$
$Pbc'm'$ (57.383) $\mathbb{Z}_2 \times \mathbb{Z}_2$	• $\boldsymbol{B} \parallel [100]$
Pc' (7.26) $\mathbb{Z}_1$	• $\boldsymbol{E} \parallel [010]$ and $\boldsymbol{B} \parallel [010]$ and strain $\perp [100]$<br>• $\boldsymbol{E} \parallel [010]$ and $\boldsymbol{B} \parallel [001]$ and strain $\perp [100]$<br>• $\boldsymbol{E} \parallel [010]$ and $\boldsymbol{B} \perp [100]$<br>• $\boldsymbol{E} \parallel [001]$ and $\boldsymbol{B} \parallel [010]$ and strain $\perp [100]$<br>• $\boldsymbol{E} \parallel [001]$ and $\boldsymbol{B} \parallel [001]$ and strain $\perp [100]$<br>• $\boldsymbol{E} \parallel [001]$ and $\boldsymbol{B} \perp [100]$<br>• $\boldsymbol{E} \perp [100]$ and $\boldsymbol{B} \parallel [010]$<br>• $\boldsymbol{E} \perp [100]$ and $\boldsymbol{B} \parallel [001]$<br>• $\boldsymbol{E} \perp [100]$ and $\boldsymbol{B} \perp [100]$
P_Cc (7.30) $\mathbb{Z}_1$	• $\boldsymbol{E} \parallel [010]$ and strain $\perp [100]$<br>• $\boldsymbol{E} \parallel [001]$ and strain $\perp [100]$<br>• $\boldsymbol{E} \perp [100]$
$Pna'2_1'$ (33.147) $\mathbb{Z}_1$	• $\boldsymbol{E} \parallel [010]$ and $\boldsymbol{B} \parallel [001]$
$Pc'a2_1'$ (29.101) $\mathbb{Z}_1$	• $\boldsymbol{E} \parallel [001]$ and $\boldsymbol{B} \parallel [010]$

Continued on next page

...continued

Subgroup & SI group	Perturbation
$P2_1'/c'$ (14.79) $\mathbb{Z}_2 \times \mathbb{Z}_4$	• $\boldsymbol{B} \parallel [010]$ and strain $\perp [100]$<br>• $\boldsymbol{B} \parallel [001]$ and strain $\perp [100]$<br>• $\boldsymbol{B} \perp [100]$
$P2$ (3.1) $\mathbb{Z}_2$	• $\boldsymbol{E} \parallel [010]$ and $\boldsymbol{B} \parallel [010]$ and strain $\perp [010]$
P_C2 (3.6) $\mathbb{Z}_1$	• $\boldsymbol{E} \parallel [010]$ and strain $\perp [010]$
$P2/c$ (13.65) $\mathbb{Z}_2 \times \mathbb{Z}_2$	• $\boldsymbol{B} \parallel [010]$ and strain $\perp [010]$
$Pm'a'2$ (28.91) $\mathbb{Z}_2$	• $\boldsymbol{E} \parallel [010]$ and $\boldsymbol{B} \parallel [010]$
P_Inc2 (30.122) $\mathbb{Z}_1$	• $\boldsymbol{E} \parallel [010]$
$Pbc'm'$ (57.383) $\mathbb{Z}_2 \times \mathbb{Z}_2$	• $\boldsymbol{B} \parallel [010]$
$P2$ (3.1) $\mathbb{Z}_2$	• $\boldsymbol{E} \parallel [001]$ and $\boldsymbol{B} \parallel [001]$ and strain $\perp [001]$
P_C2 (3.6) $\mathbb{Z}_1$	• $\boldsymbol{E} \parallel [001]$ and strain $\perp [001]$
$P2/c$ (13.65) $\mathbb{Z}_2 \times \mathbb{Z}_2$	• $\boldsymbol{B} \parallel [001]$ and strain $\perp [001]$
$Pc'c'2$ (27.81) $\mathbb{Z}_2$	• $\boldsymbol{E} \parallel [001]$ and $\boldsymbol{B} \parallel [001]$
P_Iba2 (32.143) $\mathbb{Z}_1$	• $\boldsymbol{E} \parallel [001]$
$Pc'c'n$ (56.369) $\mathbb{Z}_2 \times \mathbb{Z}_2$	• $\boldsymbol{B} \parallel [001]$
$P_S\bar{1}$ (2.7) $\mathbb{Z}_2 \times \mathbb{Z}_2 \times \mathbb{Z}_4$	• strain in generic direction
P_C2/c (13.74) $\mathbb{Z}_2$	• strain $\perp [100]$
P_C2/c (13.74) $\mathbb{Z}_2$	• strain $\perp [010]$
P_A2/c (13.73) $\mathbb{Z}_2$	• strain $\perp [001]$

Table. S 399: Subgroups of $Pmma$ (51.289) that can be realized by applying $\boldsymbol{E}/\boldsymbol{B}$ fields and/or uniaxial strains.

Subgroup & SI group	Perturbation
Pm (6.18) $\mathbb{Z}_1$	• $\boldsymbol{E} \parallel [100]$ and strain $\perp [010]$<br>• $\boldsymbol{E} \parallel [100]$ and $\boldsymbol{B} \parallel [010]$<br>• $\boldsymbol{E} \parallel [001]$ and strain $\perp [010]$<br>• $\boldsymbol{E} \parallel [001]$ and $\boldsymbol{B} \parallel [010]$<br>• $\boldsymbol{E} \perp [010]$

Continued on next page

…continued

Subgroup & SI group	Perturbation
Pc (7.24) $\mathbb{Z}_1$	• $\boldsymbol{E} \parallel [100]$ and strain $\perp [001]$<br>• $\boldsymbol{E} \parallel [100]$ and $\boldsymbol{B} \parallel [001]$<br>• $\boldsymbol{E} \parallel [010]$ and strain $\perp [001]$<br>• $\boldsymbol{E} \parallel [010]$ and $\boldsymbol{B} \parallel [001]$<br>• $\boldsymbol{E} \perp [001]$
$P2_1$ (4.7) $\mathbb{Z}_1$	• $\boldsymbol{E} \parallel [100]$ and strain $\perp [100]$<br>• $\boldsymbol{E} \parallel [100]$ and $\boldsymbol{B} \parallel [100]$
$Pmc2_1$ (26.66) $\mathbb{Z}_1$	• $\boldsymbol{E} \parallel [100]$
$P2$ (3.1) $\mathbb{Z}_2$	• $\boldsymbol{E} \parallel [010]$ and strain $\perp [010]$<br>• $\boldsymbol{E} \parallel [010]$ and $\boldsymbol{B} \parallel [010]$
$P\bar{1}$ (2.4) $\mathbb{Z}_2 \times \mathbb{Z}_2 \times \mathbb{Z}_2 \times \mathbb{Z}_4$	• strain in generic direction<br>• $\boldsymbol{B} \parallel [100]$ and strain $\perp [010]$<br>• $\boldsymbol{B} \parallel [100]$ and strain $\perp [001]$<br>• $\boldsymbol{B} \parallel [010]$ and strain $\perp [100]$<br>• $\boldsymbol{B} \parallel [010]$ and strain $\perp [001]$<br>• $\boldsymbol{B} \parallel [001]$ and strain $\perp [100]$<br>• $\boldsymbol{B} \parallel [001]$ and strain $\perp [010]$<br>• $\boldsymbol{B}$ in generic direction
$P2/m$ (10.42) $\mathbb{Z}_2 \times \mathbb{Z}_2 \times \mathbb{Z}_2$	• strain $\perp [010]$<br>• $\boldsymbol{B} \parallel [010]$
Pm (6.18) $\mathbb{Z}_1$	• $\boldsymbol{E} \parallel [010]$ and strain $\perp [100]$<br>• $\boldsymbol{E} \parallel [010]$ and $\boldsymbol{B} \parallel [100]$<br>• $\boldsymbol{E} \parallel [001]$ and strain $\perp [100]$<br>• $\boldsymbol{E} \parallel [001]$ and $\boldsymbol{B} \parallel [100]$<br>• $\boldsymbol{E} \perp [100]$
$Pma2$ (28.87) $\mathbb{Z}_1$	• $\boldsymbol{E} \parallel [010]$
$P2_1/m$ (11.50) $\mathbb{Z}_2$	• strain $\perp [100]$<br>• $\boldsymbol{B} \parallel [100]$
$P2$ (3.1) $\mathbb{Z}_2$	• $\boldsymbol{E} \parallel [001]$ and strain $\perp [001]$<br>• $\boldsymbol{E} \parallel [001]$ and $\boldsymbol{B} \parallel [001]$
$P2/c$ (13.65) $\mathbb{Z}_2 \times \mathbb{Z}_2$	• strain $\perp [001]$<br>• $\boldsymbol{B} \parallel [001]$
$Pmm2$ (25.57) $\mathbb{Z}_1$	• $\boldsymbol{E} \parallel [001]$

Table. S 400: Subgroups of $Pmma1'$ (51.290) that can be realized by applying $\boldsymbol{E}/\boldsymbol{B}$ fields and/or uniaxial strains.

Subgroup & SI group	Perturbation
$P11'$ (1.2) $\mathbb{Z}_1$	• $\boldsymbol{E} \parallel [100]$ and strain in generic direction<br>• $\boldsymbol{E} \parallel [010]$ and strain in generic direction<br>• $\boldsymbol{E} \parallel [001]$ and strain in generic direction<br>• $\boldsymbol{E} \perp [100]$ and strain $\perp [010]$<br>• $\boldsymbol{E} \perp [100]$ and strain $\perp [001]$<br>• $\boldsymbol{E} \perp [100]$ and strain in generic direction<br>• $\boldsymbol{E} \perp [010]$ and strain $\perp [100]$<br>• $\boldsymbol{E} \perp [010]$ and strain $\perp [001]$<br>• $\boldsymbol{E} \perp [010]$ and strain in generic direction<br>• $\boldsymbol{E} \perp [001]$ and strain $\perp [100]$<br>• $\boldsymbol{E} \perp [001]$ and strain $\perp [010]$<br>• $\boldsymbol{E} \perp [001]$ and strain in generic direction<br>• $\boldsymbol{E}$ in generic direction
Pm' (6.20) $\mathbb{Z}_1$	• $\boldsymbol{E} \parallel [100]$ and $\boldsymbol{B} \parallel [100]$ and strain $\perp [010]$<br>• $\boldsymbol{E} \parallel [100]$ and $\boldsymbol{B} \parallel [001]$ and strain $\perp [010]$<br>• $\boldsymbol{E} \parallel [100]$ and $\boldsymbol{B} \perp [010]$<br>• $\boldsymbol{E} \parallel [001]$ and $\boldsymbol{B} \parallel [100]$ and strain $\perp [010]$<br>• $\boldsymbol{E} \parallel [001]$ and $\boldsymbol{B} \parallel [001]$ and strain $\perp [010]$<br>• $\boldsymbol{E} \parallel [001]$ and $\boldsymbol{B} \perp [010]$<br>• $\boldsymbol{E} \perp [010]$ and $\boldsymbol{B} \parallel [100]$<br>• $\boldsymbol{E} \perp [010]$ and $\boldsymbol{B} \parallel [001]$<br>• $\boldsymbol{E} \perp [010]$ and $\boldsymbol{B} \perp [010]$
Pm (6.18) $\mathbb{Z}_1$	• $\boldsymbol{E} \parallel [100]$ and $\boldsymbol{B} \parallel [010]$ and strain $\perp [010]$<br>• $\boldsymbol{E} \parallel [001]$ and $\boldsymbol{B} \parallel [010]$ and strain $\perp [010]$<br>• $\boldsymbol{E} \perp [010]$ and $\boldsymbol{B} \parallel [010]$
$Pm1'$ (6.19) $\mathbb{Z}_1$	• $\boldsymbol{E} \parallel [100]$ and strain $\perp [010]$<br>• $\boldsymbol{E} \parallel [001]$ and strain $\perp [010]$<br>• $\boldsymbol{E} \perp [010]$
Pc' (7.26) $\mathbb{Z}_1$	• $\boldsymbol{E} \parallel [100]$ and $\boldsymbol{B} \parallel [100]$ and strain $\perp [001]$<br>• $\boldsymbol{E} \parallel [100]$ and $\boldsymbol{B} \parallel [010]$ and strain $\perp [001]$<br>• $\boldsymbol{E} \parallel [100]$ and $\boldsymbol{B} \perp [001]$<br>• $\boldsymbol{E} \parallel [010]$ and $\boldsymbol{B} \parallel [100]$ and strain $\perp [001]$<br>• $\boldsymbol{E} \parallel [010]$ and $\boldsymbol{B} \parallel [010]$ and strain $\perp [001]$<br>• $\boldsymbol{E} \parallel [010]$ and $\boldsymbol{B} \perp [001]$<br>• $\boldsymbol{E} \perp [001]$ and $\boldsymbol{B} \parallel [100]$<br>• $\boldsymbol{E} \perp [001]$ and $\boldsymbol{B} \parallel [010]$<br>• $\boldsymbol{E} \perp [001]$ and $\boldsymbol{B} \perp [001]$

Continued on next page

...continued

Subgroup & SI group	Perturbation
Pc (7.24) $\mathbb{Z}_1$	• $\boldsymbol{E} \parallel [100]$ and $\boldsymbol{B} \parallel [001]$ and strain $\perp [001]$<br>• $\boldsymbol{E} \parallel [010]$ and $\boldsymbol{B} \parallel [001]$ and strain $\perp [001]$<br>• $\boldsymbol{E} \perp [001]$ and $\boldsymbol{B} \parallel [001]$
$Pc1'$ (7.25) $\mathbb{Z}_1$	• $\boldsymbol{E} \parallel [100]$ and strain $\perp [001]$<br>• $\boldsymbol{E} \parallel [010]$ and strain $\perp [001]$<br>• $\boldsymbol{E} \perp [001]$
$P2_1'$ (4.9) $\mathbb{Z}_1$	• $\boldsymbol{E} \parallel [100]$ and $\boldsymbol{B} \parallel [010]$ and strain $\perp [100]$<br>• $\boldsymbol{E} \parallel [100]$ and $\boldsymbol{B} \parallel [001]$ and strain $\perp [100]$<br>• $\boldsymbol{E} \parallel [100]$ and $\boldsymbol{B} \perp [100]$
$Pmc'2_1'$ (26.69) $\mathbb{Z}_1$	• $\boldsymbol{E} \parallel [100]$ and $\boldsymbol{B} \parallel [010]$
$Pm'c2_1'$ (26.68) $\mathbb{Z}_1$	• $\boldsymbol{E} \parallel [100]$ and $\boldsymbol{B} \parallel [001]$
$P2_1$ (4.7) $\mathbb{Z}_1$	• $\boldsymbol{E} \parallel [100]$ and $\boldsymbol{B} \parallel [100]$ and strain $\perp [100]$
$Pm'c'2_1$ (26.70) $\mathbb{Z}_1$	• $\boldsymbol{E} \parallel [100]$ and $\boldsymbol{B} \parallel [100]$
$P2_11'$ (4.8) $\mathbb{Z}_1$	• $\boldsymbol{E} \parallel [100]$ and strain $\perp [100]$
$Pmc2_11'$ (26.67) $\mathbb{Z}_1$	• $\boldsymbol{E} \parallel [100]$
$P2'$ (3.3) $\mathbb{Z}_1$	• $\boldsymbol{E} \parallel [010]$ and $\boldsymbol{B} \parallel [100]$ and strain $\perp [010]$<br>• $\boldsymbol{E} \parallel [010]$ and $\boldsymbol{B} \parallel [001]$ and strain $\perp [010]$<br>• $\boldsymbol{E} \parallel [010]$ and $\boldsymbol{B} \perp [010]$
$P2$ (3.1) $\mathbb{Z}_2$	• $\boldsymbol{E} \parallel [010]$ and $\boldsymbol{B} \parallel [010]$ and strain $\perp [010]$
$P21'$ (3.2) $\mathbb{Z}_2$	• $\boldsymbol{E} \parallel [010]$ and strain $\perp [010]$
$P\bar{1}$ (2.4) $\mathbb{Z}_2 \times \mathbb{Z}_2 \times \mathbb{Z}_2 \times \mathbb{Z}_4$	• $\boldsymbol{B} \parallel [100]$ and strain in generic direction<br>• $\boldsymbol{B} \parallel [010]$ and strain in generic direction<br>• $\boldsymbol{B} \parallel [001]$ and strain in generic direction<br>• $\boldsymbol{B} \perp [100]$ and strain $\perp [010]$<br>• $\boldsymbol{B} \perp [100]$ and strain $\perp [001]$<br>• $\boldsymbol{B} \perp [100]$ and strain in generic direction<br>• $\boldsymbol{B} \perp [010]$ and strain $\perp [100]$<br>• $\boldsymbol{B} \perp [010]$ and strain $\perp [001]$<br>• $\boldsymbol{B} \perp [010]$ and strain in generic direction<br>• $\boldsymbol{B} \perp [001]$ and strain $\perp [100]$<br>• $\boldsymbol{B} \perp [001]$ and strain $\perp [010]$<br>• $\boldsymbol{B} \perp [001]$ and strain in generic direction<br>• $\boldsymbol{B}$ in generic direction

Continued on next page

…continued

Subgroup & SI group	Perturbation
$P2'/m'$ (10.46) $\mathbb{Z}_2 \times \mathbb{Z}_2 \times \mathbb{Z}_2 \times \mathbb{Z}_4$	• $\boldsymbol{B} \parallel [100]$ and strain $\perp [010]$<br>• $\boldsymbol{B} \parallel [001]$ and strain $\perp [010]$<br>• $\boldsymbol{B} \perp [010]$
$P2/m$ (10.42) $\mathbb{Z}_2 \times \mathbb{Z}_2 \times \mathbb{Z}_2$	• $\boldsymbol{B} \parallel [010]$ and strain $\perp [010]$
$P\bar{1}1'$ (2.5) $\mathbb{Z}_2 \times \mathbb{Z}_2 \times \mathbb{Z}_2 \times \mathbb{Z}_4$	• strain in generic direction
$P2/m1'$ (10.43) $\mathbb{Z}_2 \times \mathbb{Z}_2 \times \mathbb{Z}_2$	• strain $\perp [010]$
Pm' (6.20) $\mathbb{Z}_1$	• $\boldsymbol{E} \parallel [010]$ and $\boldsymbol{B} \parallel [010]$ and strain $\perp [100]$<br>• $\boldsymbol{E} \parallel [010]$ and $\boldsymbol{B} \parallel [001]$ and strain $\perp [100]$<br>• $\boldsymbol{E} \parallel [010]$ and $\boldsymbol{B} \perp [100]$<br>• $\boldsymbol{E} \parallel [001]$ and $\boldsymbol{B} \parallel [010]$ and strain $\perp [100]$<br>• $\boldsymbol{E} \parallel [001]$ and $\boldsymbol{B} \parallel [001]$ and strain $\perp [100]$<br>• $\boldsymbol{E} \parallel [001]$ and $\boldsymbol{B} \perp [100]$<br>• $\boldsymbol{E} \perp [100]$ and $\boldsymbol{B} \parallel [010]$<br>• $\boldsymbol{E} \perp [100]$ and $\boldsymbol{B} \parallel [001]$<br>• $\boldsymbol{E} \perp [100]$ and $\boldsymbol{B} \perp [100]$
$Pm'a2'$ (28.89) $\mathbb{Z}_1$	• $\boldsymbol{E} \parallel [010]$ and $\boldsymbol{B} \parallel [001]$
$Pm'a'2$ (28.91) $\mathbb{Z}_2$	• $\boldsymbol{E} \parallel [010]$ and $\boldsymbol{B} \parallel [010]$
$P2_1'/m'$ (11.54) $\mathbb{Z}_2 \times \mathbb{Z}_2 \times \mathbb{Z}_4$	• $\boldsymbol{B} \parallel [010]$ and strain $\perp [100]$<br>• $\boldsymbol{B} \parallel [001]$ and strain $\perp [100]$<br>• $\boldsymbol{B} \perp [100]$
Pm (6.18) $\mathbb{Z}_1$	• $\boldsymbol{E} \parallel [010]$ and $\boldsymbol{B} \parallel [100]$ and strain $\perp [100]$<br>• $\boldsymbol{E} \parallel [001]$ and $\boldsymbol{B} \parallel [100]$ and strain $\perp [100]$<br>• $\boldsymbol{E} \perp [100]$ and $\boldsymbol{B} \parallel [100]$
$Pma'2'$ (28.90) $\mathbb{Z}_1$	• $\boldsymbol{E} \parallel [010]$ and $\boldsymbol{B} \parallel [100]$
$P2_1/m$ (11.50) $\mathbb{Z}_2$	• $\boldsymbol{B} \parallel [100]$ and strain $\perp [100]$
$Pm1'$ (6.19) $\mathbb{Z}_1$	• $\boldsymbol{E} \parallel [010]$ and strain $\perp [100]$<br>• $\boldsymbol{E} \parallel [001]$ and strain $\perp [100]$<br>• $\boldsymbol{E} \perp [100]$
$Pma21'$ (28.88) $\mathbb{Z}_1$	• $\boldsymbol{E} \parallel [010]$
$P2_1/m1'$ (11.51) $\mathbb{Z}_2$	• strain $\perp [100]$
$P2'$ (3.3) $\mathbb{Z}_1$	• $\boldsymbol{E} \parallel [001]$ and $\boldsymbol{B} \parallel [100]$ and strain $\perp [001]$<br>• $\boldsymbol{E} \parallel [001]$ and $\boldsymbol{B} \parallel [010]$ and strain $\perp [001]$<br>• $\boldsymbol{E} \parallel [001]$ and $\boldsymbol{B} \perp [001]$

Continued on next page

…continued

Subgroup & SI group	Perturbation
$P2'/c'$ (13.69) $\mathbb{Z}_2 \times \mathbb{Z}_2 \times \mathbb{Z}_4$	• $\boldsymbol{B} \parallel [100]$ and strain $\perp [001]$<br>• $\boldsymbol{B} \parallel [010]$ and strain $\perp [001]$<br>• $\boldsymbol{B} \perp [001]$
$Pm'm2'$ (25.59) $\mathbb{Z}_1$	• $\boldsymbol{E} \parallel [001]$ and $\boldsymbol{B} \parallel [010]$
$Pm'ma'$ (51.296) $\mathbb{Z}_2 \times \mathbb{Z}_2 \times \mathbb{Z}_2$	• $\boldsymbol{B} \parallel [010]$
$Pm'm2'$ (25.59) $\mathbb{Z}_1$	• $\boldsymbol{E} \parallel [001]$ and $\boldsymbol{B} \parallel [100]$
$Pmm'a'$ (51.295) $\mathbb{Z}_2$	• $\boldsymbol{B} \parallel [100]$
$P2$ (3.1) $\mathbb{Z}_2$	• $\boldsymbol{E} \parallel [001]$ and $\boldsymbol{B} \parallel [001]$ and strain $\perp [001]$
$P2/c$ (13.65) $\mathbb{Z}_2 \times \mathbb{Z}_2$	• $\boldsymbol{B} \parallel [001]$ and strain $\perp [001]$
$Pm'm'2$ (25.60) $\mathbb{Z}_2$	• $\boldsymbol{E} \parallel [001]$ and $\boldsymbol{B} \parallel [001]$
$Pm'm'a$ (51.294) $\mathbb{Z}_2 \times \mathbb{Z}_2$	• $\boldsymbol{B} \parallel [001]$
$P21'$ (3.2) $\mathbb{Z}_2$	• $\boldsymbol{E} \parallel [001]$ and strain $\perp [001]$
$P2/c1'$ (13.66) $\mathbb{Z}_2 \times \mathbb{Z}_2$	• strain $\perp [001]$
$Pmm21'$ (25.58) $\mathbb{Z}_1$	• $\boldsymbol{E} \parallel [001]$

Table. S 401: Subgroups of $Pm'ma$ (51.291) that can be realized by applying $\boldsymbol{E}/\boldsymbol{B}$ fields and/or uniaxial strains.

Subgroup & SI group	Perturbation
Pm (6.18) $\mathbb{Z}_1$	• $\boldsymbol{B} \parallel [010]$ and strain $\perp [010]$<br>• $\boldsymbol{E} \parallel [100]$ and strain $\perp [010]$<br>• $\boldsymbol{E} \parallel [100]$ and $\boldsymbol{B} \parallel [010]$<br>• $\boldsymbol{E} \parallel [001]$ and strain $\perp [010]$<br>• $\boldsymbol{E} \perp [010]$
Pc (7.24) $\mathbb{Z}_1$	• $\boldsymbol{B} \parallel [001]$ and strain $\perp [001]$<br>• $\boldsymbol{E} \parallel [100]$ and strain $\perp [001]$<br>• $\boldsymbol{E} \parallel [100]$ and $\boldsymbol{B} \parallel [001]$<br>• $\boldsymbol{E} \parallel [010]$ and strain $\perp [001]$<br>• $\boldsymbol{E} \perp [001]$
$P2_1$ (4.7) $\mathbb{Z}_1$	• $\boldsymbol{B} \parallel [100]$ and strain $\perp [100]$<br>• $\boldsymbol{E} \parallel [100]$ and strain $\perp [100]$<br>• $\boldsymbol{E} \parallel [100]$ and $\boldsymbol{B} \parallel [100]$

Continued on next page

…continued

Subgroup & SI group	Perturbation
$Pmc2_1$ (26.66) $\mathbb{Z}_1$	• $\boldsymbol{E}$ ∥ [100]
$P2'$ (3.3) $\mathbb{Z}_1$	• $\boldsymbol{B}$ ∥ [100] and strain ⊥ [010]<br>• $\boldsymbol{B}$ ∥ [001] and strain ⊥ [010]<br>• $\boldsymbol{B}$ ⊥ [010]<br>• $\boldsymbol{E}$ ∥ [010] and strain ⊥ [010]<br>• $\boldsymbol{E}$ ∥ [010] and $\boldsymbol{B}$ ∥ [100]
$P\bar{1}'$ (2.6) $\mathbb{Z}_1$	• strain in generic direction
$P2'/m$ (10.44) $\mathbb{Z}_1$	• strain ⊥ [010]
Pm' (6.20) $\mathbb{Z}_1$	• $\boldsymbol{B}$ ∥ [010] and strain ⊥ [100]<br>• $\boldsymbol{B}$ ∥ [001] and strain ⊥ [100]<br>• $\boldsymbol{B}$ ⊥ [100]<br>• $\boldsymbol{E}$ ∥ [010] and strain ⊥ [100]<br>• $\boldsymbol{E}$ ∥ [010] and $\boldsymbol{B}$ ∥ [010]<br>• $\boldsymbol{E}$ ∥ [001] and strain ⊥ [100]<br>• $\boldsymbol{E}$ ∥ [001] and $\boldsymbol{B}$ ∥ [001]<br>• $\boldsymbol{E}$ ⊥ [100]
$Pm'a2'$ (28.89) $\mathbb{Z}_1$	• $\boldsymbol{B}$ ∥ [001]<br>• $\boldsymbol{E}$ ∥ [010]
$P2_1/m'$ (11.53) $\mathbb{Z}_1$	• strain ⊥ [100]
$P2'$ (3.3) $\mathbb{Z}_1$	• $\boldsymbol{B}$ ∥ [100] and strain ⊥ [001]<br>• $\boldsymbol{B}$ ∥ [010] and strain ⊥ [001]<br>• $\boldsymbol{B}$ ⊥ [001]<br>• $\boldsymbol{E}$ ∥ [001] and strain ⊥ [001]<br>• $\boldsymbol{E}$ ∥ [001] and $\boldsymbol{B}$ ∥ [100]
$P2'2'2_1$ (17.9) $\mathbb{Z}_1$	• $\boldsymbol{B}$ ∥ [100]
$P2'/c$ (13.67) $\mathbb{Z}_1$	• strain ⊥ [001]
$Pm'm2'$ (25.59) $\mathbb{Z}_1$	• $\boldsymbol{B}$ ∥ [010]<br>• $\boldsymbol{E}$ ∥ [001]

Table. S 402: Subgroups of $Pmm'a$ (51.292) that can be realized by applying $\boldsymbol{E}/\boldsymbol{B}$ fields and/or uniaxial strains.

Subgroup & SI group	Perturbation

Continued on next page

...continued

Subgroup & SI group	Perturbation
Pm' (6.20) $\mathbb{Z}_1$	• $\boldsymbol{B} \parallel [100]$ and strain $\perp [010]$<br>• $\boldsymbol{B} \parallel [001]$ and strain $\perp [010]$<br>• $\boldsymbol{B} \perp [010]$<br>• $\boldsymbol{E} \parallel [100]$ and strain $\perp [010]$<br>• $\boldsymbol{E} \parallel [100]$ and $\boldsymbol{B} \parallel [100]$<br>• $\boldsymbol{E} \parallel [001]$ and strain $\perp [010]$<br>• $\boldsymbol{E} \parallel [001]$ and $\boldsymbol{B} \parallel [001]$<br>• $\boldsymbol{E} \perp [010]$
Pc (7.24) $\mathbb{Z}_1$	• $\boldsymbol{B} \parallel [001]$ and strain $\perp [001]$<br>• $\boldsymbol{E} \parallel [100]$ and strain $\perp [001]$<br>• $\boldsymbol{E} \parallel [010]$ and strain $\perp [001]$<br>• $\boldsymbol{E} \parallel [010]$ and $\boldsymbol{B} \parallel [001]$<br>• $\boldsymbol{E} \perp [001]$
$P2_1'$ (4.9) $\mathbb{Z}_1$	• $\boldsymbol{B} \parallel [010]$ and strain $\perp [100]$<br>• $\boldsymbol{B} \parallel [001]$ and strain $\perp [100]$<br>• $\boldsymbol{B} \perp [100]$<br>• $\boldsymbol{E} \parallel [100]$ and strain $\perp [100]$<br>• $\boldsymbol{E} \parallel [100]$ and $\boldsymbol{B} \parallel [010]$
$Pm'c2_1'$ (26.68) $\mathbb{Z}_1$	• $\boldsymbol{B} \parallel [001]$<br>• $\boldsymbol{E} \parallel [100]$
$P2$ (3.1) $\mathbb{Z}_2$	• $\boldsymbol{B} \parallel [010]$ and strain $\perp [010]$<br>• $\boldsymbol{E} \parallel [010]$ and strain $\perp [010]$<br>• $\boldsymbol{E} \parallel [010]$ and $\boldsymbol{B} \parallel [010]$
$P\bar{1}'$ (2.6) $\mathbb{Z}_1$	• strain in generic direction
$P2/m'$ (10.45) $\mathbb{Z}_2$	• strain $\perp [010]$
Pm (6.18) $\mathbb{Z}_1$	• $\boldsymbol{B} \parallel [100]$ and strain $\perp [100]$<br>• $\boldsymbol{E} \parallel [010]$ and strain $\perp [100]$<br>• $\boldsymbol{E} \parallel [010]$ and $\boldsymbol{B} \parallel [100]$<br>• $\boldsymbol{E} \parallel [001]$ and strain $\perp [100]$<br>• $\boldsymbol{E} \perp [100]$
$Pma2$ (28.87) $\mathbb{Z}_1$	• $\boldsymbol{E} \parallel [010]$
$P2_1'/m$ (11.52) $\mathbb{Z}_1$	• strain $\perp [100]$

Continued on next page

...continued

Subgroup & SI group	Perturbation
$P2'$ (3.3) $\mathbb{Z}_1$	• $\boldsymbol{B} \parallel [100]$ and strain $\perp [001]$<br>• $\boldsymbol{B} \parallel [010]$ and strain $\perp [001]$<br>• $\boldsymbol{B} \perp [001]$<br>• $\boldsymbol{E} \parallel [001]$ and strain $\perp [001]$<br>• $\boldsymbol{E} \parallel [001]$ and $\boldsymbol{B} \parallel [010]$
$P22'2_1'$ (17.10) $\mathbb{Z}_2$	• $\boldsymbol{B} \parallel [010]$
$P2'/c$ (13.67) $\mathbb{Z}_1$	• strain $\perp [001]$
$Pm'm2'$ (25.59) $\mathbb{Z}_1$	• $\boldsymbol{B} \parallel [100]$<br>• $\boldsymbol{E} \parallel [001]$

Table. S 403: Subgroups of $Pmma'$ (51.293) that can be realized by applying $\boldsymbol{E}/\boldsymbol{B}$ fields and/or uniaxial strains.

Subgroup & SI group	Perturbation
Pm (6.18) $\mathbb{Z}_1$	• $\boldsymbol{B} \parallel [010]$ and strain $\perp [010]$<br>• $\boldsymbol{E} \parallel [100]$ and strain $\perp [010]$<br>• $\boldsymbol{E} \parallel [001]$ and strain $\perp [010]$<br>• $\boldsymbol{E} \parallel [001]$ and $\boldsymbol{B} \parallel [010]$<br>• $\boldsymbol{E} \perp [010]$
Pc' (7.26) $\mathbb{Z}_1$	• $\boldsymbol{B} \parallel [100]$ and strain $\perp [001]$<br>• $\boldsymbol{B} \parallel [010]$ and strain $\perp [001]$<br>• $\boldsymbol{B} \perp [001]$<br>• $\boldsymbol{E} \parallel [100]$ and strain $\perp [001]$<br>• $\boldsymbol{E} \parallel [100]$ and $\boldsymbol{B} \parallel [100]$<br>• $\boldsymbol{E} \parallel [010]$ and strain $\perp [001]$<br>• $\boldsymbol{E} \parallel [010]$ and $\boldsymbol{B} \parallel [010]$<br>• $\boldsymbol{E} \perp [001]$
$P2_1'$ (4.9) $\mathbb{Z}_1$	• $\boldsymbol{B} \parallel [010]$ and strain $\perp [100]$<br>• $\boldsymbol{B} \parallel [001]$ and strain $\perp [100]$<br>• $\boldsymbol{B} \perp [100]$<br>• $\boldsymbol{E} \parallel [100]$ and strain $\perp [100]$<br>• $\boldsymbol{E} \parallel [100]$ and $\boldsymbol{B} \parallel [001]$
$Pmc'2_1'$ (26.69) $\mathbb{Z}_1$	• $\boldsymbol{B} \parallel [010]$<br>• $\boldsymbol{E} \parallel [100]$

Continued on next page

...continued

Subgroup & SI group	Perturbation
$P2'$ (3.3) $\mathbb{Z}_1$	• $\boldsymbol{B} \parallel [100]$ and strain $\perp [010]$<br>• $\boldsymbol{B} \parallel [001]$ and strain $\perp [010]$<br>• $\boldsymbol{B} \perp [010]$<br>• $\boldsymbol{E} \parallel [010]$ and strain $\perp [010]$<br>• $\boldsymbol{E} \parallel [010]$ and $\boldsymbol{B} \parallel [001]$
$P\bar{1}'$ (2.6) $\mathbb{Z}_1$	• strain in generic direction
$P2'/m$ (10.44) $\mathbb{Z}_1$	• strain $\perp [010]$
Pm (6.18) $\mathbb{Z}_1$	• $\boldsymbol{B} \parallel [100]$ and strain $\perp [100]$<br>• $\boldsymbol{E} \parallel [010]$ and strain $\perp [100]$<br>• $\boldsymbol{E} \parallel [001]$ and strain $\perp [100]$<br>• $\boldsymbol{E} \parallel [001]$ and $\boldsymbol{B} \parallel [100]$<br>• $\boldsymbol{E} \perp [100]$
$Pma'2'$ (28.90) $\mathbb{Z}_1$	• $\boldsymbol{B} \parallel [100]$<br>• $\boldsymbol{E} \parallel [010]$
$P2_1'/m$ (11.52) $\mathbb{Z}_1$	• strain $\perp [100]$
$P2$ (3.1) $\mathbb{Z}_2$	• $\boldsymbol{B} \parallel [001]$ and strain $\perp [001]$<br>• $\boldsymbol{E} \parallel [001]$ and strain $\perp [001]$<br>• $\boldsymbol{E} \parallel [001]$ and $\boldsymbol{B} \parallel [001]$
$P22'2_1'$ (17.10) $\mathbb{Z}_2$	• $\boldsymbol{B} \parallel [001]$
$P2/c'$ (13.68) $\mathbb{Z}_2$	• strain $\perp [001]$
$Pmm2$ (25.57) $\mathbb{Z}_1$	• $\boldsymbol{E} \parallel [001]$

Table. S 404: Subgroups of $Pm'm'a$ (51.294) that can be realized by applying $\boldsymbol{E}/\boldsymbol{B}$ fields and/or uniaxial strains.

Subgroup & SI group	Perturbation
Pm' (6.20) $\mathbb{Z}_1$	• $\boldsymbol{E} \parallel [100]$ and strain $\perp [010]$<br>• $\boldsymbol{E} \parallel [100]$ and ($\boldsymbol{B} \parallel [100]$ or $\boldsymbol{B} \perp [010]$)<br>• $\boldsymbol{E} \parallel [001]$ and strain $\perp [010]$<br>• $\boldsymbol{E} \parallel [001]$ and ($\boldsymbol{B} \parallel [100]$ or $\boldsymbol{B} \perp [010]$)<br>• $\boldsymbol{E} \perp [010]$

Continued on next page

...continued

Subgroup & SI group	Perturbation
Pc (7.24) $\mathbb{Z}_1$	• $\boldsymbol{E}$ ‖ [100] and strain ⊥ [001]<br>• $\boldsymbol{E}$ ‖ [010] and strain ⊥ [001]<br>• $\boldsymbol{E}$ ⊥ [001]
$P2_1'$ (4.9) $\mathbb{Z}_1$	• $\boldsymbol{E}$ ‖ [100] and strain ⊥ [100]<br>• $\boldsymbol{E}$ ‖ [100] and ($\boldsymbol{B}$ ‖ [010] or $\boldsymbol{B}$ ⊥ [100])
$Pm'c2_1'$ (26.68) $\mathbb{Z}_1$	• $\boldsymbol{E}$ ‖ [100]
$P2'$ (3.3) $\mathbb{Z}_1$	• $\boldsymbol{E}$ ‖ [010] and strain ⊥ [010]<br>• $\boldsymbol{E}$ ‖ [010] and ($\boldsymbol{B}$ ‖ [100] or $\boldsymbol{B}$ ⊥ [010])
$P\bar{1}$ (2.4) $\mathbb{Z}_2 \times \mathbb{Z}_2 \times \mathbb{Z}_2 \times \mathbb{Z}_4$	• strain in generic direction<br>• ($\boldsymbol{B}$ ‖ [100] or $\boldsymbol{B}$ ⊥ [010]) and strain ⊥ [100]<br>• ($\boldsymbol{B}$ ‖ [100] or $\boldsymbol{B}$ ⊥ [010]) and strain ⊥ [001]<br>• ($\boldsymbol{B}$ ‖ [010] or $\boldsymbol{B}$ ⊥ [100]) and strain ⊥ [010]<br>• ($\boldsymbol{B}$ ‖ [010] or $\boldsymbol{B}$ ⊥ [100]) and strain ⊥ [001]<br>• $\boldsymbol{B}$ in generic direction
$P2'/m'$ (10.46) $\mathbb{Z}_2 \times \mathbb{Z}_2 \times \mathbb{Z}_2 \times \mathbb{Z}_4$	• strain ⊥ [010]<br>• ($\boldsymbol{B}$ ‖ [100] or $\boldsymbol{B}$ ⊥ [010])
Pm' (6.20) $\mathbb{Z}_1$	• $\boldsymbol{E}$ ‖ [010] and strain ⊥ [100]<br>• $\boldsymbol{E}$ ‖ [010] and ($\boldsymbol{B}$ ‖ [010] or $\boldsymbol{B}$ ⊥ [100])<br>• $\boldsymbol{E}$ ‖ [001] and strain ⊥ [100]<br>• $\boldsymbol{E}$ ‖ [001] and ($\boldsymbol{B}$ ‖ [010] or $\boldsymbol{B}$ ⊥ [100])<br>• $\boldsymbol{E}$ ⊥ [100]
$Pm'a2'$ (28.89) $\mathbb{Z}_1$	• $\boldsymbol{E}$ ‖ [010]
$P2_1'/m'$ (11.54) $\mathbb{Z}_2 \times \mathbb{Z}_2 \times \mathbb{Z}_4$	• strain ⊥ [100]<br>• ($\boldsymbol{B}$ ‖ [010] or $\boldsymbol{B}$ ⊥ [100])
$P2$ (3.1) $\mathbb{Z}_2$	• $\boldsymbol{E}$ ‖ [001] and strain ⊥ [001]
$P2/c$ (13.65) $\mathbb{Z}_2 \times \mathbb{Z}_2$	• strain ⊥ [001]
$Pm'm'2$ (25.60) $\mathbb{Z}_2$	• $\boldsymbol{E}$ ‖ [001]

Table. S 405: Subgroups of $Pmm'a'$ (51.295) that can be realized by applying $\boldsymbol{E}/\boldsymbol{B}$ fields and/or uniaxial strains.

Subgroup & SI group	Perturbation

Continued on next page

. . . continued

Subgroup & SI group	Perturbation
Pm' (6.20) $\mathbb{Z}_1$	• $\boldsymbol{E} \parallel [100]$ and strain $\perp [010]$<br>• $\boldsymbol{E} \parallel [100]$ and ($\boldsymbol{B} \parallel [001]$ or $\boldsymbol{B} \perp [010]$)<br>• $\boldsymbol{E} \parallel [001]$ and strain $\perp [010]$<br>• $\boldsymbol{E} \parallel [001]$ and ($\boldsymbol{B} \parallel [001]$ or $\boldsymbol{B} \perp [010]$)<br>• $\boldsymbol{E} \perp [010]$
Pc' (7.26) $\mathbb{Z}_1$	• $\boldsymbol{E} \parallel [100]$ and strain $\perp [001]$<br>• $\boldsymbol{E} \parallel [100]$ and ($\boldsymbol{B} \parallel [010]$ or $\boldsymbol{B} \perp [001]$)<br>• $\boldsymbol{E} \parallel [010]$ and strain $\perp [001]$<br>• $\boldsymbol{E} \parallel [010]$ and ($\boldsymbol{B} \parallel [010]$ or $\boldsymbol{B} \perp [001]$)<br>• $\boldsymbol{E} \perp [001]$
$P2_1$ (4.7) $\mathbb{Z}_1$	• $\boldsymbol{E} \parallel [100]$ and strain $\perp [100]$
$Pm'c'2_1$ (26.70) $\mathbb{Z}_1$	• $\boldsymbol{E} \parallel [100]$
$P2'$ (3.3) $\mathbb{Z}_1$	• $\boldsymbol{E} \parallel [010]$ and strain $\perp [010]$<br>• $\boldsymbol{E} \parallel [010]$ and ($\boldsymbol{B} \parallel [001]$ or $\boldsymbol{B} \perp [010]$)
$P\bar{1}$ (2.4) $\mathbb{Z}_2 \times \mathbb{Z}_2 \times \mathbb{Z}_2 \times \mathbb{Z}_4$	• strain in generic direction<br>• ($\boldsymbol{B} \parallel [010]$ or $\boldsymbol{B} \perp [001]$) and strain $\perp [100]$<br>• ($\boldsymbol{B} \parallel [010]$ or $\boldsymbol{B} \perp [001]$) and strain $\perp [010]$<br>• ($\boldsymbol{B} \parallel [001]$ or $\boldsymbol{B} \perp [010]$) and strain $\perp [100]$<br>• ($\boldsymbol{B} \parallel [001]$ or $\boldsymbol{B} \perp [010]$) and strain $\perp [001]$<br>• $\boldsymbol{B}$ in generic direction
$P2'/m'$ (10.46) $\mathbb{Z}_2 \times \mathbb{Z}_2 \times \mathbb{Z}_2 \times \mathbb{Z}_4$	• strain $\perp [010]$<br>• ($\boldsymbol{B} \parallel [001]$ or $\boldsymbol{B} \perp [010]$)
Pm (6.18) $\mathbb{Z}_1$	• $\boldsymbol{E} \parallel [010]$ and strain $\perp [100]$<br>• $\boldsymbol{E} \parallel [001]$ and strain $\perp [100]$<br>• $\boldsymbol{E} \perp [100]$
$Pma'2'$ (28.90) $\mathbb{Z}_1$	• $\boldsymbol{E} \parallel [010]$
$P2_1/m$ (11.50) $\mathbb{Z}_2$	• strain $\perp [100]$
$P2'$ (3.3) $\mathbb{Z}_1$	• $\boldsymbol{E} \parallel [001]$ and strain $\perp [001]$<br>• $\boldsymbol{E} \parallel [001]$ and ($\boldsymbol{B} \parallel [010]$ or $\boldsymbol{B} \perp [001]$)
$P2'/c'$ (13.69) $\mathbb{Z}_2 \times \mathbb{Z}_2 \times \mathbb{Z}_4$	• strain $\perp [001]$<br>• ($\boldsymbol{B} \parallel [010]$ or $\boldsymbol{B} \perp [001]$)
$Pm'm2'$ (25.59) $\mathbb{Z}_1$	• $\boldsymbol{E} \parallel [001]$

Table. S 406: Subgroups of $Pm'ma'$ (51.296) that can be realized by applying $\boldsymbol{E}/\boldsymbol{B}$ fields and/or uniaxial strains.

Subgroup & SI group	Perturbation
Pm (6.18) $\mathbb{Z}_1$	• $\boldsymbol{E} \parallel$ [100] and strain $\perp$ [010]<br>• $\boldsymbol{E} \parallel$ [001] and strain $\perp$ [010]<br>• $\boldsymbol{E} \perp$ [010]
Pc' (7.26) $\mathbb{Z}_1$	• $\boldsymbol{E} \parallel$ [100] and strain $\perp$ [001]<br>• $\boldsymbol{E} \parallel$ [100] and ($\boldsymbol{B} \parallel$ [100] or $\boldsymbol{B} \perp$ [001])<br>• $\boldsymbol{E} \parallel$ [010] and strain $\perp$ [001]<br>• $\boldsymbol{E} \parallel$ [010] and ($\boldsymbol{B} \parallel$ [100] or $\boldsymbol{B} \perp$ [001])<br>• $\boldsymbol{E} \perp$ [001]
$P2_1'$ (4.9) $\mathbb{Z}_1$	• $\boldsymbol{E} \parallel$ [100] and strain $\perp$ [100]<br>• $\boldsymbol{E} \parallel$ [100] and ($\boldsymbol{B} \parallel$ [001] or $\boldsymbol{B} \perp$ [100])
$Pmc'2_1'$ (26.69) $\mathbb{Z}_1$	• $\boldsymbol{E} \parallel$ [100]
$P2$ (3.1) $\mathbb{Z}_2$	• $\boldsymbol{E} \parallel$ [010] and strain $\perp$ [010]
$P\bar{1}$ (2.4) $\mathbb{Z}_2 \times \mathbb{Z}_2 \times \mathbb{Z}_2 \times \mathbb{Z}_4$	• strain in generic direction<br>• ($\boldsymbol{B} \parallel$ [100] or $\boldsymbol{B} \perp$ [001]) and strain $\perp$ [100]<br>• ($\boldsymbol{B} \parallel$ [100] or $\boldsymbol{B} \perp$ [001]) and strain $\perp$ [010]<br>• ($\boldsymbol{B} \parallel$ [001] or $\boldsymbol{B} \perp$ [100]) and strain $\perp$ [010]<br>• ($\boldsymbol{B} \parallel$ [001] or $\boldsymbol{B} \perp$ [100]) and strain $\perp$ [001]<br>• $\boldsymbol{B}$ in generic direction
$P2/m$ (10.42) $\mathbb{Z}_2 \times \mathbb{Z}_2 \times \mathbb{Z}_2$	• strain $\perp$ [010]
Pm' (6.20) $\mathbb{Z}_1$	• $\boldsymbol{E} \parallel$ [010] and strain $\perp$ [100]<br>• $\boldsymbol{E} \parallel$ [010] and ($\boldsymbol{B} \parallel$ [001] or $\boldsymbol{B} \perp$ [100])<br>• $\boldsymbol{E} \parallel$ [001] and strain $\perp$ [100]<br>• $\boldsymbol{E} \parallel$ [001] and ($\boldsymbol{B} \parallel$ [001] or $\boldsymbol{B} \perp$ [100])<br>• $\boldsymbol{E} \perp$ [100]
$Pm'a'2$ (28.91) $\mathbb{Z}_2$	• $\boldsymbol{E} \parallel$ [010]
$P2_1'/m'$ (11.54) $\mathbb{Z}_2 \times \mathbb{Z}_2 \times \mathbb{Z}_4$	• strain $\perp$ [100]<br>• ($\boldsymbol{B} \parallel$ [001] or $\boldsymbol{B} \perp$ [100])
$P2'$ (3.3) $\mathbb{Z}_1$	• $\boldsymbol{E} \parallel$ [001] and strain $\perp$ [001]<br>• $\boldsymbol{E} \parallel$ [001] and ($\boldsymbol{B} \parallel$ [100] or $\boldsymbol{B} \perp$ [001])
$P2'/c'$ (13.69) $\mathbb{Z}_2 \times \mathbb{Z}_2 \times \mathbb{Z}_4$	• strain $\perp$ [001]<br>• ($\boldsymbol{B} \parallel$ [100] or $\boldsymbol{B} \perp$ [001])
$Pm'm2'$ (25.59) $\mathbb{Z}_1$	• $\boldsymbol{E} \parallel$ [001]

Table. S 407: Subgroups of $Pm'm'a'$ (51.297) that can be realized by applying $\boldsymbol{E}/\boldsymbol{B}$ fields and/or uniaxial strains.

Subgroup & SI group	Perturbation
Pm' (6.20) $\mathbb{Z}_1$	• $\boldsymbol{B}$ ∥ [100] and strain ⊥ [010]<br>• $\boldsymbol{B}$ ∥ [001] and strain ⊥ [010]<br>• $\boldsymbol{B}$ ⊥ [010]<br>• $\boldsymbol{E}$ ∥ [100] and strain ⊥ [010]<br>• $\boldsymbol{E}$ ∥ [100] and $\boldsymbol{B}$ ∥ [001]<br>• $\boldsymbol{E}$ ∥ [001] and strain ⊥ [010]<br>• $\boldsymbol{E}$ ∥ [001] and $\boldsymbol{B}$ ∥ [100]<br>• $\boldsymbol{E}$ ⊥ [010]
Pc' (7.26) $\mathbb{Z}_1$	• $\boldsymbol{B}$ ∥ [100] and strain ⊥ [001]<br>• $\boldsymbol{B}$ ∥ [010] and strain ⊥ [001]<br>• $\boldsymbol{B}$ ⊥ [001]<br>• $\boldsymbol{E}$ ∥ [100] and strain ⊥ [001]<br>• $\boldsymbol{E}$ ∥ [100] and $\boldsymbol{B}$ ∥ [010]<br>• $\boldsymbol{E}$ ∥ [010] and strain ⊥ [001]<br>• $\boldsymbol{E}$ ∥ [010] and $\boldsymbol{B}$ ∥ [100]<br>• $\boldsymbol{E}$ ⊥ [001]
$P2_1$ (4.7) $\mathbb{Z}_1$	• $\boldsymbol{B}$ ∥ [100] and strain ⊥ [100]<br>• $\boldsymbol{E}$ ∥ [100] and strain ⊥ [100]
$Pm'c'2_1$ (26.70) $\mathbb{Z}_1$	• $\boldsymbol{B}$ ∥ [100]<br>• $\boldsymbol{E}$ ∥ [100]
$P2$ (3.1) $\mathbb{Z}_2$	• $\boldsymbol{B}$ ∥ [010] and strain ⊥ [010]<br>• $\boldsymbol{E}$ ∥ [010] and strain ⊥ [010]
$P\bar{1}'$ (2.6) $\mathbb{Z}_1$	• strain in generic direction
$P2/m'$ (10.45) $\mathbb{Z}_2$	• strain ⊥ [010]
Pm' (6.20) $\mathbb{Z}_1$	• $\boldsymbol{B}$ ∥ [010] and strain ⊥ [100]<br>• $\boldsymbol{B}$ ∥ [001] and strain ⊥ [100]<br>• $\boldsymbol{B}$ ⊥ [100]<br>• $\boldsymbol{E}$ ∥ [010] and strain ⊥ [100]<br>• $\boldsymbol{E}$ ∥ [010] and $\boldsymbol{B}$ ∥ [001]<br>• $\boldsymbol{E}$ ∥ [001] and strain ⊥ [100]<br>• $\boldsymbol{E}$ ∥ [001] and $\boldsymbol{B}$ ∥ [010]<br>• $\boldsymbol{E}$ ⊥ [100]
$Pm'a'2$ (28.91) $\mathbb{Z}_2$	• $\boldsymbol{B}$ ∥ [010]<br>• $\boldsymbol{E}$ ∥ [010]
$P2_1/m'$ (11.53) $\mathbb{Z}_1$	• strain ⊥ [100]

Continued on next page

...continued

Subgroup & SI group	Perturbation
$P2$ (3.1) $\mathbb{Z}_2$	• $\boldsymbol{B} \parallel [001]$ and strain $\perp [001]$<br>• $\boldsymbol{E} \parallel [001]$ and strain $\perp [001]$
$P2/c'$ (13.68) $\mathbb{Z}_2$	• strain $\perp [001]$
$Pm'm'2$ (25.60) $\mathbb{Z}_2$	• $\boldsymbol{B} \parallel [001]$<br>• $\boldsymbol{E} \parallel [001]$

Table. S 408: Subgroups of P_amma (51.298) that can be realized by applying $\boldsymbol{E}/\boldsymbol{B}$ fields and/or uniaxial strains.

Subgroup & SI group	Perturbation
Pm' (6.20) $\mathbb{Z}_1$	• $\boldsymbol{E} \parallel [100]$ and $\boldsymbol{B} \parallel [100]$ and strain $\perp [001]$<br>• $\boldsymbol{E} \parallel [100]$ and $\boldsymbol{B} \parallel [010]$ and strain $\perp [001]$<br>• $\boldsymbol{E} \parallel [100]$ and $\boldsymbol{B} \perp [001]$<br>• $\boldsymbol{E} \parallel [010]$ and $\boldsymbol{B} \parallel [100]$ and strain $\perp [001]$<br>• $\boldsymbol{E} \parallel [010]$ and $\boldsymbol{B} \parallel [010]$ and strain $\perp [001]$<br>• $\boldsymbol{E} \parallel [010]$ and $\boldsymbol{B} \perp [001]$<br>• $\boldsymbol{E} \perp [001]$ and $\boldsymbol{B} \parallel [100]$<br>• $\boldsymbol{E} \perp [001]$ and $\boldsymbol{B} \parallel [010]$<br>• $\boldsymbol{E} \perp [001]$ and $\boldsymbol{B} \perp [001]$
Pm (6.18) $\mathbb{Z}_1$	• $\boldsymbol{E} \parallel [100]$ and $\boldsymbol{B} \parallel [010]$ and strain $\perp [010]$<br>• $\boldsymbol{E} \parallel [001]$ and $\boldsymbol{B} \parallel [010]$ and strain $\perp [010]$<br>• $\boldsymbol{E} \perp [010]$ and $\boldsymbol{B} \parallel [010]$
$P2'$ (3.3) $\mathbb{Z}_1$	• $\boldsymbol{E} \parallel [100]$ and $\boldsymbol{B} \parallel [010]$ and strain $\perp [100]$<br>• $\boldsymbol{E} \parallel [100]$ and $\boldsymbol{B} \parallel [001]$ and strain $\perp [100]$<br>• $\boldsymbol{E} \parallel [100]$ and $\boldsymbol{B} \perp [100]$
$Pm'm2'$ (25.59) $\mathbb{Z}_1$	• $\boldsymbol{E} \parallel [100]$ and $\boldsymbol{B} \parallel [010]$

Continued on next page

...continued

Subgroup & SI group	Perturbation
P_S1 (1.3) $\mathbb{Z}_1$	• $\boldsymbol{E} \parallel [100]$ and strain in generic direction<br>• $\boldsymbol{E} \parallel [010]$ and strain in generic direction<br>• $\boldsymbol{E} \parallel [001]$ and strain in generic direction<br>• $\boldsymbol{E} \perp [100]$ and strain $\perp [010]$<br>• $\boldsymbol{E} \perp [100]$ and strain $\perp [001]$<br>• $\boldsymbol{E} \perp [100]$ and strain in generic direction<br>• $\boldsymbol{E} \perp [010]$ and strain $\perp [100]$<br>• $\boldsymbol{E} \perp [010]$ and strain $\perp [001]$<br>• $\boldsymbol{E} \perp [010]$ and strain in generic direction<br>• $\boldsymbol{E} \perp [001]$ and strain $\perp [100]$<br>• $\boldsymbol{E} \perp [001]$ and strain $\perp [010]$<br>• $\boldsymbol{E} \perp [001]$ and strain in generic direction<br>• $\boldsymbol{E}$ in generic direction
Pc (7.24) $\mathbb{Z}_1$	• $\boldsymbol{E} \parallel [100]$ and $\boldsymbol{B} \parallel [001]$ and strain $\perp [001]$<br>• $\boldsymbol{E} \parallel [010]$ and $\boldsymbol{B} \parallel [001]$ and strain $\perp [001]$<br>• $\boldsymbol{E} \perp [001]$ and $\boldsymbol{B} \parallel [001]$
P_cc (7.28) $\mathbb{Z}_1$	• $\boldsymbol{E} \parallel [100]$ and strain $\perp [001]$<br>• $\boldsymbol{E} \parallel [010]$ and strain $\perp [001]$<br>• $\boldsymbol{E} \perp [001]$
Pc' (7.26) $\mathbb{Z}_1$	• $\boldsymbol{E} \parallel [100]$ and $\boldsymbol{B} \parallel [100]$ and strain $\perp [010]$<br>• $\boldsymbol{E} \parallel [100]$ and $\boldsymbol{B} \parallel [001]$ and strain $\perp [010]$<br>• $\boldsymbol{E} \parallel [100]$ and $\boldsymbol{B} \perp [010]$<br>• $\boldsymbol{E} \parallel [001]$ and $\boldsymbol{B} \parallel [100]$ and strain $\perp [010]$<br>• $\boldsymbol{E} \parallel [001]$ and $\boldsymbol{B} \parallel [001]$ and strain $\perp [010]$<br>• $\boldsymbol{E} \parallel [001]$ and $\boldsymbol{B} \perp [010]$<br>• $\boldsymbol{E} \perp [010]$ and $\boldsymbol{B} \parallel [100]$<br>• $\boldsymbol{E} \perp [010]$ and $\boldsymbol{B} \parallel [001]$<br>• $\boldsymbol{E} \perp [010]$ and $\boldsymbol{B} \perp [010]$
P_am (6.21) $\mathbb{Z}_1$	• $\boldsymbol{E} \parallel [100]$ and strain $\perp [010]$<br>• $\boldsymbol{E} \parallel [001]$ and strain $\perp [010]$<br>• $\boldsymbol{E} \perp [010]$
$Pc'c2'$ (27.80) $\mathbb{Z}_1$	• $\boldsymbol{E} \parallel [100]$ and $\boldsymbol{B} \parallel [001]$
$P2_1$ (4.7) $\mathbb{Z}_1$	• $\boldsymbol{E} \parallel [100]$ and $\boldsymbol{B} \parallel [100]$ and strain $\perp [100]$
P_b2_1 (4.11) $\mathbb{Z}_1$	• $\boldsymbol{E} \parallel [100]$ and strain $\perp [100]$
$Pm'c'2_1$ (26.70) $\mathbb{Z}_1$	• $\boldsymbol{E} \parallel [100]$ and $\boldsymbol{B} \parallel [100]$
P_cmc2_1 (26.73) $\mathbb{Z}_1$	• $\boldsymbol{E} \parallel [100]$

Continued on next page

...continued

Subgroup & SI group	Perturbation
Pm' (6.20) $\mathbb{Z}_1$	• $\boldsymbol{E}$ ‖ [010] and $\boldsymbol{B}$ ‖ [010] and strain ⊥ [100]<br>• $\boldsymbol{E}$ ‖ [010] and $\boldsymbol{B}$ ‖ [001] and strain ⊥ [100]<br>• $\boldsymbol{E}$ ‖ [010] and $\boldsymbol{B}$ ⊥ [100]<br>• $\boldsymbol{E}$ ‖ [001] and $\boldsymbol{B}$ ‖ [010] and strain ⊥ [100]<br>• $\boldsymbol{E}$ ‖ [001] and $\boldsymbol{B}$ ‖ [001] and strain ⊥ [100]<br>• $\boldsymbol{E}$ ‖ [001] and $\boldsymbol{B}$ ⊥ [100]<br>• $\boldsymbol{E}$ ⊥ [100] and $\boldsymbol{B}$ ‖ [010]<br>• $\boldsymbol{E}$ ⊥ [100] and $\boldsymbol{B}$ ‖ [001]<br>• $\boldsymbol{E}$ ⊥ [100] and $\boldsymbol{B}$ ⊥ [100]
$P2$ (3.1) $\mathbb{Z}_2$	• $\boldsymbol{E}$ ‖ [010] and $\boldsymbol{B}$ ‖ [010] and strain ⊥ [010]
$Pm'm'2$ (25.60) $\mathbb{Z}_2$	• $\boldsymbol{E}$ ‖ [010] and $\boldsymbol{B}$ ‖ [010]
$P2'$ (3.3) $\mathbb{Z}_1$	• $\boldsymbol{E}$ ‖ [001] and $\boldsymbol{B}$ ‖ [100] and strain ⊥ [001]<br>• $\boldsymbol{E}$ ‖ [001] and $\boldsymbol{B}$ ‖ [010] and strain ⊥ [001]<br>• $\boldsymbol{E}$ ‖ [001] and $\boldsymbol{B}$ ⊥ [001]
$Pm'm2'$ (25.59) $\mathbb{Z}_1$	• $\boldsymbol{E}$ ‖ [001] and $\boldsymbol{B}$ ‖ [010]
$P\bar{1}$ (2.4) $\mathbb{Z}_2 \times \mathbb{Z}_2 \times \mathbb{Z}_2 \times \mathbb{Z}_4$	• $\boldsymbol{B}$ ‖ [100] and strain in generic direction<br>• $\boldsymbol{B}$ ‖ [010] and strain in generic direction<br>• $\boldsymbol{B}$ ‖ [001] and strain in generic direction<br>• $\boldsymbol{B}$ ⊥ [100] and strain ⊥ [010]<br>• $\boldsymbol{B}$ ⊥ [100] and strain ⊥ [001]<br>• $\boldsymbol{B}$ ⊥ [100] and strain in generic direction<br>• $\boldsymbol{B}$ ⊥ [010] and strain ⊥ [100]<br>• $\boldsymbol{B}$ ⊥ [010] and strain ⊥ [001]<br>• $\boldsymbol{B}$ ⊥ [010] and strain in generic direction<br>• $\boldsymbol{B}$ ⊥ [001] and strain ⊥ [100]<br>• $\boldsymbol{B}$ ⊥ [001] and strain ⊥ [010]<br>• $\boldsymbol{B}$ ⊥ [001] and strain in generic direction<br>• $\boldsymbol{B}$ in generic direction
$P2'/m'$ (10.46) $\mathbb{Z}_2 \times \mathbb{Z}_2 \times \mathbb{Z}_2 \times \mathbb{Z}_4$	• $\boldsymbol{B}$ ‖ [010] and strain ⊥ [100]<br>• $\boldsymbol{B}$ ‖ [001] and strain ⊥ [100]<br>• $\boldsymbol{B}$ ⊥ [100]
$P2/m$ (10.42) $\mathbb{Z}_2 \times \mathbb{Z}_2 \times \mathbb{Z}_2$	• $\boldsymbol{B}$ ‖ [010] and strain ⊥ [010]
$P2'/m'$ (10.46) $\mathbb{Z}_2 \times \mathbb{Z}_2 \times \mathbb{Z}_2 \times \mathbb{Z}_4$	• $\boldsymbol{B}$ ‖ [100] and strain ⊥ [001]<br>• $\boldsymbol{B}$ ‖ [010] and strain ⊥ [001]<br>• $\boldsymbol{B}$ ⊥ [001]
$Pm'm'm$ (47.252) $\mathbb{Z}_2 \times \mathbb{Z}_2 \times \mathbb{Z}_2$	• $\boldsymbol{B}$ ‖ [010]

Continued on next page

…continued

Subgroup & SI group	Perturbation
Pm (6.18) $\mathbb{Z}_1$	• $\boldsymbol{E}$ ∥ [010] and $\boldsymbol{B}$ ∥ [100] and strain ⊥ [100]<br>• $\boldsymbol{E}$ ∥ [001] and $\boldsymbol{B}$ ∥ [100] and strain ⊥ [100]<br>• $\boldsymbol{E}$ ⊥ [100] and $\boldsymbol{B}$ ∥ [100]
P_bm (6.22) $\mathbb{Z}_1$	• $\boldsymbol{E}$ ∥ [010] and strain ⊥ [100]<br>• $\boldsymbol{E}$ ∥ [001] and strain ⊥ [100]<br>• $\boldsymbol{E}$ ⊥ [100]
$Pma'2'$ (28.90) $\mathbb{Z}_1$	• $\boldsymbol{E}$ ∥ [001] and $\boldsymbol{B}$ ∥ [100]
$P2_1/m$ (11.50) $\mathbb{Z}_2$	• $\boldsymbol{B}$ ∥ [100] and strain ⊥ [100]
$P2'$ (3.3) $\mathbb{Z}_1$	• $\boldsymbol{E}$ ∥ [010] and $\boldsymbol{B}$ ∥ [100] and strain ⊥ [010]<br>• $\boldsymbol{E}$ ∥ [010] and $\boldsymbol{B}$ ∥ [001] and strain ⊥ [010]<br>• $\boldsymbol{E}$ ∥ [010] and $\boldsymbol{B}$ ⊥ [010]
$Pm'a2'$ (28.89) $\mathbb{Z}_1$	• $\boldsymbol{E}$ ∥ [010] and $\boldsymbol{B}$ ∥ [001]
P_a2 (3.4) $\mathbb{Z}_2$	• $\boldsymbol{E}$ ∥ [010] and strain ⊥ [010]
$P2'/c'$ (13.69) $\mathbb{Z}_2 \times \mathbb{Z}_2 \times \mathbb{Z}_4$	• $\boldsymbol{B}$ ∥ [100] and strain ⊥ [010]<br>• $\boldsymbol{B}$ ∥ [001] and strain ⊥ [010]<br>• $\boldsymbol{B}$ ⊥ [010]
$Pm'm2'$ (25.59) $\mathbb{Z}_1$	• $\boldsymbol{E}$ ∥ [010] and $\boldsymbol{B}$ ∥ [100]
P_ama2 (28.92) $\mathbb{Z}_1$	• $\boldsymbol{E}$ ∥ [010]
$Pmm'a'$ (51.295) $\mathbb{Z}_2$	• $\boldsymbol{B}$ ∥ [100]
$P2$ (3.1) $\mathbb{Z}_2$	• $\boldsymbol{E}$ ∥ [001] and $\boldsymbol{B}$ ∥ [001] and strain ⊥ [001]
$Pm'a'2$ (28.91) $\mathbb{Z}_2$	• $\boldsymbol{E}$ ∥ [001] and $\boldsymbol{B}$ ∥ [001]
P_a2 (3.4) $\mathbb{Z}_2$	• $\boldsymbol{E}$ ∥ [001] and strain ⊥ [001]
$P2/c$ (13.65) $\mathbb{Z}_2 \times \mathbb{Z}_2$	• $\boldsymbol{B}$ ∥ [001] and strain ⊥ [001]
P_amm2 (25.62) $\mathbb{Z}_1$	• $\boldsymbol{E}$ ∥ [001]
$Pc'cm'$ (49.270) $\mathbb{Z}_2 \times \mathbb{Z}_2$	• $\boldsymbol{B}$ ∥ [001]
$P_S\bar{1}$ (2.7) $\mathbb{Z}_2 \times \mathbb{Z}_2 \times \mathbb{Z}_4$	• strain in generic direction
P_b2_1/m (11.56) $\mathbb{Z}_2$	• strain ⊥ [100]
P_a2/m (10.47) $\mathbb{Z}_2 \times \mathbb{Z}_2 \times \mathbb{Z}_2$	• strain ⊥ [010]
P_c2/c (13.72) $\mathbb{Z}_2 \times \mathbb{Z}_2$	• strain ⊥ [001]

Table. S 409: Subgroups of P_bmma (51.299) that can be realized by applying $\boldsymbol{E}/\boldsymbol{B}$ fields and/or uniaxial strains.

Subgroup & SI group	Perturbation
P_S1 (1.3) $\mathbb{Z}_1$	• $\boldsymbol{E}$ ∥ [100] and strain in generic direction<br>• $\boldsymbol{E}$ ∥ [010] and strain in generic direction<br>• $\boldsymbol{E}$ ∥ [001] and strain in generic direction<br>• $\boldsymbol{E}$ ⊥ [100] and strain ⊥ [010]<br>• $\boldsymbol{E}$ ⊥ [100] and strain ⊥ [001]<br>• $\boldsymbol{E}$ ⊥ [100] and strain in generic direction<br>• $\boldsymbol{E}$ ⊥ [010] and strain ⊥ [100]<br>• $\boldsymbol{E}$ ⊥ [010] and strain ⊥ [001]<br>• $\boldsymbol{E}$ ⊥ [010] and strain in generic direction<br>• $\boldsymbol{E}$ ⊥ [001] and strain ⊥ [100]<br>• $\boldsymbol{E}$ ⊥ [001] and strain ⊥ [010]<br>• $\boldsymbol{E}$ ⊥ [001] and strain in generic direction<br>• $\boldsymbol{E}$ in generic direction
Pm (6.18) $\mathbb{Z}_1$	• $\boldsymbol{E}$ ∥ [100] and $\boldsymbol{B}$ ∥ [010] and strain ⊥ [010]<br>• $\boldsymbol{E}$ ∥ [001] and $\boldsymbol{B}$ ∥ [010] and strain ⊥ [010]<br>• $\boldsymbol{E}$ ⊥ [010] and $\boldsymbol{B}$ ∥ [010]
Pm' (6.20) $\mathbb{Z}_1$	• $\boldsymbol{E}$ ∥ [100] and $\boldsymbol{B}$ ∥ [100] and strain ⊥ [010]<br>• $\boldsymbol{E}$ ∥ [100] and $\boldsymbol{B}$ ∥ [001] and strain ⊥ [010]<br>• $\boldsymbol{E}$ ∥ [100] and $\boldsymbol{B}$ ⊥ [010]<br>• $\boldsymbol{E}$ ∥ [001] and $\boldsymbol{B}$ ∥ [100] and strain ⊥ [010]<br>• $\boldsymbol{E}$ ∥ [001] and $\boldsymbol{B}$ ∥ [001] and strain ⊥ [010]<br>• $\boldsymbol{E}$ ∥ [001] and $\boldsymbol{B}$ ⊥ [010]<br>• $\boldsymbol{E}$ ⊥ [010] and $\boldsymbol{B}$ ∥ [100]<br>• $\boldsymbol{E}$ ⊥ [010] and $\boldsymbol{B}$ ∥ [001]<br>• $\boldsymbol{E}$ ⊥ [010] and $\boldsymbol{B}$ ⊥ [010]
P_bm (6.22) $\mathbb{Z}_1$	• $\boldsymbol{E}$ ∥ [100] and strain ⊥ [010]<br>• $\boldsymbol{E}$ ∥ [001] and strain ⊥ [010]<br>• $\boldsymbol{E}$ ⊥ [010]
Pc (7.24) $\mathbb{Z}_1$	• $\boldsymbol{E}$ ∥ [100] and $\boldsymbol{B}$ ∥ [001] and strain ⊥ [001]<br>• $\boldsymbol{E}$ ∥ [010] and $\boldsymbol{B}$ ∥ [001] and strain ⊥ [001]<br>• $\boldsymbol{E}$ ⊥ [001] and $\boldsymbol{B}$ ∥ [001]

Continued on next page

. . . continued

Subgroup & SI group	Perturbation
Pc' (7.26) $\mathbb{Z}_1$	• $\boldsymbol{E} \parallel [100]$ and $\boldsymbol{B} \parallel [100]$ and strain $\perp [001]$<br>• $\boldsymbol{E} \parallel [100]$ and $\boldsymbol{B} \parallel [010]$ and strain $\perp [001]$<br>• $\boldsymbol{E} \parallel [100]$ and $\boldsymbol{B} \perp [001]$<br>• $\boldsymbol{E} \parallel [010]$ and $\boldsymbol{B} \parallel [100]$ and strain $\perp [001]$<br>• $\boldsymbol{E} \parallel [010]$ and $\boldsymbol{B} \parallel [010]$ and strain $\perp [001]$<br>• $\boldsymbol{E} \parallel [010]$ and $\boldsymbol{B} \perp [001]$<br>• $\boldsymbol{E} \perp [001]$ and $\boldsymbol{B} \parallel [100]$<br>• $\boldsymbol{E} \perp [001]$ and $\boldsymbol{B} \parallel [010]$<br>• $\boldsymbol{E} \perp [001]$ and $\boldsymbol{B} \perp [001]$
P_ac (7.27) $\mathbb{Z}_1$	• $\boldsymbol{E} \parallel [100]$ and strain $\perp [001]$<br>• $\boldsymbol{E} \parallel [010]$ and strain $\perp [001]$<br>• $\boldsymbol{E} \perp [001]$
$P2_1$ (4.7) $\mathbb{Z}_1$	• $\boldsymbol{E} \parallel [100]$ and $\boldsymbol{B} \parallel [100]$ and strain $\perp [100]$
$Pm'n'2_1$ (31.127) $\mathbb{Z}_1$	• $\boldsymbol{E} \parallel [100]$ and $\boldsymbol{B} \parallel [100]$
$P2_1'$ (4.9) $\mathbb{Z}_1$	• $\boldsymbol{E} \parallel [100]$ and $\boldsymbol{B} \parallel [010]$ and strain $\perp [100]$<br>• $\boldsymbol{E} \parallel [100]$ and $\boldsymbol{B} \parallel [001]$ and strain $\perp [100]$<br>• $\boldsymbol{E} \parallel [100]$ and $\boldsymbol{B} \perp [100]$
$Pm'c2_1'$ (26.68) $\mathbb{Z}_1$	• $\boldsymbol{E} \parallel [100]$ and $\boldsymbol{B} \parallel [001]$
$Pmn'2_1'$ (31.126) $\mathbb{Z}_1$	• $\boldsymbol{E} \parallel [100]$ and $\boldsymbol{B} \parallel [010]$
P_a2_1 (4.10) $\mathbb{Z}_1$	• $\boldsymbol{E} \parallel [100]$ and strain $\perp [100]$
P_amc2_1 (26.71) $\mathbb{Z}_1$	• $\boldsymbol{E} \parallel [100]$
$P2$ (3.1) $\mathbb{Z}_2$	• $\boldsymbol{E} \parallel [010]$ and $\boldsymbol{B} \parallel [010]$ and strain $\perp [010]$
$P2_1'$ (4.9) $\mathbb{Z}_1$	• $\boldsymbol{E} \parallel [010]$ and $\boldsymbol{B} \parallel [100]$ and strain $\perp [010]$<br>• $\boldsymbol{E} \parallel [010]$ and $\boldsymbol{B} \parallel [001]$ and strain $\perp [010]$<br>• $\boldsymbol{E} \parallel [010]$ and $\boldsymbol{B} \perp [010]$
P_b2 (3.5) $\mathbb{Z}_2$	• $\boldsymbol{E} \parallel [010]$ and strain $\perp [010]$

Continued on next page

...continued

Subgroup & SI group	Perturbation
$P\bar{1}$ (2.4) $\mathbb{Z}_2 \times \mathbb{Z}_2 \times \mathbb{Z}_2 \times \mathbb{Z}_4$	• $\boldsymbol{B} \parallel [100]$ and strain in generic direction<br>• $\boldsymbol{B} \parallel [010]$ and strain in generic direction<br>• $\boldsymbol{B} \parallel [001]$ and strain in generic direction<br>• $\boldsymbol{B} \perp [100]$ and strain $\perp [010]$<br>• $\boldsymbol{B} \perp [100]$ and strain $\perp [001]$<br>• $\boldsymbol{B} \perp [100]$ and strain in generic direction<br>• $\boldsymbol{B} \perp [010]$ and strain $\perp [100]$<br>• $\boldsymbol{B} \perp [010]$ and strain $\perp [001]$<br>• $\boldsymbol{B} \perp [010]$ and strain in generic direction<br>• $\boldsymbol{B} \perp [001]$ and strain $\perp [100]$<br>• $\boldsymbol{B} \perp [001]$ and strain $\perp [010]$<br>• $\boldsymbol{B} \perp [001]$ and strain in generic direction<br>• $\boldsymbol{B}$ in generic direction
$P2/m$ (10.42) $\mathbb{Z}_2 \times \mathbb{Z}_2 \times \mathbb{Z}_2$	• $\boldsymbol{B} \parallel [010]$ and strain $\perp [010]$
$P2_1'/m'$ (11.54) $\mathbb{Z}_2 \times \mathbb{Z}_2 \times \mathbb{Z}_4$	• $\boldsymbol{B} \parallel [100]$ and strain $\perp [010]$<br>• $\boldsymbol{B} \parallel [001]$ and strain $\perp [010]$<br>• $\boldsymbol{B} \perp [010]$
$P_S\bar{1}$ (2.7) $\mathbb{Z}_2 \times \mathbb{Z}_2 \times \mathbb{Z}_4$	• strain in generic direction
P_b2/m (10.48) $\mathbb{Z}_2 \times \mathbb{Z}_2$	• strain $\perp [010]$
Pm (6.18) $\mathbb{Z}_1$	• $\boldsymbol{E} \parallel [010]$ and $\boldsymbol{B} \parallel [100]$ and strain $\perp [100]$<br>• $\boldsymbol{E} \parallel [001]$ and $\boldsymbol{B} \parallel [100]$ and strain $\perp [100]$<br>• $\boldsymbol{E} \perp [100]$ and $\boldsymbol{B} \parallel [100]$
$Pmn'2_1'$ (31.126) $\mathbb{Z}_1$	• $\boldsymbol{E} \parallel [010]$ and $\boldsymbol{B} \parallel [100]$
$P2_1/m$ (11.50) $\mathbb{Z}_2$	• $\boldsymbol{B} \parallel [100]$ and strain $\perp [100]$
Pc' (7.26) $\mathbb{Z}_1$	• $\boldsymbol{E} \parallel [010]$ and $\boldsymbol{B} \parallel [010]$ and strain $\perp [100]$<br>• $\boldsymbol{E} \parallel [010]$ and $\boldsymbol{B} \parallel [001]$ and strain $\perp [100]$<br>• $\boldsymbol{E} \parallel [010]$ and $\boldsymbol{B} \perp [100]$<br>• $\boldsymbol{E} \parallel [001]$ and $\boldsymbol{B} \parallel [010]$ and strain $\perp [100]$<br>• $\boldsymbol{E} \parallel [001]$ and $\boldsymbol{B} \parallel [001]$ and strain $\perp [100]$<br>• $\boldsymbol{E} \parallel [001]$ and $\boldsymbol{B} \perp [100]$<br>• $\boldsymbol{E} \perp [100]$ and $\boldsymbol{B} \parallel [010]$<br>• $\boldsymbol{E} \perp [100]$ and $\boldsymbol{B} \parallel [001]$<br>• $\boldsymbol{E} \perp [100]$ and $\boldsymbol{B} \perp [100]$
$Pn'c'2$ (30.115) $\mathbb{Z}_1$	• $\boldsymbol{E} \parallel [010]$ and $\boldsymbol{B} \parallel [010]$
$Pc'a2_1'$ (29.101) $\mathbb{Z}_1$	• $\boldsymbol{E} \parallel [010]$ and $\boldsymbol{B} \parallel [001]$

Continued on next page

...continued

Subgroup & SI group	Perturbation
$P2_1'/c'$ (14.79) $\mathbb{Z}_2 \times \mathbb{Z}_4$	• $\boldsymbol{B} \parallel [010]$ and strain $\perp [100]$<br>• $\boldsymbol{B} \parallel [001]$ and strain $\perp [100]$<br>• $\boldsymbol{B} \perp [100]$
P_am (6.21) $\mathbb{Z}_1$	• $\boldsymbol{E} \parallel [010]$ and strain $\perp [100]$<br>• $\boldsymbol{E} \parallel [001]$ and strain $\perp [100]$<br>• $\boldsymbol{E} \perp [100]$
P_cma2 (28.94) $\mathbb{Z}_1$	• $\boldsymbol{E} \parallel [010]$
P_a2_1/m (11.55) $\mathbb{Z}_2$	• strain $\perp [100]$
$P2$ (3.1) $\mathbb{Z}_2$	• $\boldsymbol{E} \parallel [001]$ and $\boldsymbol{B} \parallel [001]$ and strain $\perp [001]$
$P2/c$ (13.65) $\mathbb{Z}_2 \times \mathbb{Z}_2$	• $\boldsymbol{B} \parallel [001]$ and strain $\perp [001]$
$Pm'a'2$ (28.91) $\mathbb{Z}_2$	• $\boldsymbol{E} \parallel [001]$ and $\boldsymbol{B} \parallel [001]$
$Pbc'm'$ (57.383) $\mathbb{Z}_2 \times \mathbb{Z}_2$	• $\boldsymbol{B} \parallel [001]$
$P2'$ (3.3) $\mathbb{Z}_1$	• $\boldsymbol{E} \parallel [001]$ and $\boldsymbol{B} \parallel [100]$ and strain $\perp [001]$<br>• $\boldsymbol{E} \parallel [001]$ and $\boldsymbol{B} \parallel [010]$ and strain $\perp [001]$<br>• $\boldsymbol{E} \parallel [001]$ and $\boldsymbol{B} \perp [001]$
$P2'/c'$ (13.69) $\mathbb{Z}_2 \times \mathbb{Z}_2 \times \mathbb{Z}_4$	• $\boldsymbol{B} \parallel [100]$ and strain $\perp [001]$<br>• $\boldsymbol{B} \parallel [010]$ and strain $\perp [001]$<br>• $\boldsymbol{B} \perp [001]$
$Pm'm2'$ (25.59) $\mathbb{Z}_1$	• $\boldsymbol{E} \parallel [001]$ and $\boldsymbol{B} \parallel [100]$
$Pmm'n'$ (59.410) $\mathbb{Z}_2$	• $\boldsymbol{B} \parallel [100]$
$Pma'2'$ (28.90) $\mathbb{Z}_1$	• $\boldsymbol{E} \parallel [001]$ and $\boldsymbol{B} \parallel [010]$
$Pmn'a'$ (53.327) $\mathbb{Z}_2 \times \mathbb{Z}_2$	• $\boldsymbol{B} \parallel [010]$
P_a2 (3.4) $\mathbb{Z}_2$	• $\boldsymbol{E} \parallel [001]$ and strain $\perp [001]$
P_a2/c (13.70) $\mathbb{Z}_2 \times \mathbb{Z}_2$	• strain $\perp [001]$
P_amm2 (25.62) $\mathbb{Z}_1$	• $\boldsymbol{E} \parallel [001]$

Table. S 410: Subgroups of P_cmma (51.300) that can be realized by applying $\boldsymbol{E}/\boldsymbol{B}$ fields and/or uniaxial strains.

Subgroup & SI group	Perturbation

Continued on next page

...continued

Subgroup & SI group	Perturbation
P_S1 (1.3) $\mathbb{Z}_1$	• $\boldsymbol{E}$ ∥ [100] and strain in generic direction<br>• $\boldsymbol{E}$ ∥ [010] and strain in generic direction<br>• $\boldsymbol{E}$ ∥ [001] and strain in generic direction<br>• $\boldsymbol{E} \perp$ [100] and strain $\perp$ [010]<br>• $\boldsymbol{E} \perp$ [100] and strain $\perp$ [001]<br>• $\boldsymbol{E} \perp$ [100] and strain in generic direction<br>• $\boldsymbol{E} \perp$ [010] and strain $\perp$ [100]<br>• $\boldsymbol{E} \perp$ [010] and strain $\perp$ [001]<br>• $\boldsymbol{E} \perp$ [010] and strain in generic direction<br>• $\boldsymbol{E} \perp$ [001] and strain $\perp$ [100]<br>• $\boldsymbol{E} \perp$ [001] and strain $\perp$ [010]<br>• $\boldsymbol{E} \perp$ [001] and strain in generic direction<br>• $\boldsymbol{E}$ in generic direction
Pm (6.18) $\mathbb{Z}_1$	• $\boldsymbol{E}$ ∥ [100] and $\boldsymbol{B}$ ∥ [010] and strain $\perp$ [010]<br>• $\boldsymbol{E}$ ∥ [001] and $\boldsymbol{B}$ ∥ [010] and strain $\perp$ [010]<br>• $\boldsymbol{E} \perp$ [010] and $\boldsymbol{B}$ ∥ [010]
Pc' (7.26) $\mathbb{Z}_1$	• $\boldsymbol{E}$ ∥ [100] and $\boldsymbol{B}$ ∥ [100] and strain $\perp$ [010]<br>• $\boldsymbol{E}$ ∥ [100] and $\boldsymbol{B}$ ∥ [001] and strain $\perp$ [010]<br>• $\boldsymbol{E}$ ∥ [100] and $\boldsymbol{B} \perp$ [010]<br>• $\boldsymbol{E}$ ∥ [001] and $\boldsymbol{B}$ ∥ [100] and strain $\perp$ [010]<br>• $\boldsymbol{E}$ ∥ [001] and $\boldsymbol{B}$ ∥ [001] and strain $\perp$ [010]<br>• $\boldsymbol{E}$ ∥ [001] and $\boldsymbol{B} \perp$ [010]<br>• $\boldsymbol{E} \perp$ [010] and $\boldsymbol{B}$ ∥ [100]<br>• $\boldsymbol{E} \perp$ [010] and $\boldsymbol{B}$ ∥ [001]<br>• $\boldsymbol{E} \perp$ [010] and $\boldsymbol{B} \perp$ [010]
P_am (6.21) $\mathbb{Z}_1$	• $\boldsymbol{E}$ ∥ [100] and strain $\perp$ [010]<br>• $\boldsymbol{E}$ ∥ [001] and strain $\perp$ [010]<br>• $\boldsymbol{E} \perp$ [010]
Pc (7.24) $\mathbb{Z}_1$	• $\boldsymbol{E}$ ∥ [100] and $\boldsymbol{B}$ ∥ [001] and strain $\perp$ [001]<br>• $\boldsymbol{E}$ ∥ [010] and $\boldsymbol{B}$ ∥ [001] and strain $\perp$ [001]<br>• $\boldsymbol{E} \perp$ [001] and $\boldsymbol{B}$ ∥ [001]

Continued on next page

...continued

Subgroup & SI group	Perturbation
Pc' (7.26) $\mathbb{Z}_1$	• $\boldsymbol{E} \parallel [100]$ and $\boldsymbol{B} \parallel [100]$ and strain $\perp [001]$<br>• $\boldsymbol{E} \parallel [100]$ and $\boldsymbol{B} \parallel [010]$ and strain $\perp [001]$<br>• $\boldsymbol{E} \parallel [100]$ and $\boldsymbol{B} \perp [001]$<br>• $\boldsymbol{E} \parallel [010]$ and $\boldsymbol{B} \parallel [100]$ and strain $\perp [001]$<br>• $\boldsymbol{E} \parallel [010]$ and $\boldsymbol{B} \parallel [010]$ and strain $\perp [001]$<br>• $\boldsymbol{E} \parallel [010]$ and $\boldsymbol{B} \perp [001]$<br>• $\boldsymbol{E} \perp [001]$ and $\boldsymbol{B} \parallel [100]$<br>• $\boldsymbol{E} \perp [001]$ and $\boldsymbol{B} \parallel [010]$<br>• $\boldsymbol{E} \perp [001]$ and $\boldsymbol{B} \perp [001]$
P_bc (7.29) $\mathbb{Z}_1$	• $\boldsymbol{E} \parallel [100]$ and strain $\perp [001]$<br>• $\boldsymbol{E} \parallel [010]$ and strain $\perp [001]$<br>• $\boldsymbol{E} \perp [001]$
$P2_1$ (4.7) $\mathbb{Z}_1$	• $\boldsymbol{E} \parallel [100]$ and $\boldsymbol{B} \parallel [100]$ and strain $\perp [100]$
$Pc'a'2_1$ (29.103) $\mathbb{Z}_1$	• $\boldsymbol{E} \parallel [100]$ and $\boldsymbol{B} \parallel [100]$
$P2_1'$ (4.9) $\mathbb{Z}_1$	• $\boldsymbol{E} \parallel [100]$ and $\boldsymbol{B} \parallel [010]$ and strain $\perp [100]$<br>• $\boldsymbol{E} \parallel [100]$ and $\boldsymbol{B} \parallel [001]$ and strain $\perp [100]$<br>• $\boldsymbol{E} \parallel [100]$ and $\boldsymbol{B} \perp [100]$
$Pca'2_1'$ (29.102) $\mathbb{Z}_1$	• $\boldsymbol{E} \parallel [100]$ and $\boldsymbol{B} \parallel [001]$
$Pmc'2_1'$ (26.69) $\mathbb{Z}_1$	• $\boldsymbol{E} \parallel [100]$ and $\boldsymbol{B} \parallel [010]$
P_a2_1 (4.10) $\mathbb{Z}_1$	• $\boldsymbol{E} \parallel [100]$ and strain $\perp [100]$
P_bmc2_1 (26.72) $\mathbb{Z}_1$	• $\boldsymbol{E} \parallel [100]$
$P2$ (3.1) $\mathbb{Z}_2$	• $\boldsymbol{E} \parallel [010]$ and $\boldsymbol{B} \parallel [010]$ and strain $\perp [010]$
$P2'$ (3.3) $\mathbb{Z}_1$	• $\boldsymbol{E} \parallel [010]$ and $\boldsymbol{B} \parallel [100]$ and strain $\perp [010]$<br>• $\boldsymbol{E} \parallel [010]$ and $\boldsymbol{B} \parallel [001]$ and strain $\perp [010]$<br>• $\boldsymbol{E} \parallel [010]$ and $\boldsymbol{B} \perp [010]$
P_a2 (3.4) $\mathbb{Z}_2$	• $\boldsymbol{E} \parallel [010]$ and strain $\perp [010]$

Continued on next page

…continued

Subgroup & SI group	Perturbation
$P\bar{1}$ (2.4) $\mathbb{Z}_2 \times \mathbb{Z}_2 \times \mathbb{Z}_2 \times \mathbb{Z}_4$	• $\boldsymbol{B}$ ∥ [100] and strain in generic direction<br>• $\boldsymbol{B}$ ∥ [010] and strain in generic direction<br>• $\boldsymbol{B}$ ∥ [001] and strain in generic direction<br>• $\boldsymbol{B} \perp$ [100] and strain $\perp$ [010]<br>• $\boldsymbol{B} \perp$ [100] and strain $\perp$ [001]<br>• $\boldsymbol{B} \perp$ [100] and strain in generic direction<br>• $\boldsymbol{B} \perp$ [010] and strain $\perp$ [100]<br>• $\boldsymbol{B} \perp$ [010] and strain $\perp$ [001]<br>• $\boldsymbol{B} \perp$ [010] and strain in generic direction<br>• $\boldsymbol{B} \perp$ [001] and strain $\perp$ [100]<br>• $\boldsymbol{B} \perp$ [001] and strain $\perp$ [010]<br>• $\boldsymbol{B} \perp$ [001] and strain in generic direction<br>• $\boldsymbol{B}$ in generic direction
$P2/m$ (10.42) $\mathbb{Z}_2 \times \mathbb{Z}_2 \times \mathbb{Z}_2$	• $\boldsymbol{B}$ ∥ [010] and strain $\perp$ [010]
$P2'/c'$ (13.69) $\mathbb{Z}_2 \times \mathbb{Z}_2 \times \mathbb{Z}_4$	• $\boldsymbol{B}$ ∥ [100] and strain $\perp$ [010]<br>• $\boldsymbol{B}$ ∥ [001] and strain $\perp$ [010]<br>• $\boldsymbol{B} \perp$ [010]
$P_S\bar{1}$ (2.7) $\mathbb{Z}_2 \times \mathbb{Z}_2 \times \mathbb{Z}_4$	• strain in generic direction
P_a2/m (10.47) $\mathbb{Z}_2 \times \mathbb{Z}_2 \times \mathbb{Z}_2$	• strain $\perp$ [010]
Pm (6.18) $\mathbb{Z}_1$	• $\boldsymbol{E}$ ∥ [010] and $\boldsymbol{B}$ ∥ [100] and strain $\perp$ [100]<br>• $\boldsymbol{E}$ ∥ [001] and $\boldsymbol{B}$ ∥ [100] and strain $\perp$ [100]<br>• $\boldsymbol{E} \perp$ [100] and $\boldsymbol{B}$ ∥ [100]
$Pma'2'$ (28.90) $\mathbb{Z}_1$	• $\boldsymbol{E}$ ∥ [010] and $\boldsymbol{B}$ ∥ [100]
$P2_1/m$ (11.50) $\mathbb{Z}_2$	• $\boldsymbol{B}$ ∥ [100] and strain $\perp$ [100]
Pc' (7.26) $\mathbb{Z}_1$	• $\boldsymbol{E}$ ∥ [010] and $\boldsymbol{B}$ ∥ [010] and strain $\perp$ [100]<br>• $\boldsymbol{E}$ ∥ [010] and $\boldsymbol{B}$ ∥ [001] and strain $\perp$ [100]<br>• $\boldsymbol{E}$ ∥ [010] and $\boldsymbol{B} \perp$ [100]<br>• $\boldsymbol{E}$ ∥ [001] and $\boldsymbol{B}$ ∥ [010] and strain $\perp$ [100]<br>• $\boldsymbol{E}$ ∥ [001] and $\boldsymbol{B}$ ∥ [001] and strain $\perp$ [100]<br>• $\boldsymbol{E}$ ∥ [001] and $\boldsymbol{B} \perp$ [100]<br>• $\boldsymbol{E} \perp$ [100] and $\boldsymbol{B}$ ∥ [010]<br>• $\boldsymbol{E} \perp$ [100] and $\boldsymbol{B}$ ∥ [001]<br>• $\boldsymbol{E} \perp$ [100] and $\boldsymbol{B} \perp$ [100]
$Pb'a'2$ (32.138) $\mathbb{Z}_2$	• $\boldsymbol{E}$ ∥ [010] and $\boldsymbol{B}$ ∥ [010]
$Pb'a2'$ (32.137) $\mathbb{Z}_1$	• $\boldsymbol{E}$ ∥ [010] and $\boldsymbol{B}$ ∥ [001]

Continued on next page

...continued

Subgroup & SI group	Perturbation
$P2_1'/c'$ (14.79) $\mathbb{Z}_2 \times \mathbb{Z}_4$	• $\boldsymbol{B} \parallel [010]$ and strain $\perp [100]$<br>• $\boldsymbol{B} \parallel [001]$ and strain $\perp [100]$<br>• $\boldsymbol{B} \perp [100]$
P_am (6.21) $\mathbb{Z}_1$	• $\boldsymbol{E} \parallel [010]$ and strain $\perp [100]$<br>• $\boldsymbol{E} \parallel [001]$ and strain $\perp [100]$<br>• $\boldsymbol{E} \perp [100]$
P_bma2 (28.93) $\mathbb{Z}_1$	• $\boldsymbol{E} \parallel [010]$
P_a2_1/m (11.55) $\mathbb{Z}_2$	• strain $\perp [100]$
$P2$ (3.1) $\mathbb{Z}_2$	• $\boldsymbol{E} \parallel [001]$ and $\boldsymbol{B} \parallel [001]$ and strain $\perp [001]$
$P2/c$ (13.65) $\mathbb{Z}_2 \times \mathbb{Z}_2$	• $\boldsymbol{B} \parallel [001]$ and strain $\perp [001]$
$Pc'c'2$ (27.81) $\mathbb{Z}_2$	• $\boldsymbol{E} \parallel [001]$ and $\boldsymbol{B} \parallel [001]$
$Pc'c'a$ (54.342) $\mathbb{Z}_2 \times \mathbb{Z}_2$	• $\boldsymbol{B} \parallel [001]$
$P2_1'$ (4.9) $\mathbb{Z}_1$	• $\boldsymbol{E} \parallel [001]$ and $\boldsymbol{B} \parallel [100]$ and strain $\perp [001]$<br>• $\boldsymbol{E} \parallel [001]$ and $\boldsymbol{B} \parallel [010]$ and strain $\perp [001]$<br>• $\boldsymbol{E} \parallel [001]$ and $\boldsymbol{B} \perp [001]$
$P2_1'/c'$ (14.79) $\mathbb{Z}_2 \times \mathbb{Z}_4$	• $\boldsymbol{B} \parallel [100]$ and strain $\perp [001]$<br>• $\boldsymbol{B} \parallel [010]$ and strain $\perp [001]$<br>• $\boldsymbol{B} \perp [001]$
$Pmc'2_1'$ (26.69) $\mathbb{Z}_1$	• $\boldsymbol{E} \parallel [001]$ and $\boldsymbol{B} \parallel [100]$
$Pb'c'm$ (57.382) $\mathbb{Z}_2$	• $\boldsymbol{B} \parallel [100]$
$Pmc'2_1'$ (26.69) $\mathbb{Z}_1$	• $\boldsymbol{E} \parallel [001]$ and $\boldsymbol{B} \parallel [010]$
$Pb'a'm$ (55.357) $\mathbb{Z}_2 \times \mathbb{Z}_2 \times \mathbb{Z}_2$	• $\boldsymbol{B} \parallel [010]$
P_b2 (3.5) $\mathbb{Z}_2$	• $\boldsymbol{E} \parallel [001]$ and strain $\perp [001]$
P_b2/c (13.71) $\mathbb{Z}_2 \times \mathbb{Z}_2$	• strain $\perp [001]$
P_cmm2 (25.61) $\mathbb{Z}_1$	• $\boldsymbol{E} \parallel [001]$

Table. S 411: Subgroups of P_Amma (51.301) that can be realized by applying $\boldsymbol{E}/\boldsymbol{B}$ fields and/or uniaxial strains.

Subgroup & SI group	Perturbation

Continued on next page

Subgroup & SI group	Perturbation
P_S1 (1.3) $\mathbb{Z}_1$	• $\boldsymbol{E} \parallel [100]$ and strain in generic direction<br>• $\boldsymbol{E} \parallel [010]$ and strain in generic direction<br>• $\boldsymbol{E} \parallel [001]$ and strain in generic direction<br>• $\boldsymbol{E} \perp [100]$ and strain $\perp [010]$<br>• $\boldsymbol{E} \perp [100]$ and strain $\perp [001]$<br>• $\boldsymbol{E} \perp [100]$ and strain in generic direction<br>• $\boldsymbol{E} \perp [010]$ and strain $\perp [100]$<br>• $\boldsymbol{E} \perp [010]$ and strain $\perp [001]$<br>• $\boldsymbol{E} \perp [010]$ and strain in generic direction<br>• $\boldsymbol{E} \perp [001]$ and strain $\perp [100]$<br>• $\boldsymbol{E} \perp [001]$ and strain $\perp [010]$<br>• $\boldsymbol{E} \perp [001]$ and strain in generic direction<br>• $\boldsymbol{E}$ in generic direction
Pm (6.18) $\mathbb{Z}_1$	• $\boldsymbol{E} \parallel [100]$ and $\boldsymbol{B} \parallel [010]$ and strain $\perp [010]$<br>• $\boldsymbol{E} \parallel [001]$ and $\boldsymbol{B} \parallel [010]$ and strain $\perp [010]$<br>• $\boldsymbol{E} \perp [010]$ and $\boldsymbol{B} \parallel [010]$
Pc' (7.26) $\mathbb{Z}_1$	• $\boldsymbol{E} \parallel [100]$ and $\boldsymbol{B} \parallel [100]$ and strain $\perp [010]$<br>• $\boldsymbol{E} \parallel [100]$ and $\boldsymbol{B} \parallel [001]$ and strain $\perp [010]$<br>• $\boldsymbol{E} \parallel [100]$ and $\boldsymbol{B} \perp [010]$<br>• $\boldsymbol{E} \parallel [001]$ and $\boldsymbol{B} \parallel [100]$ and strain $\perp [010]$<br>• $\boldsymbol{E} \parallel [001]$ and $\boldsymbol{B} \parallel [001]$ and strain $\perp [010]$<br>• $\boldsymbol{E} \parallel [001]$ and $\boldsymbol{B} \perp [010]$<br>• $\boldsymbol{E} \perp [010]$ and $\boldsymbol{B} \parallel [100]$<br>• $\boldsymbol{E} \perp [010]$ and $\boldsymbol{B} \parallel [001]$<br>• $\boldsymbol{E} \perp [010]$ and $\boldsymbol{B} \perp [010]$
P_Cm (6.23) $\mathbb{Z}_1$	• $\boldsymbol{E} \parallel [100]$ and strain $\perp [010]$<br>• $\boldsymbol{E} \parallel [001]$ and strain $\perp [010]$<br>• $\boldsymbol{E} \perp [010]$
Pc (7.24) $\mathbb{Z}_1$	• $\boldsymbol{E} \parallel [100]$ and $\boldsymbol{B} \parallel [001]$ and strain $\perp [001]$<br>• $\boldsymbol{E} \parallel [010]$ and $\boldsymbol{B} \parallel [001]$ and strain $\perp [001]$<br>• $\boldsymbol{E} \perp [001]$ and $\boldsymbol{B} \parallel [001]$

Continued on next page

...continued

Subgroup & SI group	Perturbation
Pc' (7.26) $\mathbb{Z}_1$	• $\boldsymbol{E} \parallel [100]$ and $\boldsymbol{B} \parallel [100]$ and strain $\perp [001]$<br>• $\boldsymbol{E} \parallel [100]$ and $\boldsymbol{B} \parallel [010]$ and strain $\perp [001]$<br>• $\boldsymbol{E} \parallel [100]$ and $\boldsymbol{B} \perp [001]$<br>• $\boldsymbol{E} \parallel [010]$ and $\boldsymbol{B} \parallel [100]$ and strain $\perp [001]$<br>• $\boldsymbol{E} \parallel [010]$ and $\boldsymbol{B} \parallel [010]$ and strain $\perp [001]$<br>• $\boldsymbol{E} \parallel [010]$ and $\boldsymbol{B} \perp [001]$<br>• $\boldsymbol{E} \perp [001]$ and $\boldsymbol{B} \parallel [100]$<br>• $\boldsymbol{E} \perp [001]$ and $\boldsymbol{B} \parallel [010]$<br>• $\boldsymbol{E} \perp [001]$ and $\boldsymbol{B} \perp [001]$
P_Cc (7.30) $\mathbb{Z}_1$	• $\boldsymbol{E} \parallel [100]$ and strain $\perp [001]$<br>• $\boldsymbol{E} \parallel [010]$ and strain $\perp [001]$<br>• $\boldsymbol{E} \perp [001]$
$P2_1$ (4.7) $\mathbb{Z}_1$	• $\boldsymbol{E} \parallel [100]$ and $\boldsymbol{B} \parallel [100]$ and strain $\perp [100]$
$Pn'a'2_1$ (33.148) $\mathbb{Z}_1$	• $\boldsymbol{E} \parallel [100]$ and $\boldsymbol{B} \parallel [100]$
$P2_1'$ (4.9) $\mathbb{Z}_1$	• $\boldsymbol{E} \parallel [100]$ and $\boldsymbol{B} \parallel [010]$ and strain $\perp [100]$<br>• $\boldsymbol{E} \parallel [100]$ and $\boldsymbol{B} \parallel [001]$ and strain $\perp [100]$<br>• $\boldsymbol{E} \parallel [100]$ and $\boldsymbol{B} \perp [100]$
$Pca'2_1'$ (29.102) $\mathbb{Z}_1$	• $\boldsymbol{E} \parallel [100]$ and $\boldsymbol{B} \parallel [001]$
$Pmn'2_1'$ (31.126) $\mathbb{Z}_1$	• $\boldsymbol{E} \parallel [100]$ and $\boldsymbol{B} \parallel [010]$
P_a2_1 (4.10) $\mathbb{Z}_1$	• $\boldsymbol{E} \parallel [100]$ and strain $\perp [100]$
P_Cmc2_1 (26.76) $\mathbb{Z}_1$	• $\boldsymbol{E} \parallel [100]$
$P2$ (3.1) $\mathbb{Z}_2$	• $\boldsymbol{E} \parallel [010]$ and $\boldsymbol{B} \parallel [010]$ and strain $\perp [010]$
$P2_1'$ (4.9) $\mathbb{Z}_1$	• $\boldsymbol{E} \parallel [010]$ and $\boldsymbol{B} \parallel [100]$ and strain $\perp [010]$<br>• $\boldsymbol{E} \parallel [010]$ and $\boldsymbol{B} \parallel [001]$ and strain $\perp [010]$<br>• $\boldsymbol{E} \parallel [010]$ and $\boldsymbol{B} \perp [010]$
P_C2 (3.6) $\mathbb{Z}_1$	• $\boldsymbol{E} \parallel [010]$ and strain $\perp [010]$

Continued on next page

...continued

Subgroup & SI group	Perturbation
$P\bar{1}$ (2.4) $\mathbb{Z}_2 \times \mathbb{Z}_2 \times \mathbb{Z}_2 \times \mathbb{Z}_4$	• $\boldsymbol{B} \parallel [100]$ and strain in generic direction<br>• $\boldsymbol{B} \parallel [010]$ and strain in generic direction<br>• $\boldsymbol{B} \parallel [001]$ and strain in generic direction<br>• $\boldsymbol{B} \perp [100]$ and strain $\perp [010]$<br>• $\boldsymbol{B} \perp [100]$ and strain $\perp [001]$<br>• $\boldsymbol{B} \perp [100]$ and strain in generic direction<br>• $\boldsymbol{B} \perp [010]$ and strain $\perp [100]$<br>• $\boldsymbol{B} \perp [010]$ and strain $\perp [001]$<br>• $\boldsymbol{B} \perp [010]$ and strain in generic direction<br>• $\boldsymbol{B} \perp [001]$ and strain $\perp [100]$<br>• $\boldsymbol{B} \perp [001]$ and strain $\perp [010]$<br>• $\boldsymbol{B} \perp [001]$ and strain in generic direction<br>• $\boldsymbol{B}$ in generic direction
$P2/m$ (10.42) $\mathbb{Z}_2 \times \mathbb{Z}_2 \times \mathbb{Z}_2$	• $\boldsymbol{B} \parallel [010]$ and strain $\perp [010]$
$P2_1'/c'$ (14.79) $\mathbb{Z}_2 \times \mathbb{Z}_4$	• $\boldsymbol{B} \parallel [100]$ and strain $\perp [010]$<br>• $\boldsymbol{B} \parallel [001]$ and strain $\perp [010]$<br>• $\boldsymbol{B} \perp [010]$
$P_S\bar{1}$ (2.7) $\mathbb{Z}_2 \times \mathbb{Z}_2 \times \mathbb{Z}_4$	• strain in generic direction
P_C2/m (10.49) $\mathbb{Z}_2 \times \mathbb{Z}_2$	• strain $\perp [010]$
Pm (6.18) $\mathbb{Z}_1$	• $\boldsymbol{E} \parallel [010]$ and $\boldsymbol{B} \parallel [100]$ and strain $\perp [100]$<br>• $\boldsymbol{E} \parallel [001]$ and $\boldsymbol{B} \parallel [100]$ and strain $\perp [100]$<br>• $\boldsymbol{E} \perp [100]$ and $\boldsymbol{B} \parallel [100]$
$Pmn'2_1'$ (31.126) $\mathbb{Z}_1$	• $\boldsymbol{E} \parallel [010]$ and $\boldsymbol{B} \parallel [100]$
$P2_1/m$ (11.50) $\mathbb{Z}_2$	• $\boldsymbol{B} \parallel [100]$ and strain $\perp [100]$
Pc' (7.26) $\mathbb{Z}_1$	• $\boldsymbol{E} \parallel [010]$ and $\boldsymbol{B} \parallel [010]$ and strain $\perp [100]$<br>• $\boldsymbol{E} \parallel [010]$ and $\boldsymbol{B} \parallel [001]$ and strain $\perp [100]$<br>• $\boldsymbol{E} \parallel [010]$ and $\boldsymbol{B} \perp [100]$<br>• $\boldsymbol{E} \parallel [001]$ and $\boldsymbol{B} \parallel [010]$ and strain $\perp [100]$<br>• $\boldsymbol{E} \parallel [001]$ and $\boldsymbol{B} \parallel [001]$ and strain $\perp [100]$<br>• $\boldsymbol{E} \parallel [001]$ and $\boldsymbol{B} \perp [100]$<br>• $\boldsymbol{E} \perp [100]$ and $\boldsymbol{B} \parallel [010]$<br>• $\boldsymbol{E} \perp [100]$ and $\boldsymbol{B} \parallel [001]$<br>• $\boldsymbol{E} \perp [100]$ and $\boldsymbol{B} \perp [100]$
$Pn'n'2$ (34.159) $\mathbb{Z}_1$	• $\boldsymbol{E} \parallel [010]$ and $\boldsymbol{B} \parallel [010]$
$Pn'a2_1'$ (33.146) $\mathbb{Z}_1$	• $\boldsymbol{E} \parallel [010]$ and $\boldsymbol{B} \parallel [001]$

Continued on next page

…continued

Subgroup & SI group	Perturbation
$P2_1'/c'$ (14.79) $\mathbb{Z}_2 \times \mathbb{Z}_4$	• $\boldsymbol{B}$ ∥ [010] and strain ⊥ [100]<br>• $\boldsymbol{B}$ ∥ [001] and strain ⊥ [100]<br>• $\boldsymbol{B}$ ⊥ [100]
P_am (6.21) $\mathbb{Z}_1$	• $\boldsymbol{E}$ ∥ [010] and strain ⊥ [100]<br>• $\boldsymbol{E}$ ∥ [001] and strain ⊥ [100]<br>• $\boldsymbol{E}$ ⊥ [100]
P_Ama2 (28.95) $\mathbb{Z}_1$	• $\boldsymbol{E}$ ∥ [010]
P_a2_1/m (11.55) $\mathbb{Z}_2$	• strain ⊥ [100]
$P2$ (3.1) $\mathbb{Z}_2$	• $\boldsymbol{E}$ ∥ [001] and $\boldsymbol{B}$ ∥ [001] and strain ⊥ [001]
$P2/c$ (13.65) $\mathbb{Z}_2 \times \mathbb{Z}_2$	• $\boldsymbol{B}$ ∥ [001] and strain ⊥ [001]
$Pn'c'2$ (30.115) $\mathbb{Z}_1$	• $\boldsymbol{E}$ ∥ [001] and $\boldsymbol{B}$ ∥ [001]
$Pb'cn'$ (60.424) $\mathbb{Z}_2 \times \mathbb{Z}_2$	• $\boldsymbol{B}$ ∥ [001]
$P2_1'$ (4.9) $\mathbb{Z}_1$	• $\boldsymbol{E}$ ∥ [001] and $\boldsymbol{B}$ ∥ [100] and strain ⊥ [001]<br>• $\boldsymbol{E}$ ∥ [001] and $\boldsymbol{B}$ ∥ [010] and strain ⊥ [001]<br>• $\boldsymbol{E}$ ∥ [001] and $\boldsymbol{B}$ ⊥ [001]
$P2_1'/c'$ (14.79) $\mathbb{Z}_2 \times \mathbb{Z}_4$	• $\boldsymbol{B}$ ∥ [100] and strain ⊥ [001]<br>• $\boldsymbol{B}$ ∥ [010] and strain ⊥ [001]<br>• $\boldsymbol{B}$ ⊥ [001]
$Pmc'2_1'$ (26.69) $\mathbb{Z}_1$	• $\boldsymbol{E}$ ∥ [001] and $\boldsymbol{B}$ ∥ [100]
$Pn'ma'$ (62.448) $\mathbb{Z}_2$	• $\boldsymbol{B}$ ∥ [100]
$Pmn'2_1'$ (31.126) $\mathbb{Z}_1$	• $\boldsymbol{E}$ ∥ [001] and $\boldsymbol{B}$ ∥ [010]
$Pn'n'm$ (58.397) $\mathbb{Z}_2 \times \mathbb{Z}_2$	• $\boldsymbol{B}$ ∥ [010]
P_C2 (3.6) $\mathbb{Z}_1$	• $\boldsymbol{E}$ ∥ [001] and strain ⊥ [001]
P_C2/c (13.74) $\mathbb{Z}_2$	• strain ⊥ [001]
P_Amm2 (25.64) $\mathbb{Z}_1$	• $\boldsymbol{E}$ ∥ [001]

Table. S 412: Subgroups of P_Bmma (51.302) that can be realized by applying $\boldsymbol{E}/\boldsymbol{B}$ fields and/or uniaxial strains.

Subgroup & SI group	Perturbation

Continued on next page

...continued

Subgroup & SI group	Perturbation
Pm' (6.20) $\mathbb{Z}_1$	• $\boldsymbol{E}$ ∥ [100] and $\boldsymbol{B}$ ∥ [100] and strain ⊥ [001]<br>• $\boldsymbol{E}$ ∥ [100] and $\boldsymbol{B}$ ∥ [010] and strain ⊥ [001]<br>• $\boldsymbol{E}$ ∥ [100] and $\boldsymbol{B}$ ⊥ [001]<br>• $\boldsymbol{E}$ ∥ [010] and $\boldsymbol{B}$ ∥ [100] and strain ⊥ [001]<br>• $\boldsymbol{E}$ ∥ [010] and $\boldsymbol{B}$ ∥ [010] and strain ⊥ [001]<br>• $\boldsymbol{E}$ ∥ [010] and $\boldsymbol{B}$ ⊥ [001]<br>• $\boldsymbol{E}$ ⊥ [001] and $\boldsymbol{B}$ ∥ [100]<br>• $\boldsymbol{E}$ ⊥ [001] and $\boldsymbol{B}$ ∥ [010]<br>• $\boldsymbol{E}$ ⊥ [001] and $\boldsymbol{B}$ ⊥ [001]
Pm (6.18) $\mathbb{Z}_1$	• $\boldsymbol{E}$ ∥ [100] and $\boldsymbol{B}$ ∥ [010] and strain ⊥ [010]<br>• $\boldsymbol{E}$ ∥ [001] and $\boldsymbol{B}$ ∥ [010] and strain ⊥ [010]<br>• $\boldsymbol{E}$ ⊥ [010] and $\boldsymbol{B}$ ∥ [010]
$P2'$ (3.3) $\mathbb{Z}_1$	• $\boldsymbol{E}$ ∥ [100] and $\boldsymbol{B}$ ∥ [010] and strain ⊥ [100]<br>• $\boldsymbol{E}$ ∥ [100] and $\boldsymbol{B}$ ∥ [001] and strain ⊥ [100]<br>• $\boldsymbol{E}$ ∥ [100] and $\boldsymbol{B}$ ⊥ [100]
$Pm'm2'$ (25.59) $\mathbb{Z}_1$	• $\boldsymbol{E}$ ∥ [100] and $\boldsymbol{B}$ ∥ [010]
P_S1 (1.3) $\mathbb{Z}_1$	• $\boldsymbol{E}$ ∥ [100] and strain in generic direction<br>• $\boldsymbol{E}$ ∥ [010] and strain in generic direction<br>• $\boldsymbol{E}$ ∥ [001] and strain in generic direction<br>• $\boldsymbol{E}$ ⊥ [100] and strain ⊥ [010]<br>• $\boldsymbol{E}$ ⊥ [100] and strain ⊥ [001]<br>• $\boldsymbol{E}$ ⊥ [100] and strain in generic direction<br>• $\boldsymbol{E}$ ⊥ [010] and strain ⊥ [100]<br>• $\boldsymbol{E}$ ⊥ [010] and strain ⊥ [001]<br>• $\boldsymbol{E}$ ⊥ [010] and strain in generic direction<br>• $\boldsymbol{E}$ ⊥ [001] and strain ⊥ [100]<br>• $\boldsymbol{E}$ ⊥ [001] and strain ⊥ [010]<br>• $\boldsymbol{E}$ ⊥ [001] and strain in generic direction<br>• $\boldsymbol{E}$ in generic direction
Pc (7.24) $\mathbb{Z}_1$	• $\boldsymbol{E}$ ∥ [100] and $\boldsymbol{B}$ ∥ [001] and strain ⊥ [001]<br>• $\boldsymbol{E}$ ∥ [010] and $\boldsymbol{B}$ ∥ [001] and strain ⊥ [001]<br>• $\boldsymbol{E}$ ⊥ [001] and $\boldsymbol{B}$ ∥ [001]
P_Ac (7.31) $\mathbb{Z}_1$	• $\boldsymbol{E}$ ∥ [100] and strain ⊥ [001]<br>• $\boldsymbol{E}$ ∥ [010] and strain ⊥ [001]<br>• $\boldsymbol{E}$ ⊥ [001]

Continued on next page

. . . continued

Subgroup & SI group	Perturbation
Pc' (7.26) $\mathbb{Z}_1$	• $\boldsymbol{E}$ ∥ [100] and $\boldsymbol{B}$ ∥ [100] and strain ⊥ [010]<br>• $\boldsymbol{E}$ ∥ [100] and $\boldsymbol{B}$ ∥ [001] and strain ⊥ [010]<br>• $\boldsymbol{E}$ ∥ [100] and $\boldsymbol{B}$ ⊥ [010]<br>• $\boldsymbol{E}$ ∥ [001] and $\boldsymbol{B}$ ∥ [100] and strain ⊥ [010]<br>• $\boldsymbol{E}$ ∥ [001] and $\boldsymbol{B}$ ∥ [001] and strain ⊥ [010]<br>• $\boldsymbol{E}$ ∥ [001] and $\boldsymbol{B}$ ⊥ [010]<br>• $\boldsymbol{E}$ ⊥ [010] and $\boldsymbol{B}$ ∥ [100]<br>• $\boldsymbol{E}$ ⊥ [010] and $\boldsymbol{B}$ ∥ [001]<br>• $\boldsymbol{E}$ ⊥ [010] and $\boldsymbol{B}$ ⊥ [010]
P_am (6.21) $\mathbb{Z}_1$	• $\boldsymbol{E}$ ∥ [100] and strain ⊥ [010]<br>• $\boldsymbol{E}$ ∥ [001] and strain ⊥ [010]<br>• $\boldsymbol{E}$ ⊥ [010]
$Pn'c2'$ (30.113) $\mathbb{Z}_1$	• $\boldsymbol{E}$ ∥ [100] and $\boldsymbol{B}$ ∥ [001]
$P2_1$ (4.7) $\mathbb{Z}_1$	• $\boldsymbol{E}$ ∥ [100] and $\boldsymbol{B}$ ∥ [100] and strain ⊥ [100]
P_C2_1 (4.12) $\mathbb{Z}_1$	• $\boldsymbol{E}$ ∥ [100] and strain ⊥ [100]
$Pm'n'2_1$ (31.127) $\mathbb{Z}_1$	• $\boldsymbol{E}$ ∥ [100] and $\boldsymbol{B}$ ∥ [100]
P_Amc2_1 (26.74) $\mathbb{Z}_1$	• $\boldsymbol{E}$ ∥ [100]
Pc' (7.26) $\mathbb{Z}_1$	• $\boldsymbol{E}$ ∥ [010] and $\boldsymbol{B}$ ∥ [010] and strain ⊥ [100]<br>• $\boldsymbol{E}$ ∥ [010] and $\boldsymbol{B}$ ∥ [001] and strain ⊥ [100]<br>• $\boldsymbol{E}$ ∥ [010] and $\boldsymbol{B}$ ⊥ [100]<br>• $\boldsymbol{E}$ ∥ [001] and $\boldsymbol{B}$ ∥ [010] and strain ⊥ [100]<br>• $\boldsymbol{E}$ ∥ [001] and $\boldsymbol{B}$ ∥ [001] and strain ⊥ [100]<br>• $\boldsymbol{E}$ ∥ [001] and $\boldsymbol{B}$ ⊥ [100]<br>• $\boldsymbol{E}$ ⊥ [100] and $\boldsymbol{B}$ ∥ [010]<br>• $\boldsymbol{E}$ ⊥ [100] and $\boldsymbol{B}$ ∥ [001]<br>• $\boldsymbol{E}$ ⊥ [100] and $\boldsymbol{B}$ ⊥ [100]
$P2$ (3.1) $\mathbb{Z}_2$	• $\boldsymbol{E}$ ∥ [010] and $\boldsymbol{B}$ ∥ [010] and strain ⊥ [010]
$Pm'a'2$ (28.91) $\mathbb{Z}_2$	• $\boldsymbol{E}$ ∥ [010] and $\boldsymbol{B}$ ∥ [010]
$P2_1'$ (4.9) $\mathbb{Z}_1$	• $\boldsymbol{E}$ ∥ [001] and $\boldsymbol{B}$ ∥ [100] and strain ⊥ [001]<br>• $\boldsymbol{E}$ ∥ [001] and $\boldsymbol{B}$ ∥ [010] and strain ⊥ [001]<br>• $\boldsymbol{E}$ ∥ [001] and $\boldsymbol{B}$ ⊥ [001]
$Pmc'2_1'$ (26.69) $\mathbb{Z}_1$	• $\boldsymbol{E}$ ∥ [001] and $\boldsymbol{B}$ ∥ [010]

Continued on next page

…continued

Subgroup & SI group	Perturbation
$P\bar{1}$ (2.4) $\mathbb{Z}_2 \times \mathbb{Z}_2 \times \mathbb{Z}_2 \times \mathbb{Z}_4$	• $\boldsymbol{B} \parallel [100]$ and strain in generic direction<br>• $\boldsymbol{B} \parallel [010]$ and strain in generic direction<br>• $\boldsymbol{B} \parallel [001]$ and strain in generic direction<br>• $\boldsymbol{B} \perp [100]$ and strain $\perp [010]$<br>• $\boldsymbol{B} \perp [100]$ and strain $\perp [001]$<br>• $\boldsymbol{B} \perp [100]$ and strain in generic direction<br>• $\boldsymbol{B} \perp [010]$ and strain $\perp [100]$<br>• $\boldsymbol{B} \perp [010]$ and strain $\perp [001]$<br>• $\boldsymbol{B} \perp [010]$ and strain in generic direction<br>• $\boldsymbol{B} \perp [001]$ and strain $\perp [100]$<br>• $\boldsymbol{B} \perp [001]$ and strain $\perp [010]$<br>• $\boldsymbol{B} \perp [001]$ and strain in generic direction<br>• $\boldsymbol{B}$ in generic direction
$P2'/c'$ (13.69) $\mathbb{Z}_2 \times \mathbb{Z}_2 \times \mathbb{Z}_4$	• $\boldsymbol{B} \parallel [010]$ and strain $\perp [100]$<br>• $\boldsymbol{B} \parallel [001]$ and strain $\perp [100]$<br>• $\boldsymbol{B} \perp [100]$
$P2/m$ (10.42) $\mathbb{Z}_2 \times \mathbb{Z}_2 \times \mathbb{Z}_2$	• $\boldsymbol{B} \parallel [010]$ and strain $\perp [010]$
$P2_1'/m'$ (11.54) $\mathbb{Z}_2 \times \mathbb{Z}_2 \times \mathbb{Z}_4$	• $\boldsymbol{B} \parallel [100]$ and strain $\perp [001]$<br>• $\boldsymbol{B} \parallel [010]$ and strain $\perp [001]$<br>• $\boldsymbol{B} \perp [001]$
$Pm'ma'$ (51.296) $\mathbb{Z}_2 \times \mathbb{Z}_2 \times \mathbb{Z}_2$	• $\boldsymbol{B} \parallel [010]$
Pm (6.18) $\mathbb{Z}_1$	• $\boldsymbol{E} \parallel [010]$ and $\boldsymbol{B} \parallel [100]$ and strain $\perp [100]$<br>• $\boldsymbol{E} \parallel [001]$ and $\boldsymbol{B} \parallel [100]$ and strain $\perp [100]$<br>• $\boldsymbol{E} \perp [100]$ and $\boldsymbol{B} \parallel [100]$
P_Cm (6.23) $\mathbb{Z}_1$	• $\boldsymbol{E} \parallel [010]$ and strain $\perp [100]$<br>• $\boldsymbol{E} \parallel [001]$ and strain $\perp [100]$<br>• $\boldsymbol{E} \perp [100]$
$Pmn'2_1'$ (31.126) $\mathbb{Z}_1$	• $\boldsymbol{E} \parallel [001]$ and $\boldsymbol{B} \parallel [100]$
$P2_1/m$ (11.50) $\mathbb{Z}_2$	• $\boldsymbol{B} \parallel [100]$ and strain $\perp [100]$
$P2'$ (3.3) $\mathbb{Z}_1$	• $\boldsymbol{E} \parallel [010]$ and $\boldsymbol{B} \parallel [100]$ and strain $\perp [010]$<br>• $\boldsymbol{E} \parallel [010]$ and $\boldsymbol{B} \parallel [001]$ and strain $\perp [010]$<br>• $\boldsymbol{E} \parallel [010]$ and $\boldsymbol{B} \perp [010]$
$Pb'a2'$ (32.137) $\mathbb{Z}_1$	• $\boldsymbol{E} \parallel [010]$ and $\boldsymbol{B} \parallel [001]$
P_a2 (3.4) $\mathbb{Z}_2$	• $\boldsymbol{E} \parallel [010]$ and strain $\perp [010]$

Continued on next page

. . . continued

Subgroup & SI group	Perturbation
$P2'/c'$ (13.69) $\mathbb{Z}_2 \times \mathbb{Z}_2 \times \mathbb{Z}_4$	• $\boldsymbol{B}$ ∥ [100] and strain ⊥ [010]<br>• $\boldsymbol{B}$ ∥ [001] and strain ⊥ [010]<br>• $\boldsymbol{B}$ ⊥ [010]
$Pm'm2'$ (25.59) $\mathbb{Z}_1$	• $\boldsymbol{E}$ ∥ [010] and $\boldsymbol{B}$ ∥ [100]
P_Cma2 (28.97) $\mathbb{Z}_1$	• $\boldsymbol{E}$ ∥ [010]
$Pmm'n'$ (59.410) $\mathbb{Z}_2$	• $\boldsymbol{B}$ ∥ [100]
$P2$ (3.1) $\mathbb{Z}_2$	• $\boldsymbol{E}$ ∥ [001] and $\boldsymbol{B}$ ∥ [001] and strain ⊥ [001]
$Pn'c'2$ (30.115) $\mathbb{Z}_1$	• $\boldsymbol{E}$ ∥ [001] and $\boldsymbol{B}$ ∥ [001]
P_C2 (3.6) $\mathbb{Z}_1$	• $\boldsymbol{E}$ ∥ [001] and strain ⊥ [001]
$P2/c$ (13.65) $\mathbb{Z}_2 \times \mathbb{Z}_2$	• $\boldsymbol{B}$ ∥ [001] and strain ⊥ [001]
P_Amm2 (25.64) $\mathbb{Z}_1$	• $\boldsymbol{E}$ ∥ [001]
$Pb'an'$ (50.282) $\mathbb{Z}_2$	• $\boldsymbol{B}$ ∥ [001]
$P_S\bar{1}$ (2.7) $\mathbb{Z}_2 \times \mathbb{Z}_2 \times \mathbb{Z}_4$	• strain in generic direction
P_C2_1/m (11.57) $\mathbb{Z}_2$	• strain ⊥ [100]
P_a2/m (10.47) $\mathbb{Z}_2 \times \mathbb{Z}_2 \times \mathbb{Z}_2$	• strain ⊥ [010]
P_A2/c (13.73) $\mathbb{Z}_2$	• strain ⊥ [001]

Table. S 413: Subgroups of P_Cmma (51.303) that can be realized by applying $\boldsymbol{E}/\boldsymbol{B}$ fields and/or uniaxial strains.

Subgroup & SI group	Perturbation
Pc' (7.26) $\mathbb{Z}_1$	• $\boldsymbol{E}$ ∥ [100] and $\boldsymbol{B}$ ∥ [100] and strain ⊥ [001]<br>• $\boldsymbol{E}$ ∥ [100] and $\boldsymbol{B}$ ∥ [010] and strain ⊥ [001]<br>• $\boldsymbol{E}$ ∥ [100] and $\boldsymbol{B}$ ⊥ [001]<br>• $\boldsymbol{E}$ ∥ [010] and $\boldsymbol{B}$ ∥ [100] and strain ⊥ [001]<br>• $\boldsymbol{E}$ ∥ [010] and $\boldsymbol{B}$ ∥ [010] and strain ⊥ [001]<br>• $\boldsymbol{E}$ ∥ [010] and $\boldsymbol{B}$ ⊥ [001]<br>• $\boldsymbol{E}$ ⊥ [001] and $\boldsymbol{B}$ ∥ [100]<br>• $\boldsymbol{E}$ ⊥ [001] and $\boldsymbol{B}$ ∥ [010]<br>• $\boldsymbol{E}$ ⊥ [001] and $\boldsymbol{B}$ ⊥ [001]

Continued on next page

...continued

Subgroup & SI group	Perturbation
Pm (6.18) $\mathbb{Z}_1$	• $\boldsymbol{E} \parallel [100]$ and $\boldsymbol{B} \parallel [010]$ and strain $\perp [010]$<br>• $\boldsymbol{E} \parallel [001]$ and $\boldsymbol{B} \parallel [010]$ and strain $\perp [010]$<br>• $\boldsymbol{E} \perp [010]$ and $\boldsymbol{B} \parallel [010]$
$P2'$ (3.3) $\mathbb{Z}_1$	• $\boldsymbol{E} \parallel [100]$ and $\boldsymbol{B} \parallel [010]$ and strain $\perp [100]$<br>• $\boldsymbol{E} \parallel [100]$ and $\boldsymbol{B} \parallel [001]$ and strain $\perp [100]$<br>• $\boldsymbol{E} \parallel [100]$ and $\boldsymbol{B} \perp [100]$
$Pma'2'$ (28.90) $\mathbb{Z}_1$	• $\boldsymbol{E} \parallel [100]$ and $\boldsymbol{B} \parallel [010]$
Pc (7.24) $\mathbb{Z}_1$	• $\boldsymbol{E} \parallel [100]$ and $\boldsymbol{B} \parallel [001]$ and strain $\perp [001]$<br>• $\boldsymbol{E} \parallel [010]$ and $\boldsymbol{B} \parallel [001]$ and strain $\perp [001]$<br>• $\boldsymbol{E} \perp [001]$ and $\boldsymbol{B} \parallel [001]$
P_S1 (1.3) $\mathbb{Z}_1$	• $\boldsymbol{E} \parallel [100]$ and strain in generic direction<br>• $\boldsymbol{E} \parallel [010]$ and strain in generic direction<br>• $\boldsymbol{E} \parallel [001]$ and strain in generic direction<br>• $\boldsymbol{E} \perp [100]$ and strain $\perp [010]$<br>• $\boldsymbol{E} \perp [100]$ and strain $\perp [001]$<br>• $\boldsymbol{E} \perp [100]$ and strain in generic direction<br>• $\boldsymbol{E} \perp [010]$ and strain $\perp [100]$<br>• $\boldsymbol{E} \perp [010]$ and strain $\perp [001]$<br>• $\boldsymbol{E} \perp [010]$ and strain in generic direction<br>• $\boldsymbol{E} \perp [001]$ and strain $\perp [100]$<br>• $\boldsymbol{E} \perp [001]$ and strain $\perp [010]$<br>• $\boldsymbol{E} \perp [001]$ and strain in generic direction<br>• $\boldsymbol{E}$ in generic direction
P_ac (7.27) $\mathbb{Z}_1$	• $\boldsymbol{E} \parallel [100]$ and strain $\perp [001]$<br>• $\boldsymbol{E} \parallel [010]$ and strain $\perp [001]$<br>• $\boldsymbol{E} \perp [001]$
$P2_1$ (4.7) $\mathbb{Z}_1$	• $\boldsymbol{E} \parallel [100]$ and $\boldsymbol{B} \parallel [100]$ and strain $\perp [100]$
P_C2_1 (4.12) $\mathbb{Z}_1$	• $\boldsymbol{E} \parallel [100]$ and strain $\perp [100]$
Pc' (7.26) $\mathbb{Z}_1$	• $\boldsymbol{E} \parallel [100]$ and $\boldsymbol{B} \parallel [100]$ and strain $\perp [010]$<br>• $\boldsymbol{E} \parallel [100]$ and $\boldsymbol{B} \parallel [001]$ and strain $\perp [010]$<br>• $\boldsymbol{E} \parallel [100]$ and $\boldsymbol{B} \perp [010]$<br>• $\boldsymbol{E} \parallel [001]$ and $\boldsymbol{B} \parallel [100]$ and strain $\perp [010]$<br>• $\boldsymbol{E} \parallel [001]$ and $\boldsymbol{B} \parallel [001]$ and strain $\perp [010]$<br>• $\boldsymbol{E} \parallel [001]$ and $\boldsymbol{B} \perp [010]$<br>• $\boldsymbol{E} \perp [010]$ and $\boldsymbol{B} \parallel [100]$<br>• $\boldsymbol{E} \perp [010]$ and $\boldsymbol{B} \parallel [001]$<br>• $\boldsymbol{E} \perp [010]$ and $\boldsymbol{B} \perp [010]$

Continued on next page

...continued

Subgroup & SI group	Perturbation
$Pc'c2'$ (27.80) $\mathbb{Z}_1$	• $\boldsymbol{E} \parallel$ [100] and $\boldsymbol{B} \parallel$ [001]
P_Cm (6.23) $\mathbb{Z}_1$	• $\boldsymbol{E} \parallel$ [100] and strain $\perp$ [010]<br>• $\boldsymbol{E} \parallel$ [001] and strain $\perp$ [010]<br>• $\boldsymbol{E} \perp$ [010]
$Pc'a'2_1$ (29.103) $\mathbb{Z}_1$	• $\boldsymbol{E} \parallel$ [100] and $\boldsymbol{B} \parallel$ [100]
P_Bmc2_1 (26.75) $\mathbb{Z}_1$	• $\boldsymbol{E} \parallel$ [100]
$P2$ (3.1) $\mathbb{Z}_2$	• $\boldsymbol{E} \parallel$ [010] and $\boldsymbol{B} \parallel$ [010] and strain $\perp$ [010]
Pc' (7.26) $\mathbb{Z}_1$	• $\boldsymbol{E} \parallel$ [010] and $\boldsymbol{B} \parallel$ [010] and strain $\perp$ [100]<br>• $\boldsymbol{E} \parallel$ [010] and $\boldsymbol{B} \parallel$ [001] and strain $\perp$ [100]<br>• $\boldsymbol{E} \parallel$ [010] and $\boldsymbol{B} \perp$ [100]<br>• $\boldsymbol{E} \parallel$ [001] and $\boldsymbol{B} \parallel$ [010] and strain $\perp$ [100]<br>• $\boldsymbol{E} \parallel$ [001] and $\boldsymbol{B} \parallel$ [001] and strain $\perp$ [100]<br>• $\boldsymbol{E} \parallel$ [001] and $\boldsymbol{B} \perp$ [100]<br>• $\boldsymbol{E} \perp$ [100] and $\boldsymbol{B} \parallel$ [010]<br>• $\boldsymbol{E} \perp$ [100] and $\boldsymbol{B} \parallel$ [001]<br>• $\boldsymbol{E} \perp$ [100] and $\boldsymbol{B} \perp$ [100]
$Pc'c'2$ (27.81) $\mathbb{Z}_2$	• $\boldsymbol{E} \parallel$ [010] and $\boldsymbol{B} \parallel$ [010]
$P\bar{1}$ (2.4) $\mathbb{Z}_2 \times \mathbb{Z}_2 \times \mathbb{Z}_2 \times \mathbb{Z}_4$	• $\boldsymbol{B} \parallel$ [100] and strain in generic direction<br>• $\boldsymbol{B} \parallel$ [010] and strain in generic direction<br>• $\boldsymbol{B} \parallel$ [001] and strain in generic direction<br>• $\boldsymbol{B} \perp$ [100] and strain $\perp$ [010]<br>• $\boldsymbol{B} \perp$ [100] and strain $\perp$ [001]<br>• $\boldsymbol{B} \perp$ [100] and strain in generic direction<br>• $\boldsymbol{B} \perp$ [010] and strain $\perp$ [100]<br>• $\boldsymbol{B} \perp$ [010] and strain $\perp$ [001]<br>• $\boldsymbol{B} \perp$ [010] and strain in generic direction<br>• $\boldsymbol{B} \perp$ [001] and strain $\perp$ [100]<br>• $\boldsymbol{B} \perp$ [001] and strain $\perp$ [010]<br>• $\boldsymbol{B} \perp$ [001] and strain in generic direction<br>• $\boldsymbol{B}$ in generic direction
$P2/m$ (10.42) $\mathbb{Z}_2 \times \mathbb{Z}_2 \times \mathbb{Z}_2$	• $\boldsymbol{B} \parallel$ [010] and strain $\perp$ [010]
$P2'/c'$ (13.69) $\mathbb{Z}_2 \times \mathbb{Z}_2 \times \mathbb{Z}_4$	• $\boldsymbol{B} \parallel$ [010] and strain $\perp$ [100]<br>• $\boldsymbol{B} \parallel$ [001] and strain $\perp$ [100]<br>• $\boldsymbol{B} \perp$ [100]

Continued on next page

. . . continued

Subgroup & SI group	Perturbation
$P2'$ (3.3) $\mathbb{Z}_1$	• $\boldsymbol{E}$ ∥ [001] and $\boldsymbol{B}$ ∥ [100] and strain ⊥ [001]<br>• $\boldsymbol{E}$ ∥ [001] and $\boldsymbol{B}$ ∥ [010] and strain ⊥ [001]<br>• $\boldsymbol{E}$ ∥ [001] and $\boldsymbol{B}$ ⊥ [001]
$Pma'2'$ (28.90) $\mathbb{Z}_1$	• $\boldsymbol{E}$ ∥ [001] and $\boldsymbol{B}$ ∥ [010]
$P2'/c'$ (13.69) $\mathbb{Z}_2 \times \mathbb{Z}_2 \times \mathbb{Z}_4$	• $\boldsymbol{B}$ ∥ [100] and strain ⊥ [001]<br>• $\boldsymbol{B}$ ∥ [010] and strain ⊥ [001]<br>• $\boldsymbol{B}$ ⊥ [001]
$Pc'c'm$ (49.269) $\mathbb{Z}_2 \times \mathbb{Z}_2$	• $\boldsymbol{B}$ ∥ [010]
Pm (6.18) $\mathbb{Z}_1$	• $\boldsymbol{E}$ ∥ [010] and $\boldsymbol{B}$ ∥ [100] and strain ⊥ [100]<br>• $\boldsymbol{E}$ ∥ [001] and $\boldsymbol{B}$ ∥ [100] and strain ⊥ [100]<br>• $\boldsymbol{E}$ ⊥ [100] and $\boldsymbol{B}$ ∥ [100]
P_Cm (6.23) $\mathbb{Z}_1$	• $\boldsymbol{E}$ ∥ [010] and strain ⊥ [100]<br>• $\boldsymbol{E}$ ∥ [001] and strain ⊥ [100]<br>• $\boldsymbol{E}$ ⊥ [100]
$P2_1/m$ (11.50) $\mathbb{Z}_2$	• $\boldsymbol{B}$ ∥ [100] and strain ⊥ [100]
$Pma'2'$ (28.90) $\mathbb{Z}_1$	• $\boldsymbol{E}$ ∥ [001] and $\boldsymbol{B}$ ∥ [100]
$P2_1'$ (4.9) $\mathbb{Z}_1$	• $\boldsymbol{E}$ ∥ [010] and $\boldsymbol{B}$ ∥ [100] and strain ⊥ [010]<br>• $\boldsymbol{E}$ ∥ [010] and $\boldsymbol{B}$ ∥ [001] and strain ⊥ [010]<br>• $\boldsymbol{E}$ ∥ [010] and $\boldsymbol{B}$ ⊥ [010]
P_C2 (3.6) $\mathbb{Z}_1$	• $\boldsymbol{E}$ ∥ [010] and strain ⊥ [010]
$Pc'a2_1'$ (29.101) $\mathbb{Z}_1$	• $\boldsymbol{E}$ ∥ [010] and $\boldsymbol{B}$ ∥ [001]
$P2_1'/c'$ (14.79) $\mathbb{Z}_2 \times \mathbb{Z}_4$	• $\boldsymbol{B}$ ∥ [100] and strain ⊥ [010]<br>• $\boldsymbol{B}$ ∥ [001] and strain ⊥ [010]<br>• $\boldsymbol{B}$ ⊥ [010]
$Pmc'2_1'$ (26.69) $\mathbb{Z}_1$	• $\boldsymbol{E}$ ∥ [010] and $\boldsymbol{B}$ ∥ [100]
P_Bma2 (28.96) $\mathbb{Z}_1$	• $\boldsymbol{E}$ ∥ [010]
$Pb'c'm$ (57.382) $\mathbb{Z}_2$	• $\boldsymbol{B}$ ∥ [100]
$P2$ (3.1) $\mathbb{Z}_2$	• $\boldsymbol{E}$ ∥ [001] and $\boldsymbol{B}$ ∥ [001] and strain ⊥ [001]
$Pb'a'2$ (32.138) $\mathbb{Z}_2$	• $\boldsymbol{E}$ ∥ [001] and $\boldsymbol{B}$ ∥ [001]
$P2/c$ (13.65) $\mathbb{Z}_2 \times \mathbb{Z}_2$	• $\boldsymbol{B}$ ∥ [001] and strain ⊥ [001]
P_a2 (3.4) $\mathbb{Z}_2$	• $\boldsymbol{E}$ ∥ [001] and strain ⊥ [001]
P_Cmm2 (25.63) $\mathbb{Z}_1$	• $\boldsymbol{E}$ ∥ [001]

Continued on next page

…continued

Subgroup & SI group	Perturbation
$Pc'ca'$ (54.344) $\mathbb{Z}_2 \times \mathbb{Z}_2$	• $\boldsymbol{B} \parallel [001]$
$P_S\bar{1}$ (2.7) $\mathbb{Z}_2 \times \mathbb{Z}_2 \times \mathbb{Z}_4$	• strain in generic direction
$P_C 2_1/m$ (11.57) $\mathbb{Z}_2$	• strain $\perp [100]$
$P_C 2/m$ (10.49) $\mathbb{Z}_2 \times \mathbb{Z}_2$	• strain $\perp [010]$
$P_a 2/c$ (13.70) $\mathbb{Z}_2 \times \mathbb{Z}_2$	• strain $\perp [001]$

Table. S 414: Subgroups of $P_I mma$ (51.304) that can be realized by applying $\boldsymbol{E}/\boldsymbol{B}$ fields and/or uniaxial strains.

Subgroup & SI group	Perturbation
Pc' (7.26) $\mathbb{Z}_1$	• $\boldsymbol{E} \parallel [100]$ and $\boldsymbol{B} \parallel [100]$ and strain $\perp [001]$<br>• $\boldsymbol{E} \parallel [100]$ and $\boldsymbol{B} \parallel [010]$ and strain $\perp [001]$<br>• $\boldsymbol{E} \parallel [100]$ and $\boldsymbol{B} \perp [001]$<br>• $\boldsymbol{E} \parallel [010]$ and $\boldsymbol{B} \parallel [100]$ and strain $\perp [001]$<br>• $\boldsymbol{E} \parallel [010]$ and $\boldsymbol{B} \parallel [010]$ and strain $\perp [001]$<br>• $\boldsymbol{E} \parallel [010]$ and $\boldsymbol{B} \perp [001]$<br>• $\boldsymbol{E} \perp [001]$ and $\boldsymbol{B} \parallel [100]$<br>• $\boldsymbol{E} \perp [001]$ and $\boldsymbol{B} \parallel [010]$<br>• $\boldsymbol{E} \perp [001]$ and $\boldsymbol{B} \perp [001]$
Pm (6.18) $\mathbb{Z}_1$	• $\boldsymbol{E} \parallel [100]$ and $\boldsymbol{B} \parallel [010]$ and strain $\perp [010]$<br>• $\boldsymbol{E} \parallel [001]$ and $\boldsymbol{B} \parallel [010]$ and strain $\perp [010]$<br>• $\boldsymbol{E} \perp [010]$ and $\boldsymbol{B} \parallel [010]$
$P2'$ (3.3) $\mathbb{Z}_1$	• $\boldsymbol{E} \parallel [100]$ and $\boldsymbol{B} \parallel [010]$ and strain $\perp [100]$<br>• $\boldsymbol{E} \parallel [100]$ and $\boldsymbol{B} \parallel [001]$ and strain $\perp [100]$<br>• $\boldsymbol{E} \parallel [100]$ and $\boldsymbol{B} \perp [100]$
$Pma'2'$ (28.90) $\mathbb{Z}_1$	• $\boldsymbol{E} \parallel [100]$ and $\boldsymbol{B} \parallel [010]$
Pc (7.24) $\mathbb{Z}_1$	• $\boldsymbol{E} \parallel [100]$ and $\boldsymbol{B} \parallel [001]$ and strain $\perp [001]$<br>• $\boldsymbol{E} \parallel [010]$ and $\boldsymbol{B} \parallel [001]$ and strain $\perp [001]$<br>• $\boldsymbol{E} \perp [001]$ and $\boldsymbol{B} \parallel [001]$

Continued on next page

...continued

Subgroup & SI group	Perturbation
P_S1 (1.3) $\mathbb{Z}_1$	• $\boldsymbol{E}$ ∥ [100] and strain in generic direction<br>• $\boldsymbol{E}$ ∥ [010] and strain in generic direction<br>• $\boldsymbol{E}$ ∥ [001] and strain in generic direction<br>• $\boldsymbol{E} \perp$ [100] and strain $\perp$ [010]<br>• $\boldsymbol{E} \perp$ [100] and strain $\perp$ [001]<br>• $\boldsymbol{E} \perp$ [100] and strain in generic direction<br>• $\boldsymbol{E} \perp$ [010] and strain $\perp$ [100]<br>• $\boldsymbol{E} \perp$ [010] and strain $\perp$ [001]<br>• $\boldsymbol{E} \perp$ [010] and strain in generic direction<br>• $\boldsymbol{E} \perp$ [001] and strain $\perp$ [100]<br>• $\boldsymbol{E} \perp$ [001] and strain $\perp$ [010]<br>• $\boldsymbol{E} \perp$ [001] and strain in generic direction<br>• $\boldsymbol{E}$ in generic direction
P_Cc (7.30) $\mathbb{Z}_1$	• $\boldsymbol{E}$ ∥ [100] and strain $\perp$ [001]<br>• $\boldsymbol{E}$ ∥ [010] and strain $\perp$ [001]<br>• $\boldsymbol{E} \perp$ [001]
$P2_1$ (4.7) $\mathbb{Z}_1$	• $\boldsymbol{E}$ ∥ [100] and $\boldsymbol{B}$ ∥ [100] and strain $\perp$ [100]
P_C2_1 (4.12) $\mathbb{Z}_1$	• $\boldsymbol{E}$ ∥ [100] and strain $\perp$ [100]
Pc' (7.26) $\mathbb{Z}_1$	• $\boldsymbol{E}$ ∥ [100] and $\boldsymbol{B}$ ∥ [100] and strain $\perp$ [010]<br>• $\boldsymbol{E}$ ∥ [100] and $\boldsymbol{B}$ ∥ [001] and strain $\perp$ [010]<br>• $\boldsymbol{E}$ ∥ [100] and $\boldsymbol{B} \perp$ [010]<br>• $\boldsymbol{E}$ ∥ [001] and $\boldsymbol{B}$ ∥ [100] and strain $\perp$ [010]<br>• $\boldsymbol{E}$ ∥ [001] and $\boldsymbol{B}$ ∥ [001] and strain $\perp$ [010]<br>• $\boldsymbol{E}$ ∥ [001] and $\boldsymbol{B} \perp$ [010]<br>• $\boldsymbol{E} \perp$ [010] and $\boldsymbol{B}$ ∥ [100]<br>• $\boldsymbol{E} \perp$ [010] and $\boldsymbol{B}$ ∥ [001]<br>• $\boldsymbol{E} \perp$ [010] and $\boldsymbol{B} \perp$ [010]
$Pn'c2'$ (30.113) $\mathbb{Z}_1$	• $\boldsymbol{E}$ ∥ [100] and $\boldsymbol{B}$ ∥ [001]
P_Cm (6.23) $\mathbb{Z}_1$	• $\boldsymbol{E}$ ∥ [100] and strain $\perp$ [010]<br>• $\boldsymbol{E}$ ∥ [001] and strain $\perp$ [010]<br>• $\boldsymbol{E} \perp$ [010]
$Pn'a'2_1$ (33.148) $\mathbb{Z}_1$	• $\boldsymbol{E}$ ∥ [100] and $\boldsymbol{B}$ ∥ [100]
P_Imc2_1 (26.77) $\mathbb{Z}_1$	• $\boldsymbol{E}$ ∥ [100]
$P2$ (3.1) $\mathbb{Z}_2$	• $\boldsymbol{E}$ ∥ [010] and $\boldsymbol{B}$ ∥ [010] and strain $\perp$ [010]

Continued on next page

. . . continued

Subgroup & SI group	Perturbation
Pc' (7.26) $\mathbb{Z}_1$	• $\boldsymbol{E} \parallel [010]$ and $\boldsymbol{B} \parallel [010]$ and strain $\perp [100]$<br>• $\boldsymbol{E} \parallel [010]$ and $\boldsymbol{B} \parallel [001]$ and strain $\perp [100]$<br>• $\boldsymbol{E} \parallel [010]$ and $\boldsymbol{B} \perp [100]$<br>• $\boldsymbol{E} \parallel [001]$ and $\boldsymbol{B} \parallel [010]$ and strain $\perp [100]$<br>• $\boldsymbol{E} \parallel [001]$ and $\boldsymbol{B} \parallel [001]$ and strain $\perp [100]$<br>• $\boldsymbol{E} \parallel [001]$ and $\boldsymbol{B} \perp [100]$<br>• $\boldsymbol{E} \perp [100]$ and $\boldsymbol{B} \parallel [010]$<br>• $\boldsymbol{E} \perp [100]$ and $\boldsymbol{B} \parallel [001]$<br>• $\boldsymbol{E} \perp [100]$ and $\boldsymbol{B} \perp [100]$
$Pn'c'2$ (30.115) $\mathbb{Z}_1$	• $\boldsymbol{E} \parallel [010]$ and $\boldsymbol{B} \parallel [010]$
$P\bar{1}$ (2.4) $\mathbb{Z}_2 \times \mathbb{Z}_2 \times \mathbb{Z}_2 \times \mathbb{Z}_4$	• $\boldsymbol{B} \parallel [100]$ and strain in generic direction<br>• $\boldsymbol{B} \parallel [010]$ and strain in generic direction<br>• $\boldsymbol{B} \parallel [001]$ and strain in generic direction<br>• $\boldsymbol{B} \perp [100]$ and strain $\perp [010]$<br>• $\boldsymbol{B} \perp [100]$ and strain $\perp [001]$<br>• $\boldsymbol{B} \perp [100]$ and strain in generic direction<br>• $\boldsymbol{B} \perp [010]$ and strain $\perp [100]$<br>• $\boldsymbol{B} \perp [010]$ and strain $\perp [001]$<br>• $\boldsymbol{B} \perp [010]$ and strain in generic direction<br>• $\boldsymbol{B} \perp [001]$ and strain $\perp [100]$<br>• $\boldsymbol{B} \perp [001]$ and strain $\perp [010]$<br>• $\boldsymbol{B} \perp [001]$ and strain in generic direction<br>• $\boldsymbol{B}$ in generic direction
$P2/m$ (10.42) $\mathbb{Z}_2 \times \mathbb{Z}_2 \times \mathbb{Z}_2$	• $\boldsymbol{B} \parallel [010]$ and strain $\perp [010]$
$P2'/c'$ (13.69) $\mathbb{Z}_2 \times \mathbb{Z}_2 \times \mathbb{Z}_4$	• $\boldsymbol{B} \parallel [010]$ and strain $\perp [100]$<br>• $\boldsymbol{B} \parallel [001]$ and strain $\perp [100]$<br>• $\boldsymbol{B} \perp [100]$
$P2_1'$ (4.9) $\mathbb{Z}_1$	• $\boldsymbol{E} \parallel [001]$ and $\boldsymbol{B} \parallel [100]$ and strain $\perp [001]$<br>• $\boldsymbol{E} \parallel [001]$ and $\boldsymbol{B} \parallel [010]$ and strain $\perp [001]$<br>• $\boldsymbol{E} \parallel [001]$ and $\boldsymbol{B} \perp [001]$
$Pmn'2_1'$ (31.126) $\mathbb{Z}_1$	• $\boldsymbol{E} \parallel [001]$ and $\boldsymbol{B} \parallel [010]$
$P2_1'/c'$ (14.79) $\mathbb{Z}_2 \times \mathbb{Z}_4$	• $\boldsymbol{B} \parallel [100]$ and strain $\perp [001]$<br>• $\boldsymbol{B} \parallel [010]$ and strain $\perp [001]$<br>• $\boldsymbol{B} \perp [001]$
$Pmn'a'$ (53.327) $\mathbb{Z}_2 \times \mathbb{Z}_2$	• $\boldsymbol{B} \parallel [010]$

Continued on next page

…continued

Subgroup & SI group	Perturbation
Pm (6.18) $\mathbb{Z}_1$	• $\boldsymbol{E} \parallel [010]$ and $\boldsymbol{B} \parallel [100]$ and strain $\perp [100]$<br>• $\boldsymbol{E} \parallel [001]$ and $\boldsymbol{B} \parallel [100]$ and strain $\perp [100]$<br>• $\boldsymbol{E} \perp [100]$ and $\boldsymbol{B} \parallel [100]$
P_Cm (6.23) $\mathbb{Z}_1$	• $\boldsymbol{E} \parallel [010]$ and strain $\perp [100]$<br>• $\boldsymbol{E} \parallel [001]$ and strain $\perp [100]$<br>• $\boldsymbol{E} \perp [100]$
$P2_1/m$ (11.50) $\mathbb{Z}_2$	• $\boldsymbol{B} \parallel [100]$ and strain $\perp [100]$
$Pmn'2_1'$ (31.126) $\mathbb{Z}_1$	• $\boldsymbol{E} \parallel [001]$ and $\boldsymbol{B} \parallel [100]$
$P2_1'$ (4.9) $\mathbb{Z}_1$	• $\boldsymbol{E} \parallel [010]$ and $\boldsymbol{B} \parallel [100]$ and strain $\perp [010]$<br>• $\boldsymbol{E} \parallel [010]$ and $\boldsymbol{B} \parallel [001]$ and strain $\perp [010]$<br>• $\boldsymbol{E} \parallel [010]$ and $\boldsymbol{B} \perp [010]$
P_C2 (3.6) $\mathbb{Z}_1$	• $\boldsymbol{E} \parallel [010]$ and strain $\perp [010]$
$Pn'a2_1'$ (33.146) $\mathbb{Z}_1$	• $\boldsymbol{E} \parallel [010]$ and $\boldsymbol{B} \parallel [001]$
$P2_1'/c'$ (14.79) $\mathbb{Z}_2 \times \mathbb{Z}_4$	• $\boldsymbol{B} \parallel [100]$ and strain $\perp [010]$<br>• $\boldsymbol{B} \parallel [001]$ and strain $\perp [010]$<br>• $\boldsymbol{B} \perp [010]$
$Pmc'2_1'$ (26.69) $\mathbb{Z}_1$	• $\boldsymbol{E} \parallel [010]$ and $\boldsymbol{B} \parallel [100]$
P_Ima2 (28.98) $\mathbb{Z}_1$	• $\boldsymbol{E} \parallel [010]$
$Pn'ma'$ (62.448) $\mathbb{Z}_2$	• $\boldsymbol{B} \parallel [100]$
$P2$ (3.1) $\mathbb{Z}_2$	• $\boldsymbol{E} \parallel [001]$ and $\boldsymbol{B} \parallel [001]$ and strain $\perp [001]$
$Pn'n'2$ (34.159) $\mathbb{Z}_1$	• $\boldsymbol{E} \parallel [001]$ and $\boldsymbol{B} \parallel [001]$
$P2/c$ (13.65) $\mathbb{Z}_2 \times \mathbb{Z}_2$	• $\boldsymbol{B} \parallel [001]$ and strain $\perp [001]$
P_C2 (3.6) $\mathbb{Z}_1$	• $\boldsymbol{E} \parallel [001]$ and strain $\perp [001]$
P_Imm2 (25.65) $\mathbb{Z}_1$	• $\boldsymbol{E} \parallel [001]$
$Pn'n'a$ (52.310) $\mathbb{Z}_2$	• $\boldsymbol{B} \parallel [001]$
$P_S\bar{1}$ (2.7) $\mathbb{Z}_2 \times \mathbb{Z}_2 \times \mathbb{Z}_4$	• strain in generic direction
P_C2_1/m (11.57) $\mathbb{Z}_2$	• strain $\perp [100]$
P_C2/m (10.49) $\mathbb{Z}_2 \times \mathbb{Z}_2$	• strain $\perp [010]$
P_C2/c (13.74) $\mathbb{Z}_2$	• strain $\perp [001]$

Table. S 415: Subgroups of $Pnna$ (52.305) that can be realized by applying $\boldsymbol{E}/\boldsymbol{B}$ fields and/or uniaxial strains.

Subgroup & SI group	Perturbation
$P2$ (3.1) $\mathbb{Z}_2$	• $\boldsymbol{E} \parallel [100]$ and strain $\perp [100]$<br>• $\boldsymbol{E} \parallel [100]$ and $\boldsymbol{B} \parallel [100]$
Pc (7.24) $\mathbb{Z}_1$	• $\boldsymbol{E} \parallel [100]$ and strain $\perp [001]$<br>• $\boldsymbol{E} \parallel [100]$ and $\boldsymbol{B} \parallel [001]$<br>• $\boldsymbol{E} \parallel [010]$ and strain $\perp [001]$<br>• $\boldsymbol{E} \parallel [010]$ and $\boldsymbol{B} \parallel [001]$<br>• $\boldsymbol{E} \perp [001]$
Pc (7.24) $\mathbb{Z}_1$	• $\boldsymbol{E} \parallel [100]$ and strain $\perp [010]$<br>• $\boldsymbol{E} \parallel [100]$ and $\boldsymbol{B} \parallel [010]$<br>• $\boldsymbol{E} \parallel [001]$ and strain $\perp [010]$<br>• $\boldsymbol{E} \parallel [001]$ and $\boldsymbol{B} \parallel [010]$<br>• $\boldsymbol{E} \perp [010]$
$Pnc2$ (30.111) $\mathbb{Z}_1$	• $\boldsymbol{E} \parallel [100]$
Pc (7.24) $\mathbb{Z}_1$	• $\boldsymbol{E} \parallel [010]$ and strain $\perp [100]$<br>• $\boldsymbol{E} \parallel [010]$ and $\boldsymbol{B} \parallel [100]$<br>• $\boldsymbol{E} \parallel [001]$ and strain $\perp [100]$<br>• $\boldsymbol{E} \parallel [001]$ and $\boldsymbol{B} \parallel [100]$<br>• $\boldsymbol{E} \perp [100]$
$P\bar{1}$ (2.4) $\mathbb{Z}_2 \times \mathbb{Z}_2 \times \mathbb{Z}_2 \times \mathbb{Z}_4$	• strain in generic direction<br>• $\boldsymbol{B} \parallel [100]$ and strain $\perp [010]$<br>• $\boldsymbol{B} \parallel [100]$ and strain $\perp [001]$<br>• $\boldsymbol{B} \parallel [010]$ and strain $\perp [100]$<br>• $\boldsymbol{B} \parallel [010]$ and strain $\perp [001]$<br>• $\boldsymbol{B} \parallel [001]$ and strain $\perp [100]$<br>• $\boldsymbol{B} \parallel [001]$ and strain $\perp [010]$<br>• $\boldsymbol{B}$ in generic direction
$P2/c$ (13.65) $\mathbb{Z}_2 \times \mathbb{Z}_2$	• strain $\perp [100]$<br>• $\boldsymbol{B} \parallel [100]$
$P2_1$ (4.7) $\mathbb{Z}_1$	• $\boldsymbol{E} \parallel [010]$ and strain $\perp [010]$<br>• $\boldsymbol{E} \parallel [010]$ and $\boldsymbol{B} \parallel [010]$
$Pna2_1$ (33.144) $\mathbb{Z}_1$	• $\boldsymbol{E} \parallel [010]$
$P2_1/c$ (14.75) $\mathbb{Z}_2$	• strain $\perp [010]$<br>• $\boldsymbol{B} \parallel [010]$
$P2$ (3.1) $\mathbb{Z}_2$	• $\boldsymbol{E} \parallel [001]$ and strain $\perp [001]$<br>• $\boldsymbol{E} \parallel [001]$ and $\boldsymbol{B} \parallel [001]$

Continued on next page

...continued

Subgroup & SI group	Perturbation
$Pnn2$ (34.156) $\mathbb{Z}_1$	• $\boldsymbol{E}$ ∥ [001]
$P2/c$ (13.65) $\mathbb{Z}_2 \times \mathbb{Z}_2$	• strain ⊥ [001]<br>• $\boldsymbol{B}$ ∥ [001]

Table. S 416: Subgroups of $Pnna1'$ (52.306) that can be realized by applying $\boldsymbol{E}/\boldsymbol{B}$ fields and/or uniaxial strains.

Subgroup & SI group	Perturbation
$P11'$ (1.2) $\mathbb{Z}_1$	• $\boldsymbol{E}$ ∥ [100] and strain in generic direction<br>• $\boldsymbol{E}$ ∥ [010] and strain in generic direction<br>• $\boldsymbol{E}$ ∥ [001] and strain in generic direction<br>• $\boldsymbol{E}$ ⊥ [100] and strain ⊥ [010]<br>• $\boldsymbol{E}$ ⊥ [100] and strain ⊥ [001]<br>• $\boldsymbol{E}$ ⊥ [100] and strain in generic direction<br>• $\boldsymbol{E}$ ⊥ [010] and strain ⊥ [100]<br>• $\boldsymbol{E}$ ⊥ [010] and strain ⊥ [001]<br>• $\boldsymbol{E}$ ⊥ [010] and strain in generic direction<br>• $\boldsymbol{E}$ ⊥ [001] and strain ⊥ [100]<br>• $\boldsymbol{E}$ ⊥ [001] and strain ⊥ [010]<br>• $\boldsymbol{E}$ ⊥ [001] and strain in generic direction<br>• $\boldsymbol{E}$ in generic direction
$P2'$ (3.3) $\mathbb{Z}_1$	• $\boldsymbol{E}$ ∥ [100] and $\boldsymbol{B}$ ∥ [010] and strain ⊥ [100]<br>• $\boldsymbol{E}$ ∥ [100] and $\boldsymbol{B}$ ∥ [001] and strain ⊥ [100]<br>• $\boldsymbol{E}$ ∥ [100] and $\boldsymbol{B}$ ⊥ [100]
$P2$ (3.1) $\mathbb{Z}_2$	• $\boldsymbol{E}$ ∥ [100] and $\boldsymbol{B}$ ∥ [100] and strain ⊥ [100]
$P21'$ (3.2) $\mathbb{Z}_2$	• $\boldsymbol{E}$ ∥ [100] and strain ⊥ [100]
Pc' (7.26) $\mathbb{Z}_1$	• $\boldsymbol{E}$ ∥ [100] and $\boldsymbol{B}$ ∥ [100] and strain ⊥ [001]<br>• $\boldsymbol{E}$ ∥ [100] and $\boldsymbol{B}$ ∥ [010] and strain ⊥ [001]<br>• $\boldsymbol{E}$ ∥ [100] and $\boldsymbol{B}$ ⊥ [001]<br>• $\boldsymbol{E}$ ∥ [010] and $\boldsymbol{B}$ ∥ [100] and strain ⊥ [001]<br>• $\boldsymbol{E}$ ∥ [010] and $\boldsymbol{B}$ ∥ [010] and strain ⊥ [001]<br>• $\boldsymbol{E}$ ∥ [010] and $\boldsymbol{B}$ ⊥ [001]<br>• $\boldsymbol{E}$ ⊥ [001] and $\boldsymbol{B}$ ∥ [100]<br>• $\boldsymbol{E}$ ⊥ [001] and $\boldsymbol{B}$ ∥ [010]<br>• $\boldsymbol{E}$ ⊥ [001] and $\boldsymbol{B}$ ⊥ [001]

Continued on next page

. . . continued

Subgroup & SI group	Perturbation
Pc (7.24) $\mathbb{Z}_1$	• $\boldsymbol{E} \parallel [100]$ and $\boldsymbol{B} \parallel [001]$ and strain $\perp [001]$<br>• $\boldsymbol{E} \parallel [010]$ and $\boldsymbol{B} \parallel [001]$ and strain $\perp [001]$<br>• $\boldsymbol{E} \perp [001]$ and $\boldsymbol{B} \parallel [001]$
$Pc1'$ (7.25) $\mathbb{Z}_1$	• $\boldsymbol{E} \parallel [100]$ and strain $\perp [001]$<br>• $\boldsymbol{E} \parallel [010]$ and strain $\perp [001]$<br>• $\boldsymbol{E} \perp [001]$
Pc' (7.26) $\mathbb{Z}_1$	• $\boldsymbol{E} \parallel [100]$ and $\boldsymbol{B} \parallel [100]$ and strain $\perp [010]$<br>• $\boldsymbol{E} \parallel [100]$ and $\boldsymbol{B} \parallel [001]$ and strain $\perp [010]$<br>• $\boldsymbol{E} \parallel [100]$ and $\boldsymbol{B} \perp [010]$<br>• $\boldsymbol{E} \parallel [001]$ and $\boldsymbol{B} \parallel [100]$ and strain $\perp [010]$<br>• $\boldsymbol{E} \parallel [001]$ and $\boldsymbol{B} \parallel [001]$ and strain $\perp [010]$<br>• $\boldsymbol{E} \parallel [001]$ and $\boldsymbol{B} \perp [010]$<br>• $\boldsymbol{E} \perp [010]$ and $\boldsymbol{B} \parallel [100]$<br>• $\boldsymbol{E} \perp [010]$ and $\boldsymbol{B} \parallel [001]$<br>• $\boldsymbol{E} \perp [010]$ and $\boldsymbol{B} \perp [010]$
$Pn'c'2$ (30.115) $\mathbb{Z}_1$	• $\boldsymbol{E} \parallel [100]$ and $\boldsymbol{B} \parallel [100]$
$Pn'c2'$ (30.113) $\mathbb{Z}_1$	• $\boldsymbol{E} \parallel [100]$ and $\boldsymbol{B} \parallel [001]$
Pc (7.24) $\mathbb{Z}_1$	• $\boldsymbol{E} \parallel [100]$ and $\boldsymbol{B} \parallel [010]$ and strain $\perp [010]$<br>• $\boldsymbol{E} \parallel [001]$ and $\boldsymbol{B} \parallel [010]$ and strain $\perp [010]$<br>• $\boldsymbol{E} \perp [010]$ and $\boldsymbol{B} \parallel [010]$
$Pnc'2'$ (30.114) $\mathbb{Z}_1$	• $\boldsymbol{E} \parallel [100]$ and $\boldsymbol{B} \parallel [010]$
$Pc1'$ (7.25) $\mathbb{Z}_1$	• $\boldsymbol{E} \parallel [100]$ and strain $\perp [010]$<br>• $\boldsymbol{E} \parallel [001]$ and strain $\perp [010]$<br>• $\boldsymbol{E} \perp [010]$
$Pnc21'$ (30.112) $\mathbb{Z}_1$	• $\boldsymbol{E} \parallel [100]$
Pc' (7.26) $\mathbb{Z}_1$	• $\boldsymbol{E} \parallel [010]$ and $\boldsymbol{B} \parallel [010]$ and strain $\perp [100]$<br>• $\boldsymbol{E} \parallel [010]$ and $\boldsymbol{B} \parallel [001]$ and strain $\perp [100]$<br>• $\boldsymbol{E} \parallel [010]$ and $\boldsymbol{B} \perp [100]$<br>• $\boldsymbol{E} \parallel [001]$ and $\boldsymbol{B} \parallel [010]$ and strain $\perp [100]$<br>• $\boldsymbol{E} \parallel [001]$ and $\boldsymbol{B} \parallel [001]$ and strain $\perp [100]$<br>• $\boldsymbol{E} \parallel [001]$ and $\boldsymbol{B} \perp [100]$<br>• $\boldsymbol{E} \perp [100]$ and $\boldsymbol{B} \parallel [010]$<br>• $\boldsymbol{E} \perp [100]$ and $\boldsymbol{B} \parallel [001]$<br>• $\boldsymbol{E} \perp [100]$ and $\boldsymbol{B} \perp [100]$

Continued on next page

...continued

Subgroup & SI group	Perturbation
Pc (7.24) $\mathbb{Z}_1$	• $\boldsymbol{E} \parallel [010]$ and $\boldsymbol{B} \parallel [100]$ and strain $\perp [100]$<br>• $\boldsymbol{E} \parallel [001]$ and $\boldsymbol{B} \parallel [100]$ and strain $\perp [100]$<br>• $\boldsymbol{E} \perp [100]$ and $\boldsymbol{B} \parallel [100]$
$Pc1'$ (7.25) $\mathbb{Z}_1$	• $\boldsymbol{E} \parallel [010]$ and strain $\perp [100]$<br>• $\boldsymbol{E} \parallel [001]$ and strain $\perp [100]$<br>• $\boldsymbol{E} \perp [100]$
$P\bar{1}$ (2.4) $\mathbb{Z}_2 \times \mathbb{Z}_2 \times \mathbb{Z}_2 \times \mathbb{Z}_4$	• $\boldsymbol{B} \parallel [100]$ and strain in generic direction<br>• $\boldsymbol{B} \parallel [010]$ and strain in generic direction<br>• $\boldsymbol{B} \parallel [001]$ and strain in generic direction<br>• $\boldsymbol{B} \perp [100]$ and strain $\perp [010]$<br>• $\boldsymbol{B} \perp [100]$ and strain $\perp [001]$<br>• $\boldsymbol{B} \perp [100]$ and strain in generic direction<br>• $\boldsymbol{B} \perp [010]$ and strain $\perp [100]$<br>• $\boldsymbol{B} \perp [010]$ and strain $\perp [001]$<br>• $\boldsymbol{B} \perp [010]$ and strain in generic direction<br>• $\boldsymbol{B} \perp [001]$ and strain $\perp [100]$<br>• $\boldsymbol{B} \perp [001]$ and strain $\perp [010]$<br>• $\boldsymbol{B} \perp [001]$ and strain in generic direction<br>• $\boldsymbol{B}$ in generic direction
$P2'/c'$ (13.69) $\mathbb{Z}_2 \times \mathbb{Z}_2 \times \mathbb{Z}_4$	• $\boldsymbol{B} \parallel [010]$ and strain $\perp [100]$<br>• $\boldsymbol{B} \parallel [001]$ and strain $\perp [100]$<br>• $\boldsymbol{B} \perp [100]$
$P2/c$ (13.65) $\mathbb{Z}_2 \times \mathbb{Z}_2$	• $\boldsymbol{B} \parallel [100]$ and strain $\perp [100]$
$P\bar{1}1'$ (2.5) $\mathbb{Z}_2 \times \mathbb{Z}_2 \times \mathbb{Z}_2 \times \mathbb{Z}_4$	• strain in generic direction
$P2/c1'$ (13.66) $\mathbb{Z}_2 \times \mathbb{Z}_2$	• strain $\perp [100]$
$P2_1'$ (4.9) $\mathbb{Z}_1$	• $\boldsymbol{E} \parallel [010]$ and $\boldsymbol{B} \parallel [100]$ and strain $\perp [010]$<br>• $\boldsymbol{E} \parallel [010]$ and $\boldsymbol{B} \parallel [001]$ and strain $\perp [010]$<br>• $\boldsymbol{E} \parallel [010]$ and $\boldsymbol{B} \perp [010]$
$Pn'a2_1'$ (33.146) $\mathbb{Z}_1$	• $\boldsymbol{E} \parallel [010]$ and $\boldsymbol{B} \parallel [001]$
$Pna'2_1'$ (33.147) $\mathbb{Z}_1$	• $\boldsymbol{E} \parallel [010]$ and $\boldsymbol{B} \parallel [100]$
$P2_1'/c'$ (14.79) $\mathbb{Z}_2 \times \mathbb{Z}_4$	• $\boldsymbol{B} \parallel [100]$ and strain $\perp [010]$<br>• $\boldsymbol{B} \parallel [001]$ and strain $\perp [010]$<br>• $\boldsymbol{B} \perp [010]$
$P2_1$ (4.7) $\mathbb{Z}_1$	• $\boldsymbol{E} \parallel [010]$ and $\boldsymbol{B} \parallel [010]$ and strain $\perp [010]$
$Pn'a'2_1$ (33.148) $\mathbb{Z}_1$	• $\boldsymbol{E} \parallel [010]$ and $\boldsymbol{B} \parallel [010]$

Continued on next page

. . . continued

Subgroup & SI group	Perturbation
$P2_1/c$ (14.75) $\mathbb{Z}_2$	• $\boldsymbol{B}$ ∥ [010] and strain ⊥ [010]
$P2_11'$ (4.8) $\mathbb{Z}_1$	• $\boldsymbol{E}$ ∥ [010] and strain ⊥ [010]
$Pna2_11'$ (33.145) $\mathbb{Z}_1$	• $\boldsymbol{E}$ ∥ [010]
$P2_1/c1'$ (14.76) $\mathbb{Z}_2$	• strain ⊥ [010]
$P2'$ (3.3) $\mathbb{Z}_1$	• $\boldsymbol{E}$ ∥ [001] and $\boldsymbol{B}$ ∥ [100] and strain ⊥ [001]<br>• $\boldsymbol{E}$ ∥ [001] and $\boldsymbol{B}$ ∥ [010] and strain ⊥ [001]<br>• $\boldsymbol{E}$ ∥ [001] and $\boldsymbol{B}$ ⊥ [001]
$Pn'n2'$ (34.158) $\mathbb{Z}_1$	• $\boldsymbol{E}$ ∥ [001] and $\boldsymbol{B}$ ∥ [010]
$Pn'n2'$ (34.158) $\mathbb{Z}_1$	• $\boldsymbol{E}$ ∥ [001] and $\boldsymbol{B}$ ∥ [100]
$P2'/c'$ (13.69) $\mathbb{Z}_2 \times \mathbb{Z}_2 \times \mathbb{Z}_4$	• $\boldsymbol{B}$ ∥ [100] and strain ⊥ [001]<br>• $\boldsymbol{B}$ ∥ [010] and strain ⊥ [001]<br>• $\boldsymbol{B}$ ⊥ [001]
$Pnn'a'$ (52.311) $\mathbb{Z}_2$	• $\boldsymbol{B}$ ∥ [100]
$Pn'na'$ (52.312) $\mathbb{Z}_2$	• $\boldsymbol{B}$ ∥ [010]
$P2$ (3.1) $\mathbb{Z}_2$	• $\boldsymbol{E}$ ∥ [001] and $\boldsymbol{B}$ ∥ [001] and strain ⊥ [001]
$Pn'n'2$ (34.159) $\mathbb{Z}_1$	• $\boldsymbol{E}$ ∥ [001] and $\boldsymbol{B}$ ∥ [001]
$P2/c$ (13.65) $\mathbb{Z}_2 \times \mathbb{Z}_2$	• $\boldsymbol{B}$ ∥ [001] and strain ⊥ [001]
$Pn'n'a$ (52.310) $\mathbb{Z}_2$	• $\boldsymbol{B}$ ∥ [001]
$P21'$ (3.2) $\mathbb{Z}_2$	• $\boldsymbol{E}$ ∥ [001] and strain ⊥ [001]
$Pnn21'$ (34.157) $\mathbb{Z}_1$	• $\boldsymbol{E}$ ∥ [001]
$P2/c1'$ (13.66) $\mathbb{Z}_2 \times \mathbb{Z}_2$	• strain ⊥ [001]

Table. S 417: Subgroups of $Pn'na$ (52.307) that can be realized by applying $\boldsymbol{E}/\boldsymbol{B}$ fields and/or uniaxial strains.

Subgroup & SI group	Perturbation
$P2$ (3.1) $\mathbb{Z}_2$	• $\boldsymbol{B}$ ∥ [100] and strain ⊥ [100]<br>• $\boldsymbol{E}$ ∥ [100] and strain ⊥ [100]<br>• $\boldsymbol{E}$ ∥ [100] and $\boldsymbol{B}$ ∥ [100]

Continued on next page

...continued

Subgroup & SI group	Perturbation
Pc (7.24) $\mathbb{Z}_1$	• $\boldsymbol{B} \parallel [001]$ and strain $\perp [001]$<br>• $\boldsymbol{E} \parallel [100]$ and strain $\perp [001]$<br>• $\boldsymbol{E} \parallel [100]$ and $\boldsymbol{B} \parallel [001]$<br>• $\boldsymbol{E} \parallel [010]$ and strain $\perp [001]$<br>• $\boldsymbol{E} \perp [001]$
Pc (7.24) $\mathbb{Z}_1$	• $\boldsymbol{B} \parallel [010]$ and strain $\perp [010]$<br>• $\boldsymbol{E} \parallel [100]$ and strain $\perp [010]$<br>• $\boldsymbol{E} \parallel [100]$ and $\boldsymbol{B} \parallel [010]$<br>• $\boldsymbol{E} \parallel [001]$ and strain $\perp [010]$<br>• $\boldsymbol{E} \perp [010]$
$Pnc2$ (30.111) $\mathbb{Z}_1$	• $\boldsymbol{E} \parallel [100]$
Pc' (7.26) $\mathbb{Z}_1$	• $\boldsymbol{B} \parallel [010]$ and strain $\perp [100]$<br>• $\boldsymbol{B} \parallel [001]$ and strain $\perp [100]$<br>• $\boldsymbol{B} \perp [100]$<br>• $\boldsymbol{E} \parallel [010]$ and strain $\perp [100]$<br>• $\boldsymbol{E} \parallel [010]$ and $\boldsymbol{B} \parallel [010]$<br>• $\boldsymbol{E} \parallel [001]$ and strain $\perp [100]$<br>• $\boldsymbol{E} \parallel [001]$ and $\boldsymbol{B} \parallel [001]$<br>• $\boldsymbol{E} \perp [100]$
$P\bar{1}'$ (2.6) $\mathbb{Z}_1$	• strain in generic direction
$P2/c'$ (13.68) $\mathbb{Z}_2$	• strain $\perp [100]$
$P2_1'$ (4.9) $\mathbb{Z}_1$	• $\boldsymbol{B} \parallel [100]$ and strain $\perp [010]$<br>• $\boldsymbol{B} \parallel [001]$ and strain $\perp [010]$<br>• $\boldsymbol{B} \perp [010]$<br>• $\boldsymbol{E} \parallel [010]$ and strain $\perp [010]$<br>• $\boldsymbol{E} \parallel [010]$ and $\boldsymbol{B} \parallel [100]$
$Pn'a2_1'$ (33.146) $\mathbb{Z}_1$	• $\boldsymbol{B} \parallel [001]$<br>• $\boldsymbol{E} \parallel [010]$
$P2_1'/c$ (14.77) $\mathbb{Z}_1$	• strain $\perp [010]$
$P2'$ (3.3) $\mathbb{Z}_1$	• $\boldsymbol{B} \parallel [100]$ and strain $\perp [001]$<br>• $\boldsymbol{B} \parallel [010]$ and strain $\perp [001]$<br>• $\boldsymbol{B} \perp [001]$<br>• $\boldsymbol{E} \parallel [001]$ and strain $\perp [001]$<br>• $\boldsymbol{E} \parallel [001]$ and $\boldsymbol{B} \parallel [100]$
$Pn'n2'$ (34.158) $\mathbb{Z}_1$	• $\boldsymbol{B} \parallel [010]$<br>• $\boldsymbol{E} \parallel [001]$

Continued on next page

…continued

Subgroup & SI group	Perturbation
$P2'/c$ (13.67) $\mathbb{Z}_1$	• strain ⊥ [001]
$P22'2_1'$ (17.10) $\mathbb{Z}_2$	• $\boldsymbol{B}$ ∥ [100]

Table. S 418: Subgroups of $Pnn'a$ (52.308) that can be realized by applying $\boldsymbol{E}/\boldsymbol{B}$ fields and/or uniaxial strains.

Subgroup & SI group	Perturbation
$P2'$ (3.3) $\mathbb{Z}_1$	• $\boldsymbol{B}$ ∥ [010] and strain ⊥ [100]<br>• $\boldsymbol{B}$ ∥ [001] and strain ⊥ [100]<br>• $\boldsymbol{B}$ ⊥ [100]<br>• $\boldsymbol{E}$ ∥ [100] and strain ⊥ [100]<br>• $\boldsymbol{E}$ ∥ [100] and $\boldsymbol{B}$ ∥ [010]
Pc (7.24) $\mathbb{Z}_1$	• $\boldsymbol{B}$ ∥ [001] and strain ⊥ [001]<br>• $\boldsymbol{E}$ ∥ [100] and strain ⊥ [001]<br>• $\boldsymbol{E}$ ∥ [010] and strain ⊥ [001]<br>• $\boldsymbol{E}$ ∥ [010] and $\boldsymbol{B}$ ∥ [001]<br>• $\boldsymbol{E}$ ⊥ [001]
Pc' (7.26) $\mathbb{Z}_1$	• $\boldsymbol{B}$ ∥ [100] and strain ⊥ [010]<br>• $\boldsymbol{B}$ ∥ [001] and strain ⊥ [010]<br>• $\boldsymbol{B}$ ⊥ [010]<br>• $\boldsymbol{E}$ ∥ [100] and strain ⊥ [010]<br>• $\boldsymbol{E}$ ∥ [100] and $\boldsymbol{B}$ ∥ [100]<br>• $\boldsymbol{E}$ ∥ [001] and strain ⊥ [010]<br>• $\boldsymbol{E}$ ∥ [001] and $\boldsymbol{B}$ ∥ [001]<br>• $\boldsymbol{E}$ ⊥ [010]
$Pn'c2'$ (30.113) $\mathbb{Z}_1$	• $\boldsymbol{B}$ ∥ [001]<br>• $\boldsymbol{E}$ ∥ [100]
Pc (7.24) $\mathbb{Z}_1$	• $\boldsymbol{B}$ ∥ [100] and strain ⊥ [100]<br>• $\boldsymbol{E}$ ∥ [010] and strain ⊥ [100]<br>• $\boldsymbol{E}$ ∥ [010] and $\boldsymbol{B}$ ∥ [100]<br>• $\boldsymbol{E}$ ∥ [001] and strain ⊥ [100]<br>• $\boldsymbol{E}$ ⊥ [100]
$P\bar{1}'$ (2.6) $\mathbb{Z}_1$	• strain in generic direction
$P2'/c$ (13.67) $\mathbb{Z}_1$	• strain ⊥ [100]

Continued on next page

...continued

Subgroup & SI group	Perturbation
$P2_1$ (4.7) $\mathbb{Z}_1$	• $\boldsymbol{B}$ ∥ [010] and strain ⊥ [010]<br>• $\boldsymbol{E}$ ∥ [010] and strain ⊥ [010]<br>• $\boldsymbol{E}$ ∥ [010] and $\boldsymbol{B}$ ∥ [010]
$Pna2_1$ (33.144) $\mathbb{Z}_1$	• $\boldsymbol{E}$ ∥ [010]
$P2_1/c'$ (14.78) $\mathbb{Z}_1$	• strain ⊥ [010]
$P2'$ (3.3) $\mathbb{Z}_1$	• $\boldsymbol{B}$ ∥ [100] and strain ⊥ [001]<br>• $\boldsymbol{B}$ ∥ [010] and strain ⊥ [001]<br>• $\boldsymbol{B}$ ⊥ [001]<br>• $\boldsymbol{E}$ ∥ [001] and strain ⊥ [001]<br>• $\boldsymbol{E}$ ∥ [001] and $\boldsymbol{B}$ ∥ [010]
$Pn'n2'$ (34.158) $\mathbb{Z}_1$	• $\boldsymbol{B}$ ∥ [100]<br>• $\boldsymbol{E}$ ∥ [001]
$P2'/c$ (13.67) $\mathbb{Z}_1$	• strain ⊥ [001]
$P2'2'2_1$ (17.9) $\mathbb{Z}_1$	• $\boldsymbol{B}$ ∥ [010]

Table. S 419: Subgroups of $Pnna'$ (52.309) that can be realized by applying $\boldsymbol{E}/\boldsymbol{B}$ fields and/or uniaxial strains.

Subgroup & SI group	Perturbation
$P2'$ (3.3) $\mathbb{Z}_1$	• $\boldsymbol{B}$ ∥ [010] and strain ⊥ [100]<br>• $\boldsymbol{B}$ ∥ [001] and strain ⊥ [100]<br>• $\boldsymbol{B}$ ⊥ [100]<br>• $\boldsymbol{E}$ ∥ [100] and strain ⊥ [100]<br>• $\boldsymbol{E}$ ∥ [100] and $\boldsymbol{B}$ ∥ [001]
Pc' (7.26) $\mathbb{Z}_1$	• $\boldsymbol{B}$ ∥ [100] and strain ⊥ [001]<br>• $\boldsymbol{B}$ ∥ [010] and strain ⊥ [001]<br>• $\boldsymbol{B}$ ⊥ [001]<br>• $\boldsymbol{E}$ ∥ [100] and strain ⊥ [001]<br>• $\boldsymbol{E}$ ∥ [100] and $\boldsymbol{B}$ ∥ [100]<br>• $\boldsymbol{E}$ ∥ [010] and strain ⊥ [001]<br>• $\boldsymbol{E}$ ∥ [010] and $\boldsymbol{B}$ ∥ [010]<br>• $\boldsymbol{E}$ ⊥ [001]

Continued on next page

. . . continued

Subgroup & SI group	Perturbation
Pc (7.24) $\mathbb{Z}_1$	• $\boldsymbol{B} \parallel [010]$ and strain $\perp [010]$<br>• $\boldsymbol{E} \parallel [100]$ and strain $\perp [010]$<br>• $\boldsymbol{E} \parallel [001]$ and strain $\perp [010]$<br>• $\boldsymbol{E} \parallel [001]$ and $\boldsymbol{B} \parallel [010]$<br>• $\boldsymbol{E} \perp [010]$
$Pnc'2'$ (30.114) $\mathbb{Z}_1$	• $\boldsymbol{B} \parallel [010]$<br>• $\boldsymbol{E} \parallel [100]$
Pc (7.24) $\mathbb{Z}_1$	• $\boldsymbol{B} \parallel [100]$ and strain $\perp [100]$<br>• $\boldsymbol{E} \parallel [010]$ and strain $\perp [100]$<br>• $\boldsymbol{E} \parallel [001]$ and strain $\perp [100]$<br>• $\boldsymbol{E} \parallel [001]$ and $\boldsymbol{B} \parallel [100]$<br>• $\boldsymbol{E} \perp [100]$
$P\bar{1}'$ (2.6) $\mathbb{Z}_1$	• strain in generic direction
$P2'/c$ (13.67) $\mathbb{Z}_1$	• strain $\perp [100]$
$P2_1'$ (4.9) $\mathbb{Z}_1$	• $\boldsymbol{B} \parallel [100]$ and strain $\perp [010]$<br>• $\boldsymbol{B} \parallel [001]$ and strain $\perp [010]$<br>• $\boldsymbol{B} \perp [010]$<br>• $\boldsymbol{E} \parallel [010]$ and strain $\perp [010]$<br>• $\boldsymbol{E} \parallel [010]$ and $\boldsymbol{B} \parallel [001]$
$Pna'2_1'$ (33.147) $\mathbb{Z}_1$	• $\boldsymbol{B} \parallel [100]$<br>• $\boldsymbol{E} \parallel [010]$
$P2_1'/c$ (14.77) $\mathbb{Z}_1$	• strain $\perp [010]$
$P2$ (3.1) $\mathbb{Z}_2$	• $\boldsymbol{B} \parallel [001]$ and strain $\perp [001]$<br>• $\boldsymbol{E} \parallel [001]$ and strain $\perp [001]$<br>• $\boldsymbol{E} \parallel [001]$ and $\boldsymbol{B} \parallel [001]$
$Pnn2$ (34.156) $\mathbb{Z}_1$	• $\boldsymbol{E} \parallel [001]$
$P2/c'$ (13.68) $\mathbb{Z}_2$	• strain $\perp [001]$
$P22'2_1'$ (17.10) $\mathbb{Z}_2$	• $\boldsymbol{B} \parallel [001]$

Table. S 420: Subgroups of $Pn'n'a$ (52.310) that can be realized by applying $\boldsymbol{E}/\boldsymbol{B}$ fields and/or uniaxial strains.

Subgroup & SI group	Perturbation
$P2'$ (3.3) $\mathbb{Z}_1$	• $\boldsymbol{E} \parallel [100]$ and strain $\perp [100]$<br>• $\boldsymbol{E} \parallel [100]$ and ($\boldsymbol{B} \parallel [010]$ or $\boldsymbol{B} \perp [100]$)
Pc (7.24) $\mathbb{Z}_1$	• $\boldsymbol{E} \parallel [100]$ and strain $\perp [001]$<br>• $\boldsymbol{E} \parallel [010]$ and strain $\perp [001]$<br>• $\boldsymbol{E} \perp [001]$
Pc' (7.26) $\mathbb{Z}_1$	• $\boldsymbol{E} \parallel [100]$ and strain $\perp [010]$<br>• $\boldsymbol{E} \parallel [100]$ and ($\boldsymbol{B} \parallel [100]$ or $\boldsymbol{B} \perp [010]$)<br>• $\boldsymbol{E} \parallel [001]$ and strain $\perp [010]$<br>• $\boldsymbol{E} \parallel [001]$ and ($\boldsymbol{B} \parallel [100]$ or $\boldsymbol{B} \perp [010]$)<br>• $\boldsymbol{E} \perp [010]$
$Pn'c2'$ (30.113) $\mathbb{Z}_1$	• $\boldsymbol{E} \parallel [100]$
Pc' (7.26) $\mathbb{Z}_1$	• $\boldsymbol{E} \parallel [010]$ and strain $\perp [100]$<br>• $\boldsymbol{E} \parallel [010]$ and ($\boldsymbol{B} \parallel [010]$ or $\boldsymbol{B} \perp [100]$)<br>• $\boldsymbol{E} \parallel [001]$ and strain $\perp [100]$<br>• $\boldsymbol{E} \parallel [001]$ and ($\boldsymbol{B} \parallel [010]$ or $\boldsymbol{B} \perp [100]$)<br>• $\boldsymbol{E} \perp [100]$
$P\bar{1}$ (2.4) $\mathbb{Z}_2 \times \mathbb{Z}_2 \times \mathbb{Z}_2 \times \mathbb{Z}_4$	• strain in generic direction<br>• ($\boldsymbol{B} \parallel [100]$ or $\boldsymbol{B} \perp [010]$) and strain $\perp [100]$<br>• ($\boldsymbol{B} \parallel [100]$ or $\boldsymbol{B} \perp [010]$) and strain $\perp [001]$<br>• ($\boldsymbol{B} \parallel [010]$ or $\boldsymbol{B} \perp [100]$) and strain $\perp [010]$<br>• ($\boldsymbol{B} \parallel [010]$ or $\boldsymbol{B} \perp [100]$) and strain $\perp [001]$<br>• $\boldsymbol{B}$ in generic direction
$P2'/c'$ (13.69) $\mathbb{Z}_2 \times \mathbb{Z}_2 \times \mathbb{Z}_4$	• strain $\perp [100]$<br>• ($\boldsymbol{B} \parallel [010]$ or $\boldsymbol{B} \perp [100]$)
$P2_1'$ (4.9) $\mathbb{Z}_1$	• $\boldsymbol{E} \parallel [010]$ and strain $\perp [010]$<br>• $\boldsymbol{E} \parallel [010]$ and ($\boldsymbol{B} \parallel [100]$ or $\boldsymbol{B} \perp [010]$)
$Pn'a2_1'$ (33.146) $\mathbb{Z}_1$	• $\boldsymbol{E} \parallel [010]$
$P2_1'/c'$ (14.79) $\mathbb{Z}_2 \times \mathbb{Z}_4$	• strain $\perp [010]$<br>• ($\boldsymbol{B} \parallel [100]$ or $\boldsymbol{B} \perp [010]$)
$P2$ (3.1) $\mathbb{Z}_2$	• $\boldsymbol{E} \parallel [001]$ and strain $\perp [001]$
$Pn'n'2$ (34.159) $\mathbb{Z}_1$	• $\boldsymbol{E} \parallel [001]$
$P2/c$ (13.65) $\mathbb{Z}_2 \times \mathbb{Z}_2$	• strain $\perp [001]$

Table. S 421: Subgroups of $Pnn'a'$ (52.311) that can be realized by applying $\boldsymbol{E}/\boldsymbol{B}$ fields and/or uniaxial strains.

Subgroup & SI group	Perturbation
$P2$ (3.1) $\mathbb{Z}_2$	• $\boldsymbol{E}$ ∥ [100] and strain ⊥ [100]
Pc' (7.26) $\mathbb{Z}_1$	• $\boldsymbol{E}$ ∥ [100] and strain ⊥ [001]<br>• $\boldsymbol{E}$ ∥ [100] and ($\boldsymbol{B}$ ∥ [010] or $\boldsymbol{B}$ ⊥ [001])<br>• $\boldsymbol{E}$ ∥ [010] and strain ⊥ [001]<br>• $\boldsymbol{E}$ ∥ [010] and ($\boldsymbol{B}$ ∥ [010] or $\boldsymbol{B}$ ⊥ [001])<br>• $\boldsymbol{E}$ ⊥ [001]
Pc' (7.26) $\mathbb{Z}_1$	• $\boldsymbol{E}$ ∥ [100] and strain ⊥ [010]<br>• $\boldsymbol{E}$ ∥ [100] and ($\boldsymbol{B}$ ∥ [001] or $\boldsymbol{B}$ ⊥ [010])<br>• $\boldsymbol{E}$ ∥ [001] and strain ⊥ [010]<br>• $\boldsymbol{E}$ ∥ [001] and ($\boldsymbol{B}$ ∥ [001] or $\boldsymbol{B}$ ⊥ [010])<br>• $\boldsymbol{E}$ ⊥ [010]
$Pn'c'2$ (30.115) $\mathbb{Z}_1$	• $\boldsymbol{E}$ ∥ [100]
Pc (7.24) $\mathbb{Z}_1$	• $\boldsymbol{E}$ ∥ [010] and strain ⊥ [100]<br>• $\boldsymbol{E}$ ∥ [001] and strain ⊥ [100]<br>• $\boldsymbol{E}$ ⊥ [100]
$P\bar{1}$ (2.4) $\mathbb{Z}_2 \times \mathbb{Z}_2 \times \mathbb{Z}_2 \times \mathbb{Z}_4$	• strain in generic direction<br>• ($\boldsymbol{B}$ ∥ [010] or $\boldsymbol{B}$ ⊥ [001]) and strain ⊥ [100]<br>• ($\boldsymbol{B}$ ∥ [010] or $\boldsymbol{B}$ ⊥ [001]) and strain ⊥ [010]<br>• ($\boldsymbol{B}$ ∥ [001] or $\boldsymbol{B}$ ⊥ [010]) and strain ⊥ [100]<br>• ($\boldsymbol{B}$ ∥ [001] or $\boldsymbol{B}$ ⊥ [010]) and strain ⊥ [001]<br>• $\boldsymbol{B}$ in generic direction
$P2/c$ (13.65) $\mathbb{Z}_2 \times \mathbb{Z}_2$	• strain ⊥ [100]
$P2_1'$ (4.9) $\mathbb{Z}_1$	• $\boldsymbol{E}$ ∥ [010] and strain ⊥ [010]<br>• $\boldsymbol{E}$ ∥ [010] and ($\boldsymbol{B}$ ∥ [001] or $\boldsymbol{B}$ ⊥ [010])
$Pna'2_1'$ (33.147) $\mathbb{Z}_1$	• $\boldsymbol{E}$ ∥ [010]
$P2_1'/c'$ (14.79) $\mathbb{Z}_2 \times \mathbb{Z}_4$	• strain ⊥ [010]<br>• ($\boldsymbol{B}$ ∥ [001] or $\boldsymbol{B}$ ⊥ [010])
$P2'$ (3.3) $\mathbb{Z}_1$	• $\boldsymbol{E}$ ∥ [001] and strain ⊥ [001]<br>• $\boldsymbol{E}$ ∥ [001] and ($\boldsymbol{B}$ ∥ [010] or $\boldsymbol{B}$ ⊥ [001])
$Pn'n2'$ (34.158) $\mathbb{Z}_1$	• $\boldsymbol{E}$ ∥ [001]
$P2'/c'$ (13.69) $\mathbb{Z}_2 \times \mathbb{Z}_2 \times \mathbb{Z}_4$	• strain ⊥ [001]<br>• ($\boldsymbol{B}$ ∥ [010] or $\boldsymbol{B}$ ⊥ [001])

Table. S 422: Subgroups of $Pn'na'$ (52.312) that can be realized by applying $\boldsymbol{E}/\boldsymbol{B}$ fields and/or uniaxial strains.

Subgroup & SI group	Perturbation
$P2'$ (3.3) $\mathbb{Z}_1$	• $\boldsymbol{E} \parallel [100]$ and strain $\perp [100]$<br>• $\boldsymbol{E} \parallel [100]$ and ($\boldsymbol{B} \parallel [001]$ or $\boldsymbol{B} \perp [100]$)
Pc' (7.26) $\mathbb{Z}_1$	• $\boldsymbol{E} \parallel [100]$ and strain $\perp [001]$<br>• $\boldsymbol{E} \parallel [100]$ and ($\boldsymbol{B} \parallel [100]$ or $\boldsymbol{B} \perp [001]$)<br>• $\boldsymbol{E} \parallel [010]$ and strain $\perp [001]$<br>• $\boldsymbol{E} \parallel [010]$ and ($\boldsymbol{B} \parallel [100]$ or $\boldsymbol{B} \perp [001]$)<br>• $\boldsymbol{E} \perp [001]$
Pc (7.24) $\mathbb{Z}_1$	• $\boldsymbol{E} \parallel [100]$ and strain $\perp [010]$<br>• $\boldsymbol{E} \parallel [001]$ and strain $\perp [010]$<br>• $\boldsymbol{E} \perp [010]$
$Pnc'2'$ (30.114) $\mathbb{Z}_1$	• $\boldsymbol{E} \parallel [100]$
Pc' (7.26) $\mathbb{Z}_1$	• $\boldsymbol{E} \parallel [010]$ and strain $\perp [100]$<br>• $\boldsymbol{E} \parallel [010]$ and ($\boldsymbol{B} \parallel [001]$ or $\boldsymbol{B} \perp [100]$)<br>• $\boldsymbol{E} \parallel [001]$ and strain $\perp [100]$<br>• $\boldsymbol{E} \parallel [001]$ and ($\boldsymbol{B} \parallel [001]$ or $\boldsymbol{B} \perp [100]$)<br>• $\boldsymbol{E} \perp [100]$
$P\bar{1}$ (2.4) $\mathbb{Z}_2 \times \mathbb{Z}_2 \times \mathbb{Z}_2 \times \mathbb{Z}_4$	• strain in generic direction<br>• ($\boldsymbol{B} \parallel [100]$ or $\boldsymbol{B} \perp [001]$) and strain $\perp [100]$<br>• ($\boldsymbol{B} \parallel [100]$ or $\boldsymbol{B} \perp [001]$) and strain $\perp [010]$<br>• ($\boldsymbol{B} \parallel [001]$ or $\boldsymbol{B} \perp [100]$) and strain $\perp [010]$<br>• ($\boldsymbol{B} \parallel [001]$ or $\boldsymbol{B} \perp [100]$) and strain $\perp [001]$<br>• $\boldsymbol{B}$ in generic direction
$P2'/c'$ (13.69) $\mathbb{Z}_2 \times \mathbb{Z}_2 \times \mathbb{Z}_4$	• strain $\perp [100]$<br>• ($\boldsymbol{B} \parallel [001]$ or $\boldsymbol{B} \perp [100]$)
$P2_1$ (4.7) $\mathbb{Z}_1$	• $\boldsymbol{E} \parallel [010]$ and strain $\perp [010]$
$Pn'a'2_1$ (33.148) $\mathbb{Z}_1$	• $\boldsymbol{E} \parallel [010]$
$P2_1/c$ (14.75) $\mathbb{Z}_2$	• strain $\perp [010]$
$P2'$ (3.3) $\mathbb{Z}_1$	• $\boldsymbol{E} \parallel [001]$ and strain $\perp [001]$<br>• $\boldsymbol{E} \parallel [001]$ and ($\boldsymbol{B} \parallel [100]$ or $\boldsymbol{B} \perp [001]$)
$Pn'n2'$ (34.158) $\mathbb{Z}_1$	• $\boldsymbol{E} \parallel [001]$
$P2'/c'$ (13.69) $\mathbb{Z}_2 \times \mathbb{Z}_2 \times \mathbb{Z}_4$	• strain $\perp [001]$<br>• ($\boldsymbol{B} \parallel [100]$ or $\boldsymbol{B} \perp [001]$)

Table. S 423: Subgroups of $Pn'n'a'$ (52.313) that can be realized by applying $\boldsymbol{E}/\boldsymbol{B}$ fields and/or uniaxial strains.

Subgroup & SI group	Perturbation
$P2$ (3.1) $\mathbb{Z}_2$	• $\boldsymbol{B} \parallel [100]$ and strain $\perp [100]$<br>• $\boldsymbol{E} \parallel [100]$ and strain $\perp [100]$
Pc' (7.26) $\mathbb{Z}_1$	• $\boldsymbol{B} \parallel [100]$ and strain $\perp [001]$<br>• $\boldsymbol{B} \parallel [010]$ and strain $\perp [001]$<br>• $\boldsymbol{B} \perp [001]$<br>• $\boldsymbol{E} \parallel [100]$ and strain $\perp [001]$<br>• $\boldsymbol{E} \parallel [100]$ and $\boldsymbol{B} \parallel [010]$<br>• $\boldsymbol{E} \parallel [010]$ and strain $\perp [001]$<br>• $\boldsymbol{E} \parallel [010]$ and $\boldsymbol{B} \parallel [100]$<br>• $\boldsymbol{E} \perp [001]$
Pc' (7.26) $\mathbb{Z}_1$	• $\boldsymbol{B} \parallel [100]$ and strain $\perp [010]$<br>• $\boldsymbol{B} \parallel [001]$ and strain $\perp [010]$<br>• $\boldsymbol{B} \perp [010]$<br>• $\boldsymbol{E} \parallel [100]$ and strain $\perp [010]$<br>• $\boldsymbol{E} \parallel [100]$ and $\boldsymbol{B} \parallel [001]$<br>• $\boldsymbol{E} \parallel [001]$ and strain $\perp [010]$<br>• $\boldsymbol{E} \parallel [001]$ and $\boldsymbol{B} \parallel [100]$<br>• $\boldsymbol{E} \perp [010]$
$Pn'c'2$ (30.115) $\mathbb{Z}_1$	• $\boldsymbol{B} \parallel [100]$<br>• $\boldsymbol{E} \parallel [100]$
Pc' (7.26) $\mathbb{Z}_1$	• $\boldsymbol{B} \parallel [010]$ and strain $\perp [100]$<br>• $\boldsymbol{B} \parallel [001]$ and strain $\perp [100]$<br>• $\boldsymbol{B} \perp [100]$<br>• $\boldsymbol{E} \parallel [010]$ and strain $\perp [100]$<br>• $\boldsymbol{E} \parallel [010]$ and $\boldsymbol{B} \parallel [001]$<br>• $\boldsymbol{E} \parallel [001]$ and strain $\perp [100]$<br>• $\boldsymbol{E} \parallel [001]$ and $\boldsymbol{B} \parallel [010]$<br>• $\boldsymbol{E} \perp [100]$
$P\bar{1}'$ (2.6) $\mathbb{Z}_1$	• strain in generic direction
$P2/c'$ (13.68) $\mathbb{Z}_2$	• strain $\perp [100]$
$P2_1$ (4.7) $\mathbb{Z}_1$	• $\boldsymbol{B} \parallel [010]$ and strain $\perp [010]$<br>• $\boldsymbol{E} \parallel [010]$ and strain $\perp [010]$
$Pn'a'2_1$ (33.148) $\mathbb{Z}_1$	• $\boldsymbol{B} \parallel [010]$<br>• $\boldsymbol{E} \parallel [010]$
$P2_1/c'$ (14.78) $\mathbb{Z}_1$	• strain $\perp [010]$

Continued on next page

. . . continued

Subgroup & SI group	Perturbation
$P2$ (3.1) $\mathbb{Z}_2$	• $\boldsymbol{B}$ ∥ [001] and strain ⊥ [001]<br>• $\boldsymbol{E}$ ∥ [001] and strain ⊥ [001]
$Pn'n'2$ (34.159) $\mathbb{Z}_1$	• $\boldsymbol{B}$ ∥ [001]<br>• $\boldsymbol{E}$ ∥ [001]
$P2/c'$ (13.68) $\mathbb{Z}_2$	• strain ⊥ [001]

Table. S 424: Subgroups of P_anna (52.314) that can be realized by applying $\boldsymbol{E}/\boldsymbol{B}$ fields and/or uniaxial strains.

Subgroup & SI group	Perturbation
Pm' (6.20) $\mathbb{Z}_1$	• $\boldsymbol{E}$ ∥ [100] and $\boldsymbol{B}$ ∥ [100] and strain ⊥ [001]<br>• $\boldsymbol{E}$ ∥ [100] and $\boldsymbol{B}$ ∥ [010] and strain ⊥ [001]<br>• $\boldsymbol{E}$ ∥ [100] and $\boldsymbol{B}$ ⊥ [001]<br>• $\boldsymbol{E}$ ∥ [010] and $\boldsymbol{B}$ ∥ [100] and strain ⊥ [001]<br>• $\boldsymbol{E}$ ∥ [010] and $\boldsymbol{B}$ ∥ [010] and strain ⊥ [001]<br>• $\boldsymbol{E}$ ∥ [010] and $\boldsymbol{B}$ ⊥ [001]<br>• $\boldsymbol{E}$ ⊥ [001] and $\boldsymbol{B}$ ∥ [100]<br>• $\boldsymbol{E}$ ⊥ [001] and $\boldsymbol{B}$ ∥ [010]<br>• $\boldsymbol{E}$ ⊥ [001] and $\boldsymbol{B}$ ⊥ [001]
Pc' (7.26) $\mathbb{Z}_1$	• $\boldsymbol{E}$ ∥ [100] and $\boldsymbol{B}$ ∥ [100] and strain ⊥ [010]<br>• $\boldsymbol{E}$ ∥ [100] and $\boldsymbol{B}$ ∥ [001] and strain ⊥ [010]<br>• $\boldsymbol{E}$ ∥ [100] and $\boldsymbol{B}$ ⊥ [010]<br>• $\boldsymbol{E}$ ∥ [001] and $\boldsymbol{B}$ ∥ [100] and strain ⊥ [010]<br>• $\boldsymbol{E}$ ∥ [001] and $\boldsymbol{B}$ ∥ [001] and strain ⊥ [010]<br>• $\boldsymbol{E}$ ∥ [001] and $\boldsymbol{B}$ ⊥ [010]<br>• $\boldsymbol{E}$ ⊥ [010] and $\boldsymbol{B}$ ∥ [100]<br>• $\boldsymbol{E}$ ⊥ [010] and $\boldsymbol{B}$ ∥ [001]<br>• $\boldsymbol{E}$ ⊥ [010] and $\boldsymbol{B}$ ⊥ [010]
$P2$ (3.1) $\mathbb{Z}_2$	• $\boldsymbol{E}$ ∥ [100] and $\boldsymbol{B}$ ∥ [100] and strain ⊥ [100]
$Pm'a'2$ (28.91) $\mathbb{Z}_2$	• $\boldsymbol{E}$ ∥ [100] and $\boldsymbol{B}$ ∥ [100]

Continued on next page

...continued

Subgroup & SI group	Perturbation
P_S1 (1.3) $\mathbb{Z}_1$	• $\boldsymbol{E}$ ∥ [100] and strain in generic direction<br>• $\boldsymbol{E}$ ∥ [010] and strain in generic direction<br>• $\boldsymbol{E}$ ∥ [001] and strain in generic direction<br>• $\boldsymbol{E} \perp$ [100] and strain $\perp$ [010]<br>• $\boldsymbol{E} \perp$ [100] and strain $\perp$ [001]<br>• $\boldsymbol{E} \perp$ [100] and strain in generic direction<br>• $\boldsymbol{E} \perp$ [010] and strain $\perp$ [100]<br>• $\boldsymbol{E} \perp$ [010] and strain $\perp$ [001]<br>• $\boldsymbol{E} \perp$ [010] and strain in generic direction<br>• $\boldsymbol{E} \perp$ [001] and strain $\perp$ [100]<br>• $\boldsymbol{E} \perp$ [001] and strain $\perp$ [010]<br>• $\boldsymbol{E} \perp$ [001] and strain in generic direction<br>• $\boldsymbol{E}$ in generic direction
Pc (7.24) $\mathbb{Z}_1$	• $\boldsymbol{E}$ ∥ [100] and $\boldsymbol{B}$ ∥ [001] and strain $\perp$ [001]<br>• $\boldsymbol{E}$ ∥ [010] and $\boldsymbol{B}$ ∥ [001] and strain $\perp$ [001]<br>• $\boldsymbol{E} \perp$ [001] and $\boldsymbol{B}$ ∥ [001]
P_cc (7.28) $\mathbb{Z}_1$	• $\boldsymbol{E}$ ∥ [100] and strain $\perp$ [001]<br>• $\boldsymbol{E}$ ∥ [010] and strain $\perp$ [001]<br>• $\boldsymbol{E} \perp$ [001]
Pc (7.24) $\mathbb{Z}_1$	• $\boldsymbol{E}$ ∥ [100] and $\boldsymbol{B}$ ∥ [010] and strain $\perp$ [010]<br>• $\boldsymbol{E}$ ∥ [001] and $\boldsymbol{B}$ ∥ [010] and strain $\perp$ [010]<br>• $\boldsymbol{E} \perp$ [010] and $\boldsymbol{B}$ ∥ [010]
P_ac (7.27) $\mathbb{Z}_1$	• $\boldsymbol{E}$ ∥ [100] and strain $\perp$ [010]<br>• $\boldsymbol{E}$ ∥ [001] and strain $\perp$ [010]<br>• $\boldsymbol{E} \perp$ [010]
$P2_1'$ (4.9) $\mathbb{Z}_1$	• $\boldsymbol{E}$ ∥ [100] and $\boldsymbol{B}$ ∥ [010] and strain $\perp$ [100]<br>• $\boldsymbol{E}$ ∥ [100] and $\boldsymbol{B}$ ∥ [001] and strain $\perp$ [100]<br>• $\boldsymbol{E}$ ∥ [100] and $\boldsymbol{B} \perp$ [100]
P_b2 (3.5) $\mathbb{Z}_2$	• $\boldsymbol{E}$ ∥ [100] and strain $\perp$ [100]
$Pca'2_1'$ (29.102) $\mathbb{Z}_1$	• $\boldsymbol{E}$ ∥ [100] and $\boldsymbol{B}$ ∥ [001]
$Pm'n2_1'$ (31.125) $\mathbb{Z}_1$	• $\boldsymbol{E}$ ∥ [100] and $\boldsymbol{B}$ ∥ [010]
P_cnc2 (30.118) $\mathbb{Z}_2$	• $\boldsymbol{E}$ ∥ [100]
Pc (7.24) $\mathbb{Z}_1$	• $\boldsymbol{E}$ ∥ [010] and $\boldsymbol{B}$ ∥ [100] and strain $\perp$ [100]<br>• $\boldsymbol{E}$ ∥ [001] and $\boldsymbol{B}$ ∥ [100] and strain $\perp$ [100]<br>• $\boldsymbol{E} \perp$ [100] and $\boldsymbol{B}$ ∥ [100]

Continued on next page

…continued

Subgroup & SI group	Perturbation
$P2_1'$ (4.9) $\mathbb{Z}_1$	• $\boldsymbol{E} \parallel [010]$ and $\boldsymbol{B} \parallel [100]$ and strain $\perp [010]$<br>• $\boldsymbol{E} \parallel [010]$ and $\boldsymbol{B} \parallel [001]$ and strain $\perp [010]$<br>• $\boldsymbol{E} \parallel [010]$ and $\boldsymbol{B} \perp [010]$
$Pm'n2_1'$ (31.125) $\mathbb{Z}_1$	• $\boldsymbol{E} \parallel [010]$ and $\boldsymbol{B} \parallel [100]$
$P2'$ (3.3) $\mathbb{Z}_1$	• $\boldsymbol{E} \parallel [001]$ and $\boldsymbol{B} \parallel [100]$ and strain $\perp [001]$<br>• $\boldsymbol{E} \parallel [001]$ and $\boldsymbol{B} \parallel [010]$ and strain $\perp [001]$<br>• $\boldsymbol{E} \parallel [001]$ and $\boldsymbol{B} \perp [001]$
$Pnc'2'$ (30.114) $\mathbb{Z}_1$	• $\boldsymbol{E} \parallel [001]$ and $\boldsymbol{B} \parallel [100]$
$P\bar{1}$ (2.4) $\mathbb{Z}_2 \times \mathbb{Z}_2 \times \mathbb{Z}_2 \times \mathbb{Z}_4$	• $\boldsymbol{B} \parallel [100]$ and strain in generic direction<br>• $\boldsymbol{B} \parallel [010]$ and strain in generic direction<br>• $\boldsymbol{B} \parallel [001]$ and strain in generic direction<br>• $\boldsymbol{B} \perp [100]$ and strain $\perp [010]$<br>• $\boldsymbol{B} \perp [100]$ and strain $\perp [001]$<br>• $\boldsymbol{B} \perp [100]$ and strain in generic direction<br>• $\boldsymbol{B} \perp [010]$ and strain $\perp [100]$<br>• $\boldsymbol{B} \perp [010]$ and strain $\perp [001]$<br>• $\boldsymbol{B} \perp [010]$ and strain in generic direction<br>• $\boldsymbol{B} \perp [001]$ and strain $\perp [100]$<br>• $\boldsymbol{B} \perp [001]$ and strain $\perp [010]$<br>• $\boldsymbol{B} \perp [001]$ and strain in generic direction<br>• $\boldsymbol{B}$ in generic direction
$P2/c$ (13.65) $\mathbb{Z}_2 \times \mathbb{Z}_2$	• $\boldsymbol{B} \parallel [100]$ and strain $\perp [100]$
$P2_1'/c'$ (14.79) $\mathbb{Z}_2 \times \mathbb{Z}_4$	• $\boldsymbol{B} \parallel [100]$ and strain $\perp [010]$<br>• $\boldsymbol{B} \parallel [001]$ and strain $\perp [010]$<br>• $\boldsymbol{B} \perp [010]$
$P2'/m'$ (10.46) $\mathbb{Z}_2 \times \mathbb{Z}_2 \times \mathbb{Z}_2 \times \mathbb{Z}_4$	• $\boldsymbol{B} \parallel [100]$ and strain $\perp [001]$<br>• $\boldsymbol{B} \parallel [010]$ and strain $\perp [001]$<br>• $\boldsymbol{B} \perp [001]$
$Pm'na'$ (53.328) $\mathbb{Z}_2 \times \mathbb{Z}_2$	• $\boldsymbol{B} \parallel [100]$

Continued on next page

…continued

Subgroup & SI group	Perturbation
Pc' (7.26) $\mathbb{Z}_1$	• $\boldsymbol{E} \parallel [010]$ and $\boldsymbol{B} \parallel [010]$ and strain $\perp [100]$<br>• $\boldsymbol{E} \parallel [010]$ and $\boldsymbol{B} \parallel [001]$ and strain $\perp [100]$<br>• $\boldsymbol{E} \parallel [010]$ and $\boldsymbol{B} \perp [100]$<br>• $\boldsymbol{E} \parallel [001]$ and $\boldsymbol{B} \parallel [010]$ and strain $\perp [100]$<br>• $\boldsymbol{E} \parallel [001]$ and $\boldsymbol{B} \parallel [001]$ and strain $\perp [100]$<br>• $\boldsymbol{E} \parallel [001]$ and $\boldsymbol{B} \perp [100]$<br>• $\boldsymbol{E} \perp [100]$ and $\boldsymbol{B} \parallel [010]$<br>• $\boldsymbol{E} \perp [100]$ and $\boldsymbol{B} \parallel [001]$<br>• $\boldsymbol{E} \perp [100]$ and $\boldsymbol{B} \perp [100]$
P_bc (7.29) $\mathbb{Z}_1$	• $\boldsymbol{E} \parallel [010]$ and strain $\perp [100]$<br>• $\boldsymbol{E} \parallel [001]$ and strain $\perp [100]$<br>• $\boldsymbol{E} \perp [100]$
$Pn'a2_1'$ (33.146) $\mathbb{Z}_1$	• $\boldsymbol{E} \parallel [010]$ and $\boldsymbol{B} \parallel [001]$
$Pn'n2'$ (34.158) $\mathbb{Z}_1$	• $\boldsymbol{E} \parallel [001]$ and $\boldsymbol{B} \parallel [010]$
$P2_1'/c'$ (14.79) $\mathbb{Z}_2 \times \mathbb{Z}_4$	• $\boldsymbol{B} \parallel [010]$ and strain $\perp [100]$<br>• $\boldsymbol{B} \parallel [001]$ and strain $\perp [100]$<br>• $\boldsymbol{B} \perp [100]$
$P2_1$ (4.7) $\mathbb{Z}_1$	• $\boldsymbol{E} \parallel [010]$ and $\boldsymbol{B} \parallel [010]$ and strain $\perp [010]$
P_a2_1 (4.10) $\mathbb{Z}_1$	• $\boldsymbol{E} \parallel [010]$ and strain $\perp [010]$
$P2_1/c$ (14.75) $\mathbb{Z}_2$	• $\boldsymbol{B} \parallel [010]$ and strain $\perp [010]$
$Pm'n'2_1$ (31.127) $\mathbb{Z}_1$	• $\boldsymbol{E} \parallel [010]$ and $\boldsymbol{B} \parallel [010]$
P_ana2_1 (33.149) $\mathbb{Z}_1$	• $\boldsymbol{E} \parallel [010]$
$Pnn'm'$ (58.398) $\mathbb{Z}_2$	• $\boldsymbol{B} \parallel [010]$
$P2$ (3.1) $\mathbb{Z}_2$	• $\boldsymbol{E} \parallel [001]$ and $\boldsymbol{B} \parallel [001]$ and strain $\perp [001]$
P_a2 (3.4) $\mathbb{Z}_2$	• $\boldsymbol{E} \parallel [001]$ and strain $\perp [001]$
$P2/c$ (13.65) $\mathbb{Z}_2 \times \mathbb{Z}_2$	• $\boldsymbol{B} \parallel [001]$ and strain $\perp [001]$
$Pn'c'2$ (30.115) $\mathbb{Z}_1$	• $\boldsymbol{E} \parallel [001]$ and $\boldsymbol{B} \parallel [001]$
P_ann2 (34.160) $\mathbb{Z}_1$	• $\boldsymbol{E} \parallel [001]$
$Pb'cn'$ (60.424) $\mathbb{Z}_2 \times \mathbb{Z}_2$	• $\boldsymbol{B} \parallel [001]$
$P_S\bar{1}$ (2.7) $\mathbb{Z}_2 \times \mathbb{Z}_2 \times \mathbb{Z}_4$	• strain in generic direction
P_b2/c (13.71) $\mathbb{Z}_2 \times \mathbb{Z}_2$	• strain $\perp [100]$
P_a2_1/c (14.80) $\mathbb{Z}_2$	• strain $\perp [010]$
P_c2/c (13.72) $\mathbb{Z}_2 \times \mathbb{Z}_2$	• strain $\perp [001]$

Table. S 425: Subgroups of P_bnna (52.315) that can be realized by applying $\boldsymbol{E}/\boldsymbol{B}$ fields and/or uniaxial strains.

Subgroup & SI group	Perturbation
P_S1 (1.3) $\mathbb{Z}_1$	• $\boldsymbol{E}$ ∥ [100] and strain in generic direction<br>• $\boldsymbol{E}$ ∥ [010] and strain in generic direction<br>• $\boldsymbol{E}$ ∥ [001] and strain in generic direction<br>• $\boldsymbol{E}$ ⊥ [100] and strain ⊥ [010]<br>• $\boldsymbol{E}$ ⊥ [100] and strain ⊥ [001]<br>• $\boldsymbol{E}$ ⊥ [100] and strain in generic direction<br>• $\boldsymbol{E}$ ⊥ [010] and strain ⊥ [100]<br>• $\boldsymbol{E}$ ⊥ [010] and strain ⊥ [001]<br>• $\boldsymbol{E}$ ⊥ [010] and strain in generic direction<br>• $\boldsymbol{E}$ ⊥ [001] and strain ⊥ [100]<br>• $\boldsymbol{E}$ ⊥ [001] and strain ⊥ [010]<br>• $\boldsymbol{E}$ ⊥ [001] and strain in generic direction<br>• $\boldsymbol{E}$ in generic direction
$P2'$ (3.3) $\mathbb{Z}_1$	• $\boldsymbol{E}$ ∥ [100] and $\boldsymbol{B}$ ∥ [010] and strain ⊥ [100]<br>• $\boldsymbol{E}$ ∥ [100] and $\boldsymbol{B}$ ∥ [001] and strain ⊥ [100]<br>• $\boldsymbol{E}$ ∥ [100] and $\boldsymbol{B}$ ⊥ [100]
$P2$ (3.1) $\mathbb{Z}_2$	• $\boldsymbol{E}$ ∥ [100] and $\boldsymbol{B}$ ∥ [100] and strain ⊥ [100]
P_a2 (3.4) $\mathbb{Z}_2$	• $\boldsymbol{E}$ ∥ [100] and strain ⊥ [100]
Pc (7.24) $\mathbb{Z}_1$	• $\boldsymbol{E}$ ∥ [100] and $\boldsymbol{B}$ ∥ [001] and strain ⊥ [001]<br>• $\boldsymbol{E}$ ∥ [010] and $\boldsymbol{B}$ ∥ [001] and strain ⊥ [001]<br>• $\boldsymbol{E}$ ⊥ [001] and $\boldsymbol{B}$ ∥ [001]
Pc' (7.26) $\mathbb{Z}_1$	• $\boldsymbol{E}$ ∥ [100] and $\boldsymbol{B}$ ∥ [100] and strain ⊥ [001]<br>• $\boldsymbol{E}$ ∥ [100] and $\boldsymbol{B}$ ∥ [010] and strain ⊥ [001]<br>• $\boldsymbol{E}$ ∥ [100] and $\boldsymbol{B}$ ⊥ [001]<br>• $\boldsymbol{E}$ ∥ [010] and $\boldsymbol{B}$ ∥ [100] and strain ⊥ [001]<br>• $\boldsymbol{E}$ ∥ [010] and $\boldsymbol{B}$ ∥ [010] and strain ⊥ [001]<br>• $\boldsymbol{E}$ ∥ [010] and $\boldsymbol{B}$ ⊥ [001]<br>• $\boldsymbol{E}$ ⊥ [001] and $\boldsymbol{B}$ ∥ [100]<br>• $\boldsymbol{E}$ ⊥ [001] and $\boldsymbol{B}$ ∥ [010]<br>• $\boldsymbol{E}$ ⊥ [001] and $\boldsymbol{B}$ ⊥ [001]
P_ac (7.27) $\mathbb{Z}_1$	• $\boldsymbol{E}$ ∥ [100] and strain ⊥ [001]<br>• $\boldsymbol{E}$ ∥ [010] and strain ⊥ [001]<br>• $\boldsymbol{E}$ ⊥ [001]

Continued on next page

. . . continued

Subgroup & SI group	Perturbation
Pc' (7.26) $\mathbb{Z}_1$	• $\boldsymbol{E}$ || [100] and $\boldsymbol{B}$ || [100] and strain ⊥ [010]<br>• $\boldsymbol{E}$ || [100] and $\boldsymbol{B}$ || [001] and strain ⊥ [010]<br>• $\boldsymbol{E}$ || [100] and $\boldsymbol{B}$ ⊥ [010]<br>• $\boldsymbol{E}$ || [001] and $\boldsymbol{B}$ || [100] and strain ⊥ [010]<br>• $\boldsymbol{E}$ || [001] and $\boldsymbol{B}$ || [001] and strain ⊥ [010]<br>• $\boldsymbol{E}$ || [001] and $\boldsymbol{B}$ ⊥ [010]<br>• $\boldsymbol{E}$ ⊥ [010] and $\boldsymbol{B}$ || [100]<br>• $\boldsymbol{E}$ ⊥ [010] and $\boldsymbol{B}$ || [001]<br>• $\boldsymbol{E}$ ⊥ [010] and $\boldsymbol{B}$ ⊥ [010]
$Pn'c2'$ (30.113) $\mathbb{Z}_1$	• $\boldsymbol{E}$ || [100] and $\boldsymbol{B}$ || [001]
$Pn'n'2$ (34.159) $\mathbb{Z}_1$	• $\boldsymbol{E}$ || [100] and $\boldsymbol{B}$ || [100]
Pc (7.24) $\mathbb{Z}_1$	• $\boldsymbol{E}$ || [100] and $\boldsymbol{B}$ || [010] and strain ⊥ [010]<br>• $\boldsymbol{E}$ || [001] and $\boldsymbol{B}$ || [010] and strain ⊥ [010]<br>• $\boldsymbol{E}$ ⊥ [010] and $\boldsymbol{B}$ || [010]
$Pn'n2'$ (34.158) $\mathbb{Z}_1$	• $\boldsymbol{E}$ || [100] and $\boldsymbol{B}$ || [010]
P_bc (7.29) $\mathbb{Z}_1$	• $\boldsymbol{E}$ || [100] and strain ⊥ [010]<br>• $\boldsymbol{E}$ || [001] and strain ⊥ [010]<br>• $\boldsymbol{E}$ ⊥ [010]
P_anc2 (30.116) $\mathbb{Z}_1$	• $\boldsymbol{E}$ || [100]
Pc' (7.26) $\mathbb{Z}_1$	• $\boldsymbol{E}$ || [010] and $\boldsymbol{B}$ || [010] and strain ⊥ [100]<br>• $\boldsymbol{E}$ || [010] and $\boldsymbol{B}$ || [001] and strain ⊥ [100]<br>• $\boldsymbol{E}$ || [010] and $\boldsymbol{B}$ ⊥ [100]<br>• $\boldsymbol{E}$ || [001] and $\boldsymbol{B}$ || [010] and strain ⊥ [100]<br>• $\boldsymbol{E}$ || [001] and $\boldsymbol{B}$ || [001] and strain ⊥ [100]<br>• $\boldsymbol{E}$ || [001] and $\boldsymbol{B}$ ⊥ [100]<br>• $\boldsymbol{E}$ ⊥ [100] and $\boldsymbol{B}$ || [010]<br>• $\boldsymbol{E}$ ⊥ [100] and $\boldsymbol{B}$ || [001]<br>• $\boldsymbol{E}$ ⊥ [100] and $\boldsymbol{B}$ ⊥ [100]
Pc (7.24) $\mathbb{Z}_1$	• $\boldsymbol{E}$ || [010] and $\boldsymbol{B}$ || [100] and strain ⊥ [100]<br>• $\boldsymbol{E}$ || [001] and $\boldsymbol{B}$ || [100] and strain ⊥ [100]<br>• $\boldsymbol{E}$ ⊥ [100] and $\boldsymbol{B}$ || [100]
P_ac (7.27) $\mathbb{Z}_1$	• $\boldsymbol{E}$ || [010] and strain ⊥ [100]<br>• $\boldsymbol{E}$ || [001] and strain ⊥ [100]<br>• $\boldsymbol{E}$ ⊥ [100]

Continued on next page

...continued

Subgroup & SI group	Perturbation
$P\bar{1}$ (2.4) $\mathbb{Z}_2 \times \mathbb{Z}_2 \times \mathbb{Z}_2 \times \mathbb{Z}_4$	• $\boldsymbol{B} \parallel [100]$ and strain in generic direction<br>• $\boldsymbol{B} \parallel [010]$ and strain in generic direction<br>• $\boldsymbol{B} \parallel [001]$ and strain in generic direction<br>• $\boldsymbol{B} \perp [100]$ and strain $\perp [010]$<br>• $\boldsymbol{B} \perp [100]$ and strain $\perp [001]$<br>• $\boldsymbol{B} \perp [100]$ and strain in generic direction<br>• $\boldsymbol{B} \perp [010]$ and strain $\perp [100]$<br>• $\boldsymbol{B} \perp [010]$ and strain $\perp [001]$<br>• $\boldsymbol{B} \perp [010]$ and strain in generic direction<br>• $\boldsymbol{B} \perp [001]$ and strain $\perp [100]$<br>• $\boldsymbol{B} \perp [001]$ and strain $\perp [010]$<br>• $\boldsymbol{B} \perp [001]$ and strain in generic direction<br>• $\boldsymbol{B}$ in generic direction
$P2'/c'$ (13.69) $\mathbb{Z}_2 \times \mathbb{Z}_2 \times \mathbb{Z}_4$	• $\boldsymbol{B} \parallel [010]$ and strain $\perp [100]$<br>• $\boldsymbol{B} \parallel [001]$ and strain $\perp [100]$<br>• $\boldsymbol{B} \perp [100]$
$P2/c$ (13.65) $\mathbb{Z}_2 \times \mathbb{Z}_2$	• $\boldsymbol{B} \parallel [100]$ and strain $\perp [100]$
$P_S\bar{1}$ (2.7) $\mathbb{Z}_2 \times \mathbb{Z}_2 \times \mathbb{Z}_4$	• strain in generic direction
P_a2/c (13.70) $\mathbb{Z}_2 \times \mathbb{Z}_2$	• strain $\perp [100]$
$P2'$ (3.3) $\mathbb{Z}_1$	• $\boldsymbol{E} \parallel [010]$ and $\boldsymbol{B} \parallel [100]$ and strain $\perp [010]$<br>• $\boldsymbol{E} \parallel [010]$ and $\boldsymbol{B} \parallel [001]$ and strain $\perp [010]$<br>• $\boldsymbol{E} \parallel [010]$ and $\boldsymbol{B} \perp [010]$
$Pb'a2'$ (32.137) $\mathbb{Z}_1$	• $\boldsymbol{E} \parallel [010]$ and $\boldsymbol{B} \parallel [001]$
$Pn'n2'$ (34.158) $\mathbb{Z}_1$	• $\boldsymbol{E} \parallel [010]$ and $\boldsymbol{B} \parallel [100]$
$P2'/c'$ (13.69) $\mathbb{Z}_2 \times \mathbb{Z}_2 \times \mathbb{Z}_4$	• $\boldsymbol{B} \parallel [100]$ and strain $\perp [010]$<br>• $\boldsymbol{B} \parallel [001]$ and strain $\perp [010]$<br>• $\boldsymbol{B} \perp [010]$
$P2_1$ (4.7) $\mathbb{Z}_1$	• $\boldsymbol{E} \parallel [010]$ and $\boldsymbol{B} \parallel [010]$ and strain $\perp [010]$
$Pn'a'2_1$ (33.148) $\mathbb{Z}_1$	• $\boldsymbol{E} \parallel [010]$ and $\boldsymbol{B} \parallel [010]$
$P2_1/c$ (14.75) $\mathbb{Z}_2$	• $\boldsymbol{B} \parallel [010]$ and strain $\perp [010]$
P_b2_1 (4.11) $\mathbb{Z}_1$	• $\boldsymbol{E} \parallel [010]$ and strain $\perp [010]$
P_cna2_1 (33.151) $\mathbb{Z}_1$	• $\boldsymbol{E} \parallel [010]$
P_b2_1/c (14.81) $\mathbb{Z}_2$	• strain $\perp [010]$
$P2$ (3.1) $\mathbb{Z}_2$	• $\boldsymbol{E} \parallel [001]$ and $\boldsymbol{B} \parallel [001]$ and strain $\perp [001]$

Continued on next page

. . . continued

Subgroup & SI group	Perturbation
$Pn'c'2$ (30.115) $\mathbb{Z}_1$	• $\boldsymbol{E}$ ∥ [001] and $\boldsymbol{B}$ ∥ [001]
$P2/c$ (13.65) $\mathbb{Z}_2 \times \mathbb{Z}_2$	• $\boldsymbol{B}$ ∥ [001] and strain ⊥ [001]
$Pb'an'$ (50.282) $\mathbb{Z}_2$	• $\boldsymbol{B}$ ∥ [001]
$P2'$ (3.3) $\mathbb{Z}_1$	• $\boldsymbol{E}$ ∥ [001] and $\boldsymbol{B}$ ∥ [100] and strain ⊥ [001]<br>• $\boldsymbol{E}$ ∥ [001] and $\boldsymbol{B}$ ∥ [010] and strain ⊥ [001]<br>• $\boldsymbol{E}$ ∥ [001] and $\boldsymbol{B}$ ⊥ [001]
$Pnc'2'$ (30.114) $\mathbb{Z}_1$	• $\boldsymbol{E}$ ∥ [001] and $\boldsymbol{B}$ ∥ [010]
$Pn'n2'$ (34.158) $\mathbb{Z}_1$	• $\boldsymbol{E}$ ∥ [001] and $\boldsymbol{B}$ ∥ [100]
$P2'/c'$ (13.69) $\mathbb{Z}_2 \times \mathbb{Z}_2 \times \mathbb{Z}_4$	• $\boldsymbol{B}$ ∥ [100] and strain ⊥ [001]<br>• $\boldsymbol{B}$ ∥ [010] and strain ⊥ [001]<br>• $\boldsymbol{B}$ ⊥ [001]
$Pn'n'n$ (48.260) $\mathbb{Z}_2$	• $\boldsymbol{B}$ ∥ [100]
$Pn'na'$ (52.312) $\mathbb{Z}_2$	• $\boldsymbol{B}$ ∥ [010]
P_a2 (3.4) $\mathbb{Z}_2$	• $\boldsymbol{E}$ ∥ [001] and strain ⊥ [001]
P_ann2 (34.160) $\mathbb{Z}_1$	• $\boldsymbol{E}$ ∥ [001]
P_a2/c (13.70) $\mathbb{Z}_2 \times \mathbb{Z}_2$	• strain ⊥ [001]

Table. S 426: Subgroups of P_cnna (52.316) that can be realized by applying $\boldsymbol{E}/\boldsymbol{B}$ fields and/or uniaxial strains.

Subgroup & SI group	Perturbation

Continued on next page

...continued

Subgroup & SI group	Perturbation
P_S1 (1.3) $\mathbb{Z}_1$	• $\boldsymbol{E} \parallel [100]$ and strain in generic direction<br>• $\boldsymbol{E} \parallel [010]$ and strain in generic direction<br>• $\boldsymbol{E} \parallel [001]$ and strain in generic direction<br>• $\boldsymbol{E} \perp [100]$ and strain $\perp [010]$<br>• $\boldsymbol{E} \perp [100]$ and strain $\perp [001]$<br>• $\boldsymbol{E} \perp [100]$ and strain in generic direction<br>• $\boldsymbol{E} \perp [010]$ and strain $\perp [100]$<br>• $\boldsymbol{E} \perp [010]$ and strain $\perp [001]$<br>• $\boldsymbol{E} \perp [010]$ and strain in generic direction<br>• $\boldsymbol{E} \perp [001]$ and strain $\perp [100]$<br>• $\boldsymbol{E} \perp [001]$ and strain $\perp [010]$<br>• $\boldsymbol{E} \perp [001]$ and strain in generic direction<br>• $\boldsymbol{E}$ in generic direction
$P2'$ (3.3) $\mathbb{Z}_1$	• $\boldsymbol{E} \parallel [100]$ and $\boldsymbol{B} \parallel [010]$ and strain $\perp [100]$<br>• $\boldsymbol{E} \parallel [100]$ and $\boldsymbol{B} \parallel [001]$ and strain $\perp [100]$<br>• $\boldsymbol{E} \parallel [100]$ and $\boldsymbol{B} \perp [100]$
$P2$ (3.1) $\mathbb{Z}_2$	• $\boldsymbol{E} \parallel [100]$ and $\boldsymbol{B} \parallel [100]$ and strain $\perp [100]$
P_a2 (3.4) $\mathbb{Z}_2$	• $\boldsymbol{E} \parallel [100]$ and strain $\perp [100]$
Pc (7.24) $\mathbb{Z}_1$	• $\boldsymbol{E} \parallel [100]$ and $\boldsymbol{B} \parallel [001]$ and strain $\perp [001]$<br>• $\boldsymbol{E} \parallel [010]$ and $\boldsymbol{B} \parallel [001]$ and strain $\perp [001]$<br>• $\boldsymbol{E} \perp [001]$ and $\boldsymbol{B} \parallel [001]$
Pc' (7.26) $\mathbb{Z}_1$	• $\boldsymbol{E} \parallel [100]$ and $\boldsymbol{B} \parallel [100]$ and strain $\perp [001]$<br>• $\boldsymbol{E} \parallel [100]$ and $\boldsymbol{B} \parallel [010]$ and strain $\perp [001]$<br>• $\boldsymbol{E} \parallel [100]$ and $\boldsymbol{B} \perp [001]$<br>• $\boldsymbol{E} \parallel [010]$ and $\boldsymbol{B} \parallel [100]$ and strain $\perp [001]$<br>• $\boldsymbol{E} \parallel [010]$ and $\boldsymbol{B} \parallel [010]$ and strain $\perp [001]$<br>• $\boldsymbol{E} \parallel [010]$ and $\boldsymbol{B} \perp [001]$<br>• $\boldsymbol{E} \perp [001]$ and $\boldsymbol{B} \parallel [100]$<br>• $\boldsymbol{E} \perp [001]$ and $\boldsymbol{B} \parallel [010]$<br>• $\boldsymbol{E} \perp [001]$ and $\boldsymbol{B} \perp [001]$
P_bc (7.29) $\mathbb{Z}_1$	• $\boldsymbol{E} \parallel [100]$ and strain $\perp [001]$<br>• $\boldsymbol{E} \parallel [010]$ and strain $\perp [001]$<br>• $\boldsymbol{E} \perp [001]$

Continued on next page

. . . continued

Subgroup & SI group	Perturbation
Pc' (7.26) $\mathbb{Z}_1$	• $\boldsymbol{E} \parallel [100]$ and $\boldsymbol{B} \parallel [100]$ and strain $\perp [010]$<br>• $\boldsymbol{E} \parallel [100]$ and $\boldsymbol{B} \parallel [001]$ and strain $\perp [010]$<br>• $\boldsymbol{E} \parallel [100]$ and $\boldsymbol{B} \perp [010]$<br>• $\boldsymbol{E} \parallel [001]$ and $\boldsymbol{B} \parallel [100]$ and strain $\perp [010]$<br>• $\boldsymbol{E} \parallel [001]$ and $\boldsymbol{B} \parallel [001]$ and strain $\perp [010]$<br>• $\boldsymbol{E} \parallel [001]$ and $\boldsymbol{B} \perp [010]$<br>• $\boldsymbol{E} \perp [010]$ and $\boldsymbol{B} \parallel [100]$<br>• $\boldsymbol{E} \perp [010]$ and $\boldsymbol{B} \parallel [001]$<br>• $\boldsymbol{E} \perp [010]$ and $\boldsymbol{B} \perp [010]$
$Pc'c2'$ (27.80) $\mathbb{Z}_1$	• $\boldsymbol{E} \parallel [100]$ and $\boldsymbol{B} \parallel [001]$
$Pc'c'2$ (27.81) $\mathbb{Z}_2$	• $\boldsymbol{E} \parallel [100]$ and $\boldsymbol{B} \parallel [100]$
Pc (7.24) $\mathbb{Z}_1$	• $\boldsymbol{E} \parallel [100]$ and $\boldsymbol{B} \parallel [010]$ and strain $\perp [010]$<br>• $\boldsymbol{E} \parallel [001]$ and $\boldsymbol{B} \parallel [010]$ and strain $\perp [010]$<br>• $\boldsymbol{E} \perp [010]$ and $\boldsymbol{B} \parallel [010]$
$Pnc'2'$ (30.114) $\mathbb{Z}_1$	• $\boldsymbol{E} \parallel [100]$ and $\boldsymbol{B} \parallel [010]$
P_ac (7.27) $\mathbb{Z}_1$	• $\boldsymbol{E} \parallel [100]$ and strain $\perp [010]$<br>• $\boldsymbol{E} \parallel [001]$ and strain $\perp [010]$<br>• $\boldsymbol{E} \perp [010]$
P_bnc2 (30.117) $\mathbb{Z}_2$	• $\boldsymbol{E} \parallel [100]$
Pc' (7.26) $\mathbb{Z}_1$	• $\boldsymbol{E} \parallel [010]$ and $\boldsymbol{B} \parallel [010]$ and strain $\perp [100]$<br>• $\boldsymbol{E} \parallel [010]$ and $\boldsymbol{B} \parallel [001]$ and strain $\perp [100]$<br>• $\boldsymbol{E} \parallel [010]$ and $\boldsymbol{B} \perp [100]$<br>• $\boldsymbol{E} \parallel [001]$ and $\boldsymbol{B} \parallel [010]$ and strain $\perp [100]$<br>• $\boldsymbol{E} \parallel [001]$ and $\boldsymbol{B} \parallel [001]$ and strain $\perp [100]$<br>• $\boldsymbol{E} \parallel [001]$ and $\boldsymbol{B} \perp [100]$<br>• $\boldsymbol{E} \perp [100]$ and $\boldsymbol{B} \parallel [010]$<br>• $\boldsymbol{E} \perp [100]$ and $\boldsymbol{B} \parallel [001]$<br>• $\boldsymbol{E} \perp [100]$ and $\boldsymbol{B} \perp [100]$
Pc (7.24) $\mathbb{Z}_1$	• $\boldsymbol{E} \parallel [010]$ and $\boldsymbol{B} \parallel [100]$ and strain $\perp [100]$<br>• $\boldsymbol{E} \parallel [001]$ and $\boldsymbol{B} \parallel [100]$ and strain $\perp [100]$<br>• $\boldsymbol{E} \perp [100]$ and $\boldsymbol{B} \parallel [100]$
P_ac (7.27) $\mathbb{Z}_1$	• $\boldsymbol{E} \parallel [010]$ and strain $\perp [100]$<br>• $\boldsymbol{E} \parallel [001]$ and strain $\perp [100]$<br>• $\boldsymbol{E} \perp [100]$

Continued on next page

...continued

Subgroup & SI group	Perturbation
$P\bar{1}$ (2.4) $\mathbb{Z}_2 \times \mathbb{Z}_2 \times \mathbb{Z}_2 \times \mathbb{Z}_4$	• $\boldsymbol{B} \parallel [100]$ and strain in generic direction<br>• $\boldsymbol{B} \parallel [010]$ and strain in generic direction<br>• $\boldsymbol{B} \parallel [001]$ and strain in generic direction<br>• $\boldsymbol{B} \perp [100]$ and strain $\perp [010]$<br>• $\boldsymbol{B} \perp [100]$ and strain $\perp [001]$<br>• $\boldsymbol{B} \perp [100]$ and strain in generic direction<br>• $\boldsymbol{B} \perp [010]$ and strain $\perp [100]$<br>• $\boldsymbol{B} \perp [010]$ and strain $\perp [001]$<br>• $\boldsymbol{B} \perp [010]$ and strain in generic direction<br>• $\boldsymbol{B} \perp [001]$ and strain $\perp [100]$<br>• $\boldsymbol{B} \perp [001]$ and strain $\perp [010]$<br>• $\boldsymbol{B} \perp [001]$ and strain in generic direction<br>• $\boldsymbol{B}$ in generic direction
$P2'/c'$ (13.69) $\mathbb{Z}_2 \times \mathbb{Z}_2 \times \mathbb{Z}_4$	• $\boldsymbol{B} \parallel [010]$ and strain $\perp [100]$<br>• $\boldsymbol{B} \parallel [001]$ and strain $\perp [100]$<br>• $\boldsymbol{B} \perp [100]$
$P2/c$ (13.65) $\mathbb{Z}_2 \times \mathbb{Z}_2$	• $\boldsymbol{B} \parallel [100]$ and strain $\perp [100]$
$P_S\bar{1}$ (2.7) $\mathbb{Z}_2 \times \mathbb{Z}_2 \times \mathbb{Z}_4$	• strain in generic direction
P_a2/c (13.70) $\mathbb{Z}_2 \times \mathbb{Z}_2$	• strain $\perp [100]$
$P2_1'$ (4.9) $\mathbb{Z}_1$	• $\boldsymbol{E} \parallel [010]$ and $\boldsymbol{B} \parallel [100]$ and strain $\perp [010]$<br>• $\boldsymbol{E} \parallel [010]$ and $\boldsymbol{B} \parallel [001]$ and strain $\perp [010]$<br>• $\boldsymbol{E} \parallel [010]$ and $\boldsymbol{B} \perp [010]$
$Pc'a2_1'$ (29.101) $\mathbb{Z}_1$	• $\boldsymbol{E} \parallel [010]$ and $\boldsymbol{B} \parallel [001]$
$Pna'2_1'$ (33.147) $\mathbb{Z}_1$	• $\boldsymbol{E} \parallel [010]$ and $\boldsymbol{B} \parallel [100]$
$P2_1'/c'$ (14.79) $\mathbb{Z}_2 \times \mathbb{Z}_4$	• $\boldsymbol{B} \parallel [100]$ and strain $\perp [010]$<br>• $\boldsymbol{B} \parallel [001]$ and strain $\perp [010]$<br>• $\boldsymbol{B} \perp [010]$
$P2_1$ (4.7) $\mathbb{Z}_1$	• $\boldsymbol{E} \parallel [010]$ and $\boldsymbol{B} \parallel [010]$ and strain $\perp [010]$
$Pc'a'2_1$ (29.103) $\mathbb{Z}_1$	• $\boldsymbol{E} \parallel [010]$ and $\boldsymbol{B} \parallel [010]$
$P2_1/c$ (14.75) $\mathbb{Z}_2$	• $\boldsymbol{B} \parallel [010]$ and strain $\perp [010]$
P_a2_1 (4.10) $\mathbb{Z}_1$	• $\boldsymbol{E} \parallel [010]$ and strain $\perp [010]$
P_bna2_1 (33.150) $\mathbb{Z}_1$	• $\boldsymbol{E} \parallel [010]$
P_a2_1/c (14.80) $\mathbb{Z}_2$	• strain $\perp [010]$
$P2$ (3.1) $\mathbb{Z}_2$	• $\boldsymbol{E} \parallel [001]$ and $\boldsymbol{B} \parallel [001]$ and strain $\perp [001]$

Continued on next page

. . . continued

Subgroup & SI group	Perturbation
$Pb'a'2$ (32.138) $\mathbb{Z}_2$	• $\boldsymbol{E}$ ∥ [001] and $\boldsymbol{B}$ ∥ [001]
$P2/c$ (13.65) $\mathbb{Z}_2\times\mathbb{Z}_2$	• $\boldsymbol{B}$ ∥ [001] and strain ⊥ [001]
$Pc'ca'$ (54.344) $\mathbb{Z}_2\times\mathbb{Z}_2$	• $\boldsymbol{B}$ ∥ [001]
$P2_1'$ (4.9) $\mathbb{Z}_1$	• $\boldsymbol{E}$ ∥ [001] and $\boldsymbol{B}$ ∥ [100] and strain ⊥ [001]<br>• $\boldsymbol{E}$ ∥ [001] and $\boldsymbol{B}$ ∥ [010] and strain ⊥ [001]<br>• $\boldsymbol{E}$ ∥ [001] and $\boldsymbol{B}$ ⊥ [001]
$Pna'2_1'$ (33.147) $\mathbb{Z}_1$	• $\boldsymbol{E}$ ∥ [001] and $\boldsymbol{B}$ ∥ [010]
$Pna'2_1'$ (33.147) $\mathbb{Z}_1$	• $\boldsymbol{E}$ ∥ [001] and $\boldsymbol{B}$ ∥ [100]
$P2_1'/c'$ (14.79) $\mathbb{Z}_2\times\mathbb{Z}_4$	• $\boldsymbol{B}$ ∥ [100] and strain ⊥ [001]<br>• $\boldsymbol{B}$ ∥ [010] and strain ⊥ [001]<br>• $\boldsymbol{B}$ ⊥ [001]
$Pc'c'n$ (56.369) $\mathbb{Z}_2\times\mathbb{Z}_2$	• $\boldsymbol{B}$ ∥ [100]
$Pb'c'n$ (60.422) $\mathbb{Z}_2$	• $\boldsymbol{B}$ ∥ [010]
P_b2 (3.5) $\mathbb{Z}_2$	• $\boldsymbol{E}$ ∥ [001] and strain ⊥ [001]
P_cnn2 (34.161) $\mathbb{Z}_2$	• $\boldsymbol{E}$ ∥ [001]
P_b2/c (13.71) $\mathbb{Z}_2\times\mathbb{Z}_2$	• strain ⊥ [001]

Table. S 427: Subgroups of P_Anna (52.317) that can be realized by applying $\boldsymbol{E}/\boldsymbol{B}$ fields and/or uniaxial strains.

Subgroup & SI group	Perturbation

Continued on next page

...continued

Subgroup & SI group	Perturbation
P_S1 (1.3) $\mathbb{Z}_1$	• $\boldsymbol{E} \parallel [100]$ and strain in generic direction<br>• $\boldsymbol{E} \parallel [010]$ and strain in generic direction<br>• $\boldsymbol{E} \parallel [001]$ and strain in generic direction<br>• $\boldsymbol{E} \perp [100]$ and strain $\perp [010]$<br>• $\boldsymbol{E} \perp [100]$ and strain $\perp [001]$<br>• $\boldsymbol{E} \perp [100]$ and strain in generic direction<br>• $\boldsymbol{E} \perp [010]$ and strain $\perp [100]$<br>• $\boldsymbol{E} \perp [010]$ and strain $\perp [001]$<br>• $\boldsymbol{E} \perp [010]$ and strain in generic direction<br>• $\boldsymbol{E} \perp [001]$ and strain $\perp [100]$<br>• $\boldsymbol{E} \perp [001]$ and strain $\perp [010]$<br>• $\boldsymbol{E} \perp [001]$ and strain in generic direction<br>• $\boldsymbol{E}$ in generic direction
$P2'$ (3.3) $\mathbb{Z}_1$	• $\boldsymbol{E} \parallel [100]$ and $\boldsymbol{B} \parallel [010]$ and strain $\perp [100]$<br>• $\boldsymbol{E} \parallel [100]$ and $\boldsymbol{B} \parallel [001]$ and strain $\perp [100]$<br>• $\boldsymbol{E} \parallel [100]$ and $\boldsymbol{B} \perp [100]$
$P2$ (3.1) $\mathbb{Z}_2$	• $\boldsymbol{E} \parallel [100]$ and $\boldsymbol{B} \parallel [100]$ and strain $\perp [100]$
P_a2 (3.4) $\mathbb{Z}_2$	• $\boldsymbol{E} \parallel [100]$ and strain $\perp [100]$
Pc (7.24) $\mathbb{Z}_1$	• $\boldsymbol{E} \parallel [100]$ and $\boldsymbol{B} \parallel [001]$ and strain $\perp [001]$<br>• $\boldsymbol{E} \parallel [010]$ and $\boldsymbol{B} \parallel [001]$ and strain $\perp [001]$<br>• $\boldsymbol{E} \perp [001]$ and $\boldsymbol{B} \parallel [001]$
Pc' (7.26) $\mathbb{Z}_1$	• $\boldsymbol{E} \parallel [100]$ and $\boldsymbol{B} \parallel [100]$ and strain $\perp [001]$<br>• $\boldsymbol{E} \parallel [100]$ and $\boldsymbol{B} \parallel [010]$ and strain $\perp [001]$<br>• $\boldsymbol{E} \parallel [100]$ and $\boldsymbol{B} \perp [001]$<br>• $\boldsymbol{E} \parallel [010]$ and $\boldsymbol{B} \parallel [100]$ and strain $\perp [001]$<br>• $\boldsymbol{E} \parallel [010]$ and $\boldsymbol{B} \parallel [010]$ and strain $\perp [001]$<br>• $\boldsymbol{E} \parallel [010]$ and $\boldsymbol{B} \perp [001]$<br>• $\boldsymbol{E} \perp [001]$ and $\boldsymbol{B} \parallel [100]$<br>• $\boldsymbol{E} \perp [001]$ and $\boldsymbol{B} \parallel [010]$<br>• $\boldsymbol{E} \perp [001]$ and $\boldsymbol{B} \perp [001]$
P_Cc (7.30) $\mathbb{Z}_1$	• $\boldsymbol{E} \parallel [100]$ and strain $\perp [001]$<br>• $\boldsymbol{E} \parallel [010]$ and strain $\perp [001]$<br>• $\boldsymbol{E} \perp [001]$

Continued on next page

…continued

Subgroup & SI group	Perturbation
Pc' (7.26) $\mathbb{Z}_1$	• $\boldsymbol{E}$ ∥ [100] and $\boldsymbol{B}$ ∥ [100] and strain ⊥ [010]<br>• $\boldsymbol{E}$ ∥ [100] and $\boldsymbol{B}$ ∥ [001] and strain ⊥ [010]<br>• $\boldsymbol{E}$ ∥ [100] and $\boldsymbol{B}$ ⊥ [010]<br>• $\boldsymbol{E}$ ∥ [001] and $\boldsymbol{B}$ ∥ [100] and strain ⊥ [010]<br>• $\boldsymbol{E}$ ∥ [001] and $\boldsymbol{B}$ ∥ [001] and strain ⊥ [010]<br>• $\boldsymbol{E}$ ∥ [001] and $\boldsymbol{B}$ ⊥ [010]<br>• $\boldsymbol{E}$ ⊥ [010] and $\boldsymbol{B}$ ∥ [100]<br>• $\boldsymbol{E}$ ⊥ [010] and $\boldsymbol{B}$ ∥ [001]<br>• $\boldsymbol{E}$ ⊥ [010] and $\boldsymbol{B}$ ⊥ [010]
$Pc'c2'$ (27.80) $\mathbb{Z}_1$	• $\boldsymbol{E}$ ∥ [100] and $\boldsymbol{B}$ ∥ [001]
$Pn'c'2$ (30.115) $\mathbb{Z}_1$	• $\boldsymbol{E}$ ∥ [100] and $\boldsymbol{B}$ ∥ [100]
Pc (7.24) $\mathbb{Z}_1$	• $\boldsymbol{E}$ ∥ [100] and $\boldsymbol{B}$ ∥ [010] and strain ⊥ [010]<br>• $\boldsymbol{E}$ ∥ [001] and $\boldsymbol{B}$ ∥ [010] and strain ⊥ [010]<br>• $\boldsymbol{E}$ ⊥ [010] and $\boldsymbol{B}$ ∥ [010]
$Pn'n2'$ (34.158) $\mathbb{Z}_1$	• $\boldsymbol{E}$ ∥ [100] and $\boldsymbol{B}$ ∥ [010]
P_Cc (7.30) $\mathbb{Z}_1$	• $\boldsymbol{E}$ ∥ [100] and strain ⊥ [010]<br>• $\boldsymbol{E}$ ∥ [001] and strain ⊥ [010]<br>• $\boldsymbol{E}$ ⊥ [010]
P_Cnc2 (30.121) $\mathbb{Z}_1$	• $\boldsymbol{E}$ ∥ [100]
Pm' (6.20) $\mathbb{Z}_1$	• $\boldsymbol{E}$ ∥ [010] and $\boldsymbol{B}$ ∥ [010] and strain ⊥ [100]<br>• $\boldsymbol{E}$ ∥ [010] and $\boldsymbol{B}$ ∥ [001] and strain ⊥ [100]<br>• $\boldsymbol{E}$ ∥ [010] and $\boldsymbol{B}$ ⊥ [100]<br>• $\boldsymbol{E}$ ∥ [001] and $\boldsymbol{B}$ ∥ [010] and strain ⊥ [100]<br>• $\boldsymbol{E}$ ∥ [001] and $\boldsymbol{B}$ ∥ [001] and strain ⊥ [100]<br>• $\boldsymbol{E}$ ∥ [001] and $\boldsymbol{B}$ ⊥ [100]<br>• $\boldsymbol{E}$ ⊥ [100] and $\boldsymbol{B}$ ∥ [010]<br>• $\boldsymbol{E}$ ⊥ [100] and $\boldsymbol{B}$ ∥ [001]<br>• $\boldsymbol{E}$ ⊥ [100] and $\boldsymbol{B}$ ⊥ [100]
Pc (7.24) $\mathbb{Z}_1$	• $\boldsymbol{E}$ ∥ [010] and $\boldsymbol{B}$ ∥ [100] and strain ⊥ [100]<br>• $\boldsymbol{E}$ ∥ [001] and $\boldsymbol{B}$ ∥ [100] and strain ⊥ [100]<br>• $\boldsymbol{E}$ ⊥ [100] and $\boldsymbol{B}$ ∥ [100]
P_cc (7.28) $\mathbb{Z}_1$	• $\boldsymbol{E}$ ∥ [010] and strain ⊥ [100]<br>• $\boldsymbol{E}$ ∥ [001] and strain ⊥ [100]<br>• $\boldsymbol{E}$ ⊥ [100]

Continued on next page

...continued

Subgroup & SI group	Perturbation
$P\bar{1}$ (2.4) $\mathbb{Z}_2 \times \mathbb{Z}_2 \times \mathbb{Z}_2 \times \mathbb{Z}_4$	• $\boldsymbol{B} \parallel [100]$ and strain in generic direction<br>• $\boldsymbol{B} \parallel [010]$ and strain in generic direction<br>• $\boldsymbol{B} \parallel [001]$ and strain in generic direction<br>• $\boldsymbol{B} \perp [100]$ and strain $\perp [010]$<br>• $\boldsymbol{B} \perp [100]$ and strain $\perp [001]$<br>• $\boldsymbol{B} \perp [100]$ and strain in generic direction<br>• $\boldsymbol{B} \perp [010]$ and strain $\perp [100]$<br>• $\boldsymbol{B} \perp [010]$ and strain $\perp [001]$<br>• $\boldsymbol{B} \perp [010]$ and strain in generic direction<br>• $\boldsymbol{B} \perp [001]$ and strain $\perp [100]$<br>• $\boldsymbol{B} \perp [001]$ and strain $\perp [010]$<br>• $\boldsymbol{B} \perp [001]$ and strain in generic direction<br>• $\boldsymbol{B}$ in generic direction
$P2'/m'$ (10.46) $\mathbb{Z}_2 \times \mathbb{Z}_2 \times \mathbb{Z}_2 \times \mathbb{Z}_4$	• $\boldsymbol{B} \parallel [010]$ and strain $\perp [100]$<br>• $\boldsymbol{B} \parallel [001]$ and strain $\perp [100]$<br>• $\boldsymbol{B} \perp [100]$
$P2/c$ (13.65) $\mathbb{Z}_2 \times \mathbb{Z}_2$	• $\boldsymbol{B} \parallel [100]$ and strain $\perp [100]$
$P_S\bar{1}$ (2.7) $\mathbb{Z}_2 \times \mathbb{Z}_2 \times \mathbb{Z}_4$	• strain in generic direction
P_c2/c (13.72) $\mathbb{Z}_2 \times \mathbb{Z}_2$	• strain $\perp [100]$
$P2'$ (3.3) $\mathbb{Z}_1$	• $\boldsymbol{E} \parallel [010]$ and $\boldsymbol{B} \parallel [100]$ and strain $\perp [010]$<br>• $\boldsymbol{E} \parallel [010]$ and $\boldsymbol{B} \parallel [001]$ and strain $\perp [010]$<br>• $\boldsymbol{E} \parallel [010]$ and $\boldsymbol{B} \perp [010]$
$Pm'a2'$ (28.89) $\mathbb{Z}_1$	• $\boldsymbol{E} \parallel [010]$ and $\boldsymbol{B} \parallel [001]$
$Pn'n2'$ (34.158) $\mathbb{Z}_1$	• $\boldsymbol{E} \parallel [010]$ and $\boldsymbol{B} \parallel [100]$
$P2'/c'$ (13.69) $\mathbb{Z}_2 \times \mathbb{Z}_2 \times \mathbb{Z}_4$	• $\boldsymbol{B} \parallel [100]$ and strain $\perp [010]$<br>• $\boldsymbol{B} \parallel [001]$ and strain $\perp [010]$<br>• $\boldsymbol{B} \perp [010]$
$P2_1$ (4.7) $\mathbb{Z}_1$	• $\boldsymbol{E} \parallel [010]$ and $\boldsymbol{B} \parallel [010]$ and strain $\perp [010]$
$Pm'n'2_1$ (31.127) $\mathbb{Z}_1$	• $\boldsymbol{E} \parallel [010]$ and $\boldsymbol{B} \parallel [010]$
$P2_1/c$ (14.75) $\mathbb{Z}_2$	• $\boldsymbol{B} \parallel [010]$ and strain $\perp [010]$
P_C2_1 (4.12) $\mathbb{Z}_1$	• $\boldsymbol{E} \parallel [010]$ and strain $\perp [010]$
P_Ana2_1 (33.152) $\mathbb{Z}_1$	• $\boldsymbol{E} \parallel [010]$
P_C2_1/c (14.84) $\mathbb{Z}_2$	• strain $\perp [010]$
$P2$ (3.1) $\mathbb{Z}_2$	• $\boldsymbol{E} \parallel [001]$ and $\boldsymbol{B} \parallel [001]$ and strain $\perp [001]$

Continued on next page

. . . continued

Subgroup & SI group	Perturbation
$Pm'a'2$ (28.91) $\mathbb{Z}_2$	• $\boldsymbol{E}$ ∥ [001] and $\boldsymbol{B}$ ∥ [001]
$P2/c$ (13.65) $\mathbb{Z}_2 \times \mathbb{Z}_2$	• $\boldsymbol{B}$ ∥ [001] and strain ⊥ [001]
$Pc'cm'$ (49.270) $\mathbb{Z}_2 \times \mathbb{Z}_2$	• $\boldsymbol{B}$ ∥ [001]
$P2_1'$ (4.9) $\mathbb{Z}_1$	• $\boldsymbol{E}$ ∥ [001] and $\boldsymbol{B}$ ∥ [100] and strain ⊥ [001]<br>• $\boldsymbol{E}$ ∥ [001] and $\boldsymbol{B}$ ∥ [010] and strain ⊥ [001]<br>• $\boldsymbol{E}$ ∥ [001] and $\boldsymbol{B}$ ⊥ [001]
$Pm'n2_1'$ (31.125) $\mathbb{Z}_1$	• $\boldsymbol{E}$ ∥ [001] and $\boldsymbol{B}$ ∥ [010]
$Pna'2_1'$ (33.147) $\mathbb{Z}_1$	• $\boldsymbol{E}$ ∥ [001] and $\boldsymbol{B}$ ∥ [100]
$P2_1'/c'$ (14.79) $\mathbb{Z}_2 \times \mathbb{Z}_4$	• $\boldsymbol{B}$ ∥ [100] and strain ⊥ [001]<br>• $\boldsymbol{B}$ ∥ [010] and strain ⊥ [001]<br>• $\boldsymbol{B}$ ⊥ [001]
$Pnn'a'$ (52.311) $\mathbb{Z}_2$	• $\boldsymbol{B}$ ∥ [100]
$Pnn'm'$ (58.398) $\mathbb{Z}_2$	• $\boldsymbol{B}$ ∥ [010]
P_C2 (3.6) $\mathbb{Z}_1$	• $\boldsymbol{E}$ ∥ [001] and strain ⊥ [001]
P_Ann2 (34.162) $\mathbb{Z}_1$	• $\boldsymbol{E}$ ∥ [001]
P_C2/c (13.74) $\mathbb{Z}_2$	• strain ⊥ [001]

Table. S 428: Subgroups of P_Bnna (52.318) that can be realized by applying $\boldsymbol{E}/\boldsymbol{B}$ fields and/or uniaxial strains.

Subgroup & SI group	Perturbation
Pm' (6.20) $\mathbb{Z}_1$	• $\boldsymbol{E}$ ∥ [100] and $\boldsymbol{B}$ ∥ [100] and strain ⊥ [001]<br>• $\boldsymbol{E}$ ∥ [100] and $\boldsymbol{B}$ ∥ [010] and strain ⊥ [001]<br>• $\boldsymbol{E}$ ∥ [100] and $\boldsymbol{B}$ ⊥ [001]<br>• $\boldsymbol{E}$ ∥ [010] and $\boldsymbol{B}$ ∥ [100] and strain ⊥ [001]<br>• $\boldsymbol{E}$ ∥ [010] and $\boldsymbol{B}$ ∥ [010] and strain ⊥ [001]<br>• $\boldsymbol{E}$ ∥ [010] and $\boldsymbol{B}$ ⊥ [001]<br>• $\boldsymbol{E}$ ⊥ [001] and $\boldsymbol{B}$ ∥ [100]<br>• $\boldsymbol{E}$ ⊥ [001] and $\boldsymbol{B}$ ∥ [010]<br>• $\boldsymbol{E}$ ⊥ [001] and $\boldsymbol{B}$ ⊥ [001]

Continued on next page

...continued

Subgroup & SI group	Perturbation
Pm' (6.20) $\mathbb{Z}_1$	• $\boldsymbol{E}$ ∥ [100] and $\boldsymbol{B}$ ∥ [100] and strain ⊥ [010]<br>• $\boldsymbol{E}$ ∥ [100] and $\boldsymbol{B}$ ∥ [001] and strain ⊥ [010]<br>• $\boldsymbol{E}$ ∥ [100] and $\boldsymbol{B}$ ⊥ [010]<br>• $\boldsymbol{E}$ ∥ [001] and $\boldsymbol{B}$ ∥ [100] and strain ⊥ [010]<br>• $\boldsymbol{E}$ ∥ [001] and $\boldsymbol{B}$ ∥ [001] and strain ⊥ [010]<br>• $\boldsymbol{E}$ ∥ [001] and $\boldsymbol{B}$ ⊥ [010]<br>• $\boldsymbol{E}$ ⊥ [010] and $\boldsymbol{B}$ ∥ [100]<br>• $\boldsymbol{E}$ ⊥ [010] and $\boldsymbol{B}$ ∥ [001]<br>• $\boldsymbol{E}$ ⊥ [010] and $\boldsymbol{B}$ ⊥ [010]
$P2$ (3.1) $\mathbb{Z}_2$	• $\boldsymbol{E}$ ∥ [100] and $\boldsymbol{B}$ ∥ [100] and strain ⊥ [100]
$Pm'm'2$ (25.60) $\mathbb{Z}_2$	• $\boldsymbol{E}$ ∥ [100] and $\boldsymbol{B}$ ∥ [100]
P_S1 (1.3) $\mathbb{Z}_1$	• $\boldsymbol{E}$ ∥ [100] and strain in generic direction<br>• $\boldsymbol{E}$ ∥ [010] and strain in generic direction<br>• $\boldsymbol{E}$ ∥ [001] and strain in generic direction<br>• $\boldsymbol{E}$ ⊥ [100] and strain ⊥ [010]<br>• $\boldsymbol{E}$ ⊥ [100] and strain ⊥ [001]<br>• $\boldsymbol{E}$ ⊥ [100] and strain in generic direction<br>• $\boldsymbol{E}$ ⊥ [010] and strain ⊥ [100]<br>• $\boldsymbol{E}$ ⊥ [010] and strain ⊥ [001]<br>• $\boldsymbol{E}$ ⊥ [010] and strain in generic direction<br>• $\boldsymbol{E}$ ⊥ [001] and strain ⊥ [100]<br>• $\boldsymbol{E}$ ⊥ [001] and strain ⊥ [010]<br>• $\boldsymbol{E}$ ⊥ [001] and strain in generic direction<br>• $\boldsymbol{E}$ in generic direction
Pc (7.24) $\mathbb{Z}_1$	• $\boldsymbol{E}$ ∥ [100] and $\boldsymbol{B}$ ∥ [001] and strain ⊥ [001]<br>• $\boldsymbol{E}$ ∥ [010] and $\boldsymbol{B}$ ∥ [001] and strain ⊥ [001]<br>• $\boldsymbol{E}$ ⊥ [001] and $\boldsymbol{B}$ ∥ [001]
P_Ac (7.31) $\mathbb{Z}_1$	• $\boldsymbol{E}$ ∥ [100] and strain ⊥ [001]<br>• $\boldsymbol{E}$ ∥ [010] and strain ⊥ [001]<br>• $\boldsymbol{E}$ ⊥ [001]
Pc (7.24) $\mathbb{Z}_1$	• $\boldsymbol{E}$ ∥ [100] and $\boldsymbol{B}$ ∥ [010] and strain ⊥ [010]<br>• $\boldsymbol{E}$ ∥ [001] and $\boldsymbol{B}$ ∥ [010] and strain ⊥ [010]<br>• $\boldsymbol{E}$ ⊥ [010] and $\boldsymbol{B}$ ∥ [010]
P_cc (7.28) $\mathbb{Z}_1$	• $\boldsymbol{E}$ ∥ [100] and strain ⊥ [010]<br>• $\boldsymbol{E}$ ∥ [001] and strain ⊥ [010]<br>• $\boldsymbol{E}$ ⊥ [010]

Continued on next page

…continued

Subgroup & SI group	Perturbation
$P2_1'$ (4.9) $\mathbb{Z}_1$	• $\boldsymbol{E} \parallel [100]$ and $\boldsymbol{B} \parallel [010]$ and strain $\perp [100]$<br>• $\boldsymbol{E} \parallel [100]$ and $\boldsymbol{B} \parallel [001]$ and strain $\perp [100]$<br>• $\boldsymbol{E} \parallel [100]$ and $\boldsymbol{B} \perp [100]$
P_C2 (3.6) $\mathbb{Z}_1$	• $\boldsymbol{E} \parallel [100]$ and strain $\perp [100]$
$Pm'c2_1'$ (26.68) $\mathbb{Z}_1$	• $\boldsymbol{E} \parallel [100]$ and $\boldsymbol{B} \parallel [001]$
$Pm'n2_1'$ (31.125) $\mathbb{Z}_1$	• $\boldsymbol{E} \parallel [100]$ and $\boldsymbol{B} \parallel [010]$
P_Anc2 (30.119) $\mathbb{Z}_1$	• $\boldsymbol{E} \parallel [100]$
Pc (7.24) $\mathbb{Z}_1$	• $\boldsymbol{E} \parallel [010]$ and $\boldsymbol{B} \parallel [100]$ and strain $\perp [100]$<br>• $\boldsymbol{E} \parallel [001]$ and $\boldsymbol{B} \parallel [100]$ and strain $\perp [100]$<br>• $\boldsymbol{E} \perp [100]$ and $\boldsymbol{B} \parallel [100]$
$P2_1'$ (4.9) $\mathbb{Z}_1$	• $\boldsymbol{E} \parallel [010]$ and $\boldsymbol{B} \parallel [100]$ and strain $\perp [010]$<br>• $\boldsymbol{E} \parallel [010]$ and $\boldsymbol{B} \parallel [001]$ and strain $\perp [010]$<br>• $\boldsymbol{E} \parallel [010]$ and $\boldsymbol{B} \perp [010]$
$Pm'n2_1'$ (31.125) $\mathbb{Z}_1$	• $\boldsymbol{E} \parallel [010]$ and $\boldsymbol{B} \parallel [100]$
$P2_1'$ (4.9) $\mathbb{Z}_1$	• $\boldsymbol{E} \parallel [001]$ and $\boldsymbol{B} \parallel [100]$ and strain $\perp [001]$<br>• $\boldsymbol{E} \parallel [001]$ and $\boldsymbol{B} \parallel [010]$ and strain $\perp [001]$<br>• $\boldsymbol{E} \parallel [001]$ and $\boldsymbol{B} \perp [001]$
$Pm'n2_1'$ (31.125) $\mathbb{Z}_1$	• $\boldsymbol{E} \parallel [001]$ and $\boldsymbol{B} \parallel [100]$
$P\bar{1}$ (2.4) $\mathbb{Z}_2 \times \mathbb{Z}_2 \times \mathbb{Z}_2 \times \mathbb{Z}_4$	• $\boldsymbol{B} \parallel [100]$ and strain in generic direction<br>• $\boldsymbol{B} \parallel [010]$ and strain in generic direction<br>• $\boldsymbol{B} \parallel [001]$ and strain in generic direction<br>• $\boldsymbol{B} \perp [100]$ and strain $\perp [010]$<br>• $\boldsymbol{B} \perp [100]$ and strain $\perp [001]$<br>• $\boldsymbol{B} \perp [100]$ and strain in generic direction<br>• $\boldsymbol{B} \perp [010]$ and strain $\perp [100]$<br>• $\boldsymbol{B} \perp [010]$ and strain $\perp [001]$<br>• $\boldsymbol{B} \perp [010]$ and strain in generic direction<br>• $\boldsymbol{B} \perp [001]$ and strain $\perp [100]$<br>• $\boldsymbol{B} \perp [001]$ and strain $\perp [010]$<br>• $\boldsymbol{B} \perp [001]$ and strain in generic direction<br>• $\boldsymbol{B}$ in generic direction
$P2/c$ (13.65) $\mathbb{Z}_2 \times \mathbb{Z}_2$	• $\boldsymbol{B} \parallel [100]$ and strain $\perp [100]$
$P2_1'/m'$ (11.54) $\mathbb{Z}_2 \times \mathbb{Z}_2 \times \mathbb{Z}_4$	• $\boldsymbol{B} \parallel [100]$ and strain $\perp [010]$<br>• $\boldsymbol{B} \parallel [001]$ and strain $\perp [010]$<br>• $\boldsymbol{B} \perp [010]$

Continued on next page

...continued

Subgroup & SI group	Perturbation
$P2_1'/m'$ (11.54) $\mathbb{Z}_2 \times \mathbb{Z}_2 \times \mathbb{Z}_4$	• $\boldsymbol{B}$ ∥ [100] and strain ⊥ [001]<br>• $\boldsymbol{B}$ ∥ [010] and strain ⊥ [001]<br>• $\boldsymbol{B}$ ⊥ [001]
$Pm'm'n$ (59.409) $\mathbb{Z}_2 \times \mathbb{Z}_2$	• $\boldsymbol{B}$ ∥ [100]
Pc' (7.26) $\mathbb{Z}_1$	• $\boldsymbol{E}$ ∥ [010] and $\boldsymbol{B}$ ∥ [010] and strain ⊥ [100]<br>• $\boldsymbol{E}$ ∥ [010] and $\boldsymbol{B}$ ∥ [001] and strain ⊥ [100]<br>• $\boldsymbol{E}$ ∥ [010] and $\boldsymbol{B}$ ⊥ [100]<br>• $\boldsymbol{E}$ ∥ [001] and $\boldsymbol{B}$ ∥ [010] and strain ⊥ [100]<br>• $\boldsymbol{E}$ ∥ [001] and $\boldsymbol{B}$ ∥ [001] and strain ⊥ [100]<br>• $\boldsymbol{E}$ ∥ [001] and $\boldsymbol{B}$ ⊥ [100]<br>• $\boldsymbol{E}$ ⊥ [100] and $\boldsymbol{B}$ ∥ [010]<br>• $\boldsymbol{E}$ ⊥ [100] and $\boldsymbol{B}$ ∥ [001]<br>• $\boldsymbol{E}$ ⊥ [100] and $\boldsymbol{B}$ ⊥ [100]
P_Cc (7.30) $\mathbb{Z}_1$	• $\boldsymbol{E}$ ∥ [010] and strain ⊥ [100]<br>• $\boldsymbol{E}$ ∥ [001] and strain ⊥ [100]<br>• $\boldsymbol{E}$ ⊥ [100]
$Pc'a2_1'$ (29.101) $\mathbb{Z}_1$	• $\boldsymbol{E}$ ∥ [010] and $\boldsymbol{B}$ ∥ [001]
$Pna'2_1'$ (33.147) $\mathbb{Z}_1$	• $\boldsymbol{E}$ ∥ [001] and $\boldsymbol{B}$ ∥ [010]
$P2_1'/c'$ (14.79) $\mathbb{Z}_2 \times \mathbb{Z}_4$	• $\boldsymbol{B}$ ∥ [010] and strain ⊥ [100]<br>• $\boldsymbol{B}$ ∥ [001] and strain ⊥ [100]<br>• $\boldsymbol{B}$ ⊥ [100]
$P2_1$ (4.7) $\mathbb{Z}_1$	• $\boldsymbol{E}$ ∥ [010] and $\boldsymbol{B}$ ∥ [010] and strain ⊥ [010]
P_a2_1 (4.10) $\mathbb{Z}_1$	• $\boldsymbol{E}$ ∥ [010] and strain ⊥ [010]
$P2_1/c$ (14.75) $\mathbb{Z}_2$	• $\boldsymbol{B}$ ∥ [010] and strain ⊥ [010]
$Pm'c'2_1$ (26.70) $\mathbb{Z}_1$	• $\boldsymbol{E}$ ∥ [010] and $\boldsymbol{B}$ ∥ [010]
P_Cna2_1 (33.154) $\mathbb{Z}_1$	• $\boldsymbol{E}$ ∥ [010]
$Pnm'a'$ (62.447) $\mathbb{Z}_2$	• $\boldsymbol{B}$ ∥ [010]
$P2$ (3.1) $\mathbb{Z}_2$	• $\boldsymbol{E}$ ∥ [001] and $\boldsymbol{B}$ ∥ [001] and strain ⊥ [001]
P_C2 (3.6) $\mathbb{Z}_1$	• $\boldsymbol{E}$ ∥ [001] and strain ⊥ [001]
$P2/c$ (13.65) $\mathbb{Z}_2 \times \mathbb{Z}_2$	• $\boldsymbol{B}$ ∥ [001] and strain ⊥ [001]
$Pm'a'2$ (28.91) $\mathbb{Z}_2$	• $\boldsymbol{E}$ ∥ [001] and $\boldsymbol{B}$ ∥ [001]
P_Ann2 (34.162) $\mathbb{Z}_1$	• $\boldsymbol{E}$ ∥ [001]
$Pbc'm'$ (57.383) $\mathbb{Z}_2 \times \mathbb{Z}_2$	• $\boldsymbol{B}$ ∥ [001]

Continued on next page

. . . continued

Subgroup & SI group	Perturbation
$P_S\bar{1}$ (2.7) $\mathbb{Z}_2 \times \mathbb{Z}_2 \times \mathbb{Z}_4$	• strain in generic direction
P_C2/c (13.74) $\mathbb{Z}_2$	• strain $\perp$ [100]
P_c2_1/c (14.82) $\mathbb{Z}_2$	• strain $\perp$ [010]
P_A2/c (13.73) $\mathbb{Z}_2$	• strain $\perp$ [001]

Table. S 429: Subgroups of P_Cnna (52.319) that can be realized by applying $\boldsymbol{E}/\boldsymbol{B}$ fields and/or uniaxial strains.

Subgroup & SI group	Perturbation
Pc' (7.26) $\mathbb{Z}_1$	• $\boldsymbol{E}$ $\parallel$ [100] and $\boldsymbol{B}$ $\parallel$ [100] and strain $\perp$ [001]<br>• $\boldsymbol{E}$ $\parallel$ [100] and $\boldsymbol{B}$ $\parallel$ [010] and strain $\perp$ [001]<br>• $\boldsymbol{E}$ $\parallel$ [100] and $\boldsymbol{B}$ $\perp$ [001]<br>• $\boldsymbol{E}$ $\parallel$ [010] and $\boldsymbol{B}$ $\parallel$ [100] and strain $\perp$ [001]<br>• $\boldsymbol{E}$ $\parallel$ [010] and $\boldsymbol{B}$ $\parallel$ [010] and strain $\perp$ [001]<br>• $\boldsymbol{E}$ $\parallel$ [010] and $\boldsymbol{B}$ $\perp$ [001]<br>• $\boldsymbol{E}$ $\perp$ [001] and $\boldsymbol{B}$ $\parallel$ [100]<br>• $\boldsymbol{E}$ $\perp$ [001] and $\boldsymbol{B}$ $\parallel$ [010]<br>• $\boldsymbol{E}$ $\perp$ [001] and $\boldsymbol{B}$ $\perp$ [001]
Pc' (7.26) $\mathbb{Z}_1$	• $\boldsymbol{E}$ $\parallel$ [100] and $\boldsymbol{B}$ $\parallel$ [100] and strain $\perp$ [010]<br>• $\boldsymbol{E}$ $\parallel$ [100] and $\boldsymbol{B}$ $\parallel$ [001] and strain $\perp$ [010]<br>• $\boldsymbol{E}$ $\parallel$ [100] and $\boldsymbol{B}$ $\perp$ [010]<br>• $\boldsymbol{E}$ $\parallel$ [001] and $\boldsymbol{B}$ $\parallel$ [100] and strain $\perp$ [010]<br>• $\boldsymbol{E}$ $\parallel$ [001] and $\boldsymbol{B}$ $\parallel$ [001] and strain $\perp$ [010]<br>• $\boldsymbol{E}$ $\parallel$ [001] and $\boldsymbol{B}$ $\perp$ [010]<br>• $\boldsymbol{E}$ $\perp$ [010] and $\boldsymbol{B}$ $\parallel$ [100]<br>• $\boldsymbol{E}$ $\perp$ [010] and $\boldsymbol{B}$ $\parallel$ [001]<br>• $\boldsymbol{E}$ $\perp$ [010] and $\boldsymbol{B}$ $\perp$ [010]
$P2$ (3.1) $\mathbb{Z}_2$	• $\boldsymbol{E}$ $\parallel$ [100] and $\boldsymbol{B}$ $\parallel$ [100] and strain $\perp$ [100]
$Pb'a'2$ (32.138) $\mathbb{Z}_2$	• $\boldsymbol{E}$ $\parallel$ [100] and $\boldsymbol{B}$ $\parallel$ [100]
Pc (7.24) $\mathbb{Z}_1$	• $\boldsymbol{E}$ $\parallel$ [100] and $\boldsymbol{B}$ $\parallel$ [001] and strain $\perp$ [001]<br>• $\boldsymbol{E}$ $\parallel$ [010] and $\boldsymbol{B}$ $\parallel$ [001] and strain $\perp$ [001]<br>• $\boldsymbol{E}$ $\perp$ [001] and $\boldsymbol{B}$ $\parallel$ [001]

Continued on next page

…continued

Subgroup & SI group	Perturbation
P_S1 (1.3) $\mathbb{Z}_1$	• $\boldsymbol{E}$ $\parallel$ [100] and strain in generic direction<br>• $\boldsymbol{E}$ $\parallel$ [010] and strain in generic direction<br>• $\boldsymbol{E}$ $\parallel$ [001] and strain in generic direction<br>• $\boldsymbol{E}$ $\perp$ [100] and strain $\perp$ [010]<br>• $\boldsymbol{E}$ $\perp$ [100] and strain $\perp$ [001]<br>• $\boldsymbol{E}$ $\perp$ [100] and strain in generic direction<br>• $\boldsymbol{E}$ $\perp$ [010] and strain $\perp$ [100]<br>• $\boldsymbol{E}$ $\perp$ [010] and strain $\perp$ [001]<br>• $\boldsymbol{E}$ $\perp$ [010] and strain in generic direction<br>• $\boldsymbol{E}$ $\perp$ [001] and strain $\perp$ [100]<br>• $\boldsymbol{E}$ $\perp$ [001] and strain $\perp$ [010]<br>• $\boldsymbol{E}$ $\perp$ [001] and strain in generic direction<br>• $\boldsymbol{E}$ in generic direction
P_ac (7.27) $\mathbb{Z}_1$	• $\boldsymbol{E}$ $\parallel$ [100] and strain $\perp$ [001]<br>• $\boldsymbol{E}$ $\parallel$ [010] and strain $\perp$ [001]<br>• $\boldsymbol{E}$ $\perp$ [001]
$P2_1'$ (4.9) $\mathbb{Z}_1$	• $\boldsymbol{E}$ $\parallel$ [100] and $\boldsymbol{B}$ $\parallel$ [010] and strain $\perp$ [100]<br>• $\boldsymbol{E}$ $\parallel$ [100] and $\boldsymbol{B}$ $\parallel$ [001] and strain $\perp$ [100]<br>• $\boldsymbol{E}$ $\parallel$ [100] and $\boldsymbol{B}$ $\perp$ [100]
$Pca'2_1'$ (29.102) $\mathbb{Z}_1$	• $\boldsymbol{E}$ $\parallel$ [100] and $\boldsymbol{B}$ $\parallel$ [001]
P_C2 (3.6) $\mathbb{Z}_1$	• $\boldsymbol{E}$ $\parallel$ [100] and strain $\perp$ [100]
Pc (7.24) $\mathbb{Z}_1$	• $\boldsymbol{E}$ $\parallel$ [100] and $\boldsymbol{B}$ $\parallel$ [010] and strain $\perp$ [010]<br>• $\boldsymbol{E}$ $\parallel$ [001] and $\boldsymbol{B}$ $\parallel$ [010] and strain $\perp$ [010]<br>• $\boldsymbol{E}$ $\perp$ [010] and $\boldsymbol{B}$ $\parallel$ [010]
P_Cc (7.30) $\mathbb{Z}_1$	• $\boldsymbol{E}$ $\parallel$ [100] and strain $\perp$ [010]<br>• $\boldsymbol{E}$ $\parallel$ [001] and strain $\perp$ [010]<br>• $\boldsymbol{E}$ $\perp$ [010]
$Pna'2_1'$ (33.147) $\mathbb{Z}_1$	• $\boldsymbol{E}$ $\parallel$ [100] and $\boldsymbol{B}$ $\parallel$ [010]
P_Bnc2 (30.120) $\mathbb{Z}_2$	• $\boldsymbol{E}$ $\parallel$ [100]
$P2'$ (3.3) $\mathbb{Z}_1$	• $\boldsymbol{E}$ $\parallel$ [010] and $\boldsymbol{B}$ $\parallel$ [100] and strain $\perp$ [010]<br>• $\boldsymbol{E}$ $\parallel$ [010] and $\boldsymbol{B}$ $\parallel$ [001] and strain $\perp$ [010]<br>• $\boldsymbol{E}$ $\parallel$ [010] and $\boldsymbol{B}$ $\perp$ [010]
Pc (7.24) $\mathbb{Z}_1$	• $\boldsymbol{E}$ $\parallel$ [010] and $\boldsymbol{B}$ $\parallel$ [100] and strain $\perp$ [100]<br>• $\boldsymbol{E}$ $\parallel$ [001] and $\boldsymbol{B}$ $\parallel$ [100] and strain $\perp$ [100]<br>• $\boldsymbol{E}$ $\perp$ [100] and $\boldsymbol{B}$ $\parallel$ [100]
$Pnc'2'$ (30.114) $\mathbb{Z}_1$	• $\boldsymbol{E}$ $\parallel$ [010] and $\boldsymbol{B}$ $\parallel$ [100]

Continued on next page

...continued

Subgroup & SI group	Perturbation
$P\bar{1}$ (2.4) $\mathbb{Z}_2 \times \mathbb{Z}_2 \times \mathbb{Z}_2 \times \mathbb{Z}_4$	• $\boldsymbol{B}$ ∥ [100] and strain in generic direction<br>• $\boldsymbol{B}$ ∥ [010] and strain in generic direction<br>• $\boldsymbol{B}$ ∥ [001] and strain in generic direction<br>• $\boldsymbol{B} \perp$ [100] and strain $\perp$ [010]<br>• $\boldsymbol{B} \perp$ [100] and strain $\perp$ [001]<br>• $\boldsymbol{B} \perp$ [100] and strain in generic direction<br>• $\boldsymbol{B} \perp$ [010] and strain $\perp$ [100]<br>• $\boldsymbol{B} \perp$ [010] and strain $\perp$ [001]<br>• $\boldsymbol{B} \perp$ [010] and strain in generic direction<br>• $\boldsymbol{B} \perp$ [001] and strain $\perp$ [100]<br>• $\boldsymbol{B} \perp$ [001] and strain $\perp$ [010]<br>• $\boldsymbol{B} \perp$ [001] and strain in generic direction<br>• $\boldsymbol{B}$ in generic direction
$P2'/c'$ (13.69) $\mathbb{Z}_2 \times \mathbb{Z}_2 \times \mathbb{Z}_4$	• $\boldsymbol{B}$ ∥ [100] and strain $\perp$ [010]<br>• $\boldsymbol{B}$ ∥ [001] and strain $\perp$ [010]<br>• $\boldsymbol{B} \perp$ [010]
$P2/c$ (13.65) $\mathbb{Z}_2 \times \mathbb{Z}_2$	• $\boldsymbol{B}$ ∥ [100] and strain $\perp$ [100]
$P2'$ (3.3) $\mathbb{Z}_1$	• $\boldsymbol{E}$ ∥ [001] and $\boldsymbol{B}$ ∥ [100] and strain $\perp$ [001]<br>• $\boldsymbol{E}$ ∥ [001] and $\boldsymbol{B}$ ∥ [010] and strain $\perp$ [001]<br>• $\boldsymbol{E}$ ∥ [001] and $\boldsymbol{B} \perp$ [001]
$Pnc'2'$ (30.114) $\mathbb{Z}_1$	• $\boldsymbol{E}$ ∥ [001] and $\boldsymbol{B}$ ∥ [100]
$P2'/c'$ (13.69) $\mathbb{Z}_2 \times \mathbb{Z}_2 \times \mathbb{Z}_4$	• $\boldsymbol{B}$ ∥ [100] and strain $\perp$ [001]<br>• $\boldsymbol{B}$ ∥ [010] and strain $\perp$ [001]<br>• $\boldsymbol{B} \perp$ [001]
$Pb'a'n$ (50.281) $\mathbb{Z}_2 \times \mathbb{Z}_2$	• $\boldsymbol{B}$ ∥ [100]
Pc' (7.26) $\mathbb{Z}_1$	• $\boldsymbol{E}$ ∥ [010] and $\boldsymbol{B}$ ∥ [010] and strain $\perp$ [100]<br>• $\boldsymbol{E}$ ∥ [010] and $\boldsymbol{B}$ ∥ [001] and strain $\perp$ [100]<br>• $\boldsymbol{E}$ ∥ [010] and $\boldsymbol{B} \perp$ [100]<br>• $\boldsymbol{E}$ ∥ [001] and $\boldsymbol{B}$ ∥ [010] and strain $\perp$ [100]<br>• $\boldsymbol{E}$ ∥ [001] and $\boldsymbol{B}$ ∥ [001] and strain $\perp$ [100]<br>• $\boldsymbol{E}$ ∥ [001] and $\boldsymbol{B} \perp$ [100]<br>• $\boldsymbol{E} \perp$ [100] and $\boldsymbol{B}$ ∥ [010]<br>• $\boldsymbol{E} \perp$ [100] and $\boldsymbol{B}$ ∥ [001]<br>• $\boldsymbol{E} \perp$ [100] and $\boldsymbol{B} \perp$ [100]
$Pb'a2'$ (32.137) $\mathbb{Z}_1$	• $\boldsymbol{E}$ ∥ [010] and $\boldsymbol{B}$ ∥ [001]

Continued on next page

. . . continued

Subgroup & SI group	Perturbation
P_Cc (7.30) $\mathbb{Z}_1$	• $\boldsymbol{E}$ ∥ [010] and strain ⊥ [100]<br>• $\boldsymbol{E}$ ∥ [001] and strain ⊥ [100]<br>• $\boldsymbol{E}$ ⊥ [100]
$P2_1'/c'$ (14.79) $\mathbb{Z}_2 \times \mathbb{Z}_4$	• $\boldsymbol{B}$ ∥ [010] and strain ⊥ [100]<br>• $\boldsymbol{B}$ ∥ [001] and strain ⊥ [100]<br>• $\boldsymbol{B}$ ⊥ [100]
$Pnc'2'$ (30.114) $\mathbb{Z}_1$	• $\boldsymbol{E}$ ∥ [001] and $\boldsymbol{B}$ ∥ [010]
$P2_1$ (4.7) $\mathbb{Z}_1$	• $\boldsymbol{E}$ ∥ [010] and $\boldsymbol{B}$ ∥ [010] and strain ⊥ [010]
P_C2_1 (4.12) $\mathbb{Z}_1$	• $\boldsymbol{E}$ ∥ [010] and strain ⊥ [010]
$P2_1/c$ (14.75) $\mathbb{Z}_2$	• $\boldsymbol{B}$ ∥ [010] and strain ⊥ [010]
$Pc'a'2_1$ (29.103) $\mathbb{Z}_1$	• $\boldsymbol{E}$ ∥ [010] and $\boldsymbol{B}$ ∥ [010]
P_Bna2_1 (33.153) $\mathbb{Z}_1$	• $\boldsymbol{E}$ ∥ [010]
$Pb'c'n$ (60.422) $\mathbb{Z}_2$	• $\boldsymbol{B}$ ∥ [010]
$P2$ (3.1) $\mathbb{Z}_2$	• $\boldsymbol{E}$ ∥ [001] and $\boldsymbol{B}$ ∥ [001] and strain ⊥ [001]
$P2/c$ (13.65) $\mathbb{Z}_2 \times \mathbb{Z}_2$	• $\boldsymbol{B}$ ∥ [001] and strain ⊥ [001]
P_a2 (3.4) $\mathbb{Z}_2$	• $\boldsymbol{E}$ ∥ [001] and strain ⊥ [001]
$Pc'c'2$ (27.81) $\mathbb{Z}_2$	• $\boldsymbol{E}$ ∥ [001] and $\boldsymbol{B}$ ∥ [001]
$Pc'c'a$ (54.342) $\mathbb{Z}_2 \times \mathbb{Z}_2$	• $\boldsymbol{B}$ ∥ [001]
P_Cnn2 (34.163) $\mathbb{Z}_2$	• $\boldsymbol{E}$ ∥ [001]
$P_S\bar{1}$ (2.7) $\mathbb{Z}_2 \times \mathbb{Z}_2 \times \mathbb{Z}_4$	• strain in generic direction
P_C2/c (13.74) $\mathbb{Z}_2$	• strain ⊥ [100]
P_C2_1/c (14.84) $\mathbb{Z}_2$	• strain ⊥ [010]
P_a2/c (13.70) $\mathbb{Z}_2 \times \mathbb{Z}_2$	• strain ⊥ [001]

Table. S 430: Subgroups of P_Inna (52.320) that can be realized by applying $\boldsymbol{E}/\boldsymbol{B}$ fields and/or uniaxial strains.

Subgroup & SI group	Perturbation

Continued on next page

...continued

Subgroup & SI group	Perturbation
Pc' (7.26) $\mathbb{Z}_1$	• $\boldsymbol{E}$ ∥ [100] and $\boldsymbol{B}$ ∥ [100] and strain ⊥ [001]<br>• $\boldsymbol{E}$ ∥ [100] and $\boldsymbol{B}$ ∥ [010] and strain ⊥ [001]<br>• $\boldsymbol{E}$ ∥ [100] and $\boldsymbol{B}$ ⊥ [001]<br>• $\boldsymbol{E}$ ∥ [010] and $\boldsymbol{B}$ ∥ [100] and strain ⊥ [001]<br>• $\boldsymbol{E}$ ∥ [010] and $\boldsymbol{B}$ ∥ [010] and strain ⊥ [001]<br>• $\boldsymbol{E}$ ∥ [010] and $\boldsymbol{B}$ ⊥ [001]<br>• $\boldsymbol{E}$ ⊥ [001] and $\boldsymbol{B}$ ∥ [100]<br>• $\boldsymbol{E}$ ⊥ [001] and $\boldsymbol{B}$ ∥ [010]<br>• $\boldsymbol{E}$ ⊥ [001] and $\boldsymbol{B}$ ⊥ [001]
Pm' (6.20) $\mathbb{Z}_1$	• $\boldsymbol{E}$ ∥ [100] and $\boldsymbol{B}$ ∥ [100] and strain ⊥ [010]<br>• $\boldsymbol{E}$ ∥ [100] and $\boldsymbol{B}$ ∥ [001] and strain ⊥ [010]<br>• $\boldsymbol{E}$ ∥ [100] and $\boldsymbol{B}$ ⊥ [010]<br>• $\boldsymbol{E}$ ∥ [001] and $\boldsymbol{B}$ ∥ [100] and strain ⊥ [010]<br>• $\boldsymbol{E}$ ∥ [001] and $\boldsymbol{B}$ ∥ [001] and strain ⊥ [010]<br>• $\boldsymbol{E}$ ∥ [001] and $\boldsymbol{B}$ ⊥ [010]<br>• $\boldsymbol{E}$ ⊥ [010] and $\boldsymbol{B}$ ∥ [100]<br>• $\boldsymbol{E}$ ⊥ [010] and $\boldsymbol{B}$ ∥ [001]<br>• $\boldsymbol{E}$ ⊥ [010] and $\boldsymbol{B}$ ⊥ [010]
$P2$ (3.1) $\mathbb{Z}_2$	• $\boldsymbol{E}$ ∥ [100] and $\boldsymbol{B}$ ∥ [100] and strain ⊥ [100]
$Pm'a'2$ (28.91) $\mathbb{Z}_2$	• $\boldsymbol{E}$ ∥ [100] and $\boldsymbol{B}$ ∥ [100]
Pc (7.24) $\mathbb{Z}_1$	• $\boldsymbol{E}$ ∥ [100] and $\boldsymbol{B}$ ∥ [001] and strain ⊥ [001]<br>• $\boldsymbol{E}$ ∥ [010] and $\boldsymbol{B}$ ∥ [001] and strain ⊥ [001]<br>• $\boldsymbol{E}$ ⊥ [001] and $\boldsymbol{B}$ ∥ [001]
P_S1 (1.3) $\mathbb{Z}_1$	• $\boldsymbol{E}$ ∥ [100] and strain in generic direction<br>• $\boldsymbol{E}$ ∥ [010] and strain in generic direction<br>• $\boldsymbol{E}$ ∥ [001] and strain in generic direction<br>• $\boldsymbol{E}$ ⊥ [100] and strain ⊥ [010]<br>• $\boldsymbol{E}$ ⊥ [100] and strain ⊥ [001]<br>• $\boldsymbol{E}$ ⊥ [100] and strain in generic direction<br>• $\boldsymbol{E}$ ⊥ [010] and strain ⊥ [100]<br>• $\boldsymbol{E}$ ⊥ [010] and strain ⊥ [001]<br>• $\boldsymbol{E}$ ⊥ [010] and strain in generic direction<br>• $\boldsymbol{E}$ ⊥ [001] and strain ⊥ [100]<br>• $\boldsymbol{E}$ ⊥ [001] and strain ⊥ [010]<br>• $\boldsymbol{E}$ ⊥ [001] and strain in generic direction<br>• $\boldsymbol{E}$ in generic direction

Continued on next page

. . . continued

Subgroup & SI group	Perturbation
P_Cc (7.30) $\mathbb{Z}_1$	• $\boldsymbol{E} \parallel [100]$ and strain $\perp [001]$<br>• $\boldsymbol{E} \parallel [010]$ and strain $\perp [001]$<br>• $\boldsymbol{E} \perp [001]$
$P2_1'$ (4.9) $\mathbb{Z}_1$	• $\boldsymbol{E} \parallel [100]$ and $\boldsymbol{B} \parallel [010]$ and strain $\perp [100]$<br>• $\boldsymbol{E} \parallel [100]$ and $\boldsymbol{B} \parallel [001]$ and strain $\perp [100]$<br>• $\boldsymbol{E} \parallel [100]$ and $\boldsymbol{B} \perp [100]$
$Pm'c2_1'$ (26.68) $\mathbb{Z}_1$	• $\boldsymbol{E} \parallel [100]$ and $\boldsymbol{B} \parallel [001]$
P_C2 (3.6) $\mathbb{Z}_1$	• $\boldsymbol{E} \parallel [100]$ and strain $\perp [100]$
Pc (7.24) $\mathbb{Z}_1$	• $\boldsymbol{E} \parallel [100]$ and $\boldsymbol{B} \parallel [010]$ and strain $\perp [010]$<br>• $\boldsymbol{E} \parallel [001]$ and $\boldsymbol{B} \parallel [010]$ and strain $\perp [010]$<br>• $\boldsymbol{E} \perp [010]$ and $\boldsymbol{B} \parallel [010]$
P_Ac (7.31) $\mathbb{Z}_1$	• $\boldsymbol{E} \parallel [100]$ and strain $\perp [010]$<br>• $\boldsymbol{E} \parallel [001]$ and strain $\perp [010]$<br>• $\boldsymbol{E} \perp [010]$
$Pna'2_1'$ (33.147) $\mathbb{Z}_1$	• $\boldsymbol{E} \parallel [100]$ and $\boldsymbol{B} \parallel [010]$
P_Inc2 (30.122) $\mathbb{Z}_1$	• $\boldsymbol{E} \parallel [100]$
$P2'$ (3.3) $\mathbb{Z}_1$	• $\boldsymbol{E} \parallel [010]$ and $\boldsymbol{B} \parallel [100]$ and strain $\perp [010]$<br>• $\boldsymbol{E} \parallel [010]$ and $\boldsymbol{B} \parallel [001]$ and strain $\perp [010]$<br>• $\boldsymbol{E} \parallel [010]$ and $\boldsymbol{B} \perp [010]$
Pc (7.24) $\mathbb{Z}_1$	• $\boldsymbol{E} \parallel [010]$ and $\boldsymbol{B} \parallel [100]$ and strain $\perp [100]$<br>• $\boldsymbol{E} \parallel [001]$ and $\boldsymbol{B} \parallel [100]$ and strain $\perp [100]$<br>• $\boldsymbol{E} \perp [100]$ and $\boldsymbol{B} \parallel [100]$
$Pnc'2'$ (30.114) $\mathbb{Z}_1$	• $\boldsymbol{E} \parallel [010]$ and $\boldsymbol{B} \parallel [100]$
$P\bar{1}$ (2.4) $\mathbb{Z}_2 \times \mathbb{Z}_2 \times \mathbb{Z}_2 \times \mathbb{Z}_4$	• $\boldsymbol{B} \parallel [100]$ and strain in generic direction<br>• $\boldsymbol{B} \parallel [010]$ and strain in generic direction<br>• $\boldsymbol{B} \parallel [001]$ and strain in generic direction<br>• $\boldsymbol{B} \perp [100]$ and strain $\perp [010]$<br>• $\boldsymbol{B} \perp [100]$ and strain $\perp [001]$<br>• $\boldsymbol{B} \perp [100]$ and strain in generic direction<br>• $\boldsymbol{B} \perp [010]$ and strain $\perp [100]$<br>• $\boldsymbol{B} \perp [010]$ and strain $\perp [001]$<br>• $\boldsymbol{B} \perp [010]$ and strain in generic direction<br>• $\boldsymbol{B} \perp [001]$ and strain $\perp [100]$<br>• $\boldsymbol{B} \perp [001]$ and strain $\perp [010]$<br>• $\boldsymbol{B} \perp [001]$ and strain in generic direction<br>• $\boldsymbol{B}$ in generic direction

Continued on next page

…continued

Subgroup & SI group	Perturbation
$P2'/m'$ (10.46) $\mathbb{Z}_2 \times \mathbb{Z}_2 \times \mathbb{Z}_2 \times \mathbb{Z}_4$	• $\boldsymbol{B} \parallel [100]$ and strain $\perp [010]$<br>• $\boldsymbol{B} \parallel [001]$ and strain $\perp [010]$<br>• $\boldsymbol{B} \perp [010]$
$P2/c$ (13.65) $\mathbb{Z}_2 \times \mathbb{Z}_2$	• $\boldsymbol{B} \parallel [100]$ and strain $\perp [100]$
$P2_1'$ (4.9) $\mathbb{Z}_1$	• $\boldsymbol{E} \parallel [001]$ and $\boldsymbol{B} \parallel [100]$ and strain $\perp [001]$<br>• $\boldsymbol{E} \parallel [001]$ and $\boldsymbol{B} \parallel [010]$ and strain $\perp [001]$<br>• $\boldsymbol{E} \parallel [001]$ and $\boldsymbol{B} \perp [001]$
$Pm'n2_1'$ (31.125) $\mathbb{Z}_1$	• $\boldsymbol{E} \parallel [001]$ and $\boldsymbol{B} \parallel [100]$
$P2_1'/c'$ (14.79) $\mathbb{Z}_2 \times \mathbb{Z}_4$	• $\boldsymbol{B} \parallel [100]$ and strain $\perp [001]$<br>• $\boldsymbol{B} \parallel [010]$ and strain $\perp [001]$<br>• $\boldsymbol{B} \perp [001]$
$Pm'na'$ (53.328) $\mathbb{Z}_2 \times \mathbb{Z}_2$	• $\boldsymbol{B} \parallel [100]$
Pm' (6.20) $\mathbb{Z}_1$	• $\boldsymbol{E} \parallel [010]$ and $\boldsymbol{B} \parallel [010]$ and strain $\perp [100]$<br>• $\boldsymbol{E} \parallel [010]$ and $\boldsymbol{B} \parallel [001]$ and strain $\perp [100]$<br>• $\boldsymbol{E} \parallel [010]$ and $\boldsymbol{B} \perp [100]$<br>• $\boldsymbol{E} \parallel [001]$ and $\boldsymbol{B} \parallel [010]$ and strain $\perp [100]$<br>• $\boldsymbol{E} \parallel [001]$ and $\boldsymbol{B} \parallel [001]$ and strain $\perp [100]$<br>• $\boldsymbol{E} \parallel [001]$ and $\boldsymbol{B} \perp [100]$<br>• $\boldsymbol{E} \perp [100]$ and $\boldsymbol{B} \parallel [010]$<br>• $\boldsymbol{E} \perp [100]$ and $\boldsymbol{B} \parallel [001]$<br>• $\boldsymbol{E} \perp [100]$ and $\boldsymbol{B} \perp [100]$
$Pm'a2'$ (28.89) $\mathbb{Z}_1$	• $\boldsymbol{E} \parallel [010]$ and $\boldsymbol{B} \parallel [001]$
P_Ac (7.31) $\mathbb{Z}_1$	• $\boldsymbol{E} \parallel [010]$ and strain $\perp [100]$<br>• $\boldsymbol{E} \parallel [001]$ and strain $\perp [100]$<br>• $\boldsymbol{E} \perp [100]$
$P2_1'/m'$ (11.54) $\mathbb{Z}_2 \times \mathbb{Z}_2 \times \mathbb{Z}_4$	• $\boldsymbol{B} \parallel [010]$ and strain $\perp [100]$<br>• $\boldsymbol{B} \parallel [001]$ and strain $\perp [100]$<br>• $\boldsymbol{B} \perp [100]$
$Pm'n2_1'$ (31.125) $\mathbb{Z}_1$	• $\boldsymbol{E} \parallel [001]$ and $\boldsymbol{B} \parallel [010]$
$P2_1$ (4.7) $\mathbb{Z}_1$	• $\boldsymbol{E} \parallel [010]$ and $\boldsymbol{B} \parallel [010]$ and strain $\perp [010]$
P_C2_1 (4.12) $\mathbb{Z}_1$	• $\boldsymbol{E} \parallel [010]$ and strain $\perp [010]$
$P2_1/c$ (14.75) $\mathbb{Z}_2$	• $\boldsymbol{B} \parallel [010]$ and strain $\perp [010]$
$Pm'c'2_1$ (26.70) $\mathbb{Z}_1$	• $\boldsymbol{E} \parallel [010]$ and $\boldsymbol{B} \parallel [010]$
P_Ina2_1 (33.155) $\mathbb{Z}_1$	• $\boldsymbol{E} \parallel [010]$

Continued on next page

. . . continued

Subgroup & SI group	Perturbation
$Pnm'a'$ (62.447) $\mathbb{Z}_2$	• $\boldsymbol{B}$ ∥ [010]
$P2$ (3.1) $\mathbb{Z}_2$	• $\boldsymbol{E}$ ∥ [001] and $\boldsymbol{B}$ ∥ [001] and strain ⊥ [001]
$P2/c$ (13.65) $\mathbb{Z}_2 \times \mathbb{Z}_2$	• $\boldsymbol{B}$ ∥ [001] and strain ⊥ [001]
P_C2 (3.6) $\mathbb{Z}_1$	• $\boldsymbol{E}$ ∥ [001] and strain ⊥ [001]
$Pm'm'2$ (25.60) $\mathbb{Z}_2$	• $\boldsymbol{E}$ ∥ [001] and $\boldsymbol{B}$ ∥ [001]
$Pm'm'a$ (51.294) $\mathbb{Z}_2 \times \mathbb{Z}_2$	• $\boldsymbol{B}$ ∥ [001]
P_Inn2 (34.164) $\mathbb{Z}_1$	• $\boldsymbol{E}$ ∥ [001]
$P_S\bar{1}$ (2.7) $\mathbb{Z}_2 \times \mathbb{Z}_2 \times \mathbb{Z}_4$	• strain in generic direction
P_A2/c (13.73) $\mathbb{Z}_2$	• strain ⊥ [100]
P_A2_1/c (14.83) $\mathbb{Z}_2$	• strain ⊥ [010]
P_C2/c (13.74) $\mathbb{Z}_2$	• strain ⊥ [001]

Table. S 431: Subgroups of $Pmna$ (53.321) that can be realized by applying $\boldsymbol{E}/\boldsymbol{B}$ fields and/or uniaxial strains.

Subgroup & SI group	Perturbation
$P2$ (3.1) $\mathbb{Z}_2$	• $\boldsymbol{E}$ ∥ [100] and strain ⊥ [100]<br>• $\boldsymbol{E}$ ∥ [100] and $\boldsymbol{B}$ ∥ [100]
Pc (7.24) $\mathbb{Z}_1$	• $\boldsymbol{E}$ ∥ [100] and strain ⊥ [001]<br>• $\boldsymbol{E}$ ∥ [100] and $\boldsymbol{B}$ ∥ [001]<br>• $\boldsymbol{E}$ ∥ [010] and strain ⊥ [001]<br>• $\boldsymbol{E}$ ∥ [010] and $\boldsymbol{B}$ ∥ [001]<br>• $\boldsymbol{E}$ ⊥ [001]
Pc (7.24) $\mathbb{Z}_1$	• $\boldsymbol{E}$ ∥ [100] and strain ⊥ [010]<br>• $\boldsymbol{E}$ ∥ [100] and $\boldsymbol{B}$ ∥ [010]<br>• $\boldsymbol{E}$ ∥ [001] and strain ⊥ [010]<br>• $\boldsymbol{E}$ ∥ [001] and $\boldsymbol{B}$ ∥ [010]<br>• $\boldsymbol{E}$ ⊥ [010]
$Pnc2$ (30.111) $\mathbb{Z}_1$	• $\boldsymbol{E}$ ∥ [100]

Continued on next page

...continued

Subgroup & SI group	Perturbation
Pm (6.18) $\mathbb{Z}_1$	• $\boldsymbol{E} \parallel [010]$ and strain $\perp [100]$<br>• $\boldsymbol{E} \parallel [010]$ and $\boldsymbol{B} \parallel [100]$<br>• $\boldsymbol{E} \parallel [001]$ and strain $\perp [100]$<br>• $\boldsymbol{E} \parallel [001]$ and $\boldsymbol{B} \parallel [100]$<br>• $\boldsymbol{E} \perp [100]$
$P\bar{1}$ (2.4) $\mathbb{Z}_2 \times \mathbb{Z}_2 \times \mathbb{Z}_2 \times \mathbb{Z}_4$	• strain in generic direction<br>• $\boldsymbol{B} \parallel [100]$ and strain $\perp [010]$<br>• $\boldsymbol{B} \parallel [100]$ and strain $\perp [001]$<br>• $\boldsymbol{B} \parallel [010]$ and strain $\perp [100]$<br>• $\boldsymbol{B} \parallel [010]$ and strain $\perp [001]$<br>• $\boldsymbol{B} \parallel [001]$ and strain $\perp [100]$<br>• $\boldsymbol{B} \parallel [001]$ and strain $\perp [010]$<br>• $\boldsymbol{B}$ in generic direction
$P2/m$ (10.42) $\mathbb{Z}_2 \times \mathbb{Z}_2 \times \mathbb{Z}_2$	• strain $\perp [100]$<br>• $\boldsymbol{B} \parallel [100]$
$P2$ (3.1) $\mathbb{Z}_2$	• $\boldsymbol{E} \parallel [010]$ and strain $\perp [010]$<br>• $\boldsymbol{E} \parallel [010]$ and $\boldsymbol{B} \parallel [010]$
$Pma2$ (28.87) $\mathbb{Z}_1$	• $\boldsymbol{E} \parallel [010]$
$P2/c$ (13.65) $\mathbb{Z}_2 \times \mathbb{Z}_2$	• strain $\perp [010]$<br>• $\boldsymbol{B} \parallel [010]$
$P2_1$ (4.7) $\mathbb{Z}_1$	• $\boldsymbol{E} \parallel [001]$ and strain $\perp [001]$<br>• $\boldsymbol{E} \parallel [001]$ and $\boldsymbol{B} \parallel [001]$
$Pmn2_1$ (31.123) $\mathbb{Z}_1$	• $\boldsymbol{E} \parallel [001]$
$P2_1/c$ (14.75) $\mathbb{Z}_2$	• strain $\perp [001]$<br>• $\boldsymbol{B} \parallel [001]$

Table. S 432: Subgroups of $Pmna1'$ (53.322) that can be realized by applying $\boldsymbol{E}/\boldsymbol{B}$ fields and/or uniaxial strains.

Subgroup & SI group	Perturbation

Continued on next page

...continued

Subgroup & SI group	Perturbation
$P11'$ (1.2) $\mathbb{Z}_1$	• $\boldsymbol{E}$ ∥ [100] and strain in generic direction<br>• $\boldsymbol{E}$ ∥ [010] and strain in generic direction<br>• $\boldsymbol{E}$ ∥ [001] and strain in generic direction<br>• $\boldsymbol{E} \perp$ [100] and strain $\perp$ [010]<br>• $\boldsymbol{E} \perp$ [100] and strain $\perp$ [001]<br>• $\boldsymbol{E} \perp$ [100] and strain in generic direction<br>• $\boldsymbol{E} \perp$ [010] and strain $\perp$ [100]<br>• $\boldsymbol{E} \perp$ [010] and strain $\perp$ [001]<br>• $\boldsymbol{E} \perp$ [010] and strain in generic direction<br>• $\boldsymbol{E} \perp$ [001] and strain $\perp$ [100]<br>• $\boldsymbol{E} \perp$ [001] and strain $\perp$ [010]<br>• $\boldsymbol{E} \perp$ [001] and strain in generic direction<br>• $\boldsymbol{E}$ in generic direction
$P2'$ (3.3) $\mathbb{Z}_1$	• $\boldsymbol{E}$ ∥ [100] and $\boldsymbol{B}$ ∥ [010] and strain $\perp$ [100]<br>• $\boldsymbol{E}$ ∥ [100] and $\boldsymbol{B}$ ∥ [001] and strain $\perp$ [100]<br>• $\boldsymbol{E}$ ∥ [100] and $\boldsymbol{B} \perp$ [100]
$P2$ (3.1) $\mathbb{Z}_2$	• $\boldsymbol{E}$ ∥ [100] and $\boldsymbol{B}$ ∥ [100] and strain $\perp$ [100]
$P21'$ (3.2) $\mathbb{Z}_2$	• $\boldsymbol{E}$ ∥ [100] and strain $\perp$ [100]
Pc' (7.26) $\mathbb{Z}_1$	• $\boldsymbol{E}$ ∥ [100] and $\boldsymbol{B}$ ∥ [100] and strain $\perp$ [001]<br>• $\boldsymbol{E}$ ∥ [100] and $\boldsymbol{B}$ ∥ [010] and strain $\perp$ [001]<br>• $\boldsymbol{E}$ ∥ [100] and $\boldsymbol{B} \perp$ [001]<br>• $\boldsymbol{E}$ ∥ [010] and $\boldsymbol{B}$ ∥ [100] and strain $\perp$ [001]<br>• $\boldsymbol{E}$ ∥ [010] and $\boldsymbol{B}$ ∥ [010] and strain $\perp$ [001]<br>• $\boldsymbol{E}$ ∥ [010] and $\boldsymbol{B} \perp$ [001]<br>• $\boldsymbol{E} \perp$ [001] and $\boldsymbol{B}$ ∥ [100]<br>• $\boldsymbol{E} \perp$ [001] and $\boldsymbol{B}$ ∥ [010]<br>• $\boldsymbol{E} \perp$ [001] and $\boldsymbol{B} \perp$ [001]
Pc (7.24) $\mathbb{Z}_1$	• $\boldsymbol{E}$ ∥ [100] and $\boldsymbol{B}$ ∥ [001] and strain $\perp$ [001]<br>• $\boldsymbol{E}$ ∥ [010] and $\boldsymbol{B}$ ∥ [001] and strain $\perp$ [001]<br>• $\boldsymbol{E} \perp$ [001] and $\boldsymbol{B}$ ∥ [001]
$Pc1'$ (7.25) $\mathbb{Z}_1$	• $\boldsymbol{E}$ ∥ [100] and strain $\perp$ [001]<br>• $\boldsymbol{E}$ ∥ [010] and strain $\perp$ [001]<br>• $\boldsymbol{E} \perp$ [001]

Continued on next page

…continued

Subgroup & SI group	Perturbation
Pc' (7.26) $\mathbb{Z}_1$	• $\boldsymbol{E}$ ∥ [100] and $\boldsymbol{B}$ ∥ [100] and strain ⊥ [010]<br>• $\boldsymbol{E}$ ∥ [100] and $\boldsymbol{B}$ ∥ [001] and strain ⊥ [010]<br>• $\boldsymbol{E}$ ∥ [100] and $\boldsymbol{B}$ ⊥ [010]<br>• $\boldsymbol{E}$ ∥ [001] and $\boldsymbol{B}$ ∥ [100] and strain ⊥ [010]<br>• $\boldsymbol{E}$ ∥ [001] and $\boldsymbol{B}$ ∥ [001] and strain ⊥ [010]<br>• $\boldsymbol{E}$ ∥ [001] and $\boldsymbol{B}$ ⊥ [010]<br>• $\boldsymbol{E}$ ⊥ [010] and $\boldsymbol{B}$ ∥ [100]<br>• $\boldsymbol{E}$ ⊥ [010] and $\boldsymbol{B}$ ∥ [001]<br>• $\boldsymbol{E}$ ⊥ [010] and $\boldsymbol{B}$ ⊥ [010]
$Pn'c'2$ (30.115) $\mathbb{Z}_1$	• $\boldsymbol{E}$ ∥ [100] and $\boldsymbol{B}$ ∥ [100]
$Pn'c2'$ (30.113) $\mathbb{Z}_1$	• $\boldsymbol{E}$ ∥ [100] and $\boldsymbol{B}$ ∥ [001]
Pc (7.24) $\mathbb{Z}_1$	• $\boldsymbol{E}$ ∥ [100] and $\boldsymbol{B}$ ∥ [010] and strain ⊥ [010]<br>• $\boldsymbol{E}$ ∥ [001] and $\boldsymbol{B}$ ∥ [010] and strain ⊥ [010]<br>• $\boldsymbol{E}$ ⊥ [010] and $\boldsymbol{B}$ ∥ [010]
$Pnc'2'$ (30.114) $\mathbb{Z}_1$	• $\boldsymbol{E}$ ∥ [100] and $\boldsymbol{B}$ ∥ [010]
$Pc1'$ (7.25) $\mathbb{Z}_1$	• $\boldsymbol{E}$ ∥ [100] and strain ⊥ [010]<br>• $\boldsymbol{E}$ ∥ [001] and strain ⊥ [010]<br>• $\boldsymbol{E}$ ⊥ [010]
$Pnc21'$ (30.112) $\mathbb{Z}_1$	• $\boldsymbol{E}$ ∥ [100]
Pm' (6.20) $\mathbb{Z}_1$	• $\boldsymbol{E}$ ∥ [010] and $\boldsymbol{B}$ ∥ [010] and strain ⊥ [100]<br>• $\boldsymbol{E}$ ∥ [010] and $\boldsymbol{B}$ ∥ [001] and strain ⊥ [100]<br>• $\boldsymbol{E}$ ∥ [010] and $\boldsymbol{B}$ ⊥ [100]<br>• $\boldsymbol{E}$ ∥ [001] and $\boldsymbol{B}$ ∥ [010] and strain ⊥ [100]<br>• $\boldsymbol{E}$ ∥ [001] and $\boldsymbol{B}$ ∥ [001] and strain ⊥ [100]<br>• $\boldsymbol{E}$ ∥ [001] and $\boldsymbol{B}$ ⊥ [100]<br>• $\boldsymbol{E}$ ⊥ [100] and $\boldsymbol{B}$ ∥ [010]<br>• $\boldsymbol{E}$ ⊥ [100] and $\boldsymbol{B}$ ∥ [001]<br>• $\boldsymbol{E}$ ⊥ [100] and $\boldsymbol{B}$ ⊥ [100]
Pm (6.18) $\mathbb{Z}_1$	• $\boldsymbol{E}$ ∥ [010] and $\boldsymbol{B}$ ∥ [100] and strain ⊥ [100]<br>• $\boldsymbol{E}$ ∥ [001] and $\boldsymbol{B}$ ∥ [100] and strain ⊥ [100]<br>• $\boldsymbol{E}$ ⊥ [100] and $\boldsymbol{B}$ ∥ [100]
$Pm1'$ (6.19) $\mathbb{Z}_1$	• $\boldsymbol{E}$ ∥ [010] and strain ⊥ [100]<br>• $\boldsymbol{E}$ ∥ [001] and strain ⊥ [100]<br>• $\boldsymbol{E}$ ⊥ [100]

Continued on next page

...continued

Subgroup & SI group	Perturbation
$P\bar{1}$ (2.4) $\mathbb{Z}_2 \times \mathbb{Z}_2 \times \mathbb{Z}_2 \times \mathbb{Z}_4$	• $\boldsymbol{B}$ || [100] and strain in generic direction<br>• $\boldsymbol{B}$ || [010] and strain in generic direction<br>• $\boldsymbol{B}$ || [001] and strain in generic direction<br>• $\boldsymbol{B} \perp$ [100] and strain $\perp$ [010]<br>• $\boldsymbol{B} \perp$ [100] and strain $\perp$ [001]<br>• $\boldsymbol{B} \perp$ [100] and strain in generic direction<br>• $\boldsymbol{B} \perp$ [010] and strain $\perp$ [100]<br>• $\boldsymbol{B} \perp$ [010] and strain $\perp$ [001]<br>• $\boldsymbol{B} \perp$ [010] and strain in generic direction<br>• $\boldsymbol{B} \perp$ [001] and strain $\perp$ [100]<br>• $\boldsymbol{B} \perp$ [001] and strain $\perp$ [010]<br>• $\boldsymbol{B} \perp$ [001] and strain in generic direction<br>• $\boldsymbol{B}$ in generic direction
$P2'/m'$ (10.46) $\mathbb{Z}_2 \times \mathbb{Z}_2 \times \mathbb{Z}_2 \times \mathbb{Z}_4$	• $\boldsymbol{B}$ || [010] and strain $\perp$ [100]<br>• $\boldsymbol{B}$ || [001] and strain $\perp$ [100]<br>• $\boldsymbol{B} \perp$ [100]
$P2/m$ (10.42) $\mathbb{Z}_2 \times \mathbb{Z}_2 \times \mathbb{Z}_2$	• $\boldsymbol{B}$ || [100] and strain $\perp$ [100]
$P\bar{1}1'$ (2.5) $\mathbb{Z}_2 \times \mathbb{Z}_2 \times \mathbb{Z}_2 \times \mathbb{Z}_4$	• strain in generic direction
$P2/m1'$ (10.43) $\mathbb{Z}_2 \times \mathbb{Z}_2 \times \mathbb{Z}_2$	• strain $\perp$ [100]
$P2'$ (3.3) $\mathbb{Z}_1$	• $\boldsymbol{E}$ || [010] and $\boldsymbol{B}$ || [100] and strain $\perp$ [010]<br>• $\boldsymbol{E}$ || [010] and $\boldsymbol{B}$ || [001] and strain $\perp$ [010]<br>• $\boldsymbol{E}$ || [010] and $\boldsymbol{B} \perp$ [010]
$Pm'a2'$ (28.89) $\mathbb{Z}_1$	• $\boldsymbol{E}$ || [010] and $\boldsymbol{B}$ || [001]
$Pma'2'$ (28.90) $\mathbb{Z}_1$	• $\boldsymbol{E}$ || [010] and $\boldsymbol{B}$ || [100]
$P2'/c'$ (13.69) $\mathbb{Z}_2 \times \mathbb{Z}_2 \times \mathbb{Z}_4$	• $\boldsymbol{B}$ || [100] and strain $\perp$ [010]<br>• $\boldsymbol{B}$ || [001] and strain $\perp$ [010]<br>• $\boldsymbol{B} \perp$ [010]
$P2$ (3.1) $\mathbb{Z}_2$	• $\boldsymbol{E}$ || [010] and $\boldsymbol{B}$ || [010] and strain $\perp$ [010]
$Pm'a'2$ (28.91) $\mathbb{Z}_2$	• $\boldsymbol{E}$ || [010] and $\boldsymbol{B}$ || [010]
$P2/c$ (13.65) $\mathbb{Z}_2 \times \mathbb{Z}_2$	• $\boldsymbol{B}$ || [010] and strain $\perp$ [010]
$P21'$ (3.2) $\mathbb{Z}_2$	• $\boldsymbol{E}$ || [010] and strain $\perp$ [010]
$Pma21'$ (28.88) $\mathbb{Z}_1$	• $\boldsymbol{E}$ || [010]
$P2/c1'$ (13.66) $\mathbb{Z}_2 \times \mathbb{Z}_2$	• strain $\perp$ [010]

Continued on next page

...continued

Subgroup & SI group	Perturbation
$P2_1'$ (4.9) $\mathbb{Z}_1$	• $\boldsymbol{E} \parallel [001]$ and $\boldsymbol{B} \parallel [100]$ and strain $\perp [001]$<br>• $\boldsymbol{E} \parallel [001]$ and $\boldsymbol{B} \parallel [010]$ and strain $\perp [001]$<br>• $\boldsymbol{E} \parallel [001]$ and $\boldsymbol{B} \perp [001]$
$Pm'n2_1'$ (31.125) $\mathbb{Z}_1$	• $\boldsymbol{E} \parallel [001]$ and $\boldsymbol{B} \parallel [010]$
$Pmn'2_1'$ (31.126) $\mathbb{Z}_1$	• $\boldsymbol{E} \parallel [001]$ and $\boldsymbol{B} \parallel [100]$
$P2_1'/c'$ (14.79) $\mathbb{Z}_2 \times \mathbb{Z}_4$	• $\boldsymbol{B} \parallel [100]$ and strain $\perp [001]$<br>• $\boldsymbol{B} \parallel [010]$ and strain $\perp [001]$<br>• $\boldsymbol{B} \perp [001]$
$Pmn'a'$ (53.327) $\mathbb{Z}_2 \times \mathbb{Z}_2$	• $\boldsymbol{B} \parallel [100]$
$Pm'na'$ (53.328) $\mathbb{Z}_2 \times \mathbb{Z}_2$	• $\boldsymbol{B} \parallel [010]$
$P2_1$ (4.7) $\mathbb{Z}_1$	• $\boldsymbol{E} \parallel [001]$ and $\boldsymbol{B} \parallel [001]$ and strain $\perp [001]$
$Pm'n'2_1$ (31.127) $\mathbb{Z}_1$	• $\boldsymbol{E} \parallel [001]$ and $\boldsymbol{B} \parallel [001]$
$P2_1/c$ (14.75) $\mathbb{Z}_2$	• $\boldsymbol{B} \parallel [001]$ and strain $\perp [001]$
$Pm'n'a$ (53.326) $\mathbb{Z}_2$	• $\boldsymbol{B} \parallel [001]$
$P2_11'$ (4.8) $\mathbb{Z}_1$	• $\boldsymbol{E} \parallel [001]$ and strain $\perp [001]$
$Pmn2_11'$ (31.124) $\mathbb{Z}_1$	• $\boldsymbol{E} \parallel [001]$
$P2_1/c1'$ (14.76) $\mathbb{Z}_2$	• strain $\perp [001]$

Table. S 433: Subgroups of $Pm'na$ (53.323) that can be realized by applying $\boldsymbol{E}/\boldsymbol{B}$ fields and/or uniaxial strains.

Subgroup & SI group	Perturbation
$P2$ (3.1) $\mathbb{Z}_2$	• $\boldsymbol{B} \parallel [100]$ and strain $\perp [100]$<br>• $\boldsymbol{E} \parallel [100]$ and strain $\perp [100]$<br>• $\boldsymbol{E} \parallel [100]$ and $\boldsymbol{B} \parallel [100]$
Pc (7.24) $\mathbb{Z}_1$	• $\boldsymbol{B} \parallel [001]$ and strain $\perp [001]$<br>• $\boldsymbol{E} \parallel [100]$ and strain $\perp [001]$<br>• $\boldsymbol{E} \parallel [100]$ and $\boldsymbol{B} \parallel [001]$<br>• $\boldsymbol{E} \parallel [010]$ and strain $\perp [001]$<br>• $\boldsymbol{E} \perp [001]$

Continued on next page

...continued

Subgroup & SI group	Perturbation
Pc (7.24) $\mathbb{Z}_1$	• $\boldsymbol{B} \parallel [010]$ and strain $\perp [010]$<br>• $\boldsymbol{E} \parallel [100]$ and strain $\perp [010]$<br>• $\boldsymbol{E} \parallel [100]$ and $\boldsymbol{B} \parallel [010]$<br>• $\boldsymbol{E} \parallel [001]$ and strain $\perp [010]$<br>• $\boldsymbol{E} \perp [010]$
$Pnc2$ (30.111) $\mathbb{Z}_1$	• $\boldsymbol{E} \parallel [100]$
Pm' (6.20) $\mathbb{Z}_1$	• $\boldsymbol{B} \parallel [010]$ and strain $\perp [100]$<br>• $\boldsymbol{B} \parallel [001]$ and strain $\perp [100]$<br>• $\boldsymbol{B} \perp [100]$<br>• $\boldsymbol{E} \parallel [010]$ and strain $\perp [100]$<br>• $\boldsymbol{E} \parallel [010]$ and $\boldsymbol{B} \parallel [010]$<br>• $\boldsymbol{E} \parallel [001]$ and strain $\perp [100]$<br>• $\boldsymbol{E} \parallel [001]$ and $\boldsymbol{B} \parallel [001]$<br>• $\boldsymbol{E} \perp [100]$
$P\bar{1}'$ (2.6) $\mathbb{Z}_1$	• strain in generic direction
$P2/m'$ (10.45) $\mathbb{Z}_2$	• strain $\perp [100]$
$P2'$ (3.3) $\mathbb{Z}_1$	• $\boldsymbol{B} \parallel [100]$ and strain $\perp [010]$<br>• $\boldsymbol{B} \parallel [001]$ and strain $\perp [010]$<br>• $\boldsymbol{B} \perp [010]$<br>• $\boldsymbol{E} \parallel [010]$ and strain $\perp [010]$<br>• $\boldsymbol{E} \parallel [010]$ and $\boldsymbol{B} \parallel [100]$
$Pm'a2'$ (28.89) $\mathbb{Z}_1$	• $\boldsymbol{B} \parallel [001]$<br>• $\boldsymbol{E} \parallel [010]$
$P2'/c$ (13.67) $\mathbb{Z}_1$	• strain $\perp [010]$
$P2_1'$ (4.9) $\mathbb{Z}_1$	• $\boldsymbol{B} \parallel [100]$ and strain $\perp [001]$<br>• $\boldsymbol{B} \parallel [010]$ and strain $\perp [001]$<br>• $\boldsymbol{B} \perp [001]$<br>• $\boldsymbol{E} \parallel [001]$ and strain $\perp [001]$<br>• $\boldsymbol{E} \parallel [001]$ and $\boldsymbol{B} \parallel [100]$
$Pm'n2_1'$ (31.125) $\mathbb{Z}_1$	• $\boldsymbol{B} \parallel [010]$<br>• $\boldsymbol{E} \parallel [001]$
$P2_1'/c$ (14.77) $\mathbb{Z}_1$	• strain $\perp [001]$
$P22'2_1'$ (17.10) $\mathbb{Z}_2$	• $\boldsymbol{B} \parallel [100]$

Table. S 434: Subgroups of $Pmn'a$ (53.324) that can be realized by applying $\boldsymbol{E}/\boldsymbol{B}$ fields and/or uniaxial strains.

Subgroup & SI group	Perturbation
$P2'$ (3.3) $\mathbb{Z}_1$	• $\boldsymbol{B} \parallel [010]$ and strain $\perp [100]$<br>• $\boldsymbol{B} \parallel [001]$ and strain $\perp [100]$<br>• $\boldsymbol{B} \perp [100]$<br>• $\boldsymbol{E} \parallel [100]$ and strain $\perp [100]$<br>• $\boldsymbol{E} \parallel [100]$ and $\boldsymbol{B} \parallel [010]$
Pc (7.24) $\mathbb{Z}_1$	• $\boldsymbol{B} \parallel [001]$ and strain $\perp [001]$<br>• $\boldsymbol{E} \parallel [100]$ and strain $\perp [001]$<br>• $\boldsymbol{E} \parallel [010]$ and strain $\perp [001]$<br>• $\boldsymbol{E} \parallel [010]$ and $\boldsymbol{B} \parallel [001]$<br>• $\boldsymbol{E} \perp [001]$
Pc' (7.26) $\mathbb{Z}_1$	• $\boldsymbol{B} \parallel [100]$ and strain $\perp [010]$<br>• $\boldsymbol{B} \parallel [001]$ and strain $\perp [010]$<br>• $\boldsymbol{B} \perp [010]$<br>• $\boldsymbol{E} \parallel [100]$ and strain $\perp [010]$<br>• $\boldsymbol{E} \parallel [100]$ and $\boldsymbol{B} \parallel [100]$<br>• $\boldsymbol{E} \parallel [001]$ and strain $\perp [010]$<br>• $\boldsymbol{E} \parallel [001]$ and $\boldsymbol{B} \parallel [001]$<br>• $\boldsymbol{E} \perp [010]$
$Pn'c2'$ (30.113) $\mathbb{Z}_1$	• $\boldsymbol{B} \parallel [001]$<br>• $\boldsymbol{E} \parallel [100]$
Pm (6.18) $\mathbb{Z}_1$	• $\boldsymbol{B} \parallel [100]$ and strain $\perp [100]$<br>• $\boldsymbol{E} \parallel [010]$ and strain $\perp [100]$<br>• $\boldsymbol{E} \parallel [010]$ and $\boldsymbol{B} \parallel [100]$<br>• $\boldsymbol{E} \parallel [001]$ and strain $\perp [100]$<br>• $\boldsymbol{E} \perp [100]$
$P\bar{1}'$ (2.6) $\mathbb{Z}_1$	• strain in generic direction
$P2'/m$ (10.44) $\mathbb{Z}_1$	• strain $\perp [100]$
$P2$ (3.1) $\mathbb{Z}_2$	• $\boldsymbol{B} \parallel [010]$ and strain $\perp [010]$<br>• $\boldsymbol{E} \parallel [010]$ and strain $\perp [010]$<br>• $\boldsymbol{E} \parallel [010]$ and $\boldsymbol{B} \parallel [010]$
$Pma2$ (28.87) $\mathbb{Z}_1$	• $\boldsymbol{E} \parallel [010]$
$P2/c'$ (13.68) $\mathbb{Z}_2$	• strain $\perp [010]$

Continued on next page

. . . continued

Subgroup & SI group	Perturbation
$P2_1'$ (4.9) $\mathbb{Z}_1$	• $\boldsymbol{B} \parallel [100]$ and strain $\perp [001]$<br>• $\boldsymbol{B} \parallel [010]$ and strain $\perp [001]$<br>• $\boldsymbol{B} \perp [001]$<br>• $\boldsymbol{E} \parallel [001]$ and strain $\perp [001]$<br>• $\boldsymbol{E} \parallel [001]$ and $\boldsymbol{B} \parallel [010]$
$Pmn'2_1'$ (31.126) $\mathbb{Z}_1$	• $\boldsymbol{B} \parallel [100]$<br>• $\boldsymbol{E} \parallel [001]$
$P2_1'/c$ (14.77) $\mathbb{Z}_1$	• strain $\perp [001]$
$P22'2_1'$ (17.10) $\mathbb{Z}_2$	• $\boldsymbol{B} \parallel [010]$

Table. S 435: Subgroups of $Pmna'$ (53.325) that can be realized by applying $\boldsymbol{E}/\boldsymbol{B}$ fields and/or uniaxial strains.

Subgroup & SI group	Perturbation
$P2'$ (3.3) $\mathbb{Z}_1$	• $\boldsymbol{B} \parallel [010]$ and strain $\perp [100]$<br>• $\boldsymbol{B} \parallel [001]$ and strain $\perp [100]$<br>• $\boldsymbol{B} \perp [100]$<br>• $\boldsymbol{E} \parallel [100]$ and strain $\perp [100]$<br>• $\boldsymbol{E} \parallel [100]$ and $\boldsymbol{B} \parallel [001]$
Pc' (7.26) $\mathbb{Z}_1$	• $\boldsymbol{B} \parallel [100]$ and strain $\perp [001]$<br>• $\boldsymbol{B} \parallel [010]$ and strain $\perp [001]$<br>• $\boldsymbol{B} \perp [001]$<br>• $\boldsymbol{E} \parallel [100]$ and strain $\perp [001]$<br>• $\boldsymbol{E} \parallel [100]$ and $\boldsymbol{B} \parallel [100]$<br>• $\boldsymbol{E} \parallel [010]$ and strain $\perp [001]$<br>• $\boldsymbol{E} \parallel [010]$ and $\boldsymbol{B} \parallel [010]$<br>• $\boldsymbol{E} \perp [001]$
Pc (7.24) $\mathbb{Z}_1$	• $\boldsymbol{B} \parallel [010]$ and strain $\perp [010]$<br>• $\boldsymbol{E} \parallel [100]$ and strain $\perp [010]$<br>• $\boldsymbol{E} \parallel [001]$ and strain $\perp [010]$<br>• $\boldsymbol{E} \parallel [001]$ and $\boldsymbol{B} \parallel [010]$<br>• $\boldsymbol{E} \perp [010]$
$Pnc'2'$ (30.114) $\mathbb{Z}_1$	• $\boldsymbol{B} \parallel [010]$<br>• $\boldsymbol{E} \parallel [100]$

Continued on next page

...continued

Subgroup & SI group	Perturbation
Pm (6.18) $\mathbb{Z}_1$	• $\boldsymbol{B} \parallel [100]$ and strain $\perp [100]$<br>• $\boldsymbol{E} \parallel [010]$ and strain $\perp [100]$<br>• $\boldsymbol{E} \parallel [001]$ and strain $\perp [100]$<br>• $\boldsymbol{E} \parallel [001]$ and $\boldsymbol{B} \parallel [100]$<br>• $\boldsymbol{E} \perp [100]$
$P\bar{1}'$ (2.6) $\mathbb{Z}_1$	• strain in generic direction
$P2'/m$ (10.44) $\mathbb{Z}_1$	• strain $\perp [100]$
$P2'$ (3.3) $\mathbb{Z}_1$	• $\boldsymbol{B} \parallel [100]$ and strain $\perp [010]$<br>• $\boldsymbol{B} \parallel [001]$ and strain $\perp [010]$<br>• $\boldsymbol{B} \perp [010]$<br>• $\boldsymbol{E} \parallel [010]$ and strain $\perp [010]$<br>• $\boldsymbol{E} \parallel [010]$ and $\boldsymbol{B} \parallel [001]$
$Pma'2'$ (28.90) $\mathbb{Z}_1$	• $\boldsymbol{B} \parallel [100]$<br>• $\boldsymbol{E} \parallel [010]$
$P2'/c$ (13.67) $\mathbb{Z}_1$	• strain $\perp [010]$
$P2_1$ (4.7) $\mathbb{Z}_1$	• $\boldsymbol{B} \parallel [001]$ and strain $\perp [001]$<br>• $\boldsymbol{E} \parallel [001]$ and strain $\perp [001]$<br>• $\boldsymbol{E} \parallel [001]$ and $\boldsymbol{B} \parallel [001]$
$Pmn2_1$ (31.123) $\mathbb{Z}_1$	• $\boldsymbol{E} \parallel [001]$
$P2_1/c'$ (14.78) $\mathbb{Z}_1$	• strain $\perp [001]$
$P2'2'2_1$ (17.9) $\mathbb{Z}_1$	• $\boldsymbol{B} \parallel [001]$

Table. S 436: Subgroups of $Pm'n'a$ (53.326) that can be realized by applying $\boldsymbol{E}/\boldsymbol{B}$ fields and/or uniaxial strains.

Subgroup & SI group	Perturbation
$P2'$ (3.3) $\mathbb{Z}_1$	• $\boldsymbol{E} \parallel [100]$ and strain $\perp [100]$<br>• $\boldsymbol{E} \parallel [100]$ and ($\boldsymbol{B} \parallel [010]$ or $\boldsymbol{B} \perp [100]$)
Pc (7.24) $\mathbb{Z}_1$	• $\boldsymbol{E} \parallel [100]$ and strain $\perp [001]$<br>• $\boldsymbol{E} \parallel [010]$ and strain $\perp [001]$<br>• $\boldsymbol{E} \perp [001]$

Continued on next page

...continued

Subgroup & SI group	Perturbation
Pc' (7.26) $\mathbb{Z}_1$	• $\boldsymbol{E}$ ∥ [100] and strain ⊥ [010]<br>• $\boldsymbol{E}$ ∥ [100] and ($\boldsymbol{B}$ ∥ [100] or $\boldsymbol{B}$ ⊥ [010])<br>• $\boldsymbol{E}$ ∥ [001] and strain ⊥ [010]<br>• $\boldsymbol{E}$ ∥ [001] and ($\boldsymbol{B}$ ∥ [100] or $\boldsymbol{B}$ ⊥ [010])<br>• $\boldsymbol{E}$ ⊥ [010]
$Pn'c2'$ (30.113) $\mathbb{Z}_1$	• $\boldsymbol{E}$ ∥ [100]
Pm' (6.20) $\mathbb{Z}_1$	• $\boldsymbol{E}$ ∥ [010] and strain ⊥ [100]<br>• $\boldsymbol{E}$ ∥ [010] and ($\boldsymbol{B}$ ∥ [010] or $\boldsymbol{B}$ ⊥ [100])<br>• $\boldsymbol{E}$ ∥ [001] and strain ⊥ [100]<br>• $\boldsymbol{E}$ ∥ [001] and ($\boldsymbol{B}$ ∥ [010] or $\boldsymbol{B}$ ⊥ [100])<br>• $\boldsymbol{E}$ ⊥ [100]
$P\bar{1}$ (2.4) $\mathbb{Z}_2 \times \mathbb{Z}_2 \times \mathbb{Z}_2 \times \mathbb{Z}_4$	• strain in generic direction<br>• ($\boldsymbol{B}$ ∥ [100] or $\boldsymbol{B}$ ⊥ [010]) and strain ⊥ [100]<br>• ($\boldsymbol{B}$ ∥ [100] or $\boldsymbol{B}$ ⊥ [010]) and strain ⊥ [001]<br>• ($\boldsymbol{B}$ ∥ [010] or $\boldsymbol{B}$ ⊥ [100]) and strain ⊥ [010]<br>• ($\boldsymbol{B}$ ∥ [010] or $\boldsymbol{B}$ ⊥ [100]) and strain ⊥ [001]<br>• $\boldsymbol{B}$ in generic direction
$P2'/m'$ (10.46) $\mathbb{Z}_2 \times \mathbb{Z}_2 \times \mathbb{Z}_2 \times \mathbb{Z}_4$	• strain ⊥ [100]<br>• ($\boldsymbol{B}$ ∥ [010] or $\boldsymbol{B}$ ⊥ [100])
$P2'$ (3.3) $\mathbb{Z}_1$	• $\boldsymbol{E}$ ∥ [010] and strain ⊥ [010]<br>• $\boldsymbol{E}$ ∥ [010] and ($\boldsymbol{B}$ ∥ [100] or $\boldsymbol{B}$ ⊥ [010])
$Pm'a2'$ (28.89) $\mathbb{Z}_1$	• $\boldsymbol{E}$ ∥ [010]
$P2'/c'$ (13.69) $\mathbb{Z}_2 \times \mathbb{Z}_2 \times \mathbb{Z}_4$	• strain ⊥ [010]<br>• ($\boldsymbol{B}$ ∥ [100] or $\boldsymbol{B}$ ⊥ [010])
$P2_1$ (4.7) $\mathbb{Z}_1$	• $\boldsymbol{E}$ ∥ [001] and strain ⊥ [001]
$Pm'n'2_1$ (31.127) $\mathbb{Z}_1$	• $\boldsymbol{E}$ ∥ [001]
$P2_1/c$ (14.75) $\mathbb{Z}_2$	• strain ⊥ [001]

Table. S 437: Subgroups of $Pmn'a'$ (53.327) that can be realized by applying $\boldsymbol{E}/\boldsymbol{B}$ fields and/or uniaxial strains.

Subgroup & SI group	Perturbation
$P2$ (3.1) $\mathbb{Z}_2$	• $\boldsymbol{E}$ ∥ [100] and strain ⊥ [100]

Continued on next page

…continued

Subgroup & SI group	Perturbation
Pc' (7.26) $\mathbb{Z}_1$	• $\boldsymbol{E} \parallel [100]$ and strain $\perp [001]$<br>• $\boldsymbol{E} \parallel [100]$ and ($\boldsymbol{B} \parallel [010]$ or $\boldsymbol{B} \perp [001]$)<br>• $\boldsymbol{E} \parallel [010]$ and strain $\perp [001]$<br>• $\boldsymbol{E} \parallel [010]$ and ($\boldsymbol{B} \parallel [010]$ or $\boldsymbol{B} \perp [001]$)<br>• $\boldsymbol{E} \perp [001]$
Pc' (7.26) $\mathbb{Z}_1$	• $\boldsymbol{E} \parallel [100]$ and strain $\perp [010]$<br>• $\boldsymbol{E} \parallel [100]$ and ($\boldsymbol{B} \parallel [001]$ or $\boldsymbol{B} \perp [010]$)<br>• $\boldsymbol{E} \parallel [001]$ and strain $\perp [010]$<br>• $\boldsymbol{E} \parallel [001]$ and ($\boldsymbol{B} \parallel [001]$ or $\boldsymbol{B} \perp [010]$)<br>• $\boldsymbol{E} \perp [010]$
$Pn'c'2$ (30.115) $\mathbb{Z}_1$	• $\boldsymbol{E} \parallel [100]$
Pm (6.18) $\mathbb{Z}_1$	• $\boldsymbol{E} \parallel [010]$ and strain $\perp [100]$<br>• $\boldsymbol{E} \parallel [001]$ and strain $\perp [100]$<br>• $\boldsymbol{E} \perp [100]$
$P\bar{1}$ (2.4) $\mathbb{Z}_2 \times \mathbb{Z}_2 \times \mathbb{Z}_2 \times \mathbb{Z}_4$	• strain in generic direction<br>• ($\boldsymbol{B} \parallel [010]$ or $\boldsymbol{B} \perp [001]$) and strain $\perp [100]$<br>• ($\boldsymbol{B} \parallel [010]$ or $\boldsymbol{B} \perp [001]$) and strain $\perp [010]$<br>• ($\boldsymbol{B} \parallel [001]$ or $\boldsymbol{B} \perp [010]$) and strain $\perp [100]$<br>• ($\boldsymbol{B} \parallel [001]$ or $\boldsymbol{B} \perp [010]$) and strain $\perp [001]$<br>• $\boldsymbol{B}$ in generic direction
$P2/m$ (10.42) $\mathbb{Z}_2 \times \mathbb{Z}_2 \times \mathbb{Z}_2$	• strain $\perp [100]$
$P2'$ (3.3) $\mathbb{Z}_1$	• $\boldsymbol{E} \parallel [010]$ and strain $\perp [010]$<br>• $\boldsymbol{E} \parallel [010]$ and ($\boldsymbol{B} \parallel [001]$ or $\boldsymbol{B} \perp [010]$)
$Pma'2'$ (28.90) $\mathbb{Z}_1$	• $\boldsymbol{E} \parallel [010]$
$P2'/c'$ (13.69) $\mathbb{Z}_2 \times \mathbb{Z}_2 \times \mathbb{Z}_4$	• strain $\perp [010]$<br>• ($\boldsymbol{B} \parallel [001]$ or $\boldsymbol{B} \perp [010]$)
$P2_1'$ (4.9) $\mathbb{Z}_1$	• $\boldsymbol{E} \parallel [001]$ and strain $\perp [001]$<br>• $\boldsymbol{E} \parallel [001]$ and ($\boldsymbol{B} \parallel [010]$ or $\boldsymbol{B} \perp [001]$)
$Pmn'2_1'$ (31.126) $\mathbb{Z}_1$	• $\boldsymbol{E} \parallel [001]$
$P2_1'/c'$ (14.79) $\mathbb{Z}_2 \times \mathbb{Z}_4$	• strain $\perp [001]$<br>• ($\boldsymbol{B} \parallel [010]$ or $\boldsymbol{B} \perp [001]$)

Table. S 438: Subgroups of $Pm'na'$ (53.328) that can be realized by applying $\boldsymbol{E}/\boldsymbol{B}$ fields and/or uniaxial strains.

Subgroup & SI group	Perturbation
$P2'$ (3.3) $\mathbb{Z}_1$	• $\boldsymbol{E} \parallel [100]$ and strain $\perp [100]$<br>• $\boldsymbol{E} \parallel [100]$ and ($\boldsymbol{B} \parallel [001]$ or $\boldsymbol{B} \perp [100]$)
Pc' (7.26) $\mathbb{Z}_1$	• $\boldsymbol{E} \parallel [100]$ and strain $\perp [001]$<br>• $\boldsymbol{E} \parallel [100]$ and ($\boldsymbol{B} \parallel [100]$ or $\boldsymbol{B} \perp [001]$)<br>• $\boldsymbol{E} \parallel [010]$ and strain $\perp [001]$<br>• $\boldsymbol{E} \parallel [010]$ and ($\boldsymbol{B} \parallel [100]$ or $\boldsymbol{B} \perp [001]$)<br>• $\boldsymbol{E} \perp [001]$
Pc (7.24) $\mathbb{Z}_1$	• $\boldsymbol{E} \parallel [100]$ and strain $\perp [010]$<br>• $\boldsymbol{E} \parallel [001]$ and strain $\perp [010]$<br>• $\boldsymbol{E} \perp [010]$
$Pnc'2'$ (30.114) $\mathbb{Z}_1$	• $\boldsymbol{E} \parallel [100]$
Pm' (6.20) $\mathbb{Z}_1$	• $\boldsymbol{E} \parallel [010]$ and strain $\perp [100]$<br>• $\boldsymbol{E} \parallel [010]$ and ($\boldsymbol{B} \parallel [001]$ or $\boldsymbol{B} \perp [100]$)<br>• $\boldsymbol{E} \parallel [001]$ and strain $\perp [100]$<br>• $\boldsymbol{E} \parallel [001]$ and ($\boldsymbol{B} \parallel [001]$ or $\boldsymbol{B} \perp [100]$)<br>• $\boldsymbol{E} \perp [100]$
$P\bar{1}$ (2.4) $\mathbb{Z}_2 \times \mathbb{Z}_2 \times \mathbb{Z}_2 \times \mathbb{Z}_4$	• strain in generic direction<br>• ($\boldsymbol{B} \parallel [100]$ or $\boldsymbol{B} \perp [001]$) and strain $\perp [100]$<br>• ($\boldsymbol{B} \parallel [100]$ or $\boldsymbol{B} \perp [001]$) and strain $\perp [010]$<br>• ($\boldsymbol{B} \parallel [001]$ or $\boldsymbol{B} \perp [100]$) and strain $\perp [010]$<br>• ($\boldsymbol{B} \parallel [001]$ or $\boldsymbol{B} \perp [100]$) and strain $\perp [001]$<br>• $\boldsymbol{B}$ in generic direction
$P2'/m'$ (10.46) $\mathbb{Z}_2 \times \mathbb{Z}_2 \times \mathbb{Z}_2 \times \mathbb{Z}_4$	• strain $\perp [100]$<br>• ($\boldsymbol{B} \parallel [001]$ or $\boldsymbol{B} \perp [100]$)
$P2$ (3.1) $\mathbb{Z}_2$	• $\boldsymbol{E} \parallel [010]$ and strain $\perp [010]$
$Pm'a'2$ (28.91) $\mathbb{Z}_2$	• $\boldsymbol{E} \parallel [010]$
$P2/c$ (13.65) $\mathbb{Z}_2 \times \mathbb{Z}_2$	• strain $\perp [010]$
$P2_1'$ (4.9) $\mathbb{Z}_1$	• $\boldsymbol{E} \parallel [001]$ and strain $\perp [001]$<br>• $\boldsymbol{E} \parallel [001]$ and ($\boldsymbol{B} \parallel [100]$ or $\boldsymbol{B} \perp [001]$)
$Pm'n2_1'$ (31.125) $\mathbb{Z}_1$	• $\boldsymbol{E} \parallel [001]$
$P2_1'/c'$ (14.79) $\mathbb{Z}_2 \times \mathbb{Z}_4$	• strain $\perp [001]$<br>• ($\boldsymbol{B} \parallel [100]$ or $\boldsymbol{B} \perp [001]$)

Table. S 439: Subgroups of $Pm'n'a'$ (53.329) that can be realized by applying $\boldsymbol{E}/\boldsymbol{B}$ fields and/or uniaxial strains.

Subgroup & SI group	Perturbation
$P2$ (3.1) $\mathbb{Z}_2$	• $\boldsymbol{B} \parallel [100]$ and strain $\perp [100]$<br>• $\boldsymbol{E} \parallel [100]$ and strain $\perp [100]$
Pc' (7.26) $\mathbb{Z}_1$	• $\boldsymbol{B} \parallel [100]$ and strain $\perp [001]$<br>• $\boldsymbol{B} \parallel [010]$ and strain $\perp [001]$<br>• $\boldsymbol{B} \perp [001]$<br>• $\boldsymbol{E} \parallel [100]$ and strain $\perp [001]$<br>• $\boldsymbol{E} \parallel [100]$ and $\boldsymbol{B} \parallel [010]$<br>• $\boldsymbol{E} \parallel [010]$ and strain $\perp [001]$<br>• $\boldsymbol{E} \parallel [010]$ and $\boldsymbol{B} \parallel [100]$<br>• $\boldsymbol{E} \perp [001]$
Pc' (7.26) $\mathbb{Z}_1$	• $\boldsymbol{B} \parallel [100]$ and strain $\perp [010]$<br>• $\boldsymbol{B} \parallel [001]$ and strain $\perp [010]$<br>• $\boldsymbol{B} \perp [010]$<br>• $\boldsymbol{E} \parallel [100]$ and strain $\perp [010]$<br>• $\boldsymbol{E} \parallel [100]$ and $\boldsymbol{B} \parallel [001]$<br>• $\boldsymbol{E} \parallel [001]$ and strain $\perp [010]$<br>• $\boldsymbol{E} \parallel [001]$ and $\boldsymbol{B} \parallel [100]$<br>• $\boldsymbol{E} \perp [010]$
$Pn'c'2$ (30.115) $\mathbb{Z}_1$	• $\boldsymbol{B} \parallel [100]$<br>• $\boldsymbol{E} \parallel [100]$
Pm' (6.20) $\mathbb{Z}_1$	• $\boldsymbol{B} \parallel [010]$ and strain $\perp [100]$<br>• $\boldsymbol{B} \parallel [001]$ and strain $\perp [100]$<br>• $\boldsymbol{B} \perp [100]$<br>• $\boldsymbol{E} \parallel [010]$ and strain $\perp [100]$<br>• $\boldsymbol{E} \parallel [010]$ and $\boldsymbol{B} \parallel [001]$<br>• $\boldsymbol{E} \parallel [001]$ and strain $\perp [100]$<br>• $\boldsymbol{E} \parallel [001]$ and $\boldsymbol{B} \parallel [010]$<br>• $\boldsymbol{E} \perp [100]$
$P\bar{1}'$ (2.6) $\mathbb{Z}_1$	• strain in generic direction
$P2/m'$ (10.45) $\mathbb{Z}_2$	• strain $\perp [100]$
$P2$ (3.1) $\mathbb{Z}_2$	• $\boldsymbol{B} \parallel [010]$ and strain $\perp [010]$<br>• $\boldsymbol{E} \parallel [010]$ and strain $\perp [010]$
$Pm'a'2$ (28.91) $\mathbb{Z}_2$	• $\boldsymbol{B} \parallel [010]$<br>• $\boldsymbol{E} \parallel [010]$
$P2/c'$ (13.68) $\mathbb{Z}_2$	• strain $\perp [010]$

Continued on next page

…continued

Subgroup & SI group	Perturbation
$P2_1$ (4.7) $\mathbb{Z}_1$	• $\boldsymbol{B}$ ∥ [001] and strain ⊥ [001]<br>• $\boldsymbol{E}$ ∥ [001] and strain ⊥ [001]
$Pm'n'2_1$ (31.127) $\mathbb{Z}_1$	• $\boldsymbol{B}$ ∥ [001]<br>• $\boldsymbol{E}$ ∥ [001]
$P2_1/c'$ (14.78) $\mathbb{Z}_1$	• strain ⊥ [001]

Table. S 440: Subgroups of P_amna (53.330) that can be realized by applying $\boldsymbol{E}/\boldsymbol{B}$ fields and/or uniaxial strains.

Subgroup & SI group	Perturbation
Pm' (6.20) $\mathbb{Z}_1$	• $\boldsymbol{E}$ ∥ [100] and $\boldsymbol{B}$ ∥ [100] and strain ⊥ [001]<br>• $\boldsymbol{E}$ ∥ [100] and $\boldsymbol{B}$ ∥ [010] and strain ⊥ [001]<br>• $\boldsymbol{E}$ ∥ [100] and $\boldsymbol{B}$ ⊥ [001]<br>• $\boldsymbol{E}$ ∥ [010] and $\boldsymbol{B}$ ∥ [100] and strain ⊥ [001]<br>• $\boldsymbol{E}$ ∥ [010] and $\boldsymbol{B}$ ∥ [010] and strain ⊥ [001]<br>• $\boldsymbol{E}$ ∥ [010] and $\boldsymbol{B}$ ⊥ [001]<br>• $\boldsymbol{E}$ ⊥ [001] and $\boldsymbol{B}$ ∥ [100]<br>• $\boldsymbol{E}$ ⊥ [001] and $\boldsymbol{B}$ ∥ [010]<br>• $\boldsymbol{E}$ ⊥ [001] and $\boldsymbol{B}$ ⊥ [001]
Pc' (7.26) $\mathbb{Z}_1$	• $\boldsymbol{E}$ ∥ [100] and $\boldsymbol{B}$ ∥ [100] and strain ⊥ [010]<br>• $\boldsymbol{E}$ ∥ [100] and $\boldsymbol{B}$ ∥ [001] and strain ⊥ [010]<br>• $\boldsymbol{E}$ ∥ [100] and $\boldsymbol{B}$ ⊥ [010]<br>• $\boldsymbol{E}$ ∥ [001] and $\boldsymbol{B}$ ∥ [100] and strain ⊥ [010]<br>• $\boldsymbol{E}$ ∥ [001] and $\boldsymbol{B}$ ∥ [001] and strain ⊥ [010]<br>• $\boldsymbol{E}$ ∥ [001] and $\boldsymbol{B}$ ⊥ [010]<br>• $\boldsymbol{E}$ ⊥ [010] and $\boldsymbol{B}$ ∥ [100]<br>• $\boldsymbol{E}$ ⊥ [010] and $\boldsymbol{B}$ ∥ [001]<br>• $\boldsymbol{E}$ ⊥ [010] and $\boldsymbol{B}$ ⊥ [010]
$P2$ (3.1) $\mathbb{Z}_2$	• $\boldsymbol{E}$ ∥ [100] and $\boldsymbol{B}$ ∥ [100] and strain ⊥ [100]
$Pm'a'2$ (28.91) $\mathbb{Z}_2$	• $\boldsymbol{E}$ ∥ [100] and $\boldsymbol{B}$ ∥ [100]

Continued on next page

...continued

Subgroup & SI group	Perturbation
P_S1 (1.3) $\mathbb{Z}_1$	• $\boldsymbol{E}$ ∥ [100] and strain in generic direction<br>• $\boldsymbol{E}$ ∥ [010] and strain in generic direction<br>• $\boldsymbol{E}$ ∥ [001] and strain in generic direction<br>• $\boldsymbol{E} \perp$ [100] and strain $\perp$ [010]<br>• $\boldsymbol{E} \perp$ [100] and strain $\perp$ [001]<br>• $\boldsymbol{E} \perp$ [100] and strain in generic direction<br>• $\boldsymbol{E} \perp$ [010] and strain $\perp$ [100]<br>• $\boldsymbol{E} \perp$ [010] and strain $\perp$ [001]<br>• $\boldsymbol{E} \perp$ [010] and strain in generic direction<br>• $\boldsymbol{E} \perp$ [001] and strain $\perp$ [100]<br>• $\boldsymbol{E} \perp$ [001] and strain $\perp$ [010]<br>• $\boldsymbol{E} \perp$ [001] and strain in generic direction<br>• $\boldsymbol{E}$ in generic direction
Pc (7.24) $\mathbb{Z}_1$	• $\boldsymbol{E}$ ∥ [100] and $\boldsymbol{B}$ ∥ [001] and strain $\perp$ [001]<br>• $\boldsymbol{E}$ ∥ [010] and $\boldsymbol{B}$ ∥ [001] and strain $\perp$ [001]<br>• $\boldsymbol{E} \perp$ [001] and $\boldsymbol{B}$ ∥ [001]
P_cc (7.28) $\mathbb{Z}_1$	• $\boldsymbol{E}$ ∥ [100] and strain $\perp$ [001]<br>• $\boldsymbol{E}$ ∥ [010] and strain $\perp$ [001]<br>• $\boldsymbol{E} \perp$ [001]
Pc (7.24) $\mathbb{Z}_1$	• $\boldsymbol{E}$ ∥ [100] and $\boldsymbol{B}$ ∥ [010] and strain $\perp$ [010]<br>• $\boldsymbol{E}$ ∥ [001] and $\boldsymbol{B}$ ∥ [010] and strain $\perp$ [010]<br>• $\boldsymbol{E} \perp$ [010] and $\boldsymbol{B}$ ∥ [010]
P_ac (7.27) $\mathbb{Z}_1$	• $\boldsymbol{E}$ ∥ [100] and strain $\perp$ [010]<br>• $\boldsymbol{E}$ ∥ [001] and strain $\perp$ [010]<br>• $\boldsymbol{E} \perp$ [010]
$P2_1'$ (4.9) $\mathbb{Z}_1$	• $\boldsymbol{E}$ ∥ [100] and $\boldsymbol{B}$ ∥ [010] and strain $\perp$ [100]<br>• $\boldsymbol{E}$ ∥ [100] and $\boldsymbol{B}$ ∥ [001] and strain $\perp$ [100]<br>• $\boldsymbol{E}$ ∥ [100] and $\boldsymbol{B} \perp$ [100]
P_b2 (3.5) $\mathbb{Z}_2$	• $\boldsymbol{E}$ ∥ [100] and strain $\perp$ [100]
$Pca'2_1'$ (29.102) $\mathbb{Z}_1$	• $\boldsymbol{E}$ ∥ [100] and $\boldsymbol{B}$ ∥ [001]
$Pm'n2_1'$ (31.125) $\mathbb{Z}_1$	• $\boldsymbol{E}$ ∥ [100] and $\boldsymbol{B}$ ∥ [010]
P_cnc2 (30.118) $\mathbb{Z}_2$	• $\boldsymbol{E}$ ∥ [100]
Pm (6.18) $\mathbb{Z}_1$	• $\boldsymbol{E}$ ∥ [010] and $\boldsymbol{B}$ ∥ [100] and strain $\perp$ [100]<br>• $\boldsymbol{E}$ ∥ [001] and $\boldsymbol{B}$ ∥ [100] and strain $\perp$ [100]<br>• $\boldsymbol{E} \perp$ [100] and $\boldsymbol{B}$ ∥ [100]

Continued on next page

...continued

Subgroup & SI group	Perturbation
$P2'$ (3.3) $\mathbb{Z}_1$	• $\boldsymbol{E}$ ∥ [010] and $\boldsymbol{B}$ ∥ [100] and strain ⊥ [010]<br>• $\boldsymbol{E}$ ∥ [010] and $\boldsymbol{B}$ ∥ [001] and strain ⊥ [010]<br>• $\boldsymbol{E}$ ∥ [010] and $\boldsymbol{B}$ ⊥ [010]
$Pm'm2'$ (25.59) $\mathbb{Z}_1$	• $\boldsymbol{E}$ ∥ [010] and $\boldsymbol{B}$ ∥ [100]
$P2_1'$ (4.9) $\mathbb{Z}_1$	• $\boldsymbol{E}$ ∥ [001] and $\boldsymbol{B}$ ∥ [100] and strain ⊥ [001]<br>• $\boldsymbol{E}$ ∥ [001] and $\boldsymbol{B}$ ∥ [010] and strain ⊥ [001]<br>• $\boldsymbol{E}$ ∥ [001] and $\boldsymbol{B}$ ⊥ [001]
$Pmc'2_1'$ (26.69) $\mathbb{Z}_1$	• $\boldsymbol{E}$ ∥ [001] and $\boldsymbol{B}$ ∥ [100]
$P\bar{1}$ (2.4) $\mathbb{Z}_2 \times \mathbb{Z}_2 \times \mathbb{Z}_2 \times \mathbb{Z}_4$	• $\boldsymbol{B}$ ∥ [100] and strain in generic direction<br>• $\boldsymbol{B}$ ∥ [010] and strain in generic direction<br>• $\boldsymbol{B}$ ∥ [001] and strain in generic direction<br>• $\boldsymbol{B}$ ⊥ [100] and strain ⊥ [010]<br>• $\boldsymbol{B}$ ⊥ [100] and strain ⊥ [001]<br>• $\boldsymbol{B}$ ⊥ [100] and strain in generic direction<br>• $\boldsymbol{B}$ ⊥ [010] and strain ⊥ [100]<br>• $\boldsymbol{B}$ ⊥ [010] and strain ⊥ [001]<br>• $\boldsymbol{B}$ ⊥ [010] and strain in generic direction<br>• $\boldsymbol{B}$ ⊥ [001] and strain ⊥ [100]<br>• $\boldsymbol{B}$ ⊥ [001] and strain ⊥ [010]<br>• $\boldsymbol{B}$ ⊥ [001] and strain in generic direction<br>• $\boldsymbol{B}$ in generic direction
$P2/m$ (10.42) $\mathbb{Z}_2 \times \mathbb{Z}_2 \times \mathbb{Z}_2$	• $\boldsymbol{B}$ ∥ [100] and strain ⊥ [100]
$P2'/c'$ (13.69) $\mathbb{Z}_2 \times \mathbb{Z}_2 \times \mathbb{Z}_4$	• $\boldsymbol{B}$ ∥ [100] and strain ⊥ [010]<br>• $\boldsymbol{B}$ ∥ [001] and strain ⊥ [010]<br>• $\boldsymbol{B}$ ⊥ [010]
$P2_1'/m'$ (11.54) $\mathbb{Z}_2 \times \mathbb{Z}_2 \times \mathbb{Z}_4$	• $\boldsymbol{B}$ ∥ [100] and strain ⊥ [001]<br>• $\boldsymbol{B}$ ∥ [010] and strain ⊥ [001]<br>• $\boldsymbol{B}$ ⊥ [001]
$Pm'ma'$ (51.296) $\mathbb{Z}_2 \times \mathbb{Z}_2 \times \mathbb{Z}_2$	• $\boldsymbol{B}$ ∥ [100]

Continued on next page

…continued

Subgroup & SI group	Perturbation
Pm' (6.20) $\mathbb{Z}_1$	• $\boldsymbol{E}$ ∥ [010] and $\boldsymbol{B}$ ∥ [010] and strain ⊥ [100]<br>• $\boldsymbol{E}$ ∥ [010] and $\boldsymbol{B}$ ∥ [001] and strain ⊥ [100]<br>• $\boldsymbol{E}$ ∥ [010] and $\boldsymbol{B}$ ⊥ [100]<br>• $\boldsymbol{E}$ ∥ [001] and $\boldsymbol{B}$ ∥ [010] and strain ⊥ [100]<br>• $\boldsymbol{E}$ ∥ [001] and $\boldsymbol{B}$ ∥ [001] and strain ⊥ [100]<br>• $\boldsymbol{E}$ ∥ [001] and $\boldsymbol{B}$ ⊥ [100]<br>• $\boldsymbol{E}$ ⊥ [100] and $\boldsymbol{B}$ ∥ [010]<br>• $\boldsymbol{E}$ ⊥ [100] and $\boldsymbol{B}$ ∥ [001]<br>• $\boldsymbol{E}$ ⊥ [100] and $\boldsymbol{B}$ ⊥ [100]
P_bm (6.22) $\mathbb{Z}_1$	• $\boldsymbol{E}$ ∥ [010] and strain ⊥ [100]<br>• $\boldsymbol{E}$ ∥ [001] and strain ⊥ [100]<br>• $\boldsymbol{E}$ ⊥ [100]
$Pm'a2'$ (28.89) $\mathbb{Z}_1$	• $\boldsymbol{E}$ ∥ [010] and $\boldsymbol{B}$ ∥ [001]
$Pm'n2_1'$ (31.125) $\mathbb{Z}_1$	• $\boldsymbol{E}$ ∥ [001] and $\boldsymbol{B}$ ∥ [010]
$P2_1'/m'$ (11.54) $\mathbb{Z}_2 \times \mathbb{Z}_2 \times \mathbb{Z}_4$	• $\boldsymbol{B}$ ∥ [010] and strain ⊥ [100]<br>• $\boldsymbol{B}$ ∥ [001] and strain ⊥ [100]<br>• $\boldsymbol{B}$ ⊥ [100]
$P2$ (3.1) $\mathbb{Z}_2$	• $\boldsymbol{E}$ ∥ [010] and $\boldsymbol{B}$ ∥ [010] and strain ⊥ [010]
P_a2 (3.4) $\mathbb{Z}_2$	• $\boldsymbol{E}$ ∥ [010] and strain ⊥ [010]
$P2/c$ (13.65) $\mathbb{Z}_2 \times \mathbb{Z}_2$	• $\boldsymbol{B}$ ∥ [010] and strain ⊥ [010]
$Pm'm'2$ (25.60) $\mathbb{Z}_2$	• $\boldsymbol{E}$ ∥ [010] and $\boldsymbol{B}$ ∥ [010]
P_ama2 (28.92) $\mathbb{Z}_1$	• $\boldsymbol{E}$ ∥ [010]
$Pm'm'n$ (59.409) $\mathbb{Z}_2 \times \mathbb{Z}_2$	• $\boldsymbol{B}$ ∥ [010]
$P2_1$ (4.7) $\mathbb{Z}_1$	• $\boldsymbol{E}$ ∥ [001] and $\boldsymbol{B}$ ∥ [001] and strain ⊥ [001]
P_a2_1 (4.10) $\mathbb{Z}_1$	• $\boldsymbol{E}$ ∥ [001] and strain ⊥ [001]
$P2_1/c$ (14.75) $\mathbb{Z}_2$	• $\boldsymbol{B}$ ∥ [001] and strain ⊥ [001]
$Pm'c'2_1$ (26.70) $\mathbb{Z}_1$	• $\boldsymbol{E}$ ∥ [001] and $\boldsymbol{B}$ ∥ [001]
P_amn2_1 (31.128) $\mathbb{Z}_1$	• $\boldsymbol{E}$ ∥ [001]
$Pb'cm'$ (57.384) $\mathbb{Z}_2$	• $\boldsymbol{B}$ ∥ [001]
$P_S\bar{1}$ (2.7) $\mathbb{Z}_2 \times \mathbb{Z}_2 \times \mathbb{Z}_4$	• strain in generic direction
P_b2/m (10.48) $\mathbb{Z}_2 \times \mathbb{Z}_2$	• strain ⊥ [100]
P_a2/c (13.70) $\mathbb{Z}_2 \times \mathbb{Z}_2$	• strain ⊥ [010]
P_c2_1/c (14.82) $\mathbb{Z}_2$	• strain ⊥ [001]

Table. S 441: Subgroups of P_bmna (53.331) that can be realized by applying $\boldsymbol{E}/\boldsymbol{B}$ fields and/or uniaxial strains.

Subgroup & SI group	Perturbation
P_S1 (1.3) $\mathbb{Z}_1$	• $\boldsymbol{E} \parallel [100]$ and strain in generic direction<br>• $\boldsymbol{E} \parallel [010]$ and strain in generic direction<br>• $\boldsymbol{E} \parallel [001]$ and strain in generic direction<br>• $\boldsymbol{E} \perp [100]$ and strain $\perp [010]$<br>• $\boldsymbol{E} \perp [100]$ and strain $\perp [001]$<br>• $\boldsymbol{E} \perp [100]$ and strain in generic direction<br>• $\boldsymbol{E} \perp [010]$ and strain $\perp [100]$<br>• $\boldsymbol{E} \perp [010]$ and strain $\perp [001]$<br>• $\boldsymbol{E} \perp [010]$ and strain in generic direction<br>• $\boldsymbol{E} \perp [001]$ and strain $\perp [100]$<br>• $\boldsymbol{E} \perp [001]$ and strain $\perp [010]$<br>• $\boldsymbol{E} \perp [001]$ and strain in generic direction<br>• $\boldsymbol{E}$ in generic direction
$P2$ (3.1) $\mathbb{Z}_2$	• $\boldsymbol{E} \parallel [100]$ and $\boldsymbol{B} \parallel [100]$ and strain $\perp [100]$
$P2'$ (3.3) $\mathbb{Z}_1$	• $\boldsymbol{E} \parallel [100]$ and $\boldsymbol{B} \parallel [010]$ and strain $\perp [100]$<br>• $\boldsymbol{E} \parallel [100]$ and $\boldsymbol{B} \parallel [001]$ and strain $\perp [100]$<br>• $\boldsymbol{E} \parallel [100]$ and $\boldsymbol{B} \perp [100]$
P_a2 (3.4) $\mathbb{Z}_2$	• $\boldsymbol{E} \parallel [100]$ and strain $\perp [100]$
Pc (7.24) $\mathbb{Z}_1$	• $\boldsymbol{E} \parallel [100]$ and $\boldsymbol{B} \parallel [001]$ and strain $\perp [001]$<br>• $\boldsymbol{E} \parallel [010]$ and $\boldsymbol{B} \parallel [001]$ and strain $\perp [001]$<br>• $\boldsymbol{E} \perp [001]$ and $\boldsymbol{B} \parallel [001]$
Pc' (7.26) $\mathbb{Z}_1$	• $\boldsymbol{E} \parallel [100]$ and $\boldsymbol{B} \parallel [100]$ and strain $\perp [001]$<br>• $\boldsymbol{E} \parallel [100]$ and $\boldsymbol{B} \parallel [010]$ and strain $\perp [001]$<br>• $\boldsymbol{E} \parallel [100]$ and $\boldsymbol{B} \perp [001]$<br>• $\boldsymbol{E} \parallel [010]$ and $\boldsymbol{B} \parallel [100]$ and strain $\perp [001]$<br>• $\boldsymbol{E} \parallel [010]$ and $\boldsymbol{B} \parallel [010]$ and strain $\perp [001]$<br>• $\boldsymbol{E} \parallel [010]$ and $\boldsymbol{B} \perp [001]$<br>• $\boldsymbol{E} \perp [001]$ and $\boldsymbol{B} \parallel [100]$<br>• $\boldsymbol{E} \perp [001]$ and $\boldsymbol{B} \parallel [010]$<br>• $\boldsymbol{E} \perp [001]$ and $\boldsymbol{B} \perp [001]$
P_ac (7.27) $\mathbb{Z}_1$	• $\boldsymbol{E} \parallel [100]$ and strain $\perp [001]$<br>• $\boldsymbol{E} \parallel [010]$ and strain $\perp [001]$<br>• $\boldsymbol{E} \perp [001]$

Continued on next page

...continued

Subgroup & SI group	Perturbation
Pc (7.24) $\mathbb{Z}_1$	• $\boldsymbol{E}$ ∥ [100] and $\boldsymbol{B}$ ∥ [010] and strain ⊥ [010]<br>• $\boldsymbol{E}$ ∥ [001] and $\boldsymbol{B}$ ∥ [010] and strain ⊥ [010]<br>• $\boldsymbol{E}$ ⊥ [010] and $\boldsymbol{B}$ ∥ [010]
$Pn'n2'$ (34.158) $\mathbb{Z}_1$	• $\boldsymbol{E}$ ∥ [100] and $\boldsymbol{B}$ ∥ [010]
Pc' (7.26) $\mathbb{Z}_1$	• $\boldsymbol{E}$ ∥ [100] and $\boldsymbol{B}$ ∥ [100] and strain ⊥ [010]<br>• $\boldsymbol{E}$ ∥ [100] and $\boldsymbol{B}$ ∥ [001] and strain ⊥ [010]<br>• $\boldsymbol{E}$ ∥ [100] and $\boldsymbol{B}$ ⊥ [010]<br>• $\boldsymbol{E}$ ∥ [001] and $\boldsymbol{B}$ ∥ [100] and strain ⊥ [010]<br>• $\boldsymbol{E}$ ∥ [001] and $\boldsymbol{B}$ ∥ [001] and strain ⊥ [010]<br>• $\boldsymbol{E}$ ∥ [001] and $\boldsymbol{B}$ ⊥ [010]<br>• $\boldsymbol{E}$ ⊥ [010] and $\boldsymbol{B}$ ∥ [100]<br>• $\boldsymbol{E}$ ⊥ [010] and $\boldsymbol{B}$ ∥ [001]<br>• $\boldsymbol{E}$ ⊥ [010] and $\boldsymbol{B}$ ⊥ [010]
$Pn'c2'$ (30.113) $\mathbb{Z}_1$	• $\boldsymbol{E}$ ∥ [100] and $\boldsymbol{B}$ ∥ [001]
$Pn'n'2$ (34.159) $\mathbb{Z}_1$	• $\boldsymbol{E}$ ∥ [100] and $\boldsymbol{B}$ ∥ [100]
P_bc (7.29) $\mathbb{Z}_1$	• $\boldsymbol{E}$ ∥ [100] and strain ⊥ [010]<br>• $\boldsymbol{E}$ ∥ [001] and strain ⊥ [010]<br>• $\boldsymbol{E}$ ⊥ [010]
P_anc2 (30.116) $\mathbb{Z}_1$	• $\boldsymbol{E}$ ∥ [100]
Pm (6.18) $\mathbb{Z}_1$	• $\boldsymbol{E}$ ∥ [010] and $\boldsymbol{B}$ ∥ [100] and strain ⊥ [100]<br>• $\boldsymbol{E}$ ∥ [001] and $\boldsymbol{B}$ ∥ [100] and strain ⊥ [100]<br>• $\boldsymbol{E}$ ⊥ [100] and $\boldsymbol{B}$ ∥ [100]
Pc' (7.26) $\mathbb{Z}_1$	• $\boldsymbol{E}$ ∥ [010] and $\boldsymbol{B}$ ∥ [010] and strain ⊥ [100]<br>• $\boldsymbol{E}$ ∥ [010] and $\boldsymbol{B}$ ∥ [001] and strain ⊥ [100]<br>• $\boldsymbol{E}$ ∥ [010] and $\boldsymbol{B}$ ⊥ [100]<br>• $\boldsymbol{E}$ ∥ [001] and $\boldsymbol{B}$ ∥ [010] and strain ⊥ [100]<br>• $\boldsymbol{E}$ ∥ [001] and $\boldsymbol{B}$ ∥ [001] and strain ⊥ [100]<br>• $\boldsymbol{E}$ ∥ [001] and $\boldsymbol{B}$ ⊥ [100]<br>• $\boldsymbol{E}$ ⊥ [100] and $\boldsymbol{B}$ ∥ [010]<br>• $\boldsymbol{E}$ ⊥ [100] and $\boldsymbol{B}$ ∥ [001]<br>• $\boldsymbol{E}$ ⊥ [100] and $\boldsymbol{B}$ ⊥ [100]
P_am (6.21) $\mathbb{Z}_1$	• $\boldsymbol{E}$ ∥ [010] and strain ⊥ [100]<br>• $\boldsymbol{E}$ ∥ [001] and strain ⊥ [100]<br>• $\boldsymbol{E}$ ⊥ [100]

Continued on next page

...continued

Subgroup & SI group	Perturbation
$P\bar{1}$ (2.4) $\mathbb{Z}_2 \times \mathbb{Z}_2 \times \mathbb{Z}_2 \times \mathbb{Z}_4$	• $\boldsymbol{B} \parallel [100]$ and strain in generic direction<br>• $\boldsymbol{B} \parallel [010]$ and strain in generic direction<br>• $\boldsymbol{B} \parallel [001]$ and strain in generic direction<br>• $\boldsymbol{B} \perp [100]$ and strain $\perp [010]$<br>• $\boldsymbol{B} \perp [100]$ and strain $\perp [001]$<br>• $\boldsymbol{B} \perp [100]$ and strain in generic direction<br>• $\boldsymbol{B} \perp [010]$ and strain $\perp [100]$<br>• $\boldsymbol{B} \perp [010]$ and strain $\perp [001]$<br>• $\boldsymbol{B} \perp [010]$ and strain in generic direction<br>• $\boldsymbol{B} \perp [001]$ and strain $\perp [100]$<br>• $\boldsymbol{B} \perp [001]$ and strain $\perp [010]$<br>• $\boldsymbol{B} \perp [001]$ and strain in generic direction<br>• $\boldsymbol{B}$ in generic direction
$P2/m$ (10.42) $\mathbb{Z}_2 \times \mathbb{Z}_2 \times \mathbb{Z}_2$	• $\boldsymbol{B} \parallel [100]$ and strain $\perp [100]$
$P2'/c'$ (13.69) $\mathbb{Z}_2 \times \mathbb{Z}_2 \times \mathbb{Z}_4$	• $\boldsymbol{B} \parallel [010]$ and strain $\perp [100]$<br>• $\boldsymbol{B} \parallel [001]$ and strain $\perp [100]$<br>• $\boldsymbol{B} \perp [100]$
$P_S\bar{1}$ (2.7) $\mathbb{Z}_2 \times \mathbb{Z}_2 \times \mathbb{Z}_4$	• strain in generic direction
P_a2/m (10.47) $\mathbb{Z}_2 \times \mathbb{Z}_2 \times \mathbb{Z}_2$	• strain $\perp [100]$
$P2$ (3.1) $\mathbb{Z}_2$	• $\boldsymbol{E} \parallel [010]$ and $\boldsymbol{B} \parallel [010]$ and strain $\perp [010]$
$Pn'c'2$ (30.115) $\mathbb{Z}_1$	• $\boldsymbol{E} \parallel [010]$ and $\boldsymbol{B} \parallel [010]$
$P2/c$ (13.65) $\mathbb{Z}_2 \times \mathbb{Z}_2$	• $\boldsymbol{B} \parallel [010]$ and strain $\perp [010]$
$P2_1'$ (4.9) $\mathbb{Z}_1$	• $\boldsymbol{E} \parallel [010]$ and $\boldsymbol{B} \parallel [100]$ and strain $\perp [010]$<br>• $\boldsymbol{E} \parallel [010]$ and $\boldsymbol{B} \parallel [001]$ and strain $\perp [010]$<br>• $\boldsymbol{E} \parallel [010]$ and $\boldsymbol{B} \perp [010]$
$Pmn'2_1'$ (31.126) $\mathbb{Z}_1$	• $\boldsymbol{E} \parallel [010]$ and $\boldsymbol{B} \parallel [100]$
$Pc'a2_1'$ (29.101) $\mathbb{Z}_1$	• $\boldsymbol{E} \parallel [010]$ and $\boldsymbol{B} \parallel [001]$
$P2_1'/c'$ (14.79) $\mathbb{Z}_2 \times \mathbb{Z}_4$	• $\boldsymbol{B} \parallel [100]$ and strain $\perp [010]$<br>• $\boldsymbol{B} \parallel [001]$ and strain $\perp [010]$<br>• $\boldsymbol{B} \perp [010]$
P_b2 (3.5) $\mathbb{Z}_2$	• $\boldsymbol{E} \parallel [010]$ and strain $\perp [010]$
P_cma2 (28.94) $\mathbb{Z}_1$	• $\boldsymbol{E} \parallel [010]$
P_b2/c (13.71) $\mathbb{Z}_2 \times \mathbb{Z}_2$	• strain $\perp [010]$
$P2_1$ (4.7) $\mathbb{Z}_1$	• $\boldsymbol{E} \parallel [001]$ and $\boldsymbol{B} \parallel [001]$ and strain $\perp [001]$

Continued on next page

…continued

Subgroup & SI group	Perturbation
$Pn'a'2_1$ (33.148) $\mathbb{Z}_1$	• $\boldsymbol{E}$ ∥ [001] and $\boldsymbol{B}$ ∥ [001]
$P2_1/c$ (14.75) $\mathbb{Z}_2$	• $\boldsymbol{B}$ ∥ [001] and strain ⊥ [001]
$Pbc'n'$ (60.423) $\mathbb{Z}_2$	• $\boldsymbol{B}$ ∥ [001]
$P2_1'$ (4.9) $\mathbb{Z}_1$	• $\boldsymbol{E}$ ∥ [001] and $\boldsymbol{B}$ ∥ [100] and strain ⊥ [001]<br>• $\boldsymbol{E}$ ∥ [001] and $\boldsymbol{B}$ ∥ [010] and strain ⊥ [001]<br>• $\boldsymbol{E}$ ∥ [001] and $\boldsymbol{B}$ ⊥ [001]
$Pmn'2_1'$ (31.126) $\mathbb{Z}_1$	• $\boldsymbol{E}$ ∥ [001] and $\boldsymbol{B}$ ∥ [100]
$Pna'2_1'$ (33.147) $\mathbb{Z}_1$	• $\boldsymbol{E}$ ∥ [001] and $\boldsymbol{B}$ ∥ [010]
$P2_1'/c'$ (14.79) $\mathbb{Z}_2 \times \mathbb{Z}_4$	• $\boldsymbol{B}$ ∥ [100] and strain ⊥ [001]<br>• $\boldsymbol{B}$ ∥ [010] and strain ⊥ [001]<br>• $\boldsymbol{B}$ ⊥ [001]
$Pnn'a'$ (52.311) $\mathbb{Z}_2$	• $\boldsymbol{B}$ ∥ [010]
$Pn'n'm$ (58.397) $\mathbb{Z}_2 \times \mathbb{Z}_2$	• $\boldsymbol{B}$ ∥ [100]
P_a2_1 (4.10) $\mathbb{Z}_1$	• $\boldsymbol{E}$ ∥ [001] and strain ⊥ [001]
P_bmn2_1 (31.129) $\mathbb{Z}_1$	• $\boldsymbol{E}$ ∥ [001]
P_a2_1/c (14.80) $\mathbb{Z}_2$	• strain ⊥ [001]

Table. S 442: Subgroups of P_cmna (53.332) that can be realized by applying $\boldsymbol{E}/\boldsymbol{B}$ fields and/or uniaxial strains.

Subgroup & SI group	Perturbation

Continued on next page

…continued

Subgroup & SI group	Perturbation
P_S1 (1.3) $\mathbb{Z}_1$	• $\boldsymbol{E} \parallel [100]$ and strain in generic direction<br>• $\boldsymbol{E} \parallel [010]$ and strain in generic direction<br>• $\boldsymbol{E} \parallel [001]$ and strain in generic direction<br>• $\boldsymbol{E} \perp [100]$ and strain $\perp [010]$<br>• $\boldsymbol{E} \perp [100]$ and strain $\perp [001]$<br>• $\boldsymbol{E} \perp [100]$ and strain in generic direction<br>• $\boldsymbol{E} \perp [010]$ and strain $\perp [100]$<br>• $\boldsymbol{E} \perp [010]$ and strain $\perp [001]$<br>• $\boldsymbol{E} \perp [010]$ and strain in generic direction<br>• $\boldsymbol{E} \perp [001]$ and strain $\perp [100]$<br>• $\boldsymbol{E} \perp [001]$ and strain $\perp [010]$<br>• $\boldsymbol{E} \perp [001]$ and strain in generic direction<br>• $\boldsymbol{E}$ in generic direction
$P2$ (3.1) $\mathbb{Z}_2$	• $\boldsymbol{E} \parallel [100]$ and $\boldsymbol{B} \parallel [100]$ and strain $\perp [100]$
$P2'$ (3.3) $\mathbb{Z}_1$	• $\boldsymbol{E} \parallel [100]$ and $\boldsymbol{B} \parallel [010]$ and strain $\perp [100]$<br>• $\boldsymbol{E} \parallel [100]$ and $\boldsymbol{B} \parallel [001]$ and strain $\perp [100]$<br>• $\boldsymbol{E} \parallel [100]$ and $\boldsymbol{B} \perp [100]$
P_a2 (3.4) $\mathbb{Z}_2$	• $\boldsymbol{E} \parallel [100]$ and strain $\perp [100]$
Pc' (7.26) $\mathbb{Z}_1$	• $\boldsymbol{E} \parallel [100]$ and $\boldsymbol{B} \parallel [100]$ and strain $\perp [001]$<br>• $\boldsymbol{E} \parallel [100]$ and $\boldsymbol{B} \parallel [010]$ and strain $\perp [001]$<br>• $\boldsymbol{E} \parallel [100]$ and $\boldsymbol{B} \perp [001]$<br>• $\boldsymbol{E} \parallel [010]$ and $\boldsymbol{B} \parallel [100]$ and strain $\perp [001]$<br>• $\boldsymbol{E} \parallel [010]$ and $\boldsymbol{B} \parallel [010]$ and strain $\perp [001]$<br>• $\boldsymbol{E} \parallel [010]$ and $\boldsymbol{B} \perp [001]$<br>• $\boldsymbol{E} \perp [001]$ and $\boldsymbol{B} \parallel [100]$<br>• $\boldsymbol{E} \perp [001]$ and $\boldsymbol{B} \parallel [010]$<br>• $\boldsymbol{E} \perp [001]$ and $\boldsymbol{B} \perp [001]$
Pc (7.24) $\mathbb{Z}_1$	• $\boldsymbol{E} \parallel [100]$ and $\boldsymbol{B} \parallel [001]$ and strain $\perp [001]$<br>• $\boldsymbol{E} \parallel [010]$ and $\boldsymbol{B} \parallel [001]$ and strain $\perp [001]$<br>• $\boldsymbol{E} \perp [001]$ and $\boldsymbol{B} \parallel [001]$
P_bc (7.29) $\mathbb{Z}_1$	• $\boldsymbol{E} \parallel [100]$ and strain $\perp [001]$<br>• $\boldsymbol{E} \parallel [010]$ and strain $\perp [001]$<br>• $\boldsymbol{E} \perp [001]$

Continued on next page

...continued

Subgroup & SI group	Perturbation
Pc' (7.26) $\mathbb{Z}_1$	• $\boldsymbol{E}$ ∥ [100] and $\boldsymbol{B}$ ∥ [100] and strain ⊥ [010]<br>• $\boldsymbol{E}$ ∥ [100] and $\boldsymbol{B}$ ∥ [001] and strain ⊥ [010]<br>• $\boldsymbol{E}$ ∥ [100] and $\boldsymbol{B}$ ⊥ [010]<br>• $\boldsymbol{E}$ ∥ [001] and $\boldsymbol{B}$ ∥ [100] and strain ⊥ [010]<br>• $\boldsymbol{E}$ ∥ [001] and $\boldsymbol{B}$ ∥ [001] and strain ⊥ [010]<br>• $\boldsymbol{E}$ ∥ [001] and $\boldsymbol{B}$ ⊥ [010]<br>• $\boldsymbol{E}$ ⊥ [010] and $\boldsymbol{B}$ ∥ [100]<br>• $\boldsymbol{E}$ ⊥ [010] and $\boldsymbol{B}$ ∥ [001]<br>• $\boldsymbol{E}$ ⊥ [010] and $\boldsymbol{B}$ ⊥ [010]
$Pc'c'2$ (27.81) $\mathbb{Z}_2$	• $\boldsymbol{E}$ ∥ [100] and $\boldsymbol{B}$ ∥ [100]
$Pc'c2'$ (27.80) $\mathbb{Z}_1$	• $\boldsymbol{E}$ ∥ [100] and $\boldsymbol{B}$ ∥ [001]
Pc (7.24) $\mathbb{Z}_1$	• $\boldsymbol{E}$ ∥ [100] and $\boldsymbol{B}$ ∥ [010] and strain ⊥ [010]<br>• $\boldsymbol{E}$ ∥ [001] and $\boldsymbol{B}$ ∥ [010] and strain ⊥ [010]<br>• $\boldsymbol{E}$ ⊥ [010] and $\boldsymbol{B}$ ∥ [010]
$Pnc'2'$ (30.114) $\mathbb{Z}_1$	• $\boldsymbol{E}$ ∥ [100] and $\boldsymbol{B}$ ∥ [010]
P_ac (7.27) $\mathbb{Z}_1$	• $\boldsymbol{E}$ ∥ [100] and strain ⊥ [010]<br>• $\boldsymbol{E}$ ∥ [001] and strain ⊥ [010]<br>• $\boldsymbol{E}$ ⊥ [010]
P_bnc2 (30.117) $\mathbb{Z}_2$	• $\boldsymbol{E}$ ∥ [100]
Pm (6.18) $\mathbb{Z}_1$	• $\boldsymbol{E}$ ∥ [010] and $\boldsymbol{B}$ ∥ [100] and strain ⊥ [100]<br>• $\boldsymbol{E}$ ∥ [001] and $\boldsymbol{B}$ ∥ [100] and strain ⊥ [100]<br>• $\boldsymbol{E}$ ⊥ [100] and $\boldsymbol{B}$ ∥ [100]
Pc' (7.26) $\mathbb{Z}_1$	• $\boldsymbol{E}$ ∥ [010] and $\boldsymbol{B}$ ∥ [010] and strain ⊥ [100]<br>• $\boldsymbol{E}$ ∥ [010] and $\boldsymbol{B}$ ∥ [001] and strain ⊥ [100]<br>• $\boldsymbol{E}$ ∥ [010] and $\boldsymbol{B}$ ⊥ [100]<br>• $\boldsymbol{E}$ ∥ [001] and $\boldsymbol{B}$ ∥ [010] and strain ⊥ [100]<br>• $\boldsymbol{E}$ ∥ [001] and $\boldsymbol{B}$ ∥ [001] and strain ⊥ [100]<br>• $\boldsymbol{E}$ ∥ [001] and $\boldsymbol{B}$ ⊥ [100]<br>• $\boldsymbol{E}$ ⊥ [100] and $\boldsymbol{B}$ ∥ [010]<br>• $\boldsymbol{E}$ ⊥ [100] and $\boldsymbol{B}$ ∥ [001]<br>• $\boldsymbol{E}$ ⊥ [100] and $\boldsymbol{B}$ ⊥ [100]
P_am (6.21) $\mathbb{Z}_1$	• $\boldsymbol{E}$ ∥ [010] and strain ⊥ [100]<br>• $\boldsymbol{E}$ ∥ [001] and strain ⊥ [100]<br>• $\boldsymbol{E}$ ⊥ [100]

Continued on next page

...continued

Subgroup & SI group	Perturbation
$P\bar{1}$ (2.4) $\mathbb{Z}_2 \times \mathbb{Z}_2 \times \mathbb{Z}_2 \times \mathbb{Z}_4$	• $\boldsymbol{B} \parallel [100]$ and strain in generic direction<br>• $\boldsymbol{B} \parallel [010]$ and strain in generic direction<br>• $\boldsymbol{B} \parallel [001]$ and strain in generic direction<br>• $\boldsymbol{B} \perp [100]$ and strain $\perp [010]$<br>• $\boldsymbol{B} \perp [100]$ and strain $\perp [001]$<br>• $\boldsymbol{B} \perp [100]$ and strain in generic direction<br>• $\boldsymbol{B} \perp [010]$ and strain $\perp [100]$<br>• $\boldsymbol{B} \perp [010]$ and strain $\perp [001]$<br>• $\boldsymbol{B} \perp [010]$ and strain in generic direction<br>• $\boldsymbol{B} \perp [001]$ and strain $\perp [100]$<br>• $\boldsymbol{B} \perp [001]$ and strain $\perp [010]$<br>• $\boldsymbol{B} \perp [001]$ and strain in generic direction<br>• $\boldsymbol{B}$ in generic direction
$P2/m$ (10.42) $\mathbb{Z}_2 \times \mathbb{Z}_2 \times \mathbb{Z}_2$	• $\boldsymbol{B} \parallel [100]$ and strain $\perp [100]$
$P2'/c'$ (13.69) $\mathbb{Z}_2 \times \mathbb{Z}_2 \times \mathbb{Z}_4$	• $\boldsymbol{B} \parallel [010]$ and strain $\perp [100]$<br>• $\boldsymbol{B} \parallel [001]$ and strain $\perp [100]$<br>• $\boldsymbol{B} \perp [100]$
$P_S\bar{1}$ (2.7) $\mathbb{Z}_2 \times \mathbb{Z}_2 \times \mathbb{Z}_4$	• strain in generic direction
P_a2/m (10.47) $\mathbb{Z}_2 \times \mathbb{Z}_2 \times \mathbb{Z}_2$	• strain $\perp [100]$
$P2'$ (3.3) $\mathbb{Z}_1$	• $\boldsymbol{E} \parallel [010]$ and $\boldsymbol{B} \parallel [100]$ and strain $\perp [010]$<br>• $\boldsymbol{E} \parallel [010]$ and $\boldsymbol{B} \parallel [001]$ and strain $\perp [010]$<br>• $\boldsymbol{E} \parallel [010]$ and $\boldsymbol{B} \perp [010]$
$Pma'2'$ (28.90) $\mathbb{Z}_1$	• $\boldsymbol{E} \parallel [010]$ and $\boldsymbol{B} \parallel [100]$
$Pb'a2'$ (32.137) $\mathbb{Z}_1$	• $\boldsymbol{E} \parallel [010]$ and $\boldsymbol{B} \parallel [001]$
$P2'/c'$ (13.69) $\mathbb{Z}_2 \times \mathbb{Z}_2 \times \mathbb{Z}_4$	• $\boldsymbol{B} \parallel [100]$ and strain $\perp [010]$<br>• $\boldsymbol{B} \parallel [001]$ and strain $\perp [010]$<br>• $\boldsymbol{B} \perp [010]$
$P2$ (3.1) $\mathbb{Z}_2$	• $\boldsymbol{E} \parallel [010]$ and $\boldsymbol{B} \parallel [010]$ and strain $\perp [010]$
$Pb'a'2$ (32.138) $\mathbb{Z}_2$	• $\boldsymbol{E} \parallel [010]$ and $\boldsymbol{B} \parallel [010]$
$P2/c$ (13.65) $\mathbb{Z}_2 \times \mathbb{Z}_2$	• $\boldsymbol{B} \parallel [010]$ and strain $\perp [010]$
P_a2 (3.4) $\mathbb{Z}_2$	• $\boldsymbol{E} \parallel [010]$ and strain $\perp [010]$
P_bma2 (28.93) $\mathbb{Z}_1$	• $\boldsymbol{E} \parallel [010]$
P_a2/c (13.70) $\mathbb{Z}_2 \times \mathbb{Z}_2$	• strain $\perp [010]$

Continued on next page

...continued

Subgroup & SI group	Perturbation
$P2'$ (3.3) $\mathbb{Z}_1$	• $\boldsymbol{E}$ ∥ [001] and $\boldsymbol{B}$ ∥ [100] and strain ⊥ [001]<br>• $\boldsymbol{E}$ ∥ [001] and $\boldsymbol{B}$ ∥ [010] and strain ⊥ [001]<br>• $\boldsymbol{E}$ ∥ [001] and $\boldsymbol{B}$ ⊥ [001]
$Pma'2'$ (28.90) $\mathbb{Z}_1$	• $\boldsymbol{E}$ ∥ [001] and $\boldsymbol{B}$ ∥ [100]
$Pnc'2'$ (30.114) $\mathbb{Z}_1$	• $\boldsymbol{E}$ ∥ [001] and $\boldsymbol{B}$ ∥ [010]
$P2'/c'$ (13.69) $\mathbb{Z}_2 \times \mathbb{Z}_2 \times \mathbb{Z}_4$	• $\boldsymbol{B}$ ∥ [100] and strain ⊥ [001]<br>• $\boldsymbol{B}$ ∥ [010] and strain ⊥ [001]<br>• $\boldsymbol{B}$ ⊥ [001]
$Pc'c'm$ (49.269) $\mathbb{Z}_2 \times \mathbb{Z}_2$	• $\boldsymbol{B}$ ∥ [100]
$Pb'a'n$ (50.281) $\mathbb{Z}_2 \times \mathbb{Z}_2$	• $\boldsymbol{B}$ ∥ [010]
$P2_1$ (4.7) $\mathbb{Z}_1$	• $\boldsymbol{E}$ ∥ [001] and $\boldsymbol{B}$ ∥ [001] and strain ⊥ [001]
$Pc'a'2_1$ (29.103) $\mathbb{Z}_1$	• $\boldsymbol{E}$ ∥ [001] and $\boldsymbol{B}$ ∥ [001]
$P2_1/c$ (14.75) $\mathbb{Z}_2$	• $\boldsymbol{B}$ ∥ [001] and strain ⊥ [001]
$Pcc'a'$ (54.343) $\mathbb{Z}_2$	• $\boldsymbol{B}$ ∥ [001]
P_b2_1 (4.11) $\mathbb{Z}_1$	• $\boldsymbol{E}$ ∥ [001] and strain ⊥ [001]
P_cmn2_1 (31.130) $\mathbb{Z}_1$	• $\boldsymbol{E}$ ∥ [001]
P_b2_1/c (14.81) $\mathbb{Z}_2$	• strain ⊥ [001]

Table. S 443: Subgroups of P_Amna (53.333) that can be realized by applying $\boldsymbol{E}/\boldsymbol{B}$ fields and/or uniaxial strains.

Subgroup & SI group	Perturbation

Continued on next page

...continued

Subgroup & SI group	Perturbation
P_S1 (1.3) $\mathbb{Z}_1$	• $\boldsymbol{E} \parallel [100]$ and strain in generic direction<br>• $\boldsymbol{E} \parallel [010]$ and strain in generic direction<br>• $\boldsymbol{E} \parallel [001]$ and strain in generic direction<br>• $\boldsymbol{E} \perp [100]$ and strain $\perp [010]$<br>• $\boldsymbol{E} \perp [100]$ and strain $\perp [001]$<br>• $\boldsymbol{E} \perp [100]$ and strain in generic direction<br>• $\boldsymbol{E} \perp [010]$ and strain $\perp [100]$<br>• $\boldsymbol{E} \perp [010]$ and strain $\perp [001]$<br>• $\boldsymbol{E} \perp [010]$ and strain in generic direction<br>• $\boldsymbol{E} \perp [001]$ and strain $\perp [100]$<br>• $\boldsymbol{E} \perp [001]$ and strain $\perp [010]$<br>• $\boldsymbol{E} \perp [001]$ and strain in generic direction<br>• $\boldsymbol{E}$ in generic direction
$P2$ (3.1) $\mathbb{Z}_2$	• $\boldsymbol{E} \parallel [100]$ and $\boldsymbol{B} \parallel [100]$ and strain $\perp [100]$
$P2'$ (3.3) $\mathbb{Z}_1$	• $\boldsymbol{E} \parallel [100]$ and $\boldsymbol{B} \parallel [010]$ and strain $\perp [100]$<br>• $\boldsymbol{E} \parallel [100]$ and $\boldsymbol{B} \parallel [001]$ and strain $\perp [100]$<br>• $\boldsymbol{E} \parallel [100]$ and $\boldsymbol{B} \perp [100]$
P_a2 (3.4) $\mathbb{Z}_2$	• $\boldsymbol{E} \parallel [100]$ and strain $\perp [100]$
Pc (7.24) $\mathbb{Z}_1$	• $\boldsymbol{E} \parallel [100]$ and $\boldsymbol{B} \parallel [001]$ and strain $\perp [001]$<br>• $\boldsymbol{E} \parallel [010]$ and $\boldsymbol{B} \parallel [001]$ and strain $\perp [001]$<br>• $\boldsymbol{E} \perp [001]$ and $\boldsymbol{B} \parallel [001]$
Pc' (7.26) $\mathbb{Z}_1$	• $\boldsymbol{E} \parallel [100]$ and $\boldsymbol{B} \parallel [100]$ and strain $\perp [001]$<br>• $\boldsymbol{E} \parallel [100]$ and $\boldsymbol{B} \parallel [010]$ and strain $\perp [001]$<br>• $\boldsymbol{E} \parallel [100]$ and $\boldsymbol{B} \perp [001]$<br>• $\boldsymbol{E} \parallel [010]$ and $\boldsymbol{B} \parallel [100]$ and strain $\perp [001]$<br>• $\boldsymbol{E} \parallel [010]$ and $\boldsymbol{B} \parallel [010]$ and strain $\perp [001]$<br>• $\boldsymbol{E} \parallel [010]$ and $\boldsymbol{B} \perp [001]$<br>• $\boldsymbol{E} \perp [001]$ and $\boldsymbol{B} \parallel [100]$<br>• $\boldsymbol{E} \perp [001]$ and $\boldsymbol{B} \parallel [010]$<br>• $\boldsymbol{E} \perp [001]$ and $\boldsymbol{B} \perp [001]$
P_Cc (7.30) $\mathbb{Z}_1$	• $\boldsymbol{E} \parallel [100]$ and strain $\perp [001]$<br>• $\boldsymbol{E} \parallel [010]$ and strain $\perp [001]$<br>• $\boldsymbol{E} \perp [001]$
Pc (7.24) $\mathbb{Z}_1$	• $\boldsymbol{E} \parallel [100]$ and $\boldsymbol{B} \parallel [010]$ and strain $\perp [010]$<br>• $\boldsymbol{E} \parallel [001]$ and $\boldsymbol{B} \parallel [010]$ and strain $\perp [010]$<br>• $\boldsymbol{E} \perp [010]$ and $\boldsymbol{B} \parallel [010]$
$Pn'n2'$ (34.158) $\mathbb{Z}_1$	• $\boldsymbol{E} \parallel [100]$ and $\boldsymbol{B} \parallel [010]$

Continued on next page

...continued

Subgroup & SI group	Perturbation
Pc' (7.26) $\mathbb{Z}_1$	• $\boldsymbol{E} \parallel [100]$ and $\boldsymbol{B} \parallel [100]$ and strain $\perp [010]$<br>• $\boldsymbol{E} \parallel [100]$ and $\boldsymbol{B} \parallel [001]$ and strain $\perp [010]$<br>• $\boldsymbol{E} \parallel [100]$ and $\boldsymbol{B} \perp [010]$<br>• $\boldsymbol{E} \parallel [001]$ and $\boldsymbol{B} \parallel [100]$ and strain $\perp [010]$<br>• $\boldsymbol{E} \parallel [001]$ and $\boldsymbol{B} \parallel [001]$ and strain $\perp [010]$<br>• $\boldsymbol{E} \parallel [001]$ and $\boldsymbol{B} \perp [010]$<br>• $\boldsymbol{E} \perp [010]$ and $\boldsymbol{B} \parallel [100]$<br>• $\boldsymbol{E} \perp [010]$ and $\boldsymbol{B} \parallel [001]$<br>• $\boldsymbol{E} \perp [010]$ and $\boldsymbol{B} \perp [010]$
$Pc'c2'$ (27.80) $\mathbb{Z}_1$	• $\boldsymbol{E} \parallel [100]$ and $\boldsymbol{B} \parallel [001]$
$Pn'c'2$ (30.115) $\mathbb{Z}_1$	• $\boldsymbol{E} \parallel [100]$ and $\boldsymbol{B} \parallel [100]$
P_Cc (7.30) $\mathbb{Z}_1$	• $\boldsymbol{E} \parallel [100]$ and strain $\perp [010]$<br>• $\boldsymbol{E} \parallel [001]$ and strain $\perp [010]$<br>• $\boldsymbol{E} \perp [010]$
P_Cnc2 (30.121) $\mathbb{Z}_1$	• $\boldsymbol{E} \parallel [100]$
Pm (6.18) $\mathbb{Z}_1$	• $\boldsymbol{E} \parallel [010]$ and $\boldsymbol{B} \parallel [100]$ and strain $\perp [100]$<br>• $\boldsymbol{E} \parallel [001]$ and $\boldsymbol{B} \parallel [100]$ and strain $\perp [100]$<br>• $\boldsymbol{E} \perp [100]$ and $\boldsymbol{B} \parallel [100]$
Pc' (7.26) $\mathbb{Z}_1$	• $\boldsymbol{E} \parallel [010]$ and $\boldsymbol{B} \parallel [010]$ and strain $\perp [100]$<br>• $\boldsymbol{E} \parallel [010]$ and $\boldsymbol{B} \parallel [001]$ and strain $\perp [100]$<br>• $\boldsymbol{E} \parallel [010]$ and $\boldsymbol{B} \perp [100]$<br>• $\boldsymbol{E} \parallel [001]$ and $\boldsymbol{B} \parallel [010]$ and strain $\perp [100]$<br>• $\boldsymbol{E} \parallel [001]$ and $\boldsymbol{B} \parallel [001]$ and strain $\perp [100]$<br>• $\boldsymbol{E} \parallel [001]$ and $\boldsymbol{B} \perp [100]$<br>• $\boldsymbol{E} \perp [100]$ and $\boldsymbol{B} \parallel [010]$<br>• $\boldsymbol{E} \perp [100]$ and $\boldsymbol{B} \parallel [001]$<br>• $\boldsymbol{E} \perp [100]$ and $\boldsymbol{B} \perp [100]$
P_am (6.21) $\mathbb{Z}_1$	• $\boldsymbol{E} \parallel [010]$ and strain $\perp [100]$<br>• $\boldsymbol{E} \parallel [001]$ and strain $\perp [100]$<br>• $\boldsymbol{E} \perp [100]$

Continued on next page

...continued

Subgroup & SI group	Perturbation
$P\bar{1}$ (2.4) $\mathbb{Z}_2 \times \mathbb{Z}_2 \times \mathbb{Z}_2 \times \mathbb{Z}_4$	• $\boldsymbol{B}$ ∥ [100] and strain in generic direction<br>• $\boldsymbol{B}$ ∥ [010] and strain in generic direction<br>• $\boldsymbol{B}$ ∥ [001] and strain in generic direction<br>• $\boldsymbol{B} \perp$ [100] and strain $\perp$ [010]<br>• $\boldsymbol{B} \perp$ [100] and strain $\perp$ [001]<br>• $\boldsymbol{B} \perp$ [100] and strain in generic direction<br>• $\boldsymbol{B} \perp$ [010] and strain $\perp$ [100]<br>• $\boldsymbol{B} \perp$ [010] and strain $\perp$ [001]<br>• $\boldsymbol{B} \perp$ [010] and strain in generic direction<br>• $\boldsymbol{B} \perp$ [001] and strain $\perp$ [100]<br>• $\boldsymbol{B} \perp$ [001] and strain $\perp$ [010]<br>• $\boldsymbol{B} \perp$ [001] and strain in generic direction<br>• $\boldsymbol{B}$ in generic direction
$P2/m$ (10.42) $\mathbb{Z}_2 \times \mathbb{Z}_2 \times \mathbb{Z}_2$	• $\boldsymbol{B}$ ∥ [100] and strain $\perp$ [100]
$P2'/c'$ (13.69) $\mathbb{Z}_2 \times \mathbb{Z}_2 \times \mathbb{Z}_4$	• $\boldsymbol{B}$ ∥ [010] and strain $\perp$ [100]<br>• $\boldsymbol{B}$ ∥ [001] and strain $\perp$ [100]<br>• $\boldsymbol{B} \perp$ [100]
$P_S\bar{1}$ (2.7) $\mathbb{Z}_2 \times \mathbb{Z}_2 \times \mathbb{Z}_4$	• strain in generic direction
P_a2/m (10.47) $\mathbb{Z}_2 \times \mathbb{Z}_2 \times \mathbb{Z}_2$	• strain $\perp$ [100]
$P2$ (3.1) $\mathbb{Z}_2$	• $\boldsymbol{E}$ ∥ [010] and $\boldsymbol{B}$ ∥ [010] and strain $\perp$ [010]
$Pn'n'2$ (34.159) $\mathbb{Z}_1$	• $\boldsymbol{E}$ ∥ [010] and $\boldsymbol{B}$ ∥ [010]
$P2/c$ (13.65) $\mathbb{Z}_2 \times \mathbb{Z}_2$	• $\boldsymbol{B}$ ∥ [010] and strain $\perp$ [010]
$P2_1'$ (4.9) $\mathbb{Z}_1$	• $\boldsymbol{E}$ ∥ [010] and $\boldsymbol{B}$ ∥ [100] and strain $\perp$ [010]<br>• $\boldsymbol{E}$ ∥ [010] and $\boldsymbol{B}$ ∥ [001] and strain $\perp$ [010]<br>• $\boldsymbol{E}$ ∥ [010] and $\boldsymbol{B} \perp$ [010]
$Pmn'2_1'$ (31.126) $\mathbb{Z}_1$	• $\boldsymbol{E}$ ∥ [010] and $\boldsymbol{B}$ ∥ [100]
$Pn'a2_1'$ (33.146) $\mathbb{Z}_1$	• $\boldsymbol{E}$ ∥ [010] and $\boldsymbol{B}$ ∥ [001]
$P2_1'/c'$ (14.79) $\mathbb{Z}_2 \times \mathbb{Z}_4$	• $\boldsymbol{B}$ ∥ [100] and strain $\perp$ [010]<br>• $\boldsymbol{B}$ ∥ [001] and strain $\perp$ [010]<br>• $\boldsymbol{B} \perp$ [010]
P_C2 (3.6) $\mathbb{Z}_1$	• $\boldsymbol{E}$ ∥ [010] and strain $\perp$ [010]
P_Ama2 (28.95) $\mathbb{Z}_1$	• $\boldsymbol{E}$ ∥ [010]
P_C2/c (13.74) $\mathbb{Z}_2$	• strain $\perp$ [010]
$P2_1$ (4.7) $\mathbb{Z}_1$	• $\boldsymbol{E}$ ∥ [001] and $\boldsymbol{B}$ ∥ [001] and strain $\perp$ [001]

Continued on next page

…continued

Subgroup & SI group	Perturbation
$Pn'a'2_1$ (33.148) $\mathbb{Z}_1$	• $\boldsymbol{E}$ ∥ [001] and $\boldsymbol{B}$ ∥ [001]
$P2_1/c$ (14.75) $\mathbb{Z}_2$	• $\boldsymbol{B}$ ∥ [001] and strain ⊥ [001]
$Pc'cn'$ (56.370) $\mathbb{Z}_2$	• $\boldsymbol{B}$ ∥ [001]
$P2'$ (3.3) $\mathbb{Z}_1$	• $\boldsymbol{E}$ ∥ [001] and $\boldsymbol{B}$ ∥ [100] and strain ⊥ [001]<br>• $\boldsymbol{E}$ ∥ [001] and $\boldsymbol{B}$ ∥ [010] and strain ⊥ [001]<br>• $\boldsymbol{E}$ ∥ [001] and $\boldsymbol{B}$ ⊥ [001]
$Pma'2'$ (28.90) $\mathbb{Z}_1$	• $\boldsymbol{E}$ ∥ [001] and $\boldsymbol{B}$ ∥ [100]
$Pn'n2'$ (34.158) $\mathbb{Z}_1$	• $\boldsymbol{E}$ ∥ [001] and $\boldsymbol{B}$ ∥ [010]
$P2'/c'$ (13.69) $\mathbb{Z}_2 \times \mathbb{Z}_2 \times \mathbb{Z}_4$	• $\boldsymbol{B}$ ∥ [100] and strain ⊥ [001]<br>• $\boldsymbol{B}$ ∥ [010] and strain ⊥ [001]<br>• $\boldsymbol{B}$ ⊥ [001]
$Pn'n'n$ (48.260) $\mathbb{Z}_2$	• $\boldsymbol{B}$ ∥ [010]
$Pmn'a'$ (53.327) $\mathbb{Z}_2 \times \mathbb{Z}_2$	• $\boldsymbol{B}$ ∥ [100]
P_C2_1 (4.12) $\mathbb{Z}_1$	• $\boldsymbol{E}$ ∥ [001] and strain ⊥ [001]
P_Amn2_1 (31.131) $\mathbb{Z}_1$	• $\boldsymbol{E}$ ∥ [001]
P_C2_1/c (14.84) $\mathbb{Z}_2$	• strain ⊥ [001]

Table. S 444: Subgroups of P_Bmna (53.334) that can be realized by applying $\boldsymbol{E}/\boldsymbol{B}$ fields and/or uniaxial strains.

Subgroup & SI group	Perturbation
Pm' (6.20) $\mathbb{Z}_1$	• $\boldsymbol{E}$ ∥ [100] and $\boldsymbol{B}$ ∥ [100] and strain ⊥ [001]<br>• $\boldsymbol{E}$ ∥ [100] and $\boldsymbol{B}$ ∥ [010] and strain ⊥ [001]<br>• $\boldsymbol{E}$ ∥ [100] and $\boldsymbol{B}$ ⊥ [001]<br>• $\boldsymbol{E}$ ∥ [010] and $\boldsymbol{B}$ ∥ [100] and strain ⊥ [001]<br>• $\boldsymbol{E}$ ∥ [010] and $\boldsymbol{B}$ ∥ [010] and strain ⊥ [001]<br>• $\boldsymbol{E}$ ∥ [010] and $\boldsymbol{B}$ ⊥ [001]<br>• $\boldsymbol{E}$ ⊥ [001] and $\boldsymbol{B}$ ∥ [100]<br>• $\boldsymbol{E}$ ⊥ [001] and $\boldsymbol{B}$ ∥ [010]<br>• $\boldsymbol{E}$ ⊥ [001] and $\boldsymbol{B}$ ⊥ [001]

Continued on next page

...continued

Subgroup & SI group	Perturbation
Pm' (6.20) $\mathbb{Z}_1$	• $\boldsymbol{E} \parallel [100]$ and $\boldsymbol{B} \parallel [100]$ and strain $\perp [010]$<br>• $\boldsymbol{E} \parallel [100]$ and $\boldsymbol{B} \parallel [001]$ and strain $\perp [010]$<br>• $\boldsymbol{E} \parallel [100]$ and $\boldsymbol{B} \perp [010]$<br>• $\boldsymbol{E} \parallel [001]$ and $\boldsymbol{B} \parallel [100]$ and strain $\perp [010]$<br>• $\boldsymbol{E} \parallel [001]$ and $\boldsymbol{B} \parallel [001]$ and strain $\perp [010]$<br>• $\boldsymbol{E} \parallel [001]$ and $\boldsymbol{B} \perp [010]$<br>• $\boldsymbol{E} \perp [010]$ and $\boldsymbol{B} \parallel [100]$<br>• $\boldsymbol{E} \perp [010]$ and $\boldsymbol{B} \parallel [001]$<br>• $\boldsymbol{E} \perp [010]$ and $\boldsymbol{B} \perp [010]$
$P2$ (3.1) $\mathbb{Z}_2$	• $\boldsymbol{E} \parallel [100]$ and $\boldsymbol{B} \parallel [100]$ and strain $\perp [100]$
$Pm'm'2$ (25.60) $\mathbb{Z}_2$	• $\boldsymbol{E} \parallel [100]$ and $\boldsymbol{B} \parallel [100]$
P_S1 (1.3) $\mathbb{Z}_1$	• $\boldsymbol{E} \parallel [100]$ and strain in generic direction<br>• $\boldsymbol{E} \parallel [010]$ and strain in generic direction<br>• $\boldsymbol{E} \parallel [001]$ and strain in generic direction<br>• $\boldsymbol{E} \perp [100]$ and strain $\perp [010]$<br>• $\boldsymbol{E} \perp [100]$ and strain $\perp [001]$<br>• $\boldsymbol{E} \perp [100]$ and strain in generic direction<br>• $\boldsymbol{E} \perp [010]$ and strain $\perp [100]$<br>• $\boldsymbol{E} \perp [010]$ and strain $\perp [001]$<br>• $\boldsymbol{E} \perp [010]$ and strain in generic direction<br>• $\boldsymbol{E} \perp [001]$ and strain $\perp [100]$<br>• $\boldsymbol{E} \perp [001]$ and strain $\perp [010]$<br>• $\boldsymbol{E} \perp [001]$ and strain in generic direction<br>• $\boldsymbol{E}$ in generic direction
Pc (7.24) $\mathbb{Z}_1$	• $\boldsymbol{E} \parallel [100]$ and $\boldsymbol{B} \parallel [001]$ and strain $\perp [001]$<br>• $\boldsymbol{E} \parallel [010]$ and $\boldsymbol{B} \parallel [001]$ and strain $\perp [001]$<br>• $\boldsymbol{E} \perp [001]$ and $\boldsymbol{B} \parallel [001]$
P_Ac (7.31) $\mathbb{Z}_1$	• $\boldsymbol{E} \parallel [100]$ and strain $\perp [001]$<br>• $\boldsymbol{E} \parallel [010]$ and strain $\perp [001]$<br>• $\boldsymbol{E} \perp [001]$
Pc (7.24) $\mathbb{Z}_1$	• $\boldsymbol{E} \parallel [100]$ and $\boldsymbol{B} \parallel [010]$ and strain $\perp [010]$<br>• $\boldsymbol{E} \parallel [001]$ and $\boldsymbol{B} \parallel [010]$ and strain $\perp [010]$<br>• $\boldsymbol{E} \perp [010]$ and $\boldsymbol{B} \parallel [010]$
P_cc (7.28) $\mathbb{Z}_1$	• $\boldsymbol{E} \parallel [100]$ and strain $\perp [010]$<br>• $\boldsymbol{E} \parallel [001]$ and strain $\perp [010]$<br>• $\boldsymbol{E} \perp [010]$

Continued on next page

…continued

Subgroup & SI group	Perturbation
$P2_1'$ (4.9) $\mathbb{Z}_1$	• $\boldsymbol{E} \parallel [100]$ and $\boldsymbol{B} \parallel [010]$ and strain $\perp [100]$<br>• $\boldsymbol{E} \parallel [100]$ and $\boldsymbol{B} \parallel [001]$ and strain $\perp [100]$<br>• $\boldsymbol{E} \parallel [100]$ and $\boldsymbol{B} \perp [100]$
P_C2 (3.6) $\mathbb{Z}_1$	• $\boldsymbol{E} \parallel [100]$ and strain $\perp [100]$
$Pm'c2_1'$ (26.68) $\mathbb{Z}_1$	• $\boldsymbol{E} \parallel [100]$ and $\boldsymbol{B} \parallel [001]$
$Pm'n2_1'$ (31.125) $\mathbb{Z}_1$	• $\boldsymbol{E} \parallel [100]$ and $\boldsymbol{B} \parallel [010]$
P_Anc2 (30.119) $\mathbb{Z}_1$	• $\boldsymbol{E} \parallel [100]$
Pm (6.18) $\mathbb{Z}_1$	• $\boldsymbol{E} \parallel [010]$ and $\boldsymbol{B} \parallel [100]$ and strain $\perp [100]$<br>• $\boldsymbol{E} \parallel [001]$ and $\boldsymbol{B} \parallel [100]$ and strain $\perp [100]$<br>• $\boldsymbol{E} \perp [100]$ and $\boldsymbol{B} \parallel [100]$
$P2'$ (3.3) $\mathbb{Z}_1$	• $\boldsymbol{E} \parallel [010]$ and $\boldsymbol{B} \parallel [100]$ and strain $\perp [010]$<br>• $\boldsymbol{E} \parallel [010]$ and $\boldsymbol{B} \parallel [001]$ and strain $\perp [010]$<br>• $\boldsymbol{E} \parallel [010]$ and $\boldsymbol{B} \perp [010]$
$Pm'm2'$ (25.59) $\mathbb{Z}_1$	• $\boldsymbol{E} \parallel [010]$ and $\boldsymbol{B} \parallel [100]$
$P2'$ (3.3) $\mathbb{Z}_1$	• $\boldsymbol{E} \parallel [001]$ and $\boldsymbol{B} \parallel [100]$ and strain $\perp [001]$<br>• $\boldsymbol{E} \parallel [001]$ and $\boldsymbol{B} \parallel [010]$ and strain $\perp [001]$<br>• $\boldsymbol{E} \parallel [001]$ and $\boldsymbol{B} \perp [001]$
$Pm'm2'$ (25.59) $\mathbb{Z}_1$	• $\boldsymbol{E} \parallel [001]$ and $\boldsymbol{B} \parallel [100]$
$P\bar{1}$ (2.4) $\mathbb{Z}_2 \times \mathbb{Z}_2 \times \mathbb{Z}_2 \times \mathbb{Z}_4$	• $\boldsymbol{B} \parallel [100]$ and strain in generic direction<br>• $\boldsymbol{B} \parallel [010]$ and strain in generic direction<br>• $\boldsymbol{B} \parallel [001]$ and strain in generic direction<br>• $\boldsymbol{B} \perp [100]$ and strain $\perp [010]$<br>• $\boldsymbol{B} \perp [100]$ and strain $\perp [001]$<br>• $\boldsymbol{B} \perp [100]$ and strain in generic direction<br>• $\boldsymbol{B} \perp [010]$ and strain $\perp [100]$<br>• $\boldsymbol{B} \perp [010]$ and strain $\perp [001]$<br>• $\boldsymbol{B} \perp [010]$ and strain in generic direction<br>• $\boldsymbol{B} \perp [001]$ and strain $\perp [100]$<br>• $\boldsymbol{B} \perp [001]$ and strain $\perp [010]$<br>• $\boldsymbol{B} \perp [001]$ and strain in generic direction<br>• $\boldsymbol{B}$ in generic direction
$P2/m$ (10.42) $\mathbb{Z}_2 \times \mathbb{Z}_2 \times \mathbb{Z}_2$	• $\boldsymbol{B} \parallel [100]$ and strain $\perp [100]$
$P2'/m'$ (10.46) $\mathbb{Z}_2 \times \mathbb{Z}_2 \times \mathbb{Z}_2 \times \mathbb{Z}_4$	• $\boldsymbol{B} \parallel [100]$ and strain $\perp [010]$<br>• $\boldsymbol{B} \parallel [001]$ and strain $\perp [010]$<br>• $\boldsymbol{B} \perp [010]$

Continued on next page

. . . continued

Subgroup & SI group	Perturbation
$P2'/m'$ (10.46) $\mathbb{Z}_2 \times \mathbb{Z}_2 \times \mathbb{Z}_2 \times \mathbb{Z}_4$	• $\boldsymbol{B} \parallel [100]$ and strain $\perp [001]$<br>• $\boldsymbol{B} \parallel [010]$ and strain $\perp [001]$<br>• $\boldsymbol{B} \perp [001]$
$Pm'm'm$ (47.252) $\mathbb{Z}_2 \times \mathbb{Z}_2 \times \mathbb{Z}_2$	• $\boldsymbol{B} \parallel [100]$
Pc' (7.26) $\mathbb{Z}_1$	• $\boldsymbol{E} \parallel [010]$ and $\boldsymbol{B} \parallel [010]$ and strain $\perp [100]$<br>• $\boldsymbol{E} \parallel [010]$ and $\boldsymbol{B} \parallel [001]$ and strain $\perp [100]$<br>• $\boldsymbol{E} \parallel [010]$ and $\boldsymbol{B} \perp [100]$<br>• $\boldsymbol{E} \parallel [001]$ and $\boldsymbol{B} \parallel [010]$ and strain $\perp [100]$<br>• $\boldsymbol{E} \parallel [001]$ and $\boldsymbol{B} \parallel [001]$ and strain $\perp [100]$<br>• $\boldsymbol{E} \parallel [001]$ and $\boldsymbol{B} \perp [100]$<br>• $\boldsymbol{E} \perp [100]$ and $\boldsymbol{B} \parallel [010]$<br>• $\boldsymbol{E} \perp [100]$ and $\boldsymbol{B} \parallel [001]$<br>• $\boldsymbol{E} \perp [100]$ and $\boldsymbol{B} \perp [100]$
P_Cm (6.23) $\mathbb{Z}_1$	• $\boldsymbol{E} \parallel [010]$ and strain $\perp [100]$<br>• $\boldsymbol{E} \parallel [001]$ and strain $\perp [100]$<br>• $\boldsymbol{E} \perp [100]$
$Pb'a2'$ (32.137) $\mathbb{Z}_1$	• $\boldsymbol{E} \parallel [010]$ and $\boldsymbol{B} \parallel [001]$
$Pnc'2'$ (30.114) $\mathbb{Z}_1$	• $\boldsymbol{E} \parallel [001]$ and $\boldsymbol{B} \parallel [010]$
$P2_1'/c'$ (14.79) $\mathbb{Z}_2 \times \mathbb{Z}_4$	• $\boldsymbol{B} \parallel [010]$ and strain $\perp [100]$<br>• $\boldsymbol{B} \parallel [001]$ and strain $\perp [100]$<br>• $\boldsymbol{B} \perp [100]$
$P2$ (3.1) $\mathbb{Z}_2$	• $\boldsymbol{E} \parallel [010]$ and $\boldsymbol{B} \parallel [010]$ and strain $\perp [010]$
P_a2 (3.4) $\mathbb{Z}_2$	• $\boldsymbol{E} \parallel [010]$ and strain $\perp [010]$
$P2/c$ (13.65) $\mathbb{Z}_2 \times \mathbb{Z}_2$	• $\boldsymbol{B} \parallel [010]$ and strain $\perp [010]$
$Pm'a'2$ (28.91) $\mathbb{Z}_2$	• $\boldsymbol{E} \parallel [010]$ and $\boldsymbol{B} \parallel [010]$
P_Cma2 (28.97) $\mathbb{Z}_1$	• $\boldsymbol{E} \parallel [010]$
$Pm'na'$ (53.328) $\mathbb{Z}_2 \times \mathbb{Z}_2$	• $\boldsymbol{B} \parallel [010]$
$P2_1$ (4.7) $\mathbb{Z}_1$	• $\boldsymbol{E} \parallel [001]$ and $\boldsymbol{B} \parallel [001]$ and strain $\perp [001]$
P_C2_1 (4.12) $\mathbb{Z}_1$	• $\boldsymbol{E} \parallel [001]$ and strain $\perp [001]$
$P2_1/c$ (14.75) $\mathbb{Z}_2$	• $\boldsymbol{B} \parallel [001]$ and strain $\perp [001]$
$Pm'c'2_1$ (26.70) $\mathbb{Z}_1$	• $\boldsymbol{E} \parallel [001]$ and $\boldsymbol{B} \parallel [001]$
P_Bmn2_1 (31.132) $\mathbb{Z}_1$	• $\boldsymbol{E} \parallel [001]$
$Pb'am'$ (55.358) $\mathbb{Z}_2$	• $\boldsymbol{B} \parallel [001]$

Continued on next page

. . . continued

Subgroup & SI group	Perturbation
$P_S\bar{1}$ (2.7) $\mathbb{Z}_2 \times \mathbb{Z}_2 \times \mathbb{Z}_4$	• strain in generic direction
P_C2/m (10.49) $\mathbb{Z}_2 \times \mathbb{Z}_2$	• strain ⊥ [100]
P_c2/c (13.72) $\mathbb{Z}_2 \times \mathbb{Z}_2$	• strain ⊥ [010]
P_A2_1/c (14.83) $\mathbb{Z}_2$	• strain ⊥ [001]

Table. S 445: Subgroups of P_Cmna (53.335) that can be realized by applying $\boldsymbol{E}/\boldsymbol{B}$ fields and/or uniaxial strains.

Subgroup & SI group	Perturbation
Pc' (7.26) $\mathbb{Z}_1$	• $\boldsymbol{E}$ ∥ [100] and $\boldsymbol{B}$ ∥ [100] and strain ⊥ [001]<br>• $\boldsymbol{E}$ ∥ [100] and $\boldsymbol{B}$ ∥ [010] and strain ⊥ [001]<br>• $\boldsymbol{E}$ ∥ [100] and $\boldsymbol{B}$ ⊥ [001]<br>• $\boldsymbol{E}$ ∥ [010] and $\boldsymbol{B}$ ∥ [100] and strain ⊥ [001]<br>• $\boldsymbol{E}$ ∥ [010] and $\boldsymbol{B}$ ∥ [010] and strain ⊥ [001]<br>• $\boldsymbol{E}$ ∥ [010] and $\boldsymbol{B}$ ⊥ [001]<br>• $\boldsymbol{E}$ ⊥ [001] and $\boldsymbol{B}$ ∥ [100]<br>• $\boldsymbol{E}$ ⊥ [001] and $\boldsymbol{B}$ ∥ [010]<br>• $\boldsymbol{E}$ ⊥ [001] and $\boldsymbol{B}$ ⊥ [001]
$P2$ (3.1) $\mathbb{Z}_2$	• $\boldsymbol{E}$ ∥ [100] and $\boldsymbol{B}$ ∥ [100] and strain ⊥ [100]
Pc' (7.26) $\mathbb{Z}_1$	• $\boldsymbol{E}$ ∥ [100] and $\boldsymbol{B}$ ∥ [100] and strain ⊥ [010]<br>• $\boldsymbol{E}$ ∥ [100] and $\boldsymbol{B}$ ∥ [001] and strain ⊥ [010]<br>• $\boldsymbol{E}$ ∥ [100] and $\boldsymbol{B}$ ⊥ [010]<br>• $\boldsymbol{E}$ ∥ [001] and $\boldsymbol{B}$ ∥ [100] and strain ⊥ [010]<br>• $\boldsymbol{E}$ ∥ [001] and $\boldsymbol{B}$ ∥ [001] and strain ⊥ [010]<br>• $\boldsymbol{E}$ ∥ [001] and $\boldsymbol{B}$ ⊥ [010]<br>• $\boldsymbol{E}$ ⊥ [010] and $\boldsymbol{B}$ ∥ [100]<br>• $\boldsymbol{E}$ ⊥ [010] and $\boldsymbol{B}$ ∥ [001]<br>• $\boldsymbol{E}$ ⊥ [010] and $\boldsymbol{B}$ ⊥ [010]
$Pb'a'2$ (32.138) $\mathbb{Z}_2$	• $\boldsymbol{E}$ ∥ [100] and $\boldsymbol{B}$ ∥ [100]
Pc (7.24) $\mathbb{Z}_1$	• $\boldsymbol{E}$ ∥ [100] and $\boldsymbol{B}$ ∥ [001] and strain ⊥ [001]<br>• $\boldsymbol{E}$ ∥ [010] and $\boldsymbol{B}$ ∥ [001] and strain ⊥ [001]<br>• $\boldsymbol{E}$ ⊥ [001] and $\boldsymbol{B}$ ∥ [001]

Continued on next page

...continued

Subgroup & SI group	Perturbation
P_S1 (1.3) $\mathbb{Z}_1$	• $\boldsymbol{E}$ ∥ [100] and strain in generic direction<br>• $\boldsymbol{E}$ ∥ [010] and strain in generic direction<br>• $\boldsymbol{E}$ ∥ [001] and strain in generic direction<br>• $\boldsymbol{E} \perp$ [100] and strain $\perp$ [010]<br>• $\boldsymbol{E} \perp$ [100] and strain $\perp$ [001]<br>• $\boldsymbol{E} \perp$ [100] and strain in generic direction<br>• $\boldsymbol{E} \perp$ [010] and strain $\perp$ [100]<br>• $\boldsymbol{E} \perp$ [010] and strain $\perp$ [001]<br>• $\boldsymbol{E} \perp$ [010] and strain in generic direction<br>• $\boldsymbol{E} \perp$ [001] and strain $\perp$ [100]<br>• $\boldsymbol{E} \perp$ [001] and strain $\perp$ [010]<br>• $\boldsymbol{E} \perp$ [001] and strain in generic direction<br>• $\boldsymbol{E}$ in generic direction
P_ac (7.27) $\mathbb{Z}_1$	• $\boldsymbol{E}$ ∥ [100] and strain $\perp$ [001]<br>• $\boldsymbol{E}$ ∥ [010] and strain $\perp$ [001]<br>• $\boldsymbol{E} \perp$ [001]
Pc (7.24) $\mathbb{Z}_1$	• $\boldsymbol{E}$ ∥ [100] and $\boldsymbol{B}$ ∥ [010] and strain $\perp$ [010]<br>• $\boldsymbol{E}$ ∥ [001] and $\boldsymbol{B}$ ∥ [010] and strain $\perp$ [010]<br>• $\boldsymbol{E} \perp$ [010] and $\boldsymbol{B}$ ∥ [010]
P_Cc (7.30) $\mathbb{Z}_1$	• $\boldsymbol{E}$ ∥ [100] and strain $\perp$ [010]<br>• $\boldsymbol{E}$ ∥ [001] and strain $\perp$ [010]<br>• $\boldsymbol{E} \perp$ [010]
$P2_1'$ (4.9) $\mathbb{Z}_1$	• $\boldsymbol{E}$ ∥ [100] and $\boldsymbol{B}$ ∥ [010] and strain $\perp$ [100]<br>• $\boldsymbol{E}$ ∥ [100] and $\boldsymbol{B}$ ∥ [001] and strain $\perp$ [100]<br>• $\boldsymbol{E}$ ∥ [100] and $\boldsymbol{B} \perp$ [100]
$Pca'2_1'$ (29.102) $\mathbb{Z}_1$	• $\boldsymbol{E}$ ∥ [100] and $\boldsymbol{B}$ ∥ [001]
P_C2 (3.6) $\mathbb{Z}_1$	• $\boldsymbol{E}$ ∥ [100] and strain $\perp$ [100]
$Pna'2_1'$ (33.147) $\mathbb{Z}_1$	• $\boldsymbol{E}$ ∥ [100] and $\boldsymbol{B}$ ∥ [010]
P_Bnc2 (30.120) $\mathbb{Z}_2$	• $\boldsymbol{E}$ ∥ [100]
Pm (6.18) $\mathbb{Z}_1$	• $\boldsymbol{E}$ ∥ [010] and $\boldsymbol{B}$ ∥ [100] and strain $\perp$ [100]<br>• $\boldsymbol{E}$ ∥ [001] and $\boldsymbol{B}$ ∥ [100] and strain $\perp$ [100]<br>• $\boldsymbol{E} \perp$ [100] and $\boldsymbol{B}$ ∥ [100]
$P2_1'$ (4.9) $\mathbb{Z}_1$	• $\boldsymbol{E}$ ∥ [010] and $\boldsymbol{B}$ ∥ [100] and strain $\perp$ [010]<br>• $\boldsymbol{E}$ ∥ [010] and $\boldsymbol{B}$ ∥ [001] and strain $\perp$ [010]<br>• $\boldsymbol{E}$ ∥ [010] and $\boldsymbol{B} \perp$ [010]
$Pmc'2_1'$ (26.69) $\mathbb{Z}_1$	• $\boldsymbol{E}$ ∥ [010] and $\boldsymbol{B}$ ∥ [100]

Continued on next page

...continued

Subgroup & SI group	Perturbation
$P\bar{1}$ (2.4) $\mathbb{Z}_2 \times \mathbb{Z}_2 \times \mathbb{Z}_2 \times \mathbb{Z}_4$	• $\boldsymbol{B} \parallel [100]$ and strain in generic direction<br>• $\boldsymbol{B} \parallel [010]$ and strain in generic direction<br>• $\boldsymbol{B} \parallel [001]$ and strain in generic direction<br>• $\boldsymbol{B} \perp [100]$ and strain $\perp [010]$<br>• $\boldsymbol{B} \perp [100]$ and strain $\perp [001]$<br>• $\boldsymbol{B} \perp [100]$ and strain in generic direction<br>• $\boldsymbol{B} \perp [010]$ and strain $\perp [100]$<br>• $\boldsymbol{B} \perp [010]$ and strain $\perp [001]$<br>• $\boldsymbol{B} \perp [010]$ and strain in generic direction<br>• $\boldsymbol{B} \perp [001]$ and strain $\perp [100]$<br>• $\boldsymbol{B} \perp [001]$ and strain $\perp [010]$<br>• $\boldsymbol{B} \perp [001]$ and strain in generic direction<br>• $\boldsymbol{B}$ in generic direction
$P2/m$ (10.42) $\mathbb{Z}_2 \times \mathbb{Z}_2 \times \mathbb{Z}_2$	• $\boldsymbol{B} \parallel [100]$ and strain $\perp [100]$
$P2_1'/c'$ (14.79) $\mathbb{Z}_2 \times \mathbb{Z}_4$	• $\boldsymbol{B} \parallel [100]$ and strain $\perp [010]$<br>• $\boldsymbol{B} \parallel [001]$ and strain $\perp [010]$<br>• $\boldsymbol{B} \perp [010]$
$P2_1'$ (4.9) $\mathbb{Z}_1$	• $\boldsymbol{E} \parallel [001]$ and $\boldsymbol{B} \parallel [100]$ and strain $\perp [001]$<br>• $\boldsymbol{E} \parallel [001]$ and $\boldsymbol{B} \parallel [010]$ and strain $\perp [001]$<br>• $\boldsymbol{E} \parallel [001]$ and $\boldsymbol{B} \perp [001]$
$Pmc'2_1'$ (26.69) $\mathbb{Z}_1$	• $\boldsymbol{E} \parallel [001]$ and $\boldsymbol{B} \parallel [100]$
$P2_1'/c'$ (14.79) $\mathbb{Z}_2 \times \mathbb{Z}_4$	• $\boldsymbol{B} \parallel [100]$ and strain $\perp [001]$<br>• $\boldsymbol{B} \parallel [010]$ and strain $\perp [001]$<br>• $\boldsymbol{B} \perp [001]$
$Pb'a'm$ (55.357) $\mathbb{Z}_2 \times \mathbb{Z}_2 \times \mathbb{Z}_2$	• $\boldsymbol{B} \parallel [100]$
$P2$ (3.1) $\mathbb{Z}_2$	• $\boldsymbol{E} \parallel [010]$ and $\boldsymbol{B} \parallel [010]$ and strain $\perp [010]$
P_C2 (3.6) $\mathbb{Z}_1$	• $\boldsymbol{E} \parallel [010]$ and strain $\perp [010]$
$P2/c$ (13.65) $\mathbb{Z}_2 \times \mathbb{Z}_2$	• $\boldsymbol{B} \parallel [010]$ and strain $\perp [010]$

Continued on next page

…continued

Subgroup & SI group	Perturbation
Pc' (7.26) $\mathbb{Z}_1$	• $\boldsymbol{E}$ ∥ [010] and $\boldsymbol{B}$ ∥ [010] and strain ⊥ [100]<br>• $\boldsymbol{E}$ ∥ [010] and $\boldsymbol{B}$ ∥ [001] and strain ⊥ [100]<br>• $\boldsymbol{E}$ ∥ [010] and $\boldsymbol{B}$ ⊥ [100]<br>• $\boldsymbol{E}$ ∥ [001] and $\boldsymbol{B}$ ∥ [010] and strain ⊥ [100]<br>• $\boldsymbol{E}$ ∥ [001] and $\boldsymbol{B}$ ∥ [001] and strain ⊥ [100]<br>• $\boldsymbol{E}$ ∥ [001] and $\boldsymbol{B}$ ⊥ [100]<br>• $\boldsymbol{E}$ ⊥ [100] and $\boldsymbol{B}$ ∥ [010]<br>• $\boldsymbol{E}$ ⊥ [100] and $\boldsymbol{B}$ ∥ [001]<br>• $\boldsymbol{E}$ ⊥ [100] and $\boldsymbol{B}$ ⊥ [100]
P_Cm (6.23) $\mathbb{Z}_1$	• $\boldsymbol{E}$ ∥ [010] and strain ⊥ [100]<br>• $\boldsymbol{E}$ ∥ [001] and strain ⊥ [100]<br>• $\boldsymbol{E}$ ⊥ [100]
$Pc'a2_1'$ (29.101) $\mathbb{Z}_1$	• $\boldsymbol{E}$ ∥ [010] and $\boldsymbol{B}$ ∥ [001]
$P2_1'/c'$ (14.79) $\mathbb{Z}_2 \times \mathbb{Z}_4$	• $\boldsymbol{B}$ ∥ [010] and strain ⊥ [100]<br>• $\boldsymbol{B}$ ∥ [001] and strain ⊥ [100]<br>• $\boldsymbol{B}$ ⊥ [100]
$Pna'2_1'$ (33.147) $\mathbb{Z}_1$	• $\boldsymbol{E}$ ∥ [001] and $\boldsymbol{B}$ ∥ [010]
$Pc'c'2$ (27.81) $\mathbb{Z}_2$	• $\boldsymbol{E}$ ∥ [010] and $\boldsymbol{B}$ ∥ [010]
P_Bma2 (28.96) $\mathbb{Z}_1$	• $\boldsymbol{E}$ ∥ [010]
$Pc'c'n$ (56.369) $\mathbb{Z}_2 \times \mathbb{Z}_2$	• $\boldsymbol{B}$ ∥ [010]
$P2_1$ (4.7) $\mathbb{Z}_1$	• $\boldsymbol{E}$ ∥ [001] and $\boldsymbol{B}$ ∥ [001] and strain ⊥ [001]
$P2_1/c$ (14.75) $\mathbb{Z}_2$	• $\boldsymbol{B}$ ∥ [001] and strain ⊥ [001]
P_a2_1 (4.10) $\mathbb{Z}_1$	• $\boldsymbol{E}$ ∥ [001] and strain ⊥ [001]
$Pc'a'2_1$ (29.103) $\mathbb{Z}_1$	• $\boldsymbol{E}$ ∥ [001] and $\boldsymbol{B}$ ∥ [001]
$Pb'c'a$ (61.436) $\mathbb{Z}_2$	• $\boldsymbol{B}$ ∥ [001]
P_Cmn2_1 (31.133) $\mathbb{Z}_1$	• $\boldsymbol{E}$ ∥ [001]
$P_S\bar{1}$ (2.7) $\mathbb{Z}_2 \times \mathbb{Z}_2 \times \mathbb{Z}_4$	• strain in generic direction
P_C2/c (13.74) $\mathbb{Z}_2$	• strain ⊥ [010]
P_C2/m (10.49) $\mathbb{Z}_2 \times \mathbb{Z}_2$	• strain ⊥ [100]
P_a2_1/c (14.80) $\mathbb{Z}_2$	• strain ⊥ [001]

Table. S 446: Subgroups of $P_I mna$ (53.336) that can be realized by applying $\boldsymbol{E}/\boldsymbol{B}$ fields and/or uniaxial strains.

Subgroup & SI group	Perturbation
Pc' (7.26) $\mathbb{Z}_1$	• $\boldsymbol{E} \parallel [100]$ and $\boldsymbol{B} \parallel [100]$ and strain $\perp [001]$<br>• $\boldsymbol{E} \parallel [100]$ and $\boldsymbol{B} \parallel [010]$ and strain $\perp [001]$<br>• $\boldsymbol{E} \parallel [100]$ and $\boldsymbol{B} \perp [001]$<br>• $\boldsymbol{E} \parallel [010]$ and $\boldsymbol{B} \parallel [100]$ and strain $\perp [001]$<br>• $\boldsymbol{E} \parallel [010]$ and $\boldsymbol{B} \parallel [010]$ and strain $\perp [001]$<br>• $\boldsymbol{E} \parallel [010]$ and $\boldsymbol{B} \perp [001]$<br>• $\boldsymbol{E} \perp [001]$ and $\boldsymbol{B} \parallel [100]$<br>• $\boldsymbol{E} \perp [001]$ and $\boldsymbol{B} \parallel [010]$<br>• $\boldsymbol{E} \perp [001]$ and $\boldsymbol{B} \perp [001]$
$P2$ (3.1) $\mathbb{Z}_2$	• $\boldsymbol{E} \parallel [100]$ and $\boldsymbol{B} \parallel [100]$ and strain $\perp [100]$
Pm' (6.20) $\mathbb{Z}_1$	• $\boldsymbol{E} \parallel [100]$ and $\boldsymbol{B} \parallel [100]$ and strain $\perp [010]$<br>• $\boldsymbol{E} \parallel [100]$ and $\boldsymbol{B} \parallel [001]$ and strain $\perp [010]$<br>• $\boldsymbol{E} \parallel [100]$ and $\boldsymbol{B} \perp [010]$<br>• $\boldsymbol{E} \parallel [001]$ and $\boldsymbol{B} \parallel [100]$ and strain $\perp [010]$<br>• $\boldsymbol{E} \parallel [001]$ and $\boldsymbol{B} \parallel [001]$ and strain $\perp [010]$<br>• $\boldsymbol{E} \parallel [001]$ and $\boldsymbol{B} \perp [010]$<br>• $\boldsymbol{E} \perp [010]$ and $\boldsymbol{B} \parallel [100]$<br>• $\boldsymbol{E} \perp [010]$ and $\boldsymbol{B} \parallel [001]$<br>• $\boldsymbol{E} \perp [010]$ and $\boldsymbol{B} \perp [010]$
$Pm'a'2$ (28.91) $\mathbb{Z}_2$	• $\boldsymbol{E} \parallel [100]$ and $\boldsymbol{B} \parallel [100]$
Pc (7.24) $\mathbb{Z}_1$	• $\boldsymbol{E} \parallel [100]$ and $\boldsymbol{B} \parallel [001]$ and strain $\perp [001]$<br>• $\boldsymbol{E} \parallel [010]$ and $\boldsymbol{B} \parallel [001]$ and strain $\perp [001]$<br>• $\boldsymbol{E} \perp [001]$ and $\boldsymbol{B} \parallel [001]$
P_S1 (1.3) $\mathbb{Z}_1$	• $\boldsymbol{E} \parallel [100]$ and strain in generic direction<br>• $\boldsymbol{E} \parallel [010]$ and strain in generic direction<br>• $\boldsymbol{E} \parallel [001]$ and strain in generic direction<br>• $\boldsymbol{E} \perp [100]$ and strain $\perp [010]$<br>• $\boldsymbol{E} \perp [100]$ and strain $\perp [001]$<br>• $\boldsymbol{E} \perp [100]$ and strain in generic direction<br>• $\boldsymbol{E} \perp [010]$ and strain $\perp [100]$<br>• $\boldsymbol{E} \perp [010]$ and strain $\perp [001]$<br>• $\boldsymbol{E} \perp [010]$ and strain in generic direction<br>• $\boldsymbol{E} \perp [001]$ and strain $\perp [100]$<br>• $\boldsymbol{E} \perp [001]$ and strain $\perp [010]$<br>• $\boldsymbol{E} \perp [001]$ and strain in generic direction<br>• $\boldsymbol{E}$ in generic direction

Continued on next page

...continued

Subgroup & SI group	Perturbation
P_Cc (7.30) $\mathbb{Z}_1$	• $\boldsymbol{E} \parallel [100]$ and strain $\perp [001]$<br>• $\boldsymbol{E} \parallel [010]$ and strain $\perp [001]$<br>• $\boldsymbol{E} \perp [001]$
Pc (7.24) $\mathbb{Z}_1$	• $\boldsymbol{E} \parallel [100]$ and $\boldsymbol{B} \parallel [010]$ and strain $\perp [010]$<br>• $\boldsymbol{E} \parallel [001]$ and $\boldsymbol{B} \parallel [010]$ and strain $\perp [010]$<br>• $\boldsymbol{E} \perp [010]$ and $\boldsymbol{B} \parallel [010]$
P_Ac (7.31) $\mathbb{Z}_1$	• $\boldsymbol{E} \parallel [100]$ and strain $\perp [010]$<br>• $\boldsymbol{E} \parallel [001]$ and strain $\perp [010]$<br>• $\boldsymbol{E} \perp [010]$
$P2_1'$ (4.9) $\mathbb{Z}_1$	• $\boldsymbol{E} \parallel [100]$ and $\boldsymbol{B} \parallel [010]$ and strain $\perp [100]$<br>• $\boldsymbol{E} \parallel [100]$ and $\boldsymbol{B} \parallel [001]$ and strain $\perp [100]$<br>• $\boldsymbol{E} \parallel [100]$ and $\boldsymbol{B} \perp [100]$
$Pm'c2_1'$ (26.68) $\mathbb{Z}_1$	• $\boldsymbol{E} \parallel [100]$ and $\boldsymbol{B} \parallel [001]$
P_C2 (3.6) $\mathbb{Z}_1$	• $\boldsymbol{E} \parallel [100]$ and strain $\perp [100]$
$Pna'2_1'$ (33.147) $\mathbb{Z}_1$	• $\boldsymbol{E} \parallel [100]$ and $\boldsymbol{B} \parallel [010]$
P_Inc2 (30.122) $\mathbb{Z}_1$	• $\boldsymbol{E} \parallel [100]$
Pm (6.18) $\mathbb{Z}_1$	• $\boldsymbol{E} \parallel [010]$ and $\boldsymbol{B} \parallel [100]$ and strain $\perp [100]$<br>• $\boldsymbol{E} \parallel [001]$ and $\boldsymbol{B} \parallel [100]$ and strain $\perp [100]$<br>• $\boldsymbol{E} \perp [100]$ and $\boldsymbol{B} \parallel [100]$
$P2_1'$ (4.9) $\mathbb{Z}_1$	• $\boldsymbol{E} \parallel [010]$ and $\boldsymbol{B} \parallel [100]$ and strain $\perp [010]$<br>• $\boldsymbol{E} \parallel [010]$ and $\boldsymbol{B} \parallel [001]$ and strain $\perp [010]$<br>• $\boldsymbol{E} \parallel [010]$ and $\boldsymbol{B} \perp [010]$
$Pmc'2_1'$ (26.69) $\mathbb{Z}_1$	• $\boldsymbol{E} \parallel [010]$ and $\boldsymbol{B} \parallel [100]$
$P\bar{1}$ (2.4) $\mathbb{Z}_2 \times \mathbb{Z}_2 \times \mathbb{Z}_2 \times \mathbb{Z}_4$	• $\boldsymbol{B} \parallel [100]$ and strain in generic direction<br>• $\boldsymbol{B} \parallel [010]$ and strain in generic direction<br>• $\boldsymbol{B} \parallel [001]$ and strain in generic direction<br>• $\boldsymbol{B} \perp [100]$ and strain $\perp [010]$<br>• $\boldsymbol{B} \perp [100]$ and strain $\perp [001]$<br>• $\boldsymbol{B} \perp [100]$ and strain in generic direction<br>• $\boldsymbol{B} \perp [010]$ and strain $\perp [100]$<br>• $\boldsymbol{B} \perp [010]$ and strain $\perp [001]$<br>• $\boldsymbol{B} \perp [010]$ and strain in generic direction<br>• $\boldsymbol{B} \perp [001]$ and strain $\perp [100]$<br>• $\boldsymbol{B} \perp [001]$ and strain $\perp [010]$<br>• $\boldsymbol{B} \perp [001]$ and strain in generic direction<br>• $\boldsymbol{B}$ in generic direction

Continued on next page

...continued

Subgroup & SI group	Perturbation
$P2/m$ (10.42) $\mathbb{Z}_2\times\mathbb{Z}_2\times\mathbb{Z}_2$	• $\boldsymbol{B}$ ∥ [100] and strain ⊥ [100]
$P2_1'/m'$ (11.54) $\mathbb{Z}_2\times\mathbb{Z}_2\times\mathbb{Z}_4$	• $\boldsymbol{B}$ ∥ [100] and strain ⊥ [010]<br>• $\boldsymbol{B}$ ∥ [001] and strain ⊥ [010]<br>• $\boldsymbol{B}$ ⊥ [010]
$P2'$ (3.3) $\mathbb{Z}_1$	• $\boldsymbol{E}$ ∥ [001] and $\boldsymbol{B}$ ∥ [100] and strain ⊥ [001]<br>• $\boldsymbol{E}$ ∥ [001] and $\boldsymbol{B}$ ∥ [010] and strain ⊥ [001]<br>• $\boldsymbol{E}$ ∥ [001] and $\boldsymbol{B}$ ⊥ [001]
$Pm'm2'$ (25.59) $\mathbb{Z}_1$	• $\boldsymbol{E}$ ∥ [001] and $\boldsymbol{B}$ ∥ [100]
$P2'/c'$ (13.69) $\mathbb{Z}_2\times\mathbb{Z}_2\times\mathbb{Z}_4$	• $\boldsymbol{B}$ ∥ [100] and strain ⊥ [001]<br>• $\boldsymbol{B}$ ∥ [010] and strain ⊥ [001]<br>• $\boldsymbol{B}$ ⊥ [001]
$Pm'ma'$ (51.296) $\mathbb{Z}_2\times\mathbb{Z}_2\times\mathbb{Z}_2$	• $\boldsymbol{B}$ ∥ [100]
$P2$ (3.1) $\mathbb{Z}_2$	• $\boldsymbol{E}$ ∥ [010] and $\boldsymbol{B}$ ∥ [010] and strain ⊥ [010]
P_C2 (3.6) $\mathbb{Z}_1$	• $\boldsymbol{E}$ ∥ [010] and strain ⊥ [010]
$P2/c$ (13.65) $\mathbb{Z}_2\times\mathbb{Z}_2$	• $\boldsymbol{B}$ ∥ [010] and strain ⊥ [010]
Pc' (7.26) $\mathbb{Z}_1$	• $\boldsymbol{E}$ ∥ [010] and $\boldsymbol{B}$ ∥ [010] and strain ⊥ [100]<br>• $\boldsymbol{E}$ ∥ [010] and $\boldsymbol{B}$ ∥ [001] and strain ⊥ [100]<br>• $\boldsymbol{E}$ ∥ [010] and $\boldsymbol{B}$ ⊥ [100]<br>• $\boldsymbol{E}$ ∥ [001] and $\boldsymbol{B}$ ∥ [010] and strain ⊥ [100]<br>• $\boldsymbol{E}$ ∥ [001] and $\boldsymbol{B}$ ∥ [001] and strain ⊥ [100]<br>• $\boldsymbol{E}$ ∥ [001] and $\boldsymbol{B}$ ⊥ [100]<br>• $\boldsymbol{E}$ ⊥ [100] and $\boldsymbol{B}$ ∥ [010]<br>• $\boldsymbol{E}$ ⊥ [100] and $\boldsymbol{B}$ ∥ [001]<br>• $\boldsymbol{E}$ ⊥ [100] and $\boldsymbol{B}$ ⊥ [100]
P_Cm (6.23) $\mathbb{Z}_1$	• $\boldsymbol{E}$ ∥ [010] and strain ⊥ [100]<br>• $\boldsymbol{E}$ ∥ [001] and strain ⊥ [100]<br>• $\boldsymbol{E}$ ⊥ [100]
$Pn'a2_1'$ (33.146) $\mathbb{Z}_1$	• $\boldsymbol{E}$ ∥ [010] and $\boldsymbol{B}$ ∥ [001]
$P2_1'/c'$ (14.79) $\mathbb{Z}_2\times\mathbb{Z}_4$	• $\boldsymbol{B}$ ∥ [010] and strain ⊥ [100]<br>• $\boldsymbol{B}$ ∥ [001] and strain ⊥ [100]<br>• $\boldsymbol{B}$ ⊥ [100]
$Pn'n2'$ (34.158) $\mathbb{Z}_1$	• $\boldsymbol{E}$ ∥ [001] and $\boldsymbol{B}$ ∥ [010]
$Pn'c'2$ (30.115) $\mathbb{Z}_1$	• $\boldsymbol{E}$ ∥ [010] and $\boldsymbol{B}$ ∥ [010]
P_Ima2 (28.98) $\mathbb{Z}_1$	• $\boldsymbol{E}$ ∥ [010]

Continued on next page

. . . continued

Subgroup & SI group	Perturbation
$Pnn'a'$ (52.311) $\mathbb{Z}_2$	• $\boldsymbol{B}$ ∥ [010]
$P2_1$ (4.7) $\mathbb{Z}_1$	• $\boldsymbol{E}$ ∥ [001] and $\boldsymbol{B}$ ∥ [001] and strain ⊥ [001]
$P2_1/c$ (14.75) $\mathbb{Z}_2$	• $\boldsymbol{B}$ ∥ [001] and strain ⊥ [001]
P_C2_1 (4.12) $\mathbb{Z}_1$	• $\boldsymbol{E}$ ∥ [001] and strain ⊥ [001]
$Pm'n'2_1$ (31.127) $\mathbb{Z}_1$	• $\boldsymbol{E}$ ∥ [001] and $\boldsymbol{B}$ ∥ [001]
$Pn'm'a$ (62.446) $\mathbb{Z}_2$	• $\boldsymbol{B}$ ∥ [001]
P_Imn2_1 (31.134) $\mathbb{Z}_1$	• $\boldsymbol{E}$ ∥ [001]
$P_S\bar{1}$ (2.7) $\mathbb{Z}_2 \times \mathbb{Z}_2 \times \mathbb{Z}_4$	• strain in generic direction
P_A2/c (13.73) $\mathbb{Z}_2$	• strain ⊥ [010]
P_C2/m (10.49) $\mathbb{Z}_2 \times \mathbb{Z}_2$	• strain ⊥ [100]
P_C2_1/c (14.84) $\mathbb{Z}_2$	• strain ⊥ [001]

Table. S 447: Subgroups of $Pcca$ (54.337) that can be realized by applying $\boldsymbol{E}/\boldsymbol{B}$ fields and/or uniaxial strains.

Subgroup & SI group	Perturbation
Pc (7.24) $\mathbb{Z}_1$	• $\boldsymbol{E}$ ∥ [100] and strain ⊥ [010]<br>• $\boldsymbol{E}$ ∥ [100] and $\boldsymbol{B}$ ∥ [010]<br>• $\boldsymbol{E}$ ∥ [001] and strain ⊥ [010]<br>• $\boldsymbol{E}$ ∥ [001] and $\boldsymbol{B}$ ∥ [010]<br>• $\boldsymbol{E}$ ⊥ [010]
Pc (7.24) $\mathbb{Z}_1$	• $\boldsymbol{E}$ ∥ [100] and strain ⊥ [001]<br>• $\boldsymbol{E}$ ∥ [100] and $\boldsymbol{B}$ ∥ [001]<br>• $\boldsymbol{E}$ ∥ [010] and strain ⊥ [001]<br>• $\boldsymbol{E}$ ∥ [010] and $\boldsymbol{B}$ ∥ [001]<br>• $\boldsymbol{E}$ ⊥ [001]
$P2_1$ (4.7) $\mathbb{Z}_1$	• $\boldsymbol{E}$ ∥ [100] and strain ⊥ [100]<br>• $\boldsymbol{E}$ ∥ [100] and $\boldsymbol{B}$ ∥ [100]
$Pca2_1$ (29.99) $\mathbb{Z}_1$	• $\boldsymbol{E}$ ∥ [100]
$P2$ (3.1) $\mathbb{Z}_2$	• $\boldsymbol{E}$ ∥ [010] and strain ⊥ [010]<br>• $\boldsymbol{E}$ ∥ [010] and $\boldsymbol{B}$ ∥ [010]

Continued on next page

...continued

Subgroup & SI group	Perturbation
$P\bar{1}$ (2.4) $\mathbb{Z}_2\times\mathbb{Z}_2\times\mathbb{Z}_2\times\mathbb{Z}_4$	• strain in generic direction<br>• $\boldsymbol{B}\parallel[100]$ and strain $\perp[010]$<br>• $\boldsymbol{B}\parallel[100]$ and strain $\perp[001]$<br>• $\boldsymbol{B}\parallel[010]$ and strain $\perp[100]$<br>• $\boldsymbol{B}\parallel[010]$ and strain $\perp[001]$<br>• $\boldsymbol{B}\parallel[001]$ and strain $\perp[100]$<br>• $\boldsymbol{B}\parallel[001]$ and strain $\perp[010]$<br>• $\boldsymbol{B}$ in generic direction
$P2/c$ (13.65) $\mathbb{Z}_2\times\mathbb{Z}_2$	• strain $\perp[010]$<br>• $\boldsymbol{B}\parallel[010]$
Pc (7.24) $\mathbb{Z}_1$	• $\boldsymbol{E}\parallel[010]$ and strain $\perp[100]$<br>• $\boldsymbol{E}\parallel[010]$ and $\boldsymbol{B}\parallel[100]$<br>• $\boldsymbol{E}\parallel[001]$ and strain $\perp[100]$<br>• $\boldsymbol{E}\parallel[001]$ and $\boldsymbol{B}\parallel[100]$<br>• $\boldsymbol{E}\perp[100]$
$Pba2$ (32.135) $\mathbb{Z}_1$	• $\boldsymbol{E}\parallel[010]$
$P2_1/c$ (14.75) $\mathbb{Z}_2$	• strain $\perp[100]$<br>• $\boldsymbol{B}\parallel[100]$
$P2$ (3.1) $\mathbb{Z}_2$	• $\boldsymbol{E}\parallel[001]$ and strain $\perp[001]$<br>• $\boldsymbol{E}\parallel[001]$ and $\boldsymbol{B}\parallel[001]$
$P2/c$ (13.65) $\mathbb{Z}_2\times\mathbb{Z}_2$	• strain $\perp[001]$<br>• $\boldsymbol{B}\parallel[001]$
$Pcc2$ (27.78) $\mathbb{Z}_2$	• $\boldsymbol{E}\parallel[001]$

Table. S 448: Subgroups of $Pcca1'$ (54.338) that can be realized by applying $\boldsymbol{E}/\boldsymbol{B}$ fields and/or uniaxial strains.

Subgroup & SI group	Perturbation

Continued on next page

...continued

Subgroup & SI group	Perturbation
$P11'$ (1.2) $\mathbb{Z}_1$	• $\boldsymbol{E}$ ∥ [100] and strain in generic direction<br>• $\boldsymbol{E}$ ∥ [010] and strain in generic direction<br>• $\boldsymbol{E}$ ∥ [001] and strain in generic direction<br>• $\boldsymbol{E}$ ⊥ [100] and strain ⊥ [010]<br>• $\boldsymbol{E}$ ⊥ [100] and strain ⊥ [001]<br>• $\boldsymbol{E}$ ⊥ [100] and strain in generic direction<br>• $\boldsymbol{E}$ ⊥ [010] and strain ⊥ [100]<br>• $\boldsymbol{E}$ ⊥ [010] and strain ⊥ [001]<br>• $\boldsymbol{E}$ ⊥ [010] and strain in generic direction<br>• $\boldsymbol{E}$ ⊥ [001] and strain ⊥ [100]<br>• $\boldsymbol{E}$ ⊥ [001] and strain ⊥ [010]<br>• $\boldsymbol{E}$ ⊥ [001] and strain in generic direction<br>• $\boldsymbol{E}$ in generic direction
Pc' (7.26) $\mathbb{Z}_1$	• $\boldsymbol{E}$ ∥ [100] and $\boldsymbol{B}$ ∥ [100] and strain ⊥ [010]<br>• $\boldsymbol{E}$ ∥ [100] and $\boldsymbol{B}$ ∥ [001] and strain ⊥ [010]<br>• $\boldsymbol{E}$ ∥ [100] and $\boldsymbol{B}$ ⊥ [010]<br>• $\boldsymbol{E}$ ∥ [001] and $\boldsymbol{B}$ ∥ [100] and strain ⊥ [010]<br>• $\boldsymbol{E}$ ∥ [001] and $\boldsymbol{B}$ ∥ [001] and strain ⊥ [010]<br>• $\boldsymbol{E}$ ∥ [001] and $\boldsymbol{B}$ ⊥ [010]<br>• $\boldsymbol{E}$ ⊥ [010] and $\boldsymbol{B}$ ∥ [100]<br>• $\boldsymbol{E}$ ⊥ [010] and $\boldsymbol{B}$ ∥ [001]<br>• $\boldsymbol{E}$ ⊥ [010] and $\boldsymbol{B}$ ⊥ [010]
Pc (7.24) $\mathbb{Z}_1$	• $\boldsymbol{E}$ ∥ [100] and $\boldsymbol{B}$ ∥ [010] and strain ⊥ [010]<br>• $\boldsymbol{E}$ ∥ [001] and $\boldsymbol{B}$ ∥ [010] and strain ⊥ [010]<br>• $\boldsymbol{E}$ ⊥ [010] and $\boldsymbol{B}$ ∥ [010]
$Pc1'$ (7.25) $\mathbb{Z}_1$	• $\boldsymbol{E}$ ∥ [100] and strain ⊥ [010]<br>• $\boldsymbol{E}$ ∥ [001] and strain ⊥ [010]<br>• $\boldsymbol{E}$ ⊥ [010]
Pc' (7.26) $\mathbb{Z}_1$	• $\boldsymbol{E}$ ∥ [100] and $\boldsymbol{B}$ ∥ [100] and strain ⊥ [001]<br>• $\boldsymbol{E}$ ∥ [100] and $\boldsymbol{B}$ ∥ [010] and strain ⊥ [001]<br>• $\boldsymbol{E}$ ∥ [100] and $\boldsymbol{B}$ ⊥ [001]<br>• $\boldsymbol{E}$ ∥ [010] and $\boldsymbol{B}$ ∥ [100] and strain ⊥ [001]<br>• $\boldsymbol{E}$ ∥ [010] and $\boldsymbol{B}$ ∥ [010] and strain ⊥ [001]<br>• $\boldsymbol{E}$ ∥ [010] and $\boldsymbol{B}$ ⊥ [001]<br>• $\boldsymbol{E}$ ⊥ [001] and $\boldsymbol{B}$ ∥ [100]<br>• $\boldsymbol{E}$ ⊥ [001] and $\boldsymbol{B}$ ∥ [010]<br>• $\boldsymbol{E}$ ⊥ [001] and $\boldsymbol{B}$ ⊥ [001]

Continued on next page

...continued

Subgroup & SI group	Perturbation
Pc (7.24) $\mathbb{Z}_1$	• $\boldsymbol{E} \parallel [100]$ and $\boldsymbol{B} \parallel [001]$ and strain $\perp [001]$<br>• $\boldsymbol{E} \parallel [010]$ and $\boldsymbol{B} \parallel [001]$ and strain $\perp [001]$<br>• $\boldsymbol{E} \perp [001]$ and $\boldsymbol{B} \parallel [001]$
$Pc1'$ (7.25) $\mathbb{Z}_1$	• $\boldsymbol{E} \parallel [100]$ and strain $\perp [001]$<br>• $\boldsymbol{E} \parallel [010]$ and strain $\perp [001]$<br>• $\boldsymbol{E} \perp [001]$
$P2_1'$ (4.9) $\mathbb{Z}_1$	• $\boldsymbol{E} \parallel [100]$ and $\boldsymbol{B} \parallel [010]$ and strain $\perp [100]$<br>• $\boldsymbol{E} \parallel [100]$ and $\boldsymbol{B} \parallel [001]$ and strain $\perp [100]$<br>• $\boldsymbol{E} \parallel [100]$ and $\boldsymbol{B} \perp [100]$
$Pc'a2_1'$ (29.101) $\mathbb{Z}_1$	• $\boldsymbol{E} \parallel [100]$ and $\boldsymbol{B} \parallel [010]$
$Pca'2_1'$ (29.102) $\mathbb{Z}_1$	• $\boldsymbol{E} \parallel [100]$ and $\boldsymbol{B} \parallel [001]$
$P2_1$ (4.7) $\mathbb{Z}_1$	• $\boldsymbol{E} \parallel [100]$ and $\boldsymbol{B} \parallel [100]$ and strain $\perp [100]$
$Pc'a'2_1$ (29.103) $\mathbb{Z}_1$	• $\boldsymbol{E} \parallel [100]$ and $\boldsymbol{B} \parallel [100]$
$P2_11'$ (4.8) $\mathbb{Z}_1$	• $\boldsymbol{E} \parallel [100]$ and strain $\perp [100]$
$Pca2_11'$ (29.100) $\mathbb{Z}_1$	• $\boldsymbol{E} \parallel [100]$
$P2'$ (3.3) $\mathbb{Z}_1$	• $\boldsymbol{E} \parallel [010]$ and $\boldsymbol{B} \parallel [100]$ and strain $\perp [010]$<br>• $\boldsymbol{E} \parallel [010]$ and $\boldsymbol{B} \parallel [001]$ and strain $\perp [010]$<br>• $\boldsymbol{E} \parallel [010]$ and $\boldsymbol{B} \perp [010]$
$P2$ (3.1) $\mathbb{Z}_2$	• $\boldsymbol{E} \parallel [010]$ and $\boldsymbol{B} \parallel [010]$ and strain $\perp [010]$
$P21'$ (3.2) $\mathbb{Z}_2$	• $\boldsymbol{E} \parallel [010]$ and strain $\perp [010]$
$P\bar{1}$ (2.4) $\mathbb{Z}_2 \times \mathbb{Z}_2 \times \mathbb{Z}_2 \times \mathbb{Z}_4$	• $\boldsymbol{B} \parallel [100]$ and strain in generic direction<br>• $\boldsymbol{B} \parallel [010]$ and strain in generic direction<br>• $\boldsymbol{B} \parallel [001]$ and strain in generic direction<br>• $\boldsymbol{B} \perp [100]$ and strain $\perp [010]$<br>• $\boldsymbol{B} \perp [100]$ and strain $\perp [001]$<br>• $\boldsymbol{B} \perp [100]$ and strain in generic direction<br>• $\boldsymbol{B} \perp [010]$ and strain $\perp [100]$<br>• $\boldsymbol{B} \perp [010]$ and strain $\perp [001]$<br>• $\boldsymbol{B} \perp [010]$ and strain in generic direction<br>• $\boldsymbol{B} \perp [001]$ and strain $\perp [100]$<br>• $\boldsymbol{B} \perp [001]$ and strain $\perp [010]$<br>• $\boldsymbol{B} \perp [001]$ and strain in generic direction<br>• $\boldsymbol{B}$ in generic direction

Continued on next page

...continued

Subgroup & SI group	Perturbation
$P2'/c'$ (13.69) $\mathbb{Z}_2 \times \mathbb{Z}_2 \times \mathbb{Z}_4$	• $\boldsymbol{B} \parallel [100]$ and strain $\perp [010]$<br>• $\boldsymbol{B} \parallel [001]$ and strain $\perp [010]$<br>• $\boldsymbol{B} \perp [010]$
$P2/c$ (13.65) $\mathbb{Z}_2 \times \mathbb{Z}_2$	• $\boldsymbol{B} \parallel [010]$ and strain $\perp [010]$
$P\bar{1}1'$ (2.5) $\mathbb{Z}_2 \times \mathbb{Z}_2 \times \mathbb{Z}_2 \times \mathbb{Z}_4$	• strain in generic direction
$P2/c1'$ (13.66) $\mathbb{Z}_2 \times \mathbb{Z}_2$	• strain $\perp [010]$
Pc' (7.26) $\mathbb{Z}_1$	• $\boldsymbol{E} \parallel [010]$ and $\boldsymbol{B} \parallel [010]$ and strain $\perp [100]$<br>• $\boldsymbol{E} \parallel [010]$ and $\boldsymbol{B} \parallel [001]$ and strain $\perp [100]$<br>• $\boldsymbol{E} \parallel [010]$ and $\boldsymbol{B} \perp [100]$<br>• $\boldsymbol{E} \parallel [001]$ and $\boldsymbol{B} \parallel [010]$ and strain $\perp [100]$<br>• $\boldsymbol{E} \parallel [001]$ and $\boldsymbol{B} \parallel [001]$ and strain $\perp [100]$<br>• $\boldsymbol{E} \parallel [001]$ and $\boldsymbol{B} \perp [100]$<br>• $\boldsymbol{E} \perp [100]$ and $\boldsymbol{B} \parallel [010]$<br>• $\boldsymbol{E} \perp [100]$ and $\boldsymbol{B} \parallel [001]$<br>• $\boldsymbol{E} \perp [100]$ and $\boldsymbol{B} \perp [100]$
$Pb'a2'$ (32.137) $\mathbb{Z}_1$	• $\boldsymbol{E} \parallel [010]$ and $\boldsymbol{B} \parallel [001]$
$Pb'a'2$ (32.138) $\mathbb{Z}_2$	• $\boldsymbol{E} \parallel [010]$ and $\boldsymbol{B} \parallel [010]$
$P2_1'/c'$ (14.79) $\mathbb{Z}_2 \times \mathbb{Z}_4$	• $\boldsymbol{B} \parallel [010]$ and strain $\perp [100]$<br>• $\boldsymbol{B} \parallel [001]$ and strain $\perp [100]$<br>• $\boldsymbol{B} \perp [100]$
Pc (7.24) $\mathbb{Z}_1$	• $\boldsymbol{E} \parallel [010]$ and $\boldsymbol{B} \parallel [100]$ and strain $\perp [100]$<br>• $\boldsymbol{E} \parallel [001]$ and $\boldsymbol{B} \parallel [100]$ and strain $\perp [100]$<br>• $\boldsymbol{E} \perp [100]$ and $\boldsymbol{B} \parallel [100]$
$Pb'a2'$ (32.137) $\mathbb{Z}_1$	• $\boldsymbol{E} \parallel [010]$ and $\boldsymbol{B} \parallel [100]$
$P2_1/c$ (14.75) $\mathbb{Z}_2$	• $\boldsymbol{B} \parallel [100]$ and strain $\perp [100]$
$Pc1'$ (7.25) $\mathbb{Z}_1$	• $\boldsymbol{E} \parallel [010]$ and strain $\perp [100]$<br>• $\boldsymbol{E} \parallel [001]$ and strain $\perp [100]$<br>• $\boldsymbol{E} \perp [100]$
$Pba21'$ (32.136) $\mathbb{Z}_1$	• $\boldsymbol{E} \parallel [010]$
$P2_1/c1'$ (14.76) $\mathbb{Z}_2$	• strain $\perp [100]$
$P2'$ (3.3) $\mathbb{Z}_1$	• $\boldsymbol{E} \parallel [001]$ and $\boldsymbol{B} \parallel [100]$ and strain $\perp [001]$<br>• $\boldsymbol{E} \parallel [001]$ and $\boldsymbol{B} \parallel [010]$ and strain $\perp [001]$<br>• $\boldsymbol{E} \parallel [001]$ and $\boldsymbol{B} \perp [001]$

Continued on next page

...continued

Subgroup & SI group	Perturbation
$P2'/c'$ (13.69) $\mathbb{Z}_2\times\mathbb{Z}_2\times\mathbb{Z}_4$	• $\boldsymbol{B}$ $\parallel$ [100] and strain $\perp$ [001]<br>• $\boldsymbol{B}$ $\parallel$ [010] and strain $\perp$ [001]<br>• $\boldsymbol{B}$ $\perp$ [001]
$Pc'c2'$ (27.80) $\mathbb{Z}_1$	• $\boldsymbol{E}$ $\parallel$ [001] and $\boldsymbol{B}$ $\parallel$ [010]
$Pc'ca'$ (54.344) $\mathbb{Z}_2\times\mathbb{Z}_2$	• $\boldsymbol{B}$ $\parallel$ [010]
$Pc'c2'$ (27.80) $\mathbb{Z}_1$	• $\boldsymbol{E}$ $\parallel$ [001] and $\boldsymbol{B}$ $\parallel$ [100]
$Pcc'a'$ (54.343) $\mathbb{Z}_2$	• $\boldsymbol{B}$ $\parallel$ [100]
$P2$ (3.1) $\mathbb{Z}_2$	• $\boldsymbol{E}$ $\parallel$ [001] and $\boldsymbol{B}$ $\parallel$ [001] and strain $\perp$ [001]
$P2/c$ (13.65) $\mathbb{Z}_2\times\mathbb{Z}_2$	• $\boldsymbol{B}$ $\parallel$ [001] and strain $\perp$ [001]
$Pc'c'2$ (27.81) $\mathbb{Z}_2$	• $\boldsymbol{E}$ $\parallel$ [001] and $\boldsymbol{B}$ $\parallel$ [001]
$Pc'c'a$ (54.342) $\mathbb{Z}_2\times\mathbb{Z}_2$	• $\boldsymbol{B}$ $\parallel$ [001]
$P21'$ (3.2) $\mathbb{Z}_2$	• $\boldsymbol{E}$ $\parallel$ [001] and strain $\perp$ [001]
$P2/c1'$ (13.66) $\mathbb{Z}_2\times\mathbb{Z}_2$	• strain $\perp$ [001]
$Pcc21'$ (27.79) $\mathbb{Z}_2$	• $\boldsymbol{E}$ $\parallel$ [001]

Table. S 449: Subgroups of $Pc'ca$ (54.339) that can be realized by applying $\boldsymbol{E}/\boldsymbol{B}$ fields and/or uniaxial strains.

Subgroup & SI group	Perturbation
Pc (7.24) $\mathbb{Z}_1$	• $\boldsymbol{B}$ $\parallel$ [010] and strain $\perp$ [010]<br>• $\boldsymbol{E}$ $\parallel$ [100] and strain $\perp$ [010]<br>• $\boldsymbol{E}$ $\parallel$ [100] and $\boldsymbol{B}$ $\parallel$ [010]<br>• $\boldsymbol{E}$ $\parallel$ [001] and strain $\perp$ [010]<br>• $\boldsymbol{E}$ $\perp$ [010]
Pc (7.24) $\mathbb{Z}_1$	• $\boldsymbol{B}$ $\parallel$ [001] and strain $\perp$ [001]<br>• $\boldsymbol{E}$ $\parallel$ [100] and strain $\perp$ [001]<br>• $\boldsymbol{E}$ $\parallel$ [100] and $\boldsymbol{B}$ $\parallel$ [001]<br>• $\boldsymbol{E}$ $\parallel$ [010] and strain $\perp$ [001]<br>• $\boldsymbol{E}$ $\perp$ [001]
$P2_1$ (4.7) $\mathbb{Z}_1$	• $\boldsymbol{B}$ $\parallel$ [100] and strain $\perp$ [100]<br>• $\boldsymbol{E}$ $\parallel$ [100] and strain $\perp$ [100]<br>• $\boldsymbol{E}$ $\parallel$ [100] and $\boldsymbol{B}$ $\parallel$ [100]

Continued on next page

…continued

Subgroup & SI group	Perturbation
$Pca2_1$ (29.99) $\mathbb{Z}_1$	• $\boldsymbol{E}$ ∥ [100]
$P2'$ (3.3) $\mathbb{Z}_1$	• $\boldsymbol{B}$ ∥ [100] and strain ⊥ [010]<br>• $\boldsymbol{B}$ ∥ [001] and strain ⊥ [010]<br>• $\boldsymbol{B}$ ⊥ [010]<br>• $\boldsymbol{E}$ ∥ [010] and strain ⊥ [010]<br>• $\boldsymbol{E}$ ∥ [010] and $\boldsymbol{B}$ ∥ [100]
$P\bar{1}'$ (2.6) $\mathbb{Z}_1$	• strain in generic direction
$P2'/c$ (13.67) $\mathbb{Z}_1$	• strain ⊥ [010]
Pc' (7.26) $\mathbb{Z}_1$	• $\boldsymbol{B}$ ∥ [010] and strain ⊥ [100]<br>• $\boldsymbol{B}$ ∥ [001] and strain ⊥ [100]<br>• $\boldsymbol{B}$ ⊥ [100]<br>• $\boldsymbol{E}$ ∥ [010] and strain ⊥ [100]<br>• $\boldsymbol{E}$ ∥ [010] and $\boldsymbol{B}$ ∥ [010]<br>• $\boldsymbol{E}$ ∥ [001] and strain ⊥ [100]<br>• $\boldsymbol{E}$ ∥ [001] and $\boldsymbol{B}$ ∥ [001]<br>• $\boldsymbol{E}$ ⊥ [100]
$Pb'a2'$ (32.137) $\mathbb{Z}_1$	• $\boldsymbol{B}$ ∥ [001]<br>• $\boldsymbol{E}$ ∥ [010]
$P2_1/c'$ (14.78) $\mathbb{Z}_1$	• strain ⊥ [100]
$P2'$ (3.3) $\mathbb{Z}_1$	• $\boldsymbol{B}$ ∥ [100] and strain ⊥ [001]<br>• $\boldsymbol{B}$ ∥ [010] and strain ⊥ [001]<br>• $\boldsymbol{B}$ ⊥ [001]<br>• $\boldsymbol{E}$ ∥ [001] and strain ⊥ [001]<br>• $\boldsymbol{E}$ ∥ [001] and $\boldsymbol{B}$ ∥ [100]
$P2'2'2_1$ (17.9) $\mathbb{Z}_1$	• $\boldsymbol{B}$ ∥ [100]
$P2'/c$ (13.67) $\mathbb{Z}_1$	• strain ⊥ [001]
$Pc'c2'$ (27.80) $\mathbb{Z}_1$	• $\boldsymbol{B}$ ∥ [010]<br>• $\boldsymbol{E}$ ∥ [001]

Table. S 450: Subgroups of $Pcc'a$ (54.340) that can be realized by applying $\boldsymbol{E}/\boldsymbol{B}$ fields and/or uniaxial strains.

Subgroup & SI group	Perturbation

Continued on next page

...continued

Subgroup & SI group	Perturbation
Pc' (7.26) $\mathbb{Z}_1$	• $\boldsymbol{B} \parallel [100]$ and strain $\perp [010]$<br>• $\boldsymbol{B} \parallel [001]$ and strain $\perp [010]$<br>• $\boldsymbol{B} \perp [010]$<br>• $\boldsymbol{E} \parallel [100]$ and strain $\perp [010]$<br>• $\boldsymbol{E} \parallel [100]$ and $\boldsymbol{B} \parallel [100]$<br>• $\boldsymbol{E} \parallel [001]$ and strain $\perp [010]$<br>• $\boldsymbol{E} \parallel [001]$ and $\boldsymbol{B} \parallel [001]$<br>• $\boldsymbol{E} \perp [010]$
Pc (7.24) $\mathbb{Z}_1$	• $\boldsymbol{B} \parallel [001]$ and strain $\perp [001]$<br>• $\boldsymbol{E} \parallel [100]$ and strain $\perp [001]$<br>• $\boldsymbol{E} \parallel [010]$ and strain $\perp [001]$<br>• $\boldsymbol{E} \parallel [010]$ and $\boldsymbol{B} \parallel [001]$<br>• $\boldsymbol{E} \perp [001]$
$P2_1'$ (4.9) $\mathbb{Z}_1$	• $\boldsymbol{B} \parallel [010]$ and strain $\perp [100]$<br>• $\boldsymbol{B} \parallel [001]$ and strain $\perp [100]$<br>• $\boldsymbol{B} \perp [100]$<br>• $\boldsymbol{E} \parallel [100]$ and strain $\perp [100]$<br>• $\boldsymbol{E} \parallel [100]$ and $\boldsymbol{B} \parallel [010]$
$Pca'2_1'$ (29.102) $\mathbb{Z}_1$	• $\boldsymbol{B} \parallel [001]$<br>• $\boldsymbol{E} \parallel [100]$
$P2$ (3.1) $\mathbb{Z}_2$	• $\boldsymbol{B} \parallel [010]$ and strain $\perp [010]$<br>• $\boldsymbol{E} \parallel [010]$ and strain $\perp [010]$<br>• $\boldsymbol{E} \parallel [010]$ and $\boldsymbol{B} \parallel [010]$
$P\bar{1}'$ (2.6) $\mathbb{Z}_1$	• strain in generic direction
$P2/c'$ (13.68) $\mathbb{Z}_2$	• strain $\perp [010]$
Pc (7.24) $\mathbb{Z}_1$	• $\boldsymbol{B} \parallel [100]$ and strain $\perp [100]$<br>• $\boldsymbol{E} \parallel [010]$ and strain $\perp [100]$<br>• $\boldsymbol{E} \parallel [010]$ and $\boldsymbol{B} \parallel [100]$<br>• $\boldsymbol{E} \parallel [001]$ and strain $\perp [100]$<br>• $\boldsymbol{E} \perp [100]$
$Pba2$ (32.135) $\mathbb{Z}_1$	• $\boldsymbol{E} \parallel [010]$
$P2_1'/c$ (14.77) $\mathbb{Z}_1$	• strain $\perp [100]$

Continued on next page

…continued

Subgroup & SI group	Perturbation
$P2'$ (3.3) $\mathbb{Z}_1$	• $\boldsymbol{B}$ ∥ [100] and strain ⊥ [001]<br>• $\boldsymbol{B}$ ∥ [010] and strain ⊥ [001]<br>• $\boldsymbol{B}$ ⊥ [001]<br>• $\boldsymbol{E}$ ∥ [001] and strain ⊥ [001]<br>• $\boldsymbol{E}$ ∥ [001] and $\boldsymbol{B}$ ∥ [010]
$P22'2'_1$ (17.10) $\mathbb{Z}_2$	• $\boldsymbol{B}$ ∥ [010]
$P2'/c$ (13.67) $\mathbb{Z}_1$	• strain ⊥ [001]
$Pc'c2'$ (27.80) $\mathbb{Z}_1$	• $\boldsymbol{B}$ ∥ [100]<br>• $\boldsymbol{E}$ ∥ [001]

Table. S 451: Subgroups of $Pcca'$ (54.341) that can be realized by applying $\boldsymbol{E}/\boldsymbol{B}$ fields and/or uniaxial strains.

Subgroup & SI group	Perturbation
Pc (7.24) $\mathbb{Z}_1$	• $\boldsymbol{B}$ ∥ [010] and strain ⊥ [010]<br>• $\boldsymbol{E}$ ∥ [100] and strain ⊥ [010]<br>• $\boldsymbol{E}$ ∥ [001] and strain ⊥ [010]<br>• $\boldsymbol{E}$ ∥ [001] and $\boldsymbol{B}$ ∥ [010]<br>• $\boldsymbol{E}$ ⊥ [010]
Pc' (7.26) $\mathbb{Z}_1$	• $\boldsymbol{B}$ ∥ [100] and strain ⊥ [001]<br>• $\boldsymbol{B}$ ∥ [010] and strain ⊥ [001]<br>• $\boldsymbol{B}$ ⊥ [001]<br>• $\boldsymbol{E}$ ∥ [100] and strain ⊥ [001]<br>• $\boldsymbol{E}$ ∥ [100] and $\boldsymbol{B}$ ∥ [100]<br>• $\boldsymbol{E}$ ∥ [010] and strain ⊥ [001]<br>• $\boldsymbol{E}$ ∥ [010] and $\boldsymbol{B}$ ∥ [010]<br>• $\boldsymbol{E}$ ⊥ [001]
$P2'_1$ (4.9) $\mathbb{Z}_1$	• $\boldsymbol{B}$ ∥ [010] and strain ⊥ [100]<br>• $\boldsymbol{B}$ ∥ [001] and strain ⊥ [100]<br>• $\boldsymbol{B}$ ⊥ [100]<br>• $\boldsymbol{E}$ ∥ [100] and strain ⊥ [100]<br>• $\boldsymbol{E}$ ∥ [100] and $\boldsymbol{B}$ ∥ [001]
$Pc'a2'_1$ (29.101) $\mathbb{Z}_1$	• $\boldsymbol{B}$ ∥ [010]<br>• $\boldsymbol{E}$ ∥ [100]

Continued on next page

...continued

Subgroup & SI group	Perturbation
$P2'$ (3.3) $\mathbb{Z}_1$	• $\boldsymbol{B}$ ∥ [100] and strain ⊥ [010]<br>• $\boldsymbol{B}$ ∥ [001] and strain ⊥ [010]<br>• $\boldsymbol{B}$ ⊥ [010]<br>• $\boldsymbol{E}$ ∥ [010] and strain ⊥ [010]<br>• $\boldsymbol{E}$ ∥ [010] and $\boldsymbol{B}$ ∥ [001]
$P\bar{1}'$ (2.6) $\mathbb{Z}_1$	• strain in generic direction
$P2'/c$ (13.67) $\mathbb{Z}_1$	• strain ⊥ [010]
Pc (7.24) $\mathbb{Z}_1$	• $\boldsymbol{B}$ ∥ [100] and strain ⊥ [100]<br>• $\boldsymbol{E}$ ∥ [010] and strain ⊥ [100]<br>• $\boldsymbol{E}$ ∥ [001] and strain ⊥ [100]<br>• $\boldsymbol{E}$ ∥ [001] and $\boldsymbol{B}$ ∥ [100]<br>• $\boldsymbol{E}$ ⊥ [100]
$Pb'a2'$ (32.137) $\mathbb{Z}_1$	• $\boldsymbol{B}$ ∥ [100]<br>• $\boldsymbol{E}$ ∥ [010]
$P2_1'/c$ (14.77) $\mathbb{Z}_1$	• strain ⊥ [100]
$P2$ (3.1) $\mathbb{Z}_2$	• $\boldsymbol{B}$ ∥ [001] and strain ⊥ [001]<br>• $\boldsymbol{E}$ ∥ [001] and strain ⊥ [001]<br>• $\boldsymbol{E}$ ∥ [001] and $\boldsymbol{B}$ ∥ [001]
$P22'2_1'$ (17.10) $\mathbb{Z}_2$	• $\boldsymbol{B}$ ∥ [001]
$P2/c'$ (13.68) $\mathbb{Z}_2$	• strain ⊥ [001]
$Pcc2$ (27.78) $\mathbb{Z}_2$	• $\boldsymbol{E}$ ∥ [001]

Table. S 452: Subgroups of $Pc'c'a$ (54.342) that can be realized by applying $\boldsymbol{E}/\boldsymbol{B}$ fields and/or uniaxial strains.

Subgroup & SI group	Perturbation
Pc' (7.26) $\mathbb{Z}_1$	• $\boldsymbol{E}$ ∥ [100] and strain ⊥ [010]<br>• $\boldsymbol{E}$ ∥ [100] and ($\boldsymbol{B}$ ∥ [100] or $\boldsymbol{B}$ ⊥ [010])<br>• $\boldsymbol{E}$ ∥ [001] and strain ⊥ [010]<br>• $\boldsymbol{E}$ ∥ [001] and ($\boldsymbol{B}$ ∥ [100] or $\boldsymbol{B}$ ⊥ [010])<br>• $\boldsymbol{E}$ ⊥ [010]

Continued on next page

…continued

Subgroup & SI group	Perturbation
Pc (7.24) $\mathbb{Z}_1$	• $\boldsymbol{E}$ ∥ [100] and strain ⊥ [001]<br>• $\boldsymbol{E}$ ∥ [010] and strain ⊥ [001]<br>• $\boldsymbol{E}$ ⊥ [001]
$P2_1'$ (4.9) $\mathbb{Z}_1$	• $\boldsymbol{E}$ ∥ [100] and strain ⊥ [100]<br>• $\boldsymbol{E}$ ∥ [100] and ($\boldsymbol{B}$ ∥ [010] or $\boldsymbol{B}$ ⊥ [100])
$Pca'2_1'$ (29.102) $\mathbb{Z}_1$	• $\boldsymbol{E}$ ∥ [100]
$P2'$ (3.3) $\mathbb{Z}_1$	• $\boldsymbol{E}$ ∥ [010] and strain ⊥ [010]<br>• $\boldsymbol{E}$ ∥ [010] and ($\boldsymbol{B}$ ∥ [100] or $\boldsymbol{B}$ ⊥ [010])
$P\bar{1}$ (2.4) $\mathbb{Z}_2\times\mathbb{Z}_2\times\mathbb{Z}_2\times\mathbb{Z}_4$	• strain in generic direction<br>• ($\boldsymbol{B}$ ∥ [100] or $\boldsymbol{B}$ ⊥ [010]) and strain ⊥ [100]<br>• ($\boldsymbol{B}$ ∥ [100] or $\boldsymbol{B}$ ⊥ [010]) and strain ⊥ [001]<br>• ($\boldsymbol{B}$ ∥ [010] or $\boldsymbol{B}$ ⊥ [100]) and strain ⊥ [010]<br>• ($\boldsymbol{B}$ ∥ [010] or $\boldsymbol{B}$ ⊥ [100]) and strain ⊥ [001]<br>• $\boldsymbol{B}$ in generic direction
$P2'/c'$ (13.69) $\mathbb{Z}_2\times\mathbb{Z}_2\times\mathbb{Z}_4$	• strain ⊥ [010]<br>• ($\boldsymbol{B}$ ∥ [100] or $\boldsymbol{B}$ ⊥ [010])
Pc' (7.26) $\mathbb{Z}_1$	• $\boldsymbol{E}$ ∥ [010] and strain ⊥ [100]<br>• $\boldsymbol{E}$ ∥ [010] and ($\boldsymbol{B}$ ∥ [010] or $\boldsymbol{B}$ ⊥ [100])<br>• $\boldsymbol{E}$ ∥ [001] and strain ⊥ [100]<br>• $\boldsymbol{E}$ ∥ [001] and ($\boldsymbol{B}$ ∥ [010] or $\boldsymbol{B}$ ⊥ [100])<br>• $\boldsymbol{E}$ ⊥ [100]
$Pb'a2'$ (32.137) $\mathbb{Z}_1$	• $\boldsymbol{E}$ ∥ [010]
$P2_1'/c'$ (14.79) $\mathbb{Z}_2\times\mathbb{Z}_4$	• strain ⊥ [100]<br>• ($\boldsymbol{B}$ ∥ [010] or $\boldsymbol{B}$ ⊥ [100])
$P2$ (3.1) $\mathbb{Z}_2$	• $\boldsymbol{E}$ ∥ [001] and strain ⊥ [001]
$P2/c$ (13.65) $\mathbb{Z}_2\times\mathbb{Z}_2$	• strain ⊥ [001]
$Pc'c'2$ (27.81) $\mathbb{Z}_2$	• $\boldsymbol{E}$ ∥ [001]

Table. S 453: Subgroups of $Pcc'a'$ (54.343) that can be realized by applying $\boldsymbol{E}/\boldsymbol{B}$ fields and/or uniaxial strains.

Subgroup & SI group	Perturbation

Continued on next page

…continued

Subgroup & SI group	Perturbation
Pc' (7.26) $\mathbb{Z}_1$	• $\boldsymbol{E} \parallel [100]$ and strain $\perp [010]$<br>• $\boldsymbol{E} \parallel [100]$ and ($\boldsymbol{B} \parallel [001]$ or $\boldsymbol{B} \perp [010]$)<br>• $\boldsymbol{E} \parallel [001]$ and strain $\perp [010]$<br>• $\boldsymbol{E} \parallel [001]$ and ($\boldsymbol{B} \parallel [001]$ or $\boldsymbol{B} \perp [010]$)<br>• $\boldsymbol{E} \perp [010]$
Pc' (7.26) $\mathbb{Z}_1$	• $\boldsymbol{E} \parallel [100]$ and strain $\perp [001]$<br>• $\boldsymbol{E} \parallel [100]$ and ($\boldsymbol{B} \parallel [010]$ or $\boldsymbol{B} \perp [001]$)<br>• $\boldsymbol{E} \parallel [010]$ and strain $\perp [001]$<br>• $\boldsymbol{E} \parallel [010]$ and ($\boldsymbol{B} \parallel [010]$ or $\boldsymbol{B} \perp [001]$)<br>• $\boldsymbol{E} \perp [001]$
$P2_1$ (4.7) $\mathbb{Z}_1$	• $\boldsymbol{E} \parallel [100]$ and strain $\perp [100]$
$Pc'a'2_1$ (29.103) $\mathbb{Z}_1$	• $\boldsymbol{E} \parallel [100]$
$P2'$ (3.3) $\mathbb{Z}_1$	• $\boldsymbol{E} \parallel [010]$ and strain $\perp [010]$<br>• $\boldsymbol{E} \parallel [010]$ and ($\boldsymbol{B} \parallel [001]$ or $\boldsymbol{B} \perp [010]$)
$P\bar{1}$ (2.4) $\mathbb{Z}_2 \times \mathbb{Z}_2 \times \mathbb{Z}_2 \times \mathbb{Z}_4$	• strain in generic direction<br>• ($\boldsymbol{B} \parallel [010]$ or $\boldsymbol{B} \perp [001]$) and strain $\perp [100]$<br>• ($\boldsymbol{B} \parallel [010]$ or $\boldsymbol{B} \perp [001]$) and strain $\perp [010]$<br>• ($\boldsymbol{B} \parallel [001]$ or $\boldsymbol{B} \perp [010]$) and strain $\perp [100]$<br>• ($\boldsymbol{B} \parallel [001]$ or $\boldsymbol{B} \perp [010]$) and strain $\perp [001]$<br>• $\boldsymbol{B}$ in generic direction
$P2'/c'$ (13.69) $\mathbb{Z}_2 \times \mathbb{Z}_2 \times \mathbb{Z}_4$	• strain $\perp [010]$<br>• ($\boldsymbol{B} \parallel [001]$ or $\boldsymbol{B} \perp [010]$)
Pc (7.24) $\mathbb{Z}_1$	• $\boldsymbol{E} \parallel [010]$ and strain $\perp [100]$<br>• $\boldsymbol{E} \parallel [001]$ and strain $\perp [100]$<br>• $\boldsymbol{E} \perp [100]$
$Pb'a2'$ (32.137) $\mathbb{Z}_1$	• $\boldsymbol{E} \parallel [010]$
$P2_1/c$ (14.75) $\mathbb{Z}_2$	• strain $\perp [100]$
$P2'$ (3.3) $\mathbb{Z}_1$	• $\boldsymbol{E} \parallel [001]$ and strain $\perp [001]$<br>• $\boldsymbol{E} \parallel [001]$ and ($\boldsymbol{B} \parallel [010]$ or $\boldsymbol{B} \perp [001]$)
$P2'/c'$ (13.69) $\mathbb{Z}_2 \times \mathbb{Z}_2 \times \mathbb{Z}_4$	• strain $\perp [001]$<br>• ($\boldsymbol{B} \parallel [010]$ or $\boldsymbol{B} \perp [001]$)
$Pc'c2'$ (27.80) $\mathbb{Z}_1$	• $\boldsymbol{E} \parallel [001]$

Table. S 454: Subgroups of $Pc'ca'$ (54.344) that can be realized by applying $\boldsymbol{E}/\boldsymbol{B}$ fields and/or uniaxial strains.

Subgroup & SI group	Perturbation
Pc (7.24) $\mathbb{Z}_1$	• $\boldsymbol{E} \parallel [100]$ and strain $\perp [010]$<br>• $\boldsymbol{E} \parallel [001]$ and strain $\perp [010]$<br>• $\boldsymbol{E} \perp [010]$
Pc' (7.26) $\mathbb{Z}_1$	• $\boldsymbol{E} \parallel [100]$ and strain $\perp [001]$<br>• $\boldsymbol{E} \parallel [100]$ and ($\boldsymbol{B} \parallel [100]$ or $\boldsymbol{B} \perp [001]$)<br>• $\boldsymbol{E} \parallel [010]$ and strain $\perp [001]$<br>• $\boldsymbol{E} \parallel [010]$ and ($\boldsymbol{B} \parallel [100]$ or $\boldsymbol{B} \perp [001]$)<br>• $\boldsymbol{E} \perp [001]$
$P2_1'$ (4.9) $\mathbb{Z}_1$	• $\boldsymbol{E} \parallel [100]$ and strain $\perp [100]$<br>• $\boldsymbol{E} \parallel [100]$ and ($\boldsymbol{B} \parallel [001]$ or $\boldsymbol{B} \perp [100]$)
$Pc'a2_1'$ (29.101) $\mathbb{Z}_1$	• $\boldsymbol{E} \parallel [100]$
$P2$ (3.1) $\mathbb{Z}_2$	• $\boldsymbol{E} \parallel [010]$ and strain $\perp [010]$
$P\bar{1}$ (2.4) $\mathbb{Z}_2 \times \mathbb{Z}_2 \times \mathbb{Z}_2 \times \mathbb{Z}_4$	• strain in generic direction<br>• ($\boldsymbol{B} \parallel [100]$ or $\boldsymbol{B} \perp [001]$) and strain $\perp [100]$<br>• ($\boldsymbol{B} \parallel [100]$ or $\boldsymbol{B} \perp [001]$) and strain $\perp [010]$<br>• ($\boldsymbol{B} \parallel [001]$ or $\boldsymbol{B} \perp [100]$) and strain $\perp [010]$<br>• ($\boldsymbol{B} \parallel [001]$ or $\boldsymbol{B} \perp [100]$) and strain $\perp [001]$<br>• $\boldsymbol{B}$ in generic direction
$P2/c$ (13.65) $\mathbb{Z}_2 \times \mathbb{Z}_2$	• strain $\perp [010]$
Pc' (7.26) $\mathbb{Z}_1$	• $\boldsymbol{E} \parallel [010]$ and strain $\perp [100]$<br>• $\boldsymbol{E} \parallel [010]$ and ($\boldsymbol{B} \parallel [001]$ or $\boldsymbol{B} \perp [100]$)<br>• $\boldsymbol{E} \parallel [001]$ and strain $\perp [100]$<br>• $\boldsymbol{E} \parallel [001]$ and ($\boldsymbol{B} \parallel [001]$ or $\boldsymbol{B} \perp [100]$)<br>• $\boldsymbol{E} \perp [100]$
$Pb'a'2$ (32.138) $\mathbb{Z}_2$	• $\boldsymbol{E} \parallel [010]$
$P2_1'/c'$ (14.79) $\mathbb{Z}_2 \times \mathbb{Z}_4$	• strain $\perp [100]$<br>• ($\boldsymbol{B} \parallel [001]$ or $\boldsymbol{B} \perp [100]$)
$P2'$ (3.3) $\mathbb{Z}_1$	• $\boldsymbol{E} \parallel [001]$ and strain $\perp [001]$<br>• $\boldsymbol{E} \parallel [001]$ and ($\boldsymbol{B} \parallel [100]$ or $\boldsymbol{B} \perp [001]$)
$P2'/c'$ (13.69) $\mathbb{Z}_2 \times \mathbb{Z}_2 \times \mathbb{Z}_4$	• strain $\perp [001]$<br>• ($\boldsymbol{B} \parallel [100]$ or $\boldsymbol{B} \perp [001]$)
$Pc'c2'$ (27.80) $\mathbb{Z}_1$	• $\boldsymbol{E} \parallel [001]$

Table. S 455: Subgroups of $Pc'c'a'$ (54.345) that can be realized by applying $\boldsymbol{E}/\boldsymbol{B}$ fields and/or uniaxial strains.

Subgroup & SI group	Perturbation
Pc' (7.26) $\mathbb{Z}_1$	• $\boldsymbol{B} \parallel [100]$ and strain $\perp [010]$<br>• $\boldsymbol{B} \parallel [001]$ and strain $\perp [010]$<br>• $\boldsymbol{B} \perp [010]$<br>• $\boldsymbol{E} \parallel [100]$ and strain $\perp [010]$<br>• $\boldsymbol{E} \parallel [100]$ and $\boldsymbol{B} \parallel [001]$<br>• $\boldsymbol{E} \parallel [001]$ and strain $\perp [010]$<br>• $\boldsymbol{E} \parallel [001]$ and $\boldsymbol{B} \parallel [100]$<br>• $\boldsymbol{E} \perp [010]$
Pc' (7.26) $\mathbb{Z}_1$	• $\boldsymbol{B} \parallel [100]$ and strain $\perp [001]$<br>• $\boldsymbol{B} \parallel [010]$ and strain $\perp [001]$<br>• $\boldsymbol{B} \perp [001]$<br>• $\boldsymbol{E} \parallel [100]$ and strain $\perp [001]$<br>• $\boldsymbol{E} \parallel [100]$ and $\boldsymbol{B} \parallel [010]$<br>• $\boldsymbol{E} \parallel [010]$ and strain $\perp [001]$<br>• $\boldsymbol{E} \parallel [010]$ and $\boldsymbol{B} \parallel [100]$<br>• $\boldsymbol{E} \perp [001]$
$P2_1$ (4.7) $\mathbb{Z}_1$	• $\boldsymbol{B} \parallel [100]$ and strain $\perp [100]$<br>• $\boldsymbol{E} \parallel [100]$ and strain $\perp [100]$
$Pc'a'2_1$ (29.103) $\mathbb{Z}_1$	• $\boldsymbol{B} \parallel [100]$<br>• $\boldsymbol{E} \parallel [100]$
$P2$ (3.1) $\mathbb{Z}_2$	• $\boldsymbol{B} \parallel [010]$ and strain $\perp [010]$<br>• $\boldsymbol{E} \parallel [010]$ and strain $\perp [010]$
$P\bar{1}'$ (2.6) $\mathbb{Z}_1$	• strain in generic direction
$P2/c'$ (13.68) $\mathbb{Z}_2$	• strain $\perp [010]$
Pc' (7.26) $\mathbb{Z}_1$	• $\boldsymbol{B} \parallel [010]$ and strain $\perp [100]$<br>• $\boldsymbol{B} \parallel [001]$ and strain $\perp [100]$<br>• $\boldsymbol{B} \perp [100]$<br>• $\boldsymbol{E} \parallel [010]$ and strain $\perp [100]$<br>• $\boldsymbol{E} \parallel [010]$ and $\boldsymbol{B} \parallel [001]$<br>• $\boldsymbol{E} \parallel [001]$ and strain $\perp [100]$<br>• $\boldsymbol{E} \parallel [001]$ and $\boldsymbol{B} \parallel [010]$<br>• $\boldsymbol{E} \perp [100]$
$Pb'a'2$ (32.138) $\mathbb{Z}_2$	• $\boldsymbol{B} \parallel [010]$<br>• $\boldsymbol{E} \parallel [010]$
$P2_1/c'$ (14.78) $\mathbb{Z}_1$	• strain $\perp [100]$

Continued on next page

…continued

Subgroup & SI group	Perturbation
$P2$ (3.1) $\mathbb{Z}_2$	• $\boldsymbol{B}$ ∥ [001] and strain ⊥ [001]<br>• $\boldsymbol{E}$ ∥ [001] and strain ⊥ [001]
$P2/c'$ (13.68) $\mathbb{Z}_2$	• strain ⊥ [001]
$Pc'c'2$ (27.81) $\mathbb{Z}_2$	• $\boldsymbol{B}$ ∥ [001]<br>• $\boldsymbol{E}$ ∥ [001]

Table. S 456: Subgroups of P_acca (54.346) that can be realized by applying $\boldsymbol{E}/\boldsymbol{B}$ fields and/or uniaxial strains.

Subgroup & SI group	Perturbation
Pm' (6.20) $\mathbb{Z}_1$	• $\boldsymbol{E}$ ∥ [100] and $\boldsymbol{B}$ ∥ [100] and strain ⊥ [001]<br>• $\boldsymbol{E}$ ∥ [100] and $\boldsymbol{B}$ ∥ [010] and strain ⊥ [001]<br>• $\boldsymbol{E}$ ∥ [100] and $\boldsymbol{B}$ ⊥ [001]<br>• $\boldsymbol{E}$ ∥ [010] and $\boldsymbol{B}$ ∥ [100] and strain ⊥ [001]<br>• $\boldsymbol{E}$ ∥ [010] and $\boldsymbol{B}$ ∥ [010] and strain ⊥ [001]<br>• $\boldsymbol{E}$ ∥ [010] and $\boldsymbol{B}$ ⊥ [001]<br>• $\boldsymbol{E}$ ⊥ [001] and $\boldsymbol{B}$ ∥ [100]<br>• $\boldsymbol{E}$ ⊥ [001] and $\boldsymbol{B}$ ∥ [010]<br>• $\boldsymbol{E}$ ⊥ [001] and $\boldsymbol{B}$ ⊥ [001]
Pc (7.24) $\mathbb{Z}_1$	• $\boldsymbol{E}$ ∥ [100] and $\boldsymbol{B}$ ∥ [010] and strain ⊥ [010]<br>• $\boldsymbol{E}$ ∥ [001] and $\boldsymbol{B}$ ∥ [010] and strain ⊥ [010]<br>• $\boldsymbol{E}$ ⊥ [010] and $\boldsymbol{B}$ ∥ [010]
$P2'$ (3.3) $\mathbb{Z}_1$	• $\boldsymbol{E}$ ∥ [100] and $\boldsymbol{B}$ ∥ [010] and strain ⊥ [100]<br>• $\boldsymbol{E}$ ∥ [100] and $\boldsymbol{B}$ ∥ [001] and strain ⊥ [100]<br>• $\boldsymbol{E}$ ∥ [100] and $\boldsymbol{B}$ ⊥ [100]
$Pm'a2'$ (28.89) $\mathbb{Z}_1$	• $\boldsymbol{E}$ ∥ [100] and $\boldsymbol{B}$ ∥ [010]

Continued on next page

…continued

Subgroup & SI group	Perturbation
P_S1 (1.3) $\mathbb{Z}_1$	• $\boldsymbol{E} \parallel [100]$ and strain in generic direction<br>• $\boldsymbol{E} \parallel [010]$ and strain in generic direction<br>• $\boldsymbol{E} \parallel [001]$ and strain in generic direction<br>• $\boldsymbol{E} \perp [100]$ and strain $\perp [010]$<br>• $\boldsymbol{E} \perp [100]$ and strain $\perp [001]$<br>• $\boldsymbol{E} \perp [100]$ and strain in generic direction<br>• $\boldsymbol{E} \perp [010]$ and strain $\perp [100]$<br>• $\boldsymbol{E} \perp [010]$ and strain $\perp [001]$<br>• $\boldsymbol{E} \perp [010]$ and strain in generic direction<br>• $\boldsymbol{E} \perp [001]$ and strain $\perp [100]$<br>• $\boldsymbol{E} \perp [001]$ and strain $\perp [010]$<br>• $\boldsymbol{E} \perp [001]$ and strain in generic direction<br>• $\boldsymbol{E}$ in generic direction
Pc (7.24) $\mathbb{Z}_1$	• $\boldsymbol{E} \parallel [100]$ and $\boldsymbol{B} \parallel [001]$ and strain $\perp [001]$<br>• $\boldsymbol{E} \parallel [010]$ and $\boldsymbol{B} \parallel [001]$ and strain $\perp [001]$<br>• $\boldsymbol{E} \perp [001]$ and $\boldsymbol{B} \parallel [001]$
P_cc (7.28) $\mathbb{Z}_1$	• $\boldsymbol{E} \parallel [100]$ and strain $\perp [001]$<br>• $\boldsymbol{E} \parallel [010]$ and strain $\perp [001]$<br>• $\boldsymbol{E} \perp [001]$
Pc' (7.26) $\mathbb{Z}_1$	• $\boldsymbol{E} \parallel [100]$ and $\boldsymbol{B} \parallel [100]$ and strain $\perp [010]$<br>• $\boldsymbol{E} \parallel [100]$ and $\boldsymbol{B} \parallel [001]$ and strain $\perp [010]$<br>• $\boldsymbol{E} \parallel [100]$ and $\boldsymbol{B} \perp [010]$<br>• $\boldsymbol{E} \parallel [001]$ and $\boldsymbol{B} \parallel [100]$ and strain $\perp [010]$<br>• $\boldsymbol{E} \parallel [001]$ and $\boldsymbol{B} \parallel [001]$ and strain $\perp [010]$<br>• $\boldsymbol{E} \parallel [001]$ and $\boldsymbol{B} \perp [010]$<br>• $\boldsymbol{E} \perp [010]$ and $\boldsymbol{B} \parallel [100]$<br>• $\boldsymbol{E} \perp [010]$ and $\boldsymbol{B} \parallel [001]$<br>• $\boldsymbol{E} \perp [010]$ and $\boldsymbol{B} \perp [010]$
P_ac (7.27) $\mathbb{Z}_1$	• $\boldsymbol{E} \parallel [100]$ and strain $\perp [010]$<br>• $\boldsymbol{E} \parallel [001]$ and strain $\perp [010]$<br>• $\boldsymbol{E} \perp [010]$
$Pn'c2'$ (30.113) $\mathbb{Z}_1$	• $\boldsymbol{E} \parallel [100]$ and $\boldsymbol{B} \parallel [001]$
$P2_1$ (4.7) $\mathbb{Z}_1$	• $\boldsymbol{E} \parallel [100]$ and $\boldsymbol{B} \parallel [100]$ and strain $\perp [100]$
P_b2_1 (4.11) $\mathbb{Z}_1$	• $\boldsymbol{E} \parallel [100]$ and strain $\perp [100]$
$Pm'n'2_1$ (31.127) $\mathbb{Z}_1$	• $\boldsymbol{E} \parallel [100]$ and $\boldsymbol{B} \parallel [100]$
P_cca2_1 (29.106) $\mathbb{Z}_1$	• $\boldsymbol{E} \parallel [100]$

Continued on next page

. . . continued

Subgroup & SI group	Perturbation
Pc' (7.26) $\mathbb{Z}_1$	• $\boldsymbol{E}$ ∥ [010] and $\boldsymbol{B}$ ∥ [010] and strain ⊥ [100]<br>• $\boldsymbol{E}$ ∥ [010] and $\boldsymbol{B}$ ∥ [001] and strain ⊥ [100]<br>• $\boldsymbol{E}$ ∥ [010] and $\boldsymbol{B}$ ⊥ [100]<br>• $\boldsymbol{E}$ ∥ [001] and $\boldsymbol{B}$ ∥ [010] and strain ⊥ [100]<br>• $\boldsymbol{E}$ ∥ [001] and $\boldsymbol{B}$ ∥ [001] and strain ⊥ [100]<br>• $\boldsymbol{E}$ ∥ [001] and $\boldsymbol{B}$ ⊥ [100]<br>• $\boldsymbol{E}$ ⊥ [100] and $\boldsymbol{B}$ ∥ [010]<br>• $\boldsymbol{E}$ ⊥ [100] and $\boldsymbol{B}$ ∥ [001]<br>• $\boldsymbol{E}$ ⊥ [100] and $\boldsymbol{B}$ ⊥ [100]
$P2$ (3.1) $\mathbb{Z}_2$	• $\boldsymbol{E}$ ∥ [010] and $\boldsymbol{B}$ ∥ [010] and strain ⊥ [010]
$Pm'a'2$ (28.91) $\mathbb{Z}_2$	• $\boldsymbol{E}$ ∥ [010] and $\boldsymbol{B}$ ∥ [010]
$P2'$ (3.3) $\mathbb{Z}_1$	• $\boldsymbol{E}$ ∥ [001] and $\boldsymbol{B}$ ∥ [100] and strain ⊥ [001]<br>• $\boldsymbol{E}$ ∥ [001] and $\boldsymbol{B}$ ∥ [010] and strain ⊥ [001]<br>• $\boldsymbol{E}$ ∥ [001] and $\boldsymbol{B}$ ⊥ [001]
$Pc'c2'$ (27.80) $\mathbb{Z}_1$	• $\boldsymbol{E}$ ∥ [001] and $\boldsymbol{B}$ ∥ [010]
$P\bar{1}$ (2.4) $\mathbb{Z}_2 \times \mathbb{Z}_2 \times \mathbb{Z}_2 \times \mathbb{Z}_4$	• $\boldsymbol{B}$ ∥ [100] and strain in generic direction<br>• $\boldsymbol{B}$ ∥ [010] and strain in generic direction<br>• $\boldsymbol{B}$ ∥ [001] and strain in generic direction<br>• $\boldsymbol{B}$ ⊥ [100] and strain ⊥ [010]<br>• $\boldsymbol{B}$ ⊥ [100] and strain ⊥ [001]<br>• $\boldsymbol{B}$ ⊥ [100] and strain in generic direction<br>• $\boldsymbol{B}$ ⊥ [010] and strain ⊥ [100]<br>• $\boldsymbol{B}$ ⊥ [010] and strain ⊥ [001]<br>• $\boldsymbol{B}$ ⊥ [010] and strain in generic direction<br>• $\boldsymbol{B}$ ⊥ [001] and strain ⊥ [100]<br>• $\boldsymbol{B}$ ⊥ [001] and strain ⊥ [010]<br>• $\boldsymbol{B}$ ⊥ [001] and strain in generic direction<br>• $\boldsymbol{B}$ in generic direction
$P2'/c'$ (13.69) $\mathbb{Z}_2 \times \mathbb{Z}_2 \times \mathbb{Z}_4$	• $\boldsymbol{B}$ ∥ [010] and strain ⊥ [100]<br>• $\boldsymbol{B}$ ∥ [001] and strain ⊥ [100]<br>• $\boldsymbol{B}$ ⊥ [100]
$P2/c$ (13.65) $\mathbb{Z}_2 \times \mathbb{Z}_2$	• $\boldsymbol{B}$ ∥ [010] and strain ⊥ [010]
$P2'/m'$ (10.46) $\mathbb{Z}_2 \times \mathbb{Z}_2 \times \mathbb{Z}_2 \times \mathbb{Z}_4$	• $\boldsymbol{B}$ ∥ [100] and strain ⊥ [001]<br>• $\boldsymbol{B}$ ∥ [010] and strain ⊥ [001]<br>• $\boldsymbol{B}$ ⊥ [001]
$Pc'cm'$ (49.270) $\mathbb{Z}_2 \times \mathbb{Z}_2$	• $\boldsymbol{B}$ ∥ [010]

Continued on next page

...continued

Subgroup & SI group	Perturbation
Pc (7.24) $\mathbb{Z}_1$	• $\boldsymbol{E} \parallel [010]$ and $\boldsymbol{B} \parallel [100]$ and strain $\perp [100]$<br>• $\boldsymbol{E} \parallel [001]$ and $\boldsymbol{B} \parallel [100]$ and strain $\perp [100]$<br>• $\boldsymbol{E} \perp [100]$ and $\boldsymbol{B} \parallel [100]$
P_bc (7.29) $\mathbb{Z}_1$	• $\boldsymbol{E} \parallel [010]$ and strain $\perp [100]$<br>• $\boldsymbol{E} \parallel [001]$ and strain $\perp [100]$<br>• $\boldsymbol{E} \perp [100]$
$Pn'c2'$ (30.113) $\mathbb{Z}_1$	• $\boldsymbol{E} \parallel [001]$ and $\boldsymbol{B} \parallel [100]$
$P2_1/c$ (14.75) $\mathbb{Z}_2$	• $\boldsymbol{B} \parallel [100]$ and strain $\perp [100]$
$P2'$ (3.3) $\mathbb{Z}_1$	• $\boldsymbol{E} \parallel [010]$ and $\boldsymbol{B} \parallel [100]$ and strain $\perp [010]$<br>• $\boldsymbol{E} \parallel [010]$ and $\boldsymbol{B} \parallel [001]$ and strain $\perp [010]$<br>• $\boldsymbol{E} \parallel [010]$ and $\boldsymbol{B} \perp [010]$
$Pb'a2'$ (32.137) $\mathbb{Z}_1$	• $\boldsymbol{E} \parallel [010]$ and $\boldsymbol{B} \parallel [001]$
P_a2 (3.4) $\mathbb{Z}_2$	• $\boldsymbol{E} \parallel [010]$ and strain $\perp [010]$
$P2'/c'$ (13.69) $\mathbb{Z}_2 \times \mathbb{Z}_2 \times \mathbb{Z}_4$	• $\boldsymbol{B} \parallel [100]$ and strain $\perp [010]$<br>• $\boldsymbol{B} \parallel [001]$ and strain $\perp [010]$<br>• $\boldsymbol{B} \perp [010]$
$Pm'a2'$ (28.89) $\mathbb{Z}_1$	• $\boldsymbol{E} \parallel [010]$ and $\boldsymbol{B} \parallel [100]$
P_bba2 (32.140) $\mathbb{Z}_1$	• $\boldsymbol{E} \parallel [010]$
$Pm'n'a$ (53.326) $\mathbb{Z}_2$	• $\boldsymbol{B} \parallel [100]$
$P2$ (3.1) $\mathbb{Z}_2$	• $\boldsymbol{E} \parallel [001]$ and $\boldsymbol{B} \parallel [001]$ and strain $\perp [001]$
$Pn'c'2$ (30.115) $\mathbb{Z}_1$	• $\boldsymbol{E} \parallel [001]$ and $\boldsymbol{B} \parallel [001]$
P_a2 (3.4) $\mathbb{Z}_2$	• $\boldsymbol{E} \parallel [001]$ and strain $\perp [001]$
$P2/c$ (13.65) $\mathbb{Z}_2 \times \mathbb{Z}_2$	• $\boldsymbol{B} \parallel [001]$ and strain $\perp [001]$
P_acc2 (27.83) $\mathbb{Z}_2$	• $\boldsymbol{E} \parallel [001]$
$Pb'an'$ (50.282) $\mathbb{Z}_2$	• $\boldsymbol{B} \parallel [001]$
$P_S\bar{1}$ (2.7) $\mathbb{Z}_2 \times \mathbb{Z}_2 \times \mathbb{Z}_4$	• strain in generic direction
P_b2_1/c (14.81) $\mathbb{Z}_2$	• strain $\perp [100]$
P_a2/c (13.70) $\mathbb{Z}_2 \times \mathbb{Z}_2$	• strain $\perp [010]$
P_c2/c (13.72) $\mathbb{Z}_2 \times \mathbb{Z}_2$	• strain $\perp [001]$

Table. S 457: Subgroups of P_bcca (54.347) that can be realized by applying $\boldsymbol{E}/\boldsymbol{B}$ fields and/or uniaxial strains.

Subgroup & SI group	Perturbation
P_S1 (1.3) $\mathbb{Z}_1$	• $\boldsymbol{E} \parallel [100]$ and strain in generic direction<br>• $\boldsymbol{E} \parallel [010]$ and strain in generic direction<br>• $\boldsymbol{E} \parallel [001]$ and strain in generic direction<br>• $\boldsymbol{E} \perp [100]$ and strain $\perp [010]$<br>• $\boldsymbol{E} \perp [100]$ and strain $\perp [001]$<br>• $\boldsymbol{E} \perp [100]$ and strain in generic direction<br>• $\boldsymbol{E} \perp [010]$ and strain $\perp [100]$<br>• $\boldsymbol{E} \perp [010]$ and strain $\perp [001]$<br>• $\boldsymbol{E} \perp [010]$ and strain in generic direction<br>• $\boldsymbol{E} \perp [001]$ and strain $\perp [100]$<br>• $\boldsymbol{E} \perp [001]$ and strain $\perp [010]$<br>• $\boldsymbol{E} \perp [001]$ and strain in generic direction<br>• $\boldsymbol{E}$ in generic direction
Pc (7.24) $\mathbb{Z}_1$	• $\boldsymbol{E} \parallel [100]$ and $\boldsymbol{B} \parallel [010]$ and strain $\perp [010]$<br>• $\boldsymbol{E} \parallel [001]$ and $\boldsymbol{B} \parallel [010]$ and strain $\perp [010]$<br>• $\boldsymbol{E} \perp [010]$ and $\boldsymbol{B} \parallel [010]$
Pc' (7.26) $\mathbb{Z}_1$	• $\boldsymbol{E} \parallel [100]$ and $\boldsymbol{B} \parallel [100]$ and strain $\perp [010]$<br>• $\boldsymbol{E} \parallel [100]$ and $\boldsymbol{B} \parallel [001]$ and strain $\perp [010]$<br>• $\boldsymbol{E} \parallel [100]$ and $\boldsymbol{B} \perp [010]$<br>• $\boldsymbol{E} \parallel [001]$ and $\boldsymbol{B} \parallel [100]$ and strain $\perp [010]$<br>• $\boldsymbol{E} \parallel [001]$ and $\boldsymbol{B} \parallel [001]$ and strain $\perp [010]$<br>• $\boldsymbol{E} \parallel [001]$ and $\boldsymbol{B} \perp [010]$<br>• $\boldsymbol{E} \perp [010]$ and $\boldsymbol{B} \parallel [100]$<br>• $\boldsymbol{E} \perp [010]$ and $\boldsymbol{B} \parallel [001]$<br>• $\boldsymbol{E} \perp [010]$ and $\boldsymbol{B} \perp [010]$
P_bc (7.29) $\mathbb{Z}_1$	• $\boldsymbol{E} \parallel [100]$ and strain $\perp [010]$<br>• $\boldsymbol{E} \parallel [001]$ and strain $\perp [010]$<br>• $\boldsymbol{E} \perp [010]$
Pc (7.24) $\mathbb{Z}_1$	• $\boldsymbol{E} \parallel [100]$ and $\boldsymbol{B} \parallel [001]$ and strain $\perp [001]$<br>• $\boldsymbol{E} \parallel [010]$ and $\boldsymbol{B} \parallel [001]$ and strain $\perp [001]$<br>• $\boldsymbol{E} \perp [001]$ and $\boldsymbol{B} \parallel [001]$

Continued on next page

...continued

Subgroup & SI group	Perturbation
Pc' (7.26) $\mathbb{Z}_1$	• $\boldsymbol{E} \parallel [100]$ and $\boldsymbol{B} \parallel [100]$ and strain $\perp [001]$<br>• $\boldsymbol{E} \parallel [100]$ and $\boldsymbol{B} \parallel [010]$ and strain $\perp [001]$<br>• $\boldsymbol{E} \parallel [100]$ and $\boldsymbol{B} \perp [001]$<br>• $\boldsymbol{E} \parallel [010]$ and $\boldsymbol{B} \parallel [100]$ and strain $\perp [001]$<br>• $\boldsymbol{E} \parallel [010]$ and $\boldsymbol{B} \parallel [010]$ and strain $\perp [001]$<br>• $\boldsymbol{E} \parallel [010]$ and $\boldsymbol{B} \perp [001]$<br>• $\boldsymbol{E} \perp [001]$ and $\boldsymbol{B} \parallel [100]$<br>• $\boldsymbol{E} \perp [001]$ and $\boldsymbol{B} \parallel [010]$<br>• $\boldsymbol{E} \perp [001]$ and $\boldsymbol{B} \perp [001]$
P_ac (7.27) $\mathbb{Z}_1$	• $\boldsymbol{E} \parallel [100]$ and strain $\perp [001]$<br>• $\boldsymbol{E} \parallel [010]$ and strain $\perp [001]$<br>• $\boldsymbol{E} \perp [001]$
$P2_1$ (4.7) $\mathbb{Z}_1$	• $\boldsymbol{E} \parallel [100]$ and $\boldsymbol{B} \parallel [100]$ and strain $\perp [100]$
$Pn'a'2_1$ (33.148) $\mathbb{Z}_1$	• $\boldsymbol{E} \parallel [100]$ and $\boldsymbol{B} \parallel [100]$
$P2_1'$ (4.9) $\mathbb{Z}_1$	• $\boldsymbol{E} \parallel [100]$ and $\boldsymbol{B} \parallel [010]$ and strain $\perp [100]$<br>• $\boldsymbol{E} \parallel [100]$ and $\boldsymbol{B} \parallel [001]$ and strain $\perp [100]$<br>• $\boldsymbol{E} \parallel [100]$ and $\boldsymbol{B} \perp [100]$
$Pca'2_1'$ (29.102) $\mathbb{Z}_1$	• $\boldsymbol{E} \parallel [100]$ and $\boldsymbol{B} \parallel [001]$
$Pn'a2_1'$ (33.146) $\mathbb{Z}_1$	• $\boldsymbol{E} \parallel [100]$ and $\boldsymbol{B} \parallel [010]$
P_a2_1 (4.10) $\mathbb{Z}_1$	• $\boldsymbol{E} \parallel [100]$ and strain $\perp [100]$
P_bca2_1 (29.105) $\mathbb{Z}_2$	• $\boldsymbol{E} \parallel [100]$
$P2$ (3.1) $\mathbb{Z}_2$	• $\boldsymbol{E} \parallel [010]$ and $\boldsymbol{B} \parallel [010]$ and strain $\perp [010]$
$P2_1'$ (4.9) $\mathbb{Z}_1$	• $\boldsymbol{E} \parallel [010]$ and $\boldsymbol{B} \parallel [100]$ and strain $\perp [010]$<br>• $\boldsymbol{E} \parallel [010]$ and $\boldsymbol{B} \parallel [001]$ and strain $\perp [010]$<br>• $\boldsymbol{E} \parallel [010]$ and $\boldsymbol{B} \perp [010]$
P_b2 (3.5) $\mathbb{Z}_2$	• $\boldsymbol{E} \parallel [010]$ and strain $\perp [010]$

Continued on next page

...continued

Subgroup & SI group	Perturbation
$P\bar{1}$ (2.4) $\mathbb{Z}_2 \times \mathbb{Z}_2 \times \mathbb{Z}_2 \times \mathbb{Z}_4$	• $\boldsymbol{B} \parallel [100]$ and strain in generic direction<br>• $\boldsymbol{B} \parallel [010]$ and strain in generic direction<br>• $\boldsymbol{B} \parallel [001]$ and strain in generic direction<br>• $\boldsymbol{B} \perp [100]$ and strain $\perp [010]$<br>• $\boldsymbol{B} \perp [100]$ and strain $\perp [001]$<br>• $\boldsymbol{B} \perp [100]$ and strain in generic direction<br>• $\boldsymbol{B} \perp [010]$ and strain $\perp [100]$<br>• $\boldsymbol{B} \perp [010]$ and strain $\perp [001]$<br>• $\boldsymbol{B} \perp [010]$ and strain in generic direction<br>• $\boldsymbol{B} \perp [001]$ and strain $\perp [100]$<br>• $\boldsymbol{B} \perp [001]$ and strain $\perp [010]$<br>• $\boldsymbol{B} \perp [001]$ and strain in generic direction<br>• $\boldsymbol{B}$ in generic direction
$P2/c$ (13.65) $\mathbb{Z}_2 \times \mathbb{Z}_2$	• $\boldsymbol{B} \parallel [010]$ and strain $\perp [010]$
$P2_1'/c'$ (14.79) $\mathbb{Z}_2 \times \mathbb{Z}_4$	• $\boldsymbol{B} \parallel [100]$ and strain $\perp [010]$<br>• $\boldsymbol{B} \parallel [001]$ and strain $\perp [010]$<br>• $\boldsymbol{B} \perp [010]$
$P_S\bar{1}$ (2.7) $\mathbb{Z}_2 \times \mathbb{Z}_2 \times \mathbb{Z}_4$	• strain in generic direction
P_b2/c (13.71) $\mathbb{Z}_2 \times \mathbb{Z}_2$	• strain $\perp [010]$
Pc (7.24) $\mathbb{Z}_1$	• $\boldsymbol{E} \parallel [010]$ and $\boldsymbol{B} \parallel [100]$ and strain $\perp [100]$<br>• $\boldsymbol{E} \parallel [001]$ and $\boldsymbol{B} \parallel [100]$ and strain $\perp [100]$<br>• $\boldsymbol{E} \perp [100]$ and $\boldsymbol{B} \parallel [100]$
$Pn'a2_1'$ (33.146) $\mathbb{Z}_1$	• $\boldsymbol{E} \parallel [010]$ and $\boldsymbol{B} \parallel [100]$
$P2_1/c$ (14.75) $\mathbb{Z}_2$	• $\boldsymbol{B} \parallel [100]$ and strain $\perp [100]$
Pc' (7.26) $\mathbb{Z}_1$	• $\boldsymbol{E} \parallel [010]$ and $\boldsymbol{B} \parallel [010]$ and strain $\perp [100]$<br>• $\boldsymbol{E} \parallel [010]$ and $\boldsymbol{B} \parallel [001]$ and strain $\perp [100]$<br>• $\boldsymbol{E} \parallel [010]$ and $\boldsymbol{B} \perp [100]$<br>• $\boldsymbol{E} \parallel [001]$ and $\boldsymbol{B} \parallel [010]$ and strain $\perp [100]$<br>• $\boldsymbol{E} \parallel [001]$ and $\boldsymbol{B} \parallel [001]$ and strain $\perp [100]$<br>• $\boldsymbol{E} \parallel [001]$ and $\boldsymbol{B} \perp [100]$<br>• $\boldsymbol{E} \perp [100]$ and $\boldsymbol{B} \parallel [010]$<br>• $\boldsymbol{E} \perp [100]$ and $\boldsymbol{B} \parallel [001]$<br>• $\boldsymbol{E} \perp [100]$ and $\boldsymbol{B} \perp [100]$
$Pn'n'2$ (34.159) $\mathbb{Z}_1$	• $\boldsymbol{E} \parallel [010]$ and $\boldsymbol{B} \parallel [010]$
$Pn'a2_1'$ (33.146) $\mathbb{Z}_1$	• $\boldsymbol{E} \parallel [010]$ and $\boldsymbol{B} \parallel [001]$

Continued on next page

. . . continued

Subgroup & SI group	Perturbation
$P2_1'/c'$ (14.79) $\mathbb{Z}_2 \times \mathbb{Z}_4$	• $\boldsymbol{B} \parallel [010]$ and strain $\perp [100]$<br>• $\boldsymbol{B} \parallel [001]$ and strain $\perp [100]$<br>• $\boldsymbol{B} \perp [100]$
P_ac (7.27) $\mathbb{Z}_1$	• $\boldsymbol{E} \parallel [010]$ and strain $\perp [100]$<br>• $\boldsymbol{E} \parallel [001]$ and strain $\perp [100]$<br>• $\boldsymbol{E} \perp [100]$
P_cba2 (32.139) $\mathbb{Z}_2$	• $\boldsymbol{E} \parallel [010]$
P_a2_1/c (14.80) $\mathbb{Z}_2$	• strain $\perp [100]$
$P2$ (3.1) $\mathbb{Z}_2$	• $\boldsymbol{E} \parallel [001]$ and $\boldsymbol{B} \parallel [001]$ and strain $\perp [001]$
$P2/c$ (13.65) $\mathbb{Z}_2 \times \mathbb{Z}_2$	• $\boldsymbol{B} \parallel [001]$ and strain $\perp [001]$
$Pn'c'2$ (30.115) $\mathbb{Z}_1$	• $\boldsymbol{E} \parallel [001]$ and $\boldsymbol{B} \parallel [001]$
$Pb'cn'$ (60.424) $\mathbb{Z}_2 \times \mathbb{Z}_2$	• $\boldsymbol{B} \parallel [001]$
$P2'$ (3.3) $\mathbb{Z}_1$	• $\boldsymbol{E} \parallel [001]$ and $\boldsymbol{B} \parallel [100]$ and strain $\perp [001]$<br>• $\boldsymbol{E} \parallel [001]$ and $\boldsymbol{B} \parallel [010]$ and strain $\perp [001]$<br>• $\boldsymbol{E} \parallel [001]$ and $\boldsymbol{B} \perp [001]$
$P2'/c'$ (13.69) $\mathbb{Z}_2 \times \mathbb{Z}_2 \times \mathbb{Z}_4$	• $\boldsymbol{B} \parallel [100]$ and strain $\perp [001]$<br>• $\boldsymbol{B} \parallel [010]$ and strain $\perp [001]$<br>• $\boldsymbol{B} \perp [001]$
$Pc'c2'$ (27.80) $\mathbb{Z}_1$	• $\boldsymbol{E} \parallel [001]$ and $\boldsymbol{B} \parallel [100]$
$Pc'cn'$ (56.370) $\mathbb{Z}_2$	• $\boldsymbol{B} \parallel [100]$
$Pn'c2'$ (30.113) $\mathbb{Z}_1$	• $\boldsymbol{E} \parallel [001]$ and $\boldsymbol{B} \parallel [010]$
$Pn'n'a$ (52.310) $\mathbb{Z}_2$	• $\boldsymbol{B} \parallel [010]$
P_a2 (3.4) $\mathbb{Z}_2$	• $\boldsymbol{E} \parallel [001]$ and strain $\perp [001]$
P_a2/c (13.70) $\mathbb{Z}_2 \times \mathbb{Z}_2$	• strain $\perp [001]$
P_acc2 (27.83) $\mathbb{Z}_2$	• $\boldsymbol{E} \parallel [001]$

Table. S 458: Subgroups of P_ccca (54.348) that can be realized by applying $\boldsymbol{E}/\boldsymbol{B}$ fields and/or uniaxial strains.

Subgroup & SI group	Perturbation

Continued on next page

...continued

Subgroup & SI group	Perturbation
P_S1 (1.3) $\mathbb{Z}_1$	• $\boldsymbol{E} \parallel [100]$ and strain in generic direction<br>• $\boldsymbol{E} \parallel [010]$ and strain in generic direction<br>• $\boldsymbol{E} \parallel [001]$ and strain in generic direction<br>• $\boldsymbol{E} \perp [100]$ and strain $\perp [010]$<br>• $\boldsymbol{E} \perp [100]$ and strain $\perp [001]$<br>• $\boldsymbol{E} \perp [100]$ and strain in generic direction<br>• $\boldsymbol{E} \perp [010]$ and strain $\perp [100]$<br>• $\boldsymbol{E} \perp [010]$ and strain $\perp [001]$<br>• $\boldsymbol{E} \perp [010]$ and strain in generic direction<br>• $\boldsymbol{E} \perp [001]$ and strain $\perp [100]$<br>• $\boldsymbol{E} \perp [001]$ and strain $\perp [010]$<br>• $\boldsymbol{E} \perp [001]$ and strain in generic direction<br>• $\boldsymbol{E}$ in generic direction
Pm' (6.20) $\mathbb{Z}_1$	• $\boldsymbol{E} \parallel [100]$ and $\boldsymbol{B} \parallel [100]$ and strain $\perp [010]$<br>• $\boldsymbol{E} \parallel [100]$ and $\boldsymbol{B} \parallel [001]$ and strain $\perp [010]$<br>• $\boldsymbol{E} \parallel [100]$ and $\boldsymbol{B} \perp [010]$<br>• $\boldsymbol{E} \parallel [001]$ and $\boldsymbol{B} \parallel [100]$ and strain $\perp [010]$<br>• $\boldsymbol{E} \parallel [001]$ and $\boldsymbol{B} \parallel [001]$ and strain $\perp [010]$<br>• $\boldsymbol{E} \parallel [001]$ and $\boldsymbol{B} \perp [010]$<br>• $\boldsymbol{E} \perp [010]$ and $\boldsymbol{B} \parallel [100]$<br>• $\boldsymbol{E} \perp [010]$ and $\boldsymbol{B} \parallel [001]$<br>• $\boldsymbol{E} \perp [010]$ and $\boldsymbol{B} \perp [010]$
Pc (7.24) $\mathbb{Z}_1$	• $\boldsymbol{E} \parallel [100]$ and $\boldsymbol{B} \parallel [010]$ and strain $\perp [010]$<br>• $\boldsymbol{E} \parallel [001]$ and $\boldsymbol{B} \parallel [010]$ and strain $\perp [010]$<br>• $\boldsymbol{E} \perp [010]$ and $\boldsymbol{B} \parallel [010]$
P_cc (7.28) $\mathbb{Z}_1$	• $\boldsymbol{E} \parallel [100]$ and strain $\perp [010]$<br>• $\boldsymbol{E} \parallel [001]$ and strain $\perp [010]$<br>• $\boldsymbol{E} \perp [010]$
Pc (7.24) $\mathbb{Z}_1$	• $\boldsymbol{E} \parallel [100]$ and $\boldsymbol{B} \parallel [001]$ and strain $\perp [001]$<br>• $\boldsymbol{E} \parallel [010]$ and $\boldsymbol{B} \parallel [001]$ and strain $\perp [001]$<br>• $\boldsymbol{E} \perp [001]$ and $\boldsymbol{B} \parallel [001]$

Continued on next page

. . . continued

Subgroup & SI group	Perturbation
Pc' (7.26) $\mathbb{Z}_1$	• $\boldsymbol{E} \parallel [100]$ and $\boldsymbol{B} \parallel [100]$ and strain $\perp [001]$<br>• $\boldsymbol{E} \parallel [100]$ and $\boldsymbol{B} \parallel [010]$ and strain $\perp [001]$<br>• $\boldsymbol{E} \parallel [100]$ and $\boldsymbol{B} \perp [001]$<br>• $\boldsymbol{E} \parallel [010]$ and $\boldsymbol{B} \parallel [100]$ and strain $\perp [001]$<br>• $\boldsymbol{E} \parallel [010]$ and $\boldsymbol{B} \parallel [010]$ and strain $\perp [001]$<br>• $\boldsymbol{E} \parallel [010]$ and $\boldsymbol{B} \perp [001]$<br>• $\boldsymbol{E} \perp [001]$ and $\boldsymbol{B} \parallel [100]$<br>• $\boldsymbol{E} \perp [001]$ and $\boldsymbol{B} \parallel [010]$<br>• $\boldsymbol{E} \perp [001]$ and $\boldsymbol{B} \perp [001]$
P_bc (7.29) $\mathbb{Z}_1$	• $\boldsymbol{E} \parallel [100]$ and strain $\perp [001]$<br>• $\boldsymbol{E} \parallel [010]$ and strain $\perp [001]$<br>• $\boldsymbol{E} \perp [001]$
$P2_1'$ (4.9) $\mathbb{Z}_1$	• $\boldsymbol{E} \parallel [100]$ and $\boldsymbol{B} \parallel [010]$ and strain $\perp [100]$<br>• $\boldsymbol{E} \parallel [100]$ and $\boldsymbol{B} \parallel [001]$ and strain $\perp [100]$<br>• $\boldsymbol{E} \parallel [100]$ and $\boldsymbol{B} \perp [100]$
$Pm'c2_1'$ (26.68) $\mathbb{Z}_1$	• $\boldsymbol{E} \parallel [100]$ and $\boldsymbol{B} \parallel [001]$
$Pc'a2_1'$ (29.101) $\mathbb{Z}_1$	• $\boldsymbol{E} \parallel [100]$ and $\boldsymbol{B} \parallel [010]$
$P2_1$ (4.7) $\mathbb{Z}_1$	• $\boldsymbol{E} \parallel [100]$ and $\boldsymbol{B} \parallel [100]$ and strain $\perp [100]$
$Pm'c'2_1$ (26.70) $\mathbb{Z}_1$	• $\boldsymbol{E} \parallel [100]$ and $\boldsymbol{B} \parallel [100]$
P_a2_1 (4.10) $\mathbb{Z}_1$	• $\boldsymbol{E} \parallel [100]$ and strain $\perp [100]$
P_aca2_1 (29.104) $\mathbb{Z}_1$	• $\boldsymbol{E} \parallel [100]$
$P2'$ (3.3) $\mathbb{Z}_1$	• $\boldsymbol{E} \parallel [010]$ and $\boldsymbol{B} \parallel [100]$ and strain $\perp [010]$<br>• $\boldsymbol{E} \parallel [010]$ and $\boldsymbol{B} \parallel [001]$ and strain $\perp [010]$<br>• $\boldsymbol{E} \parallel [010]$ and $\boldsymbol{B} \perp [010]$
$P2$ (3.1) $\mathbb{Z}_2$	• $\boldsymbol{E} \parallel [010]$ and $\boldsymbol{B} \parallel [010]$ and strain $\perp [010]$
P_a2 (3.4) $\mathbb{Z}_2$	• $\boldsymbol{E} \parallel [010]$ and strain $\perp [010]$

Continued on next page

...continued

Subgroup & SI group	Perturbation
$P\bar{1}$ (2.4) $\mathbb{Z}_2 \times \mathbb{Z}_2 \times \mathbb{Z}_2 \times \mathbb{Z}_4$	• $\boldsymbol{B} \parallel [100]$ and strain in generic direction<br>• $\boldsymbol{B} \parallel [010]$ and strain in generic direction<br>• $\boldsymbol{B} \parallel [001]$ and strain in generic direction<br>• $\boldsymbol{B} \perp [100]$ and strain $\perp [010]$<br>• $\boldsymbol{B} \perp [100]$ and strain $\perp [001]$<br>• $\boldsymbol{B} \perp [100]$ and strain in generic direction<br>• $\boldsymbol{B} \perp [010]$ and strain $\perp [100]$<br>• $\boldsymbol{B} \perp [010]$ and strain $\perp [001]$<br>• $\boldsymbol{B} \perp [010]$ and strain in generic direction<br>• $\boldsymbol{B} \perp [001]$ and strain $\perp [100]$<br>• $\boldsymbol{B} \perp [001]$ and strain $\perp [010]$<br>• $\boldsymbol{B} \perp [001]$ and strain in generic direction<br>• $\boldsymbol{B}$ in generic direction
$P2'/m'$ (10.46) $\mathbb{Z}_2 \times \mathbb{Z}_2 \times \mathbb{Z}_2 \times \mathbb{Z}_4$	• $\boldsymbol{B} \parallel [100]$ and strain $\perp [010]$<br>• $\boldsymbol{B} \parallel [001]$ and strain $\perp [010]$<br>• $\boldsymbol{B} \perp [010]$
$P2/c$ (13.65) $\mathbb{Z}_2 \times \mathbb{Z}_2$	• $\boldsymbol{B} \parallel [010]$ and strain $\perp [010]$
$P_S\bar{1}$ (2.7) $\mathbb{Z}_2 \times \mathbb{Z}_2 \times \mathbb{Z}_4$	• strain in generic direction
P_c2/c (13.72) $\mathbb{Z}_2 \times \mathbb{Z}_2$	• strain $\perp [010]$
Pm' (6.20) $\mathbb{Z}_1$	• $\boldsymbol{E} \parallel [010]$ and $\boldsymbol{B} \parallel [010]$ and strain $\perp [100]$<br>• $\boldsymbol{E} \parallel [010]$ and $\boldsymbol{B} \parallel [001]$ and strain $\perp [100]$<br>• $\boldsymbol{E} \parallel [010]$ and $\boldsymbol{B} \perp [100]$<br>• $\boldsymbol{E} \parallel [001]$ and $\boldsymbol{B} \parallel [010]$ and strain $\perp [100]$<br>• $\boldsymbol{E} \parallel [001]$ and $\boldsymbol{B} \parallel [001]$ and strain $\perp [100]$<br>• $\boldsymbol{E} \parallel [001]$ and $\boldsymbol{B} \perp [100]$<br>• $\boldsymbol{E} \perp [100]$ and $\boldsymbol{B} \parallel [010]$<br>• $\boldsymbol{E} \perp [100]$ and $\boldsymbol{B} \parallel [001]$<br>• $\boldsymbol{E} \perp [100]$ and $\boldsymbol{B} \perp [100]$
$Pm'a2'$ (28.89) $\mathbb{Z}_1$	• $\boldsymbol{E} \parallel [010]$ and $\boldsymbol{B} \parallel [001]$
$Pm'a'2$ (28.91) $\mathbb{Z}_2$	• $\boldsymbol{E} \parallel [010]$ and $\boldsymbol{B} \parallel [010]$
$P2_1'/m'$ (11.54) $\mathbb{Z}_2 \times \mathbb{Z}_2 \times \mathbb{Z}_4$	• $\boldsymbol{B} \parallel [010]$ and strain $\perp [100]$<br>• $\boldsymbol{B} \parallel [001]$ and strain $\perp [100]$<br>• $\boldsymbol{B} \perp [100]$
Pc (7.24) $\mathbb{Z}_1$	• $\boldsymbol{E} \parallel [010]$ and $\boldsymbol{B} \parallel [100]$ and strain $\perp [100]$<br>• $\boldsymbol{E} \parallel [001]$ and $\boldsymbol{B} \parallel [100]$ and strain $\perp [100]$<br>• $\boldsymbol{E} \perp [100]$ and $\boldsymbol{B} \parallel [100]$

Continued on next page

...continued

Subgroup & SI group	Perturbation
$Pb'a2'$ (32.137) $\mathbb{Z}_1$	• $\boldsymbol{E} \parallel [010]$ and $\boldsymbol{B} \parallel [100]$
$P2_1/c$ (14.75) $\mathbb{Z}_2$	• $\boldsymbol{B} \parallel [100]$ and strain $\perp [100]$
P_cc (7.28) $\mathbb{Z}_1$	• $\boldsymbol{E} \parallel [010]$ and strain $\perp [100]$<br>• $\boldsymbol{E} \parallel [001]$ and strain $\perp [100]$<br>• $\boldsymbol{E} \perp [100]$
P_bba2 (32.140) $\mathbb{Z}_1$	• $\boldsymbol{E} \parallel [010]$
P_c2_1/c (14.82) $\mathbb{Z}_2$	• strain $\perp [100]$
$P2$ (3.1) $\mathbb{Z}_2$	• $\boldsymbol{E} \parallel [001]$ and $\boldsymbol{B} \parallel [001]$ and strain $\perp [001]$
$P2/c$ (13.65) $\mathbb{Z}_2 \times \mathbb{Z}_2$	• $\boldsymbol{B} \parallel [001]$ and strain $\perp [001]$
$Pm'm'2$ (25.60) $\mathbb{Z}_2$	• $\boldsymbol{E} \parallel [001]$ and $\boldsymbol{B} \parallel [001]$
$Pm'm'a$ (51.294) $\mathbb{Z}_2 \times \mathbb{Z}_2$	• $\boldsymbol{B} \parallel [001]$
$P2_1'$ (4.9) $\mathbb{Z}_1$	• $\boldsymbol{E} \parallel [001]$ and $\boldsymbol{B} \parallel [100]$ and strain $\perp [001]$<br>• $\boldsymbol{E} \parallel [001]$ and $\boldsymbol{B} \parallel [010]$ and strain $\perp [001]$<br>• $\boldsymbol{E} \parallel [001]$ and $\boldsymbol{B} \perp [001]$
$P2_1'/c'$ (14.79) $\mathbb{Z}_2 \times \mathbb{Z}_4$	• $\boldsymbol{B} \parallel [100]$ and strain $\perp [001]$<br>• $\boldsymbol{B} \parallel [010]$ and strain $\perp [001]$<br>• $\boldsymbol{B} \perp [001]$
$Pm'c2_1'$ (26.68) $\mathbb{Z}_1$	• $\boldsymbol{E} \parallel [001]$ and $\boldsymbol{B} \parallel [010]$
$Pbc'm'$ (57.383) $\mathbb{Z}_2 \times \mathbb{Z}_2$	• $\boldsymbol{B} \parallel [010]$
$Pm'c2_1'$ (26.68) $\mathbb{Z}_1$	• $\boldsymbol{E} \parallel [001]$ and $\boldsymbol{B} \parallel [100]$
$Pb'am'$ (55.358) $\mathbb{Z}_2$	• $\boldsymbol{B} \parallel [100]$
P_b2 (3.5) $\mathbb{Z}_2$	• $\boldsymbol{E} \parallel [001]$ and strain $\perp [001]$
P_b2/c (13.71) $\mathbb{Z}_2 \times \mathbb{Z}_2$	• strain $\perp [001]$
P_ccc2 (27.82) $\mathbb{Z}_2$	• $\boldsymbol{E} \parallel [001]$

Table. S 459: Subgroups of P_Acca (54.349) that can be realized by applying $\boldsymbol{E}/\boldsymbol{B}$ fields and/or uniaxial strains.

Subgroup & SI group	Perturbation

Continued on next page

...continued

Subgroup & SI group	Perturbation
P_S1 (1.3) $\mathbb{Z}_1$	• $\boldsymbol{E}$ ∥ [100] and strain in generic direction<br>• $\boldsymbol{E}$ ∥ [010] and strain in generic direction<br>• $\boldsymbol{E}$ ∥ [001] and strain in generic direction<br>• $\boldsymbol{E}$ ⊥ [100] and strain ⊥ [010]<br>• $\boldsymbol{E}$ ⊥ [100] and strain ⊥ [001]<br>• $\boldsymbol{E}$ ⊥ [100] and strain in generic direction<br>• $\boldsymbol{E}$ ⊥ [010] and strain ⊥ [100]<br>• $\boldsymbol{E}$ ⊥ [010] and strain ⊥ [001]<br>• $\boldsymbol{E}$ ⊥ [010] and strain in generic direction<br>• $\boldsymbol{E}$ ⊥ [001] and strain ⊥ [100]<br>• $\boldsymbol{E}$ ⊥ [001] and strain ⊥ [010]<br>• $\boldsymbol{E}$ ⊥ [001] and strain in generic direction<br>• $\boldsymbol{E}$ in generic direction
Pc (7.24) $\mathbb{Z}_1$	• $\boldsymbol{E}$ ∥ [100] and $\boldsymbol{B}$ ∥ [010] and strain ⊥ [010]<br>• $\boldsymbol{E}$ ∥ [001] and $\boldsymbol{B}$ ∥ [010] and strain ⊥ [010]<br>• $\boldsymbol{E}$ ⊥ [010] and $\boldsymbol{B}$ ∥ [010]
Pm' (6.20) $\mathbb{Z}_1$	• $\boldsymbol{E}$ ∥ [100] and $\boldsymbol{B}$ ∥ [100] and strain ⊥ [010]<br>• $\boldsymbol{E}$ ∥ [100] and $\boldsymbol{B}$ ∥ [001] and strain ⊥ [010]<br>• $\boldsymbol{E}$ ∥ [100] and $\boldsymbol{B}$ ⊥ [010]<br>• $\boldsymbol{E}$ ∥ [001] and $\boldsymbol{B}$ ∥ [100] and strain ⊥ [010]<br>• $\boldsymbol{E}$ ∥ [001] and $\boldsymbol{B}$ ∥ [001] and strain ⊥ [010]<br>• $\boldsymbol{E}$ ∥ [001] and $\boldsymbol{B}$ ⊥ [010]<br>• $\boldsymbol{E}$ ⊥ [010] and $\boldsymbol{B}$ ∥ [100]<br>• $\boldsymbol{E}$ ⊥ [010] and $\boldsymbol{B}$ ∥ [001]<br>• $\boldsymbol{E}$ ⊥ [010] and $\boldsymbol{B}$ ⊥ [010]
P_Ac (7.31) $\mathbb{Z}_1$	• $\boldsymbol{E}$ ∥ [100] and strain ⊥ [010]<br>• $\boldsymbol{E}$ ∥ [001] and strain ⊥ [010]<br>• $\boldsymbol{E}$ ⊥ [010]
Pc (7.24) $\mathbb{Z}_1$	• $\boldsymbol{E}$ ∥ [100] and $\boldsymbol{B}$ ∥ [001] and strain ⊥ [001]<br>• $\boldsymbol{E}$ ∥ [010] and $\boldsymbol{B}$ ∥ [001] and strain ⊥ [001]<br>• $\boldsymbol{E}$ ⊥ [001] and $\boldsymbol{B}$ ∥ [001]

Continued on next page

. . . continued

Subgroup & SI group	Perturbation
Pc' (7.26) $\mathbb{Z}_1$	• $\boldsymbol{E}$ ∥ [100] and $\boldsymbol{B}$ ∥ [100] and strain ⊥ [001]<br>• $\boldsymbol{E}$ ∥ [100] and $\boldsymbol{B}$ ∥ [010] and strain ⊥ [001]<br>• $\boldsymbol{E}$ ∥ [100] and $\boldsymbol{B}$ ⊥ [001]<br>• $\boldsymbol{E}$ ∥ [010] and $\boldsymbol{B}$ ∥ [100] and strain ⊥ [001]<br>• $\boldsymbol{E}$ ∥ [010] and $\boldsymbol{B}$ ∥ [010] and strain ⊥ [001]<br>• $\boldsymbol{E}$ ∥ [010] and $\boldsymbol{B}$ ⊥ [001]<br>• $\boldsymbol{E}$ ⊥ [001] and $\boldsymbol{B}$ ∥ [100]<br>• $\boldsymbol{E}$ ⊥ [001] and $\boldsymbol{B}$ ∥ [010]<br>• $\boldsymbol{E}$ ⊥ [001] and $\boldsymbol{B}$ ⊥ [001]
P_Cc (7.30) $\mathbb{Z}_1$	• $\boldsymbol{E}$ ∥ [100] and strain ⊥ [001]<br>• $\boldsymbol{E}$ ∥ [010] and strain ⊥ [001]<br>• $\boldsymbol{E}$ ⊥ [001]
$P2_1$ (4.7) $\mathbb{Z}_1$	• $\boldsymbol{E}$ ∥ [100] and $\boldsymbol{B}$ ∥ [100] and strain ⊥ [100]
$Pm'n'2_1$ (31.127) $\mathbb{Z}_1$	• $\boldsymbol{E}$ ∥ [100] and $\boldsymbol{B}$ ∥ [100]
$P2_1'$ (4.9) $\mathbb{Z}_1$	• $\boldsymbol{E}$ ∥ [100] and $\boldsymbol{B}$ ∥ [010] and strain ⊥ [100]<br>• $\boldsymbol{E}$ ∥ [100] and $\boldsymbol{B}$ ∥ [001] and strain ⊥ [100]<br>• $\boldsymbol{E}$ ∥ [100] and $\boldsymbol{B}$ ⊥ [100]
$Pm'c2_1'$ (26.68) $\mathbb{Z}_1$	• $\boldsymbol{E}$ ∥ [100] and $\boldsymbol{B}$ ∥ [001]
$Pn'a2_1'$ (33.146) $\mathbb{Z}_1$	• $\boldsymbol{E}$ ∥ [100] and $\boldsymbol{B}$ ∥ [010]
P_a2_1 (4.10) $\mathbb{Z}_1$	• $\boldsymbol{E}$ ∥ [100] and strain ⊥ [100]
P_Cca2_1 (29.109) $\mathbb{Z}_1$	• $\boldsymbol{E}$ ∥ [100]
$P2$ (3.1) $\mathbb{Z}_2$	• $\boldsymbol{E}$ ∥ [010] and $\boldsymbol{B}$ ∥ [010] and strain ⊥ [010]
$P2_1'$ (4.9) $\mathbb{Z}_1$	• $\boldsymbol{E}$ ∥ [010] and $\boldsymbol{B}$ ∥ [100] and strain ⊥ [010]<br>• $\boldsymbol{E}$ ∥ [010] and $\boldsymbol{B}$ ∥ [001] and strain ⊥ [010]<br>• $\boldsymbol{E}$ ∥ [010] and $\boldsymbol{B}$ ⊥ [010]
P_C2 (3.6) $\mathbb{Z}_1$	• $\boldsymbol{E}$ ∥ [010] and strain ⊥ [010]

Continued on next page

...continued

Subgroup & SI group	Perturbation
$P\bar{1}$ (2.4) $\mathbb{Z}_2 \times \mathbb{Z}_2 \times \mathbb{Z}_2 \times \mathbb{Z}_4$	• $\boldsymbol{B} \parallel [100]$ and strain in generic direction<br>• $\boldsymbol{B} \parallel [010]$ and strain in generic direction<br>• $\boldsymbol{B} \parallel [001]$ and strain in generic direction<br>• $\boldsymbol{B} \perp [100]$ and strain $\perp [010]$<br>• $\boldsymbol{B} \perp [100]$ and strain $\perp [001]$<br>• $\boldsymbol{B} \perp [100]$ and strain in generic direction<br>• $\boldsymbol{B} \perp [010]$ and strain $\perp [100]$<br>• $\boldsymbol{B} \perp [010]$ and strain $\perp [001]$<br>• $\boldsymbol{B} \perp [010]$ and strain in generic direction<br>• $\boldsymbol{B} \perp [001]$ and strain $\perp [100]$<br>• $\boldsymbol{B} \perp [001]$ and strain $\perp [010]$<br>• $\boldsymbol{B} \perp [001]$ and strain in generic direction<br>• $\boldsymbol{B}$ in generic direction
$P2/c$ (13.65) $\mathbb{Z}_2 \times \mathbb{Z}_2$	• $\boldsymbol{B} \parallel [010]$ and strain $\perp [010]$
$P2_1'/m'$ (11.54) $\mathbb{Z}_2 \times \mathbb{Z}_2 \times \mathbb{Z}_4$	• $\boldsymbol{B} \parallel [100]$ and strain $\perp [010]$<br>• $\boldsymbol{B} \parallel [001]$ and strain $\perp [010]$<br>• $\boldsymbol{B} \perp [010]$
$P_S\bar{1}$ (2.7) $\mathbb{Z}_2 \times \mathbb{Z}_2 \times \mathbb{Z}_4$	• strain in generic direction
P_A2/c (13.73) $\mathbb{Z}_2$	• strain $\perp [010]$
Pc (7.24) $\mathbb{Z}_1$	• $\boldsymbol{E} \parallel [010]$ and $\boldsymbol{B} \parallel [100]$ and strain $\perp [100]$<br>• $\boldsymbol{E} \parallel [001]$ and $\boldsymbol{B} \parallel [100]$ and strain $\perp [100]$<br>• $\boldsymbol{E} \perp [100]$ and $\boldsymbol{B} \parallel [100]$
$Pn'a2_1'$ (33.146) $\mathbb{Z}_1$	• $\boldsymbol{E} \parallel [010]$ and $\boldsymbol{B} \parallel [100]$
$P2_1/c$ (14.75) $\mathbb{Z}_2$	• $\boldsymbol{B} \parallel [100]$ and strain $\perp [100]$
Pc' (7.26) $\mathbb{Z}_1$	• $\boldsymbol{E} \parallel [010]$ and $\boldsymbol{B} \parallel [010]$ and strain $\perp [100]$<br>• $\boldsymbol{E} \parallel [010]$ and $\boldsymbol{B} \parallel [001]$ and strain $\perp [100]$<br>• $\boldsymbol{E} \parallel [010]$ and $\boldsymbol{B} \perp [100]$<br>• $\boldsymbol{E} \parallel [001]$ and $\boldsymbol{B} \parallel [010]$ and strain $\perp [100]$<br>• $\boldsymbol{E} \parallel [001]$ and $\boldsymbol{B} \parallel [001]$ and strain $\perp [100]$<br>• $\boldsymbol{E} \parallel [001]$ and $\boldsymbol{B} \perp [100]$<br>• $\boldsymbol{E} \perp [100]$ and $\boldsymbol{B} \parallel [010]$<br>• $\boldsymbol{E} \perp [100]$ and $\boldsymbol{B} \parallel [001]$<br>• $\boldsymbol{E} \perp [100]$ and $\boldsymbol{B} \perp [100]$
$Pn'c'2$ (30.115) $\mathbb{Z}_1$	• $\boldsymbol{E} \parallel [010]$ and $\boldsymbol{B} \parallel [010]$
$Pc'a2_1'$ (29.101) $\mathbb{Z}_1$	• $\boldsymbol{E} \parallel [010]$ and $\boldsymbol{B} \parallel [001]$

Continued on next page

...continued

Subgroup & SI group	Perturbation
$P2_1'/c'$ (14.79) $\mathbb{Z}_2\times\mathbb{Z}_4$	• $\boldsymbol{B}$ ∥ [010] and strain ⊥ [100]<br>• $\boldsymbol{B}$ ∥ [001] and strain ⊥ [100]<br>• $\boldsymbol{B}$ ⊥ [100]
P_ac (7.27) $\mathbb{Z}_1$	• $\boldsymbol{E}$ ∥ [010] and strain ⊥ [100]<br>• $\boldsymbol{E}$ ∥ [001] and strain ⊥ [100]<br>• $\boldsymbol{E}$ ⊥ [100]
P_Aba2 (32.142) $\mathbb{Z}_1$	• $\boldsymbol{E}$ ∥ [010]
P_a2_1/c (14.80) $\mathbb{Z}_2$	• strain ⊥ [100]
$P2$ (3.1) $\mathbb{Z}_2$	• $\boldsymbol{E}$ ∥ [001] and $\boldsymbol{B}$ ∥ [001] and strain ⊥ [001]
$P2/c$ (13.65) $\mathbb{Z}_2\times\mathbb{Z}_2$	• $\boldsymbol{B}$ ∥ [001] and strain ⊥ [001]
$Pm'a'2$ (28.91) $\mathbb{Z}_2$	• $\boldsymbol{E}$ ∥ [001] and $\boldsymbol{B}$ ∥ [001]
$Pbc'm'$ (57.383) $\mathbb{Z}_2\times\mathbb{Z}_2$	• $\boldsymbol{B}$ ∥ [001]
$P2_1'$ (4.9) $\mathbb{Z}_1$	• $\boldsymbol{E}$ ∥ [001] and $\boldsymbol{B}$ ∥ [100] and strain ⊥ [001]<br>• $\boldsymbol{E}$ ∥ [001] and $\boldsymbol{B}$ ∥ [010] and strain ⊥ [001]<br>• $\boldsymbol{E}$ ∥ [001] and $\boldsymbol{B}$ ⊥ [001]
$P2_1'/c'$ (14.79) $\mathbb{Z}_2\times\mathbb{Z}_4$	• $\boldsymbol{B}$ ∥ [100] and strain ⊥ [001]<br>• $\boldsymbol{B}$ ∥ [010] and strain ⊥ [001]<br>• $\boldsymbol{B}$ ⊥ [001]
$Pm'c2_1'$ (26.68) $\mathbb{Z}_1$	• $\boldsymbol{E}$ ∥ [001] and $\boldsymbol{B}$ ∥ [100]
$Pn'm'a$ (62.446) $\mathbb{Z}_2$	• $\boldsymbol{B}$ ∥ [100]
$Pca'2_1'$ (29.102) $\mathbb{Z}_1$	• $\boldsymbol{E}$ ∥ [001] and $\boldsymbol{B}$ ∥ [010]
$Pb'cn'$ (60.424) $\mathbb{Z}_2\times\mathbb{Z}_2$	• $\boldsymbol{B}$ ∥ [010]
P_C2 (3.6) $\mathbb{Z}_1$	• $\boldsymbol{E}$ ∥ [001] and strain ⊥ [001]
P_C2/c (13.74) $\mathbb{Z}_2$	• strain ⊥ [001]
P_Acc2 (27.85) $\mathbb{Z}_2$	• $\boldsymbol{E}$ ∥ [001]

Table. S 460: Subgroups of P_Bcca (54.350) that can be realized by applying $\boldsymbol{E}/\boldsymbol{B}$ fields and/or uniaxial strains.

Subgroup & SI group	Perturbation

Continued on next page

. . . continued

Subgroup & SI group	Perturbation
Pm' (6.20) $\mathbb{Z}_1$	• $\boldsymbol{E}$ ∥ [100] and $\boldsymbol{B}$ ∥ [100] and strain ⊥ [001]<br>• $\boldsymbol{E}$ ∥ [100] and $\boldsymbol{B}$ ∥ [010] and strain ⊥ [001]<br>• $\boldsymbol{E}$ ∥ [100] and $\boldsymbol{B}$ ⊥ [001]<br>• $\boldsymbol{E}$ ∥ [010] and $\boldsymbol{B}$ ∥ [100] and strain ⊥ [001]<br>• $\boldsymbol{E}$ ∥ [010] and $\boldsymbol{B}$ ∥ [010] and strain ⊥ [001]<br>• $\boldsymbol{E}$ ∥ [010] and $\boldsymbol{B}$ ⊥ [001]<br>• $\boldsymbol{E}$ ⊥ [001] and $\boldsymbol{B}$ ∥ [100]<br>• $\boldsymbol{E}$ ⊥ [001] and $\boldsymbol{B}$ ∥ [010]<br>• $\boldsymbol{E}$ ⊥ [001] and $\boldsymbol{B}$ ⊥ [001]
Pc (7.24) $\mathbb{Z}_1$	• $\boldsymbol{E}$ ∥ [100] and $\boldsymbol{B}$ ∥ [010] and strain ⊥ [010]<br>• $\boldsymbol{E}$ ∥ [001] and $\boldsymbol{B}$ ∥ [010] and strain ⊥ [010]<br>• $\boldsymbol{E}$ ⊥ [010] and $\boldsymbol{B}$ ∥ [010]
$P2'$ (3.3) $\mathbb{Z}_1$	• $\boldsymbol{E}$ ∥ [100] and $\boldsymbol{B}$ ∥ [010] and strain ⊥ [100]<br>• $\boldsymbol{E}$ ∥ [100] and $\boldsymbol{B}$ ∥ [001] and strain ⊥ [100]<br>• $\boldsymbol{E}$ ∥ [100] and $\boldsymbol{B}$ ⊥ [100]
$Pm'a2'$ (28.89) $\mathbb{Z}_1$	• $\boldsymbol{E}$ ∥ [100] and $\boldsymbol{B}$ ∥ [010]
P_S1 (1.3) $\mathbb{Z}_1$	• $\boldsymbol{E}$ ∥ [100] and strain in generic direction<br>• $\boldsymbol{E}$ ∥ [010] and strain in generic direction<br>• $\boldsymbol{E}$ ∥ [001] and strain in generic direction<br>• $\boldsymbol{E}$ ⊥ [100] and strain ⊥ [010]<br>• $\boldsymbol{E}$ ⊥ [100] and strain ⊥ [001]<br>• $\boldsymbol{E}$ ⊥ [100] and strain in generic direction<br>• $\boldsymbol{E}$ ⊥ [010] and strain ⊥ [100]<br>• $\boldsymbol{E}$ ⊥ [010] and strain ⊥ [001]<br>• $\boldsymbol{E}$ ⊥ [010] and strain in generic direction<br>• $\boldsymbol{E}$ ⊥ [001] and strain ⊥ [100]<br>• $\boldsymbol{E}$ ⊥ [001] and strain ⊥ [010]<br>• $\boldsymbol{E}$ ⊥ [001] and strain in generic direction<br>• $\boldsymbol{E}$ in generic direction
Pc (7.24) $\mathbb{Z}_1$	• $\boldsymbol{E}$ ∥ [100] and $\boldsymbol{B}$ ∥ [001] and strain ⊥ [001]<br>• $\boldsymbol{E}$ ∥ [010] and $\boldsymbol{B}$ ∥ [001] and strain ⊥ [001]<br>• $\boldsymbol{E}$ ⊥ [001] and $\boldsymbol{B}$ ∥ [001]
P_Ac (7.31) $\mathbb{Z}_1$	• $\boldsymbol{E}$ ∥ [100] and strain ⊥ [001]<br>• $\boldsymbol{E}$ ∥ [010] and strain ⊥ [001]<br>• $\boldsymbol{E}$ ⊥ [001]

Continued on next page

...continued

Subgroup & SI group	Perturbation
Pc' (7.26) $\mathbb{Z}_1$	• $\boldsymbol{E}$ ∥ [100] and $\boldsymbol{B}$ ∥ [100] and strain ⊥ [010]<br>• $\boldsymbol{E}$ ∥ [100] and $\boldsymbol{B}$ ∥ [001] and strain ⊥ [010]<br>• $\boldsymbol{E}$ ∥ [100] and $\boldsymbol{B}$ ⊥ [010]<br>• $\boldsymbol{E}$ ∥ [001] and $\boldsymbol{B}$ ∥ [100] and strain ⊥ [010]<br>• $\boldsymbol{E}$ ∥ [001] and $\boldsymbol{B}$ ∥ [001] and strain ⊥ [010]<br>• $\boldsymbol{E}$ ∥ [001] and $\boldsymbol{B}$ ⊥ [010]<br>• $\boldsymbol{E}$ ⊥ [010] and $\boldsymbol{B}$ ∥ [100]<br>• $\boldsymbol{E}$ ⊥ [010] and $\boldsymbol{B}$ ∥ [001]<br>• $\boldsymbol{E}$ ⊥ [010] and $\boldsymbol{B}$ ⊥ [010]
P_ac (7.27) $\mathbb{Z}_1$	• $\boldsymbol{E}$ ∥ [100] and strain ⊥ [010]<br>• $\boldsymbol{E}$ ∥ [001] and strain ⊥ [010]<br>• $\boldsymbol{E}$ ⊥ [010]
$Pc'c2'$ (27.80) $\mathbb{Z}_1$	• $\boldsymbol{E}$ ∥ [100] and $\boldsymbol{B}$ ∥ [001]
$P2_1$ (4.7) $\mathbb{Z}_1$	• $\boldsymbol{E}$ ∥ [100] and $\boldsymbol{B}$ ∥ [100] and strain ⊥ [100]
P_C2_1 (4.12) $\mathbb{Z}_1$	• $\boldsymbol{E}$ ∥ [100] and strain ⊥ [100]
$Pm'c'2_1$ (26.70) $\mathbb{Z}_1$	• $\boldsymbol{E}$ ∥ [100] and $\boldsymbol{B}$ ∥ [100]
P_Bca2_1 (29.108) $\mathbb{Z}_1$	• $\boldsymbol{E}$ ∥ [100]
Pm' (6.20) $\mathbb{Z}_1$	• $\boldsymbol{E}$ ∥ [010] and $\boldsymbol{B}$ ∥ [010] and strain ⊥ [100]<br>• $\boldsymbol{E}$ ∥ [010] and $\boldsymbol{B}$ ∥ [001] and strain ⊥ [100]<br>• $\boldsymbol{E}$ ∥ [010] and $\boldsymbol{B}$ ⊥ [100]<br>• $\boldsymbol{E}$ ∥ [001] and $\boldsymbol{B}$ ∥ [010] and strain ⊥ [100]<br>• $\boldsymbol{E}$ ∥ [001] and $\boldsymbol{B}$ ∥ [001] and strain ⊥ [100]<br>• $\boldsymbol{E}$ ∥ [001] and $\boldsymbol{B}$ ⊥ [100]<br>• $\boldsymbol{E}$ ⊥ [100] and $\boldsymbol{B}$ ∥ [010]<br>• $\boldsymbol{E}$ ⊥ [100] and $\boldsymbol{B}$ ∥ [001]<br>• $\boldsymbol{E}$ ⊥ [100] and $\boldsymbol{B}$ ⊥ [100]
$P2$ (3.1) $\mathbb{Z}_2$	• $\boldsymbol{E}$ ∥ [010] and $\boldsymbol{B}$ ∥ [010] and strain ⊥ [010]
$Pm'm'2$ (25.60) $\mathbb{Z}_2$	• $\boldsymbol{E}$ ∥ [010] and $\boldsymbol{B}$ ∥ [010]
$P2_1'$ (4.9) $\mathbb{Z}_1$	• $\boldsymbol{E}$ ∥ [001] and $\boldsymbol{B}$ ∥ [100] and strain ⊥ [001]<br>• $\boldsymbol{E}$ ∥ [001] and $\boldsymbol{B}$ ∥ [010] and strain ⊥ [001]<br>• $\boldsymbol{E}$ ∥ [001] and $\boldsymbol{B}$ ⊥ [001]
$Pm'c2_1'$ (26.68) $\mathbb{Z}_1$	• $\boldsymbol{E}$ ∥ [001] and $\boldsymbol{B}$ ∥ [010]

Continued on next page

...continued

Subgroup & SI group	Perturbation
$P\bar{1}$ (2.4) $\mathbb{Z}_2 \times \mathbb{Z}_2 \times \mathbb{Z}_2 \times \mathbb{Z}_4$	• $\boldsymbol{B} \parallel [100]$ and strain in generic direction<br>• $\boldsymbol{B} \parallel [010]$ and strain in generic direction<br>• $\boldsymbol{B} \parallel [001]$ and strain in generic direction<br>• $\boldsymbol{B} \perp [100]$ and strain $\perp [010]$<br>• $\boldsymbol{B} \perp [100]$ and strain $\perp [001]$<br>• $\boldsymbol{B} \perp [100]$ and strain in generic direction<br>• $\boldsymbol{B} \perp [010]$ and strain $\perp [100]$<br>• $\boldsymbol{B} \perp [010]$ and strain $\perp [001]$<br>• $\boldsymbol{B} \perp [010]$ and strain in generic direction<br>• $\boldsymbol{B} \perp [001]$ and strain $\perp [100]$<br>• $\boldsymbol{B} \perp [001]$ and strain $\perp [010]$<br>• $\boldsymbol{B} \perp [001]$ and strain in generic direction<br>• $\boldsymbol{B}$ in generic direction
$P2'/m'$ (10.46) $\mathbb{Z}_2 \times \mathbb{Z}_2 \times \mathbb{Z}_2 \times \mathbb{Z}_4$	• $\boldsymbol{B} \parallel [010]$ and strain $\perp [100]$<br>• $\boldsymbol{B} \parallel [001]$ and strain $\perp [100]$<br>• $\boldsymbol{B} \perp [100]$
$P2/c$ (13.65) $\mathbb{Z}_2 \times \mathbb{Z}_2$	• $\boldsymbol{B} \parallel [010]$ and strain $\perp [010]$
$P2_1'/m'$ (11.54) $\mathbb{Z}_2 \times \mathbb{Z}_2 \times \mathbb{Z}_4$	• $\boldsymbol{B} \parallel [100]$ and strain $\perp [001]$<br>• $\boldsymbol{B} \parallel [010]$ and strain $\perp [001]$<br>• $\boldsymbol{B} \perp [001]$
$Pm'm'a$ (51.294) $\mathbb{Z}_2 \times \mathbb{Z}_2$	• $\boldsymbol{B} \parallel [010]$
Pc (7.24) $\mathbb{Z}_1$	• $\boldsymbol{E} \parallel [010]$ and $\boldsymbol{B} \parallel [100]$ and strain $\perp [100]$<br>• $\boldsymbol{E} \parallel [001]$ and $\boldsymbol{B} \parallel [100]$ and strain $\perp [100]$<br>• $\boldsymbol{E} \perp [100]$ and $\boldsymbol{B} \parallel [100]$
P_Ac (7.31) $\mathbb{Z}_1$	• $\boldsymbol{E} \parallel [010]$ and strain $\perp [100]$<br>• $\boldsymbol{E} \parallel [001]$ and strain $\perp [100]$<br>• $\boldsymbol{E} \perp [100]$
$Pca'2_1'$ (29.102) $\mathbb{Z}_1$	• $\boldsymbol{E} \parallel [001]$ and $\boldsymbol{B} \parallel [100]$
$P2_1/c$ (14.75) $\mathbb{Z}_2$	• $\boldsymbol{B} \parallel [100]$ and strain $\perp [100]$
$P2'$ (3.3) $\mathbb{Z}_1$	• $\boldsymbol{E} \parallel [010]$ and $\boldsymbol{B} \parallel [100]$ and strain $\perp [010]$<br>• $\boldsymbol{E} \parallel [010]$ and $\boldsymbol{B} \parallel [001]$ and strain $\perp [010]$<br>• $\boldsymbol{E} \parallel [010]$ and $\boldsymbol{B} \perp [010]$
$Pm'a2'$ (28.89) $\mathbb{Z}_1$	• $\boldsymbol{E} \parallel [010]$ and $\boldsymbol{B} \parallel [001]$
P_a2 (3.4) $\mathbb{Z}_2$	• $\boldsymbol{E} \parallel [010]$ and strain $\perp [010]$

Continued on next page

...continued

Subgroup & SI group	Perturbation
$P2'/c'$ (13.69) $\mathbb{Z}_2 \times \mathbb{Z}_2 \times \mathbb{Z}_4$	• $\boldsymbol{B} \parallel [100]$ and strain $\perp [010]$<br>• $\boldsymbol{B} \parallel [001]$ and strain $\perp [010]$<br>• $\boldsymbol{B} \perp [010]$
$Pm'a2'$ (28.89) $\mathbb{Z}_1$	• $\boldsymbol{E} \parallel [010]$ and $\boldsymbol{B} \parallel [100]$
P_Cba2 (32.141) $\mathbb{Z}_1$	• $\boldsymbol{E} \parallel [010]$
$Pb'cm'$ (57.384) $\mathbb{Z}_2$	• $\boldsymbol{B} \parallel [100]$
$P2$ (3.1) $\mathbb{Z}_2$	• $\boldsymbol{E} \parallel [001]$ and $\boldsymbol{B} \parallel [001]$ and strain $\perp [001]$
$Pm'a'2$ (28.91) $\mathbb{Z}_2$	• $\boldsymbol{E} \parallel [001]$ and $\boldsymbol{B} \parallel [001]$
P_C2 (3.6) $\mathbb{Z}_1$	• $\boldsymbol{E} \parallel [001]$ and strain $\perp [001]$
$P2/c$ (13.65) $\mathbb{Z}_2 \times \mathbb{Z}_2$	• $\boldsymbol{B} \parallel [001]$ and strain $\perp [001]$
P_Acc2 (27.85) $\mathbb{Z}_2$	• $\boldsymbol{E} \parallel [001]$
$Pc'cm'$ (49.270) $\mathbb{Z}_2 \times \mathbb{Z}_2$	• $\boldsymbol{B} \parallel [001]$
$P_S\bar{1}$ (2.7) $\mathbb{Z}_2 \times \mathbb{Z}_2 \times \mathbb{Z}_4$	• strain in generic direction
P_A2_1/c (14.83) $\mathbb{Z}_2$	• strain $\perp [100]$
P_a2/c (13.70) $\mathbb{Z}_2 \times \mathbb{Z}_2$	• strain $\perp [010]$
P_A2/c (13.73) $\mathbb{Z}_2$	• strain $\perp [001]$

Table. S 461: Subgroups of P_Ccca (54.351) that can be realized by applying $\boldsymbol{E}/\boldsymbol{B}$ fields and/or uniaxial strains.

Subgroup & SI group	Perturbation
Pc' (7.26) $\mathbb{Z}_1$	• $\boldsymbol{E} \parallel [100]$ and $\boldsymbol{B} \parallel [100]$ and strain $\perp [001]$<br>• $\boldsymbol{E} \parallel [100]$ and $\boldsymbol{B} \parallel [010]$ and strain $\perp [001]$<br>• $\boldsymbol{E} \parallel [100]$ and $\boldsymbol{B} \perp [001]$<br>• $\boldsymbol{E} \parallel [010]$ and $\boldsymbol{B} \parallel [100]$ and strain $\perp [001]$<br>• $\boldsymbol{E} \parallel [010]$ and $\boldsymbol{B} \parallel [010]$ and strain $\perp [001]$<br>• $\boldsymbol{E} \parallel [010]$ and $\boldsymbol{B} \perp [001]$<br>• $\boldsymbol{E} \perp [001]$ and $\boldsymbol{B} \parallel [100]$<br>• $\boldsymbol{E} \perp [001]$ and $\boldsymbol{B} \parallel [010]$<br>• $\boldsymbol{E} \perp [001]$ and $\boldsymbol{B} \perp [001]$

Continued on next page

...continued

Subgroup & SI group	Perturbation
Pc (7.24) $\mathbb{Z}_1$	• $\boldsymbol{E} \parallel [100]$ and $\boldsymbol{B} \parallel [010]$ and strain $\perp [010]$<br>• $\boldsymbol{E} \parallel [001]$ and $\boldsymbol{B} \parallel [010]$ and strain $\perp [010]$<br>• $\boldsymbol{E} \perp [010]$ and $\boldsymbol{B} \parallel [010]$
$P2'$ (3.3) $\mathbb{Z}_1$	• $\boldsymbol{E} \parallel [100]$ and $\boldsymbol{B} \parallel [010]$ and strain $\perp [100]$<br>• $\boldsymbol{E} \parallel [100]$ and $\boldsymbol{B} \parallel [001]$ and strain $\perp [100]$<br>• $\boldsymbol{E} \parallel [100]$ and $\boldsymbol{B} \perp [100]$
$Pb'a2'$ (32.137) $\mathbb{Z}_1$	• $\boldsymbol{E} \parallel [100]$ and $\boldsymbol{B} \parallel [010]$
Pc (7.24) $\mathbb{Z}_1$	• $\boldsymbol{E} \parallel [100]$ and $\boldsymbol{B} \parallel [001]$ and strain $\perp [001]$<br>• $\boldsymbol{E} \parallel [010]$ and $\boldsymbol{B} \parallel [001]$ and strain $\perp [001]$<br>• $\boldsymbol{E} \perp [001]$ and $\boldsymbol{B} \parallel [001]$
P_S1 (1.3) $\mathbb{Z}_1$	• $\boldsymbol{E} \parallel [100]$ and strain in generic direction<br>• $\boldsymbol{E} \parallel [010]$ and strain in generic direction<br>• $\boldsymbol{E} \parallel [001]$ and strain in generic direction<br>• $\boldsymbol{E} \perp [100]$ and strain $\perp [010]$<br>• $\boldsymbol{E} \perp [100]$ and strain $\perp [001]$<br>• $\boldsymbol{E} \perp [100]$ and strain in generic direction<br>• $\boldsymbol{E} \perp [010]$ and strain $\perp [100]$<br>• $\boldsymbol{E} \perp [010]$ and strain $\perp [001]$<br>• $\boldsymbol{E} \perp [010]$ and strain in generic direction<br>• $\boldsymbol{E} \perp [001]$ and strain $\perp [100]$<br>• $\boldsymbol{E} \perp [001]$ and strain $\perp [010]$<br>• $\boldsymbol{E} \perp [001]$ and strain in generic direction<br>• $\boldsymbol{E}$ in generic direction
P_ac (7.27) $\mathbb{Z}_1$	• $\boldsymbol{E} \parallel [100]$ and strain $\perp [001]$<br>• $\boldsymbol{E} \parallel [010]$ and strain $\perp [001]$<br>• $\boldsymbol{E} \perp [001]$
$P2_1$ (4.7) $\mathbb{Z}_1$	• $\boldsymbol{E} \parallel [100]$ and $\boldsymbol{B} \parallel [100]$ and strain $\perp [100]$
P_C2_1 (4.12) $\mathbb{Z}_1$	• $\boldsymbol{E} \parallel [100]$ and strain $\perp [100]$
Pc' (7.26) $\mathbb{Z}_1$	• $\boldsymbol{E} \parallel [100]$ and $\boldsymbol{B} \parallel [100]$ and strain $\perp [010]$<br>• $\boldsymbol{E} \parallel [100]$ and $\boldsymbol{B} \parallel [001]$ and strain $\perp [010]$<br>• $\boldsymbol{E} \parallel [100]$ and $\boldsymbol{B} \perp [010]$<br>• $\boldsymbol{E} \parallel [001]$ and $\boldsymbol{B} \parallel [100]$ and strain $\perp [010]$<br>• $\boldsymbol{E} \parallel [001]$ and $\boldsymbol{B} \parallel [001]$ and strain $\perp [010]$<br>• $\boldsymbol{E} \parallel [001]$ and $\boldsymbol{B} \perp [010]$<br>• $\boldsymbol{E} \perp [010]$ and $\boldsymbol{B} \parallel [100]$<br>• $\boldsymbol{E} \perp [010]$ and $\boldsymbol{B} \parallel [001]$<br>• $\boldsymbol{E} \perp [010]$ and $\boldsymbol{B} \perp [010]$

Continued on next page

...continued

Subgroup & SI group	Perturbation
$Pn'c2'$ (30.113) $\mathbb{Z}_1$	• $\boldsymbol{E}$ ∥ [100] and $\boldsymbol{B}$ ∥ [001]
P_Cc (7.30) $\mathbb{Z}_1$	• $\boldsymbol{E}$ ∥ [100] and strain ⊥ [010]<br>• $\boldsymbol{E}$ ∥ [001] and strain ⊥ [010]<br>• $\boldsymbol{E}$ ⊥ [010]
$Pn'a'2_1$ (33.148) $\mathbb{Z}_1$	• $\boldsymbol{E}$ ∥ [100] and $\boldsymbol{B}$ ∥ [100]
P_Aca2_1 (29.107) $\mathbb{Z}_1$	• $\boldsymbol{E}$ ∥ [100]
$P2$ (3.1) $\mathbb{Z}_2$	• $\boldsymbol{E}$ ∥ [010] and $\boldsymbol{B}$ ∥ [010] and strain ⊥ [010]
Pc' (7.26) $\mathbb{Z}_1$	• $\boldsymbol{E}$ ∥ [010] and $\boldsymbol{B}$ ∥ [010] and strain ⊥ [100]<br>• $\boldsymbol{E}$ ∥ [010] and $\boldsymbol{B}$ ∥ [001] and strain ⊥ [100]<br>• $\boldsymbol{E}$ ∥ [010] and $\boldsymbol{B}$ ⊥ [100]<br>• $\boldsymbol{E}$ ∥ [001] and $\boldsymbol{B}$ ∥ [010] and strain ⊥ [100]<br>• $\boldsymbol{E}$ ∥ [001] and $\boldsymbol{B}$ ∥ [001] and strain ⊥ [100]<br>• $\boldsymbol{E}$ ∥ [001] and $\boldsymbol{B}$ ⊥ [100]<br>• $\boldsymbol{E}$ ⊥ [100] and $\boldsymbol{B}$ ∥ [010]<br>• $\boldsymbol{E}$ ⊥ [100] and $\boldsymbol{B}$ ∥ [001]<br>• $\boldsymbol{E}$ ⊥ [100] and $\boldsymbol{B}$ ⊥ [100]
$Pn'c'2$ (30.115) $\mathbb{Z}_1$	• $\boldsymbol{E}$ ∥ [010] and $\boldsymbol{B}$ ∥ [010]
$P\bar{1}$ (2.4) $\mathbb{Z}_2 \times \mathbb{Z}_2 \times \mathbb{Z}_2 \times \mathbb{Z}_4$	• $\boldsymbol{B}$ ∥ [100] and strain in generic direction<br>• $\boldsymbol{B}$ ∥ [010] and strain in generic direction<br>• $\boldsymbol{B}$ ∥ [001] and strain in generic direction<br>• $\boldsymbol{B}$ ⊥ [100] and strain ⊥ [010]<br>• $\boldsymbol{B}$ ⊥ [100] and strain ⊥ [001]<br>• $\boldsymbol{B}$ ⊥ [100] and strain in generic direction<br>• $\boldsymbol{B}$ ⊥ [010] and strain ⊥ [100]<br>• $\boldsymbol{B}$ ⊥ [010] and strain ⊥ [001]<br>• $\boldsymbol{B}$ ⊥ [010] and strain in generic direction<br>• $\boldsymbol{B}$ ⊥ [001] and strain ⊥ [100]<br>• $\boldsymbol{B}$ ⊥ [001] and strain ⊥ [010]<br>• $\boldsymbol{B}$ ⊥ [001] and strain in generic direction<br>• $\boldsymbol{B}$ in generic direction
$P2/c$ (13.65) $\mathbb{Z}_2 \times \mathbb{Z}_2$	• $\boldsymbol{B}$ ∥ [010] and strain ⊥ [010]
$P2'/c'$ (13.69) $\mathbb{Z}_2 \times \mathbb{Z}_2 \times \mathbb{Z}_4$	• $\boldsymbol{B}$ ∥ [010] and strain ⊥ [100]<br>• $\boldsymbol{B}$ ∥ [001] and strain ⊥ [100]<br>• $\boldsymbol{B}$ ⊥ [100]

Continued on next page

...continued

Subgroup & SI group	Perturbation
$P2'$ (3.3) $\mathbb{Z}_1$	• $\boldsymbol{E}$ ∥ [001] and $\boldsymbol{B}$ ∥ [100] and strain ⊥ [001]<br>• $\boldsymbol{E}$ ∥ [001] and $\boldsymbol{B}$ ∥ [010] and strain ⊥ [001]<br>• $\boldsymbol{E}$ ∥ [001] and $\boldsymbol{B}$ ⊥ [001]
$Pn'c2'$ (30.113) $\mathbb{Z}_1$	• $\boldsymbol{E}$ ∥ [001] and $\boldsymbol{B}$ ∥ [010]
$P2'/c'$ (13.69) $\mathbb{Z}_2 \times \mathbb{Z}_2 \times \mathbb{Z}_4$	• $\boldsymbol{B}$ ∥ [100] and strain ⊥ [001]<br>• $\boldsymbol{B}$ ∥ [010] and strain ⊥ [001]<br>• $\boldsymbol{B}$ ⊥ [001]
$Pb'an'$ (50.282) $\mathbb{Z}_2$	• $\boldsymbol{B}$ ∥ [010]
Pc (7.24) $\mathbb{Z}_1$	• $\boldsymbol{E}$ ∥ [010] and $\boldsymbol{B}$ ∥ [100] and strain ⊥ [100]<br>• $\boldsymbol{E}$ ∥ [001] and $\boldsymbol{B}$ ∥ [100] and strain ⊥ [100]<br>• $\boldsymbol{E}$ ⊥ [100] and $\boldsymbol{B}$ ∥ [100]
P_Cc (7.30) $\mathbb{Z}_1$	• $\boldsymbol{E}$ ∥ [010] and strain ⊥ [100]<br>• $\boldsymbol{E}$ ∥ [001] and strain ⊥ [100]<br>• $\boldsymbol{E}$ ⊥ [100]
$P2_1/c$ (14.75) $\mathbb{Z}_2$	• $\boldsymbol{B}$ ∥ [100] and strain ⊥ [100]
$Pn'c2'$ (30.113) $\mathbb{Z}_1$	• $\boldsymbol{E}$ ∥ [001] and $\boldsymbol{B}$ ∥ [100]
$P2_1'$ (4.9) $\mathbb{Z}_1$	• $\boldsymbol{E}$ ∥ [010] and $\boldsymbol{B}$ ∥ [100] and strain ⊥ [010]<br>• $\boldsymbol{E}$ ∥ [010] and $\boldsymbol{B}$ ∥ [001] and strain ⊥ [010]<br>• $\boldsymbol{E}$ ∥ [010] and $\boldsymbol{B}$ ⊥ [010]
P_C2 (3.6) $\mathbb{Z}_1$	• $\boldsymbol{E}$ ∥ [010] and strain ⊥ [010]
$Pn'a2_1'$ (33.146) $\mathbb{Z}_1$	• $\boldsymbol{E}$ ∥ [010] and $\boldsymbol{B}$ ∥ [001]
$P2_1'/c'$ (14.79) $\mathbb{Z}_2 \times \mathbb{Z}_4$	• $\boldsymbol{B}$ ∥ [100] and strain ⊥ [010]<br>• $\boldsymbol{B}$ ∥ [001] and strain ⊥ [010]<br>• $\boldsymbol{B}$ ⊥ [010]
$Pc'a2_1'$ (29.101) $\mathbb{Z}_1$	• $\boldsymbol{E}$ ∥ [010] and $\boldsymbol{B}$ ∥ [100]
P_Aba2 (32.142) $\mathbb{Z}_1$	• $\boldsymbol{E}$ ∥ [010]
$Pbc'n'$ (60.423) $\mathbb{Z}_2$	• $\boldsymbol{B}$ ∥ [100]
$P2$ (3.1) $\mathbb{Z}_2$	• $\boldsymbol{E}$ ∥ [001] and $\boldsymbol{B}$ ∥ [001] and strain ⊥ [001]
$Pn'n'2$ (34.159) $\mathbb{Z}_1$	• $\boldsymbol{E}$ ∥ [001] and $\boldsymbol{B}$ ∥ [001]
$P2/c$ (13.65) $\mathbb{Z}_2 \times \mathbb{Z}_2$	• $\boldsymbol{B}$ ∥ [001] and strain ⊥ [001]
P_a2 (3.4) $\mathbb{Z}_2$	• $\boldsymbol{E}$ ∥ [001] and strain ⊥ [001]
P_Ccc2 (27.84) $\mathbb{Z}_2$	• $\boldsymbol{E}$ ∥ [001]

Continued on next page

...continued

Subgroup & SI group	Perturbation
$Pn'n'a$ (52.310) $\mathbb{Z}_2$	• $\boldsymbol{B}$ ∥ [001]
$P_S\bar{1}$ (2.7) $\mathbb{Z}_2\times\mathbb{Z}_2\times\mathbb{Z}_4$	• strain in generic direction
P_C2_1/c (14.84) $\mathbb{Z}_2$	• strain ⊥ [100]
P_C2/c (13.74) $\mathbb{Z}_2$	• strain ⊥ [010]
P_a2/c (13.70) $\mathbb{Z}_2\times\mathbb{Z}_2$	• strain ⊥ [001]

Table. S 462: Subgroups of P_Icca (54.352) that can be realized by applying $\boldsymbol{E}/\boldsymbol{B}$ fields and/or uniaxial strains.

Subgroup & SI group	Perturbation
Pc' (7.26) $\mathbb{Z}_1$	• $\boldsymbol{E}$ ∥ [100] and $\boldsymbol{B}$ ∥ [100] and strain ⊥ [001]<br>• $\boldsymbol{E}$ ∥ [100] and $\boldsymbol{B}$ ∥ [010] and strain ⊥ [001]<br>• $\boldsymbol{E}$ ∥ [100] and $\boldsymbol{B}$ ⊥ [001]<br>• $\boldsymbol{E}$ ∥ [010] and $\boldsymbol{B}$ ∥ [100] and strain ⊥ [001]<br>• $\boldsymbol{E}$ ∥ [010] and $\boldsymbol{B}$ ∥ [010] and strain ⊥ [001]<br>• $\boldsymbol{E}$ ∥ [010] and $\boldsymbol{B}$ ⊥ [001]<br>• $\boldsymbol{E}$ ⊥ [001] and $\boldsymbol{B}$ ∥ [100]<br>• $\boldsymbol{E}$ ⊥ [001] and $\boldsymbol{B}$ ∥ [010]<br>• $\boldsymbol{E}$ ⊥ [001] and $\boldsymbol{B}$ ⊥ [001]
Pc (7.24) $\mathbb{Z}_1$	• $\boldsymbol{E}$ ∥ [100] and $\boldsymbol{B}$ ∥ [010] and strain ⊥ [010]<br>• $\boldsymbol{E}$ ∥ [001] and $\boldsymbol{B}$ ∥ [010] and strain ⊥ [010]<br>• $\boldsymbol{E}$ ⊥ [010] and $\boldsymbol{B}$ ∥ [010]
$P2'$ (3.3) $\mathbb{Z}_1$	• $\boldsymbol{E}$ ∥ [100] and $\boldsymbol{B}$ ∥ [010] and strain ⊥ [100]<br>• $\boldsymbol{E}$ ∥ [100] and $\boldsymbol{B}$ ∥ [001] and strain ⊥ [100]<br>• $\boldsymbol{E}$ ∥ [100] and $\boldsymbol{B}$ ⊥ [100]
$Pb'a2'$ (32.137) $\mathbb{Z}_1$	• $\boldsymbol{E}$ ∥ [100] and $\boldsymbol{B}$ ∥ [010]
Pc (7.24) $\mathbb{Z}_1$	• $\boldsymbol{E}$ ∥ [100] and $\boldsymbol{B}$ ∥ [001] and strain ⊥ [001]<br>• $\boldsymbol{E}$ ∥ [010] and $\boldsymbol{B}$ ∥ [001] and strain ⊥ [001]<br>• $\boldsymbol{E}$ ⊥ [001] and $\boldsymbol{B}$ ∥ [001]

Continued on next page

...continued

Subgroup & SI group	Perturbation
P_S1 (1.3) $\mathbb{Z}_1$	• $\boldsymbol{E}$ ∥ [100] and strain in generic direction<br>• $\boldsymbol{E}$ ∥ [010] and strain in generic direction<br>• $\boldsymbol{E}$ ∥ [001] and strain in generic direction<br>• $\boldsymbol{E}$ ⊥ [100] and strain ⊥ [010]<br>• $\boldsymbol{E}$ ⊥ [100] and strain ⊥ [001]<br>• $\boldsymbol{E}$ ⊥ [100] and strain in generic direction<br>• $\boldsymbol{E}$ ⊥ [010] and strain ⊥ [100]<br>• $\boldsymbol{E}$ ⊥ [010] and strain ⊥ [001]<br>• $\boldsymbol{E}$ ⊥ [010] and strain in generic direction<br>• $\boldsymbol{E}$ ⊥ [001] and strain ⊥ [100]<br>• $\boldsymbol{E}$ ⊥ [001] and strain ⊥ [010]<br>• $\boldsymbol{E}$ ⊥ [001] and strain in generic direction<br>• $\boldsymbol{E}$ in generic direction
P_Cc (7.30) $\mathbb{Z}_1$	• $\boldsymbol{E}$ ∥ [100] and strain ⊥ [001]<br>• $\boldsymbol{E}$ ∥ [010] and strain ⊥ [001]<br>• $\boldsymbol{E}$ ⊥ [001]
$P2_1$ (4.7) $\mathbb{Z}_1$	• $\boldsymbol{E}$ ∥ [100] and $\boldsymbol{B}$ ∥ [100] and strain ⊥ [100]
P_C2_1 (4.12) $\mathbb{Z}_1$	• $\boldsymbol{E}$ ∥ [100] and strain ⊥ [100]
Pc' (7.26) $\mathbb{Z}_1$	• $\boldsymbol{E}$ ∥ [100] and $\boldsymbol{B}$ ∥ [100] and strain ⊥ [010]<br>• $\boldsymbol{E}$ ∥ [100] and $\boldsymbol{B}$ ∥ [001] and strain ⊥ [010]<br>• $\boldsymbol{E}$ ∥ [100] and $\boldsymbol{B}$ ⊥ [010]<br>• $\boldsymbol{E}$ ∥ [001] and $\boldsymbol{B}$ ∥ [100] and strain ⊥ [010]<br>• $\boldsymbol{E}$ ∥ [001] and $\boldsymbol{B}$ ∥ [001] and strain ⊥ [010]<br>• $\boldsymbol{E}$ ∥ [001] and $\boldsymbol{B}$ ⊥ [010]<br>• $\boldsymbol{E}$ ⊥ [010] and $\boldsymbol{B}$ ∥ [100]<br>• $\boldsymbol{E}$ ⊥ [010] and $\boldsymbol{B}$ ∥ [001]<br>• $\boldsymbol{E}$ ⊥ [010] and $\boldsymbol{B}$ ⊥ [010]
$Pc'c2'$ (27.80) $\mathbb{Z}_1$	• $\boldsymbol{E}$ ∥ [100] and $\boldsymbol{B}$ ∥ [001]
P_Cc (7.30) $\mathbb{Z}_1$	• $\boldsymbol{E}$ ∥ [100] and strain ⊥ [010]<br>• $\boldsymbol{E}$ ∥ [001] and strain ⊥ [010]<br>• $\boldsymbol{E}$ ⊥ [010]
$Pc'a'2_1$ (29.103) $\mathbb{Z}_1$	• $\boldsymbol{E}$ ∥ [100] and $\boldsymbol{B}$ ∥ [100]
P_Ica2_1 (29.110) $\mathbb{Z}_1$	• $\boldsymbol{E}$ ∥ [100]
$P2$ (3.1) $\mathbb{Z}_2$	• $\boldsymbol{E}$ ∥ [010] and $\boldsymbol{B}$ ∥ [010] and strain ⊥ [010]

Continued on next page

...continued

Subgroup & SI group	Perturbation
Pc' (7.26) $\mathbb{Z}_1$	• $\boldsymbol{E}$ $\parallel$ [010] and $\boldsymbol{B}$ $\parallel$ [010] and strain $\perp$ [100]<br>• $\boldsymbol{E}$ $\parallel$ [010] and $\boldsymbol{B}$ $\parallel$ [001] and strain $\perp$ [100]<br>• $\boldsymbol{E}$ $\parallel$ [010] and $\boldsymbol{B}$ $\perp$ [100]<br>• $\boldsymbol{E}$ $\parallel$ [001] and $\boldsymbol{B}$ $\parallel$ [010] and strain $\perp$ [100]<br>• $\boldsymbol{E}$ $\parallel$ [001] and $\boldsymbol{B}$ $\parallel$ [001] and strain $\perp$ [100]<br>• $\boldsymbol{E}$ $\parallel$ [001] and $\boldsymbol{B}$ $\perp$ [100]<br>• $\boldsymbol{E}$ $\perp$ [100] and $\boldsymbol{B}$ $\parallel$ [010]<br>• $\boldsymbol{E}$ $\perp$ [100] and $\boldsymbol{B}$ $\parallel$ [001]<br>• $\boldsymbol{E}$ $\perp$ [100] and $\boldsymbol{B}$ $\perp$ [100]
$Pc'c'2$ (27.81) $\mathbb{Z}_2$	• $\boldsymbol{E}$ $\parallel$ [010] and $\boldsymbol{B}$ $\parallel$ [010]
$P\bar{1}$ (2.4) $\mathbb{Z}_2 \times \mathbb{Z}_2 \times \mathbb{Z}_2 \times \mathbb{Z}_4$	• $\boldsymbol{B}$ $\parallel$ [100] and strain in generic direction<br>• $\boldsymbol{B}$ $\parallel$ [010] and strain in generic direction<br>• $\boldsymbol{B}$ $\parallel$ [001] and strain in generic direction<br>• $\boldsymbol{B}$ $\perp$ [100] and strain $\perp$ [010]<br>• $\boldsymbol{B}$ $\perp$ [100] and strain $\perp$ [001]<br>• $\boldsymbol{B}$ $\perp$ [100] and strain in generic direction<br>• $\boldsymbol{B}$ $\perp$ [010] and strain $\perp$ [100]<br>• $\boldsymbol{B}$ $\perp$ [010] and strain $\perp$ [001]<br>• $\boldsymbol{B}$ $\perp$ [010] and strain in generic direction<br>• $\boldsymbol{B}$ $\perp$ [001] and strain $\perp$ [100]<br>• $\boldsymbol{B}$ $\perp$ [001] and strain $\perp$ [010]<br>• $\boldsymbol{B}$ $\perp$ [001] and strain in generic direction<br>• $\boldsymbol{B}$ in generic direction
$P2/c$ (13.65) $\mathbb{Z}_2 \times \mathbb{Z}_2$	• $\boldsymbol{B}$ $\parallel$ [010] and strain $\perp$ [010]
$P2'/c'$ (13.69) $\mathbb{Z}_2 \times \mathbb{Z}_2 \times \mathbb{Z}_4$	• $\boldsymbol{B}$ $\parallel$ [010] and strain $\perp$ [100]<br>• $\boldsymbol{B}$ $\parallel$ [001] and strain $\perp$ [100]<br>• $\boldsymbol{B}$ $\perp$ [100]
$P2_1'$ (4.9) $\mathbb{Z}_1$	• $\boldsymbol{E}$ $\parallel$ [001] and $\boldsymbol{B}$ $\parallel$ [100] and strain $\perp$ [001]<br>• $\boldsymbol{E}$ $\parallel$ [001] and $\boldsymbol{B}$ $\parallel$ [010] and strain $\perp$ [001]<br>• $\boldsymbol{E}$ $\parallel$ [001] and $\boldsymbol{B}$ $\perp$ [001]
$Pca'2_1'$ (29.102) $\mathbb{Z}_1$	• $\boldsymbol{E}$ $\parallel$ [001] and $\boldsymbol{B}$ $\parallel$ [010]
$P2_1'/c'$ (14.79) $\mathbb{Z}_2 \times \mathbb{Z}_4$	• $\boldsymbol{B}$ $\parallel$ [100] and strain $\perp$ [001]<br>• $\boldsymbol{B}$ $\parallel$ [010] and strain $\perp$ [001]<br>• $\boldsymbol{B}$ $\perp$ [001]
$Pc'c'a$ (54.342) $\mathbb{Z}_2 \times \mathbb{Z}_2$	• $\boldsymbol{B}$ $\parallel$ [010]

Continued on next page

...continued

Subgroup & SI group	Perturbation
Pc (7.24) $\mathbb{Z}_1$	• $\boldsymbol{E} \parallel$ [010] and $\boldsymbol{B} \parallel$ [100] and strain $\perp$ [100]<br>• $\boldsymbol{E} \parallel$ [001] and $\boldsymbol{B} \parallel$ [100] and strain $\perp$ [100]<br>• $\boldsymbol{E} \perp$ [100] and $\boldsymbol{B} \parallel$ [100]
P_Cc (7.30) $\mathbb{Z}_1$	• $\boldsymbol{E} \parallel$ [010] and strain $\perp$ [100]<br>• $\boldsymbol{E} \parallel$ [001] and strain $\perp$ [100]<br>• $\boldsymbol{E} \perp$ [100]
$P2_1/c$ (14.75) $\mathbb{Z}_2$	• $\boldsymbol{B} \parallel$ [100] and strain $\perp$ [100]
$Pca'2_1'$ (29.102) $\mathbb{Z}_1$	• $\boldsymbol{E} \parallel$ [001] and $\boldsymbol{B} \parallel$ [100]
$P2_1'$ (4.9) $\mathbb{Z}_1$	• $\boldsymbol{E} \parallel$ [010] and $\boldsymbol{B} \parallel$ [100] and strain $\perp$ [010]<br>• $\boldsymbol{E} \parallel$ [010] and $\boldsymbol{B} \parallel$ [001] and strain $\perp$ [010]<br>• $\boldsymbol{E} \parallel$ [010] and $\boldsymbol{B} \perp$ [010]
P_C2 (3.6) $\mathbb{Z}_1$	• $\boldsymbol{E} \parallel$ [010] and strain $\perp$ [010]
$Pc'a2_1'$ (29.101) $\mathbb{Z}_1$	• $\boldsymbol{E} \parallel$ [010] and $\boldsymbol{B} \parallel$ [001]
$P2_1'/c'$ (14.79) $\mathbb{Z}_2 \times \mathbb{Z}_4$	• $\boldsymbol{B} \parallel$ [100] and strain $\perp$ [010]<br>• $\boldsymbol{B} \parallel$ [001] and strain $\perp$ [010]<br>• $\boldsymbol{B} \perp$ [010]
$Pc'a2_1'$ (29.101) $\mathbb{Z}_1$	• $\boldsymbol{E} \parallel$ [010] and $\boldsymbol{B} \parallel$ [100]
P_Iba2 (32.143) $\mathbb{Z}_1$	• $\boldsymbol{E} \parallel$ [010]
$Pb'c'a$ (61.436) $\mathbb{Z}_2$	• $\boldsymbol{B} \parallel$ [100]
$P2$ (3.1) $\mathbb{Z}_2$	• $\boldsymbol{E} \parallel$ [001] and $\boldsymbol{B} \parallel$ [001] and strain $\perp$ [001]
$Pb'a'2$ (32.138) $\mathbb{Z}_2$	• $\boldsymbol{E} \parallel$ [001] and $\boldsymbol{B} \parallel$ [001]
$P2/c$ (13.65) $\mathbb{Z}_2 \times \mathbb{Z}_2$	• $\boldsymbol{B} \parallel$ [001] and strain $\perp$ [001]
P_C2 (3.6) $\mathbb{Z}_1$	• $\boldsymbol{E} \parallel$ [001] and strain $\perp$ [001]
P_Icc2 (27.86) $\mathbb{Z}_2$	• $\boldsymbol{E} \parallel$ [001]
$Pc'ca'$ (54.344) $\mathbb{Z}_2 \times \mathbb{Z}_2$	• $\boldsymbol{B} \parallel$ [001]
$P_S\bar{1}$ (2.7) $\mathbb{Z}_2 \times \mathbb{Z}_2 \times \mathbb{Z}_4$	• strain in generic direction
P_C2_1/c (14.84) $\mathbb{Z}_2$	• strain $\perp$ [100]
P_C2/c (13.74) $\mathbb{Z}_2$	• strain $\perp$ [010]
P_C2/c (13.74) $\mathbb{Z}_2$	• strain $\perp$ [001]

Table. S 463: Subgroups of $Pbam$ (55.353) that can be realized by applying $\boldsymbol{E}/\boldsymbol{B}$ fields and/or uniaxial strains.

Subgroup & SI group	Perturbation
Pm (6.18) $\mathbb{Z}_1$	• $\boldsymbol{E} \parallel [100]$ and strain $\perp [001]$<br>• $\boldsymbol{E} \parallel [100]$ and $\boldsymbol{B} \parallel [001]$<br>• $\boldsymbol{E} \parallel [010]$ and strain $\perp [001]$<br>• $\boldsymbol{E} \parallel [010]$ and $\boldsymbol{B} \parallel [001]$<br>• $\boldsymbol{E} \perp [001]$
Pc (7.24) $\mathbb{Z}_1$	• $\boldsymbol{E} \parallel [100]$ and strain $\perp [010]$<br>• $\boldsymbol{E} \parallel [100]$ and $\boldsymbol{B} \parallel [010]$<br>• $\boldsymbol{E} \parallel [001]$ and strain $\perp [010]$<br>• $\boldsymbol{E} \parallel [001]$ and $\boldsymbol{B} \parallel [010]$<br>• $\boldsymbol{E} \perp [010]$
$P2_1$ (4.7) $\mathbb{Z}_1$	• $\boldsymbol{E} \parallel [100]$ and strain $\perp [100]$<br>• $\boldsymbol{E} \parallel [100]$ and $\boldsymbol{B} \parallel [100]$
$Pmc2_1$ (26.66) $\mathbb{Z}_1$	• $\boldsymbol{E} \parallel [100]$
$P2$ (3.1) $\mathbb{Z}_2$	• $\boldsymbol{E} \parallel [001]$ and strain $\perp [001]$<br>• $\boldsymbol{E} \parallel [001]$ and $\boldsymbol{B} \parallel [001]$
$P\bar{1}$ (2.4) $\mathbb{Z}_2 \times \mathbb{Z}_2 \times \mathbb{Z}_2 \times \mathbb{Z}_4$	• strain in generic direction<br>• $\boldsymbol{B} \parallel [100]$ and strain $\perp [010]$<br>• $\boldsymbol{B} \parallel [100]$ and strain $\perp [001]$<br>• $\boldsymbol{B} \parallel [010]$ and strain $\perp [100]$<br>• $\boldsymbol{B} \parallel [010]$ and strain $\perp [001]$<br>• $\boldsymbol{B} \parallel [001]$ and strain $\perp [100]$<br>• $\boldsymbol{B} \parallel [001]$ and strain $\perp [010]$<br>• $\boldsymbol{B}$ in generic direction
$P2/m$ (10.42) $\mathbb{Z}_2 \times \mathbb{Z}_2 \times \mathbb{Z}_2$	• strain $\perp [001]$<br>• $\boldsymbol{B} \parallel [001]$
Pc (7.24) $\mathbb{Z}_1$	• $\boldsymbol{E} \parallel [010]$ and strain $\perp [100]$<br>• $\boldsymbol{E} \parallel [010]$ and $\boldsymbol{B} \parallel [100]$<br>• $\boldsymbol{E} \parallel [001]$ and strain $\perp [100]$<br>• $\boldsymbol{E} \parallel [001]$ and $\boldsymbol{B} \parallel [100]$<br>• $\boldsymbol{E} \perp [100]$
$Pba2$ (32.135) $\mathbb{Z}_1$	• $\boldsymbol{E} \parallel [001]$
$P2_1/c$ (14.75) $\mathbb{Z}_2$	• strain $\perp [100]$<br>• $\boldsymbol{B} \parallel [100]$
$P2_1$ (4.7) $\mathbb{Z}_1$	• $\boldsymbol{E} \parallel [010]$ and strain $\perp [010]$<br>• $\boldsymbol{E} \parallel [010]$ and $\boldsymbol{B} \parallel [010]$

Continued on next page

...continued

Subgroup & SI group	Perturbation
$P2_1/c$ (14.75) $\mathbb{Z}_2$	• strain ⊥ [010]<br>• $\boldsymbol{B}$ ∥ [010]
$Pmc2_1$ (26.66) $\mathbb{Z}_1$	• $\boldsymbol{E}$ ∥ [010]

Table. S 464: Subgroups of $Pbam1'$ (55.354) that can be realized by applying $\boldsymbol{E}/\boldsymbol{B}$ fields and/or uniaxial strains.

Subgroup & SI group	Perturbation
$P11'$ (1.2) $\mathbb{Z}_1$	• $\boldsymbol{E}$ ∥ [100] and strain in generic direction<br>• $\boldsymbol{E}$ ∥ [010] and strain in generic direction<br>• $\boldsymbol{E}$ ∥ [001] and strain in generic direction<br>• $\boldsymbol{E}$ ⊥ [100] and strain ⊥ [010]<br>• $\boldsymbol{E}$ ⊥ [100] and strain ⊥ [001]<br>• $\boldsymbol{E}$ ⊥ [100] and strain in generic direction<br>• $\boldsymbol{E}$ ⊥ [010] and strain ⊥ [100]<br>• $\boldsymbol{E}$ ⊥ [010] and strain ⊥ [001]<br>• $\boldsymbol{E}$ ⊥ [010] and strain in generic direction<br>• $\boldsymbol{E}$ ⊥ [001] and strain ⊥ [100]<br>• $\boldsymbol{E}$ ⊥ [001] and strain ⊥ [010]<br>• $\boldsymbol{E}$ ⊥ [001] and strain in generic direction<br>• $\boldsymbol{E}$ in generic direction
Pm' (6.20) $\mathbb{Z}_1$	• $\boldsymbol{E}$ ∥ [100] and $\boldsymbol{B}$ ∥ [100] and strain ⊥ [001]<br>• $\boldsymbol{E}$ ∥ [100] and $\boldsymbol{B}$ ∥ [010] and strain ⊥ [001]<br>• $\boldsymbol{E}$ ∥ [100] and $\boldsymbol{B}$ ⊥ [001]<br>• $\boldsymbol{E}$ ∥ [010] and $\boldsymbol{B}$ ∥ [100] and strain ⊥ [001]<br>• $\boldsymbol{E}$ ∥ [010] and $\boldsymbol{B}$ ∥ [010] and strain ⊥ [001]<br>• $\boldsymbol{E}$ ∥ [010] and $\boldsymbol{B}$ ⊥ [001]<br>• $\boldsymbol{E}$ ⊥ [001] and $\boldsymbol{B}$ ∥ [100]<br>• $\boldsymbol{E}$ ⊥ [001] and $\boldsymbol{B}$ ∥ [010]<br>• $\boldsymbol{E}$ ⊥ [001] and $\boldsymbol{B}$ ⊥ [001]
Pm (6.18) $\mathbb{Z}_1$	• $\boldsymbol{E}$ ∥ [100] and $\boldsymbol{B}$ ∥ [001] and strain ⊥ [001]<br>• $\boldsymbol{E}$ ∥ [010] and $\boldsymbol{B}$ ∥ [001] and strain ⊥ [001]<br>• $\boldsymbol{E}$ ⊥ [001] and $\boldsymbol{B}$ ∥ [001]
$Pm1'$ (6.19) $\mathbb{Z}_1$	• $\boldsymbol{E}$ ∥ [100] and strain ⊥ [001]<br>• $\boldsymbol{E}$ ∥ [010] and strain ⊥ [001]<br>• $\boldsymbol{E}$ ⊥ [001]

Continued on next page

...continued

Subgroup & SI group	Perturbation
Pc' (7.26) $\mathbb{Z}_1$	• $\boldsymbol{E}$ $\parallel$ [100] and $\boldsymbol{B}$ $\parallel$ [100] and strain $\perp$ [010]<br>• $\boldsymbol{E}$ $\parallel$ [100] and $\boldsymbol{B}$ $\parallel$ [001] and strain $\perp$ [010]<br>• $\boldsymbol{E}$ $\parallel$ [100] and $\boldsymbol{B}$ $\perp$ [010]<br>• $\boldsymbol{E}$ $\parallel$ [001] and $\boldsymbol{B}$ $\parallel$ [100] and strain $\perp$ [010]<br>• $\boldsymbol{E}$ $\parallel$ [001] and $\boldsymbol{B}$ $\parallel$ [001] and strain $\perp$ [010]<br>• $\boldsymbol{E}$ $\parallel$ [001] and $\boldsymbol{B}$ $\perp$ [010]<br>• $\boldsymbol{E}$ $\perp$ [010] and $\boldsymbol{B}$ $\parallel$ [100]<br>• $\boldsymbol{E}$ $\perp$ [010] and $\boldsymbol{B}$ $\parallel$ [001]<br>• $\boldsymbol{E}$ $\perp$ [010] and $\boldsymbol{B}$ $\perp$ [010]
Pc (7.24) $\mathbb{Z}_1$	• $\boldsymbol{E}$ $\parallel$ [100] and $\boldsymbol{B}$ $\parallel$ [010] and strain $\perp$ [010]<br>• $\boldsymbol{E}$ $\parallel$ [001] and $\boldsymbol{B}$ $\parallel$ [010] and strain $\perp$ [010]<br>• $\boldsymbol{E}$ $\perp$ [010] and $\boldsymbol{B}$ $\parallel$ [010]
$Pc1'$ (7.25) $\mathbb{Z}_1$	• $\boldsymbol{E}$ $\parallel$ [100] and strain $\perp$ [010]<br>• $\boldsymbol{E}$ $\parallel$ [001] and strain $\perp$ [010]<br>• $\boldsymbol{E}$ $\perp$ [010]
$P2_1'$ (4.9) $\mathbb{Z}_1$	• $\boldsymbol{E}$ $\parallel$ [100] and $\boldsymbol{B}$ $\parallel$ [010] and strain $\perp$ [100]<br>• $\boldsymbol{E}$ $\parallel$ [100] and $\boldsymbol{B}$ $\parallel$ [001] and strain $\perp$ [100]<br>• $\boldsymbol{E}$ $\parallel$ [100] and $\boldsymbol{B}$ $\perp$ [100]
$Pmc'2_1'$ (26.69) $\mathbb{Z}_1$	• $\boldsymbol{E}$ $\parallel$ [100] and $\boldsymbol{B}$ $\parallel$ [001]
$Pm'c2_1'$ (26.68) $\mathbb{Z}_1$	• $\boldsymbol{E}$ $\parallel$ [100] and $\boldsymbol{B}$ $\parallel$ [010]
$P2_1$ (4.7) $\mathbb{Z}_1$	• $\boldsymbol{E}$ $\parallel$ [100] and $\boldsymbol{B}$ $\parallel$ [100] and strain $\perp$ [100]
$Pm'c'2_1$ (26.70) $\mathbb{Z}_1$	• $\boldsymbol{E}$ $\parallel$ [100] and $\boldsymbol{B}$ $\parallel$ [100]
$P2_11'$ (4.8) $\mathbb{Z}_1$	• $\boldsymbol{E}$ $\parallel$ [100] and strain $\perp$ [100]
$Pmc2_11'$ (26.67) $\mathbb{Z}_1$	• $\boldsymbol{E}$ $\parallel$ [100]
$P2'$ (3.3) $\mathbb{Z}_1$	• $\boldsymbol{E}$ $\parallel$ [001] and $\boldsymbol{B}$ $\parallel$ [100] and strain $\perp$ [001]<br>• $\boldsymbol{E}$ $\parallel$ [001] and $\boldsymbol{B}$ $\parallel$ [010] and strain $\perp$ [001]<br>• $\boldsymbol{E}$ $\parallel$ [001] and $\boldsymbol{B}$ $\perp$ [001]
$P2$ (3.1) $\mathbb{Z}_2$	• $\boldsymbol{E}$ $\parallel$ [001] and $\boldsymbol{B}$ $\parallel$ [001] and strain $\perp$ [001]
$P21'$ (3.2) $\mathbb{Z}_2$	• $\boldsymbol{E}$ $\parallel$ [001] and strain $\perp$ [001]

Continued on next page

...continued

Subgroup & SI group	Perturbation
$P\bar{1}$ (2.4) $\mathbb{Z}_2 \times \mathbb{Z}_2 \times \mathbb{Z}_2 \times \mathbb{Z}_4$	• $\boldsymbol{B} \parallel [100]$ and strain in generic direction<br>• $\boldsymbol{B} \parallel [010]$ and strain in generic direction<br>• $\boldsymbol{B} \parallel [001]$ and strain in generic direction<br>• $\boldsymbol{B} \perp [100]$ and strain $\perp [010]$<br>• $\boldsymbol{B} \perp [100]$ and strain $\perp [001]$<br>• $\boldsymbol{B} \perp [100]$ and strain in generic direction<br>• $\boldsymbol{B} \perp [010]$ and strain $\perp [100]$<br>• $\boldsymbol{B} \perp [010]$ and strain $\perp [001]$<br>• $\boldsymbol{B} \perp [010]$ and strain in generic direction<br>• $\boldsymbol{B} \perp [001]$ and strain $\perp [100]$<br>• $\boldsymbol{B} \perp [001]$ and strain $\perp [010]$<br>• $\boldsymbol{B} \perp [001]$ and strain in generic direction<br>• $\boldsymbol{B}$ in generic direction
$P2'/m'$ (10.46) $\mathbb{Z}_2 \times \mathbb{Z}_2 \times \mathbb{Z}_2 \times \mathbb{Z}_4$	• $\boldsymbol{B} \parallel [100]$ and strain $\perp [001]$<br>• $\boldsymbol{B} \parallel [010]$ and strain $\perp [001]$<br>• $\boldsymbol{B} \perp [001]$
$P2/m$ (10.42) $\mathbb{Z}_2 \times \mathbb{Z}_2 \times \mathbb{Z}_2$	• $\boldsymbol{B} \parallel [001]$ and strain $\perp [001]$
$P\bar{1}1'$ (2.5) $\mathbb{Z}_2 \times \mathbb{Z}_2 \times \mathbb{Z}_2 \times \mathbb{Z}_4$	• strain in generic direction
$P2/m1'$ (10.43) $\mathbb{Z}_2 \times \mathbb{Z}_2 \times \mathbb{Z}_2$	• strain $\perp [001]$
Pc' (7.26) $\mathbb{Z}_1$	• $\boldsymbol{E} \parallel [010]$ and $\boldsymbol{B} \parallel [010]$ and strain $\perp [100]$<br>• $\boldsymbol{E} \parallel [010]$ and $\boldsymbol{B} \parallel [001]$ and strain $\perp [100]$<br>• $\boldsymbol{E} \parallel [010]$ and $\boldsymbol{B} \perp [100]$<br>• $\boldsymbol{E} \parallel [001]$ and $\boldsymbol{B} \parallel [010]$ and strain $\perp [100]$<br>• $\boldsymbol{E} \parallel [001]$ and $\boldsymbol{B} \parallel [001]$ and strain $\perp [100]$<br>• $\boldsymbol{E} \parallel [001]$ and $\boldsymbol{B} \perp [100]$<br>• $\boldsymbol{E} \perp [100]$ and $\boldsymbol{B} \parallel [010]$<br>• $\boldsymbol{E} \perp [100]$ and $\boldsymbol{B} \parallel [001]$<br>• $\boldsymbol{E} \perp [100]$ and $\boldsymbol{B} \perp [100]$
$Pb'a2'$ (32.137) $\mathbb{Z}_1$	• $\boldsymbol{E} \parallel [001]$ and $\boldsymbol{B} \parallel [010]$
$Pb'a'2$ (32.138) $\mathbb{Z}_2$	• $\boldsymbol{E} \parallel [001]$ and $\boldsymbol{B} \parallel [001]$
$P2_1'/c'$ (14.79) $\mathbb{Z}_2 \times \mathbb{Z}_4$	• $\boldsymbol{B} \parallel [010]$ and strain $\perp [100]$<br>• $\boldsymbol{B} \parallel [001]$ and strain $\perp [100]$<br>• $\boldsymbol{B} \perp [100]$
Pc (7.24) $\mathbb{Z}_1$	• $\boldsymbol{E} \parallel [010]$ and $\boldsymbol{B} \parallel [100]$ and strain $\perp [100]$<br>• $\boldsymbol{E} \parallel [001]$ and $\boldsymbol{B} \parallel [100]$ and strain $\perp [100]$<br>• $\boldsymbol{E} \perp [100]$ and $\boldsymbol{B} \parallel [100]$

Continued on next page

…continued

Subgroup & SI group	Perturbation
$Pb'a2'$ (32.137) $\mathbb{Z}_1$	• $\boldsymbol{E}$ ∥ [001] and $\boldsymbol{B}$ ∥ [100]
$P2_1/c$ (14.75) $\mathbb{Z}_2$	• $\boldsymbol{B}$ ∥ [100] and strain ⊥ [100]
$Pc1'$ (7.25) $\mathbb{Z}_1$	• $\boldsymbol{E}$ ∥ [010] and strain ⊥ [100]<br>• $\boldsymbol{E}$ ∥ [001] and strain ⊥ [100]<br>• $\boldsymbol{E}$ ⊥ [100]
$Pba21'$ (32.136) $\mathbb{Z}_1$	• $\boldsymbol{E}$ ∥ [001]
$P2_1/c1'$ (14.76) $\mathbb{Z}_2$	• strain ⊥ [100]
$P2_1'$ (4.9) $\mathbb{Z}_1$	• $\boldsymbol{E}$ ∥ [010] and $\boldsymbol{B}$ ∥ [100] and strain ⊥ [010]<br>• $\boldsymbol{E}$ ∥ [010] and $\boldsymbol{B}$ ∥ [001] and strain ⊥ [010]<br>• $\boldsymbol{E}$ ∥ [010] and $\boldsymbol{B}$ ⊥ [010]
$P2_1'/c'$ (14.79) $\mathbb{Z}_2\times\mathbb{Z}_4$	• $\boldsymbol{B}$ ∥ [100] and strain ⊥ [010]<br>• $\boldsymbol{B}$ ∥ [001] and strain ⊥ [010]<br>• $\boldsymbol{B}$ ⊥ [010]
$Pmc'2_1'$ (26.69) $\mathbb{Z}_1$	• $\boldsymbol{E}$ ∥ [010] and $\boldsymbol{B}$ ∥ [001]
$Pb'a'm$ (55.357) $\mathbb{Z}_2\times\mathbb{Z}_2\times\mathbb{Z}_2$	• $\boldsymbol{B}$ ∥ [001]
$Pm'c2_1'$ (26.68) $\mathbb{Z}_1$	• $\boldsymbol{E}$ ∥ [010] and $\boldsymbol{B}$ ∥ [100]
$Pb'am'$ (55.358) $\mathbb{Z}_2$	• $\boldsymbol{B}$ ∥ [100]
$P2_1$ (4.7) $\mathbb{Z}_1$	• $\boldsymbol{E}$ ∥ [010] and $\boldsymbol{B}$ ∥ [010] and strain ⊥ [010]
$P2_1/c$ (14.75) $\mathbb{Z}_2$	• $\boldsymbol{B}$ ∥ [010] and strain ⊥ [010]
$Pm'c'2_1$ (26.70) $\mathbb{Z}_1$	• $\boldsymbol{E}$ ∥ [010] and $\boldsymbol{B}$ ∥ [010]
$Pb'am'$ (55.358) $\mathbb{Z}_2$	• $\boldsymbol{B}$ ∥ [010]
$P2_11'$ (4.8) $\mathbb{Z}_1$	• $\boldsymbol{E}$ ∥ [010] and strain ⊥ [010]
$P2_1/c1'$ (14.76) $\mathbb{Z}_2$	• strain ⊥ [010]
$Pmc2_11'$ (26.67) $\mathbb{Z}_1$	• $\boldsymbol{E}$ ∥ [010]

Table. S 465: Subgroups of $Pb'am$ (55.355) that can be realized by applying $\boldsymbol{E}/\boldsymbol{B}$ fields and/or uniaxial strains.

Subgroup & SI group	Perturbation

Continued on next page

...continued

Subgroup & SI group	Perturbation
Pm (6.18) $\mathbb{Z}_1$	• $\boldsymbol{B} \parallel [001]$ and strain $\perp [001]$<br>• $\boldsymbol{E} \parallel [100]$ and strain $\perp [001]$<br>• $\boldsymbol{E} \parallel [100]$ and $\boldsymbol{B} \parallel [001]$<br>• $\boldsymbol{E} \parallel [010]$ and strain $\perp [001]$<br>• $\boldsymbol{E} \perp [001]$
Pc (7.24) $\mathbb{Z}_1$	• $\boldsymbol{B} \parallel [010]$ and strain $\perp [010]$<br>• $\boldsymbol{E} \parallel [100]$ and strain $\perp [010]$<br>• $\boldsymbol{E} \parallel [100]$ and $\boldsymbol{B} \parallel [010]$<br>• $\boldsymbol{E} \parallel [001]$ and strain $\perp [010]$<br>• $\boldsymbol{E} \perp [010]$
$P2_1$ (4.7) $\mathbb{Z}_1$	• $\boldsymbol{B} \parallel [100]$ and strain $\perp [100]$<br>• $\boldsymbol{E} \parallel [100]$ and strain $\perp [100]$<br>• $\boldsymbol{E} \parallel [100]$ and $\boldsymbol{B} \parallel [100]$
$Pmc2_1$ (26.66) $\mathbb{Z}_1$	• $\boldsymbol{E} \parallel [100]$
$P2'$ (3.3) $\mathbb{Z}_1$	• $\boldsymbol{B} \parallel [100]$ and strain $\perp [001]$<br>• $\boldsymbol{B} \parallel [010]$ and strain $\perp [001]$<br>• $\boldsymbol{B} \perp [001]$<br>• $\boldsymbol{E} \parallel [001]$ and strain $\perp [001]$<br>• $\boldsymbol{E} \parallel [001]$ and $\boldsymbol{B} \parallel [100]$
$P\bar{1}'$ (2.6) $\mathbb{Z}_1$	• strain in generic direction
$P2'/m$ (10.44) $\mathbb{Z}_1$	• strain $\perp [001]$
Pc' (7.26) $\mathbb{Z}_1$	• $\boldsymbol{B} \parallel [010]$ and strain $\perp [100]$<br>• $\boldsymbol{B} \parallel [001]$ and strain $\perp [100]$<br>• $\boldsymbol{B} \perp [100]$<br>• $\boldsymbol{E} \parallel [010]$ and strain $\perp [100]$<br>• $\boldsymbol{E} \parallel [010]$ and $\boldsymbol{B} \parallel [010]$<br>• $\boldsymbol{E} \parallel [001]$ and strain $\perp [100]$<br>• $\boldsymbol{E} \parallel [001]$ and $\boldsymbol{B} \parallel [001]$<br>• $\boldsymbol{E} \perp [100]$
$Pb'a2'$ (32.137) $\mathbb{Z}_1$	• $\boldsymbol{B} \parallel [010]$<br>• $\boldsymbol{E} \parallel [001]$
$P2_1/c'$ (14.78) $\mathbb{Z}_1$	• strain $\perp [100]$

Continued on next page

. . . continued

Subgroup & SI group	Perturbation
$P2_1'$ (4.9) $\mathbb{Z}_1$	• $\boldsymbol{B}$ ∥ [100] and strain ⊥ [010]<br>• $\boldsymbol{B}$ ∥ [001] and strain ⊥ [010]<br>• $\boldsymbol{B}$ ⊥ [010]<br>• $\boldsymbol{E}$ ∥ [010] and strain ⊥ [010]<br>• $\boldsymbol{E}$ ∥ [010] and $\boldsymbol{B}$ ∥ [100]
$P2_12_1'2'$ (18.19) $\mathbb{Z}_1$	• $\boldsymbol{B}$ ∥ [100]
$P2_1'/c$ (14.77) $\mathbb{Z}_1$	• strain ⊥ [010]
$Pmc'2_1'$ (26.69) $\mathbb{Z}_1$	• $\boldsymbol{B}$ ∥ [001]<br>• $\boldsymbol{E}$ ∥ [010]

Table. S 466: Subgroups of $Pbam'$ (55.356) that can be realized by applying $\boldsymbol{E}/\boldsymbol{B}$ fields and/or uniaxial strains.

Subgroup & SI group	Perturbation
Pm' (6.20) $\mathbb{Z}_1$	• $\boldsymbol{B}$ ∥ [100] and strain ⊥ [001]<br>• $\boldsymbol{B}$ ∥ [010] and strain ⊥ [001]<br>• $\boldsymbol{B}$ ⊥ [001]<br>• $\boldsymbol{E}$ ∥ [100] and strain ⊥ [001]<br>• $\boldsymbol{E}$ ∥ [100] and $\boldsymbol{B}$ ∥ [100]<br>• $\boldsymbol{E}$ ∥ [010] and strain ⊥ [001]<br>• $\boldsymbol{E}$ ∥ [010] and $\boldsymbol{B}$ ∥ [010]<br>• $\boldsymbol{E}$ ⊥ [001]
Pc (7.24) $\mathbb{Z}_1$	• $\boldsymbol{B}$ ∥ [010] and strain ⊥ [010]<br>• $\boldsymbol{E}$ ∥ [100] and strain ⊥ [010]<br>• $\boldsymbol{E}$ ∥ [001] and strain ⊥ [010]<br>• $\boldsymbol{E}$ ∥ [001] and $\boldsymbol{B}$ ∥ [010]<br>• $\boldsymbol{E}$ ⊥ [010]
$P2_1'$ (4.9) $\mathbb{Z}_1$	• $\boldsymbol{B}$ ∥ [010] and strain ⊥ [100]<br>• $\boldsymbol{B}$ ∥ [001] and strain ⊥ [100]<br>• $\boldsymbol{B}$ ⊥ [100]<br>• $\boldsymbol{E}$ ∥ [100] and strain ⊥ [100]<br>• $\boldsymbol{E}$ ∥ [100] and $\boldsymbol{B}$ ∥ [001]
$Pm'c2_1'$ (26.68) $\mathbb{Z}_1$	• $\boldsymbol{B}$ ∥ [010]<br>• $\boldsymbol{E}$ ∥ [100]

Continued on next page

...continued

Subgroup & SI group	Perturbation
$P2$ (3.1) $\mathbb{Z}_2$	• $\boldsymbol{B} \parallel [001]$ and strain $\perp [001]$<br>• $\boldsymbol{E} \parallel [001]$ and strain $\perp [001]$<br>• $\boldsymbol{E} \parallel [001]$ and $\boldsymbol{B} \parallel [001]$
$P\bar{1}'$ (2.6) $\mathbb{Z}_1$	• strain in generic direction
$P2/m'$ (10.45) $\mathbb{Z}_2$	• strain $\perp [001]$
Pc (7.24) $\mathbb{Z}_1$	• $\boldsymbol{B} \parallel [100]$ and strain $\perp [100]$<br>• $\boldsymbol{E} \parallel [010]$ and strain $\perp [100]$<br>• $\boldsymbol{E} \parallel [001]$ and strain $\perp [100]$<br>• $\boldsymbol{E} \parallel [001]$ and $\boldsymbol{B} \parallel [100]$<br>• $\boldsymbol{E} \perp [100]$
$Pba2$ (32.135) $\mathbb{Z}_1$	• $\boldsymbol{E} \parallel [001]$
$P2_1'/c$ (14.77) $\mathbb{Z}_1$	• strain $\perp [100]$
$P2_1'$ (4.9) $\mathbb{Z}_1$	• $\boldsymbol{B} \parallel [100]$ and strain $\perp [010]$<br>• $\boldsymbol{B} \parallel [001]$ and strain $\perp [010]$<br>• $\boldsymbol{B} \perp [010]$<br>• $\boldsymbol{E} \parallel [010]$ and strain $\perp [010]$<br>• $\boldsymbol{E} \parallel [010]$ and $\boldsymbol{B} \parallel [001]$
$P2_1'2_1'2$ (18.18) $\mathbb{Z}_2$	• $\boldsymbol{B} \parallel [001]$
$P2_1'/c$ (14.77) $\mathbb{Z}_1$	• strain $\perp [010]$
$Pm'c2_1'$ (26.68) $\mathbb{Z}_1$	• $\boldsymbol{B} \parallel [100]$<br>• $\boldsymbol{E} \parallel [010]$

Table. S 467: Subgroups of $Pb'a'm$ (55.357) that can be realized by applying $\boldsymbol{E}/\boldsymbol{B}$ fields and/or uniaxial strains.

Subgroup & SI group	Perturbation
Pm (6.18) $\mathbb{Z}_1$	• $\boldsymbol{E} \parallel [100]$ and strain $\perp [001]$<br>• $\boldsymbol{E} \parallel [010]$ and strain $\perp [001]$<br>• $\boldsymbol{E} \perp [001]$

Continued on next page

…continued

Subgroup & SI group	Perturbation
Pc' (7.26) $\mathbb{Z}_1$	• $\boldsymbol{E} \parallel [100]$ and strain $\perp [010]$<br>• $\boldsymbol{E} \parallel [100]$ and ($\boldsymbol{B} \parallel [100]$ or $\boldsymbol{B} \perp [010]$)<br>• $\boldsymbol{E} \parallel [001]$ and strain $\perp [010]$<br>• $\boldsymbol{E} \parallel [001]$ and ($\boldsymbol{B} \parallel [100]$ or $\boldsymbol{B} \perp [010]$)<br>• $\boldsymbol{E} \perp [010]$
$P2_1'$ (4.9) $\mathbb{Z}_1$	• $\boldsymbol{E} \parallel [100]$ and strain $\perp [100]$<br>• $\boldsymbol{E} \parallel [100]$ and ($\boldsymbol{B} \parallel [010]$ or $\boldsymbol{B} \perp [100]$)
$Pmc'2_1'$ (26.69) $\mathbb{Z}_1$	• $\boldsymbol{E} \parallel [100]$
$P2$ (3.1) $\mathbb{Z}_2$	• $\boldsymbol{E} \parallel [001]$ and strain $\perp [001]$
$P\bar{1}$ (2.4) $\mathbb{Z}_2 \times \mathbb{Z}_2 \times \mathbb{Z}_2 \times \mathbb{Z}_4$	• strain in generic direction<br>• ($\boldsymbol{B} \parallel [100]$ or $\boldsymbol{B} \perp [010]$) and strain $\perp [100]$<br>• ($\boldsymbol{B} \parallel [100]$ or $\boldsymbol{B} \perp [010]$) and strain $\perp [001]$<br>• ($\boldsymbol{B} \parallel [010]$ or $\boldsymbol{B} \perp [100]$) and strain $\perp [010]$<br>• ($\boldsymbol{B} \parallel [010]$ or $\boldsymbol{B} \perp [100]$) and strain $\perp [001]$<br>• $\boldsymbol{B}$ in generic direction
$P2/m$ (10.42) $\mathbb{Z}_2 \times \mathbb{Z}_2 \times \mathbb{Z}_2$	• strain $\perp [001]$
Pc' (7.26) $\mathbb{Z}_1$	• $\boldsymbol{E} \parallel [010]$ and strain $\perp [100]$<br>• $\boldsymbol{E} \parallel [010]$ and ($\boldsymbol{B} \parallel [010]$ or $\boldsymbol{B} \perp [100]$)<br>• $\boldsymbol{E} \parallel [001]$ and strain $\perp [100]$<br>• $\boldsymbol{E} \parallel [001]$ and ($\boldsymbol{B} \parallel [010]$ or $\boldsymbol{B} \perp [100]$)<br>• $\boldsymbol{E} \perp [100]$
$Pb'a'2$ (32.138) $\mathbb{Z}_2$	• $\boldsymbol{E} \parallel [001]$
$P2_1'/c'$ (14.79) $\mathbb{Z}_2 \times \mathbb{Z}_4$	• strain $\perp [100]$<br>• ($\boldsymbol{B} \parallel [010]$ or $\boldsymbol{B} \perp [100]$)
$P2_1'$ (4.9) $\mathbb{Z}_1$	• $\boldsymbol{E} \parallel [010]$ and strain $\perp [010]$<br>• $\boldsymbol{E} \parallel [010]$ and ($\boldsymbol{B} \parallel [100]$ or $\boldsymbol{B} \perp [010]$)
$P2_1'/c'$ (14.79) $\mathbb{Z}_2 \times \mathbb{Z}_4$	• strain $\perp [010]$<br>• ($\boldsymbol{B} \parallel [100]$ or $\boldsymbol{B} \perp [010]$)
$Pmc'2_1'$ (26.69) $\mathbb{Z}_1$	• $\boldsymbol{E} \parallel [010]$

Table. S 468: Subgroups of $Pb'am'$ (55.358) that can be realized by applying $\boldsymbol{E}/\boldsymbol{B}$ fields and/or uniaxial strains.

Subgroup & SI group	Perturbation
Pm' (6.20) $\mathbb{Z}_1$	• $\boldsymbol{E} \parallel [100]$ and strain $\perp [001]$<br>• $\boldsymbol{E} \parallel [100]$ and ($\boldsymbol{B} \parallel [100]$ or $\boldsymbol{B} \perp [001]$)<br>• $\boldsymbol{E} \parallel [010]$ and strain $\perp [001]$<br>• $\boldsymbol{E} \parallel [010]$ and ($\boldsymbol{B} \parallel [100]$ or $\boldsymbol{B} \perp [001]$)<br>• $\boldsymbol{E} \perp [001]$
Pc (7.24) $\mathbb{Z}_1$	• $\boldsymbol{E} \parallel [100]$ and strain $\perp [010]$<br>• $\boldsymbol{E} \parallel [001]$ and strain $\perp [010]$<br>• $\boldsymbol{E} \perp [010]$
$P2_1'$ (4.9) $\mathbb{Z}_1$	• $\boldsymbol{E} \parallel [100]$ and strain $\perp [100]$<br>• $\boldsymbol{E} \parallel [100]$ and ($\boldsymbol{B} \parallel [001]$ or $\boldsymbol{B} \perp [100]$)
$Pm'c2_1'$ (26.68) $\mathbb{Z}_1$	• $\boldsymbol{E} \parallel [100]$
$P2'$ (3.3) $\mathbb{Z}_1$	• $\boldsymbol{E} \parallel [001]$ and strain $\perp [001]$<br>• $\boldsymbol{E} \parallel [001]$ and ($\boldsymbol{B} \parallel [100]$ or $\boldsymbol{B} \perp [001]$)
$P\bar{1}$ (2.4) $\mathbb{Z}_2 \times \mathbb{Z}_2 \times \mathbb{Z}_2 \times \mathbb{Z}_4$	• strain in generic direction<br>• ($\boldsymbol{B} \parallel [100]$ or $\boldsymbol{B} \perp [001]$) and strain $\perp [100]$<br>• ($\boldsymbol{B} \parallel [100]$ or $\boldsymbol{B} \perp [001]$) and strain $\perp [010]$<br>• ($\boldsymbol{B} \parallel [001]$ or $\boldsymbol{B} \perp [100]$) and strain $\perp [010]$<br>• ($\boldsymbol{B} \parallel [001]$ or $\boldsymbol{B} \perp [100]$) and strain $\perp [001]$<br>• $\boldsymbol{B}$ in generic direction
$P2'/m'$ (10.46) $\mathbb{Z}_2 \times \mathbb{Z}_2 \times \mathbb{Z}_2 \times \mathbb{Z}_4$	• strain $\perp [001]$<br>• ($\boldsymbol{B} \parallel [100]$ or $\boldsymbol{B} \perp [001]$)
Pc' (7.26) $\mathbb{Z}_1$	• $\boldsymbol{E} \parallel [010]$ and strain $\perp [100]$<br>• $\boldsymbol{E} \parallel [010]$ and ($\boldsymbol{B} \parallel [001]$ or $\boldsymbol{B} \perp [100]$)<br>• $\boldsymbol{E} \parallel [001]$ and strain $\perp [100]$<br>• $\boldsymbol{E} \parallel [001]$ and ($\boldsymbol{B} \parallel [001]$ or $\boldsymbol{B} \perp [100]$)<br>• $\boldsymbol{E} \perp [100]$
$Pb'a2'$ (32.137) $\mathbb{Z}_1$	• $\boldsymbol{E} \parallel [001]$
$P2_1'/c'$ (14.79) $\mathbb{Z}_2 \times \mathbb{Z}_4$	• strain $\perp [100]$<br>• ($\boldsymbol{B} \parallel [001]$ or $\boldsymbol{B} \perp [100]$)
$P2_1$ (4.7) $\mathbb{Z}_1$	• $\boldsymbol{E} \parallel [010]$ and strain $\perp [010]$
$P2_1/c$ (14.75) $\mathbb{Z}_2$	• strain $\perp [010]$
$Pm'c'2_1$ (26.70) $\mathbb{Z}_1$	• $\boldsymbol{E} \parallel [010]$

Table. S 469: Subgroups of $Pb'a'm'$ (55.359) that can be realized by applying $\boldsymbol{E}/\boldsymbol{B}$ fields and/or uniaxial strains.

Subgroup & SI group	Perturbation
Pm' (6.20) $\mathbb{Z}_1$	• $\boldsymbol{B}$ ∥ [100] and strain ⊥ [001]<br>• $\boldsymbol{B}$ ∥ [010] and strain ⊥ [001]<br>• $\boldsymbol{B}$ ⊥ [001]<br>• $\boldsymbol{E}$ ∥ [100] and strain ⊥ [001]<br>• $\boldsymbol{E}$ ∥ [100] and $\boldsymbol{B}$ ∥ [010]<br>• $\boldsymbol{E}$ ∥ [010] and strain ⊥ [001]<br>• $\boldsymbol{E}$ ∥ [010] and $\boldsymbol{B}$ ∥ [100]<br>• $\boldsymbol{E}$ ⊥ [001]
Pc' (7.26) $\mathbb{Z}_1$	• $\boldsymbol{B}$ ∥ [100] and strain ⊥ [010]<br>• $\boldsymbol{B}$ ∥ [001] and strain ⊥ [010]<br>• $\boldsymbol{B}$ ⊥ [010]<br>• $\boldsymbol{E}$ ∥ [100] and strain ⊥ [010]<br>• $\boldsymbol{E}$ ∥ [100] and $\boldsymbol{B}$ ∥ [001]<br>• $\boldsymbol{E}$ ∥ [001] and strain ⊥ [010]<br>• $\boldsymbol{E}$ ∥ [001] and $\boldsymbol{B}$ ∥ [100]<br>• $\boldsymbol{E}$ ⊥ [010]
$P2_1$ (4.7) $\mathbb{Z}_1$	• $\boldsymbol{B}$ ∥ [100] and strain ⊥ [100]<br>• $\boldsymbol{E}$ ∥ [100] and strain ⊥ [100]
$Pm'c'2_1$ (26.70) $\mathbb{Z}_1$	• $\boldsymbol{B}$ ∥ [100]<br>• $\boldsymbol{E}$ ∥ [100]
$P2$ (3.1) $\mathbb{Z}_2$	• $\boldsymbol{B}$ ∥ [001] and strain ⊥ [001]<br>• $\boldsymbol{E}$ ∥ [001] and strain ⊥ [001]
$P\bar{1}'$ (2.6) $\mathbb{Z}_1$	• strain in generic direction
$P2/m'$ (10.45) $\mathbb{Z}_2$	• strain ⊥ [001]
Pc' (7.26) $\mathbb{Z}_1$	• $\boldsymbol{B}$ ∥ [010] and strain ⊥ [100]<br>• $\boldsymbol{B}$ ∥ [001] and strain ⊥ [100]<br>• $\boldsymbol{B}$ ⊥ [100]<br>• $\boldsymbol{E}$ ∥ [010] and strain ⊥ [100]<br>• $\boldsymbol{E}$ ∥ [010] and $\boldsymbol{B}$ ∥ [001]<br>• $\boldsymbol{E}$ ∥ [001] and strain ⊥ [100]<br>• $\boldsymbol{E}$ ∥ [001] and $\boldsymbol{B}$ ∥ [010]<br>• $\boldsymbol{E}$ ⊥ [100]
$Pb'a'2$ (32.138) $\mathbb{Z}_2$	• $\boldsymbol{B}$ ∥ [001]<br>• $\boldsymbol{E}$ ∥ [001]
$P2_1/c'$ (14.78) $\mathbb{Z}_1$	• strain ⊥ [100]

Continued on next page

…continued

Subgroup & SI group	Perturbation
$P2_1$ (4.7) $\mathbb{Z}_1$	• $\boldsymbol{B} \parallel [010]$ and strain $\perp [010]$<br>• $\boldsymbol{E} \parallel [010]$ and strain $\perp [010]$
$P2_1/c'$ (14.78) $\mathbb{Z}_1$	• strain $\perp [010]$
$Pm'c'2_1$ (26.70) $\mathbb{Z}_1$	• $\boldsymbol{B} \parallel [010]$<br>• $\boldsymbol{E} \parallel [010]$

Table. S 470: Subgroups of P_abam (55.360) that can be realized by applying $\boldsymbol{E}/\boldsymbol{B}$ fields and/or uniaxial strains.

Subgroup & SI group	Perturbation
Pm (6.18) $\mathbb{Z}_1$	• $\boldsymbol{E} \parallel [100]$ and $\boldsymbol{B} \parallel [001]$ and strain $\perp [001]$<br>• $\boldsymbol{E} \parallel [010]$ and $\boldsymbol{B} \parallel [001]$ and strain $\perp [001]$<br>• $\boldsymbol{E} \perp [001]$ and $\boldsymbol{B} \parallel [001]$
Pm' (6.20) $\mathbb{Z}_1$	• $\boldsymbol{E} \parallel [100]$ and $\boldsymbol{B} \parallel [100]$ and strain $\perp [010]$<br>• $\boldsymbol{E} \parallel [100]$ and $\boldsymbol{B} \parallel [001]$ and strain $\perp [010]$<br>• $\boldsymbol{E} \parallel [100]$ and $\boldsymbol{B} \perp [010]$<br>• $\boldsymbol{E} \parallel [001]$ and $\boldsymbol{B} \parallel [100]$ and strain $\perp [010]$<br>• $\boldsymbol{E} \parallel [001]$ and $\boldsymbol{B} \parallel [001]$ and strain $\perp [010]$<br>• $\boldsymbol{E} \parallel [001]$ and $\boldsymbol{B} \perp [010]$<br>• $\boldsymbol{E} \perp [010]$ and $\boldsymbol{B} \parallel [100]$<br>• $\boldsymbol{E} \perp [010]$ and $\boldsymbol{B} \parallel [001]$<br>• $\boldsymbol{E} \perp [010]$ and $\boldsymbol{B} \perp [010]$
$P2'$ (3.3) $\mathbb{Z}_1$	• $\boldsymbol{E} \parallel [100]$ and $\boldsymbol{B} \parallel [010]$ and strain $\perp [100]$<br>• $\boldsymbol{E} \parallel [100]$ and $\boldsymbol{B} \parallel [001]$ and strain $\perp [100]$<br>• $\boldsymbol{E} \parallel [100]$ and $\boldsymbol{B} \perp [100]$
$Pm'm2'$ (25.59) $\mathbb{Z}_1$	• $\boldsymbol{E} \parallel [100]$ and $\boldsymbol{B} \parallel [001]$

Continued on next page

...continued

Subgroup & SI group	Perturbation
P_S1 (1.3) $\mathbb{Z}_1$	• $\boldsymbol{E} \parallel [100]$ and strain in generic direction<br>• $\boldsymbol{E} \parallel [010]$ and strain in generic direction<br>• $\boldsymbol{E} \parallel [001]$ and strain in generic direction<br>• $\boldsymbol{E} \perp [100]$ and strain $\perp [010]$<br>• $\boldsymbol{E} \perp [100]$ and strain $\perp [001]$<br>• $\boldsymbol{E} \perp [100]$ and strain in generic direction<br>• $\boldsymbol{E} \perp [010]$ and strain $\perp [100]$<br>• $\boldsymbol{E} \perp [010]$ and strain $\perp [001]$<br>• $\boldsymbol{E} \perp [010]$ and strain in generic direction<br>• $\boldsymbol{E} \perp [001]$ and strain $\perp [100]$<br>• $\boldsymbol{E} \perp [001]$ and strain $\perp [010]$<br>• $\boldsymbol{E} \perp [001]$ and strain in generic direction<br>• $\boldsymbol{E}$ in generic direction
Pc' (7.26) $\mathbb{Z}_1$	• $\boldsymbol{E} \parallel [100]$ and $\boldsymbol{B} \parallel [100]$ and strain $\perp [001]$<br>• $\boldsymbol{E} \parallel [100]$ and $\boldsymbol{B} \parallel [010]$ and strain $\perp [001]$<br>• $\boldsymbol{E} \parallel [100]$ and $\boldsymbol{B} \perp [001]$<br>• $\boldsymbol{E} \parallel [010]$ and $\boldsymbol{B} \parallel [100]$ and strain $\perp [001]$<br>• $\boldsymbol{E} \parallel [010]$ and $\boldsymbol{B} \parallel [010]$ and strain $\perp [001]$<br>• $\boldsymbol{E} \parallel [010]$ and $\boldsymbol{B} \perp [001]$<br>• $\boldsymbol{E} \perp [001]$ and $\boldsymbol{B} \parallel [100]$<br>• $\boldsymbol{E} \perp [001]$ and $\boldsymbol{B} \parallel [010]$<br>• $\boldsymbol{E} \perp [001]$ and $\boldsymbol{B} \perp [001]$
P_am (6.21) $\mathbb{Z}_1$	• $\boldsymbol{E} \parallel [100]$ and strain $\perp [001]$<br>• $\boldsymbol{E} \parallel [010]$ and strain $\perp [001]$<br>• $\boldsymbol{E} \perp [001]$
Pc (7.24) $\mathbb{Z}_1$	• $\boldsymbol{E} \parallel [100]$ and $\boldsymbol{B} \parallel [010]$ and strain $\perp [010]$<br>• $\boldsymbol{E} \parallel [001]$ and $\boldsymbol{B} \parallel [010]$ and strain $\perp [010]$<br>• $\boldsymbol{E} \perp [010]$ and $\boldsymbol{B} \parallel [010]$
P_cc (7.28) $\mathbb{Z}_1$	• $\boldsymbol{E} \parallel [100]$ and strain $\perp [010]$<br>• $\boldsymbol{E} \parallel [001]$ and strain $\perp [010]$<br>• $\boldsymbol{E} \perp [010]$
$Pc'c2'$ (27.80) $\mathbb{Z}_1$	• $\boldsymbol{E} \parallel [100]$ and $\boldsymbol{B} \parallel [010]$
$P2_1$ (4.7) $\mathbb{Z}_1$	• $\boldsymbol{E} \parallel [100]$ and $\boldsymbol{B} \parallel [100]$ and strain $\perp [100]$
P_b2_1 (4.11) $\mathbb{Z}_1$	• $\boldsymbol{E} \parallel [100]$ and strain $\perp [100]$
$Pm'c'2_1$ (26.70) $\mathbb{Z}_1$	• $\boldsymbol{E} \parallel [100]$ and $\boldsymbol{B} \parallel [100]$
P_cmc2_1 (26.73) $\mathbb{Z}_1$	• $\boldsymbol{E} \parallel [100]$

Continued on next page

…continued

Subgroup & SI group	Perturbation
Pc' (7.26) $\mathbb{Z}_1$	• $\boldsymbol{E}$ ‖ [010] and $\boldsymbol{B}$ ‖ [010] and strain ⊥ [100]<br>• $\boldsymbol{E}$ ‖ [010] and $\boldsymbol{B}$ ‖ [001] and strain ⊥ [100]<br>• $\boldsymbol{E}$ ‖ [010] and $\boldsymbol{B}$ ⊥ [100]<br>• $\boldsymbol{E}$ ‖ [001] and $\boldsymbol{B}$ ‖ [010] and strain ⊥ [100]<br>• $\boldsymbol{E}$ ‖ [001] and $\boldsymbol{B}$ ‖ [001] and strain ⊥ [100]<br>• $\boldsymbol{E}$ ‖ [001] and $\boldsymbol{B}$ ⊥ [100]<br>• $\boldsymbol{E}$ ⊥ [100] and $\boldsymbol{B}$ ‖ [010]<br>• $\boldsymbol{E}$ ⊥ [100] and $\boldsymbol{B}$ ‖ [001]<br>• $\boldsymbol{E}$ ⊥ [100] and $\boldsymbol{B}$ ⊥ [100]
$P2_1'$ (4.9) $\mathbb{Z}_1$	• $\boldsymbol{E}$ ‖ [010] and $\boldsymbol{B}$ ‖ [100] and strain ⊥ [010]<br>• $\boldsymbol{E}$ ‖ [010] and $\boldsymbol{B}$ ‖ [001] and strain ⊥ [010]<br>• $\boldsymbol{E}$ ‖ [010] and $\boldsymbol{B}$ ⊥ [010]
$Pmc'2_1'$ (26.69) $\mathbb{Z}_1$	• $\boldsymbol{E}$ ‖ [010] and $\boldsymbol{B}$ ‖ [001]
$P2$ (3.1) $\mathbb{Z}_2$	• $\boldsymbol{E}$ ‖ [001] and $\boldsymbol{B}$ ‖ [001] and strain ⊥ [001]
$Pm'a'2$ (28.91) $\mathbb{Z}_2$	• $\boldsymbol{E}$ ‖ [001] and $\boldsymbol{B}$ ‖ [001]
$P\bar{1}$ (2.4) $\mathbb{Z}_2 \times \mathbb{Z}_2 \times \mathbb{Z}_2 \times \mathbb{Z}_4$	• $\boldsymbol{B}$ ‖ [100] and strain in generic direction<br>• $\boldsymbol{B}$ ‖ [010] and strain in generic direction<br>• $\boldsymbol{B}$ ‖ [001] and strain in generic direction<br>• $\boldsymbol{B}$ ⊥ [100] and strain ⊥ [010]<br>• $\boldsymbol{B}$ ⊥ [100] and strain ⊥ [001]<br>• $\boldsymbol{B}$ ⊥ [100] and strain in generic direction<br>• $\boldsymbol{B}$ ⊥ [010] and strain ⊥ [100]<br>• $\boldsymbol{B}$ ⊥ [010] and strain ⊥ [001]<br>• $\boldsymbol{B}$ ⊥ [010] and strain in generic direction<br>• $\boldsymbol{B}$ ⊥ [001] and strain ⊥ [100]<br>• $\boldsymbol{B}$ ⊥ [001] and strain ⊥ [010]<br>• $\boldsymbol{B}$ ⊥ [001] and strain in generic direction<br>• $\boldsymbol{B}$ in generic direction
$P2'/c'$ (13.69) $\mathbb{Z}_2 \times \mathbb{Z}_2 \times \mathbb{Z}_4$	• $\boldsymbol{B}$ ‖ [010] and strain ⊥ [100]<br>• $\boldsymbol{B}$ ‖ [001] and strain ⊥ [100]<br>• $\boldsymbol{B}$ ⊥ [100]
$P2_1'/m'$ (11.54) $\mathbb{Z}_2 \times \mathbb{Z}_2 \times \mathbb{Z}_4$	• $\boldsymbol{B}$ ‖ [100] and strain ⊥ [010]<br>• $\boldsymbol{B}$ ‖ [001] and strain ⊥ [010]<br>• $\boldsymbol{B}$ ⊥ [010]
$P2/m$ (10.42) $\mathbb{Z}_2 \times \mathbb{Z}_2 \times \mathbb{Z}_2$	• $\boldsymbol{B}$ ‖ [001] and strain ⊥ [001]
$Pm'ma'$ (51.296) $\mathbb{Z}_2 \times \mathbb{Z}_2 \times \mathbb{Z}_2$	• $\boldsymbol{B}$ ‖ [001]

Continued on next page

...continued

Subgroup & SI group	Perturbation
Pc (7.24) $\mathbb{Z}_1$	• $\boldsymbol{E} \parallel [010]$ and $\boldsymbol{B} \parallel [100]$ and strain $\perp [100]$<br>• $\boldsymbol{E} \parallel [001]$ and $\boldsymbol{B} \parallel [100]$ and strain $\perp [100]$<br>• $\boldsymbol{E} \perp [100]$ and $\boldsymbol{B} \parallel [100]$
P_bc (7.29) $\mathbb{Z}_1$	• $\boldsymbol{E} \parallel [010]$ and strain $\perp [100]$<br>• $\boldsymbol{E} \parallel [001]$ and strain $\perp [100]$<br>• $\boldsymbol{E} \perp [100]$
$Pca'2_1'$ (29.102) $\mathbb{Z}_1$	• $\boldsymbol{E} \parallel [010]$ and $\boldsymbol{B} \parallel [100]$
$P2_1/c$ (14.75) $\mathbb{Z}_2$	• $\boldsymbol{B} \parallel [100]$ and strain $\perp [100]$
$P2_1$ (4.7) $\mathbb{Z}_1$	• $\boldsymbol{E} \parallel [010]$ and $\boldsymbol{B} \parallel [010]$ and strain $\perp [010]$
$Pc'a'2_1$ (29.103) $\mathbb{Z}_1$	• $\boldsymbol{E} \parallel [010]$ and $\boldsymbol{B} \parallel [010]$
P_a2_1 (4.10) $\mathbb{Z}_1$	• $\boldsymbol{E} \parallel [010]$ and strain $\perp [010]$
$P2_1/c$ (14.75) $\mathbb{Z}_2$	• $\boldsymbol{B} \parallel [010]$ and strain $\perp [010]$
P_bmc2_1 (26.72) $\mathbb{Z}_1$	• $\boldsymbol{E} \parallel [010]$
$P2'$ (3.3) $\mathbb{Z}_1$	• $\boldsymbol{E} \parallel [001]$ and $\boldsymbol{B} \parallel [100]$ and strain $\perp [001]$<br>• $\boldsymbol{E} \parallel [001]$ and $\boldsymbol{B} \parallel [010]$ and strain $\perp [001]$<br>• $\boldsymbol{E} \parallel [001]$ and $\boldsymbol{B} \perp [001]$
$Pb'a2'$ (32.137) $\mathbb{Z}_1$	• $\boldsymbol{E} \parallel [001]$ and $\boldsymbol{B} \parallel [010]$
P_a2 (3.4) $\mathbb{Z}_2$	• $\boldsymbol{E} \parallel [001]$ and strain $\perp [001]$
$P2'/c'$ (13.69) $\mathbb{Z}_2 \times \mathbb{Z}_2 \times \mathbb{Z}_4$	• $\boldsymbol{B} \parallel [100]$ and strain $\perp [001]$<br>• $\boldsymbol{B} \parallel [010]$ and strain $\perp [001]$<br>• $\boldsymbol{B} \perp [001]$
$Pm'a2'$ (28.89) $\mathbb{Z}_1$	• $\boldsymbol{E} \parallel [001]$ and $\boldsymbol{B} \parallel [100]$
P_bba2 (32.140) $\mathbb{Z}_1$	• $\boldsymbol{E} \parallel [001]$
$Pb'cm'$ (57.384) $\mathbb{Z}_2$	• $\boldsymbol{B} \parallel [100]$
$Pcc'a'$ (54.343) $\mathbb{Z}_2$	• $\boldsymbol{B} \parallel [010]$
$P_S\bar{1}$ (2.7) $\mathbb{Z}_2 \times \mathbb{Z}_2 \times \mathbb{Z}_4$	• strain in generic direction
P_b2_1/c (14.81) $\mathbb{Z}_2$	• strain $\perp [100]$
P_c2_1/c (14.82) $\mathbb{Z}_2$	• strain $\perp [010]$
P_a2/m (10.47) $\mathbb{Z}_2 \times \mathbb{Z}_2 \times \mathbb{Z}_2$	• strain $\perp [001]$

Table. S 471: Subgroups of P_cbam (55.361) that can be realized by applying $\boldsymbol{E}/\boldsymbol{B}$ fields and/or uniaxial strains.

Subgroup & SI group	Perturbation
P_S1 (1.3) $\mathbb{Z}_1$	• $\boldsymbol{E} \parallel [100]$ and strain in generic direction<br>• $\boldsymbol{E} \parallel [010]$ and strain in generic direction<br>• $\boldsymbol{E} \parallel [001]$ and strain in generic direction<br>• $\boldsymbol{E} \perp [100]$ and strain $\perp [010]$<br>• $\boldsymbol{E} \perp [100]$ and strain $\perp [001]$<br>• $\boldsymbol{E} \perp [100]$ and strain in generic direction<br>• $\boldsymbol{E} \perp [010]$ and strain $\perp [100]$<br>• $\boldsymbol{E} \perp [010]$ and strain $\perp [001]$<br>• $\boldsymbol{E} \perp [010]$ and strain in generic direction<br>• $\boldsymbol{E} \perp [001]$ and strain $\perp [100]$<br>• $\boldsymbol{E} \perp [001]$ and strain $\perp [010]$<br>• $\boldsymbol{E} \perp [001]$ and strain in generic direction<br>• $\boldsymbol{E}$ in generic direction
Pm (6.18) $\mathbb{Z}_1$	• $\boldsymbol{E} \parallel [100]$ and $\boldsymbol{B} \parallel [001]$ and strain $\perp [001]$<br>• $\boldsymbol{E} \parallel [010]$ and $\boldsymbol{B} \parallel [001]$ and strain $\perp [001]$<br>• $\boldsymbol{E} \perp [001]$ and $\boldsymbol{B} \parallel [001]$
Pm' (6.20) $\mathbb{Z}_1$	• $\boldsymbol{E} \parallel [100]$ and $\boldsymbol{B} \parallel [100]$ and strain $\perp [001]$<br>• $\boldsymbol{E} \parallel [100]$ and $\boldsymbol{B} \parallel [010]$ and strain $\perp [001]$<br>• $\boldsymbol{E} \parallel [100]$ and $\boldsymbol{B} \perp [001]$<br>• $\boldsymbol{E} \parallel [010]$ and $\boldsymbol{B} \parallel [100]$ and strain $\perp [001]$<br>• $\boldsymbol{E} \parallel [010]$ and $\boldsymbol{B} \parallel [010]$ and strain $\perp [001]$<br>• $\boldsymbol{E} \parallel [010]$ and $\boldsymbol{B} \perp [001]$<br>• $\boldsymbol{E} \perp [001]$ and $\boldsymbol{B} \parallel [100]$<br>• $\boldsymbol{E} \perp [001]$ and $\boldsymbol{B} \parallel [010]$<br>• $\boldsymbol{E} \perp [001]$ and $\boldsymbol{B} \perp [001]$
P_bm (6.22) $\mathbb{Z}_1$	• $\boldsymbol{E} \parallel [100]$ and strain $\perp [001]$<br>• $\boldsymbol{E} \parallel [010]$ and strain $\perp [001]$<br>• $\boldsymbol{E} \perp [001]$
Pc (7.24) $\mathbb{Z}_1$	• $\boldsymbol{E} \parallel [100]$ and $\boldsymbol{B} \parallel [010]$ and strain $\perp [010]$<br>• $\boldsymbol{E} \parallel [001]$ and $\boldsymbol{B} \parallel [010]$ and strain $\perp [010]$<br>• $\boldsymbol{E} \perp [010]$ and $\boldsymbol{B} \parallel [010]$

Continued on next page

. . . continued

Subgroup & SI group	Perturbation
Pc' (7.26) $\mathbb{Z}_1$	• $\boldsymbol{E}$ ∥ [100] and $\boldsymbol{B}$ ∥ [100] and strain ⊥ [010]<br>• $\boldsymbol{E}$ ∥ [100] and $\boldsymbol{B}$ ∥ [001] and strain ⊥ [010]<br>• $\boldsymbol{E}$ ∥ [100] and $\boldsymbol{B}$ ⊥ [010]<br>• $\boldsymbol{E}$ ∥ [001] and $\boldsymbol{B}$ ∥ [100] and strain ⊥ [010]<br>• $\boldsymbol{E}$ ∥ [001] and $\boldsymbol{B}$ ∥ [001] and strain ⊥ [010]<br>• $\boldsymbol{E}$ ∥ [001] and $\boldsymbol{B}$ ⊥ [010]<br>• $\boldsymbol{E}$ ⊥ [010] and $\boldsymbol{B}$ ∥ [100]<br>• $\boldsymbol{E}$ ⊥ [010] and $\boldsymbol{B}$ ∥ [001]<br>• $\boldsymbol{E}$ ⊥ [010] and $\boldsymbol{B}$ ⊥ [010]
P_ac (7.27) $\mathbb{Z}_1$	• $\boldsymbol{E}$ ∥ [100] and strain ⊥ [010]<br>• $\boldsymbol{E}$ ∥ [001] and strain ⊥ [010]<br>• $\boldsymbol{E}$ ⊥ [010]
$P2_1$ (4.7) $\mathbb{Z}_1$	• $\boldsymbol{E}$ ∥ [100] and $\boldsymbol{B}$ ∥ [100] and strain ⊥ [100]
$Pm'n'2_1$ (31.127) $\mathbb{Z}_1$	• $\boldsymbol{E}$ ∥ [100] and $\boldsymbol{B}$ ∥ [100]
$P2_1'$ (4.9) $\mathbb{Z}_1$	• $\boldsymbol{E}$ ∥ [100] and $\boldsymbol{B}$ ∥ [010] and strain ⊥ [100]<br>• $\boldsymbol{E}$ ∥ [100] and $\boldsymbol{B}$ ∥ [001] and strain ⊥ [100]<br>• $\boldsymbol{E}$ ∥ [100] and $\boldsymbol{B}$ ⊥ [100]
$Pm'c2_1'$ (26.68) $\mathbb{Z}_1$	• $\boldsymbol{E}$ ∥ [100] and $\boldsymbol{B}$ ∥ [010]
$Pmn'2_1'$ (31.126) $\mathbb{Z}_1$	• $\boldsymbol{E}$ ∥ [100] and $\boldsymbol{B}$ ∥ [001]
P_a2_1 (4.10) $\mathbb{Z}_1$	• $\boldsymbol{E}$ ∥ [100] and strain ⊥ [100]
P_amc2_1 (26.71) $\mathbb{Z}_1$	• $\boldsymbol{E}$ ∥ [100]
$P2$ (3.1) $\mathbb{Z}_2$	• $\boldsymbol{E}$ ∥ [001] and $\boldsymbol{B}$ ∥ [001] and strain ⊥ [001]
$P2_1'$ (4.9) $\mathbb{Z}_1$	• $\boldsymbol{E}$ ∥ [001] and $\boldsymbol{B}$ ∥ [100] and strain ⊥ [001]<br>• $\boldsymbol{E}$ ∥ [001] and $\boldsymbol{B}$ ∥ [010] and strain ⊥ [001]<br>• $\boldsymbol{E}$ ∥ [001] and $\boldsymbol{B}$ ⊥ [001]
P_b2 (3.5) $\mathbb{Z}_2$	• $\boldsymbol{E}$ ∥ [001] and strain ⊥ [001]

Continued on next page

...continued

Subgroup & SI group	Perturbation
$P\bar{1}$ (2.4) $\mathbb{Z}_2 \times \mathbb{Z}_2 \times \mathbb{Z}_2 \times \mathbb{Z}_4$	• $\boldsymbol{B} \parallel [100]$ and strain in generic direction<br>• $\boldsymbol{B} \parallel [010]$ and strain in generic direction<br>• $\boldsymbol{B} \parallel [001]$ and strain in generic direction<br>• $\boldsymbol{B} \perp [100]$ and strain $\perp [010]$<br>• $\boldsymbol{B} \perp [100]$ and strain $\perp [001]$<br>• $\boldsymbol{B} \perp [100]$ and strain in generic direction<br>• $\boldsymbol{B} \perp [010]$ and strain $\perp [100]$<br>• $\boldsymbol{B} \perp [010]$ and strain $\perp [001]$<br>• $\boldsymbol{B} \perp [010]$ and strain in generic direction<br>• $\boldsymbol{B} \perp [001]$ and strain $\perp [100]$<br>• $\boldsymbol{B} \perp [001]$ and strain $\perp [010]$<br>• $\boldsymbol{B} \perp [001]$ and strain in generic direction<br>• $\boldsymbol{B}$ in generic direction
$P2/m$ (10.42) $\mathbb{Z}_2 \times \mathbb{Z}_2 \times \mathbb{Z}_2$	• $\boldsymbol{B} \parallel [001]$ and strain $\perp [001]$
$P2_1'/m'$ (11.54) $\mathbb{Z}_2 \times \mathbb{Z}_2 \times \mathbb{Z}_4$	• $\boldsymbol{B} \parallel [100]$ and strain $\perp [001]$<br>• $\boldsymbol{B} \parallel [010]$ and strain $\perp [001]$<br>• $\boldsymbol{B} \perp [001]$
$P_S\bar{1}$ (2.7) $\mathbb{Z}_2 \times \mathbb{Z}_2 \times \mathbb{Z}_4$	• strain in generic direction
P_b2/m (10.48) $\mathbb{Z}_2 \times \mathbb{Z}_2$	• strain $\perp [001]$
Pc (7.24) $\mathbb{Z}_1$	• $\boldsymbol{E} \parallel [010]$ and $\boldsymbol{B} \parallel [100]$ and strain $\perp [100]$<br>• $\boldsymbol{E} \parallel [001]$ and $\boldsymbol{B} \parallel [100]$ and strain $\perp [100]$<br>• $\boldsymbol{E} \perp [100]$ and $\boldsymbol{B} \parallel [100]$
$Pn'a2_1'$ (33.146) $\mathbb{Z}_1$	• $\boldsymbol{E} \parallel [001]$ and $\boldsymbol{B} \parallel [100]$
$P2_1/c$ (14.75) $\mathbb{Z}_2$	• $\boldsymbol{B} \parallel [100]$ and strain $\perp [100]$
Pc' (7.26) $\mathbb{Z}_1$	• $\boldsymbol{E} \parallel [010]$ and $\boldsymbol{B} \parallel [010]$ and strain $\perp [100]$<br>• $\boldsymbol{E} \parallel [010]$ and $\boldsymbol{B} \parallel [001]$ and strain $\perp [100]$<br>• $\boldsymbol{E} \parallel [010]$ and $\boldsymbol{B} \perp [100]$<br>• $\boldsymbol{E} \parallel [001]$ and $\boldsymbol{B} \parallel [010]$ and strain $\perp [100]$<br>• $\boldsymbol{E} \parallel [001]$ and $\boldsymbol{B} \parallel [001]$ and strain $\perp [100]$<br>• $\boldsymbol{E} \parallel [001]$ and $\boldsymbol{B} \perp [100]$<br>• $\boldsymbol{E} \perp [100]$ and $\boldsymbol{B} \parallel [010]$<br>• $\boldsymbol{E} \perp [100]$ and $\boldsymbol{B} \parallel [001]$<br>• $\boldsymbol{E} \perp [100]$ and $\boldsymbol{B} \perp [100]$
$Pn'n'2$ (34.159) $\mathbb{Z}_1$	• $\boldsymbol{E} \parallel [001]$ and $\boldsymbol{B} \parallel [001]$
$Pn'a2_1'$ (33.146) $\mathbb{Z}_1$	• $\boldsymbol{E} \parallel [001]$ and $\boldsymbol{B} \parallel [010]$

Continued on next page

. . . continued

Subgroup & SI group	Perturbation
$P2_1'/c'$ (14.79) $\mathbb{Z}_2 \times \mathbb{Z}_4$	• $\boldsymbol{B}$ ∥ [010] and strain ⊥ [100]<br>• $\boldsymbol{B}$ ∥ [001] and strain ⊥ [100]<br>• $\boldsymbol{B}$ ⊥ [100]
$P_a c$ (7.27) $\mathbb{Z}_1$	• $\boldsymbol{E}$ ∥ [010] and strain ⊥ [100]<br>• $\boldsymbol{E}$ ∥ [001] and strain ⊥ [100]<br>• $\boldsymbol{E}$ ⊥ [100]
$P_c ba2$ (32.139) $\mathbb{Z}_2$	• $\boldsymbol{E}$ ∥ [001]
$P_a 2_1/c$ (14.80) $\mathbb{Z}_2$	• strain ⊥ [100]
$P2_1$ (4.7) $\mathbb{Z}_1$	• $\boldsymbol{E}$ ∥ [010] and $\boldsymbol{B}$ ∥ [010] and strain ⊥ [010]
$P2_1/c$ (14.75) $\mathbb{Z}_2$	• $\boldsymbol{B}$ ∥ [010] and strain ⊥ [010]
$Pm'n'2_1$ (31.127) $\mathbb{Z}_1$	• $\boldsymbol{E}$ ∥ [010] and $\boldsymbol{B}$ ∥ [010]
$Pn'm'a$ (62.446) $\mathbb{Z}_2$	• $\boldsymbol{B}$ ∥ [010]
$P2_1'$ (4.9) $\mathbb{Z}_1$	• $\boldsymbol{E}$ ∥ [010] and $\boldsymbol{B}$ ∥ [100] and strain ⊥ [010]<br>• $\boldsymbol{E}$ ∥ [010] and $\boldsymbol{B}$ ∥ [001] and strain ⊥ [010]<br>• $\boldsymbol{E}$ ∥ [010] and $\boldsymbol{B}$ ⊥ [010]
$P2_1'/c'$ (14.79) $\mathbb{Z}_2 \times \mathbb{Z}_4$	• $\boldsymbol{B}$ ∥ [100] and strain ⊥ [010]<br>• $\boldsymbol{B}$ ∥ [001] and strain ⊥ [010]<br>• $\boldsymbol{B}$ ⊥ [010]
$Pm'c2_1'$ (26.68) $\mathbb{Z}_1$	• $\boldsymbol{E}$ ∥ [010] and $\boldsymbol{B}$ ∥ [100]
$Pn'm'a$ (62.446) $\mathbb{Z}_2$	• $\boldsymbol{B}$ ∥ [100]
$Pmn'2_1'$ (31.126) $\mathbb{Z}_1$	• $\boldsymbol{E}$ ∥ [010] and $\boldsymbol{B}$ ∥ [001]
$Pn'n'm$ (58.397) $\mathbb{Z}_2 \times \mathbb{Z}_2$	• $\boldsymbol{B}$ ∥ [001]
$P_a 2_1$ (4.10) $\mathbb{Z}_1$	• $\boldsymbol{E}$ ∥ [010] and strain ⊥ [010]
$P_a 2_1/c$ (14.80) $\mathbb{Z}_2$	• strain ⊥ [010]
$P_a mc2_1$ (26.71) $\mathbb{Z}_1$	• $\boldsymbol{E}$ ∥ [010]

Table. S 472: Subgroups of $P_A bam$ (55.362) that can be realized by applying $\boldsymbol{E}/\boldsymbol{B}$ fields and/or uniaxial strains.

Subgroup & SI group	Perturbation

Continued on next page

...continued

Subgroup & SI group	Perturbation
Pm (6.18) $\mathbb{Z}_1$	• $\boldsymbol{E}$ ∥ [100] and $\boldsymbol{B}$ ∥ [001] and strain ⊥ [001]<br>• $\boldsymbol{E}$ ∥ [010] and $\boldsymbol{B}$ ∥ [001] and strain ⊥ [001]<br>• $\boldsymbol{E}$ ⊥ [001] and $\boldsymbol{B}$ ∥ [001]
P_S1 (1.3) $\mathbb{Z}_1$	• $\boldsymbol{E}$ ∥ [100] and strain in generic direction<br>• $\boldsymbol{E}$ ∥ [010] and strain in generic direction<br>• $\boldsymbol{E}$ ∥ [001] and strain in generic direction<br>• $\boldsymbol{E}$ ⊥ [100] and strain ⊥ [010]<br>• $\boldsymbol{E}$ ⊥ [100] and strain ⊥ [001]<br>• $\boldsymbol{E}$ ⊥ [100] and strain in generic direction<br>• $\boldsymbol{E}$ ⊥ [010] and strain ⊥ [100]<br>• $\boldsymbol{E}$ ⊥ [010] and strain ⊥ [001]<br>• $\boldsymbol{E}$ ⊥ [010] and strain in generic direction<br>• $\boldsymbol{E}$ ⊥ [001] and strain ⊥ [100]<br>• $\boldsymbol{E}$ ⊥ [001] and strain ⊥ [010]<br>• $\boldsymbol{E}$ ⊥ [001] and strain in generic direction<br>• $\boldsymbol{E}$ in generic direction
Pc' (7.26) $\mathbb{Z}_1$	• $\boldsymbol{E}$ ∥ [100] and $\boldsymbol{B}$ ∥ [100] and strain ⊥ [001]<br>• $\boldsymbol{E}$ ∥ [100] and $\boldsymbol{B}$ ∥ [010] and strain ⊥ [001]<br>• $\boldsymbol{E}$ ∥ [100] and $\boldsymbol{B}$ ⊥ [001]<br>• $\boldsymbol{E}$ ∥ [010] and $\boldsymbol{B}$ ∥ [100] and strain ⊥ [001]<br>• $\boldsymbol{E}$ ∥ [010] and $\boldsymbol{B}$ ∥ [010] and strain ⊥ [001]<br>• $\boldsymbol{E}$ ∥ [010] and $\boldsymbol{B}$ ⊥ [001]<br>• $\boldsymbol{E}$ ⊥ [001] and $\boldsymbol{B}$ ∥ [100]<br>• $\boldsymbol{E}$ ⊥ [001] and $\boldsymbol{B}$ ∥ [010]<br>• $\boldsymbol{E}$ ⊥ [001] and $\boldsymbol{B}$ ⊥ [001]
P_Cm (6.23) $\mathbb{Z}_1$	• $\boldsymbol{E}$ ∥ [100] and strain ⊥ [001]<br>• $\boldsymbol{E}$ ∥ [010] and strain ⊥ [001]<br>• $\boldsymbol{E}$ ⊥ [001]
Pc' (7.26) $\mathbb{Z}_1$	• $\boldsymbol{E}$ ∥ [100] and $\boldsymbol{B}$ ∥ [100] and strain ⊥ [010]<br>• $\boldsymbol{E}$ ∥ [100] and $\boldsymbol{B}$ ∥ [001] and strain ⊥ [010]<br>• $\boldsymbol{E}$ ∥ [100] and $\boldsymbol{B}$ ⊥ [010]<br>• $\boldsymbol{E}$ ∥ [001] and $\boldsymbol{B}$ ∥ [100] and strain ⊥ [010]<br>• $\boldsymbol{E}$ ∥ [001] and $\boldsymbol{B}$ ∥ [001] and strain ⊥ [010]<br>• $\boldsymbol{E}$ ∥ [001] and $\boldsymbol{B}$ ⊥ [010]<br>• $\boldsymbol{E}$ ⊥ [010] and $\boldsymbol{B}$ ∥ [100]<br>• $\boldsymbol{E}$ ⊥ [010] and $\boldsymbol{B}$ ∥ [001]<br>• $\boldsymbol{E}$ ⊥ [010] and $\boldsymbol{B}$ ⊥ [010]

Continued on next page

...continued

Subgroup & SI group	Perturbation
$P2_1'$ (4.9) $\mathbb{Z}_1$	• $\boldsymbol{E} \parallel [100]$ and $\boldsymbol{B} \parallel [010]$ and strain $\perp [100]$<br>• $\boldsymbol{E} \parallel [100]$ and $\boldsymbol{B} \parallel [001]$ and strain $\perp [100]$<br>• $\boldsymbol{E} \parallel [100]$ and $\boldsymbol{B} \perp [100]$
$Pmn'2_1'$ (31.126) $\mathbb{Z}_1$	• $\boldsymbol{E} \parallel [100]$ and $\boldsymbol{B} \parallel [001]$
Pc (7.24) $\mathbb{Z}_1$	• $\boldsymbol{E} \parallel [100]$ and $\boldsymbol{B} \parallel [010]$ and strain $\perp [010]$<br>• $\boldsymbol{E} \parallel [001]$ and $\boldsymbol{B} \parallel [010]$ and strain $\perp [010]$<br>• $\boldsymbol{E} \perp [010]$ and $\boldsymbol{B} \parallel [010]$
P_Cc (7.30) $\mathbb{Z}_1$	• $\boldsymbol{E} \parallel [100]$ and strain $\perp [010]$<br>• $\boldsymbol{E} \parallel [001]$ and strain $\perp [010]$<br>• $\boldsymbol{E} \perp [010]$
$Pca'2_1'$ (29.102) $\mathbb{Z}_1$	• $\boldsymbol{E} \parallel [100]$ and $\boldsymbol{B} \parallel [010]$
$P2_1$ (4.7) $\mathbb{Z}_1$	• $\boldsymbol{E} \parallel [100]$ and $\boldsymbol{B} \parallel [100]$ and strain $\perp [100]$
$Pn'a'2_1$ (33.148) $\mathbb{Z}_1$	• $\boldsymbol{E} \parallel [100]$ and $\boldsymbol{B} \parallel [100]$
P_a2_1 (4.10) $\mathbb{Z}_1$	• $\boldsymbol{E} \parallel [100]$ and strain $\perp [100]$
P_Cmc2_1 (26.76) $\mathbb{Z}_1$	• $\boldsymbol{E} \parallel [100]$
$P2$ (3.1) $\mathbb{Z}_2$	• $\boldsymbol{E} \parallel [001]$ and $\boldsymbol{B} \parallel [001]$ and strain $\perp [001]$
$P\bar{1}$ (2.4) $\mathbb{Z}_2 \times \mathbb{Z}_2 \times \mathbb{Z}_2 \times \mathbb{Z}_4$	• $\boldsymbol{B} \parallel [100]$ and strain in generic direction<br>• $\boldsymbol{B} \parallel [010]$ and strain in generic direction<br>• $\boldsymbol{B} \parallel [001]$ and strain in generic direction<br>• $\boldsymbol{B} \perp [100]$ and strain $\perp [010]$<br>• $\boldsymbol{B} \perp [100]$ and strain $\perp [001]$<br>• $\boldsymbol{B} \perp [100]$ and strain in generic direction<br>• $\boldsymbol{B} \perp [010]$ and strain $\perp [100]$<br>• $\boldsymbol{B} \perp [010]$ and strain $\perp [001]$<br>• $\boldsymbol{B} \perp [010]$ and strain in generic direction<br>• $\boldsymbol{B} \perp [001]$ and strain $\perp [100]$<br>• $\boldsymbol{B} \perp [001]$ and strain $\perp [010]$<br>• $\boldsymbol{B} \perp [001]$ and strain in generic direction<br>• $\boldsymbol{B}$ in generic direction
$P2/m$ (10.42) $\mathbb{Z}_2 \times \mathbb{Z}_2 \times \mathbb{Z}_2$	• $\boldsymbol{B} \parallel [001]$ and strain $\perp [001]$
$P2_1'$ (4.9) $\mathbb{Z}_1$	• $\boldsymbol{E} \parallel [001]$ and $\boldsymbol{B} \parallel [100]$ and strain $\perp [001]$<br>• $\boldsymbol{E} \parallel [001]$ and $\boldsymbol{B} \parallel [010]$ and strain $\perp [001]$<br>• $\boldsymbol{E} \parallel [001]$ and $\boldsymbol{B} \perp [001]$
P_C2 (3.6) $\mathbb{Z}_1$	• $\boldsymbol{E} \parallel [001]$ and strain $\perp [001]$

Continued on next page

…continued

Subgroup & SI group	Perturbation
$P2_1'/c'$ (14.79) $\mathbb{Z}_2 \times \mathbb{Z}_4$	• $\boldsymbol{B}$ ∥ [100] and strain ⊥ [001]<br>• $\boldsymbol{B}$ ∥ [010] and strain ⊥ [001]<br>• $\boldsymbol{B}$ ⊥ [001]
$P_S\bar{1}$ (2.7) $\mathbb{Z}_2 \times \mathbb{Z}_2 \times \mathbb{Z}_4$	• strain in generic direction
P_C2/m (10.49) $\mathbb{Z}_2 \times \mathbb{Z}_2$	• strain ⊥ [001]
Pc' (7.26) $\mathbb{Z}_1$	• $\boldsymbol{E}$ ∥ [010] and $\boldsymbol{B}$ ∥ [010] and strain ⊥ [100]<br>• $\boldsymbol{E}$ ∥ [010] and $\boldsymbol{B}$ ∥ [001] and strain ⊥ [100]<br>• $\boldsymbol{E}$ ∥ [010] and $\boldsymbol{B}$ ⊥ [100]<br>• $\boldsymbol{E}$ ∥ [001] and $\boldsymbol{B}$ ∥ [010] and strain ⊥ [100]<br>• $\boldsymbol{E}$ ∥ [001] and $\boldsymbol{B}$ ∥ [001] and strain ⊥ [100]<br>• $\boldsymbol{E}$ ∥ [001] and $\boldsymbol{B}$ ⊥ [100]<br>• $\boldsymbol{E}$ ⊥ [100] and $\boldsymbol{B}$ ∥ [010]<br>• $\boldsymbol{E}$ ⊥ [100] and $\boldsymbol{B}$ ∥ [001]<br>• $\boldsymbol{E}$ ⊥ [100] and $\boldsymbol{B}$ ⊥ [100]
$Pn'c'2$ (30.115) $\mathbb{Z}_1$	• $\boldsymbol{E}$ ∥ [001] and $\boldsymbol{B}$ ∥ [001]
$P2_1'/c'$ (14.79) $\mathbb{Z}_2 \times \mathbb{Z}_4$	• $\boldsymbol{B}$ ∥ [010] and strain ⊥ [100]<br>• $\boldsymbol{B}$ ∥ [001] and strain ⊥ [100]<br>• $\boldsymbol{B}$ ⊥ [100]
$Pc'a2_1'$ (29.101) $\mathbb{Z}_1$	• $\boldsymbol{E}$ ∥ [001] and $\boldsymbol{B}$ ∥ [010]
$P2'$ (3.3) $\mathbb{Z}_1$	• $\boldsymbol{E}$ ∥ [010] and $\boldsymbol{B}$ ∥ [100] and strain ⊥ [010]<br>• $\boldsymbol{E}$ ∥ [010] and $\boldsymbol{B}$ ∥ [001] and strain ⊥ [010]<br>• $\boldsymbol{E}$ ∥ [010] and $\boldsymbol{B}$ ⊥ [010]
$P2'/c'$ (13.69) $\mathbb{Z}_2 \times \mathbb{Z}_2 \times \mathbb{Z}_4$	• $\boldsymbol{B}$ ∥ [100] and strain ⊥ [010]<br>• $\boldsymbol{B}$ ∥ [001] and strain ⊥ [010]<br>• $\boldsymbol{B}$ ⊥ [010]
$Pma'2'$ (28.90) $\mathbb{Z}_1$	• $\boldsymbol{E}$ ∥ [010] and $\boldsymbol{B}$ ∥ [001]
$Pmn'a'$ (53.327) $\mathbb{Z}_2 \times \mathbb{Z}_2$	• $\boldsymbol{B}$ ∥ [001]
Pc (7.24) $\mathbb{Z}_1$	• $\boldsymbol{E}$ ∥ [010] and $\boldsymbol{B}$ ∥ [100] and strain ⊥ [100]<br>• $\boldsymbol{E}$ ∥ [001] and $\boldsymbol{B}$ ∥ [100] and strain ⊥ [100]<br>• $\boldsymbol{E}$ ⊥ [100] and $\boldsymbol{B}$ ∥ [100]
$P2_1/c$ (14.75) $\mathbb{Z}_2$	• $\boldsymbol{B}$ ∥ [100] and strain ⊥ [100]
$Pn'a2_1'$ (33.146) $\mathbb{Z}_1$	• $\boldsymbol{E}$ ∥ [001] and $\boldsymbol{B}$ ∥ [100]
P_ac (7.27) $\mathbb{Z}_1$	• $\boldsymbol{E}$ ∥ [010] and strain ⊥ [100]<br>• $\boldsymbol{E}$ ∥ [001] and strain ⊥ [100]<br>• $\boldsymbol{E}$ ⊥ [100]

Continued on next page

...continued

Subgroup & SI group	Perturbation
$P_{A}ba2$ (32.142) $\mathbb{Z}_1$	• $\boldsymbol{E} \parallel [001]$
$P_{a}2_1/c$ (14.80) $\mathbb{Z}_2$	• strain $\perp$ [100]
$Pc'c2'$ (27.80) $\mathbb{Z}_1$	• $\boldsymbol{E} \parallel [010]$ and $\boldsymbol{B} \parallel [100]$
$Pc'cn'$ (56.370) $\mathbb{Z}_2$	• $\boldsymbol{B} \parallel [100]$
$P2_1$ (4.7) $\mathbb{Z}_1$	• $\boldsymbol{E} \parallel [010]$ and $\boldsymbol{B} \parallel [010]$ and strain $\perp$ [010]
$P2_1/c$ (14.75) $\mathbb{Z}_2$	• $\boldsymbol{B} \parallel [010]$ and strain $\perp$ [010]
$Pc'a'2_1$ (29.103) $\mathbb{Z}_1$	• $\boldsymbol{E} \parallel [010]$ and $\boldsymbol{B} \parallel [010]$
$Pb'c'a$ (61.436) $\mathbb{Z}_2$	• $\boldsymbol{B} \parallel [010]$
$P_{C}2_1$ (4.12) $\mathbb{Z}_1$	• $\boldsymbol{E} \parallel [010]$ and strain $\perp$ [010]
$P_{C}2_1/c$ (14.84) $\mathbb{Z}_2$	• strain $\perp$ [010]
$P_{B}mc2_1$ (26.75) $\mathbb{Z}_1$	• $\boldsymbol{E} \parallel [010]$

Table. S 473: Subgroups of $P_{C}bam$ (55.363) that can be realized by applying $\boldsymbol{E}/\boldsymbol{B}$ fields and/or uniaxial strains.

Subgroup & SI group	Perturbation
Pm (6.18) $\mathbb{Z}_1$	• $\boldsymbol{E} \parallel [100]$ and $\boldsymbol{B} \parallel [001]$ and strain $\perp$ [001]<br>• $\boldsymbol{E} \parallel [010]$ and $\boldsymbol{B} \parallel [001]$ and strain $\perp$ [001]<br>• $\boldsymbol{E} \perp [001]$ and $\boldsymbol{B} \parallel [001]$
Pm' (6.20) $\mathbb{Z}_1$	• $\boldsymbol{E} \parallel [100]$ and $\boldsymbol{B} \parallel [100]$ and strain $\perp$ [010]<br>• $\boldsymbol{E} \parallel [100]$ and $\boldsymbol{B} \parallel [001]$ and strain $\perp$ [010]<br>• $\boldsymbol{E} \parallel [100]$ and $\boldsymbol{B} \perp [010]$<br>• $\boldsymbol{E} \parallel [001]$ and $\boldsymbol{B} \parallel [100]$ and strain $\perp$ [010]<br>• $\boldsymbol{E} \parallel [001]$ and $\boldsymbol{B} \parallel [001]$ and strain $\perp$ [010]<br>• $\boldsymbol{E} \parallel [001]$ and $\boldsymbol{B} \perp [010]$<br>• $\boldsymbol{E} \perp [010]$ and $\boldsymbol{B} \parallel [100]$<br>• $\boldsymbol{E} \perp [010]$ and $\boldsymbol{B} \parallel [001]$<br>• $\boldsymbol{E} \perp [010]$ and $\boldsymbol{B} \perp [010]$
$P2'$ (3.3) $\mathbb{Z}_1$	• $\boldsymbol{E} \parallel [100]$ and $\boldsymbol{B} \parallel [010]$ and strain $\perp$ [100]<br>• $\boldsymbol{E} \parallel [100]$ and $\boldsymbol{B} \parallel [001]$ and strain $\perp$ [100]<br>• $\boldsymbol{E} \parallel [100]$ and $\boldsymbol{B} \perp [100]$
$Pm'm2'$ (25.59) $\mathbb{Z}_1$	• $\boldsymbol{E} \parallel [100]$ and $\boldsymbol{B} \parallel [001]$

Continued on next page

...continued

Subgroup & SI group	Perturbation
P_S1 (1.3) $\mathbb{Z}_1$	• $\boldsymbol{E} \parallel [100]$ and strain in generic direction<br>• $\boldsymbol{E} \parallel [010]$ and strain in generic direction<br>• $\boldsymbol{E} \parallel [001]$ and strain in generic direction<br>• $\boldsymbol{E} \perp [100]$ and strain $\perp [010]$<br>• $\boldsymbol{E} \perp [100]$ and strain $\perp [001]$<br>• $\boldsymbol{E} \perp [100]$ and strain in generic direction<br>• $\boldsymbol{E} \perp [010]$ and strain $\perp [100]$<br>• $\boldsymbol{E} \perp [010]$ and strain $\perp [001]$<br>• $\boldsymbol{E} \perp [010]$ and strain in generic direction<br>• $\boldsymbol{E} \perp [001]$ and strain $\perp [100]$<br>• $\boldsymbol{E} \perp [001]$ and strain $\perp [010]$<br>• $\boldsymbol{E} \perp [001]$ and strain in generic direction<br>• $\boldsymbol{E}$ in generic direction
Pc' (7.26) $\mathbb{Z}_1$	• $\boldsymbol{E} \parallel [100]$ and $\boldsymbol{B} \parallel [100]$ and strain $\perp [001]$<br>• $\boldsymbol{E} \parallel [100]$ and $\boldsymbol{B} \parallel [010]$ and strain $\perp [001]$<br>• $\boldsymbol{E} \parallel [100]$ and $\boldsymbol{B} \perp [001]$<br>• $\boldsymbol{E} \parallel [010]$ and $\boldsymbol{B} \parallel [100]$ and strain $\perp [001]$<br>• $\boldsymbol{E} \parallel [010]$ and $\boldsymbol{B} \parallel [010]$ and strain $\perp [001]$<br>• $\boldsymbol{E} \parallel [010]$ and $\boldsymbol{B} \perp [001]$<br>• $\boldsymbol{E} \perp [001]$ and $\boldsymbol{B} \parallel [100]$<br>• $\boldsymbol{E} \perp [001]$ and $\boldsymbol{B} \parallel [010]$<br>• $\boldsymbol{E} \perp [001]$ and $\boldsymbol{B} \perp [001]$
P_am (6.21) $\mathbb{Z}_1$	• $\boldsymbol{E} \parallel [100]$ and strain $\perp [001]$<br>• $\boldsymbol{E} \parallel [010]$ and strain $\perp [001]$<br>• $\boldsymbol{E} \perp [001]$
Pc (7.24) $\mathbb{Z}_1$	• $\boldsymbol{E} \parallel [100]$ and $\boldsymbol{B} \parallel [010]$ and strain $\perp [010]$<br>• $\boldsymbol{E} \parallel [001]$ and $\boldsymbol{B} \parallel [010]$ and strain $\perp [010]$<br>• $\boldsymbol{E} \perp [010]$ and $\boldsymbol{B} \parallel [010]$
P_Ac (7.31) $\mathbb{Z}_1$	• $\boldsymbol{E} \parallel [100]$ and strain $\perp [010]$<br>• $\boldsymbol{E} \parallel [001]$ and strain $\perp [010]$<br>• $\boldsymbol{E} \perp [010]$
$Pn'c2'$ (30.113) $\mathbb{Z}_1$	• $\boldsymbol{E} \parallel [100]$ and $\boldsymbol{B} \parallel [010]$
$P2_1$ (4.7) $\mathbb{Z}_1$	• $\boldsymbol{E} \parallel [100]$ and $\boldsymbol{B} \parallel [100]$ and strain $\perp [100]$
P_C2_1 (4.12) $\mathbb{Z}_1$	• $\boldsymbol{E} \parallel [100]$ and strain $\perp [100]$
$Pm'n'2_1$ (31.127) $\mathbb{Z}_1$	• $\boldsymbol{E} \parallel [100]$ and $\boldsymbol{B} \parallel [100]$
P_Amc2_1 (26.74) $\mathbb{Z}_1$	• $\boldsymbol{E} \parallel [100]$

Continued on next page

...continued

Subgroup & SI group	Perturbation
Pm' (6.20) $\mathbb{Z}_1$	• $\boldsymbol{E} \parallel [010]$ and $\boldsymbol{B} \parallel [010]$ and strain $\perp [100]$<br>• $\boldsymbol{E} \parallel [010]$ and $\boldsymbol{B} \parallel [001]$ and strain $\perp [100]$<br>• $\boldsymbol{E} \parallel [010]$ and $\boldsymbol{B} \perp [100]$<br>• $\boldsymbol{E} \parallel [001]$ and $\boldsymbol{B} \parallel [010]$ and strain $\perp [100]$<br>• $\boldsymbol{E} \parallel [001]$ and $\boldsymbol{B} \parallel [001]$ and strain $\perp [100]$<br>• $\boldsymbol{E} \parallel [001]$ and $\boldsymbol{B} \perp [100]$<br>• $\boldsymbol{E} \perp [100]$ and $\boldsymbol{B} \parallel [010]$<br>• $\boldsymbol{E} \perp [100]$ and $\boldsymbol{B} \parallel [001]$<br>• $\boldsymbol{E} \perp [100]$ and $\boldsymbol{B} \perp [100]$
$P2'$ (3.3) $\mathbb{Z}_1$	• $\boldsymbol{E} \parallel [010]$ and $\boldsymbol{B} \parallel [100]$ and strain $\perp [010]$<br>• $\boldsymbol{E} \parallel [010]$ and $\boldsymbol{B} \parallel [001]$ and strain $\perp [010]$<br>• $\boldsymbol{E} \parallel [010]$ and $\boldsymbol{B} \perp [010]$
$Pm'm2'$ (25.59) $\mathbb{Z}_1$	• $\boldsymbol{E} \parallel [010]$ and $\boldsymbol{B} \parallel [001]$
$P2$ (3.1) $\mathbb{Z}_2$	• $\boldsymbol{E} \parallel [001]$ and $\boldsymbol{B} \parallel [001]$ and strain $\perp [001]$
$Pm'm'2$ (25.60) $\mathbb{Z}_2$	• $\boldsymbol{E} \parallel [001]$ and $\boldsymbol{B} \parallel [001]$
$P\bar{1}$ (2.4) $\mathbb{Z}_2 \times \mathbb{Z}_2 \times \mathbb{Z}_2 \times \mathbb{Z}_4$	• $\boldsymbol{B} \parallel [100]$ and strain in generic direction<br>• $\boldsymbol{B} \parallel [010]$ and strain in generic direction<br>• $\boldsymbol{B} \parallel [001]$ and strain in generic direction<br>• $\boldsymbol{B} \perp [100]$ and strain $\perp [010]$<br>• $\boldsymbol{B} \perp [100]$ and strain $\perp [001]$<br>• $\boldsymbol{B} \perp [100]$ and strain in generic direction<br>• $\boldsymbol{B} \perp [010]$ and strain $\perp [100]$<br>• $\boldsymbol{B} \perp [010]$ and strain $\perp [001]$<br>• $\boldsymbol{B} \perp [010]$ and strain in generic direction<br>• $\boldsymbol{B} \perp [001]$ and strain $\perp [100]$<br>• $\boldsymbol{B} \perp [001]$ and strain $\perp [010]$<br>• $\boldsymbol{B} \perp [001]$ and strain in generic direction<br>• $\boldsymbol{B}$ in generic direction
$P2'/m'$ (10.46) $\mathbb{Z}_2 \times \mathbb{Z}_2 \times \mathbb{Z}_2 \times \mathbb{Z}_4$	• $\boldsymbol{B} \parallel [010]$ and strain $\perp [100]$<br>• $\boldsymbol{B} \parallel [001]$ and strain $\perp [100]$<br>• $\boldsymbol{B} \perp [100]$
$P2'/m'$ (10.46) $\mathbb{Z}_2 \times \mathbb{Z}_2 \times \mathbb{Z}_2 \times \mathbb{Z}_4$	• $\boldsymbol{B} \parallel [100]$ and strain $\perp [010]$<br>• $\boldsymbol{B} \parallel [001]$ and strain $\perp [010]$<br>• $\boldsymbol{B} \perp [010]$
$P2/m$ (10.42) $\mathbb{Z}_2 \times \mathbb{Z}_2 \times \mathbb{Z}_2$	• $\boldsymbol{B} \parallel [001]$ and strain $\perp [001]$
$Pm'm'm$ (47.252) $\mathbb{Z}_2 \times \mathbb{Z}_2 \times \mathbb{Z}_2$	• $\boldsymbol{B} \parallel [001]$

Continued on next page

...continued

Subgroup & SI group	Perturbation
Pc (7.24) $\mathbb{Z}_1$	• $\boldsymbol{E} \parallel [010]$ and $\boldsymbol{B} \parallel [100]$ and strain $\perp [100]$<br>• $\boldsymbol{E} \parallel [001]$ and $\boldsymbol{B} \parallel [100]$ and strain $\perp [100]$<br>• $\boldsymbol{E} \perp [100]$ and $\boldsymbol{B} \parallel [100]$
P_Ac (7.31) $\mathbb{Z}_1$	• $\boldsymbol{E} \parallel [010]$ and strain $\perp [100]$<br>• $\boldsymbol{E} \parallel [001]$ and strain $\perp [100]$<br>• $\boldsymbol{E} \perp [100]$
$Pn'c2'$ (30.113) $\mathbb{Z}_1$	• $\boldsymbol{E} \parallel [010]$ and $\boldsymbol{B} \parallel [100]$
$P2_1/c$ (14.75) $\mathbb{Z}_2$	• $\boldsymbol{B} \parallel [100]$ and strain $\perp [100]$
$P2_1$ (4.7) $\mathbb{Z}_1$	• $\boldsymbol{E} \parallel [010]$ and $\boldsymbol{B} \parallel [010]$ and strain $\perp [010]$
$Pm'n'2_1$ (31.127) $\mathbb{Z}_1$	• $\boldsymbol{E} \parallel [010]$ and $\boldsymbol{B} \parallel [010]$
P_C2_1 (4.12) $\mathbb{Z}_1$	• $\boldsymbol{E} \parallel [010]$ and strain $\perp [010]$
$P2_1/c$ (14.75) $\mathbb{Z}_2$	• $\boldsymbol{B} \parallel [010]$ and strain $\perp [010]$
P_Amc2_1 (26.74) $\mathbb{Z}_1$	• $\boldsymbol{E} \parallel [010]$
$P2'$ (3.3) $\mathbb{Z}_1$	• $\boldsymbol{E} \parallel [001]$ and $\boldsymbol{B} \parallel [100]$ and strain $\perp [001]$<br>• $\boldsymbol{E} \parallel [001]$ and $\boldsymbol{B} \parallel [010]$ and strain $\perp [001]$<br>• $\boldsymbol{E} \parallel [001]$ and $\boldsymbol{B} \perp [001]$
$Pm'a2'$ (28.89) $\mathbb{Z}_1$	• $\boldsymbol{E} \parallel [001]$ and $\boldsymbol{B} \parallel [010]$
P_a2 (3.4) $\mathbb{Z}_2$	• $\boldsymbol{E} \parallel [001]$ and strain $\perp [001]$
$P2'/c'$ (13.69) $\mathbb{Z}_2 \times \mathbb{Z}_2 \times \mathbb{Z}_4$	• $\boldsymbol{B} \parallel [100]$ and strain $\perp [001]$<br>• $\boldsymbol{B} \parallel [010]$ and strain $\perp [001]$<br>• $\boldsymbol{B} \perp [001]$
$Pm'a2'$ (28.89) $\mathbb{Z}_1$	• $\boldsymbol{E} \parallel [001]$ and $\boldsymbol{B} \parallel [100]$
P_Cba2 (32.141) $\mathbb{Z}_1$	• $\boldsymbol{E} \parallel [001]$
$Pm'n'a$ (53.326) $\mathbb{Z}_2$	• $\boldsymbol{B} \parallel [100]$
$Pm'n'a$ (53.326) $\mathbb{Z}_2$	• $\boldsymbol{B} \parallel [010]$
$P_S\bar{1}$ (2.7) $\mathbb{Z}_2 \times \mathbb{Z}_2 \times \mathbb{Z}_4$	• strain in generic direction
P_A2_1/c (14.83) $\mathbb{Z}_2$	• strain $\perp [100]$
P_A2_1/c (14.83) $\mathbb{Z}_2$	• strain $\perp [010]$
P_a2/m (10.47) $\mathbb{Z}_2 \times \mathbb{Z}_2 \times \mathbb{Z}_2$	• strain $\perp [001]$

Table. S 474: Subgroups of $P_I bam$ (55.364) that can be realized by applying $\boldsymbol{E}/\boldsymbol{B}$ fields and/or uniaxial strains.

Subgroup & SI group	Perturbation
Pm (6.18) $\mathbb{Z}_1$	• $\boldsymbol{E}$ ∥ [100] and $\boldsymbol{B}$ ∥ [001] and strain ⊥ [001]<br>• $\boldsymbol{E}$ ∥ [010] and $\boldsymbol{B}$ ∥ [001] and strain ⊥ [001]<br>• $\boldsymbol{E}$ ⊥ [001] and $\boldsymbol{B}$ ∥ [001]
Pc' (7.26) $\mathbb{Z}_1$	• $\boldsymbol{E}$ ∥ [100] and $\boldsymbol{B}$ ∥ [100] and strain ⊥ [010]<br>• $\boldsymbol{E}$ ∥ [100] and $\boldsymbol{B}$ ∥ [001] and strain ⊥ [010]<br>• $\boldsymbol{E}$ ∥ [100] and $\boldsymbol{B}$ ⊥ [010]<br>• $\boldsymbol{E}$ ∥ [001] and $\boldsymbol{B}$ ∥ [100] and strain ⊥ [010]<br>• $\boldsymbol{E}$ ∥ [001] and $\boldsymbol{B}$ ∥ [001] and strain ⊥ [010]<br>• $\boldsymbol{E}$ ∥ [001] and $\boldsymbol{B}$ ⊥ [010]<br>• $\boldsymbol{E}$ ⊥ [010] and $\boldsymbol{B}$ ∥ [100]<br>• $\boldsymbol{E}$ ⊥ [010] and $\boldsymbol{B}$ ∥ [001]<br>• $\boldsymbol{E}$ ⊥ [010] and $\boldsymbol{B}$ ⊥ [010]
$P2'$ (3.3) $\mathbb{Z}_1$	• $\boldsymbol{E}$ ∥ [100] and $\boldsymbol{B}$ ∥ [010] and strain ⊥ [100]<br>• $\boldsymbol{E}$ ∥ [100] and $\boldsymbol{B}$ ∥ [001] and strain ⊥ [100]<br>• $\boldsymbol{E}$ ∥ [100] and $\boldsymbol{B}$ ⊥ [100]
$Pma'2'$ (28.90) $\mathbb{Z}_1$	• $\boldsymbol{E}$ ∥ [100] and $\boldsymbol{B}$ ∥ [001]
P_S1 (1.3) $\mathbb{Z}_1$	• $\boldsymbol{E}$ ∥ [100] and strain in generic direction<br>• $\boldsymbol{E}$ ∥ [010] and strain in generic direction<br>• $\boldsymbol{E}$ ∥ [001] and strain in generic direction<br>• $\boldsymbol{E}$ ⊥ [100] and strain ⊥ [010]<br>• $\boldsymbol{E}$ ⊥ [100] and strain ⊥ [001]<br>• $\boldsymbol{E}$ ⊥ [100] and strain in generic direction<br>• $\boldsymbol{E}$ ⊥ [010] and strain ⊥ [100]<br>• $\boldsymbol{E}$ ⊥ [010] and strain ⊥ [001]<br>• $\boldsymbol{E}$ ⊥ [010] and strain in generic direction<br>• $\boldsymbol{E}$ ⊥ [001] and strain ⊥ [100]<br>• $\boldsymbol{E}$ ⊥ [001] and strain ⊥ [010]<br>• $\boldsymbol{E}$ ⊥ [001] and strain in generic direction<br>• $\boldsymbol{E}$ in generic direction

Continued on next page

...continued

Subgroup & SI group	Perturbation
Pc' (7.26) $\mathbb{Z}_1$	• $\boldsymbol{E}$ ∥ [100] and $\boldsymbol{B}$ ∥ [100] and strain ⊥ [001]<br>• $\boldsymbol{E}$ ∥ [100] and $\boldsymbol{B}$ ∥ [010] and strain ⊥ [001]<br>• $\boldsymbol{E}$ ∥ [100] and $\boldsymbol{B}$ ⊥ [001]<br>• $\boldsymbol{E}$ ∥ [010] and $\boldsymbol{B}$ ∥ [100] and strain ⊥ [001]<br>• $\boldsymbol{E}$ ∥ [010] and $\boldsymbol{B}$ ∥ [010] and strain ⊥ [001]<br>• $\boldsymbol{E}$ ∥ [010] and $\boldsymbol{B}$ ⊥ [001]<br>• $\boldsymbol{E}$ ⊥ [001] and $\boldsymbol{B}$ ∥ [100]<br>• $\boldsymbol{E}$ ⊥ [001] and $\boldsymbol{B}$ ∥ [010]<br>• $\boldsymbol{E}$ ⊥ [001] and $\boldsymbol{B}$ ⊥ [001]
P_Cm (6.23) $\mathbb{Z}_1$	• $\boldsymbol{E}$ ∥ [100] and strain ⊥ [001]<br>• $\boldsymbol{E}$ ∥ [010] and strain ⊥ [001]<br>• $\boldsymbol{E}$ ⊥ [001]
Pc (7.24) $\mathbb{Z}_1$	• $\boldsymbol{E}$ ∥ [100] and $\boldsymbol{B}$ ∥ [010] and strain ⊥ [010]<br>• $\boldsymbol{E}$ ∥ [001] and $\boldsymbol{B}$ ∥ [010] and strain ⊥ [010]<br>• $\boldsymbol{E}$ ⊥ [010] and $\boldsymbol{B}$ ∥ [010]
P_Cc (7.30) $\mathbb{Z}_1$	• $\boldsymbol{E}$ ∥ [100] and strain ⊥ [010]<br>• $\boldsymbol{E}$ ∥ [001] and strain ⊥ [010]<br>• $\boldsymbol{E}$ ⊥ [010]
$Pn'c2'$ (30.113) $\mathbb{Z}_1$	• $\boldsymbol{E}$ ∥ [100] and $\boldsymbol{B}$ ∥ [010]
$P2_1$ (4.7) $\mathbb{Z}_1$	• $\boldsymbol{E}$ ∥ [100] and $\boldsymbol{B}$ ∥ [100] and strain ⊥ [100]
P_C2_1 (4.12) $\mathbb{Z}_1$	• $\boldsymbol{E}$ ∥ [100] and strain ⊥ [100]
$Pn'a'2_1$ (33.148) $\mathbb{Z}_1$	• $\boldsymbol{E}$ ∥ [100] and $\boldsymbol{B}$ ∥ [100]
P_Imc2_1 (26.77) $\mathbb{Z}_1$	• $\boldsymbol{E}$ ∥ [100]
Pc' (7.26) $\mathbb{Z}_1$	• $\boldsymbol{E}$ ∥ [010] and $\boldsymbol{B}$ ∥ [010] and strain ⊥ [100]<br>• $\boldsymbol{E}$ ∥ [010] and $\boldsymbol{B}$ ∥ [001] and strain ⊥ [100]<br>• $\boldsymbol{E}$ ∥ [010] and $\boldsymbol{B}$ ⊥ [100]<br>• $\boldsymbol{E}$ ∥ [001] and $\boldsymbol{B}$ ∥ [010] and strain ⊥ [100]<br>• $\boldsymbol{E}$ ∥ [001] and $\boldsymbol{B}$ ∥ [001] and strain ⊥ [100]<br>• $\boldsymbol{E}$ ∥ [001] and $\boldsymbol{B}$ ⊥ [100]<br>• $\boldsymbol{E}$ ⊥ [100] and $\boldsymbol{B}$ ∥ [010]<br>• $\boldsymbol{E}$ ⊥ [100] and $\boldsymbol{B}$ ∥ [001]<br>• $\boldsymbol{E}$ ⊥ [100] and $\boldsymbol{B}$ ⊥ [100]
$P2'$ (3.3) $\mathbb{Z}_1$	• $\boldsymbol{E}$ ∥ [010] and $\boldsymbol{B}$ ∥ [100] and strain ⊥ [010]<br>• $\boldsymbol{E}$ ∥ [010] and $\boldsymbol{B}$ ∥ [001] and strain ⊥ [010]<br>• $\boldsymbol{E}$ ∥ [010] and $\boldsymbol{B}$ ⊥ [010]
$Pma'2'$ (28.90) $\mathbb{Z}_1$	• $\boldsymbol{E}$ ∥ [010] and $\boldsymbol{B}$ ∥ [001]

Continued on next page

...continued

Subgroup & SI group	Perturbation
$P2$ (3.1) $\mathbb{Z}_2$	• $\boldsymbol{E} \parallel [001]$ and $\boldsymbol{B} \parallel [001]$ and strain $\perp [001]$
$Pc'c'2$ (27.81) $\mathbb{Z}_2$	• $\boldsymbol{E} \parallel [001]$ and $\boldsymbol{B} \parallel [001]$
$P\bar{1}$ (2.4) $\mathbb{Z}_2 \times \mathbb{Z}_2 \times \mathbb{Z}_2 \times \mathbb{Z}_4$	• $\boldsymbol{B} \parallel [100]$ and strain in generic direction<br>• $\boldsymbol{B} \parallel [010]$ and strain in generic direction<br>• $\boldsymbol{B} \parallel [001]$ and strain in generic direction<br>• $\boldsymbol{B} \perp [100]$ and strain $\perp [010]$<br>• $\boldsymbol{B} \perp [100]$ and strain $\perp [001]$<br>• $\boldsymbol{B} \perp [100]$ and strain in generic direction<br>• $\boldsymbol{B} \perp [010]$ and strain $\perp [100]$<br>• $\boldsymbol{B} \perp [010]$ and strain $\perp [001]$<br>• $\boldsymbol{B} \perp [010]$ and strain in generic direction<br>• $\boldsymbol{B} \perp [001]$ and strain $\perp [100]$<br>• $\boldsymbol{B} \perp [001]$ and strain $\perp [010]$<br>• $\boldsymbol{B} \perp [001]$ and strain in generic direction<br>• $\boldsymbol{B}$ in generic direction
$P2'/c'$ (13.69) $\mathbb{Z}_2 \times \mathbb{Z}_2 \times \mathbb{Z}_4$	• $\boldsymbol{B} \parallel [010]$ and strain $\perp [100]$<br>• $\boldsymbol{B} \parallel [001]$ and strain $\perp [100]$<br>• $\boldsymbol{B} \perp [100]$
$P2'/c'$ (13.69) $\mathbb{Z}_2 \times \mathbb{Z}_2 \times \mathbb{Z}_4$	• $\boldsymbol{B} \parallel [100]$ and strain $\perp [010]$<br>• $\boldsymbol{B} \parallel [001]$ and strain $\perp [010]$<br>• $\boldsymbol{B} \perp [010]$
$P2/m$ (10.42) $\mathbb{Z}_2 \times \mathbb{Z}_2 \times \mathbb{Z}_2$	• $\boldsymbol{B} \parallel [001]$ and strain $\perp [001]$
$Pc'c'm$ (49.269) $\mathbb{Z}_2 \times \mathbb{Z}_2$	• $\boldsymbol{B} \parallel [001]$
Pc (7.24) $\mathbb{Z}_1$	• $\boldsymbol{E} \parallel [010]$ and $\boldsymbol{B} \parallel [100]$ and strain $\perp [100]$<br>• $\boldsymbol{E} \parallel [001]$ and $\boldsymbol{B} \parallel [100]$ and strain $\perp [100]$<br>• $\boldsymbol{E} \perp [100]$ and $\boldsymbol{B} \parallel [100]$
P_Cc (7.30) $\mathbb{Z}_1$	• $\boldsymbol{E} \parallel [010]$ and strain $\perp [100]$<br>• $\boldsymbol{E} \parallel [001]$ and strain $\perp [100]$<br>• $\boldsymbol{E} \perp [100]$
$Pn'c2'$ (30.113) $\mathbb{Z}_1$	• $\boldsymbol{E} \parallel [010]$ and $\boldsymbol{B} \parallel [100]$
$P2_1/c$ (14.75) $\mathbb{Z}_2$	• $\boldsymbol{B} \parallel [100]$ and strain $\perp [100]$
$P2_1$ (4.7) $\mathbb{Z}_1$	• $\boldsymbol{E} \parallel [010]$ and $\boldsymbol{B} \parallel [010]$ and strain $\perp [010]$
$Pn'a'2_1$ (33.148) $\mathbb{Z}_1$	• $\boldsymbol{E} \parallel [010]$ and $\boldsymbol{B} \parallel [010]$
P_C2_1 (4.12) $\mathbb{Z}_1$	• $\boldsymbol{E} \parallel [010]$ and strain $\perp [010]$
$P2_1/c$ (14.75) $\mathbb{Z}_2$	• $\boldsymbol{B} \parallel [010]$ and strain $\perp [010]$

Continued on next page

…continued

Subgroup & SI group	Perturbation
P_Imc2_1 (26.77) $\mathbb{Z}_1$	• $\boldsymbol{E} \parallel [010]$
$P2_1'$ (4.9) $\mathbb{Z}_1$	• $\boldsymbol{E} \parallel [001]$ and $\boldsymbol{B} \parallel [100]$ and strain $\perp [001]$<br>• $\boldsymbol{E} \parallel [001]$ and $\boldsymbol{B} \parallel [010]$ and strain $\perp [001]$<br>• $\boldsymbol{E} \parallel [001]$ and $\boldsymbol{B} \perp [001]$
$Pc'a2_1'$ (29.101) $\mathbb{Z}_1$	• $\boldsymbol{E} \parallel [001]$ and $\boldsymbol{B} \parallel [010]$
P_C2 (3.6) $\mathbb{Z}_1$	• $\boldsymbol{E} \parallel [001]$ and strain $\perp [001]$
$P2_1'/c'$ (14.79) $\mathbb{Z}_2 \times \mathbb{Z}_4$	• $\boldsymbol{B} \parallel [100]$ and strain $\perp [001]$<br>• $\boldsymbol{B} \parallel [010]$ and strain $\perp [001]$<br>• $\boldsymbol{B} \perp [001]$
$Pc'a2_1'$ (29.101) $\mathbb{Z}_1$	• $\boldsymbol{E} \parallel [001]$ and $\boldsymbol{B} \parallel [100]$
P_Iba2 (32.143) $\mathbb{Z}_1$	• $\boldsymbol{E} \parallel [001]$
$Pbc'n'$ (60.423) $\mathbb{Z}_2$	• $\boldsymbol{B} \parallel [100]$
$Pbc'n'$ (60.423) $\mathbb{Z}_2$	• $\boldsymbol{B} \parallel [010]$
$P_S\bar{1}$ (2.7) $\mathbb{Z}_2 \times \mathbb{Z}_2 \times \mathbb{Z}_4$	• strain in generic direction
P_C2_1/c (14.84) $\mathbb{Z}_2$	• strain $\perp [100]$
P_C2_1/c (14.84) $\mathbb{Z}_2$	• strain $\perp [010]$
P_C2/m (10.49) $\mathbb{Z}_2 \times \mathbb{Z}_2$	• strain $\perp [001]$

Table. S 475: Subgroups of $Pccn$ (56.365) that can be realized by applying $\boldsymbol{E}/\boldsymbol{B}$ fields and/or uniaxial strains.

Subgroup & SI group	Perturbation
Pc (7.24) $\mathbb{Z}_1$	• $\boldsymbol{E} \parallel [100]$ and strain $\perp [010]$<br>• $\boldsymbol{E} \parallel [100]$ and $\boldsymbol{B} \parallel [010]$<br>• $\boldsymbol{E} \parallel [001]$ and strain $\perp [010]$<br>• $\boldsymbol{E} \parallel [001]$ and $\boldsymbol{B} \parallel [010]$<br>• $\boldsymbol{E} \perp [010]$
Pc (7.24) $\mathbb{Z}_1$	• $\boldsymbol{E} \parallel [100]$ and strain $\perp [001]$<br>• $\boldsymbol{E} \parallel [100]$ and $\boldsymbol{B} \parallel [001]$<br>• $\boldsymbol{E} \parallel [010]$ and strain $\perp [001]$<br>• $\boldsymbol{E} \parallel [010]$ and $\boldsymbol{B} \parallel [001]$<br>• $\boldsymbol{E} \perp [001]$

Continued on next page

. . . continued

Subgroup & SI group	Perturbation
$P2_1$ (4.7) $\mathbb{Z}_1$	• $\boldsymbol{E}$ ∥ [100] and strain ⊥ [100]<br>• $\boldsymbol{E}$ ∥ [100] and $\boldsymbol{B}$ ∥ [100]
$Pna2_1$ (33.144) $\mathbb{Z}_1$	• $\boldsymbol{E}$ ∥ [100]
$P2_1$ (4.7) $\mathbb{Z}_1$	• $\boldsymbol{E}$ ∥ [010] and strain ⊥ [010]<br>• $\boldsymbol{E}$ ∥ [010] and $\boldsymbol{B}$ ∥ [010]
$P\bar{1}$ (2.4) $\mathbb{Z}_2 \times \mathbb{Z}_2 \times \mathbb{Z}_2 \times \mathbb{Z}_4$	• strain in generic direction<br>• $\boldsymbol{B}$ ∥ [100] and strain ⊥ [010]<br>• $\boldsymbol{B}$ ∥ [100] and strain ⊥ [001]<br>• $\boldsymbol{B}$ ∥ [010] and strain ⊥ [100]<br>• $\boldsymbol{B}$ ∥ [010] and strain ⊥ [001]<br>• $\boldsymbol{B}$ ∥ [001] and strain ⊥ [100]<br>• $\boldsymbol{B}$ ∥ [001] and strain ⊥ [010]<br>• $\boldsymbol{B}$ in generic direction
$P2_1/c$ (14.75) $\mathbb{Z}_2$	• strain ⊥ [010]<br>• $\boldsymbol{B}$ ∥ [010]
Pc (7.24) $\mathbb{Z}_1$	• $\boldsymbol{E}$ ∥ [010] and strain ⊥ [100]<br>• $\boldsymbol{E}$ ∥ [010] and $\boldsymbol{B}$ ∥ [100]<br>• $\boldsymbol{E}$ ∥ [001] and strain ⊥ [100]<br>• $\boldsymbol{E}$ ∥ [001] and $\boldsymbol{B}$ ∥ [100]<br>• $\boldsymbol{E}$ ⊥ [100]
$Pna2_1$ (33.144) $\mathbb{Z}_1$	• $\boldsymbol{E}$ ∥ [010]
$P2_1/c$ (14.75) $\mathbb{Z}_2$	• strain ⊥ [100]<br>• $\boldsymbol{B}$ ∥ [100]
$P2$ (3.1) $\mathbb{Z}_2$	• $\boldsymbol{E}$ ∥ [001] and strain ⊥ [001]<br>• $\boldsymbol{E}$ ∥ [001] and $\boldsymbol{B}$ ∥ [001]
$P2/c$ (13.65) $\mathbb{Z}_2 \times \mathbb{Z}_2$	• strain ⊥ [001]<br>• $\boldsymbol{B}$ ∥ [001]
$Pcc2$ (27.78) $\mathbb{Z}_2$	• $\boldsymbol{E}$ ∥ [001]

Table. S 476: Subgroups of $Pccn1'$ (56.366) that can be realized by applying $\boldsymbol{E}/\boldsymbol{B}$ fields and/or uniaxial strains.

Subgroup & SI group	Perturbation

Continued on next page

...continued

Subgroup & SI group	Perturbation
$P11'$ (1.2) $\mathbb{Z}_1$	• $\boldsymbol{E}$ ∥ [100] and strain in generic direction<br>• $\boldsymbol{E}$ ∥ [010] and strain in generic direction<br>• $\boldsymbol{E}$ ∥ [001] and strain in generic direction<br>• $\boldsymbol{E} \perp$ [100] and strain $\perp$ [010]<br>• $\boldsymbol{E} \perp$ [100] and strain $\perp$ [001]<br>• $\boldsymbol{E} \perp$ [100] and strain in generic direction<br>• $\boldsymbol{E} \perp$ [010] and strain $\perp$ [100]<br>• $\boldsymbol{E} \perp$ [010] and strain $\perp$ [001]<br>• $\boldsymbol{E} \perp$ [010] and strain in generic direction<br>• $\boldsymbol{E} \perp$ [001] and strain $\perp$ [100]<br>• $\boldsymbol{E} \perp$ [001] and strain $\perp$ [010]<br>• $\boldsymbol{E} \perp$ [001] and strain in generic direction<br>• $\boldsymbol{E}$ in generic direction
Pc' (7.26) $\mathbb{Z}_1$	• $\boldsymbol{E}$ ∥ [100] and $\boldsymbol{B}$ ∥ [100] and strain $\perp$ [010]<br>• $\boldsymbol{E}$ ∥ [100] and $\boldsymbol{B}$ ∥ [001] and strain $\perp$ [010]<br>• $\boldsymbol{E}$ ∥ [100] and $\boldsymbol{B} \perp$ [010]<br>• $\boldsymbol{E}$ ∥ [001] and $\boldsymbol{B}$ ∥ [100] and strain $\perp$ [010]<br>• $\boldsymbol{E}$ ∥ [001] and $\boldsymbol{B}$ ∥ [001] and strain $\perp$ [010]<br>• $\boldsymbol{E}$ ∥ [001] and $\boldsymbol{B} \perp$ [010]<br>• $\boldsymbol{E} \perp$ [010] and $\boldsymbol{B}$ ∥ [100]<br>• $\boldsymbol{E} \perp$ [010] and $\boldsymbol{B}$ ∥ [001]<br>• $\boldsymbol{E} \perp$ [010] and $\boldsymbol{B} \perp$ [010]
Pc (7.24) $\mathbb{Z}_1$	• $\boldsymbol{E}$ ∥ [100] and $\boldsymbol{B}$ ∥ [010] and strain $\perp$ [010]<br>• $\boldsymbol{E}$ ∥ [001] and $\boldsymbol{B}$ ∥ [010] and strain $\perp$ [010]<br>• $\boldsymbol{E} \perp$ [010] and $\boldsymbol{B}$ ∥ [010]
$Pc1'$ (7.25) $\mathbb{Z}_1$	• $\boldsymbol{E}$ ∥ [100] and strain $\perp$ [010]<br>• $\boldsymbol{E}$ ∥ [001] and strain $\perp$ [010]<br>• $\boldsymbol{E} \perp$ [010]
Pc' (7.26) $\mathbb{Z}_1$	• $\boldsymbol{E}$ ∥ [100] and $\boldsymbol{B}$ ∥ [100] and strain $\perp$ [001]<br>• $\boldsymbol{E}$ ∥ [100] and $\boldsymbol{B}$ ∥ [010] and strain $\perp$ [001]<br>• $\boldsymbol{E}$ ∥ [100] and $\boldsymbol{B} \perp$ [001]<br>• $\boldsymbol{E}$ ∥ [010] and $\boldsymbol{B}$ ∥ [100] and strain $\perp$ [001]<br>• $\boldsymbol{E}$ ∥ [010] and $\boldsymbol{B}$ ∥ [010] and strain $\perp$ [001]<br>• $\boldsymbol{E}$ ∥ [010] and $\boldsymbol{B} \perp$ [001]<br>• $\boldsymbol{E} \perp$ [001] and $\boldsymbol{B}$ ∥ [100]<br>• $\boldsymbol{E} \perp$ [001] and $\boldsymbol{B}$ ∥ [010]<br>• $\boldsymbol{E} \perp$ [001] and $\boldsymbol{B} \perp$ [001]

Continued on next page

…continued

Subgroup & SI group	Perturbation
Pc (7.24) $\mathbb{Z}_1$	• $\boldsymbol{E} \parallel [100]$ and $\boldsymbol{B} \parallel [001]$ and strain $\perp [001]$<br>• $\boldsymbol{E} \parallel [010]$ and $\boldsymbol{B} \parallel [001]$ and strain $\perp [001]$<br>• $\boldsymbol{E} \perp [001]$ and $\boldsymbol{B} \parallel [001]$
$Pc1'$ (7.25) $\mathbb{Z}_1$	• $\boldsymbol{E} \parallel [100]$ and strain $\perp [001]$<br>• $\boldsymbol{E} \parallel [010]$ and strain $\perp [001]$<br>• $\boldsymbol{E} \perp [001]$
$P2_1'$ (4.9) $\mathbb{Z}_1$	• $\boldsymbol{E} \parallel [100]$ and $\boldsymbol{B} \parallel [010]$ and strain $\perp [100]$<br>• $\boldsymbol{E} \parallel [100]$ and $\boldsymbol{B} \parallel [001]$ and strain $\perp [100]$<br>• $\boldsymbol{E} \parallel [100]$ and $\boldsymbol{B} \perp [100]$
$Pn'a2_1'$ (33.146) $\mathbb{Z}_1$	• $\boldsymbol{E} \parallel [100]$ and $\boldsymbol{B} \parallel [010]$
$Pna'2_1'$ (33.147) $\mathbb{Z}_1$	• $\boldsymbol{E} \parallel [100]$ and $\boldsymbol{B} \parallel [001]$
$P2_1$ (4.7) $\mathbb{Z}_1$	• $\boldsymbol{E} \parallel [100]$ and $\boldsymbol{B} \parallel [100]$ and strain $\perp [100]$
$Pn'a'2_1$ (33.148) $\mathbb{Z}_1$	• $\boldsymbol{E} \parallel [100]$ and $\boldsymbol{B} \parallel [100]$
$P2_11'$ (4.8) $\mathbb{Z}_1$	• $\boldsymbol{E} \parallel [100]$ and strain $\perp [100]$
$Pna2_11'$ (33.145) $\mathbb{Z}_1$	• $\boldsymbol{E} \parallel [100]$
$P2_1'$ (4.9) $\mathbb{Z}_1$	• $\boldsymbol{E} \parallel [010]$ and $\boldsymbol{B} \parallel [100]$ and strain $\perp [010]$<br>• $\boldsymbol{E} \parallel [010]$ and $\boldsymbol{B} \parallel [001]$ and strain $\perp [010]$<br>• $\boldsymbol{E} \parallel [010]$ and $\boldsymbol{B} \perp [010]$
$P2_1$ (4.7) $\mathbb{Z}_1$	• $\boldsymbol{E} \parallel [010]$ and $\boldsymbol{B} \parallel [010]$ and strain $\perp [010]$
$P2_11'$ (4.8) $\mathbb{Z}_1$	• $\boldsymbol{E} \parallel [010]$ and strain $\perp [010]$
$P\bar{1}$ (2.4) $\mathbb{Z}_2 \times \mathbb{Z}_2 \times \mathbb{Z}_2 \times \mathbb{Z}_4$	• $\boldsymbol{B} \parallel [100]$ and strain in generic direction<br>• $\boldsymbol{B} \parallel [010]$ and strain in generic direction<br>• $\boldsymbol{B} \parallel [001]$ and strain in generic direction<br>• $\boldsymbol{B} \perp [100]$ and strain $\perp [010]$<br>• $\boldsymbol{B} \perp [100]$ and strain $\perp [001]$<br>• $\boldsymbol{B} \perp [100]$ and strain in generic direction<br>• $\boldsymbol{B} \perp [010]$ and strain $\perp [100]$<br>• $\boldsymbol{B} \perp [010]$ and strain $\perp [001]$<br>• $\boldsymbol{B} \perp [010]$ and strain in generic direction<br>• $\boldsymbol{B} \perp [001]$ and strain $\perp [100]$<br>• $\boldsymbol{B} \perp [001]$ and strain $\perp [010]$<br>• $\boldsymbol{B} \perp [001]$ and strain in generic direction<br>• $\boldsymbol{B}$ in generic direction

Continued on next page

...continued

Subgroup & SI group	Perturbation
$P2_1'/c'$ (14.79) $\mathbb{Z}_2 \times \mathbb{Z}_4$	• $\boldsymbol{B} \parallel [100]$ and strain $\perp [010]$<br>• $\boldsymbol{B} \parallel [001]$ and strain $\perp [010]$<br>• $\boldsymbol{B} \perp [010]$
$P2_1/c$ (14.75) $\mathbb{Z}_2$	• $\boldsymbol{B} \parallel [010]$ and strain $\perp [010]$
$P\bar{1}1'$ (2.5) $\mathbb{Z}_2 \times \mathbb{Z}_2 \times \mathbb{Z}_2 \times \mathbb{Z}_4$	• strain in generic direction
$P2_1/c1'$ (14.76) $\mathbb{Z}_2$	• strain $\perp [010]$
Pc' (7.26) $\mathbb{Z}_1$	• $\boldsymbol{E} \parallel [010]$ and $\boldsymbol{B} \parallel [010]$ and strain $\perp [100]$<br>• $\boldsymbol{E} \parallel [010]$ and $\boldsymbol{B} \parallel [001]$ and strain $\perp [100]$<br>• $\boldsymbol{E} \parallel [010]$ and $\boldsymbol{B} \perp [100]$<br>• $\boldsymbol{E} \parallel [001]$ and $\boldsymbol{B} \parallel [010]$ and strain $\perp [100]$<br>• $\boldsymbol{E} \parallel [001]$ and $\boldsymbol{B} \parallel [001]$ and strain $\perp [100]$<br>• $\boldsymbol{E} \parallel [001]$ and $\boldsymbol{B} \perp [100]$<br>• $\boldsymbol{E} \perp [100]$ and $\boldsymbol{B} \parallel [010]$<br>• $\boldsymbol{E} \perp [100]$ and $\boldsymbol{B} \parallel [001]$<br>• $\boldsymbol{E} \perp [100]$ and $\boldsymbol{B} \perp [100]$
$Pna'2_1'$ (33.147) $\mathbb{Z}_1$	• $\boldsymbol{E} \parallel [010]$ and $\boldsymbol{B} \parallel [001]$
$Pn'a'2_1$ (33.148) $\mathbb{Z}_1$	• $\boldsymbol{E} \parallel [010]$ and $\boldsymbol{B} \parallel [010]$
$P2_1'/c'$ (14.79) $\mathbb{Z}_2 \times \mathbb{Z}_4$	• $\boldsymbol{B} \parallel [010]$ and strain $\perp [100]$<br>• $\boldsymbol{B} \parallel [001]$ and strain $\perp [100]$<br>• $\boldsymbol{B} \perp [100]$
Pc (7.24) $\mathbb{Z}_1$	• $\boldsymbol{E} \parallel [010]$ and $\boldsymbol{B} \parallel [100]$ and strain $\perp [100]$<br>• $\boldsymbol{E} \parallel [001]$ and $\boldsymbol{B} \parallel [100]$ and strain $\perp [100]$<br>• $\boldsymbol{E} \perp [100]$ and $\boldsymbol{B} \parallel [100]$
$Pn'a2_1'$ (33.146) $\mathbb{Z}_1$	• $\boldsymbol{E} \parallel [010]$ and $\boldsymbol{B} \parallel [100]$
$P2_1/c$ (14.75) $\mathbb{Z}_2$	• $\boldsymbol{B} \parallel [100]$ and strain $\perp [100]$
$Pc1'$ (7.25) $\mathbb{Z}_1$	• $\boldsymbol{E} \parallel [010]$ and strain $\perp [100]$<br>• $\boldsymbol{E} \parallel [001]$ and strain $\perp [100]$<br>• $\boldsymbol{E} \perp [100]$
$Pna2_11'$ (33.145) $\mathbb{Z}_1$	• $\boldsymbol{E} \parallel [010]$
$P2_1/c1'$ (14.76) $\mathbb{Z}_2$	• strain $\perp [100]$
$P2'$ (3.3) $\mathbb{Z}_1$	• $\boldsymbol{E} \parallel [001]$ and $\boldsymbol{B} \parallel [100]$ and strain $\perp [001]$<br>• $\boldsymbol{E} \parallel [001]$ and $\boldsymbol{B} \parallel [010]$ and strain $\perp [001]$<br>• $\boldsymbol{E} \parallel [001]$ and $\boldsymbol{B} \perp [001]$

Continued on next page

...continued

Subgroup & SI group	Perturbation
$P2'/c'$ (13.69) $\mathbb{Z}_2 \times \mathbb{Z}_2 \times \mathbb{Z}_4$	• $\boldsymbol{B} \parallel [100]$ and strain $\perp [001]$<br>• $\boldsymbol{B} \parallel [010]$ and strain $\perp [001]$<br>• $\boldsymbol{B} \perp [001]$
$Pc'c2'$ (27.80) $\mathbb{Z}_1$	• $\boldsymbol{E} \parallel [001]$ and $\boldsymbol{B} \parallel [010]$
$Pc'cn'$ (56.370) $\mathbb{Z}_2$	• $\boldsymbol{B} \parallel [010]$
$Pc'c2'$ (27.80) $\mathbb{Z}_1$	• $\boldsymbol{E} \parallel [001]$ and $\boldsymbol{B} \parallel [100]$
$Pc'cn'$ (56.370) $\mathbb{Z}_2$	• $\boldsymbol{B} \parallel [100]$
$P2$ (3.1) $\mathbb{Z}_2$	• $\boldsymbol{E} \parallel [001]$ and $\boldsymbol{B} \parallel [001]$ and strain $\perp [001]$
$P2/c$ (13.65) $\mathbb{Z}_2 \times \mathbb{Z}_2$	• $\boldsymbol{B} \parallel [001]$ and strain $\perp [001]$
$Pc'c'2$ (27.81) $\mathbb{Z}_2$	• $\boldsymbol{E} \parallel [001]$ and $\boldsymbol{B} \parallel [001]$
$Pc'c'n$ (56.369) $\mathbb{Z}_2 \times \mathbb{Z}_2$	• $\boldsymbol{B} \parallel [001]$
$P21'$ (3.2) $\mathbb{Z}_2$	• $\boldsymbol{E} \parallel [001]$ and strain $\perp [001]$
$P2/c1'$ (13.66) $\mathbb{Z}_2 \times \mathbb{Z}_2$	• strain $\perp [001]$
$Pcc21'$ (27.79) $\mathbb{Z}_2$	• $\boldsymbol{E} \parallel [001]$

Table. S 477: Subgroups of $Pc'cn$ (56.367) that can be realized by applying $\boldsymbol{E}/\boldsymbol{B}$ fields and/or uniaxial strains.

Subgroup & SI group	Perturbation
Pc (7.24) $\mathbb{Z}_1$	• $\boldsymbol{B} \parallel [010]$ and strain $\perp [010]$<br>• $\boldsymbol{E} \parallel [100]$ and strain $\perp [010]$<br>• $\boldsymbol{E} \parallel [100]$ and $\boldsymbol{B} \parallel [010]$<br>• $\boldsymbol{E} \parallel [001]$ and strain $\perp [010]$<br>• $\boldsymbol{E} \perp [010]$
Pc (7.24) $\mathbb{Z}_1$	• $\boldsymbol{B} \parallel [001]$ and strain $\perp [001]$<br>• $\boldsymbol{E} \parallel [100]$ and strain $\perp [001]$<br>• $\boldsymbol{E} \parallel [100]$ and $\boldsymbol{B} \parallel [001]$<br>• $\boldsymbol{E} \parallel [010]$ and strain $\perp [001]$<br>• $\boldsymbol{E} \perp [001]$
$P2_1$ (4.7) $\mathbb{Z}_1$	• $\boldsymbol{B} \parallel [100]$ and strain $\perp [100]$<br>• $\boldsymbol{E} \parallel [100]$ and strain $\perp [100]$<br>• $\boldsymbol{E} \parallel [100]$ and $\boldsymbol{B} \parallel [100]$

Continued on next page

. . . continued

Subgroup & SI group	Perturbation
$Pna2_1$ (33.144) $\mathbb{Z}_1$	• $\boldsymbol{E} \parallel [100]$
$P2_1'$ (4.9) $\mathbb{Z}_1$	• $\boldsymbol{B} \parallel [100]$ and strain $\perp [010]$<br>• $\boldsymbol{B} \parallel [001]$ and strain $\perp [010]$<br>• $\boldsymbol{B} \perp [010]$<br>• $\boldsymbol{E} \parallel [010]$ and strain $\perp [010]$<br>• $\boldsymbol{E} \parallel [010]$ and $\boldsymbol{B} \parallel [100]$
$P\bar{1}'$ (2.6) $\mathbb{Z}_1$	• strain in generic direction
$P2_1'/c$ (14.77) $\mathbb{Z}_1$	• strain $\perp [010]$
Pc' (7.26) $\mathbb{Z}_1$	• $\boldsymbol{B} \parallel [010]$ and strain $\perp [100]$<br>• $\boldsymbol{B} \parallel [001]$ and strain $\perp [100]$<br>• $\boldsymbol{B} \perp [100]$<br>• $\boldsymbol{E} \parallel [010]$ and strain $\perp [100]$<br>• $\boldsymbol{E} \parallel [010]$ and $\boldsymbol{B} \parallel [010]$<br>• $\boldsymbol{E} \parallel [001]$ and strain $\perp [100]$<br>• $\boldsymbol{E} \parallel [001]$ and $\boldsymbol{B} \parallel [001]$<br>• $\boldsymbol{E} \perp [100]$
$Pna'2_1'$ (33.147) $\mathbb{Z}_1$	• $\boldsymbol{B} \parallel [001]$<br>• $\boldsymbol{E} \parallel [010]$
$P2_1/c'$ (14.78) $\mathbb{Z}_1$	• strain $\perp [100]$
$P2'$ (3.3) $\mathbb{Z}_1$	• $\boldsymbol{B} \parallel [100]$ and strain $\perp [001]$<br>• $\boldsymbol{B} \parallel [010]$ and strain $\perp [001]$<br>• $\boldsymbol{B} \perp [001]$<br>• $\boldsymbol{E} \parallel [001]$ and strain $\perp [001]$<br>• $\boldsymbol{E} \parallel [001]$ and $\boldsymbol{B} \parallel [100]$
$P2_12_1'2'$ (18.19) $\mathbb{Z}_1$	• $\boldsymbol{B} \parallel [100]$
$P2'/c$ (13.67) $\mathbb{Z}_1$	• strain $\perp [001]$
$Pc'c2'$ (27.80) $\mathbb{Z}_1$	• $\boldsymbol{B} \parallel [010]$<br>• $\boldsymbol{E} \parallel [001]$

Table. S 478: Subgroups of $Pccn'$ (56.368) that can be realized by applying $\boldsymbol{E}/\boldsymbol{B}$ fields and/or uniaxial strains.

Subgroup & SI group	Perturbation

Continued on next page

…continued

Subgroup & SI group	Perturbation
Pc (7.24) $\mathbb{Z}_1$	• $\boldsymbol{B} \parallel [010]$ and strain $\perp [010]$<br>• $\boldsymbol{E} \parallel [100]$ and strain $\perp [010]$<br>• $\boldsymbol{E} \parallel [001]$ and strain $\perp [010]$<br>• $\boldsymbol{E} \parallel [001]$ and $\boldsymbol{B} \parallel [010]$<br>• $\boldsymbol{E} \perp [010]$
Pc' (7.26) $\mathbb{Z}_1$	• $\boldsymbol{B} \parallel [100]$ and strain $\perp [001]$<br>• $\boldsymbol{B} \parallel [010]$ and strain $\perp [001]$<br>• $\boldsymbol{B} \perp [001]$<br>• $\boldsymbol{E} \parallel [100]$ and strain $\perp [001]$<br>• $\boldsymbol{E} \parallel [100]$ and $\boldsymbol{B} \parallel [100]$<br>• $\boldsymbol{E} \parallel [010]$ and strain $\perp [001]$<br>• $\boldsymbol{E} \parallel [010]$ and $\boldsymbol{B} \parallel [010]$<br>• $\boldsymbol{E} \perp [001]$
$P2_1'$ (4.9) $\mathbb{Z}_1$	• $\boldsymbol{B} \parallel [010]$ and strain $\perp [100]$<br>• $\boldsymbol{B} \parallel [001]$ and strain $\perp [100]$<br>• $\boldsymbol{B} \perp [100]$<br>• $\boldsymbol{E} \parallel [100]$ and strain $\perp [100]$<br>• $\boldsymbol{E} \parallel [100]$ and $\boldsymbol{B} \parallel [001]$
$Pn'a2_1'$ (33.146) $\mathbb{Z}_1$	• $\boldsymbol{B} \parallel [010]$<br>• $\boldsymbol{E} \parallel [100]$
$P2_1'$ (4.9) $\mathbb{Z}_1$	• $\boldsymbol{B} \parallel [100]$ and strain $\perp [010]$<br>• $\boldsymbol{B} \parallel [001]$ and strain $\perp [010]$<br>• $\boldsymbol{B} \perp [010]$<br>• $\boldsymbol{E} \parallel [010]$ and strain $\perp [010]$<br>• $\boldsymbol{E} \parallel [010]$ and $\boldsymbol{B} \parallel [001]$
$P\bar{1}'$ (2.6) $\mathbb{Z}_1$	• strain in generic direction
$P2_1'/c$ (14.77) $\mathbb{Z}_1$	• strain $\perp [010]$
Pc (7.24) $\mathbb{Z}_1$	• $\boldsymbol{B} \parallel [100]$ and strain $\perp [100]$<br>• $\boldsymbol{E} \parallel [010]$ and strain $\perp [100]$<br>• $\boldsymbol{E} \parallel [001]$ and strain $\perp [100]$<br>• $\boldsymbol{E} \parallel [001]$ and $\boldsymbol{B} \parallel [100]$<br>• $\boldsymbol{E} \perp [100]$
$Pn'a2_1'$ (33.146) $\mathbb{Z}_1$	• $\boldsymbol{B} \parallel [100]$<br>• $\boldsymbol{E} \parallel [010]$
$P2_1'/c$ (14.77) $\mathbb{Z}_1$	• strain $\perp [100]$

Continued on next page

. . . continued

Subgroup & SI group	Perturbation
$P2$ (3.1) $\mathbb{Z}_2$	• $\boldsymbol{B}$ ∥ [001] and strain ⊥ [001]<br>• $\boldsymbol{E}$ ∥ [001] and strain ⊥ [001]<br>• $\boldsymbol{E}$ ∥ [001] and $\boldsymbol{B}$ ∥ [001]
$P2_1'2_1'2$ (18.18) $\mathbb{Z}_2$	• $\boldsymbol{B}$ ∥ [001]
$P2/c'$ (13.68) $\mathbb{Z}_2$	• strain ⊥ [001]
$Pcc2$ (27.78) $\mathbb{Z}_2$	• $\boldsymbol{E}$ ∥ [001]

Table. S 479: Subgroups of $Pc'c'n$ (56.369) that can be realized by applying $\boldsymbol{E}/\boldsymbol{B}$ fields and/or uniaxial strains.

Subgroup & SI group	Perturbation
Pc' (7.26) $\mathbb{Z}_1$	• $\boldsymbol{E}$ ∥ [100] and strain ⊥ [010]<br>• $\boldsymbol{E}$ ∥ [100] and ($\boldsymbol{B}$ ∥ [100] or $\boldsymbol{B}$ ⊥ [010])<br>• $\boldsymbol{E}$ ∥ [001] and strain ⊥ [010]<br>• $\boldsymbol{E}$ ∥ [001] and ($\boldsymbol{B}$ ∥ [100] or $\boldsymbol{B}$ ⊥ [010])<br>• $\boldsymbol{E}$ ⊥ [010]
Pc (7.24) $\mathbb{Z}_1$	• $\boldsymbol{E}$ ∥ [100] and strain ⊥ [001]<br>• $\boldsymbol{E}$ ∥ [010] and strain ⊥ [001]<br>• $\boldsymbol{E}$ ⊥ [001]
$P2_1'$ (4.9) $\mathbb{Z}_1$	• $\boldsymbol{E}$ ∥ [100] and strain ⊥ [100]<br>• $\boldsymbol{E}$ ∥ [100] and ($\boldsymbol{B}$ ∥ [010] or $\boldsymbol{B}$ ⊥ [100])
$Pna'2_1'$ (33.147) $\mathbb{Z}_1$	• $\boldsymbol{E}$ ∥ [100]
$P2_1'$ (4.9) $\mathbb{Z}_1$	• $\boldsymbol{E}$ ∥ [010] and strain ⊥ [010]<br>• $\boldsymbol{E}$ ∥ [010] and ($\boldsymbol{B}$ ∥ [100] or $\boldsymbol{B}$ ⊥ [010])
$P\bar{1}$ (2.4) $\mathbb{Z}_2\times\mathbb{Z}_2\times\mathbb{Z}_2\times\mathbb{Z}_4$	• strain in generic direction<br>• ($\boldsymbol{B}$ ∥ [100] or $\boldsymbol{B}$ ⊥ [010]) and strain ⊥ [100]<br>• ($\boldsymbol{B}$ ∥ [100] or $\boldsymbol{B}$ ⊥ [010]) and strain ⊥ [001]<br>• ($\boldsymbol{B}$ ∥ [010] or $\boldsymbol{B}$ ⊥ [100]) and strain ⊥ [010]<br>• ($\boldsymbol{B}$ ∥ [010] or $\boldsymbol{B}$ ⊥ [100]) and strain ⊥ [001]<br>• $\boldsymbol{B}$ in generic direction
$P2_1'/c'$ (14.79) $\mathbb{Z}_2\times\mathbb{Z}_4$	• strain ⊥ [010]<br>• ($\boldsymbol{B}$ ∥ [100] or $\boldsymbol{B}$ ⊥ [010])

Continued on next page

. . . continued

Subgroup & SI group	Perturbation
Pc' (7.26) $\mathbb{Z}_1$	• $\boldsymbol{E}$ ∥ [010] and strain ⊥ [100]<br>• $\boldsymbol{E}$ ∥ [010] and ($\boldsymbol{B}$ ∥ [010] or $\boldsymbol{B}$ ⊥ [100])<br>• $\boldsymbol{E}$ ∥ [001] and strain ⊥ [100]<br>• $\boldsymbol{E}$ ∥ [001] and ($\boldsymbol{B}$ ∥ [010] or $\boldsymbol{B}$ ⊥ [100])<br>• $\boldsymbol{E}$ ⊥ [100]
$Pna'2_1'$ (33.147) $\mathbb{Z}_1$	• $\boldsymbol{E}$ ∥ [010]
$P2_1'/c'$ (14.79) $\mathbb{Z}_2 \times \mathbb{Z}_4$	• strain ⊥ [100]<br>• ($\boldsymbol{B}$ ∥ [010] or $\boldsymbol{B}$ ⊥ [100])
$P2$ (3.1) $\mathbb{Z}_2$	• $\boldsymbol{E}$ ∥ [001] and strain ⊥ [001]
$P2/c$ (13.65) $\mathbb{Z}_2 \times \mathbb{Z}_2$	• strain ⊥ [001]
$Pc'c'2$ (27.81) $\mathbb{Z}_2$	• $\boldsymbol{E}$ ∥ [001]

Table. S 480: Subgroups of $Pc'cn'$ (56.370) that can be realized by applying $\boldsymbol{E}/\boldsymbol{B}$ fields and/or uniaxial strains.

Subgroup & SI group	Perturbation
Pc (7.24) $\mathbb{Z}_1$	• $\boldsymbol{E}$ ∥ [100] and strain ⊥ [010]<br>• $\boldsymbol{E}$ ∥ [001] and strain ⊥ [010]<br>• $\boldsymbol{E}$ ⊥ [010]
Pc' (7.26) $\mathbb{Z}_1$	• $\boldsymbol{E}$ ∥ [100] and strain ⊥ [001]<br>• $\boldsymbol{E}$ ∥ [100] and ($\boldsymbol{B}$ ∥ [100] or $\boldsymbol{B}$ ⊥ [001])<br>• $\boldsymbol{E}$ ∥ [010] and strain ⊥ [001]<br>• $\boldsymbol{E}$ ∥ [010] and ($\boldsymbol{B}$ ∥ [100] or $\boldsymbol{B}$ ⊥ [001])<br>• $\boldsymbol{E}$ ⊥ [001]
$P2_1'$ (4.9) $\mathbb{Z}_1$	• $\boldsymbol{E}$ ∥ [100] and strain ⊥ [100]<br>• $\boldsymbol{E}$ ∥ [100] and ($\boldsymbol{B}$ ∥ [001] or $\boldsymbol{B}$ ⊥ [100])
$Pn'a2_1'$ (33.146) $\mathbb{Z}_1$	• $\boldsymbol{E}$ ∥ [100]
$P2_1$ (4.7) $\mathbb{Z}_1$	• $\boldsymbol{E}$ ∥ [010] and strain ⊥ [010]

Continued on next page

…continued

Subgroup & SI group	Perturbation
$P\bar{1}$ (2.4) $\mathbb{Z}_2\times\mathbb{Z}_2\times\mathbb{Z}_2\times\mathbb{Z}_4$	• strain in generic direction<br>• ($\boldsymbol{B}\parallel[100]$ or $\boldsymbol{B}\perp[001]$) and strain $\perp[100]$<br>• ($\boldsymbol{B}\parallel[100]$ or $\boldsymbol{B}\perp[001]$) and strain $\perp[010]$<br>• ($\boldsymbol{B}\parallel[001]$ or $\boldsymbol{B}\perp[100]$) and strain $\perp[010]$<br>• ($\boldsymbol{B}\parallel[001]$ or $\boldsymbol{B}\perp[100]$) and strain $\perp[001]$<br>• $\boldsymbol{B}$ in generic direction
$P2_1/c$ (14.75) $\mathbb{Z}_2$	• strain $\perp[010]$
Pc' (7.26) $\mathbb{Z}_1$	• $\boldsymbol{E}\parallel[010]$ and strain $\perp[100]$<br>• $\boldsymbol{E}\parallel[010]$ and ($\boldsymbol{B}\parallel[001]$ or $\boldsymbol{B}\perp[100]$)<br>• $\boldsymbol{E}\parallel[001]$ and strain $\perp[100]$<br>• $\boldsymbol{E}\parallel[001]$ and ($\boldsymbol{B}\parallel[001]$ or $\boldsymbol{B}\perp[100]$)<br>• $\boldsymbol{E}\perp[100]$
$Pn'a'2_1$ (33.148) $\mathbb{Z}_1$	• $\boldsymbol{E}\parallel[010]$
$P2_1'/c'$ (14.79) $\mathbb{Z}_2\times\mathbb{Z}_4$	• strain $\perp[100]$<br>• ($\boldsymbol{B}\parallel[001]$ or $\boldsymbol{B}\perp[100]$)
$P2'$ (3.3) $\mathbb{Z}_1$	• $\boldsymbol{E}\parallel[001]$ and strain $\perp[001]$<br>• $\boldsymbol{E}\parallel[001]$ and ($\boldsymbol{B}\parallel[100]$ or $\boldsymbol{B}\perp[001]$)
$P2'/c'$ (13.69) $\mathbb{Z}_2\times\mathbb{Z}_2\times\mathbb{Z}_4$	• strain $\perp[001]$<br>• ($\boldsymbol{B}\parallel[100]$ or $\boldsymbol{B}\perp[001]$)
$Pc'c2'$ (27.80) $\mathbb{Z}_1$	• $\boldsymbol{E}\parallel[001]$

Table. S 481: Subgroups of $Pc'c'n'$ (56.371) that can be realized by applying $\boldsymbol{E}/\boldsymbol{B}$ fields and/or uniaxial strains.

Subgroup & SI group	Perturbation
Pc' (7.26) $\mathbb{Z}_1$	• $\boldsymbol{B}\parallel[100]$ and strain $\perp[010]$<br>• $\boldsymbol{B}\parallel[001]$ and strain $\perp[010]$<br>• $\boldsymbol{B}\perp[010]$<br>• $\boldsymbol{E}\parallel[100]$ and strain $\perp[010]$<br>• $\boldsymbol{E}\parallel[100]$ and $\boldsymbol{B}\parallel[001]$<br>• $\boldsymbol{E}\parallel[001]$ and strain $\perp[010]$<br>• $\boldsymbol{E}\parallel[001]$ and $\boldsymbol{B}\parallel[100]$<br>• $\boldsymbol{E}\perp[010]$

Continued on next page

…continued

Subgroup & SI group	Perturbation
Pc' (7.26) $\mathbb{Z}_1$	• $\boldsymbol{B} \parallel [100]$ and strain $\perp [001]$<br>• $\boldsymbol{B} \parallel [010]$ and strain $\perp [001]$<br>• $\boldsymbol{B} \perp [001]$<br>• $\boldsymbol{E} \parallel [100]$ and strain $\perp [001]$<br>• $\boldsymbol{E} \parallel [100]$ and $\boldsymbol{B} \parallel [010]$<br>• $\boldsymbol{E} \parallel [010]$ and strain $\perp [001]$<br>• $\boldsymbol{E} \parallel [010]$ and $\boldsymbol{B} \parallel [100]$<br>• $\boldsymbol{E} \perp [001]$
$P2_1$ (4.7) $\mathbb{Z}_1$	• $\boldsymbol{B} \parallel [100]$ and strain $\perp [100]$<br>• $\boldsymbol{E} \parallel [100]$ and strain $\perp [100]$
$Pn'a'2_1$ (33.148) $\mathbb{Z}_1$	• $\boldsymbol{B} \parallel [100]$<br>• $\boldsymbol{E} \parallel [100]$
$P2_1$ (4.7) $\mathbb{Z}_1$	• $\boldsymbol{B} \parallel [010]$ and strain $\perp [010]$<br>• $\boldsymbol{E} \parallel [010]$ and strain $\perp [010]$
$P\bar{1}'$ (2.6) $\mathbb{Z}_1$	• strain in generic direction
$P2_1/c'$ (14.78) $\mathbb{Z}_1$	• strain $\perp [010]$
Pc' (7.26) $\mathbb{Z}_1$	• $\boldsymbol{B} \parallel [010]$ and strain $\perp [100]$<br>• $\boldsymbol{B} \parallel [001]$ and strain $\perp [100]$<br>• $\boldsymbol{B} \perp [100]$<br>• $\boldsymbol{E} \parallel [010]$ and strain $\perp [100]$<br>• $\boldsymbol{E} \parallel [010]$ and $\boldsymbol{B} \parallel [001]$<br>• $\boldsymbol{E} \parallel [001]$ and strain $\perp [100]$<br>• $\boldsymbol{E} \parallel [001]$ and $\boldsymbol{B} \parallel [010]$<br>• $\boldsymbol{E} \perp [100]$
$Pn'a'2_1$ (33.148) $\mathbb{Z}_1$	• $\boldsymbol{B} \parallel [010]$<br>• $\boldsymbol{E} \parallel [010]$
$P2_1/c'$ (14.78) $\mathbb{Z}_1$	• strain $\perp [100]$
$P2$ (3.1) $\mathbb{Z}_2$	• $\boldsymbol{B} \parallel [001]$ and strain $\perp [001]$<br>• $\boldsymbol{E} \parallel [001]$ and strain $\perp [001]$
$P2/c'$ (13.68) $\mathbb{Z}_2$	• strain $\perp [001]$
$Pc'c'2$ (27.81) $\mathbb{Z}_2$	• $\boldsymbol{B} \parallel [001]$<br>• $\boldsymbol{E} \parallel [001]$

Table. S 482: Subgroups of P_bccn (56.372) that can be realized by applying $\boldsymbol{E}/\boldsymbol{B}$ fields and/or uniaxial strains.

Subgroup & SI group	Perturbation
P_S1 (1.3) $\mathbb{Z}_1$	• $\boldsymbol{E}$ ∥ [100] and strain in generic direction<br>• $\boldsymbol{E}$ ∥ [010] and strain in generic direction<br>• $\boldsymbol{E}$ ∥ [001] and strain in generic direction<br>• $\boldsymbol{E}$ ⊥ [100] and strain ⊥ [010]<br>• $\boldsymbol{E}$ ⊥ [100] and strain ⊥ [001]<br>• $\boldsymbol{E}$ ⊥ [100] and strain in generic direction<br>• $\boldsymbol{E}$ ⊥ [010] and strain ⊥ [100]<br>• $\boldsymbol{E}$ ⊥ [010] and strain ⊥ [001]<br>• $\boldsymbol{E}$ ⊥ [010] and strain in generic direction<br>• $\boldsymbol{E}$ ⊥ [001] and strain ⊥ [100]<br>• $\boldsymbol{E}$ ⊥ [001] and strain ⊥ [010]<br>• $\boldsymbol{E}$ ⊥ [001] and strain in generic direction<br>• $\boldsymbol{E}$ in generic direction
Pc' (7.26) $\mathbb{Z}_1$	• $\boldsymbol{E}$ ∥ [100] and $\boldsymbol{B}$ ∥ [100] and strain ⊥ [010]<br>• $\boldsymbol{E}$ ∥ [100] and $\boldsymbol{B}$ ∥ [001] and strain ⊥ [010]<br>• $\boldsymbol{E}$ ∥ [100] and $\boldsymbol{B}$ ⊥ [010]<br>• $\boldsymbol{E}$ ∥ [001] and $\boldsymbol{B}$ ∥ [100] and strain ⊥ [010]<br>• $\boldsymbol{E}$ ∥ [001] and $\boldsymbol{B}$ ∥ [001] and strain ⊥ [010]<br>• $\boldsymbol{E}$ ∥ [001] and $\boldsymbol{B}$ ⊥ [010]<br>• $\boldsymbol{E}$ ⊥ [010] and $\boldsymbol{B}$ ∥ [100]<br>• $\boldsymbol{E}$ ⊥ [010] and $\boldsymbol{B}$ ∥ [001]<br>• $\boldsymbol{E}$ ⊥ [010] and $\boldsymbol{B}$ ⊥ [010]
Pc (7.24) $\mathbb{Z}_1$	• $\boldsymbol{E}$ ∥ [100] and $\boldsymbol{B}$ ∥ [010] and strain ⊥ [010]<br>• $\boldsymbol{E}$ ∥ [001] and $\boldsymbol{B}$ ∥ [010] and strain ⊥ [010]<br>• $\boldsymbol{E}$ ⊥ [010] and $\boldsymbol{B}$ ∥ [010]
P_bc (7.29) $\mathbb{Z}_1$	• $\boldsymbol{E}$ ∥ [100] and strain ⊥ [010]<br>• $\boldsymbol{E}$ ∥ [001] and strain ⊥ [010]<br>• $\boldsymbol{E}$ ⊥ [010]
Pc' (7.26) $\mathbb{Z}_1$	• $\boldsymbol{E}$ ∥ [100] and $\boldsymbol{B}$ ∥ [100] and strain ⊥ [001]<br>• $\boldsymbol{E}$ ∥ [100] and $\boldsymbol{B}$ ∥ [010] and strain ⊥ [001]<br>• $\boldsymbol{E}$ ∥ [100] and $\boldsymbol{B}$ ⊥ [001]<br>• $\boldsymbol{E}$ ∥ [010] and $\boldsymbol{B}$ ∥ [100] and strain ⊥ [001]<br>• $\boldsymbol{E}$ ∥ [010] and $\boldsymbol{B}$ ∥ [010] and strain ⊥ [001]<br>• $\boldsymbol{E}$ ∥ [010] and $\boldsymbol{B}$ ⊥ [001]<br>• $\boldsymbol{E}$ ⊥ [001] and $\boldsymbol{B}$ ∥ [100]<br>• $\boldsymbol{E}$ ⊥ [001] and $\boldsymbol{B}$ ∥ [010]<br>• $\boldsymbol{E}$ ⊥ [001] and $\boldsymbol{B}$ ⊥ [001]

Continued on next page

...continued

Subgroup & SI group	Perturbation
Pc (7.24) $\mathbb{Z}_1$	• $\boldsymbol{E}$ ∥ [100] and $\boldsymbol{B}$ ∥ [001] and strain ⊥ [001]<br>• $\boldsymbol{E}$ ∥ [010] and $\boldsymbol{B}$ ∥ [001] and strain ⊥ [001]<br>• $\boldsymbol{E}$ ⊥ [001] and $\boldsymbol{B}$ ∥ [001]
P_ac (7.27) $\mathbb{Z}_1$	• $\boldsymbol{E}$ ∥ [100] and strain ⊥ [001]<br>• $\boldsymbol{E}$ ∥ [010] and strain ⊥ [001]<br>• $\boldsymbol{E}$ ⊥ [001]
$P2_1$ (4.7) $\mathbb{Z}_1$	• $\boldsymbol{E}$ ∥ [100] and $\boldsymbol{B}$ ∥ [100] and strain ⊥ [100]
$Pc'a'2_1$ (29.103) $\mathbb{Z}_1$	• $\boldsymbol{E}$ ∥ [100] and $\boldsymbol{B}$ ∥ [100]
$P2_1'$ (4.9) $\mathbb{Z}_1$	• $\boldsymbol{E}$ ∥ [100] and $\boldsymbol{B}$ ∥ [010] and strain ⊥ [100]<br>• $\boldsymbol{E}$ ∥ [100] and $\boldsymbol{B}$ ∥ [001] and strain ⊥ [100]<br>• $\boldsymbol{E}$ ∥ [100] and $\boldsymbol{B}$ ⊥ [100]
$Pc'a2_1'$ (29.101) $\mathbb{Z}_1$	• $\boldsymbol{E}$ ∥ [100] and $\boldsymbol{B}$ ∥ [010]
$Pna'2_1'$ (33.147) $\mathbb{Z}_1$	• $\boldsymbol{E}$ ∥ [100] and $\boldsymbol{B}$ ∥ [001]
P_a2_1 (4.10) $\mathbb{Z}_1$	• $\boldsymbol{E}$ ∥ [100] and strain ⊥ [100]
P_bna2_1 (33.150) $\mathbb{Z}_1$	• $\boldsymbol{E}$ ∥ [100]
$P2'$ (3.3) $\mathbb{Z}_1$	• $\boldsymbol{E}$ ∥ [010] and $\boldsymbol{B}$ ∥ [100] and strain ⊥ [010]<br>• $\boldsymbol{E}$ ∥ [010] and $\boldsymbol{B}$ ∥ [001] and strain ⊥ [010]<br>• $\boldsymbol{E}$ ∥ [010] and $\boldsymbol{B}$ ⊥ [010]
$P2_1$ (4.7) $\mathbb{Z}_1$	• $\boldsymbol{E}$ ∥ [010] and $\boldsymbol{B}$ ∥ [010] and strain ⊥ [010]
P_b2_1 (4.11) $\mathbb{Z}_1$	• $\boldsymbol{E}$ ∥ [010] and strain ⊥ [010]
$P\bar{1}$ (2.4) $\mathbb{Z}_2 \times \mathbb{Z}_2 \times \mathbb{Z}_2 \times \mathbb{Z}_4$	• $\boldsymbol{B}$ ∥ [100] and strain in generic direction<br>• $\boldsymbol{B}$ ∥ [010] and strain in generic direction<br>• $\boldsymbol{B}$ ∥ [001] and strain in generic direction<br>• $\boldsymbol{B}$ ⊥ [100] and strain ⊥ [010]<br>• $\boldsymbol{B}$ ⊥ [100] and strain ⊥ [001]<br>• $\boldsymbol{B}$ ⊥ [100] and strain in generic direction<br>• $\boldsymbol{B}$ ⊥ [010] and strain ⊥ [100]<br>• $\boldsymbol{B}$ ⊥ [010] and strain ⊥ [001]<br>• $\boldsymbol{B}$ ⊥ [010] and strain in generic direction<br>• $\boldsymbol{B}$ ⊥ [001] and strain ⊥ [100]<br>• $\boldsymbol{B}$ ⊥ [001] and strain ⊥ [010]<br>• $\boldsymbol{B}$ ⊥ [001] and strain in generic direction<br>• $\boldsymbol{B}$ in generic direction

Continued on next page

...continued

Subgroup & SI group	Perturbation
$P2'/c'$ (13.69) $\mathbb{Z}_2 \times \mathbb{Z}_2 \times \mathbb{Z}_4$	• $\boldsymbol{B} \parallel [100]$ and strain $\perp [010]$<br>• $\boldsymbol{B} \parallel [001]$ and strain $\perp [010]$<br>• $\boldsymbol{B} \perp [010]$
$P2_1/c$ (14.75) $\mathbb{Z}_2$	• $\boldsymbol{B} \parallel [010]$ and strain $\perp [010]$
$P_S\bar{1}$ (2.7) $\mathbb{Z}_2 \times \mathbb{Z}_2 \times \mathbb{Z}_4$	• strain in generic direction
P_b2_1/c (14.81) $\mathbb{Z}_2$	• strain $\perp [010]$
Pc (7.24) $\mathbb{Z}_1$	• $\boldsymbol{E} \parallel [010]$ and $\boldsymbol{B} \parallel [100]$ and strain $\perp [100]$<br>• $\boldsymbol{E} \parallel [001]$ and $\boldsymbol{B} \parallel [100]$ and strain $\perp [100]$<br>• $\boldsymbol{E} \perp [100]$ and $\boldsymbol{B} \parallel [100]$
$Pb'a2'$ (32.137) $\mathbb{Z}_1$	• $\boldsymbol{E} \parallel [010]$ and $\boldsymbol{B} \parallel [100]$
$P2_1/c$ (14.75) $\mathbb{Z}_2$	• $\boldsymbol{B} \parallel [100]$ and strain $\perp [100]$
Pc' (7.26) $\mathbb{Z}_1$	• $\boldsymbol{E} \parallel [010]$ and $\boldsymbol{B} \parallel [010]$ and strain $\perp [100]$<br>• $\boldsymbol{E} \parallel [010]$ and $\boldsymbol{B} \parallel [001]$ and strain $\perp [100]$<br>• $\boldsymbol{E} \parallel [010]$ and $\boldsymbol{B} \perp [100]$<br>• $\boldsymbol{E} \parallel [001]$ and $\boldsymbol{B} \parallel [010]$ and strain $\perp [100]$<br>• $\boldsymbol{E} \parallel [001]$ and $\boldsymbol{B} \parallel [001]$ and strain $\perp [100]$<br>• $\boldsymbol{E} \parallel [001]$ and $\boldsymbol{B} \perp [100]$<br>• $\boldsymbol{E} \perp [100]$ and $\boldsymbol{B} \parallel [010]$<br>• $\boldsymbol{E} \perp [100]$ and $\boldsymbol{B} \parallel [001]$<br>• $\boldsymbol{E} \perp [100]$ and $\boldsymbol{B} \perp [100]$
$Pn'n2'$ (34.158) $\mathbb{Z}_1$	• $\boldsymbol{E} \parallel [010]$ and $\boldsymbol{B} \parallel [001]$
$Pn'a'2_1$ (33.148) $\mathbb{Z}_1$	• $\boldsymbol{E} \parallel [010]$ and $\boldsymbol{B} \parallel [010]$
$P2_1'/c'$ (14.79) $\mathbb{Z}_2 \times \mathbb{Z}_4$	• $\boldsymbol{B} \parallel [010]$ and strain $\perp [100]$<br>• $\boldsymbol{B} \parallel [001]$ and strain $\perp [100]$<br>• $\boldsymbol{B} \perp [100]$
P_ac (7.27) $\mathbb{Z}_1$	• $\boldsymbol{E} \parallel [010]$ and strain $\perp [100]$<br>• $\boldsymbol{E} \parallel [001]$ and strain $\perp [100]$<br>• $\boldsymbol{E} \perp [100]$
P_cna2_1 (33.151) $\mathbb{Z}_1$	• $\boldsymbol{E} \parallel [010]$
P_a2_1/c (14.80) $\mathbb{Z}_2$	• strain $\perp [100]$
$P2'$ (3.3) $\mathbb{Z}_1$	• $\boldsymbol{E} \parallel [001]$ and $\boldsymbol{B} \parallel [100]$ and strain $\perp [001]$<br>• $\boldsymbol{E} \parallel [001]$ and $\boldsymbol{B} \parallel [010]$ and strain $\perp [001]$<br>• $\boldsymbol{E} \parallel [001]$ and $\boldsymbol{B} \perp [001]$

Continued on next page

...continued

Subgroup & SI group	Perturbation
$P2'/c'$ (13.69) $\mathbb{Z}_2 \times \mathbb{Z}_2 \times \mathbb{Z}_4$	• $\boldsymbol{B}$ ∥ [100] and strain ⊥ [001]<br>• $\boldsymbol{B}$ ∥ [010] and strain ⊥ [001]<br>• $\boldsymbol{B}$ ⊥ [001]
$Pc'c2'$ (27.80) $\mathbb{Z}_1$	• $\boldsymbol{E}$ ∥ [001] and $\boldsymbol{B}$ ∥ [100]
$Pcc'a'$ (54.343) $\mathbb{Z}_2$	• $\boldsymbol{B}$ ∥ [100]
$Pn'c2'$ (30.113) $\mathbb{Z}_1$	• $\boldsymbol{E}$ ∥ [001] and $\boldsymbol{B}$ ∥ [010]
$Pbc'n'$ (60.423) $\mathbb{Z}_2$	• $\boldsymbol{B}$ ∥ [010]
$P2$ (3.1) $\mathbb{Z}_2$	• $\boldsymbol{E}$ ∥ [001] and $\boldsymbol{B}$ ∥ [001] and strain ⊥ [001]
$P2/c$ (13.65) $\mathbb{Z}_2 \times \mathbb{Z}_2$	• $\boldsymbol{B}$ ∥ [001] and strain ⊥ [001]
$Pn'c'2$ (30.115) $\mathbb{Z}_1$	• $\boldsymbol{E}$ ∥ [001] and $\boldsymbol{B}$ ∥ [001]
$Pnn'a'$ (52.311) $\mathbb{Z}_2$	• $\boldsymbol{B}$ ∥ [001]
P_a2 (3.4) $\mathbb{Z}_2$	• $\boldsymbol{E}$ ∥ [001] and strain ⊥ [001]
P_a2/c (13.70) $\mathbb{Z}_2 \times \mathbb{Z}_2$	• strain ⊥ [001]
P_acc2 (27.83) $\mathbb{Z}_2$	• $\boldsymbol{E}$ ∥ [001]

Table. S 483: Subgroups of P_cccn (56.373) that can be realized by applying $\boldsymbol{E}/\boldsymbol{B}$ fields and/or uniaxial strains.

Subgroup & SI group	Perturbation
P_S1 (1.3) $\mathbb{Z}_1$	• $\boldsymbol{E}$ ∥ [100] and strain in generic direction<br>• $\boldsymbol{E}$ ∥ [010] and strain in generic direction<br>• $\boldsymbol{E}$ ∥ [001] and strain in generic direction<br>• $\boldsymbol{E}$ ⊥ [100] and strain ⊥ [010]<br>• $\boldsymbol{E}$ ⊥ [100] and strain ⊥ [001]<br>• $\boldsymbol{E}$ ⊥ [100] and strain in generic direction<br>• $\boldsymbol{E}$ ⊥ [010] and strain ⊥ [100]<br>• $\boldsymbol{E}$ ⊥ [010] and strain ⊥ [001]<br>• $\boldsymbol{E}$ ⊥ [010] and strain in generic direction<br>• $\boldsymbol{E}$ ⊥ [001] and strain ⊥ [100]<br>• $\boldsymbol{E}$ ⊥ [001] and strain ⊥ [010]<br>• $\boldsymbol{E}$ ⊥ [001] and strain in generic direction<br>• $\boldsymbol{E}$ in generic direction

Continued on next page

...continued

Subgroup & SI group	Perturbation
Pm' (6.20) $\mathbb{Z}_1$	• $\boldsymbol{E} \parallel [100]$ and $\boldsymbol{B} \parallel [100]$ and strain $\perp [010]$<br>• $\boldsymbol{E} \parallel [100]$ and $\boldsymbol{B} \parallel [001]$ and strain $\perp [010]$<br>• $\boldsymbol{E} \parallel [100]$ and $\boldsymbol{B} \perp [010]$<br>• $\boldsymbol{E} \parallel [001]$ and $\boldsymbol{B} \parallel [100]$ and strain $\perp [010]$<br>• $\boldsymbol{E} \parallel [001]$ and $\boldsymbol{B} \parallel [001]$ and strain $\perp [010]$<br>• $\boldsymbol{E} \parallel [001]$ and $\boldsymbol{B} \perp [010]$<br>• $\boldsymbol{E} \perp [010]$ and $\boldsymbol{B} \parallel [100]$<br>• $\boldsymbol{E} \perp [010]$ and $\boldsymbol{B} \parallel [001]$<br>• $\boldsymbol{E} \perp [010]$ and $\boldsymbol{B} \perp [010]$
Pc (7.24) $\mathbb{Z}_1$	• $\boldsymbol{E} \parallel [100]$ and $\boldsymbol{B} \parallel [010]$ and strain $\perp [010]$<br>• $\boldsymbol{E} \parallel [001]$ and $\boldsymbol{B} \parallel [010]$ and strain $\perp [010]$<br>• $\boldsymbol{E} \perp [010]$ and $\boldsymbol{B} \parallel [010]$
P_cc (7.28) $\mathbb{Z}_1$	• $\boldsymbol{E} \parallel [100]$ and strain $\perp [010]$<br>• $\boldsymbol{E} \parallel [001]$ and strain $\perp [010]$<br>• $\boldsymbol{E} \perp [010]$
Pc (7.24) $\mathbb{Z}_1$	• $\boldsymbol{E} \parallel [100]$ and $\boldsymbol{B} \parallel [001]$ and strain $\perp [001]$<br>• $\boldsymbol{E} \parallel [010]$ and $\boldsymbol{B} \parallel [001]$ and strain $\perp [001]$<br>• $\boldsymbol{E} \perp [001]$ and $\boldsymbol{B} \parallel [001]$
Pc' (7.26) $\mathbb{Z}_1$	• $\boldsymbol{E} \parallel [100]$ and $\boldsymbol{B} \parallel [100]$ and strain $\perp [001]$<br>• $\boldsymbol{E} \parallel [100]$ and $\boldsymbol{B} \parallel [010]$ and strain $\perp [001]$<br>• $\boldsymbol{E} \parallel [100]$ and $\boldsymbol{B} \perp [001]$<br>• $\boldsymbol{E} \parallel [010]$ and $\boldsymbol{B} \parallel [100]$ and strain $\perp [001]$<br>• $\boldsymbol{E} \parallel [010]$ and $\boldsymbol{B} \parallel [010]$ and strain $\perp [001]$<br>• $\boldsymbol{E} \parallel [010]$ and $\boldsymbol{B} \perp [001]$<br>• $\boldsymbol{E} \perp [001]$ and $\boldsymbol{B} \parallel [100]$<br>• $\boldsymbol{E} \perp [001]$ and $\boldsymbol{B} \parallel [010]$<br>• $\boldsymbol{E} \perp [001]$ and $\boldsymbol{B} \perp [001]$
P_bc (7.29) $\mathbb{Z}_1$	• $\boldsymbol{E} \parallel [100]$ and strain $\perp [001]$<br>• $\boldsymbol{E} \parallel [010]$ and strain $\perp [001]$<br>• $\boldsymbol{E} \perp [001]$
$P2_1'$ (4.9) $\mathbb{Z}_1$	• $\boldsymbol{E} \parallel [100]$ and $\boldsymbol{B} \parallel [010]$ and strain $\perp [100]$<br>• $\boldsymbol{E} \parallel [100]$ and $\boldsymbol{B} \parallel [001]$ and strain $\perp [100]$<br>• $\boldsymbol{E} \parallel [100]$ and $\boldsymbol{B} \perp [100]$
$Pm'n2_1'$ (31.125) $\mathbb{Z}_1$	• $\boldsymbol{E} \parallel [100]$ and $\boldsymbol{B} \parallel [001]$
$Pn'a2_1'$ (33.146) $\mathbb{Z}_1$	• $\boldsymbol{E} \parallel [100]$ and $\boldsymbol{B} \parallel [010]$
$P2_1$ (4.7) $\mathbb{Z}_1$	• $\boldsymbol{E} \parallel [100]$ and $\boldsymbol{B} \parallel [100]$ and strain $\perp [100]$

Continued on next page

...continued

Subgroup & SI group	Perturbation
$Pm'n'2_1$ (31.127) $\mathbb{Z}_1$	• $\boldsymbol{E}$ ∥ [100] and $\boldsymbol{B}$ ∥ [100]
P_a2_1 (4.10) $\mathbb{Z}_1$	• $\boldsymbol{E}$ ∥ [100] and strain ⊥ [100]
P_ana2_1 (33.149) $\mathbb{Z}_1$	• $\boldsymbol{E}$ ∥ [100]
$P2_1'$ (4.9) $\mathbb{Z}_1$	• $\boldsymbol{E}$ ∥ [010] and $\boldsymbol{B}$ ∥ [100] and strain ⊥ [010]<br>• $\boldsymbol{E}$ ∥ [010] and $\boldsymbol{B}$ ∥ [001] and strain ⊥ [010]<br>• $\boldsymbol{E}$ ∥ [010] and $\boldsymbol{B}$ ⊥ [010]
$P2_1$ (4.7) $\mathbb{Z}_1$	• $\boldsymbol{E}$ ∥ [010] and $\boldsymbol{B}$ ∥ [010] and strain ⊥ [010]
P_a2_1 (4.10) $\mathbb{Z}_1$	• $\boldsymbol{E}$ ∥ [010] and strain ⊥ [010]
$P\bar{1}$ (2.4) $\mathbb{Z}_2\times\mathbb{Z}_2\times\mathbb{Z}_2\times\mathbb{Z}_4$	• $\boldsymbol{B}$ ∥ [100] and strain in generic direction<br>• $\boldsymbol{B}$ ∥ [010] and strain in generic direction<br>• $\boldsymbol{B}$ ∥ [001] and strain in generic direction<br>• $\boldsymbol{B}$ ⊥ [100] and strain ⊥ [010]<br>• $\boldsymbol{B}$ ⊥ [100] and strain ⊥ [001]<br>• $\boldsymbol{B}$ ⊥ [100] and strain in generic direction<br>• $\boldsymbol{B}$ ⊥ [010] and strain ⊥ [100]<br>• $\boldsymbol{B}$ ⊥ [010] and strain ⊥ [001]<br>• $\boldsymbol{B}$ ⊥ [010] and strain in generic direction<br>• $\boldsymbol{B}$ ⊥ [001] and strain ⊥ [100]<br>• $\boldsymbol{B}$ ⊥ [001] and strain ⊥ [010]<br>• $\boldsymbol{B}$ ⊥ [001] and strain in generic direction<br>• $\boldsymbol{B}$ in generic direction
$P2_1'/m'$ (11.54) $\mathbb{Z}_2\times\mathbb{Z}_2\times\mathbb{Z}_4$	• $\boldsymbol{B}$ ∥ [100] and strain ⊥ [010]<br>• $\boldsymbol{B}$ ∥ [001] and strain ⊥ [010]<br>• $\boldsymbol{B}$ ⊥ [010]
$P2_1/c$ (14.75) $\mathbb{Z}_2$	• $\boldsymbol{B}$ ∥ [010] and strain ⊥ [010]
$P_S\bar{1}$ (2.7) $\mathbb{Z}_2\times\mathbb{Z}_2\times\mathbb{Z}_4$	• strain in generic direction
P_c2_1/c (14.82) $\mathbb{Z}_2$	• strain ⊥ [010]
Pm' (6.20) $\mathbb{Z}_1$	• $\boldsymbol{E}$ ∥ [010] and $\boldsymbol{B}$ ∥ [010] and strain ⊥ [100]<br>• $\boldsymbol{E}$ ∥ [010] and $\boldsymbol{B}$ ∥ [001] and strain ⊥ [100]<br>• $\boldsymbol{E}$ ∥ [010] and $\boldsymbol{B}$ ⊥ [100]<br>• $\boldsymbol{E}$ ∥ [001] and $\boldsymbol{B}$ ∥ [010] and strain ⊥ [100]<br>• $\boldsymbol{E}$ ∥ [001] and $\boldsymbol{B}$ ∥ [001] and strain ⊥ [100]<br>• $\boldsymbol{E}$ ∥ [001] and $\boldsymbol{B}$ ⊥ [100]<br>• $\boldsymbol{E}$ ⊥ [100] and $\boldsymbol{B}$ ∥ [010]<br>• $\boldsymbol{E}$ ⊥ [100] and $\boldsymbol{B}$ ∥ [001]<br>• $\boldsymbol{E}$ ⊥ [100] and $\boldsymbol{B}$ ⊥ [100]

Continued on next page

...continued

Subgroup & SI group	Perturbation
$Pm'n2_1'$ (31.125) $\mathbb{Z}_1$	• $\boldsymbol{E}$ || [010] and $\boldsymbol{B}$ || [001]
$Pm'n'2_1$ (31.127) $\mathbb{Z}_1$	• $\boldsymbol{E}$ || [010] and $\boldsymbol{B}$ || [010]
$P2_1'/m'$ (11.54) $\mathbb{Z}_2\times\mathbb{Z}_2\times\mathbb{Z}_4$	• $\boldsymbol{B}$ || [010] and strain $\perp$ [100]<br>• $\boldsymbol{B}$ || [001] and strain $\perp$ [100]<br>• $\boldsymbol{B}\perp$ [100]
Pc (7.24) $\mathbb{Z}_1$	• $\boldsymbol{E}$ || [010] and $\boldsymbol{B}$ || [100] and strain $\perp$ [100]<br>• $\boldsymbol{E}$ || [001] and $\boldsymbol{B}$ || [100] and strain $\perp$ [100]<br>• $\boldsymbol{E}\perp$ [100] and $\boldsymbol{B}$ || [100]
$Pn'a2_1'$ (33.146) $\mathbb{Z}_1$	• $\boldsymbol{E}$ || [010] and $\boldsymbol{B}$ || [100]
$P2_1/c$ (14.75) $\mathbb{Z}_2$	• $\boldsymbol{B}$ || [100] and strain $\perp$ [100]
P_cc (7.28) $\mathbb{Z}_1$	• $\boldsymbol{E}$ || [010] and strain $\perp$ [100]<br>• $\boldsymbol{E}$ || [001] and strain $\perp$ [100]<br>• $\boldsymbol{E}\perp$ [100]
P_ana2_1 (33.149) $\mathbb{Z}_1$	• $\boldsymbol{E}$ || [010]
P_c2_1/c (14.82) $\mathbb{Z}_2$	• strain $\perp$ [100]
$P2$ (3.1) $\mathbb{Z}_2$	• $\boldsymbol{E}$ || [001] and $\boldsymbol{B}$ || [001] and strain $\perp$ [001]
$P2/c$ (13.65) $\mathbb{Z}_2\times\mathbb{Z}_2$	• $\boldsymbol{B}$ || [001] and strain $\perp$ [001]
$Pm'm'2$ (25.60) $\mathbb{Z}_2$	• $\boldsymbol{E}$ || [001] and $\boldsymbol{B}$ || [001]
$Pm'm'n$ (59.409) $\mathbb{Z}_2\times\mathbb{Z}_2$	• $\boldsymbol{B}$ || [001]
$P2_1'$ (4.9) $\mathbb{Z}_1$	• $\boldsymbol{E}$ || [001] and $\boldsymbol{B}$ || [100] and strain $\perp$ [001]<br>• $\boldsymbol{E}$ || [001] and $\boldsymbol{B}$ || [010] and strain $\perp$ [001]<br>• $\boldsymbol{E}$ || [001] and $\boldsymbol{B}\perp$ [001]
$P2_1'/c'$ (14.79) $\mathbb{Z}_2\times\mathbb{Z}_4$	• $\boldsymbol{B}$ || [100] and strain $\perp$ [001]<br>• $\boldsymbol{B}$ || [010] and strain $\perp$ [001]<br>• $\boldsymbol{B}\perp$ [001]
$Pm'c2_1'$ (26.68) $\mathbb{Z}_1$	• $\boldsymbol{E}$ || [001] and $\boldsymbol{B}$ || [010]
$Pn'm'a$ (62.446) $\mathbb{Z}_2$	• $\boldsymbol{B}$ || [010]
$Pm'c2_1'$ (26.68) $\mathbb{Z}_1$	• $\boldsymbol{E}$ || [001] and $\boldsymbol{B}$ || [100]
$Pn'm'a$ (62.446) $\mathbb{Z}_2$	• $\boldsymbol{B}$ || [100]
P_b2 (3.5) $\mathbb{Z}_2$	• $\boldsymbol{E}$ || [001] and strain $\perp$ [001]
P_b2/c (13.71) $\mathbb{Z}_2\times\mathbb{Z}_2$	• strain $\perp$ [001]
P_ccc2 (27.82) $\mathbb{Z}_2$	• $\boldsymbol{E}$ || [001]

Table. S 484: Subgroups of P_Accn (56.374) that can be realized by applying $\boldsymbol{E}/\boldsymbol{B}$ fields and/or uniaxial strains.

Subgroup & SI group	Perturbation
P_S1 (1.3) $\mathbb{Z}_1$	• $\boldsymbol{E} \parallel [100]$ and strain in generic direction<br>• $\boldsymbol{E} \parallel [010]$ and strain in generic direction<br>• $\boldsymbol{E} \parallel [001]$ and strain in generic direction<br>• $\boldsymbol{E} \perp [100]$ and strain $\perp [010]$<br>• $\boldsymbol{E} \perp [100]$ and strain $\perp [001]$<br>• $\boldsymbol{E} \perp [100]$ and strain in generic direction<br>• $\boldsymbol{E} \perp [010]$ and strain $\perp [100]$<br>• $\boldsymbol{E} \perp [010]$ and strain $\perp [001]$<br>• $\boldsymbol{E} \perp [010]$ and strain in generic direction<br>• $\boldsymbol{E} \perp [001]$ and strain $\perp [100]$<br>• $\boldsymbol{E} \perp [001]$ and strain $\perp [010]$<br>• $\boldsymbol{E} \perp [001]$ and strain in generic direction<br>• $\boldsymbol{E}$ in generic direction
Pm' (6.20) $\mathbb{Z}_1$	• $\boldsymbol{E} \parallel [100]$ and $\boldsymbol{B} \parallel [100]$ and strain $\perp [010]$<br>• $\boldsymbol{E} \parallel [100]$ and $\boldsymbol{B} \parallel [001]$ and strain $\perp [010]$<br>• $\boldsymbol{E} \parallel [100]$ and $\boldsymbol{B} \perp [010]$<br>• $\boldsymbol{E} \parallel [001]$ and $\boldsymbol{B} \parallel [100]$ and strain $\perp [010]$<br>• $\boldsymbol{E} \parallel [001]$ and $\boldsymbol{B} \parallel [001]$ and strain $\perp [010]$<br>• $\boldsymbol{E} \parallel [001]$ and $\boldsymbol{B} \perp [010]$<br>• $\boldsymbol{E} \perp [010]$ and $\boldsymbol{B} \parallel [100]$<br>• $\boldsymbol{E} \perp [010]$ and $\boldsymbol{B} \parallel [001]$<br>• $\boldsymbol{E} \perp [010]$ and $\boldsymbol{B} \perp [010]$
Pc (7.24) $\mathbb{Z}_1$	• $\boldsymbol{E} \parallel [100]$ and $\boldsymbol{B} \parallel [010]$ and strain $\perp [010]$<br>• $\boldsymbol{E} \parallel [001]$ and $\boldsymbol{B} \parallel [010]$ and strain $\perp [010]$<br>• $\boldsymbol{E} \perp [010]$ and $\boldsymbol{B} \parallel [010]$
P_Ac (7.31) $\mathbb{Z}_1$	• $\boldsymbol{E} \parallel [100]$ and strain $\perp [010]$<br>• $\boldsymbol{E} \parallel [001]$ and strain $\perp [010]$<br>• $\boldsymbol{E} \perp [010]$

Continued on next page

...continued

Subgroup & SI group	Perturbation
Pc' (7.26) $\mathbb{Z}_1$	• $\boldsymbol{E} \parallel [100]$ and $\boldsymbol{B} \parallel [100]$ and strain $\perp [001]$<br>• $\boldsymbol{E} \parallel [100]$ and $\boldsymbol{B} \parallel [010]$ and strain $\perp [001]$<br>• $\boldsymbol{E} \parallel [100]$ and $\boldsymbol{B} \perp [001]$<br>• $\boldsymbol{E} \parallel [010]$ and $\boldsymbol{B} \parallel [100]$ and strain $\perp [001]$<br>• $\boldsymbol{E} \parallel [010]$ and $\boldsymbol{B} \parallel [010]$ and strain $\perp [001]$<br>• $\boldsymbol{E} \parallel [010]$ and $\boldsymbol{B} \perp [001]$<br>• $\boldsymbol{E} \perp [001]$ and $\boldsymbol{B} \parallel [100]$<br>• $\boldsymbol{E} \perp [001]$ and $\boldsymbol{B} \parallel [010]$<br>• $\boldsymbol{E} \perp [001]$ and $\boldsymbol{B} \perp [001]$
Pc (7.24) $\mathbb{Z}_1$	• $\boldsymbol{E} \parallel [100]$ and $\boldsymbol{B} \parallel [001]$ and strain $\perp [001]$<br>• $\boldsymbol{E} \parallel [010]$ and $\boldsymbol{B} \parallel [001]$ and strain $\perp [001]$<br>• $\boldsymbol{E} \perp [001]$ and $\boldsymbol{B} \parallel [001]$
P_Cc (7.30) $\mathbb{Z}_1$	• $\boldsymbol{E} \parallel [100]$ and strain $\perp [001]$<br>• $\boldsymbol{E} \parallel [010]$ and strain $\perp [001]$<br>• $\boldsymbol{E} \perp [001]$
$P2_1$ (4.7) $\mathbb{Z}_1$	• $\boldsymbol{E} \parallel [100]$ and $\boldsymbol{B} \parallel [100]$ and strain $\perp [100]$
$Pm'c'2_1$ (26.70) $\mathbb{Z}_1$	• $\boldsymbol{E} \parallel [100]$ and $\boldsymbol{B} \parallel [100]$
$P2_1'$ (4.9) $\mathbb{Z}_1$	• $\boldsymbol{E} \parallel [100]$ and $\boldsymbol{B} \parallel [010]$ and strain $\perp [100]$<br>• $\boldsymbol{E} \parallel [100]$ and $\boldsymbol{B} \parallel [001]$ and strain $\perp [100]$<br>• $\boldsymbol{E} \parallel [100]$ and $\boldsymbol{B} \perp [100]$
$Pc'a2_1'$ (29.101) $\mathbb{Z}_1$	• $\boldsymbol{E} \parallel [100]$ and $\boldsymbol{B} \parallel [010]$
$Pm'n2_1'$ (31.125) $\mathbb{Z}_1$	• $\boldsymbol{E} \parallel [100]$ and $\boldsymbol{B} \parallel [001]$
P_a2_1 (4.10) $\mathbb{Z}_1$	• $\boldsymbol{E} \parallel [100]$ and strain $\perp [100]$
P_Cna2_1 (33.154) $\mathbb{Z}_1$	• $\boldsymbol{E} \parallel [100]$
$P2'$ (3.3) $\mathbb{Z}_1$	• $\boldsymbol{E} \parallel [010]$ and $\boldsymbol{B} \parallel [100]$ and strain $\perp [010]$<br>• $\boldsymbol{E} \parallel [010]$ and $\boldsymbol{B} \parallel [001]$ and strain $\perp [010]$<br>• $\boldsymbol{E} \parallel [010]$ and $\boldsymbol{B} \perp [010]$
$P2_1$ (4.7) $\mathbb{Z}_1$	• $\boldsymbol{E} \parallel [010]$ and $\boldsymbol{B} \parallel [010]$ and strain $\perp [010]$
P_C2_1 (4.12) $\mathbb{Z}_1$	• $\boldsymbol{E} \parallel [010]$ and strain $\perp [010]$

Continued on next page

...continued

Subgroup & SI group	Perturbation
$P\bar{1}$ (2.4) $\mathbb{Z}_2 \times \mathbb{Z}_2 \times \mathbb{Z}_2 \times \mathbb{Z}_4$	• $\boldsymbol{B}$ ∥ [100] and strain in generic direction<br>• $\boldsymbol{B}$ ∥ [010] and strain in generic direction<br>• $\boldsymbol{B}$ ∥ [001] and strain in generic direction<br>• $\boldsymbol{B} \perp$ [100] and strain $\perp$ [010]<br>• $\boldsymbol{B} \perp$ [100] and strain $\perp$ [001]<br>• $\boldsymbol{B} \perp$ [100] and strain in generic direction<br>• $\boldsymbol{B} \perp$ [010] and strain $\perp$ [100]<br>• $\boldsymbol{B} \perp$ [010] and strain $\perp$ [001]<br>• $\boldsymbol{B} \perp$ [010] and strain in generic direction<br>• $\boldsymbol{B} \perp$ [001] and strain $\perp$ [100]<br>• $\boldsymbol{B} \perp$ [001] and strain $\perp$ [010]<br>• $\boldsymbol{B} \perp$ [001] and strain in generic direction<br>• $\boldsymbol{B}$ in generic direction
$P2'/m'$ (10.46) $\mathbb{Z}_2 \times \mathbb{Z}_2 \times \mathbb{Z}_2 \times \mathbb{Z}_4$	• $\boldsymbol{B}$ ∥ [100] and strain $\perp$ [010]<br>• $\boldsymbol{B}$ ∥ [001] and strain $\perp$ [010]<br>• $\boldsymbol{B} \perp$ [010]
$P2_1/c$ (14.75) $\mathbb{Z}_2$	• $\boldsymbol{B}$ ∥ [010] and strain $\perp$ [010]
$P_S\bar{1}$ (2.7) $\mathbb{Z}_2 \times \mathbb{Z}_2 \times \mathbb{Z}_4$	• strain in generic direction
P_A2_1/c (14.83) $\mathbb{Z}_2$	• strain $\perp$ [010]
Pc (7.24) $\mathbb{Z}_1$	• $\boldsymbol{E}$ ∥ [010] and $\boldsymbol{B}$ ∥ [100] and strain $\perp$ [100]<br>• $\boldsymbol{E}$ ∥ [001] and $\boldsymbol{B}$ ∥ [100] and strain $\perp$ [100]<br>• $\boldsymbol{E} \perp$ [100] and $\boldsymbol{B}$ ∥ [100]
$Pb'a2'$ (32.137) $\mathbb{Z}_1$	• $\boldsymbol{E}$ ∥ [010] and $\boldsymbol{B}$ ∥ [100]
$P2_1/c$ (14.75) $\mathbb{Z}_2$	• $\boldsymbol{B}$ ∥ [100] and strain $\perp$ [100]
Pc' (7.26) $\mathbb{Z}_1$	• $\boldsymbol{E}$ ∥ [010] and $\boldsymbol{B}$ ∥ [010] and strain $\perp$ [100]<br>• $\boldsymbol{E}$ ∥ [010] and $\boldsymbol{B}$ ∥ [001] and strain $\perp$ [100]<br>• $\boldsymbol{E}$ ∥ [010] and $\boldsymbol{B} \perp$ [100]<br>• $\boldsymbol{E}$ ∥ [001] and $\boldsymbol{B}$ ∥ [010] and strain $\perp$ [100]<br>• $\boldsymbol{E}$ ∥ [001] and $\boldsymbol{B}$ ∥ [001] and strain $\perp$ [100]<br>• $\boldsymbol{E}$ ∥ [001] and $\boldsymbol{B} \perp$ [100]<br>• $\boldsymbol{E} \perp$ [100] and $\boldsymbol{B}$ ∥ [010]<br>• $\boldsymbol{E} \perp$ [100] and $\boldsymbol{B}$ ∥ [001]<br>• $\boldsymbol{E} \perp$ [100] and $\boldsymbol{B} \perp$ [100]
$Pnc'2'$ (30.114) $\mathbb{Z}_1$	• $\boldsymbol{E}$ ∥ [010] and $\boldsymbol{B}$ ∥ [001]
$Pc'a'2_1$ (29.103) $\mathbb{Z}_1$	• $\boldsymbol{E}$ ∥ [010] and $\boldsymbol{B}$ ∥ [010]

Continued on next page

. . . continued

Subgroup & SI group	Perturbation
$P2_1'/c'$ (14.79) $\mathbb{Z}_2 \times \mathbb{Z}_4$	• $\boldsymbol{B}$ ∥ [010] and strain ⊥ [100]<br>• $\boldsymbol{B}$ ∥ [001] and strain ⊥ [100]<br>• $\boldsymbol{B}$ ⊥ [100]
P_ac (7.27) $\mathbb{Z}_1$	• $\boldsymbol{E}$ ∥ [010] and strain ⊥ [100]<br>• $\boldsymbol{E}$ ∥ [001] and strain ⊥ [100]<br>• $\boldsymbol{E}$ ⊥ [100]
P_Bna2_1 (33.153) $\mathbb{Z}_1$	• $\boldsymbol{E}$ ∥ [010]
P_a2_1/c (14.80) $\mathbb{Z}_2$	• strain ⊥ [100]
$P2_1'$ (4.9) $\mathbb{Z}_1$	• $\boldsymbol{E}$ ∥ [001] and $\boldsymbol{B}$ ∥ [100] and strain ⊥ [001]<br>• $\boldsymbol{E}$ ∥ [001] and $\boldsymbol{B}$ ∥ [010] and strain ⊥ [001]<br>• $\boldsymbol{E}$ ∥ [001] and $\boldsymbol{B}$ ⊥ [001]
$P2_1'/c'$ (14.79) $\mathbb{Z}_2 \times \mathbb{Z}_4$	• $\boldsymbol{B}$ ∥ [100] and strain ⊥ [001]<br>• $\boldsymbol{B}$ ∥ [010] and strain ⊥ [001]<br>• $\boldsymbol{B}$ ⊥ [001]
$Pm'c2_1'$ (26.68) $\mathbb{Z}_1$	• $\boldsymbol{E}$ ∥ [001] and $\boldsymbol{B}$ ∥ [100]
$Pb'am'$ (55.358) $\mathbb{Z}_2$	• $\boldsymbol{B}$ ∥ [100]
$Pca'2_1'$ (29.102) $\mathbb{Z}_1$	• $\boldsymbol{E}$ ∥ [001] and $\boldsymbol{B}$ ∥ [010]
$Pb'c'a$ (61.436) $\mathbb{Z}_2$	• $\boldsymbol{B}$ ∥ [010]
$P2$ (3.1) $\mathbb{Z}_2$	• $\boldsymbol{E}$ ∥ [001] and $\boldsymbol{B}$ ∥ [001] and strain ⊥ [001]
$P2/c$ (13.65) $\mathbb{Z}_2 \times \mathbb{Z}_2$	• $\boldsymbol{B}$ ∥ [001] and strain ⊥ [001]
$Pm'a'2$ (28.91) $\mathbb{Z}_2$	• $\boldsymbol{E}$ ∥ [001] and $\boldsymbol{B}$ ∥ [001]
$Pm'na'$ (53.328) $\mathbb{Z}_2 \times \mathbb{Z}_2$	• $\boldsymbol{B}$ ∥ [001]
P_C2 (3.6) $\mathbb{Z}_1$	• $\boldsymbol{E}$ ∥ [001] and strain ⊥ [001]
P_C2/c (13.74) $\mathbb{Z}_2$	• strain ⊥ [001]
P_Acc2 (27.85) $\mathbb{Z}_2$	• $\boldsymbol{E}$ ∥ [001]

Table. S 485: Subgroups of P_Cccn (56.375) that can be realized by applying $\boldsymbol{E}/\boldsymbol{B}$ fields and/or uniaxial strains.

Subgroup & SI group	Perturbation

Continued on next page

. . . continued

Subgroup & SI group	Perturbation
Pm' (6.20) $\mathbb{Z}_1$	• $\boldsymbol{E} \parallel [100]$ and $\boldsymbol{B} \parallel [100]$ and strain $\perp [001]$<br>• $\boldsymbol{E} \parallel [100]$ and $\boldsymbol{B} \parallel [010]$ and strain $\perp [001]$<br>• $\boldsymbol{E} \parallel [100]$ and $\boldsymbol{B} \perp [001]$<br>• $\boldsymbol{E} \parallel [010]$ and $\boldsymbol{B} \parallel [100]$ and strain $\perp [001]$<br>• $\boldsymbol{E} \parallel [010]$ and $\boldsymbol{B} \parallel [010]$ and strain $\perp [001]$<br>• $\boldsymbol{E} \parallel [010]$ and $\boldsymbol{B} \perp [001]$<br>• $\boldsymbol{E} \perp [001]$ and $\boldsymbol{B} \parallel [100]$<br>• $\boldsymbol{E} \perp [001]$ and $\boldsymbol{B} \parallel [010]$<br>• $\boldsymbol{E} \perp [001]$ and $\boldsymbol{B} \perp [001]$
Pc (7.24) $\mathbb{Z}_1$	• $\boldsymbol{E} \parallel [100]$ and $\boldsymbol{B} \parallel [010]$ and strain $\perp [010]$<br>• $\boldsymbol{E} \parallel [001]$ and $\boldsymbol{B} \parallel [010]$ and strain $\perp [010]$<br>• $\boldsymbol{E} \perp [010]$ and $\boldsymbol{B} \parallel [010]$
$P2'$ (3.3) $\mathbb{Z}_1$	• $\boldsymbol{E} \parallel [100]$ and $\boldsymbol{B} \parallel [010]$ and strain $\perp [100]$<br>• $\boldsymbol{E} \parallel [100]$ and $\boldsymbol{B} \parallel [001]$ and strain $\perp [100]$<br>• $\boldsymbol{E} \parallel [100]$ and $\boldsymbol{B} \perp [100]$
$Pm'a2'$ (28.89) $\mathbb{Z}_1$	• $\boldsymbol{E} \parallel [100]$ and $\boldsymbol{B} \parallel [010]$
P_S1 (1.3) $\mathbb{Z}_1$	• $\boldsymbol{E} \parallel [100]$ and strain in generic direction<br>• $\boldsymbol{E} \parallel [010]$ and strain in generic direction<br>• $\boldsymbol{E} \parallel [001]$ and strain in generic direction<br>• $\boldsymbol{E} \perp [100]$ and strain $\perp [010]$<br>• $\boldsymbol{E} \perp [100]$ and strain $\perp [001]$<br>• $\boldsymbol{E} \perp [100]$ and strain in generic direction<br>• $\boldsymbol{E} \perp [010]$ and strain $\perp [100]$<br>• $\boldsymbol{E} \perp [010]$ and strain $\perp [001]$<br>• $\boldsymbol{E} \perp [010]$ and strain in generic direction<br>• $\boldsymbol{E} \perp [001]$ and strain $\perp [100]$<br>• $\boldsymbol{E} \perp [001]$ and strain $\perp [010]$<br>• $\boldsymbol{E} \perp [001]$ and strain in generic direction<br>• $\boldsymbol{E}$ in generic direction
Pc (7.24) $\mathbb{Z}_1$	• $\boldsymbol{E} \parallel [100]$ and $\boldsymbol{B} \parallel [001]$ and strain $\perp [001]$<br>• $\boldsymbol{E} \parallel [010]$ and $\boldsymbol{B} \parallel [001]$ and strain $\perp [001]$<br>• $\boldsymbol{E} \perp [001]$ and $\boldsymbol{B} \parallel [001]$
P_cc (7.28) $\mathbb{Z}_1$	• $\boldsymbol{E} \parallel [100]$ and strain $\perp [001]$<br>• $\boldsymbol{E} \parallel [010]$ and strain $\perp [001]$<br>• $\boldsymbol{E} \perp [001]$

Continued on next page

...continued

Subgroup & SI group	Perturbation
Pc' (7.26) $\mathbb{Z}_1$	• $\boldsymbol{E}$ ∥ [100] and $\boldsymbol{B}$ ∥ [100] and strain ⊥ [010]<br>• $\boldsymbol{E}$ ∥ [100] and $\boldsymbol{B}$ ∥ [001] and strain ⊥ [010]<br>• $\boldsymbol{E}$ ∥ [100] and $\boldsymbol{B}$ ⊥ [010]<br>• $\boldsymbol{E}$ ∥ [001] and $\boldsymbol{B}$ ∥ [100] and strain ⊥ [010]<br>• $\boldsymbol{E}$ ∥ [001] and $\boldsymbol{B}$ ∥ [001] and strain ⊥ [010]<br>• $\boldsymbol{E}$ ∥ [001] and $\boldsymbol{B}$ ⊥ [010]<br>• $\boldsymbol{E}$ ⊥ [010] and $\boldsymbol{B}$ ∥ [100]<br>• $\boldsymbol{E}$ ⊥ [010] and $\boldsymbol{B}$ ∥ [001]<br>• $\boldsymbol{E}$ ⊥ [010] and $\boldsymbol{B}$ ⊥ [010]
P_Cc (7.30) $\mathbb{Z}_1$	• $\boldsymbol{E}$ ∥ [100] and strain ⊥ [010]<br>• $\boldsymbol{E}$ ∥ [001] and strain ⊥ [010]<br>• $\boldsymbol{E}$ ⊥ [010]
$Pn'n2'$ (34.158) $\mathbb{Z}_1$	• $\boldsymbol{E}$ ∥ [100] and $\boldsymbol{B}$ ∥ [001]
$P2_1$ (4.7) $\mathbb{Z}_1$	• $\boldsymbol{E}$ ∥ [100] and $\boldsymbol{B}$ ∥ [100] and strain ⊥ [100]
P_C2_1 (4.12) $\mathbb{Z}_1$	• $\boldsymbol{E}$ ∥ [100] and strain ⊥ [100]
$Pm'n'2_1$ (31.127) $\mathbb{Z}_1$	• $\boldsymbol{E}$ ∥ [100] and $\boldsymbol{B}$ ∥ [100]
P_Ana2_1 (33.152) $\mathbb{Z}_1$	• $\boldsymbol{E}$ ∥ [100]
Pc' (7.26) $\mathbb{Z}_1$	• $\boldsymbol{E}$ ∥ [010] and $\boldsymbol{B}$ ∥ [010] and strain ⊥ [100]<br>• $\boldsymbol{E}$ ∥ [010] and $\boldsymbol{B}$ ∥ [001] and strain ⊥ [100]<br>• $\boldsymbol{E}$ ∥ [010] and $\boldsymbol{B}$ ⊥ [100]<br>• $\boldsymbol{E}$ ∥ [001] and $\boldsymbol{B}$ ∥ [010] and strain ⊥ [100]<br>• $\boldsymbol{E}$ ∥ [001] and $\boldsymbol{B}$ ∥ [001] and strain ⊥ [100]<br>• $\boldsymbol{E}$ ∥ [001] and $\boldsymbol{B}$ ⊥ [100]<br>• $\boldsymbol{E}$ ⊥ [100] and $\boldsymbol{B}$ ∥ [010]<br>• $\boldsymbol{E}$ ⊥ [100] and $\boldsymbol{B}$ ∥ [001]<br>• $\boldsymbol{E}$ ⊥ [100] and $\boldsymbol{B}$ ⊥ [100]
$P2_1$ (4.7) $\mathbb{Z}_1$	• $\boldsymbol{E}$ ∥ [010] and $\boldsymbol{B}$ ∥ [010] and strain ⊥ [010]
$Pm'n'2_1$ (31.127) $\mathbb{Z}_1$	• $\boldsymbol{E}$ ∥ [010] and $\boldsymbol{B}$ ∥ [010]
$P2'$ (3.3) $\mathbb{Z}_1$	• $\boldsymbol{E}$ ∥ [001] and $\boldsymbol{B}$ ∥ [100] and strain ⊥ [001]<br>• $\boldsymbol{E}$ ∥ [001] and $\boldsymbol{B}$ ∥ [010] and strain ⊥ [001]<br>• $\boldsymbol{E}$ ∥ [001] and $\boldsymbol{B}$ ⊥ [001]
$Pn'c2'$ (30.113) $\mathbb{Z}_1$	• $\boldsymbol{E}$ ∥ [001] and $\boldsymbol{B}$ ∥ [010]

Continued on next page

...continued

Subgroup & SI group	Perturbation
$P\bar{1}$ (2.4) $\mathbb{Z}_2\times\mathbb{Z}_2\times\mathbb{Z}_2\times\mathbb{Z}_4$	• $\boldsymbol{B}$ ∥ [100] and strain in generic direction<br>• $\boldsymbol{B}$ ∥ [010] and strain in generic direction<br>• $\boldsymbol{B}$ ∥ [001] and strain in generic direction<br>• $\boldsymbol{B}\perp$ [100] and strain $\perp$ [010]<br>• $\boldsymbol{B}\perp$ [100] and strain $\perp$ [001]<br>• $\boldsymbol{B}\perp$ [100] and strain in generic direction<br>• $\boldsymbol{B}\perp$ [010] and strain $\perp$ [100]<br>• $\boldsymbol{B}\perp$ [010] and strain $\perp$ [001]<br>• $\boldsymbol{B}\perp$ [010] and strain in generic direction<br>• $\boldsymbol{B}\perp$ [001] and strain $\perp$ [100]<br>• $\boldsymbol{B}\perp$ [001] and strain $\perp$ [010]<br>• $\boldsymbol{B}\perp$ [001] and strain in generic direction<br>• $\boldsymbol{B}$ in generic direction
$P2'/c'$ (13.69) $\mathbb{Z}_2\times\mathbb{Z}_2\times\mathbb{Z}_4$	• $\boldsymbol{B}$ ∥ [010] and strain $\perp$ [100]<br>• $\boldsymbol{B}$ ∥ [001] and strain $\perp$ [100]<br>• $\boldsymbol{B}\perp$ [100]
$P2_1/c$ (14.75) $\mathbb{Z}_2$	• $\boldsymbol{B}$ ∥ [010] and strain $\perp$ [010]
$P2'/m'$ (10.46) $\mathbb{Z}_2\times\mathbb{Z}_2\times\mathbb{Z}_2\times\mathbb{Z}_4$	• $\boldsymbol{B}$ ∥ [100] and strain $\perp$ [001]<br>• $\boldsymbol{B}$ ∥ [010] and strain $\perp$ [001]<br>• $\boldsymbol{B}\perp$ [001]
$Pm'n'a$ (53.326) $\mathbb{Z}_2$	• $\boldsymbol{B}$ ∥ [010]
Pc (7.24) $\mathbb{Z}_1$	• $\boldsymbol{E}$ ∥ [010] and $\boldsymbol{B}$ ∥ [100] and strain $\perp$ [100]<br>• $\boldsymbol{E}$ ∥ [001] and $\boldsymbol{B}$ ∥ [100] and strain $\perp$ [100]<br>• $\boldsymbol{E}\perp$ [100] and $\boldsymbol{B}$ ∥ [100]
P_Cc (7.30) $\mathbb{Z}_1$	• $\boldsymbol{E}$ ∥ [010] and strain $\perp$ [100]<br>• $\boldsymbol{E}$ ∥ [001] and strain $\perp$ [100]<br>• $\boldsymbol{E}\perp$ [100]
$Pn'c2'$ (30.113) $\mathbb{Z}_1$	• $\boldsymbol{E}$ ∥ [001] and $\boldsymbol{B}$ ∥ [100]
$P2_1/c$ (14.75) $\mathbb{Z}_2$	• $\boldsymbol{B}$ ∥ [100] and strain $\perp$ [100]
$P2'$ (3.3) $\mathbb{Z}_1$	• $\boldsymbol{E}$ ∥ [010] and $\boldsymbol{B}$ ∥ [100] and strain $\perp$ [010]<br>• $\boldsymbol{E}$ ∥ [010] and $\boldsymbol{B}$ ∥ [001] and strain $\perp$ [010]<br>• $\boldsymbol{E}$ ∥ [010] and $\boldsymbol{B}\perp$ [010]
$Pn'n2'$ (34.158) $\mathbb{Z}_1$	• $\boldsymbol{E}$ ∥ [010] and $\boldsymbol{B}$ ∥ [001]
P_C2_1 (4.12) $\mathbb{Z}_1$	• $\boldsymbol{E}$ ∥ [010] and strain $\perp$ [010]

Continued on next page

…continued

Subgroup & SI group	Perturbation
$P2'/c'$ (13.69) $\mathbb{Z}_2 \times \mathbb{Z}_2 \times \mathbb{Z}_4$	• $\boldsymbol{B} \parallel [100]$ and strain $\perp [010]$<br>• $\boldsymbol{B} \parallel [001]$ and strain $\perp [010]$<br>• $\boldsymbol{B} \perp [010]$
$Pm'a2'$ (28.89) $\mathbb{Z}_1$	• $\boldsymbol{E} \parallel [010]$ and $\boldsymbol{B} \parallel [100]$
P_Ana2_1 (33.152) $\mathbb{Z}_1$	• $\boldsymbol{E} \parallel [010]$
$Pm'n'a$ (53.326) $\mathbb{Z}_2$	• $\boldsymbol{B} \parallel [100]$
$P2$ (3.1) $\mathbb{Z}_2$	• $\boldsymbol{E} \parallel [001]$ and $\boldsymbol{B} \parallel [001]$ and strain $\perp [001]$
$Pn'n'2$ (34.159) $\mathbb{Z}_1$	• $\boldsymbol{E} \parallel [001]$ and $\boldsymbol{B} \parallel [001]$
P_a2 (3.4) $\mathbb{Z}_2$	• $\boldsymbol{E} \parallel [001]$ and strain $\perp [001]$
$P2/c$ (13.65) $\mathbb{Z}_2 \times \mathbb{Z}_2$	• $\boldsymbol{B} \parallel [001]$ and strain $\perp [001]$
P_Ccc2 (27.84) $\mathbb{Z}_2$	• $\boldsymbol{E} \parallel [001]$
$Pn'n'n$ (48.260) $\mathbb{Z}_2$	• $\boldsymbol{B} \parallel [001]$
$P_S\bar{1}$ (2.7) $\mathbb{Z}_2 \times \mathbb{Z}_2 \times \mathbb{Z}_4$	• strain in generic direction
P_C2_1/c (14.84) $\mathbb{Z}_2$	• strain $\perp [100]$
P_C2_1/c (14.84) $\mathbb{Z}_2$	• strain $\perp [010]$
P_c2/c (13.72) $\mathbb{Z}_2 \times \mathbb{Z}_2$	• strain $\perp [001]$

Table. S 486: Subgroups of P_Iccn (56.376) that can be realized by applying $\boldsymbol{E}/\boldsymbol{B}$ fields and/or uniaxial strains.

Subgroup & SI group	Perturbation
Pm' (6.20) $\mathbb{Z}_1$	• $\boldsymbol{E} \parallel [100]$ and $\boldsymbol{B} \parallel [100]$ and strain $\perp [001]$<br>• $\boldsymbol{E} \parallel [100]$ and $\boldsymbol{B} \parallel [010]$ and strain $\perp [001]$<br>• $\boldsymbol{E} \parallel [100]$ and $\boldsymbol{B} \perp [001]$<br>• $\boldsymbol{E} \parallel [010]$ and $\boldsymbol{B} \parallel [100]$ and strain $\perp [001]$<br>• $\boldsymbol{E} \parallel [010]$ and $\boldsymbol{B} \parallel [010]$ and strain $\perp [001]$<br>• $\boldsymbol{E} \parallel [010]$ and $\boldsymbol{B} \perp [001]$<br>• $\boldsymbol{E} \perp [001]$ and $\boldsymbol{B} \parallel [100]$<br>• $\boldsymbol{E} \perp [001]$ and $\boldsymbol{B} \parallel [010]$<br>• $\boldsymbol{E} \perp [001]$ and $\boldsymbol{B} \perp [001]$

Continued on next page

...continued

Subgroup & SI group	Perturbation
Pc (7.24) $\mathbb{Z}_1$	• $\boldsymbol{E} \parallel [100]$ and $\boldsymbol{B} \parallel [010]$ and strain $\perp [010]$<br>• $\boldsymbol{E} \parallel [001]$ and $\boldsymbol{B} \parallel [010]$ and strain $\perp [010]$<br>• $\boldsymbol{E} \perp [010]$ and $\boldsymbol{B} \parallel [010]$
$P2'$ (3.3) $\mathbb{Z}_1$	• $\boldsymbol{E} \parallel [100]$ and $\boldsymbol{B} \parallel [010]$ and strain $\perp [100]$<br>• $\boldsymbol{E} \parallel [100]$ and $\boldsymbol{B} \parallel [001]$ and strain $\perp [100]$<br>• $\boldsymbol{E} \parallel [100]$ and $\boldsymbol{B} \perp [100]$
$Pm'a2'$ (28.89) $\mathbb{Z}_1$	• $\boldsymbol{E} \parallel [100]$ and $\boldsymbol{B} \parallel [010]$
P_S1 (1.3) $\mathbb{Z}_1$	• $\boldsymbol{E} \parallel [100]$ and strain in generic direction<br>• $\boldsymbol{E} \parallel [010]$ and strain in generic direction<br>• $\boldsymbol{E} \parallel [001]$ and strain in generic direction<br>• $\boldsymbol{E} \perp [100]$ and strain $\perp [010]$<br>• $\boldsymbol{E} \perp [100]$ and strain $\perp [001]$<br>• $\boldsymbol{E} \perp [100]$ and strain in generic direction<br>• $\boldsymbol{E} \perp [010]$ and strain $\perp [100]$<br>• $\boldsymbol{E} \perp [010]$ and strain $\perp [001]$<br>• $\boldsymbol{E} \perp [010]$ and strain in generic direction<br>• $\boldsymbol{E} \perp [001]$ and strain $\perp [100]$<br>• $\boldsymbol{E} \perp [001]$ and strain $\perp [010]$<br>• $\boldsymbol{E} \perp [001]$ and strain in generic direction<br>• $\boldsymbol{E}$ in generic direction
Pc (7.24) $\mathbb{Z}_1$	• $\boldsymbol{E} \parallel [100]$ and $\boldsymbol{B} \parallel [001]$ and strain $\perp [001]$<br>• $\boldsymbol{E} \parallel [010]$ and $\boldsymbol{B} \parallel [001]$ and strain $\perp [001]$<br>• $\boldsymbol{E} \perp [001]$ and $\boldsymbol{B} \parallel [001]$
P_Ac (7.31) $\mathbb{Z}_1$	• $\boldsymbol{E} \parallel [100]$ and strain $\perp [001]$<br>• $\boldsymbol{E} \parallel [010]$ and strain $\perp [001]$<br>• $\boldsymbol{E} \perp [001]$
Pc' (7.26) $\mathbb{Z}_1$	• $\boldsymbol{E} \parallel [100]$ and $\boldsymbol{B} \parallel [100]$ and strain $\perp [010]$<br>• $\boldsymbol{E} \parallel [100]$ and $\boldsymbol{B} \parallel [001]$ and strain $\perp [010]$<br>• $\boldsymbol{E} \parallel [100]$ and $\boldsymbol{B} \perp [010]$<br>• $\boldsymbol{E} \parallel [001]$ and $\boldsymbol{B} \parallel [100]$ and strain $\perp [010]$<br>• $\boldsymbol{E} \parallel [001]$ and $\boldsymbol{B} \parallel [001]$ and strain $\perp [010]$<br>• $\boldsymbol{E} \parallel [001]$ and $\boldsymbol{B} \perp [010]$<br>• $\boldsymbol{E} \perp [010]$ and $\boldsymbol{B} \parallel [100]$<br>• $\boldsymbol{E} \perp [010]$ and $\boldsymbol{B} \parallel [001]$<br>• $\boldsymbol{E} \perp [010]$ and $\boldsymbol{B} \perp [010]$
P_Cc (7.30) $\mathbb{Z}_1$	• $\boldsymbol{E} \parallel [100]$ and strain $\perp [010]$<br>• $\boldsymbol{E} \parallel [001]$ and strain $\perp [010]$<br>• $\boldsymbol{E} \perp [010]$

Continued on next page

. . . continued

Subgroup & SI group	Perturbation
$Pnc'2'$ (30.114) $\mathbb{Z}_1$	• $\boldsymbol{E} \parallel [100]$ and $\boldsymbol{B} \parallel [001]$
$P2_1$ (4.7) $\mathbb{Z}_1$	• $\boldsymbol{E} \parallel [100]$ and $\boldsymbol{B} \parallel [100]$ and strain $\perp [100]$
P_C2_1 (4.12) $\mathbb{Z}_1$	• $\boldsymbol{E} \parallel [100]$ and strain $\perp [100]$
$Pm'c'2_1$ (26.70) $\mathbb{Z}_1$	• $\boldsymbol{E} \parallel [100]$ and $\boldsymbol{B} \parallel [100]$
P_Ina2_1 (33.155) $\mathbb{Z}_1$	• $\boldsymbol{E} \parallel [100]$
Pc' (7.26) $\mathbb{Z}_1$	• $\boldsymbol{E} \parallel [010]$ and $\boldsymbol{B} \parallel [010]$ and strain $\perp [100]$<br>• $\boldsymbol{E} \parallel [010]$ and $\boldsymbol{B} \parallel [001]$ and strain $\perp [100]$<br>• $\boldsymbol{E} \parallel [010]$ and $\boldsymbol{B} \perp [100]$<br>• $\boldsymbol{E} \parallel [001]$ and $\boldsymbol{B} \parallel [010]$ and strain $\perp [100]$<br>• $\boldsymbol{E} \parallel [001]$ and $\boldsymbol{B} \parallel [001]$ and strain $\perp [100]$<br>• $\boldsymbol{E} \parallel [001]$ and $\boldsymbol{B} \perp [100]$<br>• $\boldsymbol{E} \perp [100]$ and $\boldsymbol{B} \parallel [010]$<br>• $\boldsymbol{E} \perp [100]$ and $\boldsymbol{B} \parallel [001]$<br>• $\boldsymbol{E} \perp [100]$ and $\boldsymbol{B} \perp [100]$
$P2_1$ (4.7) $\mathbb{Z}_1$	• $\boldsymbol{E} \parallel [010]$ and $\boldsymbol{B} \parallel [010]$ and strain $\perp [010]$
$Pm'c'2_1$ (26.70) $\mathbb{Z}_1$	• $\boldsymbol{E} \parallel [010]$ and $\boldsymbol{B} \parallel [010]$
$P2_1'$ (4.9) $\mathbb{Z}_1$	• $\boldsymbol{E} \parallel [001]$ and $\boldsymbol{B} \parallel [100]$ and strain $\perp [001]$<br>• $\boldsymbol{E} \parallel [001]$ and $\boldsymbol{B} \parallel [010]$ and strain $\perp [001]$<br>• $\boldsymbol{E} \parallel [001]$ and $\boldsymbol{B} \perp [001]$
$Pca'2_1'$ (29.102) $\mathbb{Z}_1$	• $\boldsymbol{E} \parallel [001]$ and $\boldsymbol{B} \parallel [010]$
$P\bar{1}$ (2.4) $\mathbb{Z}_2 \times \mathbb{Z}_2 \times \mathbb{Z}_2 \times \mathbb{Z}_4$	• $\boldsymbol{B} \parallel [100]$ and strain in generic direction<br>• $\boldsymbol{B} \parallel [010]$ and strain in generic direction<br>• $\boldsymbol{B} \parallel [001]$ and strain in generic direction<br>• $\boldsymbol{B} \perp [100]$ and strain $\perp [010]$<br>• $\boldsymbol{B} \perp [100]$ and strain $\perp [001]$<br>• $\boldsymbol{B} \perp [100]$ and strain in generic direction<br>• $\boldsymbol{B} \perp [010]$ and strain $\perp [100]$<br>• $\boldsymbol{B} \perp [010]$ and strain $\perp [001]$<br>• $\boldsymbol{B} \perp [010]$ and strain in generic direction<br>• $\boldsymbol{B} \perp [001]$ and strain $\perp [100]$<br>• $\boldsymbol{B} \perp [001]$ and strain $\perp [010]$<br>• $\boldsymbol{B} \perp [001]$ and strain in generic direction<br>• $\boldsymbol{B}$ in generic direction
$P2'/c'$ (13.69) $\mathbb{Z}_2 \times \mathbb{Z}_2 \times \mathbb{Z}_4$	• $\boldsymbol{B} \parallel [010]$ and strain $\perp [100]$<br>• $\boldsymbol{B} \parallel [001]$ and strain $\perp [100]$<br>• $\boldsymbol{B} \perp [100]$

Continued on next page

. . . continued

Subgroup & SI group	Perturbation
$P2_1/c$ (14.75) $\mathbb{Z}_2$	• $\boldsymbol{B}$ ∥ [010] and strain ⊥ [010]
$P2_1'/m'$ (11.54) $\mathbb{Z}_2 \times \mathbb{Z}_2 \times \mathbb{Z}_4$	• $\boldsymbol{B}$ ∥ [100] and strain ⊥ [001]<br>• $\boldsymbol{B}$ ∥ [010] and strain ⊥ [001]<br>• $\boldsymbol{B}$ ⊥ [001]
$Pb'cm'$ (57.384) $\mathbb{Z}_2$	• $\boldsymbol{B}$ ∥ [010]
Pc (7.24) $\mathbb{Z}_1$	• $\boldsymbol{E}$ ∥ [010] and $\boldsymbol{B}$ ∥ [100] and strain ⊥ [100]<br>• $\boldsymbol{E}$ ∥ [001] and $\boldsymbol{B}$ ∥ [100] and strain ⊥ [100]<br>• $\boldsymbol{E}$ ⊥ [100] and $\boldsymbol{B}$ ∥ [100]
P_Cc (7.30) $\mathbb{Z}_1$	• $\boldsymbol{E}$ ∥ [010] and strain ⊥ [100]<br>• $\boldsymbol{E}$ ∥ [001] and strain ⊥ [100]<br>• $\boldsymbol{E}$ ⊥ [100]
$Pca'2_1'$ (29.102) $\mathbb{Z}_1$	• $\boldsymbol{E}$ ∥ [001] and $\boldsymbol{B}$ ∥ [100]
$P2_1/c$ (14.75) $\mathbb{Z}_2$	• $\boldsymbol{B}$ ∥ [100] and strain ⊥ [100]
$P2'$ (3.3) $\mathbb{Z}_1$	• $\boldsymbol{E}$ ∥ [010] and $\boldsymbol{B}$ ∥ [100] and strain ⊥ [010]<br>• $\boldsymbol{E}$ ∥ [010] and $\boldsymbol{B}$ ∥ [001] and strain ⊥ [010]<br>• $\boldsymbol{E}$ ∥ [010] and $\boldsymbol{B}$ ⊥ [010]
$Pnc'2'$ (30.114) $\mathbb{Z}_1$	• $\boldsymbol{E}$ ∥ [010] and $\boldsymbol{B}$ ∥ [001]
P_C2_1 (4.12) $\mathbb{Z}_1$	• $\boldsymbol{E}$ ∥ [010] and strain ⊥ [010]
$P2'/c'$ (13.69) $\mathbb{Z}_2 \times \mathbb{Z}_2 \times \mathbb{Z}_4$	• $\boldsymbol{B}$ ∥ [100] and strain ⊥ [010]<br>• $\boldsymbol{B}$ ∥ [001] and strain ⊥ [010]<br>• $\boldsymbol{B}$ ⊥ [010]
$Pm'a2'$ (28.89) $\mathbb{Z}_1$	• $\boldsymbol{E}$ ∥ [010] and $\boldsymbol{B}$ ∥ [100]
P_Ina2_1 (33.155) $\mathbb{Z}_1$	• $\boldsymbol{E}$ ∥ [010]
$Pb'cm'$ (57.384) $\mathbb{Z}_2$	• $\boldsymbol{B}$ ∥ [100]
$P2$ (3.1) $\mathbb{Z}_2$	• $\boldsymbol{E}$ ∥ [001] and $\boldsymbol{B}$ ∥ [001] and strain ⊥ [001]
$Pb'a'2$ (32.138) $\mathbb{Z}_2$	• $\boldsymbol{E}$ ∥ [001] and $\boldsymbol{B}$ ∥ [001]
P_C2 (3.6) $\mathbb{Z}_1$	• $\boldsymbol{E}$ ∥ [001] and strain ⊥ [001]
$P2/c$ (13.65) $\mathbb{Z}_2 \times \mathbb{Z}_2$	• $\boldsymbol{B}$ ∥ [001] and strain ⊥ [001]
P_Icc2 (27.86) $\mathbb{Z}_2$	• $\boldsymbol{E}$ ∥ [001]
$Pb'a'n$ (50.281) $\mathbb{Z}_2 \times \mathbb{Z}_2$	• $\boldsymbol{B}$ ∥ [001]
$P_S\bar{1}$ (2.7) $\mathbb{Z}_2 \times \mathbb{Z}_2 \times \mathbb{Z}_4$	• strain in generic direction
P_C2_1/c (14.84) $\mathbb{Z}_2$	• strain ⊥ [100]

Continued on next page

...continued

Subgroup & SI group	Perturbation
P_C2_1/c (14.84) $\mathbb{Z}_2$	• strain $\perp$ $[010]$
P_A2/c (13.73) $\mathbb{Z}_2$	• strain $\perp$ $[001]$

Table. S 487: Subgroups of $Pbcm$ (57.377) that can be realized by applying $\boldsymbol{E}/\boldsymbol{B}$ fields and/or uniaxial strains.

Subgroup & SI group	Perturbation
Pm (6.18) $\mathbb{Z}_1$	• $\boldsymbol{E}$ $\parallel$ $[100]$ and strain $\perp$ $[001]$<br>• $\boldsymbol{E}$ $\parallel$ $[100]$ and $\boldsymbol{B}$ $\parallel$ $[001]$<br>• $\boldsymbol{E}$ $\parallel$ $[010]$ and strain $\perp$ $[001]$<br>• $\boldsymbol{E}$ $\parallel$ $[010]$ and $\boldsymbol{B}$ $\parallel$ $[001]$<br>• $\boldsymbol{E}$ $\perp$ $[001]$
Pc (7.24) $\mathbb{Z}_1$	• $\boldsymbol{E}$ $\parallel$ $[100]$ and strain $\perp$ $[010]$<br>• $\boldsymbol{E}$ $\parallel$ $[100]$ and $\boldsymbol{B}$ $\parallel$ $[010]$<br>• $\boldsymbol{E}$ $\parallel$ $[001]$ and strain $\perp$ $[010]$<br>• $\boldsymbol{E}$ $\parallel$ $[001]$ and $\boldsymbol{B}$ $\parallel$ $[010]$<br>• $\boldsymbol{E}$ $\perp$ $[010]$
$P2$ (3.1) $\mathbb{Z}_2$	• $\boldsymbol{E}$ $\parallel$ $[100]$ and strain $\perp$ $[100]$<br>• $\boldsymbol{E}$ $\parallel$ $[100]$ and $\boldsymbol{B}$ $\parallel$ $[100]$
$Pma2$ (28.87) $\mathbb{Z}_1$	• $\boldsymbol{E}$ $\parallel$ $[100]$
Pc (7.24) $\mathbb{Z}_1$	• $\boldsymbol{E}$ $\parallel$ $[010]$ and strain $\perp$ $[100]$<br>• $\boldsymbol{E}$ $\parallel$ $[010]$ and $\boldsymbol{B}$ $\parallel$ $[100]$<br>• $\boldsymbol{E}$ $\parallel$ $[001]$ and strain $\perp$ $[100]$<br>• $\boldsymbol{E}$ $\parallel$ $[001]$ and $\boldsymbol{B}$ $\parallel$ $[100]$<br>• $\boldsymbol{E}$ $\perp$ $[100]$
$P2_1$ (4.7) $\mathbb{Z}_1$	• $\boldsymbol{E}$ $\parallel$ $[010]$ and strain $\perp$ $[010]$<br>• $\boldsymbol{E}$ $\parallel$ $[010]$ and $\boldsymbol{B}$ $\parallel$ $[010]$
$Pmc2_1$ (26.66) $\mathbb{Z}_1$	• $\boldsymbol{E}$ $\parallel$ $[010]$
$P2_1$ (4.7) $\mathbb{Z}_1$	• $\boldsymbol{E}$ $\parallel$ $[001]$ and strain $\perp$ $[001]$<br>• $\boldsymbol{E}$ $\parallel$ $[001]$ and $\boldsymbol{B}$ $\parallel$ $[001]$
$Pca2_1$ (29.99) $\mathbb{Z}_1$	• $\boldsymbol{E}$ $\parallel$ $[001]$

Continued on next page

…continued

Subgroup & SI group	Perturbation
$P\bar{1}$ (2.4) $\mathbb{Z}_2 \times \mathbb{Z}_2 \times \mathbb{Z}_2 \times \mathbb{Z}_4$	• strain in generic direction<br>• $\boldsymbol{B} \parallel [100]$ and strain $\perp [010]$<br>• $\boldsymbol{B} \parallel [100]$ and strain $\perp [001]$<br>• $\boldsymbol{B} \parallel [010]$ and strain $\perp [100]$<br>• $\boldsymbol{B} \parallel [010]$ and strain $\perp [001]$<br>• $\boldsymbol{B} \parallel [001]$ and strain $\perp [100]$<br>• $\boldsymbol{B} \parallel [001]$ and strain $\perp [010]$<br>• $\boldsymbol{B}$ in generic direction
$P2/c$ (13.65) $\mathbb{Z}_2 \times \mathbb{Z}_2$	• strain $\perp [100]$<br>• $\boldsymbol{B} \parallel [100]$
$P2_1/c$ (14.75) $\mathbb{Z}_2$	• strain $\perp [010]$<br>• $\boldsymbol{B} \parallel [010]$
$P2_1/m$ (11.50) $\mathbb{Z}_2$	• strain $\perp [001]$<br>• $\boldsymbol{B} \parallel [001]$

Table. S 488: Subgroups of $Pbcm1'$ (57.378) that can be realized by applying $\boldsymbol{E}/\boldsymbol{B}$ fields and/or uniaxial strains.

Subgroup & SI group	Perturbation
$P11'$ (1.2) $\mathbb{Z}_1$	• $\boldsymbol{E} \parallel [100]$ and strain in generic direction<br>• $\boldsymbol{E} \parallel [010]$ and strain in generic direction<br>• $\boldsymbol{E} \parallel [001]$ and strain in generic direction<br>• $\boldsymbol{E} \perp [100]$ and strain $\perp [010]$<br>• $\boldsymbol{E} \perp [100]$ and strain $\perp [001]$<br>• $\boldsymbol{E} \perp [100]$ and strain in generic direction<br>• $\boldsymbol{E} \perp [010]$ and strain $\perp [100]$<br>• $\boldsymbol{E} \perp [010]$ and strain $\perp [001]$<br>• $\boldsymbol{E} \perp [010]$ and strain in generic direction<br>• $\boldsymbol{E} \perp [001]$ and strain $\perp [100]$<br>• $\boldsymbol{E} \perp [001]$ and strain $\perp [010]$<br>• $\boldsymbol{E} \perp [001]$ and strain in generic direction<br>• $\boldsymbol{E}$ in generic direction

Continued on next page

. . . continued

Subgroup & SI group	Perturbation
Pm' (6.20) $\mathbb{Z}_1$	• $\boldsymbol{E}$ ∥ [100] and $\boldsymbol{B}$ ∥ [100] and strain ⊥ [001]<br>• $\boldsymbol{E}$ ∥ [100] and $\boldsymbol{B}$ ∥ [010] and strain ⊥ [001]<br>• $\boldsymbol{E}$ ∥ [100] and $\boldsymbol{B}$ ⊥ [001]<br>• $\boldsymbol{E}$ ∥ [010] and $\boldsymbol{B}$ ∥ [100] and strain ⊥ [001]<br>• $\boldsymbol{E}$ ∥ [010] and $\boldsymbol{B}$ ∥ [010] and strain ⊥ [001]<br>• $\boldsymbol{E}$ ∥ [010] and $\boldsymbol{B}$ ⊥ [001]<br>• $\boldsymbol{E}$ ⊥ [001] and $\boldsymbol{B}$ ∥ [100]<br>• $\boldsymbol{E}$ ⊥ [001] and $\boldsymbol{B}$ ∥ [010]<br>• $\boldsymbol{E}$ ⊥ [001] and $\boldsymbol{B}$ ⊥ [001]
Pm (6.18) $\mathbb{Z}_1$	• $\boldsymbol{E}$ ∥ [100] and $\boldsymbol{B}$ ∥ [001] and strain ⊥ [001]<br>• $\boldsymbol{E}$ ∥ [010] and $\boldsymbol{B}$ ∥ [001] and strain ⊥ [001]<br>• $\boldsymbol{E}$ ⊥ [001] and $\boldsymbol{B}$ ∥ [001]
$Pm1'$ (6.19) $\mathbb{Z}_1$	• $\boldsymbol{E}$ ∥ [100] and strain ⊥ [001]<br>• $\boldsymbol{E}$ ∥ [010] and strain ⊥ [001]<br>• $\boldsymbol{E}$ ⊥ [001]
Pc' (7.26) $\mathbb{Z}_1$	• $\boldsymbol{E}$ ∥ [100] and $\boldsymbol{B}$ ∥ [100] and strain ⊥ [010]<br>• $\boldsymbol{E}$ ∥ [100] and $\boldsymbol{B}$ ∥ [001] and strain ⊥ [010]<br>• $\boldsymbol{E}$ ∥ [100] and $\boldsymbol{B}$ ⊥ [010]<br>• $\boldsymbol{E}$ ∥ [001] and $\boldsymbol{B}$ ∥ [100] and strain ⊥ [010]<br>• $\boldsymbol{E}$ ∥ [001] and $\boldsymbol{B}$ ∥ [001] and strain ⊥ [010]<br>• $\boldsymbol{E}$ ∥ [001] and $\boldsymbol{B}$ ⊥ [010]<br>• $\boldsymbol{E}$ ⊥ [010] and $\boldsymbol{B}$ ∥ [100]<br>• $\boldsymbol{E}$ ⊥ [010] and $\boldsymbol{B}$ ∥ [001]<br>• $\boldsymbol{E}$ ⊥ [010] and $\boldsymbol{B}$ ⊥ [010]
Pc (7.24) $\mathbb{Z}_1$	• $\boldsymbol{E}$ ∥ [100] and $\boldsymbol{B}$ ∥ [010] and strain ⊥ [010]<br>• $\boldsymbol{E}$ ∥ [001] and $\boldsymbol{B}$ ∥ [010] and strain ⊥ [010]<br>• $\boldsymbol{E}$ ⊥ [010] and $\boldsymbol{B}$ ∥ [010]
$Pc1'$ (7.25) $\mathbb{Z}_1$	• $\boldsymbol{E}$ ∥ [100] and strain ⊥ [010]<br>• $\boldsymbol{E}$ ∥ [001] and strain ⊥ [010]<br>• $\boldsymbol{E}$ ⊥ [010]
$P2'$ (3.3) $\mathbb{Z}_1$	• $\boldsymbol{E}$ ∥ [100] and $\boldsymbol{B}$ ∥ [010] and strain ⊥ [100]<br>• $\boldsymbol{E}$ ∥ [100] and $\boldsymbol{B}$ ∥ [001] and strain ⊥ [100]<br>• $\boldsymbol{E}$ ∥ [100] and $\boldsymbol{B}$ ⊥ [100]
$Pma'2'$ (28.90) $\mathbb{Z}_1$	• $\boldsymbol{E}$ ∥ [100] and $\boldsymbol{B}$ ∥ [001]
$Pm'a2'$ (28.89) $\mathbb{Z}_1$	• $\boldsymbol{E}$ ∥ [100] and $\boldsymbol{B}$ ∥ [010]
$P2$ (3.1) $\mathbb{Z}_2$	• $\boldsymbol{E}$ ∥ [100] and $\boldsymbol{B}$ ∥ [100] and strain ⊥ [100]

Continued on next page

...continued

Subgroup & SI group	Perturbation
$Pm'a'2$ (28.91) $\mathbb{Z}_2$	• $\boldsymbol{E}$ $\parallel$ $[100]$ and $\boldsymbol{B}$ $\parallel$ $[100]$
$P21'$ (3.2) $\mathbb{Z}_2$	• $\boldsymbol{E}$ $\parallel$ $[100]$ and strain $\perp$ $[100]$
$Pma21'$ (28.88) $\mathbb{Z}_1$	• $\boldsymbol{E}$ $\parallel$ $[100]$
Pc' (7.26) $\mathbb{Z}_1$	• $\boldsymbol{E}$ $\parallel$ $[010]$ and $\boldsymbol{B}$ $\parallel$ $[010]$ and strain $\perp$ $[100]$<br>• $\boldsymbol{E}$ $\parallel$ $[010]$ and $\boldsymbol{B}$ $\parallel$ $[001]$ and strain $\perp$ $[100]$<br>• $\boldsymbol{E}$ $\parallel$ $[010]$ and $\boldsymbol{B}$ $\perp$ $[100]$<br>• $\boldsymbol{E}$ $\parallel$ $[001]$ and $\boldsymbol{B}$ $\parallel$ $[010]$ and strain $\perp$ $[100]$<br>• $\boldsymbol{E}$ $\parallel$ $[001]$ and $\boldsymbol{B}$ $\parallel$ $[001]$ and strain $\perp$ $[100]$<br>• $\boldsymbol{E}$ $\parallel$ $[001]$ and $\boldsymbol{B}$ $\perp$ $[100]$<br>• $\boldsymbol{E}$ $\perp$ $[100]$ and $\boldsymbol{B}$ $\parallel$ $[010]$<br>• $\boldsymbol{E}$ $\perp$ $[100]$ and $\boldsymbol{B}$ $\parallel$ $[001]$<br>• $\boldsymbol{E}$ $\perp$ $[100]$ and $\boldsymbol{B}$ $\perp$ $[100]$
Pc (7.24) $\mathbb{Z}_1$	• $\boldsymbol{E}$ $\parallel$ $[010]$ and $\boldsymbol{B}$ $\parallel$ $[100]$ and strain $\perp$ $[100]$<br>• $\boldsymbol{E}$ $\parallel$ $[001]$ and $\boldsymbol{B}$ $\parallel$ $[100]$ and strain $\perp$ $[100]$<br>• $\boldsymbol{E}$ $\perp$ $[100]$ and $\boldsymbol{B}$ $\parallel$ $[100]$
$Pc1'$ (7.25) $\mathbb{Z}_1$	• $\boldsymbol{E}$ $\parallel$ $[010]$ and strain $\perp$ $[100]$<br>• $\boldsymbol{E}$ $\parallel$ $[001]$ and strain $\perp$ $[100]$<br>• $\boldsymbol{E}$ $\perp$ $[100]$
$P2_1'$ (4.9) $\mathbb{Z}_1$	• $\boldsymbol{E}$ $\parallel$ $[010]$ and $\boldsymbol{B}$ $\parallel$ $[100]$ and strain $\perp$ $[010]$<br>• $\boldsymbol{E}$ $\parallel$ $[010]$ and $\boldsymbol{B}$ $\parallel$ $[001]$ and strain $\perp$ $[010]$<br>• $\boldsymbol{E}$ $\parallel$ $[010]$ and $\boldsymbol{B}$ $\perp$ $[010]$
$Pmc'2_1'$ (26.69) $\mathbb{Z}_1$	• $\boldsymbol{E}$ $\parallel$ $[010]$ and $\boldsymbol{B}$ $\parallel$ $[001]$
$Pm'c2_1'$ (26.68) $\mathbb{Z}_1$	• $\boldsymbol{E}$ $\parallel$ $[010]$ and $\boldsymbol{B}$ $\parallel$ $[100]$
$P2_1$ (4.7) $\mathbb{Z}_1$	• $\boldsymbol{E}$ $\parallel$ $[010]$ and $\boldsymbol{B}$ $\parallel$ $[010]$ and strain $\perp$ $[010]$
$Pm'c'2_1$ (26.70) $\mathbb{Z}_1$	• $\boldsymbol{E}$ $\parallel$ $[010]$ and $\boldsymbol{B}$ $\parallel$ $[010]$
$P2_11'$ (4.8) $\mathbb{Z}_1$	• $\boldsymbol{E}$ $\parallel$ $[010]$ and strain $\perp$ $[010]$
$Pmc2_11'$ (26.67) $\mathbb{Z}_1$	• $\boldsymbol{E}$ $\parallel$ $[010]$
$P2_1'$ (4.9) $\mathbb{Z}_1$	• $\boldsymbol{E}$ $\parallel$ $[001]$ and $\boldsymbol{B}$ $\parallel$ $[100]$ and strain $\perp$ $[001]$<br>• $\boldsymbol{E}$ $\parallel$ $[001]$ and $\boldsymbol{B}$ $\parallel$ $[010]$ and strain $\perp$ $[001]$<br>• $\boldsymbol{E}$ $\parallel$ $[001]$ and $\boldsymbol{B}$ $\perp$ $[001]$
$Pca'2_1'$ (29.102) $\mathbb{Z}_1$	• $\boldsymbol{E}$ $\parallel$ $[001]$ and $\boldsymbol{B}$ $\parallel$ $[010]$
$Pc'a2_1'$ (29.101) $\mathbb{Z}_1$	• $\boldsymbol{E}$ $\parallel$ $[001]$ and $\boldsymbol{B}$ $\parallel$ $[100]$
$P2_1$ (4.7) $\mathbb{Z}_1$	• $\boldsymbol{E}$ $\parallel$ $[001]$ and $\boldsymbol{B}$ $\parallel$ $[001]$ and strain $\perp$ $[001]$

Continued on next page

...continued

Subgroup & SI group	Perturbation
$Pc'a'2_1$ (29.103) $\mathbb{Z}_1$	• $\boldsymbol{E}$ ∥ [001] and $\boldsymbol{B}$ ∥ [001]
$P2_11'$ (4.8) $\mathbb{Z}_1$	• $\boldsymbol{E}$ ∥ [001] and strain ⊥ [001]
$Pca2_11'$ (29.100) $\mathbb{Z}_1$	• $\boldsymbol{E}$ ∥ [001]
$P\bar{1}$ (2.4) $\mathbb{Z}_2 \times \mathbb{Z}_2 \times \mathbb{Z}_2 \times \mathbb{Z}_4$	• $\boldsymbol{B}$ ∥ [100] and strain in generic direction<br>• $\boldsymbol{B}$ ∥ [010] and strain in generic direction<br>• $\boldsymbol{B}$ ∥ [001] and strain in generic direction<br>• $\boldsymbol{B}$ ⊥ [100] and strain ⊥ [010]<br>• $\boldsymbol{B}$ ⊥ [100] and strain ⊥ [001]<br>• $\boldsymbol{B}$ ⊥ [100] and strain in generic direction<br>• $\boldsymbol{B}$ ⊥ [010] and strain ⊥ [100]<br>• $\boldsymbol{B}$ ⊥ [010] and strain ⊥ [001]<br>• $\boldsymbol{B}$ ⊥ [010] and strain in generic direction<br>• $\boldsymbol{B}$ ⊥ [001] and strain ⊥ [100]<br>• $\boldsymbol{B}$ ⊥ [001] and strain ⊥ [010]<br>• $\boldsymbol{B}$ ⊥ [001] and strain in generic direction<br>• $\boldsymbol{B}$ in generic direction
$P2'/c'$ (13.69) $\mathbb{Z}_2 \times \mathbb{Z}_2 \times \mathbb{Z}_4$	• $\boldsymbol{B}$ ∥ [010] and strain ⊥ [100]<br>• $\boldsymbol{B}$ ∥ [001] and strain ⊥ [100]<br>• $\boldsymbol{B}$ ⊥ [100]
$P2/c$ (13.65) $\mathbb{Z}_2 \times \mathbb{Z}_2$	• $\boldsymbol{B}$ ∥ [100] and strain ⊥ [100]
$P2_1'/c'$ (14.79) $\mathbb{Z}_2 \times \mathbb{Z}_4$	• $\boldsymbol{B}$ ∥ [100] and strain ⊥ [010]<br>• $\boldsymbol{B}$ ∥ [001] and strain ⊥ [010]<br>• $\boldsymbol{B}$ ⊥ [010]
$P2_1/c$ (14.75) $\mathbb{Z}_2$	• $\boldsymbol{B}$ ∥ [010] and strain ⊥ [010]
$P2_1'/m'$ (11.54) $\mathbb{Z}_2 \times \mathbb{Z}_2 \times \mathbb{Z}_4$	• $\boldsymbol{B}$ ∥ [100] and strain ⊥ [001]<br>• $\boldsymbol{B}$ ∥ [010] and strain ⊥ [001]<br>• $\boldsymbol{B}$ ⊥ [001]
$Pbc'm'$ (57.383) $\mathbb{Z}_2 \times \mathbb{Z}_2$	• $\boldsymbol{B}$ ∥ [100]
$Pb'cm'$ (57.384) $\mathbb{Z}_2$	• $\boldsymbol{B}$ ∥ [010]
$P2_1/m$ (11.50) $\mathbb{Z}_2$	• $\boldsymbol{B}$ ∥ [001] and strain ⊥ [001]
$Pb'c'm$ (57.382) $\mathbb{Z}_2$	• $\boldsymbol{B}$ ∥ [001]
$P\bar{1}1'$ (2.5) $\mathbb{Z}_2 \times \mathbb{Z}_2 \times \mathbb{Z}_2 \times \mathbb{Z}_4$	• strain in generic direction
$P2/c1'$ (13.66) $\mathbb{Z}_2 \times \mathbb{Z}_2$	• strain ⊥ [100]
$P2_1/c1'$ (14.76) $\mathbb{Z}_2$	• strain ⊥ [010]

Continued on next page

. . . continued

Subgroup & SI group	Perturbation
$P2_1/m1'$ (11.51) $\mathbb{Z}_2$	• strain ⊥ [001]

Table. S 489: Subgroups of $Pb'cm$ (57.379) that can be realized by applying $\boldsymbol{E}/\boldsymbol{B}$ fields and/or uniaxial strains.

Subgroup & SI group	Perturbation
Pm (6.18) $\mathbb{Z}_1$	• $\boldsymbol{B}$ ∥ [001] and strain ⊥ [001]<br>• $\boldsymbol{E}$ ∥ [100] and strain ⊥ [001]<br>• $\boldsymbol{E}$ ∥ [100] and $\boldsymbol{B}$ ∥ [001]<br>• $\boldsymbol{E}$ ∥ [010] and strain ⊥ [001]<br>• $\boldsymbol{E}$ ⊥ [001]
Pc (7.24) $\mathbb{Z}_1$	• $\boldsymbol{B}$ ∥ [010] and strain ⊥ [010]<br>• $\boldsymbol{E}$ ∥ [100] and strain ⊥ [010]<br>• $\boldsymbol{E}$ ∥ [100] and $\boldsymbol{B}$ ∥ [010]<br>• $\boldsymbol{E}$ ∥ [001] and strain ⊥ [010]<br>• $\boldsymbol{E}$ ⊥ [010]
$P2$ (3.1) $\mathbb{Z}_2$	• $\boldsymbol{B}$ ∥ [100] and strain ⊥ [100]<br>• $\boldsymbol{E}$ ∥ [100] and strain ⊥ [100]<br>• $\boldsymbol{E}$ ∥ [100] and $\boldsymbol{B}$ ∥ [100]
$Pma2$ (28.87) $\mathbb{Z}_1$	• $\boldsymbol{E}$ ∥ [100]
Pc' (7.26) $\mathbb{Z}_1$	• $\boldsymbol{B}$ ∥ [010] and strain ⊥ [100]<br>• $\boldsymbol{B}$ ∥ [001] and strain ⊥ [100]<br>• $\boldsymbol{B}$ ⊥ [100]<br>• $\boldsymbol{E}$ ∥ [010] and strain ⊥ [100]<br>• $\boldsymbol{E}$ ∥ [010] and $\boldsymbol{B}$ ∥ [010]<br>• $\boldsymbol{E}$ ∥ [001] and strain ⊥ [100]<br>• $\boldsymbol{E}$ ∥ [001] and $\boldsymbol{B}$ ∥ [001]<br>• $\boldsymbol{E}$ ⊥ [100]
$P2_1'$ (4.9) $\mathbb{Z}_1$	• $\boldsymbol{B}$ ∥ [100] and strain ⊥ [010]<br>• $\boldsymbol{B}$ ∥ [001] and strain ⊥ [010]<br>• $\boldsymbol{B}$ ⊥ [010]<br>• $\boldsymbol{E}$ ∥ [010] and strain ⊥ [010]<br>• $\boldsymbol{E}$ ∥ [010] and $\boldsymbol{B}$ ∥ [100]
$Pmc'2_1'$ (26.69) $\mathbb{Z}_1$	• $\boldsymbol{B}$ ∥ [001]<br>• $\boldsymbol{E}$ ∥ [010]

Continued on next page

. . . continued

Subgroup & SI group	Perturbation
$P2_1'$ (4.9) $\mathbb{Z}_1$	• $\boldsymbol{B} \parallel [100]$ and strain $\perp [001]$<br>• $\boldsymbol{B} \parallel [010]$ and strain $\perp [001]$<br>• $\boldsymbol{B} \perp [001]$<br>• $\boldsymbol{E} \parallel [001]$ and strain $\perp [001]$<br>• $\boldsymbol{E} \parallel [001]$ and $\boldsymbol{B} \parallel [100]$
$Pca'2_1'$ (29.102) $\mathbb{Z}_1$	• $\boldsymbol{B} \parallel [010]$<br>• $\boldsymbol{E} \parallel [001]$
$P2_1'2_1'2$ (18.18) $\mathbb{Z}_2$	• $\boldsymbol{B} \parallel [100]$
$P\bar{1}'$ (2.6) $\mathbb{Z}_1$	• strain in generic direction
$P2/c'$ (13.68) $\mathbb{Z}_2$	• strain $\perp [100]$
$P2_1'/c$ (14.77) $\mathbb{Z}_1$	• strain $\perp [010]$
$P2_1'/m$ (11.52) $\mathbb{Z}_1$	• strain $\perp [001]$

Table. S 490: Subgroups of $Pbc'm$ (57.380) that can be realized by applying $\boldsymbol{E}/\boldsymbol{B}$ fields and/or uniaxial strains.

Subgroup & SI group	Perturbation
Pm (6.18) $\mathbb{Z}_1$	• $\boldsymbol{B} \parallel [001]$ and strain $\perp [001]$<br>• $\boldsymbol{E} \parallel [100]$ and strain $\perp [001]$<br>• $\boldsymbol{E} \parallel [010]$ and strain $\perp [001]$<br>• $\boldsymbol{E} \parallel [010]$ and $\boldsymbol{B} \parallel [001]$<br>• $\boldsymbol{E} \perp [001]$
Pc' (7.26) $\mathbb{Z}_1$	• $\boldsymbol{B} \parallel [100]$ and strain $\perp [010]$<br>• $\boldsymbol{B} \parallel [001]$ and strain $\perp [010]$<br>• $\boldsymbol{B} \perp [010]$<br>• $\boldsymbol{E} \parallel [100]$ and strain $\perp [010]$<br>• $\boldsymbol{E} \parallel [100]$ and $\boldsymbol{B} \parallel [100]$<br>• $\boldsymbol{E} \parallel [001]$ and strain $\perp [010]$<br>• $\boldsymbol{E} \parallel [001]$ and $\boldsymbol{B} \parallel [001]$<br>• $\boldsymbol{E} \perp [010]$

Continued on next page

…continued

Subgroup & SI group	Perturbation
$P2'$ (3.3) $\mathbb{Z}_1$	• $\boldsymbol{B} \parallel [010]$ and strain $\perp [100]$<br>• $\boldsymbol{B} \parallel [001]$ and strain $\perp [100]$<br>• $\boldsymbol{B} \perp [100]$<br>• $\boldsymbol{E} \parallel [100]$ and strain $\perp [100]$<br>• $\boldsymbol{E} \parallel [100]$ and $\boldsymbol{B} \parallel [010]$
$Pma'2'$ (28.90) $\mathbb{Z}_1$	• $\boldsymbol{B} \parallel [001]$<br>• $\boldsymbol{E} \parallel [100]$
Pc (7.24) $\mathbb{Z}_1$	• $\boldsymbol{B} \parallel [100]$ and strain $\perp [100]$<br>• $\boldsymbol{E} \parallel [010]$ and strain $\perp [100]$<br>• $\boldsymbol{E} \parallel [010]$ and $\boldsymbol{B} \parallel [100]$<br>• $\boldsymbol{E} \parallel [001]$ and strain $\perp [100]$<br>• $\boldsymbol{E} \perp [100]$
$P2_1$ (4.7) $\mathbb{Z}_1$	• $\boldsymbol{B} \parallel [010]$ and strain $\perp [010]$<br>• $\boldsymbol{E} \parallel [010]$ and strain $\perp [010]$<br>• $\boldsymbol{E} \parallel [010]$ and $\boldsymbol{B} \parallel [010]$
$Pmc2_1$ (26.66) $\mathbb{Z}_1$	• $\boldsymbol{E} \parallel [010]$
$P2_1'$ (4.9) $\mathbb{Z}_1$	• $\boldsymbol{B} \parallel [100]$ and strain $\perp [001]$<br>• $\boldsymbol{B} \parallel [010]$ and strain $\perp [001]$<br>• $\boldsymbol{B} \perp [001]$<br>• $\boldsymbol{E} \parallel [001]$ and strain $\perp [001]$<br>• $\boldsymbol{E} \parallel [001]$ and $\boldsymbol{B} \parallel [010]$
$Pc'a2_1'$ (29.101) $\mathbb{Z}_1$	• $\boldsymbol{B} \parallel [100]$<br>• $\boldsymbol{E} \parallel [001]$
$P2_12_1'2'$ (18.19) $\mathbb{Z}_1$	• $\boldsymbol{B} \parallel [010]$
$P\bar{1}'$ (2.6) $\mathbb{Z}_1$	• strain in generic direction
$P2'/c$ (13.67) $\mathbb{Z}_1$	• strain $\perp [100]$
$P2_1/c'$ (14.78) $\mathbb{Z}_1$	• strain $\perp [010]$
$P2_1'/m$ (11.52) $\mathbb{Z}_1$	• strain $\perp [001]$

Table. S 491: Subgroups of $Pbcm'$ (57.381) that can be realized by applying $\boldsymbol{E}/\boldsymbol{B}$ fields and/or uniaxial strains.

Subgroup & SI group	Perturbation
Pm' (6.20) $\mathbb{Z}_1$	• $\boldsymbol{B} \parallel [100]$ and strain $\perp [001]$<br>• $\boldsymbol{B} \parallel [010]$ and strain $\perp [001]$<br>• $\boldsymbol{B} \perp [001]$<br>• $\boldsymbol{E} \parallel [100]$ and strain $\perp [001]$<br>• $\boldsymbol{E} \parallel [100]$ and $\boldsymbol{B} \parallel [100]$<br>• $\boldsymbol{E} \parallel [010]$ and strain $\perp [001]$<br>• $\boldsymbol{E} \parallel [010]$ and $\boldsymbol{B} \parallel [010]$<br>• $\boldsymbol{E} \perp [001]$
Pc (7.24) $\mathbb{Z}_1$	• $\boldsymbol{B} \parallel [010]$ and strain $\perp [010]$<br>• $\boldsymbol{E} \parallel [100]$ and strain $\perp [010]$<br>• $\boldsymbol{E} \parallel [001]$ and strain $\perp [010]$<br>• $\boldsymbol{E} \parallel [001]$ and $\boldsymbol{B} \parallel [010]$<br>• $\boldsymbol{E} \perp [010]$
$P2'$ (3.3) $\mathbb{Z}_1$	• $\boldsymbol{B} \parallel [010]$ and strain $\perp [100]$<br>• $\boldsymbol{B} \parallel [001]$ and strain $\perp [100]$<br>• $\boldsymbol{B} \perp [100]$<br>• $\boldsymbol{E} \parallel [100]$ and strain $\perp [100]$<br>• $\boldsymbol{E} \parallel [100]$ and $\boldsymbol{B} \parallel [001]$
$Pm'a2'$ (28.89) $\mathbb{Z}_1$	• $\boldsymbol{B} \parallel [010]$<br>• $\boldsymbol{E} \parallel [100]$
Pc (7.24) $\mathbb{Z}_1$	• $\boldsymbol{B} \parallel [100]$ and strain $\perp [100]$<br>• $\boldsymbol{E} \parallel [010]$ and strain $\perp [100]$<br>• $\boldsymbol{E} \parallel [001]$ and strain $\perp [100]$<br>• $\boldsymbol{E} \parallel [001]$ and $\boldsymbol{B} \parallel [100]$<br>• $\boldsymbol{E} \perp [100]$
$P2_1'$ (4.9) $\mathbb{Z}_1$	• $\boldsymbol{B} \parallel [100]$ and strain $\perp [010]$<br>• $\boldsymbol{B} \parallel [001]$ and strain $\perp [010]$<br>• $\boldsymbol{B} \perp [010]$<br>• $\boldsymbol{E} \parallel [010]$ and strain $\perp [010]$<br>• $\boldsymbol{E} \parallel [010]$ and $\boldsymbol{B} \parallel [001]$
$Pm'c2_1'$ (26.68) $\mathbb{Z}_1$	• $\boldsymbol{B} \parallel [100]$<br>• $\boldsymbol{E} \parallel [010]$
$P2_1$ (4.7) $\mathbb{Z}_1$	• $\boldsymbol{B} \parallel [001]$ and strain $\perp [001]$<br>• $\boldsymbol{E} \parallel [001]$ and strain $\perp [001]$<br>• $\boldsymbol{E} \parallel [001]$ and $\boldsymbol{B} \parallel [001]$
$Pca2_1$ (29.99) $\mathbb{Z}_1$	• $\boldsymbol{E} \parallel [001]$

Continued on next page

. . . continued

Subgroup & SI group	Perturbation
$P2_12_1'2'$ (18.19) $\mathbb{Z}_1$	• $\boldsymbol{B} \parallel [001]$
$P\bar{1}'$ (2.6) $\mathbb{Z}_1$	• strain in generic direction
$P2'/c$ (13.67) $\mathbb{Z}_1$	• strain $\perp$ [100]
$P2_1'/c$ (14.77) $\mathbb{Z}_1$	• strain $\perp$ [010]
$P2_1/m'$ (11.53) $\mathbb{Z}_1$	• strain $\perp$ [001]

Table. S 492: Subgroups of $Pb'c'm$ (57.382) that can be realized by applying $\boldsymbol{E}/\boldsymbol{B}$ fields and/or uniaxial strains.

Subgroup & SI group	Perturbation
Pm (6.18) $\mathbb{Z}_1$	• $\boldsymbol{E} \parallel [100]$ and strain $\perp$ [001]<br>• $\boldsymbol{E} \parallel [010]$ and strain $\perp$ [001]<br>• $\boldsymbol{E} \perp [001]$
Pc' (7.26) $\mathbb{Z}_1$	• $\boldsymbol{E} \parallel [100]$ and strain $\perp$ [010]<br>• $\boldsymbol{E} \parallel [100]$ and ($\boldsymbol{B} \parallel [100]$ or $\boldsymbol{B} \perp [010]$)<br>• $\boldsymbol{E} \parallel [001]$ and strain $\perp$ [010]<br>• $\boldsymbol{E} \parallel [001]$ and ($\boldsymbol{B} \parallel [100]$ or $\boldsymbol{B} \perp [010]$)<br>• $\boldsymbol{E} \perp [010]$
$P2'$ (3.3) $\mathbb{Z}_1$	• $\boldsymbol{E} \parallel [100]$ and strain $\perp$ [100]<br>• $\boldsymbol{E} \parallel [100]$ and ($\boldsymbol{B} \parallel [010]$ or $\boldsymbol{B} \perp [100]$)
$Pma'2'$ (28.90) $\mathbb{Z}_1$	• $\boldsymbol{E} \parallel [100]$
Pc' (7.26) $\mathbb{Z}_1$	• $\boldsymbol{E} \parallel [010]$ and strain $\perp$ [100]<br>• $\boldsymbol{E} \parallel [010]$ and ($\boldsymbol{B} \parallel [010]$ or $\boldsymbol{B} \perp [100]$)<br>• $\boldsymbol{E} \parallel [001]$ and strain $\perp$ [100]<br>• $\boldsymbol{E} \parallel [001]$ and ($\boldsymbol{B} \parallel [010]$ or $\boldsymbol{B} \perp [100]$)<br>• $\boldsymbol{E} \perp [100]$
$P2_1'$ (4.9) $\mathbb{Z}_1$	• $\boldsymbol{E} \parallel [010]$ and strain $\perp$ [010]<br>• $\boldsymbol{E} \parallel [010]$ and ($\boldsymbol{B} \parallel [100]$ or $\boldsymbol{B} \perp [010]$)
$Pmc'2_1'$ (26.69) $\mathbb{Z}_1$	• $\boldsymbol{E} \parallel [010]$
$P2_1$ (4.7) $\mathbb{Z}_1$	• $\boldsymbol{E} \parallel [001]$ and strain $\perp$ [001]
$Pc'a'2_1$ (29.103) $\mathbb{Z}_1$	• $\boldsymbol{E} \parallel [001]$

Continued on next page

...continued

Subgroup & SI group	Perturbation
$P\bar{1}$ (2.4) $\mathbb{Z}_2 \times \mathbb{Z}_2 \times \mathbb{Z}_2 \times \mathbb{Z}_4$	• strain in generic direction<br>• ($\boldsymbol{B} \parallel$ [100] or $\boldsymbol{B} \perp$ [010]) and strain $\perp$ [100]<br>• ($\boldsymbol{B} \parallel$ [100] or $\boldsymbol{B} \perp$ [010]) and strain $\perp$ [001]<br>• ($\boldsymbol{B} \parallel$ [010] or $\boldsymbol{B} \perp$ [100]) and strain $\perp$ [010]<br>• ($\boldsymbol{B} \parallel$ [010] or $\boldsymbol{B} \perp$ [100]) and strain $\perp$ [001]<br>• $\boldsymbol{B}$ in generic direction
$P2'/c'$ (13.69) $\mathbb{Z}_2 \times \mathbb{Z}_2 \times \mathbb{Z}_4$	• strain $\perp$ [100]<br>• ($\boldsymbol{B} \parallel$ [010] or $\boldsymbol{B} \perp$ [100])
$P2_1'/c'$ (14.79) $\mathbb{Z}_2 \times \mathbb{Z}_4$	• strain $\perp$ [010]<br>• ($\boldsymbol{B} \parallel$ [100] or $\boldsymbol{B} \perp$ [010])
$P2_1/m$ (11.50) $\mathbb{Z}_2$	• strain $\perp$ [001]

Table. S 493: Subgroups of $Pbc'm'$ (57.383) that can be realized by applying $\boldsymbol{E}/\boldsymbol{B}$ fields and/or uniaxial strains.

Subgroup & SI group	Perturbation
Pm' (6.20) $\mathbb{Z}_1$	• $\boldsymbol{E} \parallel$ [100] and strain $\perp$ [001]<br>• $\boldsymbol{E} \parallel$ [100] and ($\boldsymbol{B} \parallel$ [010] or $\boldsymbol{B} \perp$ [001])<br>• $\boldsymbol{E} \parallel$ [010] and strain $\perp$ [001]<br>• $\boldsymbol{E} \parallel$ [010] and ($\boldsymbol{B} \parallel$ [010] or $\boldsymbol{B} \perp$ [001])<br>• $\boldsymbol{E} \perp$ [001]
Pc' (7.26) $\mathbb{Z}_1$	• $\boldsymbol{E} \parallel$ [100] and strain $\perp$ [010]<br>• $\boldsymbol{E} \parallel$ [100] and ($\boldsymbol{B} \parallel$ [001] or $\boldsymbol{B} \perp$ [010])<br>• $\boldsymbol{E} \parallel$ [001] and strain $\perp$ [010]<br>• $\boldsymbol{E} \parallel$ [001] and ($\boldsymbol{B} \parallel$ [001] or $\boldsymbol{B} \perp$ [010])<br>• $\boldsymbol{E} \perp$ [010]
$P2$ (3.1) $\mathbb{Z}_2$	• $\boldsymbol{E} \parallel$ [100] and strain $\perp$ [100]
$Pm'a'2$ (28.91) $\mathbb{Z}_2$	• $\boldsymbol{E} \parallel$ [100]
Pc (7.24) $\mathbb{Z}_1$	• $\boldsymbol{E} \parallel$ [010] and strain $\perp$ [100]<br>• $\boldsymbol{E} \parallel$ [001] and strain $\perp$ [100]<br>• $\boldsymbol{E} \perp$ [100]
$P2_1'$ (4.9) $\mathbb{Z}_1$	• $\boldsymbol{E} \parallel$ [010] and strain $\perp$ [010]<br>• $\boldsymbol{E} \parallel$ [010] and ($\boldsymbol{B} \parallel$ [001] or $\boldsymbol{B} \perp$ [010])
$Pm'c2_1'$ (26.68) $\mathbb{Z}_1$	• $\boldsymbol{E} \parallel$ [010]

Continued on next page

...continued

Subgroup & SI group	Perturbation
$P2_1'$ (4.9) $\mathbb{Z}_1$	• $\boldsymbol{E} \parallel [001]$ and strain $\perp [001]$<br>• $\boldsymbol{E} \parallel [001]$ and ($\boldsymbol{B} \parallel [010]$ or $\boldsymbol{B} \perp [001]$)
$Pc'a2_1'$ (29.101) $\mathbb{Z}_1$	• $\boldsymbol{E} \parallel [001]$
$P\bar{1}$ (2.4) $\mathbb{Z}_2 \times \mathbb{Z}_2 \times \mathbb{Z}_2 \times \mathbb{Z}_4$	• strain in generic direction<br>• ($\boldsymbol{B} \parallel [010]$ or $\boldsymbol{B} \perp [001]$) and strain $\perp [100]$<br>• ($\boldsymbol{B} \parallel [010]$ or $\boldsymbol{B} \perp [001]$) and strain $\perp [010]$<br>• ($\boldsymbol{B} \parallel [001]$ or $\boldsymbol{B} \perp [010]$) and strain $\perp [100]$<br>• ($\boldsymbol{B} \parallel [001]$ or $\boldsymbol{B} \perp [010]$) and strain $\perp [001]$<br>• $\boldsymbol{B}$ in generic direction
$P2/c$ (13.65) $\mathbb{Z}_2 \times \mathbb{Z}_2$	• strain $\perp [100]$
$P2_1'/c'$ (14.79) $\mathbb{Z}_2 \times \mathbb{Z}_4$	• strain $\perp [010]$<br>• ($\boldsymbol{B} \parallel [001]$ or $\boldsymbol{B} \perp [010]$)
$P2_1'/m'$ (11.54) $\mathbb{Z}_2 \times \mathbb{Z}_2 \times \mathbb{Z}_4$	• strain $\perp [001]$<br>• ($\boldsymbol{B} \parallel [010]$ or $\boldsymbol{B} \perp [001]$)

Table. S 494: Subgroups of $Pb'cm'$ (57.384) that can be realized by applying $\boldsymbol{E}/\boldsymbol{B}$ fields and/or uniaxial strains.

Subgroup & SI group	Perturbation
Pm' (6.20) $\mathbb{Z}_1$	• $\boldsymbol{E} \parallel [100]$ and strain $\perp [001]$<br>• $\boldsymbol{E} \parallel [100]$ and ($\boldsymbol{B} \parallel [100]$ or $\boldsymbol{B} \perp [001]$)<br>• $\boldsymbol{E} \parallel [010]$ and strain $\perp [001]$<br>• $\boldsymbol{E} \parallel [010]$ and ($\boldsymbol{B} \parallel [100]$ or $\boldsymbol{B} \perp [001]$)<br>• $\boldsymbol{E} \perp [001]$
Pc (7.24) $\mathbb{Z}_1$	• $\boldsymbol{E} \parallel [100]$ and strain $\perp [010]$<br>• $\boldsymbol{E} \parallel [001]$ and strain $\perp [010]$<br>• $\boldsymbol{E} \perp [010]$
$P2'$ (3.3) $\mathbb{Z}_1$	• $\boldsymbol{E} \parallel [100]$ and strain $\perp [100]$<br>• $\boldsymbol{E} \parallel [100]$ and ($\boldsymbol{B} \parallel [001]$ or $\boldsymbol{B} \perp [100]$)
$Pm'a2'$ (28.89) $\mathbb{Z}_1$	• $\boldsymbol{E} \parallel [100]$

Continued on next page

...continued

Subgroup & SI group	Perturbation
Pc' (7.26) $\mathbb{Z}_1$	• $\boldsymbol{E} \parallel [010]$ and strain $\perp [100]$<br>• $\boldsymbol{E} \parallel [010]$ and ($\boldsymbol{B} \parallel [001]$ or $\boldsymbol{B} \perp [100]$)<br>• $\boldsymbol{E} \parallel [001]$ and strain $\perp [100]$<br>• $\boldsymbol{E} \parallel [001]$ and ($\boldsymbol{B} \parallel [001]$ or $\boldsymbol{B} \perp [100]$)<br>• $\boldsymbol{E} \perp [100]$
$P2_1$ (4.7) $\mathbb{Z}_1$	• $\boldsymbol{E} \parallel [010]$ and strain $\perp [010]$
$Pm'c'2_1$ (26.70) $\mathbb{Z}_1$	• $\boldsymbol{E} \parallel [010]$
$P2_1'$ (4.9) $\mathbb{Z}_1$	• $\boldsymbol{E} \parallel [001]$ and strain $\perp [001]$<br>• $\boldsymbol{E} \parallel [001]$ and ($\boldsymbol{B} \parallel [100]$ or $\boldsymbol{B} \perp [001]$)
$Pca'2_1'$ (29.102) $\mathbb{Z}_1$	• $\boldsymbol{E} \parallel [001]$
$P\bar{1}$ (2.4) $\mathbb{Z}_2 \times \mathbb{Z}_2 \times \mathbb{Z}_2 \times \mathbb{Z}_4$	• strain in generic direction<br>• ($\boldsymbol{B} \parallel [100]$ or $\boldsymbol{B} \perp [001]$) and strain $\perp [100]$<br>• ($\boldsymbol{B} \parallel [100]$ or $\boldsymbol{B} \perp [001]$) and strain $\perp [010]$<br>• ($\boldsymbol{B} \parallel [001]$ or $\boldsymbol{B} \perp [100]$) and strain $\perp [010]$<br>• ($\boldsymbol{B} \parallel [001]$ or $\boldsymbol{B} \perp [100]$) and strain $\perp [001]$<br>• $\boldsymbol{B}$ in generic direction
$P2'/c'$ (13.69) $\mathbb{Z}_2 \times \mathbb{Z}_2 \times \mathbb{Z}_4$	• strain $\perp [100]$<br>• ($\boldsymbol{B} \parallel [001]$ or $\boldsymbol{B} \perp [100]$)
$P2_1/c$ (14.75) $\mathbb{Z}_2$	• strain $\perp [010]$
$P2_1'/m'$ (11.54) $\mathbb{Z}_2 \times \mathbb{Z}_2 \times \mathbb{Z}_4$	• strain $\perp [001]$<br>• ($\boldsymbol{B} \parallel [100]$ or $\boldsymbol{B} \perp [001]$)

Table. S 495: Subgroups of $Pb'c'm'$ (57.385) that can be realized by applying $\boldsymbol{E}/\boldsymbol{B}$ fields and/or uniaxial strains.

Subgroup & SI group	Perturbation
Pm' (6.20) $\mathbb{Z}_1$	• $\boldsymbol{B} \parallel [100]$ and strain $\perp [001]$<br>• $\boldsymbol{B} \parallel [010]$ and strain $\perp [001]$<br>• $\boldsymbol{B} \perp [001]$<br>• $\boldsymbol{E} \parallel [100]$ and strain $\perp [001]$<br>• $\boldsymbol{E} \parallel [100]$ and $\boldsymbol{B} \parallel [010]$<br>• $\boldsymbol{E} \parallel [010]$ and strain $\perp [001]$<br>• $\boldsymbol{E} \parallel [010]$ and $\boldsymbol{B} \parallel [100]$<br>• $\boldsymbol{E} \perp [001]$

Continued on next page

. . . continued

Subgroup & SI group	Perturbation
Pc' (7.26) $\mathbb{Z}_1$	• $\boldsymbol{B}$ ∥ [100] and strain ⊥ [010]<br>• $\boldsymbol{B}$ ∥ [001] and strain ⊥ [010]<br>• $\boldsymbol{B}$ ⊥ [010]<br>• $\boldsymbol{E}$ ∥ [100] and strain ⊥ [010]<br>• $\boldsymbol{E}$ ∥ [100] and $\boldsymbol{B}$ ∥ [001]<br>• $\boldsymbol{E}$ ∥ [001] and strain ⊥ [010]<br>• $\boldsymbol{E}$ ∥ [001] and $\boldsymbol{B}$ ∥ [100]<br>• $\boldsymbol{E}$ ⊥ [010]
$P2$ (3.1) $\mathbb{Z}_2$	• $\boldsymbol{B}$ ∥ [100] and strain ⊥ [100]<br>• $\boldsymbol{E}$ ∥ [100] and strain ⊥ [100]
$Pm'a'2$ (28.91) $\mathbb{Z}_2$	• $\boldsymbol{B}$ ∥ [100]<br>• $\boldsymbol{E}$ ∥ [100]
Pc' (7.26) $\mathbb{Z}_1$	• $\boldsymbol{B}$ ∥ [010] and strain ⊥ [100]<br>• $\boldsymbol{B}$ ∥ [001] and strain ⊥ [100]<br>• $\boldsymbol{B}$ ⊥ [100]<br>• $\boldsymbol{E}$ ∥ [010] and strain ⊥ [100]<br>• $\boldsymbol{E}$ ∥ [010] and $\boldsymbol{B}$ ∥ [001]<br>• $\boldsymbol{E}$ ∥ [001] and strain ⊥ [100]<br>• $\boldsymbol{E}$ ∥ [001] and $\boldsymbol{B}$ ∥ [010]<br>• $\boldsymbol{E}$ ⊥ [100]
$P2_1$ (4.7) $\mathbb{Z}_1$	• $\boldsymbol{B}$ ∥ [010] and strain ⊥ [010]<br>• $\boldsymbol{E}$ ∥ [010] and strain ⊥ [010]
$Pm'c'2_1$ (26.70) $\mathbb{Z}_1$	• $\boldsymbol{B}$ ∥ [010]<br>• $\boldsymbol{E}$ ∥ [010]
$P2_1$ (4.7) $\mathbb{Z}_1$	• $\boldsymbol{B}$ ∥ [001] and strain ⊥ [001]<br>• $\boldsymbol{E}$ ∥ [001] and strain ⊥ [001]
$Pc'a'2_1$ (29.103) $\mathbb{Z}_1$	• $\boldsymbol{B}$ ∥ [001]<br>• $\boldsymbol{E}$ ∥ [001]
$P\bar{1}'$ (2.6) $\mathbb{Z}_1$	• strain in generic direction
$P2/c'$ (13.68) $\mathbb{Z}_2$	• strain ⊥ [100]
$P2_1/c'$ (14.78) $\mathbb{Z}_1$	• strain ⊥ [010]
$P2_1/m'$ (11.53) $\mathbb{Z}_1$	• strain ⊥ [001]

Table. S 496: Subgroups of P_abcm (57.386) that can be realized by applying $\boldsymbol{E}/\boldsymbol{B}$ fields and/or uniaxial strains.

Subgroup & SI group	Perturbation
Pm (6.18) $\mathbb{Z}_1$	• $\boldsymbol{E} \parallel [100]$ and $\boldsymbol{B} \parallel [001]$ and strain $\perp [001]$<br>• $\boldsymbol{E} \parallel [010]$ and $\boldsymbol{B} \parallel [001]$ and strain $\perp [001]$<br>• $\boldsymbol{E} \perp [001]$ and $\boldsymbol{B} \parallel [001]$
Pc (7.24) $\mathbb{Z}_1$	• $\boldsymbol{E} \parallel [100]$ and $\boldsymbol{B} \parallel [010]$ and strain $\perp [010]$<br>• $\boldsymbol{E} \parallel [001]$ and $\boldsymbol{B} \parallel [010]$ and strain $\perp [010]$<br>• $\boldsymbol{E} \perp [010]$ and $\boldsymbol{B} \parallel [010]$
$P2$ (3.1) $\mathbb{Z}_2$	• $\boldsymbol{E} \parallel [100]$ and $\boldsymbol{B} \parallel [100]$ and strain $\perp [100]$
P_S1 (1.3) $\mathbb{Z}_1$	• $\boldsymbol{E} \parallel [100]$ and strain in generic direction<br>• $\boldsymbol{E} \parallel [010]$ and strain in generic direction<br>• $\boldsymbol{E} \parallel [001]$ and strain in generic direction<br>• $\boldsymbol{E} \perp [100]$ and strain $\perp [010]$<br>• $\boldsymbol{E} \perp [100]$ and strain $\perp [001]$<br>• $\boldsymbol{E} \perp [100]$ and strain in generic direction<br>• $\boldsymbol{E} \perp [010]$ and strain $\perp [100]$<br>• $\boldsymbol{E} \perp [010]$ and strain $\perp [001]$<br>• $\boldsymbol{E} \perp [010]$ and strain in generic direction<br>• $\boldsymbol{E} \perp [001]$ and strain $\perp [100]$<br>• $\boldsymbol{E} \perp [001]$ and strain $\perp [010]$<br>• $\boldsymbol{E} \perp [001]$ and strain in generic direction<br>• $\boldsymbol{E}$ in generic direction
Pc' (7.26) $\mathbb{Z}_1$	• $\boldsymbol{E} \parallel [100]$ and $\boldsymbol{B} \parallel [100]$ and strain $\perp [001]$<br>• $\boldsymbol{E} \parallel [100]$ and $\boldsymbol{B} \parallel [010]$ and strain $\perp [001]$<br>• $\boldsymbol{E} \parallel [100]$ and $\boldsymbol{B} \perp [001]$<br>• $\boldsymbol{E} \parallel [010]$ and $\boldsymbol{B} \parallel [100]$ and strain $\perp [001]$<br>• $\boldsymbol{E} \parallel [010]$ and $\boldsymbol{B} \parallel [010]$ and strain $\perp [001]$<br>• $\boldsymbol{E} \parallel [010]$ and $\boldsymbol{B} \perp [001]$<br>• $\boldsymbol{E} \perp [001]$ and $\boldsymbol{B} \parallel [100]$<br>• $\boldsymbol{E} \perp [001]$ and $\boldsymbol{B} \parallel [010]$<br>• $\boldsymbol{E} \perp [001]$ and $\boldsymbol{B} \perp [001]$
P_am (6.21) $\mathbb{Z}_1$	• $\boldsymbol{E} \parallel [100]$ and strain $\perp [001]$<br>• $\boldsymbol{E} \parallel [010]$ and strain $\perp [001]$<br>• $\boldsymbol{E} \perp [001]$

Continued on next page

...continued

Subgroup & SI group	Perturbation
Pc' (7.26) $\mathbb{Z}_1$	• $\boldsymbol{E}$ ∥ [100] and $\boldsymbol{B}$ ∥ [100] and strain ⊥ [010]<br>• $\boldsymbol{E}$ ∥ [100] and $\boldsymbol{B}$ ∥ [001] and strain ⊥ [010]<br>• $\boldsymbol{E}$ ∥ [100] and $\boldsymbol{B}$ ⊥ [010]<br>• $\boldsymbol{E}$ ∥ [001] and $\boldsymbol{B}$ ∥ [100] and strain ⊥ [010]<br>• $\boldsymbol{E}$ ∥ [001] and $\boldsymbol{B}$ ∥ [001] and strain ⊥ [010]<br>• $\boldsymbol{E}$ ∥ [001] and $\boldsymbol{B}$ ⊥ [010]<br>• $\boldsymbol{E}$ ⊥ [010] and $\boldsymbol{B}$ ∥ [100]<br>• $\boldsymbol{E}$ ⊥ [010] and $\boldsymbol{B}$ ∥ [001]<br>• $\boldsymbol{E}$ ⊥ [010] and $\boldsymbol{B}$ ⊥ [010]
P_ac (7.27) $\mathbb{Z}_1$	• $\boldsymbol{E}$ ∥ [100] and strain ⊥ [010]<br>• $\boldsymbol{E}$ ∥ [001] and strain ⊥ [010]<br>• $\boldsymbol{E}$ ⊥ [010]
$Pn'c'2$ (30.115) $\mathbb{Z}_1$	• $\boldsymbol{E}$ ∥ [100] and $\boldsymbol{B}$ ∥ [100]
$P2_1'$ (4.9) $\mathbb{Z}_1$	• $\boldsymbol{E}$ ∥ [100] and $\boldsymbol{B}$ ∥ [010] and strain ⊥ [100]<br>• $\boldsymbol{E}$ ∥ [100] and $\boldsymbol{B}$ ∥ [001] and strain ⊥ [100]<br>• $\boldsymbol{E}$ ∥ [100] and $\boldsymbol{B}$ ⊥ [100]
P_b2 (3.5) $\mathbb{Z}_2$	• $\boldsymbol{E}$ ∥ [100] and strain ⊥ [100]
$Pc'a2_1'$ (29.101) $\mathbb{Z}_1$	• $\boldsymbol{E}$ ∥ [100] and $\boldsymbol{B}$ ∥ [010]
$Pmn'2_1'$ (31.126) $\mathbb{Z}_1$	• $\boldsymbol{E}$ ∥ [100] and $\boldsymbol{B}$ ∥ [001]
P_cma2 (28.94) $\mathbb{Z}_1$	• $\boldsymbol{E}$ ∥ [100]
Pc (7.24) $\mathbb{Z}_1$	• $\boldsymbol{E}$ ∥ [010] and $\boldsymbol{B}$ ∥ [100] and strain ⊥ [100]<br>• $\boldsymbol{E}$ ∥ [001] and $\boldsymbol{B}$ ∥ [100] and strain ⊥ [100]<br>• $\boldsymbol{E}$ ⊥ [100] and $\boldsymbol{B}$ ∥ [100]
$P2_1$ (4.7) $\mathbb{Z}_1$	• $\boldsymbol{E}$ ∥ [010] and $\boldsymbol{B}$ ∥ [010] and strain ⊥ [010]
$P2_1$ (4.7) $\mathbb{Z}_1$	• $\boldsymbol{E}$ ∥ [001] and $\boldsymbol{B}$ ∥ [001] and strain ⊥ [001]

Continued on next page

Subgroup & SI group	Perturbation
$P\bar{1}$ (2.4) $\mathbb{Z}_2 \times \mathbb{Z}_2 \times \mathbb{Z}_2 \times \mathbb{Z}_4$	• $\boldsymbol{B} \parallel [100]$ and strain in generic direction<br>• $\boldsymbol{B} \parallel [010]$ and strain in generic direction<br>• $\boldsymbol{B} \parallel [001]$ and strain in generic direction<br>• $\boldsymbol{B} \perp [100]$ and strain $\perp [010]$<br>• $\boldsymbol{B} \perp [100]$ and strain $\perp [001]$<br>• $\boldsymbol{B} \perp [100]$ and strain in generic direction<br>• $\boldsymbol{B} \perp [010]$ and strain $\perp [100]$<br>• $\boldsymbol{B} \perp [010]$ and strain $\perp [001]$<br>• $\boldsymbol{B} \perp [010]$ and strain in generic direction<br>• $\boldsymbol{B} \perp [001]$ and strain $\perp [100]$<br>• $\boldsymbol{B} \perp [001]$ and strain $\perp [010]$<br>• $\boldsymbol{B} \perp [001]$ and strain in generic direction<br>• $\boldsymbol{B}$ in generic direction
$P2/c$ (13.65) $\mathbb{Z}_2 \times \mathbb{Z}_2$	• $\boldsymbol{B} \parallel [100]$ and strain $\perp [100]$
$P2_1/c$ (14.75) $\mathbb{Z}_2$	• $\boldsymbol{B} \parallel [010]$ and strain $\perp [010]$
$P2_1/m$ (11.50) $\mathbb{Z}_2$	• $\boldsymbol{B} \parallel [001]$ and strain $\perp [001]$
Pc' (7.26) $\mathbb{Z}_1$	• $\boldsymbol{E} \parallel [010]$ and $\boldsymbol{B} \parallel [010]$ and strain $\perp [100]$<br>• $\boldsymbol{E} \parallel [010]$ and $\boldsymbol{B} \parallel [001]$ and strain $\perp [100]$<br>• $\boldsymbol{E} \parallel [010]$ and $\boldsymbol{B} \perp [100]$<br>• $\boldsymbol{E} \parallel [001]$ and $\boldsymbol{B} \parallel [010]$ and strain $\perp [100]$<br>• $\boldsymbol{E} \parallel [001]$ and $\boldsymbol{B} \parallel [001]$ and strain $\perp [100]$<br>• $\boldsymbol{E} \parallel [001]$ and $\boldsymbol{B} \perp [100]$<br>• $\boldsymbol{E} \perp [100]$ and $\boldsymbol{B} \parallel [010]$<br>• $\boldsymbol{E} \perp [100]$ and $\boldsymbol{B} \parallel [001]$<br>• $\boldsymbol{E} \perp [100]$ and $\boldsymbol{B} \perp [100]$
P_bc (7.29) $\mathbb{Z}_1$	• $\boldsymbol{E} \parallel [010]$ and strain $\perp [100]$<br>• $\boldsymbol{E} \parallel [001]$ and strain $\perp [100]$<br>• $\boldsymbol{E} \perp [100]$
$Pc'a'2_1$ (29.103) $\mathbb{Z}_1$	• $\boldsymbol{E} \parallel [010]$ and $\boldsymbol{B} \parallel [010]$
$Pn'a'2_1$ (33.148) $\mathbb{Z}_1$	• $\boldsymbol{E} \parallel [001]$ and $\boldsymbol{B} \parallel [001]$
$P2_1'/c'$ (14.79) $\mathbb{Z}_2 \times \mathbb{Z}_4$	• $\boldsymbol{B} \parallel [010]$ and strain $\perp [100]$<br>• $\boldsymbol{B} \parallel [001]$ and strain $\perp [100]$<br>• $\boldsymbol{B} \perp [100]$
$P2_1'$ (4.9) $\mathbb{Z}_1$	• $\boldsymbol{E} \parallel [010]$ and $\boldsymbol{B} \parallel [100]$ and strain $\perp [010]$<br>• $\boldsymbol{E} \parallel [010]$ and $\boldsymbol{B} \parallel [001]$ and strain $\perp [010]$<br>• $\boldsymbol{E} \parallel [010]$ and $\boldsymbol{B} \perp [010]$

Continued on next page

...continued

Subgroup & SI group	Perturbation
$Pca'2_1'$ (29.102) $\mathbb{Z}_1$	• $\boldsymbol{E}$ ∥ [010] and $\boldsymbol{B}$ ∥ [100]
P_a2_1 (4.10) $\mathbb{Z}_1$	• $\boldsymbol{E}$ ∥ [010] and strain ⊥ [010]
$P2_1'/c'$ (14.79) $\mathbb{Z}_2 \times \mathbb{Z}_4$	• $\boldsymbol{B}$ ∥ [100] and strain ⊥ [010]<br>• $\boldsymbol{B}$ ∥ [001] and strain ⊥ [010]<br>• $\boldsymbol{B}$ ⊥ [010]
$Pmc'2_1'$ (26.69) $\mathbb{Z}_1$	• $\boldsymbol{E}$ ∥ [010] and $\boldsymbol{B}$ ∥ [001]
P_bmc2_1 (26.72) $\mathbb{Z}_1$	• $\boldsymbol{E}$ ∥ [010]
$Pn'ma'$ (62.448) $\mathbb{Z}_2$	• $\boldsymbol{B}$ ∥ [001]
$P2_1'$ (4.9) $\mathbb{Z}_1$	• $\boldsymbol{E}$ ∥ [001] and $\boldsymbol{B}$ ∥ [100] and strain ⊥ [001]<br>• $\boldsymbol{E}$ ∥ [001] and $\boldsymbol{B}$ ∥ [010] and strain ⊥ [001]<br>• $\boldsymbol{E}$ ∥ [001] and $\boldsymbol{B}$ ⊥ [001]
$Pn'a2_1'$ (33.146) $\mathbb{Z}_1$	• $\boldsymbol{E}$ ∥ [001] and $\boldsymbol{B}$ ∥ [100]
P_a2_1 (4.10) $\mathbb{Z}_1$	• $\boldsymbol{E}$ ∥ [001] and strain ⊥ [001]
$P2_1'/c'$ (14.79) $\mathbb{Z}_2 \times \mathbb{Z}_4$	• $\boldsymbol{B}$ ∥ [100] and strain ⊥ [001]<br>• $\boldsymbol{B}$ ∥ [010] and strain ⊥ [001]<br>• $\boldsymbol{B}$ ⊥ [001]
$Pca'2_1'$ (29.102) $\mathbb{Z}_1$	• $\boldsymbol{E}$ ∥ [001] and $\boldsymbol{B}$ ∥ [010]
P_bca2_1 (29.105) $\mathbb{Z}_2$	• $\boldsymbol{E}$ ∥ [001]
$Pb'c'a$ (61.436) $\mathbb{Z}_2$	• $\boldsymbol{B}$ ∥ [010]
$Pb'cn'$ (60.424) $\mathbb{Z}_2 \times \mathbb{Z}_2$	• $\boldsymbol{B}$ ∥ [100]
$P_S\bar{1}$ (2.7) $\mathbb{Z}_2 \times \mathbb{Z}_2 \times \mathbb{Z}_4$	• strain in generic direction
P_b2/c (13.71) $\mathbb{Z}_2 \times \mathbb{Z}_2$	• strain ⊥ [100]
P_a2_1/c (14.80) $\mathbb{Z}_2$	• strain ⊥ [010]
P_a2_1/m (11.55) $\mathbb{Z}_2$	• strain ⊥ [001]

Table. S 497: Subgroups of P_bbcm (57.387) that can be realized by applying $\boldsymbol{E}/\boldsymbol{B}$ fields and/or uniaxial strains.

Subgroup & SI group	Perturbation

Continued on next page

...continued

Subgroup & SI group	Perturbation
Pm (6.18) $\mathbb{Z}_1$	• $\boldsymbol{E} \parallel [100]$ and $\boldsymbol{B} \parallel [001]$ and strain $\perp [001]$<br>• $\boldsymbol{E} \parallel [010]$ and $\boldsymbol{B} \parallel [001]$ and strain $\perp [001]$<br>• $\boldsymbol{E} \perp [001]$ and $\boldsymbol{B} \parallel [001]$
P_S1 (1.3) $\mathbb{Z}_1$	• $\boldsymbol{E} \parallel [100]$ and strain in generic direction<br>• $\boldsymbol{E} \parallel [010]$ and strain in generic direction<br>• $\boldsymbol{E} \parallel [001]$ and strain in generic direction<br>• $\boldsymbol{E} \perp [100]$ and strain $\perp [010]$<br>• $\boldsymbol{E} \perp [100]$ and strain $\perp [001]$<br>• $\boldsymbol{E} \perp [100]$ and strain in generic direction<br>• $\boldsymbol{E} \perp [010]$ and strain $\perp [100]$<br>• $\boldsymbol{E} \perp [010]$ and strain $\perp [001]$<br>• $\boldsymbol{E} \perp [010]$ and strain in generic direction<br>• $\boldsymbol{E} \perp [001]$ and strain $\perp [100]$<br>• $\boldsymbol{E} \perp [001]$ and strain $\perp [010]$<br>• $\boldsymbol{E} \perp [001]$ and strain in generic direction<br>• $\boldsymbol{E}$ in generic direction
Pc' (7.26) $\mathbb{Z}_1$	• $\boldsymbol{E} \parallel [100]$ and $\boldsymbol{B} \parallel [100]$ and strain $\perp [001]$<br>• $\boldsymbol{E} \parallel [100]$ and $\boldsymbol{B} \parallel [010]$ and strain $\perp [001]$<br>• $\boldsymbol{E} \parallel [100]$ and $\boldsymbol{B} \perp [001]$<br>• $\boldsymbol{E} \parallel [010]$ and $\boldsymbol{B} \parallel [100]$ and strain $\perp [001]$<br>• $\boldsymbol{E} \parallel [010]$ and $\boldsymbol{B} \parallel [010]$ and strain $\perp [001]$<br>• $\boldsymbol{E} \parallel [010]$ and $\boldsymbol{B} \perp [001]$<br>• $\boldsymbol{E} \perp [001]$ and $\boldsymbol{B} \parallel [100]$<br>• $\boldsymbol{E} \perp [001]$ and $\boldsymbol{B} \parallel [010]$<br>• $\boldsymbol{E} \perp [001]$ and $\boldsymbol{B} \perp [001]$
P_am (6.21) $\mathbb{Z}_1$	• $\boldsymbol{E} \parallel [100]$ and strain $\perp [001]$<br>• $\boldsymbol{E} \parallel [010]$ and strain $\perp [001]$<br>• $\boldsymbol{E} \perp [001]$
Pc' (7.26) $\mathbb{Z}_1$	• $\boldsymbol{E} \parallel [100]$ and $\boldsymbol{B} \parallel [100]$ and strain $\perp [010]$<br>• $\boldsymbol{E} \parallel [100]$ and $\boldsymbol{B} \parallel [001]$ and strain $\perp [010]$<br>• $\boldsymbol{E} \parallel [100]$ and $\boldsymbol{B} \perp [010]$<br>• $\boldsymbol{E} \parallel [001]$ and $\boldsymbol{B} \parallel [100]$ and strain $\perp [010]$<br>• $\boldsymbol{E} \parallel [001]$ and $\boldsymbol{B} \parallel [001]$ and strain $\perp [010]$<br>• $\boldsymbol{E} \parallel [001]$ and $\boldsymbol{B} \perp [010]$<br>• $\boldsymbol{E} \perp [010]$ and $\boldsymbol{B} \parallel [100]$<br>• $\boldsymbol{E} \perp [010]$ and $\boldsymbol{B} \parallel [001]$<br>• $\boldsymbol{E} \perp [010]$ and $\boldsymbol{B} \perp [010]$

Continued on next page

. . . continued

Subgroup & SI group	Perturbation
$P2'$ (3.3) $\mathbb{Z}_1$	• $\boldsymbol{E} \parallel [100]$ and $\boldsymbol{B} \parallel [010]$ and strain $\perp [100]$<br>• $\boldsymbol{E} \parallel [100]$ and $\boldsymbol{B} \parallel [001]$ and strain $\perp [100]$<br>• $\boldsymbol{E} \parallel [100]$ and $\boldsymbol{B} \perp [100]$
$Pma'2'$ (28.90) $\mathbb{Z}_1$	• $\boldsymbol{E} \parallel [100]$ and $\boldsymbol{B} \parallel [001]$
Pc (7.24) $\mathbb{Z}_1$	• $\boldsymbol{E} \parallel [100]$ and $\boldsymbol{B} \parallel [010]$ and strain $\perp [010]$<br>• $\boldsymbol{E} \parallel [001]$ and $\boldsymbol{B} \parallel [010]$ and strain $\perp [010]$<br>• $\boldsymbol{E} \perp [010]$ and $\boldsymbol{B} \parallel [010]$
P_bc (7.29) $\mathbb{Z}_1$	• $\boldsymbol{E} \parallel [100]$ and strain $\perp [010]$<br>• $\boldsymbol{E} \parallel [001]$ and strain $\perp [010]$<br>• $\boldsymbol{E} \perp [010]$
$Pb'a2'$ (32.137) $\mathbb{Z}_1$	• $\boldsymbol{E} \parallel [100]$ and $\boldsymbol{B} \parallel [010]$
$P2$ (3.1) $\mathbb{Z}_2$	• $\boldsymbol{E} \parallel [100]$ and $\boldsymbol{B} \parallel [100]$ and strain $\perp [100]$
$Pb'a'2$ (32.138) $\mathbb{Z}_2$	• $\boldsymbol{E} \parallel [100]$ and $\boldsymbol{B} \parallel [100]$
P_a2 (3.4) $\mathbb{Z}_2$	• $\boldsymbol{E} \parallel [100]$ and strain $\perp [100]$
P_bma2 (28.93) $\mathbb{Z}_1$	• $\boldsymbol{E} \parallel [100]$
Pm' (6.20) $\mathbb{Z}_1$	• $\boldsymbol{E} \parallel [010]$ and $\boldsymbol{B} \parallel [010]$ and strain $\perp [100]$<br>• $\boldsymbol{E} \parallel [010]$ and $\boldsymbol{B} \parallel [001]$ and strain $\perp [100]$<br>• $\boldsymbol{E} \parallel [010]$ and $\boldsymbol{B} \perp [100]$<br>• $\boldsymbol{E} \parallel [001]$ and $\boldsymbol{B} \parallel [010]$ and strain $\perp [100]$<br>• $\boldsymbol{E} \parallel [001]$ and $\boldsymbol{B} \parallel [001]$ and strain $\perp [100]$<br>• $\boldsymbol{E} \parallel [001]$ and $\boldsymbol{B} \perp [100]$<br>• $\boldsymbol{E} \perp [100]$ and $\boldsymbol{B} \parallel [010]$<br>• $\boldsymbol{E} \perp [100]$ and $\boldsymbol{B} \parallel [001]$<br>• $\boldsymbol{E} \perp [100]$ and $\boldsymbol{B} \perp [100]$
$P2'$ (3.3) $\mathbb{Z}_1$	• $\boldsymbol{E} \parallel [010]$ and $\boldsymbol{B} \parallel [100]$ and strain $\perp [010]$<br>• $\boldsymbol{E} \parallel [010]$ and $\boldsymbol{B} \parallel [001]$ and strain $\perp [010]$<br>• $\boldsymbol{E} \parallel [010]$ and $\boldsymbol{B} \perp [010]$
$Pm'm2'$ (25.59) $\mathbb{Z}_1$	• $\boldsymbol{E} \parallel [010]$ and $\boldsymbol{B} \parallel [001]$
Pc (7.24) $\mathbb{Z}_1$	• $\boldsymbol{E} \parallel [010]$ and $\boldsymbol{B} \parallel [100]$ and strain $\perp [100]$<br>• $\boldsymbol{E} \parallel [001]$ and $\boldsymbol{B} \parallel [100]$ and strain $\perp [100]$<br>• $\boldsymbol{E} \perp [100]$ and $\boldsymbol{B} \parallel [100]$
P_cc (7.28) $\mathbb{Z}_1$	• $\boldsymbol{E} \parallel [010]$ and strain $\perp [100]$<br>• $\boldsymbol{E} \parallel [001]$ and strain $\perp [100]$<br>• $\boldsymbol{E} \perp [100]$
$Pc'c2'$ (27.80) $\mathbb{Z}_1$	• $\boldsymbol{E} \parallel [010]$ and $\boldsymbol{B} \parallel [100]$

Continued on next page

…continued

Subgroup & SI group	Perturbation
$P2_1$ (4.7) $\mathbb{Z}_1$	• $\boldsymbol{E}$ ∥ [010] and $\boldsymbol{B}$ ∥ [010] and strain ⊥ [010]
$Pm'c'2_1$ (26.70) $\mathbb{Z}_1$	• $\boldsymbol{E}$ ∥ [010] and $\boldsymbol{B}$ ∥ [010]
P_b2_1 (4.11) $\mathbb{Z}_1$	• $\boldsymbol{E}$ ∥ [010] and strain ⊥ [010]
P_cmc2_1 (26.73) $\mathbb{Z}_1$	• $\boldsymbol{E}$ ∥ [010]
$P2_1$ (4.7) $\mathbb{Z}_1$	• $\boldsymbol{E}$ ∥ [001] and $\boldsymbol{B}$ ∥ [001] and strain ⊥ [001]
$Pm'c'2_1$ (26.70) $\mathbb{Z}_1$	• $\boldsymbol{E}$ ∥ [001] and $\boldsymbol{B}$ ∥ [001]
$P\bar{1}$ (2.4) $\mathbb{Z}_2 \times \mathbb{Z}_2 \times \mathbb{Z}_2 \times \mathbb{Z}_4$	• $\boldsymbol{B}$ ∥ [100] and strain in generic direction<br>• $\boldsymbol{B}$ ∥ [010] and strain in generic direction<br>• $\boldsymbol{B}$ ∥ [001] and strain in generic direction<br>• $\boldsymbol{B}$ ⊥ [100] and strain ⊥ [010]<br>• $\boldsymbol{B}$ ⊥ [100] and strain ⊥ [001]<br>• $\boldsymbol{B}$ ⊥ [100] and strain in generic direction<br>• $\boldsymbol{B}$ ⊥ [010] and strain ⊥ [100]<br>• $\boldsymbol{B}$ ⊥ [010] and strain ⊥ [001]<br>• $\boldsymbol{B}$ ⊥ [010] and strain in generic direction<br>• $\boldsymbol{B}$ ⊥ [001] and strain ⊥ [100]<br>• $\boldsymbol{B}$ ⊥ [001] and strain ⊥ [010]<br>• $\boldsymbol{B}$ ⊥ [001] and strain in generic direction<br>• $\boldsymbol{B}$ in generic direction
$P2'/m'$ (10.46) $\mathbb{Z}_2 \times \mathbb{Z}_2 \times \mathbb{Z}_2 \times \mathbb{Z}_4$	• $\boldsymbol{B}$ ∥ [010] and strain ⊥ [100]<br>• $\boldsymbol{B}$ ∥ [001] and strain ⊥ [100]<br>• $\boldsymbol{B}$ ⊥ [100]
$P2'/c'$ (13.69) $\mathbb{Z}_2 \times \mathbb{Z}_2 \times \mathbb{Z}_4$	• $\boldsymbol{B}$ ∥ [100] and strain ⊥ [010]<br>• $\boldsymbol{B}$ ∥ [001] and strain ⊥ [010]<br>• $\boldsymbol{B}$ ⊥ [010]
$P2/c$ (13.65) $\mathbb{Z}_2 \times \mathbb{Z}_2$	• $\boldsymbol{B}$ ∥ [100] and strain ⊥ [100]
$P2_1/c$ (14.75) $\mathbb{Z}_2$	• $\boldsymbol{B}$ ∥ [010] and strain ⊥ [010]
$P2_1/m$ (11.50) $\mathbb{Z}_2$	• $\boldsymbol{B}$ ∥ [001] and strain ⊥ [001]
$Pmm'a'$ (51.295) $\mathbb{Z}_2$	• $\boldsymbol{B}$ ∥ [001]
$P2_1'$ (4.9) $\mathbb{Z}_1$	• $\boldsymbol{E}$ ∥ [001] and $\boldsymbol{B}$ ∥ [100] and strain ⊥ [001]<br>• $\boldsymbol{E}$ ∥ [001] and $\boldsymbol{B}$ ∥ [010] and strain ⊥ [001]<br>• $\boldsymbol{E}$ ∥ [001] and $\boldsymbol{B}$ ⊥ [001]
$Pm'c2_1'$ (26.68) $\mathbb{Z}_1$	• $\boldsymbol{E}$ ∥ [001] and $\boldsymbol{B}$ ∥ [010]
$Pc'a2_1'$ (29.101) $\mathbb{Z}_1$	• $\boldsymbol{E}$ ∥ [001] and $\boldsymbol{B}$ ∥ [100]

Continued on next page

...continued

Subgroup & SI group	Perturbation
P_a2_1 (4.10) $\mathbb{Z}_1$	• $\boldsymbol{E}$ ∥ [001] and strain ⊥ [001]
P_aca2_1 (29.104) $\mathbb{Z}_1$	• $\boldsymbol{E}$ ∥ [001]
$P2_1'/c'$ (14.79) $\mathbb{Z}_2 \times \mathbb{Z}_4$	• $\boldsymbol{B}$ ∥ [100] and strain ⊥ [001]<br>• $\boldsymbol{B}$ ∥ [010] and strain ⊥ [001]<br>• $\boldsymbol{B}$ ⊥ [001]
$Pc'ca'$ (54.344) $\mathbb{Z}_2 \times \mathbb{Z}_2$	• $\boldsymbol{B}$ ∥ [100]
$Pb'am'$ (55.358) $\mathbb{Z}_2$	• $\boldsymbol{B}$ ∥ [010]
$P_S\bar{1}$ (2.7) $\mathbb{Z}_2 \times \mathbb{Z}_2 \times \mathbb{Z}_4$	• strain in generic direction
P_c2/c (13.72) $\mathbb{Z}_2 \times \mathbb{Z}_2$	• strain ⊥ [100]
P_b2_1/c (14.81) $\mathbb{Z}_2$	• strain ⊥ [010]
P_a2_1/m (11.55) $\mathbb{Z}_2$	• strain ⊥ [001]

Table. S 498: Subgroups of P_cbcm (57.388) that can be realized by applying $\boldsymbol{E}/\boldsymbol{B}$ fields and/or uniaxial strains.

Subgroup & SI group	Perturbation
P_S1 (1.3) $\mathbb{Z}_1$	• $\boldsymbol{E}$ ∥ [100] and strain in generic direction<br>• $\boldsymbol{E}$ ∥ [010] and strain in generic direction<br>• $\boldsymbol{E}$ ∥ [001] and strain in generic direction<br>• $\boldsymbol{E}$ ⊥ [100] and strain ⊥ [010]<br>• $\boldsymbol{E}$ ⊥ [100] and strain ⊥ [001]<br>• $\boldsymbol{E}$ ⊥ [100] and strain in generic direction<br>• $\boldsymbol{E}$ ⊥ [010] and strain ⊥ [100]<br>• $\boldsymbol{E}$ ⊥ [010] and strain ⊥ [001]<br>• $\boldsymbol{E}$ ⊥ [010] and strain in generic direction<br>• $\boldsymbol{E}$ ⊥ [001] and strain ⊥ [100]<br>• $\boldsymbol{E}$ ⊥ [001] and strain ⊥ [010]<br>• $\boldsymbol{E}$ ⊥ [001] and strain in generic direction<br>• $\boldsymbol{E}$ in generic direction

Continued on next page

. . . continued

Subgroup & SI group	Perturbation
Pm' (6.20) $\mathbb{Z}_1$	• $\boldsymbol{E} \parallel [100]$ and $\boldsymbol{B} \parallel [100]$ and strain $\perp [001]$<br>• $\boldsymbol{E} \parallel [100]$ and $\boldsymbol{B} \parallel [010]$ and strain $\perp [001]$<br>• $\boldsymbol{E} \parallel [100]$ and $\boldsymbol{B} \perp [001]$<br>• $\boldsymbol{E} \parallel [010]$ and $\boldsymbol{B} \parallel [100]$ and strain $\perp [001]$<br>• $\boldsymbol{E} \parallel [010]$ and $\boldsymbol{B} \parallel [010]$ and strain $\perp [001]$<br>• $\boldsymbol{E} \parallel [010]$ and $\boldsymbol{B} \perp [001]$<br>• $\boldsymbol{E} \perp [001]$ and $\boldsymbol{B} \parallel [100]$<br>• $\boldsymbol{E} \perp [001]$ and $\boldsymbol{B} \parallel [010]$<br>• $\boldsymbol{E} \perp [001]$ and $\boldsymbol{B} \perp [001]$
Pm (6.18) $\mathbb{Z}_1$	• $\boldsymbol{E} \parallel [100]$ and $\boldsymbol{B} \parallel [001]$ and strain $\perp [001]$<br>• $\boldsymbol{E} \parallel [010]$ and $\boldsymbol{B} \parallel [001]$ and strain $\perp [001]$<br>• $\boldsymbol{E} \perp [001]$ and $\boldsymbol{B} \parallel [001]$
P_bm (6.22) $\mathbb{Z}_1$	• $\boldsymbol{E} \parallel [100]$ and strain $\perp [001]$<br>• $\boldsymbol{E} \parallel [010]$ and strain $\perp [001]$<br>• $\boldsymbol{E} \perp [001]$
Pm' (6.20) $\mathbb{Z}_1$	• $\boldsymbol{E} \parallel [100]$ and $\boldsymbol{B} \parallel [100]$ and strain $\perp [010]$<br>• $\boldsymbol{E} \parallel [100]$ and $\boldsymbol{B} \parallel [001]$ and strain $\perp [010]$<br>• $\boldsymbol{E} \parallel [100]$ and $\boldsymbol{B} \perp [010]$<br>• $\boldsymbol{E} \parallel [001]$ and $\boldsymbol{B} \parallel [100]$ and strain $\perp [010]$<br>• $\boldsymbol{E} \parallel [001]$ and $\boldsymbol{B} \parallel [001]$ and strain $\perp [010]$<br>• $\boldsymbol{E} \parallel [001]$ and $\boldsymbol{B} \perp [010]$<br>• $\boldsymbol{E} \perp [010]$ and $\boldsymbol{B} \parallel [100]$<br>• $\boldsymbol{E} \perp [010]$ and $\boldsymbol{B} \parallel [001]$<br>• $\boldsymbol{E} \perp [010]$ and $\boldsymbol{B} \perp [010]$
Pc (7.24) $\mathbb{Z}_1$	• $\boldsymbol{E} \parallel [100]$ and $\boldsymbol{B} \parallel [010]$ and strain $\perp [010]$<br>• $\boldsymbol{E} \parallel [001]$ and $\boldsymbol{B} \parallel [010]$ and strain $\perp [010]$<br>• $\boldsymbol{E} \perp [010]$ and $\boldsymbol{B} \parallel [010]$
P_cc (7.28) $\mathbb{Z}_1$	• $\boldsymbol{E} \parallel [100]$ and strain $\perp [010]$<br>• $\boldsymbol{E} \parallel [001]$ and strain $\perp [010]$<br>• $\boldsymbol{E} \perp [010]$
$P2$ (3.1) $\mathbb{Z}_2$	• $\boldsymbol{E} \parallel [100]$ and $\boldsymbol{B} \parallel [100]$ and strain $\perp [100]$
$Pm'm'2$ (25.60) $\mathbb{Z}_2$	• $\boldsymbol{E} \parallel [100]$ and $\boldsymbol{B} \parallel [100]$
$P2'$ (3.3) $\mathbb{Z}_1$	• $\boldsymbol{E} \parallel [100]$ and $\boldsymbol{B} \parallel [010]$ and strain $\perp [100]$<br>• $\boldsymbol{E} \parallel [100]$ and $\boldsymbol{B} \parallel [001]$ and strain $\perp [100]$<br>• $\boldsymbol{E} \parallel [100]$ and $\boldsymbol{B} \perp [100]$
$Pm'm2'$ (25.59) $\mathbb{Z}_1$	• $\boldsymbol{E} \parallel [100]$ and $\boldsymbol{B} \parallel [001]$

Continued on next page

...continued

Subgroup & SI group	Perturbation
$Pm'a2'$ (28.89) $\mathbb{Z}_1$	• $\boldsymbol{E} \parallel [100]$ and $\boldsymbol{B} \parallel [010]$
P_a2 (3.4) $\mathbb{Z}_2$	• $\boldsymbol{E} \parallel [100]$ and strain $\perp [100]$
P_ama2 (28.92) $\mathbb{Z}_1$	• $\boldsymbol{E} \parallel [100]$
Pc (7.24) $\mathbb{Z}_1$	• $\boldsymbol{E} \parallel [010]$ and $\boldsymbol{B} \parallel [100]$ and strain $\perp [100]$<br>• $\boldsymbol{E} \parallel [001]$ and $\boldsymbol{B} \parallel [100]$ and strain $\perp [100]$<br>• $\boldsymbol{E} \perp [100]$ and $\boldsymbol{B} \parallel [100]$
Pc' (7.26) $\mathbb{Z}_1$	• $\boldsymbol{E} \parallel [010]$ and $\boldsymbol{B} \parallel [010]$ and strain $\perp [100]$<br>• $\boldsymbol{E} \parallel [010]$ and $\boldsymbol{B} \parallel [001]$ and strain $\perp [100]$<br>• $\boldsymbol{E} \parallel [010]$ and $\boldsymbol{B} \perp [100]$<br>• $\boldsymbol{E} \parallel [001]$ and $\boldsymbol{B} \parallel [010]$ and strain $\perp [100]$<br>• $\boldsymbol{E} \parallel [001]$ and $\boldsymbol{B} \parallel [001]$ and strain $\perp [100]$<br>• $\boldsymbol{E} \parallel [001]$ and $\boldsymbol{B} \perp [100]$<br>• $\boldsymbol{E} \perp [100]$ and $\boldsymbol{B} \parallel [010]$<br>• $\boldsymbol{E} \perp [100]$ and $\boldsymbol{B} \parallel [001]$<br>• $\boldsymbol{E} \perp [100]$ and $\boldsymbol{B} \perp [100]$
P_ac (7.27) $\mathbb{Z}_1$	• $\boldsymbol{E} \parallel [010]$ and strain $\perp [100]$<br>• $\boldsymbol{E} \parallel [001]$ and strain $\perp [100]$<br>• $\boldsymbol{E} \perp [100]$
$P2_1'$ (4.9) $\mathbb{Z}_1$	• $\boldsymbol{E} \parallel [010]$ and $\boldsymbol{B} \parallel [100]$ and strain $\perp [010]$<br>• $\boldsymbol{E} \parallel [010]$ and $\boldsymbol{B} \parallel [001]$ and strain $\perp [010]$<br>• $\boldsymbol{E} \parallel [010]$ and $\boldsymbol{B} \perp [010]$
$Pm'c2_1'$ (26.68) $\mathbb{Z}_1$	• $\boldsymbol{E} \parallel [010]$ and $\boldsymbol{B} \parallel [100]$
$Pmn'2_1'$ (31.126) $\mathbb{Z}_1$	• $\boldsymbol{E} \parallel [010]$ and $\boldsymbol{B} \parallel [001]$
$P2_1$ (4.7) $\mathbb{Z}_1$	• $\boldsymbol{E} \parallel [010]$ and $\boldsymbol{B} \parallel [010]$ and strain $\perp [010]$
$Pm'n'2_1$ (31.127) $\mathbb{Z}_1$	• $\boldsymbol{E} \parallel [010]$ and $\boldsymbol{B} \parallel [010]$
P_a2_1 (4.10) $\mathbb{Z}_1$	• $\boldsymbol{E} \parallel [010]$ and strain $\perp [010]$
P_amc2_1 (26.71) $\mathbb{Z}_1$	• $\boldsymbol{E} \parallel [010]$
$P2'$ (3.3) $\mathbb{Z}_1$	• $\boldsymbol{E} \parallel [001]$ and $\boldsymbol{B} \parallel [100]$ and strain $\perp [001]$<br>• $\boldsymbol{E} \parallel [001]$ and $\boldsymbol{B} \parallel [010]$ and strain $\perp [001]$<br>• $\boldsymbol{E} \parallel [001]$ and $\boldsymbol{B} \perp [001]$
$Pm'a2'$ (28.89) $\mathbb{Z}_1$	• $\boldsymbol{E} \parallel [001]$ and $\boldsymbol{B} \parallel [100]$
$Pn'c2'$ (30.113) $\mathbb{Z}_1$	• $\boldsymbol{E} \parallel [001]$ and $\boldsymbol{B} \parallel [010]$
$P2_1$ (4.7) $\mathbb{Z}_1$	• $\boldsymbol{E} \parallel [001]$ and $\boldsymbol{B} \parallel [001]$ and strain $\perp [001]$

Continued on next page

...continued

Subgroup & SI group	Perturbation
$Pm'n'2_1$ (31.127) $\mathbb{Z}_1$	• $\boldsymbol{E}$ ∥ [001] and $\boldsymbol{B}$ ∥ [001]
P_b2_1 (4.11) $\mathbb{Z}_1$	• $\boldsymbol{E}$ ∥ [001] and strain ⊥ [001]
P_cca2_1 (29.106) $\mathbb{Z}_1$	• $\boldsymbol{E}$ ∥ [001]
$P\bar{1}$ (2.4) $\mathbb{Z}_2 \times \mathbb{Z}_2 \times \mathbb{Z}_2 \times \mathbb{Z}_4$	• $\boldsymbol{B}$ ∥ [100] and strain in generic direction<br>• $\boldsymbol{B}$ ∥ [010] and strain in generic direction<br>• $\boldsymbol{B}$ ∥ [001] and strain in generic direction<br>• $\boldsymbol{B}$ ⊥ [100] and strain ⊥ [010]<br>• $\boldsymbol{B}$ ⊥ [100] and strain ⊥ [001]<br>• $\boldsymbol{B}$ ⊥ [100] and strain in generic direction<br>• $\boldsymbol{B}$ ⊥ [010] and strain ⊥ [100]<br>• $\boldsymbol{B}$ ⊥ [010] and strain ⊥ [001]<br>• $\boldsymbol{B}$ ⊥ [010] and strain in generic direction<br>• $\boldsymbol{B}$ ⊥ [001] and strain ⊥ [100]<br>• $\boldsymbol{B}$ ⊥ [001] and strain ⊥ [010]<br>• $\boldsymbol{B}$ ⊥ [001] and strain in generic direction<br>• $\boldsymbol{B}$ in generic direction
$P2/c$ (13.65) $\mathbb{Z}_2 \times \mathbb{Z}_2$	• $\boldsymbol{B}$ ∥ [100] and strain ⊥ [100]
$P2'/c'$ (13.69) $\mathbb{Z}_2 \times \mathbb{Z}_2 \times \mathbb{Z}_4$	• $\boldsymbol{B}$ ∥ [010] and strain ⊥ [100]<br>• $\boldsymbol{B}$ ∥ [001] and strain ⊥ [100]<br>• $\boldsymbol{B}$ ⊥ [100]
$P2_1'/m'$ (11.54) $\mathbb{Z}_2 \times \mathbb{Z}_2 \times \mathbb{Z}_4$	• $\boldsymbol{B}$ ∥ [100] and strain ⊥ [010]<br>• $\boldsymbol{B}$ ∥ [001] and strain ⊥ [010]<br>• $\boldsymbol{B}$ ⊥ [010]
$P2_1/c$ (14.75) $\mathbb{Z}_2$	• $\boldsymbol{B}$ ∥ [010] and strain ⊥ [010]
$P2'/m'$ (10.46) $\mathbb{Z}_2 \times \mathbb{Z}_2 \times \mathbb{Z}_2 \times \mathbb{Z}_4$	• $\boldsymbol{B}$ ∥ [100] and strain ⊥ [001]<br>• $\boldsymbol{B}$ ∥ [010] and strain ⊥ [001]<br>• $\boldsymbol{B}$ ⊥ [001]
$Pm'm'a$ (51.294) $\mathbb{Z}_2 \times \mathbb{Z}_2$	• $\boldsymbol{B}$ ∥ [100]
$Pm'n'a$ (53.326) $\mathbb{Z}_2$	• $\boldsymbol{B}$ ∥ [010]
$P2_1/m$ (11.50) $\mathbb{Z}_2$	• $\boldsymbol{B}$ ∥ [001] and strain ⊥ [001]
$Pmm'n'$ (59.410) $\mathbb{Z}_2$	• $\boldsymbol{B}$ ∥ [001]
$P_S\bar{1}$ (2.7) $\mathbb{Z}_2 \times \mathbb{Z}_2 \times \mathbb{Z}_4$	• strain in generic direction
P_a2/c (13.70) $\mathbb{Z}_2 \times \mathbb{Z}_2$	• strain ⊥ [100]
P_c2_1/c (14.82) $\mathbb{Z}_2$	• strain ⊥ [010]

Continued on next page

...continued

Subgroup & SI group	Perturbation
P_b2_1/m (11.56) $\mathbb{Z}_2$	• strain $\perp$ [001]

Table. S 499: Subgroups of P_Abcm (57.389) that can be realized by applying $\boldsymbol{E}/\boldsymbol{B}$ fields and/or uniaxial strains.

Subgroup & SI group	Perturbation
Pm (6.18) $\mathbb{Z}_1$	• $\boldsymbol{E}$ $\parallel$ [100] and $\boldsymbol{B}$ $\parallel$ [001] and strain $\perp$ [001]<br>• $\boldsymbol{E}$ $\parallel$ [010] and $\boldsymbol{B}$ $\parallel$ [001] and strain $\perp$ [001]<br>• $\boldsymbol{E}$ $\perp$ [001] and $\boldsymbol{B}$ $\parallel$ [001]
P_S1 (1.3) $\mathbb{Z}_1$	• $\boldsymbol{E}$ $\parallel$ [100] and strain in generic direction<br>• $\boldsymbol{E}$ $\parallel$ [010] and strain in generic direction<br>• $\boldsymbol{E}$ $\parallel$ [001] and strain in generic direction<br>• $\boldsymbol{E}$ $\perp$ [100] and strain $\perp$ [010]<br>• $\boldsymbol{E}$ $\perp$ [100] and strain $\perp$ [001]<br>• $\boldsymbol{E}$ $\perp$ [100] and strain in generic direction<br>• $\boldsymbol{E}$ $\perp$ [010] and strain $\perp$ [100]<br>• $\boldsymbol{E}$ $\perp$ [010] and strain $\perp$ [001]<br>• $\boldsymbol{E}$ $\perp$ [010] and strain in generic direction<br>• $\boldsymbol{E}$ $\perp$ [001] and strain $\perp$ [100]<br>• $\boldsymbol{E}$ $\perp$ [001] and strain $\perp$ [010]<br>• $\boldsymbol{E}$ $\perp$ [001] and strain in generic direction<br>• $\boldsymbol{E}$ in generic direction
Pc' (7.26) $\mathbb{Z}_1$	• $\boldsymbol{E}$ $\parallel$ [100] and $\boldsymbol{B}$ $\parallel$ [100] and strain $\perp$ [001]<br>• $\boldsymbol{E}$ $\parallel$ [100] and $\boldsymbol{B}$ $\parallel$ [010] and strain $\perp$ [001]<br>• $\boldsymbol{E}$ $\parallel$ [100] and $\boldsymbol{B}$ $\perp$ [001]<br>• $\boldsymbol{E}$ $\parallel$ [010] and $\boldsymbol{B}$ $\parallel$ [100] and strain $\perp$ [001]<br>• $\boldsymbol{E}$ $\parallel$ [010] and $\boldsymbol{B}$ $\parallel$ [010] and strain $\perp$ [001]<br>• $\boldsymbol{E}$ $\parallel$ [010] and $\boldsymbol{B}$ $\perp$ [001]<br>• $\boldsymbol{E}$ $\perp$ [001] and $\boldsymbol{B}$ $\parallel$ [100]<br>• $\boldsymbol{E}$ $\perp$ [001] and $\boldsymbol{B}$ $\parallel$ [010]<br>• $\boldsymbol{E}$ $\perp$ [001] and $\boldsymbol{B}$ $\perp$ [001]
P_Cm (6.23) $\mathbb{Z}_1$	• $\boldsymbol{E}$ $\parallel$ [100] and strain $\perp$ [001]<br>• $\boldsymbol{E}$ $\parallel$ [010] and strain $\perp$ [001]<br>• $\boldsymbol{E}$ $\perp$ [001]

Continued on next page

. . . continued

Subgroup & SI group	Perturbation
Pm' (6.20) $\mathbb{Z}_1$	• $\boldsymbol{E}$ || [100] and $\boldsymbol{B}$ || [100] and strain ⊥ [010]<br>• $\boldsymbol{E}$ || [100] and $\boldsymbol{B}$ || [001] and strain ⊥ [010]<br>• $\boldsymbol{E}$ || [100] and $\boldsymbol{B}$ ⊥ [010]<br>• $\boldsymbol{E}$ || [001] and $\boldsymbol{B}$ || [100] and strain ⊥ [010]<br>• $\boldsymbol{E}$ || [001] and $\boldsymbol{B}$ || [001] and strain ⊥ [010]<br>• $\boldsymbol{E}$ || [001] and $\boldsymbol{B}$ ⊥ [010]<br>• $\boldsymbol{E}$ ⊥ [010] and $\boldsymbol{B}$ || [100]<br>• $\boldsymbol{E}$ ⊥ [010] and $\boldsymbol{B}$ || [001]<br>• $\boldsymbol{E}$ ⊥ [010] and $\boldsymbol{B}$ ⊥ [010]
$P2'$ (3.3) $\mathbb{Z}_1$	• $\boldsymbol{E}$ || [100] and $\boldsymbol{B}$ || [010] and strain ⊥ [100]<br>• $\boldsymbol{E}$ || [100] and $\boldsymbol{B}$ || [001] and strain ⊥ [100]<br>• $\boldsymbol{E}$ || [100] and $\boldsymbol{B}$ ⊥ [100]
$Pm'm2'$ (25.59) $\mathbb{Z}_1$	• $\boldsymbol{E}$ || [100] and $\boldsymbol{B}$ || [001]
Pc (7.24) $\mathbb{Z}_1$	• $\boldsymbol{E}$ || [100] and $\boldsymbol{B}$ || [010] and strain ⊥ [010]<br>• $\boldsymbol{E}$ || [001] and $\boldsymbol{B}$ || [010] and strain ⊥ [010]<br>• $\boldsymbol{E}$ ⊥ [010] and $\boldsymbol{B}$ || [010]
P_Ac (7.31) $\mathbb{Z}_1$	• $\boldsymbol{E}$ || [100] and strain ⊥ [010]<br>• $\boldsymbol{E}$ || [001] and strain ⊥ [010]<br>• $\boldsymbol{E}$ ⊥ [010]
$Pb'a2'$ (32.137) $\mathbb{Z}_1$	• $\boldsymbol{E}$ || [100] and $\boldsymbol{B}$ || [010]
$P2$ (3.1) $\mathbb{Z}_2$	• $\boldsymbol{E}$ || [100] and $\boldsymbol{B}$ || [100] and strain ⊥ [100]
$Pm'a'2$ (28.91) $\mathbb{Z}_2$	• $\boldsymbol{E}$ || [100] and $\boldsymbol{B}$ || [100]
P_a2 (3.4) $\mathbb{Z}_2$	• $\boldsymbol{E}$ || [100] and strain ⊥ [100]
P_Cma2 (28.97) $\mathbb{Z}_1$	• $\boldsymbol{E}$ || [100]
Pc' (7.26) $\mathbb{Z}_1$	• $\boldsymbol{E}$ || [010] and $\boldsymbol{B}$ || [010] and strain ⊥ [100]<br>• $\boldsymbol{E}$ || [010] and $\boldsymbol{B}$ || [001] and strain ⊥ [100]<br>• $\boldsymbol{E}$ || [010] and $\boldsymbol{B}$ ⊥ [100]<br>• $\boldsymbol{E}$ || [001] and $\boldsymbol{B}$ || [010] and strain ⊥ [100]<br>• $\boldsymbol{E}$ || [001] and $\boldsymbol{B}$ || [001] and strain ⊥ [100]<br>• $\boldsymbol{E}$ || [001] and $\boldsymbol{B}$ ⊥ [100]<br>• $\boldsymbol{E}$ ⊥ [100] and $\boldsymbol{B}$ || [010]<br>• $\boldsymbol{E}$ ⊥ [100] and $\boldsymbol{B}$ || [001]<br>• $\boldsymbol{E}$ ⊥ [100] and $\boldsymbol{B}$ ⊥ [100]
$P2'$ (3.3) $\mathbb{Z}_1$	• $\boldsymbol{E}$ || [010] and $\boldsymbol{B}$ || [100] and strain ⊥ [010]<br>• $\boldsymbol{E}$ || [010] and $\boldsymbol{B}$ || [001] and strain ⊥ [010]<br>• $\boldsymbol{E}$ || [010] and $\boldsymbol{B}$ ⊥ [010]

Continued on next page

...continued

Subgroup & SI group	Perturbation
$Pma'2'$ (28.90) $\mathbb{Z}_1$	• $\boldsymbol{E}$ ∥ [010] and $\boldsymbol{B}$ ∥ [001]
Pc (7.24) $\mathbb{Z}_1$	• $\boldsymbol{E}$ ∥ [010] and $\boldsymbol{B}$ ∥ [100] and strain ⊥ [100]<br>• $\boldsymbol{E}$ ∥ [001] and $\boldsymbol{B}$ ∥ [100] and strain ⊥ [100]<br>• $\boldsymbol{E}$ ⊥ [100] and $\boldsymbol{B}$ ∥ [100]
P_ac (7.27) $\mathbb{Z}_1$	• $\boldsymbol{E}$ ∥ [010] and strain ⊥ [100]<br>• $\boldsymbol{E}$ ∥ [001] and strain ⊥ [100]<br>• $\boldsymbol{E}$ ⊥ [100]
$Pc'c2'$ (27.80) $\mathbb{Z}_1$	• $\boldsymbol{E}$ ∥ [010] and $\boldsymbol{B}$ ∥ [100]
$P2_1$ (4.7) $\mathbb{Z}_1$	• $\boldsymbol{E}$ ∥ [010] and $\boldsymbol{B}$ ∥ [010] and strain ⊥ [010]
$Pc'a'2_1$ (29.103) $\mathbb{Z}_1$	• $\boldsymbol{E}$ ∥ [010] and $\boldsymbol{B}$ ∥ [010]
P_C2_1 (4.12) $\mathbb{Z}_1$	• $\boldsymbol{E}$ ∥ [010] and strain ⊥ [010]
P_Bmc2_1 (26.75) $\mathbb{Z}_1$	• $\boldsymbol{E}$ ∥ [010]
$P2_1$ (4.7) $\mathbb{Z}_1$	• $\boldsymbol{E}$ ∥ [001] and $\boldsymbol{B}$ ∥ [001] and strain ⊥ [001]
$Pm'c'2_1$ (26.70) $\mathbb{Z}_1$	• $\boldsymbol{E}$ ∥ [001] and $\boldsymbol{B}$ ∥ [001]
$P\bar{1}$ (2.4) $\mathbb{Z}_2\times\mathbb{Z}_2\times\mathbb{Z}_2\times\mathbb{Z}_4$	• $\boldsymbol{B}$ ∥ [100] and strain in generic direction<br>• $\boldsymbol{B}$ ∥ [010] and strain in generic direction<br>• $\boldsymbol{B}$ ∥ [001] and strain in generic direction<br>• $\boldsymbol{B}$ ⊥ [100] and strain ⊥ [010]<br>• $\boldsymbol{B}$ ⊥ [100] and strain ⊥ [001]<br>• $\boldsymbol{B}$ ⊥ [100] and strain in generic direction<br>• $\boldsymbol{B}$ ⊥ [010] and strain ⊥ [100]<br>• $\boldsymbol{B}$ ⊥ [010] and strain ⊥ [001]<br>• $\boldsymbol{B}$ ⊥ [010] and strain in generic direction<br>• $\boldsymbol{B}$ ⊥ [001] and strain ⊥ [100]<br>• $\boldsymbol{B}$ ⊥ [001] and strain ⊥ [010]<br>• $\boldsymbol{B}$ ⊥ [001] and strain in generic direction<br>• $\boldsymbol{B}$ in generic direction
$P2'/c'$ (13.69) $\mathbb{Z}_2\times\mathbb{Z}_2\times\mathbb{Z}_4$	• $\boldsymbol{B}$ ∥ [010] and strain ⊥ [100]<br>• $\boldsymbol{B}$ ∥ [001] and strain ⊥ [100]<br>• $\boldsymbol{B}$ ⊥ [100]
$P2'/m'$ (10.46) $\mathbb{Z}_2\times\mathbb{Z}_2\times\mathbb{Z}_2\times\mathbb{Z}_4$	• $\boldsymbol{B}$ ∥ [100] and strain ⊥ [010]<br>• $\boldsymbol{B}$ ∥ [001] and strain ⊥ [010]<br>• $\boldsymbol{B}$ ⊥ [010]
$P2/c$ (13.65) $\mathbb{Z}_2\times\mathbb{Z}_2$	• $\boldsymbol{B}$ ∥ [100] and strain ⊥ [100]
$P2_1/c$ (14.75) $\mathbb{Z}_2$	• $\boldsymbol{B}$ ∥ [010] and strain ⊥ [010]

Continued on next page

...continued

Subgroup & SI group	Perturbation
$P2_1/m$ (11.50) $\mathbb{Z}_2$	• $\boldsymbol{B}$ ∥ [001] and strain ⊥ [001]
$Pmm'a'$ (51.295) $\mathbb{Z}_2$	• $\boldsymbol{B}$ ∥ [001]
$P2'$ (3.3) $\mathbb{Z}_1$	• $\boldsymbol{E}$ ∥ [001] and $\boldsymbol{B}$ ∥ [100] and strain ⊥ [001]<br>• $\boldsymbol{E}$ ∥ [001] and $\boldsymbol{B}$ ∥ [010] and strain ⊥ [001]<br>• $\boldsymbol{E}$ ∥ [001] and $\boldsymbol{B}$ ⊥ [001]
$Pc'c2'$ (27.80) $\mathbb{Z}_1$	• $\boldsymbol{E}$ ∥ [001] and $\boldsymbol{B}$ ∥ [010]
$Pm'a2'$ (28.89) $\mathbb{Z}_1$	• $\boldsymbol{E}$ ∥ [001] and $\boldsymbol{B}$ ∥ [100]
P_C2_1 (4.12) $\mathbb{Z}_1$	• $\boldsymbol{E}$ ∥ [001] and strain ⊥ [001]
P_Bca2_1 (29.108) $\mathbb{Z}_1$	• $\boldsymbol{E}$ ∥ [001]
$P2'/c'$ (13.69) $\mathbb{Z}_2 \times \mathbb{Z}_2 \times \mathbb{Z}_4$	• $\boldsymbol{B}$ ∥ [100] and strain ⊥ [001]<br>• $\boldsymbol{B}$ ∥ [010] and strain ⊥ [001]<br>• $\boldsymbol{B}$ ⊥ [001]
$Pc'cm'$ (49.270) $\mathbb{Z}_2 \times \mathbb{Z}_2$	• $\boldsymbol{B}$ ∥ [100]
$Pcc'a'$ (54.343) $\mathbb{Z}_2$	• $\boldsymbol{B}$ ∥ [010]
$P_S\bar{1}$ (2.7) $\mathbb{Z}_2 \times \mathbb{Z}_2 \times \mathbb{Z}_4$	• strain in generic direction
P_a2/c (13.70) $\mathbb{Z}_2 \times \mathbb{Z}_2$	• strain ⊥ [100]
P_A2_1/c (14.83) $\mathbb{Z}_2$	• strain ⊥ [010]
P_C2_1/m (11.57) $\mathbb{Z}_2$	• strain ⊥ [001]

Table. S 500: Subgroups of P_Bbcm (57.390) that can be realized by applying $\boldsymbol{E}/\boldsymbol{B}$ fields and/or uniaxial strains.

Subgroup & SI group	Perturbation
Pm (6.18) $\mathbb{Z}_1$	• $\boldsymbol{E}$ ∥ [100] and $\boldsymbol{B}$ ∥ [001] and strain ⊥ [001]<br>• $\boldsymbol{E}$ ∥ [010] and $\boldsymbol{B}$ ∥ [001] and strain ⊥ [001]<br>• $\boldsymbol{E}$ ⊥ [001] and $\boldsymbol{B}$ ∥ [001]
Pc (7.24) $\mathbb{Z}_1$	• $\boldsymbol{E}$ ∥ [100] and $\boldsymbol{B}$ ∥ [010] and strain ⊥ [010]<br>• $\boldsymbol{E}$ ∥ [001] and $\boldsymbol{B}$ ∥ [010] and strain ⊥ [010]<br>• $\boldsymbol{E}$ ⊥ [010] and $\boldsymbol{B}$ ∥ [010]
$P2$ (3.1) $\mathbb{Z}_2$	• $\boldsymbol{E}$ ∥ [100] and $\boldsymbol{B}$ ∥ [100] and strain ⊥ [100]

Continued on next page

...continued

Subgroup & SI group	Perturbation
P_S1 (1.3) $\mathbb{Z}_1$	• $\boldsymbol{E}$ ∥ [100] and strain in generic direction<br>• $\boldsymbol{E}$ ∥ [010] and strain in generic direction<br>• $\boldsymbol{E}$ ∥ [001] and strain in generic direction<br>• $\boldsymbol{E} \perp$ [100] and strain $\perp$ [010]<br>• $\boldsymbol{E} \perp$ [100] and strain $\perp$ [001]<br>• $\boldsymbol{E} \perp$ [100] and strain in generic direction<br>• $\boldsymbol{E} \perp$ [010] and strain $\perp$ [100]<br>• $\boldsymbol{E} \perp$ [010] and strain $\perp$ [001]<br>• $\boldsymbol{E} \perp$ [010] and strain in generic direction<br>• $\boldsymbol{E} \perp$ [001] and strain $\perp$ [100]<br>• $\boldsymbol{E} \perp$ [001] and strain $\perp$ [010]<br>• $\boldsymbol{E} \perp$ [001] and strain in generic direction<br>• $\boldsymbol{E}$ in generic direction
Pc' (7.26) $\mathbb{Z}_1$	• $\boldsymbol{E}$ ∥ [100] and $\boldsymbol{B}$ ∥ [100] and strain $\perp$ [001]<br>• $\boldsymbol{E}$ ∥ [100] and $\boldsymbol{B}$ ∥ [010] and strain $\perp$ [001]<br>• $\boldsymbol{E}$ ∥ [100] and $\boldsymbol{B} \perp$ [001]<br>• $\boldsymbol{E}$ ∥ [010] and $\boldsymbol{B}$ ∥ [100] and strain $\perp$ [001]<br>• $\boldsymbol{E}$ ∥ [010] and $\boldsymbol{B}$ ∥ [010] and strain $\perp$ [001]<br>• $\boldsymbol{E}$ ∥ [010] and $\boldsymbol{B} \perp$ [001]<br>• $\boldsymbol{E} \perp$ [001] and $\boldsymbol{B}$ ∥ [100]<br>• $\boldsymbol{E} \perp$ [001] and $\boldsymbol{B}$ ∥ [010]<br>• $\boldsymbol{E} \perp$ [001] and $\boldsymbol{B} \perp$ [001]
P_Cm (6.23) $\mathbb{Z}_1$	• $\boldsymbol{E}$ ∥ [100] and strain $\perp$ [001]<br>• $\boldsymbol{E}$ ∥ [010] and strain $\perp$ [001]<br>• $\boldsymbol{E} \perp$ [001]
Pc' (7.26) $\mathbb{Z}_1$	• $\boldsymbol{E}$ ∥ [100] and $\boldsymbol{B}$ ∥ [100] and strain $\perp$ [010]<br>• $\boldsymbol{E}$ ∥ [100] and $\boldsymbol{B}$ ∥ [001] and strain $\perp$ [010]<br>• $\boldsymbol{E}$ ∥ [100] and $\boldsymbol{B} \perp$ [010]<br>• $\boldsymbol{E}$ ∥ [001] and $\boldsymbol{B}$ ∥ [100] and strain $\perp$ [010]<br>• $\boldsymbol{E}$ ∥ [001] and $\boldsymbol{B}$ ∥ [001] and strain $\perp$ [010]<br>• $\boldsymbol{E}$ ∥ [001] and $\boldsymbol{B} \perp$ [010]<br>• $\boldsymbol{E} \perp$ [010] and $\boldsymbol{B}$ ∥ [100]<br>• $\boldsymbol{E} \perp$ [010] and $\boldsymbol{B}$ ∥ [001]<br>• $\boldsymbol{E} \perp$ [010] and $\boldsymbol{B} \perp$ [010]
P_ac (7.27) $\mathbb{Z}_1$	• $\boldsymbol{E}$ ∥ [100] and strain $\perp$ [010]<br>• $\boldsymbol{E}$ ∥ [001] and strain $\perp$ [010]<br>• $\boldsymbol{E} \perp$ [010]
$Pc'c'2$ (27.81) $\mathbb{Z}_2$	• $\boldsymbol{E}$ ∥ [100] and $\boldsymbol{B}$ ∥ [100]

Continued on next page

...continued

Subgroup & SI group	Perturbation
$P2_1'$ (4.9) $\mathbb{Z}_1$	• $\boldsymbol{E} \parallel [100]$ and $\boldsymbol{B} \parallel [010]$ and strain $\perp [100]$<br>• $\boldsymbol{E} \parallel [100]$ and $\boldsymbol{B} \parallel [001]$ and strain $\perp [100]$<br>• $\boldsymbol{E} \parallel [100]$ and $\boldsymbol{B} \perp [100]$
P_C2 (3.6) $\mathbb{Z}_1$	• $\boldsymbol{E} \parallel [100]$ and strain $\perp [100]$
$Pc'a2_1'$ (29.101) $\mathbb{Z}_1$	• $\boldsymbol{E} \parallel [100]$ and $\boldsymbol{B} \parallel [010]$
$Pmc'2_1'$ (26.69) $\mathbb{Z}_1$	• $\boldsymbol{E} \parallel [100]$ and $\boldsymbol{B} \parallel [001]$
P_Bma2 (28.96) $\mathbb{Z}_1$	• $\boldsymbol{E} \parallel [100]$
Pc (7.24) $\mathbb{Z}_1$	• $\boldsymbol{E} \parallel [010]$ and $\boldsymbol{B} \parallel [100]$ and strain $\perp [100]$<br>• $\boldsymbol{E} \parallel [001]$ and $\boldsymbol{B} \parallel [100]$ and strain $\perp [100]$<br>• $\boldsymbol{E} \perp [100]$ and $\boldsymbol{B} \parallel [100]$
$P2_1$ (4.7) $\mathbb{Z}_1$	• $\boldsymbol{E} \parallel [010]$ and $\boldsymbol{B} \parallel [010]$ and strain $\perp [010]$
$P2_1$ (4.7) $\mathbb{Z}_1$	• $\boldsymbol{E} \parallel [001]$ and $\boldsymbol{B} \parallel [001]$ and strain $\perp [001]$
$P\bar{1}$ (2.4) $\mathbb{Z}_2 \times \mathbb{Z}_2 \times \mathbb{Z}_2 \times \mathbb{Z}_4$	• $\boldsymbol{B} \parallel [100]$ and strain in generic direction<br>• $\boldsymbol{B} \parallel [010]$ and strain in generic direction<br>• $\boldsymbol{B} \parallel [001]$ and strain in generic direction<br>• $\boldsymbol{B} \perp [100]$ and strain $\perp [010]$<br>• $\boldsymbol{B} \perp [100]$ and strain $\perp [001]$<br>• $\boldsymbol{B} \perp [100]$ and strain in generic direction<br>• $\boldsymbol{B} \perp [010]$ and strain $\perp [100]$<br>• $\boldsymbol{B} \perp [010]$ and strain $\perp [001]$<br>• $\boldsymbol{B} \perp [010]$ and strain in generic direction<br>• $\boldsymbol{B} \perp [001]$ and strain $\perp [100]$<br>• $\boldsymbol{B} \perp [001]$ and strain $\perp [010]$<br>• $\boldsymbol{B} \perp [001]$ and strain in generic direction<br>• $\boldsymbol{B}$ in generic direction
$P2/c$ (13.65) $\mathbb{Z}_2 \times \mathbb{Z}_2$	• $\boldsymbol{B} \parallel [100]$ and strain $\perp [100]$
$P2_1/c$ (14.75) $\mathbb{Z}_2$	• $\boldsymbol{B} \parallel [010]$ and strain $\perp [010]$
$P2_1/m$ (11.50) $\mathbb{Z}_2$	• $\boldsymbol{B} \parallel [001]$ and strain $\perp [001]$

Continued on next page

...continued

Subgroup & SI group	Perturbation
Pc' (7.26) $\mathbb{Z}_1$	• $\boldsymbol{E}$ ∥ [010] and $\boldsymbol{B}$ ∥ [010] and strain ⊥ [100]<br>• $\boldsymbol{E}$ ∥ [010] and $\boldsymbol{B}$ ∥ [001] and strain ⊥ [100]<br>• $\boldsymbol{E}$ ∥ [010] and $\boldsymbol{B}$ ⊥ [100]<br>• $\boldsymbol{E}$ ∥ [001] and $\boldsymbol{B}$ ∥ [010] and strain ⊥ [100]<br>• $\boldsymbol{E}$ ∥ [001] and $\boldsymbol{B}$ ∥ [001] and strain ⊥ [100]<br>• $\boldsymbol{E}$ ∥ [001] and $\boldsymbol{B}$ ⊥ [100]<br>• $\boldsymbol{E}$ ⊥ [100] and $\boldsymbol{B}$ ∥ [010]<br>• $\boldsymbol{E}$ ⊥ [100] and $\boldsymbol{B}$ ∥ [001]<br>• $\boldsymbol{E}$ ⊥ [100] and $\boldsymbol{B}$ ⊥ [100]
P_Cc (7.30) $\mathbb{Z}_1$	• $\boldsymbol{E}$ ∥ [010] and strain ⊥ [100]<br>• $\boldsymbol{E}$ ∥ [001] and strain ⊥ [100]<br>• $\boldsymbol{E}$ ⊥ [100]
$Pn'a'2_1$ (33.148) $\mathbb{Z}_1$	• $\boldsymbol{E}$ ∥ [010] and $\boldsymbol{B}$ ∥ [010]
$Pn'a'2_1$ (33.148) $\mathbb{Z}_1$	• $\boldsymbol{E}$ ∥ [001] and $\boldsymbol{B}$ ∥ [001]
$P2_1'/c'$ (14.79) $\mathbb{Z}_2 \times \mathbb{Z}_4$	• $\boldsymbol{B}$ ∥ [010] and strain ⊥ [100]<br>• $\boldsymbol{B}$ ∥ [001] and strain ⊥ [100]<br>• $\boldsymbol{B}$ ⊥ [100]
$P2_1'$ (4.9) $\mathbb{Z}_1$	• $\boldsymbol{E}$ ∥ [010] and $\boldsymbol{B}$ ∥ [100] and strain ⊥ [010]<br>• $\boldsymbol{E}$ ∥ [010] and $\boldsymbol{B}$ ∥ [001] and strain ⊥ [010]<br>• $\boldsymbol{E}$ ∥ [010] and $\boldsymbol{B}$ ⊥ [010]
$Pca'2_1'$ (29.102) $\mathbb{Z}_1$	• $\boldsymbol{E}$ ∥ [010] and $\boldsymbol{B}$ ∥ [100]
P_a2_1 (4.10) $\mathbb{Z}_1$	• $\boldsymbol{E}$ ∥ [010] and strain ⊥ [010]
$P2_1'/c'$ (14.79) $\mathbb{Z}_2 \times \mathbb{Z}_4$	• $\boldsymbol{B}$ ∥ [100] and strain ⊥ [010]<br>• $\boldsymbol{B}$ ∥ [001] and strain ⊥ [010]<br>• $\boldsymbol{B}$ ⊥ [010]
$Pmn'2_1'$ (31.126) $\mathbb{Z}_1$	• $\boldsymbol{E}$ ∥ [010] and $\boldsymbol{B}$ ∥ [001]
P_Cmc2_1 (26.76) $\mathbb{Z}_1$	• $\boldsymbol{E}$ ∥ [010]
$Pn'ma'$ (62.448) $\mathbb{Z}_2$	• $\boldsymbol{B}$ ∥ [001]
$P2'$ (3.3) $\mathbb{Z}_1$	• $\boldsymbol{E}$ ∥ [001] and $\boldsymbol{B}$ ∥ [100] and strain ⊥ [001]<br>• $\boldsymbol{E}$ ∥ [001] and $\boldsymbol{B}$ ∥ [010] and strain ⊥ [001]<br>• $\boldsymbol{E}$ ∥ [001] and $\boldsymbol{B}$ ⊥ [001]
$Pb'a2'$ (32.137) $\mathbb{Z}_1$	• $\boldsymbol{E}$ ∥ [001] and $\boldsymbol{B}$ ∥ [100]
P_C2_1 (4.12) $\mathbb{Z}_1$	• $\boldsymbol{E}$ ∥ [001] and strain ⊥ [001]

Continued on next page

...continued

Subgroup & SI group	Perturbation
$P2'/c'$ (13.69) $\mathbb{Z}_2 \times \mathbb{Z}_2 \times \mathbb{Z}_4$	• $\boldsymbol{B} \parallel [100]$ and strain $\perp [001]$<br>• $\boldsymbol{B} \parallel [010]$ and strain $\perp [001]$<br>• $\boldsymbol{B} \perp [001]$
$Pn'c2'$ (30.113) $\mathbb{Z}_1$	• $\boldsymbol{E} \parallel [001]$ and $\boldsymbol{B} \parallel [010]$
P_Aca2_1 (29.107) $\mathbb{Z}_1$	• $\boldsymbol{E} \parallel [001]$
$Pbc'n'$ (60.423) $\mathbb{Z}_2$	• $\boldsymbol{B} \parallel [010]$
$Pc'c'a$ (54.342) $\mathbb{Z}_2 \times \mathbb{Z}_2$	• $\boldsymbol{B} \parallel [100]$
$P_S\bar{1}$ (2.7) $\mathbb{Z}_2 \times \mathbb{Z}_2 \times \mathbb{Z}_4$	• strain in generic direction
P_C2/c (13.74) $\mathbb{Z}_2$	• strain $\perp [100]$
P_a2_1/c (14.80) $\mathbb{Z}_2$	• strain $\perp [010]$
P_C2_1/m (11.57) $\mathbb{Z}_2$	• strain $\perp [001]$

Table. S 501: Subgroups of P_Cbcm (57.391) that can be realized by applying $\boldsymbol{E}/\boldsymbol{B}$ fields and/or uniaxial strains.

Subgroup & SI group	Perturbation
Pm (6.18) $\mathbb{Z}_1$	• $\boldsymbol{E} \parallel [100]$ and $\boldsymbol{B} \parallel [001]$ and strain $\perp [001]$<br>• $\boldsymbol{E} \parallel [010]$ and $\boldsymbol{B} \parallel [001]$ and strain $\perp [001]$<br>• $\boldsymbol{E} \perp [001]$ and $\boldsymbol{B} \parallel [001]$
Pc (7.24) $\mathbb{Z}_1$	• $\boldsymbol{E} \parallel [100]$ and $\boldsymbol{B} \parallel [010]$ and strain $\perp [010]$<br>• $\boldsymbol{E} \parallel [001]$ and $\boldsymbol{B} \parallel [010]$ and strain $\perp [010]$<br>• $\boldsymbol{E} \perp [010]$ and $\boldsymbol{B} \parallel [010]$
$P2$ (3.1) $\mathbb{Z}_2$	• $\boldsymbol{E} \parallel [100]$ and $\boldsymbol{B} \parallel [100]$ and strain $\perp [100]$

Continued on next page

. . . continued

Subgroup & SI group	Perturbation
P_S1 (1.3) $\mathbb{Z}_1$	• $\boldsymbol{E}$ ∥ [100] and strain in generic direction<br>• $\boldsymbol{E}$ ∥ [010] and strain in generic direction<br>• $\boldsymbol{E}$ ∥ [001] and strain in generic direction<br>• $\boldsymbol{E} \perp$ [100] and strain $\perp$ [010]<br>• $\boldsymbol{E} \perp$ [100] and strain $\perp$ [001]<br>• $\boldsymbol{E} \perp$ [100] and strain in generic direction<br>• $\boldsymbol{E} \perp$ [010] and strain $\perp$ [100]<br>• $\boldsymbol{E} \perp$ [010] and strain $\perp$ [001]<br>• $\boldsymbol{E} \perp$ [010] and strain in generic direction<br>• $\boldsymbol{E} \perp$ [001] and strain $\perp$ [100]<br>• $\boldsymbol{E} \perp$ [001] and strain $\perp$ [010]<br>• $\boldsymbol{E} \perp$ [001] and strain in generic direction<br>• $\boldsymbol{E}$ in generic direction
Pc' (7.26) $\mathbb{Z}_1$	• $\boldsymbol{E}$ ∥ [100] and $\boldsymbol{B}$ ∥ [100] and strain $\perp$ [001]<br>• $\boldsymbol{E}$ ∥ [100] and $\boldsymbol{B}$ ∥ [010] and strain $\perp$ [001]<br>• $\boldsymbol{E}$ ∥ [100] and $\boldsymbol{B} \perp$ [001]<br>• $\boldsymbol{E}$ ∥ [010] and $\boldsymbol{B}$ ∥ [100] and strain $\perp$ [001]<br>• $\boldsymbol{E}$ ∥ [010] and $\boldsymbol{B}$ ∥ [010] and strain $\perp$ [001]<br>• $\boldsymbol{E}$ ∥ [010] and $\boldsymbol{B} \perp$ [001]<br>• $\boldsymbol{E} \perp$ [001] and $\boldsymbol{B}$ ∥ [100]<br>• $\boldsymbol{E} \perp$ [001] and $\boldsymbol{B}$ ∥ [010]<br>• $\boldsymbol{E} \perp$ [001] and $\boldsymbol{B} \perp$ [001]
P_am (6.21) $\mathbb{Z}_1$	• $\boldsymbol{E}$ ∥ [100] and strain $\perp$ [001]<br>• $\boldsymbol{E}$ ∥ [010] and strain $\perp$ [001]<br>• $\boldsymbol{E} \perp$ [001]
Pc' (7.26) $\mathbb{Z}_1$	• $\boldsymbol{E}$ ∥ [100] and $\boldsymbol{B}$ ∥ [100] and strain $\perp$ [010]<br>• $\boldsymbol{E}$ ∥ [100] and $\boldsymbol{B}$ ∥ [001] and strain $\perp$ [010]<br>• $\boldsymbol{E}$ ∥ [100] and $\boldsymbol{B} \perp$ [010]<br>• $\boldsymbol{E}$ ∥ [001] and $\boldsymbol{B}$ ∥ [100] and strain $\perp$ [010]<br>• $\boldsymbol{E}$ ∥ [001] and $\boldsymbol{B}$ ∥ [001] and strain $\perp$ [010]<br>• $\boldsymbol{E}$ ∥ [001] and $\boldsymbol{B} \perp$ [010]<br>• $\boldsymbol{E} \perp$ [010] and $\boldsymbol{B}$ ∥ [100]<br>• $\boldsymbol{E} \perp$ [010] and $\boldsymbol{B}$ ∥ [001]<br>• $\boldsymbol{E} \perp$ [010] and $\boldsymbol{B} \perp$ [010]
P_Cc (7.30) $\mathbb{Z}_1$	• $\boldsymbol{E}$ ∥ [100] and strain $\perp$ [010]<br>• $\boldsymbol{E}$ ∥ [001] and strain $\perp$ [010]<br>• $\boldsymbol{E} \perp$ [010]
$Pn'n'2$ (34.159) $\mathbb{Z}_1$	• $\boldsymbol{E}$ ∥ [100] and $\boldsymbol{B}$ ∥ [100]

Continued on next page

. . . continued

Subgroup & SI group	Perturbation
$P2_1'$ (4.9) $\mathbb{Z}_1$	• $\boldsymbol{E} \parallel [100]$ and $\boldsymbol{B} \parallel [010]$ and strain $\perp [100]$<br>• $\boldsymbol{E} \parallel [100]$ and $\boldsymbol{B} \parallel [001]$ and strain $\perp [100]$<br>• $\boldsymbol{E} \parallel [100]$ and $\boldsymbol{B} \perp [100]$
P_C2 (3.6) $\mathbb{Z}_1$	• $\boldsymbol{E} \parallel [100]$ and strain $\perp [100]$
$Pn'a2_1'$ (33.146) $\mathbb{Z}_1$	• $\boldsymbol{E} \parallel [100]$ and $\boldsymbol{B} \parallel [010]$
$Pmn'2_1'$ (31.126) $\mathbb{Z}_1$	• $\boldsymbol{E} \parallel [100]$ and $\boldsymbol{B} \parallel [001]$
P_Ama2 (28.95) $\mathbb{Z}_1$	• $\boldsymbol{E} \parallel [100]$
Pc (7.24) $\mathbb{Z}_1$	• $\boldsymbol{E} \parallel [010]$ and $\boldsymbol{B} \parallel [100]$ and strain $\perp [100]$<br>• $\boldsymbol{E} \parallel [001]$ and $\boldsymbol{B} \parallel [100]$ and strain $\perp [100]$<br>• $\boldsymbol{E} \perp [100]$ and $\boldsymbol{B} \parallel [100]$
$P2_1$ (4.7) $\mathbb{Z}_1$	• $\boldsymbol{E} \parallel [010]$ and $\boldsymbol{B} \parallel [010]$ and strain $\perp [010]$
$P2_1$ (4.7) $\mathbb{Z}_1$	• $\boldsymbol{E} \parallel [001]$ and $\boldsymbol{B} \parallel [001]$ and strain $\perp [001]$
$P\bar{1}$ (2.4) $\mathbb{Z}_2 \times \mathbb{Z}_2 \times \mathbb{Z}_2 \times \mathbb{Z}_4$	• $\boldsymbol{B} \parallel [100]$ and strain in generic direction<br>• $\boldsymbol{B} \parallel [010]$ and strain in generic direction<br>• $\boldsymbol{B} \parallel [001]$ and strain in generic direction<br>• $\boldsymbol{B} \perp [100]$ and strain $\perp [010]$<br>• $\boldsymbol{B} \perp [100]$ and strain $\perp [001]$<br>• $\boldsymbol{B} \perp [100]$ and strain in generic direction<br>• $\boldsymbol{B} \perp [010]$ and strain $\perp [100]$<br>• $\boldsymbol{B} \perp [010]$ and strain $\perp [001]$<br>• $\boldsymbol{B} \perp [010]$ and strain in generic direction<br>• $\boldsymbol{B} \perp [001]$ and strain $\perp [100]$<br>• $\boldsymbol{B} \perp [001]$ and strain $\perp [010]$<br>• $\boldsymbol{B} \perp [001]$ and strain in generic direction<br>• $\boldsymbol{B}$ in generic direction
$P2/c$ (13.65) $\mathbb{Z}_2 \times \mathbb{Z}_2$	• $\boldsymbol{B} \parallel [100]$ and strain $\perp [100]$
$P2_1/c$ (14.75) $\mathbb{Z}_2$	• $\boldsymbol{B} \parallel [010]$ and strain $\perp [010]$
$P2_1/m$ (11.50) $\mathbb{Z}_2$	• $\boldsymbol{B} \parallel [001]$ and strain $\perp [001]$

Continued on next page

...continued

Subgroup & SI group	Perturbation
Pm' (6.20) $\mathbb{Z}_1$	• $\boldsymbol{E} \parallel [010]$ and $\boldsymbol{B} \parallel [010]$ and strain $\perp [100]$<br>• $\boldsymbol{E} \parallel [010]$ and $\boldsymbol{B} \parallel [001]$ and strain $\perp [100]$<br>• $\boldsymbol{E} \parallel [010]$ and $\boldsymbol{B} \perp [100]$<br>• $\boldsymbol{E} \parallel [001]$ and $\boldsymbol{B} \parallel [010]$ and strain $\perp [100]$<br>• $\boldsymbol{E} \parallel [001]$ and $\boldsymbol{B} \parallel [001]$ and strain $\perp [100]$<br>• $\boldsymbol{E} \parallel [001]$ and $\boldsymbol{B} \perp [100]$<br>• $\boldsymbol{E} \perp [100]$ and $\boldsymbol{B} \parallel [010]$<br>• $\boldsymbol{E} \perp [100]$ and $\boldsymbol{B} \parallel [001]$<br>• $\boldsymbol{E} \perp [100]$ and $\boldsymbol{B} \perp [100]$
P_Ac (7.31) $\mathbb{Z}_1$	• $\boldsymbol{E} \parallel [010]$ and strain $\perp [100]$<br>• $\boldsymbol{E} \parallel [001]$ and strain $\perp [100]$<br>• $\boldsymbol{E} \perp [100]$
$Pm'n'2_1$ (31.127) $\mathbb{Z}_1$	• $\boldsymbol{E} \parallel [010]$ and $\boldsymbol{B} \parallel [010]$
$Pm'n'2_1$ (31.127) $\mathbb{Z}_1$	• $\boldsymbol{E} \parallel [001]$ and $\boldsymbol{B} \parallel [001]$
$P2_1'/m'$ (11.54) $\mathbb{Z}_2 \times \mathbb{Z}_2 \times \mathbb{Z}_4$	• $\boldsymbol{B} \parallel [010]$ and strain $\perp [100]$<br>• $\boldsymbol{B} \parallel [001]$ and strain $\perp [100]$<br>• $\boldsymbol{B} \perp [100]$
$P2'$ (3.3) $\mathbb{Z}_1$	• $\boldsymbol{E} \parallel [010]$ and $\boldsymbol{B} \parallel [100]$ and strain $\perp [010]$<br>• $\boldsymbol{E} \parallel [010]$ and $\boldsymbol{B} \parallel [001]$ and strain $\perp [010]$<br>• $\boldsymbol{E} \parallel [010]$ and $\boldsymbol{B} \perp [010]$
$Pn'c2'$ (30.113) $\mathbb{Z}_1$	• $\boldsymbol{E} \parallel [010]$ and $\boldsymbol{B} \parallel [100]$
P_C2_1 (4.12) $\mathbb{Z}_1$	• $\boldsymbol{E} \parallel [010]$ and strain $\perp [010]$
$P2'/c'$ (13.69) $\mathbb{Z}_2 \times \mathbb{Z}_2 \times \mathbb{Z}_4$	• $\boldsymbol{B} \parallel [100]$ and strain $\perp [010]$<br>• $\boldsymbol{B} \parallel [001]$ and strain $\perp [010]$<br>• $\boldsymbol{B} \perp [010]$
$Pm'm2'$ (25.59) $\mathbb{Z}_1$	• $\boldsymbol{E} \parallel [010]$ and $\boldsymbol{B} \parallel [001]$
P_Amc2_1 (26.74) $\mathbb{Z}_1$	• $\boldsymbol{E} \parallel [010]$
$Pmm'n'$ (59.410) $\mathbb{Z}_2$	• $\boldsymbol{B} \parallel [001]$
$P2_1'$ (4.9) $\mathbb{Z}_1$	• $\boldsymbol{E} \parallel [001]$ and $\boldsymbol{B} \parallel [100]$ and strain $\perp [001]$<br>• $\boldsymbol{E} \parallel [001]$ and $\boldsymbol{B} \parallel [010]$ and strain $\perp [001]$<br>• $\boldsymbol{E} \parallel [001]$ and $\boldsymbol{B} \perp [001]$
$Pn'a2_1'$ (33.146) $\mathbb{Z}_1$	• $\boldsymbol{E} \parallel [001]$ and $\boldsymbol{B} \parallel [100]$
P_a2_1 (4.10) $\mathbb{Z}_1$	• $\boldsymbol{E} \parallel [001]$ and strain $\perp [001]$

Continued on next page

...continued

Subgroup & SI group	Perturbation
$P2_1'/c'$ (14.79) $\mathbb{Z}_2 \times \mathbb{Z}_4$	• $\boldsymbol{B}$ ∥ [100] and strain ⊥ [001]<br>• $\boldsymbol{B}$ ∥ [010] and strain ⊥ [001]<br>• $\boldsymbol{B}$ ⊥ [001]
$Pm'c2_1'$ (26.68) $\mathbb{Z}_1$	• $\boldsymbol{E}$ ∥ [001] and $\boldsymbol{B}$ ∥ [010]
P_Cca2_1 (29.109) $\mathbb{Z}_1$	• $\boldsymbol{E}$ ∥ [001]
$Pn'm'a$ (62.446) $\mathbb{Z}_2$	• $\boldsymbol{B}$ ∥ [010]
$Pn'n'a$ (52.310) $\mathbb{Z}_2$	• $\boldsymbol{B}$ ∥ [100]
$P_S\bar{1}$ (2.7) $\mathbb{Z}_2 \times \mathbb{Z}_2 \times \mathbb{Z}_4$	• strain in generic direction
P_A2/c (13.73) $\mathbb{Z}_2$	• strain ⊥ [100]
P_C2_1/c (14.84) $\mathbb{Z}_2$	• strain ⊥ [010]
P_a2_1/m (11.55) $\mathbb{Z}_2$	• strain ⊥ [001]

Table. S 502: Subgroups of P_Ibcm (57.392) that can be realized by applying $\boldsymbol{E}/\boldsymbol{B}$ fields and/or uniaxial strains.

Subgroup & SI group	Perturbation
Pm (6.18) $\mathbb{Z}_1$	• $\boldsymbol{E}$ ∥ [100] and $\boldsymbol{B}$ ∥ [001] and strain ⊥ [001]<br>• $\boldsymbol{E}$ ∥ [010] and $\boldsymbol{B}$ ∥ [001] and strain ⊥ [001]<br>• $\boldsymbol{E}$ ⊥ [001] and $\boldsymbol{B}$ ∥ [001]
Pc (7.24) $\mathbb{Z}_1$	• $\boldsymbol{E}$ ∥ [100] and $\boldsymbol{B}$ ∥ [010] and strain ⊥ [010]<br>• $\boldsymbol{E}$ ∥ [001] and $\boldsymbol{B}$ ∥ [010] and strain ⊥ [010]<br>• $\boldsymbol{E}$ ⊥ [010] and $\boldsymbol{B}$ ∥ [010]
$P2$ (3.1) $\mathbb{Z}_2$	• $\boldsymbol{E}$ ∥ [100] and $\boldsymbol{B}$ ∥ [100] and strain ⊥ [100]

Continued on next page

...continued

Subgroup & SI group	Perturbation
P_S1 (1.3) $\mathbb{Z}_1$	• $\boldsymbol{E}$ ∥ [100] and strain in generic direction<br>• $\boldsymbol{E}$ ∥ [010] and strain in generic direction<br>• $\boldsymbol{E}$ ∥ [001] and strain in generic direction<br>• $\boldsymbol{E} \perp$ [100] and strain $\perp$ [010]<br>• $\boldsymbol{E} \perp$ [100] and strain $\perp$ [001]<br>• $\boldsymbol{E} \perp$ [100] and strain in generic direction<br>• $\boldsymbol{E} \perp$ [010] and strain $\perp$ [100]<br>• $\boldsymbol{E} \perp$ [010] and strain $\perp$ [001]<br>• $\boldsymbol{E} \perp$ [010] and strain in generic direction<br>• $\boldsymbol{E} \perp$ [001] and strain $\perp$ [100]<br>• $\boldsymbol{E} \perp$ [001] and strain $\perp$ [010]<br>• $\boldsymbol{E} \perp$ [001] and strain in generic direction<br>• $\boldsymbol{E}$ in generic direction
Pc' (7.26) $\mathbb{Z}_1$	• $\boldsymbol{E}$ ∥ [100] and $\boldsymbol{B}$ ∥ [100] and strain $\perp$ [001]<br>• $\boldsymbol{E}$ ∥ [100] and $\boldsymbol{B}$ ∥ [010] and strain $\perp$ [001]<br>• $\boldsymbol{E}$ ∥ [100] and $\boldsymbol{B} \perp$ [001]<br>• $\boldsymbol{E}$ ∥ [010] and $\boldsymbol{B}$ ∥ [100] and strain $\perp$ [001]<br>• $\boldsymbol{E}$ ∥ [010] and $\boldsymbol{B}$ ∥ [010] and strain $\perp$ [001]<br>• $\boldsymbol{E}$ ∥ [010] and $\boldsymbol{B} \perp$ [001]<br>• $\boldsymbol{E} \perp$ [001] and $\boldsymbol{B}$ ∥ [100]<br>• $\boldsymbol{E} \perp$ [001] and $\boldsymbol{B}$ ∥ [010]<br>• $\boldsymbol{E} \perp$ [001] and $\boldsymbol{B} \perp$ [001]
P_Cm (6.23) $\mathbb{Z}_1$	• $\boldsymbol{E}$ ∥ [100] and strain $\perp$ [001]<br>• $\boldsymbol{E}$ ∥ [010] and strain $\perp$ [001]<br>• $\boldsymbol{E} \perp$ [001]
Pc' (7.26) $\mathbb{Z}_1$	• $\boldsymbol{E}$ ∥ [100] and $\boldsymbol{B}$ ∥ [100] and strain $\perp$ [010]<br>• $\boldsymbol{E}$ ∥ [100] and $\boldsymbol{B}$ ∥ [001] and strain $\perp$ [010]<br>• $\boldsymbol{E}$ ∥ [100] and $\boldsymbol{B} \perp$ [010]<br>• $\boldsymbol{E}$ ∥ [001] and $\boldsymbol{B}$ ∥ [100] and strain $\perp$ [010]<br>• $\boldsymbol{E}$ ∥ [001] and $\boldsymbol{B}$ ∥ [001] and strain $\perp$ [010]<br>• $\boldsymbol{E}$ ∥ [001] and $\boldsymbol{B} \perp$ [010]<br>• $\boldsymbol{E} \perp$ [010] and $\boldsymbol{B}$ ∥ [100]<br>• $\boldsymbol{E} \perp$ [010] and $\boldsymbol{B}$ ∥ [001]<br>• $\boldsymbol{E} \perp$ [010] and $\boldsymbol{B} \perp$ [010]
P_Cc (7.30) $\mathbb{Z}_1$	• $\boldsymbol{E}$ ∥ [100] and strain $\perp$ [010]<br>• $\boldsymbol{E}$ ∥ [001] and strain $\perp$ [010]<br>• $\boldsymbol{E} \perp$ [010]
$Pn'c'2$ (30.115) $\mathbb{Z}_1$	• $\boldsymbol{E}$ ∥ [100] and $\boldsymbol{B}$ ∥ [100]

Continued on next page

...continued

Subgroup & SI group	Perturbation
$P2_1'$ (4.9) $\mathbb{Z}_1$	• $\boldsymbol{E} \parallel [100]$ and $\boldsymbol{B} \parallel [010]$ and strain $\perp [100]$<br>• $\boldsymbol{E} \parallel [100]$ and $\boldsymbol{B} \parallel [001]$ and strain $\perp [100]$<br>• $\boldsymbol{E} \parallel [100]$ and $\boldsymbol{B} \perp [100]$
P_C2 (3.6) $\mathbb{Z}_1$	• $\boldsymbol{E} \parallel [100]$ and strain $\perp [100]$
$Pn'a2_1'$ (33.146) $\mathbb{Z}_1$	• $\boldsymbol{E} \parallel [100]$ and $\boldsymbol{B} \parallel [010]$
$Pmc'2_1'$ (26.69) $\mathbb{Z}_1$	• $\boldsymbol{E} \parallel [100]$ and $\boldsymbol{B} \parallel [001]$
P_Ima2 (28.98) $\mathbb{Z}_1$	• $\boldsymbol{E} \parallel [100]$
Pc (7.24) $\mathbb{Z}_1$	• $\boldsymbol{E} \parallel [010]$ and $\boldsymbol{B} \parallel [100]$ and strain $\perp [100]$<br>• $\boldsymbol{E} \parallel [001]$ and $\boldsymbol{B} \parallel [100]$ and strain $\perp [100]$<br>• $\boldsymbol{E} \perp [100]$ and $\boldsymbol{B} \parallel [100]$
$P2_1$ (4.7) $\mathbb{Z}_1$	• $\boldsymbol{E} \parallel [010]$ and $\boldsymbol{B} \parallel [010]$ and strain $\perp [010]$
$P2_1$ (4.7) $\mathbb{Z}_1$	• $\boldsymbol{E} \parallel [001]$ and $\boldsymbol{B} \parallel [001]$ and strain $\perp [001]$
$P\bar{1}$ (2.4) $\mathbb{Z}_2 \times \mathbb{Z}_2 \times \mathbb{Z}_2 \times \mathbb{Z}_4$	• $\boldsymbol{B} \parallel [100]$ and strain in generic direction<br>• $\boldsymbol{B} \parallel [010]$ and strain in generic direction<br>• $\boldsymbol{B} \parallel [001]$ and strain in generic direction<br>• $\boldsymbol{B} \perp [100]$ and strain $\perp [010]$<br>• $\boldsymbol{B} \perp [100]$ and strain $\perp [001]$<br>• $\boldsymbol{B} \perp [100]$ and strain in generic direction<br>• $\boldsymbol{B} \perp [010]$ and strain $\perp [100]$<br>• $\boldsymbol{B} \perp [010]$ and strain $\perp [001]$<br>• $\boldsymbol{B} \perp [010]$ and strain in generic direction<br>• $\boldsymbol{B} \perp [001]$ and strain $\perp [100]$<br>• $\boldsymbol{B} \perp [001]$ and strain $\perp [010]$<br>• $\boldsymbol{B} \perp [001]$ and strain in generic direction<br>• $\boldsymbol{B}$ in generic direction
$P2/c$ (13.65) $\mathbb{Z}_2 \times \mathbb{Z}_2$	• $\boldsymbol{B} \parallel [100]$ and strain $\perp [100]$
$P2_1/c$ (14.75) $\mathbb{Z}_2$	• $\boldsymbol{B} \parallel [010]$ and strain $\perp [010]$
$P2_1/m$ (11.50) $\mathbb{Z}_2$	• $\boldsymbol{B} \parallel [001]$ and strain $\perp [001]$

Continued on next page

...continued

Subgroup & SI group	Perturbation
Pc' (7.26) $\mathbb{Z}_1$	• $\boldsymbol{E}$ ∥ [010] and $\boldsymbol{B}$ ∥ [010] and strain ⊥ [100]<br>• $\boldsymbol{E}$ ∥ [010] and $\boldsymbol{B}$ ∥ [001] and strain ⊥ [100]<br>• $\boldsymbol{E}$ ∥ [010] and $\boldsymbol{B}$ ⊥ [100]<br>• $\boldsymbol{E}$ ∥ [001] and $\boldsymbol{B}$ ∥ [010] and strain ⊥ [100]<br>• $\boldsymbol{E}$ ∥ [001] and $\boldsymbol{B}$ ∥ [001] and strain ⊥ [100]<br>• $\boldsymbol{E}$ ∥ [001] and $\boldsymbol{B}$ ⊥ [100]<br>• $\boldsymbol{E}$ ⊥ [100] and $\boldsymbol{B}$ ∥ [010]<br>• $\boldsymbol{E}$ ⊥ [100] and $\boldsymbol{B}$ ∥ [001]<br>• $\boldsymbol{E}$ ⊥ [100] and $\boldsymbol{B}$ ⊥ [100]
P_Cc (7.30) $\mathbb{Z}_1$	• $\boldsymbol{E}$ ∥ [010] and strain ⊥ [100]<br>• $\boldsymbol{E}$ ∥ [001] and strain ⊥ [100]<br>• $\boldsymbol{E}$ ⊥ [100]
$Pn'a'2_1$ (33.148) $\mathbb{Z}_1$	• $\boldsymbol{E}$ ∥ [010] and $\boldsymbol{B}$ ∥ [010]
$Pc'a'2_1$ (29.103) $\mathbb{Z}_1$	• $\boldsymbol{E}$ ∥ [001] and $\boldsymbol{B}$ ∥ [001]
$P2_1'/c'$ (14.79) $\mathbb{Z}_2 \times \mathbb{Z}_4$	• $\boldsymbol{B}$ ∥ [010] and strain ⊥ [100]<br>• $\boldsymbol{B}$ ∥ [001] and strain ⊥ [100]<br>• $\boldsymbol{B}$ ⊥ [100]
$P2'$ (3.3) $\mathbb{Z}_1$	• $\boldsymbol{E}$ ∥ [010] and $\boldsymbol{B}$ ∥ [100] and strain ⊥ [010]<br>• $\boldsymbol{E}$ ∥ [010] and $\boldsymbol{B}$ ∥ [001] and strain ⊥ [010]<br>• $\boldsymbol{E}$ ∥ [010] and $\boldsymbol{B}$ ⊥ [010]
$Pn'c2'$ (30.113) $\mathbb{Z}_1$	• $\boldsymbol{E}$ ∥ [010] and $\boldsymbol{B}$ ∥ [100]
P_C2_1 (4.12) $\mathbb{Z}_1$	• $\boldsymbol{E}$ ∥ [010] and strain ⊥ [010]
$P2'/c'$ (13.69) $\mathbb{Z}_2 \times \mathbb{Z}_2 \times \mathbb{Z}_4$	• $\boldsymbol{B}$ ∥ [100] and strain ⊥ [010]<br>• $\boldsymbol{B}$ ∥ [001] and strain ⊥ [010]<br>• $\boldsymbol{B}$ ⊥ [010]
$Pma'2'$ (28.90) $\mathbb{Z}_1$	• $\boldsymbol{E}$ ∥ [010] and $\boldsymbol{B}$ ∥ [001]
P_Imc2_1 (26.77) $\mathbb{Z}_1$	• $\boldsymbol{E}$ ∥ [010]
$Pb'c'm$ (57.382) $\mathbb{Z}_2$	• $\boldsymbol{B}$ ∥ [001]
$P2'$ (3.3) $\mathbb{Z}_1$	• $\boldsymbol{E}$ ∥ [001] and $\boldsymbol{B}$ ∥ [100] and strain ⊥ [001]<br>• $\boldsymbol{E}$ ∥ [001] and $\boldsymbol{B}$ ∥ [010] and strain ⊥ [001]<br>• $\boldsymbol{E}$ ∥ [001] and $\boldsymbol{B}$ ⊥ [001]
$Pb'a2'$ (32.137) $\mathbb{Z}_1$	• $\boldsymbol{E}$ ∥ [001] and $\boldsymbol{B}$ ∥ [100]
P_C2_1 (4.12) $\mathbb{Z}_1$	• $\boldsymbol{E}$ ∥ [001] and strain ⊥ [001]

Continued on next page

. . . continued

Subgroup & SI group	Perturbation
$P2'/c'$ (13.69) $\mathbb{Z}_2 \times \mathbb{Z}_2 \times \mathbb{Z}_4$	• $\boldsymbol{B} \parallel [100]$ and strain $\perp [001]$<br>• $\boldsymbol{B} \parallel [010]$ and strain $\perp [001]$<br>• $\boldsymbol{B} \perp [001]$
$Pc'c2'$ (27.80) $\mathbb{Z}_1$	• $\boldsymbol{E} \parallel [001]$ and $\boldsymbol{B} \parallel [010]$
P_Ica2_1 (29.110) $\mathbb{Z}_1$	• $\boldsymbol{E} \parallel [001]$
$Pc'cn'$ (56.370) $\mathbb{Z}_2$	• $\boldsymbol{B} \parallel [010]$
$Pb'an'$ (50.282) $\mathbb{Z}_2$	• $\boldsymbol{B} \parallel [100]$
$P_S\bar{1}$ (2.7) $\mathbb{Z}_2 \times \mathbb{Z}_2 \times \mathbb{Z}_4$	• strain in generic direction
P_C2/c (13.74) $\mathbb{Z}_2$	• strain $\perp [100]$
P_C2_1/c (14.84) $\mathbb{Z}_2$	• strain $\perp [010]$
P_C2_1/m (11.57) $\mathbb{Z}_2$	• strain $\perp [001]$

Table. S 503: Subgroups of $Pnnm$ (58.393) that can be realized by applying $\boldsymbol{E}/\boldsymbol{B}$ fields and/or uniaxial strains.

Subgroup & SI group	Perturbation
Pm (6.18) $\mathbb{Z}_1$	• $\boldsymbol{E} \parallel [100]$ and strain $\perp [001]$<br>• $\boldsymbol{E} \parallel [100]$ and $\boldsymbol{B} \parallel [001]$<br>• $\boldsymbol{E} \parallel [010]$ and strain $\perp [001]$<br>• $\boldsymbol{E} \parallel [010]$ and $\boldsymbol{B} \parallel [001]$<br>• $\boldsymbol{E} \perp [001]$
Pc (7.24) $\mathbb{Z}_1$	• $\boldsymbol{E} \parallel [100]$ and strain $\perp [010]$<br>• $\boldsymbol{E} \parallel [100]$ and $\boldsymbol{B} \parallel [010]$<br>• $\boldsymbol{E} \parallel [001]$ and strain $\perp [010]$<br>• $\boldsymbol{E} \parallel [001]$ and $\boldsymbol{B} \parallel [010]$<br>• $\boldsymbol{E} \perp [010]$
$P2_1$ (4.7) $\mathbb{Z}_1$	• $\boldsymbol{E} \parallel [100]$ and strain $\perp [100]$<br>• $\boldsymbol{E} \parallel [100]$ and $\boldsymbol{B} \parallel [100]$
$Pmn2_1$ (31.123) $\mathbb{Z}_1$	• $\boldsymbol{E} \parallel [100]$
$P2$ (3.1) $\mathbb{Z}_2$	• $\boldsymbol{E} \parallel [001]$ and strain $\perp [001]$<br>• $\boldsymbol{E} \parallel [001]$ and $\boldsymbol{B} \parallel [001]$

Continued on next page

…continued

Subgroup & SI group	Perturbation
$P\bar{1}$ (2.4) $\mathbb{Z}_2 \times \mathbb{Z}_2 \times \mathbb{Z}_2 \times \mathbb{Z}_4$	• strain in generic direction<br>• $\boldsymbol{B} \parallel [100]$ and strain $\perp [010]$<br>• $\boldsymbol{B} \parallel [100]$ and strain $\perp [001]$<br>• $\boldsymbol{B} \parallel [010]$ and strain $\perp [100]$<br>• $\boldsymbol{B} \parallel [010]$ and strain $\perp [001]$<br>• $\boldsymbol{B} \parallel [001]$ and strain $\perp [100]$<br>• $\boldsymbol{B} \parallel [001]$ and strain $\perp [010]$<br>• $\boldsymbol{B}$ in generic direction
$P2/m$ (10.42) $\mathbb{Z}_2 \times \mathbb{Z}_2 \times \mathbb{Z}_2$	• strain $\perp [001]$<br>• $\boldsymbol{B} \parallel [001]$
Pc (7.24) $\mathbb{Z}_1$	• $\boldsymbol{E} \parallel [010]$ and strain $\perp [100]$<br>• $\boldsymbol{E} \parallel [010]$ and $\boldsymbol{B} \parallel [100]$<br>• $\boldsymbol{E} \parallel [001]$ and strain $\perp [100]$<br>• $\boldsymbol{E} \parallel [001]$ and $\boldsymbol{B} \parallel [100]$<br>• $\boldsymbol{E} \perp [100]$
$Pnn2$ (34.156) $\mathbb{Z}_1$	• $\boldsymbol{E} \parallel [001]$
$P2_1/c$ (14.75) $\mathbb{Z}_2$	• strain $\perp [100]$<br>• $\boldsymbol{B} \parallel [100]$
$P2_1$ (4.7) $\mathbb{Z}_1$	• $\boldsymbol{E} \parallel [010]$ and strain $\perp [010]$<br>• $\boldsymbol{E} \parallel [010]$ and $\boldsymbol{B} \parallel [010]$
$P2_1/c$ (14.75) $\mathbb{Z}_2$	• strain $\perp [010]$<br>• $\boldsymbol{B} \parallel [010]$
$Pmn2_1$ (31.123) $\mathbb{Z}_1$	• $\boldsymbol{E} \parallel [010]$

Table. S 504: Subgroups of $Pnnm1'$ (58.394) that can be realized by applying $\boldsymbol{E}/\boldsymbol{B}$ fields and/or uniaxial strains.

Subgroup & SI group	Perturbation

Continued on next page

...continued

Subgroup & SI group	Perturbation
$P11'$ (1.2) $\mathbb{Z}_1$	• $\boldsymbol{E} \parallel [100]$ and strain in generic direction<br>• $\boldsymbol{E} \parallel [010]$ and strain in generic direction<br>• $\boldsymbol{E} \parallel [001]$ and strain in generic direction<br>• $\boldsymbol{E} \perp [100]$ and strain $\perp [010]$<br>• $\boldsymbol{E} \perp [100]$ and strain $\perp [001]$<br>• $\boldsymbol{E} \perp [100]$ and strain in generic direction<br>• $\boldsymbol{E} \perp [010]$ and strain $\perp [100]$<br>• $\boldsymbol{E} \perp [010]$ and strain $\perp [001]$<br>• $\boldsymbol{E} \perp [010]$ and strain in generic direction<br>• $\boldsymbol{E} \perp [001]$ and strain $\perp [100]$<br>• $\boldsymbol{E} \perp [001]$ and strain $\perp [010]$<br>• $\boldsymbol{E} \perp [001]$ and strain in generic direction<br>• $\boldsymbol{E}$ in generic direction
Pm' (6.20) $\mathbb{Z}_1$	• $\boldsymbol{E} \parallel [100]$ and $\boldsymbol{B} \parallel [100]$ and strain $\perp [001]$<br>• $\boldsymbol{E} \parallel [100]$ and $\boldsymbol{B} \parallel [010]$ and strain $\perp [001]$<br>• $\boldsymbol{E} \parallel [100]$ and $\boldsymbol{B} \perp [001]$<br>• $\boldsymbol{E} \parallel [010]$ and $\boldsymbol{B} \parallel [100]$ and strain $\perp [001]$<br>• $\boldsymbol{E} \parallel [010]$ and $\boldsymbol{B} \parallel [010]$ and strain $\perp [001]$<br>• $\boldsymbol{E} \parallel [010]$ and $\boldsymbol{B} \perp [001]$<br>• $\boldsymbol{E} \perp [001]$ and $\boldsymbol{B} \parallel [100]$<br>• $\boldsymbol{E} \perp [001]$ and $\boldsymbol{B} \parallel [010]$<br>• $\boldsymbol{E} \perp [001]$ and $\boldsymbol{B} \perp [001]$
Pm (6.18) $\mathbb{Z}_1$	• $\boldsymbol{E} \parallel [100]$ and $\boldsymbol{B} \parallel [001]$ and strain $\perp [001]$<br>• $\boldsymbol{E} \parallel [010]$ and $\boldsymbol{B} \parallel [001]$ and strain $\perp [001]$<br>• $\boldsymbol{E} \perp [001]$ and $\boldsymbol{B} \parallel [001]$
$Pm1'$ (6.19) $\mathbb{Z}_1$	• $\boldsymbol{E} \parallel [100]$ and strain $\perp [001]$<br>• $\boldsymbol{E} \parallel [010]$ and strain $\perp [001]$<br>• $\boldsymbol{E} \perp [001]$
Pc' (7.26) $\mathbb{Z}_1$	• $\boldsymbol{E} \parallel [100]$ and $\boldsymbol{B} \parallel [100]$ and strain $\perp [010]$<br>• $\boldsymbol{E} \parallel [100]$ and $\boldsymbol{B} \parallel [001]$ and strain $\perp [010]$<br>• $\boldsymbol{E} \parallel [100]$ and $\boldsymbol{B} \perp [010]$<br>• $\boldsymbol{E} \parallel [001]$ and $\boldsymbol{B} \parallel [100]$ and strain $\perp [010]$<br>• $\boldsymbol{E} \parallel [001]$ and $\boldsymbol{B} \parallel [001]$ and strain $\perp [010]$<br>• $\boldsymbol{E} \parallel [001]$ and $\boldsymbol{B} \perp [010]$<br>• $\boldsymbol{E} \perp [010]$ and $\boldsymbol{B} \parallel [100]$<br>• $\boldsymbol{E} \perp [010]$ and $\boldsymbol{B} \parallel [001]$<br>• $\boldsymbol{E} \perp [010]$ and $\boldsymbol{B} \perp [010]$

Continued on next page

...continued

Subgroup & SI group	Perturbation
Pc (7.24) $\mathbb{Z}_1$	• $\boldsymbol{E} \parallel [100]$ and $\boldsymbol{B} \parallel [010]$ and strain $\perp [010]$<br>• $\boldsymbol{E} \parallel [001]$ and $\boldsymbol{B} \parallel [010]$ and strain $\perp [010]$<br>• $\boldsymbol{E} \perp [010]$ and $\boldsymbol{B} \parallel [010]$
$Pc1'$ (7.25) $\mathbb{Z}_1$	• $\boldsymbol{E} \parallel [100]$ and strain $\perp [010]$<br>• $\boldsymbol{E} \parallel [001]$ and strain $\perp [010]$<br>• $\boldsymbol{E} \perp [010]$
$P2_1'$ (4.9) $\mathbb{Z}_1$	• $\boldsymbol{E} \parallel [100]$ and $\boldsymbol{B} \parallel [010]$ and strain $\perp [100]$<br>• $\boldsymbol{E} \parallel [100]$ and $\boldsymbol{B} \parallel [001]$ and strain $\perp [100]$<br>• $\boldsymbol{E} \parallel [100]$ and $\boldsymbol{B} \perp [100]$
$Pmn'2_1'$ (31.126) $\mathbb{Z}_1$	• $\boldsymbol{E} \parallel [100]$ and $\boldsymbol{B} \parallel [001]$
$Pm'n2_1'$ (31.125) $\mathbb{Z}_1$	• $\boldsymbol{E} \parallel [100]$ and $\boldsymbol{B} \parallel [010]$
$P2_1$ (4.7) $\mathbb{Z}_1$	• $\boldsymbol{E} \parallel [100]$ and $\boldsymbol{B} \parallel [100]$ and strain $\perp [100]$
$Pm'n'2_1$ (31.127) $\mathbb{Z}_1$	• $\boldsymbol{E} \parallel [100]$ and $\boldsymbol{B} \parallel [100]$
$P2_11'$ (4.8) $\mathbb{Z}_1$	• $\boldsymbol{E} \parallel [100]$ and strain $\perp [100]$
$Pmn2_11'$ (31.124) $\mathbb{Z}_1$	• $\boldsymbol{E} \parallel [100]$
$P2'$ (3.3) $\mathbb{Z}_1$	• $\boldsymbol{E} \parallel [001]$ and $\boldsymbol{B} \parallel [100]$ and strain $\perp [001]$<br>• $\boldsymbol{E} \parallel [001]$ and $\boldsymbol{B} \parallel [010]$ and strain $\perp [001]$<br>• $\boldsymbol{E} \parallel [001]$ and $\boldsymbol{B} \perp [001]$
$P2$ (3.1) $\mathbb{Z}_2$	• $\boldsymbol{E} \parallel [001]$ and $\boldsymbol{B} \parallel [001]$ and strain $\perp [001]$
$P21'$ (3.2) $\mathbb{Z}_2$	• $\boldsymbol{E} \parallel [001]$ and strain $\perp [001]$
$P\bar{1}$ (2.4) $\mathbb{Z}_2 \times \mathbb{Z}_2 \times \mathbb{Z}_2 \times \mathbb{Z}_4$	• $\boldsymbol{B} \parallel [100]$ and strain in generic direction<br>• $\boldsymbol{B} \parallel [010]$ and strain in generic direction<br>• $\boldsymbol{B} \parallel [001]$ and strain in generic direction<br>• $\boldsymbol{B} \perp [100]$ and strain $\perp [010]$<br>• $\boldsymbol{B} \perp [100]$ and strain $\perp [001]$<br>• $\boldsymbol{B} \perp [100]$ and strain in generic direction<br>• $\boldsymbol{B} \perp [010]$ and strain $\perp [100]$<br>• $\boldsymbol{B} \perp [010]$ and strain $\perp [001]$<br>• $\boldsymbol{B} \perp [010]$ and strain in generic direction<br>• $\boldsymbol{B} \perp [001]$ and strain $\perp [100]$<br>• $\boldsymbol{B} \perp [001]$ and strain $\perp [010]$<br>• $\boldsymbol{B} \perp [001]$ and strain in generic direction<br>• $\boldsymbol{B}$ in generic direction

Continued on next page

...continued

Subgroup & SI group	Perturbation
$P2'/m'$ (10.46) $\mathbb{Z}_2 \times \mathbb{Z}_2 \times \mathbb{Z}_2 \times \mathbb{Z}_4$	• $\boldsymbol{B} \parallel [100]$ and strain $\perp [001]$<br>• $\boldsymbol{B} \parallel [010]$ and strain $\perp [001]$<br>• $\boldsymbol{B} \perp [001]$
$P2/m$ (10.42) $\mathbb{Z}_2 \times \mathbb{Z}_2 \times \mathbb{Z}_2$	• $\boldsymbol{B} \parallel [001]$ and strain $\perp [001]$
$P\bar{1}1'$ (2.5) $\mathbb{Z}_2 \times \mathbb{Z}_2 \times \mathbb{Z}_2 \times \mathbb{Z}_4$	• strain in generic direction
$P2/m1'$ (10.43) $\mathbb{Z}_2 \times \mathbb{Z}_2 \times \mathbb{Z}_2$	• strain $\perp [001]$
Pc' (7.26) $\mathbb{Z}_1$	• $\boldsymbol{E} \parallel [010]$ and $\boldsymbol{B} \parallel [010]$ and strain $\perp [100]$<br>• $\boldsymbol{E} \parallel [010]$ and $\boldsymbol{B} \parallel [001]$ and strain $\perp [100]$<br>• $\boldsymbol{E} \parallel [010]$ and $\boldsymbol{B} \perp [100]$<br>• $\boldsymbol{E} \parallel [001]$ and $\boldsymbol{B} \parallel [010]$ and strain $\perp [100]$<br>• $\boldsymbol{E} \parallel [001]$ and $\boldsymbol{B} \parallel [001]$ and strain $\perp [100]$<br>• $\boldsymbol{E} \parallel [001]$ and $\boldsymbol{B} \perp [100]$<br>• $\boldsymbol{E} \perp [100]$ and $\boldsymbol{B} \parallel [010]$<br>• $\boldsymbol{E} \perp [100]$ and $\boldsymbol{B} \parallel [001]$<br>• $\boldsymbol{E} \perp [100]$ and $\boldsymbol{B} \perp [100]$
$Pn'n2'$ (34.158) $\mathbb{Z}_1$	• $\boldsymbol{E} \parallel [001]$ and $\boldsymbol{B} \parallel [010]$
$Pn'n'2$ (34.159) $\mathbb{Z}_1$	• $\boldsymbol{E} \parallel [001]$ and $\boldsymbol{B} \parallel [001]$
$P2_1'/c'$ (14.79) $\mathbb{Z}_2 \times \mathbb{Z}_4$	• $\boldsymbol{B} \parallel [010]$ and strain $\perp [100]$<br>• $\boldsymbol{B} \parallel [001]$ and strain $\perp [100]$<br>• $\boldsymbol{B} \perp [100]$
Pc (7.24) $\mathbb{Z}_1$	• $\boldsymbol{E} \parallel [010]$ and $\boldsymbol{B} \parallel [100]$ and strain $\perp [100]$<br>• $\boldsymbol{E} \parallel [001]$ and $\boldsymbol{B} \parallel [100]$ and strain $\perp [100]$<br>• $\boldsymbol{E} \perp [100]$ and $\boldsymbol{B} \parallel [100]$
$Pn'n2'$ (34.158) $\mathbb{Z}_1$	• $\boldsymbol{E} \parallel [001]$ and $\boldsymbol{B} \parallel [100]$
$P2_1/c$ (14.75) $\mathbb{Z}_2$	• $\boldsymbol{B} \parallel [100]$ and strain $\perp [100]$
$Pc1'$ (7.25) $\mathbb{Z}_1$	• $\boldsymbol{E} \parallel [010]$ and strain $\perp [100]$<br>• $\boldsymbol{E} \parallel [001]$ and strain $\perp [100]$<br>• $\boldsymbol{E} \perp [100]$
$Pnn21'$ (34.157) $\mathbb{Z}_1$	• $\boldsymbol{E} \parallel [001]$
$P2_1/c1'$ (14.76) $\mathbb{Z}_2$	• strain $\perp [100]$
$P2_1'$ (4.9) $\mathbb{Z}_1$	• $\boldsymbol{E} \parallel [010]$ and $\boldsymbol{B} \parallel [100]$ and strain $\perp [010]$<br>• $\boldsymbol{E} \parallel [010]$ and $\boldsymbol{B} \parallel [001]$ and strain $\perp [010]$<br>• $\boldsymbol{E} \parallel [010]$ and $\boldsymbol{B} \perp [010]$

Continued on next page

…continued

Subgroup & SI group	Perturbation
$P2_1'/c'$ (14.79) $\mathbb{Z}_2 \times \mathbb{Z}_4$	• $\boldsymbol{B} \parallel [100]$ and strain $\perp [010]$<br>• $\boldsymbol{B} \parallel [001]$ and strain $\perp [010]$<br>• $\boldsymbol{B} \perp [010]$
$Pmn'2_1'$ (31.126) $\mathbb{Z}_1$	• $\boldsymbol{E} \parallel [010]$ and $\boldsymbol{B} \parallel [001]$
$Pn'n'm$ (58.397) $\mathbb{Z}_2 \times \mathbb{Z}_2$	• $\boldsymbol{B} \parallel [001]$
$Pm'n2_1'$ (31.125) $\mathbb{Z}_1$	• $\boldsymbol{E} \parallel [010]$ and $\boldsymbol{B} \parallel [100]$
$Pnn'm'$ (58.398) $\mathbb{Z}_2$	• $\boldsymbol{B} \parallel [100]$
$P2_1$ (4.7) $\mathbb{Z}_1$	• $\boldsymbol{E} \parallel [010]$ and $\boldsymbol{B} \parallel [010]$ and strain $\perp [010]$
$P2_1/c$ (14.75) $\mathbb{Z}_2$	• $\boldsymbol{B} \parallel [010]$ and strain $\perp [010]$
$Pm'n'2_1$ (31.127) $\mathbb{Z}_1$	• $\boldsymbol{E} \parallel [010]$ and $\boldsymbol{B} \parallel [010]$
$Pnn'm'$ (58.398) $\mathbb{Z}_2$	• $\boldsymbol{B} \parallel [010]$
$P2_11'$ (4.8) $\mathbb{Z}_1$	• $\boldsymbol{E} \parallel [010]$ and strain $\perp [010]$
$P2_1/c1'$ (14.76) $\mathbb{Z}_2$	• strain $\perp [010]$
$Pmn2_11'$ (31.124) $\mathbb{Z}_1$	• $\boldsymbol{E} \parallel [010]$

Table. S 505: Subgroups of $Pn'nm$ (58.395) that can be realized by applying $\boldsymbol{E}/\boldsymbol{B}$ fields and/or uniaxial strains.

Subgroup & SI group	Perturbation
Pm (6.18) $\mathbb{Z}_1$	• $\boldsymbol{B} \parallel [001]$ and strain $\perp [001]$<br>• $\boldsymbol{E} \parallel [100]$ and strain $\perp [001]$<br>• $\boldsymbol{E} \parallel [100]$ and $\boldsymbol{B} \parallel [001]$<br>• $\boldsymbol{E} \parallel [010]$ and strain $\perp [001]$<br>• $\boldsymbol{E} \perp [001]$
Pc (7.24) $\mathbb{Z}_1$	• $\boldsymbol{B} \parallel [010]$ and strain $\perp [010]$<br>• $\boldsymbol{E} \parallel [100]$ and strain $\perp [010]$<br>• $\boldsymbol{E} \parallel [100]$ and $\boldsymbol{B} \parallel [010]$<br>• $\boldsymbol{E} \parallel [001]$ and strain $\perp [010]$<br>• $\boldsymbol{E} \perp [010]$
$P2_1$ (4.7) $\mathbb{Z}_1$	• $\boldsymbol{B} \parallel [100]$ and strain $\perp [100]$<br>• $\boldsymbol{E} \parallel [100]$ and strain $\perp [100]$<br>• $\boldsymbol{E} \parallel [100]$ and $\boldsymbol{B} \parallel [100]$

Continued on next page

. . . continued

Subgroup & SI group	Perturbation
$Pmn2_1$ (31.123) $\mathbb{Z}_1$	• $\boldsymbol{E}$ ∥ [100]
$P2'$ (3.3) $\mathbb{Z}_1$	• $\boldsymbol{B}$ ∥ [100] and strain ⊥ [001]<br>• $\boldsymbol{B}$ ∥ [010] and strain ⊥ [001]<br>• $\boldsymbol{B}$ ⊥ [001]<br>• $\boldsymbol{E}$ ∥ [001] and strain ⊥ [001]<br>• $\boldsymbol{E}$ ∥ [001] and $\boldsymbol{B}$ ∥ [100]
$P\bar{1}'$ (2.6) $\mathbb{Z}_1$	• strain in generic direction
$P2'/m$ (10.44) $\mathbb{Z}_1$	• strain ⊥ [001]
Pc' (7.26) $\mathbb{Z}_1$	• $\boldsymbol{B}$ ∥ [010] and strain ⊥ [100]<br>• $\boldsymbol{B}$ ∥ [001] and strain ⊥ [100]<br>• $\boldsymbol{B}$ ⊥ [100]<br>• $\boldsymbol{E}$ ∥ [010] and strain ⊥ [100]<br>• $\boldsymbol{E}$ ∥ [010] and $\boldsymbol{B}$ ∥ [010]<br>• $\boldsymbol{E}$ ∥ [001] and strain ⊥ [100]<br>• $\boldsymbol{E}$ ∥ [001] and $\boldsymbol{B}$ ∥ [001]<br>• $\boldsymbol{E}$ ⊥ [100]
$Pn'n2'$ (34.158) $\mathbb{Z}_1$	• $\boldsymbol{B}$ ∥ [010]<br>• $\boldsymbol{E}$ ∥ [001]
$P2_1/c'$ (14.78) $\mathbb{Z}_1$	• strain ⊥ [100]
$P2_1'$ (4.9) $\mathbb{Z}_1$	• $\boldsymbol{B}$ ∥ [100] and strain ⊥ [010]<br>• $\boldsymbol{B}$ ∥ [001] and strain ⊥ [010]<br>• $\boldsymbol{B}$ ⊥ [010]<br>• $\boldsymbol{E}$ ∥ [010] and strain ⊥ [010]<br>• $\boldsymbol{E}$ ∥ [010] and $\boldsymbol{B}$ ∥ [100]
$P2_12_1'2'$ (18.19) $\mathbb{Z}_1$	• $\boldsymbol{B}$ ∥ [100]
$P2_1'/c$ (14.77) $\mathbb{Z}_1$	• strain ⊥ [010]
$Pmn'2_1'$ (31.126) $\mathbb{Z}_1$	• $\boldsymbol{B}$ ∥ [001]<br>• $\boldsymbol{E}$ ∥ [010]

Table. S 506: Subgroups of $Pnnm'$ (58.396) that can be realized by applying $\boldsymbol{E}/\boldsymbol{B}$ fields and/or uniaxial strains.

Subgroup & SI group	Perturbation

Continued on next page

…continued

Subgroup & SI group	Perturbation
Pm' (6.20) $\mathbb{Z}_1$	• $\boldsymbol{B} \parallel [100]$ and strain $\perp [001]$<br>• $\boldsymbol{B} \parallel [010]$ and strain $\perp [001]$<br>• $\boldsymbol{B} \perp [001]$<br>• $\boldsymbol{E} \parallel [100]$ and strain $\perp [001]$<br>• $\boldsymbol{E} \parallel [100]$ and $\boldsymbol{B} \parallel [100]$<br>• $\boldsymbol{E} \parallel [010]$ and strain $\perp [001]$<br>• $\boldsymbol{E} \parallel [010]$ and $\boldsymbol{B} \parallel [010]$<br>• $\boldsymbol{E} \perp [001]$
Pc (7.24) $\mathbb{Z}_1$	• $\boldsymbol{B} \parallel [010]$ and strain $\perp [010]$<br>• $\boldsymbol{E} \parallel [100]$ and strain $\perp [010]$<br>• $\boldsymbol{E} \parallel [001]$ and strain $\perp [010]$<br>• $\boldsymbol{E} \parallel [001]$ and $\boldsymbol{B} \parallel [010]$<br>• $\boldsymbol{E} \perp [010]$
$P2_1'$ (4.9) $\mathbb{Z}_1$	• $\boldsymbol{B} \parallel [010]$ and strain $\perp [100]$<br>• $\boldsymbol{B} \parallel [001]$ and strain $\perp [100]$<br>• $\boldsymbol{B} \perp [100]$<br>• $\boldsymbol{E} \parallel [100]$ and strain $\perp [100]$<br>• $\boldsymbol{E} \parallel [100]$ and $\boldsymbol{B} \parallel [001]$
$Pm'n2_1'$ (31.125) $\mathbb{Z}_1$	• $\boldsymbol{B} \parallel [010]$<br>• $\boldsymbol{E} \parallel [100]$
$P2$ (3.1) $\mathbb{Z}_2$	• $\boldsymbol{B} \parallel [001]$ and strain $\perp [001]$<br>• $\boldsymbol{E} \parallel [001]$ and strain $\perp [001]$<br>• $\boldsymbol{E} \parallel [001]$ and $\boldsymbol{B} \parallel [001]$
$P\bar{1}'$ (2.6) $\mathbb{Z}_1$	• strain in generic direction
$P2/m'$ (10.45) $\mathbb{Z}_2$	• strain $\perp [001]$
Pc (7.24) $\mathbb{Z}_1$	• $\boldsymbol{B} \parallel [100]$ and strain $\perp [100]$<br>• $\boldsymbol{E} \parallel [010]$ and strain $\perp [100]$<br>• $\boldsymbol{E} \parallel [001]$ and strain $\perp [100]$<br>• $\boldsymbol{E} \parallel [001]$ and $\boldsymbol{B} \parallel [100]$<br>• $\boldsymbol{E} \perp [100]$
$Pnn2$ (34.156) $\mathbb{Z}_1$	• $\boldsymbol{E} \parallel [001]$
$P2_1'/c$ (14.77) $\mathbb{Z}_1$	• strain $\perp [100]$

Continued on next page

...continued

Subgroup & SI group	Perturbation
$P2_1'$ (4.9) $\mathbb{Z}_1$	• $\boldsymbol{B}$ ∥ [100] and strain ⊥ [010]<br>• $\boldsymbol{B}$ ∥ [001] and strain ⊥ [010]<br>• $\boldsymbol{B}$ ⊥ [010]<br>• $\boldsymbol{E}$ ∥ [010] and strain ⊥ [010]<br>• $\boldsymbol{E}$ ∥ [010] and $\boldsymbol{B}$ ∥ [001]
$P2_1'2_1'2$ (18.18) $\mathbb{Z}_2$	• $\boldsymbol{B}$ ∥ [001]
$P2_1'/c$ (14.77) $\mathbb{Z}_1$	• strain ⊥ [010]
$Pm'n2_1'$ (31.125) $\mathbb{Z}_1$	• $\boldsymbol{B}$ ∥ [100]<br>• $\boldsymbol{E}$ ∥ [010]

Table. S 507: Subgroups of $Pn'n'm$ (58.397) that can be realized by applying $\boldsymbol{E}/\boldsymbol{B}$ fields and/or uniaxial strains.

Subgroup & SI group	Perturbation
Pm (6.18) $\mathbb{Z}_1$	• $\boldsymbol{E}$ ∥ [100] and strain ⊥ [001]<br>• $\boldsymbol{E}$ ∥ [010] and strain ⊥ [001]<br>• $\boldsymbol{E}$ ⊥ [001]
Pc' (7.26) $\mathbb{Z}_1$	• $\boldsymbol{E}$ ∥ [100] and strain ⊥ [010]<br>• $\boldsymbol{E}$ ∥ [100] and ($\boldsymbol{B}$ ∥ [100] or $\boldsymbol{B}$ ⊥ [010])<br>• $\boldsymbol{E}$ ∥ [001] and strain ⊥ [010]<br>• $\boldsymbol{E}$ ∥ [001] and ($\boldsymbol{B}$ ∥ [100] or $\boldsymbol{B}$ ⊥ [010])<br>• $\boldsymbol{E}$ ⊥ [010]
$P2_1'$ (4.9) $\mathbb{Z}_1$	• $\boldsymbol{E}$ ∥ [100] and strain ⊥ [100]<br>• $\boldsymbol{E}$ ∥ [100] and ($\boldsymbol{B}$ ∥ [010] or $\boldsymbol{B}$ ⊥ [100])
$Pmn'2_1'$ (31.126) $\mathbb{Z}_1$	• $\boldsymbol{E}$ ∥ [100]
$P2$ (3.1) $\mathbb{Z}_2$	• $\boldsymbol{E}$ ∥ [001] and strain ⊥ [001]
$P\bar{1}$ (2.4) $\mathbb{Z}_2\times\mathbb{Z}_2\times\mathbb{Z}_2\times\mathbb{Z}_4$	• strain in generic direction<br>• ($\boldsymbol{B}$ ∥ [100] or $\boldsymbol{B}$ ⊥ [010]) and strain ⊥ [100]<br>• ($\boldsymbol{B}$ ∥ [100] or $\boldsymbol{B}$ ⊥ [010]) and strain ⊥ [001]<br>• ($\boldsymbol{B}$ ∥ [010] or $\boldsymbol{B}$ ⊥ [100]) and strain ⊥ [010]<br>• ($\boldsymbol{B}$ ∥ [010] or $\boldsymbol{B}$ ⊥ [100]) and strain ⊥ [001]<br>• $\boldsymbol{B}$ in generic direction
$P2/m$ (10.42) $\mathbb{Z}_2\times\mathbb{Z}_2\times\mathbb{Z}_2$	• strain ⊥ [001]

Continued on next page

…continued

Subgroup & SI group	Perturbation
Pc' (7.26) $\mathbb{Z}_1$	• $\boldsymbol{E} \parallel [010]$ and strain $\perp [100]$<br>• $\boldsymbol{E} \parallel [010]$ and ($\boldsymbol{B} \parallel [010]$ or $\boldsymbol{B} \perp [100]$)<br>• $\boldsymbol{E} \parallel [001]$ and strain $\perp [100]$<br>• $\boldsymbol{E} \parallel [001]$ and ($\boldsymbol{B} \parallel [010]$ or $\boldsymbol{B} \perp [100]$)<br>• $\boldsymbol{E} \perp [100]$
$Pn'n'2$ (34.159) $\mathbb{Z}_1$	• $\boldsymbol{E} \parallel [001]$
$P2_1'/c'$ (14.79) $\mathbb{Z}_2 \times \mathbb{Z}_4$	• strain $\perp [100]$<br>• ($\boldsymbol{B} \parallel [010]$ or $\boldsymbol{B} \perp [100]$)
$P2_1'$ (4.9) $\mathbb{Z}_1$	• $\boldsymbol{E} \parallel [010]$ and strain $\perp [010]$<br>• $\boldsymbol{E} \parallel [010]$ and ($\boldsymbol{B} \parallel [100]$ or $\boldsymbol{B} \perp [010]$)
$P2_1'/c'$ (14.79) $\mathbb{Z}_2 \times \mathbb{Z}_4$	• strain $\perp [010]$<br>• ($\boldsymbol{B} \parallel [100]$ or $\boldsymbol{B} \perp [010]$)
$Pmn'2_1'$ (31.126) $\mathbb{Z}_1$	• $\boldsymbol{E} \parallel [010]$

Table. S 508: Subgroups of $Pnn'm'$ (58.398) that can be realized by applying $\boldsymbol{E}/\boldsymbol{B}$ fields and/or uniaxial strains.

Subgroup & SI group	Perturbation
Pm' (6.20) $\mathbb{Z}_1$	• $\boldsymbol{E} \parallel [100]$ and strain $\perp [001]$<br>• $\boldsymbol{E} \parallel [100]$ and ($\boldsymbol{B} \parallel [010]$ or $\boldsymbol{B} \perp [001]$)<br>• $\boldsymbol{E} \parallel [010]$ and strain $\perp [001]$<br>• $\boldsymbol{E} \parallel [010]$ and ($\boldsymbol{B} \parallel [010]$ or $\boldsymbol{B} \perp [001]$)<br>• $\boldsymbol{E} \perp [001]$
Pc' (7.26) $\mathbb{Z}_1$	• $\boldsymbol{E} \parallel [100]$ and strain $\perp [010]$<br>• $\boldsymbol{E} \parallel [100]$ and ($\boldsymbol{B} \parallel [001]$ or $\boldsymbol{B} \perp [010]$)<br>• $\boldsymbol{E} \parallel [001]$ and strain $\perp [010]$<br>• $\boldsymbol{E} \parallel [001]$ and ($\boldsymbol{B} \parallel [001]$ or $\boldsymbol{B} \perp [010]$)<br>• $\boldsymbol{E} \perp [010]$
$P2_1$ (4.7) $\mathbb{Z}_1$	• $\boldsymbol{E} \parallel [100]$ and strain $\perp [100]$
$Pm'n'2_1$ (31.127) $\mathbb{Z}_1$	• $\boldsymbol{E} \parallel [100]$
$P2'$ (3.3) $\mathbb{Z}_1$	• $\boldsymbol{E} \parallel [001]$ and strain $\perp [001]$<br>• $\boldsymbol{E} \parallel [001]$ and ($\boldsymbol{B} \parallel [010]$ or $\boldsymbol{B} \perp [001]$)

Continued on next page

...continued

Subgroup & SI group	Perturbation
$P\bar{1}$ (2.4) $\mathbb{Z}_2 \times \mathbb{Z}_2 \times \mathbb{Z}_2 \times \mathbb{Z}_4$	• strain in generic direction<br>• ($\boldsymbol{B} \parallel [010]$ or $\boldsymbol{B} \perp [001]$) and strain $\perp [100]$<br>• ($\boldsymbol{B} \parallel [010]$ or $\boldsymbol{B} \perp [001]$) and strain $\perp [010]$<br>• ($\boldsymbol{B} \parallel [001]$ or $\boldsymbol{B} \perp [010]$) and strain $\perp [100]$<br>• ($\boldsymbol{B} \parallel [001]$ or $\boldsymbol{B} \perp [010]$) and strain $\perp [001]$<br>• $\boldsymbol{B}$ in generic direction
$P2'/m'$ (10.46) $\mathbb{Z}_2 \times \mathbb{Z}_2 \times \mathbb{Z}_2 \times \mathbb{Z}_4$	• strain $\perp [001]$<br>• ($\boldsymbol{B} \parallel [010]$ or $\boldsymbol{B} \perp [001]$)
Pc (7.24) $\mathbb{Z}_1$	• $\boldsymbol{E} \parallel [010]$ and strain $\perp [100]$<br>• $\boldsymbol{E} \parallel [001]$ and strain $\perp [100]$<br>• $\boldsymbol{E} \perp [100]$
$Pn'n2'$ (34.158) $\mathbb{Z}_1$	• $\boldsymbol{E} \parallel [001]$
$P2_1/c$ (14.75) $\mathbb{Z}_2$	• strain $\perp [100]$
$P2_1'$ (4.9) $\mathbb{Z}_1$	• $\boldsymbol{E} \parallel [010]$ and strain $\perp [010]$<br>• $\boldsymbol{E} \parallel [010]$ and ($\boldsymbol{B} \parallel [001]$ or $\boldsymbol{B} \perp [010]$)
$P2_1'/c'$ (14.79) $\mathbb{Z}_2 \times \mathbb{Z}_4$	• strain $\perp [010]$<br>• ($\boldsymbol{B} \parallel [001]$ or $\boldsymbol{B} \perp [010]$)
$Pm'n2_1'$ (31.125) $\mathbb{Z}_1$	• $\boldsymbol{E} \parallel [010]$

Table. S 509: Subgroups of $Pn'n'm'$ (58.399) that can be realized by applying $\boldsymbol{E}/\boldsymbol{B}$ fields and/or uniaxial strains.

Subgroup & SI group	Perturbation
Pm' (6.20) $\mathbb{Z}_1$	• $\boldsymbol{B} \parallel [100]$ and strain $\perp [001]$<br>• $\boldsymbol{B} \parallel [010]$ and strain $\perp [001]$<br>• $\boldsymbol{B} \perp [001]$<br>• $\boldsymbol{E} \parallel [100]$ and strain $\perp [001]$<br>• $\boldsymbol{E} \parallel [100]$ and $\boldsymbol{B} \parallel [010]$<br>• $\boldsymbol{E} \parallel [010]$ and strain $\perp [001]$<br>• $\boldsymbol{E} \parallel [010]$ and $\boldsymbol{B} \parallel [100]$<br>• $\boldsymbol{E} \perp [001]$

Continued on next page

...continued

Subgroup & SI group	Perturbation
Pc' (7.26) $\mathbb{Z}_1$	• $\boldsymbol{B}$ ∥ [100] and strain ⊥ [010]<br>• $\boldsymbol{B}$ ∥ [001] and strain ⊥ [010]<br>• $\boldsymbol{B}$ ⊥ [010]<br>• $\boldsymbol{E}$ ∥ [100] and strain ⊥ [010]<br>• $\boldsymbol{E}$ ∥ [100] and $\boldsymbol{B}$ ∥ [001]<br>• $\boldsymbol{E}$ ∥ [001] and strain ⊥ [010]<br>• $\boldsymbol{E}$ ∥ [001] and $\boldsymbol{B}$ ∥ [100]<br>• $\boldsymbol{E}$ ⊥ [010]
$P2_1$ (4.7) $\mathbb{Z}_1$	• $\boldsymbol{B}$ ∥ [100] and strain ⊥ [100]<br>• $\boldsymbol{E}$ ∥ [100] and strain ⊥ [100]
$Pm'n'2_1$ (31.127) $\mathbb{Z}_1$	• $\boldsymbol{B}$ ∥ [100]<br>• $\boldsymbol{E}$ ∥ [100]
$P2$ (3.1) $\mathbb{Z}_2$	• $\boldsymbol{B}$ ∥ [001] and strain ⊥ [001]<br>• $\boldsymbol{E}$ ∥ [001] and strain ⊥ [001]
$P\bar{1}'$ (2.6) $\mathbb{Z}_1$	• strain in generic direction
$P2/m'$ (10.45) $\mathbb{Z}_2$	• strain ⊥ [001]
Pc' (7.26) $\mathbb{Z}_1$	• $\boldsymbol{B}$ ∥ [010] and strain ⊥ [100]<br>• $\boldsymbol{B}$ ∥ [001] and strain ⊥ [100]<br>• $\boldsymbol{B}$ ⊥ [100]<br>• $\boldsymbol{E}$ ∥ [010] and strain ⊥ [100]<br>• $\boldsymbol{E}$ ∥ [010] and $\boldsymbol{B}$ ∥ [001]<br>• $\boldsymbol{E}$ ∥ [001] and strain ⊥ [100]<br>• $\boldsymbol{E}$ ∥ [001] and $\boldsymbol{B}$ ∥ [010]<br>• $\boldsymbol{E}$ ⊥ [100]
$Pn'n'2$ (34.159) $\mathbb{Z}_1$	• $\boldsymbol{B}$ ∥ [001]<br>• $\boldsymbol{E}$ ∥ [001]
$P2_1/c'$ (14.78) $\mathbb{Z}_1$	• strain ⊥ [100]
$P2_1$ (4.7) $\mathbb{Z}_1$	• $\boldsymbol{B}$ ∥ [010] and strain ⊥ [010]<br>• $\boldsymbol{E}$ ∥ [010] and strain ⊥ [010]
$P2_1/c'$ (14.78) $\mathbb{Z}_1$	• strain ⊥ [010]
$Pm'n'2_1$ (31.127) $\mathbb{Z}_1$	• $\boldsymbol{B}$ ∥ [010]<br>• $\boldsymbol{E}$ ∥ [010]

Table. S 510: Subgroups of $P_{a}nnm$ (58.400) that can be realized by applying $\boldsymbol{E}/\boldsymbol{B}$ fields and/or uniaxial strains.

Subgroup & SI group	Perturbation
Pm (6.18) $\mathbb{Z}_1$	• $\boldsymbol{E}$ ∥ [100] and $\boldsymbol{B}$ ∥ [001] and strain ⊥ [001]<br>• $\boldsymbol{E}$ ∥ [010] and $\boldsymbol{B}$ ∥ [001] and strain ⊥ [001]<br>• $\boldsymbol{E}$ ⊥ [001] and $\boldsymbol{B}$ ∥ [001]
Pc' (7.26) $\mathbb{Z}_1$	• $\boldsymbol{E}$ ∥ [100] and $\boldsymbol{B}$ ∥ [100] and strain ⊥ [010]<br>• $\boldsymbol{E}$ ∥ [100] and $\boldsymbol{B}$ ∥ [001] and strain ⊥ [010]<br>• $\boldsymbol{E}$ ∥ [100] and $\boldsymbol{B}$ ⊥ [010]<br>• $\boldsymbol{E}$ ∥ [001] and $\boldsymbol{B}$ ∥ [100] and strain ⊥ [010]<br>• $\boldsymbol{E}$ ∥ [001] and $\boldsymbol{B}$ ∥ [001] and strain ⊥ [010]<br>• $\boldsymbol{E}$ ∥ [001] and $\boldsymbol{B}$ ⊥ [010]<br>• $\boldsymbol{E}$ ⊥ [010] and $\boldsymbol{B}$ ∥ [100]<br>• $\boldsymbol{E}$ ⊥ [010] and $\boldsymbol{B}$ ∥ [001]<br>• $\boldsymbol{E}$ ⊥ [010] and $\boldsymbol{B}$ ⊥ [010]
$P2'$ (3.3) $\mathbb{Z}_1$	• $\boldsymbol{E}$ ∥ [100] and $\boldsymbol{B}$ ∥ [010] and strain ⊥ [100]<br>• $\boldsymbol{E}$ ∥ [100] and $\boldsymbol{B}$ ∥ [001] and strain ⊥ [100]<br>• $\boldsymbol{E}$ ∥ [100] and $\boldsymbol{B}$ ⊥ [100]
$Pma'2'$ (28.90) $\mathbb{Z}_1$	• $\boldsymbol{E}$ ∥ [100] and $\boldsymbol{B}$ ∥ [001]
P_S1 (1.3) $\mathbb{Z}_1$	• $\boldsymbol{E}$ ∥ [100] and strain in generic direction<br>• $\boldsymbol{E}$ ∥ [010] and strain in generic direction<br>• $\boldsymbol{E}$ ∥ [001] and strain in generic direction<br>• $\boldsymbol{E}$ ⊥ [100] and strain ⊥ [010]<br>• $\boldsymbol{E}$ ⊥ [100] and strain ⊥ [001]<br>• $\boldsymbol{E}$ ⊥ [100] and strain in generic direction<br>• $\boldsymbol{E}$ ⊥ [010] and strain ⊥ [100]<br>• $\boldsymbol{E}$ ⊥ [010] and strain ⊥ [001]<br>• $\boldsymbol{E}$ ⊥ [010] and strain in generic direction<br>• $\boldsymbol{E}$ ⊥ [001] and strain ⊥ [100]<br>• $\boldsymbol{E}$ ⊥ [001] and strain ⊥ [010]<br>• $\boldsymbol{E}$ ⊥ [001] and strain in generic direction<br>• $\boldsymbol{E}$ in generic direction

Continued on next page

…continued

Subgroup & SI group	Perturbation
Pc' (7.26) $\mathbb{Z}_1$	• $\boldsymbol{E} \parallel$ [100] and $\boldsymbol{B} \parallel$ [100] and strain $\perp$ [001]<br>• $\boldsymbol{E} \parallel$ [100] and $\boldsymbol{B} \parallel$ [010] and strain $\perp$ [001]<br>• $\boldsymbol{E} \parallel$ [100] and $\boldsymbol{B} \perp$ [001]<br>• $\boldsymbol{E} \parallel$ [010] and $\boldsymbol{B} \parallel$ [100] and strain $\perp$ [001]<br>• $\boldsymbol{E} \parallel$ [010] and $\boldsymbol{B} \parallel$ [010] and strain $\perp$ [001]<br>• $\boldsymbol{E} \parallel$ [010] and $\boldsymbol{B} \perp$ [001]<br>• $\boldsymbol{E} \perp$ [001] and $\boldsymbol{B} \parallel$ [100]<br>• $\boldsymbol{E} \perp$ [001] and $\boldsymbol{B} \parallel$ [010]<br>• $\boldsymbol{E} \perp$ [001] and $\boldsymbol{B} \perp$ [001]
P_am (6.21) $\mathbb{Z}_1$	• $\boldsymbol{E} \parallel$ [100] and strain $\perp$ [001]<br>• $\boldsymbol{E} \parallel$ [010] and strain $\perp$ [001]<br>• $\boldsymbol{E} \perp$ [001]
Pc (7.24) $\mathbb{Z}_1$	• $\boldsymbol{E} \parallel$ [100] and $\boldsymbol{B} \parallel$ [010] and strain $\perp$ [010]<br>• $\boldsymbol{E} \parallel$ [001] and $\boldsymbol{B} \parallel$ [010] and strain $\perp$ [010]<br>• $\boldsymbol{E} \perp$ [010] and $\boldsymbol{B} \parallel$ [010]
P_ac (7.27) $\mathbb{Z}_1$	• $\boldsymbol{E} \parallel$ [100] and strain $\perp$ [010]<br>• $\boldsymbol{E} \parallel$ [001] and strain $\perp$ [010]<br>• $\boldsymbol{E} \perp$ [010]
$Pnc'2'$ (30.114) $\mathbb{Z}_1$	• $\boldsymbol{E} \parallel$ [100] and $\boldsymbol{B} \parallel$ [010]
$P2_1$ (4.7) $\mathbb{Z}_1$	• $\boldsymbol{E} \parallel$ [100] and $\boldsymbol{B} \parallel$ [100] and strain $\perp$ [100]
P_b2_1 (4.11) $\mathbb{Z}_1$	• $\boldsymbol{E} \parallel$ [100] and strain $\perp$ [100]
$Pc'a'2_1$ (29.103) $\mathbb{Z}_1$	• $\boldsymbol{E} \parallel$ [100] and $\boldsymbol{B} \parallel$ [100]
P_cmn2_1 (31.130) $\mathbb{Z}_1$	• $\boldsymbol{E} \parallel$ [100]
Pc' (7.26) $\mathbb{Z}_1$	• $\boldsymbol{E} \parallel$ [010] and $\boldsymbol{B} \parallel$ [010] and strain $\perp$ [100]<br>• $\boldsymbol{E} \parallel$ [010] and $\boldsymbol{B} \parallel$ [001] and strain $\perp$ [100]<br>• $\boldsymbol{E} \parallel$ [010] and $\boldsymbol{B} \perp$ [100]<br>• $\boldsymbol{E} \parallel$ [001] and $\boldsymbol{B} \parallel$ [010] and strain $\perp$ [100]<br>• $\boldsymbol{E} \parallel$ [001] and $\boldsymbol{B} \parallel$ [001] and strain $\perp$ [100]<br>• $\boldsymbol{E} \parallel$ [001] and $\boldsymbol{B} \perp$ [100]<br>• $\boldsymbol{E} \perp$ [100] and $\boldsymbol{B} \parallel$ [010]<br>• $\boldsymbol{E} \perp$ [100] and $\boldsymbol{B} \parallel$ [001]<br>• $\boldsymbol{E} \perp$ [100] and $\boldsymbol{B} \perp$ [100]
$P2_1'$ (4.9) $\mathbb{Z}_1$	• $\boldsymbol{E} \parallel$ [010] and $\boldsymbol{B} \parallel$ [100] and strain $\perp$ [010]<br>• $\boldsymbol{E} \parallel$ [010] and $\boldsymbol{B} \parallel$ [001] and strain $\perp$ [010]<br>• $\boldsymbol{E} \parallel$ [010] and $\boldsymbol{B} \perp$ [010]
$Pmn'2_1'$ (31.126) $\mathbb{Z}_1$	• $\boldsymbol{E} \parallel$ [010] and $\boldsymbol{B} \parallel$ [001]

Continued on next page

. . . continued

Subgroup & SI group	Perturbation
$P2$ (3.1) $\mathbb{Z}_2$	• $\boldsymbol{E}$ ∥ [001] and $\boldsymbol{B}$ ∥ [001] and strain ⊥ [001]
$Pn'c'2$ (30.115) $\mathbb{Z}_1$	• $\boldsymbol{E}$ ∥ [001] and $\boldsymbol{B}$ ∥ [001]
$P\bar{1}$ (2.4) $\mathbb{Z}_2 \times \mathbb{Z}_2 \times \mathbb{Z}_2 \times \mathbb{Z}_4$	• $\boldsymbol{B}$ ∥ [100] and strain in generic direction<br>• $\boldsymbol{B}$ ∥ [010] and strain in generic direction<br>• $\boldsymbol{B}$ ∥ [001] and strain in generic direction<br>• $\boldsymbol{B}$ ⊥ [100] and strain ⊥ [010]<br>• $\boldsymbol{B}$ ⊥ [100] and strain ⊥ [001]<br>• $\boldsymbol{B}$ ⊥ [100] and strain in generic direction<br>• $\boldsymbol{B}$ ⊥ [010] and strain ⊥ [100]<br>• $\boldsymbol{B}$ ⊥ [010] and strain ⊥ [001]<br>• $\boldsymbol{B}$ ⊥ [010] and strain in generic direction<br>• $\boldsymbol{B}$ ⊥ [001] and strain ⊥ [100]<br>• $\boldsymbol{B}$ ⊥ [001] and strain ⊥ [010]<br>• $\boldsymbol{B}$ ⊥ [001] and strain in generic direction<br>• $\boldsymbol{B}$ in generic direction
$P2'/c'$ (13.69) $\mathbb{Z}_2 \times \mathbb{Z}_2 \times \mathbb{Z}_4$	• $\boldsymbol{B}$ ∥ [010] and strain ⊥ [100]<br>• $\boldsymbol{B}$ ∥ [001] and strain ⊥ [100]<br>• $\boldsymbol{B}$ ⊥ [100]
$P2_1'/c'$ (14.79) $\mathbb{Z}_2 \times \mathbb{Z}_4$	• $\boldsymbol{B}$ ∥ [100] and strain ⊥ [010]<br>• $\boldsymbol{B}$ ∥ [001] and strain ⊥ [010]<br>• $\boldsymbol{B}$ ⊥ [010]
$P2/m$ (10.42) $\mathbb{Z}_2 \times \mathbb{Z}_2 \times \mathbb{Z}_2$	• $\boldsymbol{B}$ ∥ [001] and strain ⊥ [001]
$Pmn'a'$ (53.327) $\mathbb{Z}_2 \times \mathbb{Z}_2$	• $\boldsymbol{B}$ ∥ [001]
Pc (7.24) $\mathbb{Z}_1$	• $\boldsymbol{E}$ ∥ [010] and $\boldsymbol{B}$ ∥ [100] and strain ⊥ [100]<br>• $\boldsymbol{E}$ ∥ [001] and $\boldsymbol{B}$ ∥ [100] and strain ⊥ [100]<br>• $\boldsymbol{E}$ ⊥ [100] and $\boldsymbol{B}$ ∥ [100]
P_bc (7.29) $\mathbb{Z}_1$	• $\boldsymbol{E}$ ∥ [010] and strain ⊥ [100]<br>• $\boldsymbol{E}$ ∥ [001] and strain ⊥ [100]<br>• $\boldsymbol{E}$ ⊥ [100]
$Pna'2_1'$ (33.147) $\mathbb{Z}_1$	• $\boldsymbol{E}$ ∥ [010] and $\boldsymbol{B}$ ∥ [100]
$P2_1/c$ (14.75) $\mathbb{Z}_2$	• $\boldsymbol{B}$ ∥ [100] and strain ⊥ [100]
$P2_1$ (4.7) $\mathbb{Z}_1$	• $\boldsymbol{E}$ ∥ [010] and $\boldsymbol{B}$ ∥ [010] and strain ⊥ [010]
$Pn'a'2_1$ (33.148) $\mathbb{Z}_1$	• $\boldsymbol{E}$ ∥ [010] and $\boldsymbol{B}$ ∥ [010]
P_a2_1 (4.10) $\mathbb{Z}_1$	• $\boldsymbol{E}$ ∥ [010] and strain ⊥ [010]
$P2_1/c$ (14.75) $\mathbb{Z}_2$	• $\boldsymbol{B}$ ∥ [010] and strain ⊥ [010]

Continued on next page

…continued

Subgroup & SI group	Perturbation
P_bmn2_1 (31.129) $\mathbb{Z}_1$	• $\boldsymbol{E} \parallel [010]$
$P2'$ (3.3) $\mathbb{Z}_1$	• $\boldsymbol{E} \parallel [001]$ and $\boldsymbol{B} \parallel [100]$ and strain $\perp [001]$<br>• $\boldsymbol{E} \parallel [001]$ and $\boldsymbol{B} \parallel [010]$ and strain $\perp [001]$<br>• $\boldsymbol{E} \parallel [001]$ and $\boldsymbol{B} \perp [001]$
$Pn'n2'$ (34.158) $\mathbb{Z}_1$	• $\boldsymbol{E} \parallel [001]$ and $\boldsymbol{B} \parallel [010]$
P_a2 (3.4) $\mathbb{Z}_2$	• $\boldsymbol{E} \parallel [001]$ and strain $\perp [001]$
$P2'/c'$ (13.69) $\mathbb{Z}_2 \times \mathbb{Z}_2 \times \mathbb{Z}_4$	• $\boldsymbol{B} \parallel [100]$ and strain $\perp [001]$<br>• $\boldsymbol{B} \parallel [010]$ and strain $\perp [001]$<br>• $\boldsymbol{B} \perp [001]$
$Pnc'2'$ (30.114) $\mathbb{Z}_1$	• $\boldsymbol{E} \parallel [001]$ and $\boldsymbol{B} \parallel [100]$
P_ann2 (34.160) $\mathbb{Z}_1$	• $\boldsymbol{E} \parallel [001]$
$Pb'c'n$ (60.422) $\mathbb{Z}_2$	• $\boldsymbol{B} \parallel [100]$
$Pn'na'$ (52.312) $\mathbb{Z}_2$	• $\boldsymbol{B} \parallel [010]$
$P_S\bar{1}$ (2.7) $\mathbb{Z}_2 \times \mathbb{Z}_2 \times \mathbb{Z}_4$	• strain in generic direction
P_b2_1/c (14.81) $\mathbb{Z}_2$	• strain $\perp [100]$
P_a2_1/c (14.80) $\mathbb{Z}_2$	• strain $\perp [010]$
P_a2/m (10.47) $\mathbb{Z}_2 \times \mathbb{Z}_2 \times \mathbb{Z}_2$	• strain $\perp [001]$

Table. S 511: Subgroups of P_cnnm (58.401) that can be realized by applying $\boldsymbol{E}/\boldsymbol{B}$ fields and/or uniaxial strains.

Subgroup & SI group	Perturbation

Continued on next page

...continued

Subgroup & SI group	Perturbation
P_S1 (1.3) $\mathbb{Z}_1$	• $\boldsymbol{E}$ ∥ [100] and strain in generic direction<br>• $\boldsymbol{E}$ ∥ [010] and strain in generic direction<br>• $\boldsymbol{E}$ ∥ [001] and strain in generic direction<br>• $\boldsymbol{E}$ ⊥ [100] and strain ⊥ [010]<br>• $\boldsymbol{E}$ ⊥ [100] and strain ⊥ [001]<br>• $\boldsymbol{E}$ ⊥ [100] and strain in generic direction<br>• $\boldsymbol{E}$ ⊥ [010] and strain ⊥ [100]<br>• $\boldsymbol{E}$ ⊥ [010] and strain ⊥ [001]<br>• $\boldsymbol{E}$ ⊥ [010] and strain in generic direction<br>• $\boldsymbol{E}$ ⊥ [001] and strain ⊥ [100]<br>• $\boldsymbol{E}$ ⊥ [001] and strain ⊥ [010]<br>• $\boldsymbol{E}$ ⊥ [001] and strain in generic direction<br>• $\boldsymbol{E}$ in generic direction
Pm (6.18) $\mathbb{Z}_1$	• $\boldsymbol{E}$ ∥ [100] and $\boldsymbol{B}$ ∥ [001] and strain ⊥ [001]<br>• $\boldsymbol{E}$ ∥ [010] and $\boldsymbol{B}$ ∥ [001] and strain ⊥ [001]<br>• $\boldsymbol{E}$ ⊥ [001] and $\boldsymbol{B}$ ∥ [001]
Pm' (6.20) $\mathbb{Z}_1$	• $\boldsymbol{E}$ ∥ [100] and $\boldsymbol{B}$ ∥ [100] and strain ⊥ [001]<br>• $\boldsymbol{E}$ ∥ [100] and $\boldsymbol{B}$ ∥ [010] and strain ⊥ [001]<br>• $\boldsymbol{E}$ ∥ [100] and $\boldsymbol{B}$ ⊥ [001]<br>• $\boldsymbol{E}$ ∥ [010] and $\boldsymbol{B}$ ∥ [100] and strain ⊥ [001]<br>• $\boldsymbol{E}$ ∥ [010] and $\boldsymbol{B}$ ∥ [010] and strain ⊥ [001]<br>• $\boldsymbol{E}$ ∥ [010] and $\boldsymbol{B}$ ⊥ [001]<br>• $\boldsymbol{E}$ ⊥ [001] and $\boldsymbol{B}$ ∥ [100]<br>• $\boldsymbol{E}$ ⊥ [001] and $\boldsymbol{B}$ ∥ [010]<br>• $\boldsymbol{E}$ ⊥ [001] and $\boldsymbol{B}$ ⊥ [001]
P_bm (6.22) $\mathbb{Z}_1$	• $\boldsymbol{E}$ ∥ [100] and strain ⊥ [001]<br>• $\boldsymbol{E}$ ∥ [010] and strain ⊥ [001]<br>• $\boldsymbol{E}$ ⊥ [001]
Pc' (7.26) $\mathbb{Z}_1$	• $\boldsymbol{E}$ ∥ [100] and $\boldsymbol{B}$ ∥ [100] and strain ⊥ [010]<br>• $\boldsymbol{E}$ ∥ [100] and $\boldsymbol{B}$ ∥ [001] and strain ⊥ [010]<br>• $\boldsymbol{E}$ ∥ [100] and $\boldsymbol{B}$ ⊥ [010]<br>• $\boldsymbol{E}$ ∥ [001] and $\boldsymbol{B}$ ∥ [100] and strain ⊥ [010]<br>• $\boldsymbol{E}$ ∥ [001] and $\boldsymbol{B}$ ∥ [001] and strain ⊥ [010]<br>• $\boldsymbol{E}$ ∥ [001] and $\boldsymbol{B}$ ⊥ [010]<br>• $\boldsymbol{E}$ ⊥ [010] and $\boldsymbol{B}$ ∥ [100]<br>• $\boldsymbol{E}$ ⊥ [010] and $\boldsymbol{B}$ ∥ [001]<br>• $\boldsymbol{E}$ ⊥ [010] and $\boldsymbol{B}$ ⊥ [010]

Continued on next page

...continued

Subgroup & SI group	Perturbation
Pc (7.24) $\mathbb{Z}_1$	• $\boldsymbol{E} \parallel [100]$ and $\boldsymbol{B} \parallel [010]$ and strain $\perp [010]$<br>• $\boldsymbol{E} \parallel [001]$ and $\boldsymbol{B} \parallel [010]$ and strain $\perp [010]$<br>• $\boldsymbol{E} \perp [010]$ and $\boldsymbol{B} \parallel [010]$
P_ac (7.27) $\mathbb{Z}_1$	• $\boldsymbol{E} \parallel [100]$ and strain $\perp [010]$<br>• $\boldsymbol{E} \parallel [001]$ and strain $\perp [010]$<br>• $\boldsymbol{E} \perp [010]$
$P2_1'$ (4.9) $\mathbb{Z}_1$	• $\boldsymbol{E} \parallel [100]$ and $\boldsymbol{B} \parallel [010]$ and strain $\perp [100]$<br>• $\boldsymbol{E} \parallel [100]$ and $\boldsymbol{B} \parallel [001]$ and strain $\perp [100]$<br>• $\boldsymbol{E} \parallel [100]$ and $\boldsymbol{B} \perp [100]$
$Pmc'2_1'$ (26.69) $\mathbb{Z}_1$	• $\boldsymbol{E} \parallel [100]$ and $\boldsymbol{B} \parallel [001]$
$Pm'n2_1'$ (31.125) $\mathbb{Z}_1$	• $\boldsymbol{E} \parallel [100]$ and $\boldsymbol{B} \parallel [010]$
$P2_1$ (4.7) $\mathbb{Z}_1$	• $\boldsymbol{E} \parallel [100]$ and $\boldsymbol{B} \parallel [100]$ and strain $\perp [100]$
$Pm'c'2_1$ (26.70) $\mathbb{Z}_1$	• $\boldsymbol{E} \parallel [100]$ and $\boldsymbol{B} \parallel [100]$
P_a2_1 (4.10) $\mathbb{Z}_1$	• $\boldsymbol{E} \parallel [100]$ and strain $\perp [100]$
P_amn2_1 (31.128) $\mathbb{Z}_1$	• $\boldsymbol{E} \parallel [100]$
$P2$ (3.1) $\mathbb{Z}_2$	• $\boldsymbol{E} \parallel [001]$ and $\boldsymbol{B} \parallel [001]$ and strain $\perp [001]$
$P2_1'$ (4.9) $\mathbb{Z}_1$	• $\boldsymbol{E} \parallel [001]$ and $\boldsymbol{B} \parallel [100]$ and strain $\perp [001]$<br>• $\boldsymbol{E} \parallel [001]$ and $\boldsymbol{B} \parallel [010]$ and strain $\perp [001]$<br>• $\boldsymbol{E} \parallel [001]$ and $\boldsymbol{B} \perp [001]$
P_b2 (3.5) $\mathbb{Z}_2$	• $\boldsymbol{E} \parallel [001]$ and strain $\perp [001]$
$P\bar{1}$ (2.4) $\mathbb{Z}_2 \times \mathbb{Z}_2 \times \mathbb{Z}_2 \times \mathbb{Z}_4$	• $\boldsymbol{B} \parallel [100]$ and strain in generic direction<br>• $\boldsymbol{B} \parallel [010]$ and strain in generic direction<br>• $\boldsymbol{B} \parallel [001]$ and strain in generic direction<br>• $\boldsymbol{B} \perp [100]$ and strain $\perp [010]$<br>• $\boldsymbol{B} \perp [100]$ and strain $\perp [001]$<br>• $\boldsymbol{B} \perp [100]$ and strain in generic direction<br>• $\boldsymbol{B} \perp [010]$ and strain $\perp [100]$<br>• $\boldsymbol{B} \perp [010]$ and strain $\perp [001]$<br>• $\boldsymbol{B} \perp [010]$ and strain in generic direction<br>• $\boldsymbol{B} \perp [001]$ and strain $\perp [100]$<br>• $\boldsymbol{B} \perp [001]$ and strain $\perp [010]$<br>• $\boldsymbol{B} \perp [001]$ and strain in generic direction<br>• $\boldsymbol{B}$ in generic direction
$P2/m$ (10.42) $\mathbb{Z}_2 \times \mathbb{Z}_2 \times \mathbb{Z}_2$	• $\boldsymbol{B} \parallel [001]$ and strain $\perp [001]$

Continued on next page

...continued

Subgroup & SI group	Perturbation
$P2_1'/m'$ (11.54) $\mathbb{Z}_2 \times \mathbb{Z}_2 \times \mathbb{Z}_4$	• $\boldsymbol{B} \parallel [100]$ and strain $\perp [001]$<br>• $\boldsymbol{B} \parallel [010]$ and strain $\perp [001]$<br>• $\boldsymbol{B} \perp [001]$
$P_S\bar{1}$ (2.7) $\mathbb{Z}_2 \times \mathbb{Z}_2 \times \mathbb{Z}_4$	• strain in generic direction
P_b2/m (10.48) $\mathbb{Z}_2 \times \mathbb{Z}_2$	• strain $\perp [001]$
Pc' (7.26) $\mathbb{Z}_1$	• $\boldsymbol{E} \parallel [010]$ and $\boldsymbol{B} \parallel [010]$ and strain $\perp [100]$<br>• $\boldsymbol{E} \parallel [010]$ and $\boldsymbol{B} \parallel [001]$ and strain $\perp [100]$<br>• $\boldsymbol{E} \parallel [010]$ and $\boldsymbol{B} \perp [100]$<br>• $\boldsymbol{E} \parallel [001]$ and $\boldsymbol{B} \parallel [010]$ and strain $\perp [100]$<br>• $\boldsymbol{E} \parallel [001]$ and $\boldsymbol{B} \parallel [001]$ and strain $\perp [100]$<br>• $\boldsymbol{E} \parallel [001]$ and $\boldsymbol{B} \perp [100]$<br>• $\boldsymbol{E} \perp [100]$ and $\boldsymbol{B} \parallel [010]$<br>• $\boldsymbol{E} \perp [100]$ and $\boldsymbol{B} \parallel [001]$<br>• $\boldsymbol{E} \perp [100]$ and $\boldsymbol{B} \perp [100]$
$Pb'a'2$ (32.138) $\mathbb{Z}_2$	• $\boldsymbol{E} \parallel [001]$ and $\boldsymbol{B} \parallel [001]$
$Pna'2_1'$ (33.147) $\mathbb{Z}_1$	• $\boldsymbol{E} \parallel [001]$ and $\boldsymbol{B} \parallel [010]$
$P2_1'/c'$ (14.79) $\mathbb{Z}_2 \times \mathbb{Z}_4$	• $\boldsymbol{B} \parallel [010]$ and strain $\perp [100]$<br>• $\boldsymbol{B} \parallel [001]$ and strain $\perp [100]$<br>• $\boldsymbol{B} \perp [100]$
Pc (7.24) $\mathbb{Z}_1$	• $\boldsymbol{E} \parallel [010]$ and $\boldsymbol{B} \parallel [100]$ and strain $\perp [100]$<br>• $\boldsymbol{E} \parallel [001]$ and $\boldsymbol{B} \parallel [100]$ and strain $\perp [100]$<br>• $\boldsymbol{E} \perp [100]$ and $\boldsymbol{B} \parallel [100]$
$Pna'2_1'$ (33.147) $\mathbb{Z}_1$	• $\boldsymbol{E} \parallel [001]$ and $\boldsymbol{B} \parallel [100]$
$P2_1/c$ (14.75) $\mathbb{Z}_2$	• $\boldsymbol{B} \parallel [100]$ and strain $\perp [100]$
P_ac (7.27) $\mathbb{Z}_1$	• $\boldsymbol{E} \parallel [010]$ and strain $\perp [100]$<br>• $\boldsymbol{E} \parallel [001]$ and strain $\perp [100]$<br>• $\boldsymbol{E} \perp [100]$
P_cnn2 (34.161) $\mathbb{Z}_2$	• $\boldsymbol{E} \parallel [001]$
P_a2_1/c (14.80) $\mathbb{Z}_2$	• strain $\perp [100]$
$P2_1'$ (4.9) $\mathbb{Z}_1$	• $\boldsymbol{E} \parallel [010]$ and $\boldsymbol{B} \parallel [100]$ and strain $\perp [010]$<br>• $\boldsymbol{E} \parallel [010]$ and $\boldsymbol{B} \parallel [001]$ and strain $\perp [010]$<br>• $\boldsymbol{E} \parallel [010]$ and $\boldsymbol{B} \perp [010]$
$P2_1'/c'$ (14.79) $\mathbb{Z}_2 \times \mathbb{Z}_4$	• $\boldsymbol{B} \parallel [100]$ and strain $\perp [010]$<br>• $\boldsymbol{B} \parallel [001]$ and strain $\perp [010]$<br>• $\boldsymbol{B} \perp [010]$

Continued on next page

…continued

Subgroup & SI group	Perturbation
$Pmc'2_1'$ (26.69) $\mathbb{Z}_1$	• $\boldsymbol{E} \parallel [010]$ and $\boldsymbol{B} \parallel [001]$
$Pb'a'm$ (55.357) $\mathbb{Z}_2 \times \mathbb{Z}_2 \times \mathbb{Z}_2$	• $\boldsymbol{B} \parallel [001]$
$Pm'n2_1'$ (31.125) $\mathbb{Z}_1$	• $\boldsymbol{E} \parallel [010]$ and $\boldsymbol{B} \parallel [100]$
$Pnm'a'$ (62.447) $\mathbb{Z}_2$	• $\boldsymbol{B} \parallel [100]$
$P2_1$ (4.7) $\mathbb{Z}_1$	• $\boldsymbol{E} \parallel [010]$ and $\boldsymbol{B} \parallel [010]$ and strain $\perp [010]$
$P2_1/c$ (14.75) $\mathbb{Z}_2$	• $\boldsymbol{B} \parallel [010]$ and strain $\perp [010]$
$Pm'c'2_1$ (26.70) $\mathbb{Z}_1$	• $\boldsymbol{E} \parallel [010]$ and $\boldsymbol{B} \parallel [010]$
$Pnm'a'$ (62.447) $\mathbb{Z}_2$	• $\boldsymbol{B} \parallel [010]$
P_a2_1 (4.10) $\mathbb{Z}_1$	• $\boldsymbol{E} \parallel [010]$ and strain $\perp [010]$
P_a2_1/c (14.80) $\mathbb{Z}_2$	• strain $\perp [010]$
P_amn2_1 (31.128) $\mathbb{Z}_1$	• $\boldsymbol{E} \parallel [010]$

Table. S 512: Subgroups of P_Bnnm (58.402) that can be realized by applying $\boldsymbol{E}/\boldsymbol{B}$ fields and/or uniaxial strains.

Subgroup & SI group	Perturbation
Pm (6.18) $\mathbb{Z}_1$	• $\boldsymbol{E} \parallel [100]$ and $\boldsymbol{B} \parallel [001]$ and strain $\perp [001]$<br>• $\boldsymbol{E} \parallel [010]$ and $\boldsymbol{B} \parallel [001]$ and strain $\perp [001]$<br>• $\boldsymbol{E} \perp [001]$ and $\boldsymbol{B} \parallel [001]$
Pm' (6.20) $\mathbb{Z}_1$	• $\boldsymbol{E} \parallel [100]$ and $\boldsymbol{B} \parallel [100]$ and strain $\perp [010]$<br>• $\boldsymbol{E} \parallel [100]$ and $\boldsymbol{B} \parallel [001]$ and strain $\perp [010]$<br>• $\boldsymbol{E} \parallel [100]$ and $\boldsymbol{B} \perp [010]$<br>• $\boldsymbol{E} \parallel [001]$ and $\boldsymbol{B} \parallel [100]$ and strain $\perp [010]$<br>• $\boldsymbol{E} \parallel [001]$ and $\boldsymbol{B} \parallel [001]$ and strain $\perp [010]$<br>• $\boldsymbol{E} \parallel [001]$ and $\boldsymbol{B} \perp [010]$<br>• $\boldsymbol{E} \perp [010]$ and $\boldsymbol{B} \parallel [100]$<br>• $\boldsymbol{E} \perp [010]$ and $\boldsymbol{B} \parallel [001]$<br>• $\boldsymbol{E} \perp [010]$ and $\boldsymbol{B} \perp [010]$
$P2'$ (3.3) $\mathbb{Z}_1$	• $\boldsymbol{E} \parallel [100]$ and $\boldsymbol{B} \parallel [010]$ and strain $\perp [100]$<br>• $\boldsymbol{E} \parallel [100]$ and $\boldsymbol{B} \parallel [001]$ and strain $\perp [100]$<br>• $\boldsymbol{E} \parallel [100]$ and $\boldsymbol{B} \perp [100]$
$Pm'm2'$ (25.59) $\mathbb{Z}_1$	• $\boldsymbol{E} \parallel [100]$ and $\boldsymbol{B} \parallel [001]$

Continued on next page

...continued

Subgroup & SI group	Perturbation
P_S1 (1.3) $\mathbb{Z}_1$	• $\boldsymbol{E} \parallel [100]$ and strain in generic direction<br>• $\boldsymbol{E} \parallel [010]$ and strain in generic direction<br>• $\boldsymbol{E} \parallel [001]$ and strain in generic direction<br>• $\boldsymbol{E} \perp [100]$ and strain $\perp [010]$<br>• $\boldsymbol{E} \perp [100]$ and strain $\perp [001]$<br>• $\boldsymbol{E} \perp [100]$ and strain in generic direction<br>• $\boldsymbol{E} \perp [010]$ and strain $\perp [100]$<br>• $\boldsymbol{E} \perp [010]$ and strain $\perp [001]$<br>• $\boldsymbol{E} \perp [010]$ and strain in generic direction<br>• $\boldsymbol{E} \perp [001]$ and strain $\perp [100]$<br>• $\boldsymbol{E} \perp [001]$ and strain $\perp [010]$<br>• $\boldsymbol{E} \perp [001]$ and strain in generic direction<br>• $\boldsymbol{E}$ in generic direction
Pc' (7.26) $\mathbb{Z}_1$	• $\boldsymbol{E} \parallel [100]$ and $\boldsymbol{B} \parallel [100]$ and strain $\perp [001]$<br>• $\boldsymbol{E} \parallel [100]$ and $\boldsymbol{B} \parallel [010]$ and strain $\perp [001]$<br>• $\boldsymbol{E} \parallel [100]$ and $\boldsymbol{B} \perp [001]$<br>• $\boldsymbol{E} \parallel [010]$ and $\boldsymbol{B} \parallel [100]$ and strain $\perp [001]$<br>• $\boldsymbol{E} \parallel [010]$ and $\boldsymbol{B} \parallel [010]$ and strain $\perp [001]$<br>• $\boldsymbol{E} \parallel [010]$ and $\boldsymbol{B} \perp [001]$<br>• $\boldsymbol{E} \perp [001]$ and $\boldsymbol{B} \parallel [100]$<br>• $\boldsymbol{E} \perp [001]$ and $\boldsymbol{B} \parallel [010]$<br>• $\boldsymbol{E} \perp [001]$ and $\boldsymbol{B} \perp [001]$
P_Cm (6.23) $\mathbb{Z}_1$	• $\boldsymbol{E} \parallel [100]$ and strain $\perp [001]$<br>• $\boldsymbol{E} \parallel [010]$ and strain $\perp [001]$<br>• $\boldsymbol{E} \perp [001]$
Pc (7.24) $\mathbb{Z}_1$	• $\boldsymbol{E} \parallel [100]$ and $\boldsymbol{B} \parallel [010]$ and strain $\perp [010]$<br>• $\boldsymbol{E} \parallel [001]$ and $\boldsymbol{B} \parallel [010]$ and strain $\perp [010]$<br>• $\boldsymbol{E} \perp [010]$ and $\boldsymbol{B} \parallel [010]$
P_cc (7.28) $\mathbb{Z}_1$	• $\boldsymbol{E} \parallel [100]$ and strain $\perp [010]$<br>• $\boldsymbol{E} \parallel [001]$ and strain $\perp [010]$<br>• $\boldsymbol{E} \perp [010]$
$Pnc'2'$ (30.114) $\mathbb{Z}_1$	• $\boldsymbol{E} \parallel [100]$ and $\boldsymbol{B} \parallel [010]$
$P2_1$ (4.7) $\mathbb{Z}_1$	• $\boldsymbol{E} \parallel [100]$ and $\boldsymbol{B} \parallel [100]$ and strain $\perp [100]$
P_C2_1 (4.12) $\mathbb{Z}_1$	• $\boldsymbol{E} \parallel [100]$ and strain $\perp [100]$
$Pm'c'2_1$ (26.70) $\mathbb{Z}_1$	• $\boldsymbol{E} \parallel [100]$ and $\boldsymbol{B} \parallel [100]$
P_Bmn2_1 (31.132) $\mathbb{Z}_1$	• $\boldsymbol{E} \parallel [100]$

Continued on next page

...continued

Subgroup & SI group	Perturbation
Pc' (7.26) $\mathbb{Z}_1$	• $\boldsymbol{E}$ ∥ [010] and $\boldsymbol{B}$ ∥ [010] and strain ⊥ [100]<br>• $\boldsymbol{E}$ ∥ [010] and $\boldsymbol{B}$ ∥ [001] and strain ⊥ [100]<br>• $\boldsymbol{E}$ ∥ [010] and $\boldsymbol{B}$ ⊥ [100]<br>• $\boldsymbol{E}$ ∥ [001] and $\boldsymbol{B}$ ∥ [010] and strain ⊥ [100]<br>• $\boldsymbol{E}$ ∥ [001] and $\boldsymbol{B}$ ∥ [001] and strain ⊥ [100]<br>• $\boldsymbol{E}$ ∥ [001] and $\boldsymbol{B}$ ⊥ [100]<br>• $\boldsymbol{E}$ ⊥ [100] and $\boldsymbol{B}$ ∥ [010]<br>• $\boldsymbol{E}$ ⊥ [100] and $\boldsymbol{B}$ ∥ [001]<br>• $\boldsymbol{E}$ ⊥ [100] and $\boldsymbol{B}$ ⊥ [100]
$P2_1'$ (4.9) $\mathbb{Z}_1$	• $\boldsymbol{E}$ ∥ [010] and $\boldsymbol{B}$ ∥ [100] and strain ⊥ [010]<br>• $\boldsymbol{E}$ ∥ [010] and $\boldsymbol{B}$ ∥ [001] and strain ⊥ [010]<br>• $\boldsymbol{E}$ ∥ [010] and $\boldsymbol{B}$ ⊥ [010]
$Pmc'2_1'$ (26.69) $\mathbb{Z}_1$	• $\boldsymbol{E}$ ∥ [010] and $\boldsymbol{B}$ ∥ [001]
$P2$ (3.1) $\mathbb{Z}_2$	• $\boldsymbol{E}$ ∥ [001] and $\boldsymbol{B}$ ∥ [001] and strain ⊥ [001]
$Pm'a'2$ (28.91) $\mathbb{Z}_2$	• $\boldsymbol{E}$ ∥ [001] and $\boldsymbol{B}$ ∥ [001]
$P\bar{1}$ (2.4) $\mathbb{Z}_2 \times \mathbb{Z}_2 \times \mathbb{Z}_2 \times \mathbb{Z}_4$	• $\boldsymbol{B}$ ∥ [100] and strain in generic direction<br>• $\boldsymbol{B}$ ∥ [010] and strain in generic direction<br>• $\boldsymbol{B}$ ∥ [001] and strain in generic direction<br>• $\boldsymbol{B}$ ⊥ [100] and strain ⊥ [010]<br>• $\boldsymbol{B}$ ⊥ [100] and strain ⊥ [001]<br>• $\boldsymbol{B}$ ⊥ [100] and strain in generic direction<br>• $\boldsymbol{B}$ ⊥ [010] and strain ⊥ [100]<br>• $\boldsymbol{B}$ ⊥ [010] and strain ⊥ [001]<br>• $\boldsymbol{B}$ ⊥ [010] and strain in generic direction<br>• $\boldsymbol{B}$ ⊥ [001] and strain ⊥ [100]<br>• $\boldsymbol{B}$ ⊥ [001] and strain ⊥ [010]<br>• $\boldsymbol{B}$ ⊥ [001] and strain in generic direction<br>• $\boldsymbol{B}$ in generic direction
$P2'/c'$ (13.69) $\mathbb{Z}_2 \times \mathbb{Z}_2 \times \mathbb{Z}_4$	• $\boldsymbol{B}$ ∥ [010] and strain ⊥ [100]<br>• $\boldsymbol{B}$ ∥ [001] and strain ⊥ [100]<br>• $\boldsymbol{B}$ ⊥ [100]
$P2_1'/m'$ (11.54) $\mathbb{Z}_2 \times \mathbb{Z}_2 \times \mathbb{Z}_4$	• $\boldsymbol{B}$ ∥ [100] and strain ⊥ [010]<br>• $\boldsymbol{B}$ ∥ [001] and strain ⊥ [010]<br>• $\boldsymbol{B}$ ⊥ [010]
$P2/m$ (10.42) $\mathbb{Z}_2 \times \mathbb{Z}_2 \times \mathbb{Z}_2$	• $\boldsymbol{B}$ ∥ [001] and strain ⊥ [001]
$Pm'ma'$ (51.296) $\mathbb{Z}_2 \times \mathbb{Z}_2 \times \mathbb{Z}_2$	• $\boldsymbol{B}$ ∥ [001]

Continued on next page

…continued

Subgroup & SI group	Perturbation
Pc (7.24) $\mathbb{Z}_1$	• $\boldsymbol{E} \parallel [010]$ and $\boldsymbol{B} \parallel [100]$ and strain $\perp [100]$<br>• $\boldsymbol{E} \parallel [001]$ and $\boldsymbol{B} \parallel [100]$ and strain $\perp [100]$<br>• $\boldsymbol{E} \perp [100]$ and $\boldsymbol{B} \parallel [100]$
P_Cc (7.30) $\mathbb{Z}_1$	• $\boldsymbol{E} \parallel [010]$ and strain $\perp [100]$<br>• $\boldsymbol{E} \parallel [001]$ and strain $\perp [100]$<br>• $\boldsymbol{E} \perp [100]$
$Pna'2_1'$ (33.147) $\mathbb{Z}_1$	• $\boldsymbol{E} \parallel [010]$ and $\boldsymbol{B} \parallel [100]$
$P2_1/c$ (14.75) $\mathbb{Z}_2$	• $\boldsymbol{B} \parallel [100]$ and strain $\perp [100]$
$P2_1$ (4.7) $\mathbb{Z}_1$	• $\boldsymbol{E} \parallel [010]$ and $\boldsymbol{B} \parallel [010]$ and strain $\perp [010]$
$Pc'a'2_1$ (29.103) $\mathbb{Z}_1$	• $\boldsymbol{E} \parallel [010]$ and $\boldsymbol{B} \parallel [010]$
P_a2_1 (4.10) $\mathbb{Z}_1$	• $\boldsymbol{E} \parallel [010]$ and strain $\perp [010]$
$P2_1/c$ (14.75) $\mathbb{Z}_2$	• $\boldsymbol{B} \parallel [010]$ and strain $\perp [010]$
P_Cmn2_1 (31.133) $\mathbb{Z}_1$	• $\boldsymbol{E} \parallel [010]$
$P2_1'$ (4.9) $\mathbb{Z}_1$	• $\boldsymbol{E} \parallel [001]$ and $\boldsymbol{B} \parallel [100]$ and strain $\perp [001]$<br>• $\boldsymbol{E} \parallel [001]$ and $\boldsymbol{B} \parallel [010]$ and strain $\perp [001]$<br>• $\boldsymbol{E} \parallel [001]$ and $\boldsymbol{B} \perp [001]$
$Pna'2_1'$ (33.147) $\mathbb{Z}_1$	• $\boldsymbol{E} \parallel [001]$ and $\boldsymbol{B} \parallel [010]$
P_C2 (3.6) $\mathbb{Z}_1$	• $\boldsymbol{E} \parallel [001]$ and strain $\perp [001]$
$P2_1'/c'$ (14.79) $\mathbb{Z}_2 \times \mathbb{Z}_4$	• $\boldsymbol{B} \parallel [100]$ and strain $\perp [001]$<br>• $\boldsymbol{B} \parallel [010]$ and strain $\perp [001]$<br>• $\boldsymbol{B} \perp [001]$
$Pm'n2_1'$ (31.125) $\mathbb{Z}_1$	• $\boldsymbol{E} \parallel [001]$ and $\boldsymbol{B} \parallel [100]$
P_Ann2 (34.162) $\mathbb{Z}_1$	• $\boldsymbol{E} \parallel [001]$
$Pnm'a'$ (62.447) $\mathbb{Z}_2$	• $\boldsymbol{B} \parallel [100]$
$Pb'c'n$ (60.422) $\mathbb{Z}_2$	• $\boldsymbol{B} \parallel [010]$
$P_S\bar{1}$ (2.7) $\mathbb{Z}_2 \times \mathbb{Z}_2 \times \mathbb{Z}_4$	• strain in generic direction
P_C2_1/c (14.84) $\mathbb{Z}_2$	• strain $\perp [100]$
P_c2_1/c (14.82) $\mathbb{Z}_2$	• strain $\perp [010]$
P_C2/m (10.49) $\mathbb{Z}_2 \times \mathbb{Z}_2$	• strain $\perp [001]$

Table. S 513: Subgroups of $P_{C}nnm$ (58.403) that can be realized by applying $\boldsymbol{E}/\boldsymbol{B}$ fields and/or uniaxial strains.

Subgroup & SI group	Perturbation
Pm (6.18) $\mathbb{Z}_1$	• $\boldsymbol{E}$ ∥ [100] and $\boldsymbol{B}$ ∥ [001] and strain ⊥ [001]<br>• $\boldsymbol{E}$ ∥ [010] and $\boldsymbol{B}$ ∥ [001] and strain ⊥ [001]<br>• $\boldsymbol{E}$ ⊥ [001] and $\boldsymbol{B}$ ∥ [001]
Pc' (7.26) $\mathbb{Z}_1$	• $\boldsymbol{E}$ ∥ [100] and $\boldsymbol{B}$ ∥ [100] and strain ⊥ [010]<br>• $\boldsymbol{E}$ ∥ [100] and $\boldsymbol{B}$ ∥ [001] and strain ⊥ [010]<br>• $\boldsymbol{E}$ ∥ [100] and $\boldsymbol{B}$ ⊥ [010]<br>• $\boldsymbol{E}$ ∥ [001] and $\boldsymbol{B}$ ∥ [100] and strain ⊥ [010]<br>• $\boldsymbol{E}$ ∥ [001] and $\boldsymbol{B}$ ∥ [001] and strain ⊥ [010]<br>• $\boldsymbol{E}$ ∥ [001] and $\boldsymbol{B}$ ⊥ [010]<br>• $\boldsymbol{E}$ ⊥ [010] and $\boldsymbol{B}$ ∥ [100]<br>• $\boldsymbol{E}$ ⊥ [010] and $\boldsymbol{B}$ ∥ [001]<br>• $\boldsymbol{E}$ ⊥ [010] and $\boldsymbol{B}$ ⊥ [010]
$P2'$ (3.3) $\mathbb{Z}_1$	• $\boldsymbol{E}$ ∥ [100] and $\boldsymbol{B}$ ∥ [010] and strain ⊥ [100]<br>• $\boldsymbol{E}$ ∥ [100] and $\boldsymbol{B}$ ∥ [001] and strain ⊥ [100]<br>• $\boldsymbol{E}$ ∥ [100] and $\boldsymbol{B}$ ⊥ [100]
$Pma'2'$ (28.90) $\mathbb{Z}_1$	• $\boldsymbol{E}$ ∥ [100] and $\boldsymbol{B}$ ∥ [001]
$P_{S}1$ (1.3) $\mathbb{Z}_1$	• $\boldsymbol{E}$ ∥ [100] and strain in generic direction<br>• $\boldsymbol{E}$ ∥ [010] and strain in generic direction<br>• $\boldsymbol{E}$ ∥ [001] and strain in generic direction<br>• $\boldsymbol{E}$ ⊥ [100] and strain ⊥ [010]<br>• $\boldsymbol{E}$ ⊥ [100] and strain ⊥ [001]<br>• $\boldsymbol{E}$ ⊥ [100] and strain in generic direction<br>• $\boldsymbol{E}$ ⊥ [010] and strain ⊥ [100]<br>• $\boldsymbol{E}$ ⊥ [010] and strain ⊥ [001]<br>• $\boldsymbol{E}$ ⊥ [010] and strain in generic direction<br>• $\boldsymbol{E}$ ⊥ [001] and strain ⊥ [100]<br>• $\boldsymbol{E}$ ⊥ [001] and strain ⊥ [010]<br>• $\boldsymbol{E}$ ⊥ [001] and strain in generic direction<br>• $\boldsymbol{E}$ in generic direction

Continued on next page

. . . continued

Subgroup & SI group	Perturbation
Pc' (7.26) $\mathbb{Z}_1$	• $\boldsymbol{E}$ ∥ [100] and $\boldsymbol{B}$ ∥ [100] and strain ⊥ [001]<br>• $\boldsymbol{E}$ ∥ [100] and $\boldsymbol{B}$ ∥ [010] and strain ⊥ [001]<br>• $\boldsymbol{E}$ ∥ [100] and $\boldsymbol{B}$ ⊥ [001]<br>• $\boldsymbol{E}$ ∥ [010] and $\boldsymbol{B}$ ∥ [100] and strain ⊥ [001]<br>• $\boldsymbol{E}$ ∥ [010] and $\boldsymbol{B}$ ∥ [010] and strain ⊥ [001]<br>• $\boldsymbol{E}$ ∥ [010] and $\boldsymbol{B}$ ⊥ [001]<br>• $\boldsymbol{E}$ ⊥ [001] and $\boldsymbol{B}$ ∥ [100]<br>• $\boldsymbol{E}$ ⊥ [001] and $\boldsymbol{B}$ ∥ [010]<br>• $\boldsymbol{E}$ ⊥ [001] and $\boldsymbol{B}$ ⊥ [001]
P_am (6.21) $\mathbb{Z}_1$	• $\boldsymbol{E}$ ∥ [100] and strain ⊥ [001]<br>• $\boldsymbol{E}$ ∥ [010] and strain ⊥ [001]<br>• $\boldsymbol{E}$ ⊥ [001]
Pc (7.24) $\mathbb{Z}_1$	• $\boldsymbol{E}$ ∥ [100] and $\boldsymbol{B}$ ∥ [010] and strain ⊥ [010]<br>• $\boldsymbol{E}$ ∥ [001] and $\boldsymbol{B}$ ∥ [010] and strain ⊥ [010]<br>• $\boldsymbol{E}$ ⊥ [010] and $\boldsymbol{B}$ ∥ [010]
P_Cc (7.30) $\mathbb{Z}_1$	• $\boldsymbol{E}$ ∥ [100] and strain ⊥ [010]<br>• $\boldsymbol{E}$ ∥ [001] and strain ⊥ [010]<br>• $\boldsymbol{E}$ ⊥ [010]
$Pn'n2'$ (34.158) $\mathbb{Z}_1$	• $\boldsymbol{E}$ ∥ [100] and $\boldsymbol{B}$ ∥ [010]
$P2_1$ (4.7) $\mathbb{Z}_1$	• $\boldsymbol{E}$ ∥ [100] and $\boldsymbol{B}$ ∥ [100] and strain ⊥ [100]
P_C2_1 (4.12) $\mathbb{Z}_1$	• $\boldsymbol{E}$ ∥ [100] and strain ⊥ [100]
$Pn'a'2_1$ (33.148) $\mathbb{Z}_1$	• $\boldsymbol{E}$ ∥ [100] and $\boldsymbol{B}$ ∥ [100]
P_Amn2_1 (31.131) $\mathbb{Z}_1$	• $\boldsymbol{E}$ ∥ [100]
Pc' (7.26) $\mathbb{Z}_1$	• $\boldsymbol{E}$ ∥ [010] and $\boldsymbol{B}$ ∥ [010] and strain ⊥ [100]<br>• $\boldsymbol{E}$ ∥ [010] and $\boldsymbol{B}$ ∥ [001] and strain ⊥ [100]<br>• $\boldsymbol{E}$ ∥ [010] and $\boldsymbol{B}$ ⊥ [100]<br>• $\boldsymbol{E}$ ∥ [001] and $\boldsymbol{B}$ ∥ [010] and strain ⊥ [100]<br>• $\boldsymbol{E}$ ∥ [001] and $\boldsymbol{B}$ ∥ [001] and strain ⊥ [100]<br>• $\boldsymbol{E}$ ∥ [001] and $\boldsymbol{B}$ ⊥ [100]<br>• $\boldsymbol{E}$ ⊥ [100] and $\boldsymbol{B}$ ∥ [010]<br>• $\boldsymbol{E}$ ⊥ [100] and $\boldsymbol{B}$ ∥ [001]<br>• $\boldsymbol{E}$ ⊥ [100] and $\boldsymbol{B}$ ⊥ [100]
$P2'$ (3.3) $\mathbb{Z}_1$	• $\boldsymbol{E}$ ∥ [010] and $\boldsymbol{B}$ ∥ [100] and strain ⊥ [010]<br>• $\boldsymbol{E}$ ∥ [010] and $\boldsymbol{B}$ ∥ [001] and strain ⊥ [010]<br>• $\boldsymbol{E}$ ∥ [010] and $\boldsymbol{B}$ ⊥ [010]
$Pma'2'$ (28.90) $\mathbb{Z}_1$	• $\boldsymbol{E}$ ∥ [010] and $\boldsymbol{B}$ ∥ [001]

Continued on next page

...continued

Subgroup & SI group	Perturbation
$P2$ (3.1) $\mathbb{Z}_2$	• $\boldsymbol{E}$ ∥ [001] and $\boldsymbol{B}$ ∥ [001] and strain ⊥ [001]
$Pc'c'2$ (27.81) $\mathbb{Z}_2$	• $\boldsymbol{E}$ ∥ [001] and $\boldsymbol{B}$ ∥ [001]
$P\bar{1}$ (2.4) $\mathbb{Z}_2 \times \mathbb{Z}_2 \times \mathbb{Z}_2 \times \mathbb{Z}_4$	• $\boldsymbol{B}$ ∥ [100] and strain in generic direction<br>• $\boldsymbol{B}$ ∥ [010] and strain in generic direction<br>• $\boldsymbol{B}$ ∥ [001] and strain in generic direction<br>• $\boldsymbol{B}$ ⊥ [100] and strain ⊥ [010]<br>• $\boldsymbol{B}$ ⊥ [100] and strain ⊥ [001]<br>• $\boldsymbol{B}$ ⊥ [100] and strain in generic direction<br>• $\boldsymbol{B}$ ⊥ [010] and strain ⊥ [100]<br>• $\boldsymbol{B}$ ⊥ [010] and strain ⊥ [001]<br>• $\boldsymbol{B}$ ⊥ [010] and strain in generic direction<br>• $\boldsymbol{B}$ ⊥ [001] and strain ⊥ [100]<br>• $\boldsymbol{B}$ ⊥ [001] and strain ⊥ [010]<br>• $\boldsymbol{B}$ ⊥ [001] and strain in generic direction<br>• $\boldsymbol{B}$ in generic direction
$P2'/c'$ (13.69) $\mathbb{Z}_2 \times \mathbb{Z}_2 \times \mathbb{Z}_4$	• $\boldsymbol{B}$ ∥ [010] and strain ⊥ [100]<br>• $\boldsymbol{B}$ ∥ [001] and strain ⊥ [100]<br>• $\boldsymbol{B}$ ⊥ [100]
$P2'/c'$ (13.69) $\mathbb{Z}_2 \times \mathbb{Z}_2 \times \mathbb{Z}_4$	• $\boldsymbol{B}$ ∥ [100] and strain ⊥ [010]<br>• $\boldsymbol{B}$ ∥ [001] and strain ⊥ [010]<br>• $\boldsymbol{B}$ ⊥ [010]
$P2/m$ (10.42) $\mathbb{Z}_2 \times \mathbb{Z}_2 \times \mathbb{Z}_2$	• $\boldsymbol{B}$ ∥ [001] and strain ⊥ [001]
$Pc'c'm$ (49.269) $\mathbb{Z}_2 \times \mathbb{Z}_2$	• $\boldsymbol{B}$ ∥ [001]
Pc (7.24) $\mathbb{Z}_1$	• $\boldsymbol{E}$ ∥ [010] and $\boldsymbol{B}$ ∥ [100] and strain ⊥ [100]<br>• $\boldsymbol{E}$ ∥ [001] and $\boldsymbol{B}$ ∥ [100] and strain ⊥ [100]<br>• $\boldsymbol{E}$ ⊥ [100] and $\boldsymbol{B}$ ∥ [100]
P_Cc (7.30) $\mathbb{Z}_1$	• $\boldsymbol{E}$ ∥ [010] and strain ⊥ [100]<br>• $\boldsymbol{E}$ ∥ [001] and strain ⊥ [100]<br>• $\boldsymbol{E}$ ⊥ [100]
$Pn'n2'$ (34.158) $\mathbb{Z}_1$	• $\boldsymbol{E}$ ∥ [010] and $\boldsymbol{B}$ ∥ [100]
$P2_1/c$ (14.75) $\mathbb{Z}_2$	• $\boldsymbol{B}$ ∥ [100] and strain ⊥ [100]
$P2_1$ (4.7) $\mathbb{Z}_1$	• $\boldsymbol{E}$ ∥ [010] and $\boldsymbol{B}$ ∥ [010] and strain ⊥ [010]
$Pn'a'2_1$ (33.148) $\mathbb{Z}_1$	• $\boldsymbol{E}$ ∥ [010] and $\boldsymbol{B}$ ∥ [010]
P_C2_1 (4.12) $\mathbb{Z}_1$	• $\boldsymbol{E}$ ∥ [010] and strain ⊥ [010]
$P2_1/c$ (14.75) $\mathbb{Z}_2$	• $\boldsymbol{B}$ ∥ [010] and strain ⊥ [010]

Continued on next page

…continued

Subgroup & SI group	Perturbation
$P_A mn2_1$ (31.131) $\mathbb{Z}_1$	• $\boldsymbol{E} \parallel [010]$
$P2'$ (3.3) $\mathbb{Z}_1$	• $\boldsymbol{E} \parallel [001]$ and $\boldsymbol{B} \parallel [100]$ and strain $\perp [001]$<br>• $\boldsymbol{E} \parallel [001]$ and $\boldsymbol{B} \parallel [010]$ and strain $\perp [001]$<br>• $\boldsymbol{E} \parallel [001]$ and $\boldsymbol{B} \perp [001]$
$Pnc'2'$ (30.114) $\mathbb{Z}_1$	• $\boldsymbol{E} \parallel [001]$ and $\boldsymbol{B} \parallel [010]$
P_a2 (3.4) $\mathbb{Z}_2$	• $\boldsymbol{E} \parallel [001]$ and strain $\perp [001]$
$P2'/c'$ (13.69) $\mathbb{Z}_2 \times \mathbb{Z}_2 \times \mathbb{Z}_4$	• $\boldsymbol{B} \parallel [100]$ and strain $\perp [001]$<br>• $\boldsymbol{B} \parallel [010]$ and strain $\perp [001]$<br>• $\boldsymbol{B} \perp [001]$
$Pnc'2'$ (30.114) $\mathbb{Z}_1$	• $\boldsymbol{E} \parallel [001]$ and $\boldsymbol{B} \parallel [100]$
$P_C nn2$ (34.163) $\mathbb{Z}_2$	• $\boldsymbol{E} \parallel [001]$
$Pn'na'$ (52.312) $\mathbb{Z}_2$	• $\boldsymbol{B} \parallel [100]$
$Pn'na'$ (52.312) $\mathbb{Z}_2$	• $\boldsymbol{B} \parallel [010]$
$P_S\bar{1}$ (2.7) $\mathbb{Z}_2 \times \mathbb{Z}_2 \times \mathbb{Z}_4$	• strain in generic direction
$P_C 2_1/c$ (14.84) $\mathbb{Z}_2$	• strain $\perp [100]$
$P_C 2_1/c$ (14.84) $\mathbb{Z}_2$	• strain $\perp [010]$
$P_a 2/m$ (10.47) $\mathbb{Z}_2 \times \mathbb{Z}_2 \times \mathbb{Z}_2$	• strain $\perp [001]$

Table. S 514: Subgroups of $P_I nnm$ (58.404) that can be realized by applying $\boldsymbol{E}/\boldsymbol{B}$ fields and/or uniaxial strains.

Subgroup & SI group	Perturbation
Pm (6.18) $\mathbb{Z}_1$	• $\boldsymbol{E} \parallel [100]$ and $\boldsymbol{B} \parallel [001]$ and strain $\perp [001]$<br>• $\boldsymbol{E} \parallel [010]$ and $\boldsymbol{B} \parallel [001]$ and strain $\perp [001]$<br>• $\boldsymbol{E} \perp [001]$ and $\boldsymbol{B} \parallel [001]$

Continued on next page

...continued

Subgroup & SI group	Perturbation
Pm' (6.20) $\mathbb{Z}_1$	• $\boldsymbol{E}$ ∥ [100] and $\boldsymbol{B}$ ∥ [100] and strain ⊥ [010]<br>• $\boldsymbol{E}$ ∥ [100] and $\boldsymbol{B}$ ∥ [001] and strain ⊥ [010]<br>• $\boldsymbol{E}$ ∥ [100] and $\boldsymbol{B}$ ⊥ [010]<br>• $\boldsymbol{E}$ ∥ [001] and $\boldsymbol{B}$ ∥ [100] and strain ⊥ [010]<br>• $\boldsymbol{E}$ ∥ [001] and $\boldsymbol{B}$ ∥ [001] and strain ⊥ [010]<br>• $\boldsymbol{E}$ ∥ [001] and $\boldsymbol{B}$ ⊥ [010]<br>• $\boldsymbol{E}$ ⊥ [010] and $\boldsymbol{B}$ ∥ [100]<br>• $\boldsymbol{E}$ ⊥ [010] and $\boldsymbol{B}$ ∥ [001]<br>• $\boldsymbol{E}$ ⊥ [010] and $\boldsymbol{B}$ ⊥ [010]
$P2'$ (3.3) $\mathbb{Z}_1$	• $\boldsymbol{E}$ ∥ [100] and $\boldsymbol{B}$ ∥ [010] and strain ⊥ [100]<br>• $\boldsymbol{E}$ ∥ [100] and $\boldsymbol{B}$ ∥ [001] and strain ⊥ [100]<br>• $\boldsymbol{E}$ ∥ [100] and $\boldsymbol{B}$ ⊥ [100]
$Pm'm2'$ (25.59) $\mathbb{Z}_1$	• $\boldsymbol{E}$ ∥ [100] and $\boldsymbol{B}$ ∥ [001]
P_S1 (1.3) $\mathbb{Z}_1$	• $\boldsymbol{E}$ ∥ [100] and strain in generic direction<br>• $\boldsymbol{E}$ ∥ [010] and strain in generic direction<br>• $\boldsymbol{E}$ ∥ [001] and strain in generic direction<br>• $\boldsymbol{E}$ ⊥ [100] and strain ⊥ [010]<br>• $\boldsymbol{E}$ ⊥ [100] and strain ⊥ [001]<br>• $\boldsymbol{E}$ ⊥ [100] and strain in generic direction<br>• $\boldsymbol{E}$ ⊥ [010] and strain ⊥ [100]<br>• $\boldsymbol{E}$ ⊥ [010] and strain ⊥ [001]<br>• $\boldsymbol{E}$ ⊥ [010] and strain in generic direction<br>• $\boldsymbol{E}$ ⊥ [001] and strain ⊥ [100]<br>• $\boldsymbol{E}$ ⊥ [001] and strain ⊥ [010]<br>• $\boldsymbol{E}$ ⊥ [001] and strain in generic direction<br>• $\boldsymbol{E}$ in generic direction
Pc' (7.26) $\mathbb{Z}_1$	• $\boldsymbol{E}$ ∥ [100] and $\boldsymbol{B}$ ∥ [100] and strain ⊥ [001]<br>• $\boldsymbol{E}$ ∥ [100] and $\boldsymbol{B}$ ∥ [010] and strain ⊥ [001]<br>• $\boldsymbol{E}$ ∥ [100] and $\boldsymbol{B}$ ⊥ [001]<br>• $\boldsymbol{E}$ ∥ [010] and $\boldsymbol{B}$ ∥ [100] and strain ⊥ [001]<br>• $\boldsymbol{E}$ ∥ [010] and $\boldsymbol{B}$ ∥ [010] and strain ⊥ [001]<br>• $\boldsymbol{E}$ ∥ [010] and $\boldsymbol{B}$ ⊥ [001]<br>• $\boldsymbol{E}$ ⊥ [001] and $\boldsymbol{B}$ ∥ [100]<br>• $\boldsymbol{E}$ ⊥ [001] and $\boldsymbol{B}$ ∥ [010]<br>• $\boldsymbol{E}$ ⊥ [001] and $\boldsymbol{B}$ ⊥ [001]
P_Cm (6.23) $\mathbb{Z}_1$	• $\boldsymbol{E}$ ∥ [100] and strain ⊥ [001]<br>• $\boldsymbol{E}$ ∥ [010] and strain ⊥ [001]<br>• $\boldsymbol{E}$ ⊥ [001]

Continued on next page

...continued

Subgroup & SI group	Perturbation
Pc (7.24) $\mathbb{Z}_1$	• $\boldsymbol{E} \parallel [100]$ and $\boldsymbol{B} \parallel [010]$ and strain $\perp [010]$<br>• $\boldsymbol{E} \parallel [001]$ and $\boldsymbol{B} \parallel [010]$ and strain $\perp [010]$<br>• $\boldsymbol{E} \perp [010]$ and $\boldsymbol{B} \parallel [010]$
P_Ac (7.31) $\mathbb{Z}_1$	• $\boldsymbol{E} \parallel [100]$ and strain $\perp [010]$<br>• $\boldsymbol{E} \parallel [001]$ and strain $\perp [010]$<br>• $\boldsymbol{E} \perp [010]$
$Pn'n2'$ (34.158) $\mathbb{Z}_1$	• $\boldsymbol{E} \parallel [100]$ and $\boldsymbol{B} \parallel [010]$
$P2_1$ (4.7) $\mathbb{Z}_1$	• $\boldsymbol{E} \parallel [100]$ and $\boldsymbol{B} \parallel [100]$ and strain $\perp [100]$
P_C2_1 (4.12) $\mathbb{Z}_1$	• $\boldsymbol{E} \parallel [100]$ and strain $\perp [100]$
$Pm'n'2_1$ (31.127) $\mathbb{Z}_1$	• $\boldsymbol{E} \parallel [100]$ and $\boldsymbol{B} \parallel [100]$
P_Imn2_1 (31.134) $\mathbb{Z}_1$	• $\boldsymbol{E} \parallel [100]$
Pm' (6.20) $\mathbb{Z}_1$	• $\boldsymbol{E} \parallel [010]$ and $\boldsymbol{B} \parallel [010]$ and strain $\perp [100]$<br>• $\boldsymbol{E} \parallel [010]$ and $\boldsymbol{B} \parallel [001]$ and strain $\perp [100]$<br>• $\boldsymbol{E} \parallel [010]$ and $\boldsymbol{B} \perp [100]$<br>• $\boldsymbol{E} \parallel [001]$ and $\boldsymbol{B} \parallel [010]$ and strain $\perp [100]$<br>• $\boldsymbol{E} \parallel [001]$ and $\boldsymbol{B} \parallel [001]$ and strain $\perp [100]$<br>• $\boldsymbol{E} \parallel [001]$ and $\boldsymbol{B} \perp [100]$<br>• $\boldsymbol{E} \perp [100]$ and $\boldsymbol{B} \parallel [010]$<br>• $\boldsymbol{E} \perp [100]$ and $\boldsymbol{B} \parallel [001]$<br>• $\boldsymbol{E} \perp [100]$ and $\boldsymbol{B} \perp [100]$
$P2'$ (3.3) $\mathbb{Z}_1$	• $\boldsymbol{E} \parallel [010]$ and $\boldsymbol{B} \parallel [100]$ and strain $\perp [010]$<br>• $\boldsymbol{E} \parallel [010]$ and $\boldsymbol{B} \parallel [001]$ and strain $\perp [010]$<br>• $\boldsymbol{E} \parallel [010]$ and $\boldsymbol{B} \perp [010]$
$Pm'm2'$ (25.59) $\mathbb{Z}_1$	• $\boldsymbol{E} \parallel [010]$ and $\boldsymbol{B} \parallel [001]$
$P2$ (3.1) $\mathbb{Z}_2$	• $\boldsymbol{E} \parallel [001]$ and $\boldsymbol{B} \parallel [001]$ and strain $\perp [001]$
$Pm'm'2$ (25.60) $\mathbb{Z}_2$	• $\boldsymbol{E} \parallel [001]$ and $\boldsymbol{B} \parallel [001]$

Continued on next page

...continued

Subgroup & SI group	Perturbation
$P\bar{1}$ (2.4) $\mathbb{Z}_2 \times \mathbb{Z}_2 \times \mathbb{Z}_2 \times \mathbb{Z}_4$	• $\boldsymbol{B} \parallel [100]$ and strain in generic direction<br>• $\boldsymbol{B} \parallel [010]$ and strain in generic direction<br>• $\boldsymbol{B} \parallel [001]$ and strain in generic direction<br>• $\boldsymbol{B} \perp [100]$ and strain $\perp [010]$<br>• $\boldsymbol{B} \perp [100]$ and strain $\perp [001]$<br>• $\boldsymbol{B} \perp [100]$ and strain in generic direction<br>• $\boldsymbol{B} \perp [010]$ and strain $\perp [100]$<br>• $\boldsymbol{B} \perp [010]$ and strain $\perp [001]$<br>• $\boldsymbol{B} \perp [010]$ and strain in generic direction<br>• $\boldsymbol{B} \perp [001]$ and strain $\perp [100]$<br>• $\boldsymbol{B} \perp [001]$ and strain $\perp [010]$<br>• $\boldsymbol{B} \perp [001]$ and strain in generic direction<br>• $\boldsymbol{B}$ in generic direction
$P2'/m'$ (10.46) $\mathbb{Z}_2 \times \mathbb{Z}_2 \times \mathbb{Z}_2 \times \mathbb{Z}_4$	• $\boldsymbol{B} \parallel [010]$ and strain $\perp [100]$<br>• $\boldsymbol{B} \parallel [001]$ and strain $\perp [100]$<br>• $\boldsymbol{B} \perp [100]$
$P2'/m'$ (10.46) $\mathbb{Z}_2 \times \mathbb{Z}_2 \times \mathbb{Z}_2 \times \mathbb{Z}_4$	• $\boldsymbol{B} \parallel [100]$ and strain $\perp [010]$<br>• $\boldsymbol{B} \parallel [001]$ and strain $\perp [010]$<br>• $\boldsymbol{B} \perp [010]$
$P2/m$ (10.42) $\mathbb{Z}_2 \times \mathbb{Z}_2 \times \mathbb{Z}_2$	• $\boldsymbol{B} \parallel [001]$ and strain $\perp [001]$
$Pm'm'm$ (47.252) $\mathbb{Z}_2 \times \mathbb{Z}_2 \times \mathbb{Z}_2$	• $\boldsymbol{B} \parallel [001]$
Pc (7.24) $\mathbb{Z}_1$	• $\boldsymbol{E} \parallel [010]$ and $\boldsymbol{B} \parallel [100]$ and strain $\perp [100]$<br>• $\boldsymbol{E} \parallel [001]$ and $\boldsymbol{B} \parallel [100]$ and strain $\perp [100]$<br>• $\boldsymbol{E} \perp [100]$ and $\boldsymbol{B} \parallel [100]$
P_Ac (7.31) $\mathbb{Z}_1$	• $\boldsymbol{E} \parallel [010]$ and strain $\perp [100]$<br>• $\boldsymbol{E} \parallel [001]$ and strain $\perp [100]$<br>• $\boldsymbol{E} \perp [100]$
$Pn'n2'$ (34.158) $\mathbb{Z}_1$	• $\boldsymbol{E} \parallel [010]$ and $\boldsymbol{B} \parallel [100]$
$P2_1/c$ (14.75) $\mathbb{Z}_2$	• $\boldsymbol{B} \parallel [100]$ and strain $\perp [100]$
$P2_1$ (4.7) $\mathbb{Z}_1$	• $\boldsymbol{E} \parallel [010]$ and $\boldsymbol{B} \parallel [010]$ and strain $\perp [010]$
$Pm'n'2_1$ (31.127) $\mathbb{Z}_1$	• $\boldsymbol{E} \parallel [010]$ and $\boldsymbol{B} \parallel [010]$
P_C2_1 (4.12) $\mathbb{Z}_1$	• $\boldsymbol{E} \parallel [010]$ and strain $\perp [010]$
$P2_1/c$ (14.75) $\mathbb{Z}_2$	• $\boldsymbol{B} \parallel [010]$ and strain $\perp [010]$
P_Imn2_1 (31.134) $\mathbb{Z}_1$	• $\boldsymbol{E} \parallel [010]$

Continued on next page

...continued

Subgroup & SI group	Perturbation
$P2_1'$ (4.9) $\mathbb{Z}_1$	• $\boldsymbol{E} \parallel [001]$ and $\boldsymbol{B} \parallel [100]$ and strain $\perp [001]$<br>• $\boldsymbol{E} \parallel [001]$ and $\boldsymbol{B} \parallel [010]$ and strain $\perp [001]$<br>• $\boldsymbol{E} \parallel [001]$ and $\boldsymbol{B} \perp [001]$
$Pm'n2_1'$ (31.125) $\mathbb{Z}_1$	• $\boldsymbol{E} \parallel [001]$ and $\boldsymbol{B} \parallel [010]$
P_C2 (3.6) $\mathbb{Z}_1$	• $\boldsymbol{E} \parallel [001]$ and strain $\perp [001]$
$P2_1'/c'$ (14.79) $\mathbb{Z}_2 \times \mathbb{Z}_4$	• $\boldsymbol{B} \parallel [100]$ and strain $\perp [001]$<br>• $\boldsymbol{B} \parallel [010]$ and strain $\perp [001]$<br>• $\boldsymbol{B} \perp [001]$
$Pm'n2_1'$ (31.125) $\mathbb{Z}_1$	• $\boldsymbol{E} \parallel [001]$ and $\boldsymbol{B} \parallel [100]$
P_Inn2 (34.164) $\mathbb{Z}_1$	• $\boldsymbol{E} \parallel [001]$
$Pnn'm'$ (58.398) $\mathbb{Z}_2$	• $\boldsymbol{B} \parallel [100]$
$Pnn'm'$ (58.398) $\mathbb{Z}_2$	• $\boldsymbol{B} \parallel [010]$
$P_S\bar{1}$ (2.7) $\mathbb{Z}_2 \times \mathbb{Z}_2 \times \mathbb{Z}_4$	• strain in generic direction
P_A2_1/c (14.83) $\mathbb{Z}_2$	• strain $\perp [100]$
P_A2_1/c (14.83) $\mathbb{Z}_2$	• strain $\perp [010]$
P_C2/m (10.49) $\mathbb{Z}_2 \times \mathbb{Z}_2$	• strain $\perp [001]$

Table. S 515: Subgroups of $Pmmn$ (59.405) that can be realized by applying $\boldsymbol{E}/\boldsymbol{B}$ fields and/or uniaxial strains.

Subgroup & SI group	Perturbation
Pm (6.18) $\mathbb{Z}_1$	• $\boldsymbol{E} \parallel [100]$ and strain $\perp [010]$<br>• $\boldsymbol{E} \parallel [100]$ and $\boldsymbol{B} \parallel [010]$<br>• $\boldsymbol{E} \parallel [001]$ and strain $\perp [010]$<br>• $\boldsymbol{E} \parallel [001]$ and $\boldsymbol{B} \parallel [010]$<br>• $\boldsymbol{E} \perp [010]$
Pc (7.24) $\mathbb{Z}_1$	• $\boldsymbol{E} \parallel [100]$ and strain $\perp [001]$<br>• $\boldsymbol{E} \parallel [100]$ and $\boldsymbol{B} \parallel [001]$<br>• $\boldsymbol{E} \parallel [010]$ and strain $\perp [001]$<br>• $\boldsymbol{E} \parallel [010]$ and $\boldsymbol{B} \parallel [001]$<br>• $\boldsymbol{E} \perp [001]$

Continued on next page

...continued

Subgroup & SI group	Perturbation
$P2_1$ (4.7) $\mathbb{Z}_1$	• $\boldsymbol{E}$ ∥ [100] and strain ⊥ [100]<br>• $\boldsymbol{E}$ ∥ [100] and $\boldsymbol{B}$ ∥ [100]
$Pmn2_1$ (31.123) $\mathbb{Z}_1$	• $\boldsymbol{E}$ ∥ [100]
$P2_1$ (4.7) $\mathbb{Z}_1$	• $\boldsymbol{E}$ ∥ [010] and strain ⊥ [010]<br>• $\boldsymbol{E}$ ∥ [010] and $\boldsymbol{B}$ ∥ [010]
$P\bar{1}$ (2.4) $\mathbb{Z}_2 \times \mathbb{Z}_2 \times \mathbb{Z}_2 \times \mathbb{Z}_4$	• strain in generic direction<br>• $\boldsymbol{B}$ ∥ [100] and strain ⊥ [010]<br>• $\boldsymbol{B}$ ∥ [100] and strain ⊥ [001]<br>• $\boldsymbol{B}$ ∥ [010] and strain ⊥ [100]<br>• $\boldsymbol{B}$ ∥ [010] and strain ⊥ [001]<br>• $\boldsymbol{B}$ ∥ [001] and strain ⊥ [100]<br>• $\boldsymbol{B}$ ∥ [001] and strain ⊥ [010]<br>• $\boldsymbol{B}$ in generic direction
$P2_1/m$ (11.50) $\mathbb{Z}_2$	• strain ⊥ [010]<br>• $\boldsymbol{B}$ ∥ [010]
Pm (6.18) $\mathbb{Z}_1$	• $\boldsymbol{E}$ ∥ [010] and strain ⊥ [100]<br>• $\boldsymbol{E}$ ∥ [010] and $\boldsymbol{B}$ ∥ [100]<br>• $\boldsymbol{E}$ ∥ [001] and strain ⊥ [100]<br>• $\boldsymbol{E}$ ∥ [001] and $\boldsymbol{B}$ ∥ [100]<br>• $\boldsymbol{E}$ ⊥ [100]
$Pmn2_1$ (31.123) $\mathbb{Z}_1$	• $\boldsymbol{E}$ ∥ [010]
$P2_1/m$ (11.50) $\mathbb{Z}_2$	• strain ⊥ [100]<br>• $\boldsymbol{B}$ ∥ [100]
$P2$ (3.1) $\mathbb{Z}_2$	• $\boldsymbol{E}$ ∥ [001] and strain ⊥ [001]<br>• $\boldsymbol{E}$ ∥ [001] and $\boldsymbol{B}$ ∥ [001]
$P2/c$ (13.65) $\mathbb{Z}_2 \times \mathbb{Z}_2$	• strain ⊥ [001]<br>• $\boldsymbol{B}$ ∥ [001]
$Pmm2$ (25.57) $\mathbb{Z}_1$	• $\boldsymbol{E}$ ∥ [001]

Table. S 516: Subgroups of $Pmmn1'$ (59.406) that can be realized by applying $\boldsymbol{E}/\boldsymbol{B}$ fields and/or uniaxial strains.

Subgroup & SI group	Perturbation

Continued on next page

. . . continued

Subgroup & SI group	Perturbation
$P11'$ (1.2) $\mathbb{Z}_1$	• $\boldsymbol{E}$ ∥ [100] and strain in generic direction<br>• $\boldsymbol{E}$ ∥ [010] and strain in generic direction<br>• $\boldsymbol{E}$ ∥ [001] and strain in generic direction<br>• $\boldsymbol{E} \perp$ [100] and strain $\perp$ [010]<br>• $\boldsymbol{E} \perp$ [100] and strain $\perp$ [001]<br>• $\boldsymbol{E} \perp$ [100] and strain in generic direction<br>• $\boldsymbol{E} \perp$ [010] and strain $\perp$ [100]<br>• $\boldsymbol{E} \perp$ [010] and strain $\perp$ [001]<br>• $\boldsymbol{E} \perp$ [010] and strain in generic direction<br>• $\boldsymbol{E} \perp$ [001] and strain $\perp$ [100]<br>• $\boldsymbol{E} \perp$ [001] and strain $\perp$ [010]<br>• $\boldsymbol{E} \perp$ [001] and strain in generic direction<br>• $\boldsymbol{E}$ in generic direction
Pm' (6.20) $\mathbb{Z}_1$	• $\boldsymbol{E}$ ∥ [100] and $\boldsymbol{B}$ ∥ [100] and strain $\perp$ [010]<br>• $\boldsymbol{E}$ ∥ [100] and $\boldsymbol{B}$ ∥ [001] and strain $\perp$ [010]<br>• $\boldsymbol{E}$ ∥ [100] and $\boldsymbol{B} \perp$ [010]<br>• $\boldsymbol{E}$ ∥ [001] and $\boldsymbol{B}$ ∥ [100] and strain $\perp$ [010]<br>• $\boldsymbol{E}$ ∥ [001] and $\boldsymbol{B}$ ∥ [001] and strain $\perp$ [010]<br>• $\boldsymbol{E}$ ∥ [001] and $\boldsymbol{B} \perp$ [010]<br>• $\boldsymbol{E} \perp$ [010] and $\boldsymbol{B}$ ∥ [100]<br>• $\boldsymbol{E} \perp$ [010] and $\boldsymbol{B}$ ∥ [001]<br>• $\boldsymbol{E} \perp$ [010] and $\boldsymbol{B} \perp$ [010]
Pm (6.18) $\mathbb{Z}_1$	• $\boldsymbol{E}$ ∥ [100] and $\boldsymbol{B}$ ∥ [010] and strain $\perp$ [010]<br>• $\boldsymbol{E}$ ∥ [001] and $\boldsymbol{B}$ ∥ [010] and strain $\perp$ [010]<br>• $\boldsymbol{E} \perp$ [010] and $\boldsymbol{B}$ ∥ [010]
$Pm1'$ (6.19) $\mathbb{Z}_1$	• $\boldsymbol{E}$ ∥ [100] and strain $\perp$ [010]<br>• $\boldsymbol{E}$ ∥ [001] and strain $\perp$ [010]<br>• $\boldsymbol{E} \perp$ [010]
Pc' (7.26) $\mathbb{Z}_1$	• $\boldsymbol{E}$ ∥ [100] and $\boldsymbol{B}$ ∥ [100] and strain $\perp$ [001]<br>• $\boldsymbol{E}$ ∥ [100] and $\boldsymbol{B}$ ∥ [010] and strain $\perp$ [001]<br>• $\boldsymbol{E}$ ∥ [100] and $\boldsymbol{B} \perp$ [001]<br>• $\boldsymbol{E}$ ∥ [010] and $\boldsymbol{B}$ ∥ [100] and strain $\perp$ [001]<br>• $\boldsymbol{E}$ ∥ [010] and $\boldsymbol{B}$ ∥ [010] and strain $\perp$ [001]<br>• $\boldsymbol{E}$ ∥ [010] and $\boldsymbol{B} \perp$ [001]<br>• $\boldsymbol{E} \perp$ [001] and $\boldsymbol{B}$ ∥ [100]<br>• $\boldsymbol{E} \perp$ [001] and $\boldsymbol{B}$ ∥ [010]<br>• $\boldsymbol{E} \perp$ [001] and $\boldsymbol{B} \perp$ [001]

Continued on next page

...continued

Subgroup & SI group	Perturbation
Pc (7.24) $\mathbb{Z}_1$	• $\boldsymbol{E} \parallel [100]$ and $\boldsymbol{B} \parallel [001]$ and strain $\perp [001]$<br>• $\boldsymbol{E} \parallel [010]$ and $\boldsymbol{B} \parallel [001]$ and strain $\perp [001]$<br>• $\boldsymbol{E} \perp [001]$ and $\boldsymbol{B} \parallel [001]$
$Pc1'$ (7.25) $\mathbb{Z}_1$	• $\boldsymbol{E} \parallel [100]$ and strain $\perp [001]$<br>• $\boldsymbol{E} \parallel [010]$ and strain $\perp [001]$<br>• $\boldsymbol{E} \perp [001]$
$P2_1'$ (4.9) $\mathbb{Z}_1$	• $\boldsymbol{E} \parallel [100]$ and $\boldsymbol{B} \parallel [010]$ and strain $\perp [100]$<br>• $\boldsymbol{E} \parallel [100]$ and $\boldsymbol{B} \parallel [001]$ and strain $\perp [100]$<br>• $\boldsymbol{E} \parallel [100]$ and $\boldsymbol{B} \perp [100]$
$Pmn'2_1'$ (31.126) $\mathbb{Z}_1$	• $\boldsymbol{E} \parallel [100]$ and $\boldsymbol{B} \parallel [010]$
$Pm'n2_1'$ (31.125) $\mathbb{Z}_1$	• $\boldsymbol{E} \parallel [100]$ and $\boldsymbol{B} \parallel [001]$
$P2_1$ (4.7) $\mathbb{Z}_1$	• $\boldsymbol{E} \parallel [100]$ and $\boldsymbol{B} \parallel [100]$ and strain $\perp [100]$
$Pm'n'2_1$ (31.127) $\mathbb{Z}_1$	• $\boldsymbol{E} \parallel [100]$ and $\boldsymbol{B} \parallel [100]$
$P2_11'$ (4.8) $\mathbb{Z}_1$	• $\boldsymbol{E} \parallel [100]$ and strain $\perp [100]$
$Pmn2_11'$ (31.124) $\mathbb{Z}_1$	• $\boldsymbol{E} \parallel [100]$
$P2_1'$ (4.9) $\mathbb{Z}_1$	• $\boldsymbol{E} \parallel [010]$ and $\boldsymbol{B} \parallel [100]$ and strain $\perp [010]$<br>• $\boldsymbol{E} \parallel [010]$ and $\boldsymbol{B} \parallel [001]$ and strain $\perp [010]$<br>• $\boldsymbol{E} \parallel [010]$ and $\boldsymbol{B} \perp [010]$
$P2_1$ (4.7) $\mathbb{Z}_1$	• $\boldsymbol{E} \parallel [010]$ and $\boldsymbol{B} \parallel [010]$ and strain $\perp [010]$
$P2_11'$ (4.8) $\mathbb{Z}_1$	• $\boldsymbol{E} \parallel [010]$ and strain $\perp [010]$
$P\bar{1}$ (2.4) $\mathbb{Z}_2 \times \mathbb{Z}_2 \times \mathbb{Z}_2 \times \mathbb{Z}_4$	• $\boldsymbol{B} \parallel [100]$ and strain in generic direction<br>• $\boldsymbol{B} \parallel [010]$ and strain in generic direction<br>• $\boldsymbol{B} \parallel [001]$ and strain in generic direction<br>• $\boldsymbol{B} \perp [100]$ and strain $\perp [010]$<br>• $\boldsymbol{B} \perp [100]$ and strain $\perp [001]$<br>• $\boldsymbol{B} \perp [100]$ and strain in generic direction<br>• $\boldsymbol{B} \perp [010]$ and strain $\perp [100]$<br>• $\boldsymbol{B} \perp [010]$ and strain $\perp [001]$<br>• $\boldsymbol{B} \perp [010]$ and strain in generic direction<br>• $\boldsymbol{B} \perp [001]$ and strain $\perp [100]$<br>• $\boldsymbol{B} \perp [001]$ and strain $\perp [010]$<br>• $\boldsymbol{B} \perp [001]$ and strain in generic direction<br>• $\boldsymbol{B}$ in generic direction

Continued on next page

...continued

Subgroup & SI group	Perturbation
$P2_1'/m'$ (11.54) $\mathbb{Z}_2 \times \mathbb{Z}_2 \times \mathbb{Z}_4$	• $\boldsymbol{B} \parallel [100]$ and strain $\perp [010]$<br>• $\boldsymbol{B} \parallel [001]$ and strain $\perp [010]$<br>• $\boldsymbol{B} \perp [010]$
$P2_1/m$ (11.50) $\mathbb{Z}_2$	• $\boldsymbol{B} \parallel [010]$ and strain $\perp [010]$
$P\bar{1}1'$ (2.5) $\mathbb{Z}_2 \times \mathbb{Z}_2 \times \mathbb{Z}_2 \times \mathbb{Z}_4$	• strain in generic direction
$P2_1/m1'$ (11.51) $\mathbb{Z}_2$	• strain $\perp [010]$
Pm' (6.20) $\mathbb{Z}_1$	• $\boldsymbol{E} \parallel [010]$ and $\boldsymbol{B} \parallel [010]$ and strain $\perp [100]$<br>• $\boldsymbol{E} \parallel [010]$ and $\boldsymbol{B} \parallel [001]$ and strain $\perp [100]$<br>• $\boldsymbol{E} \parallel [010]$ and $\boldsymbol{B} \perp [100]$<br>• $\boldsymbol{E} \parallel [001]$ and $\boldsymbol{B} \parallel [010]$ and strain $\perp [100]$<br>• $\boldsymbol{E} \parallel [001]$ and $\boldsymbol{B} \parallel [001]$ and strain $\perp [100]$<br>• $\boldsymbol{E} \parallel [001]$ and $\boldsymbol{B} \perp [100]$<br>• $\boldsymbol{E} \perp [100]$ and $\boldsymbol{B} \parallel [010]$<br>• $\boldsymbol{E} \perp [100]$ and $\boldsymbol{B} \parallel [001]$<br>• $\boldsymbol{E} \perp [100]$ and $\boldsymbol{B} \perp [100]$
$Pm'n2_1'$ (31.125) $\mathbb{Z}_1$	• $\boldsymbol{E} \parallel [010]$ and $\boldsymbol{B} \parallel [001]$
$Pm'n'2_1$ (31.127) $\mathbb{Z}_1$	• $\boldsymbol{E} \parallel [010]$ and $\boldsymbol{B} \parallel [010]$
$P2_1'/m'$ (11.54) $\mathbb{Z}_2 \times \mathbb{Z}_2 \times \mathbb{Z}_4$	• $\boldsymbol{B} \parallel [010]$ and strain $\perp [100]$<br>• $\boldsymbol{B} \parallel [001]$ and strain $\perp [100]$<br>• $\boldsymbol{B} \perp [100]$
Pm (6.18) $\mathbb{Z}_1$	• $\boldsymbol{E} \parallel [010]$ and $\boldsymbol{B} \parallel [100]$ and strain $\perp [100]$<br>• $\boldsymbol{E} \parallel [001]$ and $\boldsymbol{B} \parallel [100]$ and strain $\perp [100]$<br>• $\boldsymbol{E} \perp [100]$ and $\boldsymbol{B} \parallel [100]$
$Pmn'2_1'$ (31.126) $\mathbb{Z}_1$	• $\boldsymbol{E} \parallel [010]$ and $\boldsymbol{B} \parallel [100]$
$P2_1/m$ (11.50) $\mathbb{Z}_2$	• $\boldsymbol{B} \parallel [100]$ and strain $\perp [100]$
$Pm1'$ (6.19) $\mathbb{Z}_1$	• $\boldsymbol{E} \parallel [010]$ and strain $\perp [100]$<br>• $\boldsymbol{E} \parallel [001]$ and strain $\perp [100]$<br>• $\boldsymbol{E} \perp [100]$
$Pmn2_11'$ (31.124) $\mathbb{Z}_1$	• $\boldsymbol{E} \parallel [010]$
$P2_1/m1'$ (11.51) $\mathbb{Z}_2$	• strain $\perp [100]$
$P2'$ (3.3) $\mathbb{Z}_1$	• $\boldsymbol{E} \parallel [001]$ and $\boldsymbol{B} \parallel [100]$ and strain $\perp [001]$<br>• $\boldsymbol{E} \parallel [001]$ and $\boldsymbol{B} \parallel [010]$ and strain $\perp [001]$<br>• $\boldsymbol{E} \parallel [001]$ and $\boldsymbol{B} \perp [001]$

Continued on next page

. . . continued

Subgroup & SI group	Perturbation
$P2'/c'$ (13.69) $\mathbb{Z}_2 \times \mathbb{Z}_2 \times \mathbb{Z}_4$	• $\boldsymbol{B}$ ∥ [100] and strain ⊥ [001]<br>• $\boldsymbol{B}$ ∥ [010] and strain ⊥ [001]<br>• $\boldsymbol{B}$ ⊥ [001]
$Pm'm2'$ (25.59) $\mathbb{Z}_1$	• $\boldsymbol{E}$ ∥ [001] and $\boldsymbol{B}$ ∥ [010]
$Pmm'n'$ (59.410) $\mathbb{Z}_2$	• $\boldsymbol{B}$ ∥ [010]
$Pm'm2'$ (25.59) $\mathbb{Z}_1$	• $\boldsymbol{E}$ ∥ [001] and $\boldsymbol{B}$ ∥ [100]
$Pmm'n'$ (59.410) $\mathbb{Z}_2$	• $\boldsymbol{B}$ ∥ [100]
$P2$ (3.1) $\mathbb{Z}_2$	• $\boldsymbol{E}$ ∥ [001] and $\boldsymbol{B}$ ∥ [001] and strain ⊥ [001]
$P2/c$ (13.65) $\mathbb{Z}_2 \times \mathbb{Z}_2$	• $\boldsymbol{B}$ ∥ [001] and strain ⊥ [001]
$Pm'm'2$ (25.60) $\mathbb{Z}_2$	• $\boldsymbol{E}$ ∥ [001] and $\boldsymbol{B}$ ∥ [001]
$Pm'm'n$ (59.409) $\mathbb{Z}_2 \times \mathbb{Z}_2$	• $\boldsymbol{B}$ ∥ [001]
$P21'$ (3.2) $\mathbb{Z}_2$	• $\boldsymbol{E}$ ∥ [001] and strain ⊥ [001]
$P2/c1'$ (13.66) $\mathbb{Z}_2 \times \mathbb{Z}_2$	• strain ⊥ [001]
$Pmm21'$ (25.58) $\mathbb{Z}_1$	• $\boldsymbol{E}$ ∥ [001]

Table. S 517: Subgroups of $Pm'mn$ (59.407) that can be realized by applying $\boldsymbol{E}/\boldsymbol{B}$ fields and/or uniaxial strains.

Subgroup & SI group	Perturbation
Pm (6.18) $\mathbb{Z}_1$	• $\boldsymbol{B}$ ∥ [010] and strain ⊥ [010]<br>• $\boldsymbol{E}$ ∥ [100] and strain ⊥ [010]<br>• $\boldsymbol{E}$ ∥ [100] and $\boldsymbol{B}$ ∥ [010]<br>• $\boldsymbol{E}$ ∥ [001] and strain ⊥ [010]<br>• $\boldsymbol{E}$ ⊥ [010]
Pc (7.24) $\mathbb{Z}_1$	• $\boldsymbol{B}$ ∥ [001] and strain ⊥ [001]<br>• $\boldsymbol{E}$ ∥ [100] and strain ⊥ [001]<br>• $\boldsymbol{E}$ ∥ [100] and $\boldsymbol{B}$ ∥ [001]<br>• $\boldsymbol{E}$ ∥ [010] and strain ⊥ [001]<br>• $\boldsymbol{E}$ ⊥ [001]
$P2_1$ (4.7) $\mathbb{Z}_1$	• $\boldsymbol{B}$ ∥ [100] and strain ⊥ [100]<br>• $\boldsymbol{E}$ ∥ [100] and strain ⊥ [100]<br>• $\boldsymbol{E}$ ∥ [100] and $\boldsymbol{B}$ ∥ [100]

Continued on next page

...continued

Subgroup & SI group	Perturbation
$Pmn2_1$ (31.123) $\mathbb{Z}_1$	• $\boldsymbol{E} \parallel [100]$
$P2_1'$ (4.9) $\mathbb{Z}_1$	• $\boldsymbol{B} \parallel [100]$ and strain $\perp [010]$<br>• $\boldsymbol{B} \parallel [001]$ and strain $\perp [010]$<br>• $\boldsymbol{B} \perp [010]$<br>• $\boldsymbol{E} \parallel [010]$ and strain $\perp [010]$<br>• $\boldsymbol{E} \parallel [010]$ and $\boldsymbol{B} \parallel [100]$
$P\bar{1}'$ (2.6) $\mathbb{Z}_1$	• strain in generic direction
$P2_1'/m$ (11.52) $\mathbb{Z}_1$	• strain $\perp [010]$
Pm' (6.20) $\mathbb{Z}_1$	• $\boldsymbol{B} \parallel [010]$ and strain $\perp [100]$<br>• $\boldsymbol{B} \parallel [001]$ and strain $\perp [100]$<br>• $\boldsymbol{B} \perp [100]$<br>• $\boldsymbol{E} \parallel [010]$ and strain $\perp [100]$<br>• $\boldsymbol{E} \parallel [010]$ and $\boldsymbol{B} \parallel [010]$<br>• $\boldsymbol{E} \parallel [001]$ and strain $\perp [100]$<br>• $\boldsymbol{E} \parallel [001]$ and $\boldsymbol{B} \parallel [001]$<br>• $\boldsymbol{E} \perp [100]$
$Pm'n2_1'$ (31.125) $\mathbb{Z}_1$	• $\boldsymbol{B} \parallel [001]$<br>• $\boldsymbol{E} \parallel [010]$
$P2_1/m'$ (11.53) $\mathbb{Z}_1$	• strain $\perp [100]$
$P2'$ (3.3) $\mathbb{Z}_1$	• $\boldsymbol{B} \parallel [100]$ and strain $\perp [001]$<br>• $\boldsymbol{B} \parallel [010]$ and strain $\perp [001]$<br>• $\boldsymbol{B} \perp [001]$<br>• $\boldsymbol{E} \parallel [001]$ and strain $\perp [001]$<br>• $\boldsymbol{E} \parallel [001]$ and $\boldsymbol{B} \parallel [100]$
$P2_12_1'2'$ (18.19) $\mathbb{Z}_1$	• $\boldsymbol{B} \parallel [100]$
$P2'/c$ (13.67) $\mathbb{Z}_1$	• strain $\perp [001]$
$Pm'm2'$ (25.59) $\mathbb{Z}_1$	• $\boldsymbol{B} \parallel [010]$<br>• $\boldsymbol{E} \parallel [001]$

Table. S 518: Subgroups of $Pmmn'$ (59.408) that can be realized by applying $\boldsymbol{E}/\boldsymbol{B}$ fields and/or uniaxial strains.

Subgroup & SI group	Perturbation

Continued on next page

…continued

Subgroup & SI group	Perturbation
Pm (6.18) $\mathbb{Z}_1$	• $\boldsymbol{B} \parallel [010]$ and strain $\perp [010]$<br>• $\boldsymbol{E} \parallel [100]$ and strain $\perp [010]$<br>• $\boldsymbol{E} \parallel [001]$ and strain $\perp [010]$<br>• $\boldsymbol{E} \parallel [001]$ and $\boldsymbol{B} \parallel [010]$<br>• $\boldsymbol{E} \perp [010]$
Pc' (7.26) $\mathbb{Z}_1$	• $\boldsymbol{B} \parallel [100]$ and strain $\perp [001]$<br>• $\boldsymbol{B} \parallel [010]$ and strain $\perp [001]$<br>• $\boldsymbol{B} \perp [001]$<br>• $\boldsymbol{E} \parallel [100]$ and strain $\perp [001]$<br>• $\boldsymbol{E} \parallel [100]$ and $\boldsymbol{B} \parallel [100]$<br>• $\boldsymbol{E} \parallel [010]$ and strain $\perp [001]$<br>• $\boldsymbol{E} \parallel [010]$ and $\boldsymbol{B} \parallel [010]$<br>• $\boldsymbol{E} \perp [001]$
$P2_1'$ (4.9) $\mathbb{Z}_1$	• $\boldsymbol{B} \parallel [010]$ and strain $\perp [100]$<br>• $\boldsymbol{B} \parallel [001]$ and strain $\perp [100]$<br>• $\boldsymbol{B} \perp [100]$<br>• $\boldsymbol{E} \parallel [100]$ and strain $\perp [100]$<br>• $\boldsymbol{E} \parallel [100]$ and $\boldsymbol{B} \parallel [001]$
$Pmn'2_1'$ (31.126) $\mathbb{Z}_1$	• $\boldsymbol{B} \parallel [010]$<br>• $\boldsymbol{E} \parallel [100]$
$P2_1'$ (4.9) $\mathbb{Z}_1$	• $\boldsymbol{B} \parallel [100]$ and strain $\perp [010]$<br>• $\boldsymbol{B} \parallel [001]$ and strain $\perp [010]$<br>• $\boldsymbol{B} \perp [010]$<br>• $\boldsymbol{E} \parallel [010]$ and strain $\perp [010]$<br>• $\boldsymbol{E} \parallel [010]$ and $\boldsymbol{B} \parallel [001]$
$P\bar{1}'$ (2.6) $\mathbb{Z}_1$	• strain in generic direction
$P2_1'/m$ (11.52) $\mathbb{Z}_1$	• strain $\perp [010]$
Pm (6.18) $\mathbb{Z}_1$	• $\boldsymbol{B} \parallel [100]$ and strain $\perp [100]$<br>• $\boldsymbol{E} \parallel [010]$ and strain $\perp [100]$<br>• $\boldsymbol{E} \parallel [001]$ and strain $\perp [100]$<br>• $\boldsymbol{E} \parallel [001]$ and $\boldsymbol{B} \parallel [100]$<br>• $\boldsymbol{E} \perp [100]$
$Pmn'2_1'$ (31.126) $\mathbb{Z}_1$	• $\boldsymbol{B} \parallel [100]$<br>• $\boldsymbol{E} \parallel [010]$
$P2_1'/m$ (11.52) $\mathbb{Z}_1$	• strain $\perp [100]$

Continued on next page

...continued

Subgroup & SI group	Perturbation
$P2$ (3.1) $\mathbb{Z}_2$	• $\boldsymbol{B}$ || [001] and strain ⊥ [001]<br>• $\boldsymbol{E}$ || [001] and strain ⊥ [001]<br>• $\boldsymbol{E}$ || [001] and $\boldsymbol{B}$ || [001]
$P2_1'2_1'2$ (18.18) $\mathbb{Z}_2$	• $\boldsymbol{B}$ || [001]
$P2/c'$ (13.68) $\mathbb{Z}_2$	• strain ⊥ [001]
$Pmm2$ (25.57) $\mathbb{Z}_1$	• $\boldsymbol{E}$ || [001]

Table. S 519: Subgroups of $Pm'm'n$ (59.409) that can be realized by applying $\boldsymbol{E}/\boldsymbol{B}$ fields and/or uniaxial strains.

Subgroup & SI group	Perturbation
Pm' (6.20) $\mathbb{Z}_1$	• $\boldsymbol{E}$ || [100] and strain ⊥ [010]<br>• $\boldsymbol{E}$ || [100] and ($\boldsymbol{B}$ || [100] or $\boldsymbol{B}$ ⊥ [010])<br>• $\boldsymbol{E}$ || [001] and strain ⊥ [010]<br>• $\boldsymbol{E}$ || [001] and ($\boldsymbol{B}$ || [100] or $\boldsymbol{B}$ ⊥ [010])<br>• $\boldsymbol{E}$ ⊥ [010]
Pc (7.24) $\mathbb{Z}_1$	• $\boldsymbol{E}$ || [100] and strain ⊥ [001]<br>• $\boldsymbol{E}$ || [010] and strain ⊥ [001]<br>• $\boldsymbol{E}$ ⊥ [001]
$P2_1'$ (4.9) $\mathbb{Z}_1$	• $\boldsymbol{E}$ || [100] and strain ⊥ [100]<br>• $\boldsymbol{E}$ || [100] and ($\boldsymbol{B}$ || [010] or $\boldsymbol{B}$ ⊥ [100])
$Pm'n2_1'$ (31.125) $\mathbb{Z}_1$	• $\boldsymbol{E}$ || [100]
$P2_1'$ (4.9) $\mathbb{Z}_1$	• $\boldsymbol{E}$ || [010] and strain ⊥ [010]<br>• $\boldsymbol{E}$ || [010] and ($\boldsymbol{B}$ || [100] or $\boldsymbol{B}$ ⊥ [010])
$P\bar{1}$ (2.4) $\mathbb{Z}_2 \times \mathbb{Z}_2 \times \mathbb{Z}_2 \times \mathbb{Z}_4$	• strain in generic direction<br>• ($\boldsymbol{B}$ || [100] or $\boldsymbol{B}$ ⊥ [010]) and strain ⊥ [100]<br>• ($\boldsymbol{B}$ || [100] or $\boldsymbol{B}$ ⊥ [010]) and strain ⊥ [001]<br>• ($\boldsymbol{B}$ || [010] or $\boldsymbol{B}$ ⊥ [100]) and strain ⊥ [010]<br>• ($\boldsymbol{B}$ || [010] or $\boldsymbol{B}$ ⊥ [100]) and strain ⊥ [001]<br>• $\boldsymbol{B}$ in generic direction
$P2_1'/m'$ (11.54) $\mathbb{Z}_2 \times \mathbb{Z}_2 \times \mathbb{Z}_4$	• strain ⊥ [010]<br>• ($\boldsymbol{B}$ || [100] or $\boldsymbol{B}$ ⊥ [010])

Continued on next page

...continued

Subgroup & SI group	Perturbation
Pm' (6.20) $\mathbb{Z}_1$	• $\boldsymbol{E}$ ∥ [010] and strain ⊥ [100]<br>• $\boldsymbol{E}$ ∥ [010] and ($\boldsymbol{B}$ ∥ [010] or $\boldsymbol{B}$ ⊥ [100])<br>• $\boldsymbol{E}$ ∥ [001] and strain ⊥ [100]<br>• $\boldsymbol{E}$ ∥ [001] and ($\boldsymbol{B}$ ∥ [010] or $\boldsymbol{B}$ ⊥ [100])<br>• $\boldsymbol{E}$ ⊥ [100]
$Pm'n2_1'$ (31.125) $\mathbb{Z}_1$	• $\boldsymbol{E}$ ∥ [010]
$P2_1'/m'$ (11.54) $\mathbb{Z}_2 \times \mathbb{Z}_2 \times \mathbb{Z}_4$	• strain ⊥ [100]<br>• ($\boldsymbol{B}$ ∥ [010] or $\boldsymbol{B}$ ⊥ [100])
$P2$ (3.1) $\mathbb{Z}_2$	• $\boldsymbol{E}$ ∥ [001] and strain ⊥ [001]
$P2/c$ (13.65) $\mathbb{Z}_2 \times \mathbb{Z}_2$	• strain ⊥ [001]
$Pm'm'2$ (25.60) $\mathbb{Z}_2$	• $\boldsymbol{E}$ ∥ [001]

Table. S 520: Subgroups of $Pmm'n'$ (59.410) that can be realized by applying $\boldsymbol{E}/\boldsymbol{B}$ fields and/or uniaxial strains.

Subgroup & SI group	Perturbation
Pm' (6.20) $\mathbb{Z}_1$	• $\boldsymbol{E}$ ∥ [100] and strain ⊥ [010]<br>• $\boldsymbol{E}$ ∥ [100] and ($\boldsymbol{B}$ ∥ [001] or $\boldsymbol{B}$ ⊥ [010])<br>• $\boldsymbol{E}$ ∥ [001] and strain ⊥ [010]<br>• $\boldsymbol{E}$ ∥ [001] and ($\boldsymbol{B}$ ∥ [001] or $\boldsymbol{B}$ ⊥ [010])<br>• $\boldsymbol{E}$ ⊥ [010]
Pc' (7.26) $\mathbb{Z}_1$	• $\boldsymbol{E}$ ∥ [100] and strain ⊥ [001]<br>• $\boldsymbol{E}$ ∥ [100] and ($\boldsymbol{B}$ ∥ [010] or $\boldsymbol{B}$ ⊥ [001])<br>• $\boldsymbol{E}$ ∥ [010] and strain ⊥ [001]<br>• $\boldsymbol{E}$ ∥ [010] and ($\boldsymbol{B}$ ∥ [010] or $\boldsymbol{B}$ ⊥ [001])<br>• $\boldsymbol{E}$ ⊥ [001]
$P2_1$ (4.7) $\mathbb{Z}_1$	• $\boldsymbol{E}$ ∥ [100] and strain ⊥ [100]
$Pm'n'2_1$ (31.127) $\mathbb{Z}_1$	• $\boldsymbol{E}$ ∥ [100]
$P2_1'$ (4.9) $\mathbb{Z}_1$	• $\boldsymbol{E}$ ∥ [010] and strain ⊥ [010]<br>• $\boldsymbol{E}$ ∥ [010] and ($\boldsymbol{B}$ ∥ [001] or $\boldsymbol{B}$ ⊥ [010])

Continued on next page

...continued

Subgroup & SI group	Perturbation
$P\bar{1}$ (2.4) $\mathbb{Z}_2\times\mathbb{Z}_2\times\mathbb{Z}_2\times\mathbb{Z}_4$	• strain in generic direction<br>• ($\boldsymbol{B}$ ∥ [010] or $\boldsymbol{B}\perp$ [001]) and strain ⊥ [100]<br>• ($\boldsymbol{B}$ ∥ [010] or $\boldsymbol{B}\perp$ [001]) and strain ⊥ [010]<br>• ($\boldsymbol{B}$ ∥ [001] or $\boldsymbol{B}\perp$ [010]) and strain ⊥ [100]<br>• ($\boldsymbol{B}$ ∥ [001] or $\boldsymbol{B}\perp$ [010]) and strain ⊥ [001]<br>• $\boldsymbol{B}$ in generic direction
$P2_1'/m'$ (11.54) $\mathbb{Z}_2\times\mathbb{Z}_2\times\mathbb{Z}_4$	• strain ⊥ [010]<br>• ($\boldsymbol{B}$ ∥ [001] or $\boldsymbol{B}\perp$ [010])
Pm (6.18) $\mathbb{Z}_1$	• $\boldsymbol{E}$ ∥ [010] and strain ⊥ [100]<br>• $\boldsymbol{E}$ ∥ [001] and strain ⊥ [100]<br>• $\boldsymbol{E}\perp$ [100]
$Pmn'2_1'$ (31.126) $\mathbb{Z}_1$	• $\boldsymbol{E}$ ∥ [010]
$P2_1/m$ (11.50) $\mathbb{Z}_2$	• strain ⊥ [100]
$P2'$ (3.3) $\mathbb{Z}_1$	• $\boldsymbol{E}$ ∥ [001] and strain ⊥ [001]<br>• $\boldsymbol{E}$ ∥ [001] and ($\boldsymbol{B}$ ∥ [010] or $\boldsymbol{B}\perp$ [001])
$P2'/c'$ (13.69) $\mathbb{Z}_2\times\mathbb{Z}_2\times\mathbb{Z}_4$	• strain ⊥ [001]<br>• ($\boldsymbol{B}$ ∥ [010] or $\boldsymbol{B}\perp$ [001])
$Pm'm2'$ (25.59) $\mathbb{Z}_1$	• $\boldsymbol{E}$ ∥ [001]

Table. S 521: Subgroups of $Pm'm'n'$ (59.411) that can be realized by applying $\boldsymbol{E}/\boldsymbol{B}$ fields and/or uniaxial strains.

Subgroup & SI group	Perturbation
Pm' (6.20) $\mathbb{Z}_1$	• $\boldsymbol{B}$ ∥ [100] and strain ⊥ [010]<br>• $\boldsymbol{B}$ ∥ [001] and strain ⊥ [010]<br>• $\boldsymbol{B}\perp$ [010]<br>• $\boldsymbol{E}$ ∥ [100] and strain ⊥ [010]<br>• $\boldsymbol{E}$ ∥ [100] and $\boldsymbol{B}$ ∥ [001]<br>• $\boldsymbol{E}$ ∥ [001] and strain ⊥ [010]<br>• $\boldsymbol{E}$ ∥ [001] and $\boldsymbol{B}$ ∥ [100]<br>• $\boldsymbol{E}\perp$ [010]

Continued on next page

…continued

Subgroup & SI group	Perturbation
Pc' (7.26) $\mathbb{Z}_1$	• $\boldsymbol{B} \parallel$ [100] and strain $\perp$ [001]<br>• $\boldsymbol{B} \parallel$ [010] and strain $\perp$ [001]<br>• $\boldsymbol{B} \perp$ [001]<br>• $\boldsymbol{E} \parallel$ [100] and strain $\perp$ [001]<br>• $\boldsymbol{E} \parallel$ [100] and $\boldsymbol{B} \parallel$ [010]<br>• $\boldsymbol{E} \parallel$ [010] and strain $\perp$ [001]<br>• $\boldsymbol{E} \parallel$ [010] and $\boldsymbol{B} \parallel$ [100]<br>• $\boldsymbol{E} \perp$ [001]
$P2_1$ (4.7) $\mathbb{Z}_1$	• $\boldsymbol{B} \parallel$ [100] and strain $\perp$ [100]<br>• $\boldsymbol{E} \parallel$ [100] and strain $\perp$ [100]
$Pm'n'2_1$ (31.127) $\mathbb{Z}_1$	• $\boldsymbol{B} \parallel$ [100]<br>• $\boldsymbol{E} \parallel$ [100]
$P2_1$ (4.7) $\mathbb{Z}_1$	• $\boldsymbol{B} \parallel$ [010] and strain $\perp$ [010]<br>• $\boldsymbol{E} \parallel$ [010] and strain $\perp$ [010]
$P\bar{1}'$ (2.6) $\mathbb{Z}_1$	• strain in generic direction
$P2_1/m'$ (11.53) $\mathbb{Z}_1$	• strain $\perp$ [010]
Pm' (6.20) $\mathbb{Z}_1$	• $\boldsymbol{B} \parallel$ [010] and strain $\perp$ [100]<br>• $\boldsymbol{B} \parallel$ [001] and strain $\perp$ [100]<br>• $\boldsymbol{B} \perp$ [100]<br>• $\boldsymbol{E} \parallel$ [010] and strain $\perp$ [100]<br>• $\boldsymbol{E} \parallel$ [010] and $\boldsymbol{B} \parallel$ [001]<br>• $\boldsymbol{E} \parallel$ [001] and strain $\perp$ [100]<br>• $\boldsymbol{E} \parallel$ [001] and $\boldsymbol{B} \parallel$ [010]<br>• $\boldsymbol{E} \perp$ [100]
$Pm'n'2_1$ (31.127) $\mathbb{Z}_1$	• $\boldsymbol{B} \parallel$ [010]<br>• $\boldsymbol{E} \parallel$ [010]
$P2_1/m'$ (11.53) $\mathbb{Z}_1$	• strain $\perp$ [100]
$P2$ (3.1) $\mathbb{Z}_2$	• $\boldsymbol{B} \parallel$ [001] and strain $\perp$ [001]<br>• $\boldsymbol{E} \parallel$ [001] and strain $\perp$ [001]
$P2/c'$ (13.68) $\mathbb{Z}_2$	• strain $\perp$ [001]
$Pm'm'2$ (25.60) $\mathbb{Z}_2$	• $\boldsymbol{B} \parallel$ [001]<br>• $\boldsymbol{E} \parallel$ [001]

Table. S 522: Subgroups of P_bmmn (59.412) that can be realized by applying $\boldsymbol{E}/\boldsymbol{B}$ fields and/or uniaxial strains.

Subgroup & SI group	Perturbation
P_S1 (1.3) $\mathbb{Z}_1$	• $\boldsymbol{E} \parallel [100]$ and strain in generic direction<br>• $\boldsymbol{E} \parallel [010]$ and strain in generic direction<br>• $\boldsymbol{E} \parallel [001]$ and strain in generic direction<br>• $\boldsymbol{E} \perp [100]$ and strain $\perp [010]$<br>• $\boldsymbol{E} \perp [100]$ and strain $\perp [001]$<br>• $\boldsymbol{E} \perp [100]$ and strain in generic direction<br>• $\boldsymbol{E} \perp [010]$ and strain $\perp [100]$<br>• $\boldsymbol{E} \perp [010]$ and strain $\perp [001]$<br>• $\boldsymbol{E} \perp [010]$ and strain in generic direction<br>• $\boldsymbol{E} \perp [001]$ and strain $\perp [100]$<br>• $\boldsymbol{E} \perp [001]$ and strain $\perp [010]$<br>• $\boldsymbol{E} \perp [001]$ and strain in generic direction<br>• $\boldsymbol{E}$ in generic direction
Pm' (6.20) $\mathbb{Z}_1$	• $\boldsymbol{E} \parallel [100]$ and $\boldsymbol{B} \parallel [100]$ and strain $\perp [010]$<br>• $\boldsymbol{E} \parallel [100]$ and $\boldsymbol{B} \parallel [001]$ and strain $\perp [010]$<br>• $\boldsymbol{E} \parallel [100]$ and $\boldsymbol{B} \perp [010]$<br>• $\boldsymbol{E} \parallel [001]$ and $\boldsymbol{B} \parallel [100]$ and strain $\perp [010]$<br>• $\boldsymbol{E} \parallel [001]$ and $\boldsymbol{B} \parallel [001]$ and strain $\perp [010]$<br>• $\boldsymbol{E} \parallel [001]$ and $\boldsymbol{B} \perp [010]$<br>• $\boldsymbol{E} \perp [010]$ and $\boldsymbol{B} \parallel [100]$<br>• $\boldsymbol{E} \perp [010]$ and $\boldsymbol{B} \parallel [001]$<br>• $\boldsymbol{E} \perp [010]$ and $\boldsymbol{B} \perp [010]$
Pm (6.18) $\mathbb{Z}_1$	• $\boldsymbol{E} \parallel [100]$ and $\boldsymbol{B} \parallel [010]$ and strain $\perp [010]$<br>• $\boldsymbol{E} \parallel [001]$ and $\boldsymbol{B} \parallel [010]$ and strain $\perp [010]$<br>• $\boldsymbol{E} \perp [010]$ and $\boldsymbol{B} \parallel [010]$
P_bm (6.22) $\mathbb{Z}_1$	• $\boldsymbol{E} \parallel [100]$ and strain $\perp [010]$<br>• $\boldsymbol{E} \parallel [001]$ and strain $\perp [010]$<br>• $\boldsymbol{E} \perp [010]$
Pc' (7.26) $\mathbb{Z}_1$	• $\boldsymbol{E} \parallel [100]$ and $\boldsymbol{B} \parallel [100]$ and strain $\perp [001]$<br>• $\boldsymbol{E} \parallel [100]$ and $\boldsymbol{B} \parallel [010]$ and strain $\perp [001]$<br>• $\boldsymbol{E} \parallel [100]$ and $\boldsymbol{B} \perp [001]$<br>• $\boldsymbol{E} \parallel [010]$ and $\boldsymbol{B} \parallel [100]$ and strain $\perp [001]$<br>• $\boldsymbol{E} \parallel [010]$ and $\boldsymbol{B} \parallel [010]$ and strain $\perp [001]$<br>• $\boldsymbol{E} \parallel [010]$ and $\boldsymbol{B} \perp [001]$<br>• $\boldsymbol{E} \perp [001]$ and $\boldsymbol{B} \parallel [100]$<br>• $\boldsymbol{E} \perp [001]$ and $\boldsymbol{B} \parallel [010]$<br>• $\boldsymbol{E} \perp [001]$ and $\boldsymbol{B} \perp [001]$

Continued on next page

…continued

Subgroup & SI group	Perturbation
Pc (7.24) $\mathbb{Z}_1$	• $\boldsymbol{E} \parallel [100]$ and $\boldsymbol{B} \parallel [001]$ and strain $\perp [001]$<br>• $\boldsymbol{E} \parallel [010]$ and $\boldsymbol{B} \parallel [001]$ and strain $\perp [001]$<br>• $\boldsymbol{E} \perp [001]$ and $\boldsymbol{B} \parallel [001]$
P_ac (7.27) $\mathbb{Z}_1$	• $\boldsymbol{E} \parallel [100]$ and strain $\perp [001]$<br>• $\boldsymbol{E} \parallel [010]$ and strain $\perp [001]$<br>• $\boldsymbol{E} \perp [001]$
$P2_1$ (4.7) $\mathbb{Z}_1$	• $\boldsymbol{E} \parallel [100]$ and $\boldsymbol{B} \parallel [100]$ and strain $\perp [100]$
$Pm'c'2_1$ (26.70) $\mathbb{Z}_1$	• $\boldsymbol{E} \parallel [100]$ and $\boldsymbol{B} \parallel [100]$
$P2_1'$ (4.9) $\mathbb{Z}_1$	• $\boldsymbol{E} \parallel [100]$ and $\boldsymbol{B} \parallel [010]$ and strain $\perp [100]$<br>• $\boldsymbol{E} \parallel [100]$ and $\boldsymbol{B} \parallel [001]$ and strain $\perp [100]$<br>• $\boldsymbol{E} \parallel [100]$ and $\boldsymbol{B} \perp [100]$
$Pmc'2_1'$ (26.69) $\mathbb{Z}_1$	• $\boldsymbol{E} \parallel [100]$ and $\boldsymbol{B} \parallel [010]$
$Pm'n2_1'$ (31.125) $\mathbb{Z}_1$	• $\boldsymbol{E} \parallel [100]$ and $\boldsymbol{B} \parallel [001]$
P_a2_1 (4.10) $\mathbb{Z}_1$	• $\boldsymbol{E} \parallel [100]$ and strain $\perp [100]$
P_amn2_1 (31.128) $\mathbb{Z}_1$	• $\boldsymbol{E} \parallel [100]$
$P2'$ (3.3) $\mathbb{Z}_1$	• $\boldsymbol{E} \parallel [010]$ and $\boldsymbol{B} \parallel [100]$ and strain $\perp [010]$<br>• $\boldsymbol{E} \parallel [010]$ and $\boldsymbol{B} \parallel [001]$ and strain $\perp [010]$<br>• $\boldsymbol{E} \parallel [010]$ and $\boldsymbol{B} \perp [010]$
$P2_1$ (4.7) $\mathbb{Z}_1$	• $\boldsymbol{E} \parallel [010]$ and $\boldsymbol{B} \parallel [010]$ and strain $\perp [010]$
P_b2_1 (4.11) $\mathbb{Z}_1$	• $\boldsymbol{E} \parallel [010]$ and strain $\perp [010]$
$P\bar{1}$ (2.4) $\mathbb{Z}_2 \times \mathbb{Z}_2 \times \mathbb{Z}_2 \times \mathbb{Z}_4$	• $\boldsymbol{B} \parallel [100]$ and strain in generic direction<br>• $\boldsymbol{B} \parallel [010]$ and strain in generic direction<br>• $\boldsymbol{B} \parallel [001]$ and strain in generic direction<br>• $\boldsymbol{B} \perp [100]$ and strain $\perp [010]$<br>• $\boldsymbol{B} \perp [100]$ and strain $\perp [001]$<br>• $\boldsymbol{B} \perp [100]$ and strain in generic direction<br>• $\boldsymbol{B} \perp [010]$ and strain $\perp [100]$<br>• $\boldsymbol{B} \perp [010]$ and strain $\perp [001]$<br>• $\boldsymbol{B} \perp [010]$ and strain in generic direction<br>• $\boldsymbol{B} \perp [001]$ and strain $\perp [100]$<br>• $\boldsymbol{B} \perp [001]$ and strain $\perp [010]$<br>• $\boldsymbol{B} \perp [001]$ and strain in generic direction<br>• $\boldsymbol{B}$ in generic direction

Continued on next page

. . . continued

Subgroup & SI group	Perturbation
$P2'/m'$ (10.46) $\mathbb{Z}_2\times\mathbb{Z}_2\times\mathbb{Z}_2\times\mathbb{Z}_4$	• $\boldsymbol{B}$ ∥ [100] and strain ⊥ [010]<br>• $\boldsymbol{B}$ ∥ [001] and strain ⊥ [010]<br>• $\boldsymbol{B}$ ⊥ [010]
$P2_1/m$ (11.50) $\mathbb{Z}_2$	• $\boldsymbol{B}$ ∥ [010] and strain ⊥ [010]
$P_S\bar{1}$ (2.7) $\mathbb{Z}_2\times\mathbb{Z}_2\times\mathbb{Z}_4$	• strain in generic direction
P_b2_1/m (11.56) $\mathbb{Z}_2$	• strain ⊥ [010]
Pm (6.18) $\mathbb{Z}_1$	• $\boldsymbol{E}$ ∥ [010] and $\boldsymbol{B}$ ∥ [100] and strain ⊥ [100]<br>• $\boldsymbol{E}$ ∥ [001] and $\boldsymbol{B}$ ∥ [100] and strain ⊥ [100]<br>• $\boldsymbol{E}$ ⊥ [100] and $\boldsymbol{B}$ ∥ [100]
$Pma'2'$ (28.90) $\mathbb{Z}_1$	• $\boldsymbol{E}$ ∥ [010] and $\boldsymbol{B}$ ∥ [100]
$P2_1/m$ (11.50) $\mathbb{Z}_2$	• $\boldsymbol{B}$ ∥ [100] and strain ⊥ [100]
Pc' (7.26) $\mathbb{Z}_1$	• $\boldsymbol{E}$ ∥ [010] and $\boldsymbol{B}$ ∥ [010] and strain ⊥ [100]<br>• $\boldsymbol{E}$ ∥ [010] and $\boldsymbol{B}$ ∥ [001] and strain ⊥ [100]<br>• $\boldsymbol{E}$ ∥ [010] and $\boldsymbol{B}$ ⊥ [100]<br>• $\boldsymbol{E}$ ∥ [001] and $\boldsymbol{B}$ ∥ [010] and strain ⊥ [100]<br>• $\boldsymbol{E}$ ∥ [001] and $\boldsymbol{B}$ ∥ [001] and strain ⊥ [100]<br>• $\boldsymbol{E}$ ∥ [001] and $\boldsymbol{B}$ ⊥ [100]<br>• $\boldsymbol{E}$ ⊥ [100] and $\boldsymbol{B}$ ∥ [010]<br>• $\boldsymbol{E}$ ⊥ [100] and $\boldsymbol{B}$ ∥ [001]<br>• $\boldsymbol{E}$ ⊥ [100] and $\boldsymbol{B}$ ⊥ [100]
$Pnc'2'$ (30.114) $\mathbb{Z}_1$	• $\boldsymbol{E}$ ∥ [010] and $\boldsymbol{B}$ ∥ [001]
$Pc'a'2_1$ (29.103) $\mathbb{Z}_1$	• $\boldsymbol{E}$ ∥ [010] and $\boldsymbol{B}$ ∥ [010]
$P2_1'/c'$ (14.79) $\mathbb{Z}_2\times\mathbb{Z}_4$	• $\boldsymbol{B}$ ∥ [010] and strain ⊥ [100]<br>• $\boldsymbol{B}$ ∥ [001] and strain ⊥ [100]<br>• $\boldsymbol{B}$ ⊥ [100]
P_am (6.21) $\mathbb{Z}_1$	• $\boldsymbol{E}$ ∥ [010] and strain ⊥ [100]<br>• $\boldsymbol{E}$ ∥ [001] and strain ⊥ [100]<br>• $\boldsymbol{E}$ ⊥ [100]
P_cmn2_1 (31.130) $\mathbb{Z}_1$	• $\boldsymbol{E}$ ∥ [010]
P_a2_1/m (11.55) $\mathbb{Z}_2$	• strain ⊥ [100]
$P2'$ (3.3) $\mathbb{Z}_1$	• $\boldsymbol{E}$ ∥ [001] and $\boldsymbol{B}$ ∥ [100] and strain ⊥ [001]<br>• $\boldsymbol{E}$ ∥ [001] and $\boldsymbol{B}$ ∥ [010] and strain ⊥ [001]<br>• $\boldsymbol{E}$ ∥ [001] and $\boldsymbol{B}$ ⊥ [001]

Continued on next page

...continued

Subgroup & SI group	Perturbation
$P2'/c'$ (13.69) $\mathbb{Z}_2 \times \mathbb{Z}_2 \times \mathbb{Z}_4$	• $\boldsymbol{B} \parallel [100]$ and strain $\perp [001]$<br>• $\boldsymbol{B} \parallel [010]$ and strain $\perp [001]$<br>• $\boldsymbol{B} \perp [001]$
$Pm'm2'$ (25.59) $\mathbb{Z}_1$	• $\boldsymbol{E} \parallel [001]$ and $\boldsymbol{B} \parallel [100]$
$Pmm'a'$ (51.295) $\mathbb{Z}_2$	• $\boldsymbol{B} \parallel [100]$
$Pma'2'$ (28.90) $\mathbb{Z}_1$	• $\boldsymbol{E} \parallel [001]$ and $\boldsymbol{B} \parallel [010]$
$Pb'c'm$ (57.382) $\mathbb{Z}_2$	• $\boldsymbol{B} \parallel [010]$
$P2$ (3.1) $\mathbb{Z}_2$	• $\boldsymbol{E} \parallel [001]$ and $\boldsymbol{B} \parallel [001]$ and strain $\perp [001]$
$P2/c$ (13.65) $\mathbb{Z}_2 \times \mathbb{Z}_2$	• $\boldsymbol{B} \parallel [001]$ and strain $\perp [001]$
$Pm'a'2$ (28.91) $\mathbb{Z}_2$	• $\boldsymbol{E} \parallel [001]$ and $\boldsymbol{B} \parallel [001]$
$Pm'na'$ (53.328) $\mathbb{Z}_2 \times \mathbb{Z}_2$	• $\boldsymbol{B} \parallel [001]$
P_a2 (3.4) $\mathbb{Z}_2$	• $\boldsymbol{E} \parallel [001]$ and strain $\perp [001]$
P_a2/c (13.70) $\mathbb{Z}_2 \times \mathbb{Z}_2$	• strain $\perp [001]$
P_amm2 (25.62) $\mathbb{Z}_1$	• $\boldsymbol{E} \parallel [001]$

Table. S 523: Subgroups of P_cmmn (59.413) that can be realized by applying $\boldsymbol{E}/\boldsymbol{B}$ fields and/or uniaxial strains.

Subgroup & SI group	Perturbation
P_S1 (1.3) $\mathbb{Z}_1$	• $\boldsymbol{E} \parallel [100]$ and strain in generic direction<br>• $\boldsymbol{E} \parallel [010]$ and strain in generic direction<br>• $\boldsymbol{E} \parallel [001]$ and strain in generic direction<br>• $\boldsymbol{E} \perp [100]$ and strain $\perp [010]$<br>• $\boldsymbol{E} \perp [100]$ and strain $\perp [001]$<br>• $\boldsymbol{E} \perp [100]$ and strain in generic direction<br>• $\boldsymbol{E} \perp [010]$ and strain $\perp [100]$<br>• $\boldsymbol{E} \perp [010]$ and strain $\perp [001]$<br>• $\boldsymbol{E} \perp [010]$ and strain in generic direction<br>• $\boldsymbol{E} \perp [001]$ and strain $\perp [100]$<br>• $\boldsymbol{E} \perp [001]$ and strain $\perp [010]$<br>• $\boldsymbol{E} \perp [001]$ and strain in generic direction<br>• $\boldsymbol{E}$ in generic direction

Continued on next page

...continued

Subgroup & SI group	Perturbation
Pm (6.18) $\mathbb{Z}_1$	• $\boldsymbol{E} \parallel [100]$ and $\boldsymbol{B} \parallel [010]$ and strain $\perp [010]$<br>• $\boldsymbol{E} \parallel [001]$ and $\boldsymbol{B} \parallel [010]$ and strain $\perp [010]$<br>• $\boldsymbol{E} \perp [010]$ and $\boldsymbol{B} \parallel [010]$
Pc' (7.26) $\mathbb{Z}_1$	• $\boldsymbol{E} \parallel [100]$ and $\boldsymbol{B} \parallel [100]$ and strain $\perp [010]$<br>• $\boldsymbol{E} \parallel [100]$ and $\boldsymbol{B} \parallel [001]$ and strain $\perp [010]$<br>• $\boldsymbol{E} \parallel [100]$ and $\boldsymbol{B} \perp [010]$<br>• $\boldsymbol{E} \parallel [001]$ and $\boldsymbol{B} \parallel [100]$ and strain $\perp [010]$<br>• $\boldsymbol{E} \parallel [001]$ and $\boldsymbol{B} \parallel [001]$ and strain $\perp [010]$<br>• $\boldsymbol{E} \parallel [001]$ and $\boldsymbol{B} \perp [010]$<br>• $\boldsymbol{E} \perp [010]$ and $\boldsymbol{B} \parallel [100]$<br>• $\boldsymbol{E} \perp [010]$ and $\boldsymbol{B} \parallel [001]$<br>• $\boldsymbol{E} \perp [010]$ and $\boldsymbol{B} \perp [010]$
P_am (6.21) $\mathbb{Z}_1$	• $\boldsymbol{E} \parallel [100]$ and strain $\perp [010]$<br>• $\boldsymbol{E} \parallel [001]$ and strain $\perp [010]$<br>• $\boldsymbol{E} \perp [010]$
Pc (7.24) $\mathbb{Z}_1$	• $\boldsymbol{E} \parallel [100]$ and $\boldsymbol{B} \parallel [001]$ and strain $\perp [001]$<br>• $\boldsymbol{E} \parallel [010]$ and $\boldsymbol{B} \parallel [001]$ and strain $\perp [001]$<br>• $\boldsymbol{E} \perp [001]$ and $\boldsymbol{B} \parallel [001]$
Pc' (7.26) $\mathbb{Z}_1$	• $\boldsymbol{E} \parallel [100]$ and $\boldsymbol{B} \parallel [100]$ and strain $\perp [001]$<br>• $\boldsymbol{E} \parallel [100]$ and $\boldsymbol{B} \parallel [010]$ and strain $\perp [001]$<br>• $\boldsymbol{E} \parallel [100]$ and $\boldsymbol{B} \perp [001]$<br>• $\boldsymbol{E} \parallel [010]$ and $\boldsymbol{B} \parallel [100]$ and strain $\perp [001]$<br>• $\boldsymbol{E} \parallel [010]$ and $\boldsymbol{B} \parallel [010]$ and strain $\perp [001]$<br>• $\boldsymbol{E} \parallel [010]$ and $\boldsymbol{B} \perp [001]$<br>• $\boldsymbol{E} \perp [001]$ and $\boldsymbol{B} \parallel [100]$<br>• $\boldsymbol{E} \perp [001]$ and $\boldsymbol{B} \parallel [010]$<br>• $\boldsymbol{E} \perp [001]$ and $\boldsymbol{B} \perp [001]$
P_bc (7.29) $\mathbb{Z}_1$	• $\boldsymbol{E} \parallel [100]$ and strain $\perp [001]$<br>• $\boldsymbol{E} \parallel [010]$ and strain $\perp [001]$<br>• $\boldsymbol{E} \perp [001]$
$P2_1$ (4.7) $\mathbb{Z}_1$	• $\boldsymbol{E} \parallel [100]$ and $\boldsymbol{B} \parallel [100]$ and strain $\perp [100]$
$Pn'a'2_1$ (33.148) $\mathbb{Z}_1$	• $\boldsymbol{E} \parallel [100]$ and $\boldsymbol{B} \parallel [100]$
$P2_1'$ (4.9) $\mathbb{Z}_1$	• $\boldsymbol{E} \parallel [100]$ and $\boldsymbol{B} \parallel [010]$ and strain $\perp [100]$<br>• $\boldsymbol{E} \parallel [100]$ and $\boldsymbol{B} \parallel [001]$ and strain $\perp [100]$<br>• $\boldsymbol{E} \parallel [100]$ and $\boldsymbol{B} \perp [100]$
$Pna'2_1'$ (33.147) $\mathbb{Z}_1$	• $\boldsymbol{E} \parallel [100]$ and $\boldsymbol{B} \parallel [001]$

Continued on next page

...continued

Subgroup & SI group	Perturbation
$Pmn'2_1'$ (31.126) $\mathbb{Z}_1$	• $\boldsymbol{E}$ ∥ [100] and $\boldsymbol{B}$ ∥ [010]
P_a2_1 (4.10) $\mathbb{Z}_1$	• $\boldsymbol{E}$ ∥ [100] and strain ⊥ [100]
P_bmn2_1 (31.129) $\mathbb{Z}_1$	• $\boldsymbol{E}$ ∥ [100]
$P2_1$ (4.7) $\mathbb{Z}_1$	• $\boldsymbol{E}$ ∥ [010] and $\boldsymbol{B}$ ∥ [010] and strain ⊥ [010]
$P2_1'$ (4.9) $\mathbb{Z}_1$	• $\boldsymbol{E}$ ∥ [010] and $\boldsymbol{B}$ ∥ [100] and strain ⊥ [010]<br>• $\boldsymbol{E}$ ∥ [010] and $\boldsymbol{B}$ ∥ [001] and strain ⊥ [010]<br>• $\boldsymbol{E}$ ∥ [010] and $\boldsymbol{B}$ ⊥ [010]
P_a2_1 (4.10) $\mathbb{Z}_1$	• $\boldsymbol{E}$ ∥ [010] and strain ⊥ [010]
$P\bar{1}$ (2.4) $\mathbb{Z}_2 \times \mathbb{Z}_2 \times \mathbb{Z}_2 \times \mathbb{Z}_4$	• $\boldsymbol{B}$ ∥ [100] and strain in generic direction<br>• $\boldsymbol{B}$ ∥ [010] and strain in generic direction<br>• $\boldsymbol{B}$ ∥ [001] and strain in generic direction<br>• $\boldsymbol{B}$ ⊥ [100] and strain ⊥ [010]<br>• $\boldsymbol{B}$ ⊥ [100] and strain ⊥ [001]<br>• $\boldsymbol{B}$ ⊥ [100] and strain in generic direction<br>• $\boldsymbol{B}$ ⊥ [010] and strain ⊥ [100]<br>• $\boldsymbol{B}$ ⊥ [010] and strain ⊥ [001]<br>• $\boldsymbol{B}$ ⊥ [010] and strain in generic direction<br>• $\boldsymbol{B}$ ⊥ [001] and strain ⊥ [100]<br>• $\boldsymbol{B}$ ⊥ [001] and strain ⊥ [010]<br>• $\boldsymbol{B}$ ⊥ [001] and strain in generic direction<br>• $\boldsymbol{B}$ in generic direction
$P2_1/m$ (11.50) $\mathbb{Z}_2$	• $\boldsymbol{B}$ ∥ [010] and strain ⊥ [010]
$P2_1'/c'$ (14.79) $\mathbb{Z}_2 \times \mathbb{Z}_4$	• $\boldsymbol{B}$ ∥ [100] and strain ⊥ [010]<br>• $\boldsymbol{B}$ ∥ [001] and strain ⊥ [010]<br>• $\boldsymbol{B}$ ⊥ [010]
$P_S\bar{1}$ (2.7) $\mathbb{Z}_2 \times \mathbb{Z}_2 \times \mathbb{Z}_4$	• strain in generic direction
P_a2_1/m (11.55) $\mathbb{Z}_2$	• strain ⊥ [010]
Pm (6.18) $\mathbb{Z}_1$	• $\boldsymbol{E}$ ∥ [010] and $\boldsymbol{B}$ ∥ [100] and strain ⊥ [100]<br>• $\boldsymbol{E}$ ∥ [001] and $\boldsymbol{B}$ ∥ [100] and strain ⊥ [100]<br>• $\boldsymbol{E}$ ⊥ [100] and $\boldsymbol{B}$ ∥ [100]
$Pmn'2_1'$ (31.126) $\mathbb{Z}_1$	• $\boldsymbol{E}$ ∥ [010] and $\boldsymbol{B}$ ∥ [100]
$P2_1/m$ (11.50) $\mathbb{Z}_2$	• $\boldsymbol{B}$ ∥ [100] and strain ⊥ [100]

Continued on next page

...continued

Subgroup & SI group	Perturbation
Pc' (7.26) $\mathbb{Z}_1$	• $\boldsymbol{E} \parallel [010]$ and $\boldsymbol{B} \parallel [010]$ and strain $\perp [100]$<br>• $\boldsymbol{E} \parallel [010]$ and $\boldsymbol{B} \parallel [001]$ and strain $\perp [100]$<br>• $\boldsymbol{E} \parallel [010]$ and $\boldsymbol{B} \perp [100]$<br>• $\boldsymbol{E} \parallel [001]$ and $\boldsymbol{B} \parallel [010]$ and strain $\perp [100]$<br>• $\boldsymbol{E} \parallel [001]$ and $\boldsymbol{B} \parallel [001]$ and strain $\perp [100]$<br>• $\boldsymbol{E} \parallel [001]$ and $\boldsymbol{B} \perp [100]$<br>• $\boldsymbol{E} \perp [100]$ and $\boldsymbol{B} \parallel [010]$<br>• $\boldsymbol{E} \perp [100]$ and $\boldsymbol{B} \parallel [001]$<br>• $\boldsymbol{E} \perp [100]$ and $\boldsymbol{B} \perp [100]$
$Pn'a'2_1$ (33.148) $\mathbb{Z}_1$	• $\boldsymbol{E} \parallel [010]$ and $\boldsymbol{B} \parallel [010]$
$Pna'2_1'$ (33.147) $\mathbb{Z}_1$	• $\boldsymbol{E} \parallel [010]$ and $\boldsymbol{B} \parallel [001]$
$P2_1'/c'$ (14.79) $\mathbb{Z}_2 \times \mathbb{Z}_4$	• $\boldsymbol{B} \parallel [010]$ and strain $\perp [100]$<br>• $\boldsymbol{B} \parallel [001]$ and strain $\perp [100]$<br>• $\boldsymbol{B} \perp [100]$
P_am (6.21) $\mathbb{Z}_1$	• $\boldsymbol{E} \parallel [010]$ and strain $\perp [100]$<br>• $\boldsymbol{E} \parallel [001]$ and strain $\perp [100]$<br>• $\boldsymbol{E} \perp [100]$
P_bmn2_1 (31.129) $\mathbb{Z}_1$	• $\boldsymbol{E} \parallel [010]$
P_a2_1/m (11.55) $\mathbb{Z}_2$	• strain $\perp [100]$
$P2$ (3.1) $\mathbb{Z}_2$	• $\boldsymbol{E} \parallel [001]$ and $\boldsymbol{B} \parallel [001]$ and strain $\perp [001]$
$P2/c$ (13.65) $\mathbb{Z}_2 \times \mathbb{Z}_2$	• $\boldsymbol{B} \parallel [001]$ and strain $\perp [001]$
$Pc'c'2$ (27.81) $\mathbb{Z}_2$	• $\boldsymbol{E} \parallel [001]$ and $\boldsymbol{B} \parallel [001]$
$Pc'c'n$ (56.369) $\mathbb{Z}_2 \times \mathbb{Z}_2$	• $\boldsymbol{B} \parallel [001]$
$P2_1'$ (4.9) $\mathbb{Z}_1$	• $\boldsymbol{E} \parallel [001]$ and $\boldsymbol{B} \parallel [100]$ and strain $\perp [001]$<br>• $\boldsymbol{E} \parallel [001]$ and $\boldsymbol{B} \parallel [010]$ and strain $\perp [001]$<br>• $\boldsymbol{E} \parallel [001]$ and $\boldsymbol{B} \perp [001]$
$P2_1'/c'$ (14.79) $\mathbb{Z}_2 \times \mathbb{Z}_4$	• $\boldsymbol{B} \parallel [100]$ and strain $\perp [001]$<br>• $\boldsymbol{B} \parallel [010]$ and strain $\perp [001]$<br>• $\boldsymbol{B} \perp [001]$
$Pmc'2_1'$ (26.69) $\mathbb{Z}_1$	• $\boldsymbol{E} \parallel [001]$ and $\boldsymbol{B} \parallel [100]$
$Pn'ma'$ (62.448) $\mathbb{Z}_2$	• $\boldsymbol{B} \parallel [100]$
$Pmc'2_1'$ (26.69) $\mathbb{Z}_1$	• $\boldsymbol{E} \parallel [001]$ and $\boldsymbol{B} \parallel [010]$
$Pn'ma'$ (62.448) $\mathbb{Z}_2$	• $\boldsymbol{B} \parallel [010]$

Continued on next page

…continued

Subgroup & SI group	Perturbation
P_b2 (3.5) $\mathbb{Z}_2$	• $\boldsymbol{E}$ $\parallel$ [001] and strain $\perp$ [001]
P_b2/c (13.71) $\mathbb{Z}_2 \times \mathbb{Z}_2$	• strain $\perp$ [001]
P_cmm2 (25.61) $\mathbb{Z}_1$	• $\boldsymbol{E}$ $\parallel$ [001]

Table. S 524: Subgroups of P_Bmmn (59.414) that can be realized by applying $\boldsymbol{E}/\boldsymbol{B}$ fields and/or uniaxial strains.

Subgroup & SI group	Perturbation
Pc' (7.26) $\mathbb{Z}_1$	• $\boldsymbol{E}$ $\parallel$ [100] and $\boldsymbol{B}$ $\parallel$ [100] and strain $\perp$ [001]<br>• $\boldsymbol{E}$ $\parallel$ [100] and $\boldsymbol{B}$ $\parallel$ [010] and strain $\perp$ [001]<br>• $\boldsymbol{E}$ $\parallel$ [100] and $\boldsymbol{B}$ $\perp$ [001]<br>• $\boldsymbol{E}$ $\parallel$ [010] and $\boldsymbol{B}$ $\parallel$ [100] and strain $\perp$ [001]<br>• $\boldsymbol{E}$ $\parallel$ [010] and $\boldsymbol{B}$ $\parallel$ [010] and strain $\perp$ [001]<br>• $\boldsymbol{E}$ $\parallel$ [010] and $\boldsymbol{B}$ $\perp$ [001]<br>• $\boldsymbol{E}$ $\perp$ [001] and $\boldsymbol{B}$ $\parallel$ [100]<br>• $\boldsymbol{E}$ $\perp$ [001] and $\boldsymbol{B}$ $\parallel$ [010]<br>• $\boldsymbol{E}$ $\perp$ [001] and $\boldsymbol{B}$ $\perp$ [001]
$P2'$ (3.3) $\mathbb{Z}_1$	• $\boldsymbol{E}$ $\parallel$ [100] and $\boldsymbol{B}$ $\parallel$ [010] and strain $\perp$ [100]<br>• $\boldsymbol{E}$ $\parallel$ [100] and $\boldsymbol{B}$ $\parallel$ [001] and strain $\perp$ [100]<br>• $\boldsymbol{E}$ $\parallel$ [100] and $\boldsymbol{B}$ $\perp$ [100]
Pm (6.18) $\mathbb{Z}_1$	• $\boldsymbol{E}$ $\parallel$ [100] and $\boldsymbol{B}$ $\parallel$ [010] and strain $\perp$ [010]<br>• $\boldsymbol{E}$ $\parallel$ [001] and $\boldsymbol{B}$ $\parallel$ [010] and strain $\perp$ [010]<br>• $\boldsymbol{E}$ $\perp$ [010] and $\boldsymbol{B}$ $\parallel$ [010]
$Pma'2'$ (28.90) $\mathbb{Z}_1$	• $\boldsymbol{E}$ $\parallel$ [100] and $\boldsymbol{B}$ $\parallel$ [010]

Continued on next page

...continued

Subgroup & SI group	Perturbation
P_S1 (1.3) $\mathbb{Z}_1$	• $\boldsymbol{E}$ ∥ [100] and strain in generic direction<br>• $\boldsymbol{E}$ ∥ [010] and strain in generic direction<br>• $\boldsymbol{E}$ ∥ [001] and strain in generic direction<br>• $\boldsymbol{E} \perp$ [100] and strain $\perp$ [010]<br>• $\boldsymbol{E} \perp$ [100] and strain $\perp$ [001]<br>• $\boldsymbol{E} \perp$ [100] and strain in generic direction<br>• $\boldsymbol{E} \perp$ [010] and strain $\perp$ [100]<br>• $\boldsymbol{E} \perp$ [010] and strain $\perp$ [001]<br>• $\boldsymbol{E} \perp$ [010] and strain in generic direction<br>• $\boldsymbol{E} \perp$ [001] and strain $\perp$ [100]<br>• $\boldsymbol{E} \perp$ [001] and strain $\perp$ [010]<br>• $\boldsymbol{E} \perp$ [001] and strain in generic direction<br>• $\boldsymbol{E}$ in generic direction
Pc (7.24) $\mathbb{Z}_1$	• $\boldsymbol{E}$ ∥ [100] and $\boldsymbol{B}$ ∥ [001] and strain $\perp$ [001]<br>• $\boldsymbol{E}$ ∥ [010] and $\boldsymbol{B}$ ∥ [001] and strain $\perp$ [001]<br>• $\boldsymbol{E} \perp$ [001] and $\boldsymbol{B}$ ∥ [001]
P_Cc (7.30) $\mathbb{Z}_1$	• $\boldsymbol{E}$ ∥ [100] and strain $\perp$ [001]<br>• $\boldsymbol{E}$ ∥ [010] and strain $\perp$ [001]<br>• $\boldsymbol{E} \perp$ [001]
$P2_1$ (4.7) $\mathbb{Z}_1$	• $\boldsymbol{E}$ ∥ [100] and $\boldsymbol{B}$ ∥ [100] and strain $\perp$ [100]
P_C2_1 (4.12) $\mathbb{Z}_1$	• $\boldsymbol{E}$ ∥ [100] and strain $\perp$ [100]
Pc' (7.26) $\mathbb{Z}_1$	• $\boldsymbol{E}$ ∥ [100] and $\boldsymbol{B}$ ∥ [100] and strain $\perp$ [010]<br>• $\boldsymbol{E}$ ∥ [100] and $\boldsymbol{B}$ ∥ [001] and strain $\perp$ [010]<br>• $\boldsymbol{E}$ ∥ [100] and $\boldsymbol{B} \perp$ [010]<br>• $\boldsymbol{E}$ ∥ [001] and $\boldsymbol{B}$ ∥ [100] and strain $\perp$ [010]<br>• $\boldsymbol{E}$ ∥ [001] and $\boldsymbol{B}$ ∥ [001] and strain $\perp$ [010]<br>• $\boldsymbol{E}$ ∥ [001] and $\boldsymbol{B} \perp$ [010]<br>• $\boldsymbol{E} \perp$ [010] and $\boldsymbol{B}$ ∥ [100]<br>• $\boldsymbol{E} \perp$ [010] and $\boldsymbol{B}$ ∥ [001]<br>• $\boldsymbol{E} \perp$ [010] and $\boldsymbol{B} \perp$ [010]
P_am (6.21) $\mathbb{Z}_1$	• $\boldsymbol{E}$ ∥ [100] and strain $\perp$ [010]<br>• $\boldsymbol{E}$ ∥ [001] and strain $\perp$ [010]<br>• $\boldsymbol{E} \perp$ [010]
$Pn'n2'$ (34.158) $\mathbb{Z}_1$	• $\boldsymbol{E}$ ∥ [100] and $\boldsymbol{B}$ ∥ [001]
$Pn'a'2_1$ (33.148) $\mathbb{Z}_1$	• $\boldsymbol{E}$ ∥ [100] and $\boldsymbol{B}$ ∥ [100]
P_Amn2_1 (31.131) $\mathbb{Z}_1$	• $\boldsymbol{E}$ ∥ [100]

Continued on next page

…continued

Subgroup & SI group	Perturbation
Pc' (7.26) $\mathbb{Z}_1$	• $\boldsymbol{E}$ ∥ [010] and $\boldsymbol{B}$ ∥ [010] and strain ⊥ [100]<br>• $\boldsymbol{E}$ ∥ [010] and $\boldsymbol{B}$ ∥ [001] and strain ⊥ [100]<br>• $\boldsymbol{E}$ ∥ [010] and $\boldsymbol{B}$ ⊥ [100]<br>• $\boldsymbol{E}$ ∥ [001] and $\boldsymbol{B}$ ∥ [010] and strain ⊥ [100]<br>• $\boldsymbol{E}$ ∥ [001] and $\boldsymbol{B}$ ∥ [001] and strain ⊥ [100]<br>• $\boldsymbol{E}$ ∥ [001] and $\boldsymbol{B}$ ⊥ [100]<br>• $\boldsymbol{E}$ ⊥ [100] and $\boldsymbol{B}$ ∥ [010]<br>• $\boldsymbol{E}$ ⊥ [100] and $\boldsymbol{B}$ ∥ [001]<br>• $\boldsymbol{E}$ ⊥ [100] and $\boldsymbol{B}$ ⊥ [100]
$P2_1$ (4.7) $\mathbb{Z}_1$	• $\boldsymbol{E}$ ∥ [010] and $\boldsymbol{B}$ ∥ [010] and strain ⊥ [010]
$Pc'a'2_1$ (29.103) $\mathbb{Z}_1$	• $\boldsymbol{E}$ ∥ [010] and $\boldsymbol{B}$ ∥ [010]
$P\bar{1}$ (2.4) $\mathbb{Z}_2 \times \mathbb{Z}_2 \times \mathbb{Z}_2 \times \mathbb{Z}_4$	• $\boldsymbol{B}$ ∥ [100] and strain in generic direction<br>• $\boldsymbol{B}$ ∥ [010] and strain in generic direction<br>• $\boldsymbol{B}$ ∥ [001] and strain in generic direction<br>• $\boldsymbol{B}$ ⊥ [100] and strain ⊥ [010]<br>• $\boldsymbol{B}$ ⊥ [100] and strain ⊥ [001]<br>• $\boldsymbol{B}$ ⊥ [100] and strain in generic direction<br>• $\boldsymbol{B}$ ⊥ [010] and strain ⊥ [100]<br>• $\boldsymbol{B}$ ⊥ [010] and strain ⊥ [001]<br>• $\boldsymbol{B}$ ⊥ [010] and strain in generic direction<br>• $\boldsymbol{B}$ ⊥ [001] and strain ⊥ [100]<br>• $\boldsymbol{B}$ ⊥ [001] and strain ⊥ [010]<br>• $\boldsymbol{B}$ ⊥ [001] and strain in generic direction<br>• $\boldsymbol{B}$ in generic direction
$P2'/c'$ (13.69) $\mathbb{Z}_2 \times \mathbb{Z}_2 \times \mathbb{Z}_4$	• $\boldsymbol{B}$ ∥ [010] and strain ⊥ [100]<br>• $\boldsymbol{B}$ ∥ [001] and strain ⊥ [100]<br>• $\boldsymbol{B}$ ⊥ [100]
$P2_1/m$ (11.50) $\mathbb{Z}_2$	• $\boldsymbol{B}$ ∥ [010] and strain ⊥ [010]
$P2_1'$ (4.9) $\mathbb{Z}_1$	• $\boldsymbol{E}$ ∥ [001] and $\boldsymbol{B}$ ∥ [100] and strain ⊥ [001]<br>• $\boldsymbol{E}$ ∥ [001] and $\boldsymbol{B}$ ∥ [010] and strain ⊥ [001]<br>• $\boldsymbol{E}$ ∥ [001] and $\boldsymbol{B}$ ⊥ [001]
$Pmc'2_1'$ (26.69) $\mathbb{Z}_1$	• $\boldsymbol{E}$ ∥ [001] and $\boldsymbol{B}$ ∥ [010]
$P2_1'/c'$ (14.79) $\mathbb{Z}_2 \times \mathbb{Z}_4$	• $\boldsymbol{B}$ ∥ [100] and strain ⊥ [001]<br>• $\boldsymbol{B}$ ∥ [010] and strain ⊥ [001]<br>• $\boldsymbol{B}$ ⊥ [001]
$Pb'c'm$ (57.382) $\mathbb{Z}_2$	• $\boldsymbol{B}$ ∥ [010]

Continued on next page

…continued

Subgroup & SI group	Perturbation
Pm (6.18) $\mathbb{Z}_1$	• $\boldsymbol{E} \parallel [010]$ and $\boldsymbol{B} \parallel [100]$ and strain $\perp [100]$<br>• $\boldsymbol{E} \parallel [001]$ and $\boldsymbol{B} \parallel [100]$ and strain $\perp [100]$<br>• $\boldsymbol{E} \perp [100]$ and $\boldsymbol{B} \parallel [100]$
P_Cm (6.23) $\mathbb{Z}_1$	• $\boldsymbol{E} \parallel [010]$ and strain $\perp [100]$<br>• $\boldsymbol{E} \parallel [001]$ and strain $\perp [100]$<br>• $\boldsymbol{E} \perp [100]$
$P2_1/m$ (11.50) $\mathbb{Z}_2$	• $\boldsymbol{B} \parallel [100]$ and strain $\perp [100]$
$Pmn'2_1'$ (31.126) $\mathbb{Z}_1$	• $\boldsymbol{E} \parallel [001]$ and $\boldsymbol{B} \parallel [100]$
$P2_1'$ (4.9) $\mathbb{Z}_1$	• $\boldsymbol{E} \parallel [010]$ and $\boldsymbol{B} \parallel [100]$ and strain $\perp [010]$<br>• $\boldsymbol{E} \parallel [010]$ and $\boldsymbol{B} \parallel [001]$ and strain $\perp [010]$<br>• $\boldsymbol{E} \parallel [010]$ and $\boldsymbol{B} \perp [010]$
$Pna'2_1'$ (33.147) $\mathbb{Z}_1$	• $\boldsymbol{E} \parallel [010]$ and $\boldsymbol{B} \parallel [001]$
P_a2_1 (4.10) $\mathbb{Z}_1$	• $\boldsymbol{E} \parallel [010]$ and strain $\perp [010]$
$P2_1'/c'$ (14.79) $\mathbb{Z}_2 \times \mathbb{Z}_4$	• $\boldsymbol{B} \parallel [100]$ and strain $\perp [010]$<br>• $\boldsymbol{B} \parallel [001]$ and strain $\perp [010]$<br>• $\boldsymbol{B} \perp [010]$
$Pmc'2_1'$ (26.69) $\mathbb{Z}_1$	• $\boldsymbol{E} \parallel [010]$ and $\boldsymbol{B} \parallel [100]$
P_Cmn2_1 (31.133) $\mathbb{Z}_1$	• $\boldsymbol{E} \parallel [010]$
$Pn'ma'$ (62.448) $\mathbb{Z}_2$	• $\boldsymbol{B} \parallel [100]$
$P_S\bar{1}$ (2.7) $\mathbb{Z}_2 \times \mathbb{Z}_2 \times \mathbb{Z}_4$	• strain in generic direction
P_C2_1/m (11.57) $\mathbb{Z}_2$	• strain $\perp [100]$
P_a2_1/m (11.55) $\mathbb{Z}_2$	• strain $\perp [010]$
$P2$ (3.1) $\mathbb{Z}_2$	• $\boldsymbol{E} \parallel [001]$ and $\boldsymbol{B} \parallel [001]$ and strain $\perp [001]$
$Pn'c'2$ (30.115) $\mathbb{Z}_1$	• $\boldsymbol{E} \parallel [001]$ and $\boldsymbol{B} \parallel [001]$
$P2/c$ (13.65) $\mathbb{Z}_2 \times \mathbb{Z}_2$	• $\boldsymbol{B} \parallel [001]$ and strain $\perp [001]$
P_C2 (3.6) $\mathbb{Z}_1$	• $\boldsymbol{E} \parallel [001]$ and strain $\perp [001]$
P_Amm2 (25.64) $\mathbb{Z}_1$	• $\boldsymbol{E} \parallel [001]$
$Pnn'a'$ (52.311) $\mathbb{Z}_2$	• $\boldsymbol{B} \parallel [001]$
P_C2/c (13.74) $\mathbb{Z}_2$	• strain $\perp [001]$

Table. S 525: Subgroups of P_Cmmn (59.415) that can be realized by applying $\boldsymbol{E}/\boldsymbol{B}$ fields and/or uniaxial strains.

Subgroup & SI group	Perturbation
Pm' (6.20) $\mathbb{Z}_1$	• $\boldsymbol{E}$ ∥ [100] and $\boldsymbol{B}$ ∥ [100] and strain ⊥ [001]<br>• $\boldsymbol{E}$ ∥ [100] and $\boldsymbol{B}$ ∥ [010] and strain ⊥ [001]<br>• $\boldsymbol{E}$ ∥ [100] and $\boldsymbol{B}$ ⊥ [001]<br>• $\boldsymbol{E}$ ∥ [010] and $\boldsymbol{B}$ ∥ [100] and strain ⊥ [001]<br>• $\boldsymbol{E}$ ∥ [010] and $\boldsymbol{B}$ ∥ [010] and strain ⊥ [001]<br>• $\boldsymbol{E}$ ∥ [010] and $\boldsymbol{B}$ ⊥ [001]<br>• $\boldsymbol{E}$ ⊥ [001] and $\boldsymbol{B}$ ∥ [100]<br>• $\boldsymbol{E}$ ⊥ [001] and $\boldsymbol{B}$ ∥ [010]<br>• $\boldsymbol{E}$ ⊥ [001] and $\boldsymbol{B}$ ⊥ [001]
Pm (6.18) $\mathbb{Z}_1$	• $\boldsymbol{E}$ ∥ [100] and $\boldsymbol{B}$ ∥ [010] and strain ⊥ [010]<br>• $\boldsymbol{E}$ ∥ [001] and $\boldsymbol{B}$ ∥ [010] and strain ⊥ [010]<br>• $\boldsymbol{E}$ ⊥ [010] and $\boldsymbol{B}$ ∥ [010]
$P2'$ (3.3) $\mathbb{Z}_1$	• $\boldsymbol{E}$ ∥ [100] and $\boldsymbol{B}$ ∥ [010] and strain ⊥ [100]<br>• $\boldsymbol{E}$ ∥ [100] and $\boldsymbol{B}$ ∥ [001] and strain ⊥ [100]<br>• $\boldsymbol{E}$ ∥ [100] and $\boldsymbol{B}$ ⊥ [100]
$Pm'm2'$ (25.59) $\mathbb{Z}_1$	• $\boldsymbol{E}$ ∥ [100] and $\boldsymbol{B}$ ∥ [010]
P_S1 (1.3) $\mathbb{Z}_1$	• $\boldsymbol{E}$ ∥ [100] and strain in generic direction<br>• $\boldsymbol{E}$ ∥ [010] and strain in generic direction<br>• $\boldsymbol{E}$ ∥ [001] and strain in generic direction<br>• $\boldsymbol{E}$ ⊥ [100] and strain ⊥ [010]<br>• $\boldsymbol{E}$ ⊥ [100] and strain ⊥ [001]<br>• $\boldsymbol{E}$ ⊥ [100] and strain in generic direction<br>• $\boldsymbol{E}$ ⊥ [010] and strain ⊥ [100]<br>• $\boldsymbol{E}$ ⊥ [010] and strain ⊥ [001]<br>• $\boldsymbol{E}$ ⊥ [010] and strain in generic direction<br>• $\boldsymbol{E}$ ⊥ [001] and strain ⊥ [100]<br>• $\boldsymbol{E}$ ⊥ [001] and strain ⊥ [010]<br>• $\boldsymbol{E}$ ⊥ [001] and strain in generic direction<br>• $\boldsymbol{E}$ in generic direction
Pc (7.24) $\mathbb{Z}_1$	• $\boldsymbol{E}$ ∥ [100] and $\boldsymbol{B}$ ∥ [001] and strain ⊥ [001]<br>• $\boldsymbol{E}$ ∥ [010] and $\boldsymbol{B}$ ∥ [001] and strain ⊥ [001]<br>• $\boldsymbol{E}$ ⊥ [001] and $\boldsymbol{B}$ ∥ [001]
P_cc (7.28) $\mathbb{Z}_1$	• $\boldsymbol{E}$ ∥ [100] and strain ⊥ [001]<br>• $\boldsymbol{E}$ ∥ [010] and strain ⊥ [001]<br>• $\boldsymbol{E}$ ⊥ [001]

Continued on next page

...continued

Subgroup & SI group	Perturbation
Pc' (7.26) $\mathbb{Z}_1$	• $\boldsymbol{E}$ ∥ [100] and $\boldsymbol{B}$ ∥ [100] and strain ⊥ [010]<br>• $\boldsymbol{E}$ ∥ [100] and $\boldsymbol{B}$ ∥ [001] and strain ⊥ [010]<br>• $\boldsymbol{E}$ ∥ [100] and $\boldsymbol{B}$ ⊥ [010]<br>• $\boldsymbol{E}$ ∥ [001] and $\boldsymbol{B}$ ∥ [100] and strain ⊥ [010]<br>• $\boldsymbol{E}$ ∥ [001] and $\boldsymbol{B}$ ∥ [001] and strain ⊥ [010]<br>• $\boldsymbol{E}$ ∥ [001] and $\boldsymbol{B}$ ⊥ [010]<br>• $\boldsymbol{E}$ ⊥ [010] and $\boldsymbol{B}$ ∥ [100]<br>• $\boldsymbol{E}$ ⊥ [010] and $\boldsymbol{B}$ ∥ [001]<br>• $\boldsymbol{E}$ ⊥ [010] and $\boldsymbol{B}$ ⊥ [010]
P_Cm (6.23) $\mathbb{Z}_1$	• $\boldsymbol{E}$ ∥ [100] and strain ⊥ [010]<br>• $\boldsymbol{E}$ ∥ [001] and strain ⊥ [010]<br>• $\boldsymbol{E}$ ⊥ [010]
$Pnc'2'$ (30.114) $\mathbb{Z}_1$	• $\boldsymbol{E}$ ∥ [100] and $\boldsymbol{B}$ ∥ [001]
$P2_1$ (4.7) $\mathbb{Z}_1$	• $\boldsymbol{E}$ ∥ [100] and $\boldsymbol{B}$ ∥ [100] and strain ⊥ [100]
P_C2_1 (4.12) $\mathbb{Z}_1$	• $\boldsymbol{E}$ ∥ [100] and strain ⊥ [100]
$Pm'c'2_1$ (26.70) $\mathbb{Z}_1$	• $\boldsymbol{E}$ ∥ [100] and $\boldsymbol{B}$ ∥ [100]
P_Bmn2_1 (31.132) $\mathbb{Z}_1$	• $\boldsymbol{E}$ ∥ [100]
Pc' (7.26) $\mathbb{Z}_1$	• $\boldsymbol{E}$ ∥ [010] and $\boldsymbol{B}$ ∥ [010] and strain ⊥ [100]<br>• $\boldsymbol{E}$ ∥ [010] and $\boldsymbol{B}$ ∥ [001] and strain ⊥ [100]<br>• $\boldsymbol{E}$ ∥ [010] and $\boldsymbol{B}$ ⊥ [100]<br>• $\boldsymbol{E}$ ∥ [001] and $\boldsymbol{B}$ ∥ [010] and strain ⊥ [100]<br>• $\boldsymbol{E}$ ∥ [001] and $\boldsymbol{B}$ ∥ [001] and strain ⊥ [100]<br>• $\boldsymbol{E}$ ∥ [001] and $\boldsymbol{B}$ ⊥ [100]<br>• $\boldsymbol{E}$ ⊥ [100] and $\boldsymbol{B}$ ∥ [010]<br>• $\boldsymbol{E}$ ⊥ [100] and $\boldsymbol{B}$ ∥ [001]<br>• $\boldsymbol{E}$ ⊥ [100] and $\boldsymbol{B}$ ⊥ [100]
$P2_1$ (4.7) $\mathbb{Z}_1$	• $\boldsymbol{E}$ ∥ [010] and $\boldsymbol{B}$ ∥ [010] and strain ⊥ [010]
$Pm'c'2_1$ (26.70) $\mathbb{Z}_1$	• $\boldsymbol{E}$ ∥ [010] and $\boldsymbol{B}$ ∥ [010]
$P2'$ (3.3) $\mathbb{Z}_1$	• $\boldsymbol{E}$ ∥ [001] and $\boldsymbol{B}$ ∥ [100] and strain ⊥ [001]<br>• $\boldsymbol{E}$ ∥ [001] and $\boldsymbol{B}$ ∥ [010] and strain ⊥ [001]<br>• $\boldsymbol{E}$ ∥ [001] and $\boldsymbol{B}$ ⊥ [001]
$Pma'2'$ (28.90) $\mathbb{Z}_1$	• $\boldsymbol{E}$ ∥ [001] and $\boldsymbol{B}$ ∥ [010]

Continued on next page

…continued

Subgroup & SI group	Perturbation
$P\bar{1}$ (2.4) $\mathbb{Z}_2 \times \mathbb{Z}_2 \times \mathbb{Z}_2 \times \mathbb{Z}_4$	• $\boldsymbol{B} \parallel [100]$ and strain in generic direction<br>• $\boldsymbol{B} \parallel [010]$ and strain in generic direction<br>• $\boldsymbol{B} \parallel [001]$ and strain in generic direction<br>• $\boldsymbol{B} \perp [100]$ and strain $\perp [010]$<br>• $\boldsymbol{B} \perp [100]$ and strain $\perp [001]$<br>• $\boldsymbol{B} \perp [100]$ and strain in generic direction<br>• $\boldsymbol{B} \perp [010]$ and strain $\perp [100]$<br>• $\boldsymbol{B} \perp [010]$ and strain $\perp [001]$<br>• $\boldsymbol{B} \perp [010]$ and strain in generic direction<br>• $\boldsymbol{B} \perp [001]$ and strain $\perp [100]$<br>• $\boldsymbol{B} \perp [001]$ and strain $\perp [010]$<br>• $\boldsymbol{B} \perp [001]$ and strain in generic direction<br>• $\boldsymbol{B}$ in generic direction
$P2'/c'$ (13.69) $\mathbb{Z}_2 \times \mathbb{Z}_2 \times \mathbb{Z}_4$	• $\boldsymbol{B} \parallel [010]$ and strain $\perp [100]$<br>• $\boldsymbol{B} \parallel [001]$ and strain $\perp [100]$<br>• $\boldsymbol{B} \perp [100]$
$P2_1/m$ (11.50) $\mathbb{Z}_2$	• $\boldsymbol{B} \parallel [010]$ and strain $\perp [010]$
$P2'/m'$ (10.46) $\mathbb{Z}_2 \times \mathbb{Z}_2 \times \mathbb{Z}_2 \times \mathbb{Z}_4$	• $\boldsymbol{B} \parallel [100]$ and strain $\perp [001]$<br>• $\boldsymbol{B} \parallel [010]$ and strain $\perp [001]$<br>• $\boldsymbol{B} \perp [001]$
$Pmm'a'$ (51.295) $\mathbb{Z}_2$	• $\boldsymbol{B} \parallel [010]$
Pm (6.18) $\mathbb{Z}_1$	• $\boldsymbol{E} \parallel [010]$ and $\boldsymbol{B} \parallel [100]$ and strain $\perp [100]$<br>• $\boldsymbol{E} \parallel [001]$ and $\boldsymbol{B} \parallel [100]$ and strain $\perp [100]$<br>• $\boldsymbol{E} \perp [100]$ and $\boldsymbol{B} \parallel [100]$
P_Cm (6.23) $\mathbb{Z}_1$	• $\boldsymbol{E} \parallel [010]$ and strain $\perp [100]$<br>• $\boldsymbol{E} \parallel [001]$ and strain $\perp [100]$<br>• $\boldsymbol{E} \perp [100]$
$Pma'2'$ (28.90) $\mathbb{Z}_1$	• $\boldsymbol{E} \parallel [001]$ and $\boldsymbol{B} \parallel [100]$
$P2_1/m$ (11.50) $\mathbb{Z}_2$	• $\boldsymbol{B} \parallel [100]$ and strain $\perp [100]$
$P2'$ (3.3) $\mathbb{Z}_1$	• $\boldsymbol{E} \parallel [010]$ and $\boldsymbol{B} \parallel [100]$ and strain $\perp [010]$<br>• $\boldsymbol{E} \parallel [010]$ and $\boldsymbol{B} \parallel [001]$ and strain $\perp [010]$<br>• $\boldsymbol{E} \parallel [010]$ and $\boldsymbol{B} \perp [010]$
$Pnc'2'$ (30.114) $\mathbb{Z}_1$	• $\boldsymbol{E} \parallel [010]$ and $\boldsymbol{B} \parallel [001]$
P_C2_1 (4.12) $\mathbb{Z}_1$	• $\boldsymbol{E} \parallel [010]$ and strain $\perp [010]$

Continued on next page

…continued

Subgroup & SI group	Perturbation
$P2'/c'$ (13.69) $\mathbb{Z}_2 \times \mathbb{Z}_2 \times \mathbb{Z}_4$	• $\boldsymbol{B} \parallel [100]$ and strain $\perp [010]$<br>• $\boldsymbol{B} \parallel [001]$ and strain $\perp [010]$<br>• $\boldsymbol{B} \perp [010]$
$Pm'm2'$ (25.59) $\mathbb{Z}_1$	• $\boldsymbol{E} \parallel [010]$ and $\boldsymbol{B} \parallel [100]$
P_Bmn2_1 (31.132) $\mathbb{Z}_1$	• $\boldsymbol{E} \parallel [010]$
$Pmm'a'$ (51.295) $\mathbb{Z}_2$	• $\boldsymbol{B} \parallel [100]$
$P2$ (3.1) $\mathbb{Z}_2$	• $\boldsymbol{E} \parallel [001]$ and $\boldsymbol{B} \parallel [001]$ and strain $\perp [001]$
$Pb'a'2$ (32.138) $\mathbb{Z}_2$	• $\boldsymbol{E} \parallel [001]$ and $\boldsymbol{B} \parallel [001]$
P_a2 (3.4) $\mathbb{Z}_2$	• $\boldsymbol{E} \parallel [001]$ and strain $\perp [001]$
$P2/c$ (13.65) $\mathbb{Z}_2 \times \mathbb{Z}_2$	• $\boldsymbol{B} \parallel [001]$ and strain $\perp [001]$
P_Cmm2 (25.63) $\mathbb{Z}_1$	• $\boldsymbol{E} \parallel [001]$
$Pb'a'n$ (50.281) $\mathbb{Z}_2 \times \mathbb{Z}_2$	• $\boldsymbol{B} \parallel [001]$
$P_S\bar{1}$ (2.7) $\mathbb{Z}_2 \times \mathbb{Z}_2 \times \mathbb{Z}_4$	• strain in generic direction
P_C2_1/m (11.57) $\mathbb{Z}_2$	• strain $\perp [100]$
P_C2_1/m (11.57) $\mathbb{Z}_2$	• strain $\perp [010]$
P_c2/c (13.72) $\mathbb{Z}_2 \times \mathbb{Z}_2$	• strain $\perp [001]$

Table. S 526: Subgroups of P_Immn (59.416) that can be realized by applying $\boldsymbol{E}/\boldsymbol{B}$ fields and/or uniaxial strains.

Subgroup & SI group	Perturbation
Pm' (6.20) $\mathbb{Z}_1$	• $\boldsymbol{E} \parallel [100]$ and $\boldsymbol{B} \parallel [100]$ and strain $\perp [001]$<br>• $\boldsymbol{E} \parallel [100]$ and $\boldsymbol{B} \parallel [010]$ and strain $\perp [001]$<br>• $\boldsymbol{E} \parallel [100]$ and $\boldsymbol{B} \perp [001]$<br>• $\boldsymbol{E} \parallel [010]$ and $\boldsymbol{B} \parallel [100]$ and strain $\perp [001]$<br>• $\boldsymbol{E} \parallel [010]$ and $\boldsymbol{B} \parallel [010]$ and strain $\perp [001]$<br>• $\boldsymbol{E} \parallel [010]$ and $\boldsymbol{B} \perp [001]$<br>• $\boldsymbol{E} \perp [001]$ and $\boldsymbol{B} \parallel [100]$<br>• $\boldsymbol{E} \perp [001]$ and $\boldsymbol{B} \parallel [010]$<br>• $\boldsymbol{E} \perp [001]$ and $\boldsymbol{B} \perp [001]$

Continued on next page

...continued

Subgroup & SI group	Perturbation
Pm (6.18) $\mathbb{Z}_1$	• $\boldsymbol{E}$ ∥ [100] and $\boldsymbol{B}$ ∥ [010] and strain ⊥ [010]<br>• $\boldsymbol{E}$ ∥ [001] and $\boldsymbol{B}$ ∥ [010] and strain ⊥ [010]<br>• $\boldsymbol{E}$ ⊥ [010] and $\boldsymbol{B}$ ∥ [010]
$P2'$ (3.3) $\mathbb{Z}_1$	• $\boldsymbol{E}$ ∥ [100] and $\boldsymbol{B}$ ∥ [010] and strain ⊥ [100]<br>• $\boldsymbol{E}$ ∥ [100] and $\boldsymbol{B}$ ∥ [001] and strain ⊥ [100]<br>• $\boldsymbol{E}$ ∥ [100] and $\boldsymbol{B}$ ⊥ [100]
$Pm'm2'$ (25.59) $\mathbb{Z}_1$	• $\boldsymbol{E}$ ∥ [100] and $\boldsymbol{B}$ ∥ [010]
P_S1 (1.3) $\mathbb{Z}_1$	• $\boldsymbol{E}$ ∥ [100] and strain in generic direction<br>• $\boldsymbol{E}$ ∥ [010] and strain in generic direction<br>• $\boldsymbol{E}$ ∥ [001] and strain in generic direction<br>• $\boldsymbol{E}$ ⊥ [100] and strain ⊥ [010]<br>• $\boldsymbol{E}$ ⊥ [100] and strain ⊥ [001]<br>• $\boldsymbol{E}$ ⊥ [100] and strain in generic direction<br>• $\boldsymbol{E}$ ⊥ [010] and strain ⊥ [100]<br>• $\boldsymbol{E}$ ⊥ [010] and strain ⊥ [001]<br>• $\boldsymbol{E}$ ⊥ [010] and strain in generic direction<br>• $\boldsymbol{E}$ ⊥ [001] and strain ⊥ [100]<br>• $\boldsymbol{E}$ ⊥ [001] and strain ⊥ [010]<br>• $\boldsymbol{E}$ ⊥ [001] and strain in generic direction<br>• $\boldsymbol{E}$ in generic direction
Pc (7.24) $\mathbb{Z}_1$	• $\boldsymbol{E}$ ∥ [100] and $\boldsymbol{B}$ ∥ [001] and strain ⊥ [001]<br>• $\boldsymbol{E}$ ∥ [010] and $\boldsymbol{B}$ ∥ [001] and strain ⊥ [001]<br>• $\boldsymbol{E}$ ⊥ [001] and $\boldsymbol{B}$ ∥ [001]
P_Ac (7.31) $\mathbb{Z}_1$	• $\boldsymbol{E}$ ∥ [100] and strain ⊥ [001]<br>• $\boldsymbol{E}$ ∥ [010] and strain ⊥ [001]<br>• $\boldsymbol{E}$ ⊥ [001]
Pc' (7.26) $\mathbb{Z}_1$	• $\boldsymbol{E}$ ∥ [100] and $\boldsymbol{B}$ ∥ [100] and strain ⊥ [010]<br>• $\boldsymbol{E}$ ∥ [100] and $\boldsymbol{B}$ ∥ [001] and strain ⊥ [010]<br>• $\boldsymbol{E}$ ∥ [100] and $\boldsymbol{B}$ ⊥ [010]<br>• $\boldsymbol{E}$ ∥ [001] and $\boldsymbol{B}$ ∥ [100] and strain ⊥ [010]<br>• $\boldsymbol{E}$ ∥ [001] and $\boldsymbol{B}$ ∥ [001] and strain ⊥ [010]<br>• $\boldsymbol{E}$ ∥ [001] and $\boldsymbol{B}$ ⊥ [010]<br>• $\boldsymbol{E}$ ⊥ [010] and $\boldsymbol{B}$ ∥ [100]<br>• $\boldsymbol{E}$ ⊥ [010] and $\boldsymbol{B}$ ∥ [001]<br>• $\boldsymbol{E}$ ⊥ [010] and $\boldsymbol{B}$ ⊥ [010]
P_Cm (6.23) $\mathbb{Z}_1$	• $\boldsymbol{E}$ ∥ [100] and strain ⊥ [010]<br>• $\boldsymbol{E}$ ∥ [001] and strain ⊥ [010]<br>• $\boldsymbol{E}$ ⊥ [010]

Continued on next page

...continued

Subgroup & SI group	Perturbation
$Pn'n2'$ (34.158) $\mathbb{Z}_1$	• $\boldsymbol{E} \parallel [100]$ and $\boldsymbol{B} \parallel [001]$
$P2_1$ (4.7) $\mathbb{Z}_1$	• $\boldsymbol{E} \parallel [100]$ and $\boldsymbol{B} \parallel [100]$ and strain $\perp [100]$
P_C2_1 (4.12) $\mathbb{Z}_1$	• $\boldsymbol{E} \parallel [100]$ and strain $\perp [100]$
$Pm'n'2_1$ (31.127) $\mathbb{Z}_1$	• $\boldsymbol{E} \parallel [100]$ and $\boldsymbol{B} \parallel [100]$
P_Imn2_1 (31.134) $\mathbb{Z}_1$	• $\boldsymbol{E} \parallel [100]$
Pc' (7.26) $\mathbb{Z}_1$	• $\boldsymbol{E} \parallel [010]$ and $\boldsymbol{B} \parallel [010]$ and strain $\perp [100]$<br>• $\boldsymbol{E} \parallel [010]$ and $\boldsymbol{B} \parallel [001]$ and strain $\perp [100]$<br>• $\boldsymbol{E} \parallel [010]$ and $\boldsymbol{B} \perp [100]$<br>• $\boldsymbol{E} \parallel [001]$ and $\boldsymbol{B} \parallel [010]$ and strain $\perp [100]$<br>• $\boldsymbol{E} \parallel [001]$ and $\boldsymbol{B} \parallel [001]$ and strain $\perp [100]$<br>• $\boldsymbol{E} \parallel [001]$ and $\boldsymbol{B} \perp [100]$<br>• $\boldsymbol{E} \perp [100]$ and $\boldsymbol{B} \parallel [010]$<br>• $\boldsymbol{E} \perp [100]$ and $\boldsymbol{B} \parallel [001]$<br>• $\boldsymbol{E} \perp [100]$ and $\boldsymbol{B} \perp [100]$
$P2_1$ (4.7) $\mathbb{Z}_1$	• $\boldsymbol{E} \parallel [010]$ and $\boldsymbol{B} \parallel [010]$ and strain $\perp [010]$
$Pm'n'2_1$ (31.127) $\mathbb{Z}_1$	• $\boldsymbol{E} \parallel [010]$ and $\boldsymbol{B} \parallel [010]$
$P2_1'$ (4.9) $\mathbb{Z}_1$	• $\boldsymbol{E} \parallel [001]$ and $\boldsymbol{B} \parallel [100]$ and strain $\perp [001]$<br>• $\boldsymbol{E} \parallel [001]$ and $\boldsymbol{B} \parallel [010]$ and strain $\perp [001]$<br>• $\boldsymbol{E} \parallel [001]$ and $\boldsymbol{B} \perp [001]$
$Pmn'2_1'$ (31.126) $\mathbb{Z}_1$	• $\boldsymbol{E} \parallel [001]$ and $\boldsymbol{B} \parallel [010]$
$P\bar{1}$ (2.4) $\mathbb{Z}_2 \times \mathbb{Z}_2 \times \mathbb{Z}_2 \times \mathbb{Z}_4$	• $\boldsymbol{B} \parallel [100]$ and strain in generic direction<br>• $\boldsymbol{B} \parallel [010]$ and strain in generic direction<br>• $\boldsymbol{B} \parallel [001]$ and strain in generic direction<br>• $\boldsymbol{B} \perp [100]$ and strain $\perp [010]$<br>• $\boldsymbol{B} \perp [100]$ and strain $\perp [001]$<br>• $\boldsymbol{B} \perp [100]$ and strain in generic direction<br>• $\boldsymbol{B} \perp [010]$ and strain $\perp [100]$<br>• $\boldsymbol{B} \perp [010]$ and strain $\perp [001]$<br>• $\boldsymbol{B} \perp [010]$ and strain in generic direction<br>• $\boldsymbol{B} \perp [001]$ and strain $\perp [100]$<br>• $\boldsymbol{B} \perp [001]$ and strain $\perp [010]$<br>• $\boldsymbol{B} \perp [001]$ and strain in generic direction<br>• $\boldsymbol{B}$ in generic direction
$P2'/c'$ (13.69) $\mathbb{Z}_2 \times \mathbb{Z}_2 \times \mathbb{Z}_4$	• $\boldsymbol{B} \parallel [010]$ and strain $\perp [100]$<br>• $\boldsymbol{B} \parallel [001]$ and strain $\perp [100]$<br>• $\boldsymbol{B} \perp [100]$

Continued on next page

...continued

Subgroup & SI group	Perturbation
$P2_1/m$ (11.50) $\mathbb{Z}_2$	• $\boldsymbol{B} \parallel [010]$ and strain $\perp [010]$
$P2_1'/m'$ (11.54) $\mathbb{Z}_2 \times \mathbb{Z}_2 \times \mathbb{Z}_4$	• $\boldsymbol{B} \parallel [100]$ and strain $\perp [001]$<br>• $\boldsymbol{B} \parallel [010]$ and strain $\perp [001]$<br>• $\boldsymbol{B} \perp [001]$
$Pmm'n'$ (59.410) $\mathbb{Z}_2$	• $\boldsymbol{B} \parallel [010]$
Pm (6.18) $\mathbb{Z}_1$	• $\boldsymbol{E} \parallel [010]$ and $\boldsymbol{B} \parallel [100]$ and strain $\perp [100]$<br>• $\boldsymbol{E} \parallel [001]$ and $\boldsymbol{B} \parallel [100]$ and strain $\perp [100]$<br>• $\boldsymbol{E} \perp [100]$ and $\boldsymbol{B} \parallel [100]$
P_Cm (6.23) $\mathbb{Z}_1$	• $\boldsymbol{E} \parallel [010]$ and strain $\perp [100]$<br>• $\boldsymbol{E} \parallel [001]$ and strain $\perp [100]$<br>• $\boldsymbol{E} \perp [100]$
$Pmn'2_1'$ (31.126) $\mathbb{Z}_1$	• $\boldsymbol{E} \parallel [001]$ and $\boldsymbol{B} \parallel [100]$
$P2_1/m$ (11.50) $\mathbb{Z}_2$	• $\boldsymbol{B} \parallel [100]$ and strain $\perp [100]$
$P2'$ (3.3) $\mathbb{Z}_1$	• $\boldsymbol{E} \parallel [010]$ and $\boldsymbol{B} \parallel [100]$ and strain $\perp [010]$<br>• $\boldsymbol{E} \parallel [010]$ and $\boldsymbol{B} \parallel [001]$ and strain $\perp [010]$<br>• $\boldsymbol{E} \parallel [010]$ and $\boldsymbol{B} \perp [010]$
$Pn'n2'$ (34.158) $\mathbb{Z}_1$	• $\boldsymbol{E} \parallel [010]$ and $\boldsymbol{B} \parallel [001]$
P_C2_1 (4.12) $\mathbb{Z}_1$	• $\boldsymbol{E} \parallel [010]$ and strain $\perp [010]$
$P2'/c'$ (13.69) $\mathbb{Z}_2 \times \mathbb{Z}_2 \times \mathbb{Z}_4$	• $\boldsymbol{B} \parallel [100]$ and strain $\perp [010]$<br>• $\boldsymbol{B} \parallel [001]$ and strain $\perp [010]$<br>• $\boldsymbol{B} \perp [010]$
$Pm'm2'$ (25.59) $\mathbb{Z}_1$	• $\boldsymbol{E} \parallel [010]$ and $\boldsymbol{B} \parallel [100]$
P_Imn2_1 (31.134) $\mathbb{Z}_1$	• $\boldsymbol{E} \parallel [010]$
$Pmm'n'$ (59.410) $\mathbb{Z}_2$	• $\boldsymbol{B} \parallel [100]$
$P2$ (3.1) $\mathbb{Z}_2$	• $\boldsymbol{E} \parallel [001]$ and $\boldsymbol{B} \parallel [001]$ and strain $\perp [001]$
$Pn'n'2$ (34.159) $\mathbb{Z}_1$	• $\boldsymbol{E} \parallel [001]$ and $\boldsymbol{B} \parallel [001]$
P_C2 (3.6) $\mathbb{Z}_1$	• $\boldsymbol{E} \parallel [001]$ and strain $\perp [001]$
$P2/c$ (13.65) $\mathbb{Z}_2 \times \mathbb{Z}_2$	• $\boldsymbol{B} \parallel [001]$ and strain $\perp [001]$
P_Imm2 (25.65) $\mathbb{Z}_1$	• $\boldsymbol{E} \parallel [001]$
$Pn'n'n$ (48.260) $\mathbb{Z}_2$	• $\boldsymbol{B} \parallel [001]$
$P_S\bar{1}$ (2.7) $\mathbb{Z}_2 \times \mathbb{Z}_2 \times \mathbb{Z}_4$	• strain in generic direction
P_C2_1/m (11.57) $\mathbb{Z}_2$	• strain $\perp [100]$

Continued on next page

...continued

Subgroup & SI group	Perturbation
$P_C 2_1/m$ (11.57) $\mathbb{Z}_2$	• strain $\perp$ [010]
$P_A 2/c$ (13.73) $\mathbb{Z}_2$	• strain $\perp$ [001]

Table. S 527: Subgroups of $Pbcn$ (60.417) that can be realized by applying $\boldsymbol{E}/\boldsymbol{B}$ fields and/or uniaxial strains.

Subgroup & SI group	Perturbation
Pc (7.24) $\mathbb{Z}_1$	• $\boldsymbol{E}$ $\parallel$ [100] and strain $\perp$ [010]<br>• $\boldsymbol{E}$ $\parallel$ [100] and $\boldsymbol{B}$ $\parallel$ [010]<br>• $\boldsymbol{E}$ $\parallel$ [001] and strain $\perp$ [010]<br>• $\boldsymbol{E}$ $\parallel$ [001] and $\boldsymbol{B}$ $\parallel$ [010]<br>• $\boldsymbol{E}$ $\perp$ [010]
Pc (7.24) $\mathbb{Z}_1$	• $\boldsymbol{E}$ $\parallel$ [100] and strain $\perp$ [001]<br>• $\boldsymbol{E}$ $\parallel$ [100] and $\boldsymbol{B}$ $\parallel$ [001]<br>• $\boldsymbol{E}$ $\parallel$ [010] and strain $\perp$ [001]<br>• $\boldsymbol{E}$ $\parallel$ [010] and $\boldsymbol{B}$ $\parallel$ [001]<br>• $\boldsymbol{E}$ $\perp$ [001]
$P2_1$ (4.7) $\mathbb{Z}_1$	• $\boldsymbol{E}$ $\parallel$ [100] and strain $\perp$ [100]<br>• $\boldsymbol{E}$ $\parallel$ [100] and $\boldsymbol{B}$ $\parallel$ [100]
$Pna2_1$ (33.144) $\mathbb{Z}_1$	• $\boldsymbol{E}$ $\parallel$ [100]
$P2$ (3.1) $\mathbb{Z}_2$	• $\boldsymbol{E}$ $\parallel$ [010] and strain $\perp$ [010]<br>• $\boldsymbol{E}$ $\parallel$ [010] and $\boldsymbol{B}$ $\parallel$ [010]
$P\bar{1}$ (2.4) $\mathbb{Z}_2 \times \mathbb{Z}_2 \times \mathbb{Z}_2 \times \mathbb{Z}_4$	• strain in generic direction<br>• $\boldsymbol{B}$ $\parallel$ [100] and strain $\perp$ [010]<br>• $\boldsymbol{B}$ $\parallel$ [100] and strain $\perp$ [001]<br>• $\boldsymbol{B}$ $\parallel$ [010] and strain $\perp$ [100]<br>• $\boldsymbol{B}$ $\parallel$ [010] and strain $\perp$ [001]<br>• $\boldsymbol{B}$ $\parallel$ [001] and strain $\perp$ [100]<br>• $\boldsymbol{B}$ $\parallel$ [001] and strain $\perp$ [010]<br>• $\boldsymbol{B}$ in generic direction
$P2/c$ (13.65) $\mathbb{Z}_2 \times \mathbb{Z}_2$	• strain $\perp$ [010]<br>• $\boldsymbol{B}$ $\parallel$ [010]

Continued on next page

...continued

Subgroup & SI group	Perturbation
Pc (7.24) $\mathbb{Z}_1$	• $\boldsymbol{E} \parallel [010]$ and strain $\perp [100]$<br>• $\boldsymbol{E} \parallel [010]$ and $\boldsymbol{B} \parallel [100]$<br>• $\boldsymbol{E} \parallel [001]$ and strain $\perp [100]$<br>• $\boldsymbol{E} \parallel [001]$ and $\boldsymbol{B} \parallel [100]$<br>• $\boldsymbol{E} \perp [100]$
$Pnc2$ (30.111) $\mathbb{Z}_1$	• $\boldsymbol{E} \parallel [010]$
$P2_1/c$ (14.75) $\mathbb{Z}_2$	• strain $\perp [100]$<br>• $\boldsymbol{B} \parallel [100]$
$P2_1$ (4.7) $\mathbb{Z}_1$	• $\boldsymbol{E} \parallel [001]$ and strain $\perp [001]$<br>• $\boldsymbol{E} \parallel [001]$ and $\boldsymbol{B} \parallel [001]$
$P2_1/c$ (14.75) $\mathbb{Z}_2$	• strain $\perp [001]$<br>• $\boldsymbol{B} \parallel [001]$
$Pca2_1$ (29.99) $\mathbb{Z}_1$	• $\boldsymbol{E} \parallel [001]$

Table. S 528: Subgroups of $Pbcn1'$ (60.418) that can be realized by applying $\boldsymbol{E}/\boldsymbol{B}$ fields and/or uniaxial strains.

Subgroup & SI group	Perturbation
$P11'$ (1.2) $\mathbb{Z}_1$	• $\boldsymbol{E} \parallel [100]$ and strain in generic direction<br>• $\boldsymbol{E} \parallel [010]$ and strain in generic direction<br>• $\boldsymbol{E} \parallel [001]$ and strain in generic direction<br>• $\boldsymbol{E} \perp [100]$ and strain $\perp [010]$<br>• $\boldsymbol{E} \perp [100]$ and strain $\perp [001]$<br>• $\boldsymbol{E} \perp [100]$ and strain in generic direction<br>• $\boldsymbol{E} \perp [010]$ and strain $\perp [100]$<br>• $\boldsymbol{E} \perp [010]$ and strain $\perp [001]$<br>• $\boldsymbol{E} \perp [010]$ and strain in generic direction<br>• $\boldsymbol{E} \perp [001]$ and strain $\perp [100]$<br>• $\boldsymbol{E} \perp [001]$ and strain $\perp [010]$<br>• $\boldsymbol{E} \perp [001]$ and strain in generic direction<br>• $\boldsymbol{E}$ in generic direction

Continued on next page

. . . continued

Subgroup & SI group	Perturbation
Pc' (7.26) $\mathbb{Z}_1$	• $\boldsymbol{E}$ ∥ [100] and $\boldsymbol{B}$ ∥ [100] and strain ⊥ [010]<br>• $\boldsymbol{E}$ ∥ [100] and $\boldsymbol{B}$ ∥ [001] and strain ⊥ [010]<br>• $\boldsymbol{E}$ ∥ [100] and $\boldsymbol{B}$ ⊥ [010]<br>• $\boldsymbol{E}$ ∥ [001] and $\boldsymbol{B}$ ∥ [100] and strain ⊥ [010]<br>• $\boldsymbol{E}$ ∥ [001] and $\boldsymbol{B}$ ∥ [001] and strain ⊥ [010]<br>• $\boldsymbol{E}$ ∥ [001] and $\boldsymbol{B}$ ⊥ [010]<br>• $\boldsymbol{E}$ ⊥ [010] and $\boldsymbol{B}$ ∥ [100]<br>• $\boldsymbol{E}$ ⊥ [010] and $\boldsymbol{B}$ ∥ [001]<br>• $\boldsymbol{E}$ ⊥ [010] and $\boldsymbol{B}$ ⊥ [010]
Pc (7.24) $\mathbb{Z}_1$	• $\boldsymbol{E}$ ∥ [100] and $\boldsymbol{B}$ ∥ [010] and strain ⊥ [010]<br>• $\boldsymbol{E}$ ∥ [001] and $\boldsymbol{B}$ ∥ [010] and strain ⊥ [010]<br>• $\boldsymbol{E}$ ⊥ [010] and $\boldsymbol{B}$ ∥ [010]
$Pc1'$ (7.25) $\mathbb{Z}_1$	• $\boldsymbol{E}$ ∥ [100] and strain ⊥ [010]<br>• $\boldsymbol{E}$ ∥ [001] and strain ⊥ [010]<br>• $\boldsymbol{E}$ ⊥ [010]
Pc' (7.26) $\mathbb{Z}_1$	• $\boldsymbol{E}$ ∥ [100] and $\boldsymbol{B}$ ∥ [100] and strain ⊥ [001]<br>• $\boldsymbol{E}$ ∥ [100] and $\boldsymbol{B}$ ∥ [010] and strain ⊥ [001]<br>• $\boldsymbol{E}$ ∥ [100] and $\boldsymbol{B}$ ⊥ [001]<br>• $\boldsymbol{E}$ ∥ [010] and $\boldsymbol{B}$ ∥ [100] and strain ⊥ [001]<br>• $\boldsymbol{E}$ ∥ [010] and $\boldsymbol{B}$ ∥ [010] and strain ⊥ [001]<br>• $\boldsymbol{E}$ ∥ [010] and $\boldsymbol{B}$ ⊥ [001]<br>• $\boldsymbol{E}$ ⊥ [001] and $\boldsymbol{B}$ ∥ [100]<br>• $\boldsymbol{E}$ ⊥ [001] and $\boldsymbol{B}$ ∥ [010]<br>• $\boldsymbol{E}$ ⊥ [001] and $\boldsymbol{B}$ ⊥ [001]
Pc (7.24) $\mathbb{Z}_1$	• $\boldsymbol{E}$ ∥ [100] and $\boldsymbol{B}$ ∥ [001] and strain ⊥ [001]<br>• $\boldsymbol{E}$ ∥ [010] and $\boldsymbol{B}$ ∥ [001] and strain ⊥ [001]<br>• $\boldsymbol{E}$ ⊥ [001] and $\boldsymbol{B}$ ∥ [001]
$Pc1'$ (7.25) $\mathbb{Z}_1$	• $\boldsymbol{E}$ ∥ [100] and strain ⊥ [001]<br>• $\boldsymbol{E}$ ∥ [010] and strain ⊥ [001]<br>• $\boldsymbol{E}$ ⊥ [001]
$P2_1'$ (4.9) $\mathbb{Z}_1$	• $\boldsymbol{E}$ ∥ [100] and $\boldsymbol{B}$ ∥ [010] and strain ⊥ [100]<br>• $\boldsymbol{E}$ ∥ [100] and $\boldsymbol{B}$ ∥ [001] and strain ⊥ [100]<br>• $\boldsymbol{E}$ ∥ [100] and $\boldsymbol{B}$ ⊥ [100]
$Pn'a2_1'$ (33.146) $\mathbb{Z}_1$	• $\boldsymbol{E}$ ∥ [100] and $\boldsymbol{B}$ ∥ [010]
$Pna'2_1'$ (33.147) $\mathbb{Z}_1$	• $\boldsymbol{E}$ ∥ [100] and $\boldsymbol{B}$ ∥ [001]
$P2_1$ (4.7) $\mathbb{Z}_1$	• $\boldsymbol{E}$ ∥ [100] and $\boldsymbol{B}$ ∥ [100] and strain ⊥ [100]

Continued on next page

...continued

Subgroup & SI group	Perturbation
$Pn'a'2_1$ (33.148) $\mathbb{Z}_1$	• $\boldsymbol{E} \parallel [100]$ and $\boldsymbol{B} \parallel [100]$
$P2_11'$ (4.8) $\mathbb{Z}_1$	• $\boldsymbol{E} \parallel [100]$ and strain $\perp [100]$
$Pna2_11'$ (33.145) $\mathbb{Z}_1$	• $\boldsymbol{E} \parallel [100]$
$P2'$ (3.3) $\mathbb{Z}_1$	• $\boldsymbol{E} \parallel [010]$ and $\boldsymbol{B} \parallel [100]$ and strain $\perp [010]$<br>• $\boldsymbol{E} \parallel [010]$ and $\boldsymbol{B} \parallel [001]$ and strain $\perp [010]$<br>• $\boldsymbol{E} \parallel [010]$ and $\boldsymbol{B} \perp [010]$
$P2$ (3.1) $\mathbb{Z}_2$	• $\boldsymbol{E} \parallel [010]$ and $\boldsymbol{B} \parallel [010]$ and strain $\perp [010]$
$P21'$ (3.2) $\mathbb{Z}_2$	• $\boldsymbol{E} \parallel [010]$ and strain $\perp [010]$
$P\bar{1}$ (2.4) $\mathbb{Z}_2 \times \mathbb{Z}_2 \times \mathbb{Z}_2 \times \mathbb{Z}_4$	• $\boldsymbol{B} \parallel [100]$ and strain in generic direction<br>• $\boldsymbol{B} \parallel [010]$ and strain in generic direction<br>• $\boldsymbol{B} \parallel [001]$ and strain in generic direction<br>• $\boldsymbol{B} \perp [100]$ and strain $\perp [010]$<br>• $\boldsymbol{B} \perp [100]$ and strain $\perp [001]$<br>• $\boldsymbol{B} \perp [100]$ and strain in generic direction<br>• $\boldsymbol{B} \perp [010]$ and strain $\perp [100]$<br>• $\boldsymbol{B} \perp [010]$ and strain $\perp [001]$<br>• $\boldsymbol{B} \perp [010]$ and strain in generic direction<br>• $\boldsymbol{B} \perp [001]$ and strain $\perp [100]$<br>• $\boldsymbol{B} \perp [001]$ and strain $\perp [010]$<br>• $\boldsymbol{B} \perp [001]$ and strain in generic direction<br>• $\boldsymbol{B}$ in generic direction
$P2'/c'$ (13.69) $\mathbb{Z}_2 \times \mathbb{Z}_2 \times \mathbb{Z}_4$	• $\boldsymbol{B} \parallel [100]$ and strain $\perp [010]$<br>• $\boldsymbol{B} \parallel [001]$ and strain $\perp [010]$<br>• $\boldsymbol{B} \perp [010]$
$P2/c$ (13.65) $\mathbb{Z}_2 \times \mathbb{Z}_2$	• $\boldsymbol{B} \parallel [010]$ and strain $\perp [010]$
$P\bar{1}1'$ (2.5) $\mathbb{Z}_2 \times \mathbb{Z}_2 \times \mathbb{Z}_2 \times \mathbb{Z}_4$	• strain in generic direction
$P2/c1'$ (13.66) $\mathbb{Z}_2 \times \mathbb{Z}_2$	• strain $\perp [010]$
Pc' (7.26) $\mathbb{Z}_1$	• $\boldsymbol{E} \parallel [010]$ and $\boldsymbol{B} \parallel [010]$ and strain $\perp [100]$<br>• $\boldsymbol{E} \parallel [010]$ and $\boldsymbol{B} \parallel [001]$ and strain $\perp [100]$<br>• $\boldsymbol{E} \parallel [010]$ and $\boldsymbol{B} \perp [100]$<br>• $\boldsymbol{E} \parallel [001]$ and $\boldsymbol{B} \parallel [010]$ and strain $\perp [100]$<br>• $\boldsymbol{E} \parallel [001]$ and $\boldsymbol{B} \parallel [001]$ and strain $\perp [100]$<br>• $\boldsymbol{E} \parallel [001]$ and $\boldsymbol{B} \perp [100]$<br>• $\boldsymbol{E} \perp [100]$ and $\boldsymbol{B} \parallel [010]$<br>• $\boldsymbol{E} \perp [100]$ and $\boldsymbol{B} \parallel [001]$<br>• $\boldsymbol{E} \perp [100]$ and $\boldsymbol{B} \perp [100]$

Continued on next page

. . . continued

Subgroup & SI group	Perturbation
$Pnc'2'$ (30.114) $\mathbb{Z}_1$	• $\boldsymbol{E}$ || [010] and $\boldsymbol{B}$ || [001]
$Pn'c'2$ (30.115) $\mathbb{Z}_1$	• $\boldsymbol{E}$ || [010] and $\boldsymbol{B}$ || [010]
$P2_1'/c'$ (14.79) $\mathbb{Z}_2 \times \mathbb{Z}_4$	• $\boldsymbol{B}$ || [010] and strain $\perp$ [100]<br>• $\boldsymbol{B}$ || [001] and strain $\perp$ [100]<br>• $\boldsymbol{B} \perp$ [100]
Pc (7.24) $\mathbb{Z}_1$	• $\boldsymbol{E}$ || [010] and $\boldsymbol{B}$ || [100] and strain $\perp$ [100]<br>• $\boldsymbol{E}$ || [001] and $\boldsymbol{B}$ || [100] and strain $\perp$ [100]<br>• $\boldsymbol{E} \perp$ [100] and $\boldsymbol{B}$ || [100]
$Pn'c2'$ (30.113) $\mathbb{Z}_1$	• $\boldsymbol{E}$ || [010] and $\boldsymbol{B}$ || [100]
$P2_1/c$ (14.75) $\mathbb{Z}_2$	• $\boldsymbol{B}$ || [100] and strain $\perp$ [100]
$Pc1'$ (7.25) $\mathbb{Z}_1$	• $\boldsymbol{E}$ || [010] and strain $\perp$ [100]<br>• $\boldsymbol{E}$ || [001] and strain $\perp$ [100]<br>• $\boldsymbol{E} \perp$ [100]
$Pnc21'$ (30.112) $\mathbb{Z}_1$	• $\boldsymbol{E}$ || [010]
$P2_1/c1'$ (14.76) $\mathbb{Z}_2$	• strain $\perp$ [100]
$P2_1'$ (4.9) $\mathbb{Z}_1$	• $\boldsymbol{E}$ || [001] and $\boldsymbol{B}$ || [100] and strain $\perp$ [001]<br>• $\boldsymbol{E}$ || [001] and $\boldsymbol{B}$ || [010] and strain $\perp$ [001]<br>• $\boldsymbol{E}$ || [001] and $\boldsymbol{B} \perp$ [001]
$P2_1'/c'$ (14.79) $\mathbb{Z}_2 \times \mathbb{Z}_4$	• $\boldsymbol{B}$ || [100] and strain $\perp$ [001]<br>• $\boldsymbol{B}$ || [010] and strain $\perp$ [001]<br>• $\boldsymbol{B} \perp$ [001]
$Pca'2_1'$ (29.102) $\mathbb{Z}_1$	• $\boldsymbol{E}$ || [001] and $\boldsymbol{B}$ || [010]
$Pb'cn'$ (60.424) $\mathbb{Z}_2 \times \mathbb{Z}_2$	• $\boldsymbol{B}$ || [010]
$Pc'a2_1'$ (29.101) $\mathbb{Z}_1$	• $\boldsymbol{E}$ || [001] and $\boldsymbol{B}$ || [100]
$Pbc'n'$ (60.423) $\mathbb{Z}_2$	• $\boldsymbol{B}$ || [100]
$P2_1$ (4.7) $\mathbb{Z}_1$	• $\boldsymbol{E}$ || [001] and $\boldsymbol{B}$ || [001] and strain $\perp$ [001]
$P2_1/c$ (14.75) $\mathbb{Z}_2$	• $\boldsymbol{B}$ || [001] and strain $\perp$ [001]
$Pc'a'2_1$ (29.103) $\mathbb{Z}_1$	• $\boldsymbol{E}$ || [001] and $\boldsymbol{B}$ || [001]
$Pb'c'n$ (60.422) $\mathbb{Z}_2$	• $\boldsymbol{B}$ || [001]
$P2_11'$ (4.8) $\mathbb{Z}_1$	• $\boldsymbol{E}$ || [001] and strain $\perp$ [001]
$P2_1/c1'$ (14.76) $\mathbb{Z}_2$	• strain $\perp$ [001]
$Pca2_11'$ (29.100) $\mathbb{Z}_1$	• $\boldsymbol{E}$ || [001]

Table. S 529: Subgroups of $Pb'cn$ (60.419) that can be realized by applying $\boldsymbol{E}/\boldsymbol{B}$ fields and/or uniaxial strains.

Subgroup & SI group	Perturbation
Pc (7.24) $\mathbb{Z}_1$	• $\boldsymbol{B}$ ∥ [010] and strain ⊥ [010]<br>• $\boldsymbol{E}$ ∥ [100] and strain ⊥ [010]<br>• $\boldsymbol{E}$ ∥ [100] and $\boldsymbol{B}$ ∥ [010]<br>• $\boldsymbol{E}$ ∥ [001] and strain ⊥ [010]<br>• $\boldsymbol{E}$ ⊥ [010]
Pc (7.24) $\mathbb{Z}_1$	• $\boldsymbol{B}$ ∥ [001] and strain ⊥ [001]<br>• $\boldsymbol{E}$ ∥ [100] and strain ⊥ [001]<br>• $\boldsymbol{E}$ ∥ [100] and $\boldsymbol{B}$ ∥ [001]<br>• $\boldsymbol{E}$ ∥ [010] and strain ⊥ [001]<br>• $\boldsymbol{E}$ ⊥ [001]
$P2_1$ (4.7) $\mathbb{Z}_1$	• $\boldsymbol{B}$ ∥ [100] and strain ⊥ [100]<br>• $\boldsymbol{E}$ ∥ [100] and strain ⊥ [100]<br>• $\boldsymbol{E}$ ∥ [100] and $\boldsymbol{B}$ ∥ [100]
$Pna2_1$ (33.144) $\mathbb{Z}_1$	• $\boldsymbol{E}$ ∥ [100]
$P2'$ (3.3) $\mathbb{Z}_1$	• $\boldsymbol{B}$ ∥ [100] and strain ⊥ [010]<br>• $\boldsymbol{B}$ ∥ [001] and strain ⊥ [010]<br>• $\boldsymbol{B}$ ⊥ [010]<br>• $\boldsymbol{E}$ ∥ [010] and strain ⊥ [010]<br>• $\boldsymbol{E}$ ∥ [010] and $\boldsymbol{B}$ ∥ [100]
$P\bar{1}'$ (2.6) $\mathbb{Z}_1$	• strain in generic direction
$P2'/c$ (13.67) $\mathbb{Z}_1$	• strain ⊥ [010]
Pc' (7.26) $\mathbb{Z}_1$	• $\boldsymbol{B}$ ∥ [010] and strain ⊥ [100]<br>• $\boldsymbol{B}$ ∥ [001] and strain ⊥ [100]<br>• $\boldsymbol{B}$ ⊥ [100]<br>• $\boldsymbol{E}$ ∥ [010] and strain ⊥ [100]<br>• $\boldsymbol{E}$ ∥ [010] and $\boldsymbol{B}$ ∥ [010]<br>• $\boldsymbol{E}$ ∥ [001] and strain ⊥ [100]<br>• $\boldsymbol{E}$ ∥ [001] and $\boldsymbol{B}$ ∥ [001]<br>• $\boldsymbol{E}$ ⊥ [100]
$Pnc'2'$ (30.114) $\mathbb{Z}_1$	• $\boldsymbol{B}$ ∥ [001]<br>• $\boldsymbol{E}$ ∥ [010]
$P2_1/c'$ (14.78) $\mathbb{Z}_1$	• strain ⊥ [100]

Continued on next page

...continued

Subgroup & SI group	Perturbation
$P2_1'$ (4.9) $\mathbb{Z}_1$	• $\boldsymbol{B} \parallel [100]$ and strain $\perp [001]$<br>• $\boldsymbol{B} \parallel [010]$ and strain $\perp [001]$<br>• $\boldsymbol{B} \perp [001]$<br>• $\boldsymbol{E} \parallel [001]$ and strain $\perp [001]$<br>• $\boldsymbol{E} \parallel [001]$ and $\boldsymbol{B} \parallel [100]$
$P2_12_1'2'$ (18.19) $\mathbb{Z}_1$	• $\boldsymbol{B} \parallel [100]$
$P2_1'/c$ (14.77) $\mathbb{Z}_1$	• strain $\perp [001]$
$Pca'2_1'$ (29.102) $\mathbb{Z}_1$	• $\boldsymbol{B} \parallel [010]$<br>• $\boldsymbol{E} \parallel [001]$

Table. S 530: Subgroups of $Pbc'n$ (60.420) that can be realized by applying $\boldsymbol{E}/\boldsymbol{B}$ fields and/or uniaxial strains.

Subgroup & SI group	Perturbation
Pc' (7.26) $\mathbb{Z}_1$	• $\boldsymbol{B} \parallel [100]$ and strain $\perp [010]$<br>• $\boldsymbol{B} \parallel [001]$ and strain $\perp [010]$<br>• $\boldsymbol{B} \perp [010]$<br>• $\boldsymbol{E} \parallel [100]$ and strain $\perp [010]$<br>• $\boldsymbol{E} \parallel [100]$ and $\boldsymbol{B} \parallel [100]$<br>• $\boldsymbol{E} \parallel [001]$ and strain $\perp [010]$<br>• $\boldsymbol{E} \parallel [001]$ and $\boldsymbol{B} \parallel [001]$<br>• $\boldsymbol{E} \perp [010]$
Pc (7.24) $\mathbb{Z}_1$	• $\boldsymbol{B} \parallel [001]$ and strain $\perp [001]$<br>• $\boldsymbol{E} \parallel [100]$ and strain $\perp [001]$<br>• $\boldsymbol{E} \parallel [010]$ and strain $\perp [001]$<br>• $\boldsymbol{E} \parallel [010]$ and $\boldsymbol{B} \parallel [001]$<br>• $\boldsymbol{E} \perp [001]$
$P2_1'$ (4.9) $\mathbb{Z}_1$	• $\boldsymbol{B} \parallel [010]$ and strain $\perp [100]$<br>• $\boldsymbol{B} \parallel [001]$ and strain $\perp [100]$<br>• $\boldsymbol{B} \perp [100]$<br>• $\boldsymbol{E} \parallel [100]$ and strain $\perp [100]$<br>• $\boldsymbol{E} \parallel [100]$ and $\boldsymbol{B} \parallel [010]$
$Pna'2_1'$ (33.147) $\mathbb{Z}_1$	• $\boldsymbol{B} \parallel [001]$<br>• $\boldsymbol{E} \parallel [100]$

Continued on next page

. . . continued

Subgroup & SI group	Perturbation
$P2$ (3.1) $\mathbb{Z}_2$	• $\boldsymbol{B} \parallel [010]$ and strain $\perp [010]$<br>• $\boldsymbol{E} \parallel [010]$ and strain $\perp [010]$<br>• $\boldsymbol{E} \parallel [010]$ and $\boldsymbol{B} \parallel [010]$
$P\bar{1}'$ (2.6) $\mathbb{Z}_1$	• strain in generic direction
$P2/c'$ (13.68) $\mathbb{Z}_2$	• strain $\perp [010]$
Pc (7.24) $\mathbb{Z}_1$	• $\boldsymbol{B} \parallel [100]$ and strain $\perp [100]$<br>• $\boldsymbol{E} \parallel [010]$ and strain $\perp [100]$<br>• $\boldsymbol{E} \parallel [010]$ and $\boldsymbol{B} \parallel [100]$<br>• $\boldsymbol{E} \parallel [001]$ and strain $\perp [100]$<br>• $\boldsymbol{E} \perp [100]$
$Pnc2$ (30.111) $\mathbb{Z}_1$	• $\boldsymbol{E} \parallel [010]$
$P2_1'/c$ (14.77) $\mathbb{Z}_1$	• strain $\perp [100]$
$P2_1'$ (4.9) $\mathbb{Z}_1$	• $\boldsymbol{B} \parallel [100]$ and strain $\perp [001]$<br>• $\boldsymbol{B} \parallel [010]$ and strain $\perp [001]$<br>• $\boldsymbol{B} \perp [001]$<br>• $\boldsymbol{E} \parallel [001]$ and strain $\perp [001]$<br>• $\boldsymbol{E} \parallel [001]$ and $\boldsymbol{B} \parallel [010]$
$P2_1'2_1'2$ (18.18) $\mathbb{Z}_2$	• $\boldsymbol{B} \parallel [010]$
$P2_1'/c$ (14.77) $\mathbb{Z}_1$	• strain $\perp [001]$
$Pc'a2_1'$ (29.101) $\mathbb{Z}_1$	• $\boldsymbol{B} \parallel [100]$<br>• $\boldsymbol{E} \parallel [001]$

Table. S 531: Subgroups of $Pbcn'$ (60.421) that can be realized by applying $\boldsymbol{E}/\boldsymbol{B}$ fields and/or uniaxial strains.

Subgroup & SI group	Perturbation
Pc (7.24) $\mathbb{Z}_1$	• $\boldsymbol{B} \parallel [010]$ and strain $\perp [010]$<br>• $\boldsymbol{E} \parallel [100]$ and strain $\perp [010]$<br>• $\boldsymbol{E} \parallel [001]$ and strain $\perp [010]$<br>• $\boldsymbol{E} \parallel [001]$ and $\boldsymbol{B} \parallel [010]$<br>• $\boldsymbol{E} \perp [010]$

Continued on next page

...continued

Subgroup & SI group	Perturbation
Pc' (7.26) $\mathbb{Z}_1$	• $\boldsymbol{B}$ ∥ [100] and strain ⊥ [001]<br>• $\boldsymbol{B}$ ∥ [010] and strain ⊥ [001]<br>• $\boldsymbol{B}$ ⊥ [001]<br>• $\boldsymbol{E}$ ∥ [100] and strain ⊥ [001]<br>• $\boldsymbol{E}$ ∥ [100] and $\boldsymbol{B}$ ∥ [100]<br>• $\boldsymbol{E}$ ∥ [010] and strain ⊥ [001]<br>• $\boldsymbol{E}$ ∥ [010] and $\boldsymbol{B}$ ∥ [010]<br>• $\boldsymbol{E}$ ⊥ [001]
$P2_1'$ (4.9) $\mathbb{Z}_1$	• $\boldsymbol{B}$ ∥ [010] and strain ⊥ [100]<br>• $\boldsymbol{B}$ ∥ [001] and strain ⊥ [100]<br>• $\boldsymbol{B}$ ⊥ [100]<br>• $\boldsymbol{E}$ ∥ [100] and strain ⊥ [100]<br>• $\boldsymbol{E}$ ∥ [100] and $\boldsymbol{B}$ ∥ [001]
$Pn'a2_1'$ (33.146) $\mathbb{Z}_1$	• $\boldsymbol{B}$ ∥ [010]<br>• $\boldsymbol{E}$ ∥ [100]
$P2'$ (3.3) $\mathbb{Z}_1$	• $\boldsymbol{B}$ ∥ [100] and strain ⊥ [010]<br>• $\boldsymbol{B}$ ∥ [001] and strain ⊥ [010]<br>• $\boldsymbol{B}$ ⊥ [010]<br>• $\boldsymbol{E}$ ∥ [010] and strain ⊥ [010]<br>• $\boldsymbol{E}$ ∥ [010] and $\boldsymbol{B}$ ∥ [001]
$P\bar{1}'$ (2.6) $\mathbb{Z}_1$	• strain in generic direction
$P2'/c$ (13.67) $\mathbb{Z}_1$	• strain ⊥ [010]
Pc (7.24) $\mathbb{Z}_1$	• $\boldsymbol{B}$ ∥ [100] and strain ⊥ [100]<br>• $\boldsymbol{E}$ ∥ [010] and strain ⊥ [100]<br>• $\boldsymbol{E}$ ∥ [001] and strain ⊥ [100]<br>• $\boldsymbol{E}$ ∥ [001] and $\boldsymbol{B}$ ∥ [100]<br>• $\boldsymbol{E}$ ⊥ [100]
$Pn'c2'$ (30.113) $\mathbb{Z}_1$	• $\boldsymbol{B}$ ∥ [100]<br>• $\boldsymbol{E}$ ∥ [010]
$P2_1'/c$ (14.77) $\mathbb{Z}_1$	• strain ⊥ [100]
$P2_1$ (4.7) $\mathbb{Z}_1$	• $\boldsymbol{B}$ ∥ [001] and strain ⊥ [001]<br>• $\boldsymbol{E}$ ∥ [001] and strain ⊥ [001]<br>• $\boldsymbol{E}$ ∥ [001] and $\boldsymbol{B}$ ∥ [001]
$P2_12_1'2'$ (18.19) $\mathbb{Z}_1$	• $\boldsymbol{B}$ ∥ [001]
$P2_1/c'$ (14.78) $\mathbb{Z}_1$	• strain ⊥ [001]
$Pca2_1$ (29.99) $\mathbb{Z}_1$	• $\boldsymbol{E}$ ∥ [001]

Table. S 532: Subgroups of $Pb'c'n$ (60.422) that can be realized by applying $\boldsymbol{E}/\boldsymbol{B}$ fields and/or uniaxial strains.

Subgroup & SI group	Perturbation
Pc' (7.26) $\mathbb{Z}_1$	• $\boldsymbol{E} \parallel [100]$ and strain $\perp [010]$<br>• $\boldsymbol{E} \parallel [100]$ and ($\boldsymbol{B} \parallel [100]$ or $\boldsymbol{B} \perp [010]$)<br>• $\boldsymbol{E} \parallel [001]$ and strain $\perp [010]$<br>• $\boldsymbol{E} \parallel [001]$ and ($\boldsymbol{B} \parallel [100]$ or $\boldsymbol{B} \perp [010]$)<br>• $\boldsymbol{E} \perp [010]$
Pc (7.24) $\mathbb{Z}_1$	• $\boldsymbol{E} \parallel [100]$ and strain $\perp [001]$<br>• $\boldsymbol{E} \parallel [010]$ and strain $\perp [001]$<br>• $\boldsymbol{E} \perp [001]$
$P2_1'$ (4.9) $\mathbb{Z}_1$	• $\boldsymbol{E} \parallel [100]$ and strain $\perp [100]$<br>• $\boldsymbol{E} \parallel [100]$ and ($\boldsymbol{B} \parallel [010]$ or $\boldsymbol{B} \perp [100]$)
$Pna'2_1'$ (33.147) $\mathbb{Z}_1$	• $\boldsymbol{E} \parallel [100]$
$P2'$ (3.3) $\mathbb{Z}_1$	• $\boldsymbol{E} \parallel [010]$ and strain $\perp [010]$<br>• $\boldsymbol{E} \parallel [010]$ and ($\boldsymbol{B} \parallel [100]$ or $\boldsymbol{B} \perp [010]$)
$P\bar{1}$ (2.4) $\mathbb{Z}_2 \times \mathbb{Z}_2 \times \mathbb{Z}_2 \times \mathbb{Z}_4$	• strain in generic direction<br>• ($\boldsymbol{B} \parallel [100]$ or $\boldsymbol{B} \perp [010]$) and strain $\perp [100]$<br>• ($\boldsymbol{B} \parallel [100]$ or $\boldsymbol{B} \perp [010]$) and strain $\perp [001]$<br>• ($\boldsymbol{B} \parallel [010]$ or $\boldsymbol{B} \perp [100]$) and strain $\perp [010]$<br>• ($\boldsymbol{B} \parallel [010]$ or $\boldsymbol{B} \perp [100]$) and strain $\perp [001]$<br>• $\boldsymbol{B}$ in generic direction
$P2'/c'$ (13.69) $\mathbb{Z}_2 \times \mathbb{Z}_2 \times \mathbb{Z}_4$	• strain $\perp [010]$<br>• ($\boldsymbol{B} \parallel [100]$ or $\boldsymbol{B} \perp [010]$)
Pc' (7.26) $\mathbb{Z}_1$	• $\boldsymbol{E} \parallel [010]$ and strain $\perp [100]$<br>• $\boldsymbol{E} \parallel [010]$ and ($\boldsymbol{B} \parallel [010]$ or $\boldsymbol{B} \perp [100]$)<br>• $\boldsymbol{E} \parallel [001]$ and strain $\perp [100]$<br>• $\boldsymbol{E} \parallel [001]$ and ($\boldsymbol{B} \parallel [010]$ or $\boldsymbol{B} \perp [100]$)<br>• $\boldsymbol{E} \perp [100]$
$Pnc'2'$ (30.114) $\mathbb{Z}_1$	• $\boldsymbol{E} \parallel [010]$
$P2_1'/c'$ (14.79) $\mathbb{Z}_2 \times \mathbb{Z}_4$	• strain $\perp [100]$<br>• ($\boldsymbol{B} \parallel [010]$ or $\boldsymbol{B} \perp [100]$)
$P2_1$ (4.7) $\mathbb{Z}_1$	• $\boldsymbol{E} \parallel [001]$ and strain $\perp [001]$
$P2_1/c$ (14.75) $\mathbb{Z}_2$	• strain $\perp [001]$
$Pc'a'2_1$ (29.103) $\mathbb{Z}_1$	• $\boldsymbol{E} \parallel [001]$

Table. S 533: Subgroups of $Pbc'n'$ (60.423) that can be realized by applying $\boldsymbol{E}/\boldsymbol{B}$ fields and/or uniaxial strains.

Subgroup & SI group	Perturbation
Pc' (7.26) $\mathbb{Z}_1$	• $\boldsymbol{E}$ ∥ [100] and strain ⊥ [010]<br>• $\boldsymbol{E}$ ∥ [100] and ($\boldsymbol{B}$ ∥ [001] or $\boldsymbol{B}$ ⊥ [010])<br>• $\boldsymbol{E}$ ∥ [001] and strain ⊥ [010]<br>• $\boldsymbol{E}$ ∥ [001] and ($\boldsymbol{B}$ ∥ [001] or $\boldsymbol{B}$ ⊥ [010])<br>• $\boldsymbol{E}$ ⊥ [010]
Pc' (7.26) $\mathbb{Z}_1$	• $\boldsymbol{E}$ ∥ [100] and strain ⊥ [001]<br>• $\boldsymbol{E}$ ∥ [100] and ($\boldsymbol{B}$ ∥ [010] or $\boldsymbol{B}$ ⊥ [001])<br>• $\boldsymbol{E}$ ∥ [010] and strain ⊥ [001]<br>• $\boldsymbol{E}$ ∥ [010] and ($\boldsymbol{B}$ ∥ [010] or $\boldsymbol{B}$ ⊥ [001])<br>• $\boldsymbol{E}$ ⊥ [001]
$P2_1$ (4.7) $\mathbb{Z}_1$	• $\boldsymbol{E}$ ∥ [100] and strain ⊥ [100]
$Pn'a'2_1$ (33.148) $\mathbb{Z}_1$	• $\boldsymbol{E}$ ∥ [100]
$P2'$ (3.3) $\mathbb{Z}_1$	• $\boldsymbol{E}$ ∥ [010] and strain ⊥ [010]<br>• $\boldsymbol{E}$ ∥ [010] and ($\boldsymbol{B}$ ∥ [001] or $\boldsymbol{B}$ ⊥ [010])
$P\bar{1}$ (2.4) $\mathbb{Z}_2 \times \mathbb{Z}_2 \times \mathbb{Z}_2 \times \mathbb{Z}_4$	• strain in generic direction<br>• ($\boldsymbol{B}$ ∥ [010] or $\boldsymbol{B}$ ⊥ [001]) and strain ⊥ [100]<br>• ($\boldsymbol{B}$ ∥ [010] or $\boldsymbol{B}$ ⊥ [001]) and strain ⊥ [010]<br>• ($\boldsymbol{B}$ ∥ [001] or $\boldsymbol{B}$ ⊥ [010]) and strain ⊥ [100]<br>• ($\boldsymbol{B}$ ∥ [001] or $\boldsymbol{B}$ ⊥ [010]) and strain ⊥ [001]<br>• $\boldsymbol{B}$ in generic direction
$P2'/c'$ (13.69) $\mathbb{Z}_2 \times \mathbb{Z}_2 \times \mathbb{Z}_4$	• strain ⊥ [010]<br>• ($\boldsymbol{B}$ ∥ [001] or $\boldsymbol{B}$ ⊥ [010])
Pc (7.24) $\mathbb{Z}_1$	• $\boldsymbol{E}$ ∥ [010] and strain ⊥ [100]<br>• $\boldsymbol{E}$ ∥ [001] and strain ⊥ [100]<br>• $\boldsymbol{E}$ ⊥ [100]
$Pn'c2'$ (30.113) $\mathbb{Z}_1$	• $\boldsymbol{E}$ ∥ [010]
$P2_1/c$ (14.75) $\mathbb{Z}_2$	• strain ⊥ [100]
$P2_1'$ (4.9) $\mathbb{Z}_1$	• $\boldsymbol{E}$ ∥ [001] and strain ⊥ [001]<br>• $\boldsymbol{E}$ ∥ [001] and ($\boldsymbol{B}$ ∥ [010] or $\boldsymbol{B}$ ⊥ [001])
$P2_1'/c'$ (14.79) $\mathbb{Z}_2 \times \mathbb{Z}_4$	• strain ⊥ [001]<br>• ($\boldsymbol{B}$ ∥ [010] or $\boldsymbol{B}$ ⊥ [001])
$Pc'a2_1'$ (29.101) $\mathbb{Z}_1$	• $\boldsymbol{E}$ ∥ [001]

Table. S 534: Subgroups of $Pb'cn'$ (60.424) that can be realized by applying $\boldsymbol{E}/\boldsymbol{B}$ fields and/or uniaxial strains.

Subgroup & SI group	Perturbation
Pc (7.24) $\mathbb{Z}_1$	• $\boldsymbol{E}$ ∥ [100] and strain ⊥ [010]<br>• $\boldsymbol{E}$ ∥ [001] and strain ⊥ [010]<br>• $\boldsymbol{E}$ ⊥ [010]
Pc' (7.26) $\mathbb{Z}_1$	• $\boldsymbol{E}$ ∥ [100] and strain ⊥ [001]<br>• $\boldsymbol{E}$ ∥ [100] and ($\boldsymbol{B}$ ∥ [100] or $\boldsymbol{B}$ ⊥ [001])<br>• $\boldsymbol{E}$ ∥ [010] and strain ⊥ [001]<br>• $\boldsymbol{E}$ ∥ [010] and ($\boldsymbol{B}$ ∥ [100] or $\boldsymbol{B}$ ⊥ [001])<br>• $\boldsymbol{E}$ ⊥ [001]
$P2_1'$ (4.9) $\mathbb{Z}_1$	• $\boldsymbol{E}$ ∥ [100] and strain ⊥ [100]<br>• $\boldsymbol{E}$ ∥ [100] and ($\boldsymbol{B}$ ∥ [001] or $\boldsymbol{B}$ ⊥ [100])
$Pn'a2_1'$ (33.146) $\mathbb{Z}_1$	• $\boldsymbol{E}$ ∥ [100]
$P2$ (3.1) $\mathbb{Z}_2$	• $\boldsymbol{E}$ ∥ [010] and strain ⊥ [010]
$P\bar{1}$ (2.4) $\mathbb{Z}_2 \times \mathbb{Z}_2 \times \mathbb{Z}_2 \times \mathbb{Z}_4$	• strain in generic direction<br>• ($\boldsymbol{B}$ ∥ [100] or $\boldsymbol{B}$ ⊥ [001]) and strain ⊥ [100]<br>• ($\boldsymbol{B}$ ∥ [100] or $\boldsymbol{B}$ ⊥ [001]) and strain ⊥ [010]<br>• ($\boldsymbol{B}$ ∥ [001] or $\boldsymbol{B}$ ⊥ [100]) and strain ⊥ [010]<br>• ($\boldsymbol{B}$ ∥ [001] or $\boldsymbol{B}$ ⊥ [100]) and strain ⊥ [001]<br>• $\boldsymbol{B}$ in generic direction
$P2/c$ (13.65) $\mathbb{Z}_2 \times \mathbb{Z}_2$	• strain ⊥ [010]
Pc' (7.26) $\mathbb{Z}_1$	• $\boldsymbol{E}$ ∥ [010] and strain ⊥ [100]<br>• $\boldsymbol{E}$ ∥ [010] and ($\boldsymbol{B}$ ∥ [001] or $\boldsymbol{B}$ ⊥ [100])<br>• $\boldsymbol{E}$ ∥ [001] and strain ⊥ [100]<br>• $\boldsymbol{E}$ ∥ [001] and ($\boldsymbol{B}$ ∥ [001] or $\boldsymbol{B}$ ⊥ [100])<br>• $\boldsymbol{E}$ ⊥ [100]
$Pn'c'2$ (30.115) $\mathbb{Z}_1$	• $\boldsymbol{E}$ ∥ [010]
$P2_1'/c'$ (14.79) $\mathbb{Z}_2 \times \mathbb{Z}_4$	• strain ⊥ [100]<br>• ($\boldsymbol{B}$ ∥ [001] or $\boldsymbol{B}$ ⊥ [100])
$P2_1'$ (4.9) $\mathbb{Z}_1$	• $\boldsymbol{E}$ ∥ [001] and strain ⊥ [001]<br>• $\boldsymbol{E}$ ∥ [001] and ($\boldsymbol{B}$ ∥ [100] or $\boldsymbol{B}$ ⊥ [001])
$P2_1'/c'$ (14.79) $\mathbb{Z}_2 \times \mathbb{Z}_4$	• strain ⊥ [001]<br>• ($\boldsymbol{B}$ ∥ [100] or $\boldsymbol{B}$ ⊥ [001])
$Pca'2_1'$ (29.102) $\mathbb{Z}_1$	• $\boldsymbol{E}$ ∥ [001]

Table. S 535: Subgroups of $Pb'c'n'$ (60.425) that can be realized by applying $\boldsymbol{E}/\boldsymbol{B}$ fields and/or uniaxial strains.

Subgroup & SI group	Perturbation
Pc' (7.26) $\mathbb{Z}_1$	• $\boldsymbol{B} \parallel [100]$ and strain $\perp [010]$<br>• $\boldsymbol{B} \parallel [001]$ and strain $\perp [010]$<br>• $\boldsymbol{B} \perp [010]$<br>• $\boldsymbol{E} \parallel [100]$ and strain $\perp [010]$<br>• $\boldsymbol{E} \parallel [100]$ and $\boldsymbol{B} \parallel [001]$<br>• $\boldsymbol{E} \parallel [001]$ and strain $\perp [010]$<br>• $\boldsymbol{E} \parallel [001]$ and $\boldsymbol{B} \parallel [100]$<br>• $\boldsymbol{E} \perp [010]$
Pc' (7.26) $\mathbb{Z}_1$	• $\boldsymbol{B} \parallel [100]$ and strain $\perp [001]$<br>• $\boldsymbol{B} \parallel [010]$ and strain $\perp [001]$<br>• $\boldsymbol{B} \perp [001]$<br>• $\boldsymbol{E} \parallel [100]$ and strain $\perp [001]$<br>• $\boldsymbol{E} \parallel [100]$ and $\boldsymbol{B} \parallel [010]$<br>• $\boldsymbol{E} \parallel [010]$ and strain $\perp [001]$<br>• $\boldsymbol{E} \parallel [010]$ and $\boldsymbol{B} \parallel [100]$<br>• $\boldsymbol{E} \perp [001]$
$P2_1$ (4.7) $\mathbb{Z}_1$	• $\boldsymbol{B} \parallel [100]$ and strain $\perp [100]$<br>• $\boldsymbol{E} \parallel [100]$ and strain $\perp [100]$
$Pn'a'2_1$ (33.148) $\mathbb{Z}_1$	• $\boldsymbol{B} \parallel [100]$<br>• $\boldsymbol{E} \parallel [100]$
$P2$ (3.1) $\mathbb{Z}_2$	• $\boldsymbol{B} \parallel [010]$ and strain $\perp [010]$<br>• $\boldsymbol{E} \parallel [010]$ and strain $\perp [010]$
$P\bar{1}'$ (2.6) $\mathbb{Z}_1$	• strain in generic direction
$P2/c'$ (13.68) $\mathbb{Z}_2$	• strain $\perp [010]$
Pc' (7.26) $\mathbb{Z}_1$	• $\boldsymbol{B} \parallel [010]$ and strain $\perp [100]$<br>• $\boldsymbol{B} \parallel [001]$ and strain $\perp [100]$<br>• $\boldsymbol{B} \perp [100]$<br>• $\boldsymbol{E} \parallel [010]$ and strain $\perp [100]$<br>• $\boldsymbol{E} \parallel [010]$ and $\boldsymbol{B} \parallel [001]$<br>• $\boldsymbol{E} \parallel [001]$ and strain $\perp [100]$<br>• $\boldsymbol{E} \parallel [001]$ and $\boldsymbol{B} \parallel [010]$<br>• $\boldsymbol{E} \perp [100]$
$Pn'c'2$ (30.115) $\mathbb{Z}_1$	• $\boldsymbol{B} \parallel [010]$<br>• $\boldsymbol{E} \parallel [010]$
$P2_1/c'$ (14.78) $\mathbb{Z}_1$	• strain $\perp [100]$

Continued on next page

...continued

Subgroup & SI group	Perturbation
$P2_1$ (4.7) $\mathbb{Z}_1$	• $\boldsymbol{B}$ ∥ [001] and strain ⊥ [001]<br>• $\boldsymbol{E}$ ∥ [001] and strain ⊥ [001]
$P2_1/c'$ (14.78) $\mathbb{Z}_1$	• strain ⊥ [001]
$Pc'a'2_1$ (29.103) $\mathbb{Z}_1$	• $\boldsymbol{B}$ ∥ [001]<br>• $\boldsymbol{E}$ ∥ [001]

Table. S 536: Subgroups of P_abcn (60.426) that can be realized by applying $\boldsymbol{E}/\boldsymbol{B}$ fields and/or uniaxial strains.

Subgroup & SI group	Perturbation
Pc' (7.26) $\mathbb{Z}_1$	• $\boldsymbol{E}$ ∥ [100] and $\boldsymbol{B}$ ∥ [100] and strain ⊥ [001]<br>• $\boldsymbol{E}$ ∥ [100] and $\boldsymbol{B}$ ∥ [010] and strain ⊥ [001]<br>• $\boldsymbol{E}$ ∥ [100] and $\boldsymbol{B}$ ⊥ [001]<br>• $\boldsymbol{E}$ ∥ [010] and $\boldsymbol{B}$ ∥ [100] and strain ⊥ [001]<br>• $\boldsymbol{E}$ ∥ [010] and $\boldsymbol{B}$ ∥ [010] and strain ⊥ [001]<br>• $\boldsymbol{E}$ ∥ [010] and $\boldsymbol{B}$ ⊥ [001]<br>• $\boldsymbol{E}$ ⊥ [001] and $\boldsymbol{B}$ ∥ [100]<br>• $\boldsymbol{E}$ ⊥ [001] and $\boldsymbol{B}$ ∥ [010]<br>• $\boldsymbol{E}$ ⊥ [001] and $\boldsymbol{B}$ ⊥ [001]
Pc (7.24) $\mathbb{Z}_1$	• $\boldsymbol{E}$ ∥ [100] and $\boldsymbol{B}$ ∥ [010] and strain ⊥ [010]<br>• $\boldsymbol{E}$ ∥ [001] and $\boldsymbol{B}$ ∥ [010] and strain ⊥ [010]<br>• $\boldsymbol{E}$ ⊥ [010] and $\boldsymbol{B}$ ∥ [010]
$P2'$ (3.3) $\mathbb{Z}_1$	• $\boldsymbol{E}$ ∥ [100] and $\boldsymbol{B}$ ∥ [010] and strain ⊥ [100]<br>• $\boldsymbol{E}$ ∥ [100] and $\boldsymbol{B}$ ∥ [001] and strain ⊥ [100]<br>• $\boldsymbol{E}$ ∥ [100] and $\boldsymbol{B}$ ⊥ [100]
$Pb'a2'$ (32.137) $\mathbb{Z}_1$	• $\boldsymbol{E}$ ∥ [100] and $\boldsymbol{B}$ ∥ [010]

Continued on next page

…continued

Subgroup & SI group	Perturbation
P_S1 (1.3) $\mathbb{Z}_1$	• $\boldsymbol{E} \parallel [100]$ and strain in generic direction<br>• $\boldsymbol{E} \parallel [010]$ and strain in generic direction<br>• $\boldsymbol{E} \parallel [001]$ and strain in generic direction<br>• $\boldsymbol{E} \perp [100]$ and strain $\perp [010]$<br>• $\boldsymbol{E} \perp [100]$ and strain $\perp [001]$<br>• $\boldsymbol{E} \perp [100]$ and strain in generic direction<br>• $\boldsymbol{E} \perp [010]$ and strain $\perp [100]$<br>• $\boldsymbol{E} \perp [010]$ and strain $\perp [001]$<br>• $\boldsymbol{E} \perp [010]$ and strain in generic direction<br>• $\boldsymbol{E} \perp [001]$ and strain $\perp [100]$<br>• $\boldsymbol{E} \perp [001]$ and strain $\perp [010]$<br>• $\boldsymbol{E} \perp [001]$ and strain in generic direction<br>• $\boldsymbol{E}$ in generic direction
Pc (7.24) $\mathbb{Z}_1$	• $\boldsymbol{E} \parallel [100]$ and $\boldsymbol{B} \parallel [001]$ and strain $\perp [001]$<br>• $\boldsymbol{E} \parallel [010]$ and $\boldsymbol{B} \parallel [001]$ and strain $\perp [001]$<br>• $\boldsymbol{E} \perp [001]$ and $\boldsymbol{B} \parallel [001]$
P_ac (7.27) $\mathbb{Z}_1$	• $\boldsymbol{E} \parallel [100]$ and strain $\perp [001]$<br>• $\boldsymbol{E} \parallel [010]$ and strain $\perp [001]$<br>• $\boldsymbol{E} \perp [001]$
Pc' (7.26) $\mathbb{Z}_1$	• $\boldsymbol{E} \parallel [100]$ and $\boldsymbol{B} \parallel [100]$ and strain $\perp [010]$<br>• $\boldsymbol{E} \parallel [100]$ and $\boldsymbol{B} \parallel [001]$ and strain $\perp [010]$<br>• $\boldsymbol{E} \parallel [100]$ and $\boldsymbol{B} \perp [010]$<br>• $\boldsymbol{E} \parallel [001]$ and $\boldsymbol{B} \parallel [100]$ and strain $\perp [010]$<br>• $\boldsymbol{E} \parallel [001]$ and $\boldsymbol{B} \parallel [001]$ and strain $\perp [010]$<br>• $\boldsymbol{E} \parallel [001]$ and $\boldsymbol{B} \perp [010]$<br>• $\boldsymbol{E} \perp [010]$ and $\boldsymbol{B} \parallel [100]$<br>• $\boldsymbol{E} \perp [010]$ and $\boldsymbol{B} \parallel [001]$<br>• $\boldsymbol{E} \perp [010]$ and $\boldsymbol{B} \perp [010]$
P_ac (7.27) $\mathbb{Z}_1$	• $\boldsymbol{E} \parallel [100]$ and strain $\perp [010]$<br>• $\boldsymbol{E} \parallel [001]$ and strain $\perp [010]$<br>• $\boldsymbol{E} \perp [010]$
$Pn'n2'$ (34.158) $\mathbb{Z}_1$	• $\boldsymbol{E} \parallel [100]$ and $\boldsymbol{B} \parallel [001]$
$P2_1$ (4.7) $\mathbb{Z}_1$	• $\boldsymbol{E} \parallel [100]$ and $\boldsymbol{B} \parallel [100]$ and strain $\perp [100]$
P_b2_1 (4.11) $\mathbb{Z}_1$	• $\boldsymbol{E} \parallel [100]$ and strain $\perp [100]$
$Pn'a'2_1$ (33.148) $\mathbb{Z}_1$	• $\boldsymbol{E} \parallel [100]$ and $\boldsymbol{B} \parallel [100]$
P_cna2_1 (33.151) $\mathbb{Z}_1$	• $\boldsymbol{E} \parallel [100]$

Continued on next page

...continued

Subgroup & SI group	Perturbation
$P2$ (3.1) $\mathbb{Z}_2$	• $\boldsymbol{E}$ || [010] and $\boldsymbol{B}$ || [010] and strain $\perp$ [010]
Pc' (7.26) $\mathbb{Z}_1$	• $\boldsymbol{E}$ || [010] and $\boldsymbol{B}$ || [010] and strain $\perp$ [100]<br>• $\boldsymbol{E}$ || [010] and $\boldsymbol{B}$ || [001] and strain $\perp$ [100]<br>• $\boldsymbol{E}$ || [010] and $\boldsymbol{B}$ $\perp$ [100]<br>• $\boldsymbol{E}$ || [001] and $\boldsymbol{B}$ || [010] and strain $\perp$ [100]<br>• $\boldsymbol{E}$ || [001] and $\boldsymbol{B}$ || [001] and strain $\perp$ [100]<br>• $\boldsymbol{E}$ || [001] and $\boldsymbol{B}$ $\perp$ [100]<br>• $\boldsymbol{E}$ $\perp$ [100] and $\boldsymbol{B}$ || [010]<br>• $\boldsymbol{E}$ $\perp$ [100] and $\boldsymbol{B}$ || [001]<br>• $\boldsymbol{E}$ $\perp$ [100] and $\boldsymbol{B}$ $\perp$ [100]
$Pc'c'2$ (27.81) $\mathbb{Z}_2$	• $\boldsymbol{E}$ || [010] and $\boldsymbol{B}$ || [010]
$P\bar{1}$ (2.4) $\mathbb{Z}_2 \times \mathbb{Z}_2 \times \mathbb{Z}_2 \times \mathbb{Z}_4$	• $\boldsymbol{B}$ || [100] and strain in generic direction<br>• $\boldsymbol{B}$ || [010] and strain in generic direction<br>• $\boldsymbol{B}$ || [001] and strain in generic direction<br>• $\boldsymbol{B}$ $\perp$ [100] and strain $\perp$ [010]<br>• $\boldsymbol{B}$ $\perp$ [100] and strain $\perp$ [001]<br>• $\boldsymbol{B}$ $\perp$ [100] and strain in generic direction<br>• $\boldsymbol{B}$ $\perp$ [010] and strain $\perp$ [100]<br>• $\boldsymbol{B}$ $\perp$ [010] and strain $\perp$ [001]<br>• $\boldsymbol{B}$ $\perp$ [010] and strain in generic direction<br>• $\boldsymbol{B}$ $\perp$ [001] and strain $\perp$ [100]<br>• $\boldsymbol{B}$ $\perp$ [001] and strain $\perp$ [010]<br>• $\boldsymbol{B}$ $\perp$ [001] and strain in generic direction<br>• $\boldsymbol{B}$ in generic direction
$P2/c$ (13.65) $\mathbb{Z}_2 \times \mathbb{Z}_2$	• $\boldsymbol{B}$ || [010] and strain $\perp$ [010]
$P2'/c'$ (13.69) $\mathbb{Z}_2 \times \mathbb{Z}_2 \times \mathbb{Z}_4$	• $\boldsymbol{B}$ || [010] and strain $\perp$ [100]<br>• $\boldsymbol{B}$ || [001] and strain $\perp$ [100]<br>• $\boldsymbol{B}$ $\perp$ [100]
$P2_1'$ (4.9) $\mathbb{Z}_1$	• $\boldsymbol{E}$ || [001] and $\boldsymbol{B}$ || [100] and strain $\perp$ [001]<br>• $\boldsymbol{E}$ || [001] and $\boldsymbol{B}$ || [010] and strain $\perp$ [001]<br>• $\boldsymbol{E}$ || [001] and $\boldsymbol{B}$ $\perp$ [001]
$Pca'2_1'$ (29.102) $\mathbb{Z}_1$	• $\boldsymbol{E}$ || [001] and $\boldsymbol{B}$ || [010]
$P2_1'/c'$ (14.79) $\mathbb{Z}_2 \times \mathbb{Z}_4$	• $\boldsymbol{B}$ || [100] and strain $\perp$ [001]<br>• $\boldsymbol{B}$ || [010] and strain $\perp$ [001]<br>• $\boldsymbol{B}$ $\perp$ [001]
$Pc'c'a$ (54.342) $\mathbb{Z}_2 \times \mathbb{Z}_2$	• $\boldsymbol{B}$ || [010]

Continued on next page

…continued

Subgroup & SI group	Perturbation
$P2'$ (3.3) $\mathbb{Z}_1$	• $\boldsymbol{E} \parallel [010]$ and $\boldsymbol{B} \parallel [100]$ and strain $\perp [010]$<br>• $\boldsymbol{E} \parallel [010]$ and $\boldsymbol{B} \parallel [001]$ and strain $\perp [010]$<br>• $\boldsymbol{E} \parallel [010]$ and $\boldsymbol{B} \perp [010]$
P_a2 (3.4) $\mathbb{Z}_2$	• $\boldsymbol{E} \parallel [010]$ and strain $\perp [010]$
$Pnc'2'$ (30.114) $\mathbb{Z}_1$	• $\boldsymbol{E} \parallel [010]$ and $\boldsymbol{B} \parallel [001]$
$P2'/c'$ (13.69) $\mathbb{Z}_2 \times \mathbb{Z}_2 \times \mathbb{Z}_4$	• $\boldsymbol{B} \parallel [100]$ and strain $\perp [010]$<br>• $\boldsymbol{B} \parallel [001]$ and strain $\perp [010]$<br>• $\boldsymbol{B} \perp [010]$
Pc (7.24) $\mathbb{Z}_1$	• $\boldsymbol{E} \parallel [010]$ and $\boldsymbol{B} \parallel [100]$ and strain $\perp [100]$<br>• $\boldsymbol{E} \parallel [001]$ and $\boldsymbol{B} \parallel [100]$ and strain $\perp [100]$<br>• $\boldsymbol{E} \perp [100]$ and $\boldsymbol{B} \parallel [100]$
P_bc (7.29) $\mathbb{Z}_1$	• $\boldsymbol{E} \parallel [010]$ and strain $\perp [100]$<br>• $\boldsymbol{E} \parallel [001]$ and strain $\perp [100]$<br>• $\boldsymbol{E} \perp [100]$
$P2_1/c$ (14.75) $\mathbb{Z}_2$	• $\boldsymbol{B} \parallel [100]$ and strain $\perp [100]$
$Pn'a2_1'$ (33.146) $\mathbb{Z}_1$	• $\boldsymbol{E} \parallel [001]$ and $\boldsymbol{B} \parallel [100]$
$Pc'c2'$ (27.80) $\mathbb{Z}_1$	• $\boldsymbol{E} \parallel [010]$ and $\boldsymbol{B} \parallel [100]$
P_bnc2 (30.117) $\mathbb{Z}_2$	• $\boldsymbol{E} \parallel [010]$
$Pc'cn'$ (56.370) $\mathbb{Z}_2$	• $\boldsymbol{B} \parallel [100]$
$P_S\bar{1}$ (2.7) $\mathbb{Z}_2 \times \mathbb{Z}_2 \times \mathbb{Z}_4$	• strain in generic direction
P_a2/c (13.70) $\mathbb{Z}_2 \times \mathbb{Z}_2$	• strain $\perp [010]$
P_b2_1/c (14.81) $\mathbb{Z}_2$	• strain $\perp [100]$
$P2_1$ (4.7) $\mathbb{Z}_1$	• $\boldsymbol{E} \parallel [001]$ and $\boldsymbol{B} \parallel [001]$ and strain $\perp [001]$
$Pn'a'2_1$ (33.148) $\mathbb{Z}_1$	• $\boldsymbol{E} \parallel [001]$ and $\boldsymbol{B} \parallel [001]$
$P2_1/c$ (14.75) $\mathbb{Z}_2$	• $\boldsymbol{B} \parallel [001]$ and strain $\perp [001]$
P_a2_1 (4.10) $\mathbb{Z}_1$	• $\boldsymbol{E} \parallel [001]$ and strain $\perp [001]$
$Pn'na'$ (52.312) $\mathbb{Z}_2$	• $\boldsymbol{B} \parallel [001]$
P_bca2_1 (29.105) $\mathbb{Z}_2$	• $\boldsymbol{E} \parallel [001]$
P_a2_1/c (14.80) $\mathbb{Z}_2$	• strain $\perp [001]$

Table. S 537: Subgroups of P_bbcn (60.427) that can be realized by applying $\boldsymbol{E}/\boldsymbol{B}$ fields and/or uniaxial strains.

Subgroup & SI group	Perturbation
P_S1 (1.3) $\mathbb{Z}_1$	• $\boldsymbol{E}$ ∥ [100] and strain in generic direction<br>• $\boldsymbol{E}$ ∥ [010] and strain in generic direction<br>• $\boldsymbol{E}$ ∥ [001] and strain in generic direction<br>• $\boldsymbol{E}$ ⊥ [100] and strain ⊥ [010]<br>• $\boldsymbol{E}$ ⊥ [100] and strain ⊥ [001]<br>• $\boldsymbol{E}$ ⊥ [100] and strain in generic direction<br>• $\boldsymbol{E}$ ⊥ [010] and strain ⊥ [100]<br>• $\boldsymbol{E}$ ⊥ [010] and strain ⊥ [001]<br>• $\boldsymbol{E}$ ⊥ [010] and strain in generic direction<br>• $\boldsymbol{E}$ ⊥ [001] and strain ⊥ [100]<br>• $\boldsymbol{E}$ ⊥ [001] and strain ⊥ [010]<br>• $\boldsymbol{E}$ ⊥ [001] and strain in generic direction<br>• $\boldsymbol{E}$ in generic direction
Pc (7.24) $\mathbb{Z}_1$	• $\boldsymbol{E}$ ∥ [100] and $\boldsymbol{B}$ ∥ [010] and strain ⊥ [010]<br>• $\boldsymbol{E}$ ∥ [001] and $\boldsymbol{B}$ ∥ [010] and strain ⊥ [010]<br>• $\boldsymbol{E}$ ⊥ [010] and $\boldsymbol{B}$ ∥ [010]
Pc' (7.26) $\mathbb{Z}_1$	• $\boldsymbol{E}$ ∥ [100] and $\boldsymbol{B}$ ∥ [100] and strain ⊥ [010]<br>• $\boldsymbol{E}$ ∥ [100] and $\boldsymbol{B}$ ∥ [001] and strain ⊥ [010]<br>• $\boldsymbol{E}$ ∥ [100] and $\boldsymbol{B}$ ⊥ [010]<br>• $\boldsymbol{E}$ ∥ [001] and $\boldsymbol{B}$ ∥ [100] and strain ⊥ [010]<br>• $\boldsymbol{E}$ ∥ [001] and $\boldsymbol{B}$ ∥ [001] and strain ⊥ [010]<br>• $\boldsymbol{E}$ ∥ [001] and $\boldsymbol{B}$ ⊥ [010]<br>• $\boldsymbol{E}$ ⊥ [010] and $\boldsymbol{B}$ ∥ [100]<br>• $\boldsymbol{E}$ ⊥ [010] and $\boldsymbol{B}$ ∥ [001]<br>• $\boldsymbol{E}$ ⊥ [010] and $\boldsymbol{B}$ ⊥ [010]
P_bc (7.29) $\mathbb{Z}_1$	• $\boldsymbol{E}$ ∥ [100] and strain ⊥ [010]<br>• $\boldsymbol{E}$ ∥ [001] and strain ⊥ [010]<br>• $\boldsymbol{E}$ ⊥ [010]
Pc' (7.26) $\mathbb{Z}_1$	• $\boldsymbol{E}$ ∥ [100] and $\boldsymbol{B}$ ∥ [100] and strain ⊥ [001]<br>• $\boldsymbol{E}$ ∥ [100] and $\boldsymbol{B}$ ∥ [010] and strain ⊥ [001]<br>• $\boldsymbol{E}$ ∥ [100] and $\boldsymbol{B}$ ⊥ [001]<br>• $\boldsymbol{E}$ ∥ [010] and $\boldsymbol{B}$ ∥ [100] and strain ⊥ [001]<br>• $\boldsymbol{E}$ ∥ [010] and $\boldsymbol{B}$ ∥ [010] and strain ⊥ [001]<br>• $\boldsymbol{E}$ ∥ [010] and $\boldsymbol{B}$ ⊥ [001]<br>• $\boldsymbol{E}$ ⊥ [001] and $\boldsymbol{B}$ ∥ [100]<br>• $\boldsymbol{E}$ ⊥ [001] and $\boldsymbol{B}$ ∥ [010]<br>• $\boldsymbol{E}$ ⊥ [001] and $\boldsymbol{B}$ ⊥ [001]

Continued on next page

. . . continued

Subgroup & SI group	Perturbation
Pc (7.24) $\mathbb{Z}_1$	• $\boldsymbol{E} \parallel [100]$ and $\boldsymbol{B} \parallel [001]$ and strain $\perp [001]$<br>• $\boldsymbol{E} \parallel [010]$ and $\boldsymbol{B} \parallel [001]$ and strain $\perp [001]$<br>• $\boldsymbol{E} \perp [001]$ and $\boldsymbol{B} \parallel [001]$
P_ac (7.27) $\mathbb{Z}_1$	• $\boldsymbol{E} \parallel [100]$ and strain $\perp [001]$<br>• $\boldsymbol{E} \parallel [010]$ and strain $\perp [001]$<br>• $\boldsymbol{E} \perp [001]$
$P2_1'$ (4.9) $\mathbb{Z}_1$	• $\boldsymbol{E} \parallel [100]$ and $\boldsymbol{B} \parallel [010]$ and strain $\perp [100]$<br>• $\boldsymbol{E} \parallel [100]$ and $\boldsymbol{B} \parallel [001]$ and strain $\perp [100]$<br>• $\boldsymbol{E} \parallel [100]$ and $\boldsymbol{B} \perp [100]$
$Pc'a2_1'$ (29.101) $\mathbb{Z}_1$	• $\boldsymbol{E} \parallel [100]$ and $\boldsymbol{B} \parallel [010]$
$Pna'2_1'$ (33.147) $\mathbb{Z}_1$	• $\boldsymbol{E} \parallel [100]$ and $\boldsymbol{B} \parallel [001]$
$P2_1$ (4.7) $\mathbb{Z}_1$	• $\boldsymbol{E} \parallel [100]$ and $\boldsymbol{B} \parallel [100]$ and strain $\perp [100]$
$Pc'a'2_1$ (29.103) $\mathbb{Z}_1$	• $\boldsymbol{E} \parallel [100]$ and $\boldsymbol{B} \parallel [100]$
P_a2_1 (4.10) $\mathbb{Z}_1$	• $\boldsymbol{E} \parallel [100]$ and strain $\perp [100]$
P_bna2_1 (33.150) $\mathbb{Z}_1$	• $\boldsymbol{E} \parallel [100]$
$P2$ (3.1) $\mathbb{Z}_2$	• $\boldsymbol{E} \parallel [010]$ and $\boldsymbol{B} \parallel [010]$ and strain $\perp [010]$
$P2_1'$ (4.9) $\mathbb{Z}_1$	• $\boldsymbol{E} \parallel [010]$ and $\boldsymbol{B} \parallel [100]$ and strain $\perp [010]$<br>• $\boldsymbol{E} \parallel [010]$ and $\boldsymbol{B} \parallel [001]$ and strain $\perp [010]$<br>• $\boldsymbol{E} \parallel [010]$ and $\boldsymbol{B} \perp [010]$
P_b2 (3.5) $\mathbb{Z}_2$	• $\boldsymbol{E} \parallel [010]$ and strain $\perp [010]$
$P\bar{1}$ (2.4) $\mathbb{Z}_2 \times \mathbb{Z}_2 \times \mathbb{Z}_2 \times \mathbb{Z}_4$	• $\boldsymbol{B} \parallel [100]$ and strain in generic direction<br>• $\boldsymbol{B} \parallel [010]$ and strain in generic direction<br>• $\boldsymbol{B} \parallel [001]$ and strain in generic direction<br>• $\boldsymbol{B} \perp [100]$ and strain $\perp [010]$<br>• $\boldsymbol{B} \perp [100]$ and strain $\perp [001]$<br>• $\boldsymbol{B} \perp [100]$ and strain in generic direction<br>• $\boldsymbol{B} \perp [010]$ and strain $\perp [100]$<br>• $\boldsymbol{B} \perp [010]$ and strain $\perp [001]$<br>• $\boldsymbol{B} \perp [010]$ and strain in generic direction<br>• $\boldsymbol{B} \perp [001]$ and strain $\perp [100]$<br>• $\boldsymbol{B} \perp [001]$ and strain $\perp [010]$<br>• $\boldsymbol{B} \perp [001]$ and strain in generic direction<br>• $\boldsymbol{B}$ in generic direction
$P2/c$ (13.65) $\mathbb{Z}_2 \times \mathbb{Z}_2$	• $\boldsymbol{B} \parallel [010]$ and strain $\perp [010]$

Continued on next page

...continued

Subgroup & SI group	Perturbation
$P2_1'/c'$ (14.79) $\mathbb{Z}_2 \times \mathbb{Z}_4$	• $\boldsymbol{B} \parallel [100]$ and strain $\perp [010]$<br>• $\boldsymbol{B} \parallel [001]$ and strain $\perp [010]$<br>• $\boldsymbol{B} \perp [010]$
$P_S\bar{1}$ (2.7) $\mathbb{Z}_2 \times \mathbb{Z}_2 \times \mathbb{Z}_4$	• strain in generic direction
P_b2/c (13.71) $\mathbb{Z}_2 \times \mathbb{Z}_2$	• strain $\perp [010]$
Pm' (6.20) $\mathbb{Z}_1$	• $\boldsymbol{E} \parallel [010]$ and $\boldsymbol{B} \parallel [010]$ and strain $\perp [100]$<br>• $\boldsymbol{E} \parallel [010]$ and $\boldsymbol{B} \parallel [001]$ and strain $\perp [100]$<br>• $\boldsymbol{E} \parallel [010]$ and $\boldsymbol{B} \perp [100]$<br>• $\boldsymbol{E} \parallel [001]$ and $\boldsymbol{B} \parallel [010]$ and strain $\perp [100]$<br>• $\boldsymbol{E} \parallel [001]$ and $\boldsymbol{B} \parallel [001]$ and strain $\perp [100]$<br>• $\boldsymbol{E} \parallel [001]$ and $\boldsymbol{B} \perp [100]$<br>• $\boldsymbol{E} \perp [100]$ and $\boldsymbol{B} \parallel [010]$<br>• $\boldsymbol{E} \perp [100]$ and $\boldsymbol{B} \parallel [001]$<br>• $\boldsymbol{E} \perp [100]$ and $\boldsymbol{B} \perp [100]$
$Pm'a'2$ (28.91) $\mathbb{Z}_2$	• $\boldsymbol{E} \parallel [010]$ and $\boldsymbol{B} \parallel [010]$
$Pm'n2_1'$ (31.125) $\mathbb{Z}_1$	• $\boldsymbol{E} \parallel [010]$ and $\boldsymbol{B} \parallel [001]$
$P2_1'/m'$ (11.54) $\mathbb{Z}_2 \times \mathbb{Z}_2 \times \mathbb{Z}_4$	• $\boldsymbol{B} \parallel [010]$ and strain $\perp [100]$<br>• $\boldsymbol{B} \parallel [001]$ and strain $\perp [100]$<br>• $\boldsymbol{B} \perp [100]$
Pc (7.24) $\mathbb{Z}_1$	• $\boldsymbol{E} \parallel [010]$ and $\boldsymbol{B} \parallel [100]$ and strain $\perp [100]$<br>• $\boldsymbol{E} \parallel [001]$ and $\boldsymbol{B} \parallel [100]$ and strain $\perp [100]$<br>• $\boldsymbol{E} \perp [100]$ and $\boldsymbol{B} \parallel [100]$
$Pca'2_1'$ (29.102) $\mathbb{Z}_1$	• $\boldsymbol{E} \parallel [010]$ and $\boldsymbol{B} \parallel [100]$
$P2_1/c$ (14.75) $\mathbb{Z}_2$	• $\boldsymbol{B} \parallel [100]$ and strain $\perp [100]$
P_cc (7.28) $\mathbb{Z}_1$	• $\boldsymbol{E} \parallel [010]$ and strain $\perp [100]$<br>• $\boldsymbol{E} \parallel [001]$ and strain $\perp [100]$<br>• $\boldsymbol{E} \perp [100]$
P_cnc2 (30.118) $\mathbb{Z}_2$	• $\boldsymbol{E} \parallel [010]$
P_c2_1/c (14.82) $\mathbb{Z}_2$	• strain $\perp [100]$
$P2_1'$ (4.9) $\mathbb{Z}_1$	• $\boldsymbol{E} \parallel [001]$ and $\boldsymbol{B} \parallel [100]$ and strain $\perp [001]$<br>• $\boldsymbol{E} \parallel [001]$ and $\boldsymbol{B} \parallel [010]$ and strain $\perp [001]$<br>• $\boldsymbol{E} \parallel [001]$ and $\boldsymbol{B} \perp [001]$
$P2_1'/c'$ (14.79) $\mathbb{Z}_2 \times \mathbb{Z}_4$	• $\boldsymbol{B} \parallel [100]$ and strain $\perp [001]$<br>• $\boldsymbol{B} \parallel [010]$ and strain $\perp [001]$<br>• $\boldsymbol{B} \perp [001]$

Continued on next page

...continued

Subgroup & SI group	Perturbation
$Pm'c2_1'$ (26.68) $\mathbb{Z}_1$	• $\boldsymbol{E}$ ∥ [001] and $\boldsymbol{B}$ ∥ [010]
$Pbc'm'$ (57.383) $\mathbb{Z}_2 \times \mathbb{Z}_2$	• $\boldsymbol{B}$ ∥ [010]
$Pc'a2_1'$ (29.101) $\mathbb{Z}_1$	• $\boldsymbol{E}$ ∥ [001] and $\boldsymbol{B}$ ∥ [100]
$Pb'c'a$ (61.436) $\mathbb{Z}_2$	• $\boldsymbol{B}$ ∥ [100]
$P2_1$ (4.7) $\mathbb{Z}_1$	• $\boldsymbol{E}$ ∥ [001] and $\boldsymbol{B}$ ∥ [001] and strain ⊥ [001]
$P2_1/c$ (14.75) $\mathbb{Z}_2$	• $\boldsymbol{B}$ ∥ [001] and strain ⊥ [001]
$Pm'c'2_1$ (26.70) $\mathbb{Z}_1$	• $\boldsymbol{E}$ ∥ [001] and $\boldsymbol{B}$ ∥ [001]
$Pnm'a'$ (62.447) $\mathbb{Z}_2$	• $\boldsymbol{B}$ ∥ [001]
P_a2_1 (4.10) $\mathbb{Z}_1$	• $\boldsymbol{E}$ ∥ [001] and strain ⊥ [001]
P_a2_1/c (14.80) $\mathbb{Z}_2$	• strain ⊥ [001]
P_aca2_1 (29.104) $\mathbb{Z}_1$	• $\boldsymbol{E}$ ∥ [001]

Table. S 538: Subgroups of P_cbcn (60.428) that can be realized by applying $\boldsymbol{E}/\boldsymbol{B}$ fields and/or uniaxial strains.

Subgroup & SI group	Perturbation
P_S1 (1.3) $\mathbb{Z}_1$	• $\boldsymbol{E}$ ∥ [100] and strain in generic direction<br>• $\boldsymbol{E}$ ∥ [010] and strain in generic direction<br>• $\boldsymbol{E}$ ∥ [001] and strain in generic direction<br>• $\boldsymbol{E}$ ⊥ [100] and strain ⊥ [010]<br>• $\boldsymbol{E}$ ⊥ [100] and strain ⊥ [001]<br>• $\boldsymbol{E}$ ⊥ [100] and strain in generic direction<br>• $\boldsymbol{E}$ ⊥ [010] and strain ⊥ [100]<br>• $\boldsymbol{E}$ ⊥ [010] and strain ⊥ [001]<br>• $\boldsymbol{E}$ ⊥ [010] and strain in generic direction<br>• $\boldsymbol{E}$ ⊥ [001] and strain ⊥ [100]<br>• $\boldsymbol{E}$ ⊥ [001] and strain ⊥ [010]<br>• $\boldsymbol{E}$ ⊥ [001] and strain in generic direction<br>• $\boldsymbol{E}$ in generic direction

Continued on next page

...continued

Subgroup & SI group	Perturbation
Pm' (6.20) $\mathbb{Z}_1$	• $\boldsymbol{E}$ ∥ [100] and $\boldsymbol{B}$ ∥ [100] and strain ⊥ [010]<br>• $\boldsymbol{E}$ ∥ [100] and $\boldsymbol{B}$ ∥ [001] and strain ⊥ [010]<br>• $\boldsymbol{E}$ ∥ [100] and $\boldsymbol{B}$ ⊥ [010]<br>• $\boldsymbol{E}$ ∥ [001] and $\boldsymbol{B}$ ∥ [100] and strain ⊥ [010]<br>• $\boldsymbol{E}$ ∥ [001] and $\boldsymbol{B}$ ∥ [001] and strain ⊥ [010]<br>• $\boldsymbol{E}$ ∥ [001] and $\boldsymbol{B}$ ⊥ [010]<br>• $\boldsymbol{E}$ ⊥ [010] and $\boldsymbol{B}$ ∥ [100]<br>• $\boldsymbol{E}$ ⊥ [010] and $\boldsymbol{B}$ ∥ [001]<br>• $\boldsymbol{E}$ ⊥ [010] and $\boldsymbol{B}$ ⊥ [010]
Pc (7.24) $\mathbb{Z}_1$	• $\boldsymbol{E}$ ∥ [100] and $\boldsymbol{B}$ ∥ [010] and strain ⊥ [010]<br>• $\boldsymbol{E}$ ∥ [001] and $\boldsymbol{B}$ ∥ [010] and strain ⊥ [010]<br>• $\boldsymbol{E}$ ⊥ [010] and $\boldsymbol{B}$ ∥ [010]
P_cc (7.28) $\mathbb{Z}_1$	• $\boldsymbol{E}$ ∥ [100] and strain ⊥ [010]<br>• $\boldsymbol{E}$ ∥ [001] and strain ⊥ [010]<br>• $\boldsymbol{E}$ ⊥ [010]
Pc' (7.26) $\mathbb{Z}_1$	• $\boldsymbol{E}$ ∥ [100] and $\boldsymbol{B}$ ∥ [100] and strain ⊥ [001]<br>• $\boldsymbol{E}$ ∥ [100] and $\boldsymbol{B}$ ∥ [010] and strain ⊥ [001]<br>• $\boldsymbol{E}$ ∥ [100] and $\boldsymbol{B}$ ⊥ [001]<br>• $\boldsymbol{E}$ ∥ [010] and $\boldsymbol{B}$ ∥ [100] and strain ⊥ [001]<br>• $\boldsymbol{E}$ ∥ [010] and $\boldsymbol{B}$ ∥ [010] and strain ⊥ [001]<br>• $\boldsymbol{E}$ ∥ [010] and $\boldsymbol{B}$ ⊥ [001]<br>• $\boldsymbol{E}$ ⊥ [001] and $\boldsymbol{B}$ ∥ [100]<br>• $\boldsymbol{E}$ ⊥ [001] and $\boldsymbol{B}$ ∥ [010]<br>• $\boldsymbol{E}$ ⊥ [001] and $\boldsymbol{B}$ ⊥ [001]
Pc (7.24) $\mathbb{Z}_1$	• $\boldsymbol{E}$ ∥ [100] and $\boldsymbol{B}$ ∥ [001] and strain ⊥ [001]<br>• $\boldsymbol{E}$ ∥ [010] and $\boldsymbol{B}$ ∥ [001] and strain ⊥ [001]<br>• $\boldsymbol{E}$ ⊥ [001] and $\boldsymbol{B}$ ∥ [001]
P_bc (7.29) $\mathbb{Z}_1$	• $\boldsymbol{E}$ ∥ [100] and strain ⊥ [001]<br>• $\boldsymbol{E}$ ∥ [010] and strain ⊥ [001]<br>• $\boldsymbol{E}$ ⊥ [001]
$P2_1$ (4.7) $\mathbb{Z}_1$	• $\boldsymbol{E}$ ∥ [100] and $\boldsymbol{B}$ ∥ [100] and strain ⊥ [100]
$Pm'n'2_1$ (31.127) $\mathbb{Z}_1$	• $\boldsymbol{E}$ ∥ [100] and $\boldsymbol{B}$ ∥ [100]
$P2_1'$ (4.9) $\mathbb{Z}_1$	• $\boldsymbol{E}$ ∥ [100] and $\boldsymbol{B}$ ∥ [010] and strain ⊥ [100]<br>• $\boldsymbol{E}$ ∥ [100] and $\boldsymbol{B}$ ∥ [001] and strain ⊥ [100]<br>• $\boldsymbol{E}$ ∥ [100] and $\boldsymbol{B}$ ⊥ [100]
$Pn'a2_1'$ (33.146) $\mathbb{Z}_1$	• $\boldsymbol{E}$ ∥ [100] and $\boldsymbol{B}$ ∥ [010]

Continued on next page

...continued

Subgroup & SI group	Perturbation
$Pm'n2_1'$ (31.125) $\mathbb{Z}_1$	• $\boldsymbol{E} \parallel [100]$ and $\boldsymbol{B} \parallel [001]$
P_a2_1 (4.10) $\mathbb{Z}_1$	• $\boldsymbol{E} \parallel [100]$ and strain $\perp [100]$
P_ana2_1 (33.149) $\mathbb{Z}_1$	• $\boldsymbol{E} \parallel [100]$
$P2'$ (3.3) $\mathbb{Z}_1$	• $\boldsymbol{E} \parallel [010]$ and $\boldsymbol{B} \parallel [100]$ and strain $\perp [010]$<br>• $\boldsymbol{E} \parallel [010]$ and $\boldsymbol{B} \parallel [001]$ and strain $\perp [010]$<br>• $\boldsymbol{E} \parallel [010]$ and $\boldsymbol{B} \perp [010]$
$P2$ (3.1) $\mathbb{Z}_2$	• $\boldsymbol{E} \parallel [010]$ and $\boldsymbol{B} \parallel [010]$ and strain $\perp [010]$
P_a2 (3.4) $\mathbb{Z}_2$	• $\boldsymbol{E} \parallel [010]$ and strain $\perp [010]$
$P\bar{1}$ (2.4) $\mathbb{Z}_2 \times \mathbb{Z}_2 \times \mathbb{Z}_2 \times \mathbb{Z}_4$	• $\boldsymbol{B} \parallel [100]$ and strain in generic direction<br>• $\boldsymbol{B} \parallel [010]$ and strain in generic direction<br>• $\boldsymbol{B} \parallel [001]$ and strain in generic direction<br>• $\boldsymbol{B} \perp [100]$ and strain $\perp [010]$<br>• $\boldsymbol{B} \perp [100]$ and strain $\perp [001]$<br>• $\boldsymbol{B} \perp [100]$ and strain in generic direction<br>• $\boldsymbol{B} \perp [010]$ and strain $\perp [100]$<br>• $\boldsymbol{B} \perp [010]$ and strain $\perp [001]$<br>• $\boldsymbol{B} \perp [010]$ and strain in generic direction<br>• $\boldsymbol{B} \perp [001]$ and strain $\perp [100]$<br>• $\boldsymbol{B} \perp [001]$ and strain $\perp [010]$<br>• $\boldsymbol{B} \perp [001]$ and strain in generic direction<br>• $\boldsymbol{B}$ in generic direction
$P2'/m'$ (10.46) $\mathbb{Z}_2 \times \mathbb{Z}_2 \times \mathbb{Z}_2 \times \mathbb{Z}_4$	• $\boldsymbol{B} \parallel [100]$ and strain $\perp [010]$<br>• $\boldsymbol{B} \parallel [001]$ and strain $\perp [010]$<br>• $\boldsymbol{B} \perp [010]$
$P2/c$ (13.65) $\mathbb{Z}_2 \times \mathbb{Z}_2$	• $\boldsymbol{B} \parallel [010]$ and strain $\perp [010]$
$P_S\bar{1}$ (2.7) $\mathbb{Z}_2 \times \mathbb{Z}_2 \times \mathbb{Z}_4$	• strain in generic direction
P_c2/c (13.72) $\mathbb{Z}_2 \times \mathbb{Z}_2$	• strain $\perp [010]$
Pc (7.24) $\mathbb{Z}_1$	• $\boldsymbol{E} \parallel [010]$ and $\boldsymbol{B} \parallel [100]$ and strain $\perp [100]$<br>• $\boldsymbol{E} \parallel [001]$ and $\boldsymbol{B} \parallel [100]$ and strain $\perp [100]$<br>• $\boldsymbol{E} \perp [100]$ and $\boldsymbol{B} \parallel [100]$
$Pn'c2'$ (30.113) $\mathbb{Z}_1$	• $\boldsymbol{E} \parallel [010]$ and $\boldsymbol{B} \parallel [100]$
$P2_1/c$ (14.75) $\mathbb{Z}_2$	• $\boldsymbol{B} \parallel [100]$ and strain $\perp [100]$

Continued on next page

...continued

Subgroup & SI group	Perturbation
Pc' (7.26) $\mathbb{Z}_1$	• $\boldsymbol{E}$ || [010] and $\boldsymbol{B}$ || [010] and strain $\perp$ [100]<br>• $\boldsymbol{E}$ || [010] and $\boldsymbol{B}$ || [001] and strain $\perp$ [100]<br>• $\boldsymbol{E}$ || [010] and $\boldsymbol{B}$ $\perp$ [100]<br>• $\boldsymbol{E}$ || [001] and $\boldsymbol{B}$ || [010] and strain $\perp$ [100]<br>• $\boldsymbol{E}$ || [001] and $\boldsymbol{B}$ || [001] and strain $\perp$ [100]<br>• $\boldsymbol{E}$ || [001] and $\boldsymbol{B}$ $\perp$ [100]<br>• $\boldsymbol{E}$ $\perp$ [100] and $\boldsymbol{B}$ || [010]<br>• $\boldsymbol{E}$ $\perp$ [100] and $\boldsymbol{B}$ || [001]<br>• $\boldsymbol{E}$ $\perp$ [100] and $\boldsymbol{B}$ $\perp$ [100]
$Pn'n2'$ (34.158) $\mathbb{Z}_1$	• $\boldsymbol{E}$ || [010] and $\boldsymbol{B}$ || [001]
$Pn'n'2$ (34.159) $\mathbb{Z}_1$	• $\boldsymbol{E}$ || [010] and $\boldsymbol{B}$ || [010]
$P2_1'/c'$ (14.79) $\mathbb{Z}_2 \times \mathbb{Z}_4$	• $\boldsymbol{B}$ || [010] and strain $\perp$ [100]<br>• $\boldsymbol{B}$ || [001] and strain $\perp$ [100]<br>• $\boldsymbol{B}$ $\perp$ [100]
P_ac (7.27) $\mathbb{Z}_1$	• $\boldsymbol{E}$ || [010] and strain $\perp$ [100]<br>• $\boldsymbol{E}$ || [001] and strain $\perp$ [100]<br>• $\boldsymbol{E}$ $\perp$ [100]
P_anc2 (30.116) $\mathbb{Z}_1$	• $\boldsymbol{E}$ || [010]
P_a2_1/c (14.80) $\mathbb{Z}_2$	• strain $\perp$ [100]
$P2'$ (3.3) $\mathbb{Z}_1$	• $\boldsymbol{E}$ || [001] and $\boldsymbol{B}$ || [100] and strain $\perp$ [001]<br>• $\boldsymbol{E}$ || [001] and $\boldsymbol{B}$ || [010] and strain $\perp$ [001]<br>• $\boldsymbol{E}$ || [001] and $\boldsymbol{B}$ $\perp$ [001]
$P2'/c'$ (13.69) $\mathbb{Z}_2 \times \mathbb{Z}_2 \times \mathbb{Z}_4$	• $\boldsymbol{B}$ || [100] and strain $\perp$ [001]<br>• $\boldsymbol{B}$ || [010] and strain $\perp$ [001]<br>• $\boldsymbol{B}$ $\perp$ [001]
$Pm'a2'$ (28.89) $\mathbb{Z}_1$	• $\boldsymbol{E}$ || [001] and $\boldsymbol{B}$ || [100]
$Pm'n'a$ (53.326) $\mathbb{Z}_2$	• $\boldsymbol{B}$ || [100]
$Pn'c2'$ (30.113) $\mathbb{Z}_1$	• $\boldsymbol{E}$ || [001] and $\boldsymbol{B}$ || [010]
$Pn'n'a$ (52.310) $\mathbb{Z}_2$	• $\boldsymbol{B}$ || [010]
$P2_1$ (4.7) $\mathbb{Z}_1$	• $\boldsymbol{E}$ || [001] and $\boldsymbol{B}$ || [001] and strain $\perp$ [001]
$P2_1/c$ (14.75) $\mathbb{Z}_2$	• $\boldsymbol{B}$ || [001] and strain $\perp$ [001]
$Pm'n'2_1$ (31.127) $\mathbb{Z}_1$	• $\boldsymbol{E}$ || [001] and $\boldsymbol{B}$ || [001]
$Pnn'm'$ (58.398) $\mathbb{Z}_2$	• $\boldsymbol{B}$ || [001]

Continued on next page

…continued

Subgroup & SI group	Perturbation
P_b2_1 (4.11) $\mathbb{Z}_1$	• $\boldsymbol{E} \parallel [001]$ and strain $\perp [001]$
P_b2_1/c (14.81) $\mathbb{Z}_2$	• strain $\perp [001]$
P_cca2_1 (29.106) $\mathbb{Z}_1$	• $\boldsymbol{E} \parallel [001]$

Table. S 539: Subgroups of P_Abcn (60.429) that can be realized by applying $\boldsymbol{E}/\boldsymbol{B}$ fields and/or uniaxial strains.

Subgroup & SI group	Perturbation
P_S1 (1.3) $\mathbb{Z}_1$	• $\boldsymbol{E} \parallel [100]$ and strain in generic direction<br>• $\boldsymbol{E} \parallel [010]$ and strain in generic direction<br>• $\boldsymbol{E} \parallel [001]$ and strain in generic direction<br>• $\boldsymbol{E} \perp [100]$ and strain $\perp [010]$<br>• $\boldsymbol{E} \perp [100]$ and strain $\perp [001]$<br>• $\boldsymbol{E} \perp [100]$ and strain in generic direction<br>• $\boldsymbol{E} \perp [010]$ and strain $\perp [100]$<br>• $\boldsymbol{E} \perp [010]$ and strain $\perp [001]$<br>• $\boldsymbol{E} \perp [010]$ and strain in generic direction<br>• $\boldsymbol{E} \perp [001]$ and strain $\perp [100]$<br>• $\boldsymbol{E} \perp [001]$ and strain $\perp [010]$<br>• $\boldsymbol{E} \perp [001]$ and strain in generic direction<br>• $\boldsymbol{E}$ in generic direction
Pc (7.24) $\mathbb{Z}_1$	• $\boldsymbol{E} \parallel [100]$ and $\boldsymbol{B} \parallel [010]$ and strain $\perp [010]$<br>• $\boldsymbol{E} \parallel [001]$ and $\boldsymbol{B} \parallel [010]$ and strain $\perp [010]$<br>• $\boldsymbol{E} \perp [010]$ and $\boldsymbol{B} \parallel [010]$
Pm' (6.20) $\mathbb{Z}_1$	• $\boldsymbol{E} \parallel [100]$ and $\boldsymbol{B} \parallel [100]$ and strain $\perp [010]$<br>• $\boldsymbol{E} \parallel [100]$ and $\boldsymbol{B} \parallel [001]$ and strain $\perp [010]$<br>• $\boldsymbol{E} \parallel [100]$ and $\boldsymbol{B} \perp [010]$<br>• $\boldsymbol{E} \parallel [001]$ and $\boldsymbol{B} \parallel [100]$ and strain $\perp [010]$<br>• $\boldsymbol{E} \parallel [001]$ and $\boldsymbol{B} \parallel [001]$ and strain $\perp [010]$<br>• $\boldsymbol{E} \parallel [001]$ and $\boldsymbol{B} \perp [010]$<br>• $\boldsymbol{E} \perp [010]$ and $\boldsymbol{B} \parallel [100]$<br>• $\boldsymbol{E} \perp [010]$ and $\boldsymbol{B} \parallel [001]$<br>• $\boldsymbol{E} \perp [010]$ and $\boldsymbol{B} \perp [010]$

Continued on next page

. . . continued

Subgroup & SI group	Perturbation
$P_A c$ (7.31) $\mathbb{Z}_1$	• $\boldsymbol{E} \parallel [100]$ and strain $\perp [010]$<br>• $\boldsymbol{E} \parallel [001]$ and strain $\perp [010]$<br>• $\boldsymbol{E} \perp [010]$
Pc' (7.26) $\mathbb{Z}_1$	• $\boldsymbol{E} \parallel [100]$ and $\boldsymbol{B} \parallel [100]$ and strain $\perp [001]$<br>• $\boldsymbol{E} \parallel [100]$ and $\boldsymbol{B} \parallel [010]$ and strain $\perp [001]$<br>• $\boldsymbol{E} \parallel [100]$ and $\boldsymbol{B} \perp [001]$<br>• $\boldsymbol{E} \parallel [010]$ and $\boldsymbol{B} \parallel [100]$ and strain $\perp [001]$<br>• $\boldsymbol{E} \parallel [010]$ and $\boldsymbol{B} \parallel [010]$ and strain $\perp [001]$<br>• $\boldsymbol{E} \parallel [010]$ and $\boldsymbol{B} \perp [001]$<br>• $\boldsymbol{E} \perp [001]$ and $\boldsymbol{B} \parallel [100]$<br>• $\boldsymbol{E} \perp [001]$ and $\boldsymbol{B} \parallel [010]$<br>• $\boldsymbol{E} \perp [001]$ and $\boldsymbol{B} \perp [001]$
Pc (7.24) $\mathbb{Z}_1$	• $\boldsymbol{E} \parallel [100]$ and $\boldsymbol{B} \parallel [001]$ and strain $\perp [001]$<br>• $\boldsymbol{E} \parallel [010]$ and $\boldsymbol{B} \parallel [001]$ and strain $\perp [001]$<br>• $\boldsymbol{E} \perp [001]$ and $\boldsymbol{B} \parallel [001]$
$P_C c$ (7.30) $\mathbb{Z}_1$	• $\boldsymbol{E} \parallel [100]$ and strain $\perp [001]$<br>• $\boldsymbol{E} \parallel [010]$ and strain $\perp [001]$<br>• $\boldsymbol{E} \perp [001]$
$P2_1'$ (4.9) $\mathbb{Z}_1$	• $\boldsymbol{E} \parallel [100]$ and $\boldsymbol{B} \parallel [010]$ and strain $\perp [100]$<br>• $\boldsymbol{E} \parallel [100]$ and $\boldsymbol{B} \parallel [001]$ and strain $\perp [100]$<br>• $\boldsymbol{E} \parallel [100]$ and $\boldsymbol{B} \perp [100]$
$Pc'a2_1'$ (29.101) $\mathbb{Z}_1$	• $\boldsymbol{E} \parallel [100]$ and $\boldsymbol{B} \parallel [010]$
$Pm'n2_1'$ (31.125) $\mathbb{Z}_1$	• $\boldsymbol{E} \parallel [100]$ and $\boldsymbol{B} \parallel [001]$
$P2_1$ (4.7) $\mathbb{Z}_1$	• $\boldsymbol{E} \parallel [100]$ and $\boldsymbol{B} \parallel [100]$ and strain $\perp [100]$
$Pm'c'2_1$ (26.70) $\mathbb{Z}_1$	• $\boldsymbol{E} \parallel [100]$ and $\boldsymbol{B} \parallel [100]$
$P_a 2_1$ (4.10) $\mathbb{Z}_1$	• $\boldsymbol{E} \parallel [100]$ and strain $\perp [100]$
$P_C na2_1$ (33.154) $\mathbb{Z}_1$	• $\boldsymbol{E} \parallel [100]$
$P2$ (3.1) $\mathbb{Z}_2$	• $\boldsymbol{E} \parallel [010]$ and $\boldsymbol{B} \parallel [010]$ and strain $\perp [010]$
$P2_1'$ (4.9) $\mathbb{Z}_1$	• $\boldsymbol{E} \parallel [010]$ and $\boldsymbol{B} \parallel [100]$ and strain $\perp [010]$<br>• $\boldsymbol{E} \parallel [010]$ and $\boldsymbol{B} \parallel [001]$ and strain $\perp [010]$<br>• $\boldsymbol{E} \parallel [010]$ and $\boldsymbol{B} \perp [010]$
$P_C 2$ (3.6) $\mathbb{Z}_1$	• $\boldsymbol{E} \parallel [010]$ and strain $\perp [010]$

Continued on next page

...continued

Subgroup & SI group	Perturbation
$P\bar{1}$ (2.4) $\mathbb{Z}_2 \times \mathbb{Z}_2 \times \mathbb{Z}_2 \times \mathbb{Z}_4$	• $\boldsymbol{B} \parallel [100]$ and strain in generic direction<br>• $\boldsymbol{B} \parallel [010]$ and strain in generic direction<br>• $\boldsymbol{B} \parallel [001]$ and strain in generic direction<br>• $\boldsymbol{B} \perp [100]$ and strain $\perp [010]$<br>• $\boldsymbol{B} \perp [100]$ and strain $\perp [001]$<br>• $\boldsymbol{B} \perp [100]$ and strain in generic direction<br>• $\boldsymbol{B} \perp [010]$ and strain $\perp [100]$<br>• $\boldsymbol{B} \perp [010]$ and strain $\perp [001]$<br>• $\boldsymbol{B} \perp [010]$ and strain in generic direction<br>• $\boldsymbol{B} \perp [001]$ and strain $\perp [100]$<br>• $\boldsymbol{B} \perp [001]$ and strain $\perp [010]$<br>• $\boldsymbol{B} \perp [001]$ and strain in generic direction<br>• $\boldsymbol{B}$ in generic direction
$P2/c$ (13.65) $\mathbb{Z}_2 \times \mathbb{Z}_2$	• $\boldsymbol{B} \parallel [010]$ and strain $\perp [010]$
$P2_1'/m'$ (11.54) $\mathbb{Z}_2 \times \mathbb{Z}_2 \times \mathbb{Z}_4$	• $\boldsymbol{B} \parallel [100]$ and strain $\perp [010]$<br>• $\boldsymbol{B} \parallel [001]$ and strain $\perp [010]$<br>• $\boldsymbol{B} \perp [010]$
$P_S\bar{1}$ (2.7) $\mathbb{Z}_2 \times \mathbb{Z}_2 \times \mathbb{Z}_4$	• strain in generic direction
P_A2/c (13.73) $\mathbb{Z}_2$	• strain $\perp [010]$
Pc' (7.26) $\mathbb{Z}_1$	• $\boldsymbol{E} \parallel [010]$ and $\boldsymbol{B} \parallel [010]$ and strain $\perp [100]$<br>• $\boldsymbol{E} \parallel [010]$ and $\boldsymbol{B} \parallel [001]$ and strain $\perp [100]$<br>• $\boldsymbol{E} \parallel [010]$ and $\boldsymbol{B} \perp [100]$<br>• $\boldsymbol{E} \parallel [001]$ and $\boldsymbol{B} \parallel [010]$ and strain $\perp [100]$<br>• $\boldsymbol{E} \parallel [001]$ and $\boldsymbol{B} \parallel [001]$ and strain $\perp [100]$<br>• $\boldsymbol{E} \parallel [001]$ and $\boldsymbol{B} \perp [100]$<br>• $\boldsymbol{E} \perp [100]$ and $\boldsymbol{B} \parallel [010]$<br>• $\boldsymbol{E} \perp [100]$ and $\boldsymbol{B} \parallel [001]$<br>• $\boldsymbol{E} \perp [100]$ and $\boldsymbol{B} \perp [100]$
$Pb'a'2$ (32.138) $\mathbb{Z}_2$	• $\boldsymbol{E} \parallel [010]$ and $\boldsymbol{B} \parallel [010]$
$Pna'2_1'$ (33.147) $\mathbb{Z}_1$	• $\boldsymbol{E} \parallel [010]$ and $\boldsymbol{B} \parallel [001]$
$P2_1'/c'$ (14.79) $\mathbb{Z}_2 \times \mathbb{Z}_4$	• $\boldsymbol{B} \parallel [010]$ and strain $\perp [100]$<br>• $\boldsymbol{B} \parallel [001]$ and strain $\perp [100]$<br>• $\boldsymbol{B} \perp [100]$
Pc (7.24) $\mathbb{Z}_1$	• $\boldsymbol{E} \parallel [010]$ and $\boldsymbol{B} \parallel [100]$ and strain $\perp [100]$<br>• $\boldsymbol{E} \parallel [001]$ and $\boldsymbol{B} \parallel [100]$ and strain $\perp [100]$<br>• $\boldsymbol{E} \perp [100]$ and $\boldsymbol{B} \parallel [100]$

Continued on next page

. . . continued

Subgroup & SI group	Perturbation
$Pca'2_1'$ (29.102) $\mathbb{Z}_1$	• $\boldsymbol{E} \parallel [010]$ and $\boldsymbol{B} \parallel [100]$
$P2_1/c$ (14.75) $\mathbb{Z}_2$	• $\boldsymbol{B} \parallel [100]$ and strain $\perp [100]$
P_ac (7.27) $\mathbb{Z}_1$	• $\boldsymbol{E} \parallel [010]$ and strain $\perp [100]$<br>• $\boldsymbol{E} \parallel [001]$ and strain $\perp [100]$<br>• $\boldsymbol{E} \perp [100]$
P_Bnc2 (30.120) $\mathbb{Z}_2$	• $\boldsymbol{E} \parallel [010]$
P_a2_1/c (14.80) $\mathbb{Z}_2$	• strain $\perp [100]$
$P2'$ (3.3) $\mathbb{Z}_1$	• $\boldsymbol{E} \parallel [001]$ and $\boldsymbol{B} \parallel [100]$ and strain $\perp [001]$<br>• $\boldsymbol{E} \parallel [001]$ and $\boldsymbol{B} \parallel [010]$ and strain $\perp [001]$<br>• $\boldsymbol{E} \parallel [001]$ and $\boldsymbol{B} \perp [001]$
$P2'/c'$ (13.69) $\mathbb{Z}_2 \times \mathbb{Z}_2 \times \mathbb{Z}_4$	• $\boldsymbol{B} \parallel [100]$ and strain $\perp [001]$<br>• $\boldsymbol{B} \parallel [010]$ and strain $\perp [001]$<br>• $\boldsymbol{B} \perp [001]$
$Pc'c2'$ (27.80) $\mathbb{Z}_1$	• $\boldsymbol{E} \parallel [001]$ and $\boldsymbol{B} \parallel [010]$
$Pc'ca'$ (54.344) $\mathbb{Z}_2 \times \mathbb{Z}_2$	• $\boldsymbol{B} \parallel [010]$
$Pm'a2'$ (28.89) $\mathbb{Z}_1$	• $\boldsymbol{E} \parallel [001]$ and $\boldsymbol{B} \parallel [100]$
$Pb'cm'$ (57.384) $\mathbb{Z}_2$	• $\boldsymbol{B} \parallel [100]$
$P2_1$ (4.7) $\mathbb{Z}_1$	• $\boldsymbol{E} \parallel [001]$ and $\boldsymbol{B} \parallel [001]$ and strain $\perp [001]$
$P2_1/c$ (14.75) $\mathbb{Z}_2$	• $\boldsymbol{B} \parallel [001]$ and strain $\perp [001]$
$Pm'c'2_1$ (26.70) $\mathbb{Z}_1$	• $\boldsymbol{E} \parallel [001]$ and $\boldsymbol{B} \parallel [001]$
$Pnm'a'$ (62.447) $\mathbb{Z}_2$	• $\boldsymbol{B} \parallel [001]$
P_C2_1 (4.12) $\mathbb{Z}_1$	• $\boldsymbol{E} \parallel [001]$ and strain $\perp [001]$
P_C2_1/c (14.84) $\mathbb{Z}_2$	• strain $\perp [001]$
P_Bca2_1 (29.108) $\mathbb{Z}_1$	• $\boldsymbol{E} \parallel [001]$

Table. S 540: Subgroups of P_Bbcn (60.430) that can be realized by applying $\boldsymbol{E}/\boldsymbol{B}$ fields and/or uniaxial strains.

Subgroup & SI group	Perturbation

Continued on next page

...continued

Subgroup & SI group	Perturbation
Pc' (7.26) $\mathbb{Z}_1$	• $\boldsymbol{E} \parallel [100]$ and $\boldsymbol{B} \parallel [100]$ and strain $\perp [001]$<br>• $\boldsymbol{E} \parallel [100]$ and $\boldsymbol{B} \parallel [010]$ and strain $\perp [001]$<br>• $\boldsymbol{E} \parallel [100]$ and $\boldsymbol{B} \perp [001]$<br>• $\boldsymbol{E} \parallel [010]$ and $\boldsymbol{B} \parallel [100]$ and strain $\perp [001]$<br>• $\boldsymbol{E} \parallel [010]$ and $\boldsymbol{B} \parallel [010]$ and strain $\perp [001]$<br>• $\boldsymbol{E} \parallel [010]$ and $\boldsymbol{B} \perp [001]$<br>• $\boldsymbol{E} \perp [001]$ and $\boldsymbol{B} \parallel [100]$<br>• $\boldsymbol{E} \perp [001]$ and $\boldsymbol{B} \parallel [010]$<br>• $\boldsymbol{E} \perp [001]$ and $\boldsymbol{B} \perp [001]$
Pc (7.24) $\mathbb{Z}_1$	• $\boldsymbol{E} \parallel [100]$ and $\boldsymbol{B} \parallel [010]$ and strain $\perp [010]$<br>• $\boldsymbol{E} \parallel [001]$ and $\boldsymbol{B} \parallel [010]$ and strain $\perp [010]$<br>• $\boldsymbol{E} \perp [010]$ and $\boldsymbol{B} \parallel [010]$
$P2'$ (3.3) $\mathbb{Z}_1$	• $\boldsymbol{E} \parallel [100]$ and $\boldsymbol{B} \parallel [010]$ and strain $\perp [100]$<br>• $\boldsymbol{E} \parallel [100]$ and $\boldsymbol{B} \parallel [001]$ and strain $\perp [100]$<br>• $\boldsymbol{E} \parallel [100]$ and $\boldsymbol{B} \perp [100]$
$Pb'a2'$ (32.137) $\mathbb{Z}_1$	• $\boldsymbol{E} \parallel [100]$ and $\boldsymbol{B} \parallel [010]$
P_S1 (1.3) $\mathbb{Z}_1$	• $\boldsymbol{E} \parallel [100]$ and strain in generic direction<br>• $\boldsymbol{E} \parallel [010]$ and strain in generic direction<br>• $\boldsymbol{E} \parallel [001]$ and strain in generic direction<br>• $\boldsymbol{E} \perp [100]$ and strain $\perp [010]$<br>• $\boldsymbol{E} \perp [100]$ and strain $\perp [001]$<br>• $\boldsymbol{E} \perp [100]$ and strain in generic direction<br>• $\boldsymbol{E} \perp [010]$ and strain $\perp [100]$<br>• $\boldsymbol{E} \perp [010]$ and strain $\perp [001]$<br>• $\boldsymbol{E} \perp [010]$ and strain in generic direction<br>• $\boldsymbol{E} \perp [001]$ and strain $\perp [100]$<br>• $\boldsymbol{E} \perp [001]$ and strain $\perp [010]$<br>• $\boldsymbol{E} \perp [001]$ and strain in generic direction<br>• $\boldsymbol{E}$ in generic direction
Pc (7.24) $\mathbb{Z}_1$	• $\boldsymbol{E} \parallel [100]$ and $\boldsymbol{B} \parallel [001]$ and strain $\perp [001]$<br>• $\boldsymbol{E} \parallel [010]$ and $\boldsymbol{B} \parallel [001]$ and strain $\perp [001]$<br>• $\boldsymbol{E} \perp [001]$ and $\boldsymbol{B} \parallel [001]$
P_Cc (7.30) $\mathbb{Z}_1$	• $\boldsymbol{E} \parallel [100]$ and strain $\perp [001]$<br>• $\boldsymbol{E} \parallel [010]$ and strain $\perp [001]$<br>• $\boldsymbol{E} \perp [001]$

Continued on next page

...continued

Subgroup & SI group	Perturbation
Pc' (7.26) $\mathbb{Z}_1$	• $\boldsymbol{E}$ ∥ [100] and $\boldsymbol{B}$ ∥ [100] and strain ⊥ [010]<br>• $\boldsymbol{E}$ ∥ [100] and $\boldsymbol{B}$ ∥ [001] and strain ⊥ [010]<br>• $\boldsymbol{E}$ ∥ [100] and $\boldsymbol{B}$ ⊥ [010]<br>• $\boldsymbol{E}$ ∥ [001] and $\boldsymbol{B}$ ∥ [100] and strain ⊥ [010]<br>• $\boldsymbol{E}$ ∥ [001] and $\boldsymbol{B}$ ∥ [001] and strain ⊥ [010]<br>• $\boldsymbol{E}$ ∥ [001] and $\boldsymbol{B}$ ⊥ [010]<br>• $\boldsymbol{E}$ ⊥ [010] and $\boldsymbol{B}$ ∥ [100]<br>• $\boldsymbol{E}$ ⊥ [010] and $\boldsymbol{B}$ ∥ [001]<br>• $\boldsymbol{E}$ ⊥ [010] and $\boldsymbol{B}$ ⊥ [010]
P_ac (7.27) $\mathbb{Z}_1$	• $\boldsymbol{E}$ ∥ [100] and strain ⊥ [010]<br>• $\boldsymbol{E}$ ∥ [001] and strain ⊥ [010]<br>• $\boldsymbol{E}$ ⊥ [010]
$Pnc'2'$ (30.114) $\mathbb{Z}_1$	• $\boldsymbol{E}$ ∥ [100] and $\boldsymbol{B}$ ∥ [001]
$P2_1$ (4.7) $\mathbb{Z}_1$	• $\boldsymbol{E}$ ∥ [100] and $\boldsymbol{B}$ ∥ [100] and strain ⊥ [100]
P_C2_1 (4.12) $\mathbb{Z}_1$	• $\boldsymbol{E}$ ∥ [100] and strain ⊥ [100]
$Pc'a'2_1$ (29.103) $\mathbb{Z}_1$	• $\boldsymbol{E}$ ∥ [100] and $\boldsymbol{B}$ ∥ [100]
P_Bna2_1 (33.153) $\mathbb{Z}_1$	• $\boldsymbol{E}$ ∥ [100]
$P2$ (3.1) $\mathbb{Z}_2$	• $\boldsymbol{E}$ ∥ [010] and $\boldsymbol{B}$ ∥ [010] and strain ⊥ [010]
Pc' (7.26) $\mathbb{Z}_1$	• $\boldsymbol{E}$ ∥ [010] and $\boldsymbol{B}$ ∥ [010] and strain ⊥ [100]<br>• $\boldsymbol{E}$ ∥ [010] and $\boldsymbol{B}$ ∥ [001] and strain ⊥ [100]<br>• $\boldsymbol{E}$ ∥ [010] and $\boldsymbol{B}$ ⊥ [100]<br>• $\boldsymbol{E}$ ∥ [001] and $\boldsymbol{B}$ ∥ [010] and strain ⊥ [100]<br>• $\boldsymbol{E}$ ∥ [001] and $\boldsymbol{B}$ ∥ [001] and strain ⊥ [100]<br>• $\boldsymbol{E}$ ∥ [001] and $\boldsymbol{B}$ ⊥ [100]<br>• $\boldsymbol{E}$ ⊥ [100] and $\boldsymbol{B}$ ∥ [010]<br>• $\boldsymbol{E}$ ⊥ [100] and $\boldsymbol{B}$ ∥ [001]<br>• $\boldsymbol{E}$ ⊥ [100] and $\boldsymbol{B}$ ⊥ [100]
$Pn'c'2$ (30.115) $\mathbb{Z}_1$	• $\boldsymbol{E}$ ∥ [010] and $\boldsymbol{B}$ ∥ [010]

Continued on next page

...continued

Subgroup & SI group	Perturbation
$P\bar{1}$ (2.4) $\mathbb{Z}_2 \times \mathbb{Z}_2 \times \mathbb{Z}_2 \times \mathbb{Z}_4$	• $\boldsymbol{B} \parallel [100]$ and strain in generic direction<br>• $\boldsymbol{B} \parallel [010]$ and strain in generic direction<br>• $\boldsymbol{B} \parallel [001]$ and strain in generic direction<br>• $\boldsymbol{B} \perp [100]$ and strain $\perp [010]$<br>• $\boldsymbol{B} \perp [100]$ and strain $\perp [001]$<br>• $\boldsymbol{B} \perp [100]$ and strain in generic direction<br>• $\boldsymbol{B} \perp [010]$ and strain $\perp [100]$<br>• $\boldsymbol{B} \perp [010]$ and strain $\perp [001]$<br>• $\boldsymbol{B} \perp [010]$ and strain in generic direction<br>• $\boldsymbol{B} \perp [001]$ and strain $\perp [100]$<br>• $\boldsymbol{B} \perp [001]$ and strain $\perp [010]$<br>• $\boldsymbol{B} \perp [001]$ and strain in generic direction<br>• $\boldsymbol{B}$ in generic direction
$P2/c$ (13.65) $\mathbb{Z}_2 \times \mathbb{Z}_2$	• $\boldsymbol{B} \parallel [010]$ and strain $\perp [010]$
$P2'/c'$ (13.69) $\mathbb{Z}_2 \times \mathbb{Z}_2 \times \mathbb{Z}_4$	• $\boldsymbol{B} \parallel [010]$ and strain $\perp [100]$<br>• $\boldsymbol{B} \parallel [001]$ and strain $\perp [100]$<br>• $\boldsymbol{B} \perp [100]$
$P2'$ (3.3) $\mathbb{Z}_1$	• $\boldsymbol{E} \parallel [001]$ and $\boldsymbol{B} \parallel [100]$ and strain $\perp [001]$<br>• $\boldsymbol{E} \parallel [001]$ and $\boldsymbol{B} \parallel [010]$ and strain $\perp [001]$<br>• $\boldsymbol{E} \parallel [001]$ and $\boldsymbol{B} \perp [001]$
$Pn'c2'$ (30.113) $\mathbb{Z}_1$	• $\boldsymbol{E} \parallel [001]$ and $\boldsymbol{B} \parallel [010]$
$P2'/c'$ (13.69) $\mathbb{Z}_2 \times \mathbb{Z}_2 \times \mathbb{Z}_4$	• $\boldsymbol{B} \parallel [100]$ and strain $\perp [001]$<br>• $\boldsymbol{B} \parallel [010]$ and strain $\perp [001]$<br>• $\boldsymbol{B} \perp [001]$
$Pb'an'$ (50.282) $\mathbb{Z}_2$	• $\boldsymbol{B} \parallel [010]$
$P2'$ (3.3) $\mathbb{Z}_1$	• $\boldsymbol{E} \parallel [010]$ and $\boldsymbol{B} \parallel [100]$ and strain $\perp [010]$<br>• $\boldsymbol{E} \parallel [010]$ and $\boldsymbol{B} \parallel [001]$ and strain $\perp [010]$<br>• $\boldsymbol{E} \parallel [010]$ and $\boldsymbol{B} \perp [010]$
P_a2 (3.4) $\mathbb{Z}_2$	• $\boldsymbol{E} \parallel [010]$ and strain $\perp [010]$
$Pn'n2'$ (34.158) $\mathbb{Z}_1$	• $\boldsymbol{E} \parallel [010]$ and $\boldsymbol{B} \parallel [001]$
$P2'/c'$ (13.69) $\mathbb{Z}_2 \times \mathbb{Z}_2 \times \mathbb{Z}_4$	• $\boldsymbol{B} \parallel [100]$ and strain $\perp [010]$<br>• $\boldsymbol{B} \parallel [001]$ and strain $\perp [010]$<br>• $\boldsymbol{B} \perp [010]$
Pc (7.24) $\mathbb{Z}_1$	• $\boldsymbol{E} \parallel [010]$ and $\boldsymbol{B} \parallel [100]$ and strain $\perp [100]$<br>• $\boldsymbol{E} \parallel [001]$ and $\boldsymbol{B} \parallel [100]$ and strain $\perp [100]$<br>• $\boldsymbol{E} \perp [100]$ and $\boldsymbol{B} \parallel [100]$

Continued on next page

. . . continued

Subgroup & SI group	Perturbation
P_Cc (7.30) $\mathbb{Z}_1$	• $\boldsymbol{E} \parallel$ [010] and strain $\perp$ [100]<br>• $\boldsymbol{E} \parallel$ [001] and strain $\perp$ [100]<br>• $\boldsymbol{E} \perp$ [100]
$P2_1/c$ (14.75) $\mathbb{Z}_2$	• $\boldsymbol{B} \parallel$ [100] and strain $\perp$ [100]
$Pb'a2'$ (32.137) $\mathbb{Z}_1$	• $\boldsymbol{E} \parallel$ [001] and $\boldsymbol{B} \parallel$ [100]
$Pc'c2'$ (27.80) $\mathbb{Z}_1$	• $\boldsymbol{E} \parallel$ [010] and $\boldsymbol{B} \parallel$ [100]
P_Cnc2 (30.121) $\mathbb{Z}_1$	• $\boldsymbol{E} \parallel$ [010]
$Pcc'a'$ (54.343) $\mathbb{Z}_2$	• $\boldsymbol{B} \parallel$ [100]
$P_S\bar{1}$ (2.7) $\mathbb{Z}_2 \times \mathbb{Z}_2 \times \mathbb{Z}_4$	• strain in generic direction
P_a2/c (13.70) $\mathbb{Z}_2 \times \mathbb{Z}_2$	• strain $\perp$ [010]
P_C2_1/c (14.84) $\mathbb{Z}_2$	• strain $\perp$ [100]
$P2_1$ (4.7) $\mathbb{Z}_1$	• $\boldsymbol{E} \parallel$ [001] and $\boldsymbol{B} \parallel$ [001] and strain $\perp$ [001]
$Pn'a'2_1$ (33.148) $\mathbb{Z}_1$	• $\boldsymbol{E} \parallel$ [001] and $\boldsymbol{B} \parallel$ [001]
$P2_1/c$ (14.75) $\mathbb{Z}_2$	• $\boldsymbol{B} \parallel$ [001] and strain $\perp$ [001]
P_C2_1 (4.12) $\mathbb{Z}_1$	• $\boldsymbol{E} \parallel$ [001] and strain $\perp$ [001]
$Pn'na'$ (52.312) $\mathbb{Z}_2$	• $\boldsymbol{B} \parallel$ [001]
P_Aca2_1 (29.107) $\mathbb{Z}_1$	• $\boldsymbol{E} \parallel$ [001]
P_C2_1/c (14.84) $\mathbb{Z}_2$	• strain $\perp$ [001]

Table. S 541: Subgroups of P_Cbcn (60.431) that can be realized by applying $\boldsymbol{E}/\boldsymbol{B}$ fields and/or uniaxial strains.

Subgroup & SI group	Perturbation

Continued on next page

…continued

Subgroup & SI group	Perturbation
Pm' (6.20) $\mathbb{Z}_1$	• $\boldsymbol{E} \parallel [100]$ and $\boldsymbol{B} \parallel [100]$ and strain $\perp [001]$<br>• $\boldsymbol{E} \parallel [100]$ and $\boldsymbol{B} \parallel [010]$ and strain $\perp [001]$<br>• $\boldsymbol{E} \parallel [100]$ and $\boldsymbol{B} \perp [001]$<br>• $\boldsymbol{E} \parallel [010]$ and $\boldsymbol{B} \parallel [100]$ and strain $\perp [001]$<br>• $\boldsymbol{E} \parallel [010]$ and $\boldsymbol{B} \parallel [010]$ and strain $\perp [001]$<br>• $\boldsymbol{E} \parallel [010]$ and $\boldsymbol{B} \perp [001]$<br>• $\boldsymbol{E} \perp [001]$ and $\boldsymbol{B} \parallel [100]$<br>• $\boldsymbol{E} \perp [001]$ and $\boldsymbol{B} \parallel [010]$<br>• $\boldsymbol{E} \perp [001]$ and $\boldsymbol{B} \perp [001]$
Pc (7.24) $\mathbb{Z}_1$	• $\boldsymbol{E} \parallel [100]$ and $\boldsymbol{B} \parallel [010]$ and strain $\perp [010]$<br>• $\boldsymbol{E} \parallel [001]$ and $\boldsymbol{B} \parallel [010]$ and strain $\perp [010]$<br>• $\boldsymbol{E} \perp [010]$ and $\boldsymbol{B} \parallel [010]$
$P2'$ (3.3) $\mathbb{Z}_1$	• $\boldsymbol{E} \parallel [100]$ and $\boldsymbol{B} \parallel [010]$ and strain $\perp [100]$<br>• $\boldsymbol{E} \parallel [100]$ and $\boldsymbol{B} \parallel [001]$ and strain $\perp [100]$<br>• $\boldsymbol{E} \parallel [100]$ and $\boldsymbol{B} \perp [100]$
$Pm'a2'$ (28.89) $\mathbb{Z}_1$	• $\boldsymbol{E} \parallel [100]$ and $\boldsymbol{B} \parallel [010]$
P_S1 (1.3) $\mathbb{Z}_1$	• $\boldsymbol{E} \parallel [100]$ and strain in generic direction<br>• $\boldsymbol{E} \parallel [010]$ and strain in generic direction<br>• $\boldsymbol{E} \parallel [001]$ and strain in generic direction<br>• $\boldsymbol{E} \perp [100]$ and strain $\perp [010]$<br>• $\boldsymbol{E} \perp [100]$ and strain $\perp [001]$<br>• $\boldsymbol{E} \perp [100]$ and strain in generic direction<br>• $\boldsymbol{E} \perp [010]$ and strain $\perp [100]$<br>• $\boldsymbol{E} \perp [010]$ and strain $\perp [001]$<br>• $\boldsymbol{E} \perp [010]$ and strain in generic direction<br>• $\boldsymbol{E} \perp [001]$ and strain $\perp [100]$<br>• $\boldsymbol{E} \perp [001]$ and strain $\perp [010]$<br>• $\boldsymbol{E} \perp [001]$ and strain in generic direction<br>• $\boldsymbol{E}$ in generic direction
Pc (7.24) $\mathbb{Z}_1$	• $\boldsymbol{E} \parallel [100]$ and $\boldsymbol{B} \parallel [001]$ and strain $\perp [001]$<br>• $\boldsymbol{E} \parallel [010]$ and $\boldsymbol{B} \parallel [001]$ and strain $\perp [001]$<br>• $\boldsymbol{E} \perp [001]$ and $\boldsymbol{B} \parallel [001]$
P_cc (7.28) $\mathbb{Z}_1$	• $\boldsymbol{E} \parallel [100]$ and strain $\perp [001]$<br>• $\boldsymbol{E} \parallel [010]$ and strain $\perp [001]$<br>• $\boldsymbol{E} \perp [001]$

Continued on next page

…continued

Subgroup & SI group	Perturbation
Pc' (7.26) $\mathbb{Z}_1$	• $\boldsymbol{E}$ ∥ [100] and $\boldsymbol{B}$ ∥ [100] and strain ⊥ [010]<br>• $\boldsymbol{E}$ ∥ [100] and $\boldsymbol{B}$ ∥ [001] and strain ⊥ [010]<br>• $\boldsymbol{E}$ ∥ [100] and $\boldsymbol{B}$ ⊥ [010]<br>• $\boldsymbol{E}$ ∥ [001] and $\boldsymbol{B}$ ∥ [100] and strain ⊥ [010]<br>• $\boldsymbol{E}$ ∥ [001] and $\boldsymbol{B}$ ∥ [001] and strain ⊥ [010]<br>• $\boldsymbol{E}$ ∥ [001] and $\boldsymbol{B}$ ⊥ [010]<br>• $\boldsymbol{E}$ ⊥ [010] and $\boldsymbol{B}$ ∥ [100]<br>• $\boldsymbol{E}$ ⊥ [010] and $\boldsymbol{B}$ ∥ [001]<br>• $\boldsymbol{E}$ ⊥ [010] and $\boldsymbol{B}$ ⊥ [010]
P_Cc (7.30) $\mathbb{Z}_1$	• $\boldsymbol{E}$ ∥ [100] and strain ⊥ [010]<br>• $\boldsymbol{E}$ ∥ [001] and strain ⊥ [010]<br>• $\boldsymbol{E}$ ⊥ [010]
$Pn'n2'$ (34.158) $\mathbb{Z}_1$	• $\boldsymbol{E}$ ∥ [100] and $\boldsymbol{B}$ ∥ [001]
$P2_1$ (4.7) $\mathbb{Z}_1$	• $\boldsymbol{E}$ ∥ [100] and $\boldsymbol{B}$ ∥ [100] and strain ⊥ [100]
P_C2_1 (4.12) $\mathbb{Z}_1$	• $\boldsymbol{E}$ ∥ [100] and strain ⊥ [100]
$Pm'n'2_1$ (31.127) $\mathbb{Z}_1$	• $\boldsymbol{E}$ ∥ [100] and $\boldsymbol{B}$ ∥ [100]
P_Ana2_1 (33.152) $\mathbb{Z}_1$	• $\boldsymbol{E}$ ∥ [100]
Pm' (6.20) $\mathbb{Z}_1$	• $\boldsymbol{E}$ ∥ [010] and $\boldsymbol{B}$ ∥ [010] and strain ⊥ [100]<br>• $\boldsymbol{E}$ ∥ [010] and $\boldsymbol{B}$ ∥ [001] and strain ⊥ [100]<br>• $\boldsymbol{E}$ ∥ [010] and $\boldsymbol{B}$ ⊥ [100]<br>• $\boldsymbol{E}$ ∥ [001] and $\boldsymbol{B}$ ∥ [010] and strain ⊥ [100]<br>• $\boldsymbol{E}$ ∥ [001] and $\boldsymbol{B}$ ∥ [001] and strain ⊥ [100]<br>• $\boldsymbol{E}$ ∥ [001] and $\boldsymbol{B}$ ⊥ [100]<br>• $\boldsymbol{E}$ ⊥ [100] and $\boldsymbol{B}$ ∥ [010]<br>• $\boldsymbol{E}$ ⊥ [100] and $\boldsymbol{B}$ ∥ [001]<br>• $\boldsymbol{E}$ ⊥ [100] and $\boldsymbol{B}$ ⊥ [100]
$P2$ (3.1) $\mathbb{Z}_2$	• $\boldsymbol{E}$ ∥ [010] and $\boldsymbol{B}$ ∥ [010] and strain ⊥ [010]
$Pm'm'2$ (25.60) $\mathbb{Z}_2$	• $\boldsymbol{E}$ ∥ [010] and $\boldsymbol{B}$ ∥ [010]
$P2_1'$ (4.9) $\mathbb{Z}_1$	• $\boldsymbol{E}$ ∥ [001] and $\boldsymbol{B}$ ∥ [100] and strain ⊥ [001]<br>• $\boldsymbol{E}$ ∥ [001] and $\boldsymbol{B}$ ∥ [010] and strain ⊥ [001]<br>• $\boldsymbol{E}$ ∥ [001] and $\boldsymbol{B}$ ⊥ [001]
$Pm'c2_1'$ (26.68) $\mathbb{Z}_1$	• $\boldsymbol{E}$ ∥ [001] and $\boldsymbol{B}$ ∥ [010]

Continued on next page

...continued

Subgroup & SI group	Perturbation
$P\bar{1}$ (2.4) $\mathbb{Z}_2 \times \mathbb{Z}_2 \times \mathbb{Z}_2 \times \mathbb{Z}_4$	• $\boldsymbol{B} \parallel [100]$ and strain in generic direction<br>• $\boldsymbol{B} \parallel [010]$ and strain in generic direction<br>• $\boldsymbol{B} \parallel [001]$ and strain in generic direction<br>• $\boldsymbol{B} \perp [100]$ and strain $\perp [010]$<br>• $\boldsymbol{B} \perp [100]$ and strain $\perp [001]$<br>• $\boldsymbol{B} \perp [100]$ and strain in generic direction<br>• $\boldsymbol{B} \perp [010]$ and strain $\perp [100]$<br>• $\boldsymbol{B} \perp [010]$ and strain $\perp [001]$<br>• $\boldsymbol{B} \perp [010]$ and strain in generic direction<br>• $\boldsymbol{B} \perp [001]$ and strain $\perp [100]$<br>• $\boldsymbol{B} \perp [001]$ and strain $\perp [010]$<br>• $\boldsymbol{B} \perp [001]$ and strain in generic direction<br>• $\boldsymbol{B}$ in generic direction
$P2'/m'$ (10.46) $\mathbb{Z}_2 \times \mathbb{Z}_2 \times \mathbb{Z}_2 \times \mathbb{Z}_4$	• $\boldsymbol{B} \parallel [010]$ and strain $\perp [100]$<br>• $\boldsymbol{B} \parallel [001]$ and strain $\perp [100]$<br>• $\boldsymbol{B} \perp [100]$
$P2/c$ (13.65) $\mathbb{Z}_2 \times \mathbb{Z}_2$	• $\boldsymbol{B} \parallel [010]$ and strain $\perp [010]$
$P2_1'/m'$ (11.54) $\mathbb{Z}_2 \times \mathbb{Z}_2 \times \mathbb{Z}_4$	• $\boldsymbol{B} \parallel [100]$ and strain $\perp [001]$<br>• $\boldsymbol{B} \parallel [010]$ and strain $\perp [001]$<br>• $\boldsymbol{B} \perp [001]$
$Pm'm'a$ (51.294) $\mathbb{Z}_2 \times \mathbb{Z}_2$	• $\boldsymbol{B} \parallel [010]$
Pc (7.24) $\mathbb{Z}_1$	• $\boldsymbol{E} \parallel [010]$ and $\boldsymbol{B} \parallel [100]$ and strain $\perp [100]$<br>• $\boldsymbol{E} \parallel [001]$ and $\boldsymbol{B} \parallel [100]$ and strain $\perp [100]$<br>• $\boldsymbol{E} \perp [100]$ and $\boldsymbol{B} \parallel [100]$
P_Ac (7.31) $\mathbb{Z}_1$	• $\boldsymbol{E} \parallel [010]$ and strain $\perp [100]$<br>• $\boldsymbol{E} \parallel [001]$ and strain $\perp [100]$<br>• $\boldsymbol{E} \perp [100]$
$Pn'a2_1'$ (33.146) $\mathbb{Z}_1$	• $\boldsymbol{E} \parallel [001]$ and $\boldsymbol{B} \parallel [100]$
$P2_1/c$ (14.75) $\mathbb{Z}_2$	• $\boldsymbol{B} \parallel [100]$ and strain $\perp [100]$
$P2_1'$ (4.9) $\mathbb{Z}_1$	• $\boldsymbol{E} \parallel [010]$ and $\boldsymbol{B} \parallel [100]$ and strain $\perp [010]$<br>• $\boldsymbol{E} \parallel [010]$ and $\boldsymbol{B} \parallel [001]$ and strain $\perp [010]$<br>• $\boldsymbol{E} \parallel [010]$ and $\boldsymbol{B} \perp [010]$
$Pm'n2_1'$ (31.125) $\mathbb{Z}_1$	• $\boldsymbol{E} \parallel [010]$ and $\boldsymbol{B} \parallel [001]$
P_C2 (3.6) $\mathbb{Z}_1$	• $\boldsymbol{E} \parallel [010]$ and strain $\perp [010]$

Continued on next page

...continued

Subgroup & SI group	Perturbation
$P2_1'/c'$ (14.79) $\mathbb{Z}_2\times\mathbb{Z}_4$	• $\boldsymbol{B}\parallel[100]$ and strain $\perp[010]$<br>• $\boldsymbol{B}\parallel[001]$ and strain $\perp[010]$<br>• $\boldsymbol{B}\perp[010]$
$Pm'c2_1'$ (26.68) $\mathbb{Z}_1$	• $\boldsymbol{E}\parallel[010]$ and $\boldsymbol{B}\parallel[100]$
P_Anc2 (30.119) $\mathbb{Z}_1$	• $\boldsymbol{E}\parallel[010]$
$Pn'm'a$ (62.446) $\mathbb{Z}_2$	• $\boldsymbol{B}\parallel[100]$
$P2_1$ (4.7) $\mathbb{Z}_1$	• $\boldsymbol{E}\parallel[001]$ and $\boldsymbol{B}\parallel[001]$ and strain $\perp[001]$
$Pm'n'2_1$ (31.127) $\mathbb{Z}_1$	• $\boldsymbol{E}\parallel[001]$ and $\boldsymbol{B}\parallel[001]$
P_a2_1 (4.10) $\mathbb{Z}_1$	• $\boldsymbol{E}\parallel[001]$ and strain $\perp[001]$
$P2_1/c$ (14.75) $\mathbb{Z}_2$	• $\boldsymbol{B}\parallel[001]$ and strain $\perp[001]$
P_Cca2_1 (29.109) $\mathbb{Z}_1$	• $\boldsymbol{E}\parallel[001]$
$Pnn'm'$ (58.398) $\mathbb{Z}_2$	• $\boldsymbol{B}\parallel[001]$
$P_S\bar{1}$ (2.7) $\mathbb{Z}_2\times\mathbb{Z}_2\times\mathbb{Z}_4$	• strain in generic direction
P_A2_1/c (14.83) $\mathbb{Z}_2$	• strain $\perp[100]$
P_C2/c (13.74) $\mathbb{Z}_2$	• strain $\perp[010]$
P_c2_1/c (14.82) $\mathbb{Z}_2$	• strain $\perp[001]$

Table. S 542: Subgroups of P_Ibcn (60.432) that can be realized by applying $\boldsymbol{E}/\boldsymbol{B}$ fields and/or uniaxial strains.

Subgroup & SI group	Perturbation
Pm' (6.20) $\mathbb{Z}_1$	• $\boldsymbol{E}\parallel[100]$ and $\boldsymbol{B}\parallel[100]$ and strain $\perp[001]$<br>• $\boldsymbol{E}\parallel[100]$ and $\boldsymbol{B}\parallel[010]$ and strain $\perp[001]$<br>• $\boldsymbol{E}\parallel[100]$ and $\boldsymbol{B}\perp[001]$<br>• $\boldsymbol{E}\parallel[010]$ and $\boldsymbol{B}\parallel[100]$ and strain $\perp[001]$<br>• $\boldsymbol{E}\parallel[010]$ and $\boldsymbol{B}\parallel[010]$ and strain $\perp[001]$<br>• $\boldsymbol{E}\parallel[010]$ and $\boldsymbol{B}\perp[001]$<br>• $\boldsymbol{E}\perp[001]$ and $\boldsymbol{B}\parallel[100]$<br>• $\boldsymbol{E}\perp[001]$ and $\boldsymbol{B}\parallel[010]$<br>• $\boldsymbol{E}\perp[001]$ and $\boldsymbol{B}\perp[001]$

Continued on next page

...continued

Subgroup & SI group	Perturbation
Pc (7.24) $\mathbb{Z}_1$	• $\boldsymbol{E} \parallel [100]$ and $\boldsymbol{B} \parallel [010]$ and strain $\perp [010]$<br>• $\boldsymbol{E} \parallel [001]$ and $\boldsymbol{B} \parallel [010]$ and strain $\perp [010]$<br>• $\boldsymbol{E} \perp [010]$ and $\boldsymbol{B} \parallel [010]$
$P2'$ (3.3) $\mathbb{Z}_1$	• $\boldsymbol{E} \parallel [100]$ and $\boldsymbol{B} \parallel [010]$ and strain $\perp [100]$<br>• $\boldsymbol{E} \parallel [100]$ and $\boldsymbol{B} \parallel [001]$ and strain $\perp [100]$<br>• $\boldsymbol{E} \parallel [100]$ and $\boldsymbol{B} \perp [100]$
$Pm'a2'$ (28.89) $\mathbb{Z}_1$	• $\boldsymbol{E} \parallel [100]$ and $\boldsymbol{B} \parallel [010]$
P_S1 (1.3) $\mathbb{Z}_1$	• $\boldsymbol{E} \parallel [100]$ and strain in generic direction<br>• $\boldsymbol{E} \parallel [010]$ and strain in generic direction<br>• $\boldsymbol{E} \parallel [001]$ and strain in generic direction<br>• $\boldsymbol{E} \perp [100]$ and strain $\perp [010]$<br>• $\boldsymbol{E} \perp [100]$ and strain $\perp [001]$<br>• $\boldsymbol{E} \perp [100]$ and strain in generic direction<br>• $\boldsymbol{E} \perp [010]$ and strain $\perp [100]$<br>• $\boldsymbol{E} \perp [010]$ and strain $\perp [001]$<br>• $\boldsymbol{E} \perp [010]$ and strain in generic direction<br>• $\boldsymbol{E} \perp [001]$ and strain $\perp [100]$<br>• $\boldsymbol{E} \perp [001]$ and strain $\perp [010]$<br>• $\boldsymbol{E} \perp [001]$ and strain in generic direction<br>• $\boldsymbol{E}$ in generic direction
Pc (7.24) $\mathbb{Z}_1$	• $\boldsymbol{E} \parallel [100]$ and $\boldsymbol{B} \parallel [001]$ and strain $\perp [001]$<br>• $\boldsymbol{E} \parallel [010]$ and $\boldsymbol{B} \parallel [001]$ and strain $\perp [001]$<br>• $\boldsymbol{E} \perp [001]$ and $\boldsymbol{B} \parallel [001]$
P_Ac (7.31) $\mathbb{Z}_1$	• $\boldsymbol{E} \parallel [100]$ and strain $\perp [001]$<br>• $\boldsymbol{E} \parallel [010]$ and strain $\perp [001]$<br>• $\boldsymbol{E} \perp [001]$
Pc' (7.26) $\mathbb{Z}_1$	• $\boldsymbol{E} \parallel [100]$ and $\boldsymbol{B} \parallel [100]$ and strain $\perp [010]$<br>• $\boldsymbol{E} \parallel [100]$ and $\boldsymbol{B} \parallel [001]$ and strain $\perp [010]$<br>• $\boldsymbol{E} \parallel [100]$ and $\boldsymbol{B} \perp [010]$<br>• $\boldsymbol{E} \parallel [001]$ and $\boldsymbol{B} \parallel [100]$ and strain $\perp [010]$<br>• $\boldsymbol{E} \parallel [001]$ and $\boldsymbol{B} \parallel [001]$ and strain $\perp [010]$<br>• $\boldsymbol{E} \parallel [001]$ and $\boldsymbol{B} \perp [010]$<br>• $\boldsymbol{E} \perp [010]$ and $\boldsymbol{B} \parallel [100]$<br>• $\boldsymbol{E} \perp [010]$ and $\boldsymbol{B} \parallel [001]$<br>• $\boldsymbol{E} \perp [010]$ and $\boldsymbol{B} \perp [010]$
P_Cc (7.30) $\mathbb{Z}_1$	• $\boldsymbol{E} \parallel [100]$ and strain $\perp [010]$<br>• $\boldsymbol{E} \parallel [001]$ and strain $\perp [010]$<br>• $\boldsymbol{E} \perp [010]$

Continued on next page

...continued

Subgroup & SI group	Perturbation
$Pnc'2'$ (30.114) $\mathbb{Z}_1$	• $\boldsymbol{E}$ ∥ [100] and $\boldsymbol{B}$ ∥ [001]
$P2_1$ (4.7) $\mathbb{Z}_1$	• $\boldsymbol{E}$ ∥ [100] and $\boldsymbol{B}$ ∥ [100] and strain ⊥ [100]
P_C2_1 (4.12) $\mathbb{Z}_1$	• $\boldsymbol{E}$ ∥ [100] and strain ⊥ [100]
$Pm'c'2_1$ (26.70) $\mathbb{Z}_1$	• $\boldsymbol{E}$ ∥ [100] and $\boldsymbol{B}$ ∥ [100]
P_Ina2_1 (33.155) $\mathbb{Z}_1$	• $\boldsymbol{E}$ ∥ [100]
Pc' (7.26) $\mathbb{Z}_1$	• $\boldsymbol{E}$ ∥ [010] and $\boldsymbol{B}$ ∥ [010] and strain ⊥ [100]<br>• $\boldsymbol{E}$ ∥ [010] and $\boldsymbol{B}$ ∥ [001] and strain ⊥ [100]<br>• $\boldsymbol{E}$ ∥ [010] and $\boldsymbol{B}$ ⊥ [100]<br>• $\boldsymbol{E}$ ∥ [001] and $\boldsymbol{B}$ ∥ [010] and strain ⊥ [100]<br>• $\boldsymbol{E}$ ∥ [001] and $\boldsymbol{B}$ ∥ [001] and strain ⊥ [100]<br>• $\boldsymbol{E}$ ∥ [001] and $\boldsymbol{B}$ ⊥ [100]<br>• $\boldsymbol{E}$ ⊥ [100] and $\boldsymbol{B}$ ∥ [010]<br>• $\boldsymbol{E}$ ⊥ [100] and $\boldsymbol{B}$ ∥ [001]<br>• $\boldsymbol{E}$ ⊥ [100] and $\boldsymbol{B}$ ⊥ [100]
$P2$ (3.1) $\mathbb{Z}_2$	• $\boldsymbol{E}$ ∥ [010] and $\boldsymbol{B}$ ∥ [010] and strain ⊥ [010]
$Pm'a'2$ (28.91) $\mathbb{Z}_2$	• $\boldsymbol{E}$ ∥ [010] and $\boldsymbol{B}$ ∥ [010]
$P2'$ (3.3) $\mathbb{Z}_1$	• $\boldsymbol{E}$ ∥ [001] and $\boldsymbol{B}$ ∥ [100] and strain ⊥ [001]<br>• $\boldsymbol{E}$ ∥ [001] and $\boldsymbol{B}$ ∥ [010] and strain ⊥ [001]<br>• $\boldsymbol{E}$ ∥ [001] and $\boldsymbol{B}$ ⊥ [001]
$Pc'c2'$ (27.80) $\mathbb{Z}_1$	• $\boldsymbol{E}$ ∥ [001] and $\boldsymbol{B}$ ∥ [010]
$P\bar{1}$ (2.4) $\mathbb{Z}_2 \times \mathbb{Z}_2 \times \mathbb{Z}_2 \times \mathbb{Z}_4$	• $\boldsymbol{B}$ ∥ [100] and strain in generic direction<br>• $\boldsymbol{B}$ ∥ [010] and strain in generic direction<br>• $\boldsymbol{B}$ ∥ [001] and strain in generic direction<br>• $\boldsymbol{B}$ ⊥ [100] and strain ⊥ [010]<br>• $\boldsymbol{B}$ ⊥ [100] and strain ⊥ [001]<br>• $\boldsymbol{B}$ ⊥ [100] and strain in generic direction<br>• $\boldsymbol{B}$ ⊥ [010] and strain ⊥ [100]<br>• $\boldsymbol{B}$ ⊥ [010] and strain ⊥ [001]<br>• $\boldsymbol{B}$ ⊥ [010] and strain in generic direction<br>• $\boldsymbol{B}$ ⊥ [001] and strain ⊥ [100]<br>• $\boldsymbol{B}$ ⊥ [001] and strain ⊥ [010]<br>• $\boldsymbol{B}$ ⊥ [001] and strain in generic direction<br>• $\boldsymbol{B}$ in generic direction
$P2'/c'$ (13.69) $\mathbb{Z}_2 \times \mathbb{Z}_2 \times \mathbb{Z}_4$	• $\boldsymbol{B}$ ∥ [010] and strain ⊥ [100]<br>• $\boldsymbol{B}$ ∥ [001] and strain ⊥ [100]<br>• $\boldsymbol{B}$ ⊥ [100]

Continued on next page

…continued

Subgroup & SI group	Perturbation
$P2/c$ (13.65) $\mathbb{Z}_2 \times \mathbb{Z}_2$	• $\boldsymbol{B} \parallel [010]$ and strain $\perp [010]$
$P2'/m'$ (10.46) $\mathbb{Z}_2 \times \mathbb{Z}_2 \times \mathbb{Z}_2 \times \mathbb{Z}_4$	• $\boldsymbol{B} \parallel [100]$ and strain $\perp [001]$<br>• $\boldsymbol{B} \parallel [010]$ and strain $\perp [001]$<br>• $\boldsymbol{B} \perp [001]$
$Pc'cm'$ (49.270) $\mathbb{Z}_2 \times \mathbb{Z}_2$	• $\boldsymbol{B} \parallel [010]$
Pc (7.24) $\mathbb{Z}_1$	• $\boldsymbol{E} \parallel [010]$ and $\boldsymbol{B} \parallel [100]$ and strain $\perp [100]$<br>• $\boldsymbol{E} \parallel [001]$ and $\boldsymbol{B} \parallel [100]$ and strain $\perp [100]$<br>• $\boldsymbol{E} \perp [100]$ and $\boldsymbol{B} \parallel [100]$
P_Cc (7.30) $\mathbb{Z}_1$	• $\boldsymbol{E} \parallel [010]$ and strain $\perp [100]$<br>• $\boldsymbol{E} \parallel [001]$ and strain $\perp [100]$<br>• $\boldsymbol{E} \perp [100]$
$Pb'a2'$ (32.137) $\mathbb{Z}_1$	• $\boldsymbol{E} \parallel [001]$ and $\boldsymbol{B} \parallel [100]$
$P2_1/c$ (14.75) $\mathbb{Z}_2$	• $\boldsymbol{B} \parallel [100]$ and strain $\perp [100]$
$P2_1'$ (4.9) $\mathbb{Z}_1$	• $\boldsymbol{E} \parallel [010]$ and $\boldsymbol{B} \parallel [100]$ and strain $\perp [010]$<br>• $\boldsymbol{E} \parallel [010]$ and $\boldsymbol{B} \parallel [001]$ and strain $\perp [010]$<br>• $\boldsymbol{E} \parallel [010]$ and $\boldsymbol{B} \perp [010]$
$Pna'2_1'$ (33.147) $\mathbb{Z}_1$	• $\boldsymbol{E} \parallel [010]$ and $\boldsymbol{B} \parallel [001]$
P_C2 (3.6) $\mathbb{Z}_1$	• $\boldsymbol{E} \parallel [010]$ and strain $\perp [010]$
$P2_1'/c'$ (14.79) $\mathbb{Z}_2 \times \mathbb{Z}_4$	• $\boldsymbol{B} \parallel [100]$ and strain $\perp [010]$<br>• $\boldsymbol{B} \parallel [001]$ and strain $\perp [010]$<br>• $\boldsymbol{B} \perp [010]$
$Pm'c2_1'$ (26.68) $\mathbb{Z}_1$	• $\boldsymbol{E} \parallel [010]$ and $\boldsymbol{B} \parallel [100]$
P_Inc2 (30.122) $\mathbb{Z}_1$	• $\boldsymbol{E} \parallel [010]$
$Pb'am'$ (55.358) $\mathbb{Z}_2$	• $\boldsymbol{B} \parallel [100]$
$P2_1$ (4.7) $\mathbb{Z}_1$	• $\boldsymbol{E} \parallel [001]$ and $\boldsymbol{B} \parallel [001]$ and strain $\perp [001]$
$Pc'a'2_1$ (29.103) $\mathbb{Z}_1$	• $\boldsymbol{E} \parallel [001]$ and $\boldsymbol{B} \parallel [001]$
P_C2_1 (4.12) $\mathbb{Z}_1$	• $\boldsymbol{E} \parallel [001]$ and strain $\perp [001]$
$P2_1/c$ (14.75) $\mathbb{Z}_2$	• $\boldsymbol{B} \parallel [001]$ and strain $\perp [001]$
P_Ica2_1 (29.110) $\mathbb{Z}_1$	• $\boldsymbol{E} \parallel [001]$
$Pb'c'n$ (60.422) $\mathbb{Z}_2$	• $\boldsymbol{B} \parallel [001]$
$P_S\bar{1}$ (2.7) $\mathbb{Z}_2 \times \mathbb{Z}_2 \times \mathbb{Z}_4$	• strain in generic direction
P_C2_1/c (14.84) $\mathbb{Z}_2$	• strain $\perp [100]$

Continued on next page

...continued

Subgroup & SI group	Perturbation
P_C2/c (13.74) $\mathbb{Z}_2$	• strain $\perp$ [010]
P_A2_1/c (14.83) $\mathbb{Z}_2$	• strain $\perp$ [001]

Table. S 543: Subgroups of $Pbca$ (61.433) that can be realized by applying $\boldsymbol{E}/\boldsymbol{B}$ fields and/or uniaxial strains.

Subgroup & SI group	Perturbation
Pc (7.24) $\mathbb{Z}_1$	• $\boldsymbol{E} \parallel [100]$ and strain $\perp [010]$<br>• $\boldsymbol{E} \parallel [100]$ and $\boldsymbol{B} \parallel [010]$<br>• $\boldsymbol{E} \parallel [001]$ and strain $\perp [010]$<br>• $\boldsymbol{E} \parallel [001]$ and $\boldsymbol{B} \parallel [010]$<br>• $\boldsymbol{E} \perp [010]$
Pc (7.24) $\mathbb{Z}_1$	• $\boldsymbol{E} \parallel [100]$ and strain $\perp [001]$<br>• $\boldsymbol{E} \parallel [100]$ and $\boldsymbol{B} \parallel [001]$<br>• $\boldsymbol{E} \parallel [010]$ and strain $\perp [001]$<br>• $\boldsymbol{E} \parallel [010]$ and $\boldsymbol{B} \parallel [001]$<br>• $\boldsymbol{E} \perp [001]$
$P2_1$ (4.7) $\mathbb{Z}_1$	• $\boldsymbol{E} \parallel [100]$ and strain $\perp [100]$<br>• $\boldsymbol{E} \parallel [100]$ and $\boldsymbol{B} \parallel [100]$
$Pca2_1$ (29.99) $\mathbb{Z}_1$	• $\boldsymbol{E} \parallel [100]$
$P2_1$ (4.7) $\mathbb{Z}_1$	• $\boldsymbol{E} \parallel [010]$ and strain $\perp [010]$<br>• $\boldsymbol{E} \parallel [010]$ and $\boldsymbol{B} \parallel [010]$
$P\bar{1}$ (2.4) $\mathbb{Z}_2 \times \mathbb{Z}_2 \times \mathbb{Z}_2 \times \mathbb{Z}_4$	• strain in generic direction<br>• $\boldsymbol{B} \parallel [100]$ and strain $\perp [010]$<br>• $\boldsymbol{B} \parallel [100]$ and strain $\perp [001]$<br>• $\boldsymbol{B} \parallel [010]$ and strain $\perp [100]$<br>• $\boldsymbol{B} \parallel [010]$ and strain $\perp [001]$<br>• $\boldsymbol{B} \parallel [001]$ and strain $\perp [100]$<br>• $\boldsymbol{B} \parallel [001]$ and strain $\perp [010]$<br>• $\boldsymbol{B}$ in generic direction
$P2_1/c$ (14.75) $\mathbb{Z}_2$	• strain $\perp$ [010]<br>• $\boldsymbol{B} \parallel [010]$

Continued on next page

...continued

Subgroup & SI group	Perturbation
Pc (7.24) $\mathbb{Z}_1$	• $\boldsymbol{E} \parallel [010]$ and strain $\perp [100]$<br>• $\boldsymbol{E} \parallel [010]$ and $\boldsymbol{B} \parallel [100]$<br>• $\boldsymbol{E} \parallel [001]$ and strain $\perp [100]$<br>• $\boldsymbol{E} \parallel [001]$ and $\boldsymbol{B} \parallel [100]$<br>• $\boldsymbol{E} \perp [100]$
$Pca2_1$ (29.99) $\mathbb{Z}_1$	• $\boldsymbol{E} \parallel [010]$
$P2_1/c$ (14.75) $\mathbb{Z}_2$	• strain $\perp [100]$<br>• $\boldsymbol{B} \parallel [100]$
$P2_1$ (4.7) $\mathbb{Z}_1$	• $\boldsymbol{E} \parallel [001]$ and strain $\perp [001]$<br>• $\boldsymbol{E} \parallel [001]$ and $\boldsymbol{B} \parallel [001]$
$P2_1/c$ (14.75) $\mathbb{Z}_2$	• strain $\perp [001]$<br>• $\boldsymbol{B} \parallel [001]$
$Pca2_1$ (29.99) $\mathbb{Z}_1$	• $\boldsymbol{E} \parallel [001]$

Table. S 544: Subgroups of $Pbca1'$ (61.434) that can be realized by applying $\boldsymbol{E}/\boldsymbol{B}$ fields and/or uniaxial strains.

Subgroup & SI group	Perturbation
$P11'$ (1.2) $\mathbb{Z}_1$	• $\boldsymbol{E} \parallel [100]$ and strain in generic direction<br>• $\boldsymbol{E} \parallel [010]$ and strain in generic direction<br>• $\boldsymbol{E} \parallel [001]$ and strain in generic direction<br>• $\boldsymbol{E} \perp [100]$ and strain $\perp [010]$<br>• $\boldsymbol{E} \perp [100]$ and strain $\perp [001]$<br>• $\boldsymbol{E} \perp [100]$ and strain in generic direction<br>• $\boldsymbol{E} \perp [010]$ and strain $\perp [100]$<br>• $\boldsymbol{E} \perp [010]$ and strain $\perp [001]$<br>• $\boldsymbol{E} \perp [010]$ and strain in generic direction<br>• $\boldsymbol{E} \perp [001]$ and strain $\perp [100]$<br>• $\boldsymbol{E} \perp [001]$ and strain $\perp [010]$<br>• $\boldsymbol{E} \perp [001]$ and strain in generic direction<br>• $\boldsymbol{E}$ in generic direction

Continued on next page

. . . continued

Subgroup & SI group	Perturbation
Pc' (7.26) $\mathbb{Z}_1$	• $\boldsymbol{E}$ ‖ [100] and $\boldsymbol{B}$ ‖ [100] and strain ⊥ [010]<br>• $\boldsymbol{E}$ ‖ [100] and $\boldsymbol{B}$ ‖ [001] and strain ⊥ [010]<br>• $\boldsymbol{E}$ ‖ [100] and $\boldsymbol{B}$ ⊥ [010]<br>• $\boldsymbol{E}$ ‖ [001] and $\boldsymbol{B}$ ‖ [100] and strain ⊥ [010]<br>• $\boldsymbol{E}$ ‖ [001] and $\boldsymbol{B}$ ‖ [001] and strain ⊥ [010]<br>• $\boldsymbol{E}$ ‖ [001] and $\boldsymbol{B}$ ⊥ [010]<br>• $\boldsymbol{E}$ ⊥ [010] and $\boldsymbol{B}$ ‖ [100]<br>• $\boldsymbol{E}$ ⊥ [010] and $\boldsymbol{B}$ ‖ [001]<br>• $\boldsymbol{E}$ ⊥ [010] and $\boldsymbol{B}$ ⊥ [010]
Pc (7.24) $\mathbb{Z}_1$	• $\boldsymbol{E}$ ‖ [100] and $\boldsymbol{B}$ ‖ [010] and strain ⊥ [010]<br>• $\boldsymbol{E}$ ‖ [001] and $\boldsymbol{B}$ ‖ [010] and strain ⊥ [010]<br>• $\boldsymbol{E}$ ⊥ [010] and $\boldsymbol{B}$ ‖ [010]
$Pc1'$ (7.25) $\mathbb{Z}_1$	• $\boldsymbol{E}$ ‖ [100] and strain ⊥ [010]<br>• $\boldsymbol{E}$ ‖ [001] and strain ⊥ [010]<br>• $\boldsymbol{E}$ ⊥ [010]
Pc' (7.26) $\mathbb{Z}_1$	• $\boldsymbol{E}$ ‖ [100] and $\boldsymbol{B}$ ‖ [100] and strain ⊥ [001]<br>• $\boldsymbol{E}$ ‖ [100] and $\boldsymbol{B}$ ‖ [010] and strain ⊥ [001]<br>• $\boldsymbol{E}$ ‖ [100] and $\boldsymbol{B}$ ⊥ [001]<br>• $\boldsymbol{E}$ ‖ [010] and $\boldsymbol{B}$ ‖ [100] and strain ⊥ [001]<br>• $\boldsymbol{E}$ ‖ [010] and $\boldsymbol{B}$ ‖ [010] and strain ⊥ [001]<br>• $\boldsymbol{E}$ ‖ [010] and $\boldsymbol{B}$ ⊥ [001]<br>• $\boldsymbol{E}$ ⊥ [001] and $\boldsymbol{B}$ ‖ [100]<br>• $\boldsymbol{E}$ ⊥ [001] and $\boldsymbol{B}$ ‖ [010]<br>• $\boldsymbol{E}$ ⊥ [001] and $\boldsymbol{B}$ ⊥ [001]
Pc (7.24) $\mathbb{Z}_1$	• $\boldsymbol{E}$ ‖ [100] and $\boldsymbol{B}$ ‖ [001] and strain ⊥ [001]<br>• $\boldsymbol{E}$ ‖ [010] and $\boldsymbol{B}$ ‖ [001] and strain ⊥ [001]<br>• $\boldsymbol{E}$ ⊥ [001] and $\boldsymbol{B}$ ‖ [001]
$Pc1'$ (7.25) $\mathbb{Z}_1$	• $\boldsymbol{E}$ ‖ [100] and strain ⊥ [001]<br>• $\boldsymbol{E}$ ‖ [010] and strain ⊥ [001]<br>• $\boldsymbol{E}$ ⊥ [001]
$P2_1'$ (4.9) $\mathbb{Z}_1$	• $\boldsymbol{E}$ ‖ [100] and $\boldsymbol{B}$ ‖ [010] and strain ⊥ [100]<br>• $\boldsymbol{E}$ ‖ [100] and $\boldsymbol{B}$ ‖ [001] and strain ⊥ [100]<br>• $\boldsymbol{E}$ ‖ [100] and $\boldsymbol{B}$ ⊥ [100]
$Pc'a2_1'$ (29.101) $\mathbb{Z}_1$	• $\boldsymbol{E}$ ‖ [100] and $\boldsymbol{B}$ ‖ [010]
$Pca'2_1'$ (29.102) $\mathbb{Z}_1$	• $\boldsymbol{E}$ ‖ [100] and $\boldsymbol{B}$ ‖ [001]
$P2_1$ (4.7) $\mathbb{Z}_1$	• $\boldsymbol{E}$ ‖ [100] and $\boldsymbol{B}$ ‖ [100] and strain ⊥ [100]

Continued on next page

...continued

Subgroup & SI group	Perturbation
$Pc'a'2_1$ (29.103) $\mathbb{Z}_1$	• $\boldsymbol{E}$ ∥ [100] and $\boldsymbol{B}$ ∥ [100]
$P2_11'$ (4.8) $\mathbb{Z}_1$	• $\boldsymbol{E}$ ∥ [100] and strain ⊥ [100]
$Pca2_11'$ (29.100) $\mathbb{Z}_1$	• $\boldsymbol{E}$ ∥ [100]
$P2_1'$ (4.9) $\mathbb{Z}_1$	• $\boldsymbol{E}$ ∥ [010] and $\boldsymbol{B}$ ∥ [100] and strain ⊥ [010]<br>• $\boldsymbol{E}$ ∥ [010] and $\boldsymbol{B}$ ∥ [001] and strain ⊥ [010]<br>• $\boldsymbol{E}$ ∥ [010] and $\boldsymbol{B}$ ⊥ [010]
$P2_1$ (4.7) $\mathbb{Z}_1$	• $\boldsymbol{E}$ ∥ [010] and $\boldsymbol{B}$ ∥ [010] and strain ⊥ [010]
$P2_11'$ (4.8) $\mathbb{Z}_1$	• $\boldsymbol{E}$ ∥ [010] and strain ⊥ [010]
$P\bar{1}$ (2.4) $\mathbb{Z}_2 \times \mathbb{Z}_2 \times \mathbb{Z}_2 \times \mathbb{Z}_4$	• $\boldsymbol{B}$ ∥ [100] and strain in generic direction<br>• $\boldsymbol{B}$ ∥ [010] and strain in generic direction<br>• $\boldsymbol{B}$ ∥ [001] and strain in generic direction<br>• $\boldsymbol{B}$ ⊥ [100] and strain ⊥ [010]<br>• $\boldsymbol{B}$ ⊥ [100] and strain ⊥ [001]<br>• $\boldsymbol{B}$ ⊥ [100] and strain in generic direction<br>• $\boldsymbol{B}$ ⊥ [010] and strain ⊥ [100]<br>• $\boldsymbol{B}$ ⊥ [010] and strain ⊥ [001]<br>• $\boldsymbol{B}$ ⊥ [010] and strain in generic direction<br>• $\boldsymbol{B}$ ⊥ [001] and strain ⊥ [100]<br>• $\boldsymbol{B}$ ⊥ [001] and strain ⊥ [010]<br>• $\boldsymbol{B}$ ⊥ [001] and strain in generic direction<br>• $\boldsymbol{B}$ in generic direction
$P2_1'/c'$ (14.79) $\mathbb{Z}_2 \times \mathbb{Z}_4$	• $\boldsymbol{B}$ ∥ [100] and strain ⊥ [010]<br>• $\boldsymbol{B}$ ∥ [001] and strain ⊥ [010]<br>• $\boldsymbol{B}$ ⊥ [010]
$P2_1/c$ (14.75) $\mathbb{Z}_2$	• $\boldsymbol{B}$ ∥ [010] and strain ⊥ [010]
$P\bar{1}1'$ (2.5) $\mathbb{Z}_2 \times \mathbb{Z}_2 \times \mathbb{Z}_2 \times \mathbb{Z}_4$	• strain in generic direction
$P2_1/c1'$ (14.76) $\mathbb{Z}_2$	• strain ⊥ [010]
Pc' (7.26) $\mathbb{Z}_1$	• $\boldsymbol{E}$ ∥ [010] and $\boldsymbol{B}$ ∥ [010] and strain ⊥ [100]<br>• $\boldsymbol{E}$ ∥ [010] and $\boldsymbol{B}$ ∥ [001] and strain ⊥ [100]<br>• $\boldsymbol{E}$ ∥ [010] and $\boldsymbol{B}$ ⊥ [100]<br>• $\boldsymbol{E}$ ∥ [001] and $\boldsymbol{B}$ ∥ [010] and strain ⊥ [100]<br>• $\boldsymbol{E}$ ∥ [001] and $\boldsymbol{B}$ ∥ [001] and strain ⊥ [100]<br>• $\boldsymbol{E}$ ∥ [001] and $\boldsymbol{B}$ ⊥ [100]<br>• $\boldsymbol{E}$ ⊥ [100] and $\boldsymbol{B}$ ∥ [010]<br>• $\boldsymbol{E}$ ⊥ [100] and $\boldsymbol{B}$ ∥ [001]<br>• $\boldsymbol{E}$ ⊥ [100] and $\boldsymbol{B}$ ⊥ [100]

Continued on next page

. . . continued

Subgroup & SI group	Perturbation
$Pc'a2_1'$ (29.101) $\mathbb{Z}_1$	• $\boldsymbol{E} \parallel [010]$ and $\boldsymbol{B} \parallel [001]$
$Pc'a'2_1$ (29.103) $\mathbb{Z}_1$	• $\boldsymbol{E} \parallel [010]$ and $\boldsymbol{B} \parallel [010]$
$P2_1'/c'$ (14.79) $\mathbb{Z}_2 \times \mathbb{Z}_4$	• $\boldsymbol{B} \parallel [010]$ and strain $\perp [100]$<br>• $\boldsymbol{B} \parallel [001]$ and strain $\perp [100]$<br>• $\boldsymbol{B} \perp [100]$
Pc (7.24) $\mathbb{Z}_1$	• $\boldsymbol{E} \parallel [010]$ and $\boldsymbol{B} \parallel [100]$ and strain $\perp [100]$<br>• $\boldsymbol{E} \parallel [001]$ and $\boldsymbol{B} \parallel [100]$ and strain $\perp [100]$<br>• $\boldsymbol{E} \perp [100]$ and $\boldsymbol{B} \parallel [100]$
$Pca'2_1'$ (29.102) $\mathbb{Z}_1$	• $\boldsymbol{E} \parallel [010]$ and $\boldsymbol{B} \parallel [100]$
$P2_1/c$ (14.75) $\mathbb{Z}_2$	• $\boldsymbol{B} \parallel [100]$ and strain $\perp [100]$
$Pc1'$ (7.25) $\mathbb{Z}_1$	• $\boldsymbol{E} \parallel [010]$ and strain $\perp [100]$<br>• $\boldsymbol{E} \parallel [001]$ and strain $\perp [100]$<br>• $\boldsymbol{E} \perp [100]$
$Pca2_11'$ (29.100) $\mathbb{Z}_1$	• $\boldsymbol{E} \parallel [010]$
$P2_1/c1'$ (14.76) $\mathbb{Z}_2$	• strain $\perp [100]$
$P2_1'$ (4.9) $\mathbb{Z}_1$	• $\boldsymbol{E} \parallel [001]$ and $\boldsymbol{B} \parallel [100]$ and strain $\perp [001]$<br>• $\boldsymbol{E} \parallel [001]$ and $\boldsymbol{B} \parallel [010]$ and strain $\perp [001]$<br>• $\boldsymbol{E} \parallel [001]$ and $\boldsymbol{B} \perp [001]$
$P2_1'/c'$ (14.79) $\mathbb{Z}_2 \times \mathbb{Z}_4$	• $\boldsymbol{B} \parallel [100]$ and strain $\perp [001]$<br>• $\boldsymbol{B} \parallel [010]$ and strain $\perp [001]$<br>• $\boldsymbol{B} \perp [001]$
$Pca'2_1'$ (29.102) $\mathbb{Z}_1$	• $\boldsymbol{E} \parallel [001]$ and $\boldsymbol{B} \parallel [010]$
$Pb'c'a$ (61.436) $\mathbb{Z}_2$	• $\boldsymbol{B} \parallel [010]$
$Pc'a2_1'$ (29.101) $\mathbb{Z}_1$	• $\boldsymbol{E} \parallel [001]$ and $\boldsymbol{B} \parallel [100]$
$Pb'c'a$ (61.436) $\mathbb{Z}_2$	• $\boldsymbol{B} \parallel [100]$
$P2_1$ (4.7) $\mathbb{Z}_1$	• $\boldsymbol{E} \parallel [001]$ and $\boldsymbol{B} \parallel [001]$ and strain $\perp [001]$
$P2_1/c$ (14.75) $\mathbb{Z}_2$	• $\boldsymbol{B} \parallel [001]$ and strain $\perp [001]$
$Pc'a'2_1$ (29.103) $\mathbb{Z}_1$	• $\boldsymbol{E} \parallel [001]$ and $\boldsymbol{B} \parallel [001]$
$Pb'c'a$ (61.436) $\mathbb{Z}_2$	• $\boldsymbol{B} \parallel [001]$
$P2_11'$ (4.8) $\mathbb{Z}_1$	• $\boldsymbol{E} \parallel [001]$ and strain $\perp [001]$
$P2_1/c1'$ (14.76) $\mathbb{Z}_2$	• strain $\perp [001]$
$Pca2_11'$ (29.100) $\mathbb{Z}_1$	• $\boldsymbol{E} \parallel [001]$

Table. S 545: Subgroups of $Pb'ca$ (61.435) that can be realized by applying $\boldsymbol{E}/\boldsymbol{B}$ fields and/or uniaxial strains.

Subgroup & SI group	Perturbation
Pc (7.24) $\mathbb{Z}_1$	• $\boldsymbol{B} \parallel [010]$ and strain $\perp [010]$<br>• $\boldsymbol{E} \parallel [100]$ and strain $\perp [010]$<br>• $\boldsymbol{E} \parallel [100]$ and $\boldsymbol{B} \parallel [010]$<br>• $\boldsymbol{E} \parallel [001]$ and strain $\perp [010]$<br>• $\boldsymbol{E} \perp [010]$
Pc (7.24) $\mathbb{Z}_1$	• $\boldsymbol{B} \parallel [001]$ and strain $\perp [001]$<br>• $\boldsymbol{E} \parallel [100]$ and strain $\perp [001]$<br>• $\boldsymbol{E} \parallel [100]$ and $\boldsymbol{B} \parallel [001]$<br>• $\boldsymbol{E} \parallel [010]$ and strain $\perp [001]$<br>• $\boldsymbol{E} \perp [001]$
$P2_1$ (4.7) $\mathbb{Z}_1$	• $\boldsymbol{B} \parallel [100]$ and strain $\perp [100]$<br>• $\boldsymbol{E} \parallel [100]$ and strain $\perp [100]$<br>• $\boldsymbol{E} \parallel [100]$ and $\boldsymbol{B} \parallel [100]$
$Pca2_1$ (29.99) $\mathbb{Z}_1$	• $\boldsymbol{E} \parallel [100]$
$P2_1'$ (4.9) $\mathbb{Z}_1$	• $\boldsymbol{B} \parallel [100]$ and strain $\perp [010]$<br>• $\boldsymbol{B} \parallel [001]$ and strain $\perp [010]$<br>• $\boldsymbol{B} \perp [010]$<br>• $\boldsymbol{E} \parallel [010]$ and strain $\perp [010]$<br>• $\boldsymbol{E} \parallel [010]$ and $\boldsymbol{B} \parallel [100]$
$P\bar{1}'$ (2.6) $\mathbb{Z}_1$	• strain in generic direction
$P2_1'/c$ (14.77) $\mathbb{Z}_1$	• strain $\perp [010]$
Pc' (7.26) $\mathbb{Z}_1$	• $\boldsymbol{B} \parallel [010]$ and strain $\perp [100]$<br>• $\boldsymbol{B} \parallel [001]$ and strain $\perp [100]$<br>• $\boldsymbol{B} \perp [100]$<br>• $\boldsymbol{E} \parallel [010]$ and strain $\perp [100]$<br>• $\boldsymbol{E} \parallel [010]$ and $\boldsymbol{B} \parallel [010]$<br>• $\boldsymbol{E} \parallel [001]$ and strain $\perp [100]$<br>• $\boldsymbol{E} \parallel [001]$ and $\boldsymbol{B} \parallel [001]$<br>• $\boldsymbol{E} \perp [100]$
$Pc'a2_1'$ (29.101) $\mathbb{Z}_1$	• $\boldsymbol{B} \parallel [001]$<br>• $\boldsymbol{E} \parallel [010]$
$P2_1/c'$ (14.78) $\mathbb{Z}_1$	• strain $\perp [100]$

Continued on next page

...continued

Subgroup & SI group	Perturbation
$P2_1'$ (4.9) $\mathbb{Z}_1$	• $\boldsymbol{B} \parallel [100]$ and strain $\perp [001]$<br>• $\boldsymbol{B} \parallel [010]$ and strain $\perp [001]$<br>• $\boldsymbol{B} \perp [001]$<br>• $\boldsymbol{E} \parallel [001]$ and strain $\perp [001]$<br>• $\boldsymbol{E} \parallel [001]$ and $\boldsymbol{B} \parallel [100]$
$P2_1'2_1'2_1$ (19.27) $\mathbb{Z}_1$	• $\boldsymbol{B} \parallel [100]$
$P2_1'/c$ (14.77) $\mathbb{Z}_1$	• strain $\perp [001]$
$Pca'2_1'$ (29.102) $\mathbb{Z}_1$	• $\boldsymbol{B} \parallel [010]$<br>• $\boldsymbol{E} \parallel [001]$

Table. S 546: Subgroups of $Pb'c'a$ (61.436) that can be realized by applying $\boldsymbol{E}/\boldsymbol{B}$ fields and/or uniaxial strains.

Subgroup & SI group	Perturbation
Pc' (7.26) $\mathbb{Z}_1$	• $\boldsymbol{E} \parallel [100]$ and strain $\perp [010]$<br>• $\boldsymbol{E} \parallel [100]$ and ($\boldsymbol{B} \parallel [100]$ or $\boldsymbol{B} \perp [010]$)<br>• $\boldsymbol{E} \parallel [001]$ and strain $\perp [010]$<br>• $\boldsymbol{E} \parallel [001]$ and ($\boldsymbol{B} \parallel [100]$ or $\boldsymbol{B} \perp [010]$)<br>• $\boldsymbol{E} \perp [010]$
Pc (7.24) $\mathbb{Z}_1$	• $\boldsymbol{E} \parallel [100]$ and strain $\perp [001]$<br>• $\boldsymbol{E} \parallel [010]$ and strain $\perp [001]$<br>• $\boldsymbol{E} \perp [001]$
$P2_1'$ (4.9) $\mathbb{Z}_1$	• $\boldsymbol{E} \parallel [100]$ and strain $\perp [100]$<br>• $\boldsymbol{E} \parallel [100]$ and ($\boldsymbol{B} \parallel [010]$ or $\boldsymbol{B} \perp [100]$)
$Pca'2_1'$ (29.102) $\mathbb{Z}_1$	• $\boldsymbol{E} \parallel [100]$
$P2_1'$ (4.9) $\mathbb{Z}_1$	• $\boldsymbol{E} \parallel [010]$ and strain $\perp [010]$<br>• $\boldsymbol{E} \parallel [010]$ and ($\boldsymbol{B} \parallel [100]$ or $\boldsymbol{B} \perp [010]$)
$P\bar{1}$ (2.4) $\mathbb{Z}_2 \times \mathbb{Z}_2 \times \mathbb{Z}_2 \times \mathbb{Z}_4$	• strain in generic direction<br>• ($\boldsymbol{B} \parallel [100]$ or $\boldsymbol{B} \perp [010]$) and strain $\perp [100]$<br>• ($\boldsymbol{B} \parallel [100]$ or $\boldsymbol{B} \perp [010]$) and strain $\perp [001]$<br>• ($\boldsymbol{B} \parallel [010]$ or $\boldsymbol{B} \perp [100]$) and strain $\perp [010]$<br>• ($\boldsymbol{B} \parallel [010]$ or $\boldsymbol{B} \perp [100]$) and strain $\perp [001]$<br>• $\boldsymbol{B}$ in generic direction

Continued on next page

…continued

Subgroup & SI group	Perturbation
$P2_1'/c'$ (14.79) $\mathbb{Z}_2\times\mathbb{Z}_4$	• strain $\perp$ [010]<br>• ($\boldsymbol{B}$ $\parallel$ [100] or $\boldsymbol{B}$ $\perp$ [010])
Pc' (7.26) $\mathbb{Z}_1$	• $\boldsymbol{E}$ $\parallel$ [010] and strain $\perp$ [100]<br>• $\boldsymbol{E}$ $\parallel$ [010] and ($\boldsymbol{B}$ $\parallel$ [010] or $\boldsymbol{B}$ $\perp$ [100])<br>• $\boldsymbol{E}$ $\parallel$ [001] and strain $\perp$ [100]<br>• $\boldsymbol{E}$ $\parallel$ [001] and ($\boldsymbol{B}$ $\parallel$ [010] or $\boldsymbol{B}$ $\perp$ [100])<br>• $\boldsymbol{E}$ $\perp$ [100]
$Pc'a2_1'$ (29.101) $\mathbb{Z}_1$	• $\boldsymbol{E}$ $\parallel$ [010]
$P2_1'/c'$ (14.79) $\mathbb{Z}_2\times\mathbb{Z}_4$	• strain $\perp$ [100]<br>• ($\boldsymbol{B}$ $\parallel$ [010] or $\boldsymbol{B}$ $\perp$ [100])
$P2_1$ (4.7) $\mathbb{Z}_1$	• $\boldsymbol{E}$ $\parallel$ [001] and strain $\perp$ [001]
$P2_1/c$ (14.75) $\mathbb{Z}_2$	• strain $\perp$ [001]
$Pc'a'2_1$ (29.103) $\mathbb{Z}_1$	• $\boldsymbol{E}$ $\parallel$ [001]

Table. S 547: Subgroups of $Pb'c'a'$ (61.437) that can be realized by applying $\boldsymbol{E}/\boldsymbol{B}$ fields and/or uniaxial strains.

Subgroup & SI group	Perturbation
Pc' (7.26) $\mathbb{Z}_1$	• $\boldsymbol{B}$ $\parallel$ [100] and strain $\perp$ [010]<br>• $\boldsymbol{B}$ $\parallel$ [001] and strain $\perp$ [010]<br>• $\boldsymbol{B}$ $\perp$ [010]<br>• $\boldsymbol{E}$ $\parallel$ [100] and strain $\perp$ [010]<br>• $\boldsymbol{E}$ $\parallel$ [100] and $\boldsymbol{B}$ $\parallel$ [001]<br>• $\boldsymbol{E}$ $\parallel$ [001] and strain $\perp$ [010]<br>• $\boldsymbol{E}$ $\parallel$ [001] and $\boldsymbol{B}$ $\parallel$ [100]<br>• $\boldsymbol{E}$ $\perp$ [010]
Pc' (7.26) $\mathbb{Z}_1$	• $\boldsymbol{B}$ $\parallel$ [100] and strain $\perp$ [001]<br>• $\boldsymbol{B}$ $\parallel$ [010] and strain $\perp$ [001]<br>• $\boldsymbol{B}$ $\perp$ [001]<br>• $\boldsymbol{E}$ $\parallel$ [100] and strain $\perp$ [001]<br>• $\boldsymbol{E}$ $\parallel$ [100] and $\boldsymbol{B}$ $\parallel$ [010]<br>• $\boldsymbol{E}$ $\parallel$ [010] and strain $\perp$ [001]<br>• $\boldsymbol{E}$ $\parallel$ [010] and $\boldsymbol{B}$ $\parallel$ [100]<br>• $\boldsymbol{E}$ $\perp$ [001]

Continued on next page

...continued

Subgroup & SI group	Perturbation
$P2_1$ (4.7) $\mathbb{Z}_1$	• $\boldsymbol{B} \parallel [100]$ and strain $\perp [100]$<br>• $\boldsymbol{E} \parallel [100]$ and strain $\perp [100]$
$Pc'a'2_1$ (29.103) $\mathbb{Z}_1$	• $\boldsymbol{B} \parallel [100]$<br>• $\boldsymbol{E} \parallel [100]$
$P2_1$ (4.7) $\mathbb{Z}_1$	• $\boldsymbol{B} \parallel [010]$ and strain $\perp [010]$<br>• $\boldsymbol{E} \parallel [010]$ and strain $\perp [010]$
$P\bar{1}'$ (2.6) $\mathbb{Z}_1$	• strain in generic direction
$P2_1/c'$ (14.78) $\mathbb{Z}_1$	• strain $\perp [010]$
Pc' (7.26) $\mathbb{Z}_1$	• $\boldsymbol{B} \parallel [010]$ and strain $\perp [100]$<br>• $\boldsymbol{B} \parallel [001]$ and strain $\perp [100]$<br>• $\boldsymbol{B} \perp [100]$<br>• $\boldsymbol{E} \parallel [010]$ and strain $\perp [100]$<br>• $\boldsymbol{E} \parallel [010]$ and $\boldsymbol{B} \parallel [001]$<br>• $\boldsymbol{E} \parallel [001]$ and strain $\perp [100]$<br>• $\boldsymbol{E} \parallel [001]$ and $\boldsymbol{B} \parallel [010]$<br>• $\boldsymbol{E} \perp [100]$
$Pc'a'2_1$ (29.103) $\mathbb{Z}_1$	• $\boldsymbol{B} \parallel [010]$<br>• $\boldsymbol{E} \parallel [010]$
$P2_1/c'$ (14.78) $\mathbb{Z}_1$	• strain $\perp [100]$
$P2_1$ (4.7) $\mathbb{Z}_1$	• $\boldsymbol{B} \parallel [001]$ and strain $\perp [001]$<br>• $\boldsymbol{E} \parallel [001]$ and strain $\perp [001]$
$P2_1/c'$ (14.78) $\mathbb{Z}_1$	• strain $\perp [001]$
$Pc'a'2_1$ (29.103) $\mathbb{Z}_1$	• $\boldsymbol{B} \parallel [001]$<br>• $\boldsymbol{E} \parallel [001]$

Table. S 548: Subgroups of P_abca (61.438) that can be realized by applying $\boldsymbol{E}/\boldsymbol{B}$ fields and/or uniaxial strains.

Subgroup & SI group	Perturbation

Continued on next page

...continued

Subgroup & SI group	Perturbation
Pm' (6.20) $\mathbb{Z}_1$	• $\boldsymbol{E}$ ∥ [100] and $\boldsymbol{B}$ ∥ [100] and strain ⊥ [001]<br>• $\boldsymbol{E}$ ∥ [100] and $\boldsymbol{B}$ ∥ [010] and strain ⊥ [001]<br>• $\boldsymbol{E}$ ∥ [100] and $\boldsymbol{B}$ ⊥ [001]<br>• $\boldsymbol{E}$ ∥ [010] and $\boldsymbol{B}$ ∥ [100] and strain ⊥ [001]<br>• $\boldsymbol{E}$ ∥ [010] and $\boldsymbol{B}$ ∥ [010] and strain ⊥ [001]<br>• $\boldsymbol{E}$ ∥ [010] and $\boldsymbol{B}$ ⊥ [001]<br>• $\boldsymbol{E}$ ⊥ [001] and $\boldsymbol{B}$ ∥ [100]<br>• $\boldsymbol{E}$ ⊥ [001] and $\boldsymbol{B}$ ∥ [010]<br>• $\boldsymbol{E}$ ⊥ [001] and $\boldsymbol{B}$ ⊥ [001]
Pc (7.24) $\mathbb{Z}_1$	• $\boldsymbol{E}$ ∥ [100] and $\boldsymbol{B}$ ∥ [010] and strain ⊥ [010]<br>• $\boldsymbol{E}$ ∥ [001] and $\boldsymbol{B}$ ∥ [010] and strain ⊥ [010]<br>• $\boldsymbol{E}$ ⊥ [010] and $\boldsymbol{B}$ ∥ [010]
$P2'$ (3.3) $\mathbb{Z}_1$	• $\boldsymbol{E}$ ∥ [100] and $\boldsymbol{B}$ ∥ [010] and strain ⊥ [100]<br>• $\boldsymbol{E}$ ∥ [100] and $\boldsymbol{B}$ ∥ [001] and strain ⊥ [100]<br>• $\boldsymbol{E}$ ∥ [100] and $\boldsymbol{B}$ ⊥ [100]
$Pm'a2'$ (28.89) $\mathbb{Z}_1$	• $\boldsymbol{E}$ ∥ [100] and $\boldsymbol{B}$ ∥ [010]
P_S1 (1.3) $\mathbb{Z}_1$	• $\boldsymbol{E}$ ∥ [100] and strain in generic direction<br>• $\boldsymbol{E}$ ∥ [010] and strain in generic direction<br>• $\boldsymbol{E}$ ∥ [001] and strain in generic direction<br>• $\boldsymbol{E}$ ⊥ [100] and strain ⊥ [010]<br>• $\boldsymbol{E}$ ⊥ [100] and strain ⊥ [001]<br>• $\boldsymbol{E}$ ⊥ [100] and strain in generic direction<br>• $\boldsymbol{E}$ ⊥ [010] and strain ⊥ [100]<br>• $\boldsymbol{E}$ ⊥ [010] and strain ⊥ [001]<br>• $\boldsymbol{E}$ ⊥ [010] and strain in generic direction<br>• $\boldsymbol{E}$ ⊥ [001] and strain ⊥ [100]<br>• $\boldsymbol{E}$ ⊥ [001] and strain ⊥ [010]<br>• $\boldsymbol{E}$ ⊥ [001] and strain in generic direction<br>• $\boldsymbol{E}$ in generic direction
Pc (7.24) $\mathbb{Z}_1$	• $\boldsymbol{E}$ ∥ [100] and $\boldsymbol{B}$ ∥ [001] and strain ⊥ [001]<br>• $\boldsymbol{E}$ ∥ [010] and $\boldsymbol{B}$ ∥ [001] and strain ⊥ [001]<br>• $\boldsymbol{E}$ ⊥ [001] and $\boldsymbol{B}$ ∥ [001]
P_cc (7.28) $\mathbb{Z}_1$	• $\boldsymbol{E}$ ∥ [100] and strain ⊥ [001]<br>• $\boldsymbol{E}$ ∥ [010] and strain ⊥ [001]<br>• $\boldsymbol{E}$ ⊥ [001]

Continued on next page

...continued

Subgroup & SI group	Perturbation
Pc' (7.26) $\mathbb{Z}_1$	• $\boldsymbol{E} \parallel [100]$ and $\boldsymbol{B} \parallel [100]$ and strain $\perp [010]$<br>• $\boldsymbol{E} \parallel [100]$ and $\boldsymbol{B} \parallel [001]$ and strain $\perp [010]$<br>• $\boldsymbol{E} \parallel [100]$ and $\boldsymbol{B} \perp [010]$<br>• $\boldsymbol{E} \parallel [001]$ and $\boldsymbol{B} \parallel [100]$ and strain $\perp [010]$<br>• $\boldsymbol{E} \parallel [001]$ and $\boldsymbol{B} \parallel [001]$ and strain $\perp [010]$<br>• $\boldsymbol{E} \parallel [001]$ and $\boldsymbol{B} \perp [010]$<br>• $\boldsymbol{E} \perp [010]$ and $\boldsymbol{B} \parallel [100]$<br>• $\boldsymbol{E} \perp [010]$ and $\boldsymbol{B} \parallel [001]$<br>• $\boldsymbol{E} \perp [010]$ and $\boldsymbol{B} \perp [010]$
P_ac (7.27) $\mathbb{Z}_1$	• $\boldsymbol{E} \parallel [100]$ and strain $\perp [010]$<br>• $\boldsymbol{E} \parallel [001]$ and strain $\perp [010]$<br>• $\boldsymbol{E} \perp [010]$
$Pn'c2'$ (30.113) $\mathbb{Z}_1$	• $\boldsymbol{E} \parallel [100]$ and $\boldsymbol{B} \parallel [001]$
$P2_1$ (4.7) $\mathbb{Z}_1$	• $\boldsymbol{E} \parallel [100]$ and $\boldsymbol{B} \parallel [100]$ and strain $\perp [100]$
P_b2_1 (4.11) $\mathbb{Z}_1$	• $\boldsymbol{E} \parallel [100]$ and strain $\perp [100]$
$Pm'n'2_1$ (31.127) $\mathbb{Z}_1$	• $\boldsymbol{E} \parallel [100]$ and $\boldsymbol{B} \parallel [100]$
P_cca2_1 (29.106) $\mathbb{Z}_1$	• $\boldsymbol{E} \parallel [100]$
Pc' (7.26) $\mathbb{Z}_1$	• $\boldsymbol{E} \parallel [010]$ and $\boldsymbol{B} \parallel [010]$ and strain $\perp [100]$<br>• $\boldsymbol{E} \parallel [010]$ and $\boldsymbol{B} \parallel [001]$ and strain $\perp [100]$<br>• $\boldsymbol{E} \parallel [010]$ and $\boldsymbol{B} \perp [100]$<br>• $\boldsymbol{E} \parallel [001]$ and $\boldsymbol{B} \parallel [010]$ and strain $\perp [100]$<br>• $\boldsymbol{E} \parallel [001]$ and $\boldsymbol{B} \parallel [001]$ and strain $\perp [100]$<br>• $\boldsymbol{E} \parallel [001]$ and $\boldsymbol{B} \perp [100]$<br>• $\boldsymbol{E} \perp [100]$ and $\boldsymbol{B} \parallel [010]$<br>• $\boldsymbol{E} \perp [100]$ and $\boldsymbol{B} \parallel [001]$<br>• $\boldsymbol{E} \perp [100]$ and $\boldsymbol{B} \perp [100]$
$P2_1$ (4.7) $\mathbb{Z}_1$	• $\boldsymbol{E} \parallel [010]$ and $\boldsymbol{B} \parallel [010]$ and strain $\perp [010]$
$Pm'c'2_1$ (26.70) $\mathbb{Z}_1$	• $\boldsymbol{E} \parallel [010]$ and $\boldsymbol{B} \parallel [010]$
$P2_1'$ (4.9) $\mathbb{Z}_1$	• $\boldsymbol{E} \parallel [001]$ and $\boldsymbol{B} \parallel [100]$ and strain $\perp [001]$<br>• $\boldsymbol{E} \parallel [001]$ and $\boldsymbol{B} \parallel [010]$ and strain $\perp [001]$<br>• $\boldsymbol{E} \parallel [001]$ and $\boldsymbol{B} \perp [001]$
$Pca'2_1'$ (29.102) $\mathbb{Z}_1$	• $\boldsymbol{E} \parallel [001]$ and $\boldsymbol{B} \parallel [010]$

Continued on next page

...continued

Subgroup & SI group	Perturbation
$P\bar{1}$ (2.4) $\mathbb{Z}_2 \times \mathbb{Z}_2 \times \mathbb{Z}_2 \times \mathbb{Z}_4$	• $\boldsymbol{B}$ ∥ [100] and strain in generic direction<br>• $\boldsymbol{B}$ ∥ [010] and strain in generic direction<br>• $\boldsymbol{B}$ ∥ [001] and strain in generic direction<br>• $\boldsymbol{B} \perp [100]$ and strain $\perp [010]$<br>• $\boldsymbol{B} \perp [100]$ and strain $\perp [001]$<br>• $\boldsymbol{B} \perp [100]$ and strain in generic direction<br>• $\boldsymbol{B} \perp [010]$ and strain $\perp [100]$<br>• $\boldsymbol{B} \perp [010]$ and strain $\perp [001]$<br>• $\boldsymbol{B} \perp [010]$ and strain in generic direction<br>• $\boldsymbol{B} \perp [001]$ and strain $\perp [100]$<br>• $\boldsymbol{B} \perp [001]$ and strain $\perp [010]$<br>• $\boldsymbol{B} \perp [001]$ and strain in generic direction<br>• $\boldsymbol{B}$ in generic direction
$P2'/c'$ (13.69) $\mathbb{Z}_2 \times \mathbb{Z}_2 \times \mathbb{Z}_4$	• $\boldsymbol{B}$ ∥ [010] and strain $\perp [100]$<br>• $\boldsymbol{B}$ ∥ [001] and strain $\perp [100]$<br>• $\boldsymbol{B} \perp [100]$
$P2_1/c$ (14.75) $\mathbb{Z}_2$	• $\boldsymbol{B}$ ∥ [010] and strain $\perp [010]$
$P2_1'/m'$ (11.54) $\mathbb{Z}_2 \times \mathbb{Z}_2 \times \mathbb{Z}_4$	• $\boldsymbol{B}$ ∥ [100] and strain $\perp [001]$<br>• $\boldsymbol{B}$ ∥ [010] and strain $\perp [001]$<br>• $\boldsymbol{B} \perp [001]$
$Pb'cm'$ (57.384) $\mathbb{Z}_2$	• $\boldsymbol{B}$ ∥ [010]
Pc (7.24) $\mathbb{Z}_1$	• $\boldsymbol{E}$ ∥ [010] and $\boldsymbol{B}$ ∥ [100] and strain $\perp [100]$<br>• $\boldsymbol{E}$ ∥ [001] and $\boldsymbol{B}$ ∥ [100] and strain $\perp [100]$<br>• $\boldsymbol{E} \perp [100]$ and $\boldsymbol{B}$ ∥ [100]
P_bc (7.29) $\mathbb{Z}_1$	• $\boldsymbol{E}$ ∥ [010] and strain $\perp [100]$<br>• $\boldsymbol{E}$ ∥ [001] and strain $\perp [100]$<br>• $\boldsymbol{E} \perp [100]$
$Pn'a2_1'$ (33.146) $\mathbb{Z}_1$	• $\boldsymbol{E}$ ∥ [001] and $\boldsymbol{B}$ ∥ [100]
$P2_1/c$ (14.75) $\mathbb{Z}_2$	• $\boldsymbol{B}$ ∥ [100] and strain $\perp [100]$
$P2_1'$ (4.9) $\mathbb{Z}_1$	• $\boldsymbol{E}$ ∥ [010] and $\boldsymbol{B}$ ∥ [100] and strain $\perp [010]$<br>• $\boldsymbol{E}$ ∥ [010] and $\boldsymbol{B}$ ∥ [001] and strain $\perp [010]$<br>• $\boldsymbol{E}$ ∥ [010] and $\boldsymbol{B} \perp [010]$
$Pc'a2_1'$ (29.101) $\mathbb{Z}_1$	• $\boldsymbol{E}$ ∥ [010] and $\boldsymbol{B}$ ∥ [001]
P_a2_1 (4.10) $\mathbb{Z}_1$	• $\boldsymbol{E}$ ∥ [010] and strain $\perp [010]$

Continued on next page

...continued

Subgroup & SI group	Perturbation
$P2_1'/c'$ (14.79) $\mathbb{Z}_2 \times \mathbb{Z}_4$	• $\boldsymbol{B} \parallel [100]$ and strain $\perp [010]$<br>• $\boldsymbol{B} \parallel [001]$ and strain $\perp [010]$<br>• $\boldsymbol{B} \perp [010]$
$Pm'c2_1'$ (26.68) $\mathbb{Z}_1$	• $\boldsymbol{E} \parallel [010]$ and $\boldsymbol{B} \parallel [100]$
P_aca2_1 (29.104) $\mathbb{Z}_1$	• $\boldsymbol{E} \parallel [010]$
$Pn'm'a$ (62.446) $\mathbb{Z}_2$	• $\boldsymbol{B} \parallel [100]$
$P2_1$ (4.7) $\mathbb{Z}_1$	• $\boldsymbol{E} \parallel [001]$ and $\boldsymbol{B} \parallel [001]$ and strain $\perp [001]$
$Pn'a'2_1$ (33.148) $\mathbb{Z}_1$	• $\boldsymbol{E} \parallel [001]$ and $\boldsymbol{B} \parallel [001]$
P_a2_1 (4.10) $\mathbb{Z}_1$	• $\boldsymbol{E} \parallel [001]$ and strain $\perp [001]$
$P2_1/c$ (14.75) $\mathbb{Z}_2$	• $\boldsymbol{B} \parallel [001]$ and strain $\perp [001]$
P_bca2_1 (29.105) $\mathbb{Z}_2$	• $\boldsymbol{E} \parallel [001]$
$Pbc'n'$ (60.423) $\mathbb{Z}_2$	• $\boldsymbol{B} \parallel [001]$
$P_S\bar{1}$ (2.7) $\mathbb{Z}_2 \times \mathbb{Z}_2 \times \mathbb{Z}_4$	• strain in generic direction
P_b2_1/c (14.81) $\mathbb{Z}_2$	• strain $\perp [100]$
P_a2_1/c (14.80) $\mathbb{Z}_2$	• strain $\perp [010]$
P_c2_1/c (14.82) $\mathbb{Z}_2$	• strain $\perp [001]$

Table. S 549: Subgroups of P_Cbca (61.439) that can be realized by applying $\boldsymbol{E}/\boldsymbol{B}$ fields and/or uniaxial strains.

Subgroup & SI group	Perturbation
Pc' (7.26) $\mathbb{Z}_1$	• $\boldsymbol{E} \parallel [100]$ and $\boldsymbol{B} \parallel [100]$ and strain $\perp [001]$<br>• $\boldsymbol{E} \parallel [100]$ and $\boldsymbol{B} \parallel [010]$ and strain $\perp [001]$<br>• $\boldsymbol{E} \parallel [100]$ and $\boldsymbol{B} \perp [001]$<br>• $\boldsymbol{E} \parallel [010]$ and $\boldsymbol{B} \parallel [100]$ and strain $\perp [001]$<br>• $\boldsymbol{E} \parallel [010]$ and $\boldsymbol{B} \parallel [010]$ and strain $\perp [001]$<br>• $\boldsymbol{E} \parallel [010]$ and $\boldsymbol{B} \perp [001]$<br>• $\boldsymbol{E} \perp [001]$ and $\boldsymbol{B} \parallel [100]$<br>• $\boldsymbol{E} \perp [001]$ and $\boldsymbol{B} \parallel [010]$<br>• $\boldsymbol{E} \perp [001]$ and $\boldsymbol{B} \perp [001]$

Continued on next page

Subgroup & SI group	Perturbation
$P2'$ (3.3) $\mathbb{Z}_1$	• $\boldsymbol{E}$ ∥ [100] and $\boldsymbol{B}$ ∥ [010] and strain ⊥ [100]<br>• $\boldsymbol{E}$ ∥ [100] and $\boldsymbol{B}$ ∥ [001] and strain ⊥ [100]<br>• $\boldsymbol{E}$ ∥ [100] and $\boldsymbol{B}$ ⊥ [100]
Pc (7.24) $\mathbb{Z}_1$	• $\boldsymbol{E}$ ∥ [100] and $\boldsymbol{B}$ ∥ [010] and strain ⊥ [010]<br>• $\boldsymbol{E}$ ∥ [001] and $\boldsymbol{B}$ ∥ [010] and strain ⊥ [010]<br>• $\boldsymbol{E}$ ⊥ [010] and $\boldsymbol{B}$ ∥ [010]
$Pb'a2'$ (32.137) $\mathbb{Z}_1$	• $\boldsymbol{E}$ ∥ [100] and $\boldsymbol{B}$ ∥ [010]
Pc (7.24) $\mathbb{Z}_1$	• $\boldsymbol{E}$ ∥ [100] and $\boldsymbol{B}$ ∥ [001] and strain ⊥ [001]<br>• $\boldsymbol{E}$ ∥ [010] and $\boldsymbol{B}$ ∥ [001] and strain ⊥ [001]<br>• $\boldsymbol{E}$ ⊥ [001] and $\boldsymbol{B}$ ∥ [001]
P_S1 (1.3) $\mathbb{Z}_1$	• $\boldsymbol{E}$ ∥ [100] and strain in generic direction<br>• $\boldsymbol{E}$ ∥ [010] and strain in generic direction<br>• $\boldsymbol{E}$ ∥ [001] and strain in generic direction<br>• $\boldsymbol{E}$ ⊥ [100] and strain ⊥ [010]<br>• $\boldsymbol{E}$ ⊥ [100] and strain ⊥ [001]<br>• $\boldsymbol{E}$ ⊥ [100] and strain in generic direction<br>• $\boldsymbol{E}$ ⊥ [010] and strain ⊥ [100]<br>• $\boldsymbol{E}$ ⊥ [010] and strain ⊥ [001]<br>• $\boldsymbol{E}$ ⊥ [010] and strain in generic direction<br>• $\boldsymbol{E}$ ⊥ [001] and strain ⊥ [100]<br>• $\boldsymbol{E}$ ⊥ [001] and strain ⊥ [010]<br>• $\boldsymbol{E}$ ⊥ [001] and strain in generic direction<br>• $\boldsymbol{E}$ in generic direction
P_ac (7.27) $\mathbb{Z}_1$	• $\boldsymbol{E}$ ∥ [100] and strain ⊥ [001]<br>• $\boldsymbol{E}$ ∥ [010] and strain ⊥ [001]<br>• $\boldsymbol{E}$ ⊥ [001]
Pc' (7.26) $\mathbb{Z}_1$	• $\boldsymbol{E}$ ∥ [100] and $\boldsymbol{B}$ ∥ [100] and strain ⊥ [010]<br>• $\boldsymbol{E}$ ∥ [100] and $\boldsymbol{B}$ ∥ [001] and strain ⊥ [010]<br>• $\boldsymbol{E}$ ∥ [100] and $\boldsymbol{B}$ ⊥ [010]<br>• $\boldsymbol{E}$ ∥ [001] and $\boldsymbol{B}$ ∥ [100] and strain ⊥ [010]<br>• $\boldsymbol{E}$ ∥ [001] and $\boldsymbol{B}$ ∥ [001] and strain ⊥ [010]<br>• $\boldsymbol{E}$ ∥ [001] and $\boldsymbol{B}$ ⊥ [010]<br>• $\boldsymbol{E}$ ⊥ [010] and $\boldsymbol{B}$ ∥ [100]<br>• $\boldsymbol{E}$ ⊥ [010] and $\boldsymbol{B}$ ∥ [001]<br>• $\boldsymbol{E}$ ⊥ [010] and $\boldsymbol{B}$ ⊥ [010]
$Pn'c2'$ (30.113) $\mathbb{Z}_1$	• $\boldsymbol{E}$ ∥ [100] and $\boldsymbol{B}$ ∥ [001]

Continued on next page

...continued

Subgroup & SI group	Perturbation
P_Cc (7.30) $\mathbb{Z}_1$	• $\boldsymbol{E}$ ∥ [100] and strain ⊥ [010]<br>• $\boldsymbol{E}$ ∥ [001] and strain ⊥ [010]<br>• $\boldsymbol{E}$ ⊥ [010]
$P2_1$ (4.7) $\mathbb{Z}_1$	• $\boldsymbol{E}$ ∥ [100] and $\boldsymbol{B}$ ∥ [100] and strain ⊥ [100]
P_C2_1 (4.12) $\mathbb{Z}_1$	• $\boldsymbol{E}$ ∥ [100] and strain ⊥ [100]
$Pn'a'2_1$ (33.148) $\mathbb{Z}_1$	• $\boldsymbol{E}$ ∥ [100] and $\boldsymbol{B}$ ∥ [100]
P_Aca2_1 (29.107) $\mathbb{Z}_1$	• $\boldsymbol{E}$ ∥ [100]
Pm' (6.20) $\mathbb{Z}_1$	• $\boldsymbol{E}$ ∥ [010] and $\boldsymbol{B}$ ∥ [010] and strain ⊥ [100]<br>• $\boldsymbol{E}$ ∥ [010] and $\boldsymbol{B}$ ∥ [001] and strain ⊥ [100]<br>• $\boldsymbol{E}$ ∥ [010] and $\boldsymbol{B}$ ⊥ [100]<br>• $\boldsymbol{E}$ ∥ [001] and $\boldsymbol{B}$ ∥ [010] and strain ⊥ [100]<br>• $\boldsymbol{E}$ ∥ [001] and $\boldsymbol{B}$ ∥ [001] and strain ⊥ [100]<br>• $\boldsymbol{E}$ ∥ [001] and $\boldsymbol{B}$ ⊥ [100]<br>• $\boldsymbol{E}$ ⊥ [100] and $\boldsymbol{B}$ ∥ [010]<br>• $\boldsymbol{E}$ ⊥ [100] and $\boldsymbol{B}$ ∥ [001]<br>• $\boldsymbol{E}$ ⊥ [100] and $\boldsymbol{B}$ ⊥ [100]
$P2_1$ (4.7) $\mathbb{Z}_1$	• $\boldsymbol{E}$ ∥ [010] and $\boldsymbol{B}$ ∥ [010] and strain ⊥ [010]
$Pm'c'2_1$ (26.70) $\mathbb{Z}_1$	• $\boldsymbol{E}$ ∥ [010] and $\boldsymbol{B}$ ∥ [010]
$P\bar{1}$ (2.4) $\mathbb{Z}_2 \times \mathbb{Z}_2 \times \mathbb{Z}_2 \times \mathbb{Z}_4$	• $\boldsymbol{B}$ ∥ [100] and strain in generic direction<br>• $\boldsymbol{B}$ ∥ [010] and strain in generic direction<br>• $\boldsymbol{B}$ ∥ [001] and strain in generic direction<br>• $\boldsymbol{B}$ ⊥ [100] and strain ⊥ [010]<br>• $\boldsymbol{B}$ ⊥ [100] and strain ⊥ [001]<br>• $\boldsymbol{B}$ ⊥ [100] and strain in generic direction<br>• $\boldsymbol{B}$ ⊥ [010] and strain ⊥ [100]<br>• $\boldsymbol{B}$ ⊥ [010] and strain ⊥ [001]<br>• $\boldsymbol{B}$ ⊥ [010] and strain in generic direction<br>• $\boldsymbol{B}$ ⊥ [001] and strain ⊥ [100]<br>• $\boldsymbol{B}$ ⊥ [001] and strain ⊥ [010]<br>• $\boldsymbol{B}$ ⊥ [001] and strain in generic direction<br>• $\boldsymbol{B}$ in generic direction
$P2'/m'$ (10.46) $\mathbb{Z}_2 \times \mathbb{Z}_2 \times \mathbb{Z}_2 \times \mathbb{Z}_4$	• $\boldsymbol{B}$ ∥ [010] and strain ⊥ [100]<br>• $\boldsymbol{B}$ ∥ [001] and strain ⊥ [100]<br>• $\boldsymbol{B}$ ⊥ [100]
$P2_1/c$ (14.75) $\mathbb{Z}_2$	• $\boldsymbol{B}$ ∥ [010] and strain ⊥ [010]

Continued on next page

...continued

Subgroup & SI group	Perturbation
$P2_1'$ (4.9) $\mathbb{Z}_1$	• $\boldsymbol{E} \parallel [001]$ and $\boldsymbol{B} \parallel [100]$ and strain $\perp [001]$<br>• $\boldsymbol{E} \parallel [001]$ and $\boldsymbol{B} \parallel [010]$ and strain $\perp [001]$<br>• $\boldsymbol{E} \parallel [001]$ and $\boldsymbol{B} \perp [001]$
$Pm'c2_1'$ (26.68) $\mathbb{Z}_1$	• $\boldsymbol{E} \parallel [001]$ and $\boldsymbol{B} \parallel [010]$
$P2_1'/c'$ (14.79) $\mathbb{Z}_2 \times \mathbb{Z}_4$	• $\boldsymbol{B} \parallel [100]$ and strain $\perp [001]$<br>• $\boldsymbol{B} \parallel [010]$ and strain $\perp [001]$<br>• $\boldsymbol{B} \perp [001]$
$Pb'am'$ (55.358) $\mathbb{Z}_2$	• $\boldsymbol{B} \parallel [010]$
$P2'$ (3.3) $\mathbb{Z}_1$	• $\boldsymbol{E} \parallel [010]$ and $\boldsymbol{B} \parallel [100]$ and strain $\perp [010]$<br>• $\boldsymbol{E} \parallel [010]$ and $\boldsymbol{B} \parallel [001]$ and strain $\perp [010]$<br>• $\boldsymbol{E} \parallel [010]$ and $\boldsymbol{B} \perp [010]$
$Pm'a2'$ (28.89) $\mathbb{Z}_1$	• $\boldsymbol{E} \parallel [010]$ and $\boldsymbol{B} \parallel [001]$
P_C2_1 (4.12) $\mathbb{Z}_1$	• $\boldsymbol{E} \parallel [010]$ and strain $\perp [010]$
$P2'/c'$ (13.69) $\mathbb{Z}_2 \times \mathbb{Z}_2 \times \mathbb{Z}_4$	• $\boldsymbol{B} \parallel [100]$ and strain $\perp [010]$<br>• $\boldsymbol{B} \parallel [001]$ and strain $\perp [010]$<br>• $\boldsymbol{B} \perp [010]$
Pc (7.24) $\mathbb{Z}_1$	• $\boldsymbol{E} \parallel [010]$ and $\boldsymbol{B} \parallel [100]$ and strain $\perp [100]$<br>• $\boldsymbol{E} \parallel [001]$ and $\boldsymbol{B} \parallel [100]$ and strain $\perp [100]$<br>• $\boldsymbol{E} \perp [100]$ and $\boldsymbol{B} \parallel [100]$
P_Ac (7.31) $\mathbb{Z}_1$	• $\boldsymbol{E} \parallel [010]$ and strain $\perp [100]$<br>• $\boldsymbol{E} \parallel [001]$ and strain $\perp [100]$<br>• $\boldsymbol{E} \perp [100]$
$P2_1/c$ (14.75) $\mathbb{Z}_2$	• $\boldsymbol{B} \parallel [100]$ and strain $\perp [100]$
$Pn'a2_1'$ (33.146) $\mathbb{Z}_1$	• $\boldsymbol{E} \parallel [001]$ and $\boldsymbol{B} \parallel [100]$
$Pc'c2'$ (27.80) $\mathbb{Z}_1$	• $\boldsymbol{E} \parallel [010]$ and $\boldsymbol{B} \parallel [100]$
P_Bca2_1 (29.108) $\mathbb{Z}_1$	• $\boldsymbol{E} \parallel [010]$
$Pc'cn'$ (56.370) $\mathbb{Z}_2$	• $\boldsymbol{B} \parallel [100]$
$P2_1$ (4.7) $\mathbb{Z}_1$	• $\boldsymbol{E} \parallel [001]$ and $\boldsymbol{B} \parallel [001]$ and strain $\perp [001]$
$Pm'n'2_1$ (31.127) $\mathbb{Z}_1$	• $\boldsymbol{E} \parallel [001]$ and $\boldsymbol{B} \parallel [001]$
$P2_1/c$ (14.75) $\mathbb{Z}_2$	• $\boldsymbol{B} \parallel [001]$ and strain $\perp [001]$
P_a2_1 (4.10) $\mathbb{Z}_1$	• $\boldsymbol{E} \parallel [001]$ and strain $\perp [001]$
$Pm'n'a$ (53.326) $\mathbb{Z}_2$	• $\boldsymbol{B} \parallel [001]$

Continued on next page

. . . continued

Subgroup & SI group	Perturbation
P_Cca2_1 (29.109) $\mathbb{Z}_1$	• $\boldsymbol{E} \parallel [001]$
$P_S\bar{1}$ (2.7) $\mathbb{Z}_2 \times \mathbb{Z}_2 \times \mathbb{Z}_4$	• strain in generic direction
P_C2_1/c (14.84) $\mathbb{Z}_2$	• strain $\perp$ [010]
P_A2_1/c (14.83) $\mathbb{Z}_2$	• strain $\perp$ [100]
P_a2_1/c (14.80) $\mathbb{Z}_2$	• strain $\perp$ [001]

Table. S 550: Subgroups of P_Ibca (61.440) that can be realized by applying $\boldsymbol{E}/\boldsymbol{B}$ fields and/or uniaxial strains.

Subgroup & SI group	Perturbation
Pc' (7.26) $\mathbb{Z}_1$	• $\boldsymbol{E} \parallel [100]$ and $\boldsymbol{B} \parallel [100]$ and strain $\perp [001]$<br>• $\boldsymbol{E} \parallel [100]$ and $\boldsymbol{B} \parallel [010]$ and strain $\perp [001]$<br>• $\boldsymbol{E} \parallel [100]$ and $\boldsymbol{B} \perp [001]$<br>• $\boldsymbol{E} \parallel [010]$ and $\boldsymbol{B} \parallel [100]$ and strain $\perp [001]$<br>• $\boldsymbol{E} \parallel [010]$ and $\boldsymbol{B} \parallel [010]$ and strain $\perp [001]$<br>• $\boldsymbol{E} \parallel [010]$ and $\boldsymbol{B} \perp [001]$<br>• $\boldsymbol{E} \perp [001]$ and $\boldsymbol{B} \parallel [100]$<br>• $\boldsymbol{E} \perp [001]$ and $\boldsymbol{B} \parallel [010]$<br>• $\boldsymbol{E} \perp [001]$ and $\boldsymbol{B} \perp [001]$
$P2'$ (3.3) $\mathbb{Z}_1$	• $\boldsymbol{E} \parallel [100]$ and $\boldsymbol{B} \parallel [010]$ and strain $\perp [100]$<br>• $\boldsymbol{E} \parallel [100]$ and $\boldsymbol{B} \parallel [001]$ and strain $\perp [100]$<br>• $\boldsymbol{E} \parallel [100]$ and $\boldsymbol{B} \perp [100]$
Pc (7.24) $\mathbb{Z}_1$	• $\boldsymbol{E} \parallel [100]$ and $\boldsymbol{B} \parallel [010]$ and strain $\perp [010]$<br>• $\boldsymbol{E} \parallel [001]$ and $\boldsymbol{B} \parallel [010]$ and strain $\perp [010]$<br>• $\boldsymbol{E} \perp [010]$ and $\boldsymbol{B} \parallel [010]$
$Pb'a2'$ (32.137) $\mathbb{Z}_1$	• $\boldsymbol{E} \parallel [100]$ and $\boldsymbol{B} \parallel [010]$
Pc (7.24) $\mathbb{Z}_1$	• $\boldsymbol{E} \parallel [100]$ and $\boldsymbol{B} \parallel [001]$ and strain $\perp [001]$<br>• $\boldsymbol{E} \parallel [010]$ and $\boldsymbol{B} \parallel [001]$ and strain $\perp [001]$<br>• $\boldsymbol{E} \perp [001]$ and $\boldsymbol{B} \parallel [001]$

Continued on next page

...continued

Subgroup & SI group	Perturbation
P_S1 (1.3) $\mathbb{Z}_1$	• $\boldsymbol{E} \parallel [100]$ and strain in generic direction<br>• $\boldsymbol{E} \parallel [010]$ and strain in generic direction<br>• $\boldsymbol{E} \parallel [001]$ and strain in generic direction<br>• $\boldsymbol{E} \perp [100]$ and strain $\perp [010]$<br>• $\boldsymbol{E} \perp [100]$ and strain $\perp [001]$<br>• $\boldsymbol{E} \perp [100]$ and strain in generic direction<br>• $\boldsymbol{E} \perp [010]$ and strain $\perp [100]$<br>• $\boldsymbol{E} \perp [010]$ and strain $\perp [001]$<br>• $\boldsymbol{E} \perp [010]$ and strain in generic direction<br>• $\boldsymbol{E} \perp [001]$ and strain $\perp [100]$<br>• $\boldsymbol{E} \perp [001]$ and strain $\perp [010]$<br>• $\boldsymbol{E} \perp [001]$ and strain in generic direction<br>• $\boldsymbol{E}$ in generic direction
P_Cc (7.30) $\mathbb{Z}_1$	• $\boldsymbol{E} \parallel [100]$ and strain $\perp [001]$<br>• $\boldsymbol{E} \parallel [010]$ and strain $\perp [001]$<br>• $\boldsymbol{E} \perp [001]$
Pc' (7.26) $\mathbb{Z}_1$	• $\boldsymbol{E} \parallel [100]$ and $\boldsymbol{B} \parallel [100]$ and strain $\perp [010]$<br>• $\boldsymbol{E} \parallel [100]$ and $\boldsymbol{B} \parallel [001]$ and strain $\perp [010]$<br>• $\boldsymbol{E} \parallel [100]$ and $\boldsymbol{B} \perp [010]$<br>• $\boldsymbol{E} \parallel [001]$ and $\boldsymbol{B} \parallel [100]$ and strain $\perp [010]$<br>• $\boldsymbol{E} \parallel [001]$ and $\boldsymbol{B} \parallel [001]$ and strain $\perp [010]$<br>• $\boldsymbol{E} \parallel [001]$ and $\boldsymbol{B} \perp [010]$<br>• $\boldsymbol{E} \perp [010]$ and $\boldsymbol{B} \parallel [100]$<br>• $\boldsymbol{E} \perp [010]$ and $\boldsymbol{B} \parallel [001]$<br>• $\boldsymbol{E} \perp [010]$ and $\boldsymbol{B} \perp [010]$
$Pc'c2'$ (27.80) $\mathbb{Z}_1$	• $\boldsymbol{E} \parallel [100]$ and $\boldsymbol{B} \parallel [001]$
P_Cc (7.30) $\mathbb{Z}_1$	• $\boldsymbol{E} \parallel [100]$ and strain $\perp [010]$<br>• $\boldsymbol{E} \parallel [001]$ and strain $\perp [010]$<br>• $\boldsymbol{E} \perp [010]$
$P2_1$ (4.7) $\mathbb{Z}_1$	• $\boldsymbol{E} \parallel [100]$ and $\boldsymbol{B} \parallel [100]$ and strain $\perp [100]$
P_C2_1 (4.12) $\mathbb{Z}_1$	• $\boldsymbol{E} \parallel [100]$ and strain $\perp [100]$
$Pc'a'2_1$ (29.103) $\mathbb{Z}_1$	• $\boldsymbol{E} \parallel [100]$ and $\boldsymbol{B} \parallel [100]$
P_Ica2_1 (29.110) $\mathbb{Z}_1$	• $\boldsymbol{E} \parallel [100]$

Continued on next page

...continued

Subgroup & SI group	Perturbation
Pc' (7.26) $\mathbb{Z}_1$	• $\boldsymbol{E} \parallel [010]$ and $\boldsymbol{B} \parallel [010]$ and strain $\perp [100]$<br>• $\boldsymbol{E} \parallel [010]$ and $\boldsymbol{B} \parallel [001]$ and strain $\perp [100]$<br>• $\boldsymbol{E} \parallel [010]$ and $\boldsymbol{B} \perp [100]$<br>• $\boldsymbol{E} \parallel [001]$ and $\boldsymbol{B} \parallel [010]$ and strain $\perp [100]$<br>• $\boldsymbol{E} \parallel [001]$ and $\boldsymbol{B} \parallel [001]$ and strain $\perp [100]$<br>• $\boldsymbol{E} \parallel [001]$ and $\boldsymbol{B} \perp [100]$<br>• $\boldsymbol{E} \perp [100]$ and $\boldsymbol{B} \parallel [010]$<br>• $\boldsymbol{E} \perp [100]$ and $\boldsymbol{B} \parallel [001]$<br>• $\boldsymbol{E} \perp [100]$ and $\boldsymbol{B} \perp [100]$
$P2_1$ (4.7) $\mathbb{Z}_1$	• $\boldsymbol{E} \parallel [010]$ and $\boldsymbol{B} \parallel [010]$ and strain $\perp [010]$
$Pc'a'2_1$ (29.103) $\mathbb{Z}_1$	• $\boldsymbol{E} \parallel [010]$ and $\boldsymbol{B} \parallel [010]$
$P\bar{1}$ (2.4) $\mathbb{Z}_2 \times \mathbb{Z}_2 \times \mathbb{Z}_2 \times \mathbb{Z}_4$	• $\boldsymbol{B} \parallel [100]$ and strain in generic direction<br>• $\boldsymbol{B} \parallel [010]$ and strain in generic direction<br>• $\boldsymbol{B} \parallel [001]$ and strain in generic direction<br>• $\boldsymbol{B} \perp [100]$ and strain $\perp [010]$<br>• $\boldsymbol{B} \perp [100]$ and strain $\perp [001]$<br>• $\boldsymbol{B} \perp [100]$ and strain in generic direction<br>• $\boldsymbol{B} \perp [010]$ and strain $\perp [100]$<br>• $\boldsymbol{B} \perp [010]$ and strain $\perp [001]$<br>• $\boldsymbol{B} \perp [010]$ and strain in generic direction<br>• $\boldsymbol{B} \perp [001]$ and strain $\perp [100]$<br>• $\boldsymbol{B} \perp [001]$ and strain $\perp [010]$<br>• $\boldsymbol{B} \perp [001]$ and strain in generic direction<br>• $\boldsymbol{B}$ in generic direction
$P2'/c'$ (13.69) $\mathbb{Z}_2 \times \mathbb{Z}_2 \times \mathbb{Z}_4$	• $\boldsymbol{B} \parallel [010]$ and strain $\perp [100]$<br>• $\boldsymbol{B} \parallel [001]$ and strain $\perp [100]$<br>• $\boldsymbol{B} \perp [100]$
$P2_1/c$ (14.75) $\mathbb{Z}_2$	• $\boldsymbol{B} \parallel [010]$ and strain $\perp [010]$
$P2'$ (3.3) $\mathbb{Z}_1$	• $\boldsymbol{E} \parallel [001]$ and $\boldsymbol{B} \parallel [100]$ and strain $\perp [001]$<br>• $\boldsymbol{E} \parallel [001]$ and $\boldsymbol{B} \parallel [010]$ and strain $\perp [001]$<br>• $\boldsymbol{E} \parallel [001]$ and $\boldsymbol{B} \perp [001]$
$Pc'c2'$ (27.80) $\mathbb{Z}_1$	• $\boldsymbol{E} \parallel [001]$ and $\boldsymbol{B} \parallel [010]$
$P2'/c'$ (13.69) $\mathbb{Z}_2 \times \mathbb{Z}_2 \times \mathbb{Z}_4$	• $\boldsymbol{B} \parallel [100]$ and strain $\perp [001]$<br>• $\boldsymbol{B} \parallel [010]$ and strain $\perp [001]$<br>• $\boldsymbol{B} \perp [001]$
$Pcc'a'$ (54.343) $\mathbb{Z}_2$	• $\boldsymbol{B} \parallel [010]$

Continued on next page

…continued

Subgroup & SI group	Perturbation
$P2'$ (3.3) $\mathbb{Z}_1$	• $\boldsymbol{E} \parallel [010]$ and $\boldsymbol{B} \parallel [100]$ and strain $\perp [010]$<br>• $\boldsymbol{E} \parallel [010]$ and $\boldsymbol{B} \parallel [001]$ and strain $\perp [010]$<br>• $\boldsymbol{E} \parallel [010]$ and $\boldsymbol{B} \perp [010]$
$Pb'a2'$ (32.137) $\mathbb{Z}_1$	• $\boldsymbol{E} \parallel [010]$ and $\boldsymbol{B} \parallel [001]$
P_C2_1 (4.12) $\mathbb{Z}_1$	• $\boldsymbol{E} \parallel [010]$ and strain $\perp [010]$
$P2'/c'$ (13.69) $\mathbb{Z}_2 \times \mathbb{Z}_2 \times \mathbb{Z}_4$	• $\boldsymbol{B} \parallel [100]$ and strain $\perp [010]$<br>• $\boldsymbol{B} \parallel [001]$ and strain $\perp [010]$<br>• $\boldsymbol{B} \perp [010]$
Pc (7.24) $\mathbb{Z}_1$	• $\boldsymbol{E} \parallel [010]$ and $\boldsymbol{B} \parallel [100]$ and strain $\perp [100]$<br>• $\boldsymbol{E} \parallel [001]$ and $\boldsymbol{B} \parallel [100]$ and strain $\perp [100]$<br>• $\boldsymbol{E} \perp [100]$ and $\boldsymbol{B} \parallel [100]$
P_Cc (7.30) $\mathbb{Z}_1$	• $\boldsymbol{E} \parallel [010]$ and strain $\perp [100]$<br>• $\boldsymbol{E} \parallel [001]$ and strain $\perp [100]$<br>• $\boldsymbol{E} \perp [100]$
$P2_1/c$ (14.75) $\mathbb{Z}_2$	• $\boldsymbol{B} \parallel [100]$ and strain $\perp [100]$
$Pb'a2'$ (32.137) $\mathbb{Z}_1$	• $\boldsymbol{E} \parallel [001]$ and $\boldsymbol{B} \parallel [100]$
$Pc'c2'$ (27.80) $\mathbb{Z}_1$	• $\boldsymbol{E} \parallel [010]$ and $\boldsymbol{B} \parallel [100]$
P_Ica2_1 (29.110) $\mathbb{Z}_1$	• $\boldsymbol{E} \parallel [010]$
$Pcc'a'$ (54.343) $\mathbb{Z}_2$	• $\boldsymbol{B} \parallel [100]$
$P2_1$ (4.7) $\mathbb{Z}_1$	• $\boldsymbol{E} \parallel [001]$ and $\boldsymbol{B} \parallel [001]$ and strain $\perp [001]$
$Pc'a'2_1$ (29.103) $\mathbb{Z}_1$	• $\boldsymbol{E} \parallel [001]$ and $\boldsymbol{B} \parallel [001]$
$P2_1/c$ (14.75) $\mathbb{Z}_2$	• $\boldsymbol{B} \parallel [001]$ and strain $\perp [001]$
P_C2_1 (4.12) $\mathbb{Z}_1$	• $\boldsymbol{E} \parallel [001]$ and strain $\perp [001]$
$Pcc'a'$ (54.343) $\mathbb{Z}_2$	• $\boldsymbol{B} \parallel [001]$
P_Ica2_1 (29.110) $\mathbb{Z}_1$	• $\boldsymbol{E} \parallel [001]$
$P_S\bar{1}$ (2.7) $\mathbb{Z}_2 \times \mathbb{Z}_2 \times \mathbb{Z}_4$	• strain in generic direction
P_C2_1/c (14.84) $\mathbb{Z}_2$	• strain $\perp [010]$
P_C2_1/c (14.84) $\mathbb{Z}_2$	• strain $\perp [100]$
P_C2_1/c (14.84) $\mathbb{Z}_2$	• strain $\perp [001]$

Table. S 551: Subgroups of $Pnma$ (62.441) that can be realized by applying $\boldsymbol{E}/\boldsymbol{B}$ fields and/or uniaxial strains.

Subgroup & SI group	Perturbation
Pm (6.18) $\mathbb{Z}_1$	• $\boldsymbol{E} \parallel [100]$ and strain $\perp [010]$<br>• $\boldsymbol{E} \parallel [100]$ and $\boldsymbol{B} \parallel [010]$<br>• $\boldsymbol{E} \parallel [001]$ and strain $\perp [010]$<br>• $\boldsymbol{E} \parallel [001]$ and $\boldsymbol{B} \parallel [010]$<br>• $\boldsymbol{E} \perp [010]$
Pc (7.24) $\mathbb{Z}_1$	• $\boldsymbol{E} \parallel [100]$ and strain $\perp [001]$<br>• $\boldsymbol{E} \parallel [100]$ and $\boldsymbol{B} \parallel [001]$<br>• $\boldsymbol{E} \parallel [010]$ and strain $\perp [001]$<br>• $\boldsymbol{E} \parallel [010]$ and $\boldsymbol{B} \parallel [001]$<br>• $\boldsymbol{E} \perp [001]$
$P2_1$ (4.7) $\mathbb{Z}_1$	• $\boldsymbol{E} \parallel [100]$ and strain $\perp [100]$<br>• $\boldsymbol{E} \parallel [100]$ and $\boldsymbol{B} \parallel [100]$
$Pmc2_1$ (26.66) $\mathbb{Z}_1$	• $\boldsymbol{E} \parallel [100]$
$P2_1$ (4.7) $\mathbb{Z}_1$	• $\boldsymbol{E} \parallel [010]$ and strain $\perp [010]$<br>• $\boldsymbol{E} \parallel [010]$ and $\boldsymbol{B} \parallel [010]$
$P\bar{1}$ (2.4) $\mathbb{Z}_2 \times \mathbb{Z}_2 \times \mathbb{Z}_2 \times \mathbb{Z}_4$	• strain in generic direction<br>• $\boldsymbol{B} \parallel [100]$ and strain $\perp [010]$<br>• $\boldsymbol{B} \parallel [100]$ and strain $\perp [001]$<br>• $\boldsymbol{B} \parallel [010]$ and strain $\perp [100]$<br>• $\boldsymbol{B} \parallel [010]$ and strain $\perp [001]$<br>• $\boldsymbol{B} \parallel [001]$ and strain $\perp [100]$<br>• $\boldsymbol{B} \parallel [001]$ and strain $\perp [010]$<br>• $\boldsymbol{B}$ in generic direction
$P2_1/m$ (11.50) $\mathbb{Z}_2$	• strain $\perp [010]$<br>• $\boldsymbol{B} \parallel [010]$
Pc (7.24) $\mathbb{Z}_1$	• $\boldsymbol{E} \parallel [010]$ and strain $\perp [100]$<br>• $\boldsymbol{E} \parallel [010]$ and $\boldsymbol{B} \parallel [100]$<br>• $\boldsymbol{E} \parallel [001]$ and strain $\perp [100]$<br>• $\boldsymbol{E} \parallel [001]$ and $\boldsymbol{B} \parallel [100]$<br>• $\boldsymbol{E} \perp [100]$
$Pna2_1$ (33.144) $\mathbb{Z}_1$	• $\boldsymbol{E} \parallel [010]$
$P2_1/c$ (14.75) $\mathbb{Z}_2$	• strain $\perp [100]$<br>• $\boldsymbol{B} \parallel [100]$
$P2_1$ (4.7) $\mathbb{Z}_1$	• $\boldsymbol{E} \parallel [001]$ and strain $\perp [001]$<br>• $\boldsymbol{E} \parallel [001]$ and $\boldsymbol{B} \parallel [001]$

Continued on next page

. . . continued

Subgroup & SI group	Perturbation
$P2_1/c$ (14.75) $\mathbb{Z}_2$	• strain $\perp$ [001]<br>• $\boldsymbol{B}$ $\parallel$ [001]
$Pmn2_1$ (31.123) $\mathbb{Z}_1$	• $\boldsymbol{E}$ $\parallel$ [001]

Table. S 552: Subgroups of $Pnma1'$ (62.442) that can be realized by applying $\boldsymbol{E}/\boldsymbol{B}$ fields and/or uniaxial strains.

Subgroup & SI group	Perturbation
$P11'$ (1.2) $\mathbb{Z}_1$	• $\boldsymbol{E}$ $\parallel$ [100] and strain in generic direction<br>• $\boldsymbol{E}$ $\parallel$ [010] and strain in generic direction<br>• $\boldsymbol{E}$ $\parallel$ [001] and strain in generic direction<br>• $\boldsymbol{E}$ $\perp$ [100] and strain $\perp$ [010]<br>• $\boldsymbol{E}$ $\perp$ [100] and strain $\perp$ [001]<br>• $\boldsymbol{E}$ $\perp$ [100] and strain in generic direction<br>• $\boldsymbol{E}$ $\perp$ [010] and strain $\perp$ [100]<br>• $\boldsymbol{E}$ $\perp$ [010] and strain $\perp$ [001]<br>• $\boldsymbol{E}$ $\perp$ [010] and strain in generic direction<br>• $\boldsymbol{E}$ $\perp$ [001] and strain $\perp$ [100]<br>• $\boldsymbol{E}$ $\perp$ [001] and strain $\perp$ [010]<br>• $\boldsymbol{E}$ $\perp$ [001] and strain in generic direction<br>• $\boldsymbol{E}$ in generic direction
Pm' (6.20) $\mathbb{Z}_1$	• $\boldsymbol{E}$ $\parallel$ [100] and $\boldsymbol{B}$ $\parallel$ [100] and strain $\perp$ [010]<br>• $\boldsymbol{E}$ $\parallel$ [100] and $\boldsymbol{B}$ $\parallel$ [001] and strain $\perp$ [010]<br>• $\boldsymbol{E}$ $\parallel$ [100] and $\boldsymbol{B}$ $\perp$ [010]<br>• $\boldsymbol{E}$ $\parallel$ [001] and $\boldsymbol{B}$ $\parallel$ [100] and strain $\perp$ [010]<br>• $\boldsymbol{E}$ $\parallel$ [001] and $\boldsymbol{B}$ $\parallel$ [001] and strain $\perp$ [010]<br>• $\boldsymbol{E}$ $\parallel$ [001] and $\boldsymbol{B}$ $\perp$ [010]<br>• $\boldsymbol{E}$ $\perp$ [010] and $\boldsymbol{B}$ $\parallel$ [100]<br>• $\boldsymbol{E}$ $\perp$ [010] and $\boldsymbol{B}$ $\parallel$ [001]<br>• $\boldsymbol{E}$ $\perp$ [010] and $\boldsymbol{B}$ $\perp$ [010]
Pm (6.18) $\mathbb{Z}_1$	• $\boldsymbol{E}$ $\parallel$ [100] and $\boldsymbol{B}$ $\parallel$ [010] and strain $\perp$ [010]<br>• $\boldsymbol{E}$ $\parallel$ [001] and $\boldsymbol{B}$ $\parallel$ [010] and strain $\perp$ [010]<br>• $\boldsymbol{E}$ $\perp$ [010] and $\boldsymbol{B}$ $\parallel$ [010]
$Pm1'$ (6.19) $\mathbb{Z}_1$	• $\boldsymbol{E}$ $\parallel$ [100] and strain $\perp$ [010]<br>• $\boldsymbol{E}$ $\parallel$ [001] and strain $\perp$ [010]<br>• $\boldsymbol{E}$ $\perp$ [010]

Continued on next page

...continued

Subgroup & SI group	Perturbation
Pc' (7.26) $\mathbb{Z}_1$	• $\boldsymbol{E}$ ∥ [100] and $\boldsymbol{B}$ ∥ [100] and strain ⊥ [001]<br>• $\boldsymbol{E}$ ∥ [100] and $\boldsymbol{B}$ ∥ [010] and strain ⊥ [001]<br>• $\boldsymbol{E}$ ∥ [100] and $\boldsymbol{B}$ ⊥ [001]<br>• $\boldsymbol{E}$ ∥ [010] and $\boldsymbol{B}$ ∥ [100] and strain ⊥ [001]<br>• $\boldsymbol{E}$ ∥ [010] and $\boldsymbol{B}$ ∥ [010] and strain ⊥ [001]<br>• $\boldsymbol{E}$ ∥ [010] and $\boldsymbol{B}$ ⊥ [001]<br>• $\boldsymbol{E}$ ⊥ [001] and $\boldsymbol{B}$ ∥ [100]<br>• $\boldsymbol{E}$ ⊥ [001] and $\boldsymbol{B}$ ∥ [010]<br>• $\boldsymbol{E}$ ⊥ [001] and $\boldsymbol{B}$ ⊥ [001]
Pc (7.24) $\mathbb{Z}_1$	• $\boldsymbol{E}$ ∥ [100] and $\boldsymbol{B}$ ∥ [001] and strain ⊥ [001]<br>• $\boldsymbol{E}$ ∥ [010] and $\boldsymbol{B}$ ∥ [001] and strain ⊥ [001]<br>• $\boldsymbol{E}$ ⊥ [001] and $\boldsymbol{B}$ ∥ [001]
$Pc1'$ (7.25) $\mathbb{Z}_1$	• $\boldsymbol{E}$ ∥ [100] and strain ⊥ [001]<br>• $\boldsymbol{E}$ ∥ [010] and strain ⊥ [001]<br>• $\boldsymbol{E}$ ⊥ [001]
$P2_1'$ (4.9) $\mathbb{Z}_1$	• $\boldsymbol{E}$ ∥ [100] and $\boldsymbol{B}$ ∥ [010] and strain ⊥ [100]<br>• $\boldsymbol{E}$ ∥ [100] and $\boldsymbol{B}$ ∥ [001] and strain ⊥ [100]<br>• $\boldsymbol{E}$ ∥ [100] and $\boldsymbol{B}$ ⊥ [100]
$Pmc'2_1'$ (26.69) $\mathbb{Z}_1$	• $\boldsymbol{E}$ ∥ [100] and $\boldsymbol{B}$ ∥ [010]
$Pm'c2_1'$ (26.68) $\mathbb{Z}_1$	• $\boldsymbol{E}$ ∥ [100] and $\boldsymbol{B}$ ∥ [001]
$P2_1$ (4.7) $\mathbb{Z}_1$	• $\boldsymbol{E}$ ∥ [100] and $\boldsymbol{B}$ ∥ [100] and strain ⊥ [100]
$Pm'c'2_1$ (26.70) $\mathbb{Z}_1$	• $\boldsymbol{E}$ ∥ [100] and $\boldsymbol{B}$ ∥ [100]
$P2_11'$ (4.8) $\mathbb{Z}_1$	• $\boldsymbol{E}$ ∥ [100] and strain ⊥ [100]
$Pmc2_11'$ (26.67) $\mathbb{Z}_1$	• $\boldsymbol{E}$ ∥ [100]
$P2_1'$ (4.9) $\mathbb{Z}_1$	• $\boldsymbol{E}$ ∥ [010] and $\boldsymbol{B}$ ∥ [100] and strain ⊥ [010]<br>• $\boldsymbol{E}$ ∥ [010] and $\boldsymbol{B}$ ∥ [001] and strain ⊥ [010]<br>• $\boldsymbol{E}$ ∥ [010] and $\boldsymbol{B}$ ⊥ [010]
$P2_1$ (4.7) $\mathbb{Z}_1$	• $\boldsymbol{E}$ ∥ [010] and $\boldsymbol{B}$ ∥ [010] and strain ⊥ [010]
$P2_11'$ (4.8) $\mathbb{Z}_1$	• $\boldsymbol{E}$ ∥ [010] and strain ⊥ [010]

Continued on next page

...continued

Subgroup & SI group	Perturbation
$P\bar{1}$ (2.4) $\mathbb{Z}_2 \times \mathbb{Z}_2 \times \mathbb{Z}_2 \times \mathbb{Z}_4$	• $\boldsymbol{B}$ ∥ [100] and strain in generic direction<br>• $\boldsymbol{B}$ ∥ [010] and strain in generic direction<br>• $\boldsymbol{B}$ ∥ [001] and strain in generic direction<br>• $\boldsymbol{B} \perp$ [100] and strain $\perp$ [010]<br>• $\boldsymbol{B} \perp$ [100] and strain $\perp$ [001]<br>• $\boldsymbol{B} \perp$ [100] and strain in generic direction<br>• $\boldsymbol{B} \perp$ [010] and strain $\perp$ [100]<br>• $\boldsymbol{B} \perp$ [010] and strain $\perp$ [001]<br>• $\boldsymbol{B} \perp$ [010] and strain in generic direction<br>• $\boldsymbol{B} \perp$ [001] and strain $\perp$ [100]<br>• $\boldsymbol{B} \perp$ [001] and strain $\perp$ [010]<br>• $\boldsymbol{B} \perp$ [001] and strain in generic direction<br>• $\boldsymbol{B}$ in generic direction
$P2_1'/m'$ (11.54) $\mathbb{Z}_2 \times \mathbb{Z}_2 \times \mathbb{Z}_4$	• $\boldsymbol{B}$ ∥ [100] and strain $\perp$ [010]<br>• $\boldsymbol{B}$ ∥ [001] and strain $\perp$ [010]<br>• $\boldsymbol{B} \perp$ [010]
$P2_1/m$ (11.50) $\mathbb{Z}_2$	• $\boldsymbol{B}$ ∥ [010] and strain $\perp$ [010]
$P\bar{1}1'$ (2.5) $\mathbb{Z}_2 \times \mathbb{Z}_2 \times \mathbb{Z}_2 \times \mathbb{Z}_4$	• strain in generic direction
$P2_1/m1'$ (11.51) $\mathbb{Z}_2$	• strain $\perp$ [010]
Pc' (7.26) $\mathbb{Z}_1$	• $\boldsymbol{E}$ ∥ [010] and $\boldsymbol{B}$ ∥ [010] and strain $\perp$ [100]<br>• $\boldsymbol{E}$ ∥ [010] and $\boldsymbol{B}$ ∥ [001] and strain $\perp$ [100]<br>• $\boldsymbol{E}$ ∥ [010] and $\boldsymbol{B} \perp$ [100]<br>• $\boldsymbol{E}$ ∥ [001] and $\boldsymbol{B}$ ∥ [010] and strain $\perp$ [100]<br>• $\boldsymbol{E}$ ∥ [001] and $\boldsymbol{B}$ ∥ [001] and strain $\perp$ [100]<br>• $\boldsymbol{E}$ ∥ [001] and $\boldsymbol{B} \perp$ [100]<br>• $\boldsymbol{E} \perp$ [100] and $\boldsymbol{B}$ ∥ [010]<br>• $\boldsymbol{E} \perp$ [100] and $\boldsymbol{B}$ ∥ [001]<br>• $\boldsymbol{E} \perp$ [100] and $\boldsymbol{B} \perp$ [100]
$Pn'a2_1'$ (33.146) $\mathbb{Z}_1$	• $\boldsymbol{E}$ ∥ [010] and $\boldsymbol{B}$ ∥ [001]
$Pn'a'2_1$ (33.148) $\mathbb{Z}_1$	• $\boldsymbol{E}$ ∥ [010] and $\boldsymbol{B}$ ∥ [010]
$P2_1'/c'$ (14.79) $\mathbb{Z}_2 \times \mathbb{Z}_4$	• $\boldsymbol{B}$ ∥ [010] and strain $\perp$ [100]<br>• $\boldsymbol{B}$ ∥ [001] and strain $\perp$ [100]<br>• $\boldsymbol{B} \perp$ [100]
Pc (7.24) $\mathbb{Z}_1$	• $\boldsymbol{E}$ ∥ [010] and $\boldsymbol{B}$ ∥ [100] and strain $\perp$ [100]<br>• $\boldsymbol{E}$ ∥ [001] and $\boldsymbol{B}$ ∥ [100] and strain $\perp$ [100]<br>• $\boldsymbol{E} \perp$ [100] and $\boldsymbol{B}$ ∥ [100]

Continued on next page

...continued

Subgroup & SI group	Perturbation
$Pna'2_1'$ (33.147) $\mathbb{Z}_1$	• $\boldsymbol{E}$ ∥ [010] and $\boldsymbol{B}$ ∥ [100]
$P2_1/c$ (14.75) $\mathbb{Z}_2$	• $\boldsymbol{B}$ ∥ [100] and strain ⊥ [100]
$Pc1'$ (7.25) $\mathbb{Z}_1$	• $\boldsymbol{E}$ ∥ [010] and strain ⊥ [100]<br>• $\boldsymbol{E}$ ∥ [001] and strain ⊥ [100]<br>• $\boldsymbol{E}$ ⊥ [100]
$Pna2_11'$ (33.145) $\mathbb{Z}_1$	• $\boldsymbol{E}$ ∥ [010]
$P2_1/c1'$ (14.76) $\mathbb{Z}_2$	• strain ⊥ [100]
$P2_1'$ (4.9) $\mathbb{Z}_1$	• $\boldsymbol{E}$ ∥ [001] and $\boldsymbol{B}$ ∥ [100] and strain ⊥ [001]<br>• $\boldsymbol{E}$ ∥ [001] and $\boldsymbol{B}$ ∥ [010] and strain ⊥ [001]<br>• $\boldsymbol{E}$ ∥ [001] and $\boldsymbol{B}$ ⊥ [001]
$P2_1'/c'$ (14.79) $\mathbb{Z}_2 \times \mathbb{Z}_4$	• $\boldsymbol{B}$ ∥ [100] and strain ⊥ [001]<br>• $\boldsymbol{B}$ ∥ [010] and strain ⊥ [001]<br>• $\boldsymbol{B}$ ⊥ [001]
$Pmn'2_1'$ (31.126) $\mathbb{Z}_1$	• $\boldsymbol{E}$ ∥ [001] and $\boldsymbol{B}$ ∥ [010]
$Pn'ma'$ (62.448) $\mathbb{Z}_2$	• $\boldsymbol{B}$ ∥ [010]
$Pm'n2_1'$ (31.125) $\mathbb{Z}_1$	• $\boldsymbol{E}$ ∥ [001] and $\boldsymbol{B}$ ∥ [100]
$Pnm'a'$ (62.447) $\mathbb{Z}_2$	• $\boldsymbol{B}$ ∥ [100]
$P2_1$ (4.7) $\mathbb{Z}_1$	• $\boldsymbol{E}$ ∥ [001] and $\boldsymbol{B}$ ∥ [001] and strain ⊥ [001]
$P2_1/c$ (14.75) $\mathbb{Z}_2$	• $\boldsymbol{B}$ ∥ [001] and strain ⊥ [001]
$Pm'n'2_1$ (31.127) $\mathbb{Z}_1$	• $\boldsymbol{E}$ ∥ [001] and $\boldsymbol{B}$ ∥ [001]
$Pn'm'a$ (62.446) $\mathbb{Z}_2$	• $\boldsymbol{B}$ ∥ [001]
$P2_11'$ (4.8) $\mathbb{Z}_1$	• $\boldsymbol{E}$ ∥ [001] and strain ⊥ [001]
$P2_1/c1'$ (14.76) $\mathbb{Z}_2$	• strain ⊥ [001]
$Pmn2_11'$ (31.124) $\mathbb{Z}_1$	• $\boldsymbol{E}$ ∥ [001]

Table. S 553: Subgroups of $Pn'ma$ (62.443) that can be realized by applying $\boldsymbol{E}/\boldsymbol{B}$ fields and/or uniaxial strains.

Subgroup & SI group	Perturbation

Continued on next page

. . . continued

Subgroup & SI group	Perturbation
Pm (6.18) $\mathbb{Z}_1$	• $\boldsymbol{B} \parallel [010]$ and strain $\perp [010]$<br>• $\boldsymbol{E} \parallel [100]$ and strain $\perp [010]$<br>• $\boldsymbol{E} \parallel [100]$ and $\boldsymbol{B} \parallel [010]$<br>• $\boldsymbol{E} \parallel [001]$ and strain $\perp [010]$<br>• $\boldsymbol{E} \perp [010]$
Pc (7.24) $\mathbb{Z}_1$	• $\boldsymbol{B} \parallel [001]$ and strain $\perp [001]$<br>• $\boldsymbol{E} \parallel [100]$ and strain $\perp [001]$<br>• $\boldsymbol{E} \parallel [100]$ and $\boldsymbol{B} \parallel [001]$<br>• $\boldsymbol{E} \parallel [010]$ and strain $\perp [001]$<br>• $\boldsymbol{E} \perp [001]$
$P2_1$ (4.7) $\mathbb{Z}_1$	• $\boldsymbol{B} \parallel [100]$ and strain $\perp [100]$<br>• $\boldsymbol{E} \parallel [100]$ and strain $\perp [100]$<br>• $\boldsymbol{E} \parallel [100]$ and $\boldsymbol{B} \parallel [100]$
$Pmc2_1$ (26.66) $\mathbb{Z}_1$	• $\boldsymbol{E} \parallel [100]$
$P2_1'$ (4.9) $\mathbb{Z}_1$	• $\boldsymbol{B} \parallel [100]$ and strain $\perp [010]$<br>• $\boldsymbol{B} \parallel [001]$ and strain $\perp [010]$<br>• $\boldsymbol{B} \perp [010]$<br>• $\boldsymbol{E} \parallel [010]$ and strain $\perp [010]$<br>• $\boldsymbol{E} \parallel [010]$ and $\boldsymbol{B} \parallel [100]$
$P\bar{1}'$ (2.6) $\mathbb{Z}_1$	• strain in generic direction
$P2_1'/m$ (11.52) $\mathbb{Z}_1$	• strain $\perp [010]$
Pc' (7.26) $\mathbb{Z}_1$	• $\boldsymbol{B} \parallel [010]$ and strain $\perp [100]$<br>• $\boldsymbol{B} \parallel [001]$ and strain $\perp [100]$<br>• $\boldsymbol{B} \perp [100]$<br>• $\boldsymbol{E} \parallel [010]$ and strain $\perp [100]$<br>• $\boldsymbol{E} \parallel [010]$ and $\boldsymbol{B} \parallel [010]$<br>• $\boldsymbol{E} \parallel [001]$ and strain $\perp [100]$<br>• $\boldsymbol{E} \parallel [001]$ and $\boldsymbol{B} \parallel [001]$<br>• $\boldsymbol{E} \perp [100]$
$Pn'a2_1'$ (33.146) $\mathbb{Z}_1$	• $\boldsymbol{B} \parallel [001]$<br>• $\boldsymbol{E} \parallel [010]$
$P2_1/c'$ (14.78) $\mathbb{Z}_1$	• strain $\perp [100]$

Continued on next page

. . . continued

Subgroup & SI group	Perturbation
$P2_1'$ (4.9) $\mathbb{Z}_1$	• $\boldsymbol{B} \parallel [100]$ and strain $\perp [001]$<br>• $\boldsymbol{B} \parallel [010]$ and strain $\perp [001]$<br>• $\boldsymbol{B} \perp [001]$<br>• $\boldsymbol{E} \parallel [001]$ and strain $\perp [001]$<br>• $\boldsymbol{E} \parallel [001]$ and $\boldsymbol{B} \parallel [100]$
$P2_1'2_1'2_1$ (19.27) $\mathbb{Z}_1$	• $\boldsymbol{B} \parallel [100]$
$P2_1'/c$ (14.77) $\mathbb{Z}_1$	• strain $\perp [001]$
$Pmn'2_1'$ (31.126) $\mathbb{Z}_1$	• $\boldsymbol{B} \parallel [010]$<br>• $\boldsymbol{E} \parallel [001]$

Table. S 554: Subgroups of $Pnm'a$ (62.444) that can be realized by applying $\boldsymbol{E}/\boldsymbol{B}$ fields and/or uniaxial strains.

Subgroup & SI group	Perturbation
Pm' (6.20) $\mathbb{Z}_1$	• $\boldsymbol{B} \parallel [100]$ and strain $\perp [010]$<br>• $\boldsymbol{B} \parallel [001]$ and strain $\perp [010]$<br>• $\boldsymbol{B} \perp [010]$<br>• $\boldsymbol{E} \parallel [100]$ and strain $\perp [010]$<br>• $\boldsymbol{E} \parallel [100]$ and $\boldsymbol{B} \parallel [100]$<br>• $\boldsymbol{E} \parallel [001]$ and strain $\perp [010]$<br>• $\boldsymbol{E} \parallel [001]$ and $\boldsymbol{B} \parallel [001]$<br>• $\boldsymbol{E} \perp [010]$
Pc (7.24) $\mathbb{Z}_1$	• $\boldsymbol{B} \parallel [001]$ and strain $\perp [001]$<br>• $\boldsymbol{E} \parallel [100]$ and strain $\perp [001]$<br>• $\boldsymbol{E} \parallel [010]$ and strain $\perp [001]$<br>• $\boldsymbol{E} \parallel [010]$ and $\boldsymbol{B} \parallel [001]$<br>• $\boldsymbol{E} \perp [001]$
$P2_1'$ (4.9) $\mathbb{Z}_1$	• $\boldsymbol{B} \parallel [010]$ and strain $\perp [100]$<br>• $\boldsymbol{B} \parallel [001]$ and strain $\perp [100]$<br>• $\boldsymbol{B} \perp [100]$<br>• $\boldsymbol{E} \parallel [100]$ and strain $\perp [100]$<br>• $\boldsymbol{E} \parallel [100]$ and $\boldsymbol{B} \parallel [010]$
$Pm'c2_1'$ (26.68) $\mathbb{Z}_1$	• $\boldsymbol{B} \parallel [001]$<br>• $\boldsymbol{E} \parallel [100]$

Continued on next page

...continued

Subgroup & SI group	Perturbation
$P2_1$ (4.7) $\mathbb{Z}_1$	• $\boldsymbol{B} \parallel [010]$ and strain $\perp [010]$<br>• $\boldsymbol{E} \parallel [010]$ and strain $\perp [010]$<br>• $\boldsymbol{E} \parallel [010]$ and $\boldsymbol{B} \parallel [010]$
$P\bar{1}'$ (2.6) $\mathbb{Z}_1$	• strain in generic direction
$P2_1/m'$ (11.53) $\mathbb{Z}_1$	• strain $\perp [010]$
Pc (7.24) $\mathbb{Z}_1$	• $\boldsymbol{B} \parallel [100]$ and strain $\perp [100]$<br>• $\boldsymbol{E} \parallel [010]$ and strain $\perp [100]$<br>• $\boldsymbol{E} \parallel [010]$ and $\boldsymbol{B} \parallel [100]$<br>• $\boldsymbol{E} \parallel [001]$ and strain $\perp [100]$<br>• $\boldsymbol{E} \perp [100]$
$Pna2_1$ (33.144) $\mathbb{Z}_1$	• $\boldsymbol{E} \parallel [010]$
$P2_1'/c$ (14.77) $\mathbb{Z}_1$	• strain $\perp [100]$
$P2_1'$ (4.9) $\mathbb{Z}_1$	• $\boldsymbol{B} \parallel [100]$ and strain $\perp [001]$<br>• $\boldsymbol{B} \parallel [010]$ and strain $\perp [001]$<br>• $\boldsymbol{B} \perp [001]$<br>• $\boldsymbol{E} \parallel [001]$ and strain $\perp [001]$<br>• $\boldsymbol{E} \parallel [001]$ and $\boldsymbol{B} \parallel [010]$
$P2_1'2_1'2_1$ (19.27) $\mathbb{Z}_1$	• $\boldsymbol{B} \parallel [010]$
$P2_1'/c$ (14.77) $\mathbb{Z}_1$	• strain $\perp [001]$
$Pm'n2_1'$ (31.125) $\mathbb{Z}_1$	• $\boldsymbol{B} \parallel [100]$<br>• $\boldsymbol{E} \parallel [001]$

Table. S 555: Subgroups of $Pnma'$ (62.445) that can be realized by applying $\boldsymbol{E}/\boldsymbol{B}$ fields and/or uniaxial strains.

Subgroup & SI group	Perturbation
Pm (6.18) $\mathbb{Z}_1$	• $\boldsymbol{B} \parallel [010]$ and strain $\perp [010]$<br>• $\boldsymbol{E} \parallel [100]$ and strain $\perp [010]$<br>• $\boldsymbol{E} \parallel [001]$ and strain $\perp [010]$<br>• $\boldsymbol{E} \parallel [001]$ and $\boldsymbol{B} \parallel [010]$<br>• $\boldsymbol{E} \perp [010]$

Continued on next page

...continued

Subgroup & SI group	Perturbation
Pc' (7.26) $\mathbb{Z}_1$	• $\boldsymbol{B} \parallel [100]$ and strain $\perp [001]$<br>• $\boldsymbol{B} \parallel [010]$ and strain $\perp [001]$<br>• $\boldsymbol{B} \perp [001]$<br>• $\boldsymbol{E} \parallel [100]$ and strain $\perp [001]$<br>• $\boldsymbol{E} \parallel [100]$ and $\boldsymbol{B} \parallel [100]$<br>• $\boldsymbol{E} \parallel [010]$ and strain $\perp [001]$<br>• $\boldsymbol{E} \parallel [010]$ and $\boldsymbol{B} \parallel [010]$<br>• $\boldsymbol{E} \perp [001]$
$P2_1'$ (4.9) $\mathbb{Z}_1$	• $\boldsymbol{B} \parallel [010]$ and strain $\perp [100]$<br>• $\boldsymbol{B} \parallel [001]$ and strain $\perp [100]$<br>• $\boldsymbol{B} \perp [100]$<br>• $\boldsymbol{E} \parallel [100]$ and strain $\perp [100]$<br>• $\boldsymbol{E} \parallel [100]$ and $\boldsymbol{B} \parallel [001]$
$Pmc'2_1'$ (26.69) $\mathbb{Z}_1$	• $\boldsymbol{B} \parallel [010]$<br>• $\boldsymbol{E} \parallel [100]$
$P2_1'$ (4.9) $\mathbb{Z}_1$	• $\boldsymbol{B} \parallel [100]$ and strain $\perp [010]$<br>• $\boldsymbol{B} \parallel [001]$ and strain $\perp [010]$<br>• $\boldsymbol{B} \perp [010]$<br>• $\boldsymbol{E} \parallel [010]$ and strain $\perp [010]$<br>• $\boldsymbol{E} \parallel [010]$ and $\boldsymbol{B} \parallel [001]$
$P\bar{1}'$ (2.6) $\mathbb{Z}_1$	• strain in generic direction
$P2_1'/m$ (11.52) $\mathbb{Z}_1$	• strain $\perp [010]$
Pc (7.24) $\mathbb{Z}_1$	• $\boldsymbol{B} \parallel [100]$ and strain $\perp [100]$<br>• $\boldsymbol{E} \parallel [010]$ and strain $\perp [100]$<br>• $\boldsymbol{E} \parallel [001]$ and strain $\perp [100]$<br>• $\boldsymbol{E} \parallel [001]$ and $\boldsymbol{B} \parallel [100]$<br>• $\boldsymbol{E} \perp [100]$
$Pna'2_1'$ (33.147) $\mathbb{Z}_1$	• $\boldsymbol{B} \parallel [100]$<br>• $\boldsymbol{E} \parallel [010]$
$P2_1'/c$ (14.77) $\mathbb{Z}_1$	• strain $\perp [100]$
[truncated: 603,684 more chars]
